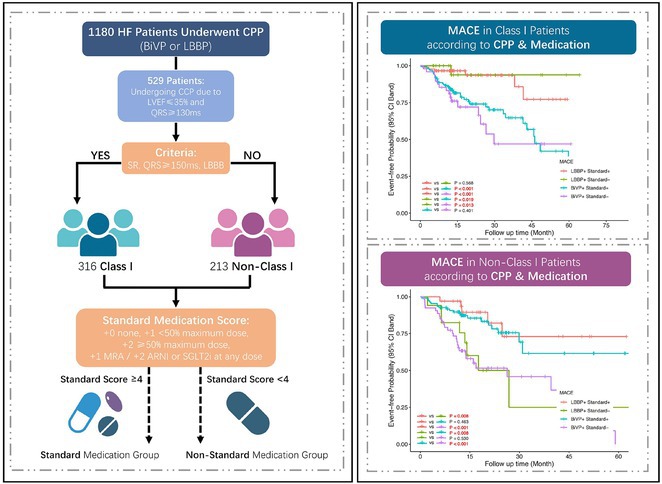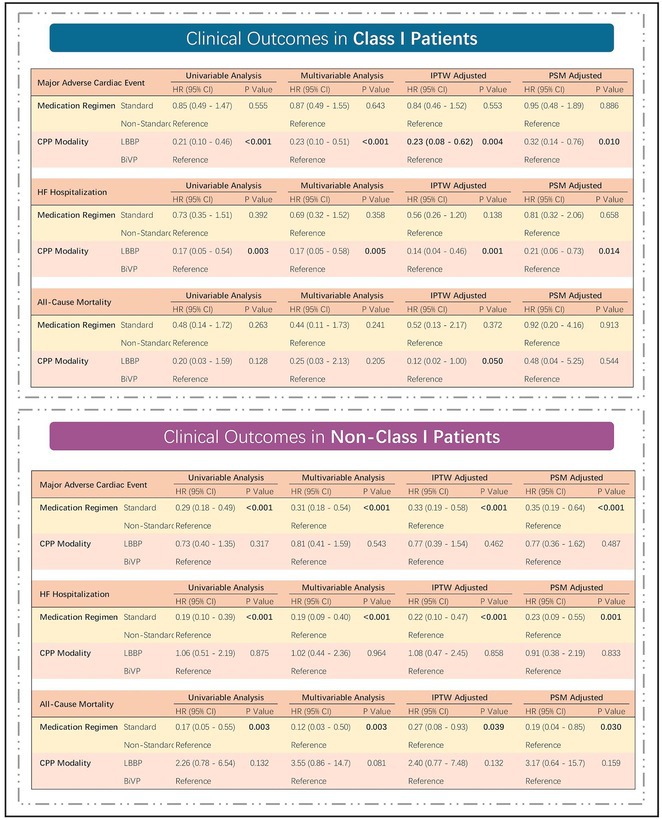# Standard Poster Abstracts for the 17th Asia Pacific Heart Rhythm Society (APHRS) Scientific Sessions

**DOI:** 10.1002/joa3.70004

**Published:** 2025-03-14

## USEFULLNESS OF HDGC (HIGH DENSITY GRID CATHETER) FOR VT STORM ABLATION

### **KAZUMASA ADACHI** ^1^, YASUTAKA HIRAYAMA^2^, NAOKI HOSOKAWA^1^, TOMOKO TOUDA^1^, HITOSHI INANAMI^1^, RIO SHIRAKI^1^, TAKASHI MURO^3^, YUKIKATSU OKADA^3^

#### ^1^Heart Rhythm Center, Midori Hospital, KOBE, Japan,^2^Akashi Medical Center, Akashi, Japan,^3^Heart Valve Center, Midori Hospital, KOBE, Japan

**Introduction:** In VT ablation, evaluation of contact bipolar potential during VT and sinus rhythm is extremely important. However, depending on the direction of beat excitation propagation, there are cases in which this potential cannot be evaluated accurately. This time, we report a case in which bipolar blindness in an ablation catheter was successfully covered by HDGC.

**Methods:** N/A

**Results:** Case: 77‐year‐old male. On October X, 2018, the patient was urgently hospitalized with VT associated with hypertrophic cardiomyopathy and apical aneurysm. Endocardial ablation was performed on October Y of the same year, and clinical VT could no longer be induced. He had a TV‐ICD implanted and was scheduled to be discharged from the hospital, but on November Z, a VT storm occurred. Clinical VT had recurred and was resistant to drug treatment, so we underwent semi‐emergency ablation. As VT could not be controlled using the endocardial approach as in the first session, we shifted to the epicardial approach. Mapping of the epicardial side revealed that VT had a focal excitation pattern. Late potential (LP) was not observed in the potential of the ablation catheter at the earliest excitation site of VT. The potential during sinus rhythm of HDGC at the same site revealed LP in the along direction, but no such potential was observed in the across direction. During VT, VT was stopped by energizing the earliest excitation region, but NSVT with the same waveform remained, so we added ablation using the LPM (Late Potential Map) guide created by HDGC, which made it impossible to induce VT. VT has not been observed since then.

**Conclusions:** Ablation using LPM by HDGC was effective for VT storm.

## ORAL PROPANOLOL ADMINISTRATION IN PRE‐EXCITED ATRIAL FIBRILLATION IN WOLFF‐PARKINSON WHITE SYNDROME WITH THYROID STORM: A CASE REPORT

### **ANDI TIARA SALENGKE ADAM**, MUZAKKIR AMIR

#### Hasanuddin University, Makassar, Indonesia

**Introduction:** Atrial fibrillation (AF) can have severe consequences in patients with pre‐existing Wolff‐Parkinson‐White (WPW) Syndrome. The combination of WPW Syndrome and AF induced by thyroid storm, a life‐threatening condition, is rare and potentially devastating. The rare combination of WPW Syndrome and AF highlights the critical need to consider the interplay between cardiac conditions and hormonal imbalances in AF diagnosis and management.

**Methods:** N/A

**Results:** A 55‐year‐old male patient was admitted to the emergency department with a history of palpitations and irregular heart beat without a clear triggered factor that started two hours prior to admission. The patient also experienced shortness of breath, dizziness, nausea and vomiting. The patient had a history of hyperthyroidism as well as history of WPW Syndrome and previously undergone ablation procedures for WPW Syndrome in the past although the ablation results were unsuccessful. ECG showed irregular broad‐complex tachycardia with varying QRS width and characteristic delta waves are best seen in V2. Due to these findings, coupled with unstable hemodynamics, we then proceeded with cardioversion but the arrhythmia stubbornly persisted. With conventional methods faltering, we then decided to administer oral propranolol along with therapy managing the thyroid storm. This strategic shift in management was a turning point, and after 9 days of treatment, the patient was then safely discharged. Propranolol, has been studied for its impact on the accessory pathway in individuals with WPW syndrome. Research indicates that propranolol can increase the effective anterograde refractory period (EARP) of the accessory pathway, suggesting that propranolol can be effective in preventing the development of tachycardias in WPW patients.

**Conclusions:** This case emphasizes the importance of considering the interplay between underlying cardiac conditions and hormonal imbalances in the diagnosis and management of atrial fibrillation. Propanolol may be adjusted to the treatment plan as needed to ensure optimal management of the patient's condition.

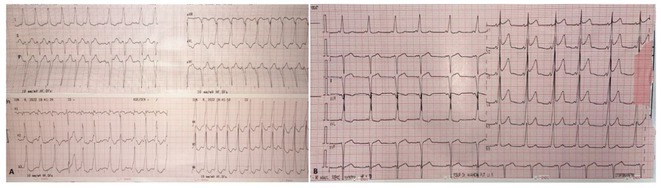

## CATHETER ABLATION TO CURE A CHILD WITH CARDIOMYOPATHY: A UNIQUE EXPERIENCE OF FIVE CASES

### **USNISH ADHIKARI**, DR. K.K. NARAYANAN NAMBOODIRI, AJIT KUMAR VALAPARAMBIL, ABHILASH S.P., KRISHNA KUMAR MOHANAN NAIR, JYOTHI VIJAY M.S.

#### Sree Chitra Tirunal Institute for Medical Sciences and Technology (SCTIMST), Trivandrum, Thiruvananthapuram, India

**Introduction:** We hereby present 5 cases over a period of last 3 years ‐ children who were diagnosed to have dilated cardiomyopathy, were being managed medically with guarded prognosis and presented to our center. Echocardiography showed severe left ventricular dysfunction with global LV hypokinesia. ECG was suggestive of narrow complex tachycardia with long RP. They were taken up for electrophysiological study under under general anaesthesia.

**Methods:** N/A

**Results:** The mean age was 6.3 years ‐ minimum age being 1 year and maximum 11 years. EP study in these children, showed regular narrow QRS tachycardia with 1:1 VA relationship and earliest activation at CS os. Response to ventricular overdrive pacing from RV showed concealed entrainment with V‐A‐V response on cessation of pacing. His refractory PVC from RV septum advanced atrial signals and also terminated tachycardia without conducting to atrium. Pre‐excitation index were < 75 ms. EP study was thus suggestive of decrementally conducting retrograde slow pathway ‐ permanent junctional reciprocating tachycardia (PJRT). 4 od the cases showed the pathway to be in typical septal location. 3D mapping (Ensite X) was used in the latest (3.5 years old) case‐ activation mapping which showed centrifugal activation with earliest atrial activation at right lateral region @ 8’o Clock of tricuspid annulus, which was a rare location for PJRT. Radiofrequency ablation in all these children led to successful termination of tachycardia. No recurrence was noted and echocardiography done on follow‐up month showed normalization of LV function and improvement in child's clinical status.

**Conclusions:** These unique cases demonstrate management of a child with tachycardiomyopathy due to PJRT, by catheter ablation. Important learning point is to carefully evaluate children with ventricular dysfunction properly with electrocardiogram/ holter and rule out incessant tachycardias like PJRT, which can be successfully cured with ablation.

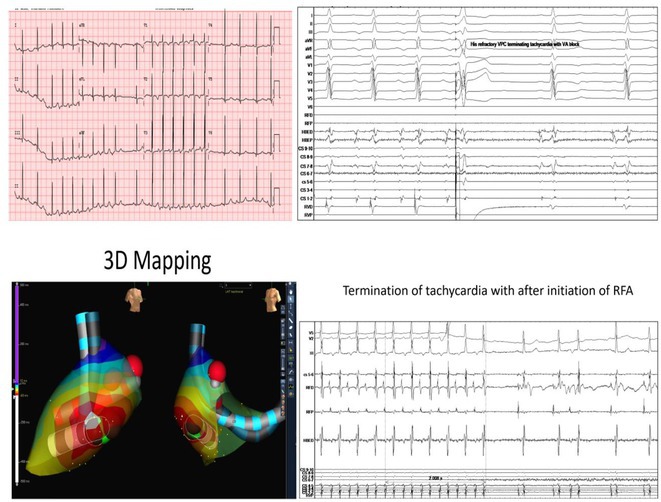

## MORTALITY OUTCOMES AND INCIDENCE OF SHOCKS IN PATIENTS IMPLANTED WITH INTRACARDIAC DEFIBRILLATORS: A SINGLE‐CENTRE EXPERIENCE OVER TWO DECADES

### **USNISH ADHIKARI**, HARSH KUMAR PANDEY, AJIT KUMAR VALAPARAMBIL, K.K. NARAYANAN NAMBOODIRI, ABHILASH S.P., KRISHNA KUMAR MOHANAN NAIR, JYOTHI VIJAY M.S.

#### Sree Chitra Tirunal Institute for Medical Sciences and Technology (SCTIMST), Trivandrum, Thiruvananthapuram, India

**Introduction:** The beneficial effects of automated implantable cardioverter defibrillators(AICDs) in primary and secondary prevention patients are well known. There is scarcity of Indian data on long‐term follow‐up of AICD recipients. This study aimed to evaluate outcomes & therapies in patients who underwent AICD implantation and also assess the differences between primary & secondary prevention groups.

**Methods:** This is a descriptive single‐center study with retrospective case enrolment and cross‐sectional follow‐up. Patients who underwent AICD/CRT‐D implantation from January 1997 to June 2020 were identified from institutional database. Study population was grouped by type of prevention (secondary or primary). Device interrogation was done for appropriate & inappropriate therapies. Patients with twelve months of missing data were considered lost to follow‐up.

**Results:** 428(81% male, mean age 55+/‐11 years) patients were included. 67.7% patients received an AICD for secondary & 32.3% for primary prevention. Incidence of appropriate shock was 14% in primary prevention & 30% in secondary prevention group. During 1913 patients‐year follow‐up(mean of 4.4+/‐2.7 years), secondary prevention patients exhibited 33% increased risk for appropriate shock compared with primary prevention group. LV dysfunction was significant predictor of appropriate shock. Atrial fibrillation was the most common cause of inappropriate shock. Overall mortality was 11.6%‐ 11.5% for primary & 11.7% for secondary prevention patients. Congestive cardiac failure was the most common mode of death in secondary prevention group, whereas non‐cardiac death was more commonly noted in primary prevention group. On multivariate analysis, appropriate shock, non‐ischemic CMP, was observed as a strong predictor of mortality.

**Conclusions:** On long‐term follow‐up, secondary prevention AICD recipients exhibited a higher risk of appropriate therapy compared to primary prevention group. Both groups showed lower & similar occurrences of inappropriate shocks. Comparable mortality patterns were observed between both groups.

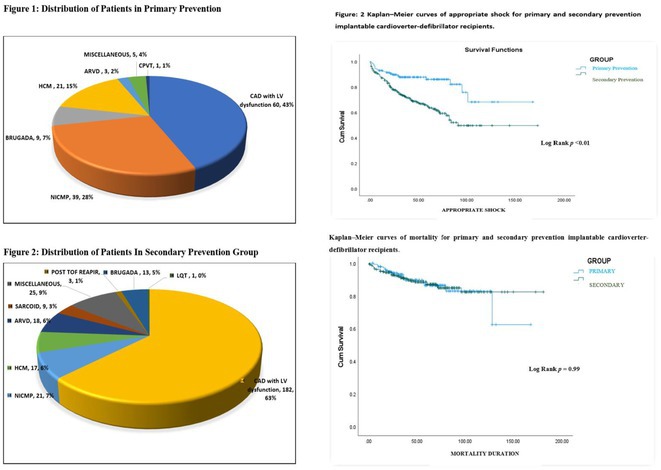

## HIGH‐DENSITY MAPPING FOR PULMONARY VEIN ISOLATION IN PATIENT WITH PAROXYSMAL ATRIAL FIBRILLATION: A SINGLE CENTRE EXPERIENCE

### **BENI AFRIANSYAH** ^1^, PAOZUL MUBTAGI^1^, GIKY KARWIKY^1^, MOHAMMAD IQBAL^2^

#### ^1^Al Ihsan General Hospital of West Java Province, Bandung, Indonesia,^2^Cardiology Department of Padjadjaran University, Bandung, Indonesia

**Introduction:** High‐density mapping of the pulmonary vein can improve the detection of conduction gaps in the radiofrequency ablation lesions after pulmonary vein isolation (PVI) for the treatment of atrial fibrillation (AF). PVI is reported to be effective in 60 to 85% of the patients, especially in patients with paroxysmal episodes of AF. Various structural diseases can underlie the risk of atrial remodeling. In this study we performed high density mapping in paroxysmal AF patients with various underlying structural abnormalities.

**Methods:** This retrospective study included patients scheduled for pulmonary vein isolation. Acute PVI was defined as an entrance and exit block and loss of pulmonary vein potential. The left atrium was mapped then remapped using the HD Grid high‐density mapping catheter to identify residual conduction gaps in the PVI lines by voltage and activation criteria.

**Results:** A total of 10 patients were included (mean age 56,7 years, 30 % female, 100% had paroxysmal AF). Structural heart disease was identified in 1 patient who had secundum atrial septal defect then underwent device closure after ablation, 1 patient had hypertrophic cardiomyopathy, 2 patients had concentric left ventricular remodeling because of hypertensive heart disease and another 6 patients had normal structural heart. Average total procedure time was 250 minutes and all patient had entrance and exit block and loss of pulmonary vein potential after ablation. Minimal scar was identified in patient with structural heart disease and in patient with normal heart we found no scar with voltage mapping. Atrial fibrillation was terminated during ablation in 1 patient, for rest of the patient sinus rhythm persisted before and after ablation. Mean follow up was 6.8 months. All patient does not had episode of AF with 24 hours Holter after 3 months of ablation. AF was recurred in one patient after 5 months of ablation.

**Conclusions:** Various structural diseases can underlie the risk of atrial remodeling in patient with paroxysmal AF. HD mapping may have the potential to improve AF ablation success rates in the long term.

## ROLE OF SEPTO‐PULMONARY BUNDLE IN SUSTAINING LEFT ATRIAL TACHYARRHYTHMIAS

### **RAKESH AGARWAL** ^1^, ANH HONG NGUYEN^1^, FADHLY SYAH AMRI^2^, RAJIV MAHAJAN^1^

#### ^1^University of Adelaide and the Lyell McEwin Hospital, Adelaide, Australia,^2^Abbott Medical, Adelaide, Australia

**Introduction:** The role of endo‐epicardial connections of Septo‐Pulmonary Bundle (SPB) in left atrial tachyarrhythmia is not well defined.

**Methods:** Two cases of left atrial tachycardia utilizing SPB are described along with their electro‐anatomic mapping and ablation strategy.

**Results:** Case 1: A 65‐year‐old patient presented with incessant left atrial flutter 8 months after AF ablation. High density mapping with HD grid demonstrated colliding wavefront around mitral annulus and anticlockwise activation inferior and anterior to right PVs. Propagation map showed focal activation in right mid posterior wall, with line of block endocardially at the right part of the roof and inferior lines. Entrainment confirmed macro reentry around right PVs (PPI‐TCL was 0ms (adjacent to right part of inferior line), 0ms (anterior LA adjacent to roof line near RSPV), 0ms (septal LA), 80ms (proximal and distal CS), and 150ms (left part of posterior wall). There was fractionated low voltage signal at the right part of the posterior wall that could only be entrained at 20A, 1ms PW(PPI‐TCL 0ms). Endocardial activation demonstrated a skip in activation from roof to mid posterior wall with missing CL corresponding to epicardial activation through the SPB. Focal ablation on the mid posterior wall terminated the tachycardia. Case 2: A 67‐year old male patient underwent SRI for persistent AF. The patient was in AF during the procedure. After completion of the SRI, the AF terminated into left atrial tachycardia with 2:1 AV conduction. High density mapping with HD grid revealed a focal left atrial tachycardia with line of endocardial block along roof line, but exiting to anterior LA through SPB (earliest activation, outside single ring, on anterior wall at proximal SPB region with later activation near ablation line). Further ablation on the roof at region of far‐field fragmented EGM (denoting overlying SPB) led to posterior wall isolation and dissociated tachycardia circuit in the posterior LA. Focal ablation at the inferior aspect of RSPV led to termination of tachycardia (Figure 2).

**Conclusions:** These cases demonstrate that SPB may play a critical role in maintenance of left atrial tachyarrhythmias.

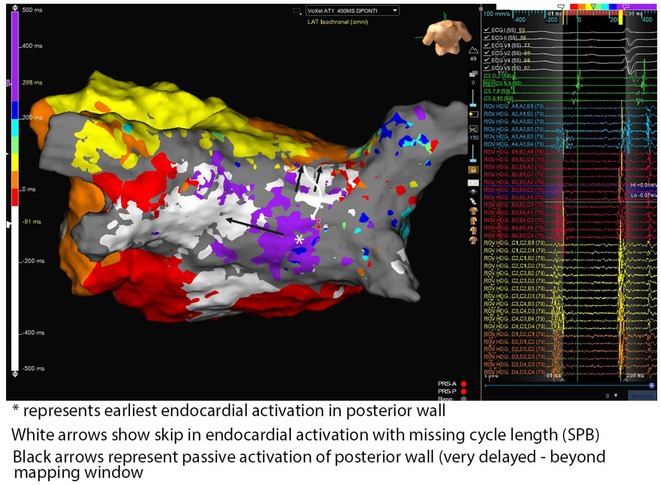

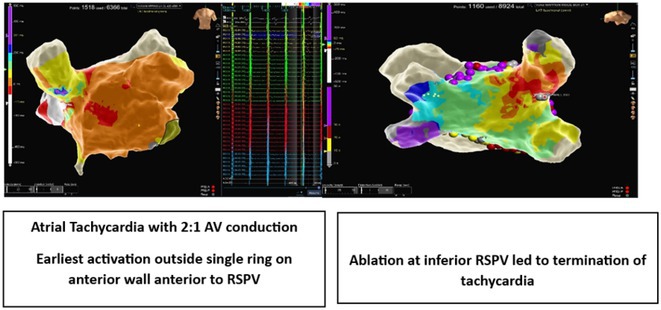

## 12 LEAD ECG GUIDED CRT OPTIMISATION (ECGCRTOPT) IMPACT IN QRS DURATION (QRSD), LEFT VENTRICULAR EJECTION FRACTION (LVEF) AND SYMPTOMS

### **SHARAD AGARWAL**, SANDRA SILVA, IAN TURNER, MARTA CUNHA, ABBIE NORRIS, MUNMOHAN VIRDEE, SIMON FYNN, PATRICK HECK

#### Royal Papworth Hospital NHS Trust, Cambridge, United Kingdom

**Introduction:** CRT optimisation can be a complex process, but we use a simpler ECG based optimisation protocol (ECGCRTOPT) which is widely applicable in hospital or in pacing clinic. We aimed to study the relation between QRSd, LVEF and symptoms following ECGCRTOPT.

**Methods:** This was a single‐centre retrospective study of consecutive HF patients with CRT implants/upgrades. A ECGCRTOPT protocol was used to produce the narrowest QRSd using the latest point of activation (qLV) and optimal AV and VV intervals, whilst avoiding LV latency, QRS fractionation or P wave truncation (by adding 40‐50ms after the end of P wave and start of BIV QRS). This was performed at post implant and at the 2 month check in clinic (visit 1). ECG's and echo's were performed pre implant and at visit 1, along with NYHA class and EQ‐5D‐5L HF questionnaire (HFQ). Blood pressure recordings were used to confirm an increase in BP after optimisation.

**Results:** 61 patients were enrolled, with a mean age of 72 years old, 77% were male and 38% had ischaemic aetiology. The mean QRSd was 167ms pre implant, 131ms pre discharge and 133ms at visit 1 (p<0.001 pre v visit1), whilst the mean EF increased from 30% pre to 44% at visit 1 showing a significant effect of optimised CRT therapy (p<0.001), in line with publishes studies. 82% of patients had an increase in EF >5% and 66% had an increase> 10% (40% had EF > 50% at visit 1). Of the patients who had an increase <5%, the majority (81%) had an ischaemic aetiology.There was also a significant shift in NYHA class pre visit 1, with a higher percentage of patients in NYHA 1 or 2 and less in class 3 or 4 (P<0.01). The HFQ showed no change in scores between visits and appears insensitive to symptoms. Analysis of change in QRSd against change in EF showed only a very weak relationship (R=0.08, p=0.54).

**Conclusions:** CRT implant coupled with the ECGCRTOPT can help reduce QRSd, improve EF and NYHA class in most patients. Change in QRSd was not predictive of the change in EF. The HFQ was not sensitive to changes in symptoms but did allow identification of patients with other needs (such as depression management) for further referral. More extended follow‐up is ongoing.

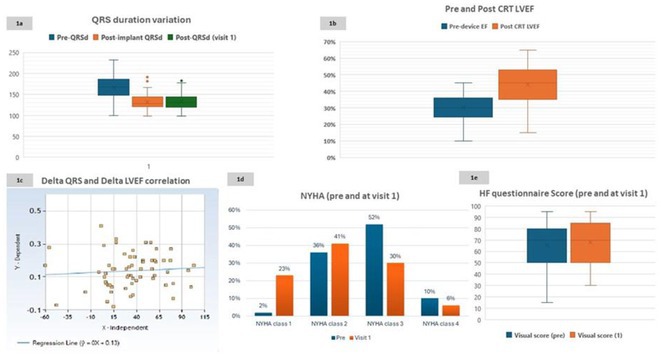

## DECIPHERING ARRHYTMOGENIC RIGHT VENTRICULAR CARDIOMYOPATHY (ARVC): DIAGNOSTIC POTENTIAL OF JT INTERVAL RATIO ACROSS PRECORDIAL LEADS IN RVOT VENTRICULAR ARRHYTHMIA

### **GANANG K. AHIMSA** ^1^, YUNIA DUANA^1^, FERA HIDAYATI^2^, ERIKA MAHARANI^2^

#### ^1^Cardiology Resident of Faculty medicine, Gadjah Mada University, Yogyakarta, Indonesia,^2^Departement of Cardiology and Vascular Medicine, Dr Sardjito General Hospital/Gadjah Mada University, Yogyakarta, Indonesia

**Introduction:** Arrhythmogenic Right Ventricular Cardiomyopathy (ARVC) is a genetic cardiomyopathy predominantly afflicting young adults, with sudden death being its leading cause of mortality. Conversely, idiopathic Ventricular Arrhythmia (VA) presents a generally benign clinical course without evident structural abnormalities. Notably, both conditions may manifest as VA originating from RVOT with a Left Bundle Branch Block (LBBB) pattern and an inferior axis. Fibrosis characteristic of ARVC disrupts right ventricular repolarization, potentially prolonging the JT interval.

**Methods:** This analytical observational study employed a cross‐sectional design to scrutinize medical records of patients at Sardjito Hospital from 2016 to 2021, characterized by VA exhibiting LBBB morphology and an inferior axis. These patients underwent series of examinations, including electrocardiography and cardiac magnetic resonance, to ascertain the diagnosis of ARVC. The study focused on the ratio of JT interval between right and left precordial leads, calculated by dividing the sum of JT interval in leads V1‐V3 by that in leads V4‐V6. Diagnosis of ARVC was adjudicated based on the PADUA criteria.

**Results:** In this study, 39 patients were enrolled: 20 with Idiopathic Ventricular Arrhythmia (VA) and 19 with ARVC. Baseline analysis found no demographic or clinical differences between the two groups. However, the JT interval ratio between right to left precordial leads was significantly higher in ARVC patients (mean 1.07 vs. 0.91 in idiopathic VA, p < 0.001). An ROC curve identified a JT interval ratio cutoff of 1.01, with 43.58% of patients having a ratio above this threshold. This cutoff showed good diagnostic performance for ARVC, with 89.74% accuracy, 84.21% sensitivity, 95% specificity, 94.11% positive predictive value, and 86.36% negative predictive value.

**Conclusions:** A JT interval ratio >1.01 between the right and left precordial leads showed strong diagnostic value in identifying ARVC in patients with ventricular arrhythmia originating from the right ventricular outflow tract (RVOT).

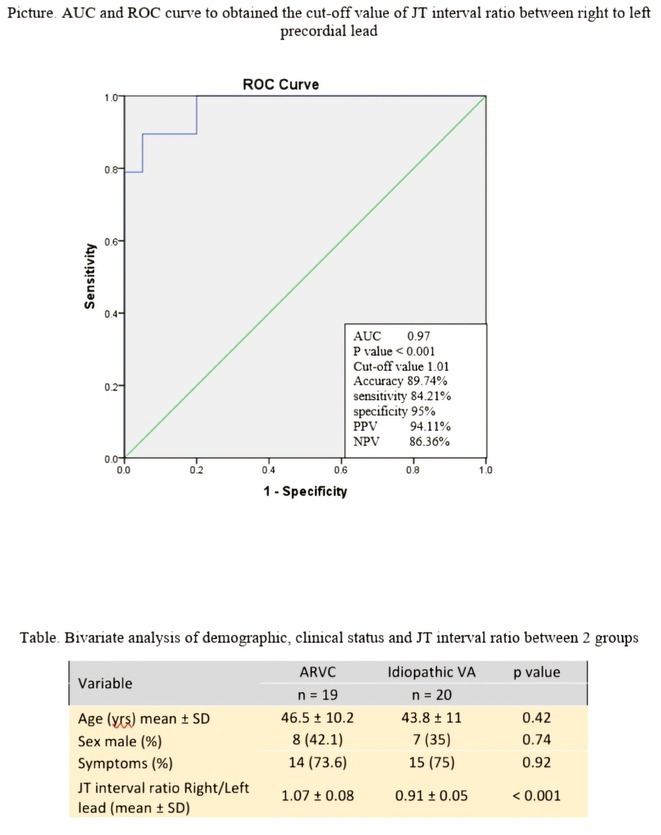

## UNRAVELING THE ENIGMA: RARE CONVERGENCE OF LEFT VENTRICULAR NON‐COMPACTION (LVNC) CARDIOMYOPATHY AND WOLFF‐PARKINSON WHITE (WPW) SYNDROME IN THE REMOTE REACHES OF RIAU, INDONESIA

### **GANANG K. AHIMSA**, M. ARIF HABIBI NASUTION, IRWANSYAH RUDIANTO PURBA

#### Awal Bros Hospital Bagan Batu, Rokan Hilir, Indonesia

**Introduction:** Left Ventricular non‐Compaction cardiomyopathy represents a rare subtype of primary cardiomyopathy often associated with a wide array of arrhythmias. Here, we present a noteworthy case of LVNC concomitant with WPW syndrome, constituting a highly uncommon co‐occurrence of two distinct cardiac diseases. This case was identified in a remote area geographically distant from sub‐specialized electrophysiology centers, underscoring the challenge of managing complex cardiac conditions in resource‐limited settings.

**Methods:** N/A

**Results:** A 54‐year‐old gentleman presented to the emergency room with complaints of palpitations and a near‐syncope episode. He reported experiencing episodic palpitations and near‐syncope for the past six months. Physical examination revealed extreme rapid heart rate. Initial electrocardiography (ECG) displayed a slightly irregular wide QRS complex with a rate ranging between 180‐200 beats per minute. Despite administration of amiodarone with close monitoring, tachyarrhythmia persisted. Subsequent electrical cardioversion at 120 Joules was performed, leading to conversion to sinus rhythm with heightened QRS voltage and intermittent pre‐excitation indicative of WPW type A pattern. Based on these ECG findings, the tachyarrhythmia was presumed to be atrial fibrillation (AF) with pre‐excitation. Echocardiography revealed mild left ventricular dysfunction (ejection fraction of 45%) with LV enlargement. Additionally, excessively prominent trabeculation and a non‐compacted (NC):(C) ratio exceeding 2 were observed, meeting echocardiography criteria for LVNC.

**Conclusions:** The coexistence of LVNC cardiomyopathy and WPW syndrome is an exceptional clinical rarity. Diligent monitoring in the emergency is imperative for early detection of potentially malignant arrhythmias. Despite resource limitations, timely intervention can prevent the progression to more severe arrhythmias and mitigate the risk of sudden cardiac death. Additional diagnostic modality is warranted in this patient to assess the need for ablation.

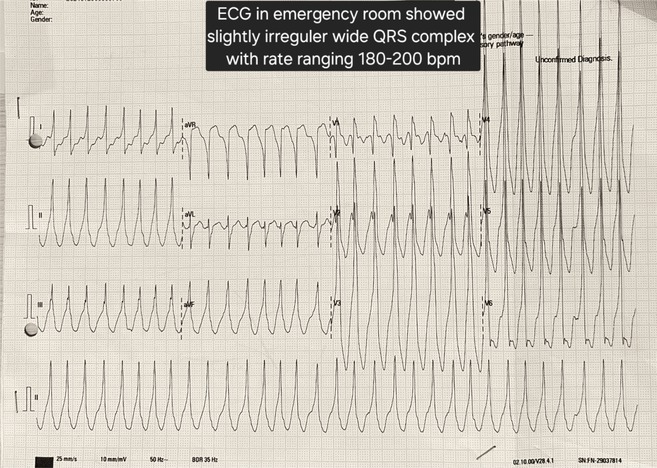

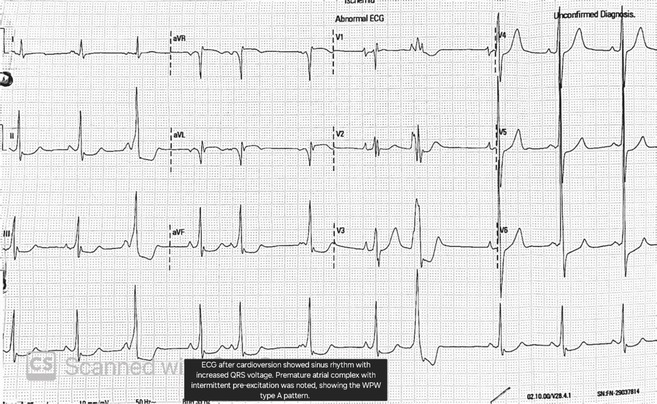

## ROLE OF CARDIAC RESYNCHRONIZATION THERAPY FOR PACING‐INDUCED CARDIOMYOPATHY IN CHILDREN WITH CONGENITAL ATRIOVENTRICULAR BLOCK

### FANDI AHMAD

#### National Cardiovascular Center Harapan Kita, Jakarta, Indonesia

**Introduction:** One in 20,000 newborns have congenital heart block and pacemaker implantation is indicated in congenital AV block. For decades, it has been treated using right ventricular (RV)‐based pacing. However, in children with RV pacing, the incidence of left ventricular (LV) dysfunction ranged from 6.0 percent to 13.4 percent over a medium follow‐up of less than ten years. The detrimental effect of RV pacing on LV systolic function has been termed Pacing‐Induced Cardiomyopathy (PICM) and defined as a ≥10% decrease in Left Ventricular Ejection Fraction (LVEF), with resultant LVEF <50%. Patients with PICM have demonstrated a consistent improvement in clinical status with cardiac resynchronization therapy with pacemaker.

**Methods:** N/A

**Results:** A 2 years old boy with a mother with Sjogren syndrome during pregnancy. He has been detected of complete AV block prenatally at 24 weeks. The mother regularly used hydroxychloroquine while pregnant. On the seventh day of life, a VVI pacemaker was implanted. A unipolar epicardial electrode was placed in the right ventricular (RV) anterior wall. At the age of two, he was referred to the hospital because of congestive heart failure (Ross criteria III). The patient was experiencing recurrent cough and failure to thrive for 6 months. The ECG showed atrioventricular dissociation, right ventricular pacing with wide QRS and the chest X‐ray showed marked cardiomegaly. Echocardiography revealed LV dilatation and severe decrease in LV function with LVEF 20%. Pacemaker interrogation showed RV pacing at 99 %. Then the patient underwent a CRT‐P implantation by left thoracotomy under general anesthesia. The VVI generator and RV electrode were explanted, and three unipolar leads were then implanted epicardially in the right atrium, the RV inferior wall, and the mid‐basal segment of the LV lateral wall. After 2 months of follow‐up post‐CRT implantation, the patient has an improvement of clinical symptoms and LVEF has increased to 42%.

**Conclusions:** High‐burden of RV pacing may lead to PICM and upgrading to biventricular pacing by CRT‐P implantation seems to have a promising result.

## CLINICAL IMPACT OF NEW‐ONSET RIGHT BUNDLE BRANCH BLOCK AFTER TRANSCATHETER AORTIC VALVEIMPLANTATION: A SYSTEMATIC REVIEW AND META‐ANALYSIS

### **MOHAMMED AHMED**, KARAN RAO, RAVINAY BHINDI

#### University of Sydney, Sydney, Australia

**Introduction:** Transcatheter aortic valve implantation (TAVI) is an effective treatment for symptomatic severe aortic stenosis, however, is often complicated by new permanent pacemaker implantation (PPI) in 9‐26% cases. Pre‐existing right bundle branch block (RBBB) and new‐onset left bundle branch block (LBBB) are well published predictors of PPI after TAVI, however the impact of new‐onset RBBB is not well understood.

**Methods:** We comprehensively searched the following databases, MEDLINE, Embase, Web of Science and PubMed, from the date of establishment until May 2024 to screen for studies on new‐onset RBBB after TAVI. Our primary outcome was the association between new‐onset RBBB and PPI at 1 year, while all‐cause mortality was the secondary outcome.

**Results:** We identified and screened 102 potential eligible studies. After abstract and full‐text screening, a total of 3 retrospective cohort studies were included with 2,812 patients for analysis. New‐onset RBBB was found in 1.7% of cases and increased the risk of PPI at 1 year (RR: 6.08; 95% CI: 4.39‐8.41, *p* < 0.001). New‐onset RBBB was not associated with the risk of all‐cause mortality at 1 year (RR: 1.74; 95% CI: 0.88‐3.46, *p* = 0.11).

Figure 1: Forest plot of PPI rates in patients with and without new‐onset RBBB after TAVI.

Figure 2: Forest plot of all‐cause mortality rates in patients with and without new‐onset RBBB after TAVI.

**Conclusions:** This meta‐analysis of 3 retrospective cohort studies suggests that new‐onset RBBB is uncommon after TAVI but significantly increases the risk of PPI but not all‐cause mortality at 1 year. Only 3 studies with moderate‐to‐high degree of heterogeneity were available for analysis. Future dedicated prospective studies with longer‐term follow ups are required to further understand this area.

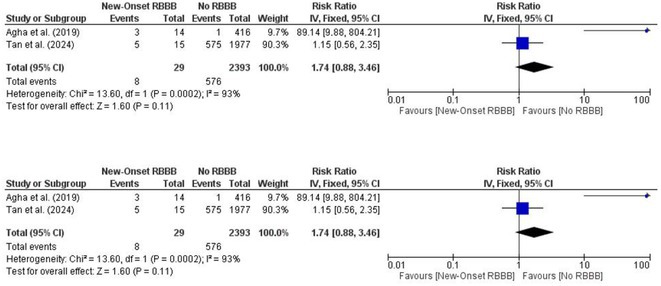

## RISK OF ATRIAL FIBRILLATION IN RELATION TO STEATOTIC LIVER DISEASE STATUS ACROSS MENOPAUSAL PHASES

### JUNGMIN CHOI^1^, KYUNG‐DO HAN^2^, SO‐RYOUNG LEE^1^, EUE‐KEUN CHOI^1^, KYUNG‐YEON LEE^1^, **HYO‐JEONG AHN** ^1^, BONGSEONG KIM^3^, SEIL OH^1^

#### ^1^Seoul National University Hospital, Seoul, Korea, Republic of,^2^Soongsil University, Seoul, Korea, Republic of,^3^The Catholic University of Korea, Seoul, Korea, Republic of

**Introduction:** Atrial fibrillation (AF) is influenced by sex hormones, highlighting a potential gender‐specific risk factor. Metabolic dysfunction‐associated steatotic liver disease (MASLD), prevalent in about one‐third of Korean adults, is also implicated in AF risk. This study assesses the impact of MASLD and its etiologies on AF risk among premenopausal and postmenopausal women.

**Methods:** Utilizing the Korean National Health Insurance Service, we analyzed participants from the 2009 national health and cancer screenings, excluding those with AF, a history of hysterectomy, liver cancer, or liver transplantation. Participants, aged 5‐30 at menarche and 30‐60 at menopause, were divided into premenopausal and postmenopausal groups, further categorized by liver disease status: No Steatosis, MASLD, MASLD with combined etiologies, Metabolic‐Associated Alcoholic Liver Disease (MetALD), Alcoholic Liver Disease (ALD), specific etiology Steatotic Liver Disease (SLD), and Cryptogenic SLD.

**Results:** Of 2,181,691 participants, 903,078 were premenopausal and 1,287,613 postmenopausal. Premenopausal women showed AF incidence rates per 1,000 person‐years ranging from 0.65 in the no steatosis group to 1.69 in the ALD group. The highest hazard ratio (HR) was 2.416 (95% CI 0.341‐7.167) in the specific etiology SLD group. Postmenopausal women had higher baseline AF incidences, from 3.43 to 5.44 in the MASLD with combined etiologies group, where the highest HR was 1.456 (95% CI 1.371‐1.546) (**Figure**). This trend was consistent across subgroups defined by age, parity, breastfeeding, and use of oral contraceptives.

**Conclusions:** AF risk is notably associated with steatotic liver disease status, more pronounced among premenopausal women. These findings underscore the necessity for targeted cardiovascular monitoring and intervention in women with these liver and menopause conditions.

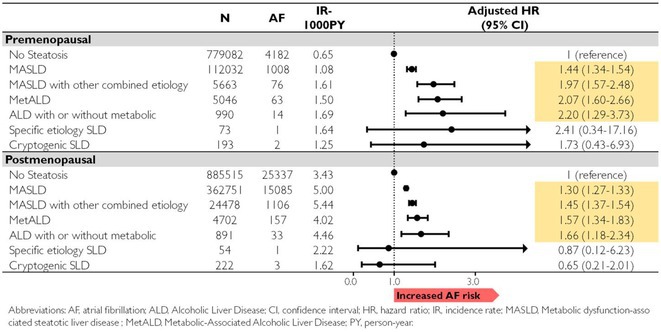

HEART RATE IN ATRIAL FIBRILLATION AND HEART FAILURE WITH PRESERVED EJECTION FRACTION

**MINSOO AHN**

Wonju College of Medicine Yonsei University, Wonju, Korea, Republic of

**Introduction:** Atrial fibrillation (AF) and heart failure with preserved ejection fraction (HFpEF) are frequently associated and can be caused or exacerbated by each other through different mechanisms. The prognostic role and target heat rate of heart rate (HR) in HFpEF with AF is less well known. Therefore, we assessed the association between heart at discharge and prognosis in admitted patients with HFpEF with AF.

**Methods:** A total of 5625 patients hospitalized for acute HF were enrolled from the Korean Acute Heart Failure Registry, of whom 687 with HFpEF and AF were selected. The patients were divided into tertiles based on their heart rate at discharge (T1 ≤69, T2 70‐80, T3 ≥81 bpm).

**Results:** Baseline characteristics were not different. Ejection fraction and brain natriuretic peptide level was not different among 3 groups. LA diameter was significantly larger in lowest heart group(T1; 56.3 ± 6.2. T2; 53.6 ± 10.9, T3; 51.7 ± 11.6, P<0.001). Kaplan‐Meier (KM) curve for 60‐day rehospitalization showed better survival in T1 group. However, it is not statistically significant. KM curve for 60‐day mortality showed no difference according to discharge heart rate.

**Conclusions:** In this study analyzing patients admitted with heart failure with preserved ejection fraction and atrial fibrillation, the analysis of discharge heart rate and prognosis revealed a trend towards lower 60‐day readmission rates for patients with lowest discharge heart rates, although this trend did not reach statistical significance.

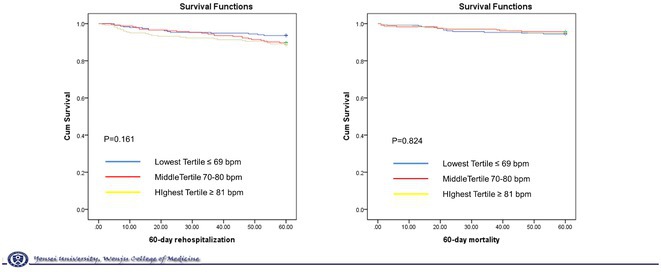

## LEAD POSITIONING IN PERMANENT PACEMAKER IMPLANTATION CAUSING PRESSURE GRADIENT ELEVATION AND TRICUSPID REGURGITATION PROGRESSION : WHAT ARE THE ODDS?

### **ADRIANUS AKBAR**, AGUNG PRASETYO WICAKSONO, FERA HIDAYATI, ERIKA MAHARANI, LUCIA KRIS DINARTI

#### Universitas Gadjah mada, Sleman, Yogyakarta, Indonesia

**Introduction:** Despite its advantages to improve lifespan and quality of life, tricuspid regurgitation (TR) might result from the implantation of a permanent pacemaker (PPM). Previous studies showed that lead impingement is the most frequent mechanism inducing pacemaker‐mediated TR, which is frequently observed and is linked to a higher risk of death in patients with chronic heart failure (HF). The purpose of this study is to assess the relationship between the development of post‐implant TR and TR pressure gradient (PG) in patients who do not currently have TR.

**Methods:** The study included 58 patients from the ALEKA registry who had single ventricular or dual chamber transvenous PPM implantation at Dr. Sardjito Hospital Yogyakarta between November 2022 and August 2023. Patients with previously abnormal TR and TRPG were excluded. Two dimension echocardiography was used to measure new TR and TRPG development prior and after PPM implantation.

**Results:** Dual chamber lead PPM was implanted in 39 patients (67.2%) which were evaluated with a mean of 8 months. Out of all patients who had PPM implants, eleven (18.9%) patients acquired new TR from which 9 (81.8%) patients were implanted with dual chamber lead PPM. Six (54.5%) patients whose ventricular lead was placed in the mid or outflowseptal region tend to have higher TRPG compared to those placed in the low septal area (30.49 ± 0.19 mmHg vs 15.18 ± 1.07 mmHg; p = 0.006).

**Conclusions:** Following transvenous ventricular‐based PPM implantation, about 18.9% of the patients had new TR. In accordance to this study, TR development and TRPG may be affected by the lead's site in the right ventricle.

## DIAGNOSTIC YIELD AND APPROPRIATENESS OF AMBULATORY ECG HOLTER MONITOR AT SULTAN QABOOS UNIVERSITY HOSPITAL

### **MOHAMED AL RAWAHI** ^1^, MOHAMMED AL HABSI^1^, ADIL AL RIYAMI^1^, MUHAMMAD ATHAR SADIQ^1^, SUNIL NADAR^2^

#### ^1^Sultan Qaboos University Hospital, Muscat, Oman,^2^Dudley group of Hospitals NHS trust, Dudley, United Kingdom

**Introduction:** Ambulatory Holter monitoring, pioneered by Norman J. Holter in the 1940s, revolutionized remote ECG monitoring for suspected cardiac arrhythmias. The choice of monitoring modality is guided by the symptomatology and suspicion of life‐threatening arrhythmias. This study evaluates the diagnostic efficacy of 24‐hour and 48‐hour ambulatory ECG Holter monitoring at Sultan Qaboos University Hospital (SQUH), while also assessing the appropriateness of monitoring requests in accordance with the 2017 ISHNE and HRS guidelines.

**Methods:** This retrospective study included all ambulatory ECG Holters performed at SQUH between April 2021 and April 2023. Data on patient demographics, cardiac risk factors, indication for the test, ordering specialty, and the findings of the Holter tests were collected.

**Results:** A total of 1050 patients were included in the analysis, with a mean age of 51.7 ± 19.04 years. The study population comprised 537 females (51.2%). The prevalence of cardiac risk factors in the study cohort was as follows: hypertension 44.2%, diabetes mellitus 32.9%, coronary artery disease 19.4%, cerebrovascular disease 6.7%, dyslipidemia 19.5%, heart failure 5.6%, and AF 5.2%.The majority of indications were deemed appropriate (90.6%) based on the 2017 ISHNE‐HRS expert consensus statement on ambulatory ECG and external cardiac monitoring. Inpatient indications accounted for 52.6% of all tests. Among Holters ordered with an indication of stroke, 14 out of 267 had pre‐existing AF, and AF was detected in 5 additional patients without pre‐existing AF. Comparisons between appropriate and inappropriate indications showed significant differences in age, ordering specialty, indication for the Holter, and patient setting (p < 0.05).

**Conclusions:** This study demonstrates the value of ambulatory ECG Holter monitoring in the management of patients with suspected cardiac arrhythmias. The high rate of appropriate indications based on international guidelines highlights the importance of using evidence‐based criteria in ordering diagnostic tests as practiced in our University Hospital.

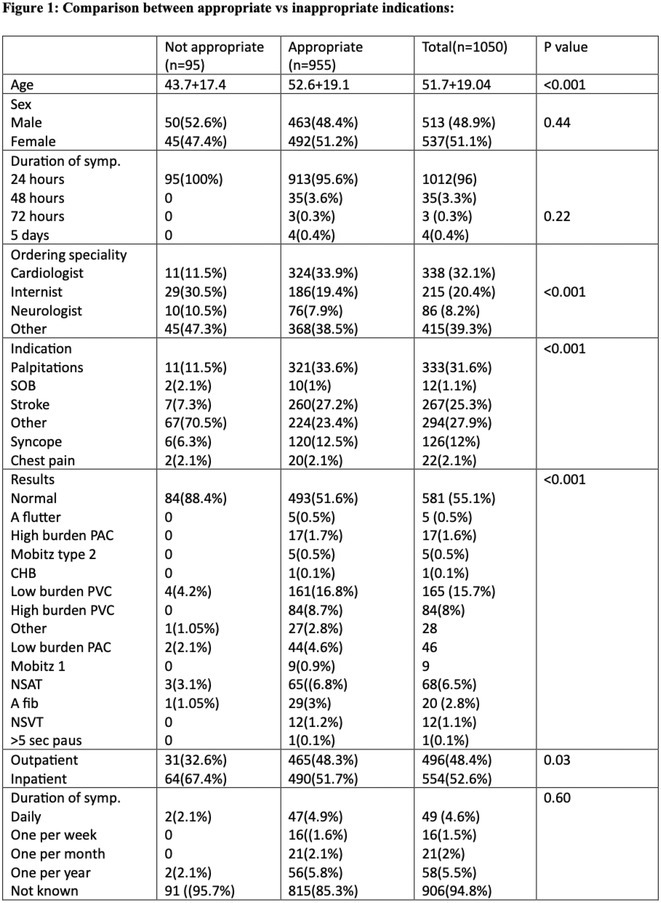

## NAVIGATING RECURRENT ICD SHOCKS CHALLENGES AND THERAPEUTIC INTERVENTIONS

### **MOHAMED AL RAWAHI** ^1^, ISMAIL AL ABRI^2^, GHALIB AL HINAI^2^, NAJIB AL RAWAHI^2^

#### ^1^Sultan Qaboos University Hospital, Muscat, Oman,^2^National Heart Center, Royal Hospital, Muscat, Oman

**Introduction:** A 39‐year‐old female with a history of successful resuscitation of cardiac arrest in 2019 was found to have hypertrophic cardiomyopathy. She received a secondary prevention single chamber Medtronic Mirro ICD. She is known to have hypertension and end‐stage kidney disease on intermittent hemodialysis. She presented to the emergency room in December 2021 with recurrent ICD shocks.

**Methods:** NA

**Results:** The patient was hemodynamically stable. The ECG showed sinus tachycardia at 110 bpm. Telemetry showed sinus tachycardia while she was shocked. A magnet was applied immediately over the pulse generator, and the ICD shocks stopped. A chest X‐ray showed no obvious lead fracture. Transthoracic echocardiography showed a left ventricular ejection fraction of 45% with apical hypertrophy. She was admitted to the coronary care unit. ICD interrogation showed 16 ICD shocks for T wave oversensing [TWOS]. Figure 1. These events occurred in a span of 2 hours. We report a challenging case of TWOS secondary to hypertrophic cardiomyopathy. Since the Medtronic Mirro ICD has no T‐wave discrimination algorithms, the treatment options were very limited with the current device. Due to the presence of an AV fistula on the right forearm and blocked venous access in the left venous system, the viable options were very limited. Changing the pulse generator with a more advanced pulse generator with various T‐ wave oversensing algorithms was our first option. If failed, subcutaneous ICD implantation was the alternative possibility. SJM Fortify Assura VR ICD pulse generator was implanted. At baseline, there was TWOS noted. SenseAbility Settings were modified, as shown in Figure 2. TWOS at baseline was eliminated. Isoprenaline was given to accelerate the heart rate which showed no TWOS.DFT was done, which showed successful detection & defibrillation of VF with the new sensitivity threshold.

**Conclusions:** The patient was regularly monitored in the arrhythmia clinic at six‐month intervals and was in stable condition. During these follow‐up visits, device interrogation consistently revealed no ICD shocks.

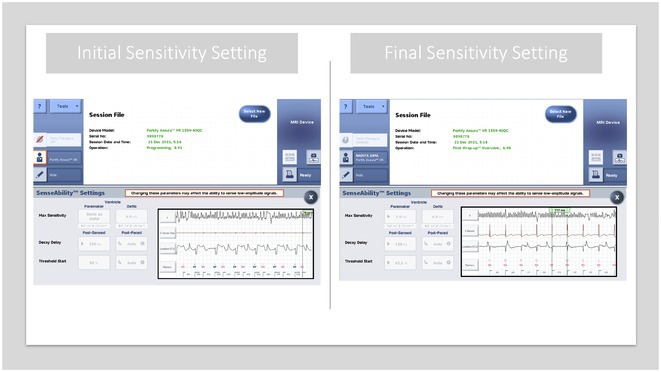

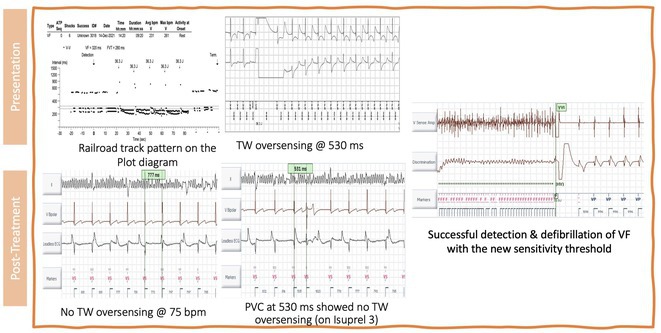

## UNEXPECTED CULPRIT VENTRICULAR FIBRILLATION TRIGGERED BY ICD PROGRAMMING

### **MOHAMED AL RAWAHI** ^1^, KHALID EL SHARNOUBY^2^

#### ^1^Sultan Qaboos University Hospital, Muscat, Oman,^2^National Heart Center, Royal Hospital, Muscat, Oman

**Introduction:** A 25‐year‐old male with a history of successful resuscitation of cardiac arrest in 2015 was found to have an anomalous origin of the right coronary artery from the left coronary cusp with an inter‐arterial course. Evidence of left ventricular non‐compaction was also noted on cardiac imaging. He underwent surgical correction of the anomalous coronary artery and received a secondary prevention single chamber Medtronic implantable cardioverter defibrillator (ICD). Over the years, he developed atrial fibrillation (AF) and underwent AF ablation in 2017. He also had bradycardia‐induced ventricular fibrillation (VF), leading to an upgrade to a dual‐chamber ICD in October 2017. He presented to the emergency room in April 2021 with recurrent ICD shocks.

**Methods:** NA

**Results:** The patient was hemodynamically stable. 12‐lead ECG, Echocardiography, CXR and blood work were all normal. He was admitted to the coronary care unit. Amiodarone was started. ICD interrogation showed four episodes of VF successfully terminated by a single ICD shock for each VF event. All 4 events were triggered by MVP mode programming, as shown in figure 1. These events occurred in a span of 24 hours.

**Conclusions:** We report a rare cause of recurrent VF that is triggered by the ICD programming. The ICD was programmed AAIR‐DDDR (MVP mode) to minimize right ventricular pacing and promote intrinsic conduction. In this patient, all 4 episodes of VF were triggered by the backup pacing after the dropped V sense beat. Reprogramming the device to DDDR mode resulted in no shocks during admission. Amiodarone was discontinued, and the patient was discharged after 48 hours. The patient was regularly monitored in the arrhythmia clinic at six‐month intervals and was in stable condition. During these follow‐up visits, device interrogation consistently revealed no ventricular events or ICD shocks. The patient approaches the completion of a three‐year period free from ventricular arrhythmias or ICD shocks; it is notable that he remains off anti‐arrhythmic medications, indicating a sustained and favourable clinical course.

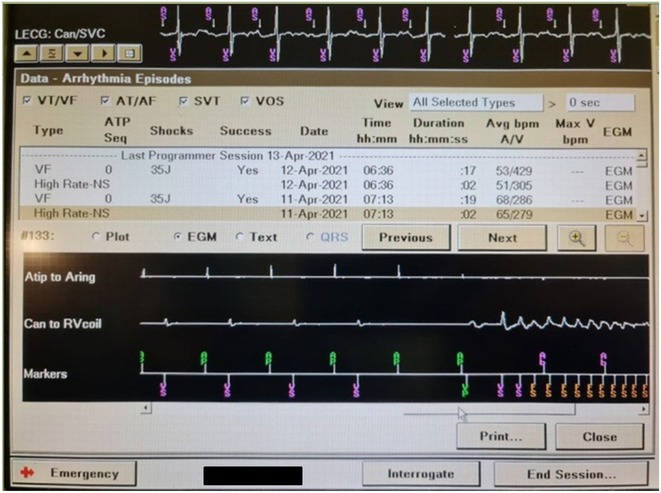

## CORRECTING QT INTERVAL AFTER CARDIAC RESYNCHRONIZATION THERAPY

### **MOHAMMAD ALASTI** ^1,2^, AMIN ESMAILIAN^2^, COLIN MACHADO^1^, JEFFREY ALISON^1^

#### ^1^Monash Health, Clayton, Australia,^2^Monash University, Clayton, Australia

**Introduction:** This study investigates various formulae utilized for correcting the QT interval in individuals with broad QRS complexes to calculate QTc following cardiac resynchronization therapy (CRT).

**Methods:** Patients with advanced heart failure and left bundle branch block (LBBB) pattern, with a QRS duration of at least 120 milliseconds, who underwent successful CRT implantation were included. Patients with LV leads in non‐lateral veins, metabolic disorders, atrial fibrillation (AF), atrial tachycardia, or high‐degree atrioventricular (AV) block rhythms pre‐implantation were excluded. Pre‐ and post‐implant QT intervals were measured and corrected for QRS duration and heart rate using the Boggosian, Wand, Tang & Rabkin, Bazett, Framingham, and Fredericia formulae.

**Results:** Among the patients who underwentCRT, 51 met the criteria. QRS duration significantly decreased from 189.68 ±18.06 ms to 165.25 ± 18.78 ms, while QT corrected with Bazett and Fredericiaformulae did not exhibit any significant change (522.06 ± 30 ms versus 524.06 ±36.52 ms). Amongdifferent formulae, only using the Fredericiaformula for heartrate correction followed by the Tang andRabkin formula,showed relatively similar pre‐ and post‐CRT implant QTc intervals (437.57±49.99ms versus 436.38± 36.91ms).

**Conclusions:** Our datasuggest that employing the Tang andRabkin formula (0.945 QTc− 26) after QTcorrection with the formula Fredericia(QTc: QT/cycle length^1/3^) may be recommended.

## CORRELATION BETWEEN QRS DURATION CHANGES AND PLASMA FLECAINIDE LEVELS IN PAEDIATRIC PATIENTS

### **MUSAB AL‐ESSA** ^1^, FATME CHARAFEDDINE^2^, ANDREAS PFLAUMER^3^, ANDREW DAVIS^3^

#### ^1^Royal Children's Hospital, Melbourne, Australia,^2^American university of Beirut, Beirut, Lebanon,^3^Royal children's hospital, Parkville, Australia

**Introduction:** Flecainide has a narrow therapeutic index and has the highest number of adverse events for any paediatric anti‐arrhythmic drug. Safe usage requires careful ECG monitoring. Flecainide levels are frequently used as complimentary information to optimise safe drug dosing. European guidelines recommend careful consideration of a decrease in dose or discontinuation of flecainide when there is an increase in QRS duration of 25% from baseline. Previous studies in adults have shown a correlation between serum flecainide concentration and QRS prolongation. Data in paediatric populations is lacking.

**Methods:** After multiple exclusions, 16 patients (aged 18 days to 20.5 years) who initiated on flecainide treatment at the Royal Children's Hospital, had 54 plasma levels from 2001 to 2018. Exclusion criteria were absence of a baseline ECG preceding therapy, concurrent administration of other antiarrhythmic drugs, presence of Wolff‐Parkinson‐White syndrome, cardiomyopathy, congenital heart disease, ventricular tachycardia (except CPVT) and myocarditis. Flecainide level was performed at St Vincent's Hospital Melbourne using a HPLC assay. ECGs were measured in V5 and changes and expressed as a ratio of QRS post‐treatment /QRS pre‐treatment.

**Results:** The research uncovered a direct relationship between flecainide levels and QRS ratio. Results featured a significant regression line (R squared 0.38; p <0.0001) and a box‐whisker plot portraying data distribution between the 5th and 95th centiles.

**Conclusions:** A positive correlation exists between flecainide level and QRS ratio. More data to define this relationship may prove useful to decrease the need for level monitoring.

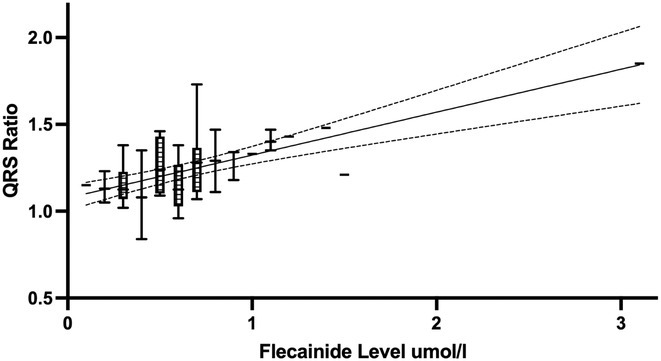

## ADVANCED ECG HEART AGE: A PROGNOSTIC, EXPLAINABLE MACHINE LEARNING APPROACH APPLICABLE TO SINUS AND NON‐SINUS RHYTHMS

### **ZAIDON AL‐FALAHI** ^1^, TODD T SCHLEGEL^2,3^, ISRAEL LAMELA‐PALENCIA^1^, ANNIE LI^1^, ERIK B SCHELBERT^4^, LOUISE NIKLASSON^4^, MAREN MAANJA^2^, THOMAS LINDOW^1,5^, MARTIN UGANDER^1,2^

#### ^1^Kolling Institute, Royal North Shore Hospital, and University of Sydney, Sydney, Australia,^2^Department of Clinical Physiology, Karolinska University Hospital, and Karolinska Institutet, Stockholm, Sweden,^3^Nicollier‐Schlegel SARL, Trélex, Switzerland,^4^Minneapolis Heart Institute East, United Hospital, Minneapolis, MN,^5^Clinical Physiology, Clinical Sciences, Lund University, Lund, Sweden

**Introduction:** An explainable advanced electrocardiography (A‐ECG) Heart Age gap is the difference between A‐ECG Heart Age and chronological age. This gap is an estimate of accelerated cardiovascular ageing expressed in years of healthy human aging, and can intuitively communicate cardiovascular risk to the general population. However, existing A‐ECG Heart Age requires sinus rhythm. **Aims**: To develop and prognostically validate a revised, explainable A‐ECG Heart Age applicable to both sinus and non‐sinus rhythms.

**Methods:** An A‐ECG Heart Age excluding P‐wave measures was derived from the 10‐second 12‐lead ECG in a derivation cohort using multivariable regression machine learning with Bayesian 5‐minute 12‐lead A‐ECG Heart Age as reference. The Heart Age was externally validated in a separate cohort of patients referred for cardiovascular magnetic resonance imaging by describing its association with heart failure hospitalization or death using Cox regression, and its association with comorbidities.

**Results:** In the derivation cohort (n=2771), A‐ECG Heart Age agreed with the 5‐min Heart Age (R^2^=0.91, bias 0.0±6.7 years), andincreased with increasing co‐morbidity. In the validation cohort (n=731, mean age 54±15 years, 43% female, n=139 events over 5.7 [4.8‐6.7] years follow‐up), increased A‐ECG Heart Age gap (≥10 years) associated with events (hazard ratio [95% confidence interval] 2.04 [1.38‐3.00], C‐statistic 0.58 [0.54‐0.62], and the presence of hypertension, diabetes mellitus, hypercholesterolemia, and heart failure (p≤0.009 for all).

**Conclusions:** An explainable A‐ECG Heart Age gap applicable to both sinus and non‐sinus rhythm associates with cardiovascular risk, cardiovascular morbidity, and survival.

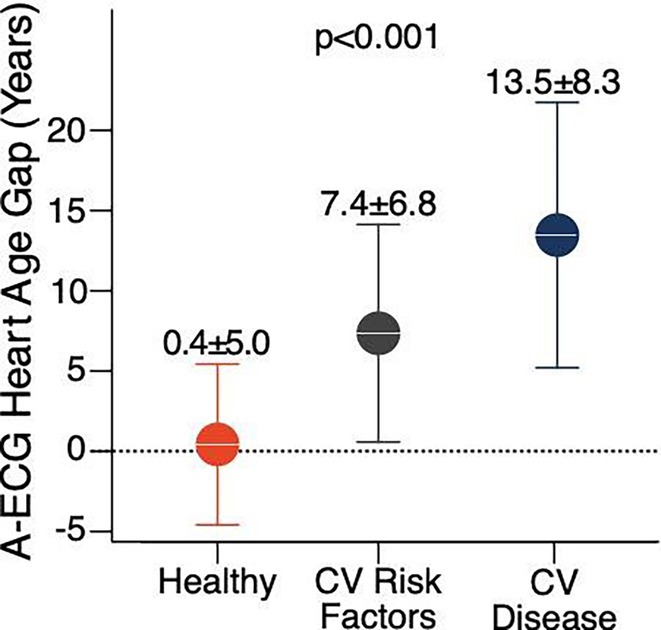

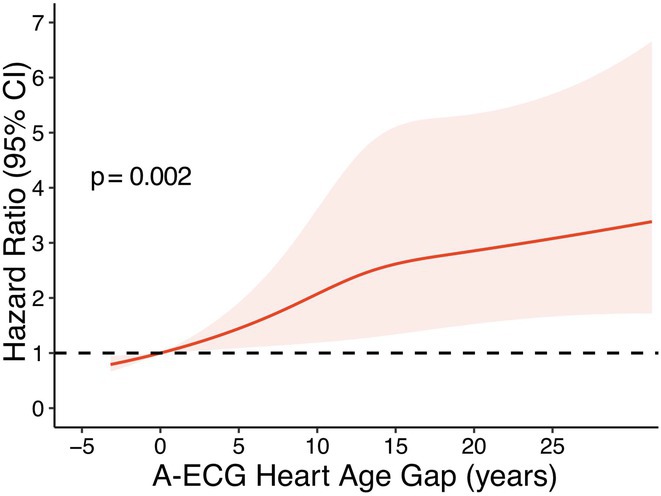

## CLINICAL PROFILE OF CARDIAC IMPLANTABLE ELECTRONIC DEVICE INFECTION IN BANGLADESH

### MD ALI

#### Evercare Hospital Dhaka Bangladesh, Dhaka, Bangladesh

**Introduction:** Bangladesh is a south Asian country, hosts about 160 million population. There are about 35 cardiac centres where cardiac implantable electronic devices (CIED) implantation can be done. Per year new implantation rate is about 2500 to 3000.Rate of implantation is increasing steadily. CIED includes pacemaker, Implantable cardiverter‐defibrillator (ICD) and cardiac resynchronization therapy (CRT). Access to therapy for CIED infection is difficult due to limited resources.

**Methods:** This is a single centre observation at Evercare hospital, a tertiary multidisciplinary hospital. Retrospective analysis was done from hospital records.

**Results:** Study included 35 cases of CIED infection during the year 2021 to 2023. All cases had CIED pocket infection. Cases were referred for further management. Study had 31 pacemaker, 3 CRT and 1 ICD cases. Age range from 32 to 76 years. The study consisted of 28 male and 7 female patients. Symptoms were, swelling in 5 cases, discharge in 15 cases, erosion and perforation in 15 cases. Before attending this centre, all patients received more than 2 courses of 10 days antibiotics and 6 cases received surgical dressings and repositioning at same pockets by plastic surgeons without useful results. Pocket fluid or discharge were cultured for common bacteria. Culture were negative in 27 cases. 8 cases were positive culture for Staph aureus and staph epidermidis. Results of treatment given at this centre were; 2 patients died from endocarditis and 33 cases received new implantation. Before new implantation the whole systems were explanted using device explanation tools in 8 cases and without tools in 26 cases. Resterilized devices were used in 3 cases and new devices used in 30 cases.

**Conclusions:** The results do not reflect the true national infection rate. After infection is diagnosed both the patients and physicians took longer time before going for extraction. Whole system extraction facilities are very limited in this country and for this reason only generator extraction are done by many implanters, keeping the lead in situ, leading the whole management procedure more difficult. Because of financial reason devices are reused in many cases.

## ABLATION OF AVNRT AND AT IN CCTGA WITH LARGE OS ASD

### SURESH ALLAMSETTY

#### Medicover Hospitals Visakhapatnam, Visakhapatnam, India

**Introduction:** Congenital transposition of Great arteries (CCTGA/ LTGA) is characterized by atrio ventricular (AV) discordance and ventriculoarterial (VA) discordance. CCTGA may be associated with many arrhythmias. However AV nodal reentrant tachycardia is rare. We present a patient with aforementioned Congenital heart disease (CHD) having AVNRT and Atrial tachycardia (AT).

**Methods:** N/A

**Results:** A 38 years old lady presented with complaints of palpitations off and on since 10 years. She is known case of CHD, CCTGA with large ostium secundum Atrial septal defect. She underwent ablation elsewhere in past and is on antiarrhythmics since then. She had recurrent palpitations in last 6 months requiring multiple hospitalizations for Supraventricular tachycardia (SVT, Figure 1B). Inj. Adenosine 12 mg IV reverted the SVT to sinus rhythm (Figure 1 A). As she was symptomatic inspite of taking antiarrhythmics, she was taken up for Electrophysiologic study (EPS) after stopping antiarrhythmics for 5 days. EPS induced typical AVNRT (Figure1 C). Radiofrequency ablation (RFA) was done using 4mm tip non irrigated catheter. Slow pathway was ablated with electrogram guided approach at posterior pulmonary cusp and there was junctional rhythm during ablation (Figure 1 D). There was another SVT induced (Figure 1 E) and ventricular overdrive pacing showed VAAV response, suggestive of AT. Activation mapping was done during SVT using HD grid catheter and Ensite Precision 3D Mapping system. It showed a focal AT arising from septum and the earliest site was 49ms before P wave. Ablation at the site terminated the AT. Post RFA there was neither AVNRT nor AT inducible with programmed atrial & ventricular stimuli under inj. Isoproterenol 4mcg/mt. Two years post RFA patient is asymptomatic and doing well.

**Conclusions:** In CHD multiple arrhythmias may be present and hence meticulous EP study has to be done. Slow pathway ablation in CCTGA is feasible in posterior aspect of pulmonary artery cusp. Ablation of SVT is feasible, safe and effective in CCTGA with the use of electroanatomic mapping.

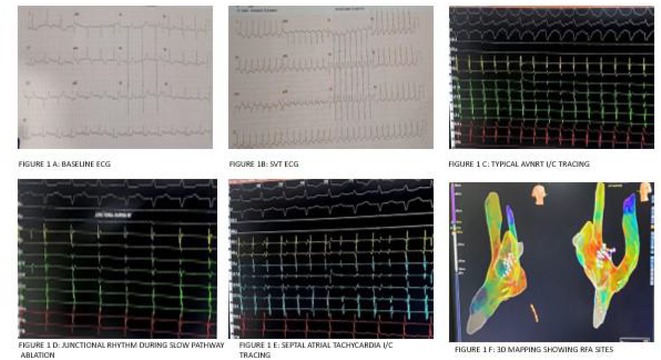

## MARKED FIRST DEGREE AV BLOCK WITH SYNCOPE; IS PACEMAKER IMPLANTATION MANDATORY?

### **SURESH ALLAMSETTY**, ARCHANA BEHERA

#### Medicover Hospitals Visakhapatnam, Visakhapatnam, India

**Introduction:** Marked first degree AV Block (PR >300ms) with syncope is a Class IIa indication for dual chamber pacemaker implantation. We describe a patient where the patient had aforementioned condition and was evaluated meticulously and managed conservatively.

**Methods:** N/A

**Results:** A 20 years old female, Nurse by profession, presented with shortness of breath and easy fatiguability class II since 1 year. She had three episodes of syncope all during prolonged standing in 1 month. Baseline ECG ( Figure 1 A) is suggestive of PR interval of 310ms. 2D Echo revealed she had structurally normal Heart. TMT was done and she could achieve 76% of her target heart rate. Holter monitoring showed that she had only marked first degree AV Block and there was neither significant sinus pauses nor high degree AV block. She underwent Cardiac PET CT scan to rule out Sarcoidosis or any other infectious/ inflammatory conditions. Biochemical investigations revealed that she had Iron deficiency anemia and Iron supplements were given. Electrophysiology study was done which showed that she had dual AV node physiology, normal SA node and AV node conduction. Baseline EP parameters are shown in Figure 1 B. AV one to one was 330ms. There was no SVT induced. She underwent Head up tilt table test(HUTT). During passive stage there was no bradycardia (Figure 1 C) or hypotensive response. After 15 minutes of sublingual Nitrate patient i.e. active stage of HUTT, she developed syncope and there was bradycardia, heart rate was 44/minute ( Figure 1 D), ECG showed 2 to 1 AV block and there was vasodepressor response. Tilt table test established the diagnosis of Vasovagal syncope. Patient was started on Tab Midodrine 2.5mg twice daily and was given all non pharmacological advise. Patient is on regular follow up and is totally asymptomatic.

**Conclusions:** Symptomatic marked first degree AV block especially young, as in our patient must be evaluated with tilt table test to confirm the diagnosis of Vasovagal syncope. Pacemaker implantation in this patient would not have benefitted as she had Vaso depressive response in HUTT. However closer monitoring is required in these subset of patients.

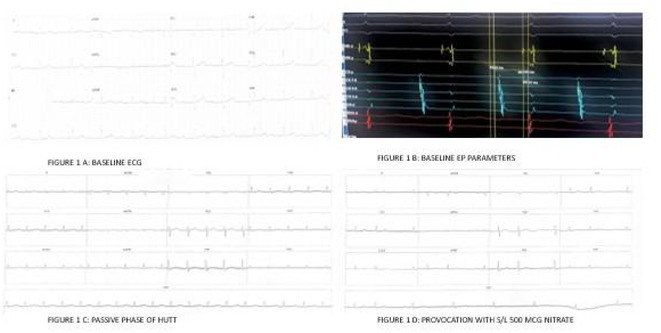

## VPC UNVEIL ALCAPA; AF WORSENS HEART FAILURE: SAGA OF MANAGEMENT

### **SURESH ALLAMSETTY** ^1^, JAIDEEP TRIVEDI^2^

#### ^1^Medicover Hospital, MVP Branch, Visakhapatnam, India,^2^Apollo Hospital, Visakhapatnam, India

**Introduction:** Anomalous Left coronary artery from pulmonary artery (ALCAPA) is a rare congenital coronary artery anomaly and accounts for 0.25‐0.5% of all congenital heart diseases. There are two forms based on the onset of disease viz. Infantile and adult type. We describe an adult type of ALCAPA, where ventricular arrhythmia unveiled the rare diagnosis.

**Methods:** N/A

**Results:** A 36 years old gentleman presented with recurrent palpitations with presyncope off and on of 3 months duration in May 2018. He underwent Mitral valve replacement in 2016 for severe Mitral regurgitation (MR) which was probably thought of Rheumatic origin, as it remains a major public health problem in India. His Electrocardiogram showed LBBB with ventricular premature complexes(VPCs) (Figure 1 A). 2D Echo was suggestive of global hypokinesia of LV, normally functioning prosthetic mitral valve and severe LV dysfunction (EF 30%). He underwent Coronary angiogram which revealed ALCAPA and ectatic right coronary artery and the same was also documented by doing CT Coronary Angiogram (Figure 1 B). He underwent definitive ALCAPA repair. Post Surgery, there were no VPCs (Figure 1 C). He symptomatically improved. He had COVID 19 Pneumonia in 2021 and then he developed Atrial Fibrillation. He had multiple hospital admissions for Heart failure and AF with fast ventricular rates since then. He had severe LV EF of 20%. He was symptomatic (NYHA class III‐IV) inspite of taking guideline directed medical therapy. He underwent CRT‐D implantation (Figure 1 E) and AV nodal ablation. Post CRT‐D implantation his LV EF was 37%. His ECG showed biventricular pacing ( Figure 1 F). He symptomatically improved.

**Conclusions:** This case brings to light a rare congenital coronary anomaly which was investigated due to VPCs and the diagnosis of ALCAPA was unveiled. In young patient with MR with LV dysfunction, broader look into etiologies should be sought. AF was another arrhythmia which worsened HF which developed during further course, which was eventually managed successfully with AV node ablation and CRT‐D implantation.

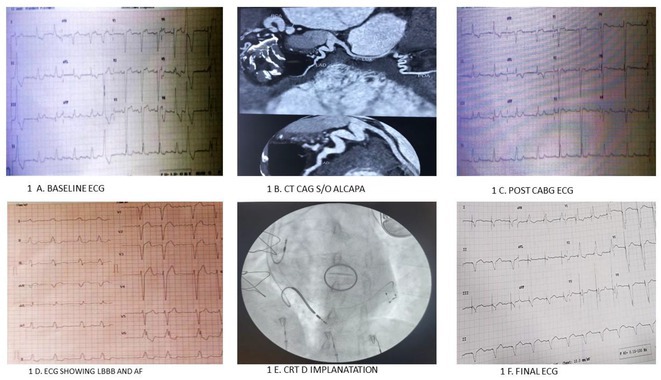

## A NOVEL MULTISTEP ALGORITHM FOR PREDICTING VENTRICULAR ARRHYTHMIAS ORIGINATING FROM THE RIGHT VENTRICULAR OUTFLOW TRACT WITH A LEFT BUNDLE BRANCH BLOCK PATTERN AND INFERIOR AXIS

### **MUHAMMAD RAFDI AMADIS** ^1^, SIMON SALIM^2^, LI‐WEI LO^3^, MUHAMMAD YAMIN^2^, YENN‐JIANG LIN^3^, SHIH‐LIN CHANG^3^, YU‐FENG HU^3^, FA‐PO CHUNG^3^, RUBIANA SUKARDI^4^, CHIN‐YU LIN^3^, TING‐YUNG CHANG^3^, LING KUO^3^, ANGGA PRAMUDITA PUDIANTO^2^, CHIH‐MIN LIU^3^, SHIN‐HUEI LIU^3^, CHENG‐I WU^3^, YU‐SHAN HUANG^5^, DINH SON NGOC NGUYEN^6^, DAT CAO TRAN^7^, SHIH‐ANN CHEN^8^

#### ^1^Heart Rhythm Center, Division of Cardiology, Department of Medicine, Taipei Veterans General Hospital, Taipei, Taiwan ‐ Department of Cardiology and Vascular Medicine Universitas Airlangga, Surabaya, Indonesia,^2^Cardiology Division, Department of Internal Medicine. Dr. Cipto Mangunkusumo National General Hospital ‐ Faculty of Medicine Universitas Indonesia, Jakarta, Indonesia,^3^Heart Rhythm Center, Division of Cardiology, Department of Medicine, Taipei Veterans General Hospital ‐ Department of Medicine, National Yang Ming Chiao Tung University, Taipei, Taiwan,^4^Cardiology Division, Department of Child Health. Dr. Cipto Mangunkusumo National General Hospital ‐ Faculty of Medicine Universitas Indonesia, Jakarta, Indonesia,^5^Heart Rhythm Center, Division of Cardiology, Department of Medicine, Taipei Veterans General Hospital, Taipei, Taiwan,^6^Heart Rhythm Center, Division of Cardiology, Department of Medicine, Taipei Veterans General Hospital ‐ University Medical Center, Ho Chi Minh City, Viet Nam,^7^Heart Rhythm Center, Division of Cardiology, Department of Medicine, Taipei Veterans General Hospital ‐ Cho Ray Hospital, Ho Chi Minh City, Viet Nam,^8^Cardiovascular Center, Taichung Veterans General Hospital ‐ National Chung Hsing University, Taichung, Taiwan

**Introduction:** Numerous criteria have been established for predicting premature ventricular contractions (PVC) originating from the right ventricular outflow tract (RVOT).

**Methods:** We hypothesized and validated a novel multistep algorithm to differentiate PVC within the RVOT and the non‐RVOT. We formulated an algorithm using data from 65 patients with PVC characterized by a left bundle branch block (LBBB) pattern with an inferior axis underwent ablation at Cipto Mangunkusumo National General Hospital, Indonesia. Diagnostic accuracy was assessed through scrutiny of nine criteria: 1) earliest onset of QRS or peak in V_2_; 2) V_1_ R‐wave duration index and R/S‐wave amplitude index; 3) S‐R amplitude difference in V_1_ through V_2_; 4) V_3_ R‐wave deflection interval and V_1_ R‐wave amplitude; 5) V_2_ transition ratio; 6) Transition zone index; 7) V_2_S/V_3_R index; 8) V_2_QRS_i40_; 9) combination index. Subsequently, we validated it from a second cohort (n=291) underwent ablation at Taipei Veterans General Hospital, Taiwan.

**Results:** Our multistep algorithm incorporates criteria 5, 8, and 1 to enhance overall diagnostic performance. The AUC, accuracy, sensitivity, specifity, PPV, and NPV was 0.802, 86.2%, 93.6%, 55.7%, 88%, and 80% respectively. Upon validation in the cohort (Figure 1), the multistep algorithm demonstrated an overall AUC of 0.775, accuracy of 85.9%, sensitivity of 90.8%, specificity of 64.2%, PPV of 91.9%, and NPV of 60.7%, indicating a good discriminatory value for the multistep algorithm.

**Conclusions:** The introduction of this novel multistep algorithm improved the accuracy in predicting the RVOT origin of the PVC when compared to reliance on a single criterion.

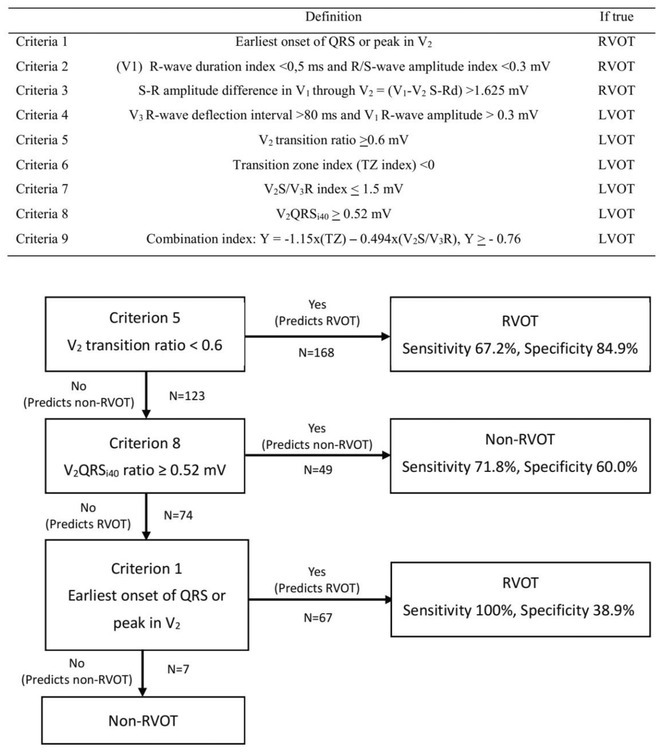

## NOVEL TECHNIQUE FOR ABLATION OF PREMATURE VENTRICULAR CONTRACTION ORIGINATING FROM MODERATOR BAND BODY USING POINT‐TO‐POINT DISTANCE

### **MUHAMMAD RAFDI AMADIS** ^1^, CHIN‐YU LIN^2^

#### ^1^Heart Rhythm Center, Division of Cardiology, Department of Medicine, Taipei Veterans General Hospital, Taipei, Taiwan ‐ Department of Cardiology and Vascular Medicine Universitas Airlangga, Surabaya, Indonesia,^2^Heart Rhythm Center, Division of Cardiology, Department of Medicine, Taipei Veterans General Hospital ‐ Department of Medicine, National Yang Ming Chiao Tung University, Taipei, Taiwan

**Introduction:** Ablation of premature ventricular contraction (PVC) originating from moderator band (MB) is challenging, as it is an intracavitary structure that is highly variable in anatomy and cannot be visualized by both fluoroscopy and electroanatomic mapping (EAM). Intracardiac echocardiography (ICE) is a useful tool to visualize the structure in real‐time and ensure ablation catheter tip location and contact during the procedure. However, the visibility of intravascular ultrasound was limited, making it difficult to accurately determine the relative position of the ablation catheter. We applied a novel method with point‐to‐point distance to measure the distance of ablation catheter to the earliest activation site (EAS) point to assist ablation.

**Methods:** N/A

**Results:** A 63‐year‐old male complained of recurrent palpitation. Previously, he underwent MB‐PVC ablation at both of free wall (FW) and septal MB insertion site facilitated by ICE. The PVC recurred and the patient underwent redo ablation. During the redo ablation, the local activation time (LAT) mapping at both FW insertion area and septal insertion area only showed the PVC 15 ms earlier than surface ECG. A suspicion of PVC originating from MB body was made. We made an imaginary line between FW and septal insertion of MB, and an attempt was made to map this area. During mapping, the ablation catheter was stuck on an intracavitary structure and spontaneous PVC resulted in even earlier LAT (23 ms) compared with FW and septal insertion area. We tagged the earliest activation site area and used point‐to‐point distance to guide the ablation. Ablation in that area successfully eliminated the PVC. The exact ablation location was confirmed by post‐procedural transthoracal echocardiography that showed hyperechogenicity at the MB body.

**Conclusions:** The use of point‐by‐point distance in EAM could help guiding the ablation PVC originating from body of moderator band.

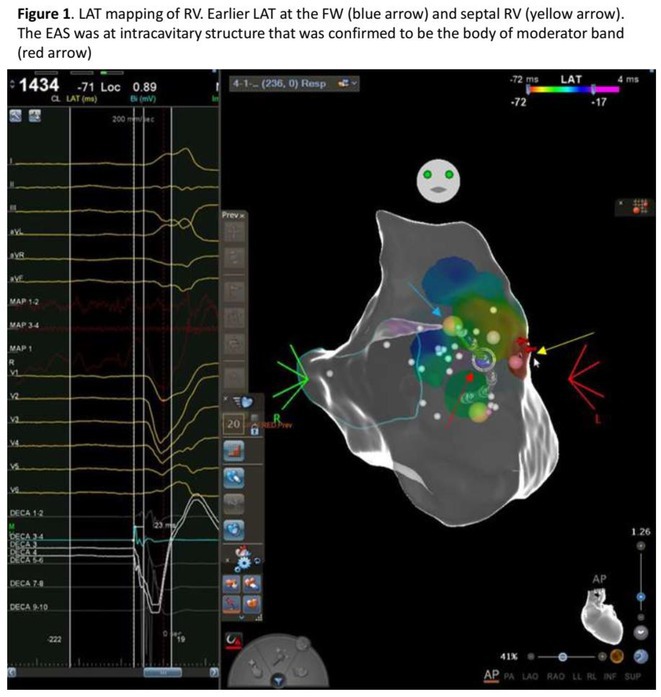

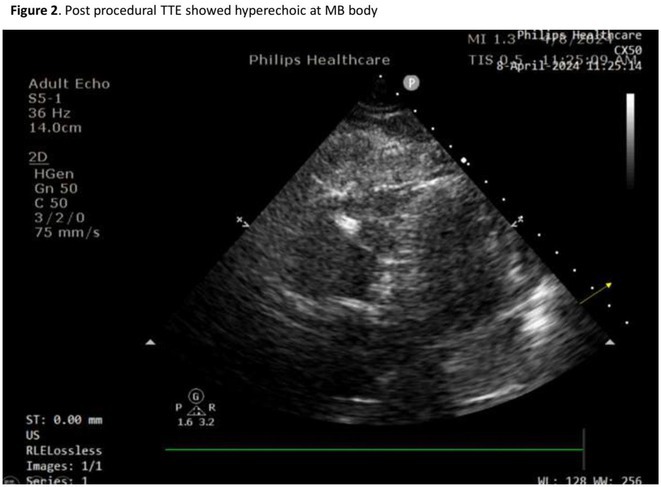

## REAPPRAISAL OF THE CLINICAL CHARACTERISTICS OF IDIOPATHIC OUTFLOW TRACT VENTRICULAR ARRHYTHMIAS WITH AN R WAVE PATTERN BREAK IN PRECORDIAL LEAD: A MULTI‐CENTER STUDY

### **MUHAMMAD RAFDI AMADIS** ^1^, SATOSHI HIGA^2^, CHIN‐YU LIN^3^, YENN‐JIANG LIN^3^, SHIH‐LIN CHANG^3^, LI‐WEI LO^3^, YU‐FENG HU^3^, FA‐PO CHUNG^3^, TING‐YUNG CHANG^3^, LING KUO^3^, CHIH‐MIN LIU^3^, SHIN‐HUEI LIU^3^, CHENG‐I WU^3^, JOSE ANTONIO L BAUTISTA^4^, YU‐SHAN HUANG^5^, BAI SITTI AMEERAH ASLEAH B TAGO^6^, MARIE KIRK PATRICH MARAMARA^7^, CHIAO‐CHIN LEE^8^, WEN‐PO FAN^9^, LO‐CHIEH LING^5^, HENDYONO LIM^10^, YU‐SHAN CHIEN^11^, YUEN HOONG PHANG^12^, HOANG NGUYEN QUOC^13^, SHIH‐ANN CHEN^14^

#### ^1^Heart Rhythm Center, Division of Cardiology, Department of Medicine, Taipei Veterans General Hospital, Taipei, Taiwan ‐ Department of Cardiology and Vascular Medicine Universitas Airlangga, Surabaya, Indonesia,^2^Cardiac Electrophysiology and Pacing Laboratory, Division of Cardiovascular Medicine, Makiminato Central Hospital, Okinawa, Japan,^3^Heart Rhythm Center, Division of Cardiology, Department of Medicine, Taipei Veterans General Hospital ‐ Department of Medicine, National Yang Ming Chiao Tung University, Taipei, Taiwan,^4^Heart Rhythm Center, Division of Cardiology, Department of Medicine, Taipei Veterans General Hospital, Taipei, Taiwan ‐ Section of Clinical Cardiac Electrophysiology, Heart Institute, St. Luke's Medical Center, Global City, Taguig City, Philippines,^5^Heart Rhythm Center, Division of Cardiology, Department of Medicine, Taipei Veterans General Hospital, Taipei, Taiwan,^6^Heart Rhythm Center, Division of Cardiology, Department of Medicine, Taipei Veterans General Hospital, Taipei, Taiwan ‐ Section of Cardiology, Department of Internal Medicine, Amaipakpak Medical Center, Marawi City, Philippines,^7^Heart Rhythm Center, Division of Cardiology, Department of Medicine, Taipei Veterans General Hospital, Taipei, Taiwan‐Division of Cardiovascular Medicine, Section of Electrophysiology and Pacing, University of the Philippines, Philippine General Hospital, Manila, Philippines,^8^Heart Rhythm Center, Division of Cardiology, Department of Medicine, Taipei Veterans General Hospital ‐ Division of Cardiology, Department of Medicine, Tri‐Service General Hospital, Taipei, Taiwan,^9^Heart Rhythm Center, Division of Cardiology, Department of Medicine, Taipei Veterans General Hospital ‐ Division of Pediatric Cardiology, Department of Pediatrics, Taipei Veterans General Hospital, Taipei, Taiwan,^10^Heart Rhythm Center, Division of Cardiology, Department of Medicine, Taipei Veterans General Hospital, Taipei, Taiwan ‐ Cardiovascular Department Universitas Pelita Harapan, Tangerang, Indonesia,^11^Heart Rhythm Center, Division of Cardiology, Department of Medicine, Taipei Veterans General Hospital, Taipei, Taiwan ‐ Cardiovascular Center, Taichung Veterans General Hospital, Taichung, Taiwan,^12^Heart Rhythm Center, Division of Cardiology, Department of Medicine, Taipei Veterans General Hospital, Taipei, Taiwan ‐ Cardiology Department, Hospital Sultanah Bahiyah, Alor Setar, Malaysia,^13^Heart Rhythm Center, Division of Cardiology, Department of Medicine, Taipei Veterans General Hospital, Taipei, Taiwan ‐ Arrhythmia Treatment Department, Cho Ray Hospital, Ho Chi Minh City, Viet Nam,^14^Cardiovascular Center, Taichung Veterans General Hospital ‐ National Chung Hsing University, Taichung, Taiwan

**Introduction:** Idiopathic outflow tract ventricular arrhythmia (OT‐VA) with a pattern break (PB) in precordial lead is considered challenging and associated with a low success rate.

**Methods:** We retrospectively reviewed the electronic medical records of all idiopathic OT‐VA patients who underwent catheter ablation at Taipei Veterans General Hospital, Taiwan and Makiminato Central Hospital, Japan. The included patients had these characteristics: a documented left bundle branch pattern and inferior axis OT‐VA with PB.

**Results:** Among 984 idiopathic OT‐VA patients, 66 patients (6.7%) had a PB in V2 (N=60) or V3 (N=6). The first clinical manifestation was frequent PVC (89.4%), and the remaining presented as VT. The acute success rate and the late success rate after median follow up of 36 months were 92.4% and 78.8%, respectively, which is significantly higher than the previous report (58.3%, p=0.006 for acute success rate, and 41.7%, p=0.013 for late success rate, N=12). The origin of VA with PB were RVOT (88.5%), intramural origin (6.6%) and LVOT (4.9%). In patients with RVOT as the successful ablation site, the subvalvular approach was more common (82.8%) than the supravalvular and both area. The VA origin from LVOT was associated with acute procedural failure compared with the VA origin from RVOT (40% vs 5.3%, p=0.048).

**Conclusions:** OT‐VA with a PB in the Asia population may not have a worse clinical outcome than previously reported in Western countries. The most common acute success site for ablation was RVOT, specifically anterior RVOT.

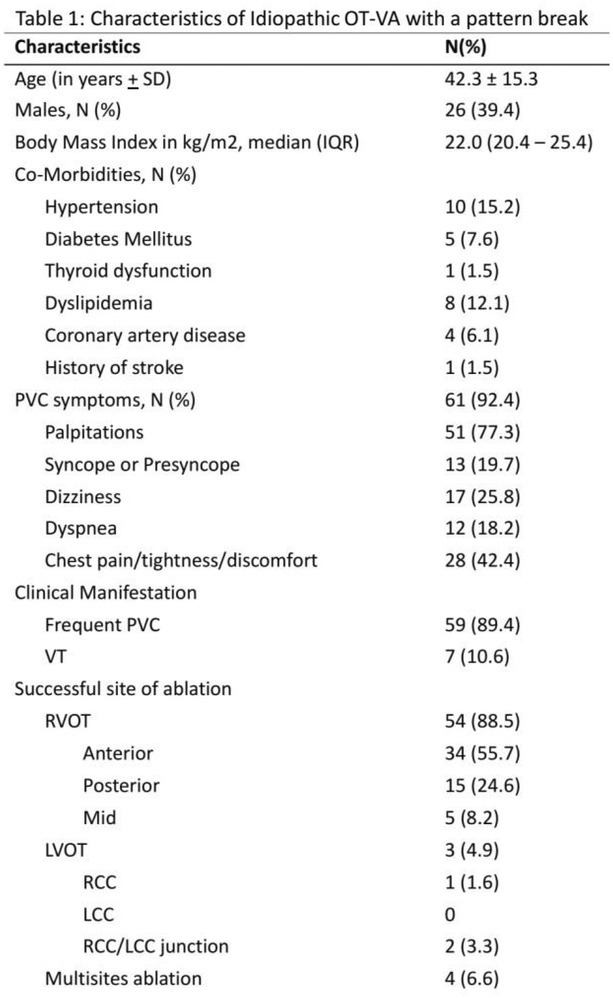

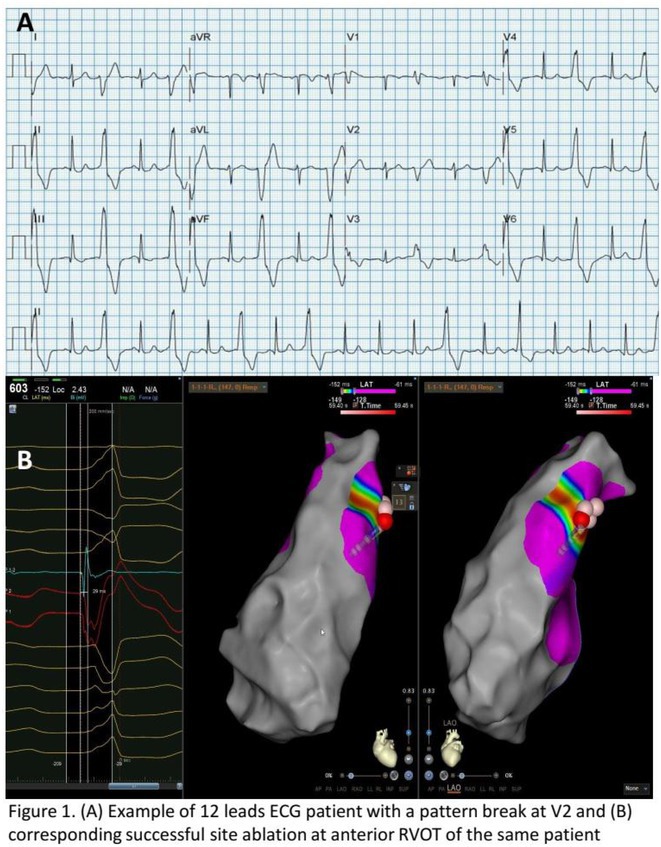

## BRUGADA SYNDROME PRECIPITATED BY AN ANTIMALARIAL AGENT: A CASE REPORT

### **MUZAKKIR AMIR**, IRMAYANTI MUKHTAR, PENDRIK TANDEAN, MUHAMMAD ZAKI RAHMANI

#### Hasanudin University, Makassar, Indonesia

**Introduction:** Cardiovascular events of antimalarial treatment remain unclear, only a few studies has reported its adverse outcome. This case presentation emphasizes cardiological assessment of brugada syndrome, a rare genetic predisposed that manifest as life threatening arrhytmia occurs during routine antimalarial consumption. Without screening and untreated, this disease leads to sudden cardiac death.

**Methods:** N/A

**Results:** We report a 23‐year‐old male initially presented with palpitation followed by syncope and shortness of breath with history of malaria infection and has switched treatment from quinidine to Dihidroartemisinin ‐ Piperaquin (DHP). Further investigations reveal ST Elevation electrocardiogram pattern related to brugada syndrome, confirmed with *flecainide challenge test*. Subsequently, we stop antimalarial drug and consent to perform Implantable Cardioverter defibrilator (ICD). Initially, patient feel clinical improvement after treatment then discharged from hospital.

**Conclusions:** Another possible cause of arrhythmic events happened following antimalarial consumption. This case highlights the possibility of proarrhytmogenic mechanism of malaria infection and antimalarial drug resulting in typical manifestation of brugada syndrome.

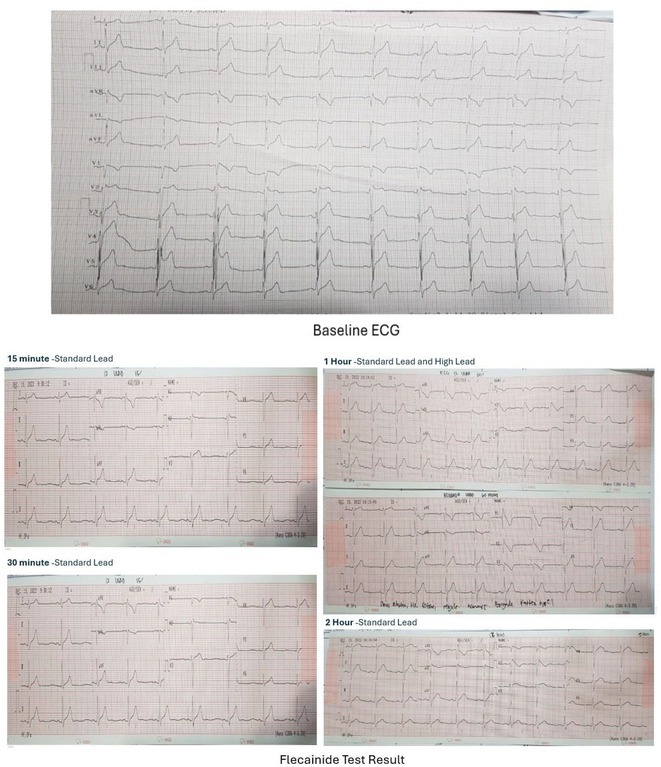

## THE IMPACT OF CONTINUOUS LOOP STIMULATION PACEMAKER ON SEVERE VASOVAGAL SYNCOPE TREATMENT

### **ISKANDAR MIRZA AMRAN**, SHARIMILA SHANMUGAM, MING YOONG LOW, AZLAN HUSSIN, SURINDER KAUR KHELAE ATMA SINGH

#### Institut Jantung Negara, Kuala Lumpur, Malaysia

**Introduction:** The Effects of severe vasovagal syncope (VVS) can be traumatic, not only due to the acute events but also because of the necessary lifestyles changes.

**Methods:** N/A

**Results:** 47‐year‐old man with underlying diabetes, hypertension, dyslipidaemia, obstructive sleep apnea, ischemic heart disease, and end stage renal disease. He presented with recurrent syncope during rest over the past week. The investigation revealed abnormal findings in various test, including elevated troponin T and NT proBNP levels, abnormal ECG results, decrease ejection fraction in the echocardiogram (37%), and stress induced ischemia at the left anterior descending artery and right coronary artery territories on technetium scan. Despite addressing the coronary perfusion through angioplasty, the patient experience multiple episodes of bradycardia and asystole, especially during sleep and apena moments. The diagnosis of cardioinhibitory syncope secondary to high vagal tone was made and a continuous loop stimulation (CLS) implant pacemaker was considered as a treatment option. The patient underwent CLS implantation, resulting in cessation of bradycardia episodes and asystole. Post‐CLS implant, the patient was discharged after 5 days.

**Conclusions:** The study emphasizes the challenges in the therapeutic approach to VVS. The evidence supports the role of pacing with CLS capability in subgroups with frequent cardioinhibitory syncope recurrence, particularly in patient aged over 40 years who are refractory to treatments. In conclusion, the case highlights the complexity of managing VVS and the potential effectiveness of CLS pacemakers, particularly in patients with cardioinhibitory syncope and lack response to other interventions. The study contributes to the evolving understanding of treatment options for severe VVS cases.

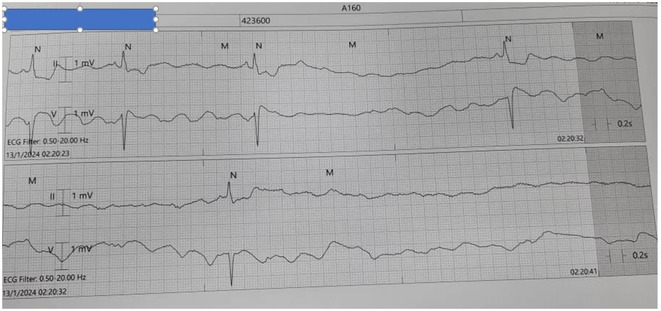

## EFFICACY AND SAFETY OF DIRECT ORAL ANTICOAGULANTS VERSUS VITAMIN K ANTAGONISTS IN ATRIAL FIBRILLATION PATIENTS WITH MODERATE OR ADVANCED CHRONIC KIDNEY DISEASE: A META‐ANALYSIS OF RANDOMIZED CONTROLLED TRIALS

### **MUAMMAR EMIR ANANTA** ^1^, CHIQUITA FEBBY PRAGITARA^2^, IGNATIUS IVAN^3^, BAYUSHI EKA PUTRA^2^, INDRA BUDI PERKASA^1^

#### ^1^Cempaka Putih Jakarta Islamic Hospital, Central Jakarta, Indonesia,^2^Berkah Regional General Hospital, Pandeglang, Indonesia,^3^Kalabahi Regional Hospital, East Nusa Tenggara, Indonesia

**Introduction:** Atrial fibrillation (AF) is common in patients with Chronic Kidney Disease (CKD) and associated with a worse prognosis. We systematically appraise the literature to compare the use of Direct Oral Anticoagulants (DOACs) versus Vitamin K Anatagonists (VKAs) in AF patients with moderate or advanced CKD.

**Methods:** We systematically searched Pubmed, Scopus, and Cochrane Library for Randomized Controlled Trials (RCT) that compare the efficacy and/or safety of DOAC (edoxaban, apixaban, dabigatran, or rivaroxaban) with VKA (warfarin or other VKAs) in AF patients with moderate CKD (CrCl 30‐50 mL/min), advanced CKD (CrCl <30 mL/min) or undergoing maintenance hemodialysis. Pairwise meta‐analysis with random effects model was performed for the primary analyses, while fixed‐effects model was employed for sensitivity analyses. Cochrane risk of bias‐2 was used to assess risk of bias.

**Results:** Out of 2,188 records, 12 records reporting 9 RCTs comprising 8,033 patients were eligible. Use of DOAC significantly decreases the risk of developing composite stroke or systemic embolism (RR 0.79 [95% CI 0.64‐0.97]; I2 = 0%) and major bleeding (RR 0.74 [95% CI 0.56‐0.98]; I2 = 59%), but it does not significantly decrease the risk of all‐cause mortality, cardiovascular death, ischemic stroke, systemic embolism, life‐threatening bleeding, myocardial infarction, or acute coronary syndrome. Subgroup analysis based on CKD category revealed a significant decrease of major bleeding and ischemic stroke only in advanced CKD patients not undergoing hemodialysis, whereas subgroup analysis based on drugs found only edoxaban (RR 0.75 [95% CI 0.58‐0.96]) and apixaban (RR 0.59 [95% CI 0.37‐0.95]; I2=23%) significantly decreases the risk of major bleeding and only rivaroxaban is associated with a decreased risk of ischemic stroke (RR 0.34 [95% CI 0.17‐0.66]; I2 = 0%).

**Conclusions:** DOAC is superior to VKA in decreasing the risk of stroke or systemic embolism and major bleeding in atrial fibrillation patients with advanced CKD not undergoing hemodialysis.

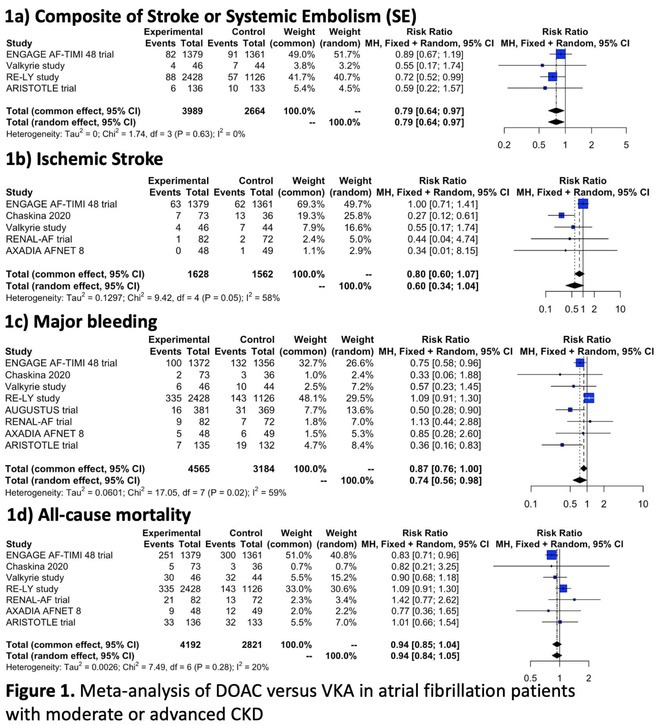

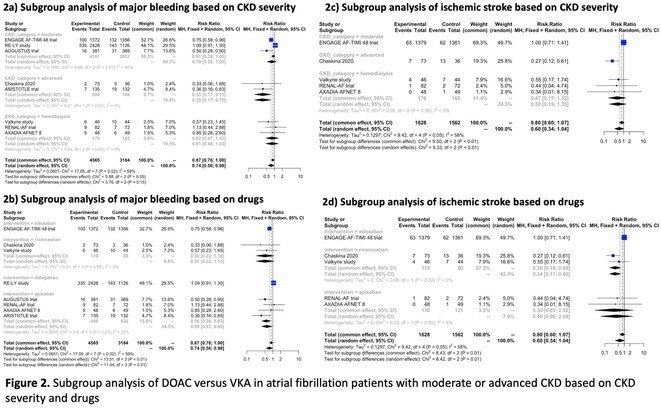

## ISOCHRONAL APPARENT DISPERSION (IAD) AT EARLY ACTIVATION SITES ACCURATELY IDENTIFIES OUTFLOW TRACT VENTRICULAR ECTOPY SITES

### **ROBERT ANDERSON** ^1^, STEPHANE MASSE^2^, JOSHUA HAWSON^1^, GEOFFREY LEE^1^, MUKUND PRABHU^3^, ABHISHEK BHASKARAN^2^, ANDREW HA^2^, KRISHNAKUMAR NAIR^2^, VIJAY CHAUHAN^2^, KUMAR NANTHAKUMAR^2^

#### ^1^Royal Melbourne Hospital, Melbourne, Australia,^2^Toronto General Hospital, Toronto, ON, Canada,^3^Kasturba Medical college, India, Australia

**Introduction:** Localization of outflow tract (OT) premature ventricular complex (PVC) sites is guided by unipolar and bipolar local activation time (LAT). However, LAT‐based localization can be inaccurate if the site is intramural or distant. Deep foci produce rapid conduction velocity (CV) if the wavefront is tangential to the surface. We evaluated if supraphysiological CV referred to as surface isochronal apparent dispersion (IAD) mapping can be used to accurately guide the successful site for OT PVC ablation.

**Methods:** Left ventricular OT (LVOT) mapping was performed if right ventricular (RVOT) mapping demonstrated a bipolar electrogram (EGM) <20ms. The earliest EGMs underwent analysis of the following: first deflection bipolar EGM (bipolar_earliest_) to QRS, bipolar_earliest_ to first deflection unipolar EGM (unipolar_earliest_), bipolar_earliest_ to unipolar ‐dV/dT_max_, unipolar ‐dV/dT_max_ to QRS, number of early LAT breakouts and the surface area of the earliest isochronal breakout. CV_Poly_ was calculated using a custom algorithm in MATLAB using cut‐offs between 1 ‐ 100,000 cm/s and was used to create IAD referred to as apparent dispersion index (ADI). The accuracy of IAD to distinguish between successful and unsuccessful OT sites was assessed and compared to conventional EGM indices.

**Results:** Bipolar_earliest_ ‐ QRS (28.5±7.3ms vs 17.8±5.7ms, P<0.05) is superior to unipolar ‐dV/dt_max_ ‐ QRS (0.4±26.4ms vs ‐6.4±13.4ms, P=0.25) to differentiate successful compared to unsuccessful OT PVC sites. An early isochronal breakout area of less than 1cm^2^ and less than 2 breakouts indicates a successful side (both P<0.05). Bipolar_earliest_ to unipolar ‐dV/dT_max_ and to unipolar_earliest_ were not predictive (28.1±27.7ms vs 24.2±13.3ms, P=0.97 and 6.4±7.3ms vs 6.4±5.8ms, P=0.8, respectively). IAD appears differentiate between successful and unsuccessful sites using an ADI cut‐off of 20,000 cm/s with an accuracy of 93.8% and area under the ROC of 0.95.

**Conclusions:** IAD is a realistic 2D interpretation of the 3D activation mapping surface that may potentially predict OT origins to guide a successful side of catheter ablation.

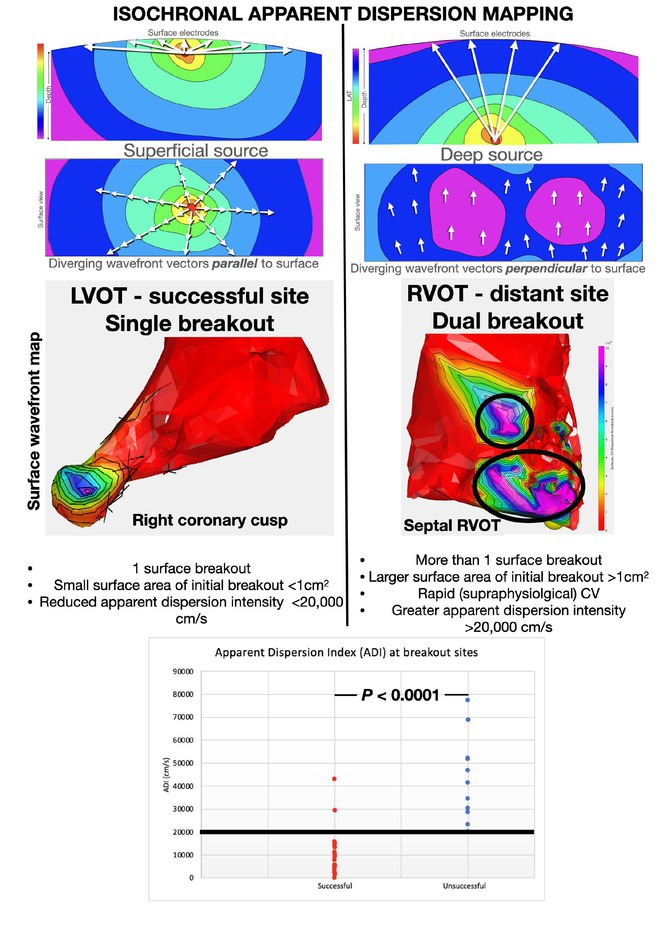

## RIGHT‐SIDED INTRAFASCICULAR RE‐ENTRANT VENTRICULAR TACHYCARDIA

### **SAM ANDERSON**, SACHIN NAYYAR

#### Gold Coast University Hospital, Southport, Australia

**Introduction:** Right‐sided intrafascicular re‐entrant ventricular tachycardia (VT) is a unique form of ventricular arrhythmia characterised by re‐entry circuit involving the right bundle branch (RBB) and adjacent myocardium, often associated with structural heart disease. Management challenges arise due to its rarity and complex electrophysiological features.

**Methods:** N/A

**Results:** A 59‐year‐old male presented in haemodynamically stable wide QRS complex tachycardia with left bundle branch morphology and left axis following exertion. Initially, acute coronary syndrome was suspected, and invasive coronary angiogram revealed severe ostial left circumflex coronary artery disease (CAD). Transthoracic echocardiogram revealed proximal sigmoidal deformity of the interventricular septum, but a structurally normal heart with no regional wall motion abnormalities. Subsequently, coronary artery bypass graft surgery was performed. Post‐operatively, electrophysiology study still demonstrated reproducible stable wide QRS tachycardia with a basal‐mid right ventricle (RV) posteroseptal exit. In addition, bidirectional activation of the RBB fascicles with fascicular‐like proximal‐to‐distal early diastolic and distal‐to‐proximal late diastolic signals spanning the tachycardia cycle length was observed. Entrainment pacing from RV apex showed manifest fusion and reset the tachycardia with a PPI‐TCL of 95ms. Overall, these features were compatible with right‐sided intrafascicular re‐entry VT with longitudinal dissociation within the RBB fascicles and participating bridging myocardium from a non‐ischemic substrate, and incidental bystander CAD. Catheter ablation was not offered due to anticipated risk of proximal conduction system injury. Patient has remained stable on follow‐up.

**Conclusions:** This case illustrates the complexities associated with right‐sided intrafascicular re‐entrant VT, particularly in a patient with concurrent bystander CAD and no structural heart disease. The rarity of this arrhythmia and the intricate electrophysiological features with proximity to the conduction system further compound the diagnostic and therapeutic challenges.

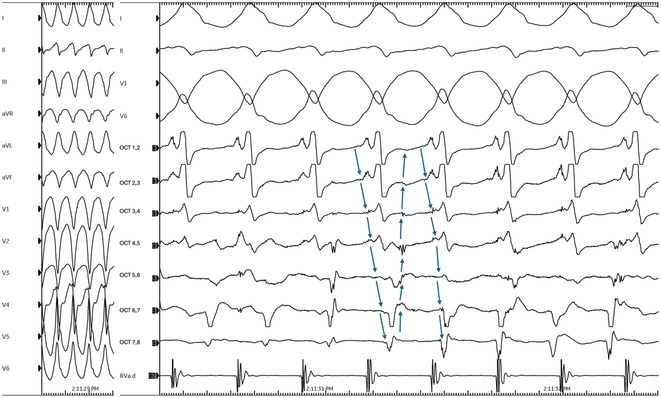

## CORRELATION BETWEEN HEART RATE VARIABILITY AND BURDEN OF PREMATURE VENTRICULAR ECTOPY IN PATIENTS AFTER MYOCARDIAL INFARCTION WITH EJECTION FRACTION ≥40% : WHAT WE NEED TO FIND OUT

### **YURIKO ANDRE** ^1^, MERLIN SARI MUTMAINDAH^1^, HAUDA EL RASYID^2^, TOMMY DAINDES^2^

#### ^1^Cardiology and Vascular Medicine, Faculty of Medicine Andalas University, Padang, Indonesia,^2^Arrhythmia Division Cardiology and Vascular Medicine, Faculty of Medicine Andalas University, Padang, Indonesia

**Introduction:** Premature ventricular complex (PVC) burden increased in patients after myocardial infarction (MI) and strongly associated with fatal ventricular arrhythmia (VA) and cardiac mortality. Heart rate variability (HRV) has been extensively studied in patients surviving MI and reduced HRV have an increased risk of mortality after MI. Burden of PVC after MI incidents is well established in patients with low ejection fraction, but in patients with left ventricular ejection fraction (LVEF)≥40% is still unclear. So it is necessary to explore factors that influence PVC burden in post‐MI patients with LVEF≥40%. Heart rate variability may be considered as risk factor that influence it. The purpose of this study was to investigated correlation between HRV and burden of PVC in post‐MI patients with LVEF≥40%.

**Methods:** This study was retrospective design at Cardiology outpatient‐clinic M. Djamil Hospital, Padang, Indonesia from February 2022 until February 2024. A total 31 consecutive patients with palpitation with history of MI performed 24 hours holter monitoring. Clinical characteristics, echocardiography (LVEF≥40%) and angiography were noted. Data on HRV and PVC burden were obtained from 24‐hours holter monitor. The heart rate variability data entered are the SDNN and RMSSD parameters. Relationships between HRV parameters and PVC burden were evaluated.

**Results:** The study sample consisted of 31 patients with a mean age 59.6±8.93 years, 67.7% were male and 32.3% were female, and mean LVEF 49.2 %±6.47. In holter monitor data could seen in table 1, mean PVC burden was 7.52 %±9.51, mean HRV SDNN 60.29 ms±37.74. There were moderate negative correlation significantly between HRV SDNN parameter and PVC burden (r = ‐0.420; p = 0.019), but between HRV RMSSD and PVC burden were low correlation (r = ‐0.285; p = 0.120), which could seen in figure 1.

**Conclusions:** This study showed moderate negative correlation significantly between HRV SDNN parameter and PVC burden (r = ‐0.420; p = 0.019). Furthermore, this study needs to be confirmed by multicenter studies with bigger sample size in the future.

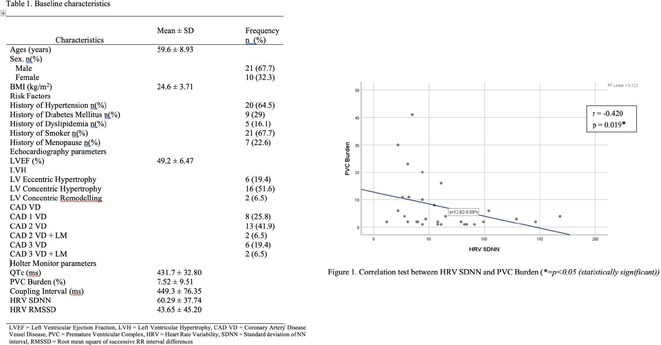

## UNCOMMON PRESENATON OF COMMON TACHYCARDIA

### **JIAN‐GANG ANG**, HAZLEENA MOHAMED HASNAN, YEW FUNG KWAN, CHUN LIANG LIM, SHEN YEW YEE, ZARRIN KANG, TAI MENG CHEN, KENGESWARI RAJA, GURPREET PAL SINGH JUGINDAR SINGH, RAMACHANDRAN SATHAPPAN, NOR HANIM MOHD AMIN

#### Hospital Raja Permaisuri Bainun, Ipoh, Malaysia

**Introduction:** Supraventricular tachycardia(SVT) can be differentiated through measurement of the RP interval. This measurement can be helpful in daily clinical practise but it might not be applicable to all cases. We present a patient with atypical presentation of SVT.

**Methods:** N/A

**Results:** A 70‐year‐old woman with underlying hypertension, dyslipidemia presented to hospital with recurrent episodes of palpitation. ECG showed short RP narrow complex tachycardia with pseudo s’ at inferior leads and pseudo r’ at V1. Vagal maneuver and IV adenosine failed to terminate the tachycardia. A subsequent electrophysiological study provided the following findings: 1) The presence of retrograde atrial activation occuring earliest at the proximal coronary sinus with a short septal VA interval (<70ms) 2)Tachycardia with wobbling tachycardia cycle length(TCL) 3) Changes of A‐A interval preceded the changes in H‐H interval 4) Unsuccessful entrainment with right ventricular overdrive pacing. Thus, we determined that patient might have septal AT. 3D mapping of right atrium and left atrium noted the earliest atrial signal was at midseptal region of right atrium which was 38ms earlier than surface p wave. AT was terminated after 2 secs of radiofrequency ablation(RFA). Post RFA, we noted that the patient had first degree AV block with a PR interval of 310ms. Although septal AT is a cause of short RP tachycardia, the likely mechanism of short RP tachycardia for this patient was delayed AV nodal conduction during tachycardia causing the P wave to be cast close to the preceding R wave. It was evident by the AH interval during AT: 312 ms and the HA interval: 40 ms, resulting in a long septal AH/HA ratio (>1) mimicking septal AH/HA ratio of typical AVNRT. This showed that the delay is confined primarily to the AV node.

**Conclusions:** In summary, our case illustrated a combination of AT and first degree heart block which mimicked typical AVNRT. Although typical slow‐fast AVNRT is the commonest cause of short RP tachycardia, the need to consider other differentials when the tachycardia is not terminated by adenosine.

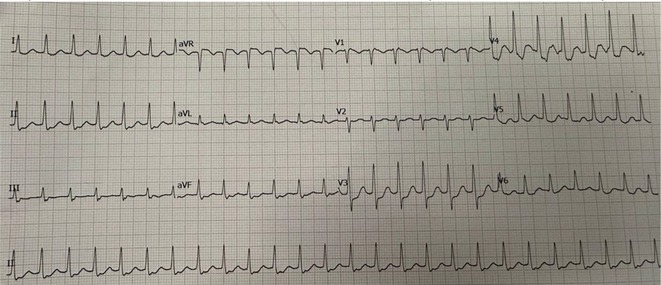

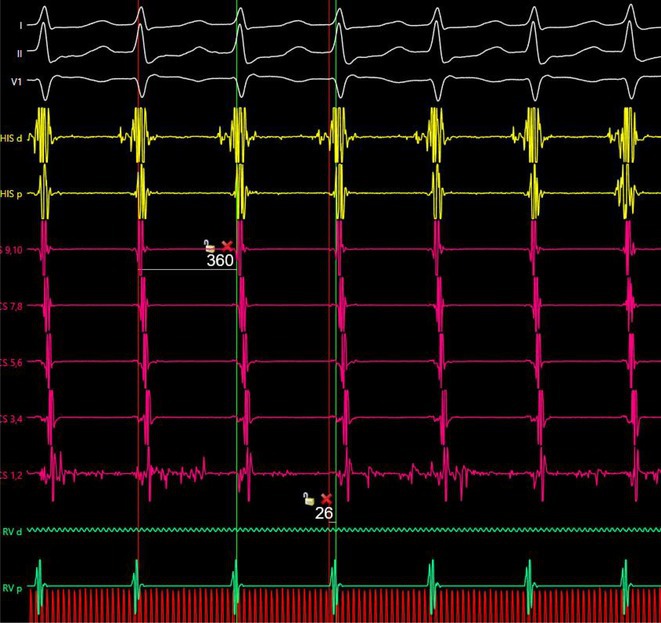

## ARTERIOVENOUS FISTULA AS UNCOMMON COMPLICATION OF CIEDS

### **SAFIRA ANJALIA** ^1^, GIKY KARWIKY^2^, MOHAMMAD IQBAL^2^, CHAERUL ACHMAD^2^

#### ^1^Department of Cardiology and Vascular Medicine, Faculty of Medicine, Universitas Padjadjaran ‐ Hasan Sadikin General Hospital, Bandung, Indonesia, Bandung, Indonesia,^2^Electrophysiology Division, Department of Cardiology and Vascular Disease, Faculty of Medicine, Universitas Padjadjaran ‐ Hasan Sadikin General Hospital, Bandung, Indonesia

**Introduction:** Vascular injury can occur during puncture of the axillary vein which is needed for various cardiac procedure such as Cardiovascular Implantable Electronic Device (CIEDs) implantation and electrophysiology study. One of the uncommon complication that may arise is the arteriovenous (AV) fistula. In some patients, AV fistula might not cause any clinical symptoms. However some late complications of AV fistula which includes chronic venous insufficiency, ischemia of the affected upper limb, or high output heart failure can occur in some patients. Immediate intervention is needed to prevent further complication of AV fistula. The use of stents to covered the fistula is preferred due to its low morbidity compared with surgical repair.

**Methods:** N/A

**Results:** A 55 years old male had a previous history Ischemic Cardiomyopathy. He underwent ICD implantation as secondary prevention 1 year prior to admission. The patient came to the outpatient clinic with a unilateral hand swelling 2 months after the ICD implantation. He was then given anticoagulation for 8 months. However, the swelling did not subside. Further examination revealed continuous murmur at deltopectoral region. The clinical suspicion of possible AV fistula at the region of ICD implantation arise. The diagnosis were confirmed by angiography. A stent was deployed at the level of AV fistula. Further angiography showed no flow at the AV fistula with the immediate resolution of the unilateral hand swelling.

**Conclusions:** Early recognition of AV fistula as the complication of ICD implantation is needed to determine the most appropriate therapeutic approach. Symptoms such as ipsilateral arm swelling and excessive bleeding during implantation should raise suspicion of this vascular injury.

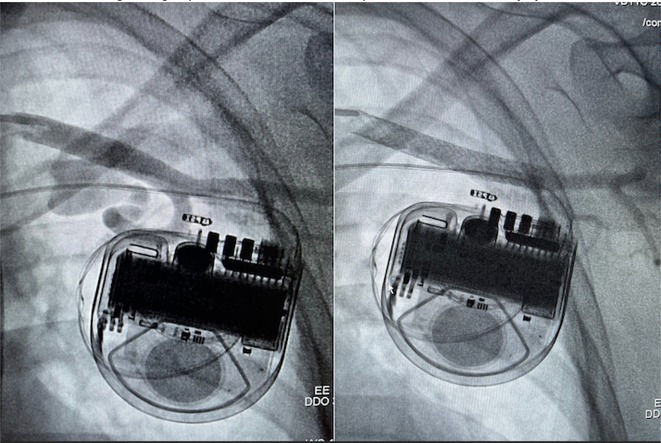

## THE OUTCOME OF THE CATHETER ABLATION AFTER FONTAN PROCEDURE

### **HISAAKI AOKI**, MASAYOSHI MORI, KUMIYO MATSUO, DAI ASADA, YOICHIRO ISHII, YOSHIHIDE NAKAMURA

#### Osaka Women's and Children's Hospital, Izumi, Japan

**Introduction:** Arrhythmias after Fontan procedures are often difficult to treat with ablation in terms of access to the heart and arrhythmic substrate. Recently, the use of conduit or inferior vena cava puncture has improved the results of ablation. We report the outcome of the catheter ablation after Fontan procedure.

**Methods:** Fourteen cases for catheter ablation performed after Fontan surgery were reviewed for type of Fontan surgery, age at procedure, type of arrhythmia, presence of conduit puncture, session time, energy, acute success rate, and complications.

**Results:** The types of Fontan procedures included 3 atrio‐pulmonary connection (APC), 2 LT, 9 EC, 8 female. Eighteen sessions were performed. Age at session was 4‐39 years; arrhythmias were atrioventricular reentrant tachycardia (AVNRT) in 6 cases, intraatrial reentrant tachycardia in 5 cases, premature ventricular contractions/ ventricular tachycarida in 2 cases, tachycardia involving twin atrioventricular nodes (TAVN) in 2 cases, atrioventricular reciprocating tachycardia in 2 cases, and junctional ectopic tachycardia in 1 case; patch/conduit puncture was performed in 16 of the 18 sessions (except 2 APCs). Session duration was a median of 335 minutes (180‐549 minutes) with a dose of 675 mGy (67‐1567 mGy). Energy consisted of 16 sessions of radiofrequency and 2 sessions of cryo. Acute success was achieved in 12 of 14 patients, but 5 had recurrence (3 AVNRT, 1 JET, 1 TAVN), 4 had no recurrence after a second session, and 1 was controlled with drug therapy. Two unsuccessful patients (APC/LT) underwent total cavo‐pulmonary connection conversion for arrhythmia control. There was no death, but one patient had a recurrence of preoperative plastic bronchitis.

**Conclusions:** Conduit puncture was safe. Ablation results have improved due to easier access to the atrium. However, the puncture itself is time‐consuming, and the evaluation and ablation of the arrhythmic substrate is often difficult. We hope to solve these problems by accumulating more cases in the future.

## COMPARISON OF THE FIRST FREEZING SUCCESS RATE OF THE CRYOBALLOON ABLATION WITH 28 MM OR 31 MM CRYOBALLOON FOR THE LEFT COMMON PULMONARY VEIN

### **HIROFUMI ARAI** ^1^, YASUTERU YAMAUCHI^1^, YUMI YASUI^1^, ATSUHITO ODA^1^, KAZUYA MURATA^1^, YUICHIRO SAGAWA^1^, TETSUO SASANO^2^

#### ^1^Japan Red Cross Yokohama City Bay Hospital, Yokohama, Japan,^2^Tokyo Medical and Dental University, Tokyo, Japan

**Introduction:** Cryoballoon ablation is an effective option for the pulmonary vein isolation (PVI) and it can be used for the left common pulmonary vein (LCPV). The POLARx is one of the devices of the cryoballon ablation which has 28 mm cryoballoon and the latest type POLARx FIT brings options of balloon size 28 mm or 31 mm. However, the difference of efficacy of the cryoballoon ablation for the LCPV with 28 mm or 31 mm cryoballon was not well elucidated.

**Methods:** We retrospectively analyzed consecutive patients with LCPV who underwent cryoballoon ablation with the POLARx or the POLARx FIT from January 2022 to April 2024. First freezing was performed with 28 mm cryoballoon of the POLARx or with 31 mm cryoballoon of the POLARx FIT. Cryoballoon size of second or more freezing was selected by operators’ discretion in the cases of the POLARx FIT. The success rate of the PVI by the first freezing, successful PVI rate by cryoablation, nadir temperature, number of applications and total freezing time were compared between the POLARx and the POLARx FIT.

**Results:** Total of 49 patients were analyzed, 30 (61.2%) were male and the mean age was 66.4 ± 11.7 years. The POLARx group was 22 and the POLARx FIT group was 27. First freezing success rate of the PVI was 1/22 (4.5%) vs 8/27 (29.6%), p=0.03. Successful PVI rate by the cryoablation was 20/22 (90.9%) vs 27/27 (100%), p=0.2. Mean nadir temperature was ‐53.5 ± 4.7°C vs. ‐54.7 ± 5.7°C, p=0.43. The number of applications was 3.2 ± 1.1 vs. 2.3 ± 1, p=0.005 and total freezing time was 516.7 ± 166.6 sec vs. 378.3 ± 132.3 sec, p=0.002.

**Conclusions:** The POLARx FIT with 31 mm cryoballoon had higher first freezing success rate. Mean nadir temperature had no significant difference but the number of applications was lower and total freezing time was shorter in the POLARx FIT.

## ASSESSMENT OF ELECTROMAGNETIC INTERFERENCE OF IMPELLA HEART PUMP AND 3D MAPPING SYSTEM

### **TAKUMI ARASHIRO**, AKIRA MIZUKAMI, MAKI OONO, NOGUCHI SOUICHI, KUMAI RYOUICHI, MIYAMOTO HINA, HIROKI ISHIZU, TAKAFUMI YAMASAKI

#### Kameda Medical Center, Kamogawa, Chiba, Japan

**Introduction:** Recently, there have been many reports of Impella heart pump (Impella)‐assisted catheter ablation. There have been concerns about the accuracy of the 3D mapping system (3D) due to electromagnetic interference of Impella. However, there have been no reports of verification of these interaciton. The aim of this study is to verify electromagnetic interference of Impella and 3D.

**Methods:** The Impella were fixed to the bottom of the perfusion‐type water tank, and various catheter tips were fixed at 1 cm intervals from the motor of impella. The support level was changed to three levels (P2, P6, P9), and the amount of displacement of position information from the reference point (P0) due to electromagnetic interference was measured. The support level during Initialization of the 3D was also changed in three steps (P0, P2, and P9), and the effects of each condition were also compared. The accuracy error of ±1mm (±3mm for multipolar catheters) was evaluated as the safety range.[Devices]Pump catheter: IMPELLA CP smart assist, IMPELLA 5.5Mapping system: CARTO3 V7.5Mapping catheter: Smart touch STSF, OCTARAY (2‐2‐2‐2‐2).

**Results:** The support level of Impella and the displacement of the location information at each distance are shown in the figure[A].

**Conclusions:** Electromagnetic interference of the Impella pump catheter alters the positional information of the mapping system. The effect was greater with closer distance and stronger support. The interference can be mitigated by making adjustments of the support level at Initialization.

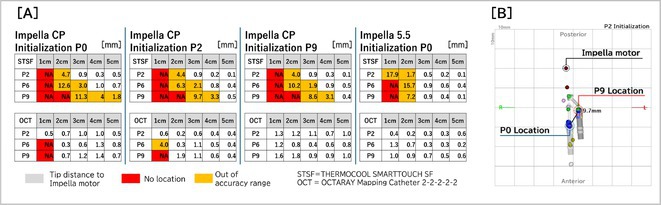

## THE IMPACT OF ADDITIONAL ABLATION FOR REGIONS WITH SHORT CYCLE LENGTHS AS INDICATED BY THE NEW CARTOFINDER ALGORISM, CYCLE LENGTH COLORING MAP

### **KEIICHI ASHIKAGA**, HIROYUKI TAKEKAWA, YASUAKI TSUMAGARI, MIWA ITO, KENGO AYABE, YOSHISATO SHIBATA

#### Miyazaki Medical Association Hospital, Miyazaki, Japan

**Introduction:** An effective additional approach beyond pulmonary vein isolation (PVI) for persistent atrial fibrillation (AF) has not yet been established.

**Methods:** We analyzed 60 consecutive patients with persistent AF (67±9 years, 84% male, median AF duration:15 months (7‐60)) who underwent left atrial (LA) mapping during sustained AF following PVI and LA posterior isolation utilizing the CARTOFINDER new module known as the cycle length coloring map (CLCM). On the CLCM, a centrifugal pattern of tachycardia cycle length gradation was observed during AF.

**Results:** After a 3‐month blanking period, 12(20%) experienced atrial tachycardia/AF recurrence during a median follow‐up period of 296±75 days. 31 patients who underwent additional ablation for regions with short cycle lengths as indicated by CLCM tended to have a lower recurrence (13% vs 28%, p=NS) compared to those without additional ablation. In particular, in the group that did not undergo additional ablation for regions with short cycle lengths, there was a recurrence of AF in 5 of 8 patients (63%), whereas in the group that underwent additional ablation, there was only one recurrence of AF (25%).

**Conclusions:** Ablation of regions with shorter cycle lengths utilizing the CARTOFINDER module might lead to a feasible and effective additional approach beyond PVI for persistent AF.

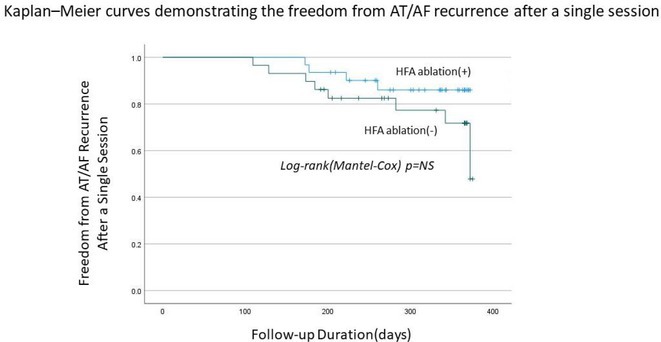

## INDUCED VENTRICULAR TACHYCARDIA IN ACUTE RHEUMATIC FEVER COMPLICATED WITH NON‐ISCHEMIC CARDIOMYOPATHY

### **TAM ADRIAN AYA‐AY**, GISELLE GERVACIO, MICHAEL‐JOSEPH AGBAYANI, WILFRED DEE, BIANCA VELANDO, ZANE OLIVER NELSON

#### University of the Philippines‐Philippine General Hospital, Manila City, Philippines

**Introduction:** Disturbances in cardiac rhythm and conduction have been reported as a common cardiac manifestations for acute rheumatic fever, however malignant ventricular arrhythmias can arise especially when accompanied by non‐ischemic cardiomyopathy. Optical medical therapy along with implantable cardioverter‐defibrillators (ICDs) have shown to reduce mortality in patients who survived an episode of life‐threatening ventricular arrhythmia.

**Methods:** N/A

**Results:** A 36‐year old male who developed monomorphic ventricular tachycardia complicated by heart failure presented with dyspnea and transient second‐ degree atrioventricular (AV) blocks Mobitz type 1 on ECG. Baseline 2D‐echocardiogram revealed depressed left ventricular systolic ejection fraction at 39%. Patient underwent emergency coronary angiogram which showed angiographically normal coronary arteries. During the hospital stay, patient also presented with migratory polyarthritis, fever, elevated inflammatory markers (ESR and CRP) and elevated ASO titer (800 IU/ml), hence diagnosed as acute rheumatic fever. Due to the history of AV blocks and non‐sustained monomorphic tachycardia, patient was then referred for an electrophysiology study which revealed normal HV interval with induced monomorphic ventricular tachycardia (220 bpm) via ventricular extrastimulation testing (600 ‐ 250ms). Due to this finding, patient underwent successful ICD insertion and then optimized with heart failure medications. On follow‐up a year after, the patient already had resolved symptoms for heart failure, however did not showed any significant improvement from baseline ejection fraction. Device interrogation showed no recurrence of life‐threatening arrhythmias and absence of pacing dependency.

**Conclusions:** Life‐threatening arrhythmias can be an important manifestation of autoimmune rheumatic fever as well as non‐ischemic cardiomyopathies which may have a serious impact on morbidity and mortality. Hence, optimization via optimal medical therapy and implantable cardioverter‐defibrillator (ICD) therapy plays a role among patients at high risk of arrhythmic death.

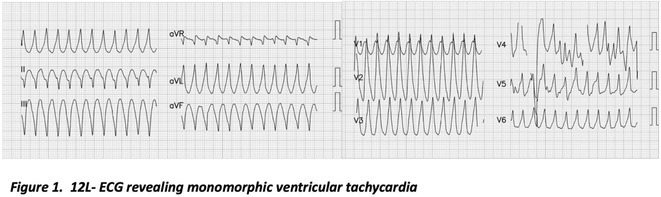

## THE FACTORS WHICH CAN INFLUENCE THE DECISION OF HIGH POWER DEVICE IMPLANTATION FOR HEART FAILURE PATIENTS

### **KENGO AYABE**, KEIICHI ASHIKAGA, HIROYUKI TAKEKAWA, YASUAKI TSUMAGARI, YOSHISATO SHIBATA

#### Miyazaki Medical Association Hospital, Miyazaki, Japan

**Introduction:** It is imperative for patients of heart failure with reduced ejection fraction (HDrEF) to undergo high power device such as Implantable Cardiac Defibrillator (ICD) to prevent sudden cardiac death. It has been reported that the implantation rate is relatively low in Asian countries as compared with that of European countries and North America. The aim of this study is to research the device implantation rate and to clarify the factors which could influence the decision of the implantation.

**Methods:** This is single center retrospective study. We selected out patients with reduced left ventricular election fraction (less than 35%) diagnosed by transthoracic echocardiogram (TTE) between January 2021 and December 2021. We collected the data of patient characteristics, the device implantation rate and the prognosis.

**Results:** 11424 cases of TTE were performed during the research period. 760 cases of TTE demonstrated low left ventricular ejection fraction. After excluding the duplicated cases which means that patients who underwent TTE more than once during the period, 336 patients were included in this study. Twenty‐eight (19.2%) patients underwent device implantation such as ICD and cardiac resynchronization therapy defibrillator (CRT‐D). The patients who deferred device implantation were older (77.4±11.5 vs 71.6±10.3 years‐old, p=0.005) and associated with the presence of motor (35% vs 3.6%, p=0.00042) or cognitive dysfunction (35% vs 3.6%, p=0.00042), such as history of stroke, dementia or mental diseases (21.7% vs 3.6%, p=0.042) respectively as compared with the patients who underwent device implantation. After the logistic regression analysis, the cognitive dysfunction is the factor which influenced the device implantation rate.

**Conclusions:** The device implantation is relatively low at our institute located in Japan. The cognitive dysfunction can influence the decision of device implantation.

## ARRHYTHMIA INCIDENCE ACCORDING TO CARDIOVASCULAR‐KIDNEY‐METABOLIC HEALTH STATUS CHANGES

### **HAN‐JOON BAE**, YOUNG SOO LEE

#### Daegu Catholic University College of Medicine, Daegu, Korea, Republic of

**Introduction:** The changes in Cardiovascular‐kidney‐metabolic (CKM) Health are dynamic. We have conducted an analysis of hospital admissions for arrhythmia events associated with changes in CKM stages.

**Methods:** Data of population‐based cohort were obtained from the Korea National Health institute of health during January 2001 to December 2020. Among 10,030 participants were enrolled. We included 8,312 participants with CKM stage 0 to 3. We categorized changes in CKM stage into three levels: aggravation, no change, and improvement, based on changes over a two‐year period.

**Results:** During a series of index time periods, there were changes in CKM stage, with approximately 15.7% showing improvement and approximately 71.8% showing no change. During about 14‐years follow up period, hospital admissions for arrhythmia events were reported 9 participants.

**Conclusions:** During the follow‐up period, no statistical significance was observed in arrhythmia events based on changes in CKM stage. Further detailed analysis and confirmation of results for other cardiovascular diseases may also be necessary.

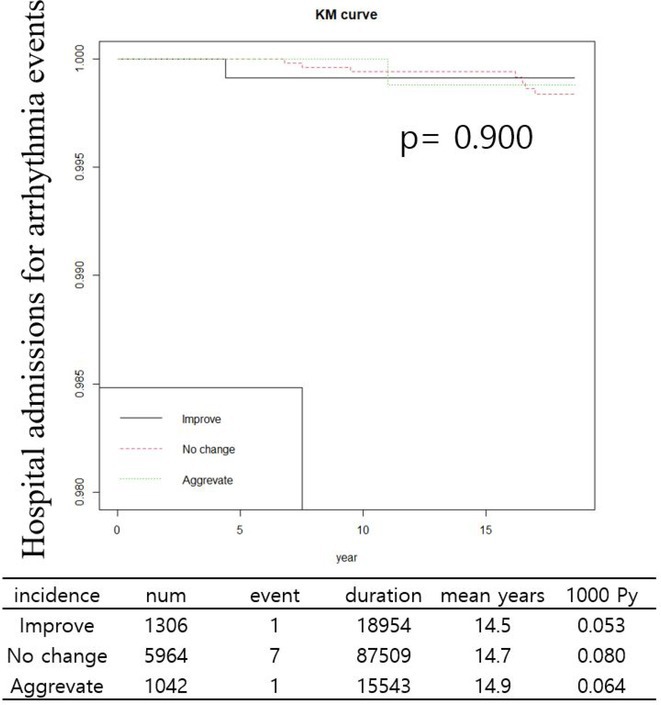

## ATRIAL FIBRILLATION AND OR ATRIAL FLUTTER INCIDENCE ACCORDING TO CARDIOVASCULAR‐KIDNEY‐METABOLIC HEALTH STATUS CHANGES

### **HAN‐JOON BAE**, YOUNG SOO LEE

#### Daegu Catholic University College of Medicine, Daegu, Korea, Republic of

**Introduction:** The changes in Cardiovascular‐kidney‐metabolic (CKM) Health are dynamic. We have conducted an analysis of hospital admissions for Atrial fibrillation and or Atrial flutter events associated with changes in CKM stages.

**Methods:** Data of population‐based cohort were obtained from the Korea National Health institute of health during January 2001 to December 2020. Among 10,030 participants were enrolled. We included 8,312 participants with CKM stage 0 to 3. We categorized changes in CKM stage into three levels: aggravation, no change, and improvement, based on changes over a two‐year period.

**Results:** During a series of index time periods, there were changes in CKM stage, with approximately 15.7% showing improvement and approximately 71.8% showing no change. During about 13‐years follow up period, hospital admissions for arrhythmia events were reported 178 participants. When adjusted for gender and age, the HR and 95% CI for the occurrence of atrial fibrillation in the no change group compared to the improved CKM status group were 1.86 (1.11 to 3.12). For the aggravation group, the HR and 95% CI were 1.19 (0.59 to 2.37). This indicates that the no change group had a statistically significantly higher risk of developing atrial fibrillation or atrial flutter compared to the improvement group.

**Conclusions:** During the follow‐up period, improvement group suffered the lower atrial arrhythmia events compared with no change group in CKM stage. Further detailed analysis and confirmation of results for other cardiovascular diseases may also be necessary.

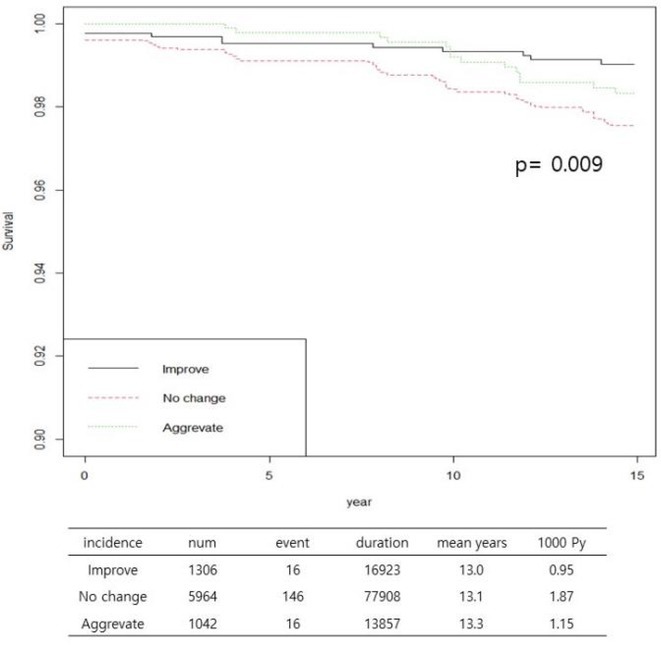

## PREDICTING ATRIAL FIBRILLATION IN PATIENTS WITH IMPLANTABLE CARDIAC MONITOR IMPLEMENTATION: A PROSPECTIVE, LONG‐TERM FOLLOW‐UP STUDY USING COMPREHENSIVE AI ECG ANALYSIS

### **YONG‐SOO BAEK** ^1,2^, DONG‐HO LEE^2^, SANG‐CHUL LEE^3,2^, WONIK CHOI^3,2^, DAE‐HYEOK KIM^1,2^, HEE‐KWON PARK^1^

#### ^1^Inha University Hospital, Incheon, Korea, Republic of,^2^DeepCardio Inc., Incheon, Korea, Republic of,^3^Inha University, Incheon, Korea, Republic of

**Introduction:** Implantable Cardiac Monitors (ICM) are crucial for long‐term atrial fibrillation (AF) monitoring. We prospectively evaluated the predictive correlation between artificial intelligence (AI) augmented electrocardiography (ECG) and AF detection during follow‐up.

**Methods:** This study involved 123 patients with ICMs, followed prospectively every three months from September 2016 to January 2023. Baseline 12‐lead ECGs in normal sinus rhythm were analyzed using AI to estimate AF risk. Patients were categorized into high and low‐risk groups based on their AI‐augmented ECG scores. Multivariate logistic analysis was performed to assess the impact of our AI‐ECG on the risk of subsequent AF.

**Results:** AF was detected in 28 of the patients (22.8%), with a mean follow‐up of 31.3 ± 15.9 months. AI‐ECG scores for AF were significantly lower in the AF‐detected group compared to the non‐AF group (50.5 ± 32.6 vs. 69.5 ± 32.4, p < 0.05) (Figure 1). Kaplan‐Meier analysis revealed a significant difference in survival probability between high and low‐risk groups by AI‐augemented ECG (p < 0.0001) (Figure 2). Multivariate analysis identified AI‐ECG (HR 3.56, 95% CI: 1.39‐9.10, p = 0.008), history of stroke (HR 6.53, 95% CI: 2.86‐14.89, p < 0.05), and age (HR 1.04, 95% CI: 1.02‐1.08, p = 0.04) as significant predictors of AF.

**Conclusions:** Our research suggests the utility of our AI‐augmented ECG in prognosticating AF in patients under prospective long‐term surveillance with ICM. It underscores the value of the AI‐augmented ECG as an effective instrument for patient risk stratification, potentially transforming the anticipatory management of AF.

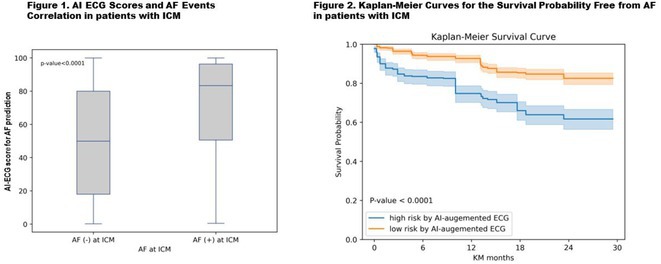

## RADIATIONTHERAPY VT CASE

### DEOKWOO BAK

#### Severace, Seoul, Korea, Republic of

**Introduction:** An 11‐year‐old Mongolian boy visited a local hospital after complaining of dyspnea on exertion for 3 years. ECG revealed an incessant monomorphic ventricular tachycardia (VT). Echocardiography revealed severely decreased global left ventricular (LV) systolic function, 10% of LV ejection fraction (EF), and severely dilated LV. Magnetic resonance imaging of the heart showed LVEF reduced to 3% and no delayed enhancement. He was diagnosed with heart failure (HF) with reduced EF. Medical therapy for HF and VT was not effective. Despite several attempts with radiofrequency catheter ablation (RFCA) for VT, the VT was sustained. At this stage, the patient was transferred to our hospital.

**Methods:** At admission, blood pressure, 84/39 mmHg; heart rate, 114/min. ECG showed sustained monomorphic VT (Figure 1A). Chest X‐ray showed cardiomegaly (Figure 1B). ECG monitoring revealed the VT was incessant. Electrophysiological study was performed and 3‐dimensional activation map revealed a focal tachycardia originating from the RV free wall (Figure 2A). The VT was terminated by RFCA performed. However, VT recurred one hour after the procedure. Several anti‐arrhythmic drugs were ineffective. We decided to perform cardiac radioablation. The cardiac radioablation was planned by transferring the 3D map of the VT to the planning CT using bone landmarks that included the ribs and vertebrae (Figure 2B). Noninvasive cardiac radioablation was performed on the VT origin, the RV mid free wall, with a single fraction of 25 Gy.

**Results:** Seven weeks after radioablation, Holter monitoring showed that the burden was reduced to 24% and echocardiography showed an LVEF of 12% and synchronous wall motion. The patient was discharged with oral medications for HF management. For 3 years, we have not been able to contact him since he returned to his country. After 3 years of cardiac radioablation, he visited the clinic. Chest X‐ray showed improvement in cardiomegaly (Figure 1C). ECG monitoring revealed no VT. Echocardiography showed that LV systolic function and LV volume were normalized.

**Conclusions:** In conclusion, cardiac radioablation can be a good treatment option for incessant and refractory VT in pediatric patients.

## ELECTRICAL STORMS: CARBOPLATIN AND DOCETAXEL‐INDUCED CARDIOTOXICITY IN A 48‐YEAR‐OLD MALE PATIENT

### **NURBAETI BAKHTIAR**, MUZAKKIR AMIR, ABDUL HAKIM ALKATIRI, RUFIAT SYAHRIR

#### Hasanuddin University, Makassar, Indonesia

**Introduction:** The persistence of cardiotoxic effects, albeit asymptomatic, not only has a negative impact on prognosis but also limits therapeutic opportunities. Clinical manifestations of chemotherapy‐induced cardiotoxicity encompass a wide spectrum, ranging from ischemia, arrhythmia, hypertension, left ventricular dysfunction, to heart failure.

**Methods:** N/A

**Results:** A 48‐year‐old male patient presented with palpitations after the fifth cycle of Docetaxel and Carboplatin chemotherapy, which started a day after the first cycle. He had controlled hypertension and diabetes. Initial evaluation showed irregular heart rate (170 bpm), atrial fibrillation with rapid ventricular response on ECG, and left ventricular hypertrophy on chest X‐ray. Thoracic MSCT scan suggested active old pulmonary tuberculosis, infected bronchiectasis, and cardiomegaly. Echocardiography revealed no decrease in left ventricular ejection fraction but abnormal left ventricular wall movement. Laboratory tests indicated anemia and leukocytosis.Initially diagnosed with atrial fibrillation rapid ventricular response, the patient received a digoxin bolus 0.5 mg/iv. However, the rhythm changed to typical AVNRT/DD Atrial Tachycardia. Modified vagal maneuvers restored sinus rhythm. Later, the patient developed Atrial Tachycardia/DD atypical AVNRT and then atrial flutter with variable conduction DD/multifocal atrial tachycardia after metoprolol bolus 5 mg/iv. Intravenous amiodarone stabilized the rhythm. By the 5th day, the patient improved and was discharged for outpatient care.

**Conclusions:** This case underscores the importance of vigilant monitoring and prompt intervention in managing chemotherapy‐induced arrhythmias. Furthermore, the case emphasizes the need for comprehensive risk assessment and close follow‐up in patients with underlying cardiovascular risk factors undergoing cancer treatment to mitigate potential cardiac complications and optimize therapeutic outcomes.

## A COMBINED IMPLANTATION OF S‐ICD AND LEADLESS PACEMAKER IN PATIENT WITH TRANSIENT TOTAL AV BLOCK AND VT: A CASE‐REPORT

### DANA ABDIMALIKOVA, **ABAY BAKYTZHANULY**, YERLAN TURURBAYEV, SERIK BAGIBAYEV, ZHANDOS YESSILBAYEV, OMIRBEK NURALINOV

#### Corporate Fund "University Medical Center", Astana, Kazakhstan

**Introduction:** Sudden Cardiac Death (SCD) is a significant global health issue, and while conventional transvenous ICD (TV‐ICD) is effective, their lead placement may be unfeasible for some patients: combining Subcutaneous ICD (S‐ICD) with leadless pacemaker (LP) offers a viable solution, particularly for those with venous access issues or recurrent complications.

**Methods:** This case involves a 52‐year‐old with a history of recurrent ventricular tachycardia and transient total AV block, presenting with palpitations, dizziness, and syncope. Echocardiography revealed a reduced ejection fraction (34%). Due to hemodynamically not‐tolerated VT, secondary prevention of sudden cardiac death was recommended, aligning with ESC 2022 guidelines (class IA). However, venography revealed subclavian vein occlusion on both sides, complicating TV‐ICD implantation. Furthermore, a history of transient 3rd degree AV block in 2022 signaled a need for a pacemaker (ESC 2021, IC). Considering limited access to the right ventricle (RV), a leadless pacemaker (LP) with bipolar pacing emerged as the optimal option.

**Results:** After extensive discussions with cardiac specialists, a combined therapy of a leadless pacemaker and an S‐ICD was chosen. A sequential approach began with LP implantation through right femoral vein access. Next step, S‐ICD implantation was successfully completed. At the 3‐month follow‐up, the patient remained asymptomatic, and all parameters were satisfactory.

**Conclusions:** This case highlights the success of a tailored therapeutic strategy, combining a leadless pacemaker and S‐ICD, in a challenging scenario with subclavian vein occlusion and recurrent arrhythmias. The comprehensive approach, considering patient‐specific factors and utilizing innovative technologies, showcases the adaptability of cardiac interventions to improve outcomes in complex cases of arrhythmias and structural challenges.

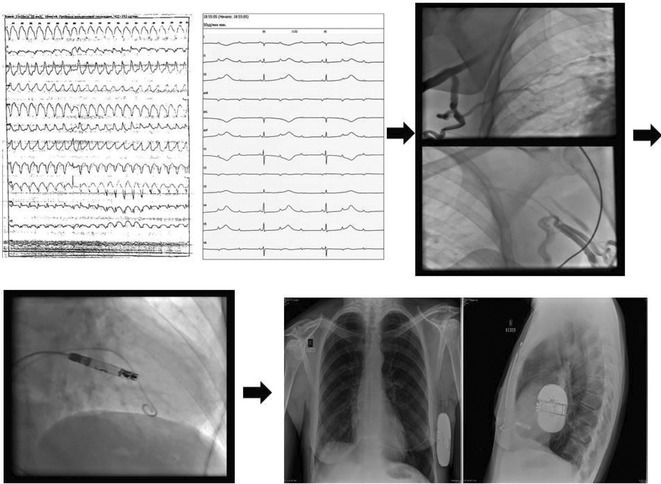

## INITIAL EXPERIENCE OF THE NEW GENERATION OF LEADLESS PACEMAKER IMPLANTATION

### **ABAY BAKYTZHANULY**, YERLAN TURUBAYEV, SERIK BAGIBAYEV, ZHANDOS YESSYLBAYEV, OMIRBEK NURALINOV

#### “University Medical Center” CF, Astana, Kazakhstan

**Introduction:** The development of leadless pacemakers has changed the pacing industry by providing a creative solution to the shortcomings associated with traditional transvenous pacemakers. This article includes a number of cases describing our first experience of implanting LP Aveir VR (Abbott, USA) in Kazakhstani patients, which was a significant step in the development of this technology in our region.

**Methods:** The case series included five male patients with a range of symptoms, including presyncope, dizziness, and dyspnea on exertion. Their age ranged from 14 to 77 years. Indications for pacing have been identified, and some individuals also have comorbidities such as AV block and atrial fibrillation. The parameters of post‐implantation stimulation and the results of the initial observation were recorded.

**Results:** The procedure was quite successful and radiographic confirmation of the location of the leadless pacemaker was obtained. All patients demonstrated clinical improvement and satisfactory pacing parameters at early follow‐up.

**Conclusions:** The Aveir leadless pacemaker has demonstrated its potential to minimize lead insertion complications, with no immediate complications observed in this case series. The study highlights the benefits of the leadless pacemaker in addressing common problems with conventional pacing systems. Our experience expands knowledge of leadless pacemaker and highlights the need for larger, multicenter trials to test the long‐term benefits and broader applicability of this new approach.

## LEADLESS PACEMAKER IMPLANTATION AFTER TRADITIONAL PACEMAKER SYSTEM EXTRACTION

### **ABAY BAKYTZHANULY**, YERLAN TURUBAYEV, TEMIRBEK ABUBAKIR, ZHANDOS ESSYLBAYEV, SERIK BAGYBAYEV, OMIRBEK NURALINOV

#### “University Medical Center” CF, Astana, Kazakhstan

**Introduction:** Conventional pacemakers (PM) have greatly improved the outcomes of patients with bradyarrhythmia. However, they can lead to complications such as infections associated with the PM pocket or lead‐related complications. Conventional PMs, which consist of both intravascular and extravascular components, are particularly susceptible to infections, mainly affecting the pocket and transvenous leads. The emergence of leadless pacemaker (LP) technology offers new possibilities for managing PM infections and reducing the risks of recurrent infection.

**Methods:** N/A

**Results:** Patient C., a 36‐year‐old female with a total atrioventricular (AV) block, had a traditional pacemaker implanted on the right side in 2000y and was replaced in 2008y and 2014y due to battery depletion. In 2016y due to pocket infection, a single‐chamber traditional pacemaker was implanted on the left side without lead extraction of the previous PM. Two years later she was hospitalized due to a lead dysfunction. All leads from the right side were mechanically extracted using the Extor set (Biotronik), and PM was replaced. Five months later, she developed a pocket infection of the left‐side pacemaker. Instrumental examinations revealed thrombosis of the left internal jugular, left subclavian and left innominate veins, multiple lung abscesses, and pneumonia. PM system was extracted and LP Micra VR (Medtronic) was implanted. Pacing parameters: threshold 0.75m V, pulse width 0.24 ms, sensing 12 mV, impedance: 790 Ohms. The fluoroscopy time was 15 min and the device was connected to the remote monitoring system Carelink. The postoperative period was without complications. The patient was discharged in satisfactory condition 14 days after the procedure. The follow‐up period showed no signs of infection and normal pacing parameters.

**Conclusions:** The LP Micra can be successfully implanted after complete removal of an infected pacemaker system, achieving low pacing thresholds and high sensitivity to R waves similar to primary implantation.

## RADIOFREQUENCY CATHETER ABLATION OF ATRIAL FLUTTER IN A ONE‐YEAR‐OLD CHILD

### **ABAY BAKYTZHANULY**, YERLAN TURUBAYEV, TEMIRBEK ABUBAKIR, ZHANDOS ESSYLBAYEV, SERIK BAGIBAYEV, OMIRBEK NURALINOV

#### “University Medical Center” CF, Astana, Kazakhstan

**Introduction:** Supraventricular tachycardia (SVT) is the most prevalent type of arrhythmia. Infants may exhibit fussiness or irritability, while older children may describe feelings of rapid heartbeats or palpitations. Additionally, SVT can lead to potential complications such as heart failure. Radiofrequency ablation could be effective and safe in drug refractory arrhythmias.

**Methods:** N/A

**Results:** An electrophysiological study of the heart and radiofrequency ablation (RFA) of the right isthmus were performed. Patient Sh., 12 months old, was diagnosed with supraventricular tachycardia. There is no history of prescribed cardiac arrhythmia medication. Laboratory analyses were within the normal range. ECG revealed atrial flutter type I with a ventricular contraction rate of 171 beats per minute. Echocardiography showed dilatation of the left ventricle (LV), reduced LV ejection fraction (EF) of 34%, moderate mitral regurgitation (MR), and a patent foramen ovale, NT‐proBNP was 243 pg/ml. Before the procedure, transesophageal echocardiography ruled out left atrial appendage thrombosis. The femoral veins were used for RFA. The right femoral vein was punctured with 7Fr (non‐irrigated ablation catheter) and the right vein with 4Fr introducer (decapolar diagnostic catheter). According to the decapolar catheter, the tachycardia cycle length (CL) was 220 ms. Pace mapping confirmed cavo‐tricuspid isthmus‐dependent re‐entry tachycardia. The ablation catheter was introduced into the right atrium and linear RFA was performed. RFA parameters: average temperature of 48‐50°C, 25 W, and 119‐150 ohms. The total ablation time was 10 minutes with a bidirectional isthmus block. The procedure was then completed. The patient, in sinus rhythm, was transferred to the department. Holter monitoring of ECG showed stable sinus rhythm and no cardiac arrhythmias. One month follow‐up echocardiography showed persistent LV dilatation but the ejection fraction increased up to 44% and decreased mitral regurgitation.

**Conclusions:** Effective RFA of atrial flutter leads to regression of heart failure. RFA is a highly effective and safe treatment in children with atrial flutter.

## SUCCESSFUL RADIOFREQUENCY ABLATION OF LEFT ATRIAL TACHYCARDIA IN CHILDREN

### **ABAY BAKYTZHANULY**, TEMIRBEK ABUBAKIR, YERLAN TURUBAYEV, SERIK BAGIBAYEV, ZHANDOS ESSYLBAYEV, OMIRBEK NURALINOV

#### “University Medical Center” CF, Astana, Kazakhstan

**Introduction:** Atrial tachycardia (AT) is a relatively uncommon but clinically significant type of arrhythmia. Persistent AT can lead to tachycardia‐induced cardiomyopathy, resulting in heart failure and carrying a similarly grave prognosis as dilated cardiomyopathies from other causes, including the risk of sudden cardiac death. Conversely, clinical studies indicate that effective radiofrequency ablation (RFA) results in the regression of heart failure symptoms. These findings underscore the substantial importance of managing this type of arrhythmia, as its resolution can significantly impact prognosis.

**Methods:** N/A

**Results:** Patient T., 7 years old, diagnosed with atrial tachycardia, suffered from rhythm disorders for the last 6 months. Antiarrhythmic drugs were ineffective in 3 months. Laboratory studies revealed no changes. ECG showed atrial tachycardia with 128 bpm and right bundle branch block. Echocardiography revealed a mild ejection fraction of 48% decrease. The patient was taken to the cath lab. Under local anesthesia, the right femoral approach was used to perform the procedure with the use of the CARTO 3 navigation system. 5Fr decapolar diagnostic catheter was positioned in the coronary sinus which revealed left atrial tachycardia. Through the foramen ovale the 3D activation mapping was performed with the SmartTouch SF catheter revealing the AT focus in the lower wall of the left atrium. RFA was effectively performed with P‐30W and achieved an ablation index of 350. After RFA, regular sinus rhythm with 80 bpm. EPS ruled out other tachycardias. Then the procedure was completed. Holter monitoring of ECG showed a stable sinus rhythm Our clinical case showed that the following picture of lower atrial rhythm, negative P wave on the II, III AVF, V5, V6 leads, and biphasic (‐/+) P wave on V3, V4 registered by ECG, perhaps indicates the arrhythmogenic focus located on the bottom wall of the left atrium.

**Conclusions:** RFA is an effective and safe treatment for ectopic atrial arrhythmias.

## THE ROLE OF ENERGY DRINKS IN ATRIAL FIBRILLATION AMONG YOUNG PATIENTS: A SINGLE‐CENTER’S RETROSPECTIVE STUDY

### DANA ABDIMALIKOVA, **ABAY BAKYTZHANULY**, YERLAN TURURBAYEV, SERIK BAGIBAYEV, ZHANDOS YESSILBAYEV, OMIRBEK NURALINOV

#### Corporate Fund "University Medical Center", Astana, Kazakhstan

**Introduction:** Atrial fibrillation (AF) stands as one of the most prevalent cardiac arrhythmias worldwide. Traditionally perceived as a condition predominantly affecting the elderly, its emergence among younger demographics has sparked growing concern within the medical community. Concurrently, the pervasive consumption of energy drinks among young individuals has garnered attention due to its potential cardiovascular implications.

**Methods:** We included all hospitalized patients between the ages of 18 and 40 with a diagnosis of AF from the Database of our center (Corporate Fund “University Medical Center”, Astana, Kazakhstan) from January 2018 and April 2024. Demographic and comorbidity data were collected and analyzed.

**Results:** Overall, 137 patients (medium age 35, IQR 38‐29, male 58.67%) were included in the study. Among them, 7 patients (5.07%) reported consuming energy drinks (median age 30, mode 29 and 30), all male and without predisposing comorbidities such as hypertension, diabetes, obesity, thyroid disease, and structural heart disease.

**Conclusions:** This study underscores the concerning rise of atrial fibrillation (AF) among young individuals and its potential link to energy drink consumption. Further research is warranted to explore the cardiovascular implications of energy drink use in this population. Healthcare providers should counsel young patients with AF on the potential risks of energy drink consumption and promote healthy lifestyle choices to mitigate the increasing burden of AF in this demographic.

## BIPOLAR ABLATION AS A BAILOUT STRATEGY FOR REFRACTORY VENTRICULAR ARRHYTHMIAS FROM THE LV SUMMIT

### **BHARATRAJ BANAVALIKAR**, AYUSH JAIN, DARSHAN KRISHNAPPA, SATISH REDDY

#### Sri Jayadeva Institute of Cardiovascular Sciences and Research, Bengaluru, India, Bengaluru, India

**Introduction:** Ventricular arrhythmia (VA) from the left ventricular summit (LVS) is frequently associated with arrhythmia‐induced cardiomyopathy. Standard unipolar radiofrequency (RF) ablation in the LVS is often ineffective owing to the presence of thick epicardial fat. The objective of the study was to determine the safety and efficacy of bipolar RF ablation for LV summit VA refractory to sequential unipolar ablation.

**Methods:** Patients with symptomatic idiopathic VA (VT and/or PVC) from the LVS refractory to standard unipolar RF ablation formed the study population. Activation mapping was performed in the distal great cardiac vein, aortic cusps, infra‐valvular LV outflow tract as well as the right ventricular outflow tract, and sequential standard irrigated ablation was performed, starting from the earliest site of activation. Bipolar RF ablation was performed if standard irrigated unipolar ablation was unsuccessful. Bipolar ablation was performed with two irrigated ablation catheters positioned on the opposing surfaces of the earliest site of activation, initially with a power of 30W, and titrated up to 50W, if necessary. All patients were closely monitored during ablation for any adverse effect.

**Results:** Between January 2022 and March 2024, 27 patients (mean age 44.8±16.4 years; 15 females) with LVS VA refractory to sequential unipolar ablation underwent bipolar ablation. The mean LVEF was 47±8.3% and 11 patients had PVC‐induced cardiomyopathy (LVEF <50%). Ten patients had high burden PVC with non‐sustained VT, two had sustained monomorphic VT and 15 had PVC only, with a mean 24‐hour burden of 31.4±8.1%. Fifteen patients had VA from the inaccessible (septal) LVS whereas in 12 patients, the PVC originated in the lateral LVS. Acute procedural success with bipolar RFA (complete elimination of the clinical PVC) was achieved in 25 patients with a mean RF power of 40.6±3.9W without any untoward effect. At a mean follow‐up of 13.3±4.2 months, 24 out of 27 patients (88.9%) were free from the clinical arrhythmia without any anti‐arrhythmic drug.

**Conclusions:** Bipolar RF ablation is a safe and effective bailout strategy for refractory VA from the LV summit.

## ATLEAST 5 INSERTION SITES OF MAHAIM PATHWAY IN YOUNG FEMALE

### PARAG BARWAD

#### PGIMER, Chandigarh, Chandigarh, India

**Introduction:** Mahaim accessory pathway has been traditionally described as an antegrade conducting pathway between RA or AV node to RV or RBB. These are rare pathways with usually single insertion at the RV end demonstrating a classical high frequency Mahaim potentials during EP study which helps in guiding the ablation. In this case we showed an extremely rare case of atleast 5 insertion site of Mahaim pathway at the RV.

**Methods:** N/A

**Results:** A 19‐year‐old female presented with wide complex tachycardia of LBBB and left axis morphology. Her baseline ECG showed no pre‐excitation. Incremental atrial pacing from the high RA showed a progressive AV prolongation and HV shortening. Diagnosis of Atrio‐fascicular pathway (Mahaim pathway) was made and for precise mapping was performed with Flexability irrigated ablation catheter. Classical high frequency Mahaim potentials were noted noted initially in the lateral tricuspid annulus. Ablation at this region led to abolition of these potentials. However, the tachycardia was still inducible easily. A dense mapping was performed over the tricuspid annulus which showed a very wide area over the TA showing the classical Mahaim potentials. It extended from the anterolateral TA to the posteromedial TA region (figure). A broad area of ablation guided by potentials was performed abolishing all of them. Ablation at each region always lead to local automaticity of the pathways. After successful ablation no tachy or pathway conduction could be demonstrated. Prior studies have demonstrated a wide area of possible insertion of these pathways, but the successful site in individual case is usually focal. In our case we found a very wide region over which these pathway potentials were clearly demonstrated, and all had to be ablated for a successful procedure. Her echocardiogram showed no evidence of Ebstein's anomaly.

**Conclusions:** Mahaim pathways are rare and may have a very variable morphology. Though multiple insertion are reported in the past, we report here a very rare case of atlest 5 insertion sites of Mahaims pathway extending from anterolateral TA to posteromedial TA. All insertions required successful ablation to achieve non‐inducibility of tachycardia.

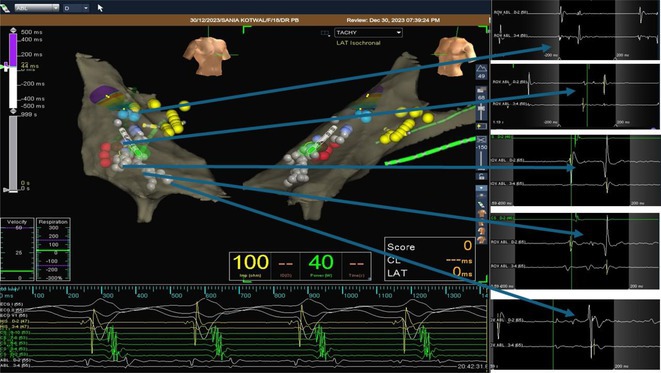

## INNOVATIVE TECHNIQUES TO STABILIZE LEFT VENTRICULAR LEAD IN DIFFICULT CORONARY SINUS ANATOMY AND ITS LONG‐TERM OUTCOME‐ A SINGLE CENTRE SINGLE OPERATOR’S EXPERIENCE

### PARAG BARWAD

#### PGIMER, Chandigarh, Chandigarh, India

**Introduction:** Introduction: Nonresponse to CRT is predominantly related to less than optimal left ventricular (LV) lead placement, its instability and displacement. Innovative techniques may be required to stabilise the LV lead in difficult coronary sinus anatomy.

**Methods:** In this retrospective study, we studied CRT parameters of the patients done by single operator from January 2014 till December 2021, where the innovative techniques to stabilize the LV lead were used. The techniques used to stabilize the LV lead and their baseline parameters were noted. All these patients were followed up and their current LV lead parameters were recorded.

**Results:** Out of 133 CRT implanted during the study period, 23 patients (17.29%) required innovative techniques for placement of LV lead due to difficult CS anatomy. All these patients had LBBB at baseline with a mean QRS duration of 160 ± 4 milliseconds. The mean LVEF of cohort at time of CRT implantation was 26.3% ± 4.7 %. The stylet and guidewire retaining techniques were used in 11/23 (47.82%) and 7/23 (30.43%) patients respectively. In two patients, a coronary stent was inflated in the CS besides the lead to stabilize the LV lead. Two patients had stenosis of CS, which was balloon dilated while one patient had tortuous postero‐lateral vein which was straightened with a coronary stent. There was technical failure of LV leads with loss of capture in 6/23 patients (26.08%) at a median follow up of 41 months (Range : 10 months ‐75 months). Out of these 6 patients, the techniques used were stylet retaining technique in 4 patients, while in two patient coronary guidewire was retained in situ.

**Conclusions:** Amongst different techniques used to stabilize LV in CRT, guidewire or stylet retaining technique was the most common. Though it provides promising short‐term results it may not be ideal in long‐term because of lack of flexibility and physical damage to LV lead. Hence these should be used as last resort in difficult CS anatomy and better leads like active fixation LV leads & other methods of physiological pacing like His Bundle pacing/Left bundle branch pacing should be used.

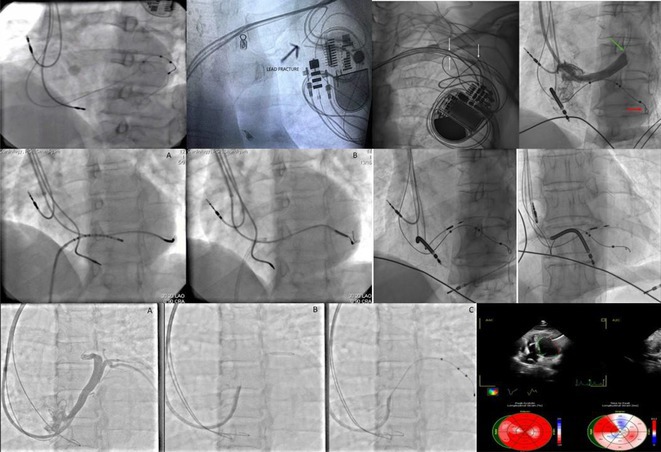

## PERIMITRAL FLUTTER IN MITRAL VALVE REPLACEMENT WITH SEVERE LV DYSFUNCTION

### PARAG BARWAD

#### PGIMER, Chandigarh, Chandigarh, India

**Introduction:** Atrial arrhythmia's are very common in patients undergoing mitral valve replacement in rheumatic heart disease. AF and perimetral flutter are amongst the most common.

**Methods:** N/A

**Results:** A 55‐year‐old male presented to us with severe LV dysfunction in a setting of mitral valve replacement with mechanical mitral valve for RHD 3 year back. Before surgery his LVEF was 55% which dropped to 20% at presentation. His ECG showed a HR of 150/min with atrial rate of 300 /min with positive P wave in V1. ECG was suggestive of AT arising form the LA. Considering the LV dysfunction to be tachycardia cardiomyopathy we considered him for RFA. Under ENSITE mapping with HD grid catheter showed large multiple areas of scarring in the LA. Only few RF burns were required to achieve pulmonary vein isolation of all 4 PV. After this detailed mapping in the LA showed a perimetral flutter with areas of delayed conduction in mitral isthmus region. Epicardial ablation through the CS made the TCL of flutter increase but had not led to termination of flutter. Endocardial ablation was performed carefully under fluoroscopy guidance to avoid entrapment of ablation catheter in the mitral valve. All areas of low voltages were ablated in the mitral isthmus region endocardial to achieve a bidirectional block across the mitral isthmus. This led to termination of tachycardia. Patient is in sinus rhythm at 8 months after surgery and his LVEF has increased to 45%.

**Conclusions:** Conclusion: Though the initial studies suggested a very poor outcome of left atrial ablation in patients with RHD, RFA should considered if patients have tachycardia cardiomyopathy. Perimitral flutter especially in post mitral valve replacement patient may require epicardial and endocardial ablation to achieve a bidirectional block.

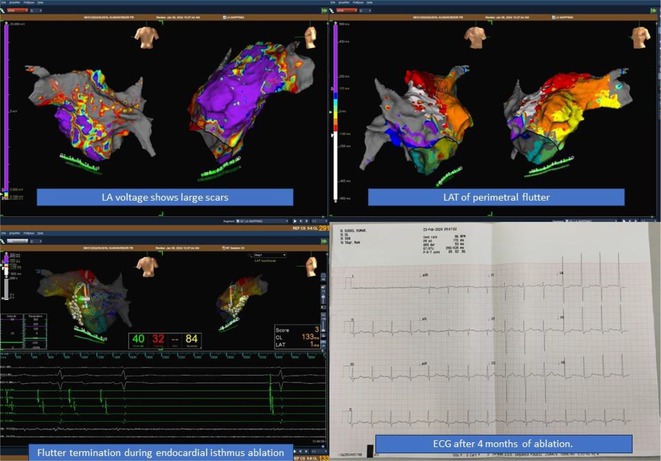

## USE OF CUT‐PIGTAIL CATHETER TO HELP NEGOTIATE AN ACUTE BEND IN THE CORONARY SINUS VENOUS SYSTEM

### PARMINDER SHARMA, **PARAG BARWAD**

#### PGIMER, CHANDIGARH, India

**Introduction:** Placement of LV lead successfully at the desired vein in CRT is the most challenging step in view of highly variable anatomy of CS. Occasionally the junction of CS to the lateral vein has an acute retroflex bend which couldn’t be negotiated with any of the available catheter and improvisation using a pigtail catheter by cutting it half at its distal loop is being demonstrated here.

**Methods:** N/A

**Results:** Case: A 50 year old female patient with diagnosis of Dilated Cardiomyopathy with Ejection fraction of 20‐25% with wide QRS of duration 160 msec and normal coronary angiogram underwent CRT‐P (St. Jude Medical) at our institute. After Coronary sinus engagement with Coronary sinus sheath, CS angiogram showed an acute bend in the origin of lateral vein and a dilated blunt stump just at the same region (image). The guidewire always went in the blunt stump and couldn’t be negotiated across in the lateral vein. Multiple catheters were used including subselect catheters of various angles. This led to dissection of the stump region. Thus, a pigtail catheter was cut at its terminal loop half way (image) to help negotiate the wire exactly in the vein. As the wire length was short the catheter has to be cut half way in its length to remove it retaining the wire at the desired vein. After this the lead could be advanced with the help of a buddy wire. Post procedure ECG showed QRS of 110 msec.

**Conclusions:** When encountered with an acute retroflexed bend in the venous system, which is not getting negotiated by any catheter, Cut‐pigtail catheter technique may be used to negotiate the wire and successful placement if lead.

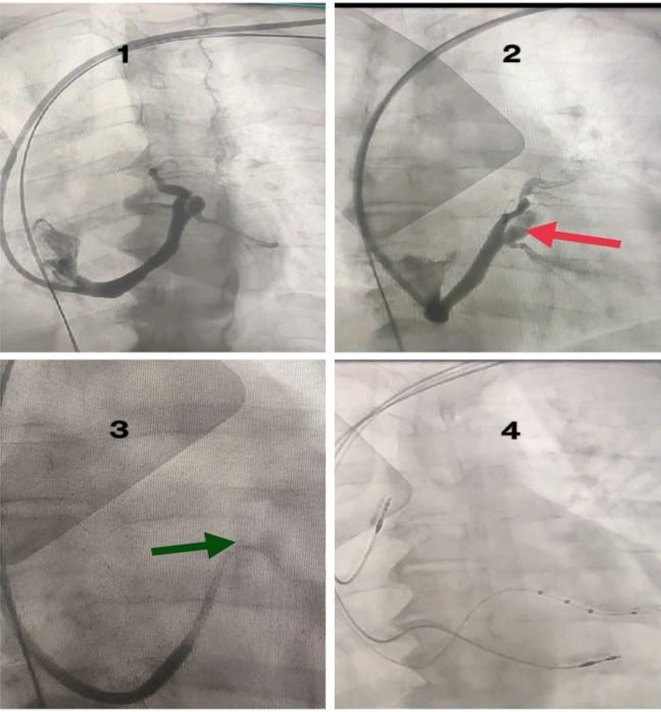

## EVERY MEAL MIGHT BE THE LAST MEAL ‐ SWALLOW SYNCOPE

### **BOON CONG BEH** ^1^, MOHAMAD MOHD SHAWAL FAIZAL^1^, MOHD RAFFALI MOHD ASYIQ AL‐FARD^1^, MOHAMAD FAROUK NOOR DIYANA^1^, CHE HASSAN HAMAT HAMDI^1^, MOHAMMAD KAZMIN NUR EZZATY^2^, SHING SHEN BAY^1^, HOW FOONG KWAN^1^

#### ^1^Faculty of Medicine, Hospital Canselor Tuanku Muhriz UKM (HCTM), Kuala Lumpur, Malaysia,^2^Faculty of medicine and health sciences, Universiti Sains Islam Malaysia, Nilai, Negeri Sembilan, Malaysia

**Introduction:** Swallow syncope is a rare form of neurally mediated situational syncope. There are various types of transient conduction abnormalities during swallowing. We report a case of swallow syncope and treated with a permanent pacemaker.

**Methods:** N/A

**Results:** A 74‐year‐old gentleman with co‐morbidities of diabetes mellitus, hypertension, dyslipidemia, advanced chronic kidney disease, with recent NSTEMI underwent coronary angioplasty to left anterior descending artery. He presented to the emergency department for uremic symptoms 1‐month post‐PCI, complicated with upper gastrointestinal bleeding. Urgent hemodialysis was initiated and he required elective intubation for oesophagogastroduodenoscopy procedure, which found to have Grade 4 esophagitis, duodenitis and multiple Forrest 2C ulcers at stomach. Post extubation, he had dizziness and fainting episodes whenever he swallowed fluid/food. The cardiac monitor showed a sinus pause (Figure 1A) after each time he swallowed, which lasted a few seconds, then reverted to sinus rhythm with regained consciousness. Electrolytes were normal. An echocardiogram showed preserved ejection fraction with left ventricular hypertrophy and no valvular abnormalities. Further history, he has been experiencing this fainting episode intermittently during mealtime for the past few months but did not seek any medical attention. Diagnosis of swallow syncope (triggered by his gastrointestinal disease) was made based on his clinical presentation and demonstrable transient sinus pause during deglutition. A transvenous temporary pacemaker was inserted as backup pacing (Figure 1B) whenever a sinus pause occurred during deglutition while he recovering from the hospital‐acquired infection. A permanent pacemaker was inserted after the infection was treated. He tolerated dialysis and remained asymptomatic during outpatient clinic follow up.

**Conclusions:** The diagnosis of swallow syncope can be suspected based on clinical presentation and demonstration of transient brady‐arrhythmia during deglutition. Treatment options vary and should be individualized based on clinical conditions.

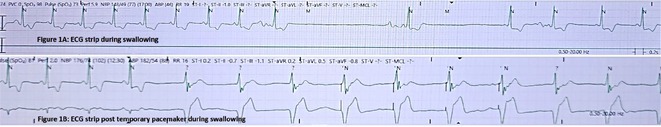

## CHARACTERISTICS AND LONG‐TERM OUTCOMES OF PATIENTS WITH IDIOPATHIC LEFT VENTRICULAR PAPILLARY MUSCLE ARRHYTHMIAS

### **LORI BELL** ^1,2^, NATASHA JONES‐LEWIS^1,2^, LUKAH Q TUAN^1,2,3^, ADRIANA TOKICH^1,2,3^, LARA G. B. HEDLEY^1,2,3^, ANUGRAH NAIR^1,2,3^, JENISH SHROFF^1,2,3^, RAJEEV K PATHAK^1,2,3,4^

#### ^1^Canberra Heart Rhythm, Canberra, Australia,^2^Canberra Heart Rhythm Foundation, Canberra, Australia,^3^Australian National University, Canberra, Australia,^4^University of Canberra, Canberra, Australia

**Introduction:** Ventricular arrhythmias (VAs) can originate from the left ventricular (LV) papillary muscles (PMs). In addition to causing potentially debilitating symptoms, VAs can cause significant left ventricular (LV) dysfunction. Electroanatomical characteristics may determine the outcome of PM ablation procedures.

**Methods:** Of 206 patients undergoing ablation for a VA, 25 (%) patients had PVCs activation and pace mapped to the LV PMs. Only patients with no scar on MRI were included for analysis in this study. The clinical characteristics, etiology and outcomes of patients were investigated.

**Results:** 25 patients were treated with radiofrequency ablation for papillary muscle PVCs. Of these patients (mean age 60 years, 55% male), 2 pts (8%) had a history of ischemic heart disease. At baseline, 69% had normal LV ejection fraction (LVEF) 30% had low LVEF (mean LVEF 53.15 ± 11.7%). PM VAs were PVCs in 28.5% and non‐sustained VT in 71.4%. Site of origin was the LV postero‐medial PM (PM PAP) in 9 (36%) and LV antero‐lateral PM (AL PAP) in 12 (48%) and both PMs in 2 pts (48%). Clinical PVC demonstrated right bundle branch morphology in all cases. Variable precordial transition is observed between papillary muscle PVCs, 37% demonstrating a V5 transition, 19% transitioning in V6 and 44% in V4. All patients with PM PAP had superior axis deviation and AL PAP had Inferior axis in limb leads. Acute success was achieved in 89% of patients. During the 46 ± 13.7 months of follow up, VA free survival was 73% after a single procedure and 84% after repeat procedure. 2 patients had worsening of mitral regurgitation post ablation transiently but got better after 3 months. On multivariate analysis, number of distinct PVC morphology (HR 1.9 CI: 1.4 to 2.3 P=0.02) was only independent predictor of VA recurrence.

**Conclusions:** Catheter ablation of frequent VAs originating from Papillary muscles are a low‐risk and effective treatment strategy. Multiple procedures may be necessary especially in patients with variable PVC morphology.

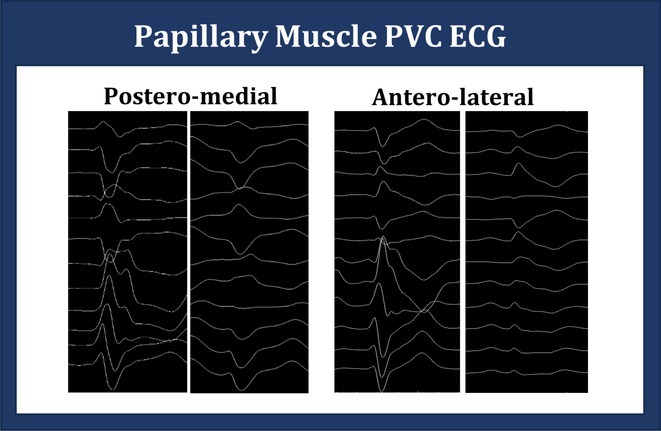

## CLINICAL, ELECTROCARDIOGRAPHIC (ECG), AND ELECTROPHYSIOLOGIC (EP) CHARACTERISTICS OF VENTRICULAR ARRHYTHMIAS (VA) ORIGINATING FROM THE RIGHT CORONARY SINUS OF VALSALVA

### **LORI BELL** ^1,2^, NATASHA JONES‐LEWIS^1,2^, LUKAH Q. TUAN^1,2,3^, ADRIANA TOKICH^1,2,3^, LARA G. B. HEDLEY^1,2,3^, ANUGRAH NAIR^1,2,3^, JENISH SHROFF^1,2,3^, RAJEEV K. PATHAK^1,2,3,4^

#### ^1^Canberra Heart Rhythm, Canberra, Australia,^2^Canberra Heart Rhythm Foundation, Canberra, Australia,^3^Australian National University, Canberra, Australia,^4^University of Canberra, Canberra, Australia

**Introduction:** Premature ventricular contractions (PVCs) can originate from the Right Coronary Cusp (RCC). Clinical and EP characteristics are important to recognize given proximity to Right Ventricular Outflow Tract (RVOT) Site 1.

**Methods:** Of 206 pts undergoing VA catheter ablation, 12 (6%) had PVCs mapped to RCC. ECG and EP characteristics are compared to 8 (5%) with PVCs from RVOT site 1.

**Results:** Of 12 pts treated with radiofrequency ablation for RCC VA (mean age 69, 66% male), 1 (8%) had history of ischemic heart disease (IHD); No h/o IHD in Site 1 (mean age 46, 10% male). Left ventricular ejection fraction (LVEF) at baseline is low in 17% and normal in 75% of RCC pts (mean LVEF 52±9.6); All Site 1 pts had normal EF (mean LVEF 61±8.1). RCC VAs are PVCs in 66% and non‐sustained VTs in 25% of pts. Site 1 had PVCs in 62% and VT in 25%. Mean baseline PVC burden is 5.7% in RCC, and 18% in Site 1. Scar seen on MRI in 25% of RCC pts, and 12% of Site 1. Clinical PVC had left bundle branch morphology in all cases. All RCC PVCs had early transition in V3; Site 1 PVCs transition in V3 in 66% and V4 in 33% of cases. Mean QRS is wider in Site I in all leads excluding lead III. Mean RCC R wave amp is higher than Site 1 in inferior leads. 58% of RCC pts had PVCs eliminated; 8.3% had significant suppression, and 1 pt had acute recurrence. Acute success seen in 100% of Site 1 pts. At follow up of 48±8 months, LVEF improved in 75% of RCC, and 50% of Site 1. Post ablation, 1 Site 1 pt had increase in aortic regurgitation; No increase seen in RCC. 1 RCC pt had pericarditis post ablation, and 1 had new onset right bundle branch block (RBBB) at 12 month‐follow up. 1 Site 1 pt had RBBB at 12 months. No heart block or stroke seen. 1 RCC pt had device implant at 12 month follow up due to unrelated conduction disease.

**Conclusions:** RCC PVCs have distinctive ECG characteristics which aid in recognition and mapping. Catheter ablation can generally be performed safely and effectively.

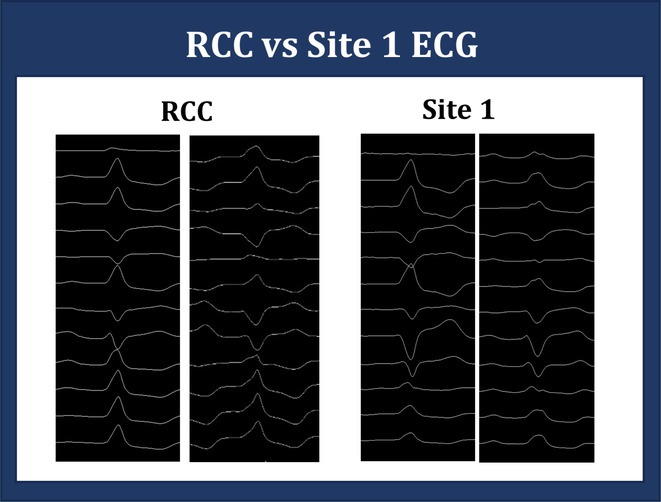

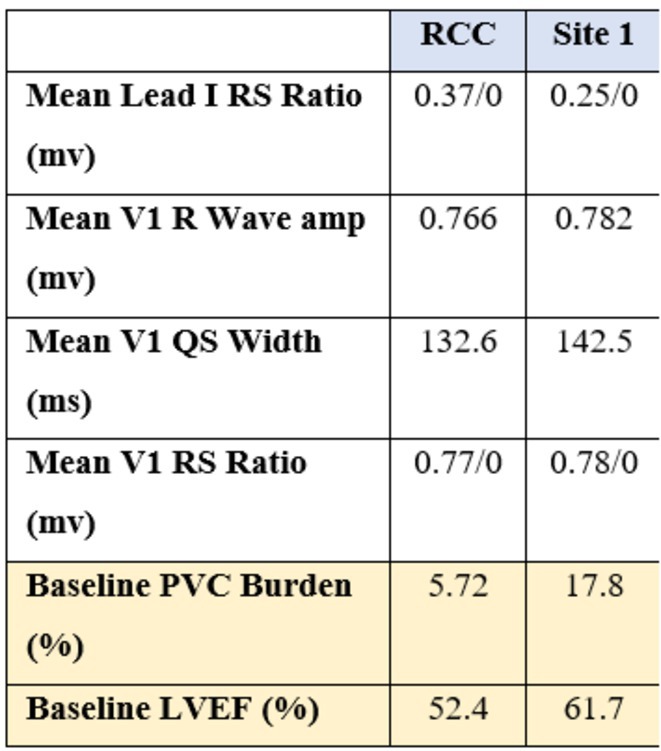

## CLINICAL, ELECTROCARDIOGRAPHIC (ECG), AND ELECTROPHYSIOLOGIC CHARACTERISTICS OF VENTRICULAR ARRHYTHMIAS (VA) ORIGINATING FROM THE LEFT CORONARY SINUS OF VALSALVA

### **LORI BELL** ^1,2^, NATASHA JONES‐LEWIS^1,2^, LUKAH Q TUAN^1,2,3^, ADRIANA TOKICH^1,2,3^, LARA G. B. HEDLEY^1,2,3^, ANUGRAH NAIR^1,2,3^, JENISH SHROFF^1,2,3^, RAJEEV K PATHAK^1,2,3,4^

#### ^1^Canberra Heart Rhythm, Canberra, Australia,^2^Canberra Heart Rhythm Foundation, Canberra, Australia,^3^Australian National University, Canberra, Australia,^4^University of Canberra, Canberra, Australia

**Introduction:** Premature ventricular contractions (PVCs) can originate from the Left Coronary Cusp (LCC). Clinical and electrophysiological (EP) characteristics are important to recognize given proximity to Right Ventricular Outflow Tract (RVOT) Site 3.

**Methods:** Of 206 pts undergoing VA catheter ablation, 22 (10%) had PVCs mapped to LCC. ECG and EP characteristics are compared to 15 pts with PVCs from RVOT Site 3.

**Results:** Of 22 pts treated with radiofrequency ablation for LCC VA (mean age 64, 58% male), 5 (23%) had history of ischemic heart disease (IHD). In Site 3 (mean age 58, 80% male), 1 (7%) had h/o IHD. LV ejection fraction (LVEF) at baseline is comparable, LVEF low in 13% and normal in 82% of LCC pts (mean LVEF 52.8±11.3); low in 7% and normal in 47% of Site 3 (mean LVEF 51.3±11.6). LCC VAs are PVCs in 68% and non‐sustained VTs in 32% of pts. Site 3 had PVCs in 79% and VT in 21%. Mean baseline PVC burden is 5% in LCC, 13% in Site 3. No scar on MRI in Site 3, 35% LCC had myocardial scar. Clinical PVC had left bundle branch morphology in all cases. All LCC PVCs had early transition (≤V3). 62% of Site 3 transition in V3, 37% in V4. Mean LCC QRS is wider in leads I‐III and R wave amp lower in all leads excluding lead I compared to Site 3. QRS in V1 is wider in Site 3. 71% of LCC pts had PVCs eliminated; 8.7% had significant suppression, 4% had acute recurrence (<48 hours) requiring redo ablation. Site 3 PVCs are eliminated in 60%, suppressed in 20%, and 6% had acute recurrence. During follow up of 48±8 months, LVEF improved in 54% of LCC pts, and 40% of site 3 pts. Post ablation, 4 LCC pts and 2 Site 3 pts had increase in aortic regurgitation. No conduction system damage in LCC, 1 Site 3 pt had new onset right bundle branch block and first‐degree AV block at 24‐month post ablation. No heart block observed in either cohort. 3 Site 3 pts and 2 LCC had device implant at 3±36 months due to unrelated conduction disease. 1 LCC pt had hematoma at access site. No stroke seen post‐procedure.

**Conclusions:** LCC PVCs have distinctive ECG characteristics which aid in recognition and mapping. Catheter ablation can generally be performed safely and effectively.

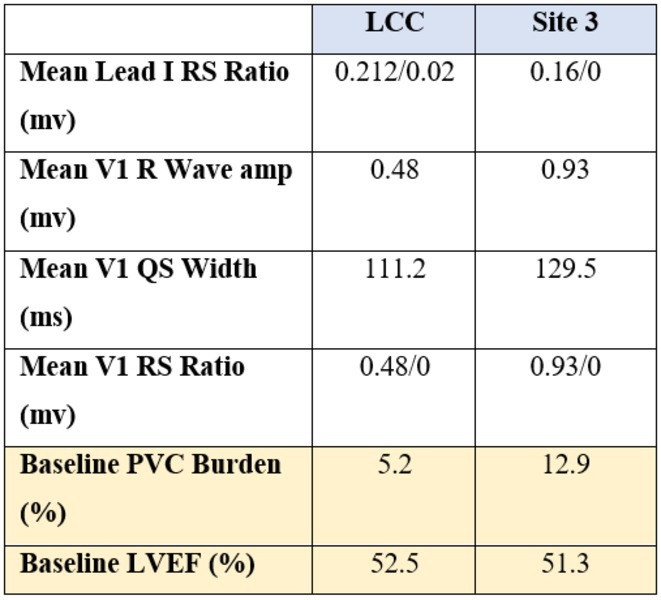

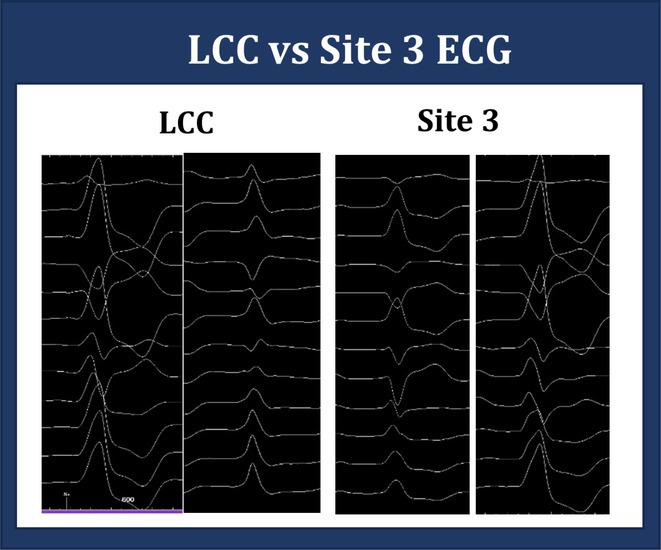

## LIMITATION OF PARA‐HISIAN PACING MANEUVER IN POSTEROSEPTAL ACCESSORY PATHWAY

### THAN HTIKE^1^, SAROJ CHOUDHURY^2^, **DEBABRATA BERA** ^2^

#### ^1^No.2 (1000‐ bedded) Defense Service General Hospital, NAYPYITAW, Myanmar,^2^NH‐RTIICS, KOLKATA, India

**Introduction:** Para‐Hisian pacing is used to differentiate the retrograde conduction over the septal accessory pathway vs AV node. This is especially useful when the tachycardia is not well sustained where maneuvers during tachycardia cannot be performed.

**Methods:** N/A

**Results:** A 15 years old male patient with structurally normal heart had documented narrow QRS tachycardia. The baseline ECG did not show any preexcitation. In EP lab, VA conduction was concentric but non‐decremental. Induced tachycardia cycle length was 450 ms and septal VA interval was 85 ms. Attempted ventricular overdrive pacing (VOP) repeatedly terminated tachycardia. His synchronous PVC advanced the next atrial electrogram (EGM) suggestive of AVRT (Fig 1A). Sequential RV apex and base pacing also showed extra nodal conduction (Fig. 1B and 1C). But para‐Hisian pacing maneuver revealed the stimulus to atrial activation (SA) interval was narrow(124ms) in narrower beat (H+Vc) and wide(140 ms) in wider beat (Vc), suggestive of a nodal response ( Fig. 1D). Mapping revealed earliest atrial activation with fused VA at the right posteroseptal region. Radiofrequency ablation was successful. Post RFA VA conduction was concentric and decremental via AV node. The explanation for discrepant nodal response was speculated to the pathway location (posteroseptal) distant from the pacing site (anteroseptal area).

**Conclusions:** Para‐Hisian pacing can sometimes fallaciously show a nodal response even in presence of retrogradely conducting non‐decremental posteroseptal accessory pathway due to the remoteness of the pacing site. Thus, it can mistakenly indicate atypical AVNRT in cases where tachycardia is non‐sustained. Hence, it is always advisable to perform other maneuvers like sequential RV pacing and His sync‐PVC in all suspected cases of AVRT where VOP repeatedly terminates the tachycardia.

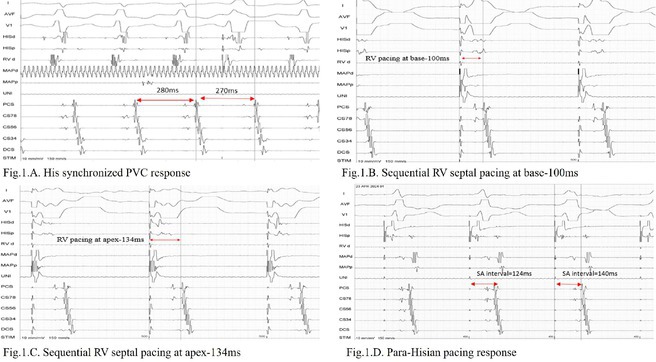

## PARADOXICAL UNDER‐SENSING OF ATRIAL LEAD AT HIGH SENSITIVITY SETTING IN DUAL CHAMBER PACEMAKER DURING ATRIAL FLUTTER ‐ THE ‘QUIET TIMER BLANKING’ PHENOMENON

### THAN HTIKE^1^, SAROJ CHOUDHURY^2^, **DEBABRATA BERA** ^2^

#### ^1^No.2 (1000‐ bedded) Defense Service General Hospital, Naypyitaw, Myanmar,^2^NH‐RTIICS, KOLKATA, India

**Introduction:** Dual chamber pacemakers are equipped with mode switch during atrial flutter / fibrillation.

**Methods:** N/A

**Results:** A 60‐year‐old male had a Medtronic Attesta dual chamber pacemaker implanted last 2 years ago for symptomatic sick sinus syndrome. The measured at the ventricular lead parameters were good. The threshold measured for the atrial lead was 0.7 V, 1.4 mA and the amplitude of the P wave (intracardiac EGM) at implant was 2.5 mV. Atrial sensitivity was set at 0.5mV. During a recent follow‐up visit, atrial flutter was noted on ECG but there was evident atrial pacing spike on ECG (Fig.1). Despite the visible flutter EGMs, the marker did not indicate many A‐EGM events (Fig.2A). Undersensing was suspected. Therefore, it was programmed to higher atrial sensitivity by lowering the value to 0.18 mV. But paradoxically the undersensing worsened with the marker annotations showing no atrial sense events (Fig. 2B). We then tried to reduce the atrial sensitivity by changing the value 1.0 mV. As the sensitivity was reduced, atrial sensing was restored (Fig.2C). Finally, the sensitivity was programmed to 2.0 mV as P waves (A‐EGM) amplitude were > 3‐3.5 mV even during the ongoing flutter (Fig.2D). Paradoxical adjustment of the atrial sensing levels resolved the paradoxical undersensing still with adequate safety margin for the detection of atrial flutter/fibrillation. This reprogramming led to the accurate detection of AF with mode switching to DDIR.

**Conclusions:** The mechanism of paradoxical atrial undersensing during atrial flutter is uncommon but reported. This phenomenon is explained by a repeated activation of the quiet timer blanking interval. Unnecessary high atrial sensing settings may predispose patients to this phenomenon and should be avoided.

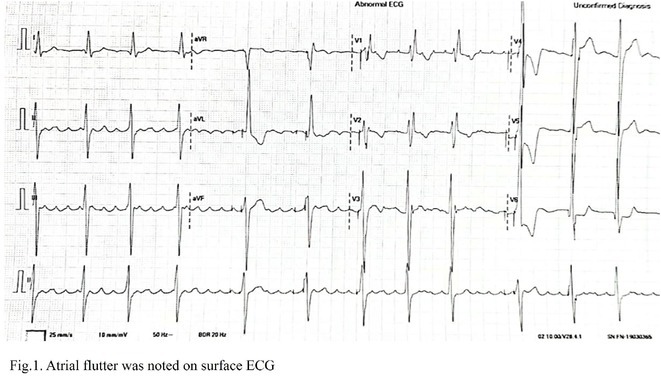

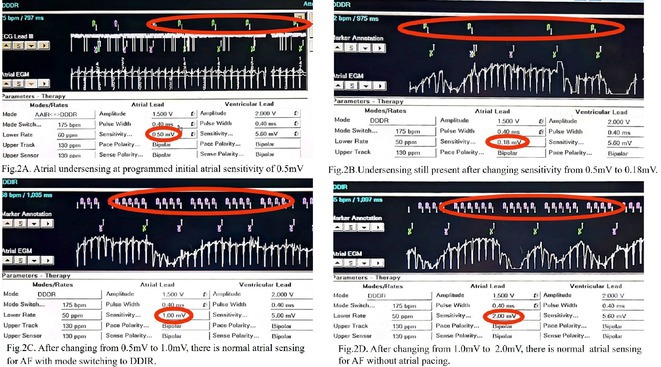

## MULTIPLE RE‐ENTRANT TACHYCARDIAS IN A PATIENT WITH TWO FREE WALL PATHWAYS

### **ANISH BHARGAV**, RAJA J SELVARAJ

#### JIPMER, Puducherry, India

**Introduction:** Patients with more than one accessory pathways can have multiple inducible tachycardias owing to the different possible combinations of reentry involving those accessory pathways and the atrioventricular node.

**Methods:** N/A

**Results:** A 32‐year‐old female underwent electrophysiology study for recurrent palpitations and documented wide complex tachycardia. Electrophysiological study revealed three different inducible tachycardias. Initially a narrow complex tachycardia with a CL of 270 ms and VA interval of 63 ms was induced with a ventricular burst pacing. Eccentric atrial activation, earliest in distal CS was noted with VA prolongation on development of LBBB suggestive of orthodromic tachycardia using left free wall accessory pathway (figure 1A). Second tachycardia was a wide complex tachycardia of LBBB morphology with a CL of 290 ms, VA interval of 121 ms and central atrial activation that was induced with ventricular extrastimulus (figure 1B). A septal refractory premature atrial complex (PAC) advanced the next ventricular activation suggestive of antidromic tachycardia involving right free wall accessory pathway. The third tachycardia was also a wide complex tachycardia of LBBB morphology with CL of 300 ms and HV interval of 55 ms, induced with an atrial extrastimulus. There was a 1:1 ventricular‐atrial relationship during tachycardia with VA interval of 110 ms and eccentric atrial activation, earliest in distal CS (figure 1C), consistent with a duodromic tachycardia proceeding antegrade through the right sided pathway and retrograde through the left sided pathway.EP study confirmed presence of a right sided atriofascicular accessory pathway and a concealed left lateral accessory pathway. The atriofascicular pathway was ablated on the lateral tricuspid annulus followed by left lateral pathway through a transseptal approach.

**Conclusions:** This case is a rare presentation of multiple tachycardias involving right atriofascicular and left lateral accessory pathway. A systematic approach for the assessment of tachycardia mechanism and elucidation of critical components of the circuit is required for the abolition of clinical tachycardia.

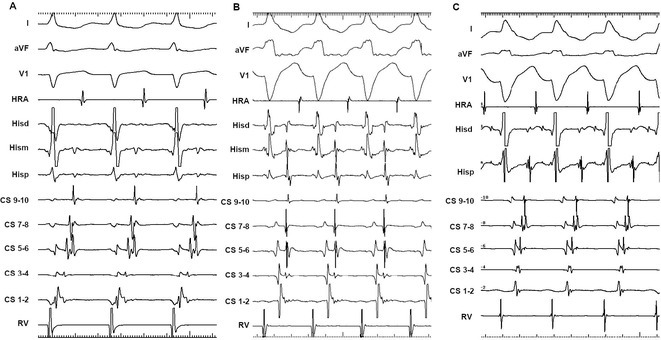

## MANAGING NECESSITIES AND UNCERTAINTIES: TEMPORARY PACEMAKER COMPLICATIONS AND SUCCESSFUL CARDIAC RESYNCHRONIZATION THERAPY PACEMAKER IMPLANTATION

### SHARIMILA SHANMUGAM, **MOHD ASYIQ AL‐FARD BIN MOHD RAFFALI**

#### National Heart Institute, Kuala Lumpur, Malaysia

**Introduction:** Atrioventricular block interrupts the heart's electrical pathways, causing complete heart block. This disrupts communication between the atria and ventricles, compromising heart rate regulation potentially reducing cardiac output. Prompt treatment is vital, often involving a permanent pacemaker.

**Methods:** Temporary pacing was initiated while awaiting permanent pacemaker placement in this case. However, this temporary measure led to pericardial effusion, requiring pericardiocentesis upon discontinuation of temporary pacing.

**Results:** A 72‐year‐old retired army officer with diabetes, dyslipidemia, and chronic obstructive airway disease presented with giddiness, seizures, and syncope. Initially treated for sinus bradycardia leading to Torsades De Pointes with intravenous magnesium sulphate and transcutaneous pacing, he then was transferred to our hospital on the 17/4/24 due to high‐grade atrioventricular block. Temporary pacemaker inserted successfully on the same day. On day 2, he complained of left‐sided chest pain. Echocardiogram revealed ejection fraction of 35% and 1.2cm pericardial effusion likely from the perforation post temporary pacemaker insertion. Due to his cardiovascular risk factors, proceeded with coronary angiogram that showed mild Left Anterior Descending Artery disease and severe stenosis of proximal right ventricular branch. Percutaneous angioplasty was performed on the right ventricular branch with no complications, followed by temporary pacemaker removal causing increasing pericardial effusion that led to pericardiocentesis. He successfully underwent Cardiac Resynchronization Therapy Pacemaker Implantation.

**Conclusions:** This case highlights the crucial role of temporary pacemakers in managing acute cardiac conditions, especially in patients with high‐grade atrioventricular block. Temporary pacing offers essential support while awaiting permanent pacemaker placement. Despite encountered challenges, successful temporary pacing stabilized the patient and enabled subsequent interventions.

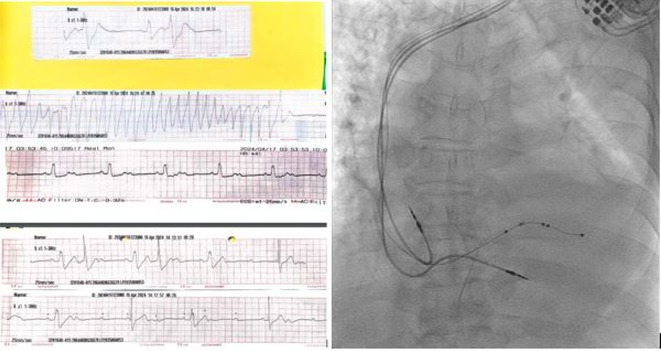

## USE OF 3‐D MAPPING REDUCES FLOUROSCOPY TIME IN CTI ABLATION BUT DOES NOT INCREASE EFFICACY

### **DEAN BODDINGTON**, SIMON LEE, PAULA BISHOP, ANIL JAYENDRAPPA

#### Tauranga Hospital, Tauranga, New Zealand

**Introduction:** In recent years proceduralists have incorporated additional strategies such as 3‐D mapping beyond simple fluoroscopy guided CTI flutter ablation. It is questionable as to how much is gained from these additional modalities.

**Methods:** This is a retrospective study of all CTI ablations performed by a single operator through the period from 2009 to the present across three institutions. Procedural success, defined by achievement of bidirectional block across the CTI line, has been evaluated along with procedural complications and requirement for repeat ablations.

**Results:** In total, 406 patients have had CTI ablation procedures performed. 24 patients required a repeat ablation procedure bringing a total of 430 procedures. Bidirectional block was achieved in 421 of the 430 procedures (98%). All procedures were performed with only fluoroscopy and no additional modalities, using a decapolar coronary sinus catheter and an ablation catheter. The majority of the procedures were performed using an irrigated 4mm tip ablation catheter and the remainder using a non‐irrigated 8mm tip catheter. 24 patients required a repeat ablation procedure giving a requirement for repeat ablation in this cohort of 6%. One patient had a delayed tamponade which occurred one week post procedure. A second patient had a significant vascular access site bleed but did not require surgical, or other repair, to stop bleeding. There were no other complications.

**Conclusions:** Simple CTI dependent atrial flutter ablation using only two catheters and fluoroscopy is highly effective in achieving bidirectional block. Additional modalities such as 3‐D mapping, or other complex systems have been shown to reduce fluoroscopy times. Additional modalities do not appear to increase the rate of achieving bidirectional block, or of reducing the requirement of repeat procedure, but do add significant extra cost. Simple fluoroscopy guided CTI flutter ablation can be effectively performed with minimal cost and a very low complication rate. CTI ablation therefore can be performed in smaller centres without the need for additional equipment beyond fluoroscopy and a radiofrequency ablation system.

## UNRAVELING THE RHYTHM: A CASE SERIES ON FASCICULAR VENTRICULAR TACHYCARDIA

### **AZEL PAOLO BONDOC**, AMRAPHEL NICOLAS, PAULA VICTORIA CATHERINE CHENG, GISELLE GERVACIO, JHOBELEEN DE LEON, MICHAEL‐JOSEPH AGBAYANI

#### Philippine General Hospital, Manila, Philippines

**Introduction:** Fascicular ventricular tachycardia (FVT), making up 10‐15% of idiopathic ventricular tachycardia from the left ventricle, categorized by ECG patterns, occurs in both normal and abnormal hearts and is often misdiagnosed as supraventricular tachycardia,. Effective management includes calcium antagonists like Verapamil and Diltiazem, with severe or drug‐resistant cases often treated with catheter ablation. This study, the first case series in the Philippines, reviews 2023 cases at the Philippine General Hospital, a tertiary national university hospital.

**Methods:** N/A

**Results:** Case 1: A 50‐year‐old male with no comorbidities developed narrow complex tachycardia after a high‐voltage injury. He was successfully cardioverted and discharged after six months.

Case 2: A 31‐year‐old pregnant at 33 weeks gestation had recurrent palpitations despite Verapamil and Metoprolol. Post‐cesarean, attempted radiofrequency ablation was done however intraoperative complications occurred requiring emergency ventriculorrhaphy, eventually discharged on Verapamil.

Case 3: A 35‐year‐old hypertensive pregnant woman with dyspnea post‐cesarean was treated with Amiodarone and Metoprolol, and discharged after 10 days.

Case 4: A 37‐year‐old male with diabetes and dyslipidemia had successful ablation after initial IV Verapamil.

Case 5: A 31‐year‐old male with palpitations and chest pain, initially diagnosed as NSTEMI, was stabilized with and discharged after five days with Verapamil.

**Conclusions:** This study highlights the challenges of diagnosing and managing idiopathic fascicular ventricular tachycardia, mainly affecting young males aged 15‐40. The most common subtype is the left posterior fascicular, showing RBBB and right axis deviation on ECG. Triggers like pregnancy and burn injuries emphasize the need for monitoring. Acute episodes respond well to intravenous antiarrhythmics, but long‐term control with oral medications varies. Radiofrequency ablation is crucial, despite potential complications. A tailored approach is essential for optimizing outcomes.

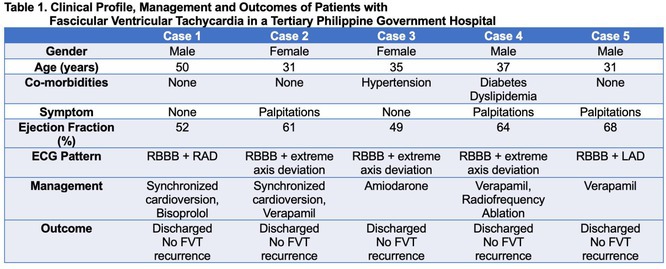

## CATHETER ABLATION OF A LEFT VENTRICULAR SUMMIT ARRHYTHMIA IN A FEMALE PATIENT

### **PAULA VICTORIA CATHERINE BROMEO**, GISELLE GERVACIO

#### Philippine General Hospital, Manila, Philippines

**Introduction:** The left ventricular (LV) summit is an infrequent location of idiopathic premature ventricular complexes (PVC).

**Methods:** N/A

**Results:** A 38‐year old female patient presented with a 20‐year history of dyspnea on exertion with associated palpitations and chest pain described as pinprick sensation. On work up with 24‐hour holter monitoring, she was noted to have high frequency PVCs (1000/hour). Treadmill stress test was done to further characterize the PVC morphology. The treadmill test showed high frequency monomorphic PVCs with inferior axis and early precordial transition in V1. An electrophysiology study and subsequent ablation procedure was performed. Activation mapping guided by a three‐dimensional electroanatomic system was performed to identify the earliest site of activation. The earliest ventricular activation was observed in the distal coronary sinus catheter (CS 1,2) when the CS catheter was advanced anteriorly. Ablation was initially attempted on the aortic cusp but the PVCs persisted. Subsequently, ablation was done near the LV summit on the endocardial surface of the LV outflow tract with the radiofrequency current delivered for 180 seconds with a maximum power of 35 watts. Post‐ablation, recurrence of the PVCs were not observed. On subsequent follow‐up, there was no note of recurrence of the patients symptoms nor PVCs during holter monitoring.

**Conclusions:** Prolonged delivery of radiofrequency energy is a viable method for ablation of left ventricular summit arrhythmias.

## CATHETER ABLATION OF CONCURRENT FOCAL ATRIAL TACHYCARDIA AND A CAVOTRICUSPID ISTHMUS DEPENDENT ATRIAL FLUTTER IN A POST‐TETRALOGY OF FALLOT REPAIR PATIENT

### **PAULA VICTORIA CATHERINE BROMEO**, GISELLE GERVACIO

#### Philippine General Hospital, Manila, Philippines

**Introduction:** Arrhythmias in adults with repaired congenital heart diseases are not uncommon. However, catheter ablation of these various arrhythmias can be challenging due to the changes in the usual anatomy of these patients. We report of a case where in two concurrent arrhythmias was treated with catheter ablation in a patient who had previously undergone repair for a congenital heart condition.

**Methods:** N/A

**Results:** A 40‐year old female presented with a 2‐year history of palpitations. She was previously diagnosed to have Tetralogy of Fallot which was repaired when she was six years old. However, 2 years prior to consult, she presented with palpitations, on workup, noted a regular wide complex tachycardia. On workup, baseline ECG showed sinus rhythm with complete right bundle branch block. Tachycardic episodes revealed a supraventricular tachycardia with aberrant conduction, considering atrial flutter. She underwent electrophysiologic study with electroanatomic mapping. Initially, a focal atrial tachycardia was induced near the crista terminalis, presumably from a previous atriotomy site during her TOF repair. Subsequent ablation of the earliest activation of the atrial tachycardia was done with no recurrence. However, post‐ablation, a cavotricuspid isthmus dependent atrial flutter was induced. Subsequent ablation of the CTI was done with demonstration of bidirectional block post‐ablation. No other arrhythmias were induced after the ablation.

**Conclusions:** Post‐congenital heart disease repair patients are at increased risk for arrhythmias due to the changes in the anatomy as well as scar tissue formation brought about by the surgery. Careful evaluation during the electrophysiology study and correlation with the electroanatomical mapping is essential in performing ablation in these patients.

## CARDIAC PUZZLE: BRUGADA ECG PATTERN MIMICKING STEMI IN A 49‐YEAR‐OLD MALE PATIENT WITH CHEST PAIN

### **INDAH CHAERUNNISA**, SUMARNI WAHYUDI, MUZAKKIR AMIR, AKHTAR FAJAR MUZAKKIR

#### Hasanuddin University, Makassar, Indonesia

**Introduction:** Brugada syndrome features a unique ECG pattern, resembling ST elevation in leads V1‐V3 with a right bundle branch block appearance. This genetic condition predisposes individuals to dangerous ventricular arrhythmias, often leading to syncope or cardiac arrest.

**Methods:** N/A

**Results:** We present a case of a 49‐year‐old male who presented with substernal chest pain. His medical history was devoid of syncope episodes, and physical examination yielded no notable findings. Electrocardiography (ECG) demonstrated ST elevation in the right precordial leads (V1‐V3) with T‐wave inversion, initially suggestive of ST‐elevation myocardial infarction (STEMI). However, urgent cardiac catheterization revealed normal coronary arteries, and echocardiogram results were unremarkable. Subsequent analysis of the ECG revealed a Brugada type 1 pattern, characterized by a coved‐type, gradually descending ST‐T segment, elevated J point (>2 mm), and T‐wave inversion. Despite the presence of this pattern, the patient had no prior history of syncope and remained asymptomatic. Consequently, he was deemed at low risk for future serious arrhythmic events.

**Conclusions:** while a history of syncope remains a crucial predictor for arrhythmic events, the utilization of electrophysiology study testing for risk stratification in asymptomatic patients with Brugada pattern remains contentious. Implantable cardioverter‐defibrillator placement in asymptomatic, non‐inducible individuals with the Brugada pattern is presently not recommended. These patients should undergo regular follow‐up with a cardiologist and be educated about potential triggers for ventricular arrhythmias.

## ELECTRICAL IMPRINTS

### CHUN YIN, VICTOR CHAN

#### Tuen Mun Hospital ‐ Hospital Athority, Tuen Mun, Hong Kong

**Introduction:** Cardiac memory refers to heart's adaptation to abnormal electrical activation, with the exact mechanism being uncertain. The hallmark of cardiac memory is the inversion of T‐waves during repolarization. While typically benign, cardiac memory can occasionally resemble severe pathological conditions such as myocardial infarction, as illustrated in the following case.

**Methods:** N/A

**Results:** Miss D, a 76‐year‐old female, has a medical history of hypertension, hyperlipidemia, and stable coronary artery disease. She underwent a percutaneous coronary intervention in 2014 where a stent was placed in the proximal right coronary artery. In 2020, she was diagnosed with atrial fibrillation with slow ventricular response, for which she was started on Apixaban for thromboprophylaxis and had a dual‐chamber permanent pacemaker implanted.

In November 2023, Miss D presented with central chest discomfort. The initial ECG displayed an atrial fibrillation rhythm with a predominance of ventricular pacing. The initial high‐sensitivity troponin I level was elevated at 166 ng/L. A follow‐up ECG revealed a sinus rhythm with intrinsic ventricular activity, showing diffuse ST depression and T wave inversion in the precordial and inferior leads, and ST elevation in lead aVR.

Given her persistent chest pain and worrying ECG findings, an emergency coronary angiogram was performed. It showed minor disease in the proximal left anterior descending artery and ostial right coronary artery, and mild in‐stent restenosis in the previously placed stent in proximal right coronary artery. These findings did not correlate with the ECG abnormalities, which were later attributed to the cardiac memory phenomenon. No percutaneous intervention was performed, and medical management for her ischemic heart disease was continued.

Due to ongoing chest discomfort, an OGD was performed, revealing multiple esophageal ulcers. A rapid test confirmed H pylori infection. She was treated accordingly, and remained asymptomatic in the most recent follow‐up.

**Conclusions:** Cardiac memory can resemble ECG signs of serious condition like myocardial infarction. Recognizing this phenomenon is crucial for accurate clinical assessment and management.

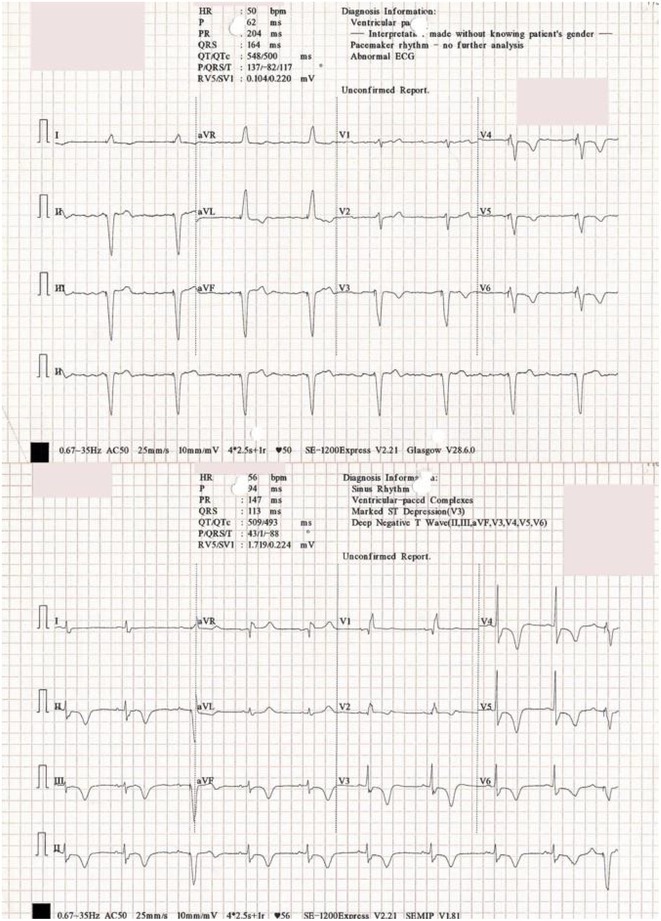

## ATRIAL FLUTTER AS THE INITIAL PRESENTATION FOR A CHILD WITH SCN5A OVERLAP SYNDROME

### **JIAHUI CHARMAINE CHAN**, AMANDA XINYI YAP, NURHAFIZAH BINTE ABDUL AZIZ, TENG HONG TAN

#### KKH, Singapore, Singapore

**Introduction:** We report a case of a child with *SCN5A* overlap syndrome who first presented with recurrent atrial flutter from the age of 2 years 7 months old.

**Methods:** N/A

**Results:** A 2 year 7‐month‐old boy first presented with atrial flutter, ventricular rate 220 beats per minute (bpm), 2:1 conduction ratio. He had a past history of Kawasaki Disease at 5 months old, complicated by small coronary aneurysms, which regressed to normal, for which he was on long term aspirin. He underwent successful electrical cardioversion and was started on oral propranolol. Echocardiography showed structurally and functionally normal heart, with no thrombi seen. A year later, he developed recurrent atrial flutters, responding to electrical cardioversion. Trial of adding on oral flecainide to propranolol revealed Type 1 Brugada pattern in leads V1/V2 after the second dose, and he also developed symptomatic sinus pauses. Flecainide was immediately stopped with resolution of the sinus pauses and Brugada pattern. He was diagnosed with Brugada syndrome (BrS). Genetic testing confirmed pathogenic heterozygous mutation in SCN5A gene c.1890G>A. He developed chronic atrial flutter, which was rate controlled by oral sotalol. At 4 years old, he was admitted for episodes of symptomatic bradycardia, lowest heart rate in the 30s, improving with isoprenaline infusion. Sinus node dysfunction (SND) was diagnosed, and he underwent dual chamber permanent pacemaker insertion, AAI mode, lower rate limit 70 bpm. In view of the presence of BrS and SND, he was diagnosed with *SCN5A* overlap syndrome. Since the pacemaker insertion, he has been mostly stable in sinus rhythm, with infrequent episodes of atrial flutter that can be converted by atrial overdrive pacing.

**Conclusions:** *SCN5A* mutations can cause varied phenotypes, such as Long QT syndrome, BrS, SND, dilated cardiomyopathy. A single mutation causing mixed phenotypes is termed as *SCN5A* overlap syndrome. It is the second most common phenotype in children with *SCN5A* mutation, next to progressive cardiac conduction defects. Diagnosis of atrial flutter in children beyond the neonatal / infantile age group is rare, and a primary arrhythmia syndrome should be considered.

## A STRATEGIC APPROACH TO TERMINATING ATRIAL FIBRILLATION WITH POST‐MITRAL VALVE REPLACEMENT

### CLARANCE CHANDRAN

#### The First Affiliated Hospital of Dalian Medical University, Dalian, China

**Introduction:** Mitral valve (MV) disease and atrial fibrillation (AF) with reduced left ventricular ejection fraction (LVEF) are increasingly prevalent and associated with high morbidity, mortality. A decade of data demonstrate promising effects on drug‐resistant AF, LVEF, and health care cost improvement following intracardiac electrophysiological examination (ICEP)‐based catheter ablation for AF in patients with post‐MV replacement (MVR). **We sought to study the relationship between LVEF, New York Heart Association (NYHA) class presentation, and the end points of mortality and recurrence of AF admissions in the north‐east Chinese population. Furthermore, predictors for AF were examined**.

**Methods:** In 90 patients (mean age, 58±11 years), post‐MVR and AF were analyzed in a single‐center prospective controlled fashion to compare catheter ablation (n = 45) versus pharmacological therapy (n = 45). LVEF, ECG, and NYHA class were assessed at baseline and at each follow‐up of 24 months.

**Results:** In the ablation group, a significantly higher number of patients experienced an improvement in their LVEF. Compared with the pharmacological therapy group. 35 (77%) patients in ablation group median basline 30% or moderate/severe (≥20% and <35%) LVEF had a significant, and NYHA class I/II patients at the time of treatment had the strongest improvement in clinical outcomes (primary end point: HR, 0.43; *P*<0.001; mortality: HR, 0.30; *P*=0.001). Among 45 patients in the ablation group, 27 (60%) had surgical MVR with CA targetted only pulmonory vein (PV) segments and mitral isthmus (MI), AF terminated in 9 of 27, 6 (13%) patients had repeat ICEP CA, and 18 (40%) had machanical MVR with ICEP ablation targetted PVs,MI,based on ICEP conduction, were found in different segments, terminated AF accordingly, and no recurrence of AF was found.

**Conclusions:** ICEP‐based AF ablation was associated with a significant improvement in common baseline cardiac characteristics. And it should be the basis for resoring the sinus rhythm in AF patients with post‐MVD.

## EARLY DETECTION OFATRIAL CARDIOMYOPATHY AND THE RISK OF CARDIOVASCULAR EVENTS IN PATIENTS WITH METABOLIC SYNDROME: A PROPENSITY SCORE‐MATCHED POPULATION‐BASED COHORT STUDY

### **CLARANCE CHANDRAN**, LIU FEI, SHABNAM FAIZ

#### The First Affiliated Hospital of Dalian Medical University, Dalian, China

**Introduction:** Atrial cardiomyopathy (ACM) as an abnormal atrial substrate was related to many cardiovascular pathologies. This study aimed to investigate whether ACM could be the early predictor of cardiovascular (CV) risk in patients with new‐onset metabolic syndrome (Mets).

**Methods:** Patients with new‐onset Mets were enrolled in this retrospective cohort study, and excluded when patients with CV diseases (CVDs) at baseline. Participants were divided into ACM (n=8222) and non‐ACM groups (n=7306). Propensity score matching (PSM) was performed to match ACM patients with non‐ACM patients in a 1:1 ratio. Cox proportional hazards regression model was established, were used to assess the predictive value of ACM on the overall rehospitalization related to CVDs and competitive (AF, IS, CHD, HF, and). A comparison of the first hospital claim for a CV event was done to evaluate long‐term outcomes using a Cox proportional hazards regression. and the hazard ratios(HR) at 95% confidence interval (CIs).

**Results:** ACM happened to 52.9% of all firstly diagnosed MetS patients. After 33.2±20.6 months’ follow‐up period, 7468 (48.1%) patients experienced once admission for CVDs. The incidences of readmission for CVDs in patients with and without ACM were 52.3% and 43.4%. Multivariate Cox regression model showed that ACM [hazard ratio= 1.20, 95% confidence interval= 1.14‐1.27, P < 0.001] was independently associated with the incidence of admission for CVDs. The restricted mean survival time indicated that individuals were expected to live free of admission for 34.08 months (95% CI, 33.68‐34.49) and 36.96 months (95% CI, 3655‐37.37) if they are with and without ACM in Mets.

**Conclusions:** ACM was common in newly diagnosed Mets patients. Patients with ACM were more likely to happen to admission for CV events and should be monitored more closely.

## ANTIBACTERIAL ENVELOPE PREVENTS CARDIAC IMPLANTABLE ELECTRONIC DEVICE INFECTIONS:THE LARGEST ASIA REAL WORLD DATA

### **CHING‐FEN CHANG**, YEN‐NIEN LIN

#### China Medical University Hospital, Taichung, Taiwan

**Introduction:** Cardiac implantable electronic device (CIED) infection is one of the most serious complications of CIED therapy associated with increased morbidity, mortality, and healthcare cost. The use of absorbable antibiotic‐eluting envelope (TYRX™, Medtronic, Minneapolis, US) has been reported to reduce the risk of CIED infection without an increased risk of additional complications. To investigate the real‐world efficacy on Taiwanese patients, we retrospectively review the outcomes of CIED patients with and without envelope use during procedure.

**Methods:** All 456 patients underwent CIED procedure since January 2022 to December 2023, including initial implantation, generator replacement, upgrade, or revision. 154 cases were treated with envelope in consideration of medical condition, operator's choice, or financial availability. The envelope group and the other 302 cases as control group both received our standard‐of‐care infection prophylaxis. Patient demography and CIED complication during serial clinical follow‐ups were analyzed.

**Results:** Both groups demonstrate comparable characteristics including age, sex, BMI, CIED type, and left ventricular ejection fraction. The envelope group consists of more dialysis (Envelope vs Control: 14% vs 7%, p=0.015) and anticoagulant use (Envelope vs Control: 36% vs 17%, p=0.048) patients with longer procedure time (Envelope vs Control: 83.42±40.3 vs 70.36±31.7 minutes, p=0.0002) needed. Major CIED‐related infections (defined as those resulted in CIED system removal) occurred in 0 Envelope patients and 4 control patients (0% vs 1.3%, p=0.19). There were no any lead dislodgment complications in Envelope group, but 2 cases happened in control group (0.6%). No cases reported allergic reactions to the components of Envelope.

**Conclusions:** In our study, the Envelope group consists of subjects with higher risk of CIED infection. Even though the bio‐absorbable antibiotic envelope demonstrated its lower occurrence of CIED related infection without an increased risk of system or procedure related complications.

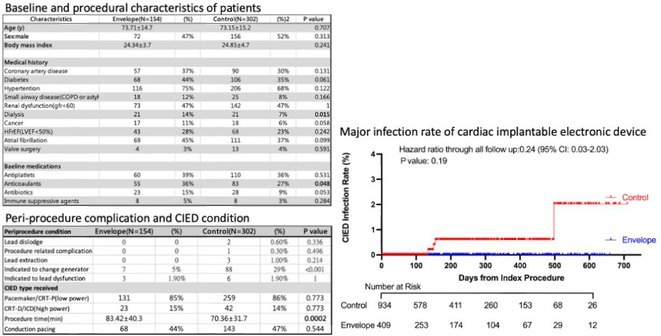

## EFFECT OF VERICIGAUT ON CARDIAC STRUCTURAL AND ELECTRICAL REMODELING IN POST‐MI HF ANIMAL MODEL

### **TING‐YUNG CHANG** ^1^, SHIH‐LIN CHANG^1^, SHIH‐ANN CHEN^2^

#### ^1^Taipei Veterans General Hospital, Taipei, Taiwan,^2^Taichung Veterans General Hospital, Taichung, Taiwan

**Introduction:** Although new therapies and management strategies have emerged, the prognosis for patients with heart failure (HF) remains poor. It is estimated that only 50% of patients survive after 5 years from their initial diagnosis. Sudden death is the common mode of death in patients with HF but its underlying mechanisms are not fully elucidated. In VICTORIA trial, vericiguat reduced the incidence of death from cardiovascular causes or hospitalization for heart failure in patients with high‐risk heart failure. However, the effect of vericiguat on ventricular arrhythmia has not been explored. In this study, we aimed to investigate the effect of vericiguat on ventricular arrhythmia and possible mechanism in the animal of post‐MI HF.

**Methods:** Sprague‐Dawley (SD) rats (n=6 per group) were assigned to normal (group 1), HF (4 weeks of left ascending artery [LAD] ligation, group 2), vericiguat (10 mg/kg/day for 3 weeks after HF, group 3). Experiments involving electrocardiography, echocardiogram and VA inducibility were performed and analyzed.

**Results:** After 4 weeks of LAD ligation, HF was confirmed with decreased ejection fraction by echocardiography (fractional shortening in M‐mode). After 7 weeks, fractional shortening was 53% in group 1, 35% in group 2 and 54% in group 3. ECG parameters showed similar QRS duration among 3 groups, QTc interval was 0.19 seconds in group 1, 0.25 seconds in group 2 and 0.17 seconds in group 3. The VA inducibility were 2% in group 1, 28% in group 2, and 7% in group 3. Figure (A) showed the comparison of fractional shortening among 3 groups, and figure (B) and (C) showed QTc interval and VA inducibility among 3 groups.

**Conclusions:** In post‐MI HF animal model, treatment of vericiguat was associated with improved heart function, shortened QT interval and a trend in reduced VT inducibility.

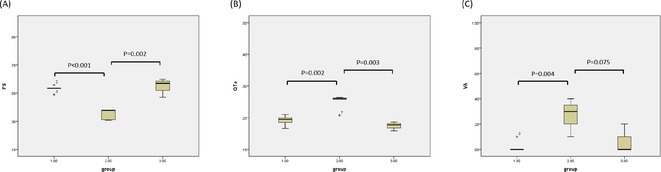

## ASSOCIATIONS OF BODY WEIGHT, BODY MASS INDEX AND CLINICAL OUTCOMES OF EDOXABAN THERAPY IN ATRIAL FIBRILLATION PATIENTS FROM 4 ASIAN REGIONS IN THE ETNA‐AF PROGRAM

### TZE‐FAN CHAO

#### Taipei Veterans General Hospital and National Yang Ming Chiao Tung University, On behalf of the Global ETNA Study Group, Taipei, Taiwan

**Introduction:** The associations between body weight (BW), body mass index (BMI) and clinical outcomes in anticoagulated Asian patients with atrial fibrillation (AF) are unclear.

**Methods:** We analyzed 2‐year data of Korea(KR), Taiwan(TW), Hong Kong(HK) and Thailand(TH) ETNA‐AF program to study the impacts of baseline BW and BMI on clinical outcomes in Asian AF patients treated with edoxaban.

**Results:** A total of 3,299 patients were enrolled from the 4 Asian countries with a mean BW and BMI of 65.9 kg and 25.1 kg/m^2^, respectively, which were lower than that of global ETNA enrollees (BW 72.2kg; BMI 26.4 kg/m^2^). The percentages of patients with a BW >100kg and BMI >27.5kg/m^2^ were much lower in Asian countries compared with global (BW 0.7% vs 6.5%; BMI 22.7% vs 34.5%). Among Asian patients, the annual risk of stroke gradually decreased from low to high BW groups (≤40 kg 2.8%/yr, >40 to 60 kg 1.3%/yr, >60 to 80 kg 1.0%/yr, >80 to ≤100 kg 0.7%/yr, >100 kg 0%/yr). For the risk of major bleeding, a “paradox” phenomenon was observed (higher risk of major bleeding in groups of ≤40 kg [1.4%/yr] and >100 kg [2.5%/yr] compared with other groups). A higher risk of intra‐cranial hemorrhage (ICH) (1.4%/yr) was observed in BW≤40 kg group than other groups. For risks of clinical events in different BMI categories, patients with a lower BMI (<18.5 kg/m^2^) were associated with the highest risks of stroke (2.3%/yr), major bleeding (1.8%/yr) and ICH (0.6%/yr) compared with other groups.

**Conclusions:** Asian AF patients had a lower BM and BMI than global enrollees in ETNA‐AF program. Anticoagulated Asian AF patients having a lower BW or BMI were high‐risk population associated with a high risk of stroke, major bleeding and ICH.

## EFFECTIVENESS AND SAFETY OF FRAIL PATIENTS WITH ATRIAL FIBRILLATION TREATED ON EDOXABAN EITHER ORAL ANTICOAGULANT NAïVE OR EXPERIENCED: A SUBGROUP ANALYSIS OF THE ETNA‐AF‐CHINA STUDY

### XUEYUAN GUO^1^, JUAN DU^2^, JIAFU WEI^3^, JUN ZHANG^4^, HAIYAN LI^5^, WEIHONG JIANG^6^, XINWEN MIN^7^, JIE LIU^8^, PENG GAO^9^, LIANJUN GAO^10^, YUAN YI^2^, CATHY CHEN^11^, MARTIN UNVERDORBEN^11^, QIANG LV^1^, CHANGSHENG MA^1^, **TZE‐FAN CHAO** ^11^

#### ^1^Beijing Anzhen Hospital, Capital Medical University, Beijing, China,^2^Daiichi Sankyo (China) Holdings Co., Ltd, Shanghai, China,^3^West China Hospital, Sichuan University, Chengdu, China,^4^Suzhou Municipal Hospital, Suzhou, China,^5^Changzhou No.2 People's Hospital, Changzhou, China,^6^The Third Xiangya Hospital of Central South University, Changsha, China,^7^Dongfeng General Hospital of Chinese Medicine, Shiyan, China,^8^The First People's Hospital of Nanning, Nanning, China,^9^Peking Union Medical College Hospital, Beijing, China,^10^The Fist Affiliated Hospital of Dalian Medical University, Dalian, China,^11^Daiichi Sankyo Inc., Basking Ridge, NJ

**Introduction:** In patients with atrial fibrillation (AF), frailty is associated with increased risks of stroke/systematic embolic events (SEE) and major bleeding. Clinical benefits of edoxaban in frail patients who were oral anticoagulant (OAC) naïve or have experienced OAC remain unclear.

**Methods:** The prospective, observational ETNA‐AF‐China study (same design as the ETNA‐AF Global programme) enrolled 5001 AF patients treated with edoxaban 60mg/30mg OD across the Chinese Mainland. Frail patients were classified as: 1) 4+ Gencer high‐risk factors (N=1365; Am Heart J. 2022May:247:24‐32), 2) CHA_2_DS_2_‐VASc score ≥4 (N=1478), 3) mFI‐5 ≥2 (N=1266); and further categorized as OAC (including VKA and DOACs) naïve treated with edoxaban, and OAC experienced.

**Results:** Overall, 4883 patients who completed 1‐year follow‐up were analysed. Of the frail patients, 61.2% of 4+ Gencer high‐risk, 39.0% of CHA_2_DS_2_‐VASc score ≥4, 37.8% of mFI‐5 criteria were OAC naïve. Frail patients had a higher risk of all stroke, major bleeding events, and all‐cause death across the 3 criteria; higher risk of cardiovascular (CV) death in 4+ Gencer high‐risk patients (Figure). In frail patients, edoxaban use in OAC naïve vs experienced reduced stroke/SEE/transient ischaemic attack (TIA), ischaemic stroke, major adverse cardiovascular events (MACE), all‐cause and CV death, net clinical outcomes (stroke, bleeding combined, or stroke, bleeding, death combined) by 4+ Gencer high‐risk criteria. In CHA_2_DS_2_‐VASc score ≥4 and mFI‐5 frail patients, OAC naïve treated with edoxaban tend to reduce the risk of stroke/SEE/TIA, MACE and CV death. There were no significant differences in major bleeding and major or clinically relevant non‐major bleeding between edoxaban use in OAC naïve or experienced.

**Conclusions:** In real‐world clinical care of frail patients with AF, edoxaban treatment in OAC naïve vs other OAC experienced had relatively better clinical benefits on stroke, CV death, and net clinical outcomes. The major bleeding risk were similar regardless of OAC naïve or experienced.

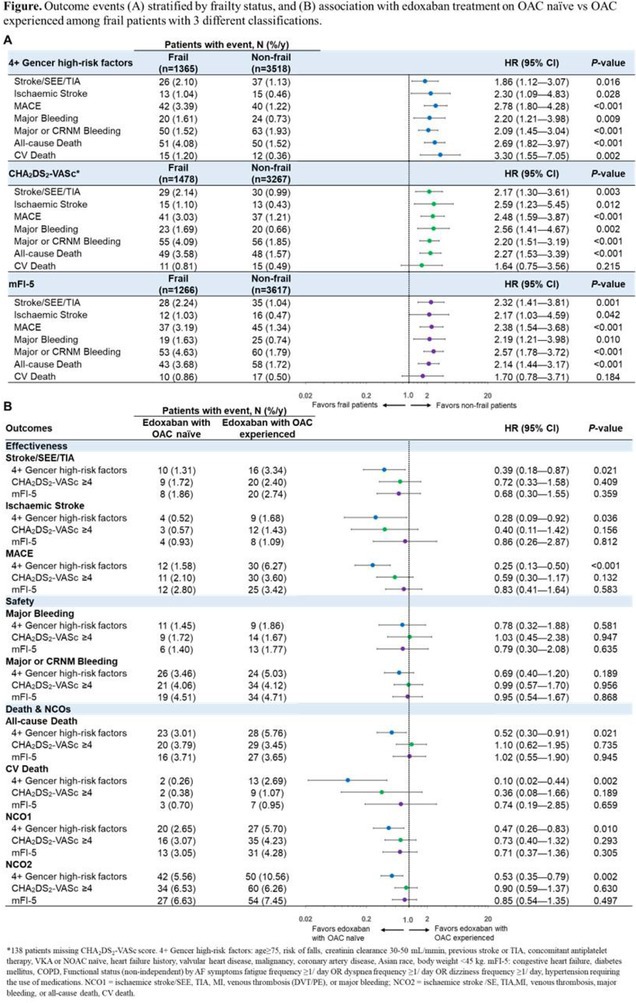

## THE RISK OF MAJOR BLEEDING IN TAIWANESE ATRIAL FIBRILLATION PATIENTS WITH CREATININE CLEARANCE OF 15 TO 30 ML/MIN AND 30 TO 50 ML/MIN RECEIVING RIVAROXABAN ‐ REPORT OF THE PROSPECTIVE XARETO REGISTRY

### **TZE‐FAN CHAO** ^1^, KUAN‐CHENG CHANG^2^, CHENG‐I CHENG^3^, CHING‐PEI CHEN^4^, JU‐YI CHEN^5^, PAO‐HSIEN CHU^6^, ZHIH‐CHERNG CHEN^7^, TSUNG‐HSIEN LIN^8^, YEN‐BIN LIU^9^, CHENG‐TING TSAI^10^, HAN‐LIN TSAI^11^, TIEN‐PING TSAO^12^, WEN‐JONE CHEN^13^, SHIH‐ANN CHEN^14^

#### ^1^Taipei Veterans General Hospital, Taipei, Taiwan,^2^China Medical University Hospital, Taichung, Taiwan,^3^Chang Gung Memorial Hospital Kaohsiung, Kaohsiung, Taiwan,^4^Changhua Christian Hospital, Changhua, Taiwan,^5^National Cheng Kung University Hospital, Tainan, Taiwan,^6^Chang Gung Memorial Hospital, Taoyuan, Taiwan,^7^Chi‐Mei Medical Center, Tainan, Taiwan,^8^Kaohsiung Medical University Hospital, Kaohsiung, Taiwan,^9^National Taiwan University Hospital, Taipei, Taiwan,^10^Mackay Memorial Hospital, Taipei, Taiwan,^11^Ditmanson Medical Foundation Chia‐Yi Christian Hospital, Chiayi, Taiwan,^12^Cheng‐Hsin General Hospital, Taipei, Taiwan,^13^Min‐Sheng General Hospital, Taoyuan, Taiwan,^14^Taichung Veterans General Hospital, Taichung, Taiwan

**Introduction:** Data about the use of direct oral anticoagulants (DOACs) in the prospective registry of Asian atrial fibrillation (AF) patients with a creatinine clearance (CCr) between 15 to 30 ml/min are limited. This study aimed to investigate and compare the risk of major bleeding in Taiwanese AF patients with a CCr of 15‐30 ml/min and 30‐50 ml/min receiving rivaroxaban.

**Methods:** XARETO (**Xa**relto for the prevention of stroke and non‐central nervous systemic embolism in non‐valvular atrial fibrillation with **RE**nal impairment in **T**aiwanese p**O**pulation) is a multi‐center, non‐interventional, single arm prospective observational cohort study performed in Taiwan. Subjects with non‐valvular AF aged >20 years with a documented CCr of 15‐50 ml/min within 6 months before the enrollment were enrolled. All subjects were prescribed rivaroxaban for stroke prevention.

**Results:** A total of 493 patients (mean age 78.3 years old, male 59.6%) were enrolled with a mean CCr of 35.19 ml/min. There were 159 (32.3%) and 334 (67.7%) patients having a CCr of 15‐29 ml/min and 30‐50 ml/min, respectively. The distributions of dose of rivaroxaban are as the following: 20mg/day 0.4%, 15mg/day 22.1%, 10mg/day 76.7%. Low‐dosed rivaroxaban was more commonly prescribed for patients having a CCr of 15‐29 ml/min than those with a CCr of 30‐50 ml/min (10mg/day: 91.8% vs 69.5%, 15mg/day: 7.5% vs 29%). During the follow up, there were 4 patients with the adjudicated major bleeding events considered as related to the rivaroxaban treatment (incidence rate 3.8 per 100 person‐years [95% CI 1.0‐9.6]) in CCr 15‐29 mL/min group. In CCr 30‐50 mL/min group, there were 11 patients with the adjudicated major bleeding events considered as related to the rivaroxaban treatment (incidence rate 4.1 per 100 person‐years [95% CI 2.1‐7.4]). The risk of major bleeding was similar between 2 groups (**Figure**).

**Conclusions:** In Taiwan AF patients with stage IV chronic kidney disease (CCr 15‐29 mL/min), the safety of rivaroxaban was confirmed in the prospective XARETO study.

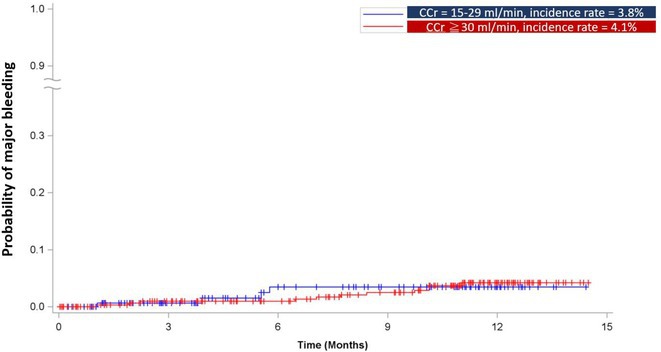

## THE CLINICAL CHARACTERISTICS OF THROMBUS FORMATION IN LOW CHA2DS2‐VASC SCORE PATIENTS UNDERGOING CATHETER ABLATION FOR ATRIAL FIBRILLATION: A RETROSPECTIVE COHORT STUDY

### **CHUN KAI CHEN** ^1^, HUI CHUN HUANG^2^, YEN BIN LIU^2^

#### ^1^National Taiwan University Hospital, Hsin‐Chu branch, Hsinchu City, Taiwan,^2^National Taiwan University Hospital, Taipei, Taiwan

**Introduction:** Three weeks of consistent oral anti‐coagulant (OAC) use or thrombus detection by transesophageal echocardiogram (TEE) before cardioversion was suggested by the European society of cardiology(ESC) atrial fibrillation (AF) guideline. We aimed to assess the prevalence and patient characteristics of thrombus formation of low CHA2DS2‐VASc score patients in Taiwan with routine TEE inspection before catheter ablation (CA).

**Methods:** A retrospective cohort study was conducted in a single medical center in Taiwan. The medical records were reviewed and collected from 2015 to 2020. The index TEE images were collected and reviewed by a core laboratory of echocardiogram.

**Results:** From 2015 to 2020, there were a total of 187 AF patients with low CHA2DS2‐VASc score (0 and 1) receiving scheduled AF catheter ablation. Ten patients (5.34%) were excluded from the procedure due to dense spontaneous echo contrast (SEC) or thrombus in left atrial appendage (LAA) detected by routine TEE before CA in our center. There was 6 (60%) of these patients did not receive OACs. In those receiving OAC, 2 of them received new OAC and 2 of them received warfarin. A diagnosis of congestive heart failure was significant higher in those patients with thrombus or dense SEC formation. (30% vs 4.5%, p=0.0146*) The percentage of persistent AF was higher in the thrombus or dense SEC formation group. (50% vs 22%, p=0.056).

**Conclusions:** The chance dense SEC or thrombus formation in LAA in low CHA2DS2‐VASc score patients was not low (5.34%), especially in those with a diagnosis of congestive heart failure. A routine TEE could be essential before AF patients undergoing catheter ablation, despite pre‐ablation OAC or not.

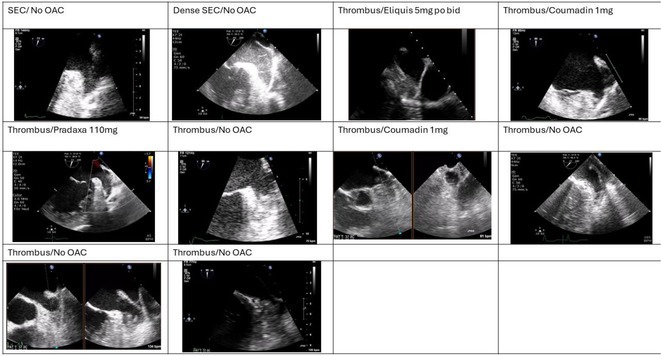

## ACUTE MESENTERIC ISCHEMIA IN AN ATRIAL FIBRILLATION PATIENT SUCCESSFULLY TREATED WITH ENDOVASCULAR THROMBOLYSIS

### **SSU‐YU CHEN** ^1^, CHENG‐AN WANG^2^, JONG‐SHIUAN YEH^2^, MING‐HSIUNG HSIEH^2^

#### ^1^University of New South Wales, Sydney, Australia,^2^Wan Fang Hospital, Taipei, Taiwan

**Introduction:** N/A

**Methods:** N/A

**Results:** A 93‐year‐old female has a medical history of hypertension, atrial fibrillation (AF), heart failure with preserved ejection fraction, and minor intracerebral hemorrhage 10 years ago. During outpatient follow ups, she was on Edoxaban 30mg daily for stroke prevention. The patient ceased her medications for 2 weeks before presenting to the Emergency Department with a 2‐week history of dizziness and 3‐day history of abdominal pain. She reported no gastrointestinal or urological symptoms. On examinations, she had a temperature of 36°C, heart rate of 72 beats/min, respiratory rate of 20 breaths/min, blood pressure of 142/93 mmHg. Murphy sign was negative with no sign of appendicitis. 12‐lead electrocardiogram (ECG) was performed and revealed AF and left ventricular hypertrophy with strain. Serum laboratory results revealed 11.6 g/dL of hemoglobin, 48 U/L of glutamic oxaloacetic transaminase (GOT), 5.9 mg/dL C‐reactive protein (CRP). Urinalysis and KUB revealed no obvious findings. During hospitalization, the pain persisted and the patient's kidney function deteriorated. Abdominal CT revealed a filling defect and low‐density thrombus at the root of the superior mesenteric artery (SMA), and a relatively poor opacification of right renal artery. Thus, SMA infarction with acute mesenteric ischemia was suspected. Enoxaparin 0.5mg was administered subcutaneously 12 hourly. Angiography was performed and a thrombus was located at the ostium of SMA. Thrombolytic therapy was attempted using Ekosonic Endovascular System (EKOS) in SMA with urokinase for thrombolysis. The patient's abdominal pain improved postoperatively and no thrombus was found in SMA on angiography the following day. The patient recovered well with no complications with acceptable renal function. Anticoagulant was resumed and she was discharged one week later.

**Conclusions:** N/A

## IMPROVED ON‐LABLE ORAL ANTICOAGULATION RATE THROUGH HOSPITAL‐WIDE ATRIAL FIBRILLATION SCREENING SYSTEM

### WEITA CHEN

#### Division of Cardiovascular Medicine, Taipei Medical Univeristy Hospital, Taipei Medical University, Taipei City, Taiwan

**Introduction:** Since 2010, direct oral anticoagulation (DOAC) therapy was approved for stroke prevention in atrial fibrillation (AF) patients. The use of DOAC markedly reduces the stroke rate, while keeps the bleeding rate no higher than warfarin. However, about 30% patients with AF were not on oral anticoagulants (OAC). In patients on OACs, about 30% of them were on off‐label dosages. Therefore, we used a hospital‐wide AF screening system to improve the on‐label dosed OAC rate.

**Methods:** Since 2021, we invented a software to screen hospital‐wide electrocardiography (ECG). All ECGs of each hospitalized patients would be screened, no matter the ECGs were obtained during or before the hospitalization. Patients with AF documented by ECG would be enrolled. The CHA2DS2‐VAS score would be calculated and appropriate on‐label dosed DOACs would be suggested to the main caring medical team. The on‐label dosed DOAC prescription rate would be calculated at discharge, at the first month and at the third month after discharge. The annual stroke rate would also be calculated.

**Results:** Before the intervention of this screening system, the on‐label dosed DOAC prescription rate was only 20.6%. After the intervention, the on‐label dosed DOAC prescription rate was markedly improved to 82.5% (Figure A). After discharge, the first month and the third month prescription rate was not decreased. In the first month of engagement of this screening system, only 40.6% patients with AF were on on‐label dosed DOAC before our intervention. However, the number was increased to 70.9% in the 12^th^ month of the engagement (Figure B). The annual stroke rate for patients on on‐label dosed DOAC before our intervention was 2.79%. The annual stroke rate for patients on on‐label dosed DOAC after our intervention was 3.12%. The annual stroke rate for patients on off‐label dosed DOAC was 9.91% (Figure C).

**Conclusions:** Hospital‐wide AF screening system could improve the on‐label dosed DOAC prescription rate and reduce the annual stroke rate for patients with AF.

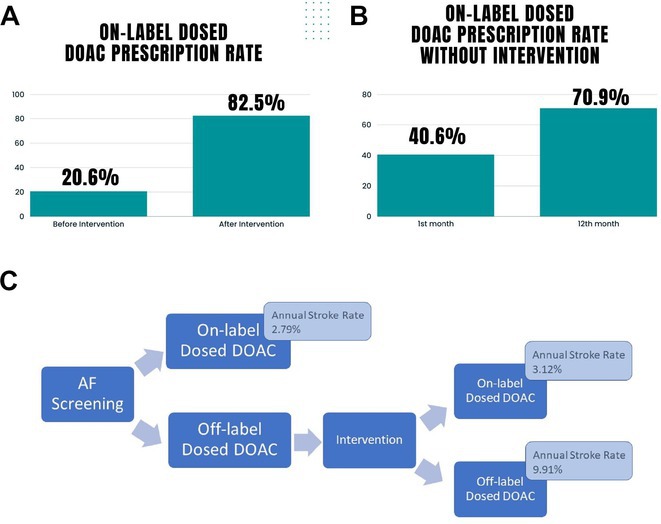

## EMERGENCY STENT IMPLANTATION FOR ACUTE CHEST PAIN DURING VENTRICULAR PREMATURE BEAT RADIOFREQUENCY ABLATION

### YUNING CHEN

#### Zhejiang Province Qiushi Cardiovascular Hospital, Hangzhou, China

**Introduction:** We present the case of a 42‐year‐old female who underwent an emergency percutaneous coronary intervention (PCI) with stent implantation during a radiofrequency catheter ablation (RFCA) procedure for symptomatic frequent ventricular premature contractions (VPCs).

**Methods:** The patient initially presented with recurrent episodes of palpitations and exertional dyspnea, associated with intermittent chest discomfort. Pre‐procedural investigations, including an electrocardiogram (ECG) and echocardiogram, revealed a structurally normal heart but frequent VPCs with an estimated burden of 18,641 beats/day.Upon initiation of the RFCA procedure under sedation, targeted at a focal trigger above left coronary cusp, ventricular premature beat disappeared during ablation,but the patient developed severe chest pain, not consistent with typical procedural discomfort. Immediate urgent coronary angiography.Coronary angiogram revealed a critical thrombotic lesion(total occlusion) in the left anterior descending artery (LAD), previously undiagnosed, likely triggered by the manipulation of catheters during the RFCA attempt.

**Results:** An emergency PCI was promptly performed, with successful deployment of a drug‐eluting stent to restore blood flow in the LAD. Post‐stenting, the patient's chest pain subsided, and ECG changes normalized.The RFCA procedure was deferred until the patient's condition stabilized, and appropriate dual antiplatelet therapy was initiated. Subsequent evaluation confirmed resolution of the acute coronary event, and the patient was discharged with a plan for follow‐up care to reassess the need for further VPC management.

**Conclusions:** This case illustrates the rare but critical complication of RFCA, highlighting the importance of prompt recognition of iatrogenic coronary artery injury and the necessity for immediate interventional response. It also underscores the value of interdisciplinary collaboration between electrophysiologists and interventional cardiologists in managing complex cases where cardiac arrhythmias coexist with occult coronary artery disease.

## INTRACARDIAC ECHO‐GUIDED BIOABSORBABLE PATENT FORAMEN OVALE CLOSURE

### YUNING CHEN

#### Zhejiang Province Qiushi Cardiovascular Hospital, Hangzhou, China

**Introduction:** Patent foramen ovale (PFO), a common congenital cardiac anomaly, is implicated in cryptogenic strokes and other paradoxical embolic events. Traditional closure devices are typically metallic and permanent. This case report details the successful deployment of a bioabsorbable occluder device under intracardiac echocardiography (ICE) guidance in a patient with a recurrent headache history, emphasizing the advantages of bioabsorption and the precision offered by ICE‐guided intervention.

**Methods:** A 28‐year‐old male with a history of recurrent headache with transient ischemic attacks (TIAs). Transesophageal echocardiography (TEE) revealed a large patent foramen ovale (PFO)(Long tunnel type). Right echocardiography hints positive. Given her young age and preference for a less invasive, temporary solution, a decision was made to proceed with a bioabsorbable PFO closure.Procedure under local anesthesia, and an ICE catheter was introduced to provide real‐time imaging guidance. The bioabsorbable PFO occluder, designed to degrade over time and minimize long‐term device‐related complications. The device's position and stability were verified, ensuring complete closure of the defect without impinging on surrounding structures.

**Results:** Post‐procedural ICE confirmed adequate closure with no residual shunt, and the patient experienced an uneventful recovery. At the 6‐month follow‐up, transthoracic echocardiography (TTE) showed continued PFO closure, and the patient reported no further neurologic events, suggesting successful prevention of future embolic incidents.

**Conclusions:** The use of bioabsorbable technology in PFO closure represents a paradigm shift towards minimally invasive and potentially more physiological interventions. Intracardiac echocardiography offers superior visualization compared to fluoroscopy alone, which is particularly beneficial in complex anatomies or when aiming for a minimally invasive approach.This case illustrates the feasibility and efficacy of a bioabsorbable PFO closure under ICE guidance, combining the latest in material science with advanced imaging technology to achieve a safe and efficacious outcome.

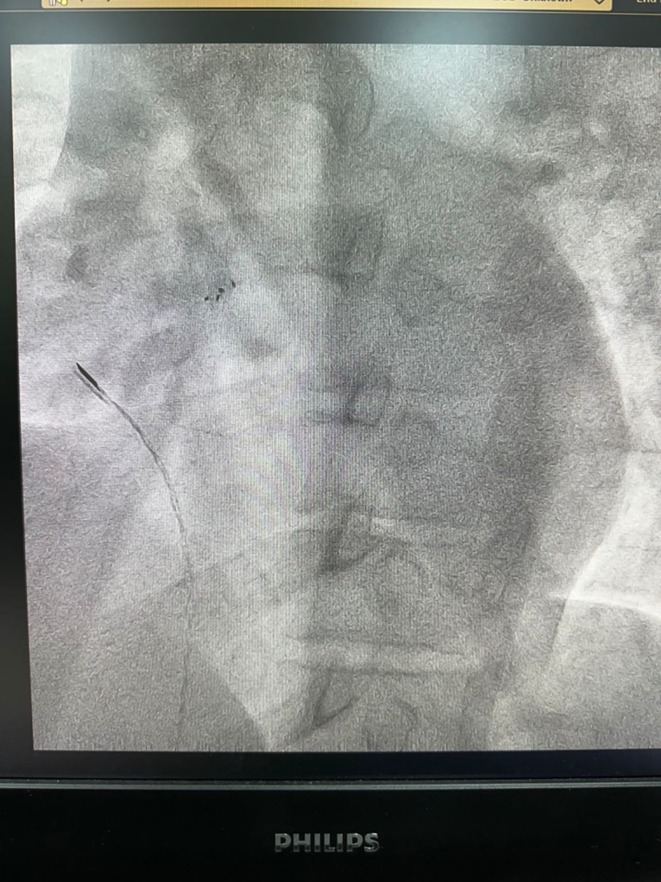

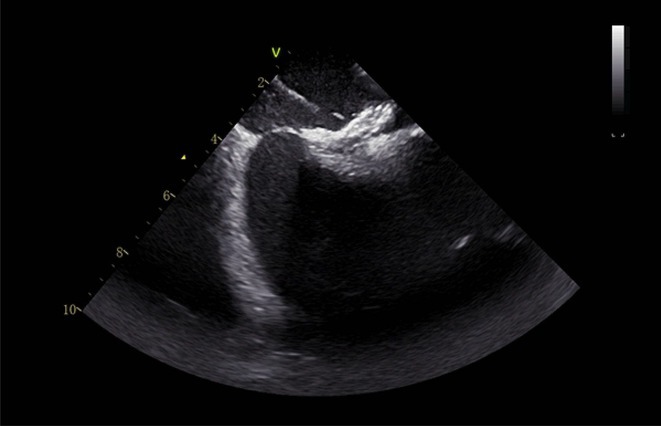

## LONG‐TERM ELECTROCARDIOGRAPHIC MONITORING FOR EARLY RECURRENCE PREDICTION OF LATE ATRIAL FIBRILLATION RECURRENCE FOLLOWING CATHETER ABLATION

### YUNING CHEN

#### Zhejiang Province Qiushi Cardiovascular Hospital, Hangzhou, China

**Introduction:** Catheter ablation is a well‐established therapeutic modality for atrial fibrillation (AF), yet post‐ablation recurrence remains a clinical challenge. This study aimed to investigate the predictive value of early recurrences, detected through extended Holter monitoring, for the occurrence of late AF recurrence following catheter ablation.

**Methods:** We conducted a prospective observational study involving 200 consecutive patients undergoing AF catheter ablation. All participants underwent continuous ambulatory electrocardiographic monitoring for 30 days post‐ablation. Early recurrence was defined as any episode of AF, atrial flutter, or atrial tachycardia lasting over 30 seconds within the first 3 months after the procedure. Late recurrence was identified as a similar arrhythmic event occurring beyond 3 months post‐ablation. Logistic regression analysis was employed to evaluate the association between early recurrence and the risk of late recurrence, adjusting for relevant clinical variables.

**Results:** Among the cohort, 35 patients (17.5%) experienced early recurrence, while 27 (13.5%) had late recurrence during a median follow‐up of 18 months. Early recurrence was significantly associated with an increased likelihood of late recurrence. Notably, patients with persistent AF before ablation and those with higher pre‐ablation levels of high‐sensitivity C‐reactive protein (hs‐CRP) were more prone to both early and late recurrences. Additionally, multivariate analysis confirmed early recurrence as an independent predictor of late AF recurrence.

**Conclusions:** Our findings suggest that early detection of AF recurrence within the first 3 months post‐ablation, through prolonged electrocardiographic monitoring, serves as a valuable predictor for the subsequent development of late recurrence. This underscores the importance of vigilant monitoring in this period and may inform tailored strategies for post‐ablation management and follow‐up, particularly in high‐risk individuals.

## SYNCOPE AS A MANIFESTATION OF TRANSTHYRETIN AMYLOIDOSIS CARDIOMYOPATHY (ATTR‐CM): A CASE REPORT AND REVIEW OF LITERATURE

### YUNING CHEN

#### Zhejiang Province Qiushi Cardiovascular Hospital, Hangzhou, China

**Introduction:** Transthyretin amyloidosis cardiomyopathy (ATTR‐CM) is a rare, underdiagnosed condition characterized by extracellular deposition of misfolded transthyretin proteins in the heart, leading to progressive cardiac dysfunction. Symptomatic presentation can vary widely; however, syncope as an initial manifestation is not commonly appreciated but holds significant clinical implications.

**Methods:** A 59‐year‐old man with a history of hyotension and unexplained syncope presented to the emergency department over the preceding month. These events were accompanied by presyncope symptoms, lasting seconds before complete recovery without neurological deficits. Initial workup, was non‐contributory. Transthoracic echocardiography revealed left ventricular hypertrophy with preserved ejection fraction. Whereas the surface ECG indicates low voltage.Further diagnostic work was pursued.Magnetic resonance imaging (MRI) and late gadolinium enhancement (LGE) showed a characteristic diffuse. Bone scintigraphy with 99mTc‐3also demonstrated increased tracer uptake, supporting systemic amyloidosis.

**Results:** The patient was started on tafamidis, a transthyretin stabilizer, to halt disease progression. Additionally. With optimized medical management and lifestyle modifications, the patient experienced a reduction in syncope episodes, blood pressure is returning to normal and his quality of life improved significantly.

**Conclusions:** Syncope in the context of ATTR‐CM is often multifactorial, attributed to arrhythmias, autonomic dysfunction, and/or hemodynamic compromise secondary to diastolic heart failure. Recognition of syncope as an early indicator of ATTR‐CM is crucial for timely diagnosis and initiation of targeted therapies.

Recurrent syncope should prompt clinicians to consider ATTR‐CM, even in the absence of overt heart failure symptoms. A high index of suspicion, combined with a comprehensive diagnostic approach, is essential for early detection and intervention, ultimately improving outcomes in this challenging patient population.

## PACEMAKER IMPLANTATION FOLLOWING TRANSCATHETER TRICUSPID VALVE REPLACEMENT

### **SING HUEY CHENG**, ALEXANDER DASHWOOD, COLIN MACHADO, JEFFREY ALISON

#### Victorian Heart Hospital, Melbourne, Australia

**Introduction:** In patients who are at high risk for traditional open‐heart surgery, transcatheter tricuspid valve replacement (TTVR) offers an alternative. A number of TTVR devices are undergoing clinical trials, including the VDYNE system. However, there remains a risk atrioventricular (AV) block requiring pacemaker implantation. Delivery of a transvenous pacemaker without crossing the new tricuspid valve (TV) prosthesis poses some challenges.

**Methods:** N/A

**Results:** Two patients with symptomatic torrential tricuspid regurgitation underwent successful VDYNE TTVR at Victorian Heart Hospital ‐ first cases in Australia. Post‐operatively, both had symptomatic high degree AV block which did not recover after two weeks. At a multidisciplinary team discussion, avoidance of crossing the new valve was a priority. Epicardial left ventricular (LV) lead via mini‐thoracotomy was also considered. Ultimately, a decision was made to attempt transvenous insertion of a LV lead via conventional coronary sinus (CS) route in the first instance. This was despite concerns of challenging CS cannulation due to partial coverage by VDYNE frame design (Figure 1A).

Pre‐device planning included a Cardiac Computed Tomography (CT) Scan and intraoperative Transesophageal Echocardiogram (TOE) to define the relationship between the coronary sinus ostia and the TV prosthesis. There were struts from the TV prosthesis close to the CS ostia which were navigated with TOE guidance during CS cannulation. In both cases, a single‐site LV lead through the CS was successfully placed to minimize interaction with the new TV prosthesis. Patients were followed up at 1 month post pacemaker implant and had stable lead parameters.

**Conclusions:** As transcatheter tricuspid valve replacements increase into the future, so will the need for pacemaker implantation for those who develop AV block. There is concern about crossing the TV prosthesis. It is unclear if this concern is justified as there is increasing evidence for conduction system pacing to optimize cardiac resynchronization but requires lead delivery across the TV prosthesis. Pacing in this select subset of patients requires further studies.

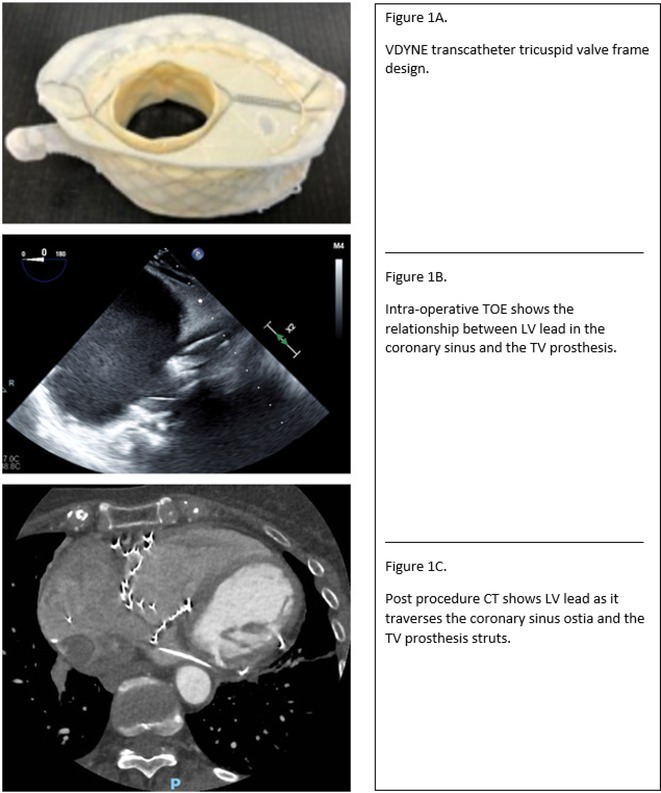

## OUTCOMES OF SUBCUTANEOUS IMPLANTABLE CARDIOVERTER DEFIBRILLATORS: A SINGLE CENTRE EXPERIENCE

### **CALLUM CHERRETT** ^1,2^, JUSTIN PHAN^1,2,3^, WILLIAM LEE^1,2,3^, BRUCE WALKER^1,2,3^, ALASDAIR WATSON^1,2,3^, RAJESH SUBBIAH^1,2,3^

#### ^1^St Vincent's, Sydney, Australia,^2^University of New South Wales, Sydney, Australia,^3^Victor Chang Cardiac Research Institute, Sydney, Australia

**Introduction:** Subcutaneous ICDs (S‐ICDs) are an alternative to transvenous (TV) ICDs for patients who do not require pacing, resynchronisation, or anti‐tachycardia pacing. S‐ICDs are often considered for young, active patients, those with risk factors for TV lead infection and patients with venous access occlusion.

**Methods:** This was a single centre study of all consecutive patients who received a S‐ICD between July 2016 and March 2024. Baseline demographics, indications, implant characteristics and outcomes (including mortality, device therapies and complications) were collected retrospectively from the medical records.

**Results:** Between 2016 and 2024, 132 patients underwent S‐ICD implantation. The average age at time of implant was 49 years and 43/132 patients (33%) required S‐ICD implantation for secondary prevention. Indications for S‐ICD are listed in table 1. Forty‐two patients (32%) underwent defibrillator threshold testing (DFT) and 73/132 (55%) underwent a 10J command shock at implant. Six patients had left ventricular thrombus at time of implant and did not undergo a DFT. At a mean follow‐up period of 595 days, 5 patients died (all non‐arhythmic), 2 patients received left‐ventricular assist devices (LVAD) and 8 (6%) patients underwent cardiac transplantation. Eleven patients (8%) had a previous TV‐ICD which required extraction (either for infection or lead fracture/complication). Five (4%) patients had concurrent pacing devices, with no device interactions noted. One patient with a LVAD had electromagnetic interference requiring S‐ICD deactivation. Ten (7.5%) patients received appropriate therapy from the device, all successful. Six (4.5%) patients had inappropriate shocks, most commonly for T‐wave oversensing. One patient received inappropriate therapy due to air entrapment. One patient required late lead repositioning due to significant weight loss. Six (4.5%) patients required a transvenous device during follow up.

**Conclusions:** Based on our single centre study, S‐ICDs were associated with high efficacy and low complication rate.

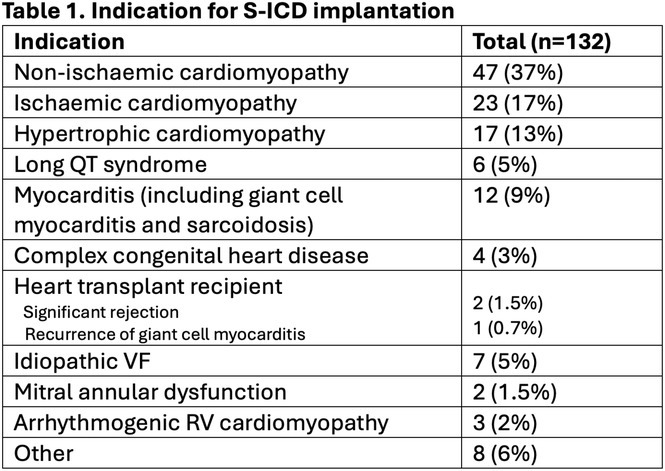

## TRICUSPID VALVE COMPLICATION FOLLOWING LEADLESS PACEMAKER IMPLANTATION REQUIRING DEVICE EXTRACTION

### **CALLUM CHERRETT**, COBI ADAMS, JUSTIN PHAN, WILLIAM LEE, BRUCE WALKER, DAVID MULLER, EMILY GRANGER, RAJESH SUBBIAH

#### St Vincent's, Sydney, Australia

**Introduction:** Leadless pacemakers (PPM) are being increasingly utilised with the hope of avoiding lead complications such as lead fracture, infection and tricuspid valve regurgitation. We present a case of inadvertent implantation of a leadless PPM into the TV subvalvular apparatus causing severe tricuspid regurgitation (TR) requiring extraction.

**Methods:** NA

**Results:** A 67 year old man presented with presyncope and was found to have intermittent 2:1 AV block. His TTE was normal. Given his active lifestyle, including rowing, he underwent a leadless PPM implantation (Micra AV, Medtronic), at another cardiac centre. Pacing was set to VVI 40, with less than 1% pacing at most recent follow up. He presented 3 years later with dyspnoea on exertion. His coronary angiogram revealed severe multi‐vessel disease and he was referred for CABG. A repeat TTE demonstrated normal ventricular function with no significant valvular abnormalities, within the limitations of poor acoustic windows. Transoesophageal echocardiography however revealed mild segmental LV dysfunction and severe eccentric TR due to attachment of the Micra AV to the subvalvular apparatus causing tethering of the septal TV leaflet (Figure 1A and B). During his CABG, he underwent extraction of the Micra AV and TV annuloplasty. A bipolar IS‐1 epicardial lead was placed on the RV and tunnelled up to the left chest wall in anticipation of PPM implantation prior to discharge. However, post‐operatively he had long episodes of non‐sustained VT, and in the context of coronary artery disease and LV dysfunction, decision was made to proceed with a transvenous ICD. During this procedure a DF4 ICD lead was implanted in the RV apico‐septum and an IS‐1 lead implanted into the right atrial appendage. The leads were then connected to a cardiac resynchronization therapy‐defibrillator, with the epicardial RV lead connected to an IS‐1 LV lead port (with the LV lead programmed off), in case of future DF4 lead failure.

**Conclusions:** Leadless pacemakers may be complicated by inadvertent tricuspid valve injury. Given the mechanism of the TV dysfunction, the TR jet may be very eccentric and can be under‐appreciated on TTE.

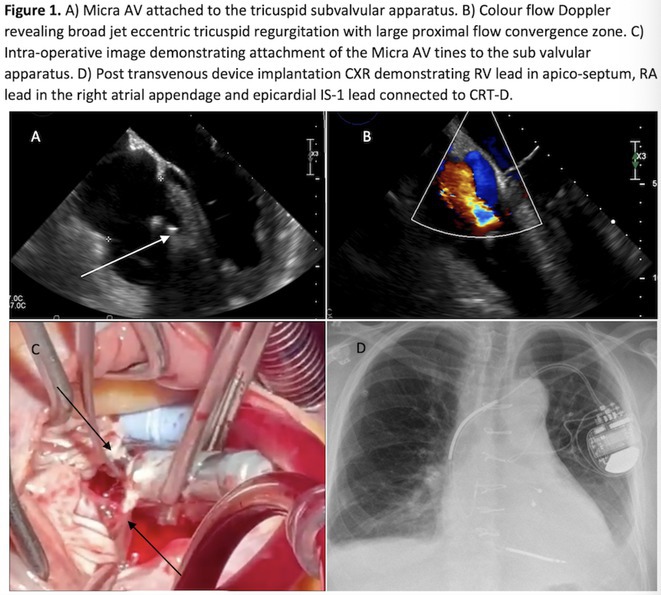

## INTERNATIONAL SURVEY OF ELECTROPHYSIOLOGISTS ON LONG‐TERM CONTINUOUS ECG MONITORING

### **CHRIS CHEUNG** ^1^, DEREK CHEW^2^, DEREK EXNER^2^

#### ^1^Sunnybrook Hospital / University of Toronto, Toronto, ON, Canada,^2^University of Calgary, Calgary, AB, Canada

**Introduction:** Identifying arrhythmias via continuous long‐term ECG monitoring allows for targeted care to improve outcomes (e.g., identification of atrial fibrillation [AF] in patients with a higher risk of stroke can be used to guide oral anticoagulant therapy to reduce recurrent strokes). Patch technologies allow for continuous ECG data collection for 14 to 28 days, but the average time to detect AF after a cryptogenic stroke typically exceeds three months. Thus, clinicians presently are required to choose between shorter duration non‐invasive monitoring or an invasive surgically implanted insertable loop recorder for patients who require long‐term ECG monitoring. However, wearable technologies that can collect continuous ECG data for three months are being developed.

**Methods:** To better understand the clinical need and requirements of these wearable long‐term monitors we conducted a brief anonymous survey of thought leaders and high‐volume heart rhythm specialists to collect their input on the desired duration of continuous ECG monitoring after a cryptogenic stroke and what percent of non‐interpretable data would be acceptable for clinical adoption.

**Results:** Requests to complete the survey were sent to 65 heart rhythm specialists in the United States, Canada, and Asia Pacific regions. A total of 35 (54% of survey requests) responses were received. Practice locations of respondents are summarized in the Figure, A. Most respondents indicated that continuous monitoring for 26 to 52 weeks (46%) or 7 to 25 weeks (26%) was desired (Figure, B). The acceptable percentage of non‐interpretable ECG desired by respondents was < 5% (20%), 5% to 10% (46%), 11% to 20% (26%), or > 20% (9%; Figure, C). Less noise was desired for briefer monitoring. No significant difference in responses was observed when responses were stratified by region.

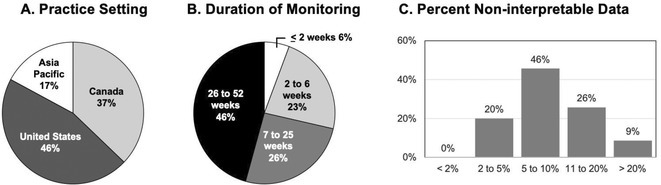

**Conclusions:** Most (72%) of the thought leaders and high‐volume heart rhythm specialist respondents indicated that longer‐term non‐invasive ECG monitoring (i.e., 8 weeks or longer) for AF is desirable after a cryptogenic stroke. Further, most respondents would prescribe a long‐term wearable device with 10% or more non‐interpretable ECG data.

## CARDIONEURAL ABLATION (CNA) AS TREATMENT MODALITY FOR ATRIOVENTRICULAR BLOCK (AVB) AFTER SLOW PATHWAY (SP) MODIFICATION FOR ATRIOVENTRICULAR NODAL REENTRY TACHYCARDIA (AVNRT) IN A YOUNG PATIENT

### **KIT HOU CHIANG**, KENG TAT KOH, LAI KUAN LEONG

#### Sarawak Heart Centre, Kuching, Malaysia

**Introduction:** Pacemaker therapy is the default treatment for symptomatic AVB after SP modification during RFA of AVNRT. However, there is reported successful treatment of AVB with CNA. We report a case of successful treatment with CNA as alternative treatment in a young girl with AVB after RFA of AVNRT.

**Methods:** N/A

**Results:** A 17‐year‐old girl with recurrent SVT for past 2 years was referred to our centre for consideration of EP study and ablation. 12 lead ECG during her symptom showed narrow complex tachycardia (NCT) with rate 195 beats per min (bpm). Her BP and rest of physical examination were normal. She underwent EP study and typical AVNRT with lower common pathway block was induced during atrial extrastimulus testing and isoprenaline infusion. SP was modified and she developed transient complete heart block which recovered to 1^st^ degree AVB. Due to persistent symptom, Kardia mobile was used and showed paroxysmal NCT. She underwent second EP study under 3D Ensite map and typical slow fast AVNRT was again induced. SP was successfully modified. Upon discharge, ECG showed sinus rhythm (SR) with PR interval 135ms. 1 month later, she developed symptomatic 2:1 AVB limiting her daily activity with lowest HR 35 bpm monitored at home. Exercise stress test showed 1:1 conduction during exercise with brief 2:1 conduction during recovery stage. Patient was counselled for CNA. Baseline ECG prior procedure showed 2:1 AVB. CNA was performed with 3D Ensite Precision mapping using the fractionation map. AF nest showing ≥2 deflections in regions which were anatomically consistent with ganglionated plexus (GP) sites were tagged as ablation targets. The GPs were ablated in the following sequence: left superior, Marshal tract, posterior medial and right superior. Post ablation ECG showed SR with HR 89 bpm and PR interval 114 ms. Her symptom completely resolved post ablation. Repeated holter at 3^rd^ month showed average HR 87 bpm, minimum HR 38 bpm (during sleep) and maximum HR 132 bpm during awake. There was transient 2:1 AVB at night during sleep, and patient was asymptomatic.

**Conclusions:** N/A

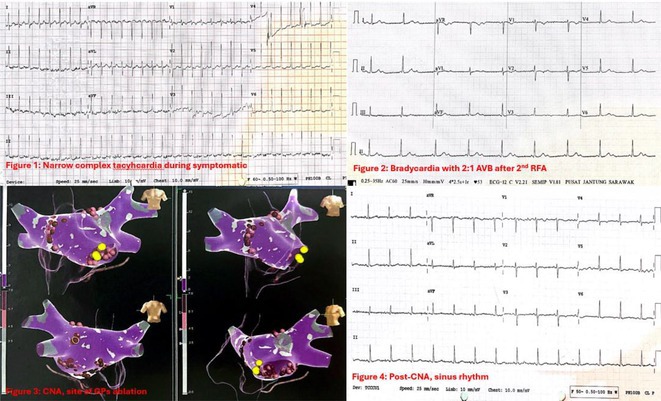

## SINGLE‐CENTRE EXPERIENCE: COMPARING RECURRENCE RATES AND CLINICAL CHARACTERISTICS IN ATRIAL FIBRILLATION PATIENTS UNDERGOING WIDE‐AREA CIRCUMFERENTIAL ABLATION (WACA) VERSUS WACA‐PLUS ADJUNCTIVE ABLATION

### **KIT HOU CHIANG**, KENG TAT KOH, LAI KUAN LEONG

#### Sarawak Heart Centre, Kuching, Malaysia

**Introduction:** Catheter ablation is effective therapy for patients with symptomatic atrial fibrillation (AF), especially in those refractory or intolerants to medical therapy. Conventional technique used is wide area circumferential ablation (WACA). Adjunctive ablation techniques such as performing additional linear lesion is still area of ongoing research. We aim to describe the demographic, clinical characteristics and outcome of symptomatic AF patients undergoing ablation in the only tertiary cardiology centre providing cardiac EP service in East Malaysia

**Methods:** This was a single centre, observational study involving 77 patients (40 males, 60.3±10.4 years) with paroxysmal AF (51) and persistent AF (26) between January 2019 to February 2023 undergoing WACA (71.4%) or WACA‐plus (28.6%) with Medtronic Cryoballoon System, Biosense Webster 3D CARTO System and Abbott 3D EnSite System (Figure 1). Mean follow up was 27±14.6 months. The outcome at 1 year follow up is the occurrence of AF as documented by ECG or holter. Multivariate regression analysis was performed to examine the factors associated with recurrence

**Results:** Mean CHA2DS2VASc was higher for WACA‐plus group, 2.5 vs 2.3 in WACA group (p=0.76). The recurrence rate was 31.5% in WACA group vs 27.3% in WACA‐plus group (p=0.71)(Figure 2). Although there was trend toward reduction in outcome, the observed change did not reach statistical significance. Our cohort showed that age (p=0.04) and LA dimension (p=0.07) were significant predictors of AF recurrence. Mean procedural duration was longer in WACA‐plus group, 192±45min vs 171±60min in WACA group. Mean LV ejection fraction change post ablation was 9.48%±14

**Conclusions:** WACA‐plus exhibited trend towards reduction in AF recurrence. The observation underscores the complexity of AF management and the importance of individualised treatment based on patient characteristics and disease profile. We highlight the need for continued innovation in cardiac EP field to optimize treatment strategy and improve long term outcome.

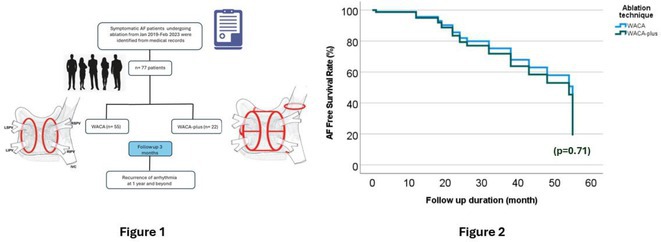

## ZERO FLUOROSCOPY "RESCUE" ABLATION FOR VENTRICULAR TACHYCARDIA INDUCED CARDIOMYOPATHY ORIGINATING FROM RIGHT VENTRICULAR OUTFLOW TRACT IN PREGNANCY

### **CHEA CHIN YUNG**, ABDUL RAQIB ABDUL GHANI, NOR HALWANI HABIZAL, HARTINI MOHD YUSOF, SATHVINDER SINGH GIAN SINGH, EMMA YAAKOP, SIOW YOON KEE, CHEE WEI SHEN, ONG YU YING

#### Sultan Idris Shah Serdang Hospital, Kajang, Malaysia

**Introduction:** During pregnancy, the increased occurrence of ventricular arrhythmias (VA) can be attributed to the unique physiological and hemodynamic changes. While most of these arrhythmias exhibit a benign course, refractory arrhythmias may precipitate cardiomyopathy. Radiofrequency ablation (RFCA) for VA in pregnancy is considered a final resort after exhausting medical therapy options

**Methods:** Ν/Α

**Results:** We present the case of a 40 y.o. gravida 3 at 26 weeks of gestation with palpitations and dyspnea for 3 months. Initial assessments revealed a structurally normal heart with preserved LVEF of 60%. She was hemodynamically stable but demonstreated sustained monomorphic ventricular tachycardia (VT) with left bundle branch block morphology on her 12‐lead ECG. Despite intravenous amiodarone bolus and oral propranolol, her condition deteriorated with hemodynamic instability necessitating intubation for respiratory support. Sustained runs of VT persisted with a reduction in LVEF to 40%. Following multidisciplinary discussion, a decision was made to proceed with a rescue RFCA for this drug refractory VA. The patient underwent an emergency rescue RFCA procedure in the EP lab, utilizing a zero‐fluoroscopy approach. Catheter navigation was facilitated using a three‐dimensional electro‐anatomical mapping system (Ensite NavX, Abbott, IL, USA). Sustained runs of VT were mapped and localized to the antero‐septal RVOT, with the earliest activation occurring 30 ms from the QRS complex (Figure 1). Radiofrequency energy was delivered to this target area, resulting in termination and suppression of VT. The patient was extubated and discharged one week post procedure in stable condition. One month following the procedure, her left ventricular function normalises and subsequently delivered her baby uneventfully at 36 weeks of gestation.

**Conclusions:** We shared our experience in the mangement of an idiopathic, sustained, drug‐refractory RVOT VT induced cardiomyopathy with a zero‐fluroscopic RFCA approach in second trimester of pregnancy, resulting in good outcome for the mother and the fetus.

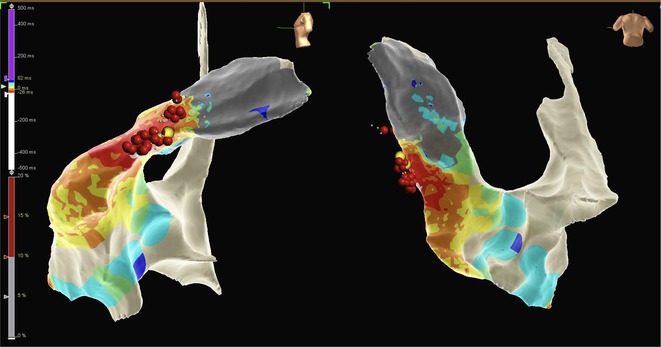

## AICD INSERTION IN AN AMPUTATED LEFT ARM PATIENT WITH AORTOSTERNAL VENOUS COMPRESSION: A CASE REPORT

### **KUMPOL CHINTANAVILAS**, APISAPOL INTHARAKANCHIT

#### Chulabhorn's international collage of medicine, Pathum Thani, Thailand

**Introduction:** ‐

**Methods:** ‐

**Results:** Venous assessment is one of the most important steps for automatic implantable cardioverter‐defibrillator (AICD) implantation. We hereby report a case of a challenging left axillary venous assessment. A case of 68‐year‐old Thai male with a history of traumatic left arm amputation 10 years ago. The patient was admitted due to heart failure precipitated by atrial fibrillation with rapid ventricular response. He required intravenous amiodarone and synchronized cardioversion. After restoration to sinus rhythm, the patient suffered from recurrent polymorphic ventricular tachycardia caused by amiodarone‐induced QT prolongation (corrected QT interval 619 ms). Dual chamber AICD implantation was planned. During the procedure, right femoral vein was retrogradely catheterized but the catheter and the wire were unable to be passed into the left brachiocephalic vein. In addition, the left arm was amputated leading to an inaccessible peripheral vein for the left axillary venogram. Fortunately, despite the limitations of this patient, the left axillary vein could be visualized via left external jugular venogram. Moreover, it was found that the patient also had an ostial left brachiocephalic vein stenosis compatible with aortosternal venous compression. After a left lateral axillary venous assessment, dual chamber AICD was successfully implanted through left brachiocephalic vein.

**Conclusions:** Aortosternal Venous Compression syndrome can be a major obstacle which could ultimately lead to a failure of retrograde approach to left brachiocephalic vein. Left external jugular venogram can used for identifying the axillary vein anatomy and ensure patency of venous drainage after an arm amputation.

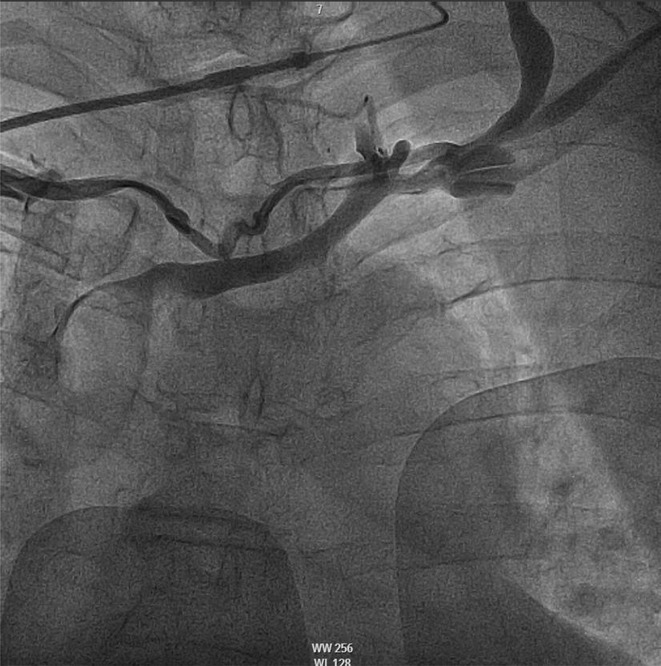

## A TWO‐STAGE DATA ANALYSIS FRAMEWORK FOR RISK ASSESSMENT OF BLEEDING IN ATRIAL FIBRILLATION PATIENTS TREATED WITH RIVAROXABAN

### **WEI‐RU CHIOU** ^1,2^, CHEN‐YANG CHENG^3^, PI‐YI CHANG^4^, PO‐LIN LIN^5^, YING‐HSIANG LEE^6^, YI‐NING HUANG^3^

#### ^1^Taitung MacKey Memorial Hospital, Taitung, Taiwan,^2^Department of Medicine, MacKay Medical College, New Taipei, Taiwan,^3^Department of Industrial Engineering and Management, National Taipei University of Technology, Taipei, Taiwan,^4^Department of Radiology, Taichung Veterans General Hospital, Taichung, Taiwan,^5^Hsinchu MacKay Memorial Hospital, Hsinchu, Taiwan,^6^Cardiovascular Center, MacKay Memorial Hospital, Taipei, Taiwan

**Introduction:** Atrial fibrillation increases stroke risk, and anticoagulants like rivaroxaban can prevent strokes but may cause bleeding. Current risk assessment tools are limited and do not account for patient differences. This study developed a new two‐stage data analysis framework to better evaluate bleeding risk in atrial fibrillation patients treated with rivaroxaban.

**Methods:** Data from 2,362 atrial fibrillation patients treated between 2011 and 2016 were analyzed retrospectively. Patients were clustered into five groups using the K‐modes algorithm based on stroke history, gender, paroxysmal or non‐paroxysmal atrial fibrillation. Association rule mining was conducted within each cluster to find rules linking patient characteristics to bleeding occurrences.

**Results:** In stroke patients, age ≥ 75, CHF history, and bleeding history are linked to higher bleeding risk. Patients with paroxysmal atrial fibrillation taking 20 mg of rivaroxaban face a higher bleeding risk, particularly men using NSAIDs and women with diabetes, eGFR < 50, and a history of bleeding. Moreover, women with these conditions are at high bleeding risk regardless of rivaroxaban dosage. Non‐paroxysmal atrial fibrillation patients showed increased bleeding risk as well: men with hypertension and alcohol use, and women with diabetes, hypertension, and bleeding history.

**Conclusions:** The two‐stage analysis framework provided interpretable risk assessment rules that reflect patient heterogeneity, identifying subgroups at higher bleeding risk. This helps clinicians balance stroke prevention with adverse event risks, enabling more personalized risk assessments compared to traditional scoring systems. However, the study's limitations include the single‐center data source; future work should validate these findings across different databases and explore advanced machine learning models for risk prediction.

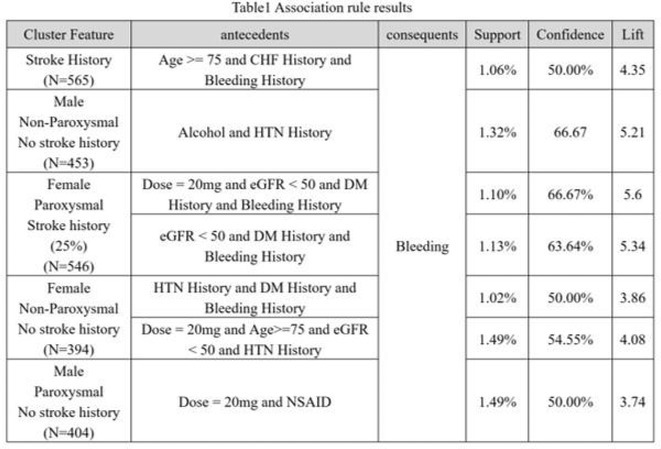

## CHICKEN OR EGG

### **SAM CHIRAMEL**, RAJESH NAIR

#### Government Medical College, Kozhikode, India

**Introduction:** Genetic mutations leading to arrhythmia are common. Here we are presenting a case with dual mutation and leading to a confusion as which one is responsible for the arrhythmia.

**Methods:** N/A

**Results:** A 64yr old female, presenting with dyspnoea from 1 week followed by syncope in a local hospital. Was given 5 shocks and referred to out hospital. In our hospital, patient again developed syncope and the monitor showed PMVT. Again needed to be defibrillated, thrice, to maintain sinus rhythm. Her echocardiogram showed DCM with severe LV dysfunction with an EF of 28%, global hypokinesia. She was started on a propranolol 160mg. Over the next three days the patient did not develop any VT but her QTC was consistently above 500ms. On the third day her heart rate dropped to less than 40bpm requiring temporary pacing. An angiogram was done which showed normal coronaries. We weren’t sure whether the DCM caused the PMVT or was there an underlying LQTS which predisposed her to the DCM. A genetic analysis was sent and the patient was managed inserting a Dx ICD with left bundle area pacing, as the patient could not afford a CRT‐D. Genetic analysis showed presence of two mutations, RBM20 gene and KCNQ1 gene. RBM20 gene has been shown to cause DCM with ventricular arrhythmia and loss KCNQ1 gene to cause LQTS 1. The patient had two uncommon mutations, leading to PMVT. There has been no case reported prior with these two mutations in the same patient.

**Conclusions:** the search for the cause of a problem can lead to interesting findings.

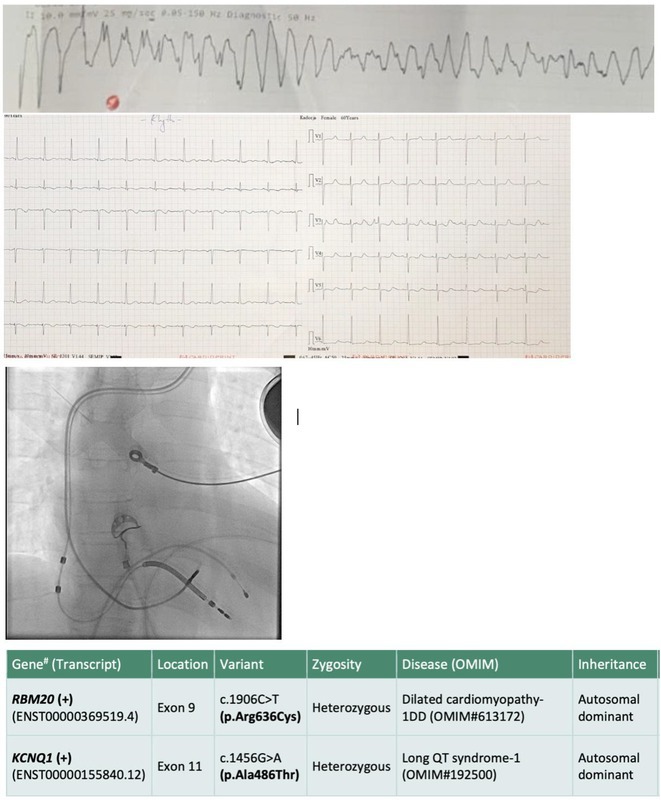

## JUMPING JACK

### **SAM CHIRAMEL**, RAJESH NAIR

#### Government Medical College, Kozhikode, India

**Introduction:** It is uncommon to see changing cycle tachycardia cycle lengths during tachycardia in AVRT, unless the patient develops a bundle branch block or has multiple pathways. presenting a teaching case of alternating tachycardia cycle length during an AVRT.

**Methods:** N/A

**Results:** 58 Years Male, with paroxysmal palpitations for the past 2 years, easily terminated with Inj. Adenosine. No other co‐morbidity. Referred to our hospital for further work up and ablation. As the patient was very symptomatic, he was taken up for an electrophysiology study even though there was no documented ECG for an SVT. The baseline ECG did not show pre‐excitation ad baseline cycle length was 840ms with AH of 79ms and HV of 42ms. The retrograde protocol showed eccentric non‐decremental pathway at CS 1,2 suggestive of concealed left lateral pathway. The tachycardia was easily inducible with atrial extrastimulus. But the tachycardia showed an alternating cycle length of 310ms and 400ms. Our differential included 1) multiple concealed pathway with signals changing the pathway during tachycardia; 2) As there was a hint of LAHB in the alternating beat, Coumel's Law showing slowing of tachycardia; 3) preferential conduction through fast and slow pathway during tachycardia. To confirm this we measured the VA interval. It was always constant ruling out the first two differential. On measuring the AH interval, it was found that the difference in AH was exactly equal to the difference in the tachycardia rate, I.e AH 100 and 200ms during alternating beats. This proved the signals alternating from fast to slow pathway during tachycardia. The left lateral pathway was ablated. Post RFA no tachycardia, neither AVRT nor AVNRT, was inducible. So slow pathway ablation was not done.

**Conclusions:** The signals will give you the answer. All we need to do is to look for it.

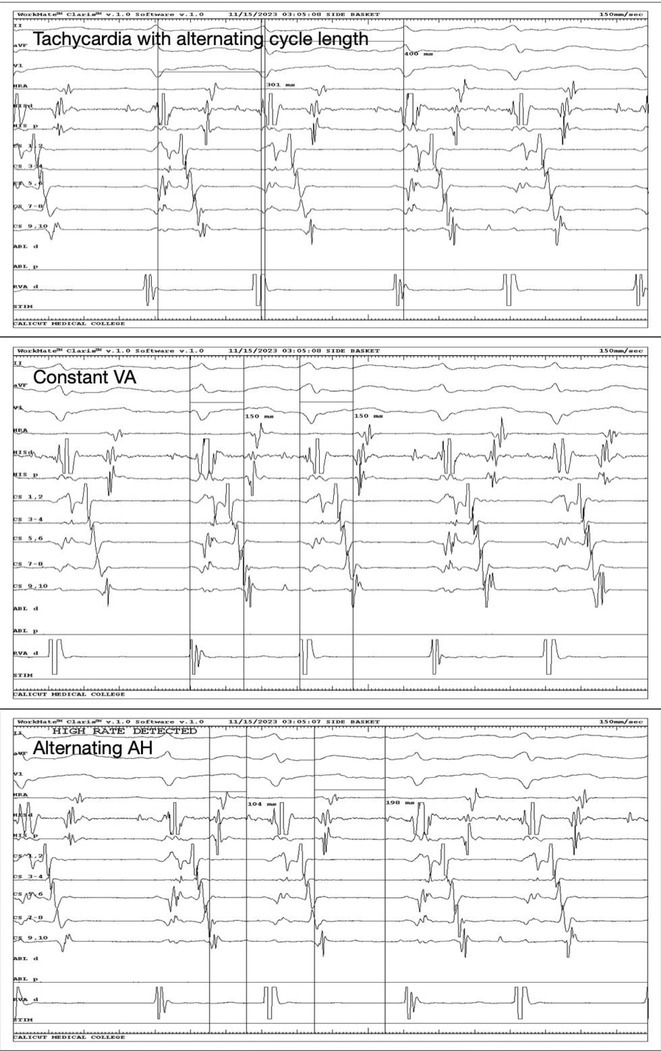

## PANDORA'S BOX

### **SAM CHIRAMEL**, RAJESH NAIR

#### Government Medical College, Kozhikode, India

**Introduction:** 31yr old Female, presenting with recurrent palpitations over the last 5‐6 years. Had been previously diagnosed as PSVT, probably AVNRT, and on Verapamil 120mg/day. On presenting to us, episodes of palpitations on medications but no tachycardia ECG was available. Her baseline ECG did not show any evidence of WPW.

**Methods:** NA

**Results:** On retrograde protocol, there was eccentric conduction followed by induction of tachycardia at TCL of 260ms. Earliest activation was at CS9,10. VOP done to conform AVRT as the diagnosis. The posterior septal region was mapped but we were unable to get fused signals. Finally fused signals were obtained at 3‐4O’clock tricuspid annulus. Confirming that there was no His signal on ablation catheter, RFA done which terminated this tachycardia. But then the patient went into a persistent tachycardia with TCL of 330ms. Earliest A wave seen in CS 9, 10. This tachycardia was mapped and fused signal were obtained at TA 5‐6 o clock position. On terminating this and trying to induce, the patient developed her third tachycardia. Which we diagnosed as AVNRT and the slow pathway was modified. On trying to induce the patient developed another tachycardia. This time it was atrial tachycardia at a TCL of 470ms. The tachycardia was non sustained but easily inducible

It was mapped to the inferior posterolateral right atrium. The patient didn’t have any symptoms due to this and was getting uncomfortable on table. We decided to wait and watch and did not ablate this tachycardia. On follow up, in the first week, the patient had short runs of AT on the ECG but no symptoms at all. But due to the salvos of AT we started her on propranolol. On her follow up after one month. As her symptoms persisted, we decided to take it under 3D mapping. The AT was localised to the 6‐7 o clock TA which was ablated. On reinducung she developed another AT around crest terminals which was also ablated. Then she developed a slow AT which was localised to the para‐hisian area. Decided not to ablate that and keep her on medical management unless the AT causes severe symptoms or tachycardiomyopathy.

**Conclusions:** Multiple tachycardia is a challenge during ablation. Unusual presentations with more than 3 tachycardia in a single patient is rare.

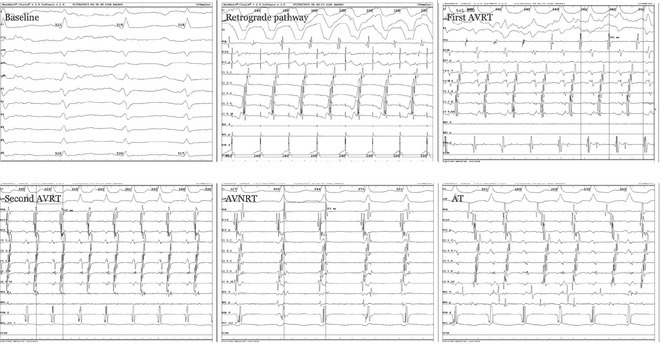

## EFFECT OF HYDROQUINIDINE ON ST‐ELEVATION ON HOLTER MONITORING IN PATIENTS WITH BRUGADA SYNDROME

### **KATHERINE CHISWELL** ^1,2^, JULIA ISBISTER^1,2^, ADRIENNE KIRBY^3^, YUN DUAN^1^, JAMIE CHAM^1,2^, LAURA YEATES^1,2,4,5^, HARIHARAN RAJU^6^, CHRISTOPHER SEMSARIAN^1,2,4^, BELINDA GRAY^1,2^, RAYMOND SY^1,2^

#### ^1^Department of Cardiology, Royal Prince Alfred Hospital, Sydney, Australia,^2^Faculty of Medicine and Health, The University of Sydney, Sydney, Australia,^3^NHMRC Clinical Trials Centre, Camperdown, Sydney, Australia,^4^Agnes Ginges Centre for Molecular Cardiology at Centenary Institute, The University of Sydney, Sydney, Australia,^5^Genomics and Inherited Disease Program, Garvan Institute of Medical Research and University of New South Wales, Sydney, Australia,^6^Faculty of Medicine, Health and Human Sciences, Macquarie University, Sydney, Australia

**Introduction:** Hydroquinidine (HQ) reduces arrhythmic events in patients with Brugada syndrome (BrS) but the mechanism remains unclear. Praecordial ST‐elevation (STE) is characteristic of BrS and STE burden may be associated with events. Based on *in vitro* studies, HQ is expected to normalise the ST segment. The project aim is to determine *in vivo* effect of HQ on STE in BrS.

**Methods:** Patients with BrS underwent 12‐lead, 24‐hour Holter monitoring at baseline and on‐treatment with HQ 300mg BD.

**Results:** Ten patients (9 males, 47.0 ± 15 years) with spontaneous type 1 BrS pattern (mean Shanghai score 4.4) were studied. There was a significant increase in temporal burden (time with ≥ 2mm STE over monitoring period) with HQ (752 ± 160 minutes at baseline vs 985 ± 134 minutes on HQ; p=0.024). While there was no difference in spatial burden of ST‐elevation across the precordial leads on‐treatment compared to baseline (ratio 1.26 [range: 0.91 ‐ 1.75], p=0.17) in whole cohort analysis, patients with therapeutic HQ levels had a greater increase in both temporal and spatial burden compared to those with subtherapeutic levels. One patient developed ventricular tachycardia originating from the right ventricular outflow tract on HQ, necessitating discontinuation. There was no change in heart rate variability on HQ.

**Conclusions:** HQ paradoxically increases the temporal burden of praecordial STE in patients with BrS. HQ may be associated with pro‐arrhythmia and 24‐hour Holter monitoring should be considered after initiating therapy in patients with BrS for monitoring safety.

## VEIN OF MARSHALL ABLATION USING TRANCATHETER INTRAVENOUS RADIOFREQUENCY ABLATION IN PATIENT WITH PERSISTENT ATRIAL FIBRILLATION: CASE REPORT

### **JIN HEE CHOI** ^1^, KI WON HWANG^1^, HYUNG OH CHOI^2^, MIN SOO CHO^3^, JUNE HONG KIM^1^

#### ^1^Pusan National University Yangsan Hospital, Yangsan, Korea, Republic of,^2^Soonchunhyang University Hospital, Soonchunhyang University College of Medicine, Bucheon, Korea, Republic of,^3^University of Ulsan College of Medicine, Asan Medical Center, Seoul, Korea, Republic of

**Introduction:** For vein of Marshall (VoM) ablation, a conventional radiofrequency ablation catheter is relatively thick and difficult to insert into the VoM, resulting in a low success rate. Alcohol ablation in the VoM makes it difficult to control the extent of the procedure and damage to other adjacent organs. The authors developed and tested a new transcatheter intravenous radiofrequency ablation (TIRA) catheter (Tau‐PNU Medical, Busan, Republic of Korea) to perform VoM ablation (Figure A).

**Methods:** N/A

**Results:** A 53‐year‐old man with a 2‐year history of persistent atrial fibrillation (AF) and tachycardia‐mediated cardiomyopathy underwent a previous pulmonary vein isolation (PVI) procedure. Despite extensive consolidation along with the PVI lesion set, AF persisted. For VoM ablation, the CS was cannulated via the right femoral vein with a 6 French (F) deflectable ablation catheter and the 8.5 F sheath was advanced into the CS body. After coronary venography and identification of the VoM, a 0.014‐inch guidewire (Asahi Sion, Asahi Intec) with a 6 F internal mammary guiding catheter (Vista Brite tip IM SH, Cordis) was advanced into the VoM (Figure B). A 2 F electrode catheter (EPstar Fix, Japan Lifeline) was then inserted to check the atrial potential in the VoM before ablation. A 4 F over‐the‐wire type TIRA catheter was advanced into the VoM and ablated twice at 50°C for 30 seconds. A 2 F electrode catheter was then used to check the atrial potential in the VoM after ablation. The voltage of the atrial potential in the VoM after ablation was significantly lower than the atrial potential before ablation (Figure C). There were no significant complications such as arrhythmias or cardiac tamponade.

**Conclusions:** VoM ablation with the TIRA catheter is a safe and attractive new approach for patients with persistent AF.

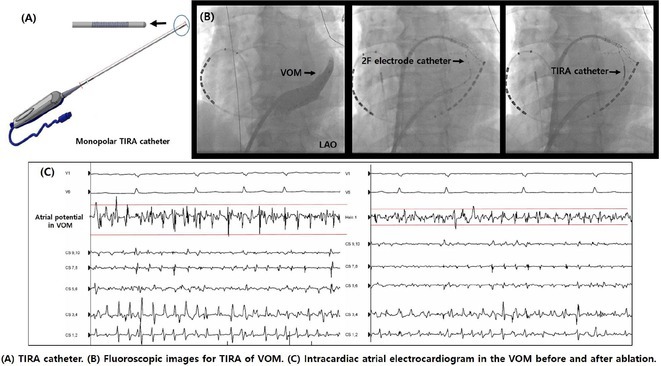

## AGE AND GENDER SPECIFIC PATTERNS IN ECG MEASUREMENTS AND DIAGNOSTIC FREQUENCIES IN A LARGE HEALTH SCREENING POPULATION

### **JINA CHOI** ^1^, HO‐YEON BANG^2^, DONG‐WON SHIN^2^, JUN‐HYUNG KIM^2^, SUNHWA KIM^1^, JI HYUN LEE^1^, YOUNGJIN CHO^1^, IL‐YOUNG OH^1^

#### ^1^Seoul National University Bundang Hospital, Seongnam‐si, Korea, Republic of,^2^Seoul National University, Seoul, Korea, Republic of

**Introduction:** Variations in electrocardiographic (ECG) parameters are associated with cardiovascular prognosis in patients with cardiovascular diseases. This study aims to investigate the differences in ECG values and the frequency of ECG‐associated diagnoses according to age and gender among large‐scale health screening participants.

**Methods:** Data from 140,559 participants at Bundang Seoul National University Hospital, comprising 296,927 ECG records, were analyzed. Four intervals, RR, PR, QRS duration, QTc, and three axes including P, QRS, T were assessed. Two‐way ANOVA was used to explore the effects of age and gender on ECG values. The frequency of 16 commonly diagnosed ECG findings was presented as counts and percentages of diagnosed ECGs, with common diagnoses further analyzed by age and gender. Additionally, the frequencies of atrial fibrillation (AF) and left ventricular hypertrophy (LVH) were examined over five‐year intervals.

**Results:** Significant differences in the four intervals and three axes were observed according to age and gender. The PR and QTc intervals increased with age regardless of gender, while the QRS axis decreased in both genders with advancing age. Comparing genders within the same age group, PR interval and QRS duration were higher in males, but QTc interval was higher in females. The RR interval was higher in males except in their 50s. The most frequent ECG diagnosis was LVH, occurring in 6.58% of cases. Most diagnoses increased with age, except for RAD, RVH, and LPFB. Over five‐year intervals, the frequency of LVH decreased from 19.01% (2005‐2009) to 1.79% (2020‐2023), while AF slightly increased from 0.34% to 0.43% over the same periods.

**Conclusions:** Significant differences were found in ECG values based on age and gender, as well as in the frequency of ECG diagnoses. These findings underscore the importance of considering age and gender when using ECG as a diagnostic tool or screening measure.

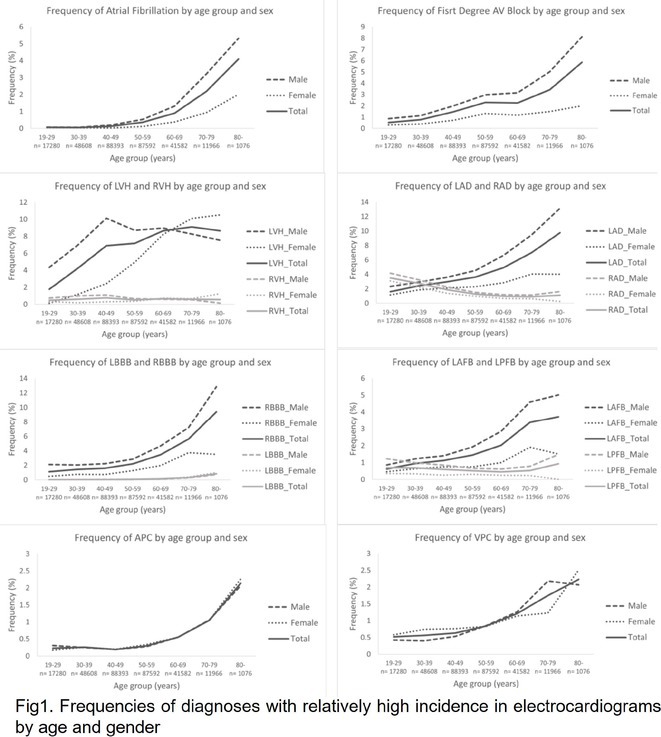

## CATHETER ABLATION FOR INTRAATRIAL MACROREENTRANT TACHYCARDIA OF WHICH CRITICAL ISTHMUS WAS RIGHT ATRIUM REMNANT IN A PATIENT WITH EXTRACARDIAC CONDUIT FONTAN CIRCULATION

### **JIYEON CHOI**, JAE‐SUN UHM, WON‐WOO YOO, GON LEE, DUK WOO PARK, HAN JIN PARK, DAEHOON KIM, HEE TAE YU, TAE HOON KIM, BOYOUNG JOUNG, HUI‐NAM PAK, MOON‐HYOUNG LEE

#### Severance Hospital, Seoul, Korea, Republic of

**Introduction:**

**Methods:** N/A

**Results:** A 44‐year‐old female complained of palpitation and dyspnea of sudden onset. She was diagnosed with unbalanced atrioventricular septal defect and functional single ventricle at birth She underwent atriopulmonary connection (APC) Fontan operation at the age of 4 years. For Fontan circulation failure, she underwent conversion surgery from APC Fontan circulation to extracardiac conduit Fontan circulation using Goretex at the age of 26 years. During Fontan conversion surgery, the most enlarged right atrium (RA) was removed but a small portion of RA remained because the sinus node needed to be preserved. Blood pressure and heart rate were 82/64 mmHg and 149 /min. ECG at palpitation showed atrial flutter with 2:1 atrioventricular conduction (Figure 1). Electrophysiological study was performed. Fontan conduit was punctured and the mapping catheter was inserted into the atria. For compete mapping of the whole atria, another Fontan puncture from the conduit to RA remnant was needed. 3‐dimensional activation map showed intraatrial macroreentrant tachycardia. The reentry circuit was low RA remnant, high RA remnant, roof of left atrium (LA), low LA, and low RA remnant (Figure 2). The critical isthmus (slow conduction zone) was mid RA remnant. Catheter ablation for the critical isthmus was performed at mid RA remnant (Figure 2). After catheter ablation, the tachycardia was not induced at programmed electrical stimuli. Tachycardia did not recur for 4 months.

**Conclusions:**

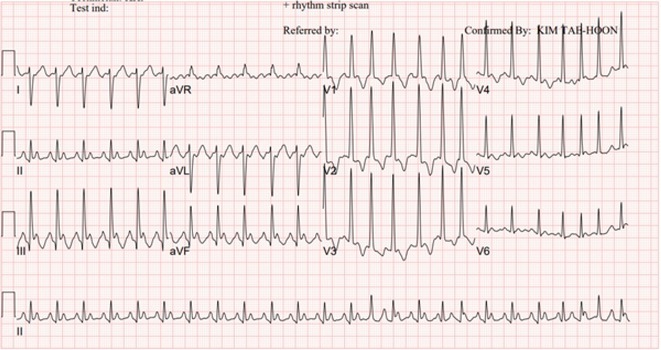

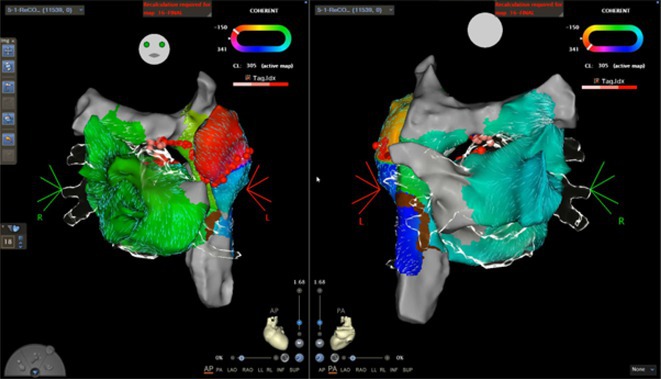

## IMPACT OF RENAL FUNCTION ON CLINICAL OUTCOMES OF EDOXABAN THERAPY IN ATRIAL FIBRILLATION PATIENTS: THE ETNA‐AF ASIAN STUDY

### JONG‐IL CHOI

#### On behalf of the Global ETNA Study Group, Korea University Medical Center, Seoul, Korea, Republic of

**Introduction:** This study aimed to evaluate the effectiveness and safety outcomes over 2 years of Edoxaban Treatment in routiNe clinical prActice (ETNA) for Atrial Fibrillation (AF) patients, categorized by renal function from a GLOBAL cohort and four Asian cohorts (Korea, Taiwan, Hong Kong, and Thailand).

**Methods:** In this post‐hoc analysis, the effectiveness and safety of edoxaban in patients with AF were analyzed by baseline CG‐CrCl (Cockcroft‐Gault creatinine clearance) categories in the global ETNA‐AF(n=26,805) and four Asian countries(n=3,299) over a 2‐year follow‐up period. Clinical outcomes assessed included all‐cause mortality, systemic embolic event (SEE), any stroke (ischemic and hemorrhagic), major bleeding, and clinically relevant non‐major bleeding (CRNMB).

**Results:** A total of 26,805 patients who received either 60 mg or 30 mg of edoxaban daily in the Global ETNA study completed the two‐year follow‐up. The 4 Asian countries’ cohorts with AF patients having baseline CG‐CrCl 15~30 ml/min constituted 6.5% compared to 3.8% in the Global cohort. In the 4 Asian countries’ cohorts with AF patients in the CG‐CrCl 15~30 ml/min category, which showed severe renal function at baseline, clinical outcomes were 14.1% for all‐cause death; 0% for SEE; 4.2% for any stroke; 5.2% for major bleeding, and 4.7% for CRNMB. In AF patients with CG‐CrCl ≥50 ml/min had annual rates of 1.5% for all‐cause death; 0% for SEE; 1.8% for any stroke; 1.4% for major bleeding, and 1.3% for CRNMB. In the Global cohort, AF patients in the CG‐CrCl 15~30 ml/min category had annual rates of 19.9% for all‐cause mortality, 0.1% for systemic embolic event (SEE), 2.3% for any stroke, 5.8% for major bleeding, and 4.5% for CRNMB. In contrast, AF patients with CG‐CrCl ≥50 ml/min had annual rates of 4.1% for all‐cause mortality, <0.1% for SEE, 1.3% for any stroke, 1.6% for major bleeding, and 2.3% for CRNMB.

**Conclusions:** In four Asian AF patients categorized by baseline renal function, at a two‐years, this study showed that edoxaban was effectiveness and safe even in patients with severe reductions in renal function.

## SAFETY OF CLASS IC ANTIARRHYTHMIC DRUGS IN ATRIAL FIBRILLATION WITH STRUCTURAL HEART DISEASE

### **JONG‐IL CHOI** ^1^, JOO HEE JEONG^1^, YUN GI KIM^1^, HYOUNG SEOK LEE^1^, YUN YOUNG CHOI^1^, JAEMIN SHIM^1^, YOUNG‐HOON KIM^1^, HOSEOB KIM^2^, YOONJONG BAE^2^

#### ^1^Korea University Medical Center, Seoul, Korea, Republic of,^2^Hanmi Pharm. Co., Ltd, Seoul, Korea, Republic of

**Introduction:** This study investigated the safety and feasibility of class IC antiarrhythmic drugs (AADs) compared with those of class III AADs in patients with AF and structural heart disease.

**Methods:** Based on the nationwide health insurance database, patients first diagnosed as AF between 2013 and 2019 were screened, and those with a diagnosis of either hypertrophic cardiomyopathy, obstructive coronary artery disease, or heart failure were included. The primary outcome was the composite of all‐cause mortality, sudden cardiac arrest, and ventricular arrhythmia. Secondary outcomes included new‐onset atrial flutter, heart failure hospitalization, and coronary revascularization.

**Results:** A total of 38,378 patients were analyzed. Class IC and III AADs were prescribed to 10,034 and 28,344 patients, respectively. Patients in the class III AAD group were older and had higher CHA_2_DS_2_‐VASc scores than those in the class IC AAD group. The class III AAD group had a higher risk of primary outcome than the class IC AAD group (hazard ratio 2.36, 95% confidence interval 1.93‐2.89, p<0.001). The risks of heart failure hospitalization and coronary revascularization were also higher in the class III AAD group than those in the class IC group. However, the risk of atrial flutter did not differ significantly.

**Conclusions:** In patients with AF and structural heart disease, the use of class IC AAD was associated with lower risk of death and adverse cardiovascular events than Class III AAD. Class IC AADs may be a reasonable choice in patients with AF and stabilized structural heart disease.

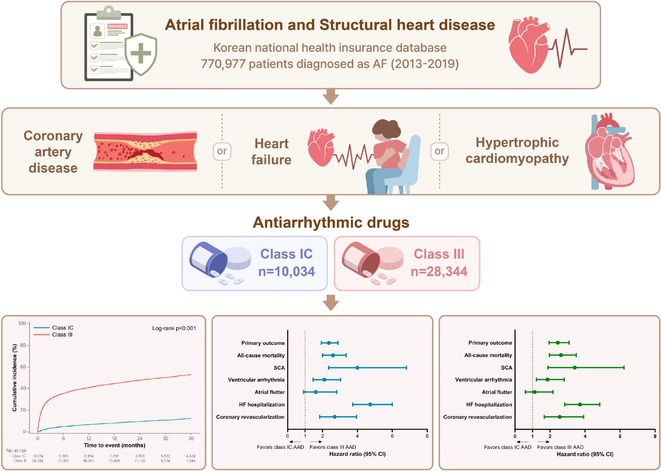

## CAUSAL EFFECT OF LONE ATRIAL FIBRILLATION ON CARDIOVASCULAR DISEASES: A MENDELIAN RANDOMIZATION STUDY

### **YOUNG CHOI**, SUNG‐HWAN KIM, YONG‐SEOG OH, DO YOUNG KIM

#### Seoul St. Mary's Hospital, Seoul, Korea, Republic of

**Introduction:** It is well established that atrial fibrillation (AF) is associated with an increased risk of ischemic stroke and heart failure (HF). However, AF commonly coexists with various cardiovascular comorbidities, making it difficult to isolate the direct effect of AF on clinical outcomes. We investigated the causal effect of lone AF on various cardiovascular phenotypes using mendelian randomization (MR).

**Methods:** A genome‐wide association study (GWAS) for lone AF were conducted using UK biobank data. Lone AF was defined as AF diagnosed without coexisting conditions including obesity, heavy alcohol use, hypertension, diabetes mellitus, coronary artery disease (CAD), valvular heart disease, cardiomyopathy, hyperthyroidism, or obstructive sleep apnea. Summary‐level data for cardiovascular phenotypes were obtained from public GWAS datasets. The causal effects were estimated through generalized summary‐data‐based mendelian randomization (GSMR).

**Results:** Lone AF significantly increased the risk for stroke (OR 2.19, 95% CI 1.87 ‐ 2.55) and HF (OR 2.00, 95% CI 1.78 ‐ 2.24), but did not show significant association with CAD (OR 0.98, 95% CI 0.84 ‐ 1.14). or cardiac death (OR 1.21, 95% CI 0.98 ‐ 1.50). Sensitivity analyses confirmed the significant association with stroke and HF, while the association with CAD (MR‐Egger: OR 0.92, 95% CI 0.68 ‐ 1.23, weighted median 0.98, 95% CI 0.76 ‐ 1.27) or cardiac death was not significant (MR‐Egger: OR 1.35, 95% CI 0.90 ‐ 2.04, weighted median: OR 1.21, 95% CI 0.86 ‐ 1.71).

**Conclusions:** This MR study showed the causal effect of lone AF on the increased risk of stroke and HF. However, a causal relationship between lone AF and CAD or cardiovascular death was not demonstrated.

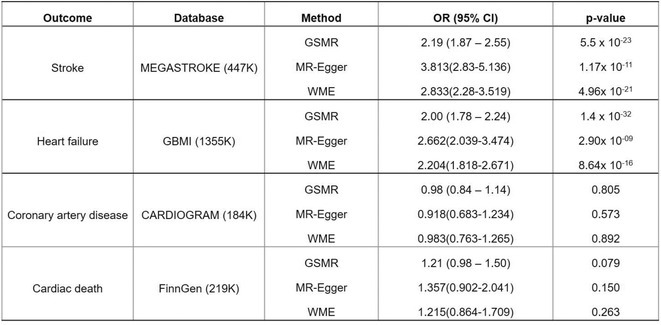

## USE OF M MODE (WITH APPLICATION OF COLOUR DOPPLER) TECHNIQUE IN THE DIAGNOSIS OF ATRIAL FLUTTER

### **JONATHAN CHOO**, MING XU, CHARMAINE CHAN

#### KK Women's and Children's Hospital, Singapore, Singapore

**Introduction:** The diagnosis of atrial flutter is typically clear on surface electrocardiograms. However, the diagnosis may be missed if there is concomitant 2:1 atrioventricular block such that the ventricular rate is between 120 ‐ 140 bpm or in clinical situations when surface ECGs may not be so readily available.

**Methods:** We describe 3 cases of atrial flutter to demonstrate the utility of M Mode Technique (with and without colour application) in the confirmation of atrial flutter. At our centre, atrial landmarks are used to enhance the definition of atrial events on M Mode trace.

**Results:**

A 13‐ year‐old boy with idiopathic pulmonary arterial hypertension was admitted for acute onset palpitations. His heart rate was 120 BPM. M Mode across the left atrium and right ventricle (from the coumadin ridge to the right ventricular free wall) demonstrated the relationship between atrial and ventricular events confirming atrial flutter with 2:1 atrioventricular block.

A 6‐year‐old girl presented with post‐operative total anomalous pulmonary venous connections presented well at her routine cardiology follow‐up visit with a heart rate of 170 beats per minute. A prominent fold was use in the posterior wall of the left atrium to obtain M Mode deflections indicating atrial events.

A 32‐year‐old with a history of post‐operative aortic coarctation and post‐operative mitral valve replacement had been complaining of palpitations for last week. The right atrial pectinate muscles were used to obtain atrial M Mode deflections. Colour‐Encoded enhancement of M Mode demonstrated atrial flutter with atrial fibrillation.

**Conclusions:** The left atrial coumadin ridge and right atrial pectinate muscles may be used to enhance the detection of atrial mechanical events on M Mode to determine atrial ‐ ventricular relationships particularly in the confirmation of atrial flutter. The use of the above atrial structures enhances the demonstration of atrial contractions indicating atrial events in the M Mode assessment of rhythm. In addition, we have used the Colour‐Encoded M Mode to highlight atrial events by correlating ventricular inflows to ventricular free wall contractions.

## RENAL DENERVATION AS A STRATEGY TO IMPROVE HEART FAILURE OUTCOME INVOLVES CONTROLLING SYMPATHETIC ACTIVITY AND MANAGING INFLAMMATION IN AN EXPERIMENTAL AUTOIMMUNE MYOCARDITIS RABBIT MODEL

### **YU‐HUI CHOU** ^1^, YA‐WEN HSIAO^1^, WEI‐LUN LIN^2^, SHIN‐HUEI LIU^1,3^, SHIH‐ANN CHEN^2,3,4^, LI‐WEI LO^1,3^

#### ^1^Taipei Veterans General Hospital, Taipei, Taiwan,^2^Taichung Veterans General Hospital, Taichung, Taiwan,^3^National Yang Ming Chiao Tung University, Taipei, Taiwan,^4^National Chung Hsing University, Taichung, Taiwan

**Introduction:** Previous studies have suggested a correlation between heart failure (HF) occurrence and myocardial inflammation. Our objective is to explore the therapeutic effects of renal denervation (RDN) in experimental autoimmune myocarditis (EAM) and to clarify the involvement of various inflammatory factors in the therapeutic mechanism.

**Methods:** Eighteen rabbits were randomly assigned to sham control, EAM, and EAM‐RDN groups. The EAM model was validated by assessing the expression of CD4, CD8, CD14, and CD163 using flow cytometry. Renal catecholamine levels were measured in all three groups, and cardiac function was assessed using echocardiography. Additionally, TNF‐α, IL‐6, and IL‐1β expression levels were determined using ELISA.

**Results:** In EAM‐induced HF rabbits, the expressions of CD4, CD8, CD14, and CD163 significantly increased, indicating successful model establishment. Following RDN treatment, a notable reduction in these inflammatory markers was observed (Fig. A). The levels of cytokines TNF‐α, IL‐6, and IL‐1β were significantly induced in the EAM groups and reduced after RDN treatment compared to the EAM group (Fig. B). Furthermore, adrenaline and norepinephrine levels, elevated during EAM, were significantly decreased post‐RDN therapy (Fig. C). While fractional shortening (FS) and left ventricular ejection fraction (LVEF) exhibited an increase in EAM, RDN treatment led to a subsequent decrease (Fig. D), indicating an amelioration of cardiac function.

**Conclusions:** Based on these results, it is suggested that RDN effectively attenuated inflammation and improved cardiac function in EAM‐induced HF rabbits. The modulation of inflammatory markers and catecholamine levels, along with the reversal of EAM‐induced cardiac dysfunction, highlights the potential therapeutic role of RDN in HF management. Overall, these findings suggest that RDN may hold promise as a therapeutic intervention for HF by modulating sympathetic activity, reducing inflammation, and ameliorating cardiac function in EAM‐induced HF rabbits.

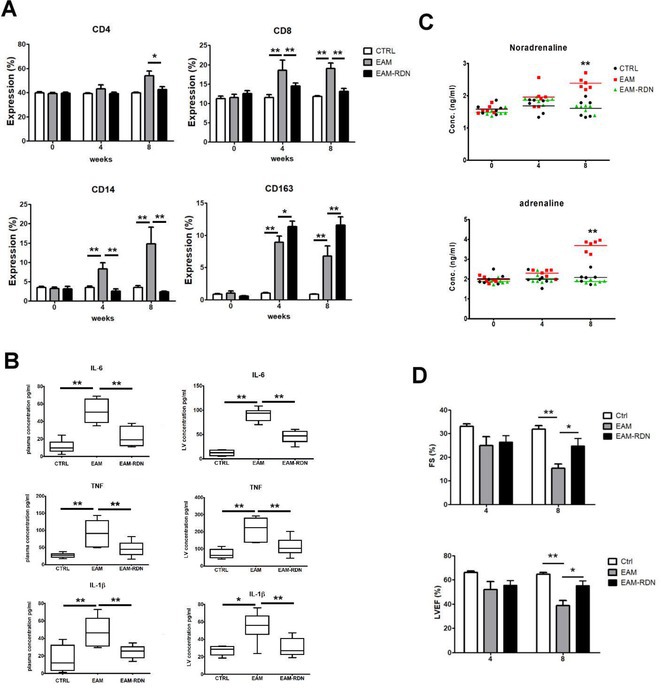

## AUTOCAPTURE ALGORITHM VALIDATION IN PERMANENT LEFT BUNDLE AREA PACING WITH STYLET DRIVEN LEAD ‐ A PILOT STUDY

### **SAROJ KUMAR CHOUDHURY**, DEBABRATA BERA

#### NH‐RTIICS, Kolkata, India

**Introduction:** AutoCapture^TM^ (AC) [Abbott] is a useful algorithm for safety in case of late threshold rise and saving battery. Its feasibility is not validated in permanent left bundle area pacing.

**Methods:** It was a prospective observational study. We attempted autocapture algorithm programming in consecutive (n=25) patients with successful PLBAP with an Abbott device with SDL. We also included patients who had anodal capture while bipolar pacing or had non selective LB capture at higher output and selective LB capture at lower output. Cases with LB capture at higher output and septal myocardial capture at lower output during initial implant were excluded to minimize heterogeneity (n=4).

**Results:** Among 21 cases ( age 64±10 yrs, 13 males), all had AC ‘recommended’ when tested immediately after the case on the same day. The mean manual threshold for LB capture was 1 V± 0.5 @ 0.4 ms PW. The AC mean threshold was 0.75+‐ 0.5 mV (p> 0.05). Over mean follow up for 2‐5 months the AC threshold remained similar. Among them 2/21 cases had loss of LB capture on follow up suggestive of micro‐dislodgement. One case the LB capture was evident on higher output 3V@ 0.4 ms. The device continued to capture at a lower myocardial capture threshold determined by the device correctly. Among 8 cases who had anodal capture and predominant myocardial capture at higher output and AC was successful to avoid anodal capture and myocardial capture.

**Conclusions:** AutoCapture algorithm is feasible among PLABP cases with Abbott SDL. It can reliably avoid anodal capture and save battery. However, monitoring at regular intervals is necessary as late loss of LB capture cannot be detected by AC algorithm where it keeps pacing at lower myocardial capture threshold.

## LIMITATION OF DIFFERENTIAL VENTRICULAR PACING IN ANTEROSEPTAL ACCESSORY PATHWAY ‐ A CASE REPORT

### **SAROJ KUMAR CHOUDHURY**, DEBABRATA BERA

#### NH‐RTIICS, Kolkata, India

**Introduction:** When retrograde VA conduction is via AV node, the VA interval is usually shorter during RV apical pacing than RV basal pacing due to early engagement of right bundle branch. When retrograde conduction is over the non‐decremental septal accessory pathway, the VA interval became shorter from RV base compared to RV apex. A discrepant response was noted in a case of 25‐year‐old female with supraventricular tachycardia.

**Methods:** N/A

**Results:** 25 year female presented with paroxysmal rapid palpitation since last 1year. In EP lab, short VA narrow complex tachycardia was induced. Attempt to entrain the tachycardia with ventricular overdrive pacing was repeatedly terminating the tachycardia. His synchronous PVC and parahisian pacing were indicative of presence of a septal AP. VA interval during apical pacing was 120ms while it was 140ms during basal pacing which was in contradiction to earlier maneuvers which were suggestive of retrograde atrial conduction through a septal AP. While mapping during the tachycardia, the earliest atrial activation with fused ventricular and atrial electrogram located the accessory pathway to superoparaseptal position over the tricuspid annulus. Repeating the maneuver with catheter at anterobasal RV instead of posteobasal RV, VA interval was reproducibly found to be shorter opposed to pacing from RV apex in consistent with presence of a septal accessory pathway. Successful accessory pathway ablation with radiofrequency application terminated the tachycardia.

**Conclusions:** In cases of SVT with concentric atrial activation, a concealed anteroseptal accessory pathway is another possibility. This type of accessory pathway is extremely rare (less than 1%). Differential RV apex‐base pacing from posterobasal RV in these cases will provide conflicting results as the accessory pathway insertion site is far away from the pacing site which is the scenario in our case. This finding is rarely reported in clinical practice. Our case highlights the fact and puts emphasis on pacing from various basal sites (antero / supero vs postero / infero‐basal RV region in cases where pacing maneuvers present conflicting results.

## T‐WAVE MORPHOLOGY CHANGES OBSERVED BY CONTINUOUS MONITORING USING INSERTABLE CARDIAC MONITORS DURING FOLLOW UP PERIOD AFTER ANTIARRHYTHMIC LOADING HOSPITALIZATION: RESULTS FROM THE LINQ QT STUDY

### **ANTONY CHU** ^1^, VENKATA SAGI^2^, GAUTHAM RAJAGOPAL^3^, KAJA PEDERSON^3^, SHANTANU SARKAR^3^, AMY LAUTENBACH^3^, ANISH AMIN^4^

#### ^1^Brown University, Providence, RI,^2^Baptist Medical Center, Jacksonville, FL,^3^Medtronic Inc., Mounds View, MN,^4^Riverside Methodist Hospital, Columbus, OH

**Introduction:** Class III Antiarrhythmic drugs (AAD) are known to cause QT prolongation and T‐wave morphology changes. Insertable cardiac monitors (ICM) have the capability to continuously monitor and detect dynamic changes in QT intervals and T‐wave morphologies.

**Methods:** The developed QT algorithm detects T‐wave and determines QTc intervals for every beat from ICM. QTc and T‐wave morphologies detected from continuously collected ICM ECG from patients undergoing Class III AAD loading and enrolled in a prospective clinical study from 3 sites were analyzed. Ensemble average of morphologies from ICM ECG during the first 3 days (period 1) of the 90‐day follow up period after AAD loading hospitalization discharge was compared with the last 3 days (period 2) of the 90‐day period to analyze differences in T‐wave and QRS morphologies and parameters such as QT, QRS width, Tpeak‐Tend were compared between the two periods.

**Results:** ICM ECG data during the study follow up period was available in 9 out of 21 patients (avg. age 71.5 years) enrolled in the QT clinical study with completed follow up and were included in this analysis. The ensemble average trends were computed from over 44,000 individual beats from ICM ECG. All 9 patients showed changes in QRS and/or T‐wave morphologies when comparing the ensemble average ICM ECG beats between periods 1 and 2 (Figure 1). 7 out of 9 patients(78%) had higher QTc (427±49 msec) during period 1 when compared to period 2 (417±50 msec). 6 patients(67%) had higher Tpeak‐Tend (107±42 msec) during period 1 compared to period 2 (87±29 msec). 4 out of 7 patients(57%) had higher QRS width (131±29 msec) during period 1 compared period 2 (129±34 msec) while 2 patients showed no change.

**Conclusions:** ICM QT monitoring performed utilizing a unique automated QT algorithm provides longitudinal dynamic QT trend assessment not previously possible. Class III AAD related changes in QTc interval and T‐wave morphology using ICM automation warrants further investigation as a tool for long term continuous cardiotoxicity assessment.

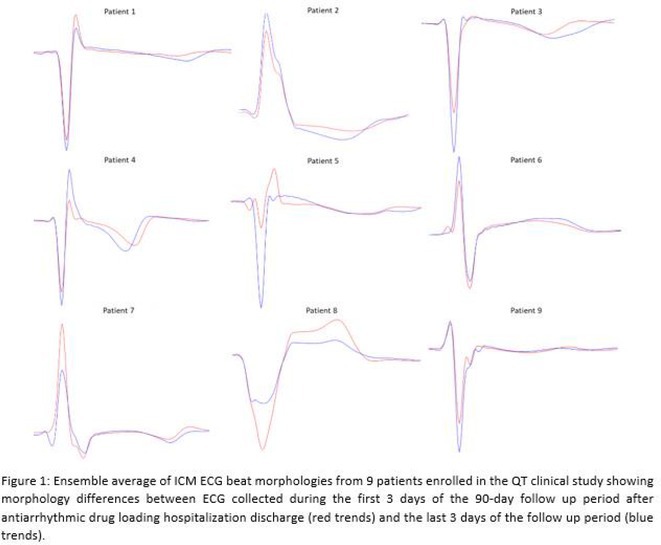

## COMPARISON OF SODIUM‐GLUCOSE COTRANSPORTER‐2 INHIBITOR AND DIPEPTIDYL PEPTIDASE‐4 INHIBITOR ON THE RISK OF NEW‐ONSET ATRIAL FIBRILLATION, STROKE, AND MORTALITY IN DIABETIC PATIENTS: A NATIONWIDE COHORT STUDY

### **KWANG JIN CHUN** ^1^, BUM SUNG KIM^2^

#### ^1^Kangwon National University Hospital, Chuncheon, Korea, Republic of,^2^Konkuk University Medical Center, Seoul, Korea, Republic of

**Introduction:** Sodium‐glucose cotransporter‐2 Inhibitor (SGLT2I) is reported to reduce new‐onset atrial fibrillation (AF). However, its cardiovascular benefits remains unclear. This study aimed to compare the risk of all‐cause mortality, new‐onset AF, and stroke between SGLT2I user and dipeptidyl peptidase‐4 inhibitor (DPP4I) user.

**Methods:** This study used data from Common Data Model of the Health Insurance Review and Assessment Service of Korea collected between January 1, 2012 and December 31, 2021. Diabetic patients without a history of AF was enrolled in this study. They were divided into two groups according to the anti‐diabetic drugs (SGLT2I or DPP4I). The primary outcome was all‐cause mortality. The secondary outcome was new‐onset AF and stroke.

**Results:** We identified 1,279,486 patients in SGLT2I group and 1,978,684 patients in DPP4I group. After 1:2 propensity score matching, 37,373 patients with SGLT2I and 63,742 patients with DPP4I were finally analyzed. SGLT2I user showed lower incidence of all‐cause mortality compared to DPP4I user (1.65% vs. 3.07%; hazard ratio (HR), 0.70; 95% confidence interval (CI), 0.63 ‐ 0.79; P<0.01). SGLT2I user also showed lower incidence of stroke (3.36% vs. 4.64%; HR, 0.87; 95% CI, 0.80 ‐ 0.95; P<0.01). However, new‐onset AF was not significantly different between the two groups (P=0.26).

**Conclusions:** In this nationwide cohort study, use of SGLT2I was associated with low risk of all cause mortality and stroke compared to use of DPP4I.

## GENDER DIFFERENCE IN LIFE THREATENING ARRHYTHMIAS IN PATIENTS WITH NON‐ISCHAEMIC CARDIOMYOPATHY

### IRENE CHUNG

#### Tan Tock Seng Hospital, Singapore, Singapore

**Introduction:** The mortality benefits of Automatic Intracardiac Defibrillator (AICD) in non‐ischaemic cardiomyopathy (NICMP) patients is less clear.

**Methods:** 447 patients with NICMP, heart failure with reduced left ventricular ejection fraction (LVEF<40% and significant coronary artery disease >70% stenosis has been excluded). Continuous variables were compared by Student t tests and categorical variables compared by Fisher exact test. Clinical endpoints include death, sudden cardiac death (SCD), ventricular arrhythmias (VA ) and Pulseless Electrical Activity (PEA)/ asystole arrests were recorded.

Mean age 63.07 ± 14.19 years, male 70%, 360 Chinese and 87 non‐chinese. Diabetes mellitus 38%, hypertension 76%, atrial fibrillation 38%, stroke 9%, ivabradine 9%, b‐blocker 88%, Angiotensin inhibitors/Angiotensin Receptor Blocker 66%, Sacubitrial/ Valsartan 26%, spironolactone 57%, SGLT‐2 inhibitor 9%. Mean follow‐up duration of 8.22 ± 4.25 years.

**Results:** 40 patients (9%) received device therapy (AICD and Cardiac resynchronization therapy), 19% secondary prevention, 81% for primary prevention. There were 21 appropriate device therapy, median time from diagnosis of NICMP to appropriate device therapy for VAs was 85.5 months (interquartile range 35‐131). 18/21 patients (86%) noted to have appropriate therapy for VT/ VT storms were males. HR for males 8, 95% CI (1.06, 60.34, p=0.044. There were 71 deaths (16%, 1.93% death/ year, 20 females and 51 males). Mean time to death 6.9 ± 3.80 year, no significance difference in gender (p=0.177). 27 SCD, 6 PEA (5 males and 1 female), 5 asystole (4 males and 1 female), 3 VAs, 13 undetermined.

**Conclusions:** Mortality in NICMP patients generally improved following guideline directed therapy. Male patients had significantly higher appropriate device therapy. Long median time from diagnosis to appropriate device therapy suggests underlying progressive scar forming cardiomyopathic process and 41% SCD were due to non‐shockable rhythm, may be related to the endstage disease.

## INCIDENCE OF ATRIAL FIBRILLATION AND CLINICAL COURSE OF PATIENTS WITH ACUTE ST‐ELEVATION MYOCARDIAL INFARCTION IN MODERN ERA OF EARLY REVASCULARIZATION IN ASIAN POPULATIONS

### IRENE CHUNG

#### Tan Tock Seng Hospital, Singapore, Singapore

**Introduction:** Current data on arrhythmic risks after acute myocardial infarction (MI) in the modern era of early revascularization in the Asian population are limited. We reported last year the arrhythmic risks after acute ST‐elevation MI is lower than previously reported. The purpose of this study was to investigate the incidence of atrial fibrillation (AF) and clinical course of patients after acute ST elevation MI in a contemporary cohort of Chinese patients in the era of early revascularization with intermediate term follow up.

**Methods:** 393 patients admitted with ST elevation myocardial infarction who underwent emergency revascularization, mean age 59.75 ± 12.85 years old, male 89%, diabetes mellitus 40%, hypertension 72%, hyperlipidaemia 78%, previous ischaemic heart disease or stroke 10%, smoker 65%, mean follow up of 4.32 ± 0.34 years. Mean Pain to Needle time (onset of chest pain to primary angioplasty) 4.88 ± 4.60 hours. Patients were divided into 2 groups, group 1 (n=267) and 2 (n=126) with pain to needle time less than or more than 6 hours respectively. Primary endpoints include atrial fibrillation and cardiovascular death.

**Results:** The left ventricular ejection fraction improved from 37.01 ± 10.42 to 45.30 ± 11.80 %. There were 31 cardiovascular deaths, 18 patients with atrial fibrillation, 13 occurred during the admission, 1 within 30 days, 2 between 1‐6 month, and 2 more than 1 year post MI. The incidence of CV death and AF was 1.83% and1.06% per year respectively. There was significantly fewer CV death in group 1 than 2 (15 vs 16, p=0.023).

**Conclusions:** Incidence of AF and CV death after acute ST elevation MI in the modern era of early revascularization in the Asian population is lower than previously reported may be due to prompt revascularization and reverse cardiac remodeling from modern day secondary prevention medications. Patient education and early admission is also recommended.

## RELATIONSHIP OF BUNDLE BRANCH BLOCK, QRS DURATION AND REVERSE CARDIAC REMODELING IN PATIENTS WITH NON‐ISCHAEMIC CARDIOMYOPATHY

### IRENE CHUNG

#### Tan Tock Seng Hospital, Singapore, Singapore

**Introduction:** QRS prolongation has been shown to predict mortality in patients with heart failure and reduced left ventricular function. Reduction of QRS duration has been reported to associate with better prognosis. We aim to study the relationship of bundle branch block, QRS duration and cardiac remodeling in patients with non‐ischaemic cardiomyopathy.

**Methods:** 455 patients with non‐ischaemic cardiomyopathy, left ventricular function <40%, significant coronary artery disease >70% stenosis excluded. Mean follow‐up duration of 8.22 ± 4.25 years. Mean age 62.95 ±14.16, 319 males (70%). 80 patients had bundle branch block (BBB), 48 left BBB, 32 right BBB. 48% diabetes mellitus, 76% hypertension, 25% atrial fibrillation, previous stroke 6%. 14% on ivabradine, 78% b‐blocker, 55% angiotensin converting enzyme inhibitor (ACE‐I)/ Angiotensin receptor blocker, 36% Sacubitrial/ Valsartan, 59% minerocorticoid receptor antagonist, 11% sodium glucose transport inhibitor. 10% Cardiac resynchronization therapy. Clinical endpoints include ventricular tachyarrhyhmias (Vas) and cardiovascular death.

**Results:** 34% cardiovascular death and mean time to diagnosis to VAs 5.26 ± 3.83 years. 12 LBBB and 10 RBBB patients had significant LVEF improvement. LVEF improvement and QRS reduction were 23 ± 10.18%, ‐30 ±21.64ms and 26.2 ±8.88%, 3.6 ±15.90ms in LBBB and RBBB respectively. LVEF improvement and QRS reduction were significant in LBBB (p<0.01) not in RBBB. 5 patients with LBBB had significant LVEF improvement after ACE‐I switched to Sacubitrial/ Valsartan and reverse cardiac remodeling may take up to 17 months.

**Conclusions:** QRS reduction correlates LVEF improvement in non‐ischaemic cardiomyopathy patients with LBBB. Sacubitrial/ Valsartan was more efficacious in 41% of these patients. Long mean time from diagnosis to appropriate device therapy suggests underlying progressive scar forming cardiomyopathic process.

## SWITCHING TO SACUBITRIL‐VALSARTAN IS ASSOCIATED WITH SHORTENING OF QRS DURATION IN NON‐ISCHAEMIC CARDIOMYOPATHY

### IRENE CHUNG

#### Tan Tock Seng Hospital, Singapore, Singapore

**Introduction:** Prolongation of QRS is associated with poorer prognosis in patients with LBBB and left ventricular systolic dysfunction. Shortening of QRS has been shown to have favourable prognosis.

**Methods:** NA

**Results:** A 72 years old woman was referred for non‐ischaemic cardiomyopathy on 18/3/22. She was known to have LBBB with QRS 148ms, left ventricular ejection fraction 35% since 02/10/2014. Past medical history includes diabetes mellitus and chronic kidney disease stage 3b with GFR 40 ml/min. She was on atenolol and losartan, switched to sacubitril‐valsartan and started on dapagliflozin same day. Serial echocardiograms showed reverse cardiac remodelling, left ventricular ejection fraction increased from 35 to 45% with reduction in QRS duration from 156 to 132ms, with NYHA improved from II to I over 17 months.

**Conclusions:** This case showed switching to Sacubitril‐valsartan is associated with shortening of QRS duration in non‐ischaemic cardiomyopathy and suggests preferential efficacy of sacubitril‐valsartan over angiotensin receptor blocker as well as reverse cardiac remodeling may take longer than previously suggested in guidelines.

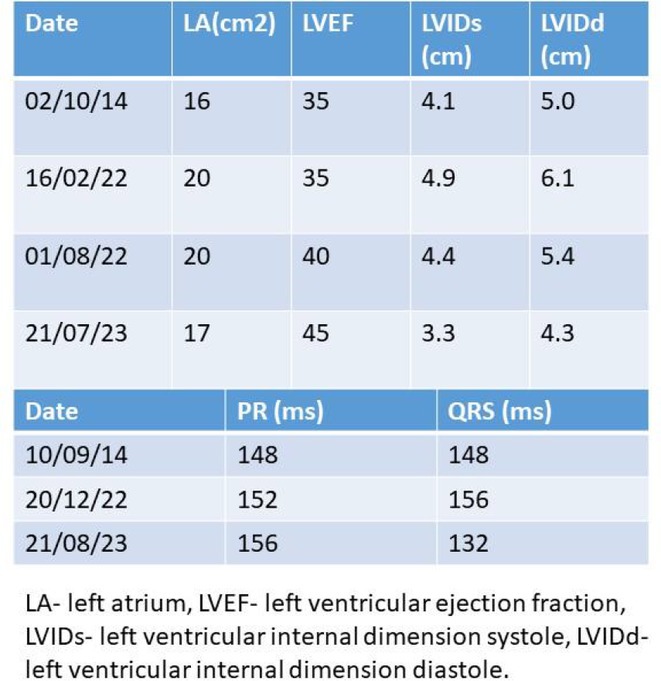

## USEFULNESS OF 7‐DAY MONITORING FOR THE EVALUATION OF DAILY VARIATION OF PVCBURDEN: A STUDY WITH SINGLE LEAD ECG (INTERIM ANALYSIS FROM THE SINGLE CENTER)

### **TAE‐WAN CHUNG**, MINSU JUNG, JONGMIN HWANG, HYOUNGSEOB PARK

#### Keimyoung University Dongsan Hospital, Daegu, Korea, Republic of

**Introduction:** Premature ventricular complex (PVC) can provoke symptoms such as palpitation, fatigue, and shortness of breath and may induce left ventricular (LV) dysfunction, especially if the daily burden is high (over 15%). In the case of PVC‐induced cardiomyopathy, catheter ablation helps improve LV systolic function. The PVC burden of the day is an essential factor in deciding the catheter ablation. The long‐term monitoring up to 14 days showed the daily variation of PVC burden from 3.6 to 9.9%. The information about the optimal monitoring duration for evaluating PVC burden is little. The purpose of this study is to evaluate the appropriate duration of the monitoring for the PVC burden.

**Methods:** It was a single‐center prospective observational study, and 20 patients with PVC were enrolled. Themean age was 55.7 ± 12.4 years old, and 7 patients (35%) were men. One was dropped out due to severecontact dermatitis, and one subject underwent only 3‐day monitoring. The primary endpoint was the differencein PVC burden between the first 24 hours and the maximal burden during the 7‐day monitoring. The PVCburden during the first 3 days and the later 4 days was also compared.

**Results:** The PVC burden on the first day was 9.5±11.6%, and the highest burden during 7‐day was12.9±12.8%, which showed a significant difference (3.39±5.54%, p=0.001). However, the PVC burden duringthe first 3 days was 11.8±12.6%, which was not different from the PVC burden during the later 4 days(12.4±13.1%, P=0.39). The average of the duration to the peak of PVC burden was 3.84 days.

**Conclusions:** The only 24 hour‐monitoring may not reflect the actual PVC burden because of the daily variationof the burden. However, 3‐day monitoring may be sufficient for evaluating PVC burden rather than longerduration. The results of the multicenter observational study will be available soon.

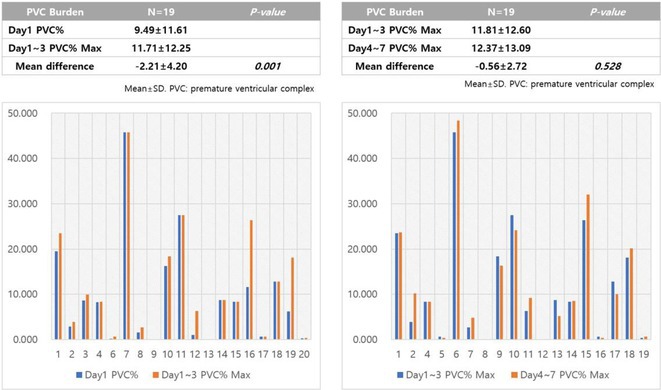

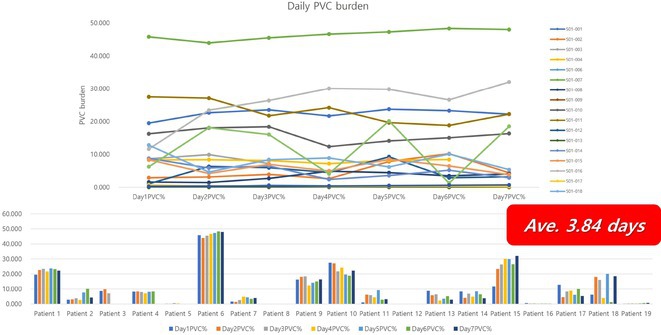

## INTRAPULMONARY VEIN LOCAL REENTRY ATRIAL FLUTTER MIMICKING TYPICAL ATRIAL FLUTTER

### **WEIHSIN CHUNG**, YEN‐NIEN LIN, KUAN‐CHENG CHANG

#### China Medical University Hospital, Taichung, Taiwan

**Introduction:** Atypical atrial flutter (AFL) is a diagnostic and therapeutic challenging arrhythmia. Here We presented a rare case of intrapulmonary vein (PV) reentry AFL after prior PV isolation.

**Methods:** N/A

**Results:** The 63‐year‐old male with a history of hyperlipidemia, paroxysmal atrial fibrillation post prior radiofrequency catheter ablation, and congenital coronary abnormality with arteriovenous fistula presented with persistent atrial flutter (Figure 1A) for 2 weeks despite antiarrhythmic drugs being given. Therefore he was admitted for electrophysiology study and catheter ablation. After femoral access was established, programmed atrial stimulation with rapid atrial pacing at 240ms induced a sustained atypical AFL which showed right atria comprised only 40% of the AFL cycle length with earliest activation sites at septal superior vena cava and propagated laterally and septally simultaneously and then collides at left lateral right atria (Figure 1B). During mapping, AFL degenerated into atrial fibrillation for which cardioversion with 200J 3 times was performed. The decision was made to perform PV isolation and map left atria for the AFL. Bilateral pulmonary veins were reconnected, and ablation was performed in a point‐by‐point fashion with a setting of 35W~40W targeting an ablation index of 380‐400. During ablation of the last point, atrial fibrillation transformed into AFL and then terminated (Figure 1C). The entrance block and exit block of 4 PVs were confirmed. Importantly, multiple potentials were recorded during mapping in the right superior PV, which contained 90% of the cycle length of the AFL with a figure‐of‐eight wavefront propagation (Figure 1D). Within the right superior PV, a slow conduction area was obtained (Figure 1E). After 5secs of ablation, the intrapulmonary vein reentry was terminated.

**Conclusions:** Intrapulmonary vein reentry AFL is a rare mechanism of atypical AFL. This should be taken into consideration in a patient with prior PV isolation presenting with a saw‐tooth AFL.

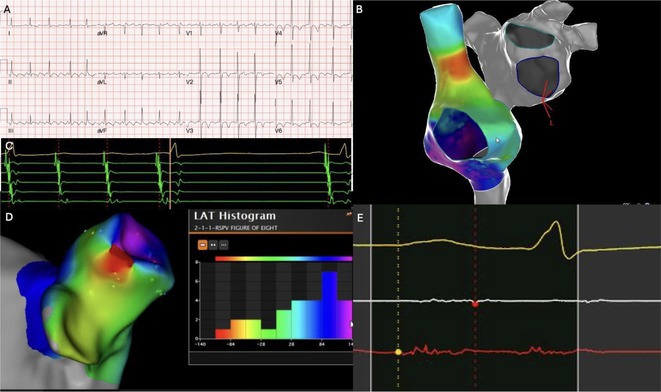

## CATHETER ABLATION FOR ATRIAL FIBRILLATION IN PATIENTS WITH REDUCED LEFT VENTRICULAR EJECTION FRACTION: ACUTE SAFETY OUTCOMES AND PROCEDURAL CHARACTERISTICS

### **ROSE CROWLEY** ^1^, JEFF MA^2^, MATTHEW MORTON^1^, SWETHA VASUDEVAN^2^, HAILEY MILLER^2^, LOUISE SEGAN^1^, JEREMY WILLIAM^1^, DAVID CHIENG^1^, HARIHARAN SUGUMAR^1^, ALEKSANDR VOSKOBOINIK^1^, SANDEEP PRABHU^1^, LIANG‐HAN LING^1^, PETER KISTLER^1^

#### ^1^Alfred Health, Melbourne, Australia,^2^Monash University, Melbourne, Australia

**Introduction:** Catheter ablation(CA) is a class 1 indication in patients with atrial fibrillation (AF) and heart failure with reduced ejection fraction (HFrEF). However, concerns exist about the procedural safety in these higher risk patients. The aim of this study was to describe the procedural characteristics and acute safety outcomes in patients with and without HFrEF (LVEF < 50%) undergoing CA for AF.

**Methods:** All AF ablations performed at a single tertiary centre between 2013 and 2023 were reviewed for procedural characteristics and in‐hospital complications.

**Results:** Of the 1071 AF ablations, 280 (26.1%) were performed in patients with LVEF<50% at time of procedure. There were higher rates of persistent AF (81.4% vs 43.4%, p<0.001), higher CHA2DS2‐VASc scores (2(1‐3) vs 1(0‐2), p<0.001) and fewer women (16% vs 33.9%, p<0.001) in the HFrEF group. Rates of left atrial appendage(LAA) thrombus on transoesophageal echo were higher (p=0.022) despite uninterrupted anticoagulation, and more patients were in AF at time of procedure (52.9% v 26.6% p<0.001) in the HFrEF group. Procedure duration (p<0.001) and RF time (p<0.001) were longer, and posterior wall isolation was performed more frequently (43.6% vs 25.8%, p<0.001) in the HFrEF group, rates of ablation at other non‐pulmonary vein sites did not differ. Major in hospital complications were more common in the HFrEF group (3.2% vs 0.8% p=0.003), as was heart failure decompensation (p=0.006), bradycardia (p=0.031) and hypotension (p=0.012). Median hospital length of stay was 1 night in both groups.

**Conclusions:** Patients with HFrEF undergoing AF ablation were more likely to have persistent AF, LAA thrombus despite anticoagulation, and major complications, compared to patients with LVEF>50% at time of procedure. These factors should be considered when selecting patients with HFrEF for CA.

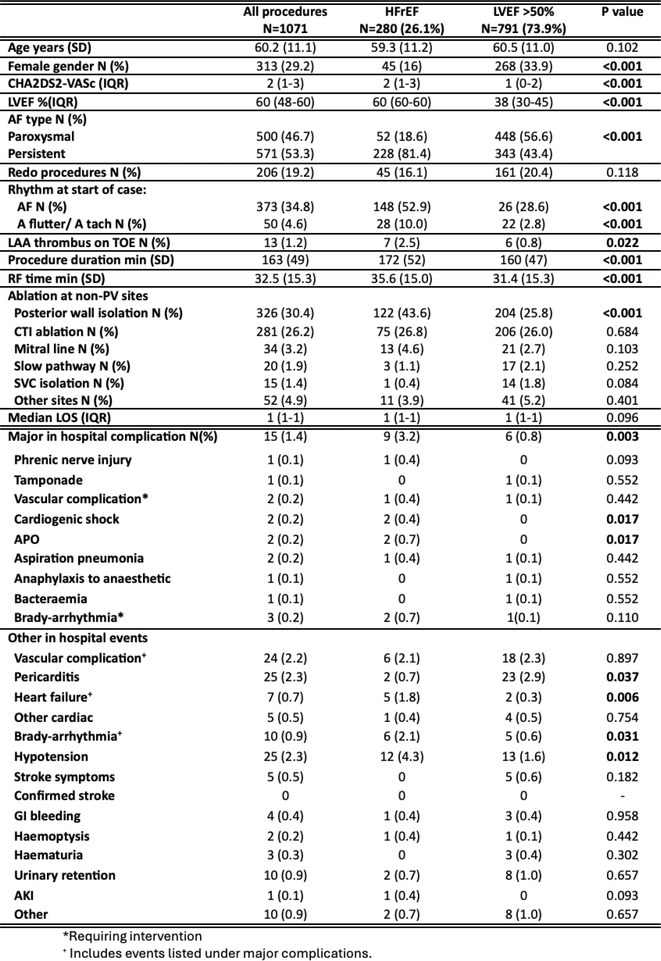

## CHALLENGING EP CASE: INCESSANT ATRIOVENTRICULAR REENTRANT TACHYCARDIA SUCCESSFULLY ABLATED FROM NON‐CORONARY SINUS CUSP

### **PHONG DANG**, KIEN VO, PHONG PHAN

#### Vietnam National Heart Institute, Hanoi, Viet Nam

**Introduction:** Atrioventricular reentrant tachycardia (AVRT) rarely results in heart failure. We present a case of incessant short RP supraventricular tachycardia leading to decompensated heart failure, which was successfully terminated by ablating a concealed accessory pathway in non‐coronary sinus cusp.

**Methods:** N/A

**Results:** A 45‐year‐old woman presented to our emergency department with signs of decompensated heart failure and palpitations with a regular heart rate of 180 beats per minute. The electrocardiogram (ECG) revealed a narrow QRS complex tachycardia with a short RP interval. The tachycardia terminated with adenosine injection and electrical cardioversion but returned almost immediately. Amiodarone also failed to maintain sinus rhythm. Because the patient deteriorated, we decided to perform an emergency ablation. Intracardiac tracings showed a 1:1 V‐A activation during the tachycardia, with concentric atrial activation. EP maneuvers (VOP, VA linking, His‐synchronous PVC) suggested the tachycardia mechanism was an AVRT with a concealed right‐sided accessory pathway (AP). The tachycardia terminated easily by overdrive pacing, but the sinus rhythm could not be maintained. We constructed a 3D activation map of the right atrium and tricuspid annulus, which revealed an anteroseptal AP. The site of earliest atrial activation during the tachycardia was proximal to His‐bundle, and attempts to ablate near the target site were unsuccessful. We decided to map the aortic non‐coronary cusp (NCC) and found a site with earlier retrograde atrial activation there. We delivered an ablation with an irrigated catheter in NCC and terminated the tachycardia in 3 seconds. The tachycardia was then non‐inducible, and there was no retrograde V‐A conduction.

**Conclusions:** Atrioventricular reentrant tachycardia can sometimes be incessant and requires emergency catheter ablation. The anteroseptal accessory pathway, which is adjacent to the His‐bundle, can be ablated safely from the non‐coronary sinus cusp.

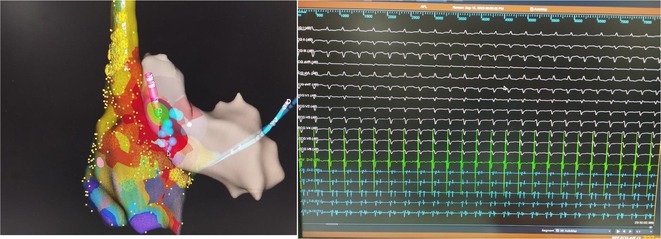

## STEREOTACTIC ARRHYTHMIA RADIOABLATION (STAR) FOR REFRACTORY VENTRICULAR TACHYCARDIA‐ INITIAL, MULTICENTRE EXPERIENCE IN AN AUSTRALIAN COHORT

### SOUVIK DAS

#### Royal Melbourne Hospital, Melbourne, Australia

**Introduction:** Stereotactic arrhythmia radioablation (STAR) is a novel, non‐invasive technique for the management of refractory ventricular tachycardia (VT). The aim of this study was to assess the feasibility, efficacy, and safety of STAR in an Australian cohort.

**Methods:** From February 2020 to August 2023, 15 patients with drug‐refractory VT, who had either failed catheter ablation or were unsuitable for it, were evaluated for suitability for STAR. Twelve suitable patients were treated in 2 Australian centres with 25 Gy in one fraction. Treatments were delivered without anaesthesia. Efficacy endpoints were defined as number of VT episodes as well as anti‐tachycardia pacing (ATP) and shocks after STAR. Mortality and adverse event data were collected over 12‐month follow up (FU).

**Results:** In the 8 patients who survived blanking period (6 weeks), a significant reduction (54.9%, P=0.017) in VT burden was observed over a 6‐month FU. However, 75% of these patients experienced VT recurrence. Over a 12‐month FU, 5 patients died, and 3 moderate adverse events were recorded (undersensing of defibrillator lead, increased rate of reflux and radiation pneumonitis).

**Conclusions:** This study summarises initial Australian experience for treating refractory VT with STAR and demonstrates that STAR can decrease VT burden significantly over a 6‐month FU with acceptable acute side‐effects profile. VT recurrence occurred in 75% of the patients over the 6‐month FU.

## COMPARATIVE ANALYSIS OF PULMONARY VEIN ISOLATION (PVI) ALONE VERSUS PVI WITH ADDITIONAL LEFT ATRIAL POSTERIOR WALL ISOLATION BY PULMONARY VEIN ABLATION: A SINGLE‐CENTRE STUDY

### **ALEXANDER DASHWOOD**, EMILY KOTSCHET, SAADAT SALEEMI, DAVID ADAM, LOGAN BITTINGER, JEFFREY ALISON, COLIN MACHADO, HUI‐CHEN HAN, SING HUEY CHENG, BRENDEN TIAN, VIVIAN KY, JULIET YOUNG, STEWART HEALY

#### Victorian Heart Hospital, Melbourne, Australia

**Introduction:** Pulsed‐field ablation (PFA) presents a novel approach for achieving left atrial posterior wall isolation (LAPWI), while simultaneously mitigating procedural complications.

**Methods:** To evaluate the practical feasibility and safety of incorporating LAPWI using PFA alongside pulmonary vein isolation (PVI), compared to PVI alone. Methods: De‐identified data from 251 patients undergoing PFA at the Victorian Heart Hospital (VHH) from October 2022 to April 2024 were analyzed.

**Results:** Among 251 patients, 100 underwent additional LAPWI following PVI, while 151 patients received PVI only. Demographic and procedural characteristics are summarized in Table 1. LAPWI was performed in patients with more persistent forms of atrial fibrillation (AF). The addition of LAPWI extended the procedure duration by an average of 5±2.2 minutes and required 24±7 additional PFA applications. In the PVI + LAPWI group, one major complication occurred at day 7, presenting as acute kidney injury. Anticoagulation was suspended, and the patient experienced a stroke with full resolution. Conversely, the PVI‐only group experienced three admissions for heart failure and two cases of pericarditis. At 30 days, four patients (5%) developed atrial arrhythmias in the PVI + LAPWI group compared to seven (5.5%) in the PVI‐only cohort.

**Conclusions:** The addition of LAPWI using PFA is both feasible and safe, with minimal extension of procedural duration. Longer‐term follow‐up is warranted to assess efficacy and durability.

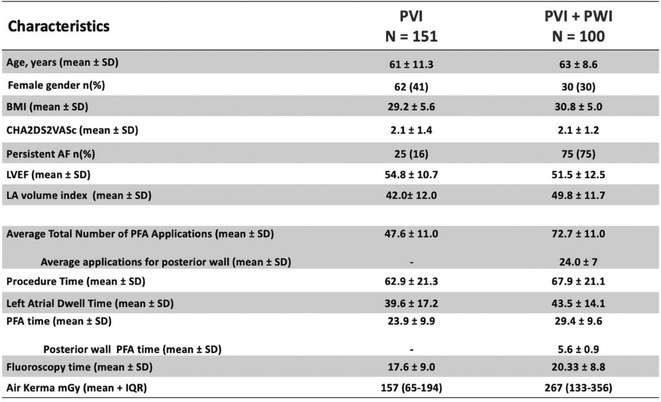

## CLINICAL AUDIT OF CARDIAC SCREENING IN NEW ZEALAND FOOTBALL

### **ANGUS J. DAVIS** ^1^, BELINDA GRAY^1^, TIM DRISCOLL^1^, JOHN W. ORCHARD^1^, BRUCE HAMILTON^2^, ROB DOUGHTY^3^, MARK FULCHER^4^, JESSICA J. ORCHARD^1^

#### ^1^The University of Sydney, University of Sydney, Australia,^2^High Performance Sport New Zealand, Auckland, New Zealand,^3^University of Auckland, Auckland, New Zealand,^4^Axis Sports Medicine Specialists, Auckland, New Zealand

**Introduction:** Cardiac screening of elite footballers for conditions associated with sudden cardiac arrest/death (SCA/D) is a requirement of Fédération Internationale De Football Association (FIFA). This study aimed to report the findings, cardiac diagnoses, and outcomes from the New Zealand Football (NZF) screening program for FIFA tournaments since 2012.

**Methods:** This was a retrospective cohort study of female and male footballers from NZF who were screened for a FIFA event from January 2012 to November 2023. Footballers were screened with a history, physical examination, resting 12‐lead electrocardiogram (ECG) and transthoracic echocardiogram (TTE). An audit (March 2024) reviewed screening records, comprising ECGs, TTEs, demographic data, follow‐up testing, and diagnoses. A footballer was included if sufficient details about the screening outcome, an ECG trace, and a TTE report in English was available. ECGs were reviewed using the International Criteria for Athlete ECGs (2017). Small numbers of results were reported as less than 5.

**Results:** In total, 147 footballers (60% female, mean age 19.2±3.7 years) were included. There were less than 5 diagnoses of conditions associated with SCA/D. 4.8% of ECGs were abnormal however no patient was subsequently identified to have underlying structural heart disease in TTEs. Female footballers were less likely to have left ventricular hypertrophy according to voltage criteria (8.4% vs 37.8%, p<0.001), and early repolarisation (4.5% vs 35.6%, p<0.001). There were no sex differences in rates of abnormal ECG findings or abnormal T‐wave inversion. In TTEs, females had significantly lower indexed left ventricular end diastolic volumes (62±8.8 ml/m^2^ vs 74±12.9 ml/m^2^, p<0.001), and indexed left ventricular end systolic volumes (22±5.5 ml/m^2^ vs 30±6.9 ml/m^2^, p<0.001). No athletes retired for cardiac reasons and there were no incidents of SCA/D during the period.

**Conclusions:** This audit represents an insight into the screening program of an individual sport in New Zealand. The findings are consistent with those from other cohorts and the low level of diagnoses, and no instances of SCA/D, indicates that NZF has a healthy group of athletes under their care.

## THORACOSCOPIC LEFT ATRIAL APPENDAGE EXCLUSION FOR CURATIVE INTENT OF ATRIAL FIBRILLATION

### TINA CHERIAN^1^, MARTIN STILES^1,2^, **ZACH DEBOARD** ^1,3^

#### ^1^Waikato Hospital, Hamilton, New Zealand,^2^Waikato Clinical School ‐ University of Auckland, Hamilton, New Zealand,^3^University of Auckland Faculty of Medical and Health Sciences, Auckland, New Zealand

**Introduction:** The left atrial appendage (LAA) is an under recognized source of atrial fibrillation (AF). A significant reduction in AF/atrial tachycardia has been shown with electrical isolation (EI) of the LAA. Epicardial ligation of the LAA has demonstrated EI and is a component of surgical ablation. We present a case of standalone surgical LAA ligation using an AtriClip for curative intent of AF.

**Methods:** N/A

**Results:** A 72‐year‐old man with persistent, symptomatic AF refractory to multiple cardioversions and antiarrhythmics had undergone 4 catheter ablations with temporary resolution post LAA isolation. Serial mapping demonstrated ongoing arrhythmia generation from the LAA. A multidisciplinary consensus felt repeat catheter ablation yielded increased risk and cost compared to surgery. Given the electrical and structural isolation afforded by epicardial LAA occlusion the patient was offered a thoracoscopic epicardial ligation clip. Open surgical ablation was not offered due to comorbidities and thoracoscopic ablative procedures not offered in New Zealand. A 3‐port VATS technique was used. A 35mm AtriCure PRO‐2 AtriClip (Mason, OH, United States) was selected for ligation. During manipulation of the LAA into the clip the LAA went into rapid AF with the heart entering a rate‐controlled AF. Upon securing the clip at the base of the LAA the heart converted to sinus rhythm and, visually, the LAA continued to fibrillate. 30 seconds post clip deployment all contractile functions of the LAA ceased. Intraoperative transesophageal echocardiography demonstrated a residual LAA stump of 2mm with no flow (see figure). Operative time was 21 minutes. The patient was discharged the following morning and has since remained in sinus rhythm beyond 6 months post‐surgery.

**Conclusions:** To our knowledge this is the first documented case of surgical ligation for EI of the LAA for curative intent of AF and isolated ablative purposes. In select patients this could be a future treatment approach in those with LAA as a trigger for AF.

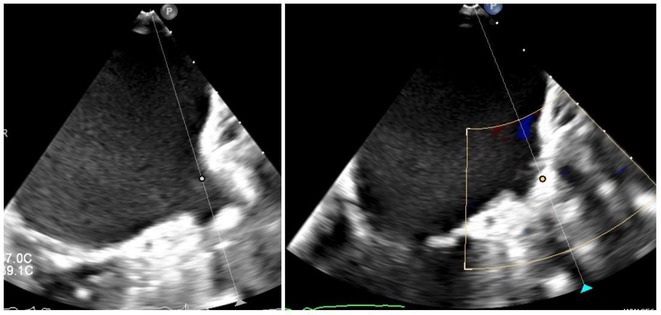

## TRENDS IN SURGICAL ABLATION FOR ATRIAL FIBRILLATION IN AOTEAROA NEW ZEALAND

### **ZACH DEBOARD** ^1,2^, STEPHANIE CH'NG^3^, MARTIN STILES^1,4^, NISHITH PATEL^1,2^

#### ^1^Waikato Hospital ‐ Health New Zealand, Hamilton, New Zealand,^2^University of Auckland Faculty of Medical and Health Sciences, Auckland, New Zealand,^3^Wellington Regional Hospital ‐ Health New Zealand, Wellington, New Zealand,^4^Waikato Clinical School ‐ University of Auckland, Hamilton, New Zealand

**Introduction:** Although guidelines support surgical ablation for atrial fibrillation incongruences remain between cardiac professional societal endorsements and real‐world application. Our goal was to examine the practises in New Zealand to increase surgical ablation and left atrial appendage management.

**Methods:** All adult cardiac surgical patients from two New Zealand public centres from 1 January 2015 to 31 December 2021 with a history of preoperative atrial fibrillation were retrospectively reviewed. Patient demographics, type of atrial fibrillation, index operation, and ablation lesion set were collected.

**Results:** 1208 patients with preoperative atrial fibrillation underwent cardiac surgery during the study period. Of these, 94 (7.8%) had some form of surgical ablation, all of which were concomitant procedures. There was a significantly greater percentage of male and younger patients receiving ablation compared to those not receiving ablation (male 88.3%, p<0.0001; mean age 63.2 vs. 66.7 years, p=0.0004). Pulmonary vein isolation and bi‐atrial Cox‐Maze IV were the most common lesion sets used; however, a large variation was observed. Twenty‐five patients underwent left atrial appendage exclusion as an isolated atrial fibrillation therapy during the index operation.

**Conclusions:** Atrial fibrillation has been undertreated surgically in New Zealand. Left atrial appendage exclusion has been an underutilised therapy which can be applied when ablation is not indicated. Surgeons are encouraged to adhere to current guidelines and perform surgical ablation where appropriate. Improved data collection and standardisation of therapy are suggested to aid with efforts to study surgical ablation and patient outcomes.

## IATROGENIC STROKE CAUSED BY INADVERTENT RIGHT ATRIAL PACING LEAD WENT TO LEFT ATRIUM

### **STEPHANIE DHARMAPUTRI** ^1^, GIKY KARWIKY^2^, MOHAMMAD IQBAL^2^, CHAERUL ACHMAD^2^

#### ^1^Cardiology and Vascular Medicine Department, Faculty of Medicine, Universitas Padjadjaran/Dr. Hasan Sadikin General Hospital, Bandung, Indonesia,^2^Electrophysiology Division, Cardiology and Vascular Medicine Department, Faculty of Medicine, Universitas Padjadjaran/Dr. Hasan Sadikin General Hospital, Bandung, Indonesia

**Introduction:** The transvenous pacing leads can pass easily through a patent foramen ovale (PFO) into the left heart but it has rarely been reported (approximately 3.4%). Some serious consequences are left heart valves damage or infection and arterial thromboembolic. Here, we present a thromboembolic case due to inadvertent pacing lead malposition.

**Methods:** N/A

**Results:** A 90‐years‐old male complained whole body weakness and tiredness with normal physical examination. The initial 12‐leads electrocardiography (ECG) revealed first degree atrioventricular block with marked prolonged PR interval and right bundle branch block. Initial transthoracic echocardiography (TTE) showed preserved LV systolic function. Implantation of dual chamber permanent pacemaker (PPM) was done, and the 12‐leads surface ECG after implantation recorded pacing rhythm with atrial sensing and ventricular pacing. Two days later, he came back with half body weakness along with speech difficulty which gradually resolved. Head scan revealed multiple lacunar infarction at regio basal ganglia and lateral periventricular substansia alba bilateral. Repeated TTE revealed undulating mass at LA, suggestive lead dislodgement and confirmed by chest radiography, which found the supposed RA lead at LA area. Lead reposition procedure was advised but he refused, even asked it to be extracted. After extraction, residual symptoms and heart rhythm was observed for 24 hours then he was discharged.

**Conclusions:** Cases of lead malposition to left heart through PFO is rare, but this patient was experienced thromboembolic event caused by possible undiagnosed PFO and abnormal thoracic anatomy due to aging. The inadvertent left heart lead placement provides a nidus for thrombus formation and when it was discovered early (particularly within the first 2 weeks), the treatment of choice is lead removal and repositioning, with reassurance from echocardiography that there is no thrombus. To reduce events like this in the future, it is important to not only identify potential risk factors, but also do an early identification with at least two fluoroscopic views at the time of device implant.

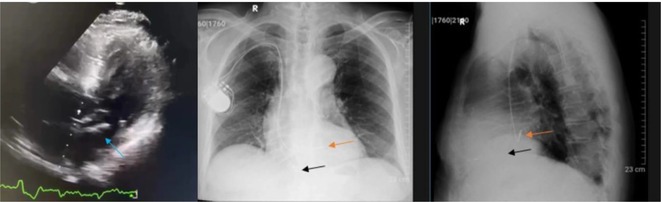

## ALTERNATIVE APPROACH TO SYSTOLIC HEART FAILURE WITH SEVERE FUCTIONAL MITRAL REGURGITATION

### **RAFAEL DIAMANTE**, NICHOLAY TEODOROVICH, MOSHE SWISSA

#### Kaplan Medical Center, Rehovot, Israel

**Introduction:** Conduction system pacing has been proved to be an efficacy method alternative to coronary sinus resynchronization in systolic heart failure patients. We present case report of ischemic cardiomyopathy patient with severe functional mitral regurgitation.

**Methods:** Is 90 years old patient with left ventricular dysfunction. functional class NYHA II\III. Under Optimal Pharmacological medical treatment. ECHOCARDIOGRAM:EF 35%, severe mitral regurgitation, moderate pulmonary hypertension 54 mmhg.ECG: Sinus 75 BPM QRS 130ms PR 180ms.Submitted to CSP procedure. After attempting to LBBBAP with suboptimal pacing QRS,was decided to add coronary sinus electrode in other to achieve resynchronization‐LOT‐CRT. CS electrode were located in Antero‐lateral branch.

**Results:** Simultaneous stimulation results in narrow complex QRS 100 ms.Echocardiogram month late after implant shows improvement of EF from 35%to 40%, disappearance of pulmonary hypertension and reduction of severity mitral regurgitation to moderate. Patient experiment increase of clinical status NYHA I\II.

**Conclusions:** This case highlight LBBBAP Optimization‐CRT as feasible and safety and seems to be great alternative resynchronization method to patients with systolic heart failure and functional moderate to severe mitral regurgitation.

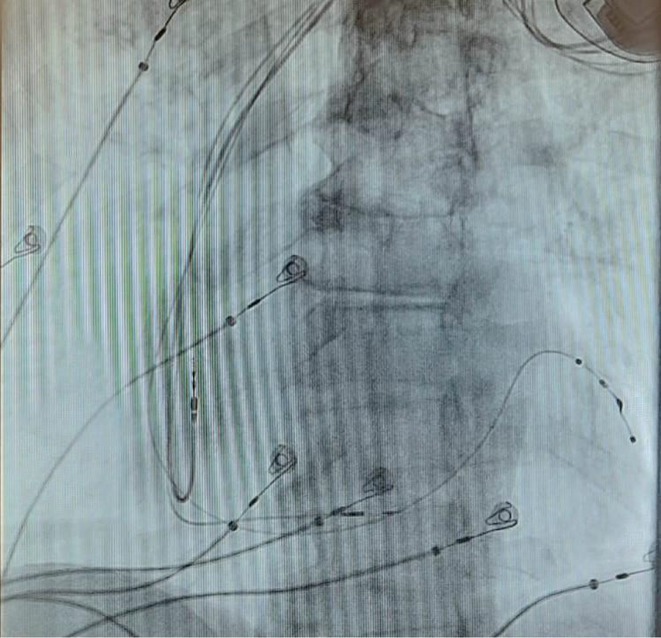

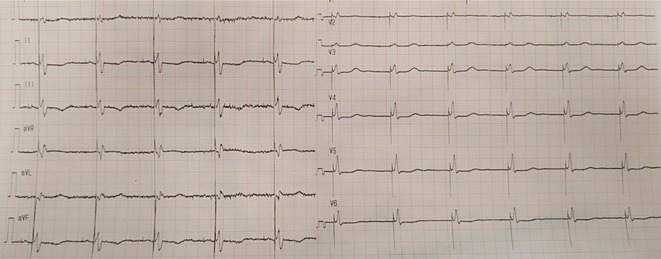

## INTRAMURAL VENOUS ETHANOL INFUSION FOR LEFT VENTRICULAR SUMMIT TACHYCARDIA IN A PATIENT WITH HYPERTROPHIC CARDIOMYOPATHY DURING EXTRACORPOREAL MEMBRANE OXYGENATION TREATMENT

### **LEI DING**, MIN TANG

#### fuwai hospital, Beijing, China

**Introduction:** For patients with unstable hemodynamic status and left ventricular (LV) summit origin ventricular tachycardia (VT), ethanol infusion under circulatory support device may be a choice.

**Methods:** N/A

**Results:** A 61‐year‐old man with hypertrophic cardiomyopathy (HCM) presented to our department. He experienced the electrical storm and multiple ICD shocks, the esmolol, amiodarone, and dexmedetomidine were continuously intravenous administrated. Echocardiography revealed non‐obstructive HCM with a LV ejection fraction (LVEF) of 40%. The ablation procedure was organized. When VT was induced, the patient was loss of conscious and became hemodynamically unstable requiring cardioversion. Thus, veno‐arterial extracorporeal membrane oxygenation (ECMO) was initiated for circulatory support. During the procedure, the patient continued to have incessant VT with a cycle length (CL) at 480ms. The VT morphology was right bundle branch block with left superior axis deviation, transition before V1, and the QRS duration is 180‐190ms **(Figure 1A)**. The morphology indicating the LV summit origin. A 3D electroanatomic mapping was performed, the earliest site in the endocardium was 21ms earlier than the QRS complex with a r wave in unipolar electrogram and the ablation was no response **(Figure 1A)**. Epicardial puncture from subxiphoid was then performed and no earlier sites or fractioned electrograms **(Figure 1B)**. We did the selective angiography of the summit region veins through posterior lateral vein access **(Figure 1C)** and infused 2ml 96% ethanol slowly. The CL was prolonged to 523ms. We tried the angiography through great cardiac vein access and infused another 2ml ethanol targeted the same region. The VT was then terminated and the myocardial staining area was seen in the LV summit region **(Figure 1D)**. The patient remained in sinus rhythm and ECMO was discontinued within 12 hours. The patient was free of ICD shock and LVEF normalized 1 month after discharge **(Figure 1E)**.

**Conclusions:** Ethanol infusion for LV summit origin VT under circulatory support was safe and efficacy.

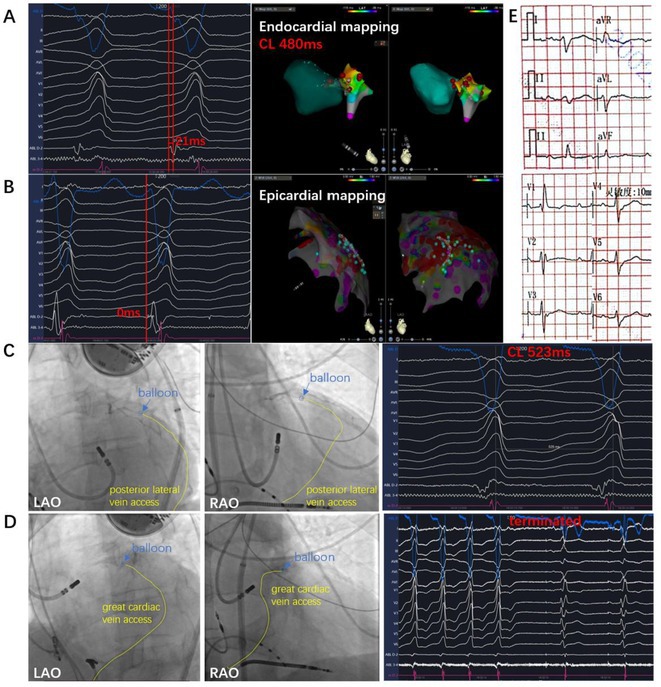

## RECURRENT PAROXYSMAL ATRIAL FIBRILLATION ORIGINATING FROM THE PERSISTENT LEFT SUPERIOR VENA CAVA IN A PATIENT WITH ABSENT RIGHT SUPERIOR VENA CAVA: A CASE REPORT

### **LEI DING**, MIN TANG

#### fuwai hospital, Beijing, China

**Introduction:** For recurrent atrial fibrillation (AF), non‐pulmonary foci are important triggers and can locate in congenitally anomalous veins. In this report, we describe a case that had persistent left superior vena cava (PLSVC) ectopy initiating AF with absent right superior vena cava (ARSVC).

**Methods:** N/A

**Results:** A 69‐year‐old man suffered from symptomatic paroxysmal AF and refractory to antiarrhythmic drugs for 10 years. Seven years ago, he underwent pulmonary veins isolation by cryoablation and recurred two years ago. He was referred for radiofrequency catheter ablation in our center. The preprocedural computed tomographic angiography (CTA) revealed a PLSVC with an ARSVC. Because of the ARSVC, the traditional pull‐down maneuver to conduct septal puncture was impossible. Therefore, we conducted the high‐pressure angiography of the right atrium first **(Figure 1A)**. Under the guidance of the angiography, spine, and the PLSVC, the transseptal puncture was completed. Then, we reconstructed the right atrium, the left atrium, and the PLSVC using a circular mapping catheter (**Figure 1B‐C**, PENTARAY, Biosense Webster, Inc., California). Although there was no reconduction in the pulmonary veins, we enlarged the ablation line to the vestibule (**Figure 1C**). Following the adenosine triphosphate infusion, the earliest local activation was recorded inside the PLSVC and then triggered the AF **(Figure 1D)**. There were fragmentated electrograms inside the PLSVC **(Figure 1E)**. We considered the PLSVC was the trigger and circumferential ablation was conducted (25‐30W, 43°C, 17 ml/min, **Figure 1F**). During the ablation, the AF was intermittent returned to the sinus rhythm. After the isolation of the PLSVC, the patient kept in sinus rhythm and AF could not be induced again (**Figure 1G**).

**Conclusions:** We presented a rare case of non‐pulmonary vein foci originating from the PLSVC in a patient with ARSVC. For this kind of patient, the preprocedural CTA and high‐pressure angiography of the right atrium may helpful for transseptal puncture. And for redo procedure, the PLSVC should be pay more attention as an ectopic trigger.

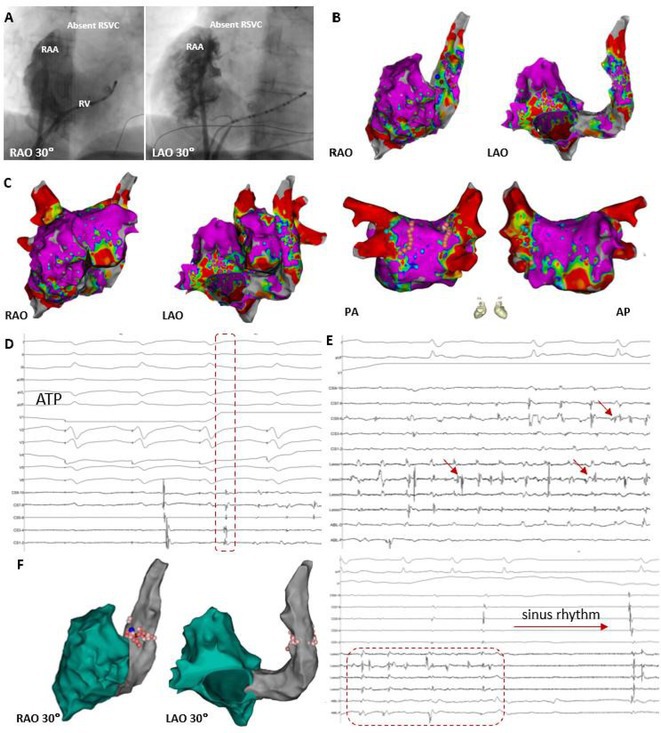

## SUCCESSFUL LEFT ATRIAL PACING WITHIN CORONARY SINUS NEAR THE OSTIUM OF MARSHALL VEIN USING A SELECTSECURE LEAD IN A CASE WITH FAILED CONVENTIONAL RIGHT ATRIAL PACING

### **JUNICHI DOI**, KAZUTO HAYASAKA, SHU YAMASHITA, TAKESHI SASAKI

#### National Hospital Organization Disaster Medical Center, Tokyo, Japan

**Introduction:** In cases with failed conventional right atrial (RA) pacing in RA appendage or septum due to anatomical and pathophysiological changes in RA myocardium for some reasons, an alternative atrial pacing site will be required. However, atrial pacing on the left atrial (LA) side is still challenging. We report a case that atrial pacing was successfully performed within the coronary sinus (CS) near the ostium of Marshall vein (VOM).

**Methods:** N/A

**Results:** A 72‐year‐old male with a dual chamber pacemaker for sick sinus syndrome and a previous history of catheter ablation for atrial tachycardia was admitted due to infectious endocarditis caused by device infection. He presented with ventricular fibrillation on QT prolongation, which required an upgrade to implantable cardioverter defibrillator (ICD) after the removal of a whole pacemaker system for device infection. ICD was implanted a month after the device extraction. A SelectSecure atrial lead (3830, Medtronic) was managed to be placed in RA septum due to lower sensing and higher pacing thresholds that were caused by severe atrial remodeling, resulting in its dislodgement the next day after implantation. In the redo‐procedure, atrial lead was successfully delivered and placed within CS near the ostium of VOM using 7Fr subselection catheter for CRT device implantation (C315, Medtronic). Atrial pacing at this site showed normal pacing parameters (sensing: 0.9mV, pacing threshold: 0.5 V/0.4ms, lead impedance: 450Ω) and similar p wave morphologies to those by lower septal pacing, and also significantly shortened PR interval compared with RA septal pacing.

**Conclusions:** Left atrial pacing within the CS or its branches, such as VOM, may be an alternative atrial pacing site in cases with failed conventional RA pacing. Further study will be required to investigate its safety and efficacy.

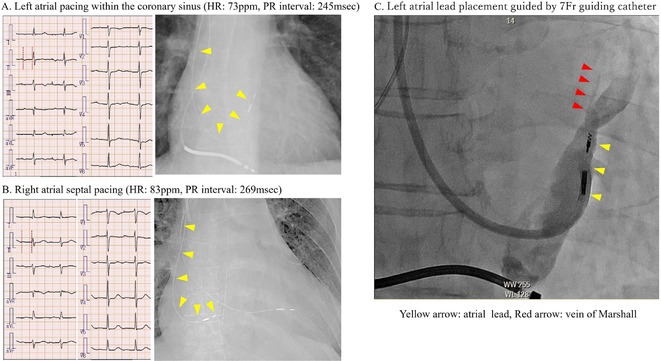

## LEFT ATRIAL APPENDAGE OCCLUSION WITH AMULETDEVICE AFTER FAILED WATCHMAN IMPLANTATION

### **RAHUL DOSHI**, AMIER AHMAD, YOAAV KRAUTHAMMER, MARK SEIFERT

#### HonorHealth, Scottsdale, AZ

**Introduction:** Both clinically approved devices for left atrial appendage (LAA) occlusion are safe and efficacious for mitigation of thromboembolism in patients with atrial fibrillation. The Amulet device requires less depth for implant compared to the Watchman device. To be enrolled in the Amulet IDE trial patients had to be deemed suitable for either implant. We sought to evaluate the effectiveness of this device in patients with failed Watchman implants.

**Methods:** Our initial 50 consecutive patients commercially implanted with the Amulet were retrospectively evaluated. Reasons for failure and implant characteristics were examined including parameters on TEE visualization at time of implant.

**Results:** All 50 patients were successively implanted acutely with average deployment time 10.3+/‐2.3 min. 72.4% of patients were screen fails on imaging (CT or TEE) with the remaining 27.6% as failed implant attempts. One screen fail was for persistent thrombus with inadequate depth to land device proximally. The average implant depth required was 15.7+/‐5.1mm. The most common LAA morphology was chicken wing (37.9% anterior and 34.5% posterior). Maximal ostial measurements were 26.5+/‐3.6mm and the maximal landing zone diameter was 19.0+/‐3.3mm. The majority of devices implanted where of median size (20‐25 mm device in 35/50 (70%) with only a small percentage with either the smallest (16 mm n=3) or the biggest device (34 mm n=1). There were no intraprocedural complications, but one patient developed a late pericardial effusion not requiring intervention. On 45‐90 day follow‐up, 3/50 (6%) were seen to have an edge leak on follow‐up imaging.

**Conclusions:** Initial Amulet implantation was highly successful in failed Watchman device implantation for LAA occlusion. These procedures were driven largely by implant depth as compared to ostial dimensions. Whether this population results in a higher incidence of edge leaks needs validation in prospective clinical trials.

## ABNORMALITIES IN CARDIOPULMONARY EXERCISE TEST ASSOCIATED WITH MORE ADVANCE ATRIAL CARDIOMYOPATHY

### **JENELLE DZIANO**, JACKSON HOWIE, JONATHAN ARIYARATNAM, MOHAMED ABBAS, ELNAZ SHAHMOHAMADI, MEHRDAD EMAMI, SHAUN EVANS, MELISSA MIDDELDORP, PRASHANTHAN SANDERS, ADRIAN ELLIOTT

#### University of Adelaide, Adelaide, Australia

**Introduction:** Cardiopulmonary Exercise Testing (CPET) provides prognostic and diagnostic value in patients with heart failure. However, the use of CPET to evaluate the extent of atrial cardiomyopathy in symptomatic AF patients has not been examined. This study sought to evaluate the association between the number of pulmonary gas exchange abnormalities during CPET and measures of LA function amongst patients with symptomatic AF.

**Methods:** Patients with symptomatic paroxysmal or persistent AF, undergoing ablation were assessed. Participants completed maximal CPET on a cycle ergometer. Oxygen consumption (VO_2peak_), ventilatory efficiency (V_E_/VCO_2_ slope) and partial pressure of end‐tidal CO_2_ (PETCO_2_) were stratified according to international criteria and patients trichotomized according to number of CPET abnormalities (0, 1 or ≥2). LA strain and LA minimum volume were assessed by resting echocardiography. LA pressure was measured invasively during ablation and monitored during continuous saline infusion into the LA (15mL/kg over 8 minutes). LA diameter was assessed by transoesophageal echocardiography throughout the saline infusion. LA Compliance was calculated as ∆LA Diameter/∆Peak LA Pressure.

**Results:** 125 participants were recruited (mean age 63±11, 72% male). Patients with more CPET abnormalities had higher LA minimum volume at rest (p=0.025), lower LA reservoir strain (p=0.001) and higher NT‐proBNP (p=0.058). In those with more CPET abnormalities, LA compliance was lower (p=0.009) and LA pressure at rest was similar (p=0.14), but significantly higher during fluid loading (p=0.05).

**Conclusions:** Abnormalities detected in CPET may provide a useful non‐invasive tool to identify patients with more advanced atrial cardiomyopathy in patients with symptomatic AF.

## EXERCISE INTOLERANCE ON CARDIOPULMONARY EXERCISE TESTING AS A KEY INDICATOR OF ATRIAL FIBRILLATION SYMPTOM BURDEN

### **JENELLE DZIANO**, JACKSON HOWIE, JONATHAN ARIYARATNAM, MOHAMED ABBAS, ELNAZ SHAHMOHAMADI, MEHRDAD EMAMI, SHAUN EVANS, MELISSA MIDDELDORP, PRASHANTHAN SANDERS, ADRIAN ELLIOTT

#### University of Adelaide, Adelaide, Australia

**Introduction:** The presence and intensity of symptoms in atrial fibrillation (AF) influences clinical decision making. However, symptoms can be non‐specific and confounded by other existing cardiovascular conditions making symptom assessment challenging.

**Methods:** We prospectively recruited patients scheduled for AF catheter ablation. Prior to procedure, participants completed a maximal cardiopulmonary exercise test (CPET) on a cycle ergometer to determine peak oxygen consumption (VO2peak). Percent of predicted oxygen consumption (%VO2peak) was calculated as VO2peak divided by validated normative values based on age, height, weight and gender. AF symptoms were assessed using a 7‐question symptom checklist (AFSS). The severity of each symptom (palpitations, dyspnoea at rest, dyspnoea during exercise, exercise intolerance, fatigue, light‐headedness and chest pain) was scored on a 0‐5‐point scale. Patients were trichotomized based on overall symptom score into low burden (0‐6, with no more than 2 in any one symptom), moderate burden (7‐12) and high burden (>12). Individual symptom scores were also trichotomized into low (0‐1), moderate (2‐3) and high (4‐5) burden.

**Results:** 126 patients (mean age 65 ± 11 years, 20% female) were included in the analysis. Among them, 36 reported low, 43 moderate, and 47 high symptom burdens, with mean age not statistically different between groups. VO2peak was lower in patients with the highest symptom burden (24.3 in low vs. 21.7 in moderate, vs.18.1mL/min/kg in high symptom group)(p<0.001). These findings remained the same when percentage of predicted VO2peak achieved were used. Significantly lower VO2peak was also demonstrated amongst patients with higher individual symptom scores for dyspnea at rest (p<0.001), dyspnea during exercise (p<0.001), exercise intolerance (p<0.001), fatigue (p=0.047), light headedness (p=0.037), and chest pain (p<0.001).

**Conclusions:** These findings suggest that reduced exercise tolerance on cardiopulmonary exercise testing is associated with higher AF symptom burden and may be a useful tool to quantify response to therapy.

## IDENTIFICATION OF NOVEL PREDICTORS OF HEART FAILURE WITH PRESERVED EJECTION FRACTION IN PATIENTS WITH ATRIAL FIBRILLATION

### **JENELLE DZIANO**, JACKSON HOWIE, JONATHAN ARIYARATNAM, MOHAMED ABBAS, ELNAZ SHAHMOHAMADI, MEHRDAD EMAMI, SHAUN EVANS, MELISSA MIDDELDORP, PRASHANTHAN SANDERS, ADRIAN ELLIOTT

#### University of Adelaide, Adelaide, Australia

**Introduction:** Currently there are two commonly used scoring systems to determine probability of HFpEF in the absence of invasive testing. However, previous analysis in patients with atrial fibrillation (AF) suggests that these scoring systems may have limited utility in this population. The purpose of this study is to assess for the presence of other important predictors of HFpEF in patients with AF.

**Methods:** Patients with symptomatic paroxysmal or persistent AF, undergoing ablation were assessed. Participants completed maximal cardiopulmonary exercise test on a cycle ergometer to determine oxygen consumption (VO2_peak_), ventilatory efficiency (VE/VCO2 _slope_) and peak partial pressure of end‐tidal CO2 (PETCO^2^_peak_). Resting echocardiography was conducted to measure LA strain, indexed LA minimum volume (LAVi) and a non‐invasive estimation of left ventricular filling pressure (E/e’). HFpEF status was determined by invasive LA pressure measurement following 500mL fluid infusion directly into the left atrium. HFpEF was defined as pressure>15mmHg following fluid challenge.

**Results:** 125 patients (mean age of 65 ± 11 years, 26% female) were included for analysis. Comparison between patients with HFpEF (n= 91, 75.8%) and those without (n= 29, 24.2%) revealed significant differences in several key parameters. Individuals without HFpEF exhibited a lower mean age (60 ± 11 years vs. 65 ± 11 years, p = 0.042) and lower mean BMI (27.3 ± 4.7 kg/m^2^ vs. 29.3 ± 4.7 kg/m^2^, p = 0.039). Additionally, the no HFpEF group demonstrated lower mean E/e' values (7.9 ± 2.2 vs. 9.9 ± 3.4, p = 0.000) and lower mean NT‐proBNP levels (175 ± 152 ng/L vs. 513 ± 588 ng/L, p = 0.000). Conversely, the HFpEF group had a lower mean VO2 (19.6 ± 6.8 mL/min/kg vs. 25.8 ± 7. mL/min/kg, p = 0.000), as well as lower LA strain (18.8 ± 8% vs. 26.5 ± 10.6%, p= 0.001). However, no statistical differences were observed between groups for LAVi (p = 0.105), PETCO2peak (p = 0.466), or VE/VCO2 slope (p = 0.864).

**Conclusions:** These findings suggest that adding variables such as LA strain and VO2peak to HFpEF scoring algorithms, could improve non‐invasive diagnostic detection of HFpEF in patients with AF.

## THE PREVALENCE OF ATRIAL FIBRILLATION ASSOCIATED WITH CONGESTIVE HEART FAILURE IN SINGLE HEART CENTER

### PONH REASEY EA

#### Khmer‐soviet friendship hospital, Phnom Penh, Cambodia

**Introduction:** The most prevalent arrhythmia in congestive heart failure (HF) is atrial fibrillation (AF), which is associated with a worse prognosis. AF increases HF symptoms and increases in prevalence with increasing New York Heart Association class. Although the exact cause of the two conditions is unknown, the coexistence of valvular, ischemic, and nonischemic structural heart disease, as well as common risk factors like age, hypertension, diabetes, and obesity, can help to explain why they exist.

**Methods:** It is a retrospective study of atrial fibrillation among 271 cases diagnosis with congestive heart failure based on clinical aspects and criteria of echocardiography (the average age of the population is 60 years with an extreme ranging from 24 years to 90 years), male 51% from January 2024 to March 2024 at Khmer‐soviet friendship hospital.

**Results:** It was recognized over a century ago that AF and heart failure were related; the reported prevalence of AF in our investigation of the prevalence of heart failure over three months at heart center, Khmer‐soviet friendship hospital, 271 patients experienced either new AF or heart failure. Out of these subjects, 72 people (or 26%) experienced both heart failure and AF which 52.8% of the patients were found to be male and 47.2% to be female, several significant risk factors (age, hypertension, coronary heart disease, and rheumatic heart disease) were identified for AF in the general population. The age group older than sixty is the most affected (Figure 1). Moreover, 66.7% of our case have preserved left ventricle ejection fraction (Figure 2).

**Conclusions:** Due to short duration and slight of sample, this study conducted on a small sample size demonstrates that AF prevalence in our heart center affect non different between male and female (52.8% in male and 47.2% in female) who having many of cardiovascular risk factors. Additionally, the prevalence of AF are predicted in the future with the increasing age of the population. Further prospective randomized studies are needed to corroborate these finding.

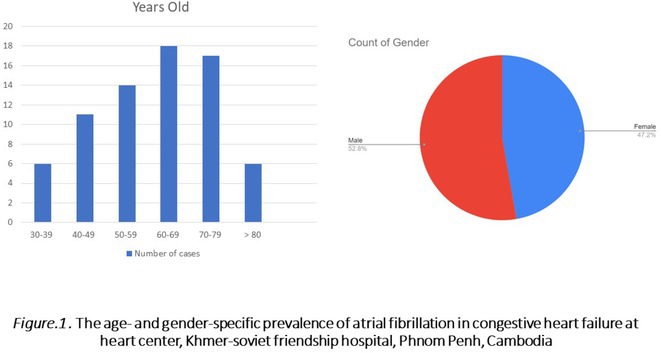

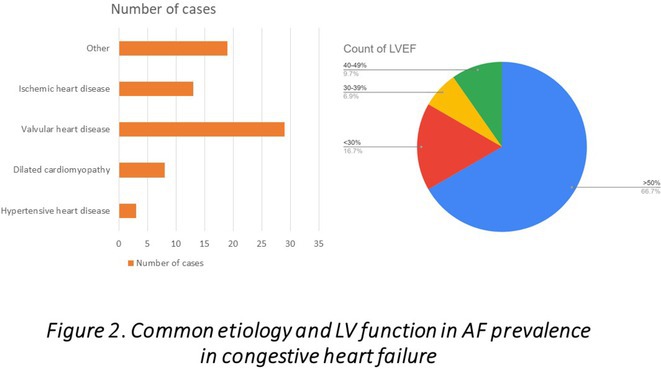

## RISK ASSESSMENT OF CARDIOGENIC CEREBRAL EMBOLISM IN PATIENTS WITH ATRIAL FIBRILLATION USING GENOMIC INFORMATION

### **YUSUKE EBANA**, TETSUO SASANO, TETSUSHI FURUKAWA

#### Tokyo Medical and Dental University, Tokyo, Japan

**Introduction:** Stroke is a leading cause of death and the primary cause of adult‐acquired disability. Patients with cardiogenic embolic stroke also have higher mortality and recurrence rates than patients with other stroke subtypes. Atrial fibrillation (AF) is a major risk factor for cerebral infarction (CI). Although the large‐scale study identified several loci, few stroke risk assessments in AF cases have been sparse. Our overall aim was to assess stroke risk with genetic information and clinical data.

**Methods:** We performed association study with CI using 8181 AF cases in previous genome‐wide association study (GWAS) and imputation data without controls. We classified AF cases into those with or without past history of CI, and the genetic associations with the CI risk were examined.

**Results:** GWAS identified several associated loci. We generated a genetic risk score (GRS) for these loci that was significantly associated with CI in patients with AF (1.46 × 10^‐8^). We estimated a bivariate logistic regression model which contained GRS and CHADS_2_ score (GRS: p‐Value 7.41 × 10^‐9^, CHADS_2_ score: p‐Value 2.0 × 10^‐16^) or CHA2DS2‐VASc scores (GRS: p‐Value 2.52 × 10^‐10^, CHA_2_DS_2_‐VASc score: p‐Value 2.0 × 10^‐16^).

**Conclusions:** We identified several genetic variants that were potentially associated with the risk of CI of AF cases and the significant GRS, whose associations were independent of the CHADS_2_ or CHA_2_DS_2_‐VASc score.

## TRANSEPTAL ACCESS USING FLEXCATH CROSS COMPARED WITH A STANDARD APPROACH FOR CRYOBALLOON PULMONARY VEIN ISOLATION

### **HEATHER EDWARDS** ^1^, ALASDAIR HAWLEY^1^, GERASIMOS DIMITRPOULOS^1^, STEVEN PODD^1^, WAQAS ULLAH^2^, KIM RAJAPPAN^3^, MATTHEW LOVELL^1^

#### ^1^Royal Devon and Exeter nhs trust, Exeter, United Kingdom,^2^University Hospital Southampton NHS Foundation Trust, Southampton, United Kingdom,^3^Oxford University Hospitals NHS Trust, Oxford, United Kingdom

**Introduction:** CryoPVI (cryoballoon pulmonary vein isolation) with a standard transseptal puncture technique requires a sheath exchange over a wire in the LA. This takes time, risks loss of transseptal access and the potential for air embolism. The FlexCath Cross system offers a simplified workflow for transseptal puncture without the need for sheath exchange.

**Methods:** This was a registry of transseptal punctures for cryoPVI. Patients with paroxysmal, persistent and longstanding persistent AF were included. Procedures were performed over 3 sites under sedation or general anaesthesia. Standard transseptal technique with BRK‐1 and SL sheath were compared with direct access with FlexCross.

**Results:** 100 patients were included in this study, 50 (50%) in the standard procedure group and 50 (50%) in the FlexCross group. There were no differences in baseline characteristics. BMI 27.1kg/m^2^ (24.2‐29.9), LAVI 37ml/m^2^ (29‐45) in standard group compared with 28.3 kg/m^2^ (25.4‐29.9) and 33ml/m^2^ (26‐41) in the FlexCross group. In the standard group 24 (48%) patients had paroxsysmal atrial fibrillation (PAF) compared with 22 (44%) in the FlexCross group. All transseptal punctures were successful. The time from local anaesthetic to FlexCath in the LA was significantly shorter in the FlexCross group 12 (9‐14) minutes compared with standard therapy 15 (12‐18) minutes p= <0.001. The overall procedure length was shorter in the FlexCross 53 (43‐61) minutes compared with standard therapy 55 (46‐63) minutes but this was not significant p=0.2. There were 2 (4%) transient phrenic nerve palsies that recovered before leaving the lab and 1 (2%) case of vasovagal syncope in the FlexCross group. There were no complications in the standard group.

**Conclusions:** Transseptal puncture using FlexCath Cross for cryoPVI is a simplified approach which shortens the time from local anaesthetic to FlexCath in the LA when compared to the standard workflow. By simplifying the transseptal approach we can shorten the overall time for ablation procedures and increase the number of cases performed in the cath lab.

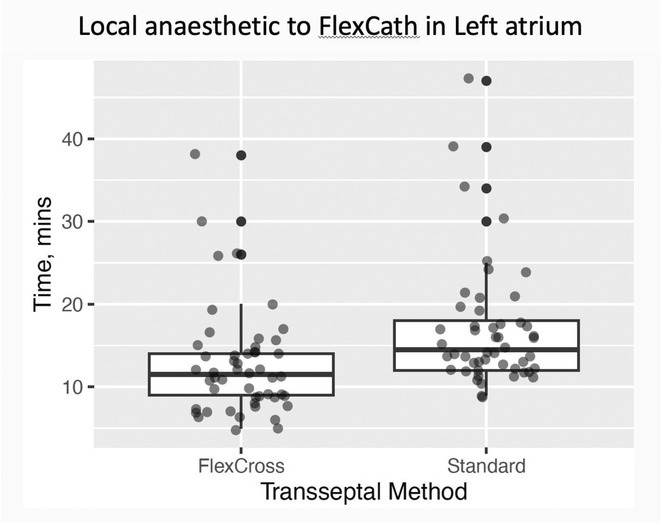

## CATHETER ABLATION OF EPICARDIAL ACCESSORY PATHWAY: A SINGLE‐CENTER CASE SERIES

### **RIFQI RIZKANI ERI**, HAIKAL BALWEEL, AGUS HARSOYO, NOVARO ADENEUR TAFRIEND

#### Gatot Soebroto Army Hospital, Central Jakarta, Indonesia

**Introduction:** Epicardial accessory pathways (EAP) can be ablated by various approaches, including via the coronary sinus, aortic cusp or endocardially. Most EAP cases are commonly treated through catheter ablation via subxyphoid access, which allows direct access to the epicardium. Here, we present three successful cases of EAP ablation without subxyphoid access.

**Methods:** N/A

**Results:** We present three patients who underwent catheter ablation for epicardial accessory pathways. The first patient, a 19‐year‐old male, had a history of atrioventricular reciprocating tachycardia (AVRT). During the procedure, the accessory pathway (AP) was identified in right anteroseptal/nodo‐Hisian region, and ablation was successfully performed from the right atrial appendage (RAA) area. The second patient, a 39‐year‐old female, had a history of AVRT due to a concealed left lateral accessory pathway. The coronary sinus catheter showed AP potential, and ablation was successfully performed via the coronary sinus approach. The third patient, a 21‐year‐old male who was about to enter Military School, had manifest pre‐excitation from a nodo‐Hisian pathway. The AP was successfully ablated from right coronary cusp (RCC).

**Conclusions:** This case series highlights the use of radio‐frequency ablation (RFA) for the treatment of epicardial accessory pathways. In some cases, EAP ablation can be safely performed using transvenous or transaortic approaches. Our cases demonstrate that it is possible to perform RFA for epicardial AP through the right atrial appendage, coronary sinus or aortic cusp rather than subxyphoid approach.

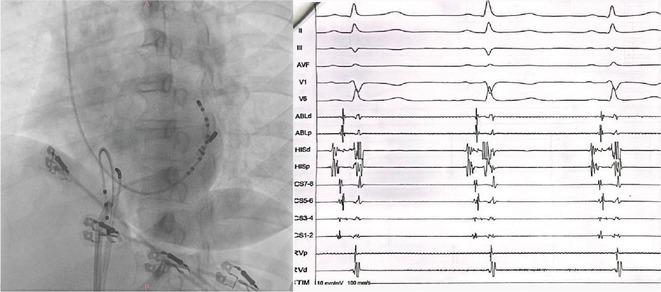

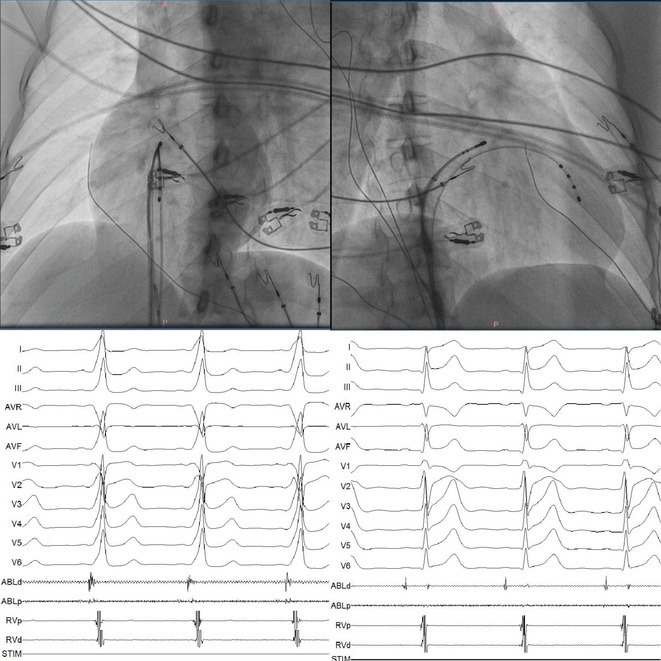

## ARTIFICIAL INTELLIGENCE (AI) ECG‐PREDICTED BIOLOGICAL AGE GAP AND MORTALITY: CAPTURING DYNAMIC RISK WITH MULTIPLE ECGS

### **SHAUN EVANS** ^1^, SARAH HOWSON^1^, ANDREW E C BOOTH^1^, MATTHEW LIM^1^, STEPHEN BACCHI^1^, MOHANARAJ JAYAKUMAR^1^, SURAYA KAMSANI^1^, JOHN FITZGERALD^1^, ANAND THIYAGARAJAH^1^, MELISSA MIDDELDORP^2^, PETER PSALTIS^1^, PRASHANTHAN SANDERS^1^

#### ^1^Royal Adelaide Hospital, Adelaide, Australia,^2^University of Adelaide, Adelaide, Australia

**Introduction:** AI‐predicted ECG biological age is a predictor of mortality. ECGs are inexpensive and readily available compared with other markers of biological age, which makes them attractive as a lifestyle counselling aid. Previously, the age gap between biological age and chronological age was modelled as a fixed hazard. The effect of using multiple ECGs to estimate a time‐varying hazard has not been explored.

**Methods:** Using AI‐ECG trained to predict age, biological age was estimated to calculate a biological age gap. Patients included were 20‐90 years old and had at least two ECGs recorded in hospital. Two Cox models were created: one using a single ECG per patient, and the other using multiple ECGs. Survival estimates were compared using the C‐index to explore the difference in model discrimination. The multiple‐ECG model was reviewed for accuracy by number of ECGs per patient.

**Results:** 46,960 patients with 337,415 ECGs were included. The median follow‐up was 4.5 years with 6622 patient deaths. Both models estimated that every year increase in age gap increased the mortality hazard by 1% (coeff.=0.01, P<0.005). The multiple‐ECG model provided a superior fit for mortality compared with a single ECG with a higher log‐likelihood ratio test (6098 vs 5136, higher is better). The survival discrimination as measured by the C‐index increased from 0.59 to 0.67 with the multiple‐ECG model. In the multiple‐ECG model, segmenting estimates by the number of ECGs undertaken showed that the C‐index increases from 0.60 with 2 ECGs performed and plateaus at 0.74 for patients with at least 5 ECGs undertaken.

**Conclusions:** The AI ECG‐derived age gap is a significant predictor of mortality, and this is further improved by using multiple ECGs. These results suggest that mortality estimates are significantly enhanced by incorporating the dynamic nature of risk conferred by biological age

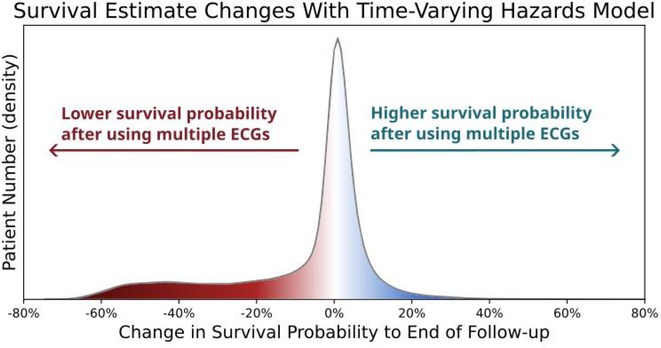

## ARTIFICIAL INTELLIGENCE AGE PREDICTION USING ELECTROCARDIOGRAM DATA: EXPLORING BIOLOGICAL AGE DIFFERENCES

### **SHAUN EVANS** ^1^, SARAH HOWSON^1^, ANDREW E C BOOTH^1^, ELNAZ SHAHMOHADI^2^, MATTHEW LIM^1^, STEPHEN BACCHI^3^, ROSS ROBERTS‐THOMSON^1^, MELISSA MIDDELDORP^2^, MEHRDAD EMAMI^1^, PETER PSALTIS^1^, PRASHANTHAN SANDERS^1^

#### ^1^Royal Adelaide Hospital, Adelaide, Australia,^2^University of Adelaide, Adelaide, Australia,^3^Lyell McEwin Hospital, Adelaide, Australia

**Introduction:** Biological age can be predicted using artificial intelligence (AI) trained on electrocardiograms (ECGs), which is prognostic for mortality and cardiovascular events. To date, there has been limited validation of these models using external datasets.

**Methods:** An AI model was trained on all ECGs from cardiology patients aged 20‐90 years at a single tertiary hospital. External validation was undertaken using data from the UK Biobank. Subgroups were compared using the difference between participants’ biological and chronological ages.

**Results:** 63,246 patients with 353,704 total ECGs were included (mean 5.6 per patient). In internal validation, our model achieved a correlation coefficient of 0.72 between predicted (biological) age and chronological age. With an unadjusted model and ECG processing algorithm, similar performance was seen in the UK Biobank external validation population. In patients aged 20‐29, AI‐ECG biological age was older than chronological age by a mean 13.9±0.2 yrs. In patients aged 80‐89 years, biological age was younger by a mean 11.2±0.1 yrs. Women were biologically younger than men by a mean of 7 months (P=0.02) and patients with a single ECG were biologically 2.6 years younger than those with multiple ECGs recorded at the hospital (P<0.0001).

**Conclusions:** The results support earlier findings that AI can predict age based on ECG. There are significant between‐group differences in AI‐ECG biological age for hospitalised cardiology patients. The youngest hospitalized patients were biologically older than chronological age, and geriatric patients were biologically younger. Women were biologically younger than men. Patients with a single ECG recorded were younger than those with multiple ECGs recorded, which we hypothesize reflects an association between accelerated biological ageing and cumulative time spent in hospital. Further work is required to examine the potential clinical utility in patient care and risk stratification.

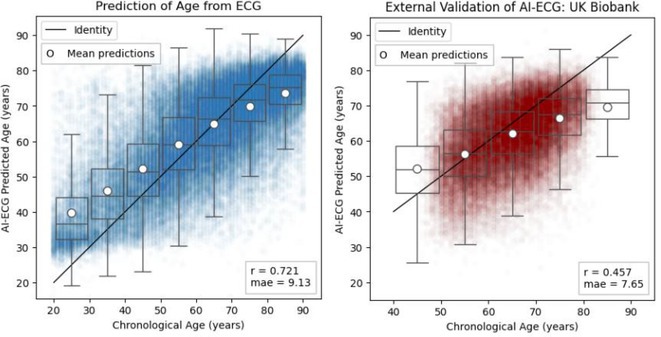

## ARTIFICIAL INTELLIGENCE ELECTROCARDIOGRAM PREDICTION OF LEFT ATRIAL DILATION: INFLUENCES OF FORM AND FUNCTION

### **SHAUN EVANS** ^1^, ANDREW E C BOOTH^1^, SARAH HOWSON^1^, MATTHEW LIM^1^, STEPHEN BACCHI^2^, ANAND THIYAGARAJAH^1^, JOHN FITZGERALD^1^, MOHANARAJ JAYAKUMAR^1^, MOHAMED ABBAS^1^, SURAYA KAMSANI^1^, MELISSA MIDDELDORP^3^, MEHRDAD EMAMI^1^, PETER PSALTIS^1^, PRASHANTHAN SANDERS^1^

#### ^1^Royal Adelaide Hospital, Adelaide, Australia,^2^Lyell McEwin Hospital, Adelaide, Australia,^3^University of Adelaide, Adelaide, Australia

**Introduction:** Left atrial (LA) size and function is hypothesised to be closely related to the pathophysiology of embolic strokes with uncertain source (ESUS). However, the diagnosis of LA myopathy remains histopathological, with no robust non‐invasive criteria. Despite observational data that LA diameter might predict recurrent stroke after ESUS, no measures or biomarkers have translated to clinical benefit in randomised controlled trials. Artificial intelligence using an ECG (AI‐ECG) may provide a novel screening strategy.

**Methods:** A single‐centre, retrospective cohort of ECG‐echocardiogram pairs was used to train an AI algorithm to predict LA volume index (LAVi) from a 12‐lead ECG. The AI algorithm was a convolutional neural network trained to estimate the probability of LAVi > 34mL/m2. The resulting model was then evaluated on a cohort of patients with atrial fibrillation (AF) prior to planned AF ablation: these patients underwent comprehensive left atrial assessment and these baseline variables were examined to identify underlying determinants of AI predictions.

**Results:** 43,022 individuals with a total of 207,147 ECGs had a corresponding echocardiogram reporting LAVi. The median LAVi was 38 mL/m2 (interquartile range 29‐49 mL/m2). The ECG‐based convolutional neural network C‐statistic was 0.77 to predict LA dilation. A cohort of 52 patients planned for AF ablation were included with accompanying comprehensive LA assessment. In this group, in addition to LAVi itself, LA reservoir strain and LA ejection fraction were significant predictors of AI‐assessed LA dilation in a multivariate model: β* coefficients were 0.104, ‐0.085, and 0.064 respectively.

**Conclusions:** AI‐ECG can successfully predict LA volume. This prediction is sensitive not only to the true LA volume index, but also to functional impairment of the LA as measured by LA ejection fraction and LA reservoir strain. This suggests that the AI‐ECG estimate may be a biomarker of LA function. To better characterise LA myopathy, ECG‐based AI warrants further characterisation, and examination into correlation with relevant clinical outcomes, particularly embolic stroke.

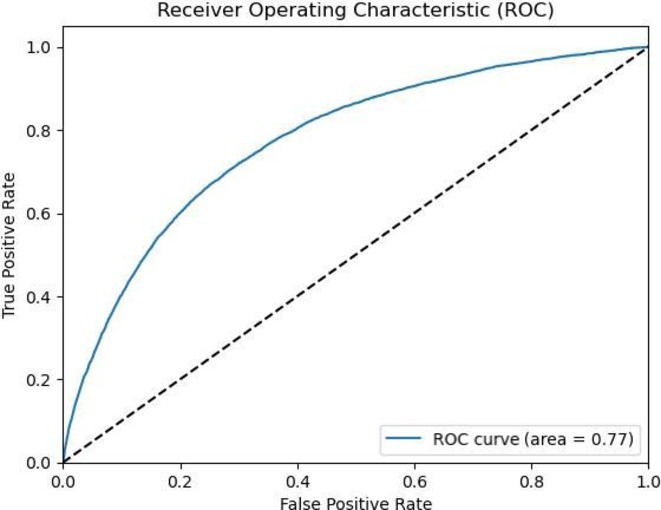

## QUALITY‐OF‐LIFE OUTCOMES FOR A DUAL‐CHAMBER LEADLESS PACEMAKER SYSTEM

### **DEREK EXNER** ^1^, RAHUL DOSHI^2^, JAMES IP^3^, PASCAL DEFAYE^4^, VIVEK REDDY^5^, ROBERT CANBY^6^, MORIO SHODA^7^, MARIA GRAZIA BONGIORNI^8^, GERHARD HINDRICKS^9^, PETR NEUZIL^10^, THOMAS CALLAHAN^11^, NICOLE HARBERT^12^, STEPHANIE DELGADO^13^, ANU BULUSU^14^, REINOUD KNOPS^15^

#### ^1^University of Calgary, Calgary, AB, Canada,^2^Honor Health Cardiac Arrhythmia Group, Scottsdale, AZ,^3^Weill Cornell Medicine/New York Presbyterian Hospital, New York, NY,^4^CHRU Albert Michallon, Grenoble, France,^5^Icahn School of Medicine at Mount Sinai, New York, NY,^6^Texas Cardiac Arrhythmia Institute, Austin, TX,^7^Tokyo Women's Medical University, Tokyo, Japan,^8^San Rossore Private Hospital and Medical Center, Pisa, Italy,^9^German Heart Center of the Charite, Berlin, Germany,^10^Na Homolce Hospital, Prague, Czech Republic,^11^Cleveland Clinic Foundation, Cleveland, OH,^12^Abbott Medical, Plano, TX,^13^Abbott Medical, Sylmar, CA,^14^Abbott Medical, Santa Clara, CA,^15^Amsterdam UMC, Amsterdam, Netherlands

**Introduction:** The safety and electrical performance of the Aveir™ DR dual‐chamber leadless pacemaker (DR‐LP) (Abbott Medical) has been demonstrated, but the health‐related quality of life (HR‐QoL) outcomes of this technology are not well understood. This analysis summarizes the temporal HR‐QoL outcomes in the Aveir DR global clinical trial.

**Methods:** Subjects completed a general HR‐QoL instrument (EQ‐5D‐5L) at pre‐implant (enrollment); those with a successful Aveir DR‐LP implant completed EQ‐5D‐5L at 1, 3, 6, and 12‐months post‐implant. The EQ‐5D‐5L assesses five health dimensions and includes a visual analog scale (EQ‐VAS) which spans from 0 (“worst”) to 100 (“best”) imaginable health state. The primary analysis was the change in HR‐QoL over the initial 6 months post‐implant and was assessed using the McNemar and Wilcoxon tests.

**Results:** A total of 442 patients (97.8 % of implant attempts) underwent successful implantation of an Aveir DR‐LP system. Of these, HR‐QoL data were available for 440 subjects at enrollment (62% male, 69.6 ± 13.3 years of age, 65% with sinus node disease [SND], and 31% with AV block) and for 433 subjects at 1 month (98.4%), 431 at 3 months (98.0%), 420 at 6 months (95.5%) and 301 at 12 months (68.4%). Due to a truncated 12‐month follow‐up in the overall study, HR‐QoL analyses were limited to the initial 6 months. Absolute changes in the proportions of patients reporting a problem for at least one dimension in EQ‐5D‐5L and change in VAS scores over time are shown in Figure 1 for subjects with paired data at enrollment and the respective timepoint. Statistically significant improvements were found in paired data comparing enrollment and each follow‐up timepoint for the EQ‐5D‐5L (Figure 1). These improvements appear larger in comparison to prior studies of patients with cardiac disease. Further, improved EQ‐VAS scores were observed at each follow‐up visit and were statistically improved over the 6 months (Figure 1).

**Conclusions:** A large improvement in HR‐QoL scores in Aveir DR‐LP recipients occurred from enrollment through 6 months post implant. The magnitude of these changes shows meaningful HR‐QOL improvements in a dual chamber leadless pacemaker population.

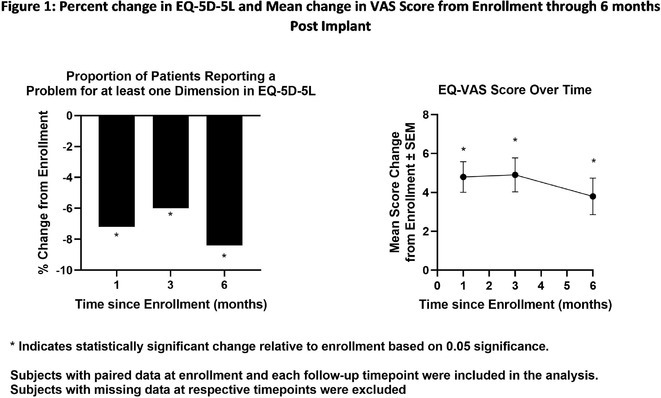

## EFFICACY AND SAFETY OF HYBRID CONVERGENT ABLATIONS IN PATIENTS WITH PERSISTENT ATRIAL FIBRILLATION FIRST‐IN ASIA CASE SERIES IN HONG KONG

### **KATHERINE FAN** ^1^, DANIEL CHAN^2^, IAN WH LING^1^, MAX KH WONG^2^, MARC YH CHENG^1^, CATHY LAM^1^

#### ^1^Grantham Hospital HK, Aberdeen, Hong Kong,^2^Queen Mary Hospital HK, Pok Fu Lam, Hong Kong

**Introduction:** Hybrid convergent procedure is a novel epicardial‐endocardial ablation approach which combined minimal invasive cardiothoracic surgical and catheter ablation techniques into a collaborative approach in managing persistent atrial fibrillation (perAF). We reported our first‐in‐Asia experiences and clinical outcomes.

**Methods:** Hybrid convergent procedures were performed in 19 consecutive patients (pts) with perAF (men 84%; mean age 63± 7.4 years). Median duration of perAF was 17.5 mth (average 35 ±39 mth). Mean EF was 57.6 ±14.7% and mean LA diameter was 4.5± 0.4 cm. 5 pts (26%) underwent endocardial pulmonary vein(PV) isolations prior but perAF remained. Epicardial LA posterior wall radiofrequency (RF) ablation (EPi‐Sense, AtriCure) was performed via subxyphoid incision while LA appendage clip‐closure and dissection of ligament of Marshall were performed via 3 ports videoscopic approach. Endocardial mapping and RF ablation (PVI, roof line, and complex fractionated atrial electrograms) are performed afterwards to complete the lesion set so that both PVs and posterior wall are electrically isolated.

**Results:** All pts underwent successful epicardial RF ablations without complications. 1 pt declined subsequent endocardial AF ablation, therefore 18 pts were included. Mean interval between 2 procedures was 1.8 ± 0.2 mth. During the 3 mths’ blanking period, AF/ AT occurred in 3 pts (17%) who underwent successful DC cardioversion to maintain SR. 17pts (95%) remained in SR at mean FU period 6.4 ± 3.4 mths. 83% pts remained anti‐arrhythmic agents and anti‐coagulants free. There was no significant difference between prior PVI ablations and de‐novo hybrid convergent procedures. 2 pts with tachycardia induced cardiomyopathy demonstrated significant improvement in EF post convergent procedures.

**Conclusions:** Longstanding perAF responded well with the hybrid convergent RF ablation approach and is safe. Selection of pts is important. This workflow represents a unique opportunity for multidisciplinary arrhythmia management in surgical‐ electrophysiological heart team collaboration.

## SUCCESSFUL ABLATION OF VENTRICULAR TACHYCARDIA IN REPAIRED TETRALOGY OF FALLOT WITH MECHANICAL PULMONARY VALVE

### **WEN‐PO FAN** ^1^, CHIEH‐MAO CHUANG^2^, PI‐CHANG LEE^2^, YU‐CHENG HSIEH^2^, CHENG‐HUNG LI^2^, YUN‐CHING FU^2^, YENN‐JIANG LIN^1^, SHIH‐LIN CHANG^1^, LI‐WEI LO^1^, YU‐FENG HU^1^, FA‐PO CHUNG^1^, CHIN‐YU LIN^1^, TING‐YUNG CHANG^1^, LING KUO^1^, CHIH‐MIN LIU^1^, SHIN‐HUEI LIU^1^, CHENG‐I WU^1^, GUAN‐YI LI^1^, YU‐SHAN HUANG^1^, MUHAMMAD RAFDI AMADIS^1^, BAI SITTI AMEERAH ASLEAH B TAGO^1^, MARIE KIRK PATRICH MARAMARA^1^, CHIAO‐CHIN LEE^1^, LO‐CHIEH LING^1^, SHIH‐ANN CHEN^2^

#### ^1^Taipei Veterans General Hospital, Taipei, Taiwan,^2^Taichung Veterans General Hospital, Taichung, Taiwan

**Introduction:** Ventricular tachycardia is the most common arrhythmia in repaired tetralogy of Fallot (TOF) and ablation is a recommended treatment. However, the interference of mechanical pulmonary valve to ablation in repaired TOF remain unknown.

**Methods:** N/A

**Results:** A 62‐year‐old female with TOF had received right ventricular outflow tract (RVOT) and ventricular septal defect (VSD) repair at the age of 20, and mechanical pulmonary valve replacement at the age of 52. Palpitation and near syncope developed since September 2023, and monomorphic VT was documented. Considering the complicated anatomy, the electrophysiological study (EPS) was guided by fluoroscopy and electroanatomic mapping system, with pre‐EPS chest CT images merged as intraprocedural references. Substrate mapping revealed low‐voltage zones over the RVOT free wall and basal ventricular septum, compatible with RVOT and VSD patch (Figure 1A). Late potentials and local abnormal ventricular activities (LAVAs) were also identified nearby. The clinical VT was induced and was hemodynamically tolerated. Activation mapping delineated the macro‐reentrant circuit circling around the pulmonary annulus (Figure 1B), and entrainment mapping identified the central isthmus was just underneath the mechanical pulmonary valve (Figure 1C). Radiofrequency ablation terminated the tachycardia after a complete linear ablation between pulmonary annulus and RVOT patch (Figure 1D). Focal ablation over areas with late potentials and LAVAs were also performed (Figure 1E). Post‐ablation EPS confirmed the non‐inducibility of the VT. The patient was symptom‐free till the present day.

**Conclusions:** The ablation strategies of VT in repaired TOF should be individualized according to the anatomy and arrhythmia substrates. VT ablation in repair TOF with a mechanical pulmonary valve is more challenging but still feasible with delicate manipulation.

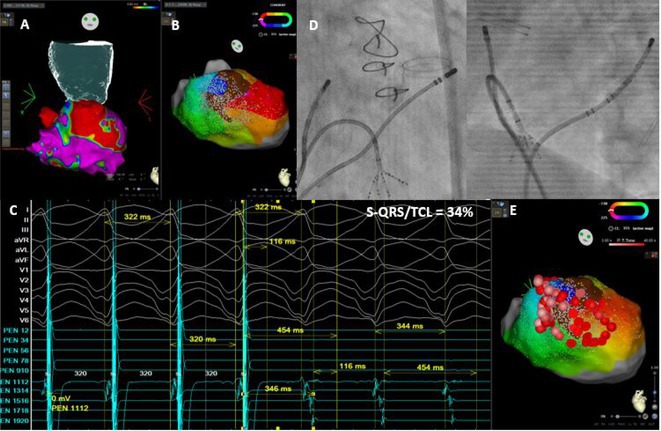

## SPACE INVADER: PAROXYSMAL ATRIAL FIBRILLATION IN ESOPHAGEAL SQUAMOUS CELL CARCINOMA WITH LEFT ATRIAL COMPRESSION

### **RYAN PAUL FERNANDEZ**, ANTHONY KING, RAMON FRANCISCO

#### Makati Medical Center, Makati, Philippines

**Introduction:** Atrial fibrillation (AF) is the most common sustained arrhythmia in adults with a global prevalence of 2‐4%. Multiple risk factors are associated with the disease and these serve as targets for therapy. However, structural and anatomic causes of the arrhythmia are not well‐established. Esophageal cancer is the seventh most common cancer, increasing by about 37% from 1990 despite having a poor prognosis. Atrial fibrillation is not a recognized as a direct clinical consequence of esophageal cancer due to external compression of the left atrium but rather as a consequence of chemotherapy or surgery.

**Methods:** N/A

**Results:** This is a case of a 51‐year‐old male diagnosed with Esophageal Squamous Cell Carcinoma Stage IIIA with first‐diagnosed atrial fibrillation. Acute episode happened during admission presenting as chest pain and palpitations with hypotension and tachycardia at a rate of 160. Synchronized cardioversion was done which converted the rhythm to sinus with noted resolution of symptoms and hypotension. Patient was subsequently given Amiodarone to resolve recurrent episodes and required higher maintenance doses to sustain sinus rhythm. Echocardiogram was done showing a normal ejection fraction and wall motion. However, it revealed an extracardiac, well‐defined, fluid‐filled structure adjacent to the left atrium, causing partial compression. Myocardial perfusion imaging was done to investigate for stress‐induced ischemia, with negative results. Findings of the echocardiogram were confirmed on cardiac MRI and chest CT scan showing a mass on the middle third of the esophagus with severe luminal narrowing compressing the left atrium without infiltration. This external compression leads to changes in the structure and architecture of the atrium causing contractile and electrophysiologic changes that could lead to the development of recurrent AF.

**Conclusions:** Mechanical compression of the left atrium causing extrinsic changes in its structure and geometry could trigger ectopic beats and can be associated with atrial fibrillation.

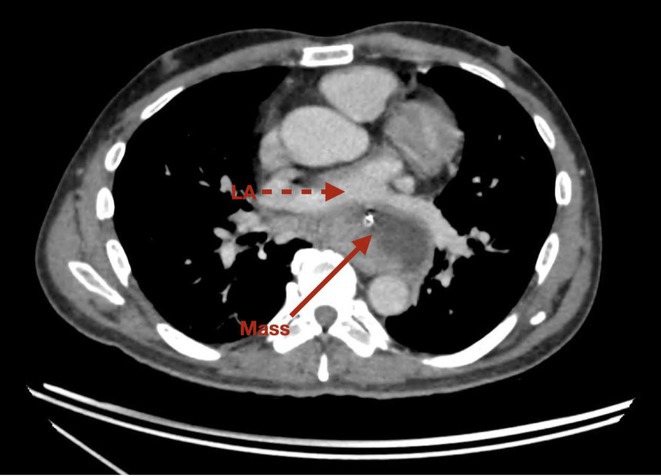

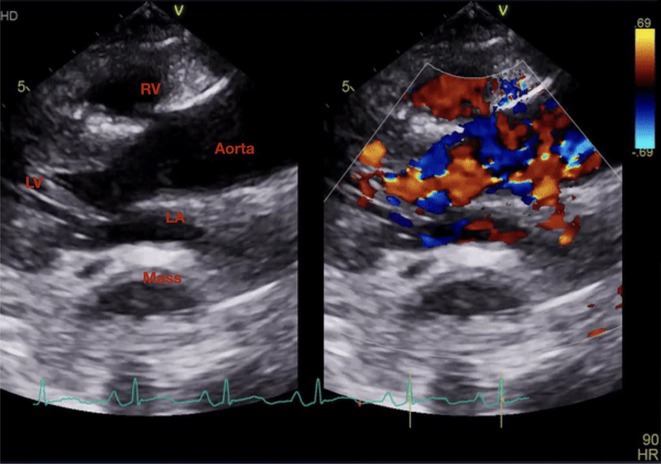

## PROGRESSION OF LEFT ATRIAL SUBSTRATE AT REPEAT LEFT ATRIAL ABLATION FOR ATRIAL FIBRILLATION

### **DAVID FERREIRA**, MICHAEL MALATY, KERISSA GOVENDER, MATTHEW FRENCH, LLOYD BUTEL‐SIMOES, AMANDEEP KAUR, GWILYM MORRIS, NICHOLAS JACKSON

#### John Hunter Hospital, Newcastle, Australia

**Introduction:** Larger left atrial (LA) size and the presence of LA low voltage areas are associated with worse outcomes following AF ablation. Patterns of low voltage and their progression over time on electroanatomic maps (EAMs) have not been carefully characterised. We aimed to assess for baseline characteristics that would predict left atrial scar progression.

**Methods:** At least 2 complete LA EAMs were created in 84 patients who had an initial pulmonary vein isolation for AF and then a repeat AF ablation. Maps were created with proximal coronary sinus pacing at 800ms with high density mapping catheters and care was taken to ensure adequate contact (prior LA imaging, signal assessment and contact force catheters where needed) and adequate point density over the complete LA. Low voltage was defined as <0.5mV and the LA was divided into 6 distinct regions for further assessment. Progression of low voltage was defined as a ≥10% relative increase in low voltage area from the index procedure.

**Results:** The average age was 65±9.5 years, 69% were male, and 74% had persistent AF. Median time between procedures was 17.6 (8.4‐38.6) months. The median number of points for an EAM was 2689 (2401‐3245). The mean change in LA volume measured by the EAM was +28.4ml/m2±77.8. Within the total cohort the mean percentage of LA low voltage was 9.0%±14.9 at index procedure and 18.4%±22.9 at redo procedure. LA low voltage was most likely to progress at the septum (in 59.4% of patients), followed by the anterior wall (54.7%) and roof (51.9%). Patients who had progression in total low voltage area at redo had a higher mean CHA2DS2‐VASc score (2.8 vs 1.8, p=0.037) but no significant difference in their left atrial volume change (+36.5 vs +27.6, p=0.44).

**Conclusions:** In this cohort of patients undergoing repeat left atrial ablation procedures for atrial fibrillation, the mean percentage low voltage area was 9.0%±14.9 at index procedure and 18.4%±22.9 at redo procedure. Higher CHA2DS2 VASc score was associated with an increase in left atrial low voltage at redo procedure but increased left atrial volume was not. This suggests that electrical remodelling can occur independently of structural remodelling of the LA.

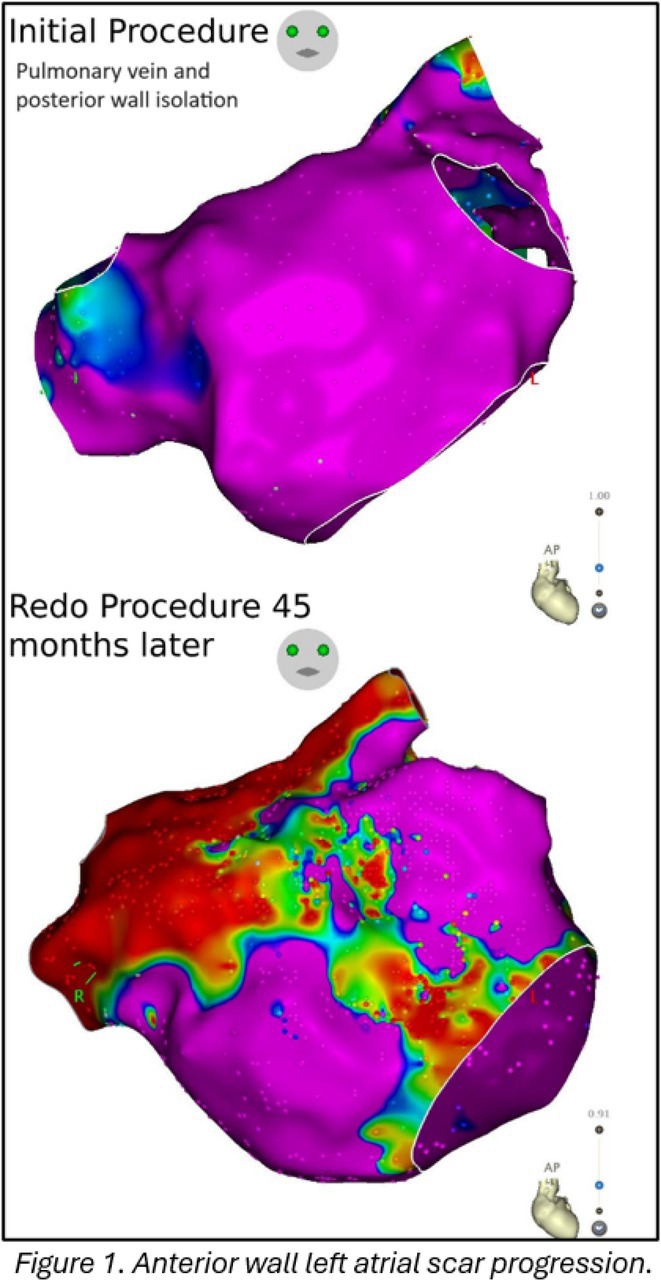

## A LOT MORE THAN MEETS THE EYE

### ROY ‐ MING CHOW LEE^1^, **JHI HUI FOO** ^2^, KENG TAT KOH^3^, LEE YEE LIM^2^, ROY‐MING CHOW LEE^2^

#### ^1^Hospital Queen Elizabeth 1, Kota Kinabalu, Sabah, Malaysia,^2^Hospital Queen Elizabeth 2, Kota Kinabalu, Sabah, Malaysia,^3^Sarawak Heart Centre, Kuching, Sarawak, Malaysia

**Introduction:** Left bundle branch‐optimised cardiac resynchronisation therapy (LOT‐CRT) offered greater electrical resynchronisation compared to conventional biventricular CRT in patients with heart failure with reduced ejection fraction (HFrEF). Upfront ventricular tachycardia (VT) ablation at the time of ICD implantation has demonstrated benefit in mortality and ICD therapy. This case illustrates LOT‐CRTD implantation and VT ablation in HFrEF with atrial fibrillation (AF), complete heart block (CHB) and incessant VT.

**Methods:** N/A

**Results:** 64‐year‐old man with non‐ischaemic dilated cardiomyopathy (LVEF < 30%, normal coronary arteries on angiogram), AF, diabetes mellitus and hypertension presented with fever, diarrhea and heart failure symptoms. His ECG showed CHB, not responding to atropine and dopamine. The scenario was complicated with stable VT which aborted with intravenous amiodarone. Temporary transvenous pacing was inserted and oral amiodarone was started as he continued to have VT while on pacing.

LOT‐CRTD was implanted with DF1/IS1 device in view of HFrEF, CHB and VT. The procedure was uneventful. However CRTD interrogation done 1 month post implantation revealed 768 episodes of non sustained VT with 146 episodes of VT appropriately treated with anti‐tachycardia pacing ICD shocks. Oral Hydroquinidine 300mg BD was started as a bridge while waiting for VT ablation. VT ablation with 3D EnSite Precision^TM^ (Abbott) successfully ablated VT1 (300 ms) over the basal septum and VT2 (400 ms) below the left coronary cusp with activation mapping and isochronal late activation mapping (ILAM). The end point of the VT ablation was non‐inducibility of VT and elimination of all deceleration zones with ILAM.

LOT‐CRTD interrogation 1 month post ablation showed no VT and ventricular fibrillation (VF) with no shocks given. He remained well with NYHA II and did not experience shocks for 2 months post ablation.

**Conclusions:** LOT‐CRTD with upfront VT ablation improved the clinical outcome in patient with HFrEF, CHB, AF and VT through electrical resynchronisation and reduction in VT burden.

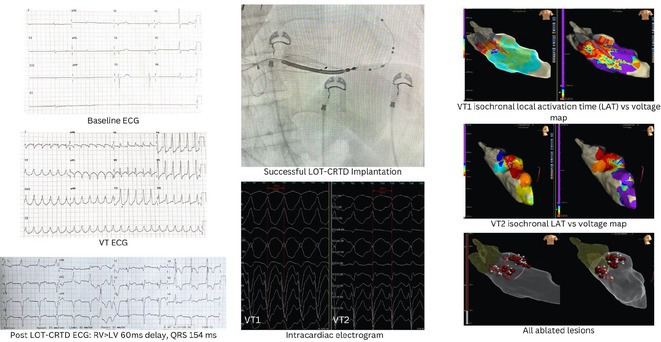

## AN ELECTROCARDIOGRAM IMAGE OF NIAGARA FALLS T WAVE IN A PATIENT WITH SUBARACHNOID HEMORRHAGE

### **LU FU**, SIJIA PU, WEIDONG LIN, YUMEI XUE

#### Guangdong Provincial People‘s Hospital, Guangzhou, China

**Introduction:** We report a case of a patient with specific giant inverted T waves after subarachnoid hemorrhage.

**Methods:** N/A

**Results:** An elderly woman with a history of hypertension was hospitalized for 17 hours after a fall with unconsciousness. Surface electrocardiogram (ECG) at admission revealed prolonged QTc, accompanied by elevated troponin levels. A head computed tomography scanning showed a subarachnoid hemorrhage, bilateral hematoma, and a suspected rupture of a vertebral artery aneurysm. An emergency therapeutic intervention and aneurysm treatment were performed to embolize and decrease the risk of rebleeding. The post‐procedural ECG showed a giant inverted T wave in the chest leads, morphologically similar to the Niagara Falls T wave. Coronary angiography or anticoagulation therapy was not performed due to the subarachnoid hemorrhage. The patient's troponin level decreased dynamically in a few days.

**Conclusions:** The characteristic ECG changes may be explained by a catecholamine storm induced by the subarachnoid hemorrhage, which stimulated the sympathetic nerve extensively and strongly and caused coronary spasms and myocardial infarction in the epicardium. The case serves as a reminder to physicians to be aware of the possibility of myocardial ischemia and potential ventricular arrhythmias following cerebrovascular accidents.

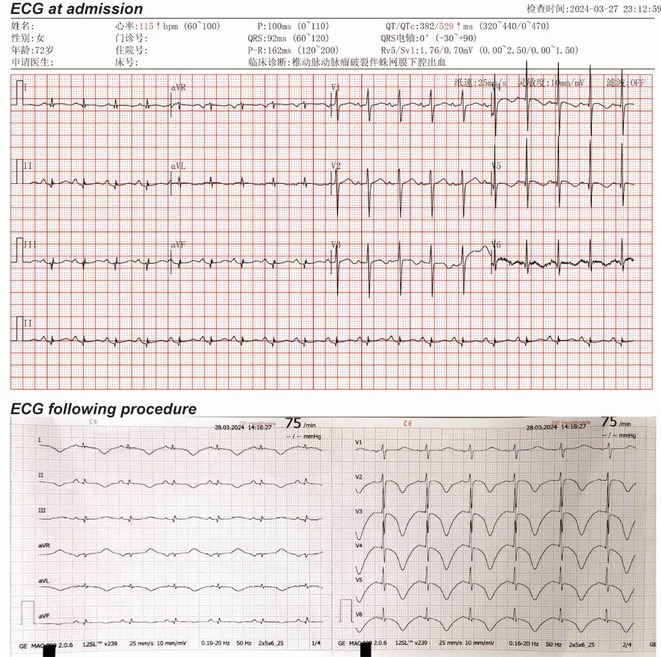

## SAFETY AND EFFICACY OF VERY HIGH‐POWER ABLATION APPROACH OF SUPERIOR VENA CAVA ISOLATION: A TWO‐CENTER STUDY

### **YUHI FUJIMOTO** ^1^, MURYO TERASAWA^2^, SHUHEI OKAJIMA^1^, MASATO HACHISUKA^1^, HIROSHIGE MURATA^1^, YOSHIYASU AIZAWA^1^, KENJI YODOGAWA^1^, YASUYUKI TAKADA^2^, KAZUHIRO SATOMI^2^, WATARU SHIMIZU^1^, KUNIYA ASAI^1^, YU‐KI IWASAKI^1^

#### ^1^Nippon Medical School, Tokyo, Japan,^2^Department of Cardiology, Tokyo Medical University Hospital, Tokyo, Japan

**Introduction:** The superior vena cava (SVC) plays an important role in non‐pulmonary vein (PV) foci to trigger atrial fibrillation (AF). A major complication in SVC isolation (SVCI) is phrenic nerve injury, and a shallower ablation depth might be less likely to cause complications. Recently, very high‐power short ablation (vHPSD) has been demonstrated to be effective in PV isolation. On the other hand, vHPSD has been reported to have shallower depth than the low‐power ablation strategies. This study aimed to examine the safety and efficacy of the vHPSD strategy in SVCI.

**Methods:** A retrospective study was conducted at two universities. Patients with AF who were undergoing first‐time SVCI were included. The first step was to perform circumferential ablation except for the sites where the phrenic nerve was captured. If the SVCI was achieved, the procedure was completed. If the isolation was inadequate, the sites of capture of the phrenic nerve were also ablated. Fifty‐nine patients who underwent the initial SVCI were evaluated. We analyzed two groups: those performing vHPSD (n=38, vHPSD group) with a power of 90 watts for 3 seconds or those using lower‐power (n=21, LP group) limited to 20‐30 watts. We analyzed all 497 in the vHPSD and 370 points in the LP group with ablation of SVC, respectively.

**Results:** All patients in the vHPSD group were successfully isolated first‐pass; the first‐pass isolation rate was higher than in the LP group (100% vs. 86%, p=0.04). The vHPSD group achieved SVCI with a higher impedance drop (12±4 vs. 7±5, p<0.001), shorter procedure time (9±5 vs. 16±5 min, p<0.001), and fewer ablation points (13±4 vs. 18±4, p<0.001) compared to the LP group. No differences were found in the distance of the SVC circumference on the isolation line (vHPSD: 70±11 vs. LP: 70±12mm, p=0.89) or the extent of phrenic nerve capture (11±7 vs. 11±9mm, p=0.93). In the vHPSD cases, ATP was administered after isolation, and only one case of dormant conduction was observed. In two cases in the LP group, transient phrenic nerve injury was observed.

**Conclusions:** The vHPSD for SVCI might be safe and result in shorter procedure time and fewer ablation points.

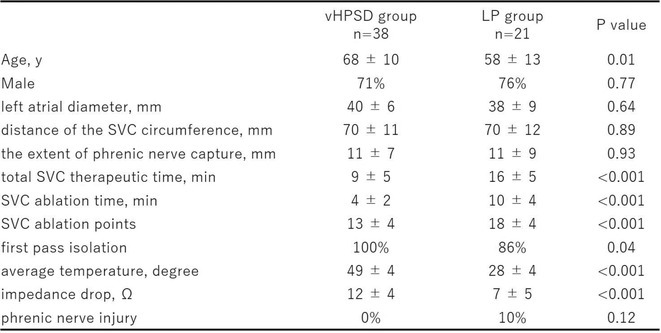

THE PROMISING IMPACT OF LEFT ATRIAL APPENDAGE CLOSURE ON HIGH STROKE RISK IN PATIENTS WITH NON‐VALVULAR ATRIAL FIBRILLATION ‐INSIGHT FROM 1‐YEAR LANDMARK ANALYSIS

**MASATO FUKUNAGA**, KENJI ANDO

Kokura Memorial Hospital, Kitakyushu, Japan

**Introduction:** Introduction: Left atrial appendage closure (LAAC) has been introduced as an alternative to anticoagulation, balancing thrombosis and bleeding risks in high‐risk populations. In prior studies with on‐treatment analysis, once the LAAC procedure was successful, the stroke rate could be lower. We, therefore, sought to elucidate the stroke rate and the prognosis after LAAC.

**Methods:** Methods: A retrospective review of 180 consecutive patients (74 WATCHMAN and 106 WATCHMAN FLX) who underwent LAAC therapy from September 2019 to December 2022 was conducted. Anticoagulation was discontinued from 45 days to 6 months after LAAC, based on their stroke and device‐related thrombus risk.

**Results:** Results: The baseline CHA2DS2‐VASc score was 4.4 ± 1.4 and the baseline HAS‐BLED score was 3.4 ± 0.9. All but one implanted procedures (99.4%) were successful and had no procedure‐related complications. During the mean follow‐up of 773 days, 9 ischemic strokes were detected, which was calculated as 2.4%/100pt‐yrs. Of these, 6 ischemic strokes in WATCHMAN Gen 2.5 and 3 in WATCHMAN FLX. As for medication at the stroke events, 6 in SAPT, 2 in anticoagulation, and 1 in no antithrombotic therapy. All but one stroke was not as severe as the Modified Rankin Scale score of 3. All antithrombotic therapy was discontinued in 51% (87/171) at 1 year after the procedure. Landmark analysis showed no differences in stroke incidence between those who discontinued and those who continued.

**Conclusions:** Conclusions: The LAAC showed a reasonably low stroke rate in this high‐risk population. The optimal medication after LAAC needs to be explored.

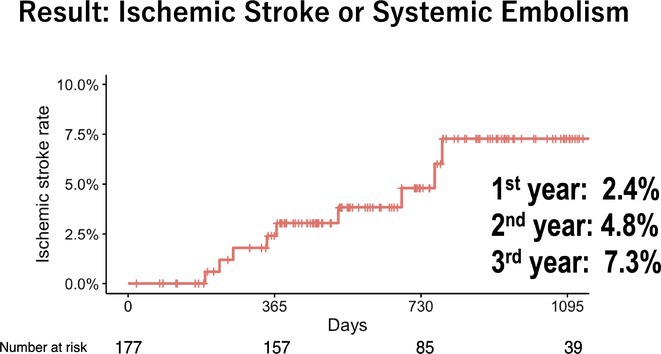

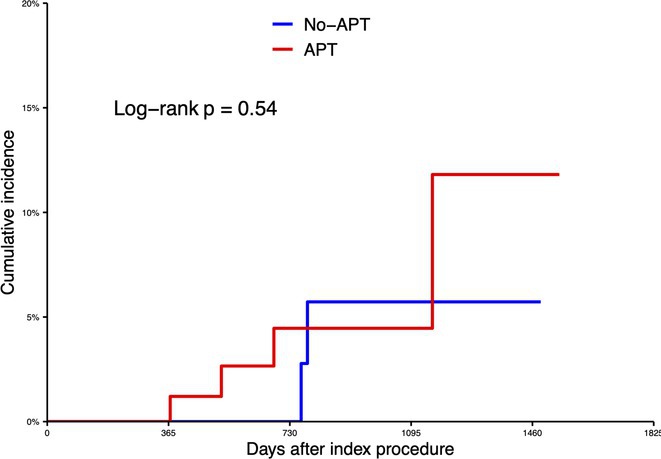

## AN ASSESSMENT WITH FIBRILLATION CYCLE LENGTH OF SURFACE ELECTROCARDIOGRAM PRIOR ABLATION FOR PERSISTENT ATRIAL FIBRILLATION

### **TAISHI FUKUSHIMA** ^1^, YOSHIHIRO SOBUE^1^, SHUHEI TAKAHARA^1^, AKANE MIYAZAKI^1^, SHUN ITO^1^, TAKEHIRO ITO^1^, YOSHINAGA MASATAKA^1^, WAKAYA FUJIWARA^1^, EIICHI WATANABE^1^, HIDEO IZAWA^2^

#### ^1^Fujita Health University Bantane Hospital, Nagoya, Aichi, Japan,^2^Fujita Health University Hospital, Toyoake, Aichi, Japan

**Introduction:** Atrial fibrillation (AF) is progressive disease due to electrical and structural remodeling. Shorten atrial refractory period due to electrical remodeling causes sustain of AF. A fibrillation cycle length (FCL) in surface ECG is related to atrial refractory period, however, little is known on assessment of AF recurrence using with FCL in patients with scheduled ablation for persistent atrial fibrillation (PerAF). Therefore we assessed the recurrence using with FCL.

**Methods:** All patients with persistent AF (PerAF) were performed ECG prior scheduled ablation. The ECG trace was obtained for 10sec, the FCL of V1 lead was assessed using with fast fourier transformation analysis. The FCL was analyzed at sampling rate 1kHz and 2048 points after subtraction of QRS wave, we assessed mean and standard deviation (SD) on FCL, mean voltage of fibrillatory wave amplitude. The ablation strategy was performed based on pulmonary vein isolation, the recurrence was defined as at least more than 30msec lasting AF.

**Results:** We enrolled 144 PerAF patients. The mean age was 72 ± 12 years, 82 patients were men. LVEF and left atrial volume index were 55 ± 12 % and 45 ± 14, respectively. During mean follow up period of 11 months, 12 (11.8%) patients met the recurrence. Although there were no significant differences on mean amplitude and SD of FCL (0.07 ± 0.05 vs. 0.05 ± 0.03 μV, 0.88 ± 0.63 ± 0.53), the FCL of recurrence groups was significant shorter than that of no recurrence groups (146 ± 24 vs. 163 ± 26 p=0.03).

**Conclusions:** The FCL may be simple and useful predictor of AF recurrence in patients with PerAF.

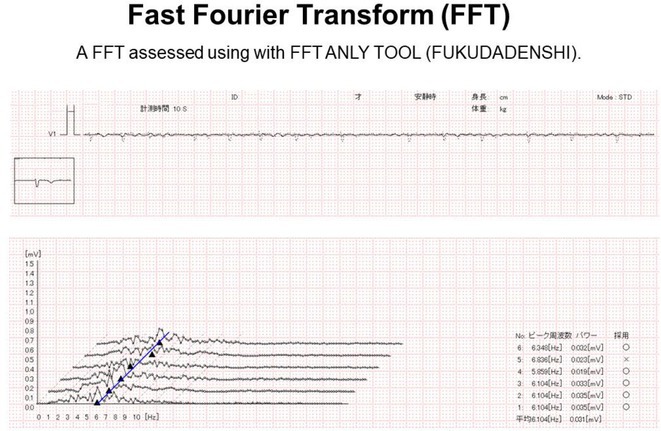

## IMPROVING THE GENETIC DIAGNOSIS OF LETHAL ARRHYTHMIC SYNDROME FOR THE PREVENTION OF SUDDEN CARDIAC DEATH

### **MEGUMI FUKUYAMA** ^1^, SEIKO OHNO^2^, KOICHI KATO^1^, KEIKO SONODA^2^, YUSUKE FUJII^1^, TOMOYA OZAWA^1^, TAKERU MAKIYAMA^3^, YOSHIHISA NAKAGAWA^1^, MINORU HORIE^1^

#### ^1^Shiga University of Medical Science, Otsu, Japan,^2^National Cerebral and Cardiovascular Center, Osaka, Japan,^3^Kyoto University Graduate School of Medicine, Kyoto, Japan

**Introduction:** The causes of sudden cardiac death (SCD) are varied, with inherited primary arrhythmic syndromes (IPAS) and ischemic heart disease being the most common. We aim to make SCD as preventable as possible by consideration of genetic diagnostic results in our cases with IPAS.

**Methods:** This cohort consists of 494 patient who received genetic testing due to lethal arrhythmic attacks (LAEs) or sudden cardiac death. Genetic testing was performed by targeted gene sequencing about 48 of arrhythmia related genes.

**Results:** As the clinical diagnosis before the testing, long QT syndrome was the most (28.9%, shown in the figure [donut chart]). In LQTS/CPVT group, identification rates of causative variants were more than 50% (shown in figure). Next, we focused on resuscitated idiopathic ventricular fibrillation (IVF, 22.7%), unrevived cardiopulmonary arrest (CPA, 1.6%), and forensic autopsy cases (n=6.3%). 14.3% of IVF cases carried any causative gene variants, and RYR2 were most frequent. Whereas, only two of likely pathogenic variants were identified in CPA or autopsy case. Thus, identification rate of causative variant is higher in resuscitated cases than that in deceased cases.

**Conclusions:** Genetic testing improved providing definitive diagnoses that is very important to prevent SCD, for resuscitated patients and family members. In addition, genomic and clinical information of as many family members as possible is very important to increase the identification rate in unrevived cases.

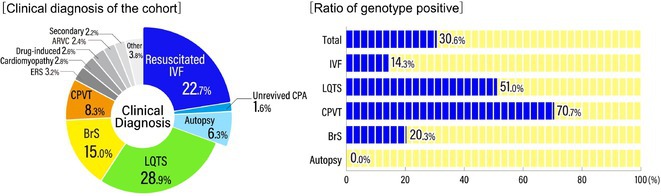

## COMPARISON OF 30 AND 15 SECONDS IN ATRIAL FIBRILLATION MAPPING USING CARTOFINDER MODULE

### **YOSHIO FURUKAWA**, TETSUROU TAKASE, AKIRA SHINODA, YUYA KOBAYASHI, TETSUSHI MITSUSHIMA, YUICHIROU IWAIDE, FUMIHARU ITAHASHI, MASATO FUJII, JUNKI YAMAMOTO, KEI ICHIHASHI, TAKUYA MAEDA, KAZUHIRO DAN, MASANORI TERAMURA, NOBUKIYO TANAKA

#### Ichinomiya Nishi Hospital, Ichinomiya, Aichi, Japan

**Introduction:** Several studies have reported high‐resolution, sequential endocardial electrograms‐based mapping (CARTOFINDER) could identify focal and rotational activities and its ablation might have a better outcome in patients with persistent AF. However, this mapping needs 30‐second electrogram acquisition at each site. This study aimed to compare the focal and rotational activities acquired in 30 seconds by CARTOFINDER system with those acquired in 15 seconds.

**Methods:** We enrolled 18 patients who were referred to our institution for undergoing catheter ablation for AF and AF was sustained during mapping. Thirty‐second CARTOFINDER was performed using OctaRay catheter, followed by 15‐second mapping at the same location. The reproducibility of focal and rotational activities were compared in each group in terms of “Site” defined as the position of center of Octaray and “Channel” defined as each electrode position.

**Results:** CARTOFINDER were obtained at 190 sites and measured at 9121 channels. With regard to focal activity, 2.9% of channels were detected in both groups and 89.7% were not detected in either groups, with an agreement rate of 92.6%. With regard to rotational activity, 3.1% of channels were detected in both group and 86.6% were not detected in either group, with an agreement rate were 89.7%. When more than 22 repetitive focal activities obtained in 30s was considered true activities, the sensitivity of focal activities in 15s was 86.0%, with a specificity of 95.6%, positive predictive value (PPV) of 19.8% and negative predictive value (NPV) of 99.8%. With regard to rotational activity, 3.1% of channels were detected in both group and 86.6% were not detected in either group, with an agreement rate were 89.7%. When more than 17 repetitive rotational activities obtained in 30s was considered true activities, the sensitivity of focal activities in 15s was 35.7%, with a specificity of 94.1%, PPV of 0.9% and NPV of 99.9%.

**Conclusions:** Fifteen‐second CARTOFINDER might reduce procedural time and detect focal activities with high reproducibility, Conversely, detection of rotational activity is less sensitive and may miss regions of interest.

## EXPLORING NOCTURNAL ARRHYTHMIA CHARACTERISTICS IN SLEEP ARCHITECTURE

### SOBHAN SALARI SHAHRBABAKI^1^, DHANI DHARMAPRANI^2^, DARIUS CHAPMAN^1^, CAMPBELL STRONG^1^, IVAYLO TONCHEV^1^, KATHRYN TIVER^1^, EVAN JENKINS^1^, **ANAND GANESAN** ^1^

#### ^1^Flinders University, Adelaide, Australia,^2^University of Adelaide, Adelaide, Australia

**Introduction:** Bursting non‐sustained nocturnal arrhythmia refers to brief irregular heart rhythms that occur during sleep, potentially leading to palpitations or chest discomfort. The characteristic feature of arrhythmia events is the abundance of short‐duration occurrences, gradually decreasing in frequency as the duration increases, following a distinct power‐law distribution pattern. Understanding the relationship between these events and sleep architecture is crucial for effective management. This study seeks to provide a quantitative analysis of the characteristics of these events, known as nocturnal arrhythmia avalanches (NAA), across different sleep stages.

**Methods:** We studied 1882 Polysomnography‐derived ECG recordings from the Multi‐ethnic Study of Atherosclerosis (MESA) to detect non‐sustained arrhythmias. We detected QRS complex and by using Pan‐Tompkins algorithm and computed R‐R time intervals to define NAA as any instance of ECG R‐R intervals decreasing by 30% of the baseline, followed by a return to 90% of the baseline. We explored the frequency and duration of NAA events during wakefulness and various sleep stages. Additionally, power‐law distribution was employed to model NAA events across each sleep stage.

**Results:** The average duration of NAA events was found to be longer during wakefulness compared to sleep (Wake: 6.51±13.11 vs. Sleep: 4.49±7.09, p<0.001), suggesting a greater occurrence of extended arrhythmias while awake. Notably, during sleep, NAA events occurring in the rapid eye movement (REM) stage were approximately 75% longer than those in deep sleep and 25% longer than those in light sleep. Additionally, the frequency of events per hour was significantly higher during sleep compared to wakefulness, with a 42% increase (p=0.007). Moreover, the power‐law exponent (α) of NAA durations in light sleep stages exceeded those in REM sleep or wakefulness by approximately 2%. Interestingly, the α value reached its peak during deep sleep.

**Conclusions:** Characteristics of nocturnal arrhythmia differ across the sleep stages. events in REM sleep were longer than those in non‐rapid eye movement.

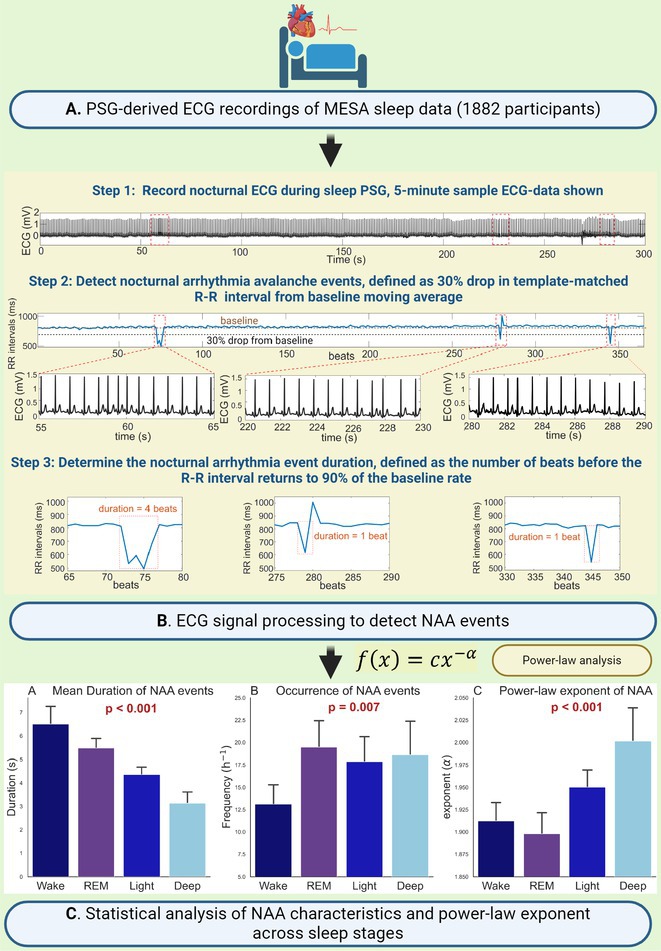

## PAEDIATRIC SODIUM CHANNEL DISEASE ‐ BRUGADA OR NOT BRUGADA?

### **CLAUDIO JR GAYETA**, HIROKO ASAKAI, CHRISTIAN TURNER, JONATHAN SKINNER, CLAIRE LAWLEY, JANINE SMITH, ANSLEY MORRISH, KAREN ROBINSON

#### The Children's Hospital at Westmead, Westmead, Australia

**Introduction:** Brugada syndrome (BrS) is diagnosed in individuals with a type 1 Brugada ECG pattern without other cause and is associated with increased risk of sudden death in adults. Relevant variants in the *SCN5A* gene are found in ~20% of cases. While the majority of childen with BrS are asymptomatic, *SCN5A* variants associated with sinus node dysfunction, atrial and/or ventricular arrhythmias have also been reported.

**Methods:** Retrospective review of all children diagnosed with BrS and/or a *SCN5A* variant (excluding gain of function in the setting of long QT 3) during the study period (January 2010‐March 2024) at a single tertiary paediatric centre in Australia. Children were identified through the departmental database. Demographic, ECG, genetic testing, and follow‐up data were extracted from medical records.

**Results:** Twenty‐six children (female n=10, 38%), were identified. The reason for presentation was an incidental type 1 Brugada pattern on ECG (n=5, 19%), family screening (n=16, 62%) and symptomatic arrhythmia (n=5, 19%). Of those with an incidental type 1 Brugada ECG, *SCN5A* gene variant status was variable (n=2 pathogenic/likely pathogenic (P/LP), n=1 VUS, n=1 negative, n=1 not known). Of those identified in family screening, 11 had inherited the P/LP familial *SCN5A* variant, 5 were diagnosed on the basis of a type 1 Brugada ECG. Of the children presenting with arrythmia, 3 had VT, one had VF, and one had AFL. Three had type 1 Brugada pattern ECG and all had *SCN5A* gene variant (4 P/LP, 1 VUS). During a mean follow‐up of 5± 4 years, there were no deaths. Four children had further arrhythmias (n=3 recurrence of previous, n=1 adolescent with a type 1 Brugada ECG and *SCN5A* variant developed atrial flutter and sinus node dysfunction). No child with an incidental type 1 Brugada ECG had documented arrythmia, although one had syncope prior to diagnosis.

**Conclusions:** Paediatric BrS and sodium channel disease represent a spectrum of disease, ranging from asymptomatic BrS to malignant tachyarrhythmia. Children presenting with a malignant arrhythmia in the setting of a relevant *SCN5A* gene variant have a high likelihood of recurrence, even in the absence of a type 1 Brugada ECG.

## RANDOMIZED COMPARISON OF LEFT BUNDLE AREA PACING USING NOVEL CPS LOCATOR SHEATH AND STYLET‐DRIVEN LEAD AND COMPARISON WITH LUMEN‐LESS LEADS

### **ANINDYA GHOSH**, ULHAS M. PANDURANGI

#### Madras Medical Mission, Chennai, India

**Introduction:** Left Bundle Branch Area Pacing (LBBaP) simulates the physiological activation of the Cardiac Conduction System.

**Methods:** The randomized prospective study enrolled the patients who underwent LBBaP implantation during January‐ April 2024 in Group A (CPS Locator 3D, Abbott, Stylet Driven Lead (Tendril STS 2088 TC 58cm) and Group B (C315‐HIS, Medtronic Select Secure 3830 69 cm, Medtronic). The primary aim was to evaluate safety, feasibility, and efficacy of the LBBaP using proprietary SDL and pre‐shaped/ fixed curve catheter while reporting procedural success.

**Results:** A total of 18 patients were randomly allocated to either Group A (n = 9, 55% (F), Mean Age: 69±12 Years, Mean LVEF: 51.44±4.77) or Group B (n = 9, 88.88% (F), Mean Age: 63±13, Mean LVEF: 54.3±4.17). The Baseline and Procedural Characteristics have been detailed in Figure 1. Both the groups performed similarly with no significant difference at implant and 1‐month follow‐up.

**Conclusions:** The CPS Locator 3D catheter is a effective tool for LBBAP using stylet‐supported pacemaker leads (Tendril STS 2088 TC) with procedural and 1 month‐follow‐up performance comparable to existing tools.

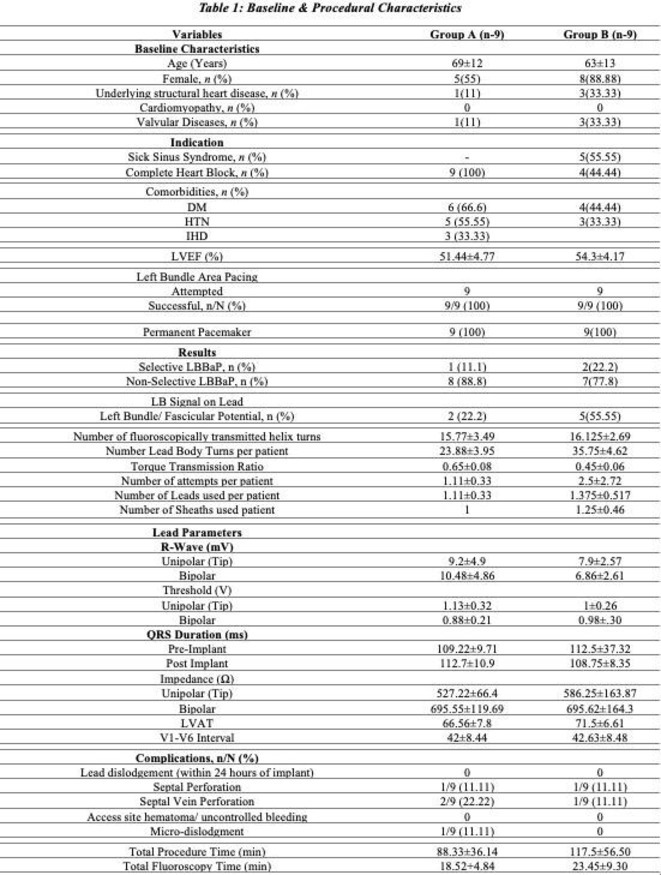

## PREMATURE VENTRICULAR CONTRACTION BURDEN IN PATIENTS WITH AN INSERTABLE CARDIAC MONITOR

### **RAKESH GOPINATHANNAIR** ^1^, DALE YOO^2^, HARISH MANYAM^3^, BABAK YASMEH^4^, FUJIAN QU^5^, NIMA BADIE^5^, KYUNGMOO RYU^5^, DANIEL KEENE^6^, DHANUNJAYA LAKKIREDDY^1^, ROY GARDNER^7^

#### ^1^Kansas City Heart Rhythm Institute, Overland Park, KS,^2^Heart Rhythm Specialists, Dallas, TX,^3^Erlanger Health System, Chattanooga, TN,^4^Aurora Medical Center, Oshkosh, WI,^5^Abbott, Sylmar, CA,^6^Imperial College London, London, United Kingdom,^7^Golden Jubilee National Hospital, Clydebank, United Kingdom

**Introduction:** Premature ventricular contractions (PVCs) exhibit day‐to‐day variation, and frequent PVCs may induce cardiomyopathy and left ventricular dysfunction. Short‐term monitoring may miss true extent of PVC burden. Continuous long‐term monitoring of PVC burden has been incorporated into a new insertable cardiac monitor (ICM) system. The aim of this study was to evaluate the PVC burden magnitude and variability in ICM patients.

**Methods:** Six hundred Assert‐IQ™ ICM devices were randomly selected from the Merlin.net™ patient care network, and those that had PVC detection enabled with more than 14 days of continuous PVC monitoring were included in this analysis. From ICM device transmissions during the monitoring period the minimum and maximum daily PVC burden, overall mean burden, and range of burden (maximum‐minimum) were reported.

**Results:** A total of 440 patients with a device PVC monitoring duration of 63 ± 23 days were analyzed. Implant indications were syncope (33%), AF (35%), cryptogenic stroke (16%), and other including palpitations and ventricular tachycardia (16%). The mean daily PVC burden exhibited a median for the entire cohort of 0.3% [IQR: 0.1‐0.8%]. However, 47 patients (40% syncope, 21% suspected AF, 15% cryptogenic stroke, 11% palpitations, and 13% others) had mean daily PVC burden >2% (see figure). This subgroup patients exhibited a median [IQR] of mean PVC burden, minimum daily burden, maximum daily burden, and daily burden range of 3.8% [2.6‐6.5%], 0.9% [0.5‐1.2%], 9.7% [6.7‐16.6%], and 8.6% [5.8‐14.9%], respectively.

**Conclusions:** Daily PVC burden is low in most ICM patients. A small subset of patients demonstrated high daily PVC burden but with significant variation over the monitoring period that could not be fully assessed by short‐term monitoring. ICM‐based long‐term PVC monitoring may provide additional diagnostic and even prognostic information for clinical decision making.

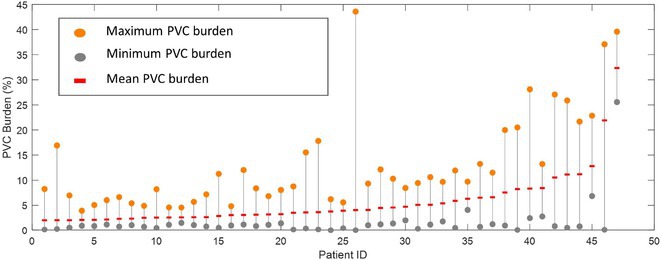

## THE RELATIONSHIP BETWEEN APICAL LEAD PLACEMENT AND RVOT IN RIGHT VENTRICULAR PACING WITH MYOCARDIAL PERFORMANCE INDEX, LEFT VENTRICULAR SYSTOLIC DYSFUNCTION AND DILATATION

### **RUTH GUNADI**, BUDI BAKTIJASA, I GDE RURUS SURYAWAN

#### Dr. Soetomo General Hospital, Surabaya, Indonesia

**Introduction:** Apical right ventricular (RV) pacing is standard practice due to facile accessibility and lead stability. However, it creates left ventricular (LV) dyssynchronization, leading to LV dilatation and decreased ejection fraction (LVEF). There is growing interest in selecting the right ventricular outflow tract (RVOT) as the primary implant site due to better physiological mechanism, thereby reducing electrical and mechanical dyssynchrony. There are not many data comparing long term outcomes of these two locations, thus further research is needed.

**Methods:** This is an analytic observational study with prospective cohort design. Single chamber, RV pacing permanent pacemaker patients with a minimum duration of 2 years in Soetomo Surabaya General Hospital were collected. We obtained baseline characteristic data, electrocardiogram recording, and PPM interrogation to assess PPM burden. Subsequently, left ventricle myocardial performance index (LVMPI), cardiac chamber dimensions, and LVEF was obtained by echocardiography.

**Results:** This study collected 56 patients (37 apical RV and 19 RVOT) with median age of 70 years (28‐85 years). The average duration of pacemaker implantation was 5.25±1.40 years. There was a significant difference (p 0.001) in the LVMPI with apical RV lead of 0.45±0.11 and the RVOT lead group of 0.35±0.07. In the RV apex group, there was a significant decrease in LVEF in baseline and post PPM conditions, with a mean baseline of 66.89±4.88% and post PPM 59.48±9.75%, p < 0.001. In addition, at post PPM there was a significant LVEF difference, RVOT group was 64.76±5.53% and apical group 59.48±9.75%, the mean difference was 5.27±2.42% (p 0.034). All samples did not experience LV dilatation.

**Conclusions:** The RV apical lead group had inferior LVMPI compared to the RVOT lead group. Left ventricle systolic function was found to be lower in the RV apical lead group compared to the RVOT lead group. There was no significant event on LV dilatation in both groups.

## EARLIEST ACTIVATION VS PACE MAPPING: THE BETTER PREDICTOR FOR SUCCESSFUL CATHETER ABLATION IN IDIOPATHIC PREMATURE VENTRICULAR COMPLEX

### AIDA FAHIRA RACHMA, ADRA ACHIRULTAN RAMAINALDO SUGIARTO, ARIIKAH DYAH LAMARA, MAKHYAN JIBRIL AL‐FARABI, **RUTH IRENA GUNADI**, RERDIN JULARIO, BUDI BAKTIJASA DHARMADJATI

#### Dr. Soetomo Regional General Hospital, Surabaya, Indonesia

**Introduction:** The identification of premature ventricular complex (PVC) origin is paramount in determining radiofrequency catheter ablation (RFCA) success. During ablation, triggered activity that underlies the pathology of PVC is identified at a focal tissue area that precedes QRS onset, thus termed as the earliest activation. Subsequently, the presence of stimulated ventricular arrhythmia (VA) is examined for its similarity to clinical VA using an algorithmic electro‐anatomical pace mapping. Although both methods are important, in some cases, the identified site using earliest activation did not produce VA with enough similarity according to pace mapping, and vice versa. The aim of this study is to compare the sensitivity and specificity of earliest activation and pace mapping as a predictor to successful PVC ablation.

**Methods:** This retrospective cohort study analyzed patients with idiopathic PVC undergoing RFCA in a single health center from the period of December 2018 to April 2024.

**Results:** A total of 67 patients were included in this study. RFCA was done using 2D mapping technique in 37 patients (56.7%). Forty three patients (64.1%) had a successful ablation. Site of origin was associated with ablation outcome (p=0.014). During RFCA, 53 patients (79.1%) were identified with PVC originating from the right ventricular outflow tract (RVOT). Area under the curve (AUC) was observed larger in pace mapping compared to earliest activation (AUC of pace mapping = 72.8% (60%‐85.6%) (p=0.002); AUC of earliest activation = 66.4% (52.6%‐80.2%) (p=0.027)). The optimal cut‐off value of earliest activation was ‐35 ms with a sensitivity of 60.5% and specificity of 54.2%. Meanwhile, the optimal cut‐off value of pace mapping was 98.5% with a sensitivity of 53.5% and specificity of 79.2%. However, this study was conducted in a burgeoning electrophysiology center with a small population. In addition, a separate analysis between different site of origin and catheter type was not conducted.

**Conclusions:** In our center, the earliest activation time had a better sensitivity while pace mapping had a better specificity.

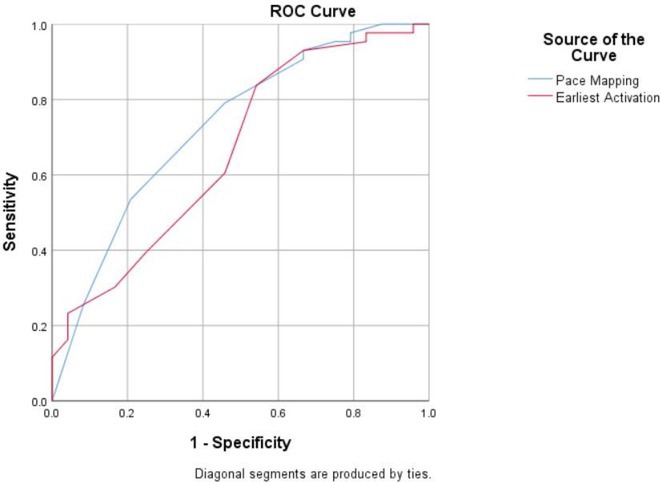

## EMERY‐DREIFUSS MUSCULAR DYSTROPHY¦FIRST SRI LANKA CASES

### **MAHENDRA GUNASEKARA** ^1^, ASUNGA DUNUWILLE^1^, ROHAN GUNAWARDENA^1^, VAJIRA H. W. DISSANAYAKE^2^

#### ^1^Institute of Cardiology, Colombo, Sri Lanka,^2^Human Genetics Unit, Faculty of Medicine, University of Colombo, Colombo, Sri Lanka

**Introduction:** Emery‐Dreifuss Muscular Dystrophy (EDMD) is a rare genetic disorder affecting skeletal muscles and the heart, characterized by early‐onset joint contractures, progressive muscle weakness, and cardiac involvement. EDMD has a global incidence of approximately 1 in 100,000 individuals. The condition has multiple inheritance patterns: X‐linked recessive, which primarily affects males due to mutations in the EMD or FHL1 genes; autosomal dominant, caused by LMNA mutations; and the rarer autosomal recessive pattern. Diagnosis requires a comprehensive clinical assessment and, when available, genetic testing. This report presents the first EDMD cases in Sri Lanka, involving two brothers with severe cardiac involvement and sick sinus syndrome, highlighting the difficulties in managing EDMD with limited resources.

**Methods:** N/A

**Results:** We report the first case series in Sri Lanka featuring two brothers, diagnosed with EDMD1.The elder brother, aged 35, had severe muscle involvement and joint contractures requiring surgery, as well as severe cardiac involvement, including a low ejection fraction and symptomatic sick sinus syndrome. He initially received VVIR pacing due to an electrically silent right atrium, but this was later upgraded to biventricular pacing with CRT‐P, given his low EF. The younger brother, aged 30, experienced muscle weakness and symptomatic sick sinus syndrome with a normal EF, necessitating DDDR pacing. Clinical exome sequencing (Figure 1) for the elder brother confirmed a likely pathogenic variant (c.397C>T, p. Gln133*) in the EMD gene, indicating X‐linked recessive EDMD1 [MIM:310300]. This diagnosis was consistent with his clinical presentation and family history, which included a maternal uncle's sudden cardiac death. The younger brother relied on clinical assessment and family history because of financial constraint

**Conclusions:** This Sri Lankan case series on Emery‐Dreifuss Muscular Dystrophy (EDMD) highlights the challenges of diagnosing and treating rare genetic disorders in resource‐limited settings. Severe cardiac complications underscore the need for timely intervention and early diagnosis to improve patient outcomes

## MITRAL VALVE ANNULAR DISJUNCTION¦ A HIDDEN CULPRIT IN STRUCTURAL MV DISEASE AND FATAL VENTRICULAR ARRHYTHMIAS

### **MAHENDRA GUNASEKARA**, ASUNGA DUNUWILLE, ROHAN GUNAWARDENA

#### Institute of Cardiology, Colombo, Sri Lanka

**Introduction:** Mitral Valve Prolapse (MVP) can lead to mitral regurgitation and arrhythmias, particularly ventricular types that heighten sudden cardiac death (SCD) risk. This risk intensifies with Mitral Annular Disjunction (MAD), where the posterior mitral valve leaflet's abnormal displacement at its insertion point causes asynchronous motion during systole. This hypermobility contributes to MVP, myxomatous degeneration, and cardiac fibrosis. While antiarrhythmic drugs may alleviate symptoms, they don't eliminate SCD risk. Surgical interventions like mitral valve repair or replacement are considered based on MR severity, symptoms, and structural changes such as left ventricular dysfunction. Risk stratification is essential in managing MVP and MAD to tailor treatment strategies.

**Methods:** N/A

**Results:** A 22‐year‐old recently married woman with a history of Mitral Valve Prolapse (MVP) and Grade 2 mitral regurgitation (MR) since adolescence, presented with worsening palpitations and fatigue. Physical examination revealed a mid‐systolic click and late systolic murmur. Repeated Holter monitoring detected ventricular ectopic beats from papillary muscles, with a burden increasing from 5.2% to 6.8% over two years. Echocardiography showed progressive left atrial enlargement and worsening MR, along with the ‘Pickelhaube Sign,’ indicative of lateral Mitral Annular Disjunction (MAD), previously overlooked. Despite financial constraints precluding a cardiac MRI, antiarrhythmic medication was initiated. The increased risk of serious arrhythmias during potential future pregnancies prompted a multidisciplinary team discussion to strategize management and emphasize family planning guidance. However, the patient has not yet confirmed her participation.

**Conclusions:** This case illustrates the complex interaction between MVP and MVAD and their combined impact on arrhythmogenic risk. MVAD, often missed during routine cardiac evaluations, is now recognized as a significant factor in severe ventricular arrhythmias in MVP patients. A comprehensive and multifaceted therapeutic approach is required in such cases.

## ARTIFICIAL INTELLIGENCE‐ENABLED ELECTROCARDIOGRAPHY TO DETECT CARDIAC DYSFUNCTION IN ASYMPTOMATIC POPULATION WITH LEFT BUNDLE BRANCH BLOCK

### HYE BIN GWAG

#### Samsung Changwon Hospital, Sungkyunkwan University School of Medicine, Changwon, Korea, Republic of

**Introduction:** Left bundle branch block (LBBB) is known to have clinical implications even among asymptomatic individuals as a manifestation or a precipitating factor of left ventricular systolic dysfunction (LVSD). Cost‐effective screening tool for LVSD is needed for proper evaluation and management of individuals with LBBB.

**Methods:** We identified subjects with LBBB on 12‐lead electrocardiogram (ECG) and left ventricular ejection fraction (LVEF) data from an echocardiography performed within 2 months from ECG acquisition during general health check‐up in our institute. We trained a convolutional neural network (CNN) model to detect severe LVSD defined as LVEF ≤35%. The residual signals between the original and reconstructed data were used as an input. Data were reconstructed by an autoencoder based anomaly detection model trained with only ECGs from those without severe LVSD. The final classification result of a subject was derived from the combination of 12 decisions for each lead using a hard voting ensemble technique. The model performance was assessed using area under the receiver operating characteristic curve (AUC), sensitivity, specificity and F1‐score.

**Results:** Among 508 subjects with LBBB, 189 (37.2%) had severe LVSD. Twelve lead data each from 408 subjects were used for training, while remaining 100 were used as a test set. The simple CNN algorithm identified severe LVSD with an AUC of 0.69, sensitivity of 0.48, specificity of 0.90, and F1‐score of 0.58, which was enhanced to 0.76, 0.57, 0.95, and 0.69, respectively, when combined with lead‐wise ensemble.

**Conclusions:** In this study, we propose an AI model using CNN and hard voting ensemble methods for prediction of LVSD in asymptomatic population with LBBB. Regarding modest performance, it warrants further validation studies with more ECG data from other populations.

## IMPACT OF IMPLANT LOOP RECORDER IMPLANT TIMING ON ATRIAL FIBRILLATION DETECTION IN EMBOLIC STROKE OF UNDETERMINED SOURCE PATIENTS

### HYE BIN GWAG

#### Samsung Changwon Hospital, Sungkyunkwan University School of Medicine, Changwon, Korea, Republic of

**Introduction:** Implantable loop recorder (ILR) has proven its efficacy to detect atrial fibrillation (AF) in embolic stroke of undetermined source (ESUS) patients. However, it remains uncertain about how long they should be monitored or when to implant ILR. To help transcend the knowledge gaps, we investigated whether timing or indication of ILR implant is associated with incidence of AF detection using National Health Insurance claims data.

**Methods:** We identified ESUS patients who received an ILR implant between July 2016 and October 2021. The first diagnosis date of stroke was considered as the date of index stroke. Patients were classified according to either implant indication (recurrent vs. non‐recurrent stroke) or ILR implant timing (early vs. late). Late implant was defined if the interval from the index stroke to ILR implant was >2 months.

**Results:** Among 1,001 patients, 197 (19.7%) were implanted for recurrent stroke and 232 (23.2%) were implanted with ILR within 2 months from index stroke. The median time between the index stroke and ILR was 578 days, which tended to be longer in those with recurrent stroke than those without (1503 vs. 1289 days, p = 0.07). There was no difference in AF incidence between recurrent and non‐recurrent stroke patients, but AF detection rate was significantly higher in the early compared to the late implant group (HR 1.47, 95% CI 0.50‐0.85 vs. p = 0.001). Patients in early implant group were younger and had lower prevalences of hypertension, diabetes, and heart failure compared with those in late implant group. Older age, diabetes, and early implant was associated with higher AF detection rate in multivariate Cox analysis.

**Conclusions:** ESUS patients with ILR in Korea have shown exceptionally long interval from the stroke to ILR implant because of reimbursement policy. Early implant of ILR (< 2 months) was associated with higher AF detection rate in ESUS patients. The reason why AF detection rate of patients with recurrent stroke was similar with that of those without seems to be a relatively long interval from the stroke to implant even in those with non‐recurrent stroke.

## REDUCING STROKE THROUGH EARLIER OPPORTUNISTIC SCREENING FOR ATRIAL FIBRILLATION FOR INDIGENOUS AUSTRALIANS

### **KYLIE GWYNNE** ^1^, VITA CHRISTIE^1,2,3^, ALENA HAINES^4^, BOE RAMBALDINI^1^, CONNIE HENSON^1,2,3^, JOSEPHINE GWYNN^4,5^, PHILLIP OBAH^1^, KAI NASH^1,6^, REKHA KHATRI^4^, DAVID BRIEGER^4,7^, JESSICA ORCHARD^8^, JUDITH M KATZENELLENBOGEN^9,10^, JOSEPH HUNG^9,11^, CHRISTOPHER X WONG^12^, LEE NEDKOFF^9,13^, TOM BRIFFA^9^, NICOLE LOWRES^5,14^, KATRINA WARD^15^, HEATHER FINLAYSON^16^, DEBBIE MCCOWEN^17^, BEN FREEDMAN^5,7,14^

#### ^1^Djurali Centre for Aboriginal and Torres Strait Islander Health Research and Education, Heart Research Institute, Newton, Australia,^2^Indigenous Studies, University of New South Wales, Sydney, Australia,^3^Health Sciences, Macquarie University, Macquarie Park, Australia,^4^Sydney School of Health Sciences, The University of Sydney, Sydney, Australia,^5^Charles Perkins Centre, The University of Sydney, Sydney, Australia,^6^HEAR Centre, Macquarie University Hearing, Macquarie Park, Australia,^7^Department of Cardiology, Concord Repatriation General Hospital, Concord, Australia,^8^Sydney School of Public Health, The University of Sydney, Sydney, Australia,^9^Cardiovascular Epidemiology Research Centre, School of Population and Global Health, University of Western Australia, Perth, Australia,^10^Wesfarmers Centre for Vaccines and Infectious Diseases, Telethon Kid's Institute, University of Western Australia, Perthj, Australia,^11^School of Medicine, University of Western Australia, Perth, Australia,^12^Centre for Heart Rhythm Disorders, University of Adelaide and Royal Adelaide Hospital, Adelaide, Australia,^13^Victor Chang Cardiac Research Institute, Darlinghurst, Australia,^14^Heart Rhythm and Stroke Prevention Group, Heart Research Institute, Newton, Australia,^15^Brewarrina Aboriginal Medical Service, Brewarrina, Australia,^16^Brewarrina Multipurpose Service, Brewarrina, Australia,^17^Armajun Aboriginal Health Service, Inverell, Australia

**Introduction:** The presence of AF at an earlier age in Indigenous peopleis likely a significant contributor to their higher incidence of stroke whencompared to other Australians. Antecedents for AF include infectious diseases such as rheumatic heartdisease, hypertension diabetes and obesity, which are more prevalent inIndigenous communities. Our national AF study demonstrated that there is a higher estimatedprevalence of AF (7.2%) in Indigenous Australians at an earlier age (55 yearsand over) than the general population.

**Methods:** We undertook a systematic reviewof literature to determine AF risk and prevalence, screening age and outcomesfor Indigenous Australians. Results were reported using PRISMA. Quality wasassessed using the JBI and CREATE tools. Indigenous researchers were involvedin all aspects of the review. An expert advisory group informedinclusion/exclusion criteria, interrogated the results and co‐createdrecommendations.

**Results:** Evidence supports earlierscreening due to higher rates of AF at a younger age, far higher AF stroke andpoorer outcomes.

**Conclusions:** Evidence from this reviewsupports opportunistic screening of Indigenous people for AF from at least age55. Further, when AF is found, guideline recommendations for management ofrate, rhythm, stroke prevention, and risk factors/co‐morbidities should befollowed and primary care providers should consider the logistics of care whendeciding on localised care pathways.

## DETECTING CONDUCTION BLOCK ZONES AS AN ABLATION TARGET SITE OF SCAR‐RELATED VENTRICULAR TACHYCARDIA WITH 3D IMAGING: INSIGHTS FROM INHEART MODELS

### **MASATO HACHISUKA**, YU‐KI IWASAKI, MAKOTO KOBAYASHI, HIROAKI HIRAYAMA, SHUHEI OKAJIMA, NOBUAKI ITO, SERINA KOBAYASHI, REI MIMURO, YUHI FUJIMOTO, HIROSHI HAYASHI, HIROSHIGE MURATA, YOSHIYASU AIZAWA, KENJI YODOGAWA, WATARU SHIMIZU, KUNIYA ASAI

#### Nippon Medical School, Tokyo, Japan

**Introduction:** Catheter ablation (CA) for ventricular tachycardia (VT) often fails to adequately assess the VT substrate and circuit due to unstable hemodynamics. Recent reports suggest that advanced cardiac imaging prior to the CA procedure can enhance treatment efficacy. However, the correlation between substrate mapping in VT ablation and imaging findings have not been fully elucidated.

**Methods:** From 2022 to 2023, 11 consecutive cases (8 men, age 58±14 years) who underwent CA of VTs were enrolled. Cardiac computed tomography (CT) or magnetic resonance (MR) were performed in 5 of 11 cases. The imaging data were retrospectively analyzed by inHeart Models, a novel 3‐dementional imaging software for detecting target arrhythmogenic substrate of VT and merged with CARTO3 or EnSite system.

**Results:** Isochronal late activation mapping was performed in all cases during sinus rhythm, right ventricular, and left ventricular pacing. Among the 5 cases analyzed with inHEART Models, deceleration zones (DZ) were identified in all cases under one or more pacing conditions. U‐shaped conduction blocks were identified in 3 cases, but only 1 case showed U‐shaped conduction blocks during VT activation mapping, consistent with the VT isthmus (Figure A). Some of these conduction blocks associated with VT circuits coincided with the margins of the dense scar in the total scar in 3 out of 4 cases (75%) evaluated by cardiac MR, suggesting that the boundaries forming the VT circuit were related to the scar heterogeneity. Furthermore, in cases where the VT isthmus was identified within the DZ, the wall thinning on cardiac CT was correlated with the presence of VT channels harboring isthmuses for VT (Figure B). Areas of wall thickness change were consistent with conduction blocks in 3 of 4 cases (75%), suggesting wall thickness changes at the boundaries contribute to the VT circuit. In other patients, the clinical VTs were unmappable due to hemodynamic instability.

**Conclusions:** Imaging assessment of scar distribution, heterogeneity and wall thinning area was associated with conduction block zones where successful ablation of VTs was achieved.

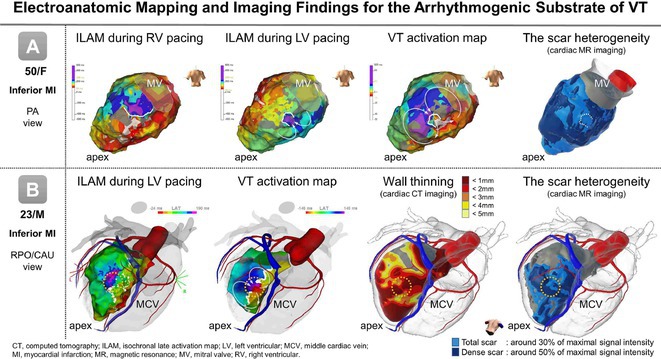

## CAN A SUPERIOR VENA CAVA FIBRILLATION BE THE CAUSE OF PERSISTENT ATRIAL FIBRILLATION?

### **HITOSHI HACHIYA**, SATOSHI HARA, TADANORI NAKATA, HIDENORI HIRANO, TAIKI ISHIZAWA, YOSHIKAZU SATO, NAOYUKI MIWA, SHIGEKI KUSA

#### Tsuchiura Kyodo Hospital, Tsuchiura, Japan

**Introduction:** There are rarely ever patients with persistent atrial fibrillation (per‐AF) due to a superior vena cava (SVC) arrhythmogenic source.

**Methods:** Nine per‐AF patients who underwent AF ablation from 2013 to 2023 at our institution had per‐AF arrhythmogenicity from the SVC. We measured the maximum SVC diameter in the long axis (SVC‐d‐long) view, that in the short axis (SVC‐d‐short) view at the location of the highest edge of the right atrial appendage on the CT image in the 9 patients. Further, the age, SVC‐d‐long, SVC‐d‐short, and left atrial diameter (LAD) in those patients (SVC‐group) were compared to those in 31 patients (non‐SVC‐group) with per‐AF.

**Results:** In all the 9 patients, AF continued or occurred after an extensive pulmonary vein isolation. Further, no AF emerged after only the SVC isolation. SVC fibrillation (Figure) was recorded during sinus rhythm after the SVC isolation in 6 (67%) of the 9 patients. However, there were no significant differences between the SVC‐group and non‐SVC‐group regarding the SVC diameter (23.9±2.4 vs. 23.4±3.8 mm for the SVC‐d‐long, p=0.67, and 19.4±3.3 vs. 19.7±3.7 mm for the SVC‐d‐short, p=0.83). The LAD in SVC‐group was significantly smaller than that in non‐SVC‐group (36.3±5.4 vs. 43.5±5.8 mm, p=0.002) and age in SVC‐group was younger than that in non‐SVC‐group (57.2±12 vs. 66.7±10 years, p=0.018). There has been no AF recurrence during a follow‐up period of 37±33 months in SVC‐group.

**Conclusions:** A superior vena cava fibrillation can be the cause of a per‐AF. Although the patients with per‐AF due to SVC arrhythmogenicity were rare, SVC should be considered as a per‐AF cause including a substrate if the LAD is relatively small and/or the patients are not elderly.

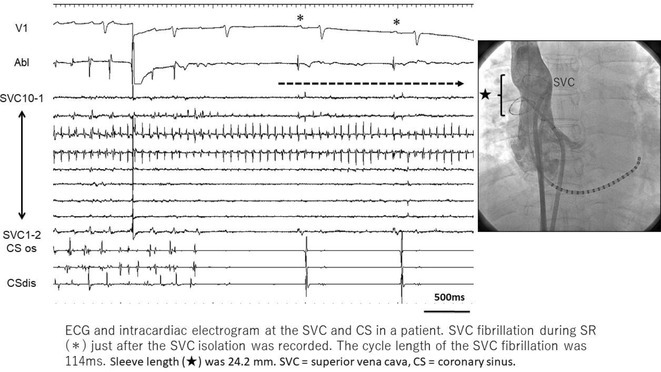

## CASE OF PERSISTENT LEFT SUPERIOR VENA CAVA AND CORONARY SINUS OSTIAL CLOSURE WITH DRAINAGE INTO LATERAL RIGHT ATRIUM

### **HIKARU HAGIWARA** ^1^, TAKURO OKAMOTO^2^, KOTOMI YOKOYAMA^2^, RUI KATANO^2^, HIROKAZU KOMORIYAMA^2^, YOSHIYA KATO^1^, TOSHIHISA ANZAI^1^

#### ^1^Hokkaido University Graduate School of Medicine School of Medicine, Sapporo, Japan,^2^Kushiro City General Hospital, Kushiro, Japan

**Introduction:** Persistent left superior vena cava (PLSVC) represents the most common thoracic venous anomaly, and is also a component of complex cardiac pathologies. Herein, we report a case of PLSVC and coronary sinus (CS) ostial closure with drainage to the posterolateral right atrium (RA) via the middle cardiac vein (MCV) and right‐sided vein.

**Methods:** br />**Results:** A 71‐year‐old man with previous myocardial infarction and left ventricular dysfunction underwent permanent pacemaker implantation for symptomatic sick sinus syndrome. Since he had decreased left ventricular function, we upgraded the conventional pacemaker to a cardiac resynchronization therapy‐defibrillator. Intraoperatively, although we tried to cannulate the CS ostium in the lower portion of the posteroseptal aspect of the RA, the ostium could not be identified. Previous coronary angiography showed that the coronary veins and PLSVC were contrasted in the venous phase, with no drainage into the posteroseptal aspect of the RA. Contrast imaging from the PLSVC revealed ostial atresia of the CS, with contrast extension along the RA floor, draining into the posterolateral RA. The angle between the PLSVC and CS was steep and the lead length was insufficient when approaching via the right‐sided vein. Therefore, the LV lead was placed in the MCV. Postoperative computed tomography showed the right‐sided vein coursing along the tricuspid annulus, which was continuous with the MCV, draining into the mid‐lateral RA.

**Conclusions:** In this case, the anomalous vessel was responsible for drainage from the coronary veins to the RA, which is a rare anomaly.

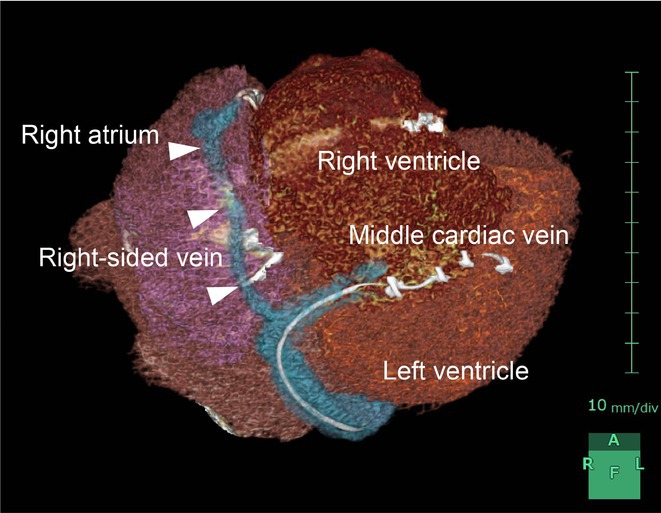

## EFFECTIVENESS OF IMPLANTABLE CARDIOVERTER DEFIBRILLATOR IN ELDERLY PATIENTS: COMPARISON OF PRIMARY VS. SECONDARY PREVENTION

### **HIKARU HAGIWARA** ^1^, NORITSUGU NAGAI^2^, KOTOMI OTSUBO^2^, SOU SASAKI^3^, HIROKAZU KOMORIYAMA^4^, YOSHIYA KATO^1^, MASAYUKI TAKAHASHI^5^, YUSUKE TOKUDA^2^, TOSHIHIRO SHIMIZU^3^, MINORU SATO^5^, TOSHIYUKI NAGAI^1^, TOSHIHISA ANZAI^1^

#### ^1^Hokkaido University Graduate School of Medicine School of Medicine, Sapporo, Japan,^2^Hakodate Municipal Hospital, Hakodate, Japan,^3^Sunagawa City Medical Center, Sunagawa, Japan,^4^Kushiro city General Hospital, Kushiro, Japan,^5^National Hospital Organization Hokkaido Medical Center, Sapporo, Japan

**Introduction:** Implantable cardioverter‐defibrillators (ICDs) are effective in preventing sudden cardiac death, regardless of their indication (primary or secondary). Previous studies reported a lower incidence of appropriate ICD therapy in primary than secondary prevention patients, although with comparable mortality rates between both groups. However, in elderly patients, a detailed analysis of the potential differences in long‐term prognosis between both groups is still lacking. We sought to investigate whether long‐term prognosis changes depending on the indication for ICD in elderly people aged > 75 years.

**Methods:** One hundred twenty consecutive patients at four tertiary hospitals who underwent ICD implantations or were upgraded from an existing permanent pacemaker between January 2011 and November 2022 were enrolled (primary prevention: 64 patients, secondary prevention: 56 patients). We compared their incidences of all‐cause death and adverse cardiovascular events, including cardiac death, appropriate ICD therapies, and heart failure hospitalization.

**Results:** There were no differences between the groups in terms of age, body mass index, or brain natriuretic peptide levels. Patients with primary prevention had lower left ventricular ejection fraction, increased left ventricular end‐diastolic diameter and larger left atrial diameter. During the median follow‐up period of 2.8 years (interquartile range 0.9‐4.8), 48 patients died. The cumulative incidence of all‐cause death (72% vs. 90%, log‐rank p = 0.01) was higher in patients with secondary prevention. The cumulative incidences of cardiac death (25% vs. 40%, log‐rank p = 0.35), appropriate ICD therapy (31% vs. 30%, log‐rank p = 0.93), and heart failure hospitalization (67% vs. 37%, log‐rank p = 0.71) were comparable between the groups.

**Conclusions:** During long‐term follow‐up, primary prevention patients showed a lower risk of all‐cause mortality, although comparable rates of cardiac death, appropriate ICD therapy and heart failure hospitalization were observed between both groups.

## CONDUCTION SYSTEM PACING FOR RESTORATION OF SYNCHRONOUS VENTRICULAR CONTRACTION IN PATIENTS WITH PACING‐INDUCED MODERATE LEFT VENTRICULAR DYSFUNCTION

### DOOYOUNG HAN

#### Asan medical center, Seoul, Korea, Republic of

**Introduction:** The restoration of synchronous ventricular contraction is an essential treatment for patients with pacing‐induced left ventricular dysfunction. Herein, we present a case of utilizing lead extraction and conduction system pacing (CSP) in a patient with moderate left ventricular dysfunction.

**Methods:** A 64‐year‐old female patient was presented at the emergency room with dyspnea, orthopnea, and generalized edema. She had received a conventional dual‐chamber pacemaker implantation for a complete atrioventricular block 10 years ago. Recent echocardiography taken 2 months before admission revealed a decreased ejection fraction (EF) of 40%, resulting from dyssynchronous left ventricular contraction from apical pacing. A moderate degree of functional mitral regurgitation and aortic regurgitation were also found. In addition, an insulation break was suspected in the atrial lead. Although her EF did not meet the criteria for cardiac resynchronization therapy, due to significant heart failure symptoms (NYHA III) and functional MR despite heart failure medications, we tried CSP to restore synchronous ventricular contraction. Due to near‐total obstruction of the subclavian vein, we extracted the dysfunctional atrial lead first. After extraction, the guidewire was advanced through the inner lumen of the mechanical extraction sheath. The left bundle branch area was targeted, and the lead was penetrated into the interventricular septum.

**Results:** The resultant pacing morphology met the non‐selective left bundle branch capture criteria (left ventricular activation time 75ms, QRS duration 144ms). There were no significant complications during the procedure. In the outpatient clinic follow‐up at one month, her dyspnea improved from NYHA class III to I, and edema had disappeared. The follow‐up echocardiography revealed an improved EF of 45% and the disappearance of moderate functional MR.

**Conclusions:** In patients with moderate LV dysfunction from chronic right ventricular apical pacing, CSP can restore synchronous contraction and improve patient symptoms.

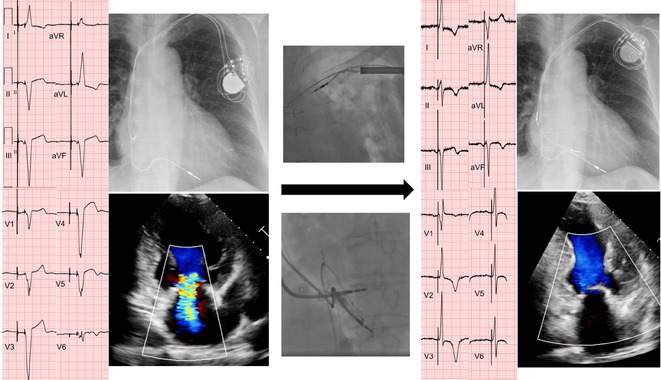

## RIGHT VENTRICULAR DYSFUNCTION IN HIGH BURDEN IDIOPATHIC INFERIOR AXIS PREMATURE VENTRICULAR CONTRACTION POPULATION

### **DICKY HANAFY**, PUTRI INDRISIA, SUNU RAHARDJO, AMILIANA SOESANTO, M. RIZKY FELANI, D. YUGO HERMANTO, YOGA YUNIARDI, V. GILANG REJEKI, A. AGITA SEMBIRING

#### National Cardiovascular Centre Harapan Kita, Jakarta, Indonesia

**Introduction:** The relationship between premature ventricular contractions (PVC) and right ventricular (RV) function is not widely known. Left ventricular dysfunction due to PVC is known as PVC‐Induced cardiomyopathy (PIC) and suppressing the PVC substrate would improve left ventricular function. The effect of PVC ablation on changes in right ventricular (RV) function in patients with subtle subclinical RV dysfunction remains unknown.

**Methods:** Basic and speckle‐tracking echocardiography has been performed on 42 individuals with symptomatic idiopathic inferior axis PVC before and one month after a successful

**Results:** The burden and QRS duration of premature ventricular contractions (PVC) were notably higher in the group with right ventricular (RV) dysfunction compared to those with normal RV function (p = 0.012 and p = 0.009, respectively). In both groups, measurements of RV function before and after ablation, specifically global longitudinal strain (GLS) and free wall longitudinal strain (FWLS), demonstrated significant changes. These improvements were more pronounced in the group with RV dysfunction (FWLS 9.7 ± 4.0, p < 0.001; GLS 7.5 ± 4.2, p < 0.001). Lower initial FWLS and GLS before ablation emerged as significant parameters in the multivariate analysis for the improvement of RV function post‐ablation.

**Conclusions:** Patients with RV dysfunction had higher PVC burden and wider QRS duration. Patients with idiopathic PVC and impaired RV function may experience improvements in RV function after successful PVC ablation.

## LEFT AND RIGHT DISCRIMINATION OF ACCESSORY PATHWAY LOCATION BASED ON EARLIEST DELTA WAVE OCCURRENCE ON PRECORDIAL LEADS

### **AHMAD HANDAYANI** ^1^, SHEILA DHIENE PUTRI^1^, FADLI ILHAMI^2^, AMRYI KARIM MAPPANYUKI^3^, MIRHANSYAH PERDANA HUTASUHUT^2,4^, ANGGIA CHAIRUDDIN LUBIS^2,4^

#### ^1^Universitas Muhammadiyah Sumatera Utara, Medan, Indonesia,^2^Universitas Sumatera Utara, Medan, Indonesia,^3^Transmedic Corporation, Medan, Indonesia,^4^Adam Malik National General Hospital, Medan, Indonesia

**Introduction:** Localization of accessory pathway (AP) in Wolf Parkinson White (WPW) syndrome is crucial as it may affect the preparation needed. Even though various algorithms have been proposed for identifying the location of an accessory pathway, uncertainty remains regarding the predictive value of the delta wave shape, particularly in lead V1. Considering that lead V1 is positioned more to the right and serves as the most anterior lead, the appearance of a delta wave should occur earlier in V1 if an AP originates from the right side of the heart.

**Methods:** The aim of the study was to assess the predictive ability of the earliest delta wave occurrence on precordial leads. It was a mixed cohort study of the patients with WPW syndrome who underwent conventional electrophysiology study and ablation in Adam Malik General Hospital Medan Indonesia since 2021 to March 2024. Only the patients with manifest delta wave on precordial leads and with successful ablation, proofing that it was the precise location of the AP, included in the study. The study assessed the appearance of the earliest delta wave on the ECG leads, recorded and analyzed at a speed of 200 ms, and validated by a blind examiner.

**Results:** In this study, 17 patients were included. Among them, 8 patients had right‐sided accessory pathways (AP), while 9 patients had left‐sided AP. Within the right‐sided AP group, the earliest delta wave appeared in lead V1 for 7 patients. Conversely, in the other group, 7 patients exhibited the earliest delta wave in non‐V1 leads (either V2‐V6). The Fisher exact test revealed a significant association between the location of AP and the precordial lead where the delta wave appeared earliest (p=0.015)

**Conclusions:** This study demonstrates that the precordial lead where the earliest delta wave appears can serve as a predictor for a left or right location of AP in WPW syndrome. However, for a more precise localization of AP, alternative algorithms must be utilized. Future studies can be conducted with better sample size.

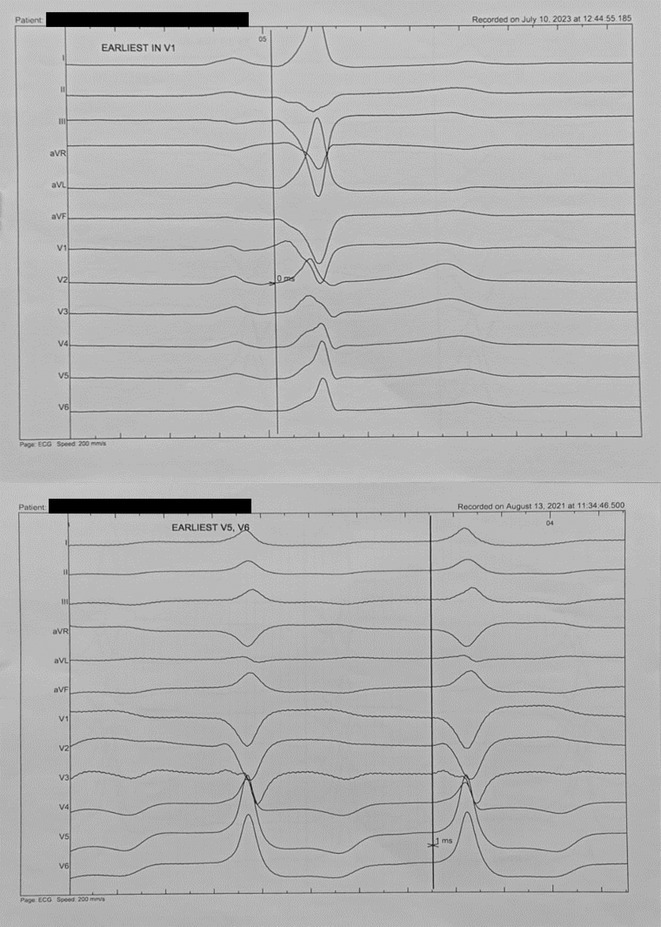

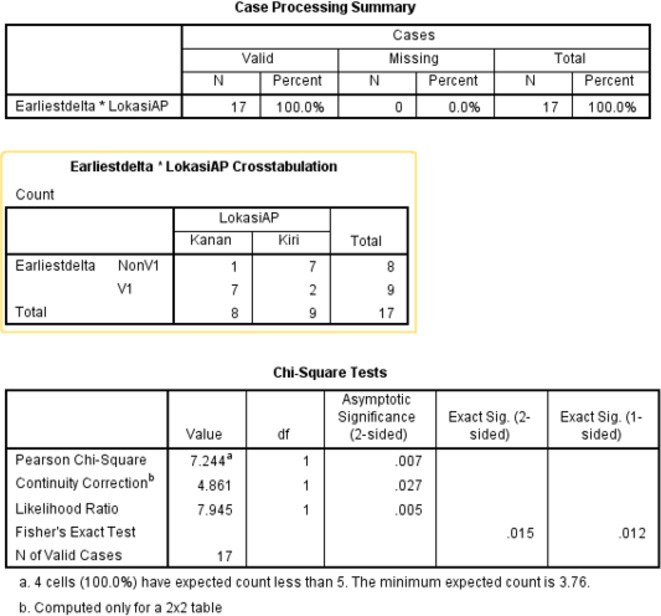

## CLINICAL EFFICACY OF OPTIMIZED CARDIAC RESYNCHRONIZATION THERAPY IN PATIENTS WITH HEART FAILURE COMPLICATED WITH ATRIOVENTRICULAR BLOCK

### SU HAO

#### THE FIRST AFFILIATED HOSPITAL OF USTC ANHUIPROVINCIALHOSPITAL, Hefei, China

**Introduction:** Left bundle branch region pacing for heart failure patients with atrioventricular conduction block offers the advantage of avoiding complications associated with dual ventricular pacing. However, its clinical application and efficacy observation in China have been limited by relatively small sample sizes and short follow‐up durations, necessitating further comprehensive clinical investigations.

**Methods:** A retrospective analysis was conducted on a cohort of patients who underwent left bundle branch region pacing for heart failure combined with third‐degree/high‐degree atrioventricular block or atrial fibrillation with atrioventricular block at our hospital, between February 2021 and April 2022. To compare the therapeutic outcomes of left bundle branch region pacing with traditional dual ventricular pacing, propensity score matching was employed to create a control group (Biv‐CRT group) consisting of patients who underwent traditional dual ventricular pacing during the corresponding timeframe. Baseline data, surgical details, and 6‐month postoperative follow‐up data were collected for both the experimental group (Lot‐CRT group) and the control group. Changes in electrocardiographic measurements, pacing parameters, clinical symptoms, and other pertinent indicators were analyzed to assess improvements in cardiac function, safety profiles, efficacy, and discrepancies between left bundle branch region pacing and traditional dual ventricular pacing.

**Results:** The results are shown in the attached figure

**Conclusions:** Left bundle branch region pacing represents a secure and effective therapeutic strategy, characterized by consistent pacing parameters. It elicits a reduction in QRS duration, an enhancement in left ventricular ejection fraction, and proves non‐inferior to traditional dual ventricular pacing in terms of improving cardiac function.

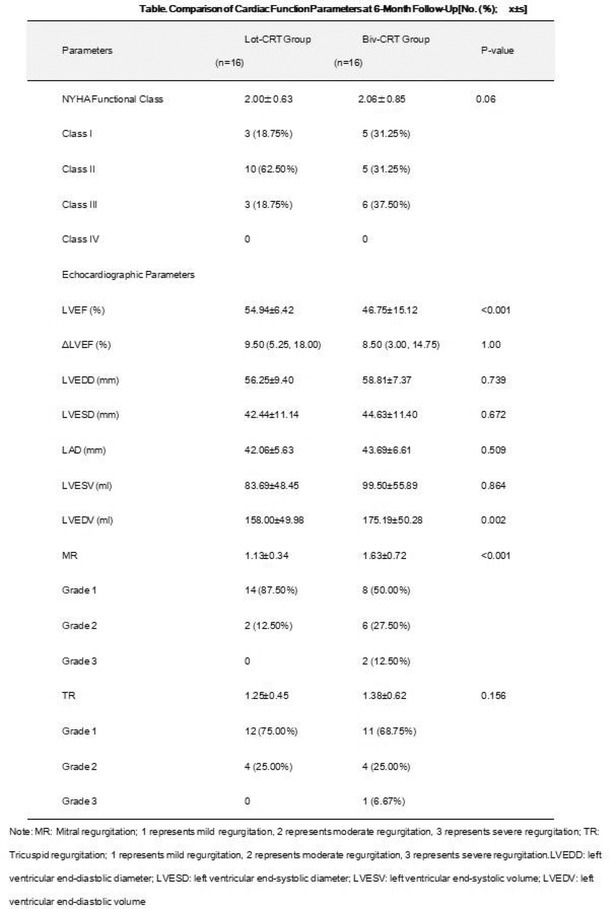

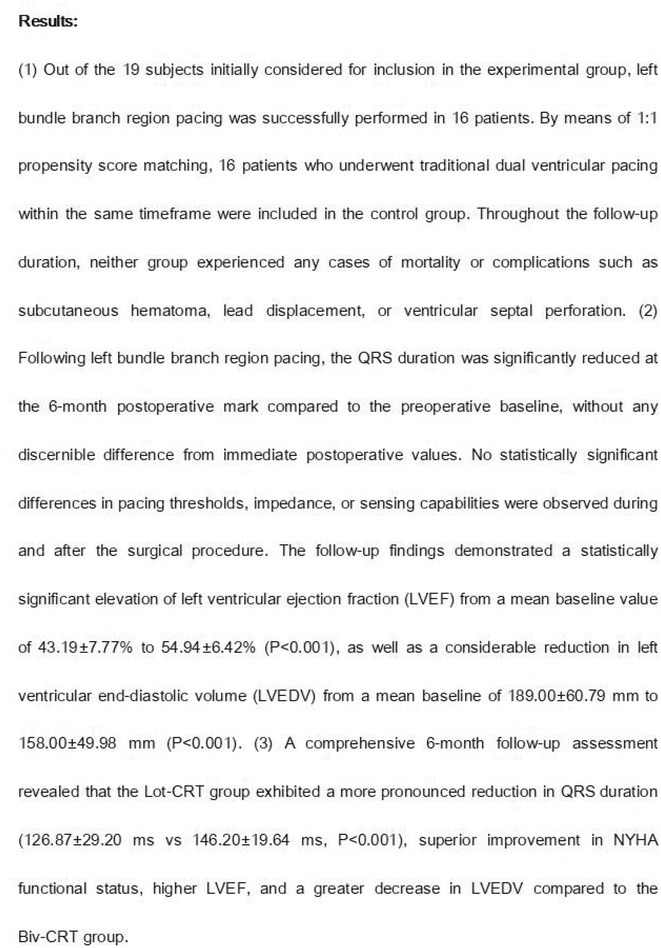

## CAN THE QRS MORPHOLOGY OF OUTFLOW VENTRICULAR ARRHYTHMIA CHANGE WHEN RIGHT BUNDLE BRANCH BLOCK EMERGES DURING SINUS RHYTHM?

### **SATOSHI HARA**, NAOYUKI MIWA, SHIGEKI KUSA, YOSHIKAZU SATO, TADANORI NAKATA, HIDENORI HIRANO, HITOSHI HACHIYA

#### Tsuchiura Kyodo Hospital, Ibaraki, Japan

**Introduction:** Bundle branch block, especially right bundle branch block (RBBB), is sometimes provoked by catheter manipulation during ablation procedures. A few reports have described the effect of procedure‐induced RBBB on outflow tract ventricular arrhythmia (OT‐VA). We investigated whether iatrogenic RBBB affects the QRS morphology of OT‐VA.

**Methods:** We retrospectively investigated 195 consecutive patients who underwent an initial ablation of VA. The study inclusion criteria were VAs that were successfully ablated in the outflow tract (OT) and in whom right bundle branch block (RBBB) was induced by catheter manipulation close to the His bundle area during sinus rhythm, before any radiofrequency application. We analyzed the QRS morphology of the VAs with and without RBBB during sinus beats.

**Results:** Twenty‐four patients (age 59.0±17.9 years, female 14) developed RBBB before radiofrequency application during their procedure. The type of RBBB was complete RBBB in 19 patients and incomplete RBBB in 4 patients. Of the remaining two patients, one had incomplete RBBB at baseline and displayed complete RBBB during the procedure. The other had both incomplete and complete RBBB provoked during the procedure. QRS duration during RBBB increased by 43.9±19.9 ms relative to normal conduction. The successful ablation sites of the VAs were the right ventricular outflow tract (RVOT) in 12 patients, pulmonary artery in 1, left coronary cusp in 5, right coronary cusp in 3, right‐left cusp junction in 2, and great cardiac vein in 2. QRS‐morphology change was observed in 5 (20%) cases. The successful ablation sites in that group were the left coronary cusp in 3 cases, right coronary cusp in 1, and RVOT septum in 1. The QRS duration of the VAs increased during RBBB.(**Figure**)

**Conclusions:** There are some cases of OT‐VAs in which the QRS waveform changes with the appearance of catheter induced RBBB. We need to be aware that when QRS morphology changes during an OT‐VA ablation, it does not necessarily mean that the origin or exit of the VA has changed.

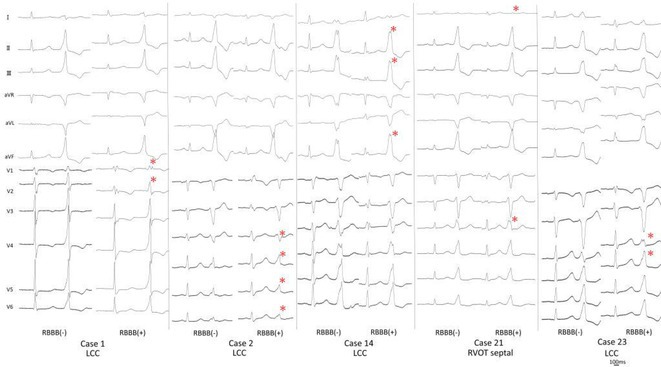

## A NEW TYPE OF BI‐ATRIAL TACHYCARDIA REVEALED BY HIGH‐DENSITY ACTIVATION MAPPING

### **OSAMU HASHIMOTO**, YU IEMURA, YASUO TOKORO

#### Shinkuki General Hospital, Kuki, Japan

**Introduction:** A 70‐year‐old man with a history of diabetes mellitus, dyslipidemia, myocardial infarction, and congestive heart failure underwent mitral valve replacement (MVR) two years ago for severe mitral regurgitation (MR). Recently, he presented with palpitations and was found to have a heart rate of 120 bpm with narrow QRS tachycardia. Chest X‐ray showed cardiomegaly but no pulmonary congestion. Echocardiography revealed a left ventricular ejection fraction of 61%, mild aortic regurgitation, and severe MR due to a paravalvular leak, with MR still moderate to severe.

His heart failure medication was adjusted, and a calcium blocker was prescribed to reduce his heart rate to 60 bpm. Further evaluation confirmed a 4:1 atrial tachycardia (AT) ratio, thought to be exacerbated by previous heart surgery. Catheter ablation was planned for AT treatment.

During 3‐dimensional mapping, surgical incision lines in the anterior left atrium (LA) and right atrium (RA) were identified. Bi‐atrial tachycardia (Bi‐AT) with a tachycardia cycle length of 270 msec featured clockwise rotation. The RA propagation of the Bi‐AT circuit moved around the tricuspid annulus, from the lower RA septum through the lateral RA to the superior vena cava (SVC), then to the LA roof via the Bachman bundle. LA propagation ran from the roof posteriorly to the bottom and anterior LA, then to the RA via the lower LA septum and the coronary sinus (CS). A single‐loop macroreentrant circuit involving parts of the tricuspid annulus and the LA septum was revealed. Radiofrequency linear ablation at the lateral RA successfully terminated the Bi‐AT.

Post‐ablation, the patient was asymptomatic with no complications. This case represented a novel Bi‐AT form, not fitting existing classifications, revealed through high‐density activation mapping.

**Methods:** N/A

**Results:** N/A

**Conclusions:** Our case presented a novel form of Bi‐AT revealed by high‐density activation mapping, which did not fit any of the known types.

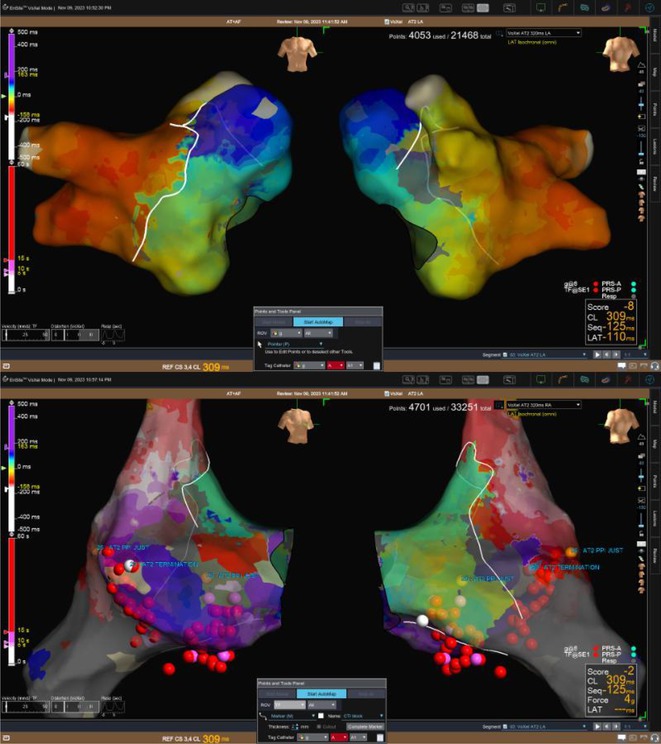

## CARDIAC PACING IN POLYMORPHIC VT STORM: HOW FAST TO PACE ? HOW LONG TO PACE?

### **PRADEEP HASIJA** ^1^, NEELAM CHHABRA^2^

#### ^1^Holy Family Hospital, Bandra West, Mumbai, India,^2^Lilawati Hospital and Research Centre, Bandra West, Mumbai, India

**Introduction:** Cardiac pacing for arrhythmic storm due to polymorphic VT is a life saving procedure. This case report provides insight into pacing dynamics as rarity of such events preclude evidence based guidelines.

**Methods:** N/A

**Results:** A 23‐year‐old lady (G1P0A0) while being managed for puerperal sepsis following caesarean delivery had suffered with frequent episodes of syncope and VT. She received approximately 36‐40 DC shocks over the 24 hours and loaded with Amiodarone and Lignocaine IV injection when referral was made to our hospital. She had hypokalemia (serum potassium was 2.8 mg/dl) for which she was being given appropriate correction along with inotropes. She had received 3 DC shocks in ambulance while in transit. Her continuous ECG monitoring showed frequent runs of polymorphic VT (Fig. 1A). She had raised BNP, Troponin T and LVEF of 35%, with global hypokinesia on echocardiography. The ECG, in the intervening VT episodes had QT prolongation, measuring 520 msec (Fig. 2A). The history of recurrent syncope since childhood, suggested a high probability of congenital long QT syndrome. as per Swartz scoring. At our centre, patient was managed on temporary pacing at cycle length of 600 msec (100bpm) (Fig. 1B)., She continued to have repeated runs of non‐sustained polymorphic VT, either reverting spontaneously or responded to overdrive pacing. The underlying rhythm was bizarre with prolonged QT even after 24 hrs (Fig. 1C). The beta‐blockers (Tab Bisoprolol ) started after withdrawal of inotropes and dose was up titrated monitoring the QT interval and heart rate. She was implanted with a dual chamber ICD programmed to AAI mode (Fig. 2B‐C). Subsequent review at 4 weeks, showed her LV function normal, QT 480 msec and pacing rate was reduced to permit sinus rhythm (Fig. 2C). On yearly follow up thereafter, for past 7years, she had no syncope, no shocks or recurrence of VT on ICD interrogation.

**Conclusions:** Atrial based pacing effectively modulates the QT interval and provides feasibility of overdrive suppression of VT episodes in Polymorphic VT storm. Pacing is required till maximum shortening of QT is achieved with tolerable beta‐blocker dose.

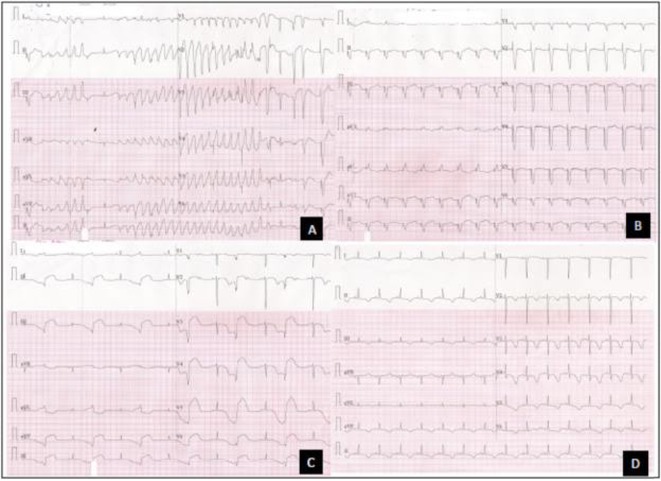

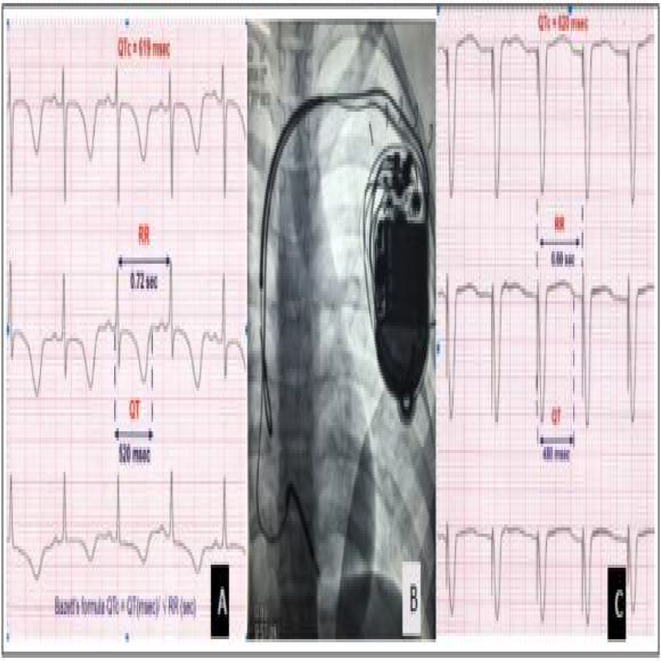

## THINLINE/FINELINE LEAD EXTRACTION OUTCOMES IN OUR HOSPITAL

### **TETSUHISA HATTORI**, TAKASHI YAMASAKI, MISUN PAK, KEN KAKITA

#### Koseikai Takeda Hospital, Kyoto, Japan

**Introduction:** Thinline/Fineline pacing leads (Intermedics/Boston scientific) have a revolutionary design consisting of two separately insulated, helically wound, parallel coils. These leads are known to be at high‐risk for disruption with traction that causes difficulties during lead extraction procedures. Thereby, the pacing leads may require special care when extracting, and are probably more difficult to completely extract. However, the actual success rate of transvenous extraction remains unclear.

**Methods:** A total of 53 patients underwent transvenous lead extraction at our hospital from December 2020 to November 2023. Of these patients, we extracted patients who had been implanted with Thinline/Fineline leads, and retrospectively assessed outcomes. Procedural complexity and results were defined as recommended by recent consensus statements.

**Results:** A total of 35 Thinline/Fineline leads were removed from 28 patients. The median age of lead extracted was 14.0 years (interquartile range 9.5‐17.1 years). Lead tip fixation was passive in three leads (9%) and active in 32 leads (91%). Locking stylets and powered sheaths were used in all the leads. Twelve leads (35.2%) were broken during the procedure. Of the broken leads, five leads were extracted without femoral approach, six leads were removed via femoral approach using snare catheters. In only one lead, the fragment between the ring and tip electrodes could not be extracted with snare catheters. The lead fixation mechanism was passive, and the dwell time was 18.7 years. These results may exhibit that success rate of transvenous extraction was not low despite a high rate of lead break during the procedure and complete lead removal was difficult in passive and older Thinline/Fineline lead.

**Conclusions:** Thinline/Fineline leads can be removed successfully though they require complex extraction.

## WEARABLES ARE A VIABLE DIGITAL HEALTH TOOL FOR OLDER INDIGENOUS ADULTS LIVING REMOTELY IN AUSTRALIA

### **CONNIE HENSON** ^1^, BEN FREEDMAN^1^, BOE RAMBALDINI^1^, BRONWYN CARLSON^2^, CHRISHAN NALLIAH^2^, FELICITY CHAPMAN^3^, GINA SHEPHERD^3^, JESSICA ORCHARD^4^, JOHN SKINNER^1^, JOSEPHINE GWYNN^4^, RONALD CASTELLINO^4^, ROBYN RAMSDEN^5^, SOPHIA SPEIER^6^, SUUD NAHDI^4^, VITA CHRISTIE^1^, YANSONG HUANG^4^, KATRINA WARD^7^, KYLIE GWYNNE^1^

#### ^1^Heart Reasearch Institute, Newtown, Australia,^2^Macquarie University, Sydney, Australia,^3^Djurali Centre, Newtown, Australia,^4^Sydney University, Sydney, Australia,^5^NSW Rural Doctors Network, Melbourne, Australia,^6^University of Victoria, Victoria, BC, Canada,^7^Brewarrina Medical Services, Brewarrina, Australia

**Introduction:** Indigenous Australians are more likely to have undetected and untreated AF and an increased risk of AF‐related stroke. Despite this higher prevalence and evidence that older Indigenous people are interested in using wearables and other digital health technologies, no research has involved older Indigenous people using wearables for heart health monitoring. To ensure the opportunities and benefits of digital health are distributed equitably, it is critical to partner with older Indigenous people to test the acceptability and feasibility of using these technologies. Health programs for Indigenous people are most effective and sustainable when prioritising Indigenous perspectives.

**Methods:** This mixed‐methods study was co‐designed and co‐implemented with the local Aboriginal Community Controlled Health Service (ACCHS) in a remote area of New South Wales, Australia. It examined the acceptability and feasibility for Indigenous people aged 55 and older living in remote locations to use wearables (patches and watches) to detect atrial fibrillation (AF) and high blood pressure. The study included active involvement and co‐design with the participants.

**Results:** Despite challenging conditions (temperature >36 degrees Celsius) and variable internet connectivity, eleven Indigenous older adults participated in a five‐day wearables program in a remote location. Participants indicated that digital health devices were acceptable and feasible for older Indigenous users. They described high levels of comfort, safety and convenience when using wearables (patches and watches) to detect AF and blood pressure. The ACCHS indicated that the delivery of the program was feasible in this setting and expressed interest in a broader implementation.

**Conclusions:** Older Indigenous Australians are motivated to use wearable health devices. Co‐design builds on Indigenous people's self‐determination and propensity to experiment with new technologies and ensures research is designed and implemented in a culturally safe and respectful manner.

## EARLY CATASTROPHE IN PATIENT WITH IMPLANTED TEMPORARY PACEMAKER

### **KATHERINE HERMANTO**, MOHAMMAD IQBAL, GIKY KARWIKY, CHAERUL ACHMAD

#### Universitas Padjadjaran, Bandung, Indonesia

**Introduction:** Thrombosis related to temporary pacemaker (TPM) implantation can be asymptomatic or lead to pulmonary embolism. We present a case of a patient who developed early‐onset deep vein thrombosis (DVT) and pulmonary embolism (PE) after TPM implantation to raise awareness clinicians about thromboembolism risk, despite the extremely rare incidence of the occurrence (approximately 0.8%).

**Methods:** N/A

**Results: Case Illustration** A 62‐year‐old male was diagnosed with high degree AV‐block 3:1 and underwent TPM implantation. He was obese and had a past medical history of hypertension and congestive heart failure. After 4 days of immobilization, the TPM was replaced with left bundle branch area pacing. He developed right lower limb edema with suspicion of DVT soon after TPM lead removal. Doppler ultrasonography found thrombi in the right iliac down to the right popliteal vein. He complained about a sudden episode of dyspnea and palpitations with hemodynamic deterioration. Laboratories were notable for thrombocytopenia, elevated cardiac troponin I and D‐dimer levels with no signs of hypercoagulability (Protein C, protein S, antithrombin‐III, and homocysteine were normal). Echocardiography findings strongly suggest acute PE with multiple thrombi founded in the tricuspid valve and right ventricle (RV) along the PPM lead. The patient was treated with fondaparinux for 7 days and switched to rivaroxaban. He was stable and discharged with a smaller residual thrombus.

**Conclusions:** TPM implantation causes injury to the vessel wall and blood stasis. The combination of the pathological processes causes the development of DVT, which embolizes to pulmonary artery in such short duration of time after implantation. This patient had several risk factors including obesity, heart failure, and immobilization. Treatment should be individualized according to the patient's clinical situation. Although the case was challenging, it was well treated with anticoagulants, despite the fact that no thrombolytics were given considering consumptive thrombocytopenia.

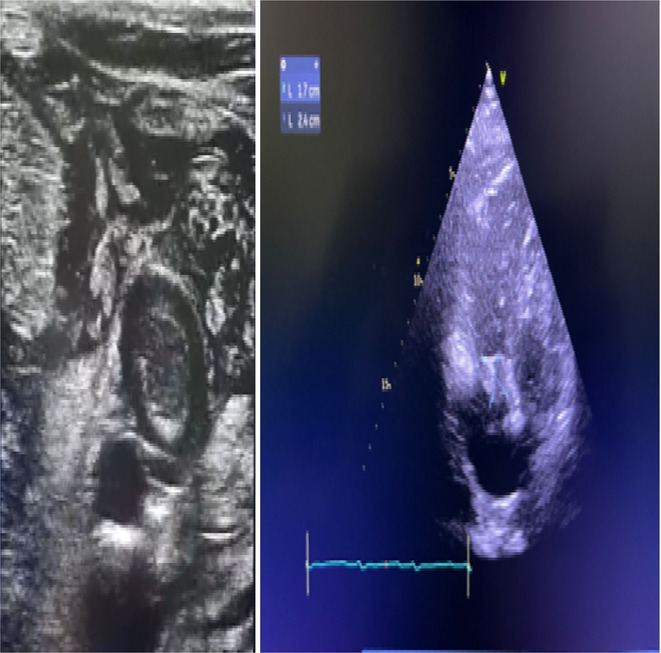

## CARDIAC QUAGMIRE:NAVIGATING THE TREACHEROUS OF ARRHYTHMIA AND CARDIOMYOPATHY IN ADULT EMERY‐DREIFUSS MUSCULAR DYSTROPHY

### **FERA HIDAYATI** ^1^, ERIKA MAHARANI^1^, KRISTY ISKANDAR^2^

#### ^1^SARDJITO HOSPITAL, SLEMAN, Indonesia,^2^UGM ACADEMIC HOSPITAL, SLEMAN, Indonesia

**Introduction:** Emery‐Dreifuss muscular dystrophy (EDMD) type 2 is a rare genetic disorder which caused by a mutation in the LMNA gene, resulting in the lamin A/C nuclear proteins. Cardiomyopathy, conduction system problems, atrial and ventricular arrhythmia are among the documented cardiac phenotypic manifestations of EDMD type 2.

**Methods:** N/A

**Results:** A 37‐year‐old male was referred to Dr. Sardjito Hospital after experiencing palpitations and syncope. Six years ago, he and his son were diagnosed with EDMD type 2, according to a genetic investigation conducted at UGM Academic Hospital. The examination revealed an LMNA gene mutation. The patient was diagnosed with unstable VT associated with acute coronary syndrome. The ECG showed monomorphic RBBB VT, 219 bpm. Following repeated cardioversion, a junctional rhythm ECG was noted, RBBB with dynamic ST‐T changes. Cardiac enzyme (hs troponin T) increased to 266 ng/L. Coroangiography revealed normocoroner, and temporary pacemaker was implanted. Reduced LVEF (34%), as well as right and left atrial dilatation, were observed on echocardiogram. Late gadolinium enhancement revealed fibrosis in the LA, RA, and LV. After the implantation of a VVIR permanent pacemaker, the patient received optimal medication therapy. A 6‐month evaluation showed high burden of V pace with frequent PVCs and decreased LVEF to 27%. He did not complain of palpitations, shortness of breath, or fainting.

**Conclusions:** Establishing a definitive diagnosis is crucial in EDMD patients so that appropriate treatment shall be given to prevent sudden cardiac death.

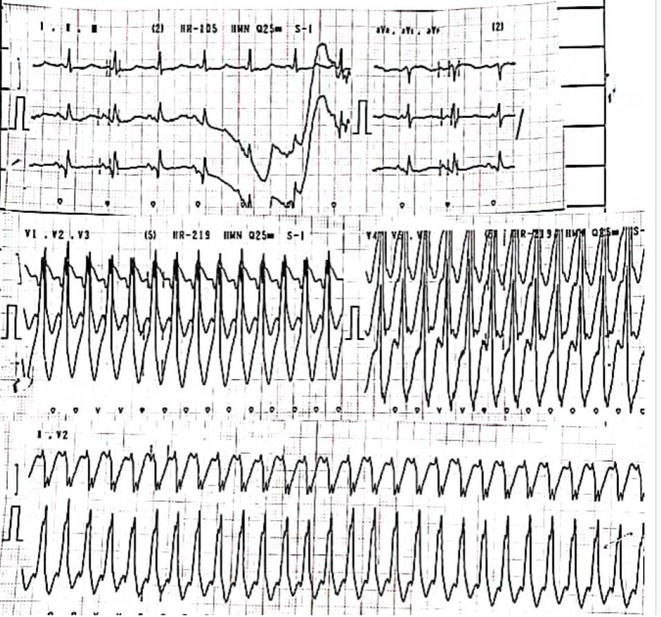

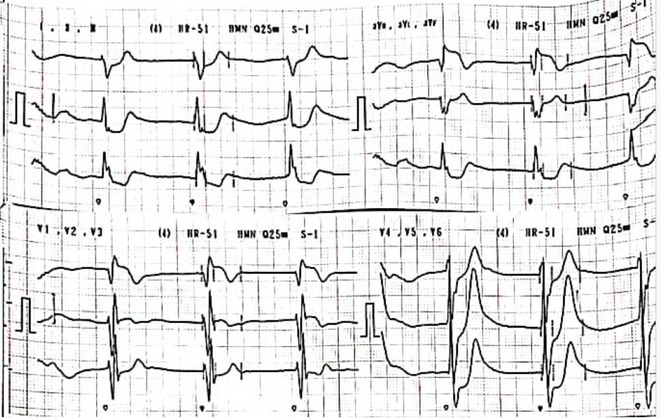

## COST‐EFFECTIVENESS OF AN ANTIBACTERIAL ENVELOPE IN PATIENTS AT HIGH RISK OF CARDIAC IMPLANTABLE ELECTRONIC DEVICE INFECTION FROM AN AUSTRALIAN PUBLIC HOSPITAL PERSPECTIVE

### **MICHELLE HILL** ^1^, BEHNOOSH HOSSEINLOUI KHALAJ^2^, MD SHAJEDUR RAHMAN SHAWON^2^, LIESL STRACHAN^1^, GABRIELLE CHALLIS^1^, KATE KING^1^, LOUISA JORM^2^, REECE HOLBROOK^3^

#### ^1^Medtronic, Macquarie Park, Australia,^2^Centre for Big Data Research in Health, University of New South Wales, Macquarie Park, Australia,^3^Medtronic, Minneapolis, MN

**Introduction:** Approximately 30,000 cardiac implantable electronic devices (CIED) are implanted annually in Australia. Patients with cardiac resynchronisation therapy‐defibrillators, or a CIED revision or replacement procedure have an increased risk of infection. The Worldwide Randomized Antibiotic Envelope Infection Prevention Trial (WRAP‐IT) demonstrated a 40% reduction of major CIED infection with the use of an absorbable antibacterial envelope in patients at high risk of infection. The objective of this analysis was to determine the cost‐effectiveness of this envelope in a high‐risk patient population in the Australian Public hospital setting.

**Methods:** A published cost‐effectiveness model was adapted with Australian costs and other local inputs derived from real‐world linked hospital admission data from the New South Wales Cardiovascular Cohort (NSWCVC) database. The decision tree model compared the use of an antibacterial envelope versus no envelope over the lifetime of a patient with a high risk of infection as defined in WRAP‐IT. Detailed costs and quality‐adjusted life‐years (QALY) were based on the first 12 months of data from WRAP‐IT, along with lumped estimates for lifetime costs and QALYs.

**Results:** From the NSWCVC database, the patient‐specific infection rate for patients at high risk of infection in an Australian public hospital was 2.1% with an average cost of AUD$102,923. The use of an antibacterial envelope results in a cost saving of AUD$157 at 12 months and an incremental cost of AUD$62 over a lifetime. Incremental QALYs with the envelope was 0.00144 at 12 months and 0.00872 over a lifetime. Both the 12 month and the lifetime cost/QALY gained resulted in an ICER that was “dominant”. That is, the envelope did not result in a significant increased cost over a lifetime, however resulted in increased QALYs.

**Conclusions:** An antibacterial envelope is highly cost‐effective in patients at high risk of infection. Use of the envelope was essentially cost neutral to the Australian Public healthcare system, but increases the quality and length of life of the patient.

## COST‐EFFECTIVENESS OF THE MICRA™ VR LEADLESS PACEMAKER IN PATIENTS WITH BRADYCARDIA AND ATRIAL FIBRILLATION IN AUSTRALIA

### **MICHELLE HILL** ^1^, KOJI MAKINO^2^, MIA MUDGE^2^, CHELSEA ZAUNMAYR^2^, DOMINIC TILDEN^2^

#### ^1^Medtronic, North Ryde, Australia,^2^THEMA, Pyrmont, Australia

**Introduction:** Approximately 4,200 single chamber pacemakers are implanted in Australia per annum. Transvenous pacemakers (TVPMs) are predominantly used and have a long history of use. More recently, a leadless single chamber pacing option has become available (Micra VR). Micra VR is much smaller than a transvenous pacemaker at 25.9mm long and 6.7mm wide and is inserted via the femoral vein and implanted directly into the right ventricle. Absence of leads and a subcutaneous pocket reduces the risk of complications associated with conventional TVPM. The objective of this cost‐utility analysis was to determine the cost‐effectiveness of Micra VR compared to single chamber TVPM for the management of patients with bradycardia with atrial fibrillation (AF).

**Methods:** A Markov model was developed from the perspective of the Australian healthcare system, comparing Micra VR to single chamber TVPM over the device battery life of 17 years. Key data inputs were drawn from the MICRA Coverage with Evidence Development (CED) study which showed Micra VR (n=6,219) had a 31% lower rate of chronic complications and a 38% lower rate of reintervention compared with transvenous patients (n=10,212) at 2 years. Costs were obtained from Australian sources.

**Results:** Over the lifetime of the device, Micra VR had an estimated incremental cost of A$4,277 and yielded 0.09 incremental quality‐adjusted life years (QALYs) when compared with TVPM. The overall incremental cost‐effectiveness ratio of Micra VR was A$47,379 per QALY gain.

**Conclusions:** Micra VR is likely to offer a cost‐effective alternative to conventional single chamber TVPM for the management of Australian patients with bradycardia and AF. As infection is a serious complication, associated with significant mortality and morbidity, a leadless cost‐effective option may be advantageous particularly for patients at high risk of infection or who will have an improved quality of life due to lack of lead restrictions and/or not having a visible reminder of a pacemaker.

## NOVEL ELECTROGRAM‐GUIDED ABLATION DERIVED FROM AUTOMATICITY SITES THAT INDUCES ATRIAL FIBRILLATION

### **SHU HIRATA**, KOICHI NAGASHIMA, RYUTA WATANABE, YUJI WAKAMATSU, MOYURU HIRATA, YASUO OKUMURA

#### Division of Cardiology, Department of Medicine, Nihon University School of Medicine, Tokyo, Japan

**Introduction:** The identification of the AF drivers, particularly non‐PV drivers, requires further elucidation. The first part of our study focused on locating and delineating the electrophysiological features of the PV drivers during AF. In the second part, we attempted additional RF applications targeting the sites with electrograms that exhibited these features, in the LA/RA body, to terminate AF that persisted after PVI.

**Methods:** First Part: In 27 AF patients (11 with paroxysmal AF and 16 with persistent AF), automaticity of PV potentials observed post‐PVI, with or without isolation of the LA posterior wall, was targeted with additional RF ablations at 40 sites. The electrograms recorded during AF before the ablation procedure were analyzed. Second Part: Additional RF energy was applied to sites where electrograms suggested potential AF drivers.

**Results:** First part: During AF, all sites of automatic PV potentials exhibited a relatively high voltage of 1.19±0.52 mV, surrounded by a low voltage area (0.54±0.18 mV; left panel, Figure). In 9 of 20 cases (45%) where the CARTOFINDER module was used, these sites were identified as focal activations. The automatic PV potential sites were also characterized by an average cycle length of 149±20 ms (range 108‐194 ms), with an interval confidence level (ICL) of 13.3±2.3 (range 10‐18), as determined by the CFAE module (right upper panel, Figure). Second part: In patients where AF persisted even after initial ablation, additional RF energy was applied to the LA/RA body at electrogram sites satisfying both criteria: a voltage of >1.2 mV and an ICL within a specific range (50±25, with 8 sites per patient). These additional RF applications successfully terminated AF (n=4) or transitioned it to atrial tachycardia (n=2) in 6 of 19 patients (32%; right lower panel, Figure).

**Conclusions:** AF drivers may be located in areas with high voltage and relatively stable cycle lengths. Additional RF ablation targeting electrograms that exhibit these characteristics could be a potential strategy for ablating AF drivers.

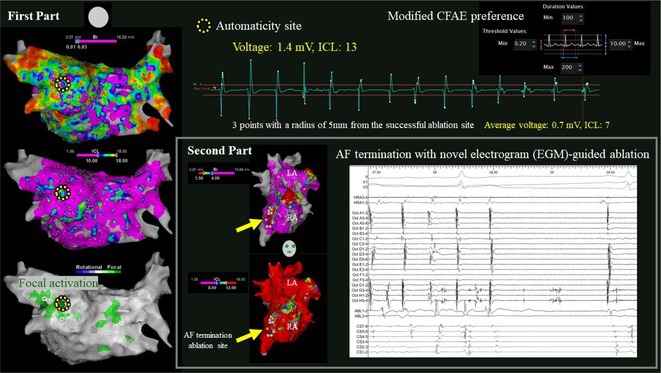

## ABLATION OF THE LEFT ATRIAL ANTRUM IN ADDITION TO PULMONARY VEIN ISOLATION IN CRYOBALLOON ABLATION FOR PAROXYSMAL ATRIAL FIBRILLATION

### **ATSUSHI HIRATSUKA**, TAKATOSHI WAKEYAMA, MASAKAZU TANAKA, NOZOMU HARADA, TAKUMA NANNO, SHINJI TANAKA, TAKAYUKI OKIMURA, TAKUTO NAKATSUKA

#### JCHO Tokuyama Central Hospital, Shunan, Japan

**Introduction:** It is unclear whether additional ablation of the antrum in pulmonary vein isolation improves outcomes in cryoballoon ablation for paroxysmal atrial fibrillation. The aim of this study was to evaluate the efficacy and safety of left atrial antrum ablation in addition to pulmonary vein isolation during cryoballoon ablation for paroxysmal atrial fibrillation.

**Methods:** We retrospectively studied 50 consecutive cases of cryoballoon ablation for paroxysmal atrial fibrillation at our hospital immediately before the introduction of additional antrum ablation (PVI group) and 50 consecutive cases immediately after the introduction of additional antrum ablation (Antrum group). Cases with a common left pulmonary vein or tachyarrhythmias other than atrial fibrillation were excluded.

**Results:** No significant differences in age (PVI vs Antrum, 69.0 vs 71.2 years), CHADS2 score (1.26 vs 1.24), left ventricular ejection fraction (64.6 vs 66.3%), left atrial diameter (38.1 vs 38.3 mm) or observation period (450 vs 385 days) between the two groups. Successful pulmonary vein isolation was achieved in all patients, with an average of 1.9 additional ablations to the antrum in the Antrum group.3D mapping using Ensite showed that although there was no significant difference in preoperative left atrial posterior wall area (18.6 vs 17.0 cm^2^), the post‐ablative left atrial posterior wall isolation area was significantly larger in the Antrum group (3.6 vs 7.6 cm^2^, p<0.01). There was no difference in procedure time, and complications included one case of cardiac tamponade in the PVI group and one case of acute gastric dilatation in the Antrum group; atrial tachyarrhythmia recurrence after the blanking period was not significantly different between the two groups (6 vs 7 cases).

**Conclusions:** In cryoballoon ablation for paroxysmal atrial fibrillation, ablation of the left atrial antrum in addition to pulmonary vein isolation was not associated with outcomes, although it significantly increased the area of left atrial posterior wall isolation without increasing procedure time or complications.

## BEPRIDIL‐INDUCED INTERSTITIAL PNEUMONIA: INCIDENCE AND CLINICAL IMPLICATIONS

### **HIROAKI HIRAYAMA**, MASATO HACHISUKA, MAKOTO KOBAYASHI, SHUHEI OKAJIMA, NOBUAKI ITO, REI MIMURO, SERINA KOBAYASHI, YUHI FUJIMOTO, YOSHIAKI KUBOTA, HIROSHIGE MURATA, YOSHIYASU AIZAWA, KENJI YODOGAWA, WATARU SHIMIZU, KUNIYA ASAI, YU‐KI IWASAKI

#### Nippon Medical School Hospital, Tokyo, Japan

**Introduction:** Bepridil, a multi‐channel blocker has been often prescribed for atrial fibrillation (AF) and ventricular tachyarrhythmias. The incidence rate and risk factors are unclear, but there has been reports of severe interstitial pneumonia (IP) as an adverse effect of bepridil. The purpose of the study was to elucidate the incidence and clinical outcomes of bepridil‐induced IP.

**Methods:** A total of 1,365 consecutive patients (951 men, age 68±12 years) who were prescribed bepridil from 2012 to 2023 at our hospital were retrospectively analyzed. The clinical parameters were compared between patients admitted with bepridil‐induced IP and who did not.

**Results:** Five patients (0.37%) developed severe IP associated with bepridil requiring hospitalization. All of the 5 patients were prescribed bepridil for the treatment of persistent atrial fibrillation, and there were no significant differences in the patient characteristics including age at bepridil prescription initiation (70.0±5.5 years vs. 67.5±11.6 years *P*=0.63), gender (men 100% vs. 69.6% *P*=0.16), and the cumulative dose administered (76.7±52.0g vs. 88.6±61.2 *P*=0.82) compared to the patients without bepridil‐induced IP. The interval between development of IP and initiation of bepridil was 17.0±14.4 months. Four patients received corticosteroid treatment for IP. Three out of 5 patients died 35.7±23.8 months after the initiation of bepridil prescription, with a mean duration of hospitalization to death of 17.4±12.3 months. Remaining survived 2 patients received corticosteroid therapy and discharged in 13 and 25 days, respectively.

**Conclusions:** Our study found no significant clinical parameter differences between patients with bepridil‐induced interstitial pneumonia (IP) and those without. Although the incidence of severe IP was rare (0.37%) in our single center cohort, the mortality rate was high (60%). It is crucial to monitor for potential development of IP following bepridil administration to promptly address this rare, but potentially life‐threatening complication.

## COMPARISON OF OUTCOMES AND SAFETY BETWEEN DIFFERENT CATHETERS FOR ELECTROANATOMIC MAPPING DURING PULSED FIELD ABLATION FOR ATRIAL FIBRILLATION

### **WILBERT HSIEN HAO HO**, JULIAN CHEONG KIAT TAY, GERMAINE JIE MIN LOO, XUANMING PUNG, HOOI KHEE TEO, ERIC TIEN SIANG LIM, DANIEL THUAN TEE CHONG, KAH LENG HO, CHI KEONG CHING

#### National Heart Centre Singapore, Singapore, Singapore

**Introduction:** Pulsed field ablation (PFA) is gaining traction as a technique for pulmonary vein isolation (PVI) for atrial fibrillation (AF). 3D electroanatomic mapping (EAM) may be performed during PVI to improve procedural success. The performance of different catheters for EAM during PVI has not been compared to date.

**Methods:** We enrolled consecutive patients with paroxysmal and persistent AF who underwent PFA using the Boston Scientific Farapulse^TM^ system guided EAM. EAM was performed using one of the following three catheter types: the same Farawave PFA catheter, an additional multipolar high density (HD) catheter, or an additional radiofrequency ablation (RFA) catheter in cases which required additional linear ablations. The primary efficacy endpoint was freedom from AF recurrence up to 1 year. Other outcomes measured were procedural time, radiation exposure and left atrial dwell time. Safety endpoints were 1 year all‐cause mortality and inpatient complications.

**Results:** 94 patients of mean age 60.1 years were included. 69 (73.4%) were male. 29 (30.9%) had structural heart disease. The mean CHADSVASc score was 1.4; 21 (22.3%) had persistent AF. Concurrent cavotricuspid isthmus ablation was performed in 42 (44.7%) and posterior wall isolation in 29 (30.9%). 8 patients (8.5%) experienced AF recurrence. No significant difference was elicited between the three catheter types with regard to AF recurrence (ANOVA p=0.296, log‐rank p=0.236). Procedural time was significantly shorter in the PFA catheter group on both omnibus testing (p<0.001) as well as on post‐hoc comparison with the multipolar HD catheter and RFA catheter separately (p<0.001). Fluoroscopy time (p=0.014) and skin dose (<0.001) were lower compared to the RFA catheter. There was no difference in all‐cause mortality, inpatient complications or left atrial dwell time between the three groups.

**Conclusions:** EAM using the Farawave catheter is safe and shortens procedural time with no difference in AF recurrence rates. Eliminating the need to deploy additional catheters for the specific purpose of EAM may save costs.

## ATRIAL ARRHYTHMIA ABLATION IN A PATIENT POST MITRAL VALVE REPLACEMENT SURGERY AND COX‐MAZE PROCEDURE

### **TRUNG KIEN HOANG**, VAN BA VU, DUC THINH DO, MANH HUNG NGUYEN, TIEN DUNG LE

#### Cardiovascular Center ‐ E Hospital, Hanoi, Viet Nam

**Introduction:** The Cox‐Maze procedure is an effective treatment for valvular atrial fibrillation, but there is still a high incidence of recurrence of atrial arrhythmia.

**Methods:** N/A.

**Results:** A 66‐years‐old female with hypertension and type‐2 diabetes, who underwent cardiac surgery involving mitral valve replacement with a bioprosthesis (Hancook No27), tricuspid valve repair with a size 29 Medtronic annuloplasty ring, and Cox‐Maze IV procedure, presented 14 months later with pneumonia, atrial tachycardia, and left ventricular dysfunction which were intolerant to medical therapy. An electrophysiology study was perform, and we identified focal atrial tachycardia originating from the anterior part of the left inferior pulmonary vein, near the mitral valve; the right pulmonary veins and left atrial posterior wall were isolated after the Cox‐Maze procedure; the left pulmonary veins were re‐connected. After successfully ablating this focal AT and re‐isolating the left pulmonary vein, the rhythm changed to a typical cavotricuspid isthmus‐dependent atrial flutter. Ablation of the CTI terminated the flutter, and the sinus rhythm was restored, with transient junctional rhythm. The symptoms improved after the procedure, the transthoracic echocardiography found an improvement of LV ejection fraction with no bioprosthesis dysfunction. The patient remained in sinus rhythm 3 months post‐procedure.

**Conclusions:** Ablation procedure with electro‐anatomy mapping is feasible, safe and effective for adjunctive ablation of atrial arrhythmia post mitral valve replacement and Cox‐Maze procedure without damage to the bioprosthesis.

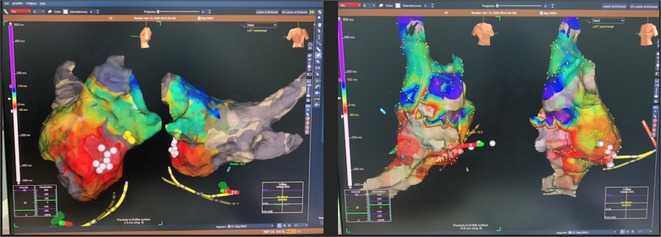

## POTENTIAL IMPLICATIONS OF RECENT ENHANCEMENTS IN LEADLESS PACEMAKER TECHNOLOGY ON DEVICE MANAGEMENT AND HEALTHCARE UTILIZATION: A VIRTUAL PATIENT ANALYSIS

### **MIKAYLE HOLM** ^1^, MIGUEL LEAL^2^, TODD SHELDON^1^, KEELIA ESCALANTE^1^, MICHELLE GALARNEAU^1^, SARAH ROSEMAS^1^, KURT STROMBERG^1^, JONATHAN PICCINI^3^

#### ^1^Medtronic, Mounds View, MN,^2^Emory University Hospital, Atlanta, GA,^3^Duke Clinical Research Institute & Duke University Medical school, Durham, NC

**Introduction:** Leadless ventricular pacemakers have been developed for single chamber VVIR and AV synchronous (AVS) pacing (VDD) applications. We sought to assess the impact of enhancements to the battery/electronics and AVS algorithms of next‐generation Micra leadless pacemakers.

**Methods:** Real‐world pacing data gathered from the Micra IDE study (VR), Medtronic's Carelink database (AV), and historical pacemaker patient survival data from Medtronic's Device Registry were used to project device longevity and estimate the proportion of patients requiring lifetime device replacements. Accelerometer data from the AccelAV study were used to create virtual patients and compare AVS between enhanced vs. original algorithms; a real‐world survey was conducted to characterize clinical time savings from AVS programming burden reduction.

**Results:** With data from 644 patients the median projected longevity of Micra VR was 12.3 years and of Micra VR2 was 16.7 years, with 91% of patients requiring a single VR2 device over their lifetime. Using data from 999 patients, the projected longevity of Micra AV was 10.8 years and Micra AV2 was 15.6 years, with 80% of patients requiring one AV2 device (Fig. 1). The improvements noted with Micra VR2 and AV2 were associated with 8 and 15 fewer projected lifetime device replacements per 100 patients, respectively (9 and 19 in those with >90% ventricular pacing). The enhanced Atrial Sensing Setup in Micra AV2 devices led to >70% AVS in 90% of Micra AV2 devices as well as improved ambulatory AVS of 84% in the 80‐100 bpm range without additional manual programming (Fig. 2). Based on survey results, the enhanced Atrial Sensing Setup algorithm accounted for a time savings of 13 minutes per post‐implant device check.

**Conclusions:** Substantial improvements were projected in device longevity and AVS with next‐generation Micra pacemakers, carrying potential to streamline device follow‐up and resulting in most patients needing only one device over their lifetime.

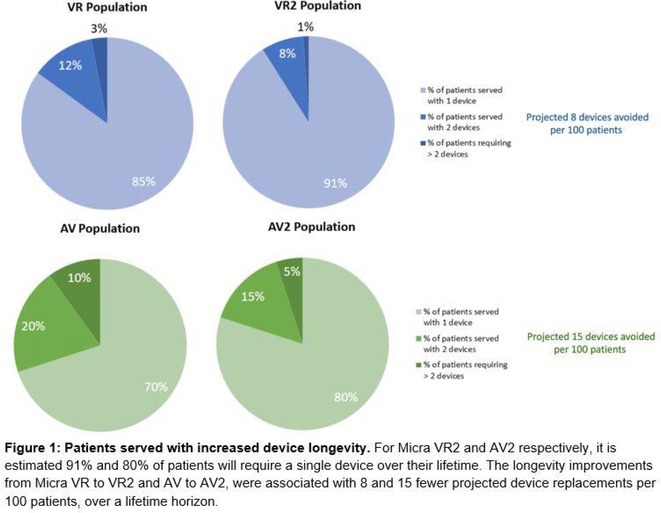

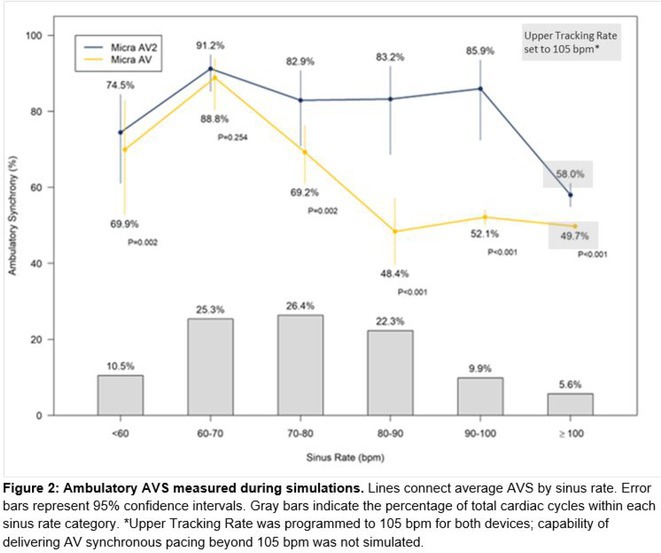

## GLYCEMIC VARIABILITY AND IN‐HOSPITAL DEATH OF CRITICALLY ILL PATIENTS AND THE ROLE OF VENTRICULAR ARRHYTHMIAS

### **KUI HONG** ^1,2,3^, WEIGUO FAN^1^, HONGYU LIU^3^

#### ^1^Department of Cardiovascular Medicine, the Second Affiliated Hospital of Nanchang University, Nanchang, China,^2^Jiangxi Key Laboratory of Molecular Medicine, Nanchang, China,^3^Department of Genetic Medicine, the Second Affiliated Hospital of Nanchang University, Nanchang, China

**Introduction:** Abnormal glycemic variability is common in the intensive care unit (ICU) and is associated with increased in‐hospital mortality and major adverse cardiovascular events, but little is known about whether adverse outcomes are partly mediated by ventricular arrhythmias (VAs). We aimed to explore the association between glycemic variability and VAs in the intensive care unit (ICU) and whether VAs related to glycemic variability mediate the increased risk of in‐hospital death.

**Methods:** We extracted all measurements of blood glucose during the ICU stay from The Medical Information Mart for Intensive Care IV (MIMIC‐IV) database version 2.0. Glycemic variability was expressed by the coefficient of variation (CV), which was calculated by the ratio of standard deviation (SD) and average blood glucose values. The outcomes included the incidence of VA and in‐hospital death. The KHB (Karlson, KB & Holm, A) is a method to analyze the mediation effect for nonlinear models, which was used to decompose the total effect of glycemic variability on in‐hospital death into a direct and VA‐mediated indirect effect.

**Results:** Finally, 17,756 ICU patients with a mean age of 64 years were enrolled; 47.2% of them were male, 64.0% were white, and 17.8% were admitted to the cardiac ICU. The total incidence of VA and in‐hospital death were 10.6% and 12.8%, respectively. In the adjusted logistic model, each unit increase in log‐transformed CV was associated with a 21% increased risk of VA (OR 1.21, 95% CI: 1.11‐1.31) and a 30% increased risk (OR 1.30, 95% CI: 1.20‐1.41) of in‐hospital death. A total of 3.85% of the effect of glycemic variability on in‐hospital death was related to the increased risk of VA.

**Conclusions:** High glycemic variability was an independent risk factor for in‐hospital death in ICU patients, and the effect was caused in part by an increased risk of VA.

## THE GROWTH AND SAFETY AFTER S‐ICD IMPLANTATION IN PATIENTS UNDER 12 YEARS‐OLD

### **JUNYA HOSODA**, TOSHIYUKI ISHIKAWA, YUKA TAGUCHI, MASATOSHI NARIKAWA, YOSHINORI OKAZAKI, SHUICHI MIYAGAWA, AKIRA HORIGOME, SATOSHI ISHII, YU YAMADA, KIYOSHI HIBI

#### Yokohama City University Hospital, Yokohama, Japan

**Introduction:** There are few reports on subcutaneous implantable cardioverter defibrillator (S‐ICD) implantation in pediatric field, and there are no studies regarding growth and safety in the distant periods. We analyzed the growth, device position, and ventricular amplitude in three S‐ICD cases under 12 years‐old.

**Methods:** Case 1 is a 11 years‐old boy (height: 145 cm, weight: 32 kg), who was suffering from ventricular fibrillation (VF) due to congenital long QT syndrome (LQT). Case 2 is a 7 years‐old boy (130 cm, 27 kg) with VF due to hypertrophic cardiomyopathy. Case 3 is a 10 years‐old girl (142 cm, 41 kg) with VF due to LQT. In all cases, S‐ICD was implanted using the two‐incision method.

**Results:** During a mean follow‐up period of 33 months, their height increased by 25 cm, 21cm, and 13 cm, respectively. Although, chest X‐rays showed that pulse generators and leads moved slightly downward, the ventricular amplitude in the suitable sensing vector at the final follow‐up increased by 0.2 mV, 0.8 mV, and 1.2mV, respectively. During the follow‐up, a total of four appropriate shocks were observed in two patients. In all episodes, VF stopped after the first shock therapy. There were no inappropriate shocks and lead‐related complications in three cases.

**Conclusions:** S‐ICD implantation in patients under 12 years‐old considered to be safe and useful.

## THE ASSOCIATION OF AGE AND LEFT ATRIAL DYSFUNCTION IN PATIENTS WITH ATRIAL FIBRILLATION

### **JACKSON HOWIE** ^1,2,3^, JENELLE DZIANO^1,2,3^, JONATHAN ARIYARATNAM^1,2,3,4^, MELISSA MIDDELDORP^1,2^, MEHRDAD EMAMI^1,2,3,4^, RICARDO MISHIMA^1^, PRASHANTHAN SANDERS^1,2,3,4^, ADRIAN ELLIOTT^1,2,3^

#### ^1^University of Adelaide, Adelaide, Australia,^2^Centre for Heart Rhythm Disorders, Adelaide, Australia,^3^SAHMRI, Adelaide, Australia,^4^Royal Adelaide Hospital, Adelaide, Australia

**Introduction:** Advancing age is a well‐established risk factor for the development of atrial fibrillation (AF). Atrial fibrillation has previously demonstrated its impact on electrical remodelling of the left atrium (LA). However, less is known about the haemodynamic and functional effects of age. Therefore, the aim of this study is to evaluate the association of aging on LA structural and functional remodelling in symptomatic AF patients.

**Methods:** Consecutive patients with symptomatic paroxysmal or persistent AF undergoing AF ablation were recruited. Invasive measures of LA remodelling included mean LA pressure (LAP) and direct assessment of LA stiffness obtained from saline infusion at the time of ablation. Non‐invasive transthoracic echocardiographic measures of function included LA reservoir strain, and LA minimum and maximum volumes. In addition, NT‐ProBNP was collected from venous blood samples. The association of age and LA features were determined using multivariable linear regression models adjusted for sex.

**Results:** In total, 125 participants (72% male, mean age 63±11 years) were recruited. In adjusted analyses, advancing age was positively associated with increased mean LAP (p=0.044) and LA stiffness (p<0.001) from invasive measures. Age was also associated with lower LA reservoir strain (p<0.001) and increased LA minimum (p<0.001) and LA maximum (p=0.001) volumes. We also observed a positive association between age and NT‐ProBNP (p=0.001).

**Conclusions:** In symptomatic AF patients, advanced ageing was associated with more advanced LA functional and haemodynamic impairment. These findings may have implications for the ongoing management of AF, and the development of comorbidities including heart failure.

## CASE REPORT: ACUTE LEAD IMPEDANCE CHANGES IN LEFT BUNDLE BRANCH AREA PACING WITH ATRIOVENTRICULAR JUNCTION ABLATION

### **RACHEL HOYLE** ^1^, KHANG LI LOOI^1^, JAMIE VOSS^2^

#### ^1^Auckland City Hospital, Auckland, New Zealand,^2^Middlmore Hospital, Auckland, New Zealand

**Introduction:** The combination of a permanent pacemaker with atrioventricular junction (AVJ) ablation has shown efficacy in patients with chronic atrial fibrillation (AF) when pharmacological rate control is not achieved. Although, left bundle branch area pacing (LBBAP) has emerged as a promising physiological pacing modality for reducing the risk of pacing‐induced cardiomyopathy, research considering acute lead impedance changes in patients having an AVJ ablation is lacking.

**Methods:** N/A

**Results:** Here, we present the case of a 76‐year‐old female with a history of bronchiectasis and persistent AF with associated cardiomyopathy. Despite optimal medical therapy, she continued to have AF with fast ventricular rates that were difficult to control. AVJ ablation was accepted by her to achieve better rate control. During the AVJ ablation the initial lesion was performed in close proximity to the LBBAP lead (Figure 1). Acute lead impedance changes were observed in the both the LBBAP lead and in the unipolar configuration (LV1tip‐Can) of the inactive left ventricular (LV) lead (Table 1). The ablation was performed using an irrigated catheter at 40watts, with a total radiofrequency (RF) time of 15 minutes. Although the lead impedances measured during the course of the procedure were variable, they improved towards a near normal value at the end of the procedure and had normalised 1 month post procedure. There were no consequences to the patient with the observed lead impedance changes.

**Conclusions:** Acute lead impedance changes can be seen with RF ablation of the AVJ without any chronic implications to lead function.

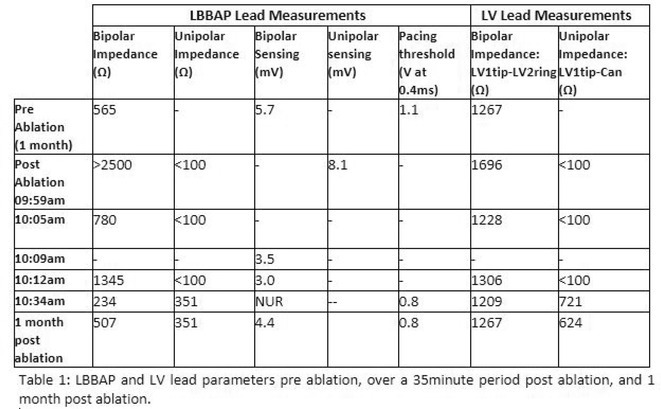

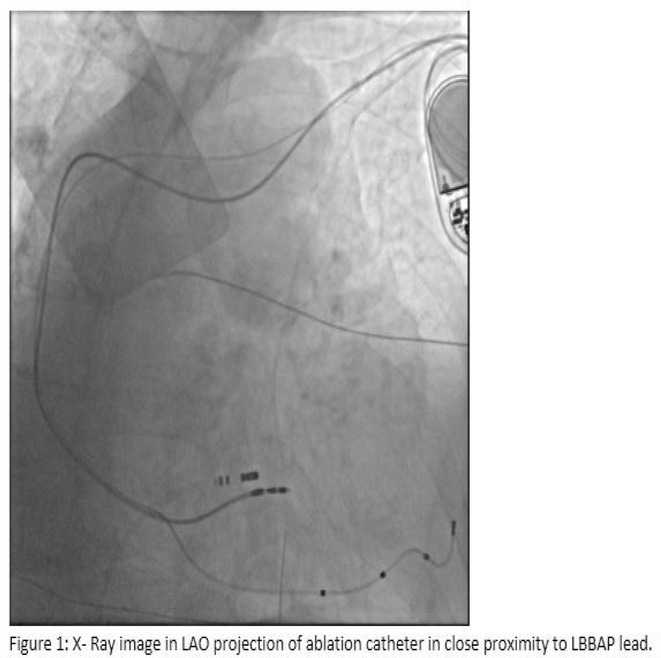

## CARDIAC TAMPONADE HAPPENED ONE WEEK AFTER IMPLANTATION OF A PERMANENT PACEMAKER AT LEFT BUNDLE BRANCH AREA: A CASE REPORT

### **MING‐HSIUNG HSIEH** ^1^, SSU‐YU CHEN^2^, JIE‐YWI ONG^1^, CHYE GEN CHIN^1^, JONG‐SHIUAN YEH^1^

#### ^1^Wan Fang Hospital, Taipei, Taiwan,^2^University of New South Wales, Sydney, Australia

**Introduction:** N

**Methods:** N

**Results:** A 70‐year‐old female underwent permanent pacemaker (PPM) implantation on a background of tachycardia (paroxysmal atrial fibrillation)‐bradycardia syndrome causing multiple episodes of syncope. The ventricular lead was successfully placed at the left bundle branch area (LBBA); however, lead perforation was suspected during the attempt. The patient was discharged two days later with stable vitals and normal CXR and ECG findings. Amiodarone was replaced with Dronedarone due to poor AF control, and rivaroxaban was continued. She was followed up five days after discharge at the outpatient clinic without any symptoms. The patient represented to the emergency department (ED) the day after her follow‐up appointment due to syncope. In ED, she was conscious with marked hypotension (blood pressure 57/41 mmHg) and tachypnea (RR 26/min). Her blood test revealed normocytic anemia (Hemoglobin 9.6 g/dl), elevated INR (2.3) and aPTT (45.8 seconds). CT scan revealed a substantial amount of pericardial effusion and bilateral pleural effusion, which is highly suggestive of cardiac tamponade. Therefore, pericardiocentesis was initiated and 500ml of bloody pericardial fluid was drained. Consequently, the patient vital signs improved; however, poor saturation and dyspnea persisted at rest. Thoracocentesis was then performed with 220ml of bloody fluid obtained suggesting hemothorax. Rivaroxaban was ceased and dronedarone was replaced by amiodarone during this admission. The patient became hemodynamically stable and was discharged 10 days later. Initially, cardiac tamponade was suspected secondary to lead perforation during the PPM procedure in addition to the use of rivaroxaban. Despite recommendations for continuing lead placement at LBBA after lead perforation, little data suggest safety concerns of coagulopathy from long‐term anticoagulant use after lead perforation. Later, coagulopathy induced simultaneous hemopericardium and hemothorax was considered, and drug‐drug interaction between the combination of rivaroxaban and dronedarone causing coagulopathy was suspected.

**Conclusions:** N

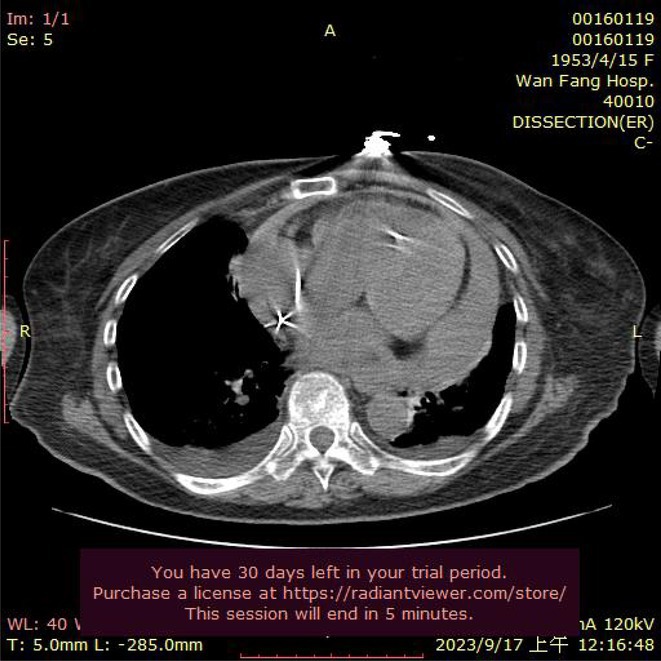

## INSULIN RESISTANCE AS A NOVEL PREDICTOR FOR ATRIAL FIBRILLATION RECURRENCE AND CARDIOVASCULAR HOSPITALIZATIONS AFTER CRYOBALLOON ABLATION FOR ATRIAL FIBRILLATION

### **YU‐CHENG HSIEH**, YU‐SHAN CHIEN, SHANG‐JU WU, CHIA‐LIN LEE, MING‐JEN KUO, YU‐YU HSIAO, CHENG‐HUNG LI, JIUNN‐CHERNG LIN, JIN‐LONG HUANG, SHIH‐ANN CHEN

#### Taichung Veterans General Hospital and Chiayi Branch, Taichung, Taiwan

**Introduction:** Insulin resistance (IR) has been identified as a risk factor for atrial fibrillation (AF). Our study aimed to assess whether IR, as determined by our previously published AI model that does not require fasting insulin for calculation, serves as an independent predictor for AF recurrence following cryoballoon ablation.

**Methods:** In this retrospective single‐center cohort study, we included patients who underwent cryoballoon ablation for AF at our hospital from 2014 to 2023. Patients with pre‐existing diabetes mellitus were excluded. IR was computed using our AI model, and the patients were stratified into two groups based on the presence or absence of IR. A multivariate Cox hazards regression model was used to evaluate the hazard ratio (HR) for AF recurrence.

**Results:** A total of 126 patients (aged 60.1±1.0 years) with AF receiving cryoballoon ablation for AF were included. Using our AI model, 17 met the criteria for IR and constituted the IR group, while the others were the non‐IR group (n=109). Over an average follow‐up period of 3.02±2.3 years post‐ablation, 43 (34.4%) patients experienced AF recurrence. After adjusting for age, gender, and Charlson comorbidity index, the average left atrium (LA) diameter was higher by 4.02 mm in the IR group comparing to the non‐IR group (p=0.021). The size of LA was positively correlated with the degree of IR risks (r=0.262, p=0.003). AF recurrence was more prevalent in patients with persistent AF (p=0.035). IR significantly increased the risk of AF recurrence in those with a LA diameter of <4.0 mm (p=0.044). IR patients also have elevated the risk for cardiovascular hospitalization (adjusted HR: 3.351, p=0.045).

**Conclusions:** IR is associated with an increased risk of AF recurrence after cryoballoon ablation for AF, particularly in individuals who have not yet developed LAE. In addition to established risk factors such as persistent AF and LAE, IR emerges as a potential novel predictor for AF recurrence and future cardiovascular hospitalizations in patients receiving cryoballoon ablation for AF.

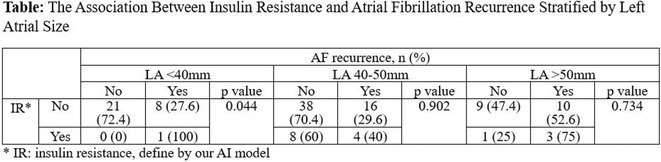

## WEARABLE, WIRELESS, NON‐CONTACT BIOMEDICAL EDDY CURRENT SENSOR FOR CARDIORESPIRATORY MONITORING

### **DONG‐YU HSU**, YEN‐LING SUNG, SHIH‐HUA NI, CHIU‐YUN HUANG, YU‐XIANG HUANG, EN‐ZHU LYU, CHUN‐HSIEN CHEN, TING‐WEI WANG

#### National Tsing Hua University, Hsinchu City, Taiwan

**Introduction:** Cardiopulmonary diseases are a predominant cause of death globally, highlighting the importance of monitoring cardiopulmonary health within public health frameworks. Cardiorespiratory coordination (CRC) is a critical indicator of cardiovascular and pulmonary coordination status. Wearable technologies enable real‐time health monitoring; however, Electrocardiography (ECG) patches and chest‐strap respiratory sensors often compromise on durability and comfort over extended periods.

**Methods:** This study presents a pocket‐sized wearable device that measures cardiac and lung activities without skin contact. It utilizes magnetic coupling between the coil and the cardiopulmonary area. The device is characterized by a compact structure with diameter of 4.53 cm, which incorporate LC tank, inductance‐to‐digital converter ASIC, and microcontroller Unit (MCU) with Bluetooth module. It provides real‐time signal visualization and analysis of heart rate (HR) and respiratory rate (RR) using fast Fourier transform (FFT). Integration of these datasets enables real‐time calculation of pulse‐respiration quotient (PRQ) for CRC monitoring.

**Results:** To demonstrate the functionality of this device for cardiopulmonary measurements, The synchronous measurements using proposed device along with commercial ECG sensors and chest‐strap respiratory sensors. The results showed that the proposed device measured the consistent heart and breathing rhythm in time‐domain analysis among two commercially available devices and confirmed its HR and RR accuracy by FFT analysis. Furthermore, we identified the resonance frequency with the highest sensitivity within the coil's bandwidth to obtain optimal sensing conditions for personalized measurement. In practical applications for CRC, this device has successfully demonstrated the changes in PRQ of subjects before and after exercise.

**Conclusions:** This study provides a solution for comfortable, long‐term monitoring, enabling CRC monitoring from synchronous HR and RR measurement, offering comprehensive cardiopulmonary health monitoring.

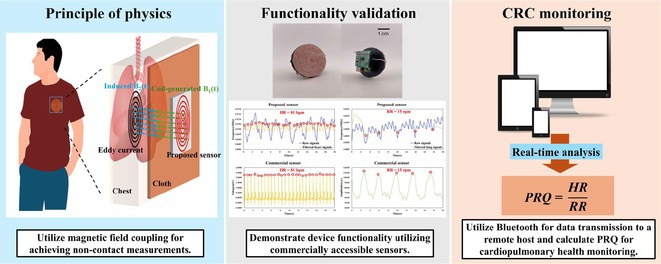

## ENHANCED ENDOTHELIALIZATION USING RESVERATROL‐LOADED POLYLACTIC‐ACID COATED LEFT ATRIAL APPENDAGE OCCLUDERS IN A CANINE MODEL

### **LIXING HU**, KUO ZHANG, HONGDA ZHANG, MIN TANG

#### Center of Arrhythmia, Fuwai Hospital, National Center for Cardiovascular Diseases, Chinese Academy of Medical Sciences, Peking Union Medical College, Beijing, China

**Introduction:** Left atrial appendage occluder (LAAO) is a well‐established alternative to anticoagulation therapy for atrial fibrillation patients with high bleeding risk. Post implantation, anticoagulation therapy is required for at least 45 days until complete LAA occlusion by neoendocardium coverage of the device. Herein, we applied polylactic acid‐resveratrol coating on the LAAO membrane to enhance endothelialization, with the goal of possibly shortening anticoagulation duration.

**Methods:** Eighteen dogs were randomly assigned into the experimental or control groups receiving coated and uncoated occluders, respectively. They were sacrificed in cohorts at day 14, 28, and 90 for anatomical observation and pathological examination to evaluate endothelialization and thrombus formation. Trans‐esophageal echocardiography (TEE) was performed before sacrifice to evaluate device related adverse events.

**Results:** According to the anatomical and pathological examination, all LAAO covers except one exhibited larger or thicker tissue or neo‐endocardium coverage in all the experimental groups than the control groups at the same sacrifice time. All connection hubs were densely covered by endothelial cells at 90 days and completely covered at 28 days in the experimental group, while all connection hubs were thinly covered at 90 days and 2 connection hubs were exposed at 28 days in the control group. In addition, the pathological examination revealed no thrombus formation in the experimental group, while a small amount of thrombus was observed in one dog at 90 days and two dogs at 28 days in the control group. Finally, TEE showed no peri‐device leakage (PDL) in the experimental group, whereas a small amount of PDL was detected in one dog (2mm) at 28 days and one dog (3mm) at 14 days in the control group.

**Conclusions:** The resveratrol loaded polylactic acid covered LAAO enhances endothelialization, potentially reducing thrombus formation and PDL. This effect could lead to a possible reduction in the duration of anticoagulation therapy.

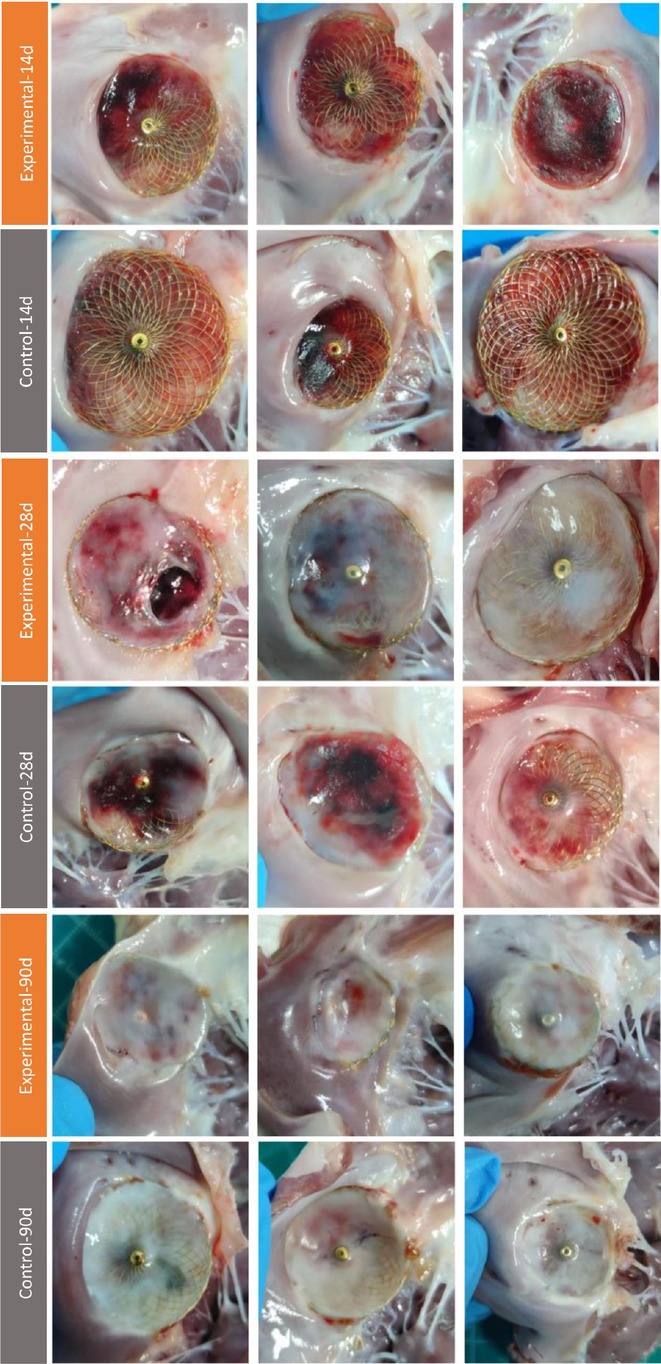

## LESS VENTRICULAR ARRHYTHMIA AND DEATH IN THE EARLY DIAGNOSIS OF ADPKD WITH CKD STAGE 3 THAN THE OTHER CKD STAGE 3

### HSIN‐TI HUANG

#### Taichung Veterans General Hospital, Taichung, Taiwan

**Introduction:** Autosomal dominant polycystic kidney disease (ADPKD) is the most frequent hereditary renal disease. It is still unknown about the cardiovascular disease (CVD) of ADPKD in the early‐stage (stage 3) chronic kidney disease (CKD). In this study, we evaluate the CVD between ADPKD and CKD from other etiologies (CKDoe) after 5 years follow‐up.

**Methods:** The data used in this study was collected from the TriNetX Global Research Network, which provides access to EHR for approximately 90 million patients from healthcare organizations, predominantly within the United States. Data displayed on the TriNetX Platform in aggregate form, or any patient‐level data provided in a data set generated by the Tri‐NetX Platform. ADPKD without Tolvaptan, (CKD stage 3) n=13734 and CKDoe (stage 3) n=13734 after propensity score matching (PSM).

**Results:** During 5‐year follow‐up period. The ADPKD has less death ( OR 0.7, 95% CI 0.65‐0.75), ventricular arrhythmia(OR 0.72, 95% CI 0.61‐0.85), atrial fibrillation (OR 0.89, 95% CI 0.81‐ 0.98), heart failure (OR 0.72, 95% CI 0.66‐ 0.78), stroke (OR 0.72, 95% CI 0.66‐0.78), hyperlipidemia (OR 0.84, 95% CI 0.78‐0.91), Diabetes mellitus (DM) (OR 0.88, 95% CI 0.80‐0.96).

**Conclusions:** This is the first real‐world report of the early diagnosis of ADPKD CKD stage 3 associated with less DM, hyperlipidemia, arrhythmia, heart failure and death than CKDoe (stage 3). Further studies are needed to study the underlying mechanisms.

## REAL‐TIME MONITORING OF AIR QUALITY INDEX PREDICTS THE IMPACT OF AIR POLLUTION ON AUTONOMIC MODULATION: INSIGHTS FROM HEART RATE VARIABILITY ANALYSIS

### **YU SHAN HUANG** ^1^, LI WEI LO^1^, YENN JIANG LIN^1^, SHIH‐LIN CHANG^1^, YU FENG HU^1^, FA PO CHUNG^1^, CHIN YU LIN^1^, TING YUNG CHANG^1^, LING KUO^1^, CHIH MIN LIU^1^, SHIN HUEI LIU^1^, SHIH ANN CHEN^2^

#### ^1^Taipei veterans general hospital, Taipei, Taiwan,^2^Taichung veterans general hospital, Taichung, Taiwan

**Introduction:** Air pollution, a proven environmental risk for increased cardiovascular events, lacks exploration into its mechanisms and impact across various daily time periods. We aimed to use the Air Quality Index (AQI), representing mixed air pollutant exposure, to assess the impact of air pollution on cardiac autonomics through heart rate variability (HRV) analysis across different time intervals.

**Methods:** We analyzed 6,912 patients (Oct 2015 ‐ Oct 2016) using Holter ECG and environmental data (PM2.5, PM10, O3, NO2, SO2, CO) to calculate AQI from Taiwan's Environmental Protection Administration. In Group 1 (n=24) with AQI 101‐200, and Group 2 (n=23) with AQI 0‐100, we explored HRV parameters'correlation with AQI for the past day, two days prior, and the three‐day average.

**Results:** There were significantly lower high frequency (HF) in Group 1 than Group 2 across 24 hours (p=0.019), 9 am to 9 pm (p=0.047), and 4 pm to 12 pm (p=0.036). Group 1 exhibited higher ratios of low frequency and high frequency (LF/HF) than Group 2 during 12 pm to 8 am (p < 0.001) and in various daytimeslots (9 am to 9 pm, p = 0.003; 8 am to 4 pm, p=0.001; 4 pm to 12 pm, p=0.031). The mean standard deviation of the NN intervals (SDNN) decline in Group 1 vs. Group 2 was significant during 9 am to 9 pm (p=0.030) and 8 am to 4 pm (p=0.037). Within Group 1, SDNN was significantly lower from 9 am to 9 pm (90.85 ms) compared to 9 pm to 9 am (114.31 ms, p=0.024) and 4 pm to 12 pm (85.89 ms) compared to 12 pm to 8 am (123.20 ms, p=0.027) (Table). No correlations were found between HRV parameters and AQI in different lags.

**Conclusions:** This study linked HRV LF/HF ratios to air pollutant exposure, indicating a significant daytime LF decrease. Mean SDNN also decreased during the daytime, suggesting a pronounced impact on cardiac autonomics, resulting in reduced HRV and sympathetic‐parasympathetic imbalance from combined air pollutant exposure.

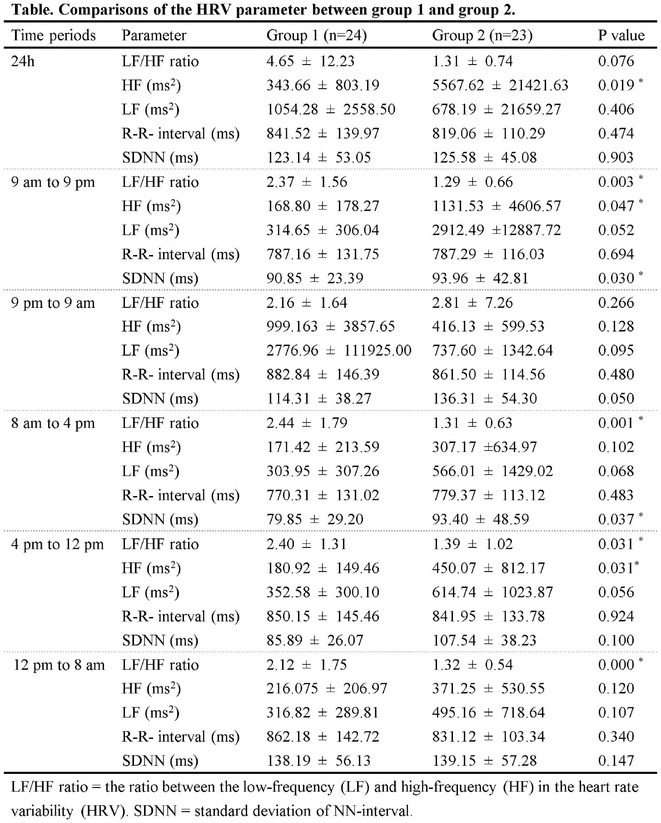

## EVALUATING SOLUBLE ST2 BIOMARKER FOR PREDICTING ATRIAL FIBRILLATION RECURRENCE AFTER ELECTRICAL CARDIOVERSION

### **KI WON HWANG** ^1^, JIN HEE CHOI^1^, HYUNG OH CHOI^2^

#### ^1^Pusan National University Yangsan Hospital, Yangsan, Korea, Republic of,^2^Soonchunhyang University Hospital, Bucheon‐si, Korea, Republic of

**Introduction:** Atrial fibrillation (AF) is a prevalent arrhythmias with high recurrence rates following electrical cardioversion. Identifying biomarkers that can predict recurrence is important for optimizing patients management. This study evaluates the role of soluble ST2 (sST2) as a predictor of AF recurrence.

**Methods:** Among 101 patients who underwent electrical cardioversion between 2022 and 2023, sST2 levels were measured in 33 patients on the day of electrical cardioversion. The primary endpoint was the failure of sinus rhythm maintenance at 6 months. Baseline characteristics and sST2 level were collected.

**Results:** A total of 14 patients (42.4%) experienced AF recurrence. The mean sST2 level was higher in patients with AF recurrence (26.7 ± 9.4 ng/mL vs. 25.5 ± 10.0 ng/mL respectively), but the difference was not statistically significant (*P* =0.735). Total energy requirements for cardioversion were significantly higher in patients with AF recurrence than those without AF recurrence (260.7 J ± 153.4 vs. 155.3 J ± 59.8, respectively; *P* = 0.027). Receiver operating characteristic curve for sST2 levels identified 19.124 ng/mL as the optimal cut‐off value to predict AF recurrence, with a sensitivity and specificity of 36.8% and 85.7%, respectively.

**Conclusions:** sST2 levels were higher in patients with AF recurrence compared to those without AF recurrence, though statistical significance was not reached. Further studies with larger sample sizes are needed to confirm these findings.

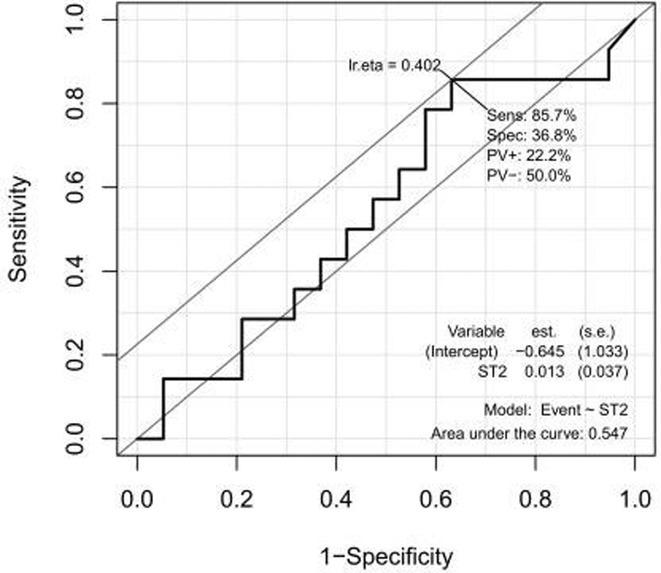

## ASSESSING THE CLINICAL EFFICACY OF VIRTUAL AMIODARONE TESTING GUIDED BY DIGITAL TWIN TECHNOLOGY IN PATIENTS AFTER ATRIAL FIBRILLATION CATHETER ABLATION

### **TAEHYUN HWANG** ^1^, BYOUNGHYUN LIM^1^, OH‐SEOK KWON^1^, MOON‐HYUN KIM^2^, DAEHOON KIM^1^, JE‐WOOK PARK^2^, HEE TAE YU^1^, TAE‐HOON KIM^1^, JAE‐SUN UHM^1^, BOYOUNG JOUNG^1^, MOON‐HYOUNG LEE^1^, CHUN HWANG^1^, HUI‐NAM PAK^1^

#### ^1^Yonsei University College of Medicine, Seoul, Korea, Republic of,^2^Yongin Severance Hospital, Yongin, Korea, Republic of

**Introduction:** We aimed to develop a predictive tool using the digital twin technique to assess amiodarone (AMD) efficacy in high‐risk atrial fibrillation (AF) recurrence after catheter ablation (AFCA).

**Methods:** This single‐center retrospective clinical study included 115 patients (27.0% female, 60.8±10.0 years of age, 26.1% paroxysmal AF) prescribed AMD within 3 months after AFCA and regular rhythm follow‐up for a year. Using a high‐density electro‐anatomical map‐integrated digital twin, we performed virtual pulmonary vein isolations and induced virtual AF under three concentrations of AMD (simulating 0μM, 1.6μM, and 3.9μM). We analyzed electrophysiologic parameters and classified the patient group as the effective (virtual AF termination within 32‐sec in at least one concentration) and ineffective (sustaining virtual AF or atrial tachycardia [AT] in all concentrations) groups. We compared 1‐year clinical AF or AT recurrence rates after the amiodarone prescription date.

**Results:** Out of 345 simulations (115 each in baseline, low and high dose AMD groups), virtual AMD terminated AF in 12.2% (28/230) and was effective in 20.9% (24/115). Compared with baseline AF, virtual AMD prolonged action potential duration (APD_90_, p<0.001) and reduced the peak upstroke velocity (dV/dt_max_, p<0.001) with dose dependent manners. Low‐dose AMD increased the maximal slope of action potential duration (Smax, p<0.001 vs. baseline), but high‐dose AMD reduced Smax significantly (p=0.016 vs. baseline). Mean Smax was significantly lower in the termination cases than in maintained AF/AT cases (p=0.006). One‐year clinical AF/AT recurrence rate was significantly lower in the virtual AMD effective group than ineffective group (20.8% vs. 45.1%, Log‐rank p=0.031, adjusted HR 0.37 [0.14‐0.98]; p=0.046) with clinically predictive value (Harrel's C‐index of model 0.70 [0.66‐0.74]).

**Conclusions:** The digital twin‐guided virtual AMD test has a predictive value of clinical AMD effectiveness among patients who underwent AFCA.

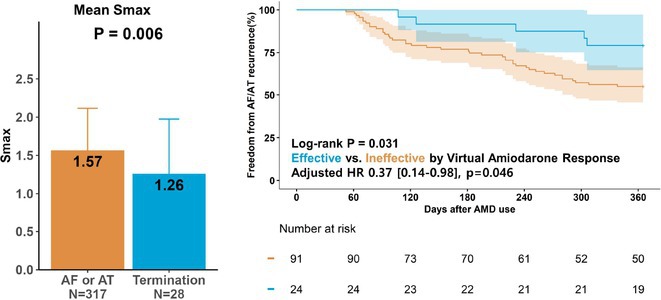

## THE COMPARISON OF LEFT ATRIAL APPENDAGE FILLING DEFECT BETWEEN SUPINE AND PRONE‐POSITION CONTRAST COMPUTED TOMOGRAPHY IN PATIENTS WITH ATRIAL FIBRILLATION

### HIROTSUGU IKEWAKI

#### Kyorin University, Tokyo, Japan

**Introduction:** Left appendage filling defects (LAAFD) revealed by supine‐position contrast computed tomography (sCT) are caused not only by thrombi but also disturbance of LAA blood flow. Prone‐position contrast CT (pCT) is a useful tool to detect LAA thrombi with high sensitivity and specificity. We evaluated the difference between these two manners of CT in patients with atrial fibrillation (AF).

**Methods:** A total of 113 contrast CT was performed to evaluate intracardiac thrombi in patients with AF. 55 patients underwent pCT, otherwise 58 patients underwent sCT.

**Results:** The LAAFD was significantly less revealed by pCT than sCT (1.9% [1/55] vs. 19.0% [11/58], p<0.005). There was no significant difference in LAA emptying flow velocity (LAAEFV) measured by transesophageal echocardiography (38.5±21.0 vs. 36.2±16.3 cm/s, p=0.71, respectively). There was also no significant difference in all common clinical factors including age, body mass index, CHADS2 score, LA diameter, left ventricular ejection fraction but serum creatinine level and NTproBNP level was significantly lower in patients underwent pCT than sCT (0.87±0.17 vs. 0.95±0.19 mg/dl, p=0.02, 400±427 vs. 722±953 pg/ml, p=0.03, respectively). Although LAAFD was detected in 80% (8/10) of patients with LAAEFV less than 30 cm/s by sCT, no patients (0/10) in pCT with low LAAEFV had LAAFD (p<0.0005). Patients with residual contrast in LAA on late phase of pCT are likely to have lower LAAEFV than those without even though there was no significant difference (27.7±5.9 vs. 43.0±23.5 cm/s, p=0.18, respectively).

**Conclusions:** The LAAFD of CT caused by disturbance of LAA blood flow was significantly reduced by change the patients position from supine to prone.

## INVESTIGATION OF PREDICTORS FOR CRT UPGRADE IN PATIENTS WITH A PACEMAKER

### **YUKITOSHI IKEYA**, TOSHIKO NAKAI, RIKITAKE KOGAWA, YUJI WAKAMATSU, RYUTA WATANABE, KOICHI NAGASHIMA, YASUO OKUMURA

#### Nihon University Itabashi Hospital, Tokyo, Japan

**Introduction:** Among patients with a pacemaker, cardiac function deteriorates over time and may require upgrading to cardiac resynchronization therapy (CRT). However, predictive factors, which patient will develop heart failure (HF) and needs upgrading to CRT, remains unclear. In this study, we investigated predictors for the development of HF in patients with pacemaker and pacing dependent.

**Methods:** Twenty‐nine patients, aged 73±10 years, Male 13, were studied. Of those 14 patients (CRT group) who developed decreased cardiac function after pacemaker implantation and were upgraded to CRT and 15 patients who were dependent on right ventricular (RV) pacing after pacemaker implantation but remained without developing HF for more than 5 years (non‐CRT group). Baseline clinical characteristics and echocardiographic parameters including left atrial diameter (LAD), left ventricular diameter (LVD), left ventricular ejection fraction (LVEF), were compared between two groups.

**Results:** There were no significant differences in age, gender, or, QRS duration after RV pacing between CRT group and non‐CRT group. Echocardiographic findings showed not only decreased LVEF (p=0.0417) associated with a tendency towards heart failure, but also a significantly enlarged LAD (p=0.0242).

**Conclusions:** Among patients with RV pacing dependent, LAD was significantly enlarged in patients who required CRT, suggesting that it may be a predictor for CRT upgrade.

## IMPACT OF PIRFENIDONE ON ARRHYTHMIC AND CLINICAL OUTCOMES IN PATIENTS WITH IDIOPATHIC PULMONARY FIBROSIS

### SUNG IL IM

#### kosin university gospel hospital, busan, Korea, Republic of

**Introduction:** Atrial fibrosis is an important substrate in atrial fibrillation (AF), particularly in the setting of structural heart disease. Previous study reported that pirfenidone, an antifibrotic agent, was beneficial at reducing myocardial fibrosis. The objective of the present study is to evaluate the effects of pirfenidone on arrhythmic and clinical outcomes in patients with idiopathic pulmonary fibrosis (IPF).

**Methods:** Our database of patients diagnosed with IPF from 2008 to 2023 was used to obtain echocardiography, electrocardiogram (ECG), and 24hr‐holter monitoring data. Inclusion criteria were all IPF patients with/without Pirfenidone. And we compared arrhythmic events including atrial fibrillation, atrial premature complex, atrial tachycardia, ventricular arrhythmia and clinical outcomes according to use of Pirfenidone.

**Results:** Among 257 patients with IPF (74.0±8.9 years), 106 (42.1%) patients took Pirfenidone. Difference in the baseline characteristics was not observed. During the median 36‐month follow‐up, a lower incidence of arrhythmic events (P=0.001) and diastolic dysfunction (P=0.025) was observed in the Pirfenidone group. In univariate analysis, hypertension, coronary artery disease, higher body weight, longer PR interval, QRS duration, and less use of Pirfenidone were associated with arrhythmic events. In multivariate analysis, higher body weight and less Pirfenidone use were independent risk factors for arrhythmic events.

**Conclusions:** The use of Pirfenidone was associated with less arrhythmic events and lower diastolic dysfunction compared with no use of Pirfenidone in the long‐term follow‐up. And obesity was associated with higher incidence of arrhythmic events in IPF patients.

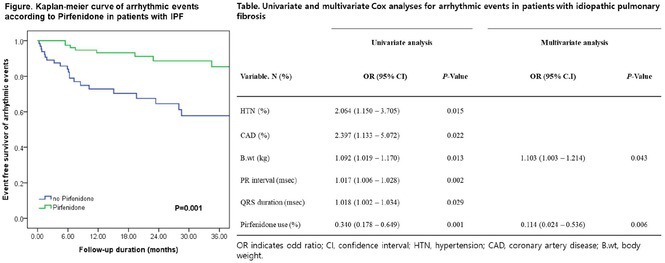

## IMPACT OF REAL‐WORLD OFF‐LABEL DOSE OF APIXABAN ON LONG‐TERM CLINICAL OUTCOMES IN PATIENTS WITH ATRIAL FIBRILLATION AND CHRONIC KIDNEY DISEASE

### SUNG IL IM

#### kosin univ gospel hospital, Division of Cardiology, busan, Korea, Republic of

**Introduction:** The choices of Apixaban dose guided by the decision‐making process of the physician, which considers specific according to kidney functions are inconsistent in the real‐world practice. the aim of our study is to evaluate the effects of real‐world Off‐label dose of Apixaban on long‐term clinical outcomes in nonvalvular atrial fibrillation (NVAF) patients with chronic kidney disease (CKD)

**Methods:** Our database of AF patients diagnosed with CKD from 2018 to 2023 was used to obtain laboratory, echocardiography, electrocardiogram (ECG), and clinical outcomes data. Inclusion criteria were all AF patients with CKD using Apixaban. And we compared bleeding, systemic thrombotic events including stroke/systemic embolism and death according to off‐label real ‐world dose of Apixaban

**Results:** Among 635 patients with AF and CKD (76.9±10.4 years), 335 (52.8%) patients took off‐label underdosed Apixaban. Difference in the baseline characteristics was not observed among patients including CHA_2_DS_2_ VASc score and HASBLED score. During the median 18‐month follow‐up, a lower incidence of bleeding events (P=0.001) was observed in the off‐label underdosed Apixaban group compared to those with standard dose or off‐label overdosed Apixaban. However, there was no significant difference of systemic thrombotic events including stroke/systemic embolism and death according to off‐label real‐world dose of Apixaban (off‐label underdose vs. standard dose, P=1.000; off‐label overdose vs. standard dose, P=1.000; off‐label underdose vs. off‐label overdose, P=0.609). In multivariate analysis, HASBLED score was independent risk factors for bleeding events (OR 9.650; CI 1.998‐46.622; P=0.005).

**Conclusions:** Compared with standard or off‐label overdose of Apixaban, off‐label underdose of Apixaban was associated with a lower risk of bleeding in patients with AF and CKD, with no difference in the risk of stroke/systemic embolism or death, supporting the apixaban dosing tailored to specific clinical features and drug pharmacokinetics of the Asian patients.

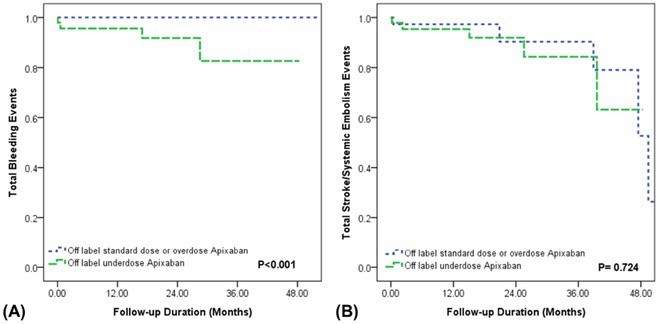

## IMPACT OF INCREASING BALLOON SIZE ON SUPERIOR VENA CAVA POTENTIAL WITH SIZE‐ADJUSTABLE CRYOBALLOON

### **YUKIHIRO INAMURA** ^1^, OSAMU INABA^1^, SHIN MEGURO^1^, KENTARO NAKATA^1^, YUHEI ISONAGA^1^, SHINICHI TACHIBANA^1^, HIROAKI OHYA^1^, TAKAMITSU TAKAGI^1^, AKIRA SATO^1^, YUTAKA MATSUMURA^1^, TETSUO SASANO^2^

#### ^1^Japanese Red Cross Saitama Hospital, Saitama, Japan,^2^Tokyo Medical and Dental University, Tokyo, Japan

**Introduction:** This is known that right superior pulmonary vein (RSPV) isolation with a cryoballoon could affect a superior vena cava (SVC) potential. This study aimed to evaluate whether cooling to RSPV on a 28 mm or 31mm cryoballoon has a different effect on the SVC potential.

**Methods:** Eighteen consecutive paroxysmal atrial fibrillation patients who underwent PV isolation with a POLARx FIT cryoballoon were prospectively enrolled. First, a voltage map of the SVC was obtained using a ring catheter before PV isolation; areas with a voltage of less than 0.1 mV were defined as scar areas. The length from the SVC‐right atrium junction to the top of the area with a voltage ≥ 0.1 mV on the SVC septal side was calculated as the SVC septal‐side sleeve length. Nest, the 4 PVs were isolated in a 28 mm cryoballoon and the SVC voltage map was generated in the same way. Next, the balloon size was increased to 31 mm, and single‐shot cooling was performed for 180 s with additional RSPVs and LSPVs, after which the SVC voltage map was again generated. We compared changes in the septal‐side sleeve length of the SVC before PV isolation, after cryoablation with a 28mm balloon, and after additional cooling with a 31 mm balloon.

**Results:** Of the 18 cases (11 males, mean age 66.9±11.4 years), all PVs were successfully isolated and the number of cryo‐application times required for isolation was 1.4, 1.0, 1.3, and 1.1 times for left superior (LS), left inferior (LI), right inferior (RI) and RSPV respectively, with a mean TTI of 55.5, 27.9, 35.4, and 27.0 seconds for LS, LI, RI, and RSPV. Transient phrenic nerve palsy during RSPV isolation was observed in one case each with 28 and 31mm balloons. The septal‐side sleeve length of the SVC was 29.0±8.4mm before PV isolation, 25.0±10.2 mm after cryoablation with a 28 mm balloon, and 22.7±10.0 mm after additional cooling with a 31 mm balloon. The septal‐side sleeve length of the SVC became shorter after the use of the 31 mm balloon with a significant difference compared to the sleeve length before PV isolation (P=0.049).

**Conclusions:** Cryoablation for RSPV has a cooling effect that extends to the septal side of the SVC, and the effect is greater the larger the balloon size.

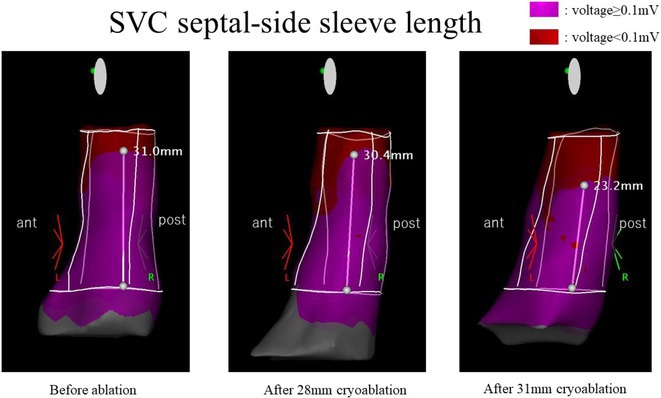

## THE INVESTIGATION OF THE RELATIONSHIP BETWEEN THE RECURRENCE OF BLOCKLINE AFTER CAVOTRICUSPID ISTHMUS ABLATION AND THE ANATOMICAL CHARACTERISTIC OF CAVOTRICUSPID ISTHMUS

### **TOMOYUKI INOUE**, MUNEKAZU TANAKA, AKIFUMI MORINAGA, FUMIYA YONEDA, SHUSHI NISHIWAKI, REO HATA, HIROHIKO KOUJITANI, SATOSHI SHIZUTA, KOH ONO

#### Kyoto university hospital, Kyoto, Japan

**Introduction:** The anatomical characteristics of the cavotricuspid isthmus (CTI) that may predict re‐conduction at the site of the blockline after CTI ablation are unknown. The aim of this study is to investigate the relationship between the re‐conduction of the CTI blockline and the anatomical characteristics of the CTI.

**Methods:** We analyzed 240 patients who underwent re‐ablation for recurrent atrial arrhythmia after initially undergoing catheter ablation for the CTI. Additionally, we measured the length(figure①), depth(②), and angle(③)of the CTI about 240 patients using CT scans, and investigated the relationship between these measurements and the re‐conduction of the CTI blockline.We defined the angle of the CTI as the angle formed between the line connecting the atrial and ventricular ends (the horizontal CTI line), and the parallel line of the inferior vena cava (IVC). We also defined the depth of the CTI as the distance from the horizontal IVC line to the bottom of the CTI curve (figure).

**Results:** Re‐conduction of the CTI blockline was observed in 39 cases (16.3%). There was no significant difference in the length or depth of the CTI between the re‐conduction group and the non‐re‐conduction group (length:35.4mm±8.8mm vs 35.0mm±7.9mm, p=0.78), (depth:5.73mm±2.5mm vs 6.27mm±2.7mm, p=0.22). However, the angle of the CTI in the recurrence group was significantly smaller compared to the non‐recurrence group (89.6°±20.5° vs 97.5°±19.2°, p=0.02).

**Conclusions:** The angle of the CTI may be associated with the re‐conduction of the CTI blockline.

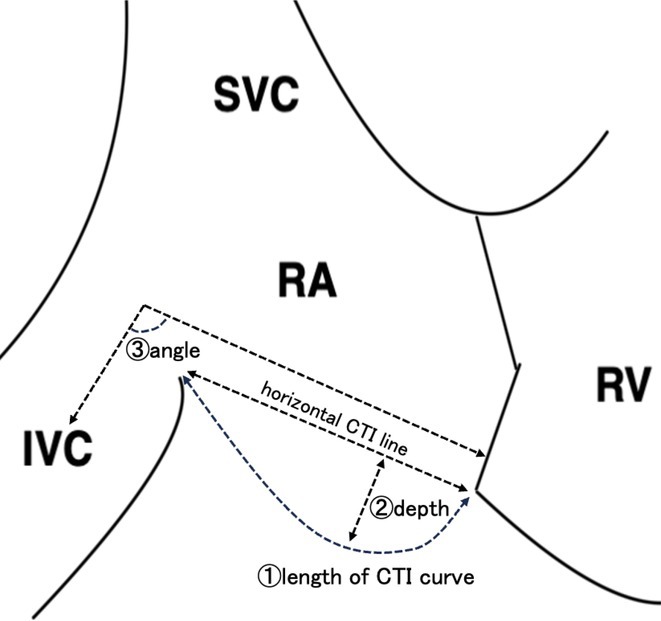

## DELAYED RIGHT VENTRICULAR SEPTAL PACING LEAD PERFORATION RESULTING IN HEMOTHORAX WITHOUT HEMOPERICARDIUM: A CASE REPORT

### **NASTITI INTANSARI**, BUDI BAKTIJASA DHARMADJATI, RERDIN JULARIO, MUHAMMAD RAFDI AMADIS, RAGIL NUR ROSYADI

#### Airlangga University, surabaya, Indonesia

**Introduction:** Permanent pacemaker lead perforation in the right ventricle is a very rare complication of PPM implantation (0.1‐0.8%). Pacemaker lead perforation can cause a variety of symptoms ranging from asymptomatic to hemodynamic compromise and tamponade. Hemothorax is a very rare complication of PPM implantation. Hemothorax is a consequence of perforation of the pacemaker through the right ventricle and pericardium into the pleural cavity, as well as direct injury to the lungs and blood vessels. Pacemaker lead perforation can be confirmed via imaging, with Computed Tomography (CT) being the standard diagnostic imaging modality.

**Methods:** N/A

**Results:** A 72 year old female patient was referred with complaints of coughing, shortness of breath and chest pain with inspiration for 1 month, with a history of VVIR mode active lead PPM installation in right ventricle septal 3 months before. Electrocardiogram revelaed PPM failed to capture. Chest x‐ray revelaed left pleural effusion. Echocardiography did not show pericardial effusion. Device interrogation revealed low lead impedance (314 ohms). Thorax MSCT examination showed a suspicion of solid mass in the anterior left lung and distal PPM leads in the right ventricle. A thoracotomy was performed on the patient, with evacuation of hemorrhagic pleural effusion, repositioning the PPM lead and repairing myocardium. The patient showed signicant clinical improvement

**Conclusions:** The risk factors and clinical course of patient showed the importance of recognizing suspected pacemaker lead perforation even if confirmation by imaging (CT) cannot be established. Less traumatic passive fixation lead might be used in high risk patient. Combination of contracting myocardium, reactive fibrosis, self‐sealing ventricle and visceral pericardium, might explain the cause of hematothorax without hemopericardium in this case

## LEFT BUNDLE BRANCH AREA PACING FOR HIGH‐GRADE ATRIOVENTRICULAR BLOCK IN A PATIENT WITH DEXTROCARDIA

### **LINA LING‐LING IP**, PING‐WA YAM

#### Tuen Mun Hospital, Hong Kong, China

**Introduction:** We report the first case of LBBAP for high‐grade atrioventricular (AV) block in a patient with dextrocardia in Hong Kong.

**Methods:** N/A

**Results:** A 73‐year‐old man had minor coronary artery disease and situs inversus dextrocardia diagnosed by computer tomography coronary angiography before. He was admitted to our hospital for dizziness and chest discomfort. Electrocardiogram (ECG) showed sinus rhythm with 4:1 AV block and ventricular rate of 30‐40bpm, and QRS duration of 144ms due to right bundle branch block. Echocardiogram showed left ventricular ejection fraction of 63%. We proceeded with dual‐chamber pacemaker implantation. As substantial ventricular pacing is anticipated, we intended to place the ventricular lead in LBBA to prevent the patient from developing right ventricular (RV) septal pacing‐induced cardiomyopathy.

Vascular access was obtained by venogram‐guided right axillary vein puncture. A lumenless lead (SelectSecure^TM^ MRI SureScan^TM^ 3830, Medtronic) was delivered into RV through a fixed‐curve sheath (C315HIS, Medtronic) after the sheath was reshaped manually to reverse the primary curve. Clockwise torque was applied, so that the sheath can reach RV basal to mid septum. The lead was rapidly rotated in clockwise direction to penetrate interventricular septum and the lead depth was determined by septography in right anterior oblique view. LBBA was successfully captured, as evidenced by the pacing stimulus to R wave peak time in lead V6 (V6RWPT) of 68ms. The pacing threshold was 0.5V at 0.4ms. The paced QRS duration was 142ms, comparable with the intrinsic QRS. The atrial lead was placed over right atrial appendage. Both leads were connected to a dual‐chamber pulse generator (Attesta^TM^ DR MRI SureScan^TM^, Medtronic).

His symptoms resolved with pacing. The pacing threshold remained stable 2 weeks later and thereafter. At 3 months after pacemaker implantation, the atrial and ventricular pacing percentages were 33.6% and 99.8% respectively with base rate of 60bpm.

**Conclusions:** This case demonstrates that LBBAP is feasible in patient with situs inversus dextrocardia by using currently available implantation tools.

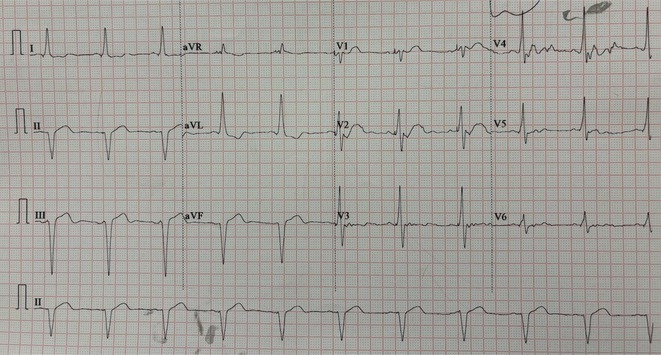

## A COMPARATIVE STUDY OF SUB‐SPECIALTY SELECTION AMONG ADULT CARDIOLOGY FELLOWS IN TRAINING AND DOES GENDER PLAY A ROLE?

### REEMA QAYOOM, MUHAMMAD MOHSIN, FAISAL QADIR, KUBBRA RAHOOJA, DEEBAJ NADEEM, ZUBAIR MUMTAZ, **GHAZALA IRFAN**

#### National Institute Of Cardio Vascular Diseases, Karachi, Pakistan

**Introduction:** The history of postgraduate medical training in Pakistan is impressive. Committing to sub‐specialty training after cardiology fellowship can be one of the most challenging decisions faced by fellows in training (FITs) in terms of work‐life balance. This study is aimed to determine the factors that influence the selection of sub‐specialty training among adult cardiology FITs and whether this differs by gender.

**Methods:** Multiple‐choice online survey conducted among Adult cardiology FITs in Pakistan pursuing cardiovascular sub‐specialties from January‐May 2023

**Results:** A total of 198 FITs completed the survey; 36% anticipated specializing in interventional cardiology (IC), 28% in Electrophysiology (EP), 12% in critical care (CC), 15% in cardiac imaging (CI) and 9% were unsure. Among IC and CC group, there was male FIT dominance (70% and 54% respectively) and likelihood of getting employment after completing training was most important factor in both sub‐specialities. Among EP and CI group, there was predominance of women FIT (76% and 56% respectively) as shown in Figure 2. The most important factor that influenced FITs to pursue EP was a strong interest in the field and female role model, while for CI minimal radiation exposure and flexibility in job were important factors.

**Conclusions:** Female FITs are less inclined to choose interventional cardiology as future career path because of increased radiation exposure and old‐boys club. Conversely there is growing trend for female FITS inclination towards choosing Electrophysiology, this is in contrast to similar study conducted internationally^.^

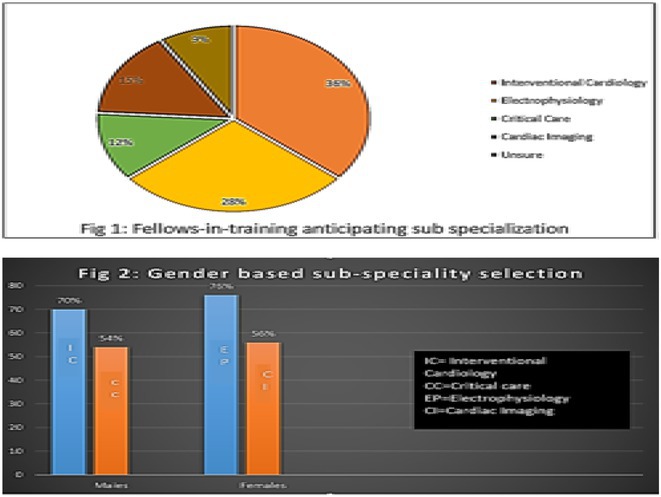

## EFFICACY OF HEAD‐UP TILT TEST FOR WORK‐UP OF UNEXPLAINED RECURRENT ATYPICAL TRANSIENT LOSS OF CONSCIOUSNESS ‐ PSYCHOGENIC PSEUDO‐SYNCOPE

### REEMA QAYOOM^1^, FAISAL QADIR^1^, ZUBAIR MUMTAZ^1^, DEEBAJ NADEEM^1^, IFRAH UROOS^1^, MUHAMMAD IMRAN KHOKHAR^2^, MUHAMMAD MOHSIN^1^, **GHAZALA IRFAN** ^1^

#### ^1^National Institute Of Cardio Vascular Diseases, Karachi, Pakistan,^2^Basildon University Hospital, Basildon, United Kingdom

**Introduction:** Clinical presentation with the sudden loss of consciousness occurs frequently and often distressing clinical entity. We aimed to evaluate the head‐up tilt table test's usefulness in the diagnosis of recurrent, unexplained, atypical loss of consciousness after initial clinical evaluation.

**Methods:** A 15‐year‐old girl with no comorbid medical illness, presented with a history of multiple episodes of loss of consciousness for the last 3 months. The "attacks" were described as frequent, sometimes occurring more than once a day, with a duration of unconsciousness lasting about 3‐5 minutes. Patient during these episodes was unresponsive and flaccid with eyes closed. Post‐recovery, the patient would appear to be lethargic. She was labelled as having vasovagal syncope. During the clinic visit, the patient looked anxious but alert and oriented. Her clinical examination was unremarkable. Baseline ECG, blood tests and echocardiography were normal. Head up tilt table test was scheduled for further evaluation.

**Results:** We performed a head‐up tilt table test according to the Italian protocol. After 1 minute of attaining an upright position, the patient was noted to suddenly become unconscious. ECG monitoring showed sinus tachycardia. On close observation, the patient was deliberately moving her eyeballs, keeping her eyes closed, and appeared aware of her surroundings as she started responding when the supervising physician asked the nurse to pass a nasogastric tube. Further testing was aborted as the diagnosis of psychogenic pseudo‐syncope was entertained. As the patient had some obvious stress from home, she was referred for cognitive behavioral therapy. The patient was followed up in the clinic after 3 months and her episode frequency had declined to just a single episode in the stated period.

**Conclusions:** Physicians who treat patients who have syncope should be aware of this illness and able to differentiate PPS from other types of syncope.

## THE EFFECT OF ORAL GLUCOSE LOAD ON VENTRICULAR DEPOLARISATION AND REPOLARISATION IN BRUGADA SYNDROME

### **JULIA ISBISTER** ^1,2^, MARINA STROCCHI^3,4^, MATTHEW RIEDY^5^, LAURA YEATES^1,2,6,7^, BELINDA GRAY^1,2^, HARIHARAN RAJU^8^, CHRISTOPHER SEMSARIAN^1,2,6^, STEVEN NIEDERER^3,4,9^, RAYMOND SY^1,2^

#### ^1^Faculty of Medicine and Heath, The University of Sydney, Sydney, Australia,^2^Royal Prince Alfred Hospital, Sydney, Australia,^3^National Heart and Lung Institute, Imperial College London, London, United Kingdom,^4^School of Biomedical Engineering and Imaging Sciences, King's College London, London, United Kingdom,^5^Medtronic Australasia, Melbourne, Australia,^6^Agnes Ginges Centre for Molecular Cardiology at Centenary Institute, Sydney, Australia,^7^h Genomics and Inherited Disease Program, Garvan Institute of Medical Research and University of New South Wales, Sydney, Australia,^8^Faculty of Medicine, Health and Human Sciences, Macquarie University, Sydney, Australia,^9^The Alan Turing Institute, London, United Kingdom

**Introduction: Background:** Glucose load, either orally or intravenously with co‐administration of insulin, has been shown to provoke ST elevation in patients with Brugada syndrome (BrS). The effect of oral glucose on ventricular depolarisation and repolarisation has not been studied *in vivo* and detailed assessment may shed light on the pathophysiology of BrS. This study sought to compare the effect of oral glucose on ventricular depolarisation and repolarisation in patients with BrS and healthy controls.

**Methods:** Eleven patients with BrS and six healthy controls underwent electrocardiographic imaging during sinus rhythm at baseline, 30, 60 and 90 minutes following an oral glucose load (75g in 300ml water). Regional differences in activation time (AT), repolarisation time (RT), gradients and activation‐recovery interval (ARI) were analysed and electroanatomical maps constructed.

**Results:** 88.2% of participants were male (10 of BrS group and 5 of control group). The average age of patients with BrS was higher than controls (46.7 ± 11.0 vs 37.2 ±7.6 years). At baseline, AT in the right ventricular outflow tract (RVOT) was longer (57.3 ± 11.7ms vs 32.8 ± 13.3ms, p = 0.004), RT (297.8 ± 22.1ms vs 301.8 ± 22.5ms, p= 0.731) was similar, and ARI was shorter (240.1 ± 18.5ms vs 269.0 ± 28.1msl p= 0.022) in patients compared to controls. Regional comparison [RVOT‐ biventricular mass (BiV)] of indices at baseline showed greater difference in AT and RT (but not ARI) in BrS patients compared to controls. Glucose accentuated regional differences in RT and ARI in patients with BrS but not in controls. Difference in mean RT between the RVOT and BiV increased significantly in patients with BrS (42.0ms to 51.1ms, p=0.002, orange in **Figure**) but not controls (22.0ms to 24.7ms, p=0.990, blue in **Figure**). A similar regional difference was observed in ARI of BrS patients but not controls.

**Conclusions:** Glucose accentuates regional differences in RT and ARI, but not AT between RVOT and remaining ventricular mass in patients with BrS but not controls.

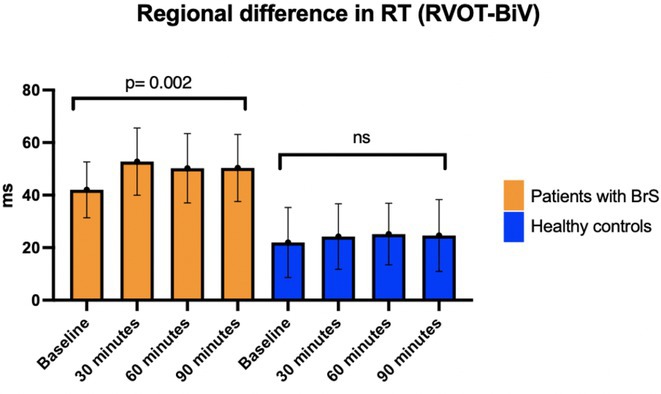

## EVALUATION OF THE REAL TIP POSITION OF LEADLESS PACEMAKERS WITH COMPUTED TOMOGRAPHY

### **SHINJI ISHIMARU**, MARIKO KAWASAKI, RUI KAMADA, TAKUYA KOIZUMI, TAKASHI TAMURA, JUNGO FURUYA, YOSHIROU MATSUI, KEIICHI HANAOKA

#### Hanaoka Seishu Memorial Cardiovascular Hospital, Sapporo, Japan

**Introduction:** Leadless pacemakers (LPM) are accepted globally to avoid lead‐ and pocket‐associated complications. On the other hand, cardiac perforation during LPM implantation is more likely to require intervention than perforation by a transvenous lead and the complication is sometimes lethal. It is the reason that implanting LPM at right ventricular septum is recommended to prevent the perforation. Furthermore, large‐scale trials of pacemakers and cardioverter‐defibrillators suggest that right ventricular apical pacing may lead to progressive left ventricular dysfunction and heart failure even in patients with normal cardiac function, owing to the electrical and mechanical dyssynchrony. Pacing at interventricular septum shows narrower QRS than apical pacing in electrocardiogram, so it might be more advantageous than right ventricular apical pacing in aspect of electrophysiology. So, it is reasonable to implant LPM at right ventricular septum (RVS). In our hospital we had tried to place the LPM at RVS. But it is difficult to determine the true pacing site during implantation procedure by contrast X‐ray alone.

**Methods:** To verify the true pacing site in cases of LPM implantation, we evaluated 24 patients (including 21 Micra™ and 3 Aveir™) who underwent CT scan under several reasons and could be recognized exact tip position retrospectively.

**Results:** The true septal pacing was detected in 11 patients (45.8%). In others the LPM were placed at the hinge position of the right ventricular wall (n =11, 45.8%), and at the ventricular free wall (n=2, 8.3%), respectively.

**Conclusions:** In contrast to physicians’ intention, about half of the LPM were placed at other than RVS. It is difficult to place the LPM at RVS properly under contrast X‐ray alone.

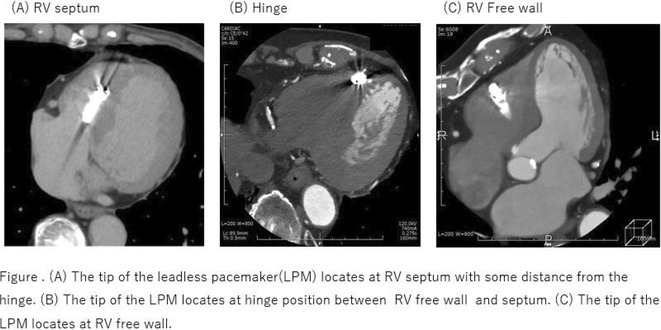

## A NOVEL APPROACH TO INVISIBLE VEIN OF MARSHALL

### **MASAYUKI ISHIMURA** ^1^, MASASHI YAMAMOTO^1^, TOSHIHARU HIMI^1^, YOSHIO KOBAYASHI^2^

#### ^1^Kimitsu Central Hospital, Kisarazu, Japan,^2^Chiba University Hospital, Chiba, Japan

**Introduction:** The utility of ethanol infusion into the vein of Marshall (EIVOM) is limited in patients without a visible vein of Marshall (VOM).

**Methods:** Our study included of 232 patients with atrial fibrillation undergoing MI ablation. We initially performed coronary sinus venography using a balloon catheter in all patients to assess VOM characteristics. EIVOM was attempt in those with adequately visible VOMs. In cases where the VOM was not visible, we utilized a guidewire to locate its entrance, using the intravenous valve as a landmark.

**Results:** Venography initially identified the VOM in 140 of 232 patients (60%) (visible VOM group). Within this group, 68 patients (29%) did not require balloon inflation for VOM visualization, while 72 patients (31%), visibility was achieved through balloon inflation. In the remaining 92 patients (39%), the VOM remained invisible even after balloon inflation. Nevertheless, in 13 of these cases, the VOM was successfully located using a landmark technique (invisible VOM group). EIVOM was fully achieved in 96% (147 of 153 patients). Additionally, full MI line blocks were achieved in 91% of (A) the visible VOM group (122 of 134 patients), 92% of (B) the invisible VOM group (12 of 13 patients), and 81% in (C) the non EIVOM group (69 of 85 patients) (A vs. B, *p* = 0.88; A vs. C, *p* .05; B vs. C, *p* = 0.32).

**Conclusions:** A novel approach to locating invisible VOM extends the clinical utility of EIVOM and potentially improves success rate of MI ablation.

## APPROPRIATE DEVICE SELECTION FOR LONG QT SYNDROME IN A PEDIATRIC PATIENT WITH REFRACTORY SYNCOPE

### **NARUYA ISHIZUE**, TAKASHI HONDA, YOICHIRO HIRATA, HIDEHIRA FUKAYA, SHO OGISO, SYUHEI KOBAYASHI, HIRONORI NAKAMURA, JUN KISHIHARA, SHINICHI NIWANO, JUNYA AKO

#### Kitasato University, Sagamihara,Kanagawa, Japan

**Introduction:** Implantable cardiac electrical device therapy, including implantable cardioverter defibrillator (ICD) and pacemaker, is considered for patients with long QT syndrome (LQTS) who experience recurrent syncope despite medical treatment such as beta‐blockers, but its appropriate indication remains unclear especially in pediatric patients with LQTS.

**Methods:** N/A

**Results:** An 11‐year‐old male with a family history of LQTS was found to have a prolonged QTc (QTc 559 msec) at the age of 6 years during a school physical examination; he scored 4 points on the Schwartz criteria (QTc greater than or equal to 480 msec, family history of LQTS) and was diagnosed with congenital LQTS. Genetic testing revealed a KCNH2 mutation, leading to the diagnosis of LQT2. He was initially treated with beta‐blockers to prevent cardiac events. However, he experienced syncope at the age of 10. Despite increasing the beta‐blocker dosage and adding mexiletine, syncopal events recurred within one year. An AAI pacemaker with epicardial leads was subsequently implanted. Because of the patient's genotype and age, a pacemaker was chosen instead of an ICD. The QTc interval was shortened to 473ms by intentional atrial pacing, and he did not experience syncope after pacemaker implantation

**Conclusions:** This case report highlights the successful suppression of drug‐resistant syncope events with an AAI pacemaker in a pediatric patient with LQTS.

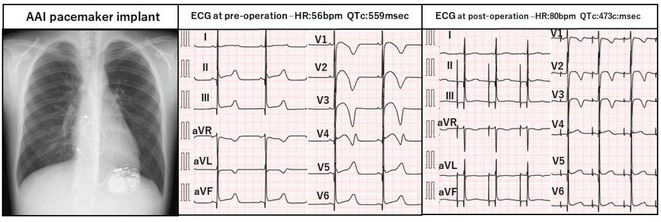

## COMPARISON OF TWO CRYOBALLOON SYSTEM FOR PULMONARY VEIN ISOLATION

### **M. ITO** ^1^, K. Ashikaga^2^, K. Ayabe^2^, Y. Tsumagari^2^, H. Takekawa^2^

#### ^1^Miyazaki Prefectural Nobeoka Hospital, Miyazaki, Japan, ^2^Miyazaki Medical Association Hospital, Miyazaki, Japan

**Introduction:** Cryoballoon (CB) ablation therapy has recommended for paroxysmal atrial fibrillation (AF). Recently there are two competing CB system; POLARx, Boston Scientific and Arctic Front Advance (AFA), Medtronic. There is insufficient data on comparison of the area of posterior left atrium (LA) after pulmonary vein isolation (PVI). We studied to compare the area after PVI by each CB and to evaluate safety, efficacy and characteristics about two CB systems.

**Methods:** Seventy‐five consecutive patients who underwent AF ablation at our hospital were prospectively evaluated. The Rhythmia mapping system and the Orion mapping catheter, or EnSite mapping system and the HD grid mapping catheter were used to systematically map the LA map before and after PVI.

**Results:** There were 20 patients (71.1±7.9 years, 35% Male) undergoing PVI with POLARx and 75 patients (69.±9.2 years, 50.9% Male) undergoing PVI with AFA. Therewere no significant between POLARx and AFA for the area of posterior LA before PVI (19.7 vs 19.2 cm2, p=0.296),fluoroscopy time (15.5±6.9 vs 16.5±8.1 min, p=0.631). While there was significant difference for the rate of contact area of CB (POLARx 58.0±3.0 vs AFA 37.5±12.7 %, p≤0.01), it had suggested that ablation area had be wide in using POLARx.Only one complication occurred in POLARx (tanponade). Atrial arrhythmia recurrence rate was low with 5.5% (POLARx) and 5% (AFA) at 6 months (p=0.712).The ablation area with POLARx was wide. Both ablation systems are comparably safe and effective.

**Conclusions:** The ablation area with POLARx was wide. Both ablation systems are comparably safe and effective.

## SAFETY AND EFFICACY OF SPOT ROI® IN REDUCING FLUOROSCOPY EXPOSURE DURING ATRIAL FIBRILLATION ABLATION

### **HIDEHIRO IWAKAWA**, NOBUHIRO SUZUKI, RYOSUKE KATO, HIYU WAKABAYASHI, HARUWO TASHIRO, KEN TERATA, HIROYUKI WATANABE

#### Akita University Graduate School of Medicine, Akita, Japan

**Introduction:** Radiofrequency catheter ablation (RFCA) for atrial fibrillation (AF) is associated with long fluoroscopic durations and significant radiation exposure. Spot region of interest (ROI)® platform (Alphenix, Canon Medical Systems) is a novel functionality aimed at reducing fluoroscopy exposure. Although its usefulness has been evaluated in neuro‐interventional procedures, little is known about its safety and effectiveness in RFCA for AF. The aim of this study was to determine whether Spot ROI can reduce radiation exposure without procedural disadvantages.

**Methods:** We enrolled 50 consecutive patients (age, 64±8 years; male, 69%) with AF scheduled for RFCA from January 2023 to March 2024. We divided them into two groups: Spot ROI (SROI), N=25 and conventional collimation (CC), N=25. Pulsed fluoroscopy in the CC group was at a rate of 7.5 pulses/s. SROI fluoroscopic imaging consists of a brighter square indicating the ROI with a normal radiation dose, surrounded by a darker area where fluoroscopic exposure is reduced by 80% compared to CC (Figure). RFCA was performed under fluoroscopic guidance and 3‐dimensional electro‐anatomical mapping systems.

**Results:** AF ablation including pulmonary vein isolation and additional liner ablation (if necessary) was successfully performed in all patients without any complications. Dose‐area product of SROI group was significantly lower than CC group (855±430 vs 1867±931 cGy cm^2^, P<0.001), with a reduction of approximately 55% in radiation exposure. There were no significant differences in fluoroscopy time (SROI, 13.9±4.3 min; CC, 15.0±4.5 min, P=0.43), total procedure time (SROI, 158.1±43.2 min; CC, 146.8±28.5 min, P=0.35) between two groups.

**Conclusions:** RFCA for AF using the SROI platform significantly reduced radiation exposure compared to conventional settings, with comparable acute success rate and procedure time.

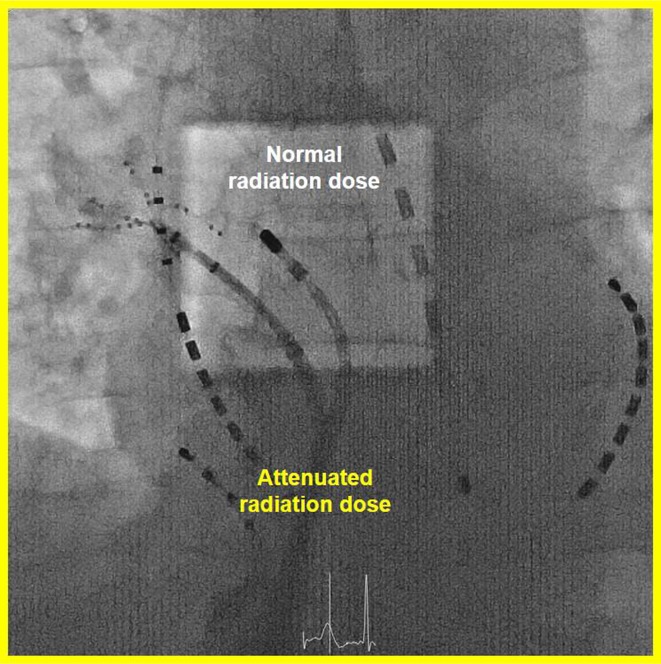

## ASSOCIATION BETWEEN SDB AND PVC IN PACEMAKER PATIENTS USING SLEEP APNEA MONITOR

### **KAORI IWAMA‐SAIGUSA**, HARUNA TABUCHI, ASUKA MINAMI‐TAKANO, ATSUSHI KIMURA, KAZUNORI MARUYAMA, MITSUHIRO KUNIMOTO, GAKU SEKITA, MASATAKA SUMIYOSHI, KIKUO ISODA, TORU MINAMINO

#### Juntendo University, Tokyo, Japan

**Introduction:** The Sleep Apnea Monitor (SAM) function of Microport pacemakers is a simple and easy way to evaluate sleep disordered breathing(SDB), with a high specificity of 85% and sensitivity of 89%. The SAM and the premature ventricular contraction(PVC) counter function can be used to clarify the relationship between SDB and PVCs, which may lead to useful patient interventions.

**Methods:** SAM was introduced for patients implanted with Microport pacemakers at Juntendo University Nerima Hospital from May 2016 to March 2023, and respiratory disturbance index (RDI) was recorded. SAM is expressed as a percentage of nights with severe sleep‐disordered breathing (RDI>20) during the analysis period (RDI%). In addition, the maximum RDI (RDI max)during the period was also evaluated, and the PVC group was defined as the group in which PVCs accounted for more than 2% of the cardiac cycle distribution, and the non‐PVC group as the group in which PVCs accounted for <1%. A total of 88 patients(PVC group:n=14, non‐PVC group :n=74)were analyzed retrospectively (median analysis period : 6 months.).

**Results:** The mean patient age was 84.3±9.7 years, and 44.3% were male.The mean Body Mass Index(BMI) was 22.5±3.7(PVC group:23.0±3.1,non‐PVC group:22.4±3.8),showing no significant difference between the PVC and non‐PVC groups(p=0.6114).The mean RDI% was 66.1±10.1% in the PVC group and 31.5±4.4% in the non‐PVC group, and was significantly higher on nights with severe sleep‐disordered breathing in the PVC group (p=0.0165). The RDI max during the analysis period was 47.1±4.8 on average in the PVC group and 34.6±2.1 on average in the non‐PVC group, and was significantly higher in the PVC group (p=0.0204). The present study suggests that SDB may be associated with the development of PVCs.

**Conclusions:** This study suggests an association between SDB and PVCs; active intervention in SDB using SAM function may be useful in preventing the development of ventricular arrhythmias.

## THE NOVEL TENSOR CARDIOGRAPHY ANALYSIS BY USING CUMULATIVE DISTRIBUTION FUNCTIONS FOR PREDICTING LIFE‐THREATENING ARRHYTHMIA

### **YU‐KI IWASAKI** ^1^, SHINGO TSUKADA^2^, YAYOI‐TETSUO TSUKADA^1^, HIROSHIGE MURATA^1^, KENJI YODOGAWA^1^, WATARU SHIMIZU^1^, KUNIYA ASAI^1^

#### ^1^Nippon Medical School, Tokyo, Japan,^2^NTT Basic Research Laboratories, Bio‐Medical Informatics Research Center, Kanagawa, Japan

**Introduction:** R on T type PVC and non‐sustained VT are considered as warning arrhythmia leading to life‐threatening arrhythmia. However, ventricular fibrillation can suddenly develop without any warning signs. We introduce the clinical application of the novel Tensor cardiography, a novel analysis by using cumulative distribution function for prediction of life‐threatening arrhythmia during sinus rhythm.

**Methods:** We developed novel Tensor cardiography (TCG) analysis that calculate the difference between two cumulative distribution functions (CDFs) as the positive‐ and negative‐epicardium potential fits well with R and T waves. The two CDFs (above fRp fRn) and two inverse CDFs (f'Tn f'Tp) obtained by ECG approximation represent the action potential transition process time series of the anode and cathode groups of the origin dipole of the target R and T waveforms, respectively (Figure). The average (μRp, μRn, μTp, μTn) and standard deviation (σRp, σRn, σTp, σTn) of the 4 cumulative CDFs during sinus rhythm immediately before onset of ventricular fibrillation were evaluated to verify the usefulness of these parameters as clinical indices in predicting ventricular fibrillation.

**Results:** Results: In the patient with early repolarization syndrome, the sinus rhythm just before the onset of VF showed prolonged and fluctuating repolarization of the cathode. The average σTp and μRTn of 10 consecutive sinus rhythms just before the onset of VF were significantly increased compared to 30 seconds before VF onset (σTp: 29.9 ± 0.8 vs. 30.8 ± 1.0, p=0.035; μRTn: 271.0 ± 2.8 vs. 276.8 ± 7.5, =0.035). However, no significant differences were observed in the RR and QT intervals between these times (RR interval [ms]: 819.5 ± 1.8 vs. 818.0 ± 10.6, p=0.066; QT [ms]: 382.0 ± 2.9 vs. 388.8 ± 12.8, p=0.12).

**Conclusions:** Conclusion: TCG analysis could potentially provide predictive parameters during sinus rhythm for identifying the risk of life‐threatening ventricular arrhythmias.

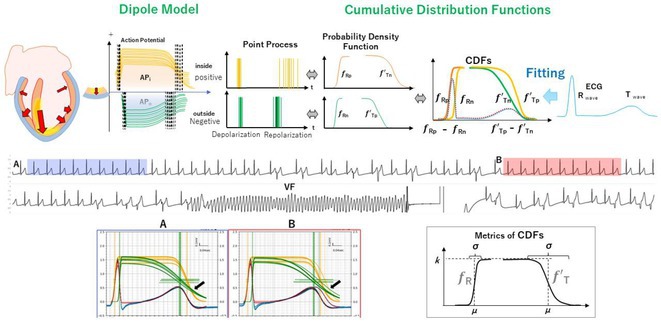

## DIFFERENCES IN ISOCHRONAL LATE ACTIVATION ZONES BETWEEN SINUS RHYTHM AND VENTRICULAR S1 AND S2 PACING AND COMPARISON WITH DELAYED EVOKED POTENTIALS INPATIENTS WITH VENTRICULAR TACHYCARDIA AND CARDIOMYOPATHY

### **JASON JACOBSON** ^1^, DANIEL READE^2^, DANIEL FRENKEL^1^, SEI IWAI^1^

#### ^1^Westchester Medical Center‐New York Medical College, Valhalla, NY,^2^Abbott, Minneapolis, MN

**Introduction:** Functional substrate mapping has become an important tool to identify the reentrant ventricular tachycardia (VT) isthmus in patients with scar‐based VT, without the need to induce VT. Isochronal late activation mapping (ILAM) is performed during sinus rhythm (SR) or ventricular pacing (VP) to identify zones of slow conduction. Delayed evoked potential (DEEP) mapping is another technique to identify the VT isthmus with S1S2 VP protocol to identify zones of delayed activation with S2 compared to S1. It is unclear if ILAM zones (IZ) are consistent during SR and VP, or between S1 and S2, and how DEEP and IZ compare.

**Methods:** Mapping was performed with Abbott Ensite X and the HD Grid using Omnipolar Technology (OT). IZ were determined during SR, and right VP S1 (500‐600ms CL) and S2 (20‐40 ms above ERP). Total activation time was divided into 8 isochrones and ILAM was defined as 3 isochrones within 1 cm, marked with 1cm tags. IZ areas were measured with Ensite marking tool around IZ. DEEP were defined as 10 ms local delay with S2 compared to S1. DEEP points were marked with lesion tags. Mean IZ area was compared with T test.

**Results:** 4 patients (2 ischemic, 2 nonischemic) had full datasets for each rhythm. 3 of the patients had largerIZ with VP than SR. The average total IZ area was similar across SR (20cm) S1 (22.7cm) and S2 (23.8cm) maps. However, there was a significant difference between the largest (17.1cm) and smallest (27.3 cm) IZ for each patient (p=0.002). A total 39 IZ were mapped with an area range of 1‐36.5 cm. Of these, 6 were consistent across all rhythms, 11 were consistent across 2 rhythms, and 22 were unique. Of 30 DEEP zones,18 at least partially overlapped with IZ. Figure Legend: Voltage maps (purple>1.5mV) with ILAM zones from each rhythm (different colored perimeters)and DEEP (brown circles).

**Conclusions:** Different IZ sizes and locations can be elicited with SR and VP, as well as with S2 VP. Additionally, DEEP zones and IZ do not always correlate. The mechanisms and significance of these findings require further study.

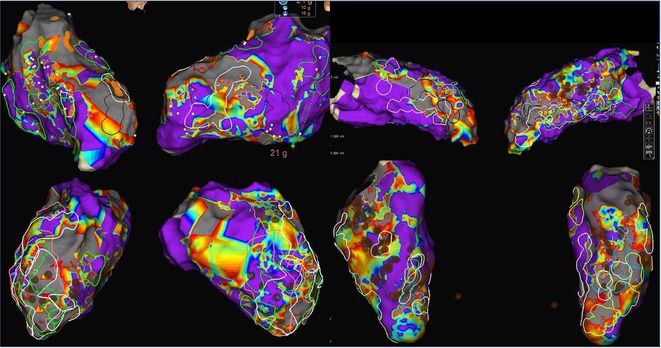

## DIRECT COMPARISON OF BIPOLAR AND OMNIPOLAR ELECTROGRAM VOLTAGE AMPLITUDE IN SINUS RHYTHM AND VENTRICULAR PACING

### **JASON JACOBSON** ^1^, DANIEL READE^2^, D. CURTIS DENO^2^, DANIEL CERVANTES^3^, MARCELLO ROTA^3^

#### ^1^Westchester Medical Center‐New York Medical College, Valhalla, NY,^2^Abbott, Minneapolis, MN,^3^New York Medical College, Valhalla, NY

**Introduction:** Standard bipolar voltage mapping (BV) depends on bipole orientation relative to wavefront direction. Omnipolar mapping technology (OT) allows direction independent voltage measure using sets of 3 electrodes yielding a maximum over all directions. However the extent to which BV and OT values differ and how voltage differs between rhythms is unclear. While prior studies have shown higher voltage with OT in general direct comparison by electrogram (EGM) site has not been reported.

**Methods:** Abbott HD Grid catheter was placed at multiple locations in the uninfarcted LV of 4 canines guided by intracardiac echocardiography and electroanatomic mapping (Abbott Precision) with preclinical research OT software. SR and VP was recorded at each location prior to moving to a new one. Within subjects non‐parametric analysis was employed for comparisons between OT and BV at each EGM site. Only EGMs with good contact and stability in SR and VP were included. The OT/BV and VP/SR ratios were calculated for each EGM site.

**Results:** 4752 EGM sites were analyzed. Median voltage was greater for OT than BV for SR and VP (Table).Voltage during VP was also greater than SR for BV and OT (Table). The median OT/BV and VP/SR are also reported. Significantly, the top quartile of OT/BV was 1.17‐1.41 for SR and 1.14‐1.41 for VP.

**Conclusions:** In this first site‐by‐site EGM comparison median OT voltage was greater than BV in SR (median7%, max 41%) and VP (median 6%, max 41%). This should be taken into consideration in defining normal voltage, otherwise substrate areas might be erroneously deemed normal, which could affect ablation sufficiency. Unexpectedly, median voltages were greater for VP using both OT (16%) and BV (13%). The mechanism and clinical significance of this is unclear and requires further study into underlying possibilities including (but not limited to) uniformity in VP conduction direction and reduced wavefront collision.

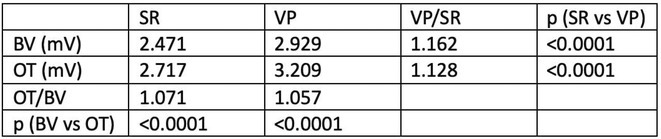

## FASCICULAR PVC WITH AN INTERFASCICULAR CONNECTION MASQUERADING AS JUNCTIONAL ECTOPY

### **JASON JACOBSON**, JONATHAN RAMALHO, MAHMOUD AHMED, BRENT KLINKHAMMER

#### Westchester Medical Center‐New York Medical College, Valhall, NY

**Introduction:** Premature ventricular complexes (PVC) may originate from the native conduction system (CS) including the right bundle branch (RBB) and left fascicular system. Junctional ectopic beats (JEB) have also been described and ablated in the proximal CS. We present a fascicular PVC that originated from the left posterior fascicle (LPF) and appeared to be a JEB.

**Methods:** NA

**Results:** 77‐year‐old woman presented with symptomatic PVCs (16% 1 week burden) despite therapy with flecainide.The initial PVC morphology showed incomplete RBB block, left inferior axis with positive V3 transition andQRS duration 90ms (Fig A). This was mapped to the distal left anterior fascicle (LAF) which was 30ms pre‐PVC with a PASO map >95%. A purkinje potential (PP) was seen at this site during SR and during PVC.Ablation at this site resulted in suppression of the PVC, but soon thereafter, a PVC with a slightly different morphology emerged. It was successfully ablated in the LAF just proximal to the first site ablation, without recurrence.Three months later, the patient returned with recurrent symptoms (13% 1 week burden) with a morphology that was identical to SR suggestive of JEB. This was presumed to be the same LAF PVC with distal LAF block and rapid conduction to the RBB as well as LPF. Activation mapping of the RV showed the earliest site in the mid‐RBB with retrograde conduction to the His. The LV was also mapped and the earliest site was found mid LPF with a Purkinje potential 50ms pre‐PVC and excellent PASO maps (Fig B). Ablation at this site resulted in suppression of the PVC without damage to the LPF. Of note, the PP was seen preceding the sinus rhythmQRS as well. The activation pattern of the RBB and LPF as well as the JEB morphology (identical to SR) is likely explained by an interfascicular connection (IFC) between the LPF and RBB. Figure Legend A: First ablation ECG and Map RAO/LAO. Dots indicate fascicles.B: Second ablation ECG and near‐field map of RBB (left) and LPF (right)

**Conclusions:** This case suggests the presence of IFC that can affect activation patterns and ECGs of PVCs.Unanswered questions include if IFC could sustain reentry and if more JEB might actually represent fascicularPVCs with IFC.

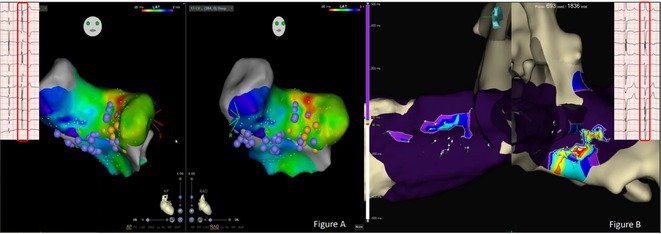

## INTRA‐PROCEDURAL ECHOCARDIOGRAPHY AND MANDATORY PHYSICIAN REFRESHER TRAINING REDUCES COMPLICATIONS FOR LEADLESS LEFT VENTRICULAR ENDOCARDIAL PACING

### **SIMON JAMES** ^1^, ANDREW TURLEY^1^, MATTEO ZIACCHI^2^, PRASHANTHAN SANDERS^3^, CHRISTOPHER ALDO RINALDI^4^, MARY WALSH^5^, JAGMEET SINGH^6^

#### ^1^James Cook University Hospital, MIDDLESBROUGH, United Kingdom,^2^University Hospital of Bologna, Bologna, Italy,^3^University of Adelaide, Adelaide, SA, Australia,^4^St. Thomas Hospital, London, United Kingdom,^5^St. Vincent Medical Group, Indianapolis, IN,^6^Mass General Hospital, Boston, MA

**Introduction:** The WiSE‐CRT System (EBR Systems, Inc.) is a novel CRT system that involves implanting a leadless electrode directly into the left ventricular (LV) endocardium. The implant procedure utilizes techniques drawn from a range of subspecialties including intercostal implantation of the ultrasound transmitter, generator implantation between serratus anterior / posterior surface of the latissimus dorsi, large bore arterial access or transseptal puncture and catheter manipulation within the LV. The pivotal SOLVE‐CRT study demonstrated the efficacy of the system albeit with a complication rate of 19.1%. Cardiac perforations were a specific concern.

**Methods:** Our aim was to assess if a mitigation strategy of either transesophageal or intra‐cardiac echocardiography during implant, plus mandatory physician refresher training prior to scheduled implants can reduce the complication rate for WiSE‐CRT implantation. Implants performed in the SOLVE‐CRT study were dichotomised to before or after the implementation of the above practice. Complication rates were then compared for both groups.

**Results:** Overall Type 1 complications were a significantly reduced from 26.8% to 14.3% (p=0.037) as were any cardiac perforations (7.0% v 1.8%, P = 0.071). There were no events requiring surgical repair in the post mitigation group. Impact of the mitigation strategy is shown in table 1.

**Conclusions:** Complications of WiSE‐CRT implant were significantly reduced by the use of real‐time echocardiography and mandatory physician retraining prior to implant. The greatest impact was related to direct complications of the endocardial electrode implantation itself, including the risk of perforation. Importantly, there were no events requiring surgical repair in the post mitigation group.

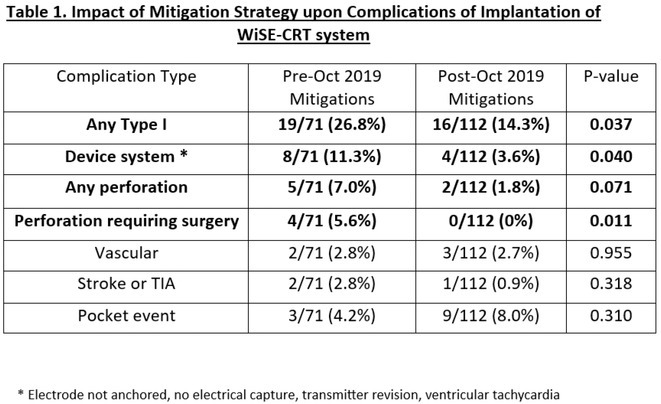

## EFFICACY OF RADIOFREQUENCY ABLATION FOR POST‐HEART TRANSPLANT RAPID ARRHYTHMIAS: A SINGLE‐CENTER EXPERIENCE

### **ZIHAN JIANG**, FENGYUAN YU, MIN TANG

#### Arrhythmia Center, State Key Laboratory of Cardiovascular Disease, Fuwai Hospital, National Center for Cardiovascular Diseases, Chinese Academy of Medical Sciences and Peking Union Medical College, Beijing, China

**Introduction:** Radiofrequency ablation (RFA) is a potential treatment option for rapid arrhythmias in post‐heart transplant patients who have not responded to pharmacological interventions. Despite its potential utility, there is limited data available on its success rates and efficacy. To address this gap in knowledge, the present study presents a case series of patients from our ward who underwent RFA for rapid arrhythmias following heart transplantation.

**Methods:** This retrospective study reviewed post‐heart transplant patients in our ward who underwent RFA for rapid arrhythmias within the past five years. Prior to RFA, all patients underwent coronary CT angiography, cardiac MRI, and/or myocardial biopsy to assess rejection and transplant vasculopathy. Recurrence was defined during follow‐up as tachycardia lasting >30 seconds on a 24‐hour Holter monitor.

**Results:** Seven patients underwent RFA for arrhythmias following heart transplantation in our ward, including three cases of atrial flutter, three cases of atrial tachycardia, and one case of atrial fibrillation (Table). All atrial flutter cases were cavotricuspid isthmus‐dependent. The atrial tachycardia foci were detected at the anterior wall of the donor superior vena cava, the right atrial scar close to His bundler, and the posterior inferior margin of the right atrial septum. One patient experienced RFA failure due to mild transplant vasculopathy indicated by coronary CTA and a history of self‐discontinuation of immunosuppressants. The remaining patients achieved successful RFA. During a median follow‐up period of 448 days, one patient relapsed 17 months after RFA and underwent a second RFA. Eight months after the second ablation, the patient passed away due to severe pneumonia without any recurrence of arrhythmia.

**Conclusions:** This study demonstrates that RFA can be effective for managing arrhythmias post‐heart transplantation over the long term. In this limited sample, non‐adherence to immunosuppressive therapy and extensive intracardiac scarring were potential factors contributing to unsuccessful RFA outcomes.

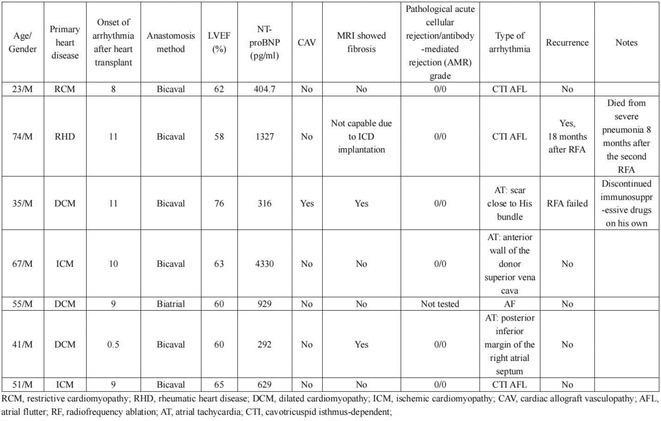

## THE VALUE OF ELECTROPHYSIOLOGICAL STUDY IN RISK STRATIFICATION OF HYPERTROPHIC CARDIOMYOPATHY

### BEE NGO LAU, **SOFIAN JOHAR**

#### Raja Isteri Pengiran Anak Saleha Hospital, Bandar Seri Begawan, Brunei Darussalam

**Introduction:** Electrophysiological study (EP) for risk stratification of hypertrophic cardiomyopathy (HCM) is a Class III recommendation in the guidelines. However we offer ventricular stimulation to asymptomatic patients clinically deemed to have a low risk of sudden death. The aim of this study is to determine the outcome of patients having EP.

**Methods:** From 2014 to 2018, we collated and analysed data of patients having HCM from electronic medical records at our local hospital.

**Results:** Out of 39 patients having HCM, 22 patients had EP (mean age 55.5 +/‐ 11.9 years, 76% male). 11 patients had positive (+ve) EP. Out of these 11 patients, 6 had implantable cardioverter defibrillators (ICDs). Out of the 11 patients having negative (‐ve) EP, only 1 had ICD due to a family history of sudden death. Out of the 17 patients with no EP, 6 had ICDs. At baseline, 1 had significant left ventricular outflow tract obstruction, 1 had family history of sudden death, 3 had non‐sustained ventricular tachycardia (NSVT), and 1 had syncope and NSVT.

During a mean follow up of 28.7 +/‐ 12.1 months, in the **EP +ve / ICD group**, 1 had appropriate shock for ventricular tachycardia (VT), 2 had NSVT, 1 had paroxysmal atrial fibrillation and 2 had no arrhythmia. In the **EP +ve / no ICD group**, 1 patient deemed to be at low risk (HCM risk‐SCD^1^ score 2.49%) had sudden death, 2 were asymptomatic, and 2 had insertable cardiac monitors. Out of these 2 patients, 1 had asymptomatic long pauses secondary to atenolol. The other patient had no arrhythmia. In the **EP ‐ve group**, the 1 patient having ICD had asymptomatic paroxysmal atrial tachycardia (PAT). The other 10 patients were asymptomatic. In the **no EP / ICD group**, 3 patients developed NSVT, 1 had PAT and 2 had no arrhythmia. In the **no EP / no ICD group**, all 11 patients were asymptomatic. There were no ICD related complications or inappropriate shocks. The mean HCM risk‐SCD score was 2.4 +/‐ 1.1 in the EP group, and 3.5 +/‐ 2.4 in the no EP group.

**Conclusions:** In our study, the event rate of sudden death / appropriate shock was 5%, and EP study may help in additional risk stratification for HCM patients.

**Ref:**

*1) Elliott P, et al. 2014 ESC Guidelines on diagnosis and management of HCM. Eur Heart J*

## ATRIAL FIBRILLATION ABLATION OUTCOMES IN PATIENTS WITH NORMAL LEFT VENTRICULAR DYSFUNCTION VS PATIENTS WITH REDUCED LEFT VENTRICULAR DYSFUNCTION

### **NATASHA JONES‐LEWIS** ^1,2^, LUKAH Q. TUAN^1,2,3^, LORI BELL^1,2^, ADRIANA TOKICH^1,2^, JENISH SHROFF^1,2,3^, ANUGRAH NAIR^1,2,3^, RAJEEV K. PATHAK^1,2,3,4^

#### ^1^Canberra Heart Rhythm, Canberra, Australia,^2^Canberra Heart Rhythm Foundation, Canberra, Australia,^3^Australian National University, Canberra, Australia,^4^University of Canberra, Canberra, Australia

**Introduction:** Pulmonary Vein Isolation catheter ablations for atrial fibrillation can result in increased left ventricular ejection fraction (LVEF) in patients presenting with atrial fibrillation (AF). This study aimed to evaluate the clinical outcomes and characteristics of AF catheter ablation on symptomatic patients who presented with normal LVEF (Group 1: EF >50%) and reduced LVEF (Group 2: EF <50%).

**Methods:** Of 314 patients between 2018‐2024, 98 patients with symptomatic AF underwent catheter ablation and were included in this study. Echocardiographic data and Holter data were analysed at baseline prior to ablation, early follow‐up and 1 year post ablation.

**Results:** Mean age was 68±9.3 with 59% male. At a mean follow up of 72 months ±6 months, LVEF improved significantly in group 2 whereas it remained the same in group 1. Patients in group also showed increased improvement in quality of life over the monitoring period with significantly increased exercise capacity. The following table shows the characteristics and outcomes of the 2 groups.

**Conclusions:** The findings suggest that PVI catheter ablation can significantly improve LVEF in patients with reduced baseline EF. Careful monitoring and routine monitoring of these patients is important to ensure they remain free from AF.

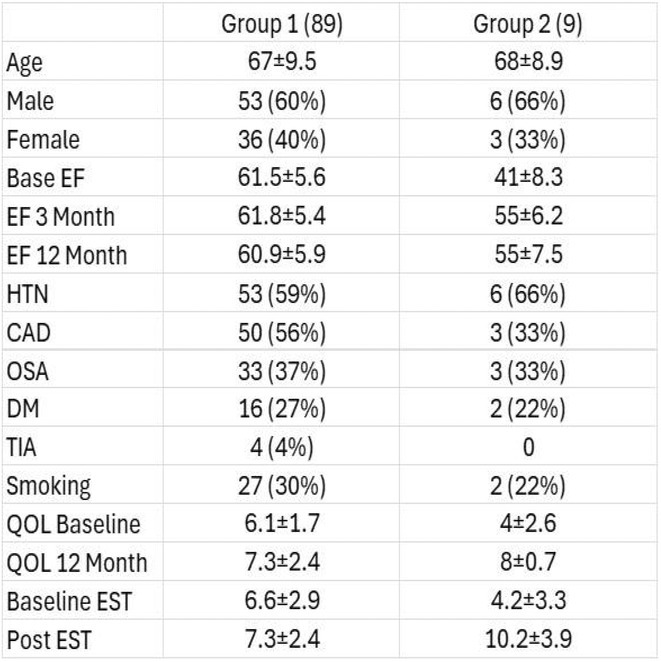

## HIGHER DETECTION OF ATRIAL FIBRILLATION WITH 14‐DAY ECG MONITORING THAN 1‐DAY HOLTER IN INDIVIDUALS WITH PALPITATION WITH HIGH RISK OF STROKE

### **BOYOUNG JOUNG** ^1^, EUE‐KEUN CHOI^2^, IL‐YOUNG OH^3^, MIN KIM^4^, KYOUNG‐MIN PARK^5^, GI‐BYOUNG NAM^6^, JONG SUNG PARK^7^, KI HONG LEE^8^, JUNBEOM PARK^9^, JAEMIN SHIM^10^

#### ^1^Yonsei University Severance Hospital, Seoul, Korea, Republic of,^2^Seoul National University Hospital, Seoul, Korea, Republic of,^3^Seoul National University Bundang Hospital, Bundang, Korea, Republic of,^4^Chungbuk National University Hospital, Cheungju, Korea, Republic of,^5^Samsung Medical Center, Seoul, Korea, Republic of,^6^Asan Medical Center, Seoul, Korea, Republic of,^7^Dong‐A University Medical Center, Busan, Korea, Republic of,^8^Chonnam National University Hospital, Gwangju, Korea, Republic of,^9^Ewha Womans University Mokdong Hospital, Seoul, Korea, Republic of,^10^Korea University Hospital, Seoul, Korea, Republic of

**Introduction:** Atrial fibrillation (AF) in adults is the most common sustained arrhythmia and routinely goes undiagnosed because they are often transient and asymptomatic. Effective diagnosis and treatment can substantially reduce the morbidity and mortality associated with AF. The MEMO Patch (Huinno Co., Ltd, Seoul, Korea) is a novel, single‐lead electrocardiographic (ECG), lightweight, continuously recording ambulatory adhesive patch monitor suitable for detecting cardiac arrhythmias in patients referred for ambulatory ECG monitoring.

**Methods:** We conducted a prospective, randomized, multicenter study of 1000 patients to assess the diagnostic sensitivity of 1‐day versus long‐term (8 to 14 days) patch‐type Holter monitoring to detect AF. Patients 75 years of age or older, or those at high risk of stroke (non‐gender CHA_2_DS_2_‐VA score ≥ 2) with no evidence of AF underwent 1:1 randomization. The primary outcome was the rate of detection of AF more than 30 seconds which resulted in the initiation of additional OAC therapy as per usual clinical practice. Data were analyzed according to the intention‐to‐treat principle.

**Results:** The study was terminated after enrolling 580 individuals (mean age 67.7 ± 9.6 years, 72.0% female, CHA_2_DS_2_‐VASc score 2.8 ± 1.1) due to a significant difference in AF detection rates. AF was identified in 6.4% of patients in the long‐term monitoring group (16 patients) compared to 1.1% in the control group (3 patients) (odds ratio, 6.3; 95% confidence interval [CI], 1.8 to 22.0; P < 0.001). Withdrawal rates were similar between the two groups, with 8.1% and 10.1% in the 1‐day and long‐term monitoring groups, respectively (p = 0.40).

**Conclusions:** In symptomatic individuals aged ≥ 75 years or those at high risk of stroke, long‐term ECG monitoring with a patch‐type device proved superior to conventional 1‐day monitoring for detecting AF. Prolonged duration monitoring for the detection of arrhythmia events using single‐lead, less‐obtrusive, adhesive‐patch monitoring platforms could replace conventional Holter monitoring in patients referred for ambulatory ECG monitoring.

## LONG‐TERM INCIDENCE OF ARRHYTHMIC DISEASE IN YOUNG OBSTRUCTIVE SLEEP APNEA PATIENTS BY TREATMENT METHODS

### **LAE‐YOUNG JUNG**, SUNGWON KIM, DONGHYUN KIM, JIN‐SIL CHOI, SANG‐HYO KIM

#### Jeonbuk National University Hospital, Jeonju, Korea, Republic of

**Introduction:** Obstructive sleep apnea (OSA) is a known risk factor for cardiac arrhythmias, with continuous positive airway pressure (CPAP) therapy being the primary non‐invasive treatment. However, patient compliance with CPAP remains a challenge. This study examines whether surgical intervention can reduce the incidence of arrhythmias in OSA patients.

**Methods:** Data from the Korean Health Insurance (January 2009 ‐ December 2020) were analyzed, involving 359,851 OSA patients (85.4% men, aged 60 years old or younger: 94.2%). Propensity score matching (PSM) at a 1:4 ratio compared surgical (23,533) and non‐surgical (94,132) patients, focusing on arrhythmia occurrence over five years.

**Results:** The cohort that underwent surgery for OSA consistently exhibited a lower incidence of arrhythmias than the non‐surgical cohort. In the PSM analysis, a statistically significant reduction in the incidence of AF, premature beats, and sudden cardiac arrest was observed in groups of patients who underwent surgery for OSA. Among those who developed AF, the group that underwent surgery for OSA had a fivefold higher incidence than the non‐surgical group (hazard ratio [HR] 5.384, 95% confidence interval [CI] 3.459‐8.381). The incidence of premature beats was more than four times higher in the non‐surgical group (HR 4.284, 95% CI 2.96‐6.177, p<0.0001), and sudden arrest was more than 24x higher in the non‐surgical group (HR 24.089, 95% CI 3.348‐173.312).

**Conclusions:** Surgical intervention for OSA appears to reduce arrhythmias in young patients, suggesting a potential preventative strategy. Further research is necessary to confirm these findings.

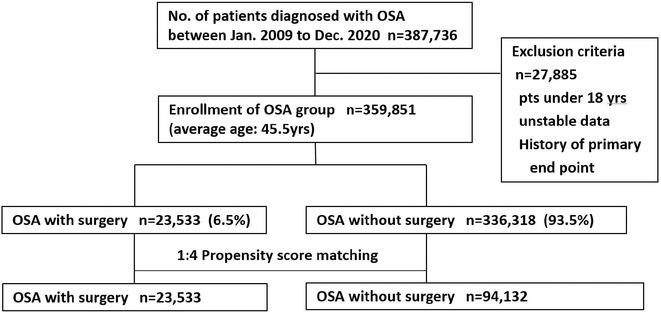

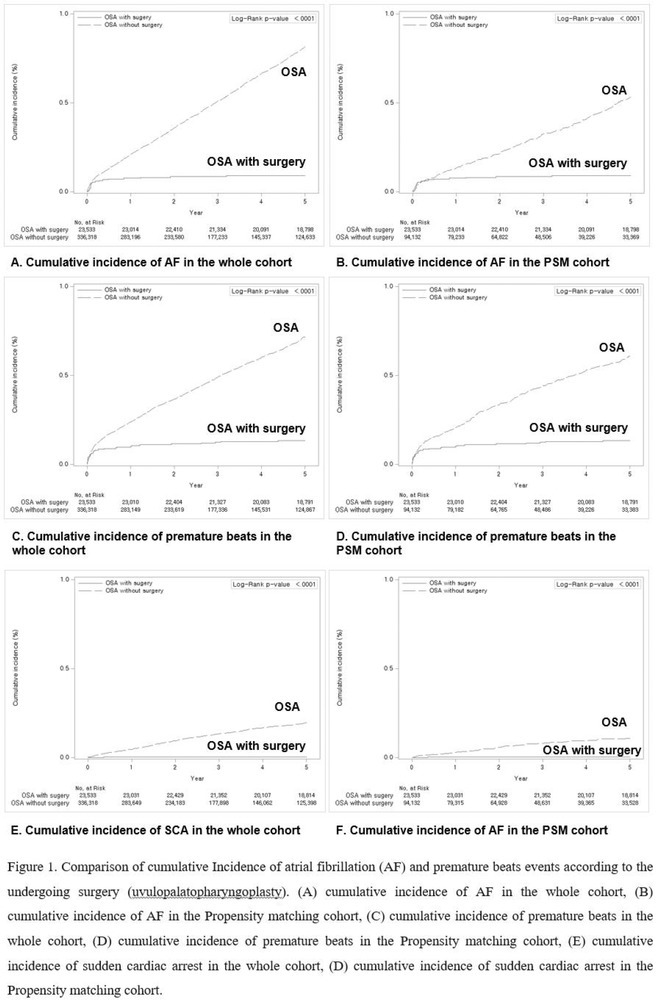

## ULTRASOUND‐GUIDED AXILLARY VEIN ACCESS AND INCISION LINE DECISION FOR THE IMPLANTATION OF CARDIAC IMPLANTABLE ELECTRONIC DEVICE LEADS

### **MIN‐SU JUNG**, JONGMIN HWANG, TAE‐WAN CHUNG, HYOUNG‐SEOB PARK

#### Keimyung University Dongsan Hospital, Daegu, Korea, Republic of

**Introduction:** Using surface landmark methods along with Byrd's first rib/fluoroscopy approach for incision line making and axillary vein puncture is the mainstream strategy for the implantation of cardiac implantable electronic device (CIED) leads. However, there are considerable inter‐individual variations in the three‐dimensional relationship between skin‐clavicle‐axillary veins. We have conducted research to determine the optimal incision line. We evaluated the “ultrasound‐guided axillary vein access and incision line decision” method for the Implantation of CIED Leads.

**Methods:** We performed the procedure as illustrated in the Figure. Since the wire is already retained, we can clearly see the course of the cephalic‐axillary‐subclavian vein. Therefore, an accurate assessment of the spatial relationship between the axillary and cephalic veins, as well as a precise determination of the point where the needle enters the axillary vein is possible. Additionally, there is an almost perfect correlation between the incision site and the entry point where the needle passes through the muscle for puncture.

**Results:** A total of 308 patients from June 2019 to September 2023 who received new CIED implantation at our center were enrolled. Historical age‐, sex‐matched cohort from our hospital were compared, and there were two instances of lead fracture, two severe hematomas due to arterial puncture, and two pneumothoraces in the historical cohort. However, such complications did not occur with this method.

**Conclusions:** Ultrasound‐guided axillary vein access and incision line decision is a very safe and effective procedural method for the implantation of CIED leads.

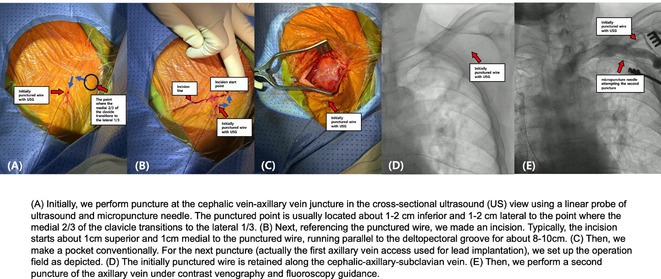

## ASSOCIATIONS OF LEFT VENTRICULAR FALSE TENDON WITH SUDDEN CARDIAC ARREST AND VENTRICULAR FIBRILLATION

### **MOONKI JUNG** ^1^, JAE‐SUN UHM^2^, HANJIN PARK^2^, DAEHOON KIM^2^, TAE‐HOON KIM^2^, HEE TAE YU^2^, BOYOUNG JOUNG^2^, HUI‐NAM PAK^2^, MOON‐HYOUNG LEE^2^

#### ^1^Chung‐Ang University, Seoul, Korea, Republic of,^2^Yonsei University, Seoul, Korea, Republic of

**Introduction:** Left ventricular false tendon is fibromuscular structures known to be associated with arrhythmogenic manifestations such as premature ventricular complexes and ventricular tachycardia (VT). However, the association between false tendon and sudden cardiac arrest (SCA) is not well‐established. This study aimed to clarify the relationship between false tendon and SCA.

**Methods:** We retrospectively reviewed medical histories, laboratory data, electrocardiograms, echocardiograms, coronary angiography, and cardiac CT/MRI for 45 patients with SCA. Prevalence of false tendon, the connection of false tendon with papillary muscle (PM), and association with ventricular fibrillation (VF) were compared between patients with SCA of unknown cause and those with known causes. False tendon is defined as stringlike structure with a free intracavitary course connected to ventricular walls and/or PM.

**Results:** Among the 45 patients, 32 had SCA of unknown cause (mean age, 53.8±19.0 years; 23 males), and 13 had SCA with known causes (mean age, 62.7±12.0 years; 12 males). Prevalence of false tendon was higher in the unknown‐cause group compared to the known‐cause group (62.5% vs. 38.5%, p=0.254). In patients with SCA of unknown cause, the VF occurrence rate was 65.6%, particularly higher in those with false tendon connected to PM (42.9% vs. 18.2%, p=0.315). False tendon emerged as independent predictors for SCA without identified causes (adjusted odds ratio [aOR] 2.33, 95% CI 0.56‐10.40, p=0.249). In patients with false tendon connected to PMs under 60 years of age, false tendon was independent predictor for VF in SCA without identified causes (age < 60 years, aOR 10.62, 95% CI 1.66‐101.05, p=0.020; FT to PM, aOR 10.91, 95% CI 1.05‐345.62, p=0.088).

**Conclusions:** False tendon is associated with SCA of unknown cause. Presence of false tendon connected to PM is identified as an independent predictor for VF in sudden cardiac arrest cases without identified causes.

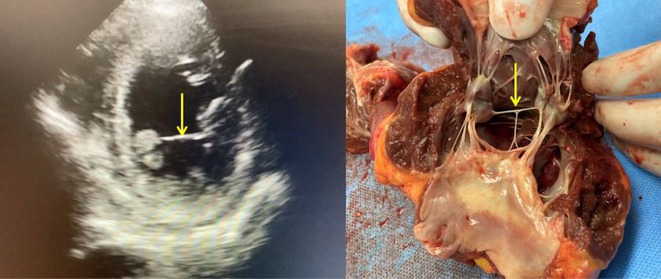

## ASSESSMENT OF ECG WAVEFORMS FROM INSERTABLE CARDIAC MONITORS AND SERUM POTASSIUM CONCENTRATIONS

### **ABHIJIT KADROLKAR** ^1^, RAJAT DEO^2^

#### ^1^Medtronic, Mounds View, MN,^2^Perelman School of Medicine at the University of Pennsylvania, Philadelphia, PA

**Introduction:** Safe and effective titration of guideline directed medical therapies (GDMT) for heart failure patients requires frequent monitoring of serum potassium (K^+^) levels. In particular, renin‐angiotensin‐aldosterone system inhibitors and β‐blockers can lead to hyperkalemia. Recent AHA guidelines recommend titration of these therapies every 1 to 2 weeks, necessitating frequent blood draws. Prior research has highlighted possibility of reducing blood draws by non‐invasively estimating serum K^+^ from ECG. We sought to develop an algorithm to estimate serum K^+^ by using ECG waveforms from an Insertable Cardiac Monitor (ICM).

**Methods:** A population‐wide, deep learning model was developed to estimate serum K^+^ from ICM ECG waveforms. Data from the Monitoring in Dialysis multicenter study were used to develop the model. Study included dialysis patients, who were implanted with a Reveal‐XT or LINQ ICM and followed over a 6‐month period. Dialysis was 3 times/week with ICM interrogation (ECG) and blood draws performed simultaneously, before and after every dialysis session. Calibrating blood draws were simultaneous ECG and K+ data pairs at a minimum of two different time‐points. The deep learning model was further fine‐tuned to every individual patient using a small number of blood draws for calibration.

**Results:** Of the 18 patients in our study, we evaluated an average of 25 data pairs (simultaneous ECG and K^+^) per patient. Fine‐tuned deep learning models estimate serum K^+^ with mean square error of 0.4 mEq/L. Models demonstrate 92% sensitivity in estimating K^+^ from ECG within ±1 mEq/L with 6 calibrating blood draws per new patient (Figure 1). Results represent improvement in estimation performance, over past efforts.

**Conclusions:** ICM generated ECG waveforms can be assessed to estimate serum K^+^. Future work will require validation of these findings in a larger group of patients. These methods may enable non‐invasive monitoring of serum K^+^ and may facilitate rapid up‐titration of GDMT drugs with fewer blood draws.

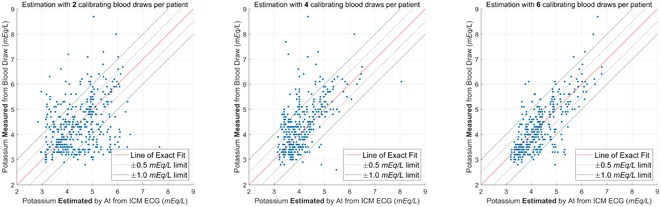

## A MATHEMATICAL MODEL FOR DERIVATION OF ELEVATED‐ELECTRODE‐PLACEMENT ELECTROCARDIOGRAM (EEP‐ECG) FROM A STANDARD 12‐LEAD ELECTROCARDIOGRAM FOR THE DIAGNOSIS OF BRUGADA SYNDROME (BRS)

### **KARAN KALANI**, RAJA SELVARAJ

#### Jawahar Institute of Postgraduate Medical Education and Research, Puducherry, India

**Introduction:** BrS is an inherited arrhythmogenic disorder associated with ventricular fibrillation and sudden cardiac death. Placement of right precordial leads in higher intercostal spaces (EEP‐ECG) increases the yield of BrS. Since such lead placement may be cumbersome and the ECG pattern associated with BrS may be transient, we derived a model to predict an EEP‐ECG from a standard 12‐lead ECG.

**Methods:** Using 2 identical ECG recorders (BioCare iE 12A), a 16‐channel recording was obtained in every patient. One recorded the standard 12‐leads while the other recorded the 4 elevated leads in the 2^nd^ and 3^rd^ ICS (intercostal spaces) (EEP‐ECG) (Figure, top left image). Using the data from the training group, a linear regression matrix was created for derivation of the EEP‐ECG from the standard leads (Figure, bottom image). The model was then tested in the validation group. The derived elevated leads were compared to the actually recorded elevated leads. Goodness of fit was assessed by the correlation coefficient and by visual assessment on a scale of 1‐3 (1=No match, 2=reasonable match, 3=good match) by two independent cardiologists (Figure, top right image).

**Results:** 43 participants (23 in training group and 20 in validation group) were included. 8 BrS patients were a part of the study (All type 1 BrS, 2 in training, 6 in validation group). The regression model was derived from the training set. In the validation set, the actual and derived leads were well correlated with r >0.85 for all but 2 participants (1 BrS, 1 Non‐BrS). On visual assessment of fit, the average score was 2.44+‐0.68 suggestive of a good performance of the model. The scores for the non‐BrS participants were numerically higher than those with BrS (2.59+‐0.62 vs 2.06+‐0.69).

**Conclusions:** A linear model was created to derive the elevated ECG leads from the standard 12 lead ECG. The EEP‐ECG derived from the model reasonably predicts the actual EEP‐ECG. Use of the model on new or previously recorded ECGs might help in diagnosing BrS.

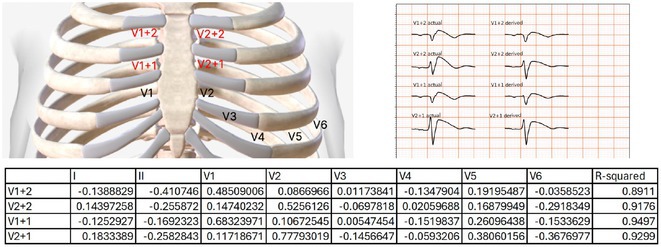

## EARLY SINGLE CENTRE EXPERIENCE IN PULSED FIELD ABLATION FOR ATRIAL FIBRILLATION

### **SURAYA HANI KAMSANI** ^1,2^, ROHITH STANISLAUS^1^, MING YOONG LOW^1^, MATHAN MUNUSAMY^1^, ZUNIDA ALI^1^, NOOR ASYIKIN SAHAT^1^, AMIRZUA AHMAD SAID^1^, AZLINA DAUD^1^, MOHAMAD HALMY AZIMAN^1^, NIRPAL SINGH^1^, ROSILA REBO^1^, HANI JALINA MOHAMED ZAMBERI^1^, AZLAN HUSSIN^1^, SURINDER KAUR KHELAE^1^

#### ^1^National Heart Institute, KUALA LUMPUR, Malaysia,^2^Centre for Heart Rhythm Disorders, University of Adelaide, Adelaide, Australia

**Introduction:** Pulsed field ablation (PFA) is an emerging, mainly non‐thermal and tissue‐selective ablative technology. We aim to illustrate our early experience in a single centre in Malaysia of PFA for AF ablation.

**Methods:** Consecutive patients undergoing PFA procedure for paroxysmal or persistent AF between May 2023 and March 2024 at a single centre were included. Baseline characteristics, procedural details and acute clinical outcomes were documented.

**Results:** A total of 100 patients (73% males, mean age: 62±10years) underwent PFA procedures for pulmonary vein isolation during the study period. Majority (73%) of these patients had paroxysmal AF and 27% had persistent AF. Hypertension is the most common comorbidity (70%) followed by dyslipidemia (53%), diabetes (37%) and renal impairment (10%); with 62% of patients having CHADSVASC2 score more than 2. Mean baseline left ventricular ejection fraction was 53.7±9.3% with mean left atrial volume index of 36.0±11.8. The most common AF symptom was palpitation (93%), while chest pain occurred in 12%, presyncope (7%), dizziness (2%) and syncope (2%). The procedures were performed under general anaesthesia in 93% of cases and deep sedation in 7%. Pulmonary veins were successfully isolated acutely in 97%, with partial success in 2% and procedure was abandoned in 1%. Mean procedure time was 53.9±27.1 minutes while total fluoroscopy time was 21.7±10.3 minutes. The procedure was safely performed in 98% of patients, while 2 procedures were complicated with pericardial effusion. All of the patients were discharged well the following day post‐procedure.

**Conclusions:** Our early experience showed that PFA can be safely performed in majority of patients with comparable procedural duration and acute efficacy to other high volume centres.

## PRE‐PROCEDURAL CARDIAC MAGNETIC RESONANCE IMAGING FOR DETECTION OF SLOW CONDUCTION CORRIDORS FOR VENTRICULAR TACHYCARDIA ABLATION

### **SURAYA HANI KAMSANI** ^1,2^, ROHITH STANISLAUS^1^, YOGESH HIWARE^3^, MING YOONG LOW^1^, MATHAN MUNUSAMY^1^, ZUNIDA ALI^1^, NOOR ASYIKIN SAHAT^1^, AMIRZUA AHMAD SAID^1^, AZLINA DAUD^1^, MOHAMAD HALMY AZIMAN^1^, NIRPAL SINGH SACHDEV^1^, SHIRLEY LOW^3^, ROSILA REBO^1^, HANI JALINA MOHAMED ZAMBER^1^, SURINDER KAUR KHELAE^1^, AZLAN HUSSIN^1^

#### ^1^National Heart Institute, Kuala Lumpur, Malaysia,^2^Centre for Heart Rhythm Disorders, University of Adelaide, Adelaide, Australia,^3^Abbott (Malaysia), Kuala Lumpur, Malaysia

**Introduction:** Scar assessment prior to VT ablation with late gadolinium enhancement cardiac magnetic resonance (LGE‐CMR) could aid in identifying relevant slow conduction corridors in different layers of the ventricle. We describe the utility of integrating pre‐procedural CMR assessment with our current VT ablation workflow.

**Methods:** Patients who underwent LGE‐CMR assessment with ADAS3D LV software prior to VT ablation were retrospectively reviewed. Ventricular scars and slow conduction corridors were identified. During VT mapping and ablation procedure, utilizing the ADAS corridors as guide, isochronal late activation maps (ILAM) during sinus rhythm and/or VT activation maps were created. Correlation between the ADAS corridors and deceleration zone (DZ) on ILAM were made to determine suitable target for ablation. Patient and procedural characteristics as well as short‐term outcomes were documented.

**Results:** Twenty‐six VT ablation procedures were conducted with ADAS3D LGE‐CMR guidance for 25 patients (88% males, mean age of 62.4 ± 12.1 years). Ischemic VT was present in 64% of patients (n=16), while 36% (n=9) had non‐ischemic cardiomyopathy. Mean LVEF was 34.5 ± 9.1%. On average, 2.3 ± 1.3 slow conduction corridors were identified per LGE‐CMR image, with 88.5% matching the DZ found on ILAM or critical isthmus on VT activation map. These corridors were predominantly localized to the endocardial layer (96.2%), with 61.5% in the mid and 57.7% in epicardial layers. In non‐ischemic VT, corridors were found in all three layers in 44.4% whereas these channels were localized in all layers in 47.1% ischemic VT (p=0.90). Total procedural time was 168.7 ± 45.6 mins, and all procedures were successfully ablated with minimal complications. After a follow up period of 2.9 ± 1.5 months, 85.7% of the patients remained free of recurrence.

**Conclusions:** Pre‐procedural ADAS LGE‐CMR for slow conduction corridor identification is useful in facilitating an efficient, safe and successful VT ablation. In most cases, more than one corridors were identified with good correlation to DZ and critical VT isthmus during electroanatomical mapping.

## PROARRHYTHMIC EFFECTS OF SOTALOL DURING THERAPY INITIATION: A SYSTEMATIC REVIEW AND META‐ANALYSIS

### **SURAYA HANI KAMSANI** ^1,2^, MELISSA MIDDELDORP^1,3^, ELNAZ SHAHMOHAMADI^1^, SHAUN EVANS^1^, PRASH SANDERS^1^

#### ^1^Centre for Heart Rhythm Disorders, University of Adelaide, Adelaide, Australia,^2^National Heart Institute, Kuala Lumpur, Malaysia,^3^Department of Cardiology, University of Groningen, University Medical Center Groningen, Groningen, Netherlands

**Introduction:** Sotalol posseses potent potassium channel‐blocking properties, raising concerns about its proarrhythmic potential, particularly torsades de pointes (TdP), and other adverse effects like bradyarrhythmia and increased mortality risk. This meta‐analysis examines whether patients are at increased risks of proarrhythmic events during sotalol initiation phase.

**Methods:** A systematic search of published literature was conducted in PubMed, Embase and Cochrane database from the earliest date possible up to December 2023. The main keyword used was ‘sotalol’. Meta‐analysis of proportions was utilized to combine the incidence of arrhythmia types, mortality and drug discontinuation across the groups. Random‐effects models were employed irrespective of heterogeneity.

**Results:** This review presented the findings across 29,046 individuals exposed to sotalol from 51 unique publications comprising of 25 randomised trials and 26 observational studies. The mean age of participants were 59.9±7.5 years with an average of 67.7% male participants. The most common route of sotalol administration was oral (42 out of 51 studies), with only 9 studies utilising intravenous (IV) infusion. Dosages at initiation varied during both oral and IV administration. The incidence of TdP was generally low. Out of 32 studies with 4,549 patients exposed to sotalol, 15 studies did not document any TdP events while 17 studies reported an incidence between 0.8 to 4.9%. The rate of bradycardia in these papers varied between 0 (3 studies) to 27.8%; affecting a total of 296 patients from an accumulative sample size of 4,120 (7.2%). Most patients were asymptomatic and only required dose adjustment. The pooled mortality rate among the patients receiving Sotalol was 1.8% (1.7‐2.0%; I2 : 0.0%, p < 0.001) by the random effects model.

**Conclusions:** Significant proarrhythmic adverse events including symptomatic bradycardia, new or increased VA and early mortality were low during the initiation phase of sotalol. Further prospective studies are needed to evaluate the safety of outpatient sotalol initation with careful patient selection, especially in the era of digital health.

## SAFETY AND FEASIBILITY OF ACCESSING THE EPICARDIUM USING CARBON DIOXIDE INSUFFLATION FOR VENTRICULAR TACHYCARDIA ABLATION

### **SURAYA HANI KAMSANI** ^1,2^, ROHITH STANISLAUS^1^, YOGESH HIWARE^3^, LOW MING YOONG^1^, MATHAN MUNUSAMY^1^, NIRPAL SINGH SACHDEV^1^, ZUNIDA ALI^1^, NOOR ASYIKIN SAHAT^1^, AMIRZUA AHMAD SAID^1^, AZLINA DAUD^1^, MOHAMAD HALMY AZIMAN^1^, SHIRLEY LOW^3^, ROSILA REBO^1^, HANI JALINA MOHAMED ZAMBERI^1^, SURINDER KAUR KHEALE^1^, AZLAN HUSSIN^1^

#### ^1^National Heart Institute, Kuala Lumpur, Malaysia,^2^Centre for Heart Rhythm Disorders, University of Adelaide, Adelaide, Australia,^3^Abbott (Malaysia), Kuala Lumpur, Malaysia

**Introduction:** Epicardial mapping and ablation is often crucial to ensure successful ventricular tachycardia (VT) ablation procedure. Standard “dry tap” technique to access the pericardial space carries a high risk of complications. A new technique involving insufflation of the epicardial space via intentional coronary vein exit has been introduced. This is an update to our single centre registry involving all‐comer from March 2022 to February 2024 who underwent pericardial carbon dioxide (CO2) insufflation technique for epicardial VT ablation.

**Methods:** After femoral or internal jugular venous access, the coronary sinus (CS) was cannulated with a decapolar catheter via a steerable sheath. CS venogram was done to determine suitable branches. Intentional distal CS branch exit was performed using a stiff coronary guidewire and microcatheter. A small volume of contrast was injected to confirm successful epicardial access prior to CO_2_ insufflation. After sufficient CO_2_ insufflation was observed on fluoroscopy, subxiphoid pericardial puncture was then performed with a Tuohy needle. A guidewire was inserted and looped in the pericardial space and a steerable sheath was subsequently advanced over the wire. The sheath was connected to a negative pressure drainage. The epicardial surface was mapped and ablation performed over the area of interest. Intrapericardial corticosteroid was infused via the epicardial sheath prior to its removal at the end of the procedure.

**Results:** In total, 7 patients (100% males, mean age 56.6±15.1 years) were included in the analysis. 3 patients were diagnosed with non‐ischemic dilated cardiomyopathy, two patients had arrhythmogenic right ventricular cardiomyopathy and one patient had ischemic cardiomyopathy with persistent left ventricular thrombus. Overall mean procedural time was 235.0 ± 56.6 minutes. An average volume of 195.8 ± 75.7 ml of CO_2_ was used. All 7 patients did not experience any intraprocedural complication.

**Conclusions:** Intentional coronary vein exit for CO_2_ insufflation allows the creation of a “buffer” zone to facilitate a safe epicardial access for VT mapping and ablation.

## SINGLE CENTRE REAL‐WORLD SHORT‐TERM OUTCOME OF PULSED FIELD ABLATION FOR ATRIAL FIBRILLATION

### **SURAYA HANI KAMSANI** ^1,2^, ROHITH STANISLAUS^1^, YOGESH HIWARE^3^, LOW MING YOONG^1^, MATHAN MUNUSAMY^1^, NIRPAL SINGH SACHDEV^1^, ZUNIDA ALI^1^, NOOR ASYIKIN SAHAT^1^, AMIRZUA AHMAD SAID^1^, AZLINA DAUD^1^, MOHAMAD HALMY AZIMAN^1^, SHIRLEY LOW^3^, ROSILA REBO^1^, HANI JALINA MOHAMED ZAMBERI^1^, SURINDER KAUR KHELAE^1^, AZLAN HUSSIN^1^

#### ^1^National Heart Institute, Kuala Lumpur, Malaysia,^2^Centre for Heart Rhythm Disorders, University of Adelaide, Adelaide, Australia,^3^Abbott (Malaysia), Kuala Lumpur, Malaysia

**Introduction:** Pulsed field ablation (PFA) has garnered much publicity recently as the latest modality for atrial fibrillation (AF) management. Early clinical outcomes from European and Australian centers shows promise, but there is a lack of data from Asian regions. This single centre study aims to determine the safety and short‐term outcomes of the procedure in real‐world AF patients.

**Methods:** All‐comer AF patients who underwent PFA procedure between May to December 2023 were prospectively included. Procedural details and short‐term clinical outcomes were collected.

**Results:** Throughout the study period, 75 patients (mean age: 62.2±9.6years, 69.3% males) underwent pulmonary vein isolation (PVI) procedures using PFA. Out of these, 74.7% (n=56) had paroxysmal AF while 25.3% (n=19) had persistent AF. Procedures were performed under general anesthesia for most cases (93.3%, n=70) while (4.0%, n=5) were performed under deep sedation. Mean procedural duration was 56.8±25.6 minutes while fluoroscopy time was 24.0±10.6 minutes. Acute PVI was successfully achieved in 97.3% of the procedures. Acute complication of pericardial effusion occurred in 2.7%. Patients were discharged on anti‐arrhythmic drugs in 50% of PAF and 57.9% of persistent AF. After a mean follow up of 234.3±87.0 days, recurrence of AT/AF occurred in 8.9% of paroxysmal AF and 21.1% of persistent AF. The Kaplan‐Meier estimate for freedom from atrial arrhythmias at 180 days after the procedure was 91% (95% confidence interval 80%‐96%) for PAF and 79% (95% confidence interval 53%‐92%) for persistent AF.

**Conclusions:** Our real‐world data demonstrates that PFA could be safely performed in relatively short duration with satisfactory short‐term outcomes for both PAF and persistent AF patients.

## THE UTILITY OF OVER‐THE‐WIRE MULTIELECTRODE CATHETER FOR TRANSCORONARY MAPPING IN LV SUMMIT VPC

### **SURAYA HANI KAMSANI** ^1,2^, ROHITH STANISLAUS^1^, YOGESH HIWARE^3^, LOW MING YOONG^1^, MATHAN MUNUSAMY^1^, NIRPAL SINGH SACHDEV^1^, ZUNIDA ALI^1^, NOOR ASYIKIN SAHAT^1^, AMIRZUA AHMAD SAID^1^, AZLINA DAUD^1^, MOHAMAD HALMY AZIMAN^1^, SHIRLEY LOW^3^, ROSILA REBO^1^, HANI JALINA MOHAMED ZAMBERI^1^, SURINDER KAUR KHELAE^1^, AZLAN HUSSIN^1^

#### ^1^National Heart Institute, Kuala Lumpur, Malaysia,^2^Centre for Heart Rhythm Disorders, University of Adelaide, Adelaide, Australia,^3^Abbott (Malaysia), Kuala Lumpur, Malaysia

**Introduction:** Ventricular arrhythmias arising from left ventricular summit (LVS) region is challenging to map and ablate due to its complex relationship with surrounding structures. The region lies in close proximity to the bifurcation of the left anterior descending (LAD) and the left circumflex (LCx) arteries and bounded laterally by the great cardiac vein. Thus, proper anatomical assessment and detailed activation mapping to determine the appropriate ablation target is crucial. The use of over‐the‐wire multielectrode catheter to facilitate transcoronary artery and venous system mapping in this region has not been previously described.

**Methods:** Two patients (a 53‐year‐old‐male and a 56‐year‐old female) presented with symptomatic LVS ventricular premature contractions (VPC) underwent simultaneous transcoronary artery and venous system mapping. Coronary angiography was performed prior to placing an over‐the‐wire decapolar catheter to create a detailed activation map of the VPC in the left coronary arteries. Following this, a deflectable sheath was advanced into the coronary sinus and contrast venography was performed. The anterior interventricular vein (AIV) was then cannulated using the over‐the‐wire multielectrode catheter to create a high‐density activation map of the VPC in the coronary venous system. The HD Grid multipolar mapping catheter was also employed to interrogate both the left and right ventricular outflow tracts.

**Results:** The earliest activation site for both patients were at the inaccessible area of LVS, bounded by the LAD and LCx superior to great cardiac vein and AIV. Successful ablation was achieved at the endocardial aspect of LV myocardium beneath the left coronary cusp. Coronary angiogram was repeated to confirm the patency of coronary arteries post ablation. The procedures were completed within 150.0±70.7 minutes with flurosocopy time of 35.0±19.8minutes.

**Conclusions:** The use of over‐the‐wire multielectrode catheter in transcoronary mapping aid in determining the proximity of the ablation target to major coronary vessels. This helps to ensure the safe delivery of radiofrequency energy during the ablation procedure.

## EFFECT OF ANGIOTENSIN RECEPTOR NEPRILYSIN INHIBITORS IN CATHETER ABLATION THERAPY FOR ATRIAL FIBRILLATION

### **SHOZO KANEKO**, YUICHIRO TSURUTA, YUTA TSURUSAKI, KOHEI MATSUNAGA, HITOSHI SUMI, TADASHI HOSHIYAMA, HISANORI KANAZAWA, KENICHI TSUJITA

#### Kumamoto University Hospital, Kumamoto, Japan

**Introduction:** Angiotensin receptor neprilysin inhibitors (ARNI) is one of the heart failure therapeutic drugs and lowers blood pressure, improves excessive water retention. ARNI also raises Atrial natriuretic peptide (ANP) levels. Higher ANP levels during heart failure have been reported to be a predictor in assessing recurrence after catheter ablation for atrial fibrillation (AF). It is not clear how ANP levels are involved in preventing recurrent of AF. We investigated how ANP levels are related to left atrial function and recurrent AF.

**Methods:** Of the 497 consecutive patients undergoing AF ablation from April 2021 to March 2023, 115 patients with AF (paroxysmal AF in 54 patients) who having ARNI or angiotensin II receptor blocker (ARB), angiotensin converting enzyme inhibitor (ACE‐I) for heart failure and undergoing first AF ablation were studied (mean age 71 ± 9 years old, 61% male). Preoperatively, we evaluate brain natriuretic peptide (BNP), ANP, and left atrial function by blood samples and echocardiography. Similarly, we performed these inspections in six months and one year after catheter ablation therapy. We divided the patients into ARNI and ARB or ACE‐I groups and compared these results and examined differences in recurrence.

**Results:** The medication of ARNI and ARB or ACE‐I were observed in 31 patients (ARNI group) and 84 patients (ARB or ACE‐I group). Preoperatively, ANP levels were significantly higher in ARNI group (101.8 ± 121.6 pg/ml versus 93.8 ± 106.9 pg/ml, p=0.042), but BNP levels and left atrial function were not different between the two groups (94.7 ± 109.9 pg/ml versus 90.5 ± 105.4 pg/ml, p=0.221). Postoperatively, BNP levels and improvement rate of left atrial function in ARNI group were significantly higher than ARB or ACE‐I group (p=0.001 and p=0.013). Moreover, the recurrence rate was also lower in ARNI group (p=0.004).

**Conclusions:** Patients taking ARNI had a lower recurrence rate and a higher rate of improvement in left atrial function after AF ablation than patients taking ARB or ACE‐I. ARNI may be a new upstream therapy in AF therapy.

## RACIAL DIFFERENCES IN BLEEDING RISKS AMONG PATIENTS WITH ATRIAL FIBRILLATION: AN ECOLOGICAL STUDY COMPARING SUBJECTS IN KOREA AND THE UNITED KINGDOM

### **DONG‐SEON KANG** ^1^, PIL‐SUNG YANG^2^, DAEHOON KIM^3^, EUNSUN JANG^3^, HEE TAE YU^3^, TAE‐HOON KIM^3^, JUNG HOON SUNG^2^, HUI‐NAM PAK^3^, MOON‐HYUNG LEE^3^, GREGORY Y.H. LIP^4^, BOYOUNG JOUNG^3^

#### ^1^Kangdong Sacred Heart Hospital, Seoul, Korea, Republic of,^2^CHA Bundang Medical Center, Seongnam, Korea, Republic of,^3^Yonsei University College of Medicine, Seoul, Korea, Republic of,^4^3Liverpool Centre for Cardiovascular Science at University of Liverpool, Liverpool, United Kingdom

**Introduction:** This study aimed to assess racial differences in bleeding risk among patients with atrial fibrillation (AF) using an ecological epidemiological approach, utilizing data sourced from Korea and the UK.

**Methods:** We included patients with AF from the Korean National Health Insurance Service‐Health Screening and UK Biobank who underwent health examinations between 2006 and 2010. The analysis involved 1786 East Asians (61.1% male, median age 60.0 years) and 4460 Caucasians (74.5% male, median age 64.0 years) were analyzed. The primary outcome was composed of intracranial hemorrhage and bleeding from the gastrointestinal, respiratory, and genitourinary systems.

**Results:** During follow‐up, the primary outcome events occurred in 114 East Asians and 427 Caucasians. East Asians had a 33% lower five‐year incidence rate compared to Caucasians (weighted incidence rate 1.35 vs. 2.02 per 100 person‐years; incidence rate ratio [IRR] 0.67, 95% confidence interval [CI] 0.45‐0.99). Contrary to bleeding from the gastrointestinal, respiratory, and genitourinary systems, which followed similar trends as the primary outcome, the incidence of intracranial hemorrhage tended to be higher among East Asians (weighted incidence rate 0.36 vs. 0.14 per 100 person‐years; IRR 2.63, 95% CI 0.89‐7.76). These results persisted even in patients naïve to antithrombotics. However, East Asians who were already taking antithrombotics at baseline showed no significant difference in the incidence of the primary outcome compared to Caucasians (weighted incidence rate 2.03 vs. 1.98 per 100 person‐years; IRR 1.02, 95% CI 0.52‐2.01).

**Conclusions:** This ecological study highlighted significant racial differences in the incidence of bleeding influenced by anatomical site and antithrombotic use. These findings underscore the necessity for race‐based tailored approaches in the management of patients with AF.

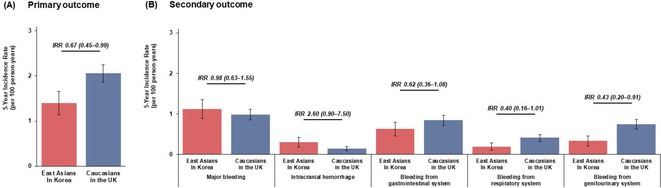

## COMBINING PRESSURE‐GUIDED BALLOON ABLATION WITH PREFREEZING TECHNIQUE FOR ENHANCED EFFICACY IN PULMONARY VEIN ISOLATION: A CASE REPORT

### **HONSA KANG**, TAKANORI WATANABE, KIYOSHI HIRONAGA

#### Fukuoka City Hospital, Fukuoka, Japan

**Introduction:** Cryoballoon ablation (CBA) is widely recognized for its effectiveness in pulmonary vein isolation worldwide. The Prefreezing Technique (PFT), involving balloon manipulation post‐cooling initiation, has demonstrated effectiveness. However, determining the initiation point of PFT and confirming occlusion often necessitate multiple contrast agent administrations. In contrast, Pressure‐guided Balloon Ablation enables continuous confirmation of balloon occlusion through balloon tip pressure measurement and observation of pressure waveform changes, without requiring contrast agents. Nonetheless, challenges in delineating anatomical positional relationships underscore the importance of avoiding intra‐pulmonary vein (PV) cooling.

**Methods:** NA

**Results:** Case: An 62‐year‐old man with symptomatic persistent atrial fibrillation underwent treatment by CBA. Right inferior pulmonary vein (RIPV) and left inferior pulmonary vein (LIPV) ablations were performed using pressure guidance. Right superior pulmonary vein (RSPV) and left superior pulmonary vein (LSPV) were treated with a combination of pressure guidance and PFT. For the upper PVs, balloon occlusion was conducted, and mean pressure and pressure waveforms were recorded pre‐ and post‐occlusion. Subsequently, the balloon was gradually retracted to the antrum, confirming the transition of the balloon pressure waveform to a non‐occlusion pattern. Cooling commenced from the point of waveform transition to non‐occlusion, followed by balloon pushing 5 seconds after cooling initiation. This method facilitated confirmation of pressure waveform changes post‐occlusion prior to freezing. Treatment was completed with a single cooling session for all four PVs without no contrast agent, and confirming PV isolation on left atrial post‐voltage mapping.

**Conclusions:** The integration of pressure‐guided ablation with PFT represents a promising strategy for enhancing the efficacy of Cryoballoon ablation, mitigating the limitations associated with contrast agent usage and improving treatment outcomes.

## DEVICE

### **NAYUN KANG**, MYUNGYONG LEE, DONGMIN KIM

#### Dankook univercity hospital, CHEONAN, Korea, Republic of

**Introduction:** Conduction system pacing has been suggested as an alternative for overcoming chronic right ventricular (RV) pacing‐related complications. Several nonrandomized multicenter studies have shown the feasibility and efficacy of LBBP as an alternative to biventricular pacing (BVP) in patients eligible for cardiac resynchronization therapy (CRT). In patients who are candidates for an implantable cardioverter‐defibrillator (ICD) and have an indication for CRT, implantation of a cardiac resynchronization therapy‐defibrillator (CRT‐D) is recommended. Recently, Left bundle branch area pacing (LBBAP) during conduction system pacing (CSP), including His bundle pacing and left bundle region pacing, has emerged as an alternative to BiVP for cardiac resynchronization. We report a patient underwent CSP and achieved the same effect as BiVP.

**Methods:** N/A

**Results:** A‐49‐years old man arrived at the emergency department cardiac arrest with successful resuscitation, Ventricular fibrillation and flutter, Left bundle‐branch block. Twelve‐lead electrocardiography showed left bundle branch block with regular beats of 58 beats per minute with QRS duration 141ms Two‐dimensional echocardiography showed global hypokinesia (ejection fraction, EF 42%) and Trivial TR (estimated RVSP 20.5mmhg). Coronary angiography showed 2 vessl deaseas. Given the impaired LV function and the deleterious effects of right ventricular apical pacing, Left bundle branch pacing‐optimized implantable cardioverter‐defibrillator (LOT‐ICD) was attempted. After LOT ICD implant QRS duration is decreaed to 116 ms.

**Conclusions:** In recent years, pacing of the conduction system (CSP) has emerged as a new standard pacing method for bradycardia indications, allowing for more physiological ventricular activation compared to conventional right ventricular pacing. CSP has also emerged as an alternative modality to conventional ventricular pacing for delivering cardiac resynchronization therapy (CRT) to patients with heart failure. For patients eligible for an implantable cardioverter‐defibrillator (ICD) and with an indication for CRT, the LOT‐ICD can serve as a BiVP for cardiac resynchronization.

## ECG

### **NAYUN KANG**, MYUNGYONG LEE, DONGMIN KIM

#### Dankook univercity hospital, CHEONAN, Korea, Republic of

**Introduction:** Delayed cardiac repolarization leading to prolongation of the QT interval is a well‐characterised precursor of arrhythmia.The purpose of this study was to analyze the effect of age on the ECG QT interval, an important predictor of cardiovascular mortality and drug‐induced cardiac arrhythmias

**Methods:** We designed a retrospective study. We have studied 69 patients we reviewed echocardiographic parameter also we measured QT and QTc parameter the resting 12‐lead ECG in these patient.

**Results:** A total of 69 patient age mean 55.3year±13.0. sex male 53(80.3%), female13(19.7%), smoking 23(35.9%), non‐smoking 41(64.1%), QTc interval 430.5ms±37.3, QT interval 391.4ms+±38.0, LVIDs32.1mm+±5.7, LVIDd 51.1mm+±5.3, EF 66%+±8.3, E/A ratio 1.04+±0.5, LAVI 30.9+± 11.9, SBPmmHg133+18. DBP 79mmHg±11, BMI 26.0+±4.7 QT interval was correlated with Age (r=0.259,p=0.037), QTc was correlated with LA size (r=0.293, p=0.018)

**Conclusions:** There is a positive correlation between QT interval and Age, between QTc and LA size

## CLINICAL SIGNIFICANCE OF VERY EARLY RHYTHM CONTROL BY CATHETER ABLATION IN PATIENTS WITH DEVICE‐DETECTED ATRIAL FIBRILLATION

### **YASUNORI KANZAKI**, ITSURO MORISHIMA, YASUHIRO MORITA, NAOKI WATANABE, NAOKI YOSHIOKA, NAOKI SHIBATA, YOSHIHITO ARAO, KAZUKI SHIMOJO, TAKUMA OHI, HIROKI GOTO, HOSHITO KARASAWA, YUTA NAKAGAWA, YUKI KAWASAKI, TATSUKI YOSHIE

#### Ogaki Muncipal Hospital, Ogaki, Japan

**Introduction:** Remote monitoring (RM) is recommended for patients with cardiac implantable electrical devices (CIEDs). Although the risks and benefits of oral anticoagulants (OACs) in patients with device‐detected atrial fibrillation (AF) have often been discussed, there is limited data on catheter ablation (CA) of device‐detected AF.

**Methods:** A total of 832 patients with CIEDs were systematically followed by RM. CA of AF was considered if the AF burden exceeded 50% / day. We studied 54 patients (77±9.1 years old, 41% female, CHADS2 score 2.1±1.2) whose one‐year follow‐up data were available among 81 patients who met the AF burden criteria.

**Results:** We performed AFCA in 22 patients and 32 continued monitoring only. All patients in the CA group and 28 patients (88%) in the monitoring group were on OACs (p=0.114). The patients in the CA group were younger than those in the monitoring group (71±7.2 vs. 81±8.6, p<0.001). The AF‐free survival was 63% in the CA group at one year. After 6 and 12 months, the AF burden was significantly decreased in the CA group (0.05 [0, 3.67] %, 0.04 [0, 0.98] %, respectively), while it remained the same in the monitoring group (7.79 [1.68, 41.1] %, 6.72 [1.22, 41.9] %, respectively) (Fig. 1A). After the long‐term follow‐up period (19.8±6.6 months), all but one patient in the CA group had continued OACs, while 28 patients in the monitoring group were on OACs (p=0.314). No patients had systemic embolisms in either group. Bleeding events occurred in 3 patients in the CA group and one in the monitoring group (log‐rank p=0.144) (Fig. 1B).

**Conclusions:** CA of device‐detected AF may reduce the AF burden and suppress the AF progression in patients with CIEDs. It is challenging to decide whether to continue oral anticoagulation in patients with a reduced AF burden after CA.

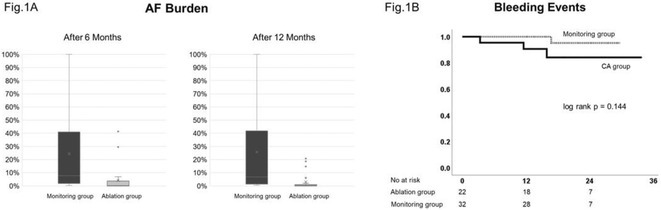

## CHALLENGES IN DIAGNOSTIC AND CATHETER ABLATION OF LONG RP SUPRAVENTRICULAR TACHYCARDIA WITH ECCENTRIC ACTIVATION AND DECREMENTAL PROPERTIES‐A CASE REPORT

### KEVIN KARIM

#### University of Padjadjaran, Hasan Sadikin General Hospital, Bandung, Indonesia

**Introduction:** In most cases, an electrophysiological study is necessary to discern the exact etiology of long RP SVT. Here, we present a challenging case of long RP SVT with eccentric activation and decremental properties.

**Methods:** N/A

**Results:** A 40 year old male patient presented with recurrent bouts of palpitations for five years prior to admission. Echocardiography was within normal limits. ECG during tachycardia showed long RP tachycardia. EP study showed eccentric atrial activation and decremental properties with earliest A at DD9‐10 (coronary sinus ostium dd/ left posteroseptal). SVT pattern: cycle length 410ms; VA interval 215ms; AH interval 93 ms; HA interval 332 ms; ratio AH/HA interval <1, V entrainment showed VAV response, PPI‐TCL 225 ms, SA‐VA 194 ms, V reset did not terminate SVT yet no atrial delay/advancement. Ablation was attempted at coronary sinus ostium during SVT which terminated, yet still inducible. We decided to perform slow pathway ablation and slow junctional rhythm was observed during ablation. Post ablation, HRA S1 showed crossover at 320 ms. SVT can still be easily induced with S1 pacing at 400 ms from the atrium. We opted to optimize medical treatment and scheduled a 3D ablation if tachycardia persisted.

**Conclusions:** We presented a complex case of long RP SVT ablation with main differentials including AVNRT fast‐slow pathway with bystander accessory pathway and PJRT with bystander dual AV nodal physiology. In these cases, left atrial access and 3D ablation based on earliest activation time might be required.

## DISAPPEARANCE OF THE LATE AND PIVOTING ACTIVATION AROUND KOCH’S TRIANGLE AFTER SUCCESSFUL ABLATION FOR TYPICAL ATRIOVENTRICULAR NODAL REENTRANT TACHYCARDIA

### **AKIRA KASAGAWA**, TOMOO HARADA, SANAMI OZAKI, IKURATO NAKAJIMA, KENICHI SASAKI, J YOSHIHIRO AKASHI

#### St Marianna Univirsity, Kawasaki, Japan

**Introduction:** Recently, high‐resolution mapping around Koch's triangle in sinus rhythm has been reported to be useful to identify ablation target sites in patients with atrioventricular nodal reentrant tachycardia (AVNRT). However, there are very few reports evaluating changes in activation pattern around those area before and after successful ablation for AVNRT.

**Methods:** Conventional catheter mapping and radiofrequency ablation (RFA) were performed in 30 consecutive patients referred for therapy of slow‐fast AVNRT. In five patients among them, latest high‐resolution activation mapping around Koch's triangle in sinus rhythm with OCTARAY catheter using CARTO mapping system (Biosense webster) was examined before and after ablation.

**Results:** In all five patients, the activation pattern pivoting around the block line (left panel in Figure) beneath the His potential (yellow tags in Figure) was observed. Late activation area were present beneath the His potential, local activation time (LAT) to the late activation area pivoting around the block line was 35±11.4 msec before ablation. The late activation electrogram characteristics (L in left panel in Figure) represented by potentials longer in duration and with fractionation. The electrogram characteristics at a pivot points (P) and outside the block line (E) were short in duration and high in amplitude. RFA was applied to the pivot point (red tags in right panel), and we confirmed the inability to induce AVNRT beyond a one‐echo beat. Activation mapping after successful ablation revealed the disappearance of a block line and pivot point (right panel in Figure), LAT to the tricuspid valve changed much shorter (4±1.5 msec, p<0.001) in all five patients.

**Conclusions:** A pivot point in Koch's triangle during sinus rhythm was a reasonable ablation site for typical AVNRT. The disappearance of the pivoting activation may be helpful for confirming successful ablation.

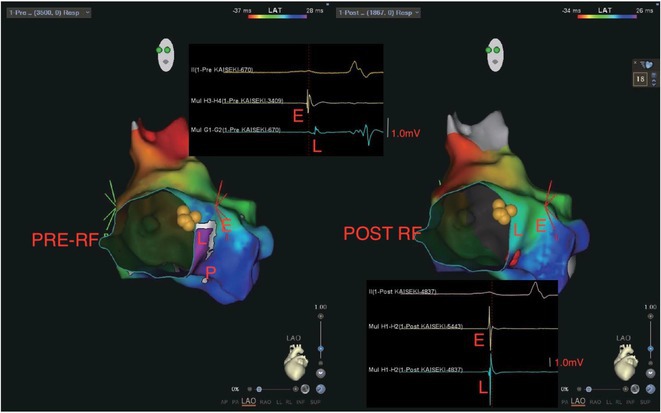

## COMPARISON OF CRYOBALLOON ABLATION AND RADIOFREQUENCY ABLATION WITH REGARD TO EFFICACY OF EXTRA PULMONARY VEIN ISOLATION IN PERSISTENT ATRIAL FIBRILLATION

### **SHUNSUKE KAWAI**, ARIHIDE OKAHARA, MASAKI TOKUTOME, HIROHIDE MATSUURA, JUNPEI ITONAGA, EICHI KOGA, HIROSHI KISANUKI, AYANO HARA, MASASHI SADA, KOSUKE OKABE, KIYOHIRO OGAWA, RYUICHI MATSUKAWA, YASUSHI MUKAI

#### Japanese Red Cross Fukuoka Hospital, Fukuoka, Japan

**Introduction:** Although some investigators reported the efficacy of left atrial roof ablation in addition to pulmonary vein isolation (PVI) using the cryoballoon (CB) for persistent atrial fibrillation (AF) cases, it remains unclear whether the clinical outcome of CB ablation is comparable to that of radiofrequency (RF) ablation.

**Methods:** We enrolled persistent AF cases who underwent index catheter ablation procedure. Mid‐term clinical outcome of RF group (N=265) was compared with that of CB group (N=44).

**Results:** Sixty‐six patients (RF group; N=33, CB group; N=33) were analyzed after propensity score adjustment (Fig. 1). In RF group, 15 cases underwent PVI alone. Prophylactic superior vena cava isolation and cavo‐tricuspid isthmus ablation were performed in 10 and 12 cases, respectively. One case underwent line ablation. In CB group, all cases underwent roof ablation in addition to PVI. Atrial tachyarrhythmia recurrence free rate at 12 months of follow‐up period in RF group and CB group were 87.4% and 81.3%, respectively. There was no significant difference between 2 groups (Fig. 2).

**Conclusions:** Efficacy of extra pulmonary vein isolation ablation is comparable between cryoballoon ablation and radiofrequency ablation in persistent atrial fibrillation cases.

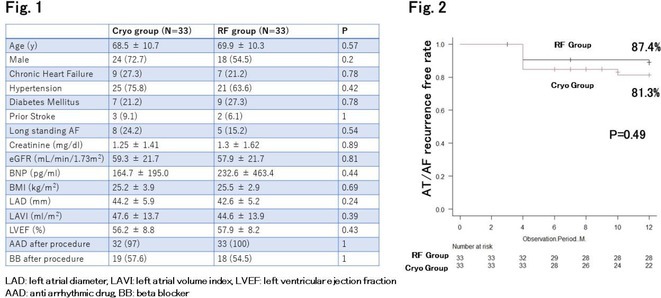

## RELATIONSHIP BETWEEN NON‐PULMONARY VEIN TRIGGER SITES AND LOW VOLTAGE AREA

### **SHUNSUKE KAWAI**, ARIHIDE OKAHARA, MASAKI TOKUTOME, HIROHIDE MATSUURA, JUNPEI ITONAGA, EICHI KOGA, HIROSHI KISANUKI, AYANO HARA, MASASHI SADA, KOSUKE OKABE, KIYOHIRO OGAWA, RYUICHI MATSUKAWA, YASUSHI MUKAI

#### Japanese Red Cross Fukuoka Hospital, Fukuoka, Japan

**Introduction:** We previously reported that non pulmonary vein (PV) triggers of atrial fibrillation (AF) arose from low voltage area (LVA) in the left atrium (LA). However, the relationship between non‐PV trigger sites and LVA is not fully clarified.

**Methods:** We enrolled 135 non‐PV trigger positive AF cases. Relationship between trigger sites and LVA was evaluated.

**Results:** LA voltage mapping was performed in 90 out of 135 cases. Relationship between trigger sites and LVA was evaluated in 96 LA non‐PV trigger sites. While almost all of the anterior/roof trigger sites arose from LVA, half of the posterior/septum/inferior trigger sites arose from intact area (Fig. 1). None of the 6 right atrial (RA) non‐PV trigger cases had LVA in the LA. We divided patients into 4 non‐PV trigger groups (thoracic vein, RA, septum and LA group) and compared mid‐term clinical outcome. Among the 4 groups, atrial tachyarrhythmia recurrence free rate at the 12 months of follow‐up period was lowest in LA group (P<0.05) (Fig. 2).

**Conclusions:** Non‐PV trigger sites are related to LVAs especially in the LA roof and anterior wall, and rather in the posterior/septum/inferior. The voltage mapping pattern may help to detect non‐PV trigger sites.

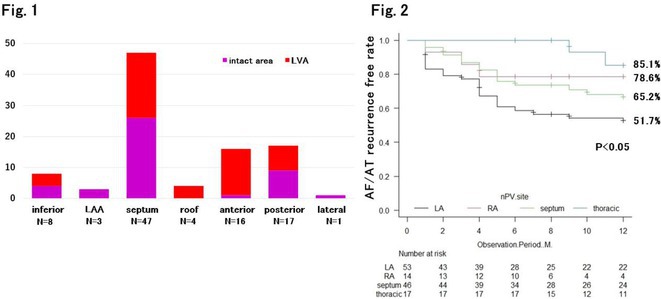

## HISTOLOGICAL BACKGROUND OF THE ABC‐DEATH SCORE IN PATIENTS WITH ATRIAL FIBRILLATION: EVIDENCE FROM ATRIAL BIOPSY

### **YUKI KAWANO** ^1^, TAKANORI YAMAGUCHI^2^, YUYA TAKAHASHI^2^, TOYOKAZU OTSUBO^2^, KANA NAKASHIMA^2^, KODAI SHINZATO^2^, YUKI NISHIMURA^2^, KOTARO TSURUTA^2^, RYOSUKE OSAKO^2^, MAKOTO EDAYOSHI^2^, SHIGEHISA AOKI^2^, ATSUSHI KAWAGUCHI^2^, TOSHIAKI KADOKAMI^1^, KOICHI NODE^2^

#### ^1^Saiseikai Futsukaichi Hospital, Chikushino, Japan,^2^Saga University, Saga, Japan

**Introduction:** The ABC‐death score consisting of the age, heart failure, and three biomarkers including NT‐proBNP, high‐sensitive troponin T (hs‐TnT), and growth differentiation factor (GDF)‐15 was developed to stratify mortality risk in anticoagulated patients with atrial fibrillation (AF). This study aimed to evaluate the histological background of the ABC‐death score.

**Methods:** A total of 245 consecutive patients (68 ± 12 years old; 78 women) undergoing AF catheter ablation and endomyocardial atrial biopsy were examined after excluding those with amyloid deposition (n = 15). The ABC‐death scores for all‐cause and cardiovascular mortality were calculated for each patient. Atrial samples were obtained from the right atrial septum and histologically analyzed to quantify the extent of fibrosis (%Fibrosis), intercellular space (%Intercellular space), myofibrillar loss severity (%Myofibrillar loss), and myocardial nuclear density. Logistic regression analysis was used to examine the relationship between the ABC‐death score and each histological factor.

**Results:** The ABC‐death scores for all‐cause mortality and cardiovascular mortality were 10.4 ± 4.0 and 8.5 ± 3.2, respectively. %Fibrosis, %Intercellular space, %Myofibrillar loss and nuclear density were 6.8 ± 3.6%, 21.8 ± 8.3%, 20.1 ± 10.2%, and 384 ± 141/mm^2^, respectively. Multivariate logistic regression analyses showed that myofibrillar loss was the only histological factor associated with ABC‐death scores for all‐cause mortality (p < 0.0001) and cardiovascular mortality (p < 0.0001) after covariate adjustment including age, sex, type of AF, and left atrial volume.

**Conclusions:** Degeneration of the myocardial parenchyma, not interstitial remodeling, is associated with the ABC‐death score.

## SUPPRESSION OF ATRIAL FIBRILLATION FOLLOWING ABLATION OF PREMATURE VENTRICULAR CONTRACTION AND ATRIOVENTRICULAR NODAL REENTRANT TACHYCARDIA: A CASE REPORT

### **KKL KHOO**, YH PHANG, FN AZIZAN, CL LEE, AS MD SAAD, C. CHHEAV, KS LIEW, HY KANG, CY TAN, KR NARASAMULOO, S. KRISHINAN

#### Hospital Sultanah Bahiyah, Alor Setar, Malaysia

**Introduction:** Atrioventricular nodal reentrant tachycardia (AVNRT) is known to coexist with other arrhythmias, yet this is underappreciated. Recognizing AVNRT as a primary trigger for other arrhythmias has important therapeutic consequences. We present a case that illustrates this point.

**Methods:** N/A

**Results:** This is a 52 year old lady who was diagnosed with recurrent atrial fibrillation (AF) and paroxysmal SVT. Her thyroid function and echocardiography were normal and she had a sinus rhythm as baseline. She was scheduled for electrophysiological studies (EPS). In the lab she was in sinus rhythm with frequent PVCs. The PVCs had a “right ventricular outflow tract (RVOT) morphology” suggested by an LBBB morphology in V6, transition at V4 and an inferior axis. Since the PVCs were frequent we decided for PVC ablation. The earliest spot of PVC was at the postero‐septal RVOT with sharp QS pattern at the unipolar EGM. During mapping, a PVC was seen to initiate a narrow complex tachycardia with a cycle length (CL) of 285ms and VA interval of 48ms. This tachycardia degenerated into AF and then atrial flutter (regular atrial CL of 204ms) with earliest atrial activation at the lower right atrium. Entrainment of this atrial flutter was unsuccessful as she went back to AF. She was then electrically cardioverted. We proceed to ablate the PVCs to prevent PVC‐induced SVT. Post PVC ablation, we re‐induced the previously seen narrow complex tachycardia via atrial extra‐stimulus pacing. The tachycardia was entrained and showed V‐A‐V response, PPI‐TCI of 239ms and SA‐VA of 139ms. Thus a diagnosis of typical slow‐fast AVNRT was made. We proceeded with slow pathway ablation. Post ablation, the AVNRT, AF and atrial flutter were no longer inducible.

**Conclusions:** In patients with co‐existing arrhythmias, it is important to consider if one arrhythmia might be the trigger for another. This case showed how PVCs can trigger AVNRT which then led to AF. Successful ablation of the PVC and slow pathway cured the AF. Thus it may be prudent to have EPS done before AF ablation to reveal unsuspected AVNRT that triggers AF in selected patients.

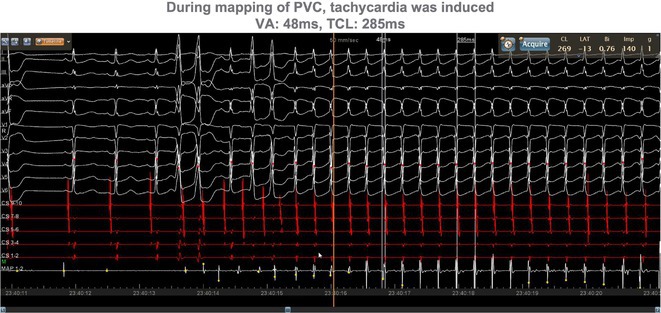

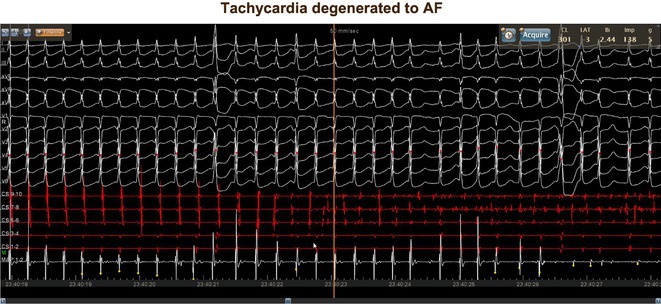

## EFFECT OF CATHETER ABLATION VS MEDICAL THERAPY ON CARDIOVASCULAR OUTCOMES AMONG ATRIAL FIBRILLATION PATIENTS WITH HYPERTROPHIC CARDIOMYOPATHY

### **DAEHOON KIM** ^1^, PIL‐SUNG YANG^2^, EUNSUN JANG^1^, HEE TAE YU^1^, TAE‐HOON KIM^1^, JAE‐SUN UHM^1^, JUNG‐HOON SUNG^2^, HUI‐NAM PAK^1^, MOON‐HYOUNG LEE^1^, BOYOUNG JOUNG^1^

#### ^1^Yonsei University College of Medicine, Seoul, Korea, Republic of,^2^CHA University, Seongnam, Korea, Republic of

**Introduction:** Atrial fibrillation (AF) increases the risks of stroke and mortality. AF ablation in patients with heart failure (HF) was associated with a lower risk of death and hospitalisation for worsening HF. Whether catheter ablation is beneficial to improve cardiovascular outcomes in patients with concomitant hypertrophic cardiomyopathy (HCM) and AF remains unclear. We aimed to investigate whether AF ablation is more effective than conventional medical therapy for improving outcomes in HCM.

**Methods:** 2,281 patients with HCM and AF undergoing catheter ablation or medical therapy (antiarrhythmic drugs or rate control drugs) in 2005‐2015 were identified from the Korean National Health Insurance Service database. The primary composite outcome of death from cardiovascular causes, ischemic stroke, hospitalization for worsening HF, or acute myocardial infarction was compared between catheter ablation and medical therapy using propensity score overlap weighting. The time‐at‐risk was counted from the first medical therapy, and catheter ablation was analyzed as a time‐varying exposure.

**Results:** Of the included (41.1% female; median age: 66 years), 145 (6.1%) underwent catheter ablation for AF during the study period. During a mean follow‐up of 3.1 (IQR 1.3‐5.8) years, a total of 831 composite outcomes occurred including 292 cardiovascular deaths and 401 strokes. Catheter ablation, compared with medical therapy, was associated with lower risks of the primary composite outcome (weighted incidence rate: 3.84 vs. 6.43 per 100 person‐years; weighted HR 0.61, 95% CI 0.38‐0.96) and ischemic stroke (weighted HR 0.43, 95% CI 0.19‐0.97). The association between ablation and a lower risk of composite outcome was more pronounced in cases of ablation success whereas no significant difference was observed in cases of ablation failure.

**Conclusions:** In patients with AF and HCM, catheter ablation was associated with a lower risk of adverse cardiovascular outcomes than medical therapy.

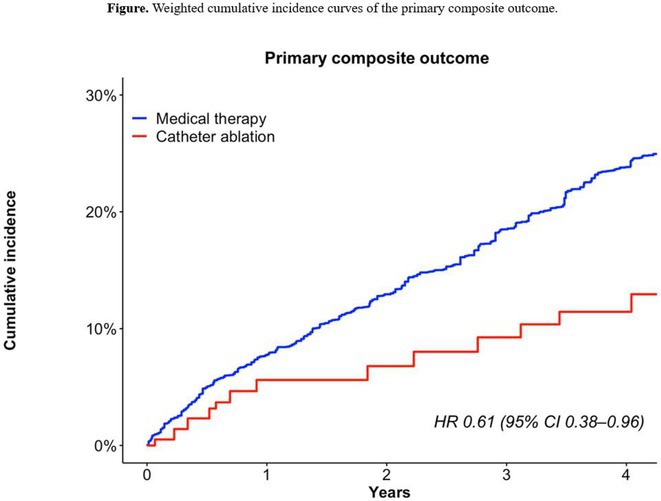

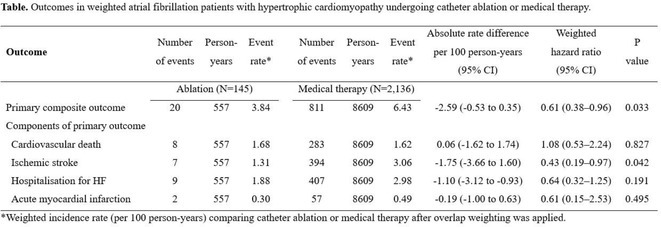

## EFFICACY AND SAFETY OF LEFT ATRIAL APPENDAGE CLOSURE IN PATIENTS WITH ATRIAL FIBRILLATION AND FAILED ANTICOAGULANT THERAPY: A NATION‐WIDE COHORT STUDY

### **DO YOUNG KIM**, EUI‐SOON KIM, EUN SOM JANG, HWA JUNG KIM, YEON‐HEE LEE, JUN‐PYO MYONG, SOOHYUN KIM, SOYOON PARK, YOUNG CHOI, YONG‐SEOG OH, SUNG‐HWAN KIM

#### The Catholic University of Korea, Seoul St. Mary's Hospital, Seoul, Korea, Republic of

**Introduction:** Stroke is one of the most lethal complications of atrial fibrillation (AF). While oral anticoagulant (OAC) effectively prevents ischemic stroke, 20‐36% of patients may still experience strokes during OAC therapy. Left atrial appendage closure (LAAC) could be an alternative for those with OAC failure, though its advantages over conventional OAC therapy are unclear.

**Methods:** Using data from the Health Insurance Review & Assessment Service in Korea, this retrospective cohort study analyzed patients with AF who developed stroke on OAC from January 2012 to December 2017. Patients who underwent LAAC were compared to a control group using 1:3 propensity score matching. The primary outcome was a composite of stroke or systemic thromboembolism.

**Results:** The study included 96 patients in the LAAC group and 286 in the control group, with mean follow‐up durations of 19.7±6.46 and 20.78±6.28 months, respectively. Numerically, the LAAC group tended to have fewer occurrences of the primary outcome compared to the control group, although the difference was not statistically significant (3.1% vs 5.9%, Hazard Ratio [HR] 0.537, 95% Confidence Interval [CI] 0.157‐1.832, p=0.321). Similar trends were observed for death from any cause (5.2% vs 7.0%, HR 0.786, 95% CI 0.295‐2.096, p=0.631) and no significant differences were noted in severe disability or hospitalization for bleeding between the groups.

**Conclusions:** LAAC may provide a potential benefit in preventing stroke among patients with OAC failure. Further prospective studies are warranted to confirm these findings.

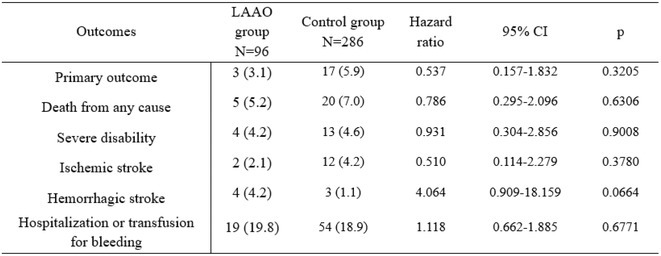

## NOCTURNAL ATRIOVENTRICULAR BLOCK: NATURAL HISTORY AND CLINICAL IMPLICATIONS

### SUNGHWAN KIM^1^, SOYOON PARK^1^, YOUNG CHOI^1^, YONG SEOK OH^1^, **DO YOUNG KIM** ^1^, HWAJUNG KIM^2^, SOOHYUN KIM^3^

#### ^1^Seoul St. Mary's hospital, Seoul, Korea, Republic of,^2^Yeouido St. Mary's hospital, Seoul, Korea, Republic of,^3^Incheon St. Mary's hospital, Incheon, Korea, Republic of

**Introduction:** Atrioventricular block (AVB) that occurs only at night has been considered the result of increased vagal tone and has a favorable outcome. Traditionally, nocturnal AVB is not an indication for pacemaker therapy; however, evidence is lacking.

**Methods:** Patients with AVB (2:1, high‐degree, or complete type) on 24‐hr electrocardiogram (ECG) monitoring only at night (21:00 PM to 07:00 AM) were enrolled in the present study. The primary outcome was implantation of a pacemaker during the follow‐up period.

**Results:** A total of 10,977 individuals underwent 24‐hour monitoring at the Department of Cardiology in Seoul St. Mary's Hospital between January 2000 and June 2020. Among them, 342 patients were diagnosed with complete atrioventricular block (AVB), high‐degree AVB, or 2:1 AVB, and 92 of these 342 patients were classified as having nocturnal AVB. Excluding 11 patients who were lost to follow‐up and 14 patients who had immediate pacemaker implantation due to daytime bradycardia symptoms, 67 patients underwent watchful waiting. Over a median follow‐up period of 2.4 years, 14 out of these 67 patients eventually received pacemaker implantation (n: CAVB 5/14, high‐degree AVB 4/14, 2:1 AVB 5/14). The indications for pacemaker insertion included the emergence of symptoms or sign (n=13) or sick sinus syndrome (n=1) during the follow‐up period. Higher grade of AVB, diabetes mellitus and absence of PR prolongation prior to occurrence of AVB were identified as risk factors for pacemaker insertion during follow‐up period. (HR 2.8 p‐value 0.038, HR 4.0, p‐value 0.002, and HR 3.1 p‐value 0.01, respectively)

**Conclusions:** Although patients did not have a pacemaker insertion at the time of diagnosis, a pacemaker insertion was needed in 21% of subjects within the median 2.4 years of follow‐up. Therefore, active surveillance should be required even for patients with nocturnal AVB.

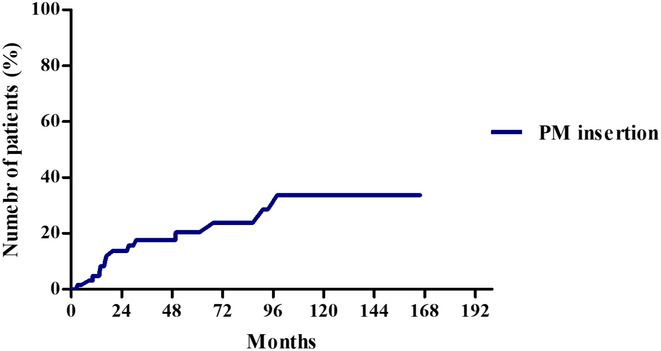

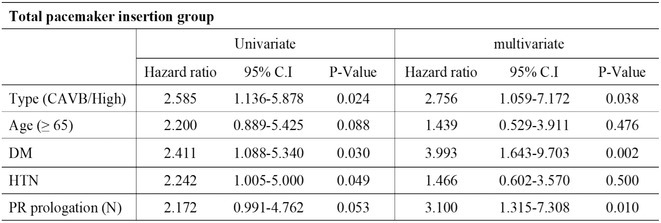

## ARTIFICIAL INTELLIGENCE‐BASED PREDICTING INCIDENT ATRIAL FIBRILLATION USING SINUS RHYTHM ELECTROCARDIOGRAM FOR POST‐MYOCARDIAL INFARCTION PATIENTS AS AN IMAGE DATA STRUCTURE

### IN‐SOO KIM

#### Yonsei University College of Medicine, Seoul, Korea, Republic of

**Introduction:** Atrial fibrillation (AF) is frequently under‐detected but linked to cardiovascular comorbidities, including stroke. Preexisting screening modalities are known to have low performance. We aimed to develop a convolutional neural network (CNN)‐based predictive tool for individual risk assessment of incident AF using sinus rhythm electrocardiogram (SRECG) data for post‐myocardial infarction (MI) patients.

**Methods:** We developed a predictive tool based on a CNN image detection algorithm, merging it with individual patient characteristics to predict incident AF using 12‐lead SRECG. We included 4,656 SRECG from 531 post‐MI patients without history of AF and allocated the SRECG to training, internal validation, and testing datasets in a 7:1:2 ratio. We classified patients with incident AF within 24 months after MI.

**Results:** Our study included 392 patients with 3,262 SRECG for training, 58 patients with 466 SRECG for internal validation, and 81 patients with 928 post‐cardioversion SRECG for the testing dataset. The mean age was 68.2 years, with mean left atrial diameter and left ventricular ejection fraction measuring 39.3mm and 62.2%, respectively. Furthermore, 77.8% had a history of hypertension, and 45.6% had diabetes. In the testing dataset, 14 (16.9%) patients were confirmed to have incident AF. The area under the curve (AUC) for predicting incident AF in post‐MI patients was 0.82 [0.75‐0.90], with a sensitivity of 0.63, specificity of 0.72, and an overall accuracy of 72.1% after training.

**Conclusions:** Our predictive tool, based on a CNN image detection algorithm merged with individual patient information, can identify post‐MI patients at risk of incident AF using SRECG.

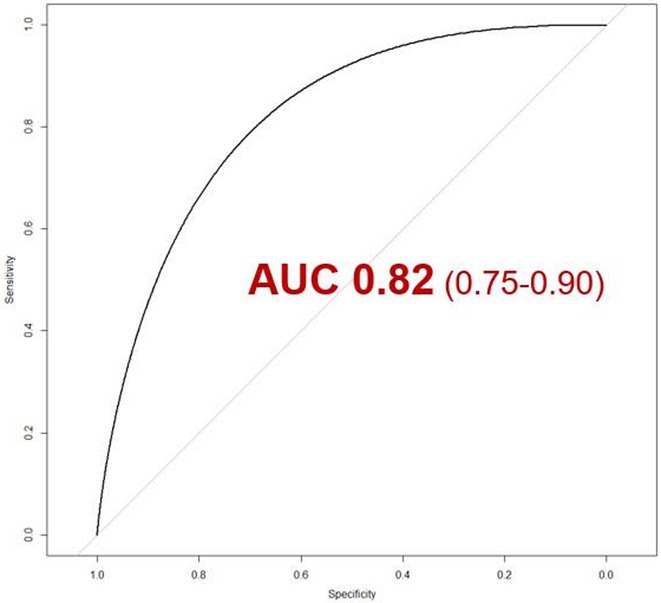

## ASSOCIATION BETWEEN FINE PARTICULATE MATTER AND VENTRICULAR HIGH‐RATE EPISODE (VHRE) IN PATIENTS WITH CARDIAC IMPLANTABLE ELECTRONIC DEVICES

### JUNG RAN CHOI, NA AHN, YOUNG SHIN LEE, **JIN‐BAE KIM**, WOO‐SHIK KIM

#### Kyung Hee University Hospital, Seoul, Korea, Republic of

**Introduction:** While atrial high rate episodes (AHREs) are crucial to recognize because AHRE is considered a harbinger of atrial fibrillation, ventricular high‐rate episodes (VHREs) may also occur and identify patients experiencing non‐sustained ventricular tachycardia (NSVT) or sustained ventricular tachycardia. Recent studies have suggested that exposure to particulate matter (PM_2.5_) increases the incidence of subclinical arrhythmia. However, evidence regarding the association between PM_2.5_ exposure and subclinical arrhythmia is limited, especially in patients with cardiac implantable electronic devices (CIED).

**Methods:** We conducted a nationwide cross‐sectional study using data from eight multicenter cohort studies. Using generalize estimating equation (GEE), we assessed the association between air pollution, such as PM_2.5_, PM_10_, O_3_, NO2, SO2 and CO, and subclinical arrhythmia episode, including AHREs and VHREs. These pollutants were estimated using high‐resolution and high‐quality spatiotemporal datasets of Air Korea's daily air pollutants.

**Results:** Among the 22,793 eligible episodes, we identified 98 patients with subclinical arrhythmia, of whom 631 experienced atrial arrhythmia episodes, and 201 had ventricular arrhythmia occurrences. Of the air pollutants, SO2 showed a significant positive association with the risk of overall arrhythmia, while no air pollutants showed a significant association with atrial arrhythmia risk. PM2.5, NO2, SO2, and CO were statistically significantly associated with an increased risk of ventricular arrhythmia (odds ratio (OR), 1.038; 95% confidence interval (CI), 1.009‐1.067; OR, 1.159; 95% CI 1.044‐1.296; OR 2.278; 95% CI 1.105‐4.696; and OR, 1.006; 95% CI, 1.002‐1.010, respectively), while O3 was significantly associated with a low risk (OR 0.914; 95% CI 0.841‐0.993).

**Conclusions:** These results suggest a statistically significant association between particulate matter and a higher risk of ventricular arrhythmia occurrence. Further retrospective studies using nationwide claims data and prospective studies are warranted.

## CRYOBALLOON ABLATION OF ATRIAL FIBRILLATION IN PATIENTS WITH HYPERTROPHIC CARDIOMYOPATHY

### **MYOUNG JUNG KIM** ^1^, CHANG HEE KWON^2^, IL‐YOUNG OH^3^, SO‐RYOUNG LEE^4^, SUNG HO LEE^5^, JUNBEOM PARK^6^, KI‐HUN KIM^7^, PIL‐SUNG YANG^8^, JUN‐HYUNG KIM^9^, JAEMIN SHIM^10^, MYUNG‐JIN CHA^11^, HONG EUY LIM^12^, JU YOUN KIM^1^

#### ^1^Samsung Medical Center, Seoul, Korea, Republic of,^2^Konkuk University Medical Center, Seoul, Korea, Republic of,^3^Seoul National University Bundang Hospital, Seongnam, Korea, Republic of,^4^Seoul National University Hospital, Seoul, Korea, Republic of,^5^Kangbuk Samsung Hospital, Seoul, Korea, Republic of,^6^Ewha Womans University Medical Center, Seoul, Korea, Republic of,^7^Haeundae Paik Hospital, Busan, Korea, Republic of,^8^CHA Bundang Medical Center, Seongnam, Korea, Republic of,^9^Chungnam National University Hospital, Daejeon, Korea, Republic of,^10^Korea University Anam Hospital, Seoul, Korea, Republic of,^11^Asan Medical Center, Seoul, Korea, Republic of,^12^Hallym University Sacred Heart Hospital, Anyang, Korea, Republic of

**Introduction:** Hypertrophic cardiomyopathy (HCM) poses a distinct challenge due to its association with atrial fibrillation (AF), significantly increasing the risk of stroke. Despite optimal antiarrhythmic medications, their efficacy in HCM patients is limited, necessitating alternative interventions. Cryoballoon ablation (CBA) has emerged as a promising treatment for AF, but its efficacy in HCM patients remains uncertain. This study aims to investigate the effectiveness of CBA in HCM patients with AF.

**Methods:** The study utilized data from the Korean CBA registry, comprising observational cohort data from 12 tertiary institutes between 2018 and 2022. Patients undergoing CBA for drug‐refractory symptomatic AF were included. HCM was defined as a left ventricular wall thickness ≥15 mm, and AF was categorized as paroxysmal or persistent. Procedures were performed according to established guidelines. The primary outcomes were recurrence of atrial fibrillation, atrial flutter, and/or atrial tachycardia lasting longer than 30 seconds at 3 months post‐procedure.

**Results:** A total of 2649 patients were included, delineating baseline characteristics such as mean age (61.7 years), male predominance (76.7%), and the prevalence of paroxysmal AF (44.4%). Patients with HCM (n=59) exhibited larger left atrial dimensions and longer AF duration compared to non‐HCM patients. AF recurrence rates were significantly higher in HCM patients at 1 and 2 years post‐procedure. Multivariate analysis identified left atrial size and persistent AF as significant predictors of AF recurrence.

**Conclusions:** CBA demonstrated safety and feasibility in HCM patients with AF; however, post‐procedural AF recurrence rates were elevated, primarily attributed to patient‐specific characteristics such as larger left atrial size and persistent AF. These findings underscore the need for tailored interventions to optimize outcomes in this patient population.

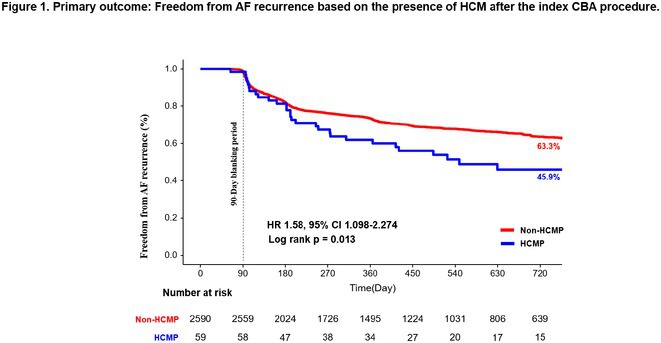

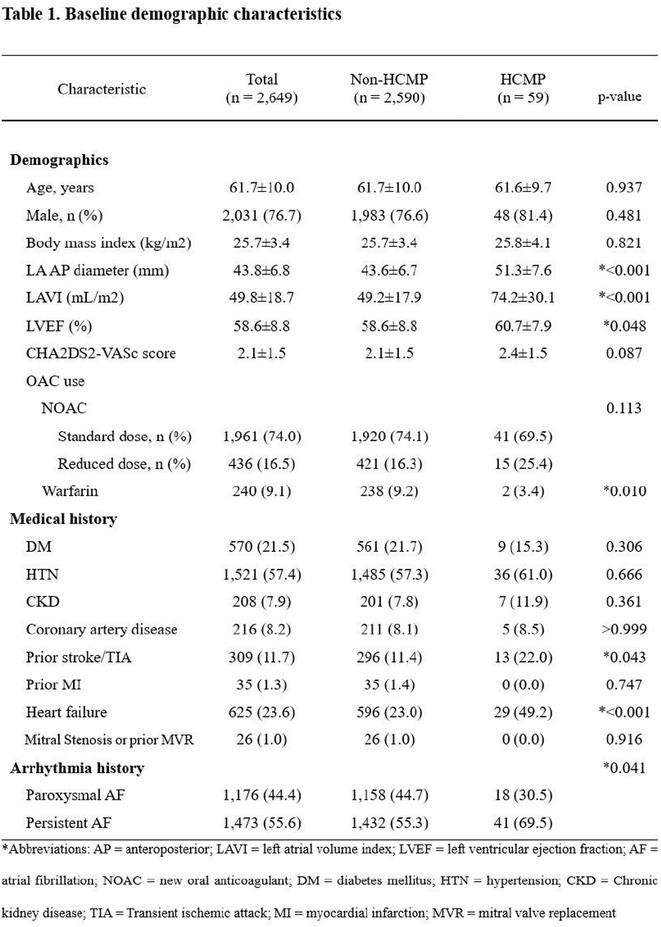

## UTILITY OF COMPUTED TOMOGRAPHY CORONARY ANGIOGRAPHY IN PATIENTS REQUIRING PERMANENT PACING

### **SAMUEL KIM**, ADAM PERKOVIC, LINDA LIN

#### Northern Beaches Hospital, Frenchs Forest, Australia

**Introduction:** Coronary artery disease (CAD) is a common cause of bradycardia. Despite the widespread use of computed tomography coronary angiography (CTCA) to non‐invasively assess for CAD, there is limited data assessing its utility in patients with bradycardia requiring permanent pacing.

**Methods:** We retrospectively studied patients requiring permanent pacing referred for CTCA to investigate for CAD as a potential etiology between January 2019 and February 2024. CTCA characteristics including coronary artery calcium (CAC) score, coronary dominance, and significant (≥50% stenosis) main vessel disease (VD) were recorded. Baseline demographics including age, gender, history of CAD, indication for pacing and subsequent coronary angiography and/or revascularization were also documented.

**Results:** 50 patients were referred for CTCA for bradycardia, of which 29 required permanent pacing. The mean age was 73.0 years with 21 (72.4%) male gender. 2 (6.9%) patients had known CAD with coronary artery bypass grafting (CABG). The indication for permanent pacing was sinus node dysfunction in 7 (24.1%) and 2^nd^‐3^rd^ degree AV block in 22 (75.9%). CAC was 0 in 6 (20.7%), 1‐99 in 7 (24.1%), 100‐399 in 5 (17.2%) and ≥400 in 11 (37.9%). The coronary circulation was right dominant in 26 (89.7%) and left in 3 (10.3%). 15 (51.7%) had 0VD, 7 (24.1%) had 1VD, 5 (17.2%) had 2VD and 1 (3.4%) had 3VD. There was 1 (3.4%) patient with significant left main disease, 12 (41.4%) patients with left anterior descending artery disease, 4 (13.8%) with left circumflex disease, and 4 (13.8%) with right coronary artery disease. Both patients with prior CABG had patent grafts. 11 (37.9%) patients underwent coronary angiography with 5 (17.2%) receiving revascularization; 3 with percutaneous coronary intervention and 2 with CABG.

**Conclusions:** CTCA in patients requiring permanent pacing identified a significant proportion of patients with angiographically significant CAD leading to revascularization. Larger studies with longitudinal follow‐up are required to determine whether this practice improves long‐term outcomes in this population.

## IMPACT OF COVID‐19 ON THE INCIDENCE, PREVALENCE AND ADVERSE OUTCOMES OF NON‐VALVULAR ATRIAL FIBRILLATION: NATIONWIDE HEALTH INSURANCE DATA COVERING THE ENTIRE KOREAN POPULATION

### **SEONJI KIM** ^1^, DAEHOON KIM^2^, SEUNG IL KIM^1^, SENG CHAN YOU^1^, PIL‐SUNG YANG^3^, BOYOUNG JOUNG^2^

#### ^1^Department of Biomedical Systems Informatics, Yonsei University College of Medicine, Seoul, Korea, Republic of,^2^Division of Cardiology, Severance Cardiovascular Hospital, Yonsei University College of Medicine, Seoul, Korea, Republic of,^3^Department of Cardiology, CHA Bundang Medical Center, CHA University, Seongnam, Korea, Republic of

**Introduction:** The incidence and prevalence of atrial fibrillation (AF) are rising worldwide. This is recognized as a global health issue due to the significant burden on morbidity and mortality rates caused by cardiovascular diseases. Although most diseases have been affected by COVID‐19, few previous studies have identified trends in AF considering the post‐pandemic period. We aimed to investigate the trends of the incidence, prevalence, and 1‐year mortality and cardiovascular event rates of non‐valvular AF in Korea.

**Methods:** We performed a cross‐sectional study using the National Health Insurance Service database involving the entire Korean population from 2010 to 2022. The incidence cases were defined with no diagnosis of AF for 4 years. Age‐ and sex‐adjusted AF incidence rate and prevalence rates were estimated by calendar year and 1‐year mortality and cardiovascular event rates following AF were assessed.

**Results:** A total of 1,358,240 patients with non‐valvular AF were identified from 2010 to 2022. In 2014, the standardized AF incidence rate was 1.95 (95% CI, 1.94‐1.96) per 1000 person‐years, which increased gradually to 2.26 (95% CI: 2.25 ‐ 2.27) in 2019 but decreased to 2.09 (95% CI: 2.08 ‐ 2.11) immediately after the COVID‐19 pandemic (Figure 1A). The AF prevalence increased from 0.71% in 2014 to 1.27% in 2022, regardless of COVID‐19. In AF patients, the annual rates were 8.41% for all‐cause mortality, 2.99% for ischemic stroke, 0.42% for myocardial infarction, 0.66% for intracranial bleeding, and 2.38% for heart failure admission. All‐cause mortality increased after the COVID‐19 pandemic from 8.06 in 2021 to 9.27 in 2022 (Figure 1B).

**Conclusions:** In this nationwide cross‐sectional study, the annual incidence rate of AF has gradually increased before the COVID‐19 pandemic, but decreased after the COVID‐19 pandemic, suggesting decreased AF screening. In contrast, all‐cause mortality continuously decreased before COVID‐19, but increased after the COVID‐19 pandemic.

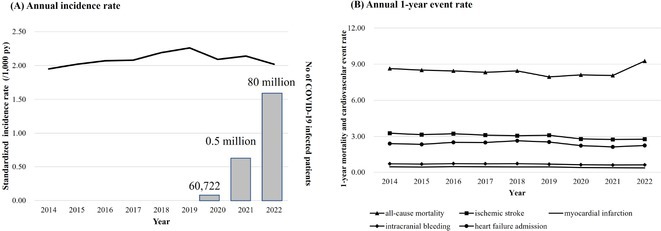

## A RANDOMIZED COMPARISON OF RIGHT SEPTAL PACING VERSUS APICAL PACING FOR PROGRESSION OF HEART FAILURE

### **SOOHYUN KIM** ^1^, SOYOON PARK^2^, HWAJUNG KIM^3^, DO YOUNG KIM^2^, YOUNG CHOI^2^, YONG‐SEOG OH^2^, SUNG‐HWAN KIM^2^

#### ^1^Incheon St. Mary's hospital, The Catholic University of Korea, Incheon, Korea, Republic of,^2^Seoul St. Mary's hospital, The Catholic University of Korea, Seoul, Korea, Republic of,^3^Yeouido St. Mary's hospital, The Catholic University of Korea, Seoul, Korea, Republic of

**Introduction:** Atrioventricular (AV) block is one of the most common indications for permanent pacemaker therapy. Although right ventricular (RV) apex had been used for over four decades without substantial damage or benefits, RV septal pacing has become an alternative position since probable association with more physiologic pacing.

**Methods:** In this prospective, randomized, single‐center study, patients who were indicated for permanent pacing due to AV block were randomly divided into apical pacing or septal pacing. The location of RV lead was assessed by electrocardiogram during implantation and confirmed by final fluoroscopy. The primary outcome is progression of heart failure, defined by reduction of left ventricular ejection fraction (LVEF) more than 5% from baseline or LVEF less than 45%.

**Results:** Total 176 patients were randomly divided into apical group and septal group by 1:1 ratio. After final fluoroscopy, RV lead was placed in apex in 95 patients and placed at septum in 81 patients. The mean age of patients was 74.0[65.0;80.0] and baseline LVEF were 63.0% [IQR 60.3;65.0] in apical group and 63.4% [IQR 61.0;66.8] in septal group (p=0.276). During median follow‐up of 3.4 years [IQR 1.8‐5.1], reduction of LVEF more than 5% from baseline was similar in both groups (41[46.6%] in apical group vs 33[37.5%] in septal group, log‐rank p=0.11). Reduction of LVEF more than 10% from baseline was numerically more frequent in apical group but there was no statistical difference between two groups (22[23.2%] in apical group vs 12[14.8%]) in septal group), log‐rank p=0.06). Progression of heart failure defined as LVEF <45% in whom baseline EF>50% was higher in apical group (11[11.6%] in apical group vs 4[5.4%] in septal group, log‐rank p=0.047), but the difference was not seen by intention‐to‐treatment analysis.

**Conclusions:** RV septal pacing is associated with better outcomes compared to RV apical pacing.

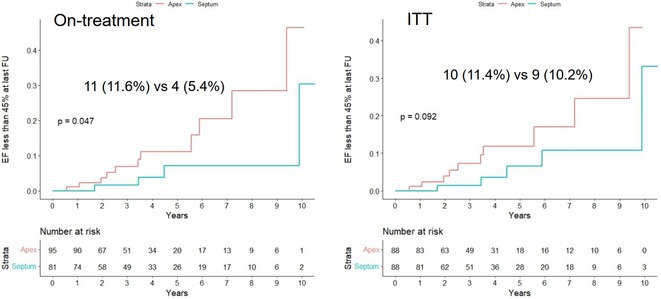

## CLINICAL OUTCOMES WITH THE USE OF ASPIRIN VERSUS CLOPIDOGREL AS A COMBINATION THERAPY WITH DIRECT ORAL ANTICOAGULANT AFTER CORONARY STENT IMPLANTATION IN PATIENTS WITH ATRIAL FIBRILLATION

### **SOOHYUN KIM** ^1^, SOYOON PARK^2^, HWAJUNG KIM^3^, SUNG‐HWAN KIM^2^, YONG‐SEOG OH^2^, YOUNG CHOI^2^, DO YOUNG KIM^2^

#### ^1^Incheon St. Mary's hospital, The Catholic University of Korea, Incheon, Korea, Republic of,^2^Seoul St. Mary's hospital, The Catholic University of Korea, Seoul, Korea, Republic of,^3^Yeouido St. Mary's hospital, The Catholic University of Korea, Seoul, Korea, Republic of

**Introduction:** Patients with atrial fibrillation (AF) who undergo percutaneous coronary intervention (PCI) are recommended to receive dual antithrombotic therapy including an antiplatelet agent and direct anticoagulants (DOAC). We compared the effectiveness between aspirin and clopidogrel for a combination therapy with DOAC.

**Methods:** We analyzed patient data from the Korea National Health Insurance Service. A total of 9,157 patients with AF who received dual antithrombotic therapy after PCI were included. Patients were classified into the clopidogrel group and aspirin group, and 1:1 propensity score matching (PSM) was performed. The major adverse cardiovascular event (MACE) was defined as a composite of cardiovascular death, myocardial infarction, stroke, or systemic thromboembolism. A major bleeding event was defined as a critical anatomical site bleeding requiring hospitalization.

**Results:** After PSM, there were each 2,882 patients each in the clopidogrel and aspirin group. During a mean follow‐up of 20.1 (±14.8) months, incidence of MACE was similar in the two groups (hazard ratio [HR] for clopidogrel group: 0.91, 95% confidence interval [CI] 0.81 ‐ 1.02). The incidence of each component of ischemic endpoint did not differ significantly between the two groups. There was no significant difference in the incidence of major bleeding events (HR 0.94, 95% CI 0.78 ‐ 1.12) and net adverse clinical events (HR 0.93, 95% CI 0.84 ‐ 1.03).

**Conclusions:** For a dual antithrombotic therapy in patients with AF who had undergone PCI, aspirin and clopidogrel did not differ in terms of long‐term ischemic and bleeding outcomes.

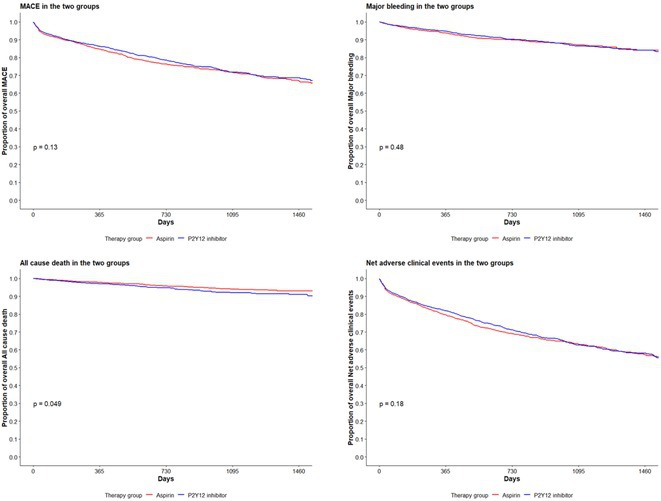

## COLCHICINE IN ADDITION TO USUAL CARE FOR HEMOPERICARDIUM AFTER CATHETER ABLATION FOR ATRIAL FIBRILLATION

### **SOOHYUN KIM** ^1^, SOYOON PARK^2^, HWAJUNG KIM^3^, DO YOUNG K^2^, YOUNG CHOI^2^, YONG‐SEOG OH^2^, SUNG‐HWAN KIM^2^

#### ^1^Incheon St. Mary's hospital, The Catholic University of Korea, Incheon, Korea, Republic of,^2^Seoul St. Mary's hospital, The Catholic University of Korea, Seoul, Korea, Republic of,^3^Yeouido St. Mary's hospital, The Catholic University of Korea, Seoul, Korea, Republic of

**Introduction:** Hemopericardium is a complication associated with radiofrequency catheter ablation (RFCA) for atrial fibrillation (AF) and may accompany pericardial inflammation. Colchicine has been shown to have anti‐inflammatory effects on acute pericarditis; however, the effect on hemopericardium that developed during RFCA is unknown.

**Methods:** Patients who developed hemopericardium during RFCA for AF were prospectively included in the present study. Percutaneous pericardiocentesis was performed in all patients. After the RFCA procedure, some patients were prescribed 0.6 mg colchicine twice daily for 14 days at the operator's discretion in addition to usual care. The patients were 1:2 propensity‐score (PS)‐matched. The outcomes were freedom from AF or atrial tachycardia (AT) within 12 months, signs of pericarditis, recurrence of pericardial effusion, and incidence of constrictive pericarditis.

**Results:** Hemopericardium requiring pericardiocentesis developed in 79 patients. Patients who received colchicine (Colchicine group, n = 21) were analyzed based on 1:2 PS‐matching with patients who received only usual care (Usual care group, n = 42). During the median 366.0 days (interquartile range (IQR) 329.5‐413.0 days) of follow‐up, freedom from AF or AT did not differ between the colchicine and usual care groups (71.4% vs. 76.2%, p = 0.83 by log‐rank test). Difference was not observed in remnant pericardial effusion at 1 month in patients in either group (p = 0.9), though all were regressed on follow‐up echocardiography. Constrictive pericarditis was not reported during follow‐up.

**Conclusions:** Colchicine in addition to usual care did not improve clinical outcomes in patients who developed hemopericardium after RFCA for AF.

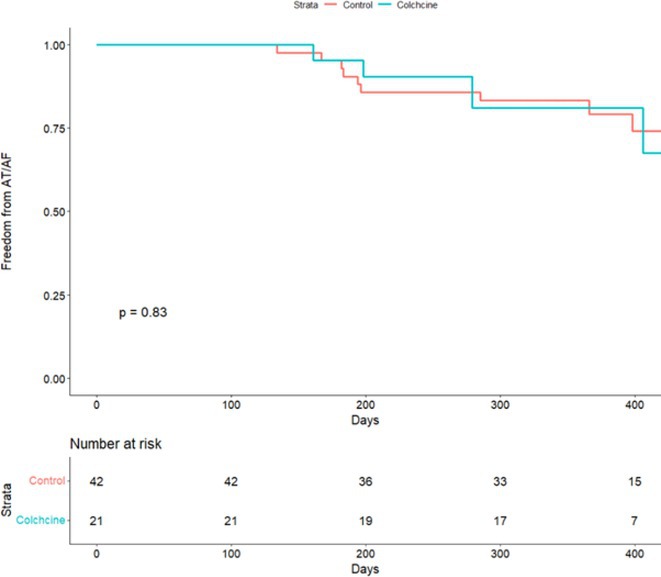

## MAINTENANCE OF SINUS RHYTHM IS ASSOCIATED WITH LOWER INCIDENCE OF STROKE IN PATIENTS WITH DRUG‐REFRACTORY ATRIAL FIBRILLATION

### **SOOHYUN KIM** ^1^, SOYOON PARK^2^, HWAJUNG KIM^3^, DO YOUNG KIM^2^, YOUNG CHOI^2^, YONG‐SEOG OH^2^, SUNG‐HWAN KIM^4^

#### ^1^Incheon St. Mary's hospital, The Catholic University of Korea, Incheon, Korea, Republic of,^2^Seoul St. Mary's hospital, The Catholic University of Korea, Seoul, Korea, Republic of,^3^Yeouido St. Mary's hospital, The Catholic University of Korea, Seoul, Korea, Republic of,^4^Korea St. Mary's hospital, The Catholic University of Korea, Korea, Korea, Republic of

**Introduction:** Recent studies have demonstrated that early rhythm control for first‐line treatment of atrial fibrillation (AF) improved cardiovascular outcomes. However, there is limited data regarding the long‐term outcome of patients who failed antiarrhythmic drugs and who refuse radiofrequency catheter ablation (RFCA).

**Methods:** Patients with AF who were refractory to antiarrhythmic drugs and had refused further rhythm control attempts via RFCA were retrospectively identified and propensity‐score matched with who had been treated with RFCA. The primary outcome of interest was all‐cause mortality or ischemic stroke.

**Results:** A total of 169 patients who refused rhythm control with RFCA and propensity‐score matched 169 patients who had been treated with RFCA were included for analysis. During a mean follow‐up of 4.3[2.3;6.9] years, maintenance of sinus rhythm was more achieved in RFCA group (7 [4.1%] in Refuse group vs. 133 [78.7%] in RFCA group, p<0.001). the incidence of ischemic stroke was significantly higher in patients who refused RFCA compared with patients who underwent RFCA (24 [14.2%] vs. 6 [3.5%], log‐rank p<0.001), but all‐cause mortality was not significantly different (log‐rank p=0.8). Refusal of attempted rhythm control via RFCA was an independent risk factor for ischemic stroke on multivariate Cox analysis (hazard ratio 3.2; 95% confidence interval 1.2 to 8.53, p=0.02). The risk of major adverse cardiovascular events (MACE) were also significantly higher in Refuse group (31 [18.3%] vs. 12 [7.1%], HR 3.83, 95% CI 1.57‐9.37, p=0.003).

**Conclusions:** In patients with antiarrhythmic drug‐refractory AF, maintenance of sinus rhythm was significantly different dependent on rhythm control via RFCA, therefore the risk of stroke was significantly higher in patients who refused rhythm control via RFCA compared with that of those treated with RFCA.

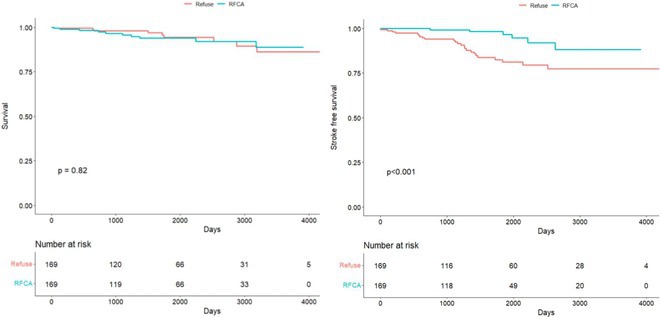

## FACTORS ASSOCIATED WITH THE DETECTION OF ATRIAL FIBRILLATION IN PATIENTS WITH EMBOLIC STROKE OF UNDETERMINED SOURCE

### **YEON‐JUNG KIM**, JAE‐HAN BAE, BUM JOON KIM

#### Asan Medical Center, Seoul, Korea, Republic of

**Introduction:** Detection of atrial fibrillation (AF) in patients with embolic stroke of undetermined source (ESUS) is important for the secondary prevention of stroke. We investigated the factors associated with the detection of newly diagnosed AF in ESUS patients during follow‐up.

**Methods:** Patients with acute ischemic stroke classified as ESUS were included. All patients underwent transthoracic echocardiography and Holter to detect the source of embolism. Structural, electrophysiological markers of left atrial cardiopathy (i.e., left atrial enlargement (LAE), non‐sustained tachycardia (NSAT)) as well as lesion patterns of ischemic stroke were examined. Implantable loop recorder (ILR) was implanted in selective patients. Sensitivity and positive predictive value analysis was used to assess the predictive value for AF detection.

**Results:** Among 312 patients with ESUS, AF was detected in 24 (7.7%) patients during follow‐up. Patients with AF had a higher prevalence of LAE, NSAT, and the imaging pattern of confluent plus additional lesions in a single vascular territory. Multivariable analysis showed that ILR implantation (hazards ratio 11.497 [95% confidence interval 3.795‐34.818]), LAE (3.204 (1.096 ‐ 9.370)), NSAT (4.070 (1.378 ‐ 12.018)), and confluent plus additional lesions (4.977 (1.649 ‐ 15.019)) were independent predictors of AF detection. The sensitivity of detecting AF in those with LAE, NSAT, or confluent plus additional lesions pattern was 91.7%. The positive predictive value of detecting AF in those with LAE, NSAT and confluent plus additional lesions pattern was 40.0%.

**Conclusions:** In conclusion, patients with LAE, NSAT, or confluent plus additional lesions may benefit from ILR monitoring detecting new AF.

## COMPARISON OF LEAD‐TYPE VS. PATCH‐TYPE WEARABLE ECG IN REAL‐WORD PRACTICE

### **YOO RI KIM** ^1^, DONG‐GEUM SHIN^2^, KI‐HONG LEE^1^, NAMSIK YOON^1^, HYUNG WOOK PARK^1^

#### ^1^Chonnam National University, Gwangju, Korea, Republic of,^2^Hanllym University, Seoul, Korea, Republic of

**Introduction:** Curren tly, there was two prevalent types of wearable ECG monitoring: a lead type and a patch type. Each method offers distinct advantages and considerations in terms of application, patient comfort, and diagnostic accuracy. This study aims to compare these two modalities, examining their respective strengths and limitations in real‐world practice

**Methods:** A total of 639 consecutive patients (mean age: 61.7±14.5, male: 56.7%) were included in the analysis between March 2022 and October 2023, retrospectively. The patient cohort was stratified into two groups: the lead type and the patch type wearable ECGs.

**Results:** Out of the total 639 patients, 466 (72.9%) were categorized under the patch‐type group, while 173 (27.1%) were assigned to the lead‐type group. Baseline characteristics, age, gender, and co‐morbidities (hypertension, diabetes, stroke, congestive heart failure, and coronary artery diseases), did not exhibit significant differences between the two groups. The detection rates of atrial fibrillation were comparable, with 24.0% in the patch‐type group and 24.9% in the lead‐type group (p=0.836). Notably, the patch‐type group demonstrated significantly longer monitoring durations compared to the lead‐type group (155.1±23.5 hours vs. 72.0±11.3 hours, p<0.0001) and a higher incidence of detecting pause events (612.3±140.8 vs. 151.3±75.8 per monitoring, p=0.004). Consequently, the patch‐type wearable ECG exhibited a higher noise rate than its lead‐type counterpart (12.9±14.9% vs. 6.9±12.4% per monitoring, p<0.0001).

**Conclusions:** In comparing lead‐type and patch‐type wearable ECG monitoring, we found both modalities to be clinically viable. Despite similar atrial fibrillation detection rates, the patch‐type demonstrated prolonged monitoring and increased detection of pause events. However, this advantage was counterbalanced by a higher noise rate. The choice between these methods should be tailored to patient needs, considering monitoring duration, diagnostic accuracy, and noise tolerance. This study contributes practical insights for optimizing wearable ECG technologies in real‐world clinical settings.

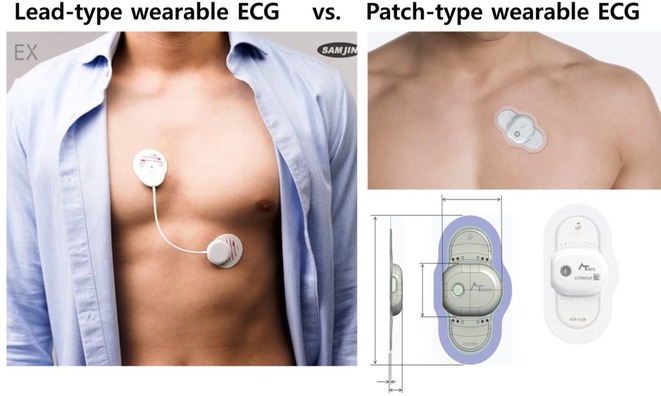

## MEASUREMENT QT INTERVAL IN ECGS WITH DEEP LEARNING TECHNIQUES

### **YOO RI KIM**, SEUNGJUN LEE, JINJU PARK, HYUNG‐JEONG YANG

#### Chonnam National University, Gwangju, Korea, Republic of

**Introduction:** In electrocardiograms (ECGs), the QT interval reflects the repolarization status of the heart. Currently, the automatical measurement of QT interval in 12‐lead ECGs is inaccurately derived as the median value, rather than utilizing the more precise mean value of QT intervals from all leads. This approach fails to provide information on heterogeneous QT prolongation crucial for predicting arrhythmias, as it does not accurately represent the values of QT intervals in each lead.

**Methods:** The dataset used in this study was the QT Database, comprising ECG data for 105 patients recorded at a sampling rate of 250Hz over a duration of 15 minutes. Each ECG was associated with annotations for two leads and information regarding the P, QRS, and T waves. We structured the dataset into a ratio of approximately 8:1:1, resulting in 51 patients for training (15,490 samples), 6 patients for validation (1,564 samples), and 7 patients for testing (1,682 samples).A model that integrates low‐pass filtering and continuous wavelet transformation eliminated baseline wander noise during ECG measurements.

For temporal features of ECG, we incorporate Convolutional Neural Networks (CNN) and Bidirectional Long Short‐Term Memory (Bi‐LSTM) to estimate QT intervals.

**Results:** For the evaluation of deep learning, we selected Accuracy and F1‐Score as metrics. We experimented with two configurations: the proposed model with two Bi‐LSTM layers and an alternative with a single Bi‐LSTM layer of 100 units. We also tested a similar recurrent neural network, GRU, with the same unit configuration. The results indicated that the method with two Bi‐LSTM layers outperformed others in both Accuracy and F1‐Score.

**Conclusions:** By incorporating CNN and Bi‐LSTM, we conducted QT interval estimation, leveraging temporal features. This methodology facilitated the elimination of diverse noise types during ECG measurements, resulting in enhanced accuracy for QT interval estimation through the consideration of temporal ECG characteristics. In our future research endeavors, we plan to deploy this model for predicting arrhythmias, specifically targeting conditions like long QT syndrome and drug‐induced QT prolongation.

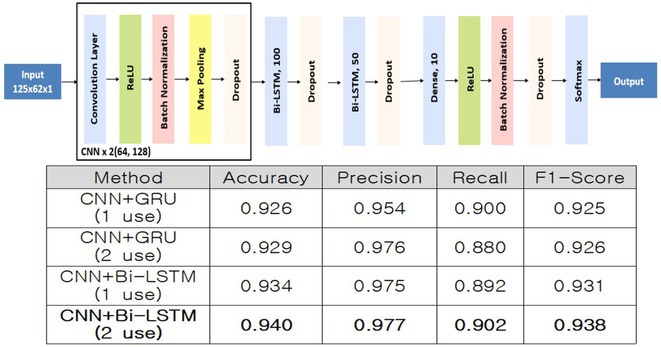

## HEART RATE SCORES IN PATIENTS WITH CARDIAC IMPLANTABLE ELECTRICAL DEVICES ARE NOT ASSOCIATED WITH PHYSICAL ACTIVITY AND REMAIN HIGH DESPITE THE USE OF THE HEART RATE RESPONSE FUNCTION

### **ATSUSHI KIMURA**, ASUKA MINAMI‐TAKANO, HARUNA TABUCHI, KAORI IWAMA‐SAIGUSA, KAZUNORI MARUYAMA, MITSUHIRO KUNIMOTO, GAKU SEKITA, MASATAKA SUMIYOSHI, KIKUO ISODA, TORU MINAMINO

#### Juntendo University, Tokyo, Japan

**Introduction:** Heart rate score (HRSc), a novel parameter, predicts the risk of incident heart failure, atrial fibrillation (AF), and mortality in patients with cardiac implantable electrical devices (CIEDs). Rate response (RR) is based on physical activity and is associated with HRSc. However, the association between HRSc and physical activity (PA) in patients with CIEDs is unclear.To clarify the physical activity of pacemaker patients and investigate its association with HRSc.

**Methods:** Enrolled consecutive patients with CIEDs under pacemaker management at Juntendo University Nerima Hospital (n=82). The observation period was from May 2023 to January 2024, and data on HRSc and PA time were retrospectively analyzed for the four months when data were available from remote monitoring.

**Results:** The mean age of the patients was 80.9 ± 9.9 years, and 51% were male. The average time of PA was 1.56 ± 0.99 hours, and there was no significant difference between males and females (p=0.62). The distribution of PA time was as follows: less than 1 hour (33.0%), 1‐2 hours (38.0%), 2‐3 hours (19.0%), and more than 3 hours (10.0%). Furthermore, the Spearman's correlation coefficient between PA time and age was ‐0.444 (p<0.001; Figure 1), indicating a moderate negative correlation between these two variables. There was no significant association between HRSc and PA time (r=0.176, p=0.112; Figure 2). RR use was observed in 43.9% (n=36) of patients, with a mean HRSc of 72.7%. RR use was 43.9% (n=36), and there was no association between rate response users and age (p=0.79). HRSc was higher in the group using RR than in the group not using RR (p<0.001).

**Conclusions:** There was a significant association between PA time and age, while HRSc did not correlate with patient PA time. These results may indicate that RR is underutilized in patients with CIEDs.

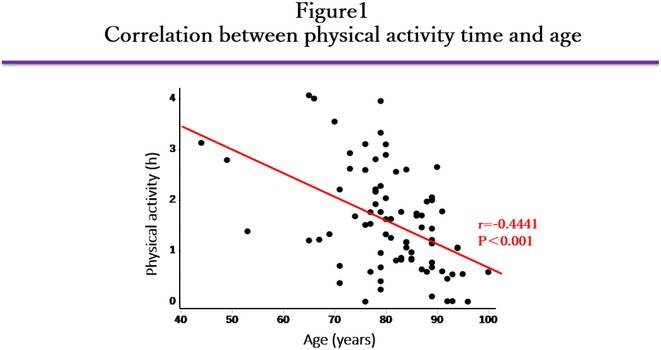

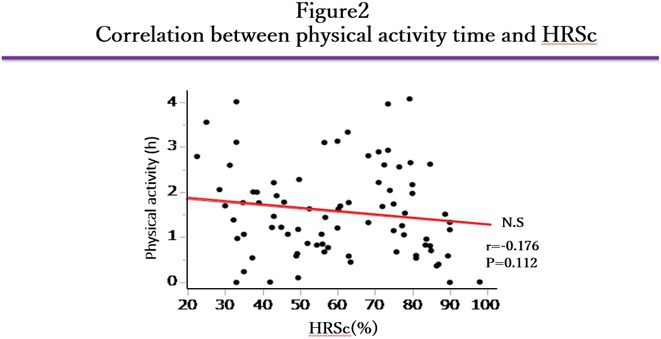

## CHARACTERISTICS OF PATIENTS REQUIRED PACEMAKER IMPLANTATION AFTER CATHETER ABLATION IN PATIENTS WITH ATRIAL FIBRILLATION

### **TOMONARI KIMURA**, ATSUYUKI WATANABE

#### National Hospital Organization Okayama Medical Center, Okayama, Japan

**Introduction:** Catheter ablation (CA) become more common as a treatment for patients with atrial fibrillation. Although pacemaker implantation was sometimes needed after CA revealing the bradycardia, the background of patients requiring pacemaker implantation after CA for atrial fibrillation has not been fully understood.

**Methods:** We conducted a retrospective analysis using database of Okayama national medical center and identified patients with atrial fibrillation who underwent CA between April 1, 2021 and March 31, 2024.

**Results:** The subjects of this study were comprised of 313 patients underwent CA and 8 patients underwent pacemaker implantation after CA for atrial fibrillation.The patients underwent pacemaker implantation includes 7 patients with paroxysmal atrial fibrillation, and 1 patient with common atrial flutter. The causes of pacemaker implantation were sick sinus syndrome in 7 patients (tachycardia‐bradycardia syndrome in 3 patients) and atrioventricular block in 1 patient. Recurrence of atrial fibrillation or atrial tachycardia was not observed in those patients who underwent pacemaker implantation. Baseline comorbidities included multiple myeloma, end‐stage renal failure requiring hemodialysis, and ischemic heart disease with coronary artery bypass surgery. The mean age was 81±6 y.o (range: 70‐88 y.o), and 4 patients were male. The mean time from CA to pacemaker implantation was 49 days (1‐165 days) and the average of CHAD2S2vasc score was 3.5 (3‐5 point). The mean left atrial diameter was 40.1±5.2mm and left atrial volume index was 55.7±19.6mL/m^2^ on echocardiography. 3D‐voltage mapping showed low voltage area in left atrium in 2 patients, and right atrium in 1 patient.The background of those patients requiring pacemaker implantation after CA was various and the specific predictor of cases required pacemaker could not be found in this study.

**Conclusions:** Although there are a certain number of cases in which pacemaker implantation is required after CA for atrial fibrillation, it is difficult to predict pacemaker implantation before ablation.

## PATIENT SATISFACTION WITH REMOTE MONITORING, CAN WE DO BETTER?

### SETH KINGSTON

#### University of Adelaide, adelaide, Australia

**Introduction:** Remote monitoring provides many benefits for patients with implantable cardiac rhythm management (CRM) devices. While its clinical benefits are increasingly recognized, patient perspectives on remote CRM monitoring utilization remains crucial for optimizing its integration into their care and is not well understood, often going unrecognized.

We aimed to develop and validate a simplified questionnaire to assess patient satisfaction with remote monitoring and determine its impact on their care.

**Methods:** A team of healthcare professionals and focused patient groups developed a comprehensive questionnaire which focused on 5 domains. Direct questions and Likert‐scales were utilized. The studied domains comprised device and monitoring set‐up, patient management of remote monitoring, symptoms management, follow‐up and expectations management, and overall satisfaction of the remote monitoring process. A pilot study was performed to help focus the questionnaire using principal components analysis. Internal consistency was quantified using Cronbach alpha. Patient demographics and device details were captured.

**Results:** A total of 192 patients (61.5% male, mean age 72.3±10.5) completed the questionnaire, 60% had pacemakers, 22% implantable cardiac defibrillators, 18% loop recorders. 89% of patients utilized at home a CRM remote monitoring device and 11% have the mobile app. The questionnaire comprised a total of 32 questions, and demonstrated good internal consistency (Cronbach's alpha of 0.671, 0.838 and 0.886 for expectations, follow‐up and overall satisfaction domains, respectively). The majority of respondents indicated their satisfaction (mean domain score of 3.9/5 (range 3.5‐4.1). Of the respondents, 98.9% indicated that remote monitoring either had a neutral (62.1%) or positive (36.8%) impact on their lives.

**Conclusions:** We demonstrate that a simple yet comprehensive patient satisfaction questionnaire with regards to remote monitoring is feasible and consistent. The majority of patients report a positive experience. Further studies are required to test the utility of this questionnaire on a larger scale.

## URGENT SEQUENTIAL CATHETER ABLATIONS WITH MECHANICAL CIRCULATORY SUPPORT FOR HEMODYNAMICALLY UNSTABLE ATRIAL FIBRILLATION AND VENTRICULAR FIBRILLATION IN A PATIENT FOLLOWING ACUTE MYOCARDIAL INFARCTION

### **TAKAYUKI KITAI**, YUHEI KASAI, JUNJI MORITA

#### Sapporo Cardiovascular Clinic, Sapporo, Japan

**Introduction:** Arrhythmia management following acute myocardial infarction (AMI) can sometimes present significant challenges. The emergence of new‐onset atrial fibrillation (AF) in the context of AMI is linked to increased morbidity and mortality, while ventricular fibrillation (VF) storm represents a life‐threatening complication that may follow AMI. We report a case where both of these arrhythmias were successfully treated with catheter ablation.

**Methods:** NA

**Results:** A 72‐year‐old male presented with acute myocardial infarction and cardiogenic shock, attributed to the left anterior descending artery as the culprit vessel, and underwent emergency percutaneous coronary intervention (PCI) with Impella support. On Day 3, pulmonary vein isolation was undertaken to manage atrial fibrillation that was contributing to hemodynamic instability. By Day 7, following hemodynamic stabilization achieved through the maintenance of sinus rhythm, the Impella device was replaced with an intra‐aortic balloon pump. However, on Day 9, recurrent premature ventricular contractions triggered ventricular fibrillation, necessitating multiple episodes of electrical defibrillation under general anesthesia and amiodarone infusion. Subsequently, catheter ablation targeting the ventricular fibrillation was performed, with successful suppression achieved through Purkinje de‐networking in the left anterior fascicle.

**Conclusions:** This case highlights the challenges and effective strategies of managing complex arrhythmias with catheter ablation in hemodynamically unstable patients following AMI.

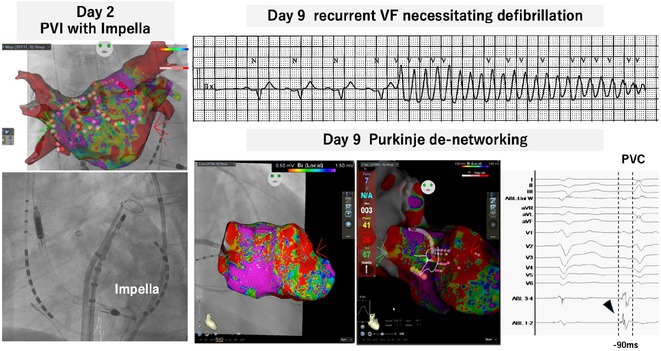

## THE RISK FACTORS AND OUTCOMES IN THE FATAL INTERSTITIAL PNEUMONIA ASSOCIATED WITH AMIODARONE

### **MAKOTO KOBAYASHI**, MASATO HACHISUKA, HIROAKI HIRAYAMA, SHUHEI OKAJIMA, NOBUAKI ITO, SERINA KOBAYASHI, REI MIMURO, YUHI FUJIMOTO, YOSHIAKI KUBOTA, HIROSHIGE MURATA, YOSHIYASU AIZAWA, KENJI YODOGAWA, WATARU SHIMIZU, KUNIYA ASAI, YU‐KI IWASAKI

#### Nippon Medical School Hospital, Tokyo, Japan

**Introduction:** Oral Amiodarone (AMD) is commonly prescribed for both atrial and ventricular tachyarrhythmias in the clinical practice, but it could cause interstitial pneumonia (IP) as a serious, potentially life‐threatening adverse effect. The clinical characteristics of severe amiodarone‐induced IP (AMD‐IP) and optimal dosage of AMD have not been fully elucidated. This study aimed to clarify risk factors associated with mortality in patients with AMD‐IP in a large number of patients prescribed AMD.

**Methods:** A total of 1159 patients (792 men, age 69±13 years) who were prescribed AMD from 1998 to 2023 at our hospital were retrospectively enrolled. Among the patients admitted with AMD‐IP, the clinical parameters were compared between the patients diagnosed with AMD‐IP and those who did not develop it.

**Results:** Nineteen patients (1.64%) were admitted with AMD‐IP. Compared these 19 patients and the remaining 1140 patients who were not developed AMD‐IP, there was no difference in age of AMD prescription initiation (72.5±11.4 years vs. 69.0±12.9 years, *P*=0.24), gender (men 68.4% vs. 68.3%, *P*=0.99) and the cumulative dose of administered AMD (168.5±250.7g vs. 77.5±107.3g, *P*=0.13) between the two groups. However, the 19 patients admitted with AMD‐IP had a significantly higher mortality rate (42.1% vs. 8.2% *P*<0.01). Furthermore, detail analyzed was performed in 19 patients with AMD‐IP to elucidate the risk factors for mortality. Among the 19 patients, the 5 patients who died directory associated with AMD‐IP were significantly heavier (67.2±15.0 kg vs. 49.4±10.5 kg *P*=0.01) and had a higher BMI (24.5±3.1 vs. 20.7±2.9 *P*=0.03) compared to the 14 survived patients. Additionally, these 5 patients were more likely to have received steroid therapy (100% vs. 50% *P*=0.07) and had a higher prevalence of previous catheter ablation for atrial tachyarrhythmias (60% vs. 7.1% P=0.04).

**Conclusions:** Although the incidence of AMD‐IP was not so often (0.43%), AMD‐IP could be fatal especially in the patients with higher BMI. Determining the optimal dosage and duration of AMD administration that minimizes the risk of such severe side effects is essential for improving patient outcomes.

## USEFULNESS OF LAT HISTOGRAM AND COMPARISON OF MAPPING CATHETERS IN PATIENTS WITH LEFT ATRIAL TACHYCARDIA AFTER ABLATION FOR ATRIAL FIBRILLATION

### **CHISATO KOCHI**, TSUNESUKE KONO, DAISUKE YAMAGISHI

#### Nagano Chuo Hospital, Nagano, Japan

**Introduction:** Some atrial tachycardia (AT) seen after treatment of atrial fibrillation (AF) have circuits that are complete only on the endocardial side, while others extend to the epicardial side. Circuit identification of such ATs and their treatment have not been established. In our clinic, the epicardial‐endocardial junction (entrances or exits) is identified and ablated using the LAT histogram from CARTO®︎3, and the effectiveness of this treatment was investigated.

**Methods:** The number of points, map time, analysis time, junction identification rate and termination rate were compared for 40 consecutive ATs mapped with PENTARAY® from October 2021 to November 2022 and 40 consecutive ATs mapped with OCTARAY^TM^ from November 2022 to November 2023. For the LAT histogram analysis method, points were acquired uniformly throughout the left atrium. The point immediately before and after the area of the trough in the LAT histogram was defined as Exit and Entrance, respectively.

**Results:** The number of points was higher in the OCTARAY^TM^ group, map time and analysis time were shorter in the OCTARAY^TM^ group, and there was no significant difference between PENTARAY® and OCTARAY^TM^ groups in terms of termination. The PENTARAY® and OCTARAY^TM^ groups combined had a 47.5% termination rate, a 72.55% CL change rate, and a 93.75% termination or CL change rate.

**Conclusions:** The number of electrodes in PENTARAY® and OCTARAY^TM^ was different, and although there was a significant difference in the number of points and map time, there was no significant difference in short‐term results. The LAT histogram is considered to be an effective analysis tool.

## PREDICTORS OF SEVERITY OF DEPRESSIVE SYMPTOMS IN ATRIAL FIBRILLATION

### **YOULIN KOH** ^1^, MICHAEL CHUN‐GEE WONG^1,2^, PETER KISTLER^3^, CHRISTOPHER DAVEY^1^, JONATHAN KALMAN^1^

#### ^1^Royal Melbourne Hospital, Melbourne, Australia,^2^Western Health, Melbourne, Australia,^3^Alfred Hospital, Melbourne, Australia

**Introduction:** Up to 30% of atrial fibrillation (AF) patients have significant depressive symptoms. Low socioeconomic status (SES) is linked to higher AF risk and may exacerbate these symptoms. In a consecutive cohort of AF patients screened for depressive symptoms, we sought to determine predictors of depression severity by examining SES factors according to the Australian Bureau of Statistics.

**Methods:** We screened 106 consecutive AF outpatients from three Melbourne hospitals using the Patient Health Questionnaire‐9 (PHQ‐9). Higher scores indicate more severe depressive symptoms (0‐27). SES data included Cultural and Linguistically Diverse (CALD) status, highest educational level, occupational skill level, weekly income and index of social support (10‐70; higher scores indicate better support). A multivariable linear regression with PHQ9 score as the outcome was performed, adjusting for age, sex, and AF type.

**Results:** Depressive symptoms varied among patients: 22.9% had minimal (PHQ‐9 score 1‐4), 21% mild (5‐9), 16.2% moderate (10‐14), 9.5% moderate to severe (15‐19), and 4.8% severe (20‐27). Model estimates show the change in PHQ‐9 score associated with each predictor:

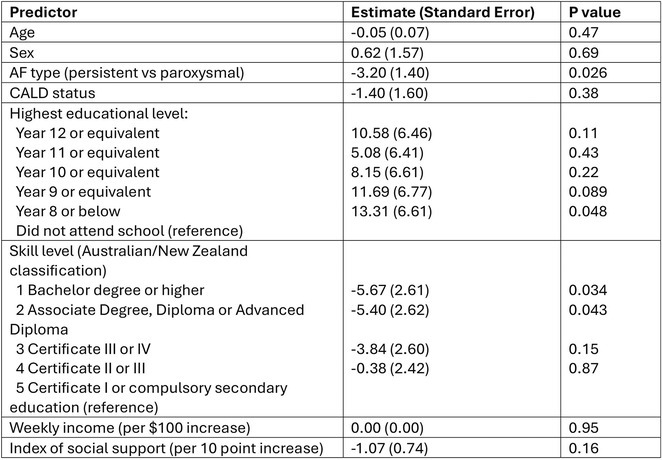

**Conclusions:** Lower secondary educational level was associated with higher PHQ9 score. Higher occupational skill and persistent AF were associated with lower PHQ9 score.

## EFFICACY OF VEIN OF MARSHALL ETHANOL INFUSION COMBINED WITH ANATOMICAL ABLATION FOR THE TREATMENT OF PERSISTENT ATRIAL FIBRILLATION

### **MASATERU KONDO**, HIDEAKI ENDO, YUTA KAGAYA, KENJIRO SATO, HIROKI SAITO, MASANORI KANAZAWA, MASANOBU MIURA, AKIHIRO NAMAMURA

#### Iwate Prefectural Central Hospital, Morioka, Japan

**Introduction:** Radiofrequency (RF) ablation alone is challenging for achieving mitral isthmus (MI) block. We here report two cases of persistent atrial fibrillation (AFib) with perimitral atrial flutter, which treated by anatomical RF ablation and added vein of Marshal (VOM) ethanol infusion for achieving MI block. We here report two cases of persistent atrial fibrillation (AFib) with perimitral atrial flutter, which treated by anatomical RF ablation and added vein of Marshal (VOM) ethanol infusion for achieving MI block.

**Methods:** N/A

**Results:** Case 1: A 62‐year‐old man was referred to our hospital because of symptomatic persistent AFib. He underwent successful catheter ablation as follows: First, pulmonary vein and superior vena cava were isolated, and cavotricuspid isthmus block line was created. Second, the pulmonary vein was isolated again, and posterior wall of left atrium (LA) was isolated. And finally, treatment for perimitral atrial tachycardia was unsuccessful with RF ablation, so the VOM ethanol infusion was performed and the MI block was created. Case 2: A 69‐year‐old man was treated for persistent AFib with perimitral atrial flutter. He had twice pulmonary vein isolation and posterior LA wall isolation. However, he needed a third session of catheter ablation due to recurrence of atrial tachycardia. We first performed the VOM ethanol infusion because perimitral flutter was induce. The tachycardia cycle length prolongation was confirmed. After additional RF ablation, the perimitral flutter was terminated and the MI block was completed.

**Conclusions:** Take together, VOM ethanol infusion showed the acute effect of mitral isthmus block creation.

## SUBSTRATE MAPPING STRATEGIES AND FRACTION MAP ANALYSIS FOR SCAR‐RELATED VENTRICULAR TACHYCARDIA

### **HIROYUKI KONO**, KENICHI HIROSHIMA, KOMEI ONUKI, MAIKO KURODA, REI KUJI, TOMONORI KATSUKI, KENGO KORAI, MASATO FUKUNAGA, MICHIO NAGASHIMA, KENJI ANDO

#### Kokura Memorial Hospital, Kitakyushu, Japan

**Introduction:** Estimating the critical isthmus and successfully eliminating delayed potentials (DPs) and local abnormal ventricular activities (LAVAs) are crucial strategies for treating scar‐related ventricular tachycardia (VT). However, the identification of slow conduction becomes complex when the myocardial substrate is intricate.

**Methods:** We analyzed data from 42 consecutive patients who underwent VT ablation using the Ensite system at our hospital from 2018 to 2023.

**Results:** The mean age of the patients was 65 ± 16 years, with 79% being male. Their preoperative left ventricular ejection fraction was 37.0 ± 11.4%. Sixteen patients had ischemic cardiomyopathy, twenty had an implantable cardioverter‐defibrillator (ICD) implanted, and nine experienced an electrical storm. Mapping was performed in the right ventricle for six patients, the left ventricle for 38 patients, and epicardially for five patients. Effective fractionation threshold counts were 4.0 for four patients, 5.0 for twenty patients, 6.0 for sixteen patients, and 7.0 for two patients. Eighty‐six percent of patients exhibited DPs, with an average coverage of 70 ± 34% within the fraction map. Sixty‐nine percent had LAVAs, with an average coverage of 92 ± 20% within the fraction map. The fraction map considered optimal covered 5.9 ± 3.9% of the points obtained.

**Conclusions:** Fractionated potentials, which characterize conduction delays, showed a strong correlation between delayed potentials and local abnormal ventricular activities. This correlation is crucial for the precise mapping and successful ablation of VT circuits.

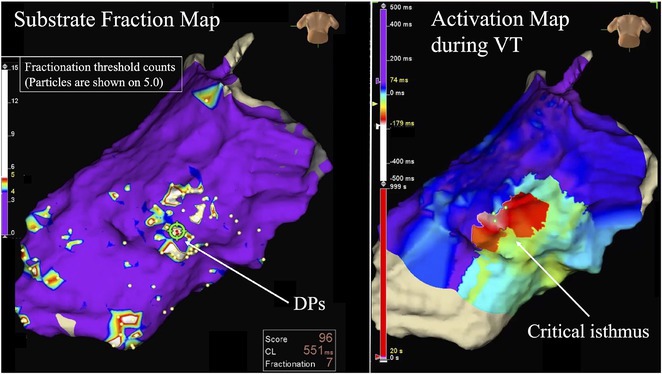

## INVESTIGATION OF THE USEFULNESS OF HIGH‐POWER SHORT‐DURATION ABLATION WITH THE QDOT MICRO™ CATHETER FOR ATRIAL FIBRILLATION

### **TSUNESUKE KONO**, CHISATO KOCHI, DAISUKE YAMAGISHI

#### Nagano Chuo Hospital, Nagano, Japan

**Introduction:** RF ablation of atrial fibrillation using the CARTO®3 system has generally been performed with the VISITAG SURPOINT® guide. With the recent introduction of the QDOT MICRO™ Catheter, high‐power short‐duration ablation (HPSD) is now possible. We report the efficacy of pulmonary vein isolation (PVI) and posterior wall isolation (PWI) with HPSD (90W4 seconds) and VISITAG SURPOINT® guide (value = 400) in 60 patients with persistent atrial fibrillation undergoing first‐time ablation in 2023.

**Methods:** Acute success rate and time were examined in the 30 cases in which HPSD was performed from 6/19/2023 to 10/30/2023 (HPSD group) and in the 30 cases in which VISITAG SURPOINT® guided (VS group) was performed from 2/2/2023 to 6/2/2023 (VS group).In both groups, the procedure was performed with one puncture and one sheath to the left atrium, PV potential monitoring was not performed during ablation, and isolation was considered complete when the 6 mm diameter automated tag overlapped.After PVI and PWI were completed, mapping was performed with OCTARAY, and if residual potentials were present with a cutoff of 0.1 mV, additional ablation was performed at 50 W up to VISITAG SURPOINT® 400.

**Results:** Door‐to‐door time (145.4±23.1 vs 132.2±18.9 P=0.02) and fluoroscopy time (17.3±6.6 vs 11.8±4.0 P<0.001) were significantly shorter in the HPSD group. The 1‐pass rate of initial ablation was significantly higher in the VS group for both PVI alone (57% vs. 20% P=0.003) and PWI alone (48% vs. 18% P=0.04).Local residual potentials were not significantly different for RPV (23% vs. 33%), while LPV (27% vs. 60% P=0.009) was significantly higher in the HPSD group.

**Conclusions:** In this study, the one‐pass rate was higher in the VS group and the therapy was more effective. However, fluoroscopy and door‐to‐door time were significantly shorter in the HPSD group, confirming the effectiveness of HPSD. However, the HPSD group had more LPV local residual potentials, which is an issue in the future.

## FACTORS ASSOCIATED WITH THE NON‐IMPROVEMENT IN LEFT VENTRICULAR EJECTION FRACTION DESPITE THE ABSENCE OF ATRIAL FIBRILLATION RECURRENCE AFTER CATHETER ABLATION

### **TERUMASA KOYAMA** ^1^, SOU TAKENAKA^2^, AYANO ENZAN^1^, TOMOKO TAMADA^1^, KOICHIRO IMAI^1^, RYOTARO YAMADA^1^, TERUYOSHI KUME^1^, YOJI NEISHI^1^, SHIRO UEMURA^1^

#### ^1^Kawasaki Medical School, Okayama, Japan,^2^Ism Katsushika Heart Center, Tokyo, Japan

**Introduction:** The maintenance of sinus rhythm through catheter ablation (CA) has been shown to significantly enhance left ventricular ejection fraction (LVEF) in patients with atrial fibrillation (AF). However, in certain cases, a decline in LVEF persists even after successful CA. This study aimed to identify predictors associated with the non‐improvement of LVEF in patients with AF who underwent CA.

**Methods:** Out of 348 patients who underwent CA for AF and remained free of AF recurrence at the 12‐month mark, 73 individuals with LVEF < 50% at baseline were analyze. Among these, 44 patients who underwent follow‐up transthoracic echocardiography (TTE) at 6 months were included in the study (age 66.0 ± 8.9 years, 75% male). The patients were categorized into two groups base on the improvement of LVEF at follow‐up, improvement group (LVEF ≧ 50% at 6 months) and the non‐improvement group (LVEF < 50% at 6 months).

**Results:** The non‐improvement group exhibited a higher prevalence of paroxysmal AF, elevated levels of E/e', and a higher CHA2DS2‐VASc score at baseline (71 vs 19 %, P<0.01, 17.0 vs 12.9, p=0.04, 3.5 vs 2.5, p=0.02, respectively). The mon‐improvement group showed a higher incidence of composite cardiac comorbidities, including coronary artery disease, between the two groups (94 vs 44 %, p<0.01).

**Conclusions:** The persistence of reduced LVEF following successful CA was associated with factors such as paroxysmal AF, elevated E/e' levels, and the presence of composite cardiac comorbidities at baseline.

## ZERO FLUOROSCOPY SVT ABLATION ‐ A SINGLE CENTER EXPERIENCE

### **PREETAM KRISHNAMURTHY**, MURALIDHARAN THODDI RAMAMURTHY, DIVYA SHRIDHAR, VINODKUMAR BALAKRISHNAN

#### Sri Ramachandra Insitute for Higher Education and Research, Chennai, India

**Introduction:** Fluoroscopy imaging has been the mainstay in majority of electrophysiology procedures. Fluoroscopy is associated with significant radiation exposure to patient, operator and paramedical workers. With evolution of technology, it is possible to achieve similar results without fluoroscopy in patients undergoing supraventricular tachycardia. This study aims to compare the operative efficacy of zero fluoroscopy with use of impedance‐based 3D mapping system (Ensite Precision) with fluoroscopy.

**Methods:** The study is a retrospective, single‐centre study in which patients who underwent Electrophysiology study with radiofrequency ablation for cardiac arrythmias from January 2021 to December 2023 at Sri Ramachandra Institute of Higher Education and Research (SRIHER) were included. The mapping and ablation was done with use of Claris system and fluoroscopy in fluoroscopy arm and with Claris system and Ensite Precision system in the zero fluoroscopy arm. Baseline characteristics including diagnosis, comorbid conditions, and interventional details were collected and analysed. No intracardiac imaging was used. The primary outcome of the study was to compare the procedural times, complications, and recurrence between zero fluoroscopy and fluoroscopy‐based electrophysiology procedures.

**Results:** The baseline characteristics including the type of arrhythmia, age, sex, height and weight were similar in both fluoroscopy and zero fluoroscopy procedures. Totally, 104 patients were included in the study. Zero fluoroscopy was done in 61 patients compared to 43 patients with conventional method. The total time of procedure (85 min vs 88 min, p value = 0.07) and the total ablation time (4 min vs 3.5 min, p value = 0.08) was not significant between the 2 groups. The success rates were similar and no recurrences were noted post procedure in both the groups

**Conclusions:** Zero fluoroscopy ablation is a feasible alternative to conventional fluoroscopy based radiofrequency ablation and has similar procedure times and success rates

## EXTERNAL VALIDATION OF ABLATION SITE CLASSIFICATION FOR ATRIAL FIBRILLATION ABLATION USING THE CARTONET® R14 MODEL

### KAZUHIKO KUINOSE

#### Saitama Medical University International Medical Center, Hidaka, Saitama Prefecture, Japan, Japan

**Introduction:** The CARTONET R14 employs deep learning algorithms to automate ablation site classification and predict reconnection sites. However, the accuracy of its site classification model and the trends of its site prediction model for potential reconnection sites remain uncertain.

**Methods:** This study included 33 cases from December 22, 2023, to March 29, 2024. Sixteen patients underwent pulmonary vein isolation (PVI), including cavotricuspid isthmus (CTI) ablation (PVI group), and 17 underwent PVI with additional ablations (e.g., box isolation) (PVI+ group). We investigated the sensitivity and positive predictive value (PPV) of automatic site classification in the total cohort and conducted a subgroup analysis comparing the PVI group with the PVI+ group. The distribution of potential reconnection sites and the confidence level for each site were also analyzed.

**Results:** A total of 2,747 points were analyzed. In the right pulmonary vein (PV), the sensitivity and PPV were as follows: inferior (92.8%, 94.2%); anterior (98.9%, 84.8%); roof (89.3%, 84.7%); posterior (94.8%, 87.0%); carina (96.4%, 67.5%). In the left PV: inferior (83.1%, 87.3%); anterior (83.5%, 100%); ridge (91.8%, 95.2%); roof (94.0%, 96.6%); posterior (85.1%, 85.6%); carina (none, 0%). In the roofline (66.7%, 40.0%), posterior line (5.0%, 3.8%), and CTI (86.5%, 94.8%), the superior vena cava was neither recognized nor analyzed. The potential reconnection prediction model indicated the highest incidence of potential reconnections in the posterior, with the highest confidence in the roof.

**Conclusions:** The CARTONET® R14 model demonstrates relatively high accuracy in classifying ablation sites in the pulmonary veins, excluding the carina. The model's prediction of potential reconnection sites tends to identify areas with poor catheter stability as likely reconnection sites.

## TRENDS OF THE PREDICTION MODEL FOR THE POTENTIAL RECONNECTION SITE USING CARTO NET R14 MODEL

### KAZUHIKO KUINOSE

#### Saitama Medical University International Medical Center, Hidaka, Saitama Prefecture, Japan, Japan

**Introduction:** The CARTONET R14 model is a cloud‐based management system that allows for retrospective analysis of the ablation procedure using deep learning technology. Among this system, a prediction model for the potential reconnection site can predict the reconnection site, based on each ablation data. However, the detailed examination of this model was not well investigated.

**Methods:** The detailed ablation data of 33 patients undergoing atrial fibrillation ablation were analyzed by using the CARTONET R14 model. The frequency of potential reconnection sites and the percentage of potential reconnection were investigated.

**Results:** A total of 158 segments were predicted as a potential reconnection site. The frequency of potential reconnection sites of the left pulmonary vein (LPV) (n=84 [64.6%]) was higher than that of the right pulmonary vein (RPV)(n=48 [35.4%]). The frequency of potential reconnection sites was highest in the posterior wall for both the RPV and LPV (RPV, n=18 [39.6%])(LPV, n=23 [35.7%]). Among the confidence of gap prediction of RPV, the percentage of potential reconnection was highest at the right roof area (14% [8‐33%]), and significantly higher than that of the right anterior and posterior area. The percentage of potential reconnection of LPV was also highest in the roof area, the posterior area, and the anterior area (16% [12‐22%], 16%[10‐22%], 16%[12‐22%],).

**Conclusions:** The proximity of the posterior wall to the esophagus, which limits sufficient ablation due to temperature rise, and the instability of the catheter at the roof due to respiratory variations, are factors that could have contributed to these potential reconnections.

## INITIAL RESULTS OF THE NEW SCREW IN LEADLESS PACEMAKER AVEIR VR

### **REI KUJI**, MICHIO NAGASHIMA, KOMEI ONUKI, TOMONORI KATSUKI, KENGO KORAI, MASATO FUKUNAGA, KENICHI HIROSHIMA, KENJI ANDO

#### Kokura Memorial Hospital, Kitakyushu, Japan

**Introduction:** Leadless pacemakers have become a major breakthrough in the management of bradyarrhythmia as an attractive alternative to the standard transvenous pacemakers. We've been able to use new leadless pacemaker (Aveir VR Abbott medical) since January 2023.The Aveir VR has a removable structure and is expected to reduce the number of implantations by pre‐mapping before implantation. We report the initial results and short‐term follow‐up of Aveir VR implantation.

**Methods:** We underwent Aveir VR implantation procedures to 55 patients between March 2023 and January 2024. The implantation criteria for pre‐mapping were an injury current of 1 mV or greater, a threshold of 2.0 V/0.4 ms or less, and a wave height of 3.0 mV or greater. Patient characteristics, implantation procedure success rate, number of deployments, and complications were reviewed.

**Results:** The mean ages were 84.1±6.6years (69‐94), 26 men (47.3%). The procedure time was 29.9±21.7 minutes, the fluoroscopy time was 4.99±2.12 minutes. Primary indication for pacemaker were sick sinus syndrome (37 cases(67.3%)), AF brady(6 cases(10.9%)), AV block (12 cases(21.8%)). Successful implantation in 54 (98.2%) of 55. In one unsuccessful case, the vessel was so tortuous that torque could not be transmitted and implantation was not possible. Dislodge was not observed. Fifty‐one (94.4%) of 54 did not require repositioning after the first implantation. There were no other complications.

**Conclusions:** The availability of pre‐mapping reduced the number of deployments and allowed for safe implantation without complications.

## COMPARISON OF TOUCH‐UP ABLATION RATE AND PULMONARY VEIN ISOLATION DURABILITY BETWEEN TWO CRYOBALLOON SYSTEMS

### **HIROFUMI KUJIRAOKA**, RINTARO HOJO, FUMIYA YOKOZEKI, MASANAO HOMMA, MASATAKA SUNAGAWA, WATARU TSUNO, YOSHIAKI MIZUNUMA, TAKAFUMI SASAKI, KOICHIRO YAMAOKA, TOMOYUKI ARAI, KIYOTAKA YOSHIDA, KENSUKE KASANO, MASAO TAKAHASHI, TAKAAKI TSUCHIYAMA, SEIJI FUKAMIZU

#### Tokyo Metropolitan Hiroo Hospital, Tokyo, Japan

**Introduction:** Single‐shot pulmonary vein isolation (PVI) utilizing cryothermal energy is an effective and safe treatment for atrial fibrillation (AF) patients. A novel cryoballoon system, POLARx™, has been recently introduced. The aim of this study was to compare PVI durability, touch‐up ablation (TUA) rate and procedural parameters of PVI between the novel cryoballoon system, POLARx™, and the standard cryoballoon system, Arctic Front Advance™ (AFA), in patients with paroxysmal AF.

**Methods:** This single‐center observational study included 150 patients diagnosed with paroxysmal AF who underwent PVI using a cryoballoon during their initial treatment session (POLARx N=44, AFA N=106). Patients were recommended to undergo electrophysiological evaluations and additional ablation procedures to check LA‐PV reconnections regardless of atrial tachyarrhythmia recurrence approximately 6 months after the first session. Patients who underwent second session were divided into two groups by cryoballoon system used. The PVI durability, TUA rate and PV antrum isolated area were compared between these two groups.

**Results:** Ninety‐one patients attended the second session (POLARx N=15, AFA N=76). There was no significant difference observed in the durability of PVI between the two groups (POLARx 49/60: 81.7% vs AFA 265/304: 87.2%; P > 0.05). The durability of each PV was also not significantly different (LS, 80.0% vs 92.1%; LI, 86.7% vs 92.1%; RS, 73.3% vs 90.8%; RI, 86.7% vs 73.7%, P>0.05 respectively). TUA was performed for 7 PVs in the POLARx group (LS, 1; LI, 2; RS, 2; RI, 2) and 37 PVs in the AFA group (LS, 4; LI, 4; RS, 2; RI, 27), respectively (POLARx 14.6% vs AFA 12.2%; P > 0.05). There was no significant difference in the ratio of PV antrum isolated area to left atrial posterior wall area (POLARx 48.4% vs AFA 53.4%; P > 0.05).

**Conclusions:** The durability of PVI was similar between the two cryoballoon systems, POLARx™ and AFA.

## THE IMPACT OF VENTRICULAR SEPTAL ANGLE ON OPERATION TIME IN LEADLESS PACEMAKER IMPLANTATION

### **MITSUHIRO KUNIMOTO** ^1^, HARUNA TABUCHI^1^, KIKUO ISODA^1^, TOHRU MINAMINO^2^

#### ^1^Juntendo University Nerima Hospital, Tokyo, Japan,^2^Juntendo University Graduate School of Medicine, Tokyo, Japan

**Introduction:** The Micra Leadless Pacemaker (MLP) has emerged as an effective alternative to conventional transvenous pacemakers, reducing the operation time. However, the potential influence of the ventricular septal angle on MLP implantation time remains unexplored. This study aimed to elucidate the relationship between the ventricular septal angle and the duration of MLP implantation.

**Methods:** A retrospective analysis was conducted on consecutive MLP implantation cases performed by a single practitioner at our institution from July 2022 to March 2024. The ventricular septal angle was manually measured from pre‐implantation computed tomography (CT) scans (Figure1). The operation time, defined from delivery sheath insertion to device release, was extracted from electronic medical records.

**Results:** Out of 19 MLP implantations, 16 cases with pre‐procedural CT were included, comprising 11 males and 5 females with an average age of 83.4 ± 9.0 years. Diagnoses included atrioventricular block (8 cases), slow atrial fibrillation (4 cases), and sick sinus syndrome (4 cases). The mean operation time was 15.6 ± 8.7 minutes, with a ventricular septal angle averaging 37.0 ± 11.6 degrees. A significant inverse correlation was observed between operation time and the ventricular septal angle (r=‐0.74, P<0.01; Figure2). Multivariate analysis identified the ventricular septal angle as a significant predictor of operation time (β=‐1.04, P<0.01).

**Conclusions:** The ventricular septal angle measured by CT scan is significantly correlated with the operation time for MLP implantation, suggesting its utility as a predictive measure in procedural planning.

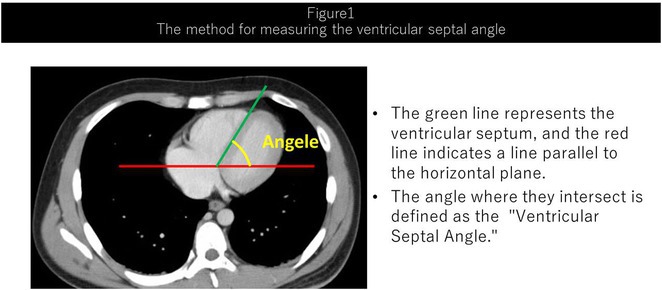

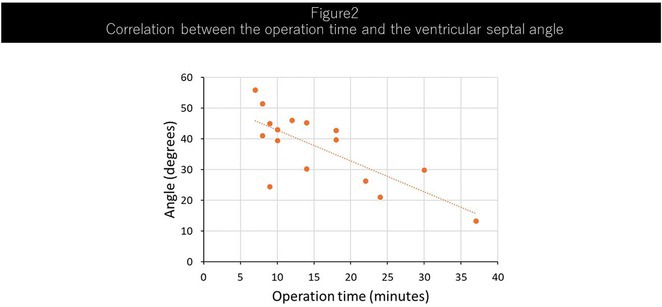

## THE POTENTIAL OF A NOVEL AUTOMATED PEAK FREQUENCY MAP: A SUPERIOR APPROACH TO MITIGATING THE IMPACT OF DIVERSE WAVEFRONTS COMPARED TO VOLTAGE MAPS

### **MINGJEN KUO** ^1^, LI‐WEI LO^2^, YENN‐JIANG LIN^2^, SHIH‐LIN CHANG^2^, YU‐FENG HU^2^, FA‐PO CHUNG^2^, CHIN‐YU LIN^2^, TING‐YUNG CHANG^2^, SHIH‐ANN CHEN^1^

#### ^1^Taichung Veterans General Hospital, Taichung, Taiwan,^2^Taipei Veterans General Hospital, Taipei, Taiwan

**Introduction:** The optimal method for assessing left atrium (LA) substrate during atrial fibrillation (AF) remains unclear. We aimed to compare the HD wave voltage and peak frequency (PF) between AF and sinus rhythm (SR).

**Methods:** Patients with persistent AF who underwent de novo pulmonary vein isolation (PVI) were included, while those exhibiting significant low voltage zones during SR were excluded. Bipolar and omnipolar voltage (BV/OV), and bipolar and omnipolar peak frequency (BPF/OPF) of LA during both AF and SR following PVI were compared.

**Results:** Fifteen patients (10 males, aged 60.9 ± 8.5 years) were included. Analysis revealed a significant difference in global LA voltage between different rhythms (BV AF vs. SR: 0.90 ± 0.36 vs. 1.74 ± 0.63, *P* < 0.01; OV AF vs. SR: 1.23 ± 0.51 vs. 2.46 ± 0.85, *P* < 0.01). However, no significant difference was observed in PF maps between different rhythms (BPF AF vs. SR: 271.93 ± 30.62 vs. 284.38 ± 41.39, *P* = 0.149; OPF AF vs. SR: 280.52 ± 32.35 vs. 287.91 ± 39.38, *P* < 0.349). As illustrated in Figure panel A, there is no significant difference for PF between AF and SR in various LA regions. Additionally, no region exhibited BPF lower than 190 Hz and OPF lower than 230 Hz during AF, values previously defined as scar in our prior study. An example case is depicted in Figure panel B.

**Conclusions:** The PF AF maps demonstrate a strong correlation with PF maps during SR than the voltage map. The PF map exhibits the potential to overcome the impact of varying wavefronts even in the presence of chaotic wavefronts during AF.

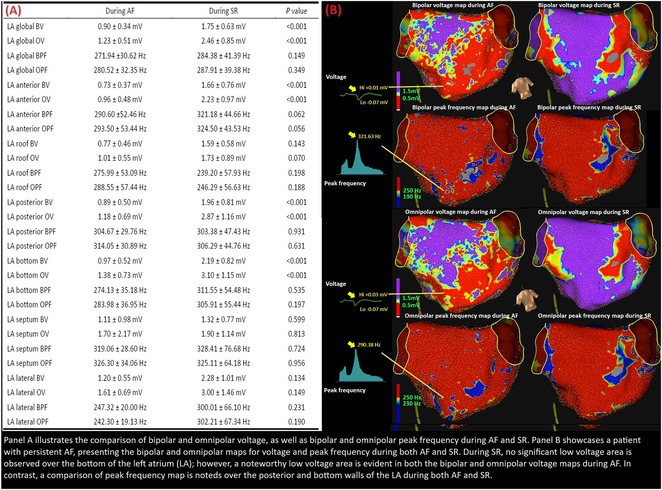

## COMPARISON OF MITRAL ISTHMUS LINEAR ABLATION BETWEEN ANTERIOR MITRAL LINE AND LATERAL MITRAL ISTHMUS

### **SAYANA KURAOKA**, MASATSUGU NOZOE, TORU KUBOTA

#### Saiseikai Fukuoka General Hospital, Fukuoka, Japan

**Introduction:** Two types of mitral linear ablation are performed for mitral flutter or as a stepwise approach for atrial fibrillation (AF). The purpose of the present study was to compare the efficacy and safety of mitral linear ablation between anterior mitral line (AML) and lateral mitral isthmus(LMI).

**Methods:** We retrospectively analyzed consecutive 41 cases of mitral flutter ablation from 2013 to 2023 (AML n=15, LMI n=26).

**Results:** Successful electrical bi‐directional block line was confirmed in 13 of 15 cases (86.7%) in AML group and 19 of 26 cases (73.1%) in LMI group. Recurrence of mitral flutter were observed 1 case of AML groups (6.67%) and 8 cases of LMI groups (72.7%). Recurrence rate of atrial arrhythmias including AF was 21.4% (3/15) in AML group and 42.3% (11/26) in LMI group (P = 0.15). One patient in AML group and 10 patients in LMI group were received re‐ablation including chemical ablation for Marshal vein. We also analyzed consecutive 31 cases of AF ablation treated by liner ablation of mitral isthmus as a stepwise approach (AML n=15, LMI n=16). Successful electrical bi‐directional block line was confirmed in 14/15 cases (93.3%) in AML group and 14/16 cases (87.5%) in LMI group. Recurrence rate of atrial arrhythmias including AF was 40% (6/15) in AML group, and 50% (8/16) in LMI group (P=0.81). 5 patients in AML group and 6 patients in LMI group were received re‐ablation. There were no cases of recurrence after 2^nd^ session in LMI group, while there were 3 cases of AF recurrence in AML group.

**Conclusions:** There was no difference in the recurrence rate of atrial arrhythmias including AF between AML group and LMI group. It remains undetermined whether poor outcomes of LMI ablation might be overcome by chemical ablation of Marshal vein.

## VENOPLASTY AT TIGHTLY STENOTIC LESION OF LEFT BRACHIOCEPHALIC VEIN IN PATIENT WITH RIGHT VENTRICULAR LEAD REPLACEMENT

### **JAE‐JIN KWAK**, JUNE NAMGUNG, JUSIK KIM, INYEONG BANG

#### Inje Univ. Ilsan Paik Hospital, Goyang, Korea, Republic of

**Introduction:** A 76‐year‐old man’ right ventricular(RV) lead polarity switched from bipolar pace‐sense to unipolar pace‐sense, spontaneously, at one‐year post‐implantation. Automatic pace‐sense polarity switch was considered as an indicator of early lead corrosion. Finally, the RV lead impedance was to values > 3,000 ohms.

**Methods:** NA

**Results:** The extraction of old RV lead was not difficult. However, a guide wire for new lead could not pass through the left brachiocephalic vein. The venography showed many collateral veins and total occlusion of left brachiocephalic and subclavian veins. We changed guide wire to Glidewire (0.035”, Terumo international system, Japan). The glidewire could pass through a tight lesion in left brachiocephalic vein with difficulty but the dilator of seven French introducer could not open the occlusion of left brachiocephalic vein. We used Mustang balloon catheter (3.0mm x 40mm x 75cm, Boston scientific, USA) which was usually used for angioplasty of calcified lesion of peripheral artery. An over the wire Mustang balloon was inserted and inflated three times up to 10 atm, but RV lead could not pass through the lesion. So, Mustang balloon was inflated twice to 24 atm. Venography showed open left brachiocephalic vein. The balloon was inflated one more to 24 atm. After then, RV lead was able to pass through the hard stenotic lesion and was affixed to RV septum.

**Conclusions:** Venoplasty with Mustang balloon catheter is feasible in tightly occlusive lesions of left brachiocephalic or subclavian vein.

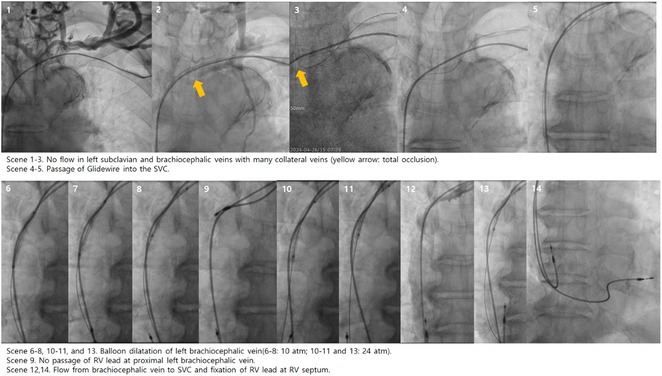

## MULTI‐TASK LEARNING‐BASED ARTIFICIAL INTELLIGENCE FOR SIMULTANEOUS AGE AND GENDER PREDICTION FROM 12‐LEAD ECGS

### **OH‐SEOK KWON**, JE‐WOOK PARK, DAEHOON KIM, HEE TAE YU, HUI‐NAM PAK

#### Yonsei University Health System, Seoul, Korea, Republic of

**Introduction:** Single‐task learning (STL) approaches typically require a separate model for each prediction task, leading to a linear increase in training costs as the number of tasks increases. We propose a multi‐task learning (MTL) model that efficiently predicts both age and gender simultaneously, leveraging shared parameters to reduce resource usage and increase computational efficiency.

**Methods:** We developed a ResNet‐based MTL model incorporating the logarithmic transformation of task loss and gradient normalization to address task imbalance. The MTL model was trained on a dataset of 500,000 ECGs and validated on a holdout dataset of 13,628 ECGs from the MIMIC4 dataset (total: 513,628 ECGs). We benchmarked the performance of the MTL model against the conventional STL models using an independent external dataset, CODE15% (345,779 ECGs).

**Results:** The MTL model was successfully validated for both age and gender prediction tasks, achieving a correlation coefficient *r*=0.79 for age prediction task (T1_age_) and an area under the curve (AUC) = 0.94 for gender prediction task (T2_gender_) on the holdout dataset. In the independent external dataset, T1_age_ achieved equivalent performance with *r* = 0.79, compared to the STL model's *r* = 0.83. Similarly, T2_gender_ also demonstrated equivalent performance with AUC=0.88, compared to the STL model's AUC=0.89. Remarkably, the training time of the MTL model was comparable to that of the STL model for just one task.

**Conclusions:** The MTL model concurrently predicts age and gender with performance equivalent to conventional STL models, significantly enhancing memory usage and training cost efficiency.

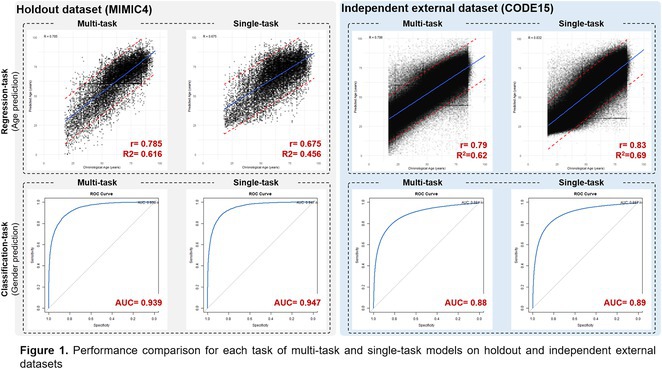

## HETEROGENEITY AND DEFICIENCIES OF CARDIAC MONITORING POST‐STROKE AMONG STROKE CENTERS: DIVERT PHASE I STUDY

### **DHANUNJAYA LAKKIREDDY** ^1^, THOMAS DEVLIN^2^, JOSHUA SNAVELY^3^, KRISHNA POTHINENI^4^, JANA BRAKLOW^4^, BIBHU MOHANTY^5^, MARLA HAIRSTON^5^, ROBERTO CARTA^6^, NORELI C. FRANCO^6^, KARAH B. NEISEN^6^, DAVID Z. ROSE^5^

#### ^1^Kansas City Heart Rhythm Institute, Overland Park, KS,^2^CHI Memorial, Chattanooga, TN,^3^Franciscan Neurology Associates at St. Joseph, Tacoma, WA,^4^Research Medical Center, Kansas City, MO,^5^University South Florida, Tampa, FL,^6^Medtronic Cardiac Rhythm Management, Minneapolis, MN

**Introduction:** Characterization of stroke care pathways and the rate of adherence to guideline‐recommended post‐stroke cardiac monitoring to identify occult atrial fibrillation (AF) across healthcare institutions is unknown.

**Methods:** The DiVERT (SeconDary Stroke PreVEntion ThRough Pathway ManagemenT) Phase I study assessed cardiac monitoring practices post‐stroke and conducted a retrospective analysis between Comprehensive Stroke Centers (CSC) and non‐Comprehensive Stroke Centers (NCSC) for patients hospitalized with ischemic stroke. Collected data included clinician interviews across cardiology and neurology subspecialities, and baseline demographics from electronic health records 6 months post‐index stroke discharge.

**Results:** Overall, 6,347 patients with cryptogenic (78.8%), large artery (15.2%) and small vessel (6.0%) ischemic strokes were identified at 13 stroke centers (CSC n=5 and NCSC n=8). Patients at CSC were younger (66.9±14.0 vs. 68.0±13.9, p=0.003) and more frequently male (52.1% vs. 48.8%, p=0.009), had more cardiology consultations during index hospitalization (14.1% vs. 10.4%, p<0.001), longer length of stay (9.8 days vs. 7.0 days, p<0.001), more frequent use of insertable cardiac monitors (ICM) (5.3% vs. 2.5%, p<0.001) and external monitors (18.0% vs. 2.0%, p<0.001), compared to NCSC. Patients who underwent monitoring had a higher rate of AF detection (9.7% vs. 4.9%, p<0.001). There were no differences observed in the number of patients experiencing cardiovascular‐related healthcare encounters or recurrent strokes through 6 months post‐index stroke at CSC vs. NCSC (31.3% vs. 30.2%, p=0.3486; 6.7% vs. 5.9%, p=0.1551).

**Conclusions:** Ischemic stroke patients were more frequently and intensively monitored for occult AF at CSC than at NCSC, resulting in higher rates of AF detection. However, this may be accounted for by demographic differences and/or disparities in healthcare related age and gender and/or due to greater frequency of cardiology consultations and longer length of stay.

## A SINGLE CENTRE EXPERIENCE OF TRANSVENOUS CARDIAC IMPLANTABLE ELECTRONIC DEVICE LEAD EXTRACTION IN CHILDREN

### **CLAIRE LAWLEY** ^1,2,3^, ANDRIANOS KONTOGEORGIS^4,3^, RONEL TALKER^3^, TSVETA RAHNEVA^3^, LEONIE WONG^3^, JANICE TILL^3^, JONATHAN CLAGUE^3^

#### ^1^Sydney Children's Hospitals Network, Sydney, Australia,^2^The University of Sydney, Sydney, Australia,^3^The Royal Brompton Hospital, London, United Kingdom,^4^Department of Cardiology and Electrophysiology, Interbalkan Medical Centre, Athens Medical Group, Thessaloniki, Greece

**Introduction:** Children with congenital, inherited or acquired electrical disease managed with a conventional transvenous cardiac implantable electronic device (CIED), may face a lifetime of procedures related to their device system. Management of malfunctioning or infected leads or leads that have simply been outgrown may include removal, or it may be required prior to an upgrade to an implantable cardioverter‐defibrillator. Extraction methods have evolved over time. Removal systems have been designed for adults but inevitably need to be used in children. The aim of this study was to explore the trends and outcomes of CIED transvenous lead extraction in a single centre paediatric cohort, 2002‐2023.

**Methods:** A retrospective chart review was undertaken, including all cases of paediatric (age <18 years) lead extraction. Cases were identified through hand searching of theatre and pacing clinic logbooks and via the departmental pacemaker databases (‘PaceNet’, ‘CCAD’). Underlying demographic data, procedural data and outcomes were extracted and examined for trends.

**Results:** Over a 20‐year period, 38 children were identified to have undergone lead removal or extraction. Average age at first transvenous lead implant was 7 years (range 0‐16 years) and average age at lead extraction procedure was 12.5 years (range 6.2‐18 years). Average lead age at time of extraction was 55 months (data for n=34), range 0‐136 months. Seven children had lead age <12 months at time of removal. Four children underwent more than one extraction procedure. The most common indication for implantation was complete congenital heart block (n=15). The most common reason for extraction was lead failure. Methods to remove included manual traction +/‐ combined locking stylets with advanced laser or mechanical rotor in select cases. There were no deaths related to the procedure.

**Conclusions:** Although important progress is being made in designing and implanting leadless systems, safe lead extraction remains a vital technique in children. It is important to understand the risk this presents when weighing the balance between epicardial, leadless and transvenous lead CIEDs.

## ADVANCED ATRIOVENTRICULAR BLOCK IN ATHLETES: PREVALENCE AND ROLE OF ANTI‐RO/SSA‐ANTIBODIES

### **PIETRO ENEA LAZZERINI** ^1^, IACOPO BERTOLOZZI^1^, ALESSANDRA CARTOCCI^1^, KRISHNA MURTHY GINJUPALLI^2^, VIOLA SALVINI^1^, RICCARDO ACCIOLI^1^, ANNA CANTORE^1^, MAURIZIO ACAMPA^1^, PIER LEOPOLDO CAPECCHI^1^, MOHAMED BOUTJDIR^2^

#### ^1^University of Siena [project ECS00000017 Tuscany‐Health Ecosystem—CUP B63C2200068007 spoke 6 Mission 4 Component 2 (M4C2)—investment 1.5 of the National Recovery and Resilience Plan (PNRR) funded by the European Union “Next Generation EU”], Siena, Italy,^2^VA New York Harbor Healthcare System, SUNY Downstate Health Sciences University, New York, NY

**Introduction:** Advanced‐atrioventricular block(AVBs), i.e. higher than second‐degree Mobitz‐1, is abnormal finding in athletes. Despite intensive investigation, in several cases the aetiology remains unknown, but frequently pacemaker implantation is still indicated. Increasing evidence points to circulating anti‐Ro/SSA‐antibodies cross‐reacting with L‐type‐calcium channel(Ca_v_1.2) and inhibiting the related current(I_CaL_) as an epidemiologically relevant and potentially reversible cause of isolated AVB in adults. Aim of this study was to determine the prevalence of anti‐Ro/SSA‐associated advanced‐AVBs in a large sample of young athletes.

**Methods:** 2536 consecutive athletes <40 years without history of cardiac diseases/interventions were enrolled in a cross‐sectional study. Resting and exercise electrocardiography was performed, and those presenting any AVB were further evaluated by 24h‐Holter ECG. Athletes with second‐degree AVBs, and their mothers, underwent anti‐Ro/SSA testing. Moreover, purified immunoglobulins‐G(IgG) from anti‐Ro/SSA‐positive and anti‐Ro/SSA‐negative advanced‐AVB subjects were tested on I_CaL_ and Ca_v_1.2 expression using tSA201 cells.

**Results:** The global prevalence of advanced‐AVB in the overall sample was ~0.1%, but the risk considerably increased(2%) when intensely trained post‐pubertal males were selectively considered. While none of the athletes with advanced‐AVB showed heart abnormalities, in 100% of cases anti‐Ro/SSA‐antibodies were detected. *Ex‐vivo* experiments showed that IgG from anti‐Ro/SSA‐positive but not ‐negative subjects with advanced‐AVB acutely inhibit I_CaL_ and chronically down‐regulate Ca_v_1.2 expression.

**Conclusions:** Our study provides evidence that advanced‐AVB occurs in young athletes, in most cases associated with anti‐Ro/SSA‐antibodies blocking L‐type calcium channels. These findings may open new avenues for immunomodulating therapies to reduce the risk of life‐threatening events in athletes, avoiding or delaying pacemaker implantation.

## SEVERE COVID‐19 ACUTELY DELAYS ATRIOVENTRICULAR CONDUCTION: THE ROLE OF CIRCULATING INTERLEUKIN‐6 LEVELS

### **PIETRO ENEA LAZZERINI** ^1^, RICCARDO ACCIOLI^1^, VIOLA SALVINI^1^, STEFANIA BISOGNO^1^, MAURIZIO ACAMPA^1^, MOHAMED BOUTJDIR^2^, PIER LEOPOLDO CAPECCHI^1^

#### ^1^University of Siena [project ECS00000017 Tuscany‐Health Ecosystem—CUP B63C2200068007 spoke 6 Mission 4 Component 2 (M4C2)—investment 1.5 of the National Recovery and Resilience Plan (PNRR) funded by the European Union “Next Generation EU”], Siena, Italy,^2^VA New York Harbor Healthcare System, SUNY Downstate Health Sciences University, New York, NY

**Introduction:** Severely ill patients with coronavirus disease‐2019(COVID‐19) show an increased risk of new‐onset atrioventricular blocks(AVB), associated with high rates of short‐term mortality. Recent data suggest that the uncontrolled inflammatory activation observed in these patients, specifically interleukin(IL)‐6 elevation, may play an important pathogenic role by directly affecting cardiac electrophysiology. Aim of our study was to assess the acute impact of IL‐6 changes on electrocardiographic indices of atrioventricular conduction in severe COVID‐19.

**Methods:** We investigated: (1) the behavior of PR‐interval and PR‐segment in patients with severe COVID‐19 during active phase and recovery, and (2) their association with circulating IL‐6 levels over time.

**Results:** During active disease, COVID‐19 patients showed a significant increase of PR‐interval and PR‐segment. Such atrioventricular delay was transient as these parameters rapidly normalized during recovery. PR‐indices significantly correlated with circulating IL‐6 levels over time. All these changes and correlations persisted also in the absence of laboratory signs of cardiac strain/injury or concomitant treatment with PR‐prolonging drugs, repurposed or not.

**Conclusions:** Our study provides evidence that in patients with severe COVID‐19 and high‐grade systemic inflammation, IL‐6 elevation is associated with a significant delay of atrioventricular conduction, independent of concomitant confounding factors. While transient, such alterations may enhance the risk of severe AVB and associated short‐term mortality.

## R WAVE IN LEAD AVR TO LOCALIZE REGION OF ABNORMAL ELECTROANATOMIC SUBSTRATE IN ARRHYTHMOGENIC RIGHT VENTRICULAR CARDIOMYOPATHY

### **CHIAO CHIN LEE** ^1,2^, CHIN‐YU LIN^2^, YENN‐JIANG LIN^2^, SHIH‐LIN CHANG^2^, LI‐WEI LO^2^, YU‐FENG HU^2^, FA‐PO CHUNG^2^, TING‐YUNG CHANG^2^, LING KUO^2^, CHENG‐I WU^2^, CHIH‐MIN LIU^2^, SHIN‐HUEI LIU^2^, YU‐SHAN HUANG^2^, MUHAMMAD RAFDI AMADIS^2,3^, BAI SITTI AMEERAH ASLEAH B TAGO^2,4^, MARIE KIRK PATRICH MARAMARA^2,5^, WEN‐PO FAN^2,6^, LO‐CHIEH LING^2^, SHIH‐ANN CHEN^7,8^

#### ^1^Tri‐Servie General Hospital, Taipei, Taiwan,^2^Heart Rhythm Center, Division of Cardiology, Department of Medicine, Taipei Veterans General Hospital, Taipei, Taiwan,^3^Dr. Soetomo General Hospital, Surabaya City, Indonesia,^4^Amaipakpak Medical Center, Marawi City, Philippines,^5^University of the Philippines, Philippine General Hospital, Manila City, Philippines,^6^Division of Pediatric Cardiology, Department of Pediatrics, Taipei Veterans General Hospital, Taipei, Taiwan,^7^Cardiovascular Center, Taichung Veterans General Hospital, Taichung, Taiwan,^8^National Chung Hsing University, Taichung, Taiwan

**Introduction:** Arrhythmogenic right ventricular cardiomyopathy (ARVC) primarily impacts the right ventricular (RV) free wall, affecting both the outflow and inflow tracts. While the aVR morphology in electrocardiogram (ECG) has been linked to RV outflow tract (RVOT) substrate in Brugada syndrome, its association with ARVC remains unexplored. Our study aims to evaluate the association between the ECG morphology of lead aVR and epicardial‐endocardial RVOT scar in patients with ARVC.

**Methods:** A total of 45 subjects fulfilling the diagnosis of definite ARVC receiving epi‐endocardial mapping and ablation for drug‐refractory VT, were enrolled. The patients were divided into two groups according to the presence of RVOT scar [Group 1: RVOT scar (‐), N =10; Group 2: RVOT scar (+), N =35]. The significance of lead aVR in the ECG, baseline parameter, electrophysiological characteristics, and clinical outcomes were analyzed.

**Results:** The baseline characteristics are demonstrated in Figure 1A. In lead aVR, Group 1 presents with QR pattern, and Group 2 exhibited a heterogeneous morphology. Using a receiver operating characteristic curve, we found an R/Q ratio ≥0.545 in lead aVR was a good discriminator of patients without RVOT scar (Figure 1B, 1C). In Group 2, aVR morphology showed a significant correlation with the size of the epicardial scar, right ventricular ejection fraction, clinical VT eliminate site, and clinical/inducible VF. Figure 1D was the algorithm to evaluate the distribution of diseased RV substrate and clinical outcomes with ECG lead aVR.

**Conclusions:** In the ARVC population, the ECG morphology of lead aVR was associated with epicardial‐endocardial RVOT substrate abnormalities.

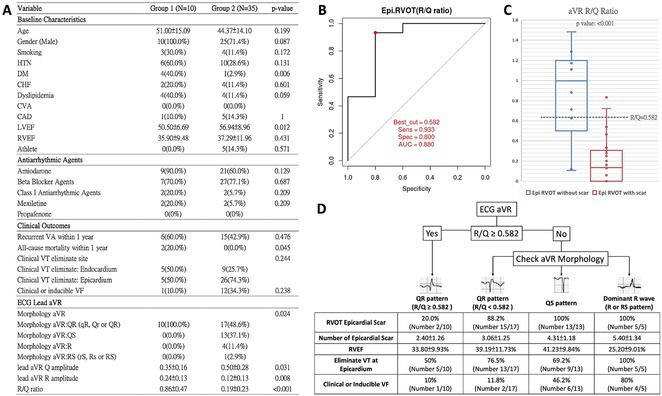

## ALL‐AROUND VERY HIGH‐POWER SHORT‐DURATION ABLATION IMPROVE THE PERFORMANCE OF ABLATION FOR ATRIAL FIBRILLATION

### **EUIJAE LEE**, WONSEOK CHOE, SANG WEON PARK

#### Bucheon Sejong Hospital, Bucheon‐si, Korea, Republic of

**Introduction:** Radiofrequency catheter ablation (RFCA) is the cornerstone in treating atrial fibrillation (AF), with its efficacy and safety continually improving due to technological advancements. The objective of this study is to determine whether very high‐power short‐duration (vHPSD) ablation differs from conventional ablation in terms of procedural efficiency and effectiveness.

**Methods:** Patients who underwent de novo AF RFCA before and after the introduction of vHPSD ablation were retrospectively analyzed. The Thermocool SmartTouch SF catheter (Biosense Webster, USA) was used for conventional ablation (SmartTouch group), and the QDOT Micro catheter (Biosense Webster, USA) was used for vHPSD ablation (QDOT group). All ablations were performed with 90 watts and 4 seconds in QDOT group. Baseline demographics and procedural characteristics, and recurrence rates were compared.

**Results:** A total of 109 patients were included (QDOT group 53, SmartTouch group 56). There were similar baseline characteristics between the groups. Despite more frequent linear ablations in the QDOT group, both total procedure time and total ablation time were significantly shorter (174.8 ± 47.4 vs. 226.8 ± 62.0 minutes, p<0.001; 17.1 ± 10.9 vs. 54.6 ± 14.8 minutes, p<0.001). One cardiac tamponade was reported in the QDOT group. The rate of freedom from AF was 67.0%, with no significant difference between the groups (69.8% in QDOT group vs. 64.3% in SmartTouch group, p=0.682).

**Conclusions:** The vHPSD ablation increases procedural efficiency and demonstrates comparable safety and clinical outcomes to conventional ablation.

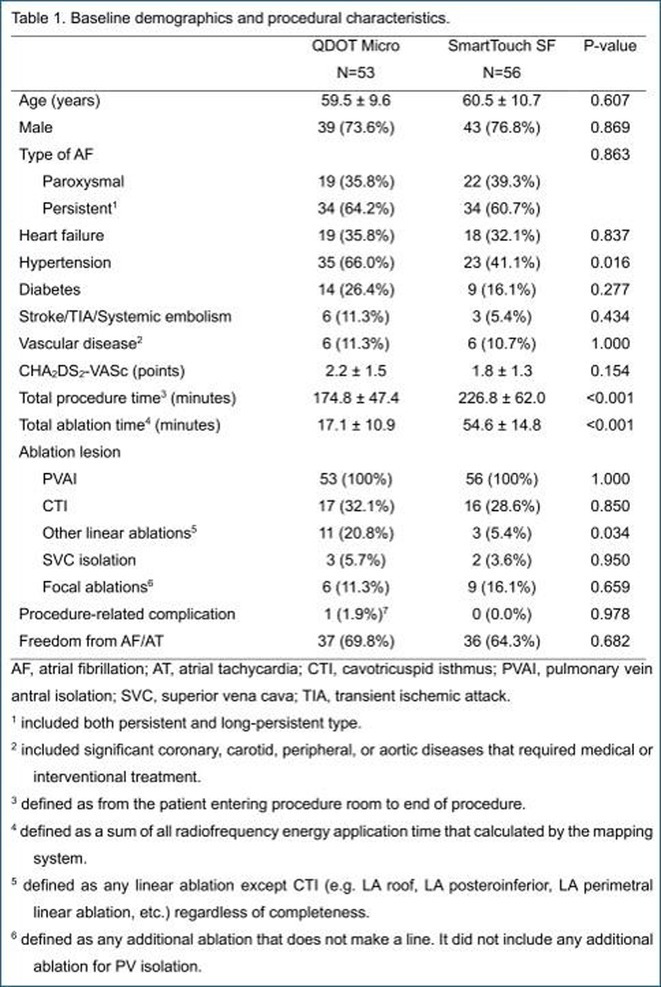

## IN‐HOSPITAL FEMORAL VASCULAR COMPLICATIONS ASSOCIATED WITH CATHETER ABLATION: AN EARLY POSTOPERATIVE DUPLEX ULTRASOUND INVESTIGATION

### **HYO JIN LEE** ^1,2^, SU HYUN LEE^1,2^, JUWON KIM^1,2^, JU YOUN KIM^1,2^, SEUNG‐JUNG PARK^1,2^, YOUNG KEUN ON^1,2^, KYOUNG‐MIN PARK^1,2^

#### ^1^Samsung Medical Center, Seoul, Korea, Republic of,^2^School of medicine, Sungkyunkwan university, Seoul, Korea, Republic of

**Introduction:** Usefulness of post‐catheter ablation duplex ultrasound and predictor for early (< 24 hours) femoral vascular complications is unclear. This study aimed to assess the incidence and the predictors of femoral vascular complications following catheter ablation using duplex ultrasound.

**Methods:** Between March 2023 and January 2024, 518 consecutive patients underwent catheter ablation for various cardiac arrhythmias. Duplex ultrasound evaluations were conducted at the femoral access site on the day following the procedure.

**Results:** Out of the 518 patients, 32 experienced femoral vascular complications (6.2%), including 20 cases of hematoma (3.9%), 5 pseudoaneurysms (1.0%), and 11 arteriovenous fistulas (AVF, 2.1%). Symptomatic pseudoaneurysms and AVFs were successfully treated with direct compression (2 patients [0.4%]) and coil embolization (2 patients [0.4%]). Procedures conducted within 6 month of venous puncture experience [Odds ratio (OR) 2.39; 95% confidence interval (CI) 1.02‐5.64; p‐value =0.05], body weight (OR 1.03; 95% CI 0.99‐1.05; p‐value =0.08), procedure on general anesthesia (OR 2.40; 95% CI 1.06‐5.45; p‐value =0.04) were associated with the femoral vascular complications.

**Conclusions:** Post‐procedural duplex ultrasound scans are valuable for the early detection of femoral vascular complications, enabling timely intervention and appropriate management strategies. Procedures conducted within 6 month of venous puncture experience and under general anesthesia were found to be associated with an increased risk of femoral vascular complications.

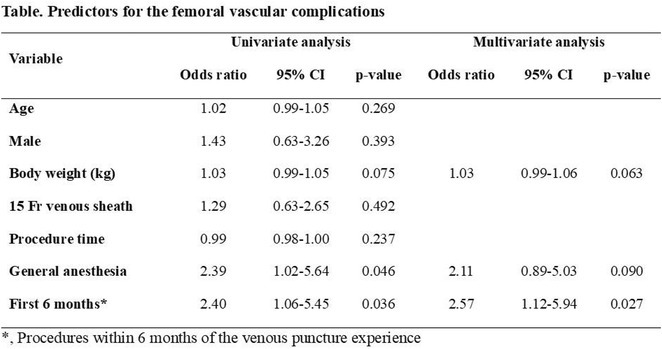

## TEMPORAL TRENDS OF THE CAUSE OF IN‐HOSPITAL CARDIAC ARREST: LIMITATIONS OF THE CURRENT ALERT SYSTEM

### **JOONG MIN LEE**, GI‐BYOUNG NAM, MIN SOO CHO, MYUNG‐JIN CHA, JUN KIM, KEE‐JOON CHOI

#### Asan Medical Center, Seoul, Korea, Republic of

**Introduction:** Cardiac arrest may occur in hospitals and causes serious medical consequences such as high mortality and residual neurologic sequelae. Etiology of in‐hospital cardiac arrest (IHCA) and its temporal trend has not been reported.

**Methods:** The medical emergency team (MET) was launched at Asan Medical Center in Seoul, Korea in March 2008. This prospective observational study was conducted based between March 2009 and December 2019. We included patients who received in‐hospital cardiopulmonary resuscitation (CPR). Patients who were ≤18 years of age, had a prior history of out‐of‐hospital cardiac arrest.

**Results:** A total of 2,344 episodes were included in this study. The mean age of IHCA patients was 62.9 years old (cardiac origin, 64.1 vs. non‐cardiac origin, 62.2 years old, p=0.002). The most common cause of IHCA was cardiac arrhythmia (331/2344, 14.1%), followed by respiratory failure (13.3%), sepsis (9.8%), airway obstruction (9.2%) and hypovolemic shock (8.5%). Acute coronary syndrome accounted for 4.3% of IHCA cases. The incidence of IHCA caused by respiratory cause decreased from 45.2% (52/115) to 9.7% (18/186) during the study period. In contrast, IHCA due to arrhythmic cause increased significantly from 8.7% (10/115) to 14.0% (26/186). The activation of the MET system prior to the IHCA event was 2.2% (51/2344). MET activation prior to IHCA was 8 cases, making up 0.9% of the cardiac cause group. In the non‐cardiac cause group, MET pre‐activation before IHCA was also rare, 43 cases (4.0%) of the group. Over the study period, MET pre‐activation due to respiratory causes was 47.2%, while cardiac causes accounted for 3.8% of the total activations.

**Conclusions:** This study elucidates a meaningful shift in the trends of IHCA over the past decade. Arrhythmia has surfaced as the principal cause of IHCA, concurrently with a significant decrease in the incidence of respiratory‐related IHCA cases. It was observed that the current alert system faces challenges in proactively screening for arrhythmias. Therefore, early prediction of cardiac arrhythmia is necessary.

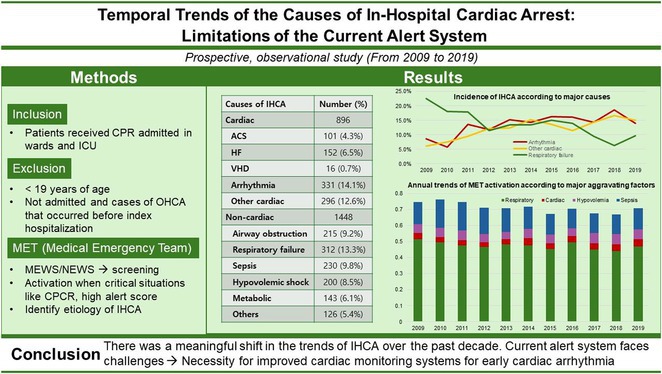

## PROLONG CARDIOPULMONARY BYPASS TIME AND HIGH LEFT ATRIAL VOLUME INDEX AS NOVEL RISK FACTORS OF ATRIAL FIBRILLATION IN THE LONG TERM AFTER TYPE A AORTIC DISSECTION SURGERY

### **KUN‐FENG LEE**, YEN‐NIEN LIN, JAN‐YOW CHEN, YOU‐CIAN LIN, WEI‐HSIN CHUNG

#### China Medical University Hospital, Taichung City, Taiwan

**Introduction:** New onset of atrial fibrillation (AF) after discharge from type A aortic dissection surgery (AD) is associated with subsequent morbidity and mortality. However, the incidence and risk factors remain unknown.

**Methods:** Patients who underwent type A AD surgery from 2015 to 2020 at a tertiary medical center in Taiwan were retrospectively analyzed with a case‐control design. Patients’ characteristics, surgical details, and outcomes were collected from electrical medical records.

**Results:** A total of 312 patients were included, within which 20 developed AF after the discharge, with an incidence of 12.7 per 1000 person‐year, and were defined as Group A. 20 patients without AF during follow‐up were defined as Group B. The baseline characteristics including age, sex, smoking, and CHA_2_DS_2_‐VASc score were comparable. Patients with AF in long term demonstrated a higher left atrial volume index (LAVI) (Group A vs. Group B, 36.44±14.1 vs. 25.74±8.5 mL/m^2^; *p*=0.006) at baseline. Surgically, these patients were associated with longer cardiopulmonary bypass time (Group A vs. Group B, 202, 95% CI, 177~313 vs.177, 95% CI, 156~199 minutes, *p*=0.055). After discharge from AD surgery to the time of detected AF, the time was 1,237.30±889.2 (IQR=964) days. During the follow‐up, patients with newly diagnosed AF were associated with a higher prevalence of heart failure with preserved ejection fraction (Group A vs. Group B, 35% vs. 5%, *p*=0.010), higher grade of diastolic dysfunction (Group A vs. Group B, 1, 95% CI 0.17~2.00 vs. 0, 95% CI 0.00~0.83, p=0.018), and higher LAVI (Group A vs. Group B, 46.37±15.2 vs. 29.21±12.3 mL/m^2^, *p*<0.001).

**Conclusions:** Type A AD surgery predisposed a risk factor for subsequent AF. Higher baseline LAVI and longer cardiopulmonary bypass time are associated with future AF independent of the CHA_2_DS_2_‐VASc score.

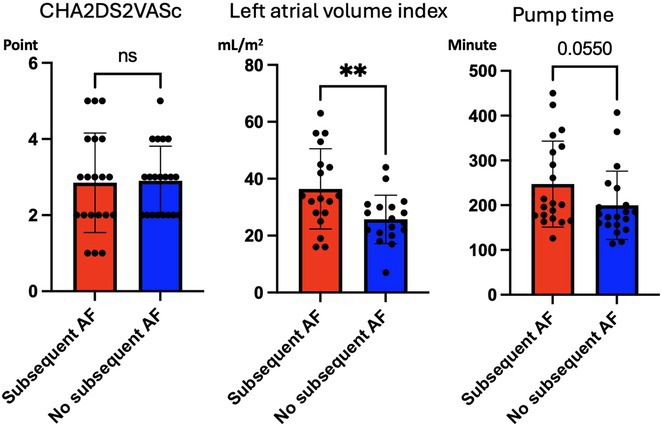

**OBESITY PARADOX IN ATRIAL FIBRILLATION PATIENTS ON ORAL ANTICOAGULATION THERAPY: A KOREAN NATIONWIDE COHORT STUDY**

**KWANG‐NO LEE** ^1^, DO‐YOUNG KIM^1^, SEUNG‐YOUNG ROH^2^, KYO‐SEUNG HWANG^1^

^1^Ajou University Medical School, Suwon, Korea, Republic of,^2^Korea Univerisity, Seoul, Korea, Republic of

**Introduction:** The obesity paradox is a phenomenon that has been observed in specific pathological conditions, wherein individuals who are overweight or obese exhibit a survival advantage over those with a normal weight. This phenomenon has also been noted in patients diagnosed with atrial fibrillation (AF), although the impact of body mass index (BMI) on clinical outcomes in AF patients undergoing oral anticoagulant (OAC) therapy remains uncertain. The aim of this study was to investigate the potential relationship between obesity and cardiovascular outcomes in patients diagnosed with AF who were receiving OAC.

**Methods:** We utilized administrative claims data from the Korean National Health Insurance Service and the combined health check‐up database of the National Health Insurance Corporation to conduct a retrospective national cohort study spanning from 2013 to 2020. The patients newly prescribed OAC for AF were included, between January 2015 and December 2019. We followed up with patients from their index date until each outcome event or the end of the study period. BMI was categorized into 5 groups based on the World Health Organization's recommended criteria for Asian populations. The predefined clinical outcomes included thromboembolism, major bleeding, all‐cause death, and the composite outcome.

**Results:** A total of 35,333 patients were included in the study. The mean age was 69 ± 10 years, and 37.1% were female. During a median follow‐up of 2.0 years (IQR, 0.4‐3.3), there were 11,634 thromboembolic events, 1,521 major bleeding events, and 2,586 deaths. The adjusted hazard ratio (HR) for thromboembolism, major bleeding, all‐cause death, and a composite outcome were lower in obese II group (HR 0.79, 95% CI 0.73‐0.86; HR 0.99, 95% CI 0.79‐1.24; HR 0.75, 95% CI 0.62‐0.91; HR 0.81, 95% CI 0.75‐0.88) compared to the normal range BMI group.

**Conclusions:** In AF patients undergoing OAC therapy, individuals classified as obese exhibited a reduced risk of thromboembolism, major bleeding, all‐cause mortality, and composite outcome compared to patients with normal weight.

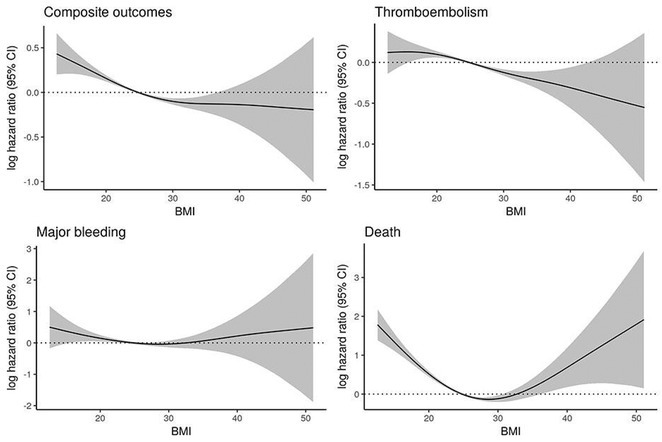

## SHANK3 OVEREXPRESSION LEADS TO CARDIAC DYSFUNCTION IN MICE BY DISRUPTING CALCIUM HOMEOSTASIS IN CARDIOMYOCYTES

### **MINJU LEE** ^1,2^, TAE HEE KO^1^, YOONHEE KIM^3^, CHUNMEI JIN^3^, BYEONGIL YU^1,2^, PHUONG KIM LUONG^4^, TRAN NGUYET TRINH^4^, YEJI YANG^5^, HYOJIN KANG^6^, YINHUA ZHANG^3^, RUIYING MA^3,2^, KWANGMIN YOO^7,2^, JUNGMIN CHOI^7,2^, JIN YOUNG KIM^5^, SUN‐HEE WOO^4^, KIHOON HAN^3,2^, JONG‐IL CHOI^1,2^

#### ^1^Division of Cardiology, Department of Internal Medicine, Korea University College of Medicine and Korea University Anam Hospital, Seoul, Korea, Republic of,^2^BK21 Graduate Program, Department of Biomedical Sciences, Korea University College of Medicine, Seoul, Korea, Republic of,^3^Department of Neuroscience, Korea University College of Medicine, Seoul, Korea, Republic of,^4^Laboratory of Pathophysiology, Chungnam National University College of Pharmacy, Daejeon, Korea, Republic of,^5^Digital Omics Research Center, Korea Basic Science Institute (KBSI), Ochang, Korea, Republic of,^6^Division of National Supercomputing, Korea Institute of Science and Technology Information (KISTI), Daejeon, Korea, Republic of,^7^Department of Biomedical Informatics, Korea University College of Medicine, Seoul, Korea, Republic of

**Introduction:** Shank3 proteins play crucial roles as neuronal postsynaptic scaffolds. Alongside neuropsychiatric symptoms, individuals with SHANK3 mutations often exhibit symptoms related to dysfunctions in other organs, including the heart. However, detailed insights into the cardiac functions of Shank3 remain limited. This study aimed to understand cardiac dysfunction with Shank3 mutations.

**Methods:** Alongside Cardiac histological analysis, electrocardiogram and echocardiogram recordings were conducted on Shank3‐overexpressing transgenic mice (Shank3 TG mice). Electrophysiological properties, including action potentials and L‐type Ca^2+^ channel (LTCC) currents, were measured in isolated cardiomyocytes. Calcium homeostasis was assessed by analyzing cytosolic Ca^2+^ transients and sarcoplasmic reticulum Ca^2+^ contents. Depolarization‐induced cell shortening was examined in cardiomyocytes. Immunoprecipitation followed by mass spectrometry‐based identification was employed to identify proteins in the cardiac Shank3 interactome. Western blot and immunocytochemical analyses were conducted to identify changes in protein expression in Shank3‐overexpressing transgenic cardiomyocytes.

**Results:** The hearts of Shank3 TG mice displayed reduced weight and increased fibrosis. In vivo, sudden cardiac death, arrhythmia, and contractility impairments were identified. Shank3‐overexpressing transgenic cardiomyocytes showed prolonged action potential duration and increased LTCC current density. Cytosolic Ca^2+^ transients were increased with prolonged decay time, while sarcoplasmic reticulum Ca^2+^ contents remained normal. Cell shortening was augmented in Shank3‐overexpressing transgenic cardiomyocytes. The cardiac Shank3 interactome comprised 78 proteins with various functions. Troponin I levels were down‐regulated in Shank3‐overexpressing transgenic cardiomyocytes.

**Conclusions:** This study revealed cardiac dysfunction in Shank3 TG mice, potentially attributed to changes in calcium homeostasis and contraction, with a notable reduction in Troponin I.

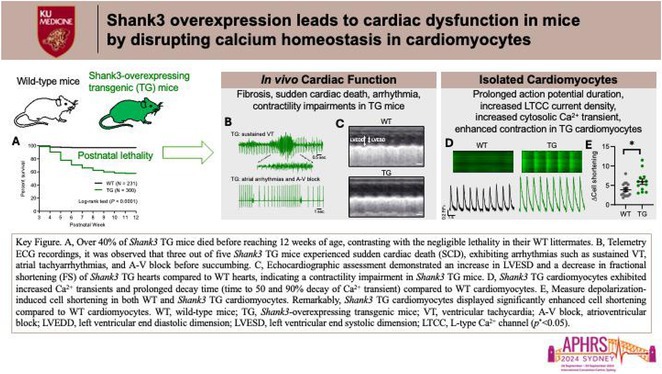

## AFTER THE IMPACT: PRESENTATIONS, DIAGNOSTIC INVESTIGATIONS, POST RESUSCITATION CARE AND COMPLICATIONS OF COMMOTIO CORDIS

### **RAFAEL LEE** ^1^, HAN LIM^2^

#### ^1^The Northern Hospital, Melbourne, Australia,^2^The Austin Hospital, Melbourne, Australia

**Introduction:** Commotio cordis survival rates have increased, owing to greater public awareness, earlier response, and defibrillation access. We explored the clinical profile, investigations, post‐resuscitation care, and long‐term complications of commotio cordis survivors.

**Methods:** PubMed and Embase were searched for all cases of commotio cordis from inception to November 11, 2023.

**Results:** Of 339 cases, the survival rate was 27%. Victims were typically young (mean 20±10 years) males (94%) with no prior cardiac history (93%). VF (85%) was the most common initial arrhythmia. Post‐resuscitation ECGs showed ischemic changes (42%), bundle branch blocks (13%), and prolonged QT (8%). Troponin (85%) and creatine kinase (86%) were elevated in most cases. Two cases reported hypokalemia with QT prolongation. TTE was the most reported imaging modality (29 cases), which was mostly normal (73%). Reduced systolic function was the most common TTE abnormality (24%) and most abnormalities resolved on follow‐up (83%). X‐ray (13 cases), angiogram (7 cases), cardiac MRI (6 cases), CT chest (6 cases) and stress testing (5 cases), were mostly normal. Post arrest, some cases required intubation (35%), antiarrhythmics (10%), and ICDs (13%). Long‐term complications were rare but included further arrhythmias (13%), hypoxic encephalopathy (10%) and persistent reduced systolic function (6%). Mean length of hospital stay was 10±12 days. Higher survival rates were observed with prompt resuscitation <3 minutes (48% vs 13%, p<0.001), on‐site AED use (79% vs 28%, p<0.001), and sport‐related settings (34% vs 14%, p<0.001). On‐site AED use had lower rates of intubation compared to later defibrillation by emergency medical teams or in hospital (25% vs 67%, p=0.04).

**Conclusions:** Workup of suspected commotio cordis involved comprehensive clinical assessment, biochemical testing, and imaging to assess for structural damage, underlying heart disease or predisposing factors. Inpatien management was mainly supportive, and most admissions were brief. Prompt resuscitation, on‐site AED use and sport‐related settings were associated with better survival.

## ADVANCED SURGICAL MAPPING AND PULSED‐FIELD ABLATION: VOLTAGE‐INDEPENDENT DEPTH CONTROL

### **SEUNGYUP LEE**, DANIEL LAURITA, CELEEN KHRESTIAN, DRAGAN JUZBASICH

#### Case Western Reserve University, Cleveland, OH

**Introduction:** Pulsed Field Ablation (PFA) is a novel ablation technology that utilizes electric field strength to ablate tissue. With the ability to electrically isolate each electrode, it is possible to dynamically map and ablate according to patient‐specific “mechanistic” targets. The objective of the study was to develop a surgical mapping/PFA system and to investigate the effects of applying the same pulse parameters to different electrode configurations.

**Methods:** PFA was delivered with a pulse generator (BTX ECM 830). Application was limited to 500V delivered due to electrical constraints of the mapping arrays. Prior to the study, we modeled the electric field distribution for 2 electrode configurations (COMSOL Metaphysics). In five swine open chest mapping/PAF, Group 1 (n=1) tested the ablating capabilities of the PFA system as well as comparing PFA lesions ablating from electrode configurations A and B (Fig. 1) on the left atrial appendage (LAA), repeating the exact same configuration B on the left ventricle (LV). PFA was delivered with constant pulse parameters per 3 applications (500V, 250μs pulse width, 500ms delay, 20 pulses), and PFA applications were delivered sequentially. Group 2 (n=4), 3 PFA applications (100ms pulse width, 1s delay, 10 pulses) in the left atrium (LA). Sinus electrograms before and after PFA sites were recorded in each group. Triphenyl tetrazolium chloride (TTC) was used to compare PFA depth postmortem.

**Results:** Group 1 configurations A and B (Fig. 1) were compared through gross examination. PFA depth varied between configurations (A: 1±0.3 mm vs. B: 2±0.3mm). In Group 2, PFA applications demonstrated a significant decrease in capture threshold at 300 ms drive train, (non‐ablated: 0.3±0.1mA vs. ablated: 4.2±0.7mA, p<0.01) and a significant decrease in electrogram amplitude (pre‐PFA: 4±2.7mV vs. post‐PFA: 1.1±0.7mV, p<0.01).

**Conclusions:** Surgical mapping/PFA was successfully performed using a custom high‐density mapping array. By using specific electrode configurations, PFA lesion depth can be controlled and applied to any tissue regardless of its thickness and diseased state, including the atrium and the ventricles.

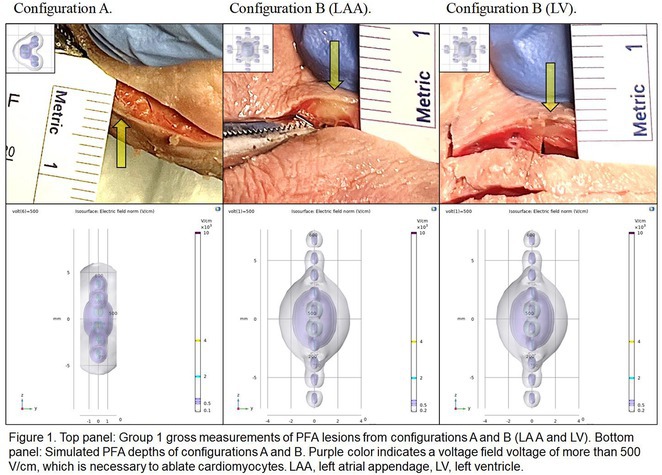

## OUTCOMES AFTER CAVOTRICUSPID ISTHMUS ABLATION AND TACHYCARDIOMYOPATHY IN TAURANGA, NEW ZEALAND

### **SIMON LEE**, PAULA BISHOP, ANIL JAYENDRAPPA, DEAN BODDINGTON

#### Tauranga Hospital, Tauranga, New Zealand

**Introduction:** Atrial flutter, along with atrial fibrillation is a common cause of arrythmia cardiomyopathy with reduced left ventricular (LV) systolic function. International guidelines now have cavotricuspid isthmus (CTI) dependent typical atrial flutter ablation as a class I indication and medical therapy as a class II indication. Patients with heart failure potentially stand to gain the most from atrial flutter ablation.

**Methods:** We aimed to assess the outcomes of patients with CTI dependent flutter with features of cardiomyopathy who underwent CTI ablation in the recently established ablation service in the Bay of Plenty District, New Zealand. All cases of CTI ablation between the 7^th^ of April 2022 up until the 12^th^ of May 2024 were included.

**Results:** In total during the two‐year period studied, there were 81 CTI dependent flutter ablation procedures performed. Bidirectional block was achieved in 75 (93%) of procedures. The average procedure time was 57.9 minutes and average fluoroscopy time was 13.4 minutes. There were zero cases of peri‐procedural complications.

56 of the patients had clinical heart failure. Of the patients who have had follow up with successive echocardiograms pre and post ablation, 68% of patients with a reduced ejection fraction had a return to normal LV function post ablation. A further 19% had improvement in their LV function post ablation. The remaining 13% of patients with impaired LV function did not have improvement in LV function post ablation. This group of patients had other co‐morbidities to account for their LV impairment, which included ischemic heart disease, infiltrative cardiomyopathy, left bundle branch block and one patient with multiple co‐morbidities.

**Conclusions:** The vast majority of patients with heart failure had improvement in LV function and 68% of patients had recovery of LV function back to normal. CTI dependent atrial flutter ablation is therefore an essential tool for the treatment of atrial flutter, and those with heart failure probably gain the most benefit. All patients with heart failure and typical atrial flutter should be considered for ablation.

## A CASE OF THE FLIRTATIOUS RIGHT BUNDLE BRANCH

### **WAI KIT LEE**, ERIC TIEN SIANG LIM, KAH LENG HO, CHI KEONG CHING, HOOI KHEE TEO

#### National Heart Centre, Singapore, Singapore

**Introduction:** Right bundle branch block (RBBB) is an ECG finding reflecting an alteration in the His‐Purkinje conduction system. It can be found in healthy individuals but may also be associated with structural, infiltrative diseases or conditions that stretch the right ventricle. Intermittent RBBB can occur during exercise and is often related to changes in the heart rate. We present a case of a young man who exhibited alternating RBBB during exercise testing.

**Methods:** N/A

**Results:** A 31‐year‐old Chinese male, BMI 27.4, with a history of asthma, Gitelman's syndrome and non‐alcoholic fatty liver disease, presented with recurrent chest pain. There was no family history of cardiac disease or sudden cardiac death. He first presented to another hospital in 2018 and a 24‐hour Holter and echocardiogram performed then, did not reveal significant abnormalities. He re‐presented end 2023 with chest pain and underwent an exercise stress echocardiography (ESE). During ESE, a pretest supine ECG showed sinus rhythm (SR) with left anterior hemiblock (LAHB). This transited into RBBB with no change in the PR or RR interval. A premature ventricular ectopic temporarily resulted in narrow QRS for the next beat but subsequent beats reverted to RBBB pattern. At Stage 1 Bruce, heart rate of 114 beats per minute, an alternating SR with RBBB at the same cycle length was noted. The patient subsequently went into complete RBBB from Stage 2. This persisted until the end of the test. The post stress echocardiography showed normal left ventricular ejection fracture and no new regional wall motion abnormalities. A cardiac computed tomography performed showed no significant atherosclerotic disease.

**Conclusions:** The occurrence of alternating RBBB during exercise is rare and more frequently reported in the elderly. Possible mechanisms have been postulated to be due to bidirectional block in the right bundle resulting in time for the bundle to recover at the next beat. Other possible explanations could be supranormal conduction on alternate beats. In this case, the LAHB may have a role to play in the difference in the antegrade and retrograde conduction, possibly contributing to the alternating SR and RBBB during exercise.

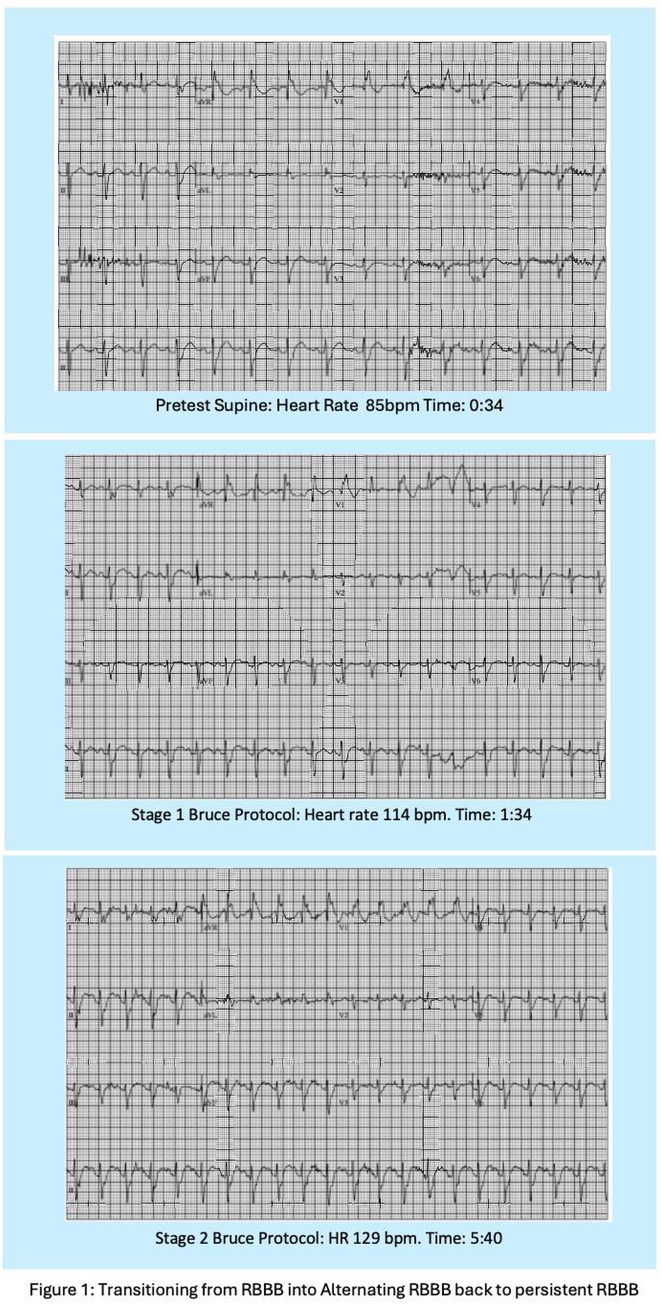

## HEART FAILURE (HF): CAN WE ACCURATELY PREDICT ACUTE DECOMPENSATION REMOTELY?

### **WAI KIT LEE**, GERMAINE JIE MIN LOO, ERIC TIEN SIANG LIM, KAH LENG HO, CHI KEONG CHING, HOOI KHEE TEO

#### National Heart Centre, Singapore, Singapore

**Introduction:** Acute HF decompensation is a major contributor to recurrent hospital attendance. There is a role for remote home monitoring in the prediction of clinical decompensation via cardiac implantable electronic devices (CIEDs). This study describes real world experience in a single tertiary centre in Singapore evaluating the feasibility of home monitoring via CIEDs with various parameters to predict HF decompensations.

**Methods:** A retrospective analysis was performed on a sample of patients who had implantable cardioverter defibrillator (ICDs) or cardiac resynchronization therapy (CRTs) implanted since 2019.

**Results:** 130 patients with a mean age of 67 years old were included. 44.6% of patients were on home monitoring whilst the rest opted for in‐office checks 6 monthly or earlier. Of our patients, 35 were admitted for heart failure decompensation. Reductions in thoracic impedance (TI) was not significantly associated with admissions, although 68.6% of patients showed a declining trend in the TI prior to admission (Figure 1). Haemoglobin (Hb) and NT‐proBNP levels were significantly linked to TI. There was no significant correlation between TI and age or creatinine levels.

**Conclusions:** Studies have not found TI a reliable sole indicator in predicting HF decompensation. This was also observed in our cohort, where heart failure admission was not associated with a significant reduction in TI, although most of our participants showed a declining trend in TI prior to admission. This suggests that an alert system based on the rate of the delta change in the TI may be more useful.

Recent studies have alluded to the potential of integrating various parameters into algorithms to predict HF decompensation. However, we did not find a significant correlation with individual parameters and clinical HF decompensation. This is likely due to the multi‐factorial nature of HF. We also found that Hb and NT‐proBNP were significantly associated with TI and these blood markers are unavailable in remote monitoring via CIEDs solely. More work is needed to truly understand how the different factors interact and contribute to clinical HF to better predict and hence reduce readmissions.

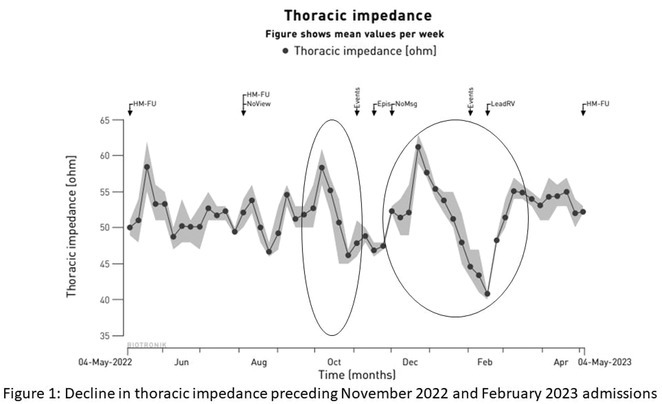

## CLINICAL OUTCOMES OF PULMONARY VEIN ISOLATION ENHANCED BY COMPLEX FRACTIONATED ATRIAL ELECTROGRAMS AND HIGH‐FREQUENCY MAPPING IN PERSISTENT ATRIAL FIBRILLATION

### **WEICHEIH LEE** ^1^, MIEN‐CHENG CHEN^2^

#### ^1^Chi Mei Medical Center, Tainan, Taiwan,^2^Kaohsiung Chang Gung Memorial Hospital, Kaohsiung, Taiwan

**Introduction:** The ablation strategy following pulmonary vein isolation (PVI) for persistent atrial fibrillation (AF) remains a subject of debate. In this approach, we utilized mapping of complex fractionated atrial electrograms (CFAE) and high‐frequency signals to identify critical sites of electrical activity. The ablation lines during PVI were fine‐tuned based on high‐frequency mapping data.

**Methods:** A total of 55 patients underwent their first ablation for AF at our hospital. Of these, 32 patients with paroxysmal AF were enrolled in group 1, and 23 patients with persistent or long‐standing persistent AF were enrolled in group 2. PVI was initially performed on all patients, followed by additional ablation if the rhythm failed to convert to sinus rhythm or if sustained atrial tachyarrhythmias could be induced. In group 2, CAFÉ and high‐frequency mapping and analysis was performed under AF and PVI was fine‐tuned based on the results of mapping.

**Results:** The average CHA2DS2‐VASc score was similar between the two groups. A larger LA volume (192.4 ± 73.7 mL vs. 113.4 ± 39.3 mL; p<0.001) was detected by computed tomography in group 2. There was also a greater need for linear ablation, including roof line (56.5% vs. 3.1%; p<0.001) and bottom line (47.8% vs. 3.1%; p<0.001), in group 2. A higher rate of early recurrence was observed in group 2 (34.8% vs. 12.5%; p=0.048), and there was a trend towards a higher incidence of late recurrence, though not statistically significant (21.7% vs. 6.3%; p=0.089). The discontinuation rates of anti‐arrhythmic agents were similar between the two groups (60.9% vs. 68.8%; p=0.547). In both univariate logistic regression analyses aimed at identifying predictors of late recurrence of AF, LA volume emerged as the sole significant predictor.

**Conclusions:** For persistent or long‐standing persistent AF, modifying PVI with CAFE and high‐frequency signals may lead to improved outcomes in terms of reduced recurrence rates. Only LA volume predicted the late recurrence of AF.

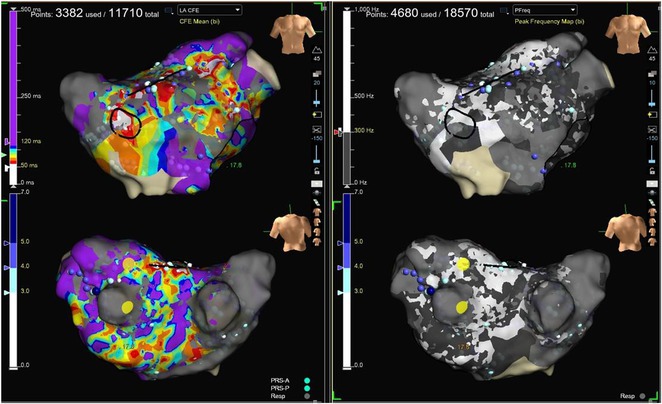

COMPARATIVE OUTCOMES OF STANDARD AND ENHANCED PULMONARY VEIN ISOLATION IN PERSISTENT ATRIAL FIBRILLATION: A STUDY ON THE INTEGRATION OF COMPLEX FRACTIONATED ATRIAL ELECTROGRAMS AND LOW VOLTAGE AREA MAPPING

**WEICHEIH LEE** ^1^, HUANG‐CHUNG CHEN^2^, MIEN‐CHENG CHEN^2^

^1^Chi Mei Medical Center, Tainan, Taiwan,^2^Kaohsiung Chang Gung Memorial Hospital, Kaohsiung, Taiwan

**Introduction:** The recurrence of atrial tachyarrhythmias (ATAs) remains a significant challenge following ablation, especially in cases of persistent or long‐standing persistent AF. In our approach, we integrated mapping of complex fractionated atrial electrograms (CFAE) and low‐voltage areas to enhance the effectiveness of pulmonary vein isolation (PVI). This study aims to evaluate the clinical outcomes of PVI supplemented by CFAE mapping and low‐voltage area targeting in patients with persistent atrial fibrillation (AF).

**Methods:** Between January 2021 and December 2023, a total of 64 patients underwent their first ablation for persistent or long‐standing persistent atrial fibrillation (AF) at two medical centers. Of these, 26 patients treated with the conventional method were placed in Group 1, while 38 patients who received PVI augmented with CFAE mapping and targeting of low‐voltage areas were assigned to Group 2.

**Results:** A larger left atrial volume was observed in Group 2 (180.9 ± 66.5 mL) compared to Group 1 (145.7 ± 38.3 mL; p=0.011), as detected by computed tomography. The need for linear ablation was comparable between the two groups, but Group 1 required significantly more additional ablation for CAFE (84.6% vs. 46.1%; P<0.001). A higher rate of early recurrence was observed in Group 1 compared to Group 2 (53.8% vs. 21.1%; p=0.009), along with a significantly higher incidence of late recurrence (57.7% vs. 21.1%; p=0.004). However, the rates of discontinuation of anti‐arrhythmic agents were similar between the two groups (23.1% vs. 26.3%; p=0.769). The recurrence rates of atrial fibrillation (AF) were similar between the two groups (34.6% for Group 1 vs. 18.4% for Group 2; p=0.145). However, the recurrence of atrial flutter (AFL) or atrial tachycardia (AT) was higher in Group 1 (23.1% vs. 2.6%; p=0.010).

**Conclusions:** For persistent or long‐standing persistent AF, enhancing PVI with CAFE and low‐voltage area targeting may lead to improved outcomes. This includes reduced recurrence of ATAs, particularly a decrease in recurrent AFL and AT.

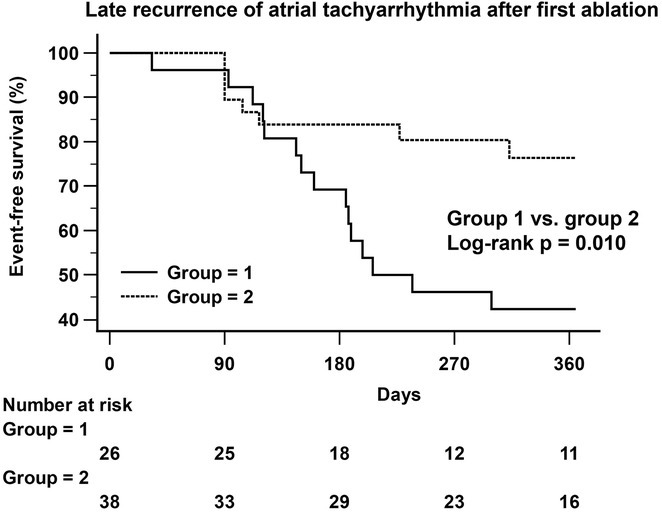

## SEX‐SPECIFIC OUTCOMES IN PULSED FIELD ABLATION OF ATRIAL FIBRILLATION

### **XIANG WEN LEE**, EHSAN MAHMOODI, RUSSELL DENMAN, HARIS HAQQANI

#### The Prince Charles Hospital, Brisbane, Australia

**Introduction:** Disparities in patient selection, procedural strategies and outcomes following ablation of atrial fibrillation (AF) between males and females have been variably demonstrated in previous studies. We present sex‐specific outcomes in a series of patients undergoing pulsed field ablation (PFA).

**Methods:** Consecutive patients undergoing PFA of AF at a single centre were prospectively included. PFA procedures were performed with the Farapulse system under general anaesthesia. Patients underwent routine clinical follow‐up. Data are presented as mean ± standard deviation or proportions. Kaplan‐Meier curves were constructed for freedom from atrial arrhythmias and compared with a log‐rank test.

**Results:** A total of 104 males (65%) and 55 females (35%) underwent PFA. There were no statistically significant differences in baseline characteristics including age (61.3±10.7 vs 62.4±9.3 years), body mass index (29.7±4.9 vs 31.3±7.0 kg/m^2^), paroxysmal AF (44.2% vs 41.8%), ejection fraction (55.2±10.4% vs 56.7±9.7%) and CHA_2_DS_2_‐VASc score (1.75±1.29 vs 2.09±1.57) in males vs females, respectively. The number of energy applications (40.8±12.2 vs 42.4±12.0), procedure times (77.5±18.4 vs 78.0±20.8 minutes) and proportion receiving adjunctive posterior wall isolation (26.9% vs 32.7%) were similar. Fluoroscopy dose‐area product was higher in males than females (506±505 vs 352±335 μGym^2^, p=0.035). Three vascular access complications occurred, of which one was in a male (1.0%) and two were in females (3.6%). There were no other major complications. Median follow‐up was 370 days. The Kaplan‐Meier estimate of freedom from recurrent atrial arrhythmias at 365 days was 80% (95% CI: 72‐88%) in males and 72% (95% CI: 56‐87%) in females (p=0.31; Figure 1).

**Conclusions:** In our series, males and females undergoing PFA of AF had similar procedural metrics and outcomes.

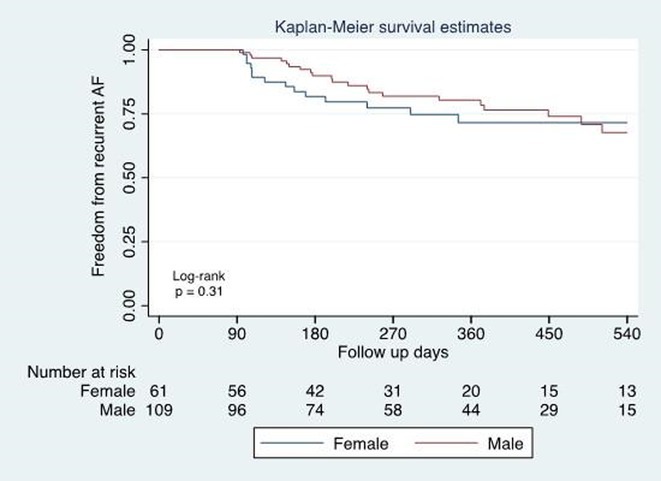

## LEADLESS PACEMAKER IMPLANTATION POSITION; AND INCIDENCE AND PROGNOSIS OF SUBCLINICAL RIGHT VENTRICULAR PERFORATION BY LEADLESS PACEMAKER; MULTICENTER RETROSPECTIVE COHORT STUDY

### **YOUNG SHIN LEE** ^1^, JAE‐SUN UHM^1^, JE‐WOOK PARK^2^, IN‐SOO KIM^3^, MOON‐HYUN KIM^2^, DAEHOON KIM^1^, HEE TAE YU^1^, TAE‐HOON KIM^1^, JONG‐YOUN KIM^3^, JIN‐BAE KIM^4^, BOYOUNG JOUNG^1^, HUI‐NAM PAK^1^, MOON‐HYOUNG LEE^1^

#### ^1^Severance Cardiovascular Hospital, Yonsei University College of Medicine, Seoul, Korea, Republic of,^2^Yongin Severance Hospital, Yonsei University College of Medicine, Yongin, Korea, Republic of,^3^Gangnam Severance Hospital, Yonsei University College of Medicine, Seoul, Korea, Republic of,^4^Kyung Hee University Hospital, Seoul, Korea, Republic of

**Introduction:** Leadless pacemakers are recommended to be implanted in the right ventricular (RV) mid‐septum to mitigate the risk of RV perforation. RV perforation cases associated with severe adverse outcomes have been reported, but reports on the exact location of device implantation are limited. Our aim was to elucidate the implantation position of the leadless pacemaker tines on computed tomography (CT) and assess the prognosis of patients accordingly.

**Methods:** Patients who underwent CT after leadless pacemaker implantation were included in the analysis. Two cardiologists and one radiologist reviewed the CT images to assess the position of tines and presence of perforation. The implantation position was categorized as septum, RV free wall, junction of septum and RV free wall, moderator band, and RV apex. Subclinical perforation was defined as the tine traversing beyond the outer myocardial contour without symptoms, hemodynamic instability, or pacemaker issues.

**Results:** A total of 62 patients (age, 72.8±12.2 years; 27 males) were included. Twenty‐nine patients (46.8%) had underlying atrial fibrillation, 24 (38.7%) of whom were taking non‐vitamin K antagonist oral anticoagulants. As the pacemaker positions, junction of septum and RV free wall was most common, observed in 48 patients (77.4%), while 3 of the patients (4.8%) having some of the tines implanted in the moderator band. RV perforation by the tines was identified in 11 patients (17.7%), with parameter results performed immediately after implantation showing significantly higher impedance compared with the no perforation group (1060.0±485.4 vs. 818.0±224.9 Ω, *P* value=0.020). None of the patients with perforation experienced an adverse event for 9.3months of follow‐up.

**Conclusions:** The most common leadless pacemaker implantation site is the septum‐free wall junction. Subclinical perforation rate is 17.7%.

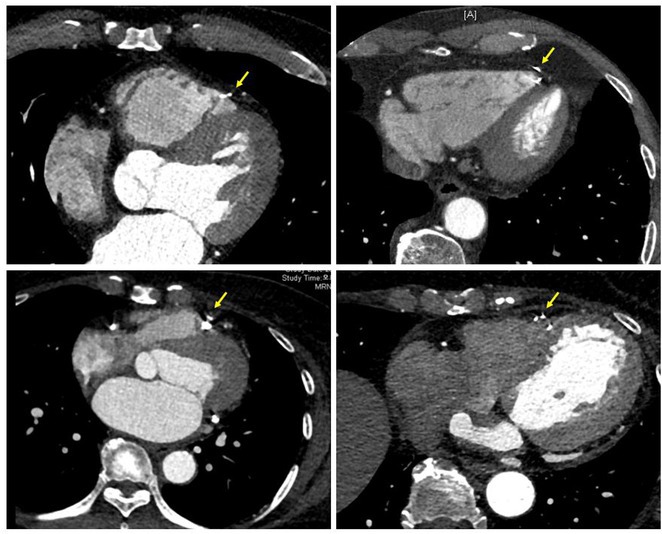

## A TALE OF ELECTRICAL HARMONY: CORONARY SINUS PACING IN INACCESSIBLE RIGHT VENTRICLE

### **LIM LEE YEE**, KHOO SONG WENG RYAN, FOO JHI HUI

#### Hospital Queen Elizabeth II, Kota Kinabalu, Malaysia

**Introduction:** Tricuspid valve surgery is commonly associated with atrioventricular conduction abnormality due to the close anatomical proximity of tricuspid valve and conduction tissue. Permanent pacemaker implantation for a complete atrioventricular block after a tricuspid valve surgery is not uncommon and it can be challenging due to the inaccessible right ventricle with a prosthetic tricuspid valve.

**Methods:** N/A

**Results:** A 22‐year‐old young woman with a permanent pacemaker for complete atrioventricular block was admitted due to epicardial lead failure with rapid depletion of battery life. She had a background history of congenital heart disease and underwent surgeries including a tricuspid valve replacement which is complicated with complete atrioventricular block requiring permanent pacemaker implantation. Unfortunately, she had two repeated surgeries for tricuspid valve replacement due to early degeneration of the bioprosthetic valve and the endocardial lead was replaced with epicardial lead during her last surgery. His Bundle pacing with AAI‐DDDR mode was planned but unsuccessful due to difficulty in screwed in and fixed the lead. Univentricular bifocal pacing via coronary sinus was performed and she was discharged well with a month follow‐up for pacemaker interrogation which showed good threshold and impedance level. Management of complete atrioventricular block in patients with prosthetic tricuspid valve can be challenging due to difficulty for the conventional right ventricular lead placement. Options available to avoid lead placement through prosthetic tricuspid valve include coronary sinus pacing, His bundle pacing, leadless pacemaker or surgical epicardial lead implantation. Univentricular bifocal pacing via coronary sinus for a better intraventricular synchronization can be considered in patients with inaccessible right ventricle if His bundle pacing is not feasible.

**Conclusions:** Permanent pacemaker via coronary sinus is an option in patients with inaccessible right ventricle.

## CARDIOVASCULAR IMPLANTABLE ELECTRONIC DEVICES INFECTIONS TREATED WITH CONTINUOUS IN SITU‐TARGETED ULTRAHIGH CONCENTRAION OF ANTIBIOTICS: A CASE SERIES OF SUCCESSFUL OUTCOMES AT A TERTIARY HEART CENTRE

### **LIM LEE YEE** ^1^, FOO JHI HUI^1^, LEE ZHAO MING^1^, CHIA LIUQING^1^, SHAHIDATUL AUNI BINTI SHAHRIMAN^1^, STEPHANIE CHANG YEN LI^1^, LIEW HOUNG BANG^1^, KOH KENG TAT^2^, ZOSIMO KEN L. JIMENO^3^, GIRI SHAN RAJAHRAM^4^, TAN SZE LING^5^, LIM WUN XIN^5^

#### ^1^Department of Cardiology, Hospital Queen Elizabeth II, Kota Kinabalu, Malaysia,^2^Department of Cardiology, Sarawak Heart Centre, Kuching, Malaysia,^3^Department of Plastic Surgery, Hospital Queen Elizabeth, Kota Kinabalu, Malaysia,^4^Department of Infectious Disease, Hospital Queen Elizabeth II, Kota Kinabalu, Malaysia,^5^Pharmacy Department, Hospital Queen Elizabeth II, Kota Kinabalu, Malaysia

**Introduction:** Cardiovascular implantable electronic devices (CIED) usage has been increasing in the recent years with indications such as pacemakers for bradyarrhythmia, implantable cardioverter defibrillator (ICD) for ventricular arrhythmias, and cardiac resynchronization therapy. CIED related infection can be a serious complication which is associated with high morbidity and mortality and guidelines recommended device and lead extraction in such cases. However, device and lead extraction is a complex procedure with potential complications of vessel injury, valve damage and cardiac perforation and continuous, in situ‐targeted, ultrahigh concentration of antibiotics (CITA) can be an alternative to the current practice.

**Methods:** N/A

**Results:** We report four cases of CIED infection treated with CITA. All of them are men, age between 32 to 90 years old, two of them with permanent pacemakers and another two with ICD. All patients received intravenous ceftriaxone and vancomycin empirically and converted to CITA once blood cultures confirmed no growth and they went through similar procedures with wound debridement, capsulectomy, wound washout, edges refashioning, submuscular pocket creation with triple lumen insertion, primary closure and incisional negative pressure wound therapy. Intrapocket high concentration of vancomycin and gentamycin were given for 14 days with dosage titration according to therapeutic drug monitoring and infusion rate of 1.2ml/hour with titration according to tolerability. However, one of the patient was unable to tolerate the pain and swelling over the infusion site and treatment was stopped after 10 days. All the four cases had good wound healing without major adverse events, and avoided device and lead extraction.

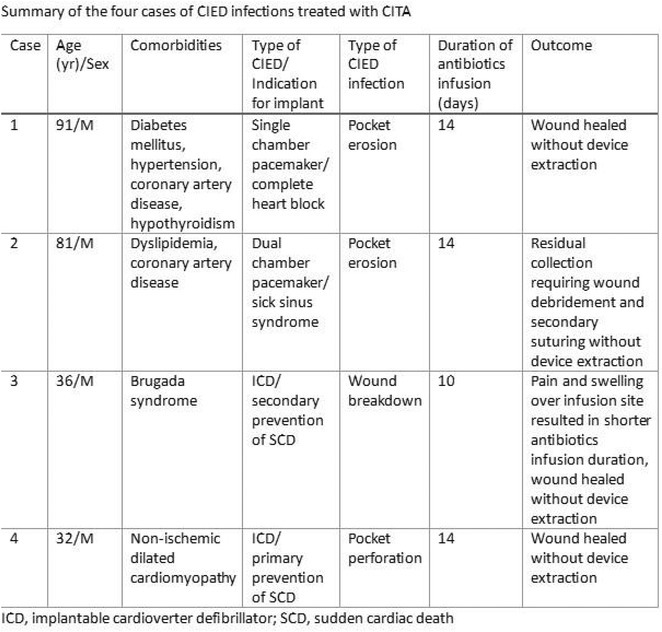

**Conclusions:** CITA is a promising alternative treatment for CIED infection as device and lead extraction carries significant risk of complications with high morbidity and mortality. More studies are needed to verify the safety and efficacy of CITA.

## REVIVING RHYTHMS: UNCONVENTIONAL CARDIONEURAL ABLATION REVERSES COMPLETE HEART BLOCK

### **LIM LEE YEE**, FOO JHI HUI, CHIA LIUQING, SHAHIDATUL AUNI BINTI SHAHRIMAN, ONG KOK HAN

#### Department of Cardiology, Hospital Queen Elizabeth II, Kota Kinabalu, Malaysia

**Introduction:** Cardioneural ablation (CNA) is a novel method used to decrease vagal innervation to the heart. It is a catheter‐based ablation technique which has been used in various conditions associated with high vagal tone such as vasovagal syncope and functional bradycardia. In selected group of patients, CNA has the potential in treating functional bradyarrhythmias which may prevent a permanent pacemaker implantation.

**Methods:** N/A

**Results:** A 50‐year‐old woman presented to district hospital with a 3‐month history of pre‐syncopal attack with frequency of 2‐3 times every day. There was no specific trigger and it lasted for a brief duration of 2 minutes. She had a background history of well controlled hypertension and dyslipidemia with losartan 50mg daily and atorvastatin 20mg daily. Upon presentation, she was alert with normal vital signs and physical examination was unremarkable. She had a normal blood result. Her electrocardiogram showed second degree atrioventricular block Morbitz II (figure 1A) and intravenous atropine challenge test was performed which showed good response with heart rate improved from 60 to 100 beats per minute. She fulfilled the selection criteria for CNA with post atropine heart rate increment of >20%. However, due to logistic issues, she turned up to our clinic 6 months later. She was asymptomatic but her heart rate was 40 beats per minute and her electrocardiogram showed a complete heart block (figure 1B). Electrophysiology study was performed which showed normal value for AH and HV interval. CNA was performed using Tolga Aksu method and achieved improvement in atrioventricular conduction to 2:1 block. 6 weeks later, she was reviewed in the clinic with a repeated electrocardiogram showed a normal sinus rhythm with heart rate of 70 beats per minute (figure 1C).

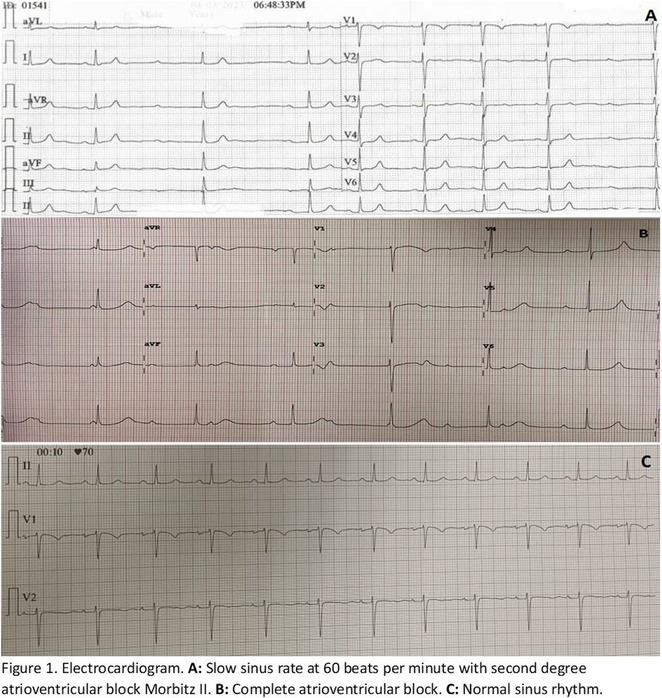

**Conclusions:** CNA has a potential in treatment for functional bradycardia to avoid a permanent pacemaker implantation. More studies are needed to define appropriate selection of patients, technique and comprehensive follow‐up and also to verify the safety and efficacy of CNA.

## TRIUMPH OVER CHALLENGES IN HYPERTROPHIC OBSTRUCTIVE CARDIOMYOPATHY WITH ALCOHOL SEPTAL ABLATION AND LEFT BUNDLE BRANCH PACING‐OPTIMIZED IMPLANTABLE CARDIOVERTER‐DEFIBRILLATOR

### **LIM LEE YEE**, LAW DE ZHI, NEERUSHA KAISBAIN, LO CHEW FUI, FOO JHI HUI

#### Hospital Queen Elizabeth II, Kota Kinabalu, Malaysia

**Introduction:** Alcohol septal ablation is one of the non‐surgical approach to hypertrophic obstructive cardiomyopathy and a complete heart block is a known complication of the procedure. Pacemaker implantation is indicated in cases of complete heart block and left bundle branch pacing is an emerging technique to achieve a physiological pacing with better interventricular synchronization. However, challenges arise when applying left bundle branch pacing in hypertrophic obstructive cardiomyopathy due to septal hypertrophy and we report a successful left bundle branch pacing‐optimized implantable cardioverter‐defibrillator implantation in a patient with hypertrophic obstructive cardiomyopathy.

**Methods:** N/A

**Results:** A 33 year old man was diagnosed with hypertrophic obstructive cardiomyopathy and underwent alcohol septal ablation in view of symptomatic and presence of high pressure gradient of left ventricular outflow tract. It was a successful procedure but complicated with complete heart block. In view of sudden cardiac risk of 2.3%, we decided for pacemaker implantation along with implantable cardioverter‐defibrillator. A more physiological conductive system pacing was chosen for this young man to reduce the risk of heart failure in the future. It was a challenging procedure as patient has thick interventricular septum, however, we were able to achieve a good left bundle branch area pacing signal at the mid‐septum of right ventricle after several attempts. Patient remained well and device interrogation during follow‐up showed good threshold and impedance.

**Conclusions:** Left bundle branch pacing‐optimized implantable cardioverter‐defibrillator offers a physiological approach for patients with hypertrophic obstructive cardiomyopathy with complete heart block, providing both pacing and defibrillation function. This case illustrates the potential of this approach as an alternative to conventional therapeutic approach, however, further research is needed to validate its safety and feasibility especially in hypertrophic cardiomyopathy.

## CARDIOVASCULAR IMPLANTABLE ELECTRONIC DEVICE (CIED) IMPLANTATION IN INFANTS AND CHILDREN: A SINGLE CENTER 17 YEAR EXPERIENCE AND FOLLOW UP

### **MANURA LEKAMWATTAGE** ^1^, SAMEERA T ELAPATHA^2^, ASUNGA N DUNUWILLE^2^, ROHAN GUNAWARDENA^2^

#### ^1^Lady Ridgeway Hospital for Children, COLOMBO, Sri Lanka,^2^The National Hospital, COLOMBO, Sri Lanka

**Introduction:** Pacemaker implants in infants and children constitutes about 1% of all pacemaker implants while other CIED implants are much rarer. Most common indication for pacing is either congenital complete heart block (CHB) or AV block following cardiac surgery. The small size of these patients, complex congenital abnormalities in some instances and the relatively large size of devices makes device implantation in this group difficult. Since most commonly available leads, are of a relatively large diameter more suited for adults, transvenous implants can be a challenge.

**Methods:** All patients who underwent CIED implants from the period 2006 to January 2024, at the Lady Ridgeway Hospital for Children in Colombo, Sri Lanka were recorded and followed up.

**Results:** 0ne hundred and sixty three children and infants underwent Pacemakers or other CIEDs during this period. The mean age of patients was 7.5 years (range 01 month to 15 years). The mean weight was 24.3Kg (range 2.3 to 55.5Kg). The indications are noted in table 1. Majority (73%), undergoing transvenous pacing with 27%, epicardial procedures. Most were implanted with single chamber VVIR pacemakers, with only 02 patients undergoing dual chamber pacing. Two patients had a Cardiac Resynchronization (CRT) device for severe Dilated Cardiomyopathy and 04 patients had Implantable Cardiac Defibrillators (ICDs) (2 for Hypertrophic Cardiomyopathy and 2 for Long QT syndrome). 06 patients had to have a lead revision or generator re‐positioning due to infection or lead displacement. 02 patients died. 28 patients had a single box change and 5 have had the second box change due to initial pacemaker reaching ERI during this period. p class="MsoNormal">Table 01: Indications for pacing

**Conclusions:** Pacing and other CIED implants in children, while being not as common as adults, is vital in certain situations. It is relatively safe and generally has a good long‐term outcome. CRTs and ICDs are much more challenging but feasible and safe when definitely indicated.

## LEFT VENTRICULAR SUMMIT PVC ABLATION‐ DAUNTING TASK BUT SATISFYING!

### LAI KUAN LEONG

#### Sarawak Heart Centre, Kota Samarahan, Malaysia

**Introduction:** Ablation within the left ventricular summit is challenging due to the proximity of the surrounding major cardiac structures that might pose a risk of complications and accessibility.

**Methods:** We reported a 59‐year‐old male with underlying heart failure reduced ejection fraction who had an implantable cardiac defibrillator for 1.5 primary prevention of sudden cardiac death. He complained of being easily lethargic and his electrocardiogram showed bigeminy PVC. 24‐hour Holter showed high burden PVC. Patient prepared in CARTO 3D mapping. Activation mapping of RVOT, LVOT, CS were performed using SmartTouch^TM^ irrigated ablation catheter 7.5Fr. Ablation catheter was negotiated to the deepest negotiable area in AIV, activation time preceded QRS onset by 44 ms with QS unipolar signal. Coronary angiogram showed the ablation site was away from the coronary artery. Ablated at 20‐30W with high flow irrigation, PVC suppressed but recurred upon stopping of ablation. On the second redo procedure, patient was prepared under Ensite 3D Precision. BMW coronary wire with Finecross microcatheter was inserted into AIV and unipolar mapping was done.

**Results:** Earliest site was found deeper in the AIV from the previous ablation site. AlFlux Blue tip 7Fr irrigated ablation catheter was able to negotiate to the AIV. Earliest site mapped and acceptable distance of 4 mm away from coronary artery, ‐35 ms early than PVC was selected for ablation. Coronary artery was flushed with normal. Ablation catheter was at maximum flow of 34 ml/min, and ablated at 10 W. PVC was silent within 2 seconds and ablation was continued for 60 seconds. Post ablation, coronary angiogram was repeated and there was no coronary artery stenosis or spasm.

**Conclusions:** Left ventricular summit PVC are less readily accessed due to the presence of thicker anteroseptal myocardium and epicardial fat. Without small size (2Fr to 2.3Fr) mapping catheter, coronary wire and microcatheter can be used for Unipolar mapping in AIV to better delineate the site of earliest activation. Smaller ablation catheter or Ethanol ablation may be attempted to this ‘inaccessible area’ in LV Summit PVC.

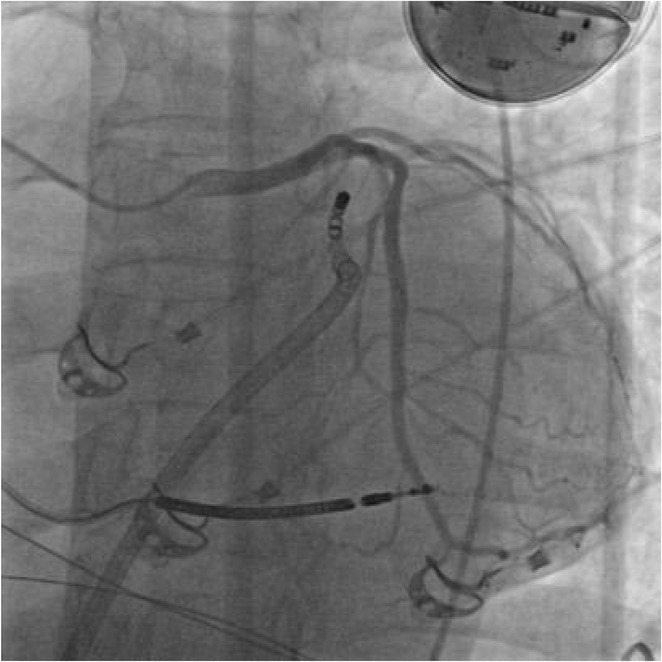

## COMPUTED TOMOGRAPHY‐VERIFIED PACING SITES OF LEADLESS MICRA™ PACEMAKERS IMPLANTED IN BRADYCARDIA PATIENTS

### JIANGHUA ZHANG^1^, BAOPENG TANG^1^, XIANHUI ZHOU^1^, YANMEI LU^1^, **YAODONG LI** ^1^, QIANG XING^1^, KELA ZU^1^, XU YANG^1^, SUOER JIA^1^, YANKAI GUO^1^, XIAOHONG ZHOU^2^, KOHNLE SAMANTHA^2^, ZILONG KANG^3^, GEORGIANA ZOU^3^

#### ^1^The First Affiliated Hospital of Xinjiang Medical University, Urumqi, China,^2^Medtronic Inc., Minneapolis, MN,^3^Medtronic (Shanghai). Co Ltd, Shanghai, China

**Introduction:** The leadless pacemakers are implanted routinely under fluoroscopic image, yet the location of the pacing sites remains unclear. This study was to determine the precise location of the leadless Micra™ pacemakers (Micra™) by usingcomputed tomography (CT) images collected post Micra™ implantation.

**Methods:** 20 consecutive patients who met the pacemaker indications for bradycardia and underwent Micra™ implantation were enrolled. The procedure for Micra™ deployment in the RV septal region was conducted under fluoroscopic image of RAO 30o±10 and confirmed under LAO 45o±10. All subjects underwent a postoperative CT scan to determine the precise location of the Micra™ pacing tip. The positioning of the Micra™ tip under the fluoroscopic RAO image was assessed using the method of the nine partitions (Figure 1A) and compared with the location identified in the CT image analysis. Paced 12‐lead ECG characteristics were analysed and correlated with the Micra™ tip location.

**Results:** In the nine partitions of fluoroscopic RAO images, 14 (70%) of 20 patients had the Micra™ tip in zone 5, 5 (25%) in zone 6 and 1 in zone 2. Reconstructed CT 3‐D cardiac images found the Micra™ tips mostly clustered near the anterior insertion between the RV septum and free wall (Figure 1B) with 12 cases in the septal side and 8 cases in the free wall side. Of the 14 cases in fluoroscopic zone 5, 7 were in the septal side and 7 in the free wall side while of the 5 cases in fluoroscopic zone 6, 4 were in the septal side and 1 in the free wall side. ECG morphological analysis found that the peak deviation index in ECG V1 lead was 0.402±0.061 for Micra™ tips in the septal side and 0.542±0.053 in the free wall side (P <0.001 between two sides), suggesting different ventricular activation propagation by pacing at different sites. Neither pericardial effusions nor other procedural complications were identified.

**Conclusions:** In routine Micra™ implantation with assistance of fluoroscopic imaging, the majority of the pacing sites located in the region of the anterior insertion between the RV septum and free wall. The ventricular activation propagation likely depended on the pacing sites.

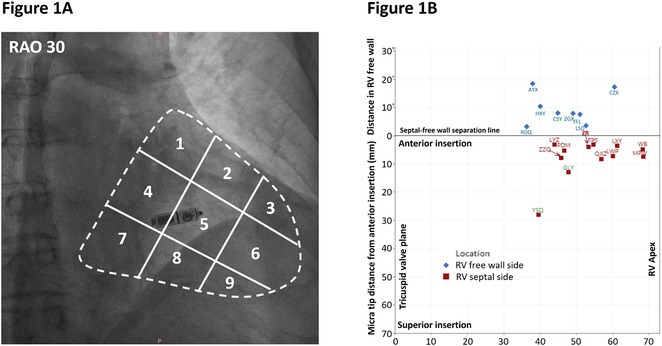

## ASSOCIATION BETWEEN COMPLETE HEART BLOCK AND STROKES

### **DANLU LIANG** ^1,2^, ZHONG YANG LI^1^, JOSHUA BOWDITCH^1^, JITHIN SAJEEV^1^, JAYA CHANDRASEKHAR^1,3^

#### ^1^Eastern Health, Melbourne, Australia,^2^University of Melbourne, Melbourne, Australia,^3^Monash University, Melbourne, Australia

**Introduction:** Complete heart block (CHB) results in complete dissociation and dyssynchrony of the atria and ventricles, causing independent contraction. This leads to atria contracting against a closed valve, potentially resulting in stasis of blood. To maintain cardiac output, there is a physiological increase in systolic blood pressure. We describe 4 patients who presented with concurrent CHB and stroke.

**Methods:** N/A

**Results:** Patient 1 was a 48‐year‐old man presenting with dizziness, confusion and headache on a background of surgical VSD repair 32‐years‐ago with MRI brain showing an acute right middle cerebral artery stroke. His electrocardiograph showed right‐bundle‐branch‐block and CHB with a ventricular rate of 42bpm. Patient 2 was a 64‐year‐old‐man presenting with dizziness, blurred vision and headache in setting of high‐grade 2:1 atrioventricular block. He had a partial 3^rd^ cranial nerve palsy and during his admission developed acute confusion with MRI brain showing multi‐territory infarcts in supratentorial brain, bilateral cerebelli and right pons. Patient 3 was a 68‐year‐old‐man presenting with light‐headedness and syncope with confusion, dysarthria and right‐sided hemiplegia. MRI brain showed left parietal and inferior temporal gyrus infarcts. On monitor he had intermittent CHB with prolonged ventricular pauses. Patient 4 was a 77‐year‐old‐man who presented with light‐headedness, left‐sided facial droop and dysarthria with MRI brain confirming acute right frontal infarct. He was haemodynamically unstable with hypotension and bradycardia 24bpm in CHB. All 4 patients received a permanent pacemaker.

**Conclusions:** CHB symptoms of dizziness, headaches and syncope can mimic that of a stroke. We describe a case series of presented with concurrent CHB and strokes. One proposed mechanism is due to potential stasis of blood in the atria during closed contraction against the atrioventricular valves and reflex hypertension, this could acutely increase the risk of stroke.

## MIRROR MIRROR IN THE LEADS, IS THERE INSULATION BREACH?

### **DANLU LIANG** ^1,2^, GREGORY KING^1^, JANET CHEN^1^, TROY WATTS^3^, HAN LIM^1,2^

#### ^1^Austin Health, Melbourne, Australia,^2^University of Melbourne, Melbourne, Australia,^3^Royal Melbourne Hospital, Melbourne, Australia

**Introduction:** Insulation defects are the commonest reason for pacemaker lead failure with increasing incidence over time, usually due to subclavian crush. Modern leads are coaxially‐designed with 2 concentric metal conductors surrounded by insulation. Breaches may be visualised on CXR and via other markers including lead noise, rising impedance, polarity switch or pacing inhibition. In this case series, we describe a lesser‐known phenomenon of “mirror image artefact" on EGM, indicating insulation breach of 2 adjacent leads.

**Methods:** N/A

**Results:** 5 patients with “mirror image artefact” all had silicone‐insulated Medtronic leads with features of lead‐failure including noise, high SIC‐counter, threshold rise or polarity switch (Table 1). 2 patients underwent lead extraction, as recommended by the manufacturer due to unpredictable course progression. This revealed an outer insulation breach exposing the conductor (Figure 1). CXR changes were only seen in Patient 2 who had increased thresholds, corresponding to more severe breach down to the inner coil. Extraction was not appropriate/necessary in the remaining 3 patients. Instead, their leads were programmed to unipolar for pacing and sensing, sensitivity adjusted and isometric exercises conducted assessing for myopotentials to exclude other causes of noise.

**Conclusions:** These cases highlight “mirror image artefact”, an under‐recognised, serious type of cross‐talk suggesting insulation breaches in adjacent leads at the same position where outer conductors are in direct contact with each other causing communication of current. Unlike conventional crosstalk, signals and noise are mirror images of the other lead's EGM (Figure 1). Concurrent markers of lead failure are present. Management includes unipolar programming, radiological assessment of lead integrity and consideration of lead extraction or replacement.

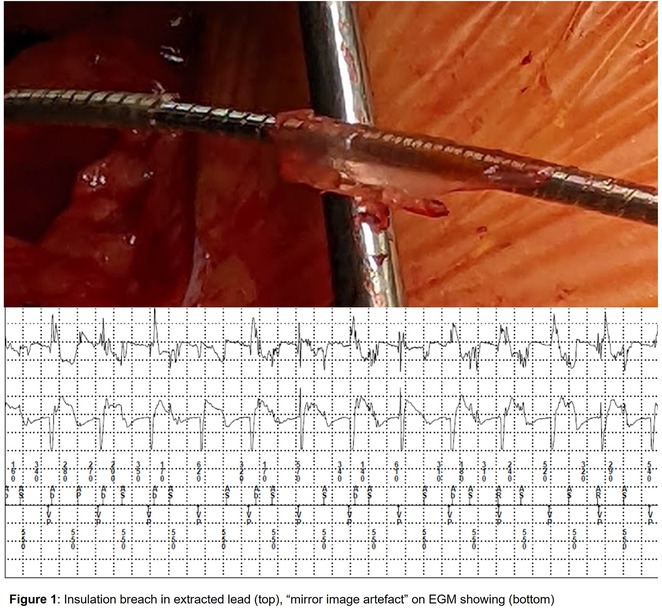

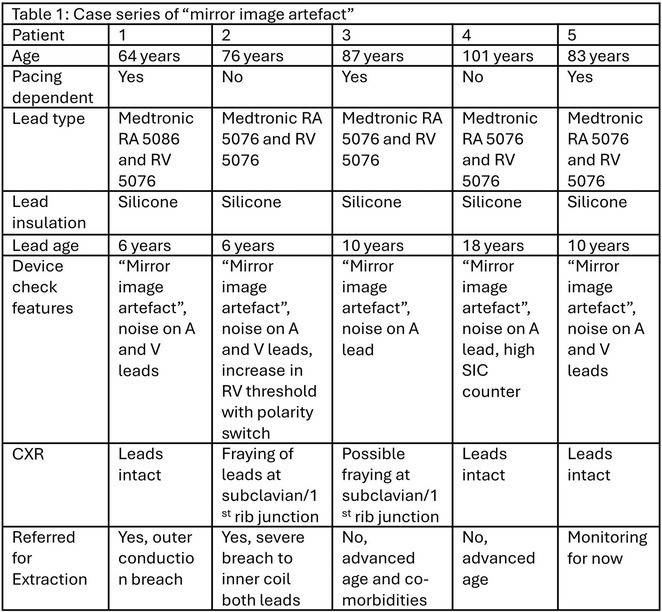

## PACING CHALLENGES FOR SINUS NODE DYSFUNCTION (SND) FROM SUPERIOR VENA CAVA (SVC) TUMOUR EXCISION

### **DANLU LIANG** ^1,2^, NATALIE WHITEHEAD^1^, SIVEN SEEVANAYAGAM^1,2^, HAN LIM^1,2^

#### ^1^Austin Health, Melbourne, Australia,^2^University of Melbourne, Melbourne, Australia

**Introduction:** Sinoatrial node (SAN) is the natural pacemaker determining heart rate (HR) and is situated in the upper right atrium (RA) at the SVC and crista terminalis junction. Damage from cardiac surgery, ischaemia, fibrosis or infiltration can cause SND whereby pacemaker function is superseded by other myocytes resulting in atrial ectopic or junctional rhythm. We describe a case of SND from SVC tumour excision causing symptomatic chronotropic incompetency (CI) requiring permanent‐pacemaker (PPM) insertion.

**Methods:** N/A

**Results:** A 58‐year‐old‐man with progressively obstructive SVC‐RA benign epithelioid haemangioma tumour (Figure 1) underwent surgical resection and SVC reconstruction using a 20mm Gore‐Tex‐graft. He was in normal sinus rhythm pre‐operatively but post‐operatively developed junctional rhythm (Figure 1). This was attributed to inadvertent SAN excision. PPM insertion was considered with challenges given potential lack of RA tissue post‐surgery and/or disruption to synthetic graft. PPM insertion was postponed after telemetry revealed intermittent ectopic low atrial rhythm and HR incrementation to 147bpm. 1‐month post‐operatively, holter monitor showed predominant junctional rhythm with periods of atrial ectopic rhythm and blunted HR 38‐84bpm, average 54bpm. Stress echocardiogram demonstrated reduced maximum HR 106bpm (65% predicted) with fatigue and dyspnoea, consistent with symptomatic CI. A dual‐chamber Medtronic endovascular PPM was inserted with a thin strip of RA tissue found for active fixation lead. At 3‐month check, he remained atrial‐paced 99% and ventricular‐paced 1%.

**Conclusions:** This interesting case of SND post‐SVC surgery highlights the vulnerability of the SAN in these operations. Pacing requirements should be considered including endocardial and epicardial options with their respective challenges. Vigilant assessment of chronotropic incompetency post‐surgical recovery identifies whether long‐term pacing is warranted.

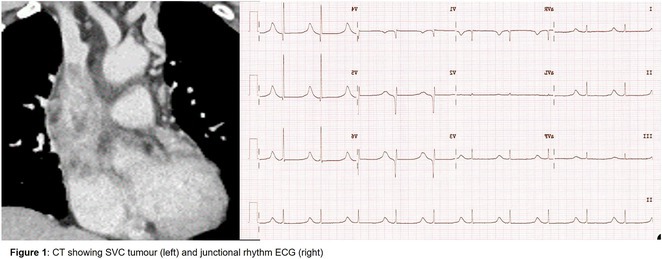

## PERFORMING STELLATE GANGLION BLOCK (SGB) FOR MANAGEMENT OF INCESSANT VENTRICULAR TACHYCARDIA (VT)

### **DANLU LIANG** ^1,2^, HASSANAIN ALTAMIMI^1^, SHAREEN JAIJEE^1^, GEOFFREY LEE^1,2^, ROBERT ANDERSON^1^

#### ^1^Royal Melbourne Hospital, Melbourne, Australia,^2^University of Melbourne, Melbourne, Australia

**Introduction:** Stellate ganglion (SG) is fusion of the inferior cervical and 1^st^ thoracic sympathetic ganglions providing sympathetic nerve branches to head, neck, upper limbs and heart. SG is anterior to C7 transverse process, prevertebral fascia (PF) and longus colli (LC), posterior to carotid (CA), jugular vein (IJV) and vertebral artery (VA), medial to scalene muscles and lateral to midline structures. SGB is typically performed for pain syndromes and hyperhidrosis. Due to sympathetic cardiac innervation, SGB can also treat ventricular arrhythmias driven by sympathetic tone. We describe a case report of incessant VT refractory to medication and ablation that was successfully managed with SGB.

**Methods:**

**Results:** 72 year‐old‐man with secondary prevention ICD and severe non‐ischaemic sarcoid cardiomyopathy presented with VT storm. Despite medical therapy with amiodarone, mexiletine, procainamide and high‐dose methylprednisolone, he had ongoing VT. He underwent endocardial VT ablation with 14 different VT morphologies visualised. Extensive LV and RV ablations resulted in 6 VT terminations however had persistent slow VT. He was referred for left SGB. SGB was performed under sedation with ultrasound (US) guidance. He was positioned supine, head up 45° turned to the right. US at neck base identified C7 level with CA adjacent to IJV and VA posterior. SG is deep to VA but block is performed at C6 to avoid VA by moving probe superiorly until no longer visible (Figure1,2). A 50mm 25G needle was attached to syringe of 0.5% ropivacaine (long‐acting sodium‐channel blocker) and 4mg dexamethasone (prolong blockade). Needle was directed to where VA was, anterior to LC, lifting CA. At targeted area, 6mL was injected. SGB was confirmed with transient ipsilateral Horner's syndrome, arm temperature increase and nasal congestion. He remained VT free for 24 hours after SGB and is planned for epicardial VT ablation when stable.

**Conclusions:** SGB is an effective, minimally invasive method to treat intractable VT refractory to medications as a bridge to more definitive/invasive therapies, particularly in unstable patients. Longer SGB can be considered with continuous infusion.

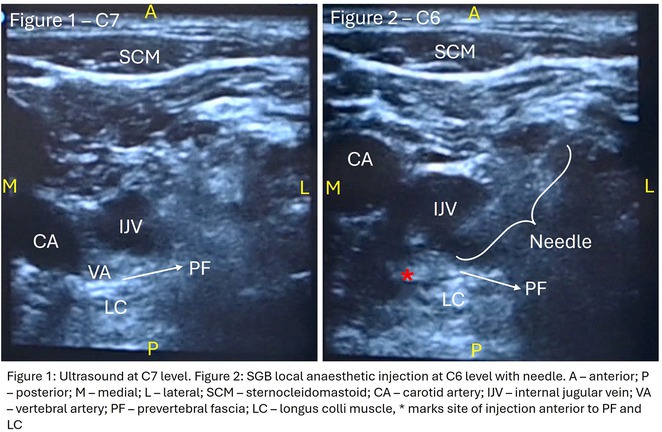

## VERY LATE PRESENTATION OF COMPLETE HEART BLOCK (CHB) POST‐SURGICAL VENTRICULAR SEPTAL DEFECT (VSD) REPAIR

### **DANLU LIANG** ^1,2^, ZHONG YANG LI^1^, JOSHUA BOWDITCH^1^, JAYA CHANDRASEKHAR^1,3^

#### ^1^Eastern Health, Melbourne, Australia,^2^University of Melbourne, Melbourne, Australia,^3^Monash University, Melbourne, Australia

**Introduction:** Surgical VSD repair is complicated by CHB in 1‐3%, often within the first 14 days. Late presentation CHB occurring beyond 30 days is rare with few cases described. We report a case of CHB decades post‐surgical VSD repair.

**Methods:** N/A

**Results:** A 48‐year‐old man presents with dizziness, confusion, headache and fevers on a background of surgical VSD repair 32‐years‐ago. On examination, he had a left‐sided homonymous hemianopia and sensory neglect with MRI brain confirming an acute right middle cerebral artery stroke. His electrocardiograph showed right‐bundle‐branch‐block and CHB with a ventricular rate of 42bpm (Figure 1). This raised the suspicion of infective endocarditis causing stroke and CHB and he was initiated on empirical antibiotics and an isoprenaline infusion. His blood cultures were negative and his transthoracic echocardiogram which showed no residual VSD or endocarditis, but there was concentric hypertrophy and a positive saline contrast study suspicious for shunt. Transoesophageal echocardiogram, however, demonstrated no aortic root abscess, no residual VSD or atrial septal defect. Cardiac MRI also showed no infiltrative processes. The impression was his CHB was chronic and a sequelae of his VSD repair. A dual chamber permanent pacemaker was inserted and he made a good recovery at neurorehabilitation.

**Conclusions:** Given all other common aetiologies of CHB were excluded and the patient's young age, it is likely his previous surgical VSD repair contributed to the development of CHB. This case highlights that CHB remains a post‐operative risk decades after the initial operation and to be vigilant in patients presenting with CHB post‐VSD repair.

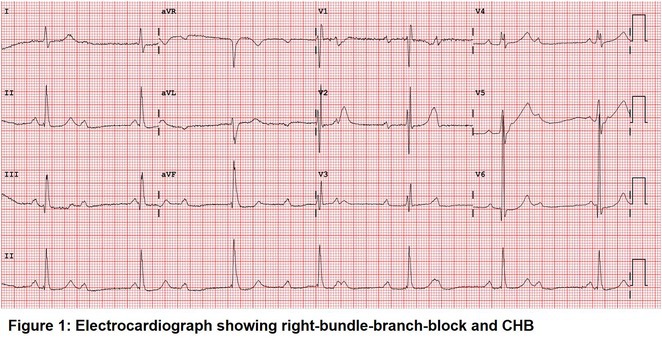

## COMPARISON OF BALLOON COMPLIANCE BETWEEN NORDICA AND ARCTIC FRONT ADVANCE CRYOBALLOON

### RONG BAI^1^, KEVIN ROSENTHAL^2^, **YU LIAO** ^3^, JUSTIN VESSEY^2^, MEITAL MAZOR^2^, XUNZHANG WANG^4^, WILBER W. SU^1^

#### ^1^Banner University Medical Center‐Phoenix, Phoenix, AZ,^2^Synaptic Medical, Carlsbad, CA,^3^National Cheng Kung University Hospital, Tainan, Taiwan,^4^Cedars‐Sinai Medical Center, Los Angles, CA

**Introduction:** The Arctic Front series cryoballoon (CB, Medtronic) has a two‐layer balloon design and holds 18psi intra‐balloon pressure during ablation, which is thought to be associated with poor balloon compliance. Although the Arctic Front CB inflates to 3psi prior to freezing, its “jump” to the high ablation pressure may result in expansion dislodgement (i.e., “pop‐out effect”) of the CB when freezing starts and subsequent incomplete pulmonary vein isolation (PVI). The novel Nordica CB developed by Synaptic Medical possesses a single‐layer polyurethane surface and holds 3psi (28mm) or 5psi (31mm) intra‐balloon pressure during inflation and ablation without the nonideal “pop‐out effect.” This design is expected to improve balloon compliance and maintain occlusion with the target pulmonary vein. This study was to test in vitro the compliance of Nordica and Arctic Front Advance CB.

**Methods:** A tensile tester was used to advance an inflated CB (Arctic Front Advance 28mm; Nordica 28mm and 31mm) at a constant speed (100mm/min) for a set distance into a 3D‐printed, plastic fixture that simulated an ovular‐shaped plastic PV ostium of approximal 19mm in diameter (Figure). The stroke length was set at 2mm. The force encountered by the CB when it was pushed into the ostium was recorded. The less force encountered suggested more balloon compliance.

**Results:** CB‐PV ostium engagement was repeated 15 times for each of the scenarios (3 CB catheters were used for each scenario, each CB catheter was tested 5 times). The Arctic Front Advance CB at 28mm encountered the highest force (982±48g) followed by the Nordica CB at 31mm (298±24g) and the Nordica CB at 28mm (188±16g), p<0.0001 for all paired comparison.

**Conclusions:** The Nordica CB, both at 28mm and 31mm exhibited significantly more compliance than the Arctic Front Advance CB, which may avoid expansion dislodgement (“pop‐out effect”) of the CB during freezing and improve efficacy of PVI.

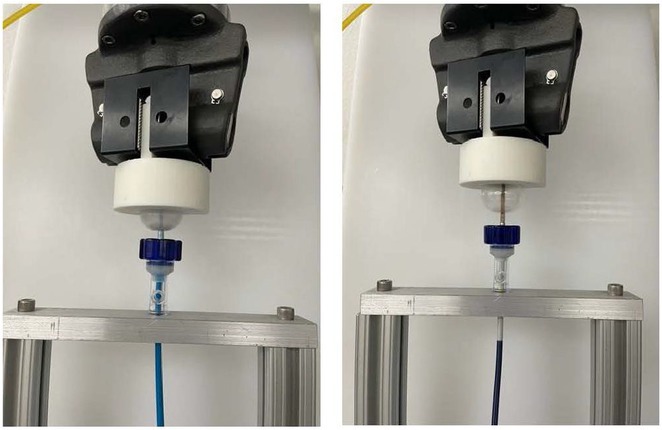

## CONVERGENT MAZE PROCEDURE IN THE TREATMENT OF ATRIALFIBRILLATION: SINGLE CENTER EXPERIENCE

### RONG BAI^1^, DALISE SHATZ^2^, **YU LIAO** ^3^, TRUC VY LAM^4^, YASUTOSHI SHINODA^1^, ASHISH SADHU^5^, RENZO M. CATALDO^6^, MICHAEL ZAWANEH^1^, PETER J. WEISS^1^, WILBER W. SU^1^, H. KENITH FANG^1^

#### ^1^Banner University Medical Center‐Phoenix, Phoenix, AZ,^2^University of Arizona College of Medicine‐Phoenix, Phoenix, AZ,^3^National Cheng Kung University Hospital, Tainan, Taiwan,^4^New York University, New York, NY,^5^Cardiovascular Institute of Scottsdale, Phoenix, AZ,^6^Northern Arizona Healthcare, Phoenix, AZ

**Introduction:** Outcome data on the safety, efficacy and long‐term durability of the Convergent Maze (CM) procedures for treatment of AF in challenging cases have been inconsistent and sparse. This study is to report our single center, single surgeon experience of CM for the treatment of symptomatic atrial fibrillation (AF).

**Methods:** Data of 43 AF patients (Table 1) who underwent standalone CM were collected. All procedures were done via subxiphoid approach. Standard lesion sets included 2‐3 rows of parallel linear ablations on the left atrium (LA) posterior wall between the left and right PVs. The cephalad extent of ablation was the pericardial reflection extending as far up as possible to the LA roof and caudal extent approximately 1cm from the CS (Figure 1A). Left atrial appendage closure (LAAC) via a thoracoscopy was performed at the operator's discretion.

**Results:** AF was longstanding persistent in 19 patients, persistent in 17 and recurrent paroxysmal in 7. 38 patients had at least 1 (median 2) failed endocardial catheter ablation (CA). Of the 5 cases without prior CA, 3 had contraindication to anticoagulant. The remaining 2 patients optioned for a hybrid strategy with surgical ablation being performed first. Concomitant LAAC was performed in 9 cases. There was 100% procedural success, no major adverse event or in‐hospital death, and only one (2.3%) perioperative complication (partial small bowel obstruction treated medically).3 patients received CA of AF or atrial tachycardia after the CM procedure. Of the patients who had a minimum of 12‐month follow‐up (n=33), 23 patients (69.6%) were free from AF (Figure 1B). Non‐procedural, non‐cardiovascular related death occurred in 2 cases at 4 and 16 months after the operation.

**Conclusions:** The CM procedure as an adjunct treatment option for recurrent AF after failed CA is a safe and reproducible treatment option. Initial 12‐month follow up data shows promise for a reasonable rate of success that is consistent with published literature.

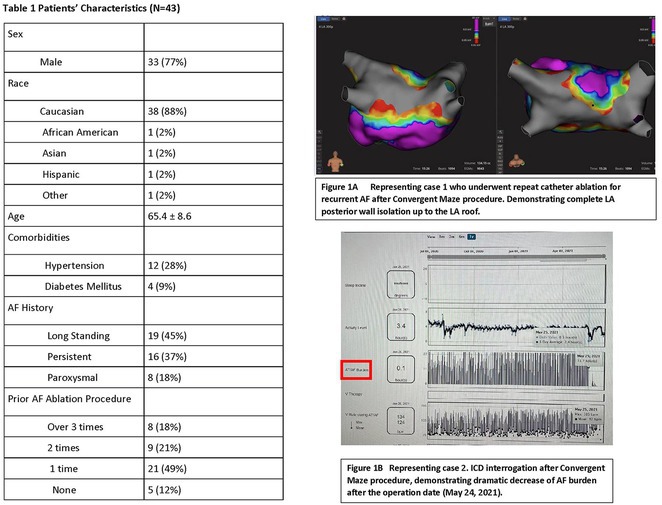

## COOLING PERFORMANCE OF A NEW CRYOABLATION SYSTEM IN COMPARISON TO THE ARCTIC FRONT CRYOBALLOON

### RONG BAI^1^, KEVIN ROSENTHAL^2^, **YU LIAO** ^3^, JUSTIN VESSEY^4^, MEITAL MAZOR^2^, XUNZHANG WANG^5^, WILBER W. SU^1^

#### ^1^Banner University Medical Center‐Phoenix, Phoenix, AZ,^2^Synaptic Medical, Carlsbad, CA,^3^National Cheng Kung University Hospital, Tainan, Taiwan,^4^Synaptic Medical, Phoenix, AZ,^5^Cedars‐Sinai Medical Center, Los Angles, CA

**Introduction:** Although the Arctic Front series cryoballoon (CB) has been the only available cryoablation system for over a decade, a few draw backs have been associated with this technology. A new cryoablation system with improved design (Nordica) was recently developed by Synaptic Medical and is expected to improve the efficacy and safety of atrial fibrillation (AF) ablation. This study was designed to test in vitro cooling performance of the Nordica CB in comparison to the Arctic Front Advance CB.

**Methods:** The test was conducted in agar molds with an array of thermocouples embedded at 0 mm, 3 mm, and 6 mm from the surface of the CBs (Nordica vs. Arctic Front). The Nordica CB has adjustable size and was inflated to 28mm (3psi) and 31mm (5psi) respectively, while the Arctic Front CB was inflated to 28mm during the test. Ablations were performed for a duration of 180 seconds while the fixture was submerged in a body temperature water bath at 37°C. The average nadir temperatures (NT) at each depth were recorded and compared for both CBs.

**Results:** 60 Nordica (30 each for 28mm and 31mm) and 12 Arctic CB applications were tested. Although the NT on the surface of CB (0mm) was similar in both groups, the Nordica CB produced significantly lower NT at 3mm and 6mm from the CB surface, as compared to the Arctic Front CB. The NT at 6mm from CB surface was above 0°C for both CBs. Within the first 30s after freezing started, the NT at CB surface (0mm) reached 1.17°C (28mm) or ‐0.63°C (31mm) for Nordica CB and 5.0°C for Arctic Front CB (Figure).

**Conclusions:** In comparison to the Arctic Front CB, the Nordica CB at both 28mm and 31mm diameter can rapidly generate lower NT in proximity without increasing the risk of cryo‐injury in the collateral area.

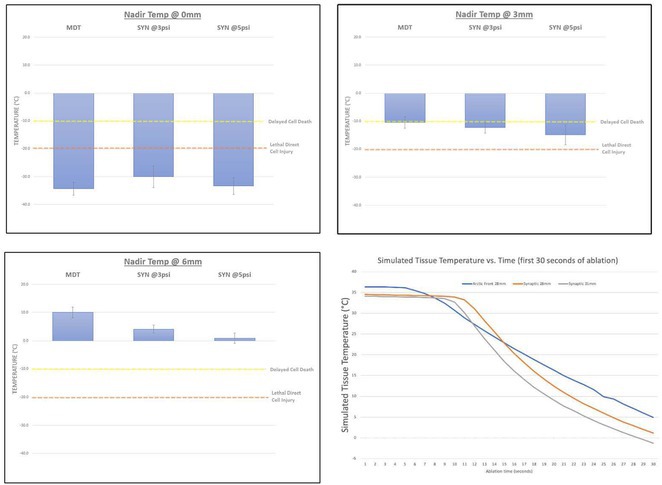

## EXPANDING OF ABLATION AREA WITH ADJUSTABLE‐SIZE CRYOBALLOON: DEMONSTRATION OF A 3‐DIMENSIONAL MODEL

### **YU LIAO** ^1^, DAVID J. SMITH^2^, SAM MURTHY^2^, MICHAEL ZAWANEH^3^, PETER J. WEISS^3^, RODERICK TUNG^4^, PRANEETH KATRAPATI^3^, JAKE MARTINEZ^3^, RONG BAI^3^, WILBER W. SU^3^

#### ^1^National Cheng Kung University Hospital, Tainan, CA,^2^Boston Scientific, St. Paul, MN,^3^Banner University Medical Center‐Phoenix, Phoenix, AZ,^4^University of Arizona College of Medicine‐Phoenix, Phoenix, AZ

**Introduction:** The expandable design of POLARx FIT cryoballoon (CB, Boston Scientific) allows it to be adjusted to 28mm in dimension, which can be expanded to 31mm. The 31mm balloon size offers 20% greater surface area that is expected to be translated into a greater balloon‐antrum contact area during freezing. This study was designed to establish a 3‐dimensional model to quantify the ablation area difference with the adjustable‐size CB.

**Methods:** During a PVI procedure, the POLARx FIT CB was initially inflated to 28mm then increased to 31mm while tracking geometrical engagement changes by continuous contrast injection and cine. Prior CT imaging stack was analyzed to identify the outlines of the LA and PV structures. The cardiac tissue of LA and PVs were modeled by finite element analysis as a two‐layered elastic structure with resistance to deformation under an applied force. The POLARx FIT CB was positioned in the 3D space based on the angle of the balloon to the cine view plane and the angle to the patient's transverse plane, which were derived mathematically with the fluoroscopy cine imaging during procedure. The model calculated the balloon‐antrum “affected area” as a function of balloon displacement toward the PV ostium. The affected area was defined as the area between the proximal edge of the balloon/tissue contact area and the ostium when the contact area fully rang the PV antrum. The final balloon‐antrum contact area was the affected area when the PV was fully occluded.

**Results:** Six PVs (2 LSPV, 1 LIPV, 1 RSPV, 2 RIPV) with full occlusion by both 28mm and 31mm CB were used for 3D modeling and analysis. Inflating the CB from 28mm to 31mm resulted in a displacement of the center of CB with 0.25±0.09 cm and an expanding of balloon‐antrum contact area with 1.31±0.7 cm2 (Example of one case shown in Figure).

**Conclusions:** A 3‐dimensional model was established to simulate and calculate balloon‐antrum contact area. It demonstrated an average of 1.31 cm2 increase in balloon‐antrum contact area when adjusting the POLARx FIT CB from 28mm to 31mm.

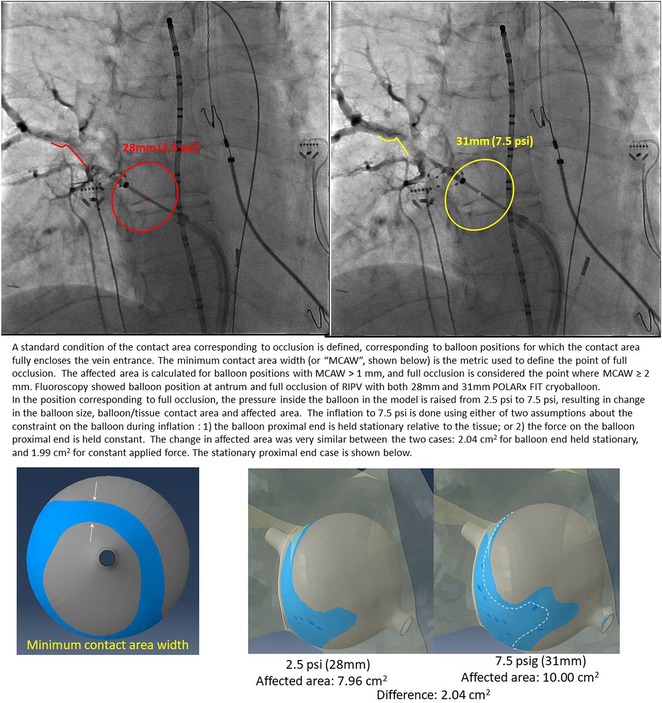

## IMPROVEMENT IN THE DESIGN OF DELIVERY SHEATH FACILITATES CRYOBALLOON MANIPULABILITY: COMPARISON BETWEEN NORDICA AND ARCTIC FRONT CRYOABLATION SYSTEMS

### RONG BAI^1^, KEVIN ROSENTHAL^2^, **YU LIAO** ^3^, JUSTIN VESSEY^2^, MEITAL MAZOR^2^, XUNZHANG WANG^4^, WILBER W. SU^1^

#### ^1^Banner University Medical Center‐Phoenix, Phoenix, AZ,^2^Synaptic Medical, Carlsbad, CA,^3^National Cheng Kung University Hospital, Tainan, Taiwan,^4^Cedars‐Sinai Medical Center, Los Angles, CA

**Introduction:** One of the drawbacks associated with the Arctic Front cryoablation system (Medtronic) is its manipulability with its corresponding sheath. A new cryoablation system with improved design of cryoballoon (Nordica CB) and its corresponding delivery sheath (NaviGo) was recently developed by Synaptic Medical. This study was designed to test in vitro the force required to advance or retract the Nordica CB within its corresponding NaviGo sheath in comparison to the force associated with the Arctic Front Advance CB and its corresponding FlexCath Advance sheath, in addition to testing the pressure buildup encountered during insertion (i.e., “piston effect”).

**Methods:** A tensile tester was used to advance/retract the CBs with a 0.032” guidewire in place in 1 cm increments to measure the peak force until the CB was fully outside of the distal tip/end of its corresponding sheath at deflection angles of 0°, 90°, and 135° (Figure). With the sheath in a straight configuration, the pressure at the distal end of the sheath (“piston effect”) was measured while inserting the CB catheter into the sheath.

**Results:** The advancement/retraction test was repeated 18 times, while the “piston effect” test was repeated 25 times. A similar force was required to advance the CBs into its corresponding sheath but significantly less force was required to retract the Nordica CB into the NaviGo sheath at each deflection angle (Figure). Significantly less pressure buildup (2.64± 0.383 psi) was encountered when inserting the Nordica CB into the NaviGo sheath, in comparison to 3.78 ±0.295 psi (p<0.0001) when inserting the Arctic Front Advance CB into FlexCath Advance sheath.

**Conclusions:** In comparison to the Arctic Front cryoablation system, significantly less force was required to retract Nordica CB into the NaviGo sheath, and less pressure buildup was encountered when inserting the Nordica CB into the NaviGo sheath, which may decrease the risk of complication associated with CB/sheath manipulation.

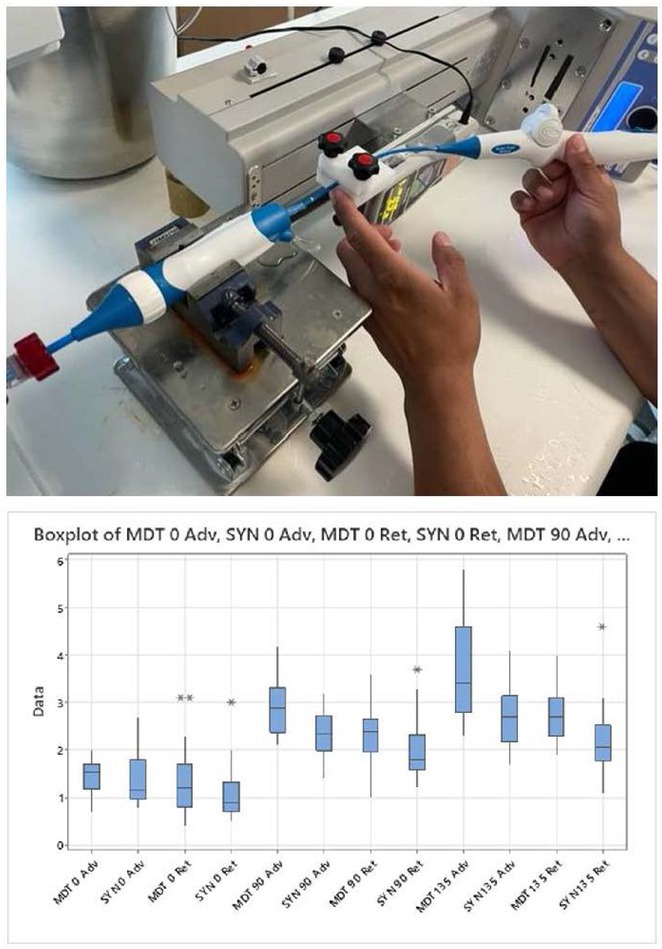

## REFRIGERANT ESCAPEMENT IN CASE OF CRYOBALLOON RUPTURE: COMPARISON BETWEEN NORDICA AND ARCTIC FRONT CRYOBALLOON

### RONG BAI^1^, KEVIN ROSENTHAL^2^, **YU LIAO** ^3^, JUSTIN VESSEY^2^, MEITAL MAZOR^2^, XUNZHANG WANG^4^, WILBER W. SU^1^

#### ^1^Banner University Medical Center‐Phoenix, Phoenix, AZ,^2^Synaptic Medical, Carlsbad, CA,^3^National Cheng Kung University Hospital, Tainan, Taiwan,^4^Cedars‐Sinai Medical Center, Los Angles, CA

**Introduction:** Balloon rupture and refrigerant leak is one of the most lethal complications during a cryoablation procedure. The Arctic Front series cryoballoon (CB) has a two‐layer balloon design and holds 18psi intra‐balloon pressure when freezing. The novel Nordica CB developed by Synaptic Medical possesses a single‐layer balloon and holds 3psi or 5psi intra‐balloon pressure during ablation when it is inflated to the same size (28mm) as an Arctic Front CB or to a larger size (31mm) respectively. Less is known about which of the two designs is associated with less refrigerant leak in case of CB rupture. This study was designed test in vitro the amount of nitrous oxide (N2O) that could leak from the CB if a balloon rupture occurs.

**Methods:** While being inflated to 28mm or 31mm and actively ablating underwater in a saline bath at 37°C, an Arctic Front Advance or a Nordica CB was manually cut with a scalpel at 120th second into the ablation and the N2O floated into an inverted graduated cylinder, allowing measurement of the “gas escapement” (Figure). This compares the volume (mL) of N2O held inside the balloon during ablation and the response time of the system to prevent any further refrigerant delivery upon detecting a balloon breach.

**Results:** A total of six Nordica CBs (3 each for 28mm and 31mm) and ten Arctic Front Advance CBs were tested with their respective cryo console. Rupture of a Nordica CB, regardless of size being inflated, is associated with significantly less refrigerant leak as compared to an Arctic Front Advance CB (Table).

**Conclusions:** In comparison to the single‐layer and low‐intra‐balloon pressure design of the Nordica CB, the two‐layer and high intra‐balloon pressure of the Arctic Front Advance CB may result in nearly 1.4 to 4.5 times more refrigerant leak in the event of balloon rupture.

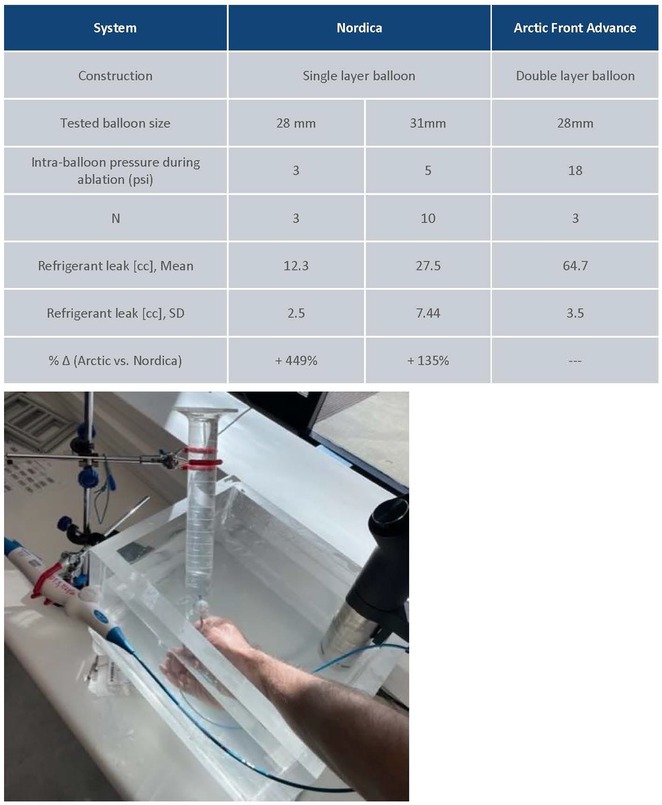

## A VERY RARE CASE OF INTER‐ATRIAL CYST RESULTING TOTAL ATRIOVENTRICULAR BLOCK: A DILEMMA OF PACING OR SURGERY FIRST

### **JEFRI LIM**, PRIMA AGUNG LAKSONO, BENNY MULYANTO SETIADI, GRATIANI EBEN H. REPPI, AGNES LUCIA PANDA

#### Department of Cardiology and Vascular Medicine Sam Ratulangi University Prof. Dr. R.D. Kandou General Hospital, Manado, Indonesia

**Introduction:** Cystic tumors of the AV node are very rare and can disrupt the conduction system. Until now, due to the small number of cases, treatments for patients with cystic tumors in the AV node which cause atrioventricular block not yet established.

**Methods:** N/A

**Results:** A 68 year old male with syncope and fatigue admitted to emergency unit diagnosed with permanent total AV block planned for installation of permanent percutaneous pacemaker. During observation in the ICCU, hemodynamic echocardiography was carried out and a suspicion of an inter‐atrial cystic mass was found. A full echocardiography examination revealed a cystic mass 1.97 x 2.11 centimeters of size in the inter‐atrial septum close to the non‐coronary cusp. Consultation was carried out with thoracic and cardiovascular surgeon to discuss the disease due to its rarity and no specific treatment for this case in the literature. From various cases that have been reported both antemortem and postmortem, although the mass is cystic in shape, its nature tends to infiltrate and damage the conduction system as obtained from histological examination. From several reported cases, the risk of TAVB due to surgical complications is also very high. We decided to install a PPM first because it was hypothesized that permanent damage had occurred to the conduction system and the nature of the cyst in this patient did not affect hemodynamics.

**Conclusions:** Total AV block caused by interatrial cysts from several case reports tends to be permanent even after surgery. PPM installation in this case where damage to the conduction system is suspected cannot be avoided.

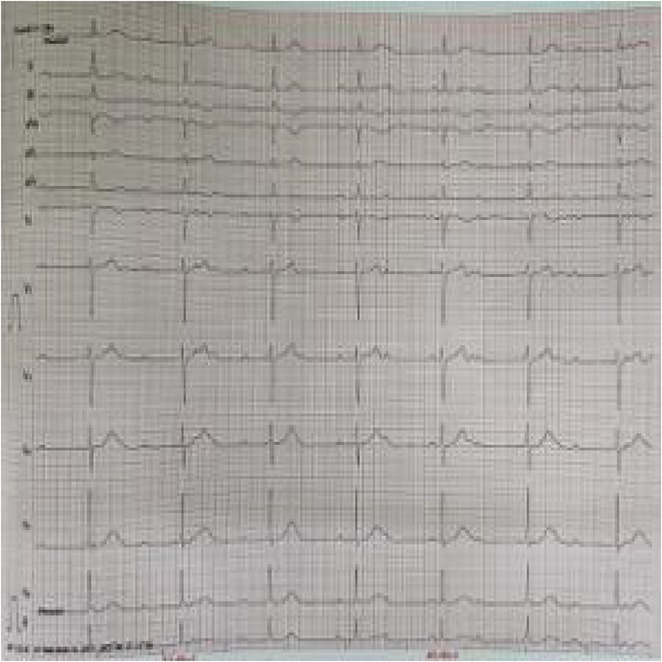

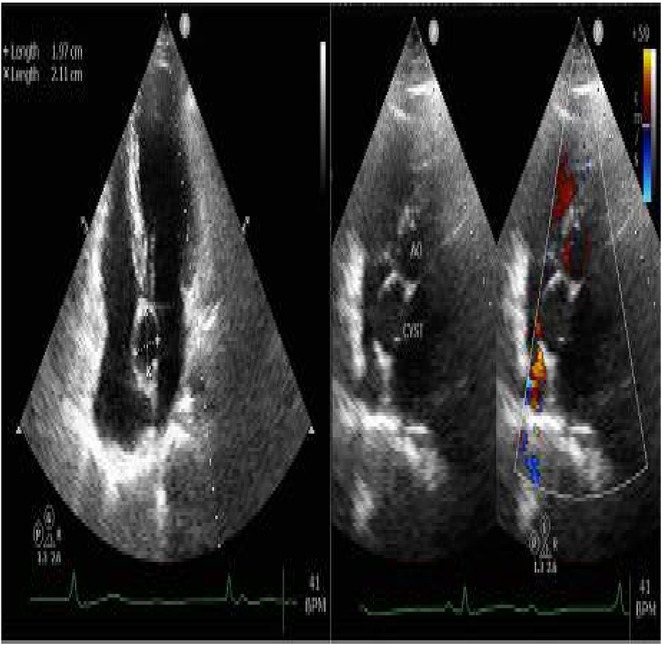

## PULSE FIELD ABLATION FOR ATRIAL FIBRILLATION ‐ AN INITIAL ASIAN POPULATION EXPERIENCE

### **PAUL CHUN YIH LIM**, KELVIN CHEOK KENG WONG, WEE SIONG TEO

#### Mount Elizabeth Hospital Singapore, Singapore, Singapore

**Introduction:** Pulse field ablation (PFA) is a novel modality of atrial fibrillation (AF) ablation proven to be effective and safe. We report our intial Asian experience with PFA.

**Methods:** 82 consecutive patients were enrolled in our PFA AF ablation study utilising the Farapulse (Boston Scientific, MA, USA) PFA system combined with electroanatomic mapping. Primary endpoints were success with acute pulmonary vein isolation (PVI) and safety endpoints were major adverse complications related to this modality of energy delivery. Secondary endpoints were efficacy of posterior wall (PW) isolation and mitral isthmus (MI) ablation

**Results:** Our cohort consisted of 58 (70.7%) with paroxysmal and 24 (29.2%) persistent AF. 12 (19.5%) of the cohort were redo‐AF ablation procedures, mostly from prior radiofrequency ablation. PFA was highly efficacious in achieving PVI for 81 of 82 patients (98.8%). The failure was due to lack of familiarity with catheter system manipulation which we feel we have overcome. PFA time to achieve complete PVI was short at 27.7 ± 9.3 mins with delivery of 46.4 ± 11.7 PFA lesions. PW isolation was performed in 39 (48.2%) patients and was completely successful, requiring only 7.8 ± 3.4 mins to deliver 14.0 ± 5.6 flower applications. MI ablation was undertaken in 15 (18.3%) patients with PFA alone achieving block in 4 of these patients (26.7%). 3 complications occurred: One air embolism from catheter exchange, one tamponade from atrial flutter ablation and one patient with variant left inferior pulmonary vein anatomy developed transient coronary artery spasm during PVI.

**Conclusions:** Our experience showed very high rates of success for AF ablation for paroxysmal or persistent AF and in redo AF ablations. It shows high efficacy in PW isolation and can achieve or facilitate MI ablation.

## INTERLEUKIN‐11 CAUSES ACUTE CARDIAC ELECTROPHYSIOLOGICAL DYSFUNCTION AND PREDISPOSES THE HEART TO ARRHYTHMIA

### **WEI‐WEN LIM** ^1,2^, TIANXIN YE^3^, CHEN XIE^1^, YU‐NING LIU^2^, LIPING SU^1^, ANGELINA CHENG^1^, MARK SWEENEY^4^, ANISSA WIDJAJA^2^, STUART COOK^1,2,4^

#### ^1^National Heart Centre Singapore, Singapore, Singapore,^2^Duke‐National University of Singapore Medical School, Singapore, Singapore,^3^Zhejiang University, Zhejiang, China,^4^MRC Laboratory of Medical Sciences, London, United Kingdom

**Introduction:** Interleukin‐11 (IL11) is used to treat chemotherapy‐induced thrombocytopenia. However, a substantial proportion of patients develop severe cardiac arrhythmias, which remain unexplained. Here we dissected the electrophysiological impact of clinically‐relevant doses of IL11 in the mouse.

**Methods:** We investigated the effect of IL11 on surface ECG morphology and its impact on caffeine‐epinephrine induced arrhythmias. We administered either recombinant mouse IL11 (rmIL11) (50 μg/kg/day; for 21 days; IP) or an equal volume of saline and monitored ECG parameters in isoflurane‐anesthetized 10‐week‐old C57BL/6J mice at intervals from day 0 (d0; first dose) to d21. At d10 and d21, arrhythmia susceptibility was evaluated by caffeine‐epinephrine challenge. Additionally, we studied age‐dependent predisposition to transesophageal pacing‐induced atrial arrhythmia prior and post single bolus rmIL11 administration (50 μg/kg; IP) in young (10‐week‐old) and aged (18‐month‐old) mice.

**Results:** ECG analyses revealed significant P‐wave prolongation immediately following IL11 administration (d0) that persisted throughout the course of treatment (to d21). Repeated dosing of IL11 progressively increased the QT interval (QT) and Tpeak‐end, suggesting abnormal cardiac repolarization in IL11‐treated mice. Heart rate variability (HRV), measured by time‐ and frequency‐domain indices, was unaffected by repeated IL11 administration suggesting that autonomic remodeling was not impacted. Progressive incidence of T‐wave alternans, non‐sustained ventricular arrhythmia (NSVA), bidirectional ventricular tachycardia (BDVT), ventricular bigeminy, and atrial fibrillation (AF) was observed in IL11‐treated mice at d10 and d21 post caffeine‐epinephrine challenge. Aged mice displayed increased AF susceptibility after one dose of rmIL11.

**Conclusions:** Our results show for the first time that IL11 causes acute electrophysiological dysfunction and predisposes the heart to atrial and ventricular arrhythmias. These findings likely explain the common occurrence of arrhythmia seen in patients receiving IL11 therapy.

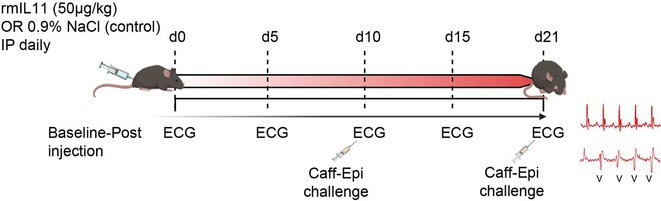

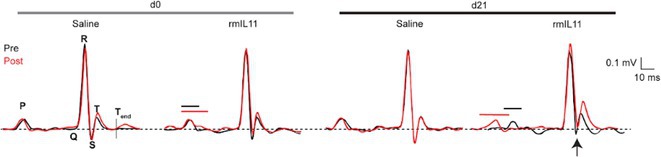

WOLFF PARKINSON WHITE WITH TWO ACCESSORY PATHWAYS

**YEE HUI LIM**, DR SOFIAN BIN DATO PADUKA DR HAJI JOHAR, DAYANGKU NUR IZYAN NADHIRAN BINTI PG HJ MOHAMMAD

Raja Isteri Pengiran Anak Saleha Hospital, Bandar seri begawan, Brunei Darussalam

**Introduction:** Patient with Wolff Parkinson White (WPW) syndrome typically have one accessory pathway; cases with multiple accessory pathways are rare. This is a case of WPW with two accessory pathways.

**Methods:** N/A

**Results:** A 15year old male with no significant medical history presented with palpitation and a brief loss of consciousness. His baseline ECG showed an R/S ratio <1 at V1 and positive delta wave II, III and avF indicative of right anteroseptal accessory pathway (AS AP). An electrophysiology (EP) study was performed, during which decremental atrial pacing and with atrial extra stimulus revealed a switch of anterograde AP conduction from right anteroseptal AP to right lateral pathway likely reaching right AS AP ERP. An electroanatomic map of right atrium using open window mapping (OWM) technique with pacing from High Right atrium (HRA) was performed. Site with fused atrioventricular (AV) signals and probable AP site was tagged on the electronic ‐ anatomical map. OWM revealed possible 2 breakthrough site along the tricuspid annulus (TA) at 1 o’clock and 9 o’clock ‐ confirming presence of 2 accessory pathways. Right anteroseptal AP with fused AV signals was tagged and distance from nearest HIS signal at 2 o’clock TA was identified. However, persistent pre‐excitation with negative delta waves on lead III was observed during pacing from the HRA. On pacing the earliest A signal on HRA, it confirmed the presence of right lateral AP. Radiofrequency Ablation was done on the right anteroseptal (1 o’clock) and right lateral AP. Following ablation, there were still residual retrograde AP conduction over right lateral AP. Patient underwent a re‐do ablation after one month. The right lateral accessor pathway was blocked. Right anteroseptal AP has weak conduction. RF over right anteroseptal AP resulted in the loss of pre‐excitation.

**Conclusions:** This is a challenging case in view of prolonged procedure duration and early recurrence of delta wave post ablation.

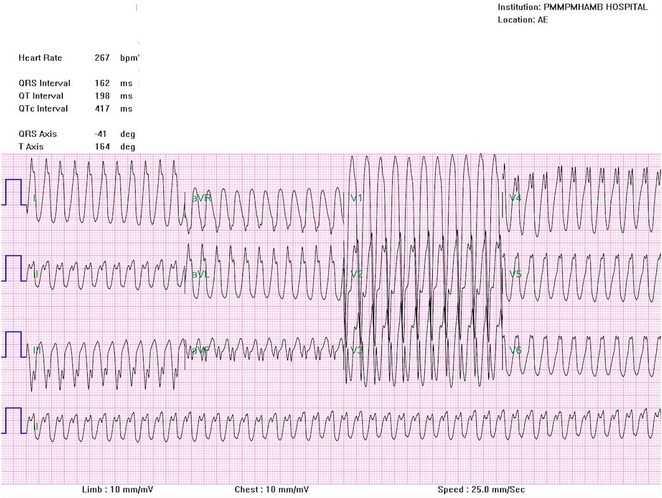

## LEADLESS AND EXTRAVASCULAR CARDIAC IMPLANTABLE ELECTRONIC DEVICES: NINE YEARS EXPERIENCE IN A THAI TERTIARY CARE CENTER, A SINGLE‐CENTER STUDY

### **CHAYAPOL LIMTRAKUL**, NAPAWAN PORNNIMITTHUM, ARISARA SUWANAGOOL

#### Mahidol University, Bangkok, Thailand

**Introduction:** Cardiac implantable electronic devices with transvenous lead are associated with significant complications, including infection and lead‐related issues. Leadless Cardiac Pacemaker (LCPM) and Subcutaneous Implantable Cardioverter‐Defibrillator (S‐ICD) are alternative options to mitigate these risks.

**Methods:** To determine the post‐implantation infection rate of S‐ICD and LCPM and to evaluate other outcomes (Intraoperative, adverse events during and after the procedure, and Follow‐up data).

This single‐center, retrospective study was conducted at Siriraj Hospital. We reviewed the medical records of 50 patients who underwent device implantation at Siriraj Hospital from January 2015 to June 2023 (24 LCPM and 26 S‐ICD).

Data collection included patients’ demographics, CIEDs information, indication for lead removal, intra‐procedural data, and length of stay. Follow‐up was conducted for up to 1 year after implantation.

**Results: LCPM:** 24 patients (66.7% male), mean age 73±12.7 years, mean LVEF of 63±7.3%, 100% implantation success rate, mean procedure time 65±39.7 minutes, with discharged within 4±5.7 days, median time 1.5 days (IQL 1, 3.75 days), 100% complication‐free rate, with no device dislodgment or unfavorable device parameters observed during follow‐up. **S‐ICD:** 26 patients (88.5% male), mean age 45±14.7 years, mean LVEF 58±15.3%, 100% implantation success rate, mean procedure time 86±25.6 minutes, with discharged within 3±3.05 days post‐implantation, median time 2 days (IQL 1, 3.25), 84.6% complication‐free rate from intraoperative to 1 year of follow‐up. Complications included 1 S‐ICD device infection and 2 inappropriate shocks. Complications were managed with definitive strategies, including 1 S‐ICD explantation, 1 lead revision, and 1 instance of off‐therapy programming adjustment.

**Conclusions:** Our study provides compelling evidence of the effectiveness and safety of both LCPM and S‐ICD with only 1 case of post S‐ICD implantation related device infection.

## USING ARTIFICIAL INTELLIGENCE MODEL TO PREDICT OUTCOME IN PATIENTS RECEIVING CATHETER ABLATION FOR PREMATURE VENTRICULAR CONTRACTION ‐ A NATIONWIDE PROSPECTIVE STUDY

### **CHUNGYU LIN** ^1,2^, BEN‐CHANG SHIA^2^, MING‐CHIH CHEN^2^

#### ^1^Division of Cardiology, Department of Cardiology, Fu Jen Catholic University Hospital, New Taipei City, Taiwan,^2^Catholic University, Graduate Institute of Business Administration, Fu Jen Catholic University, New Taipei City, Taiwan

**Introduction:** Premature ventricular contractions (PVCs) rank among the most prevalent arrhythmias in the general populace. Existing guidelines advocate intervention for patients meeting criteria.Despite prior investigations comparing clinical outcomes in patients undergoing catheter ablation for PVCs, conclusive evidence delineating high‐risk patients primed for this invasive therapy, to enhance long‐term outcomes, remains elusive. In this s, we scrutinize five artificial intelligence models to discern patients at elevated risk, thereby earmarking them as candidates for catheter ablation.

**Methods:** 3887 patients with VPC undergoing ablation therapy were enrolled in the study from the Taiwan's National Health Insurance Research Database (NHIRD) system. (1883 men and 2004 women). Five machine learning algorithms were used to analysis the data: logistic regression, decision trees, random forests, XGBoost, and LightGBM.

**Results:** LightGBM_OS demonstrated exceptional performance in this analysis, notably excelling in the AUC metric (0.822), signifying its robust discriminative prowess in predicting heart failure events. Additionally, its equilibrium between sensitivity (0.735) and specificity (0.784) renders it a dependable option for heart failure prognosis. All models view heart failure as the paramount predictor for future heart failure occurrences. Age also emerges as a significant predictor.Additionally, end‐stage renal disease, gender, hypertension, and diabetes mellitus wield notable importance in the model, suggesting a potential influence of gender on heart failure risk.

**Conclusions:** The artificial intelligence model identified age, heart failure, malignancy, end‐stage kidney disease, gender, hypertension, and diabetes mellitus as the six primary risk factors predicting outcomes in patients undergoing catheter ablation. Beyond traditional logistic regression, this AI approach offers an alternative perspective for constructing clinical predictions.

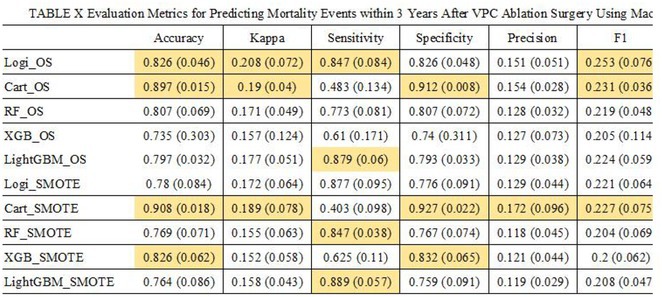

## MITIGATING ARRHYTHMIC RISKS IN MYOCARDIAL INFARCTION RATS THROUGH OPTOGENETIC STIMULATION OF LIGHT‐GATED IONIC PUMPS IN THE ROSTRAL VENTROLATERAL MEDULLA AFFECTED BY SLEEP‐DISORDERED BREATHING‐INDUCED AUTONOMIC DYSFUNCTION

### **WEI LUN LIN** ^1^, LI‐WEI LO^2^, YA‐WEN HSIAO^2^, YU‐HUI CHOU^2^, SHIN‐HUEI LIU^2^, WEN‐HAN CHENG^3^, CHERYL C.H. YANG^4^, TERRY B.J. KUO^4^, SHIH‐ANN CHEN^1^

#### ^1^Taichung Veterans General Hospital, Taichung, Taiwan,^2^Taipei Veterans General Hospital, Taipei, Taiwan,^3^National Yang Ming Chiao Tung University Hospital, Yilan, Taiwan,^4^National Yang Ming Chiao Tung University, Taipei, Taiwan

**Introduction:** Cardiac sympathovagal imbalance from myocardial infarction (MI) can cause fatal arrhythmic death. The rostral ventrolateral medulla (RVLM) regulates hypertension and heart failure by increasing sympathetic outflow. Using optogenetic technology, we manipulated neurons with light‐sensitive ion channels to study this. Our goal was to evaluate the effects of autonomic neuromodulation on MI rats by analyzing sleep patterns and heart rate variability (HRV) following optogenetic stimulation of RVLM neurons.

**Methods:** Polysomnographic data were wirelessly obtained from WKY male MI rats during daytime sleep. The study divided the rats into two groups: Group 1 (vehicle control, n=5), where MI rats received an infection with pAAV‐CaMKIIa, and Group 2 (halorhodopsin, n=5), where MI rats received an infection with pAAV‐CaMKIIa‐eNpHR3.0. Yellow LEDs stimulated the RVLM during the experiments. We conducted spectral analyses of EEG and EMG recordings to identify periods of active waking, quiet sleep (QS), and paradoxical sleep. Cardiac autonomic activity was assessed by analyzing the HRV power spectrum. Figure A illustrates the sites of optic fiber stimulation in RVLM neurons.

**Results:** Sleep interruptions occurred more frequently in Group 1 rats compared to Group 2 (Figure B). Notably, the low frequency (LF) /high frequency (HF) ratio during the QS stage significantly decreased in Group 2 compared to MI rats in Group 1 (Figure C). The RR intervals remained consistent across all sleep stages for rats in both groups (Figure D). Additionally, both HF and LF components were similar across all sleep stages in the two groups (Figures E and F).

**Conclusions:** This study found that optogenetic photostimulation effectively suppresses RVLM neuron activity, reducing sympathetic hyperactivity in MI rats and improving sleep quality. These results demonstrate optogenetic technology's potential as a novel treatment for arrhythmogenesis linked to autonomic dysfunction and disordered sleep breathing in myocardial infarction.

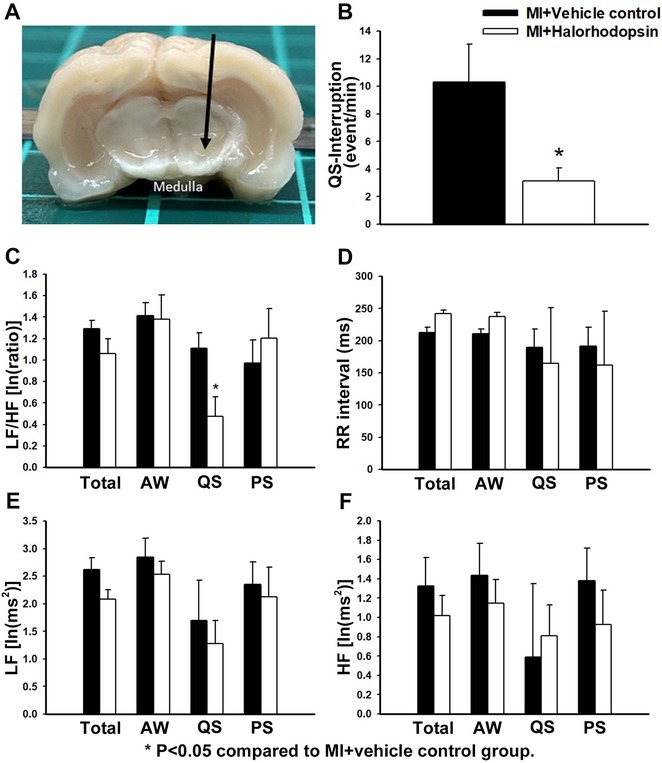

## DETECTION OF NARROW QRS TACHYCARDIA BY SINGLE LEAD ECG STRIP FROM WEARABLE DEVICES HELPS FURTHER ACTION FOR SUSPICIOUS PSVT

### **YEN‐LIANG LIN**, MIN‐TSUN LIAO

#### National Taiwan University Hospital Hsinchu Branch, Hsinchu, Taiwan

**Introduction:** Patients with PSVT may experience palpitation with short duration, resulting in difficulty to record 12‐lead ECG. It is known that wearable devices can capture single lead ECG strip during palpitation anytime, helping clinician to take further action. Here, we present two cases with single lead ECG strip detecting narrow QRS tachycardia.

**Methods:** N/A

**Results:** (Case 1) A 62‐year‐old lady had palpitation for 2 weeks, and a wearable device captured her pulse rate 140 bpm during tachycardia. The single lead ECG strip revealed regular and narrow QRS tachycardia, with retrograde P and short RP seen (figure a). The tachycardia was also terminated by a PAC. Under the diagnosis of PSVT with short RP interval, she received EP study. The EP study revealed eccentric VA conduction via CS, indicating presence of left AP. AH jump was also detected, showing existence of dual AV nodal pathway. Tachycardia could be induced with antegrade via AVN and retrograde through left AP, forming orthodromic AVRT (figure b). After ablation of left AP, another SVT could be induced after AH jump, with simultaneous HV and HA conduction, forming AVNRT (figure c). With slow pathway ablated, SVT could no longer be induced. (Case 2) A 39‐year‐old woman had palpitation for 1 year, and a wearable device detected her pulse rate 160 bpm during tachycardia. The single lead ECG strip showed regular and narrow QRS tachycardia, but no obvious P was detected (figure d). Since PSVT was suspected, she received EP study. The EP study showed total VA block and no AH jump. Rapid atrial pacing induced right atrial flutter with 2 to 1 AV conduction (figure e). Catheter ablation was performed, and the tachycardia could not be induced anymore.

**Conclusions:** Both patients brought single lead ECG strip, helping further confirmation and treatment of suspicious PSVT. Although the accuracy of diagnosing PSVT or atrial flutter has not been approved by the FDA, single lead ECG strip from wearable devices can be seen as a screening tool, providing clinician to take further action. It is recommended for further studies to evaluate the clinical benefit of wearable devices as a diagnostic tool for PSVT formally.

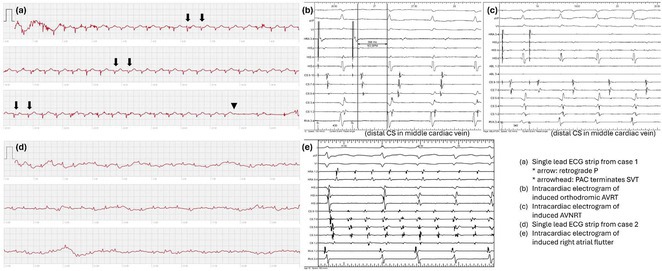

## PERFORMANCE OF THE NOVEL ANTWERP SCORE IN PREDICTING HEART FUNCTION IMPROVEMENT AFTER ATRIAL FIBRILLATION ABLATION IN ASIAN PATIENTS WITH HEART FAILURE

### **LO CHIEH LING** ^1,2^, TING‐YUNG CHANG^1,2,3^, YENN‐JIANG LIN^1,2^, CHIN‐YUN LIN^1,2^, SHIH‐LIN CHANG^1,2^, LI‐WEI LO^1,2^, YU‐FENG HU^1,2^, FA‐PO CHUNG^1,2^, SHIH‐ANN CHEN^1,2,4^

#### ^1^Taipei Veteran General Hospital, Taipei City, Taiwan,^2^National Yang Ming Chiao Tung University, Taipei City, Taiwan,^3^National Taipei University of Nursing and Health Sciences, Taipei City, Taiwan,^4^National Chung Hsing University, Taichung City, Taiwan

**Introduction:** Previous research has demonstrated that atrial fibrillation (AF) ablation improves heart function variably among patients. We hypothesized that the ANTWERP score, validated in a European cohort for assessing patients with reduced left ventricular ejection fraction (LVEF) undergoing AF ablation, would be applicable in an Asian cohort. This study aimed to investigate the ANTWERP score's performance in predicting heart failure improvement post‐AF ablation in Asian patients.

**Methods:** Previous research has demonstrated that atrial fibrillation (AF) ablation improves heart function variably among patients. We hypothesized that the ANTWERP score, validated in a European cohort for assessing patients with reduced left ventricular ejection fraction (LVEF) undergoing AF ablation, would be applicable in an Asian cohort. This study aimed to investigate the ANTWERP score's performance in predicting heart failure improvement post‐AF ablation in Asian patients.

**Results:** Similarities were observed between responders and non‐responders regarding comorbidities, AF type, and LVEF, except for the Left Ventricular Internal Diameter in Diastole (LVIDd). A higher percentage of responders had an ANTWERP score ≤ 2 (87.8%) compared to those with a score > 2 (55.6%). LVEF improvement was notably higher in the former group. Atrial reverse remodeling and recurrent atrial arrhythmia rates were similar across groups.

**Conclusions:** The ANTWERP score effectively predicts LVEF improvement post‐AF ablation in Asian patients with AF and LVEF < 50%. Patients with a score ≤ 2 demonstrated higher rates of LVEF improvement and more significant ventricular remodeling compared to those with a score > 2. This study supports the score's applicability across different ethnicities, though further research is warranted for validation.

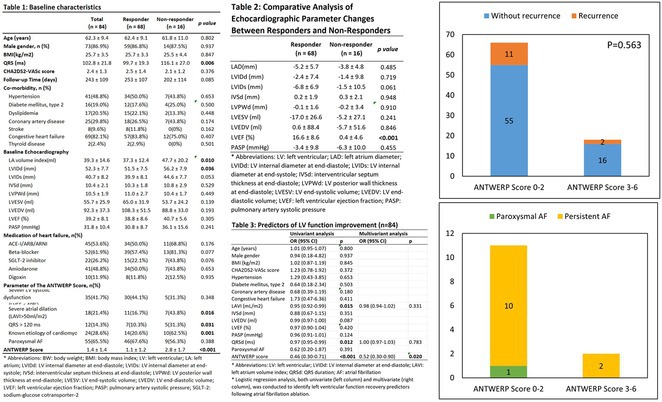

## THE VALUE OF THE AH INTERVAL DURING ATRIAL PACING AT TACHYCARDIA CYCLE LENGTH IN IDENTIFYING THE MECHANISM OF SUPRAVENTRICULAR TACHYCARDIA

### **PHAM LINH** ^1^, VU THANH^2^

#### ^1^Vietnam National Heart Institute, Hà Nội, Viet Nam,^2^vice director of Cardiac Electrophysiology department ‐ Vietnam National Heart Institute, Ha Noi, Viet Nam

**Introduction:** We aim to investigate the AH interval during supraventricular tachycardia and during atrial pacing at the tachycardia cycle length to understand the role of the differences in AH intervals in diagnosing the underlying mechanism of supraventricular tachycardias

**Methods:** Our study enrolled 41 patients diagnosed with supraventricular tachycardia who underwent electrophysiology studies to confirm the diagnosis followed by successful RF treatment at the Vietnam Heart Institute ‐ Bach Mai Hospital from July 2022 to October 2023. All patients underwent electrophysiology studies and programmed heart stimulation to induce supraventricular tachycardia. After confirming the diagnosis of the tachycardia mechanism, we carried out the atrial pacing maneuver at the same cycle length as the tachycardia, measuring AH intervals during tachycardia and during atrial pacing. These intervals were recorded on the electrogram of the His bundle

**Results:** The ∆AH index is valuable in differentiating between AVNRT and the group of AT + AVRT, with an area under the curve of 1. The selected cutoff point is 44.5ms, with sensitivity and specificity both at 100%. In distinguishing between AT and AVRT, the ∆AH index also holds its value with an area under the curve of 0.942 (p=0.008), a cutoff point of 10ms, sensitivity of 93.3%, and specificity of 100%.

**Conclusions:** Measuring the AH interval during supraventricular tachycardia and during atrial stimulation at the same rate as the tachycardia can help determine the mechanism of tachycardia such as atrial tachycardia or AVNRT or AVRT.

## CHANGING TACTICS: EARLY SINGLE CENTRE EXPERIENCE OF TACTICATH™ SE VS TACTIFLEX™ SE RF ABLATION CATHETERS FOR AF ABLATION

### SCOTT EAVES, AMELIA LUTWYCHE, JOSHUA HAWSON, RHONDA MCNEILL, **JONATHAN LIPTON**

#### Royal Hobart Hospital, Hobart, Australia

**Introduction:** Pulmonary vein isolation (PVI) via radiofrequency ablation is an effective treatment for atrial fibrillation (AF). Radiofrequency ablation combined with electroanatomical mapping is standard practice; technological advances promise improved efficacy and safety. We compared the effect of changing from the TactiCath™ Sensor Enabled Ablation (Abbott) to the TactiFlex™ Sensor Enabled Ablation Catheter (Abbott) in a single centre experience.

**Methods:** Procedural records of 135 consecutive patients undergoing pulmonary vein isolation for atrial fibrillation at the Royal Hobart Hospital from 2021‐2023 were reviewed. Tacticath™ was utilised in the first 103 patients, with Tactiflex™ having been introduced in all remaining cases. Procedural characteristics and 6‐month patient outcomes were compared.

**Results:** PVI was achieved in 100/103 (97%) of patients in the TactiCath™ group and 22/22 (100%) of patients in the TactiFlex™ group. There was no difference with regards to safety outcomes between the two groups. Left atrial dwelling time was significantly lower in the TactiFlex™ group (87 minutes, range 48‐179 vs 109 minutes, range 58‐202, p<0.005). At 6 months, there was no difference in rates of AF recurrence (11.6% vs 9.1%, p=0.72).

**Conclusions:** In our early experience, use of the TactiFlex™ catheter allows for safe and effective PVI in patients undergoing radiofrequency ablation for AF. It results in shorter procedural times when compared to use of TactiCath™ but no difference in rates of early AF recurrence.

## LATE FRACTURE OF STYLET DRIVEN LEAD IN LEFT BUNDLE BRANCH AREA PACING: A CASE STUDY

### **JONATHAN LIPTON** ^1,2,3^, JENNIFER MILLHOUSE^4^, SAM LOVIBOND^1^, JOSHUA HAWSON^1^, PAUL MACINTYRE^1^

#### ^1^Royal Hobart Hospital, Hobart, Australia,^2^University of Tasmania, Hobart, Australia,^3^Royal Melbourne Hospital, Melbourne, Australia,^4^Hobart Heart Center, Hobart, Australia

**Introduction:** Left bundle branch area pacing (LBBAP) is increasingly popular due to its physiological benefits. However, currently used leads are designed for endocardial rather than deep septal use, with limited data on medium and long‐term outcomes in this location.

**Methods:** n/a

**Results:** An 84‐year‐old female received of a dual chamber pacemaker in preparation for AV node ablation. The ventricular lead was successfully positioned in the left bundle branch area using a long sheath (Biotronik Selectra 3D 55‐39 cm) and stylet driven lead (Solia S60). She underwent AV node ablation 4 weeks later. After 6 months, she presented with sudden onset fatigue, shortness of breath, and a junctional rhythm at 30/min. Pacemaker interrogation revealed high impedance and failure of ventricular capture in both bipolar and unipolar modes. X‐ray did not indicate dislodgement or fracture. A new endocardial ventricular lead was implanted; fluoroscopy showed acute angulation at the septal hinge point of the existing lead (see Figure). Due to concerns about lead integrity, it was not removed. The lead was retrieved later after the patient's death from an unrelated cause. Manufacturer analysis confirmed a fracture at the tip hinge point. Leads positioned in an LBBAP have a different stress profile at the lead tip from the endocardial position they were designed for. Though rare (estimated at <0.1% at 2 years after implant), distal conduction fracture can occur suddenly, without warning, and have significant clinical implications, especially in pacing‐dependent patients. The incidence may be higher if the lead is exposed to torque and angulation at implant. Though evidence for the benefits of LBBAP pacing is increasing, with the technique being widely adopted, more data on long term lead performance is needed. Leads designed specifically for LBBAP may offer better performance and ease of implant.

**Conclusions:** Sudden failure of stylet driven leads in conduction system pacing is a rare complication with major clinical implications. Data on long term LBBAP‐lead performance and development of specific LBBAP leads are needed.

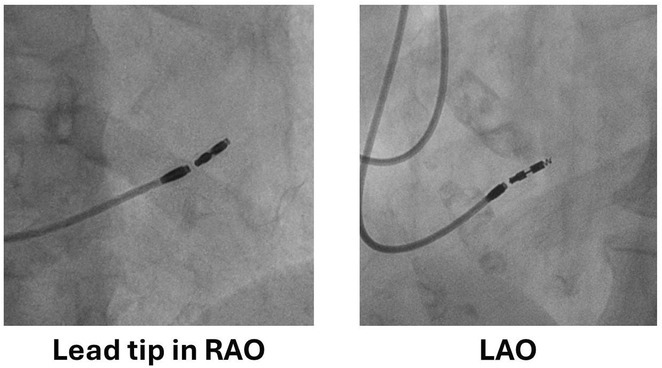

## TASMANIAN EP: THE ZERO FLUORO JOURNEY

### SCOTT EAVES, AMELIA LUTWYCHE, JOSHUA HAWSON, RHONDA MCNEILL, **JONATHAN LIPTON**

#### Royal Hobart Hospital, Hobart, Australia

**Introduction:** Radiation exposure during electrophysiology procedures can cause adverse health outcomes for patients and clinicians. Due to recent advances in electroanatomical mapping systems and use of intracardiac echocardiography, many electrophysiology procedures can now be performed safely with minimal or zero fluoroscopy. Here we present the experience of the Royal Hobart Hospital EP service zero fluoroscopy approach for supraventricular tachycardia/PVC ablation.

**Methods:** The electrophysiology database entries and electronic records of all patients who underwent an electrophysiology procedure +/‐ supraventricular tachycardia/PVC ablation at the Royal Hobart Hospital between 2019 and 2023 were reviewed. Ablations for atrial fibrillation were excluded from analysis. The average radiation dose per patient (mGy) and number of cases that were performed without the use of fluoroscopy were identified. All cases were performed by a single operator.

**Results:** Of the 424 consecutive patients that were identified, 288 (67.9%) cases were performed with zero fluoroscopy use. There was a significant decrease in the average radiation dose used per case on an annual basis: 2019, 208mGy; 2020, 45mGy; 2021, 11mGy; 2022 7mGy; 2023 2.5mGy. There was no increase in safety outcome events in the zero fluoroscopy group. Acute procedural success was comparable between the 2 groups (274/288 patients, 95% vs 124/136 patients, 91%, p = 0.12).

**Conclusions:** A zero fluoroscopy approach is safe and feasible in most electrophysiology procedures. For experienced operators, in cases where fluoroscopy is required, it is possible to minimize radiation exposure.

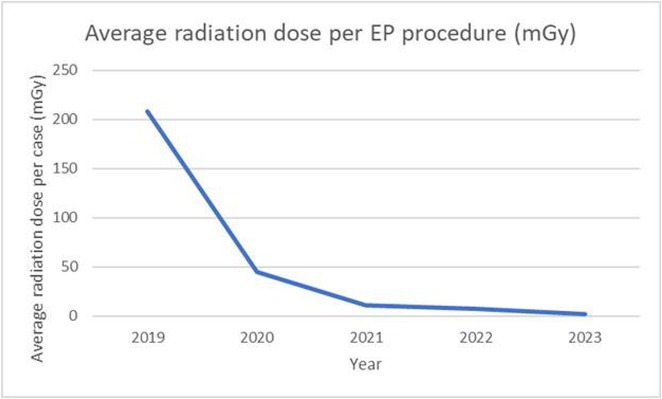

## COMPLEX FRACTIONATED ELECTROGRAMS, DISPERSIONS, AND LOW VOLTAGE AREAS IN ATRIAL FIBRILLATION: INSIGHTS FROM FOUR‐SECOND CONTINUOUS RECORDINGS

### **HAILEI LIU**, MICHAEL SHEHATA, ASHKAN EHDAIE, XUNZHANG WANG

#### Cedars‐Sinai Medical Center, Los Angeles, CA

**Introduction:** Various methodologies have been devised to identify critical regions in atrial fibrillation (AF) mapping, including Complex Fractionated Electrograms (CFEs), dispersion areas, and low voltage areas (LVAs). However, the dynamics of local activations in the left atrium evolve over time, and the interrelationship among these factors remains incompletely understood.

**Methods:** From March 1^st^ to April 14^th^, 2024, consecutive patients with AF undergoing catheter ablation were prospectively recruited at a single center. Inclusion criteria encompassed spontaneous or induced AF prior to ablation. High‐density catheters were utilized to map the left atrium, with each point recorded continuously for at least four seconds, totaling 2000 points. Following ablation, remapping of the left atrium was performed in cases of persistent or induced AF. The left atrium was divided into six segments: anterior wall, left atrial appendage, lateral wall, inferior wall, posterior wall, and septum. CFEs were characterized as atrial electrograms with a very short cycle length (≤ 150 ms). Dispersion points were automatically annotated using Volta, a proprietary AI based software, and LVAs were defined as < 0.1 mV bipolar voltage during AF.

**Results:** A total of 10 patients (9 males, mean age 63.7±11.9 years, 5 with persistent AF) were included, with mean mapping points of 3672.4±898.8. All dispersion points were located within CFE areas. The majority of CFE areas exhibited voltages exceeding 0.5 mV (94.6%±12.0%), whereas most non‐CFE areas demonstrated voltages below 0.5 mV (90.4%±19.4%). Four patients underwent post‐ablation mapping, revealing a significant reduction in both CFE areas and dispersion points, even in areas without ablation lesions.

**Conclusions:** CFE areas predominantly exhibit normal voltage and correlate closely with dispersion areas over a relatively prolonged mapping duration. Both CFEs and dispersion regions do not necessarily represent the targets for ablation, and further studies are warranted to distinguish passive from active areas of interest.

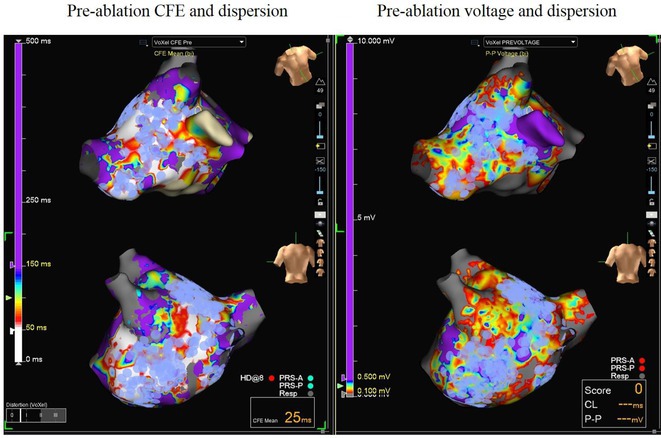

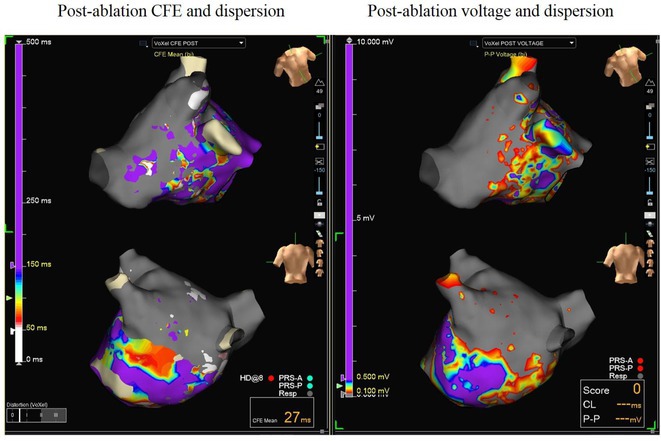

## ARRHYTHMIA INCIDENCE ON ECG MONITORING POST PERCUTANEOUS CORONARY INTERVENTION (PCI) ‐ A SINGLE CENTRE RETROSPECTIVE STUDY

### **JUSTIN LIU**, SHONDA NG, XUYAN TEOH

#### Tan Tock Seng Hospital, Singapore, Singapore

**Introduction:** Guidelines recommend routine continuous ECG monitoring post PCI. However, studies show that actionable arrhythmia alarms (AAs) detected during this period is low. Some studies propose same day discharges and shorter monitoring especially in elective PCI cases. Our study aims to report the incidence of AAs post PCI and identify risk factors.

**Methods:** We included patients who had ECG monitoring post PCI in our hospital, and described baseline demographics, PCI indications, coronary anatomy, management, ECG monitoring duration and AAs detected. AAs were defined as 1) ≥ 3 seconds pause; 2) high‐grade atrioventricular (AV) block or complete heart block; 3) ventricular fibrillation (VF); 4) ventricular tachycardia (VT) >15 beats; 5) atrial fibrillation with rapid ventricular response (AF with RVR); 6) supraventricular tachycardia> 15 beats. Using SPSS, categorical data was analysed with Fisher's exact and Chi‐Square tests. Student's t‐test was used for continuous data.

**Results:** We included 475 patients (Mean age 61.2 ± 11.7, Male 85.3%) of which 8 (1.7%) had AAs. Of the AAs, 2 (25%) were high grade AV block, 2 (25%) were VT or VF, and 4 (50%) were AF with RVR. PCI indications included acute coronary syndromes (ACS) (Unstable angina (7.8%), NSTEMI (30.9%), STEMI (41.9%)) and stable ischemic heart disease (Stable angina (7.2%), ischemic cardiomyopathy (4.8%) and positive stress test (7.4%)). Coronary anatomy was grouped into single vessel disease (39.4%); multivessel disease (54.7%); and left main (LM) disease (5.9%). Mean ECG monitoring duration was 2 ± 1.1 days. LM disease was significantly associated with more AAs (p=0.037). All AAs occurred in ACS patients. Patients with AAs had a longer period of ECG monitoring (p=0.024). There was no significant difference between patients who underwent full or partial revascularization.

**Conclusions:** Further studies can be done in more patients to assess need for routine ECG monitoring post PCI, especially in non ACS patients with no LM disease. This may aid targeted monitoring of high risk patients and improve resource allocation, cost effectiveness and shorten length of hospital stay.

## EARLY DETECTION OF ELECTROMECHANICAL DYSFUNCTION USING ACOUSTIC CARDIOGRAPHY IN VASOVAGAL SYNCOPE PATIENTS

### **SHIN HUEI LIU**, SHIH‐LIN CHANG, TING‐AN LEE, YENN‐JIANG LIN, LI‐WEI LO, YU‐FENG HU, FA‐PO CHUNG, CHIN‐YU LIN, TING‐YUNG CHANG, LING KUO, CHIH‐MIN LIU, CHENG‐I WU, SHIH‐ANN CHEN

#### Taipei Veteran General Hospital, Taipei, Taiwan

**Introduction:** Early diagnosis for syncope is challenging and essential to prevent traumatic injuries. We aimed to observe serial cases using acoustic cardiography in syncope to identify potential parameters that differentiate subgroups.

**Methods:** A case series of 7 syncope patients was included for preliminary investigation. The tilting table test (TTT) was performed under isoproterenol (ISO) infusion to provoke vasovagal syncope (VVS). Acoustic cardiography (AUDICOR, Inovise Medical) was applied on syncope patients during TTT. The ISO infusion was administrated consecutively at 1, 2, and 3 μg/min doses. The TTT was stopped if the patient developed syncope or intolerable symptoms. Cardiac parameters from the acoustic cardiography were investigated.

**Results:** Seven syncope patients were admitted to the hospital through the emergency department (Figure 1, 2). All patients had normal left ventricular systolic function, normal blood tests, and negative findings on the brain image. The EMAT in the negative patients (purple & orange line) did not reveal a reduction, whereas the VVS patients revealed a decrease before ISO infusion (Figure 1A). The EMAT coefficient variation revealed a trend of elevation in the negative patients (purple & orange line), whereas the VVS revealed a reduction after the ISO infusion (Figure 1B). The youngest VVS patient (red line) revealed compensation for EMAT after ISO infusion, followed by a steep decline before the syncope event (Figure 1A). Patients older than 55 revealed comparable LVST patterns during the TTT regardless of syncope events (Figure 2A). The youngest VVS patient (red line) revealed a significant LVST increase after ISO infusion (Figure 2A). The LVST coefficient variation pattern revealed comparable among the VVS patients.

**Conclusions:** The VVS patients had a low coefficient of variation in EMAT and a high coefficient of variation in LVST. Acoustic cardiography might identify the mechanism of syncope and differentiate the underlying subgroups among syncope, further facilitating risk stratification and management.

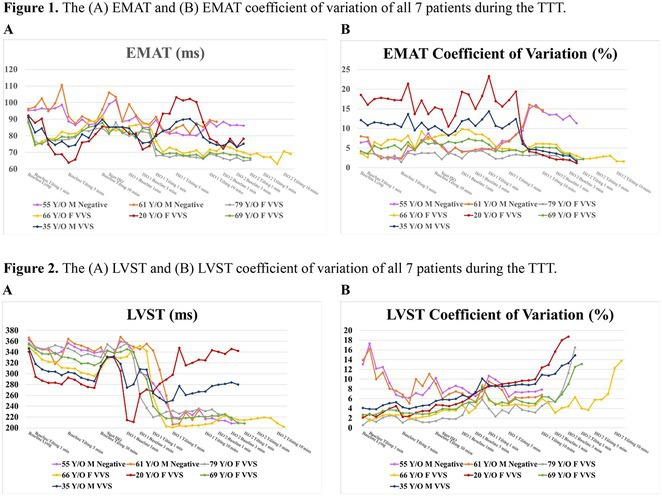

## IMPACT OF SODIUM‐GLUCOSE COTRANSPORTER 2 INHIBITORS ON ARRHYTHMOGENIC REMODELING IN CARDIORENAL SYNDROME

### **SHIN HUEI LIU**, LI‐WEI LO, YU‐HUI CHOU, WEI‐LUN LIN, SHIH‐ANN CHEN

#### Taipei Veterans General Hospital, Taipei City, Taiwan

**Introduction:** Cardiorenal syndrome (CRS) is demonstrated with cardiac structural and electrical remodeling. Sodium‐glucose cotransporter‐2 inhibitors (SGLT2i) have reduced cardiovascular (CV) events in heart failure (HF). The efficacy of SGLT2i in controlling arrhythmias in CRS remained questionable. We aimed to investigate the utility of SGLT2i on arrhythmogenicity in CRS rabbits.

**Methods:** Eighteen New Zealand white rabbits (4 kgs) were randomized into the sham (n=6), CRS (n=6), and CRS‐SGLT2i (n=6) groups. The rabbits in the CRS and CRS‐SGLT2i groups were created by the 5/6 nephrectomy. The CRS‐SGLT2i groups received oral dapagliflozin 1 mg/kg/day for 4 weeks. All rabbits received an echocardiogram, blood samples, and cardiac electrophysiology study. Myocardium was harvested for histological stain.

**Results:** The HF and renal failure were confirmed in the CRS and CRS‐SGLT2i groups. The RR interval in the CRS group was significantly longer than in the sham and CRS‐SGLT2i groups (Figure A). The effective refractory period (ERP) of all cardiac chambers in the CRS‐SGLT2i group revealed a trend of shorter ERPs than those in the CRS group (Figure B). The CRS group had significantly higher ventricular arrhythmia inducibility than the sham (32.00±10.95 vs. 0.00±0.00 %, p<0.001) and CRS‐SGLT2i (32.00±10.95 vs. 1.12±0.34 %, p<0.001) groups, respectively. The CRS group had a significantly larger fibrotic tissue area than the sham and CRS‐SGLT2i group (Figure C). The CRS‐SGLT2i group had a significantly larger fibrotic tissue area than the sham group (Figure C). Figure D demonstrates the collagen deposition (blue) in the myocardium in the 3 groups.

**Conclusions:** In a CRS rabbit model, SGLT2i demonstrated a potential antiarrhythmic effect, evidenced by attenuated VA inducibility and reversal of cardiac structural remodeling. Further studies are required to elucidate the comprehensive role of SGLT2i in managing CRS‐associated arrhythmias.

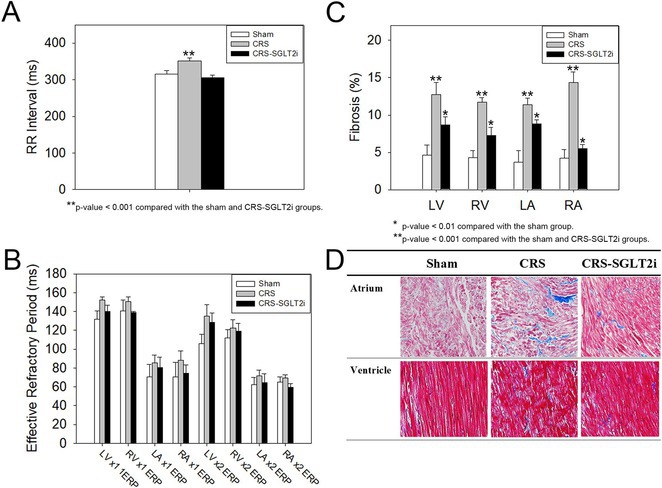

## LOW‐LEVEL SPINAL CORD STIMULATION ALTERS CARDIAC NEUROELECTRICAL ACTIVITY IN ISCHEMIC CARDIOMYOPATHY

### **SHIN HUEI LIU** ^1^, LI‐WEI LO^1^, YU‐HUI CHOU^1^, WEI‐LUN LIN^1^, TING‐AN LEE^1^, CHING‐WEN CHANG^2^, YOU‐YIN CHEN^2^, SHIH‐ANN CHEN^1^

#### ^1^Taipei Veterans General Hospital, Taipei City, Taiwan,^2^National Yang Ming Chiao Tung University, Taipei City, Taiwan

**Introduction:** Spinal cord stimulation (SCS) demonstrated autonomic nervous system (ANS) remodeling influencing arrhythmias in ischemic cardiomyopathy (ICM). The potential impact of ANS modulation through SCS remained unclear. We aimed to explore the neuromodulatory effects of SCS in ICM rats.

**Methods:** Eight Sprague‐Dawley rats were randomized into sham (n=4) or ICM (n=4) groups. The ICM model was created by coronary artery ligation. The SCS was applied by inserting a custom‐made probe into the epidural space. All rats underwent SCS at T‐spine levels 2‐3 and 3‐4, following protocols of 30 Hz/225μs/200μA and 30 Hz/225μs/300μA. Heart rate variability (HRV) was investigated.

**Results:** All rats with ICM were confirmed to have heart failure. Figures 1 and 2 demonstrated a significant alteration in RR intervals during SCS‐On and SCS‐Off in the sham group, whereas the ICM group revealed an attenuated response. In both the sham and ICM groups, RR interval changes at T‐spine 2‐3 exhibited a relatively faster response to SCS than T‐spine 3‐4 (Figure 1, 2). Under SCS‐On at 200μA and 300μA, the LF in the ICM groups was significantly higher than in the sham groups (Table 1). In the ICM groups under SCS‐Off at 200μA and 300μA, the LF was significantly lower than that under SCS‐On (Table 1). In the ICM groups under SCS‐Off at 200μA and 300μA, the LF/HF was significantly lower than under SCS‐On (Table 1). In the ICM group under SCS‐Off at 300μA, the LF was significantly lower than under SCS‐On (Table 2). In the ICM groups under SCS‐Off at 200μA and 300μA, the HF was significantly higher than that under SCS‐On (Table 2). The LF/HF in the ICM groups under SCS‐Off at 200μA and 300μA was significantly lower than that under SCS‐On (Table 2).

**Conclusions:** Low‐level SCS at the T‐spine exhibited a gradual sympathetic suppression in ICM rats at both T‐spine 2‐3 and 3‐4. After the cessation of SCS, the sympathetic suppression endured in ICM rats, accompanied by an augmentation in parasympathetic tone, indicating an ongoing ANS remodeling despite the brief duration of SCS. These findings underscore the need for additional investigations to elucidate and optimize the protocols for SCS.

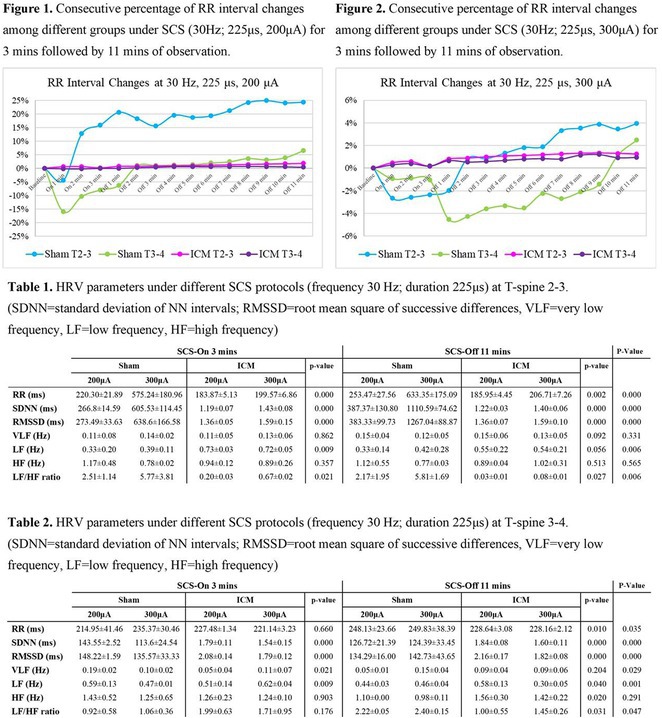

## HEARTBEATS IN SYNC: OPTIMIZING HEART FAILURE TREATMENT WITH RHYTHM CONTROL STRATEGY FOR ATRIAL FIBRILLATION AND ATRIAL FLUTTER

### **CHIA LIUQING**, FOO JHI HUI, LIM LEE YEE, LAW DE ZHI

#### Department of Cardiology, Hospital Queen Elizabeth II, Kota Kinabalu, Malaysia

**Introduction:** Atrial fibrillation (AF) affects the worldwide population which is associated with morbidity particularly stroke and increased mortality rate. Debates regarding rhythm control versus rate control in AF patients had been ongoing for decades and in recent years there is more evidence supporting rhythm control with catheter ablation among AF patients.

**Methods:** N/A

**Results:** A 55 year old man had a history of admission for heart failure in which he was found to have reduced left ventricular ejection fraction (LVEF) of 20‐25% and coronary angiogram showed no significant coronary artery disease. In the same setting, he was found to have atrial flutter (figure 1A) and atrial fibrillation (figure 1B) with mildly dilated left atrium. He was treated with main pillars of heart failure therapy and scheduled for ablation with Marshall plan. Upon elective admission for the procedure, he was not in failure, normotensive with heart rate of 90 beats per minute.

The procedure was performed under general anesthesia started with direct PA electrical cardioversion which revert the rhythm to sinus, followed by vein of Marshall alcohol ablation, then pulmonary vein isolation. After successful right and left pulmonary veins isolation with demonstrated exit block, eccentric atrial flutter with tachycardia cycle length (TCL) of 400ms was induced during coronary sinus (CS) pacing.

Ablation done over lateral mitral isthmus line and anterior left atrium wall but was unable to terminate the tachycardia. Right atrium mapping done which showed two re‐entrant tachycardia at Crista terminalis and cavotricuspid isthmus and ablation was performed. Tachycardia persist despite ablation performed over both atria with potential of epicardial connection. Direct PA electrical cardioversion was performed again and able to revert back to sinus rhythm (figure 1C).

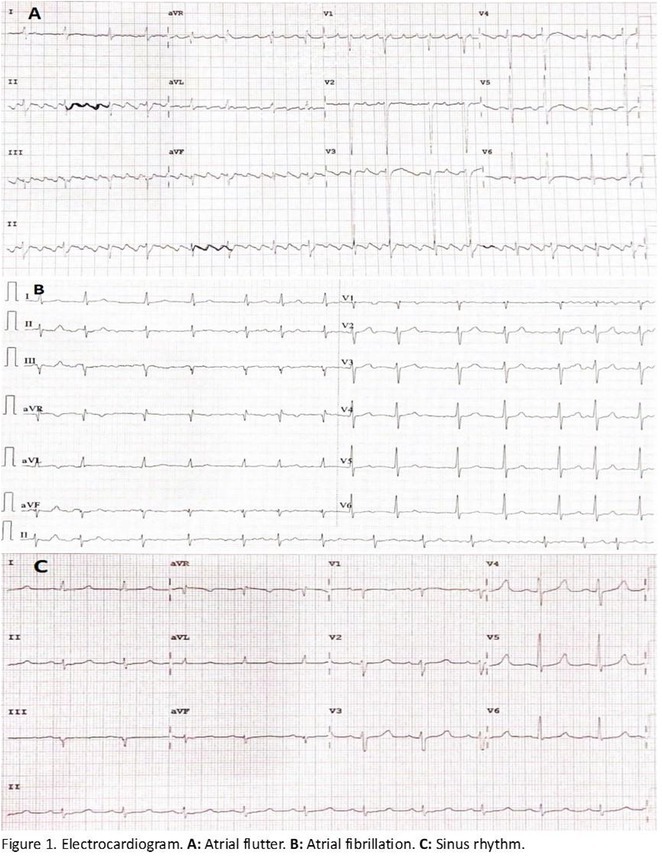

**Conclusions:** Alcohol ablation of the vein of Marshall is a therapeutic option for atrial tachyarrhythmias. The addition of this technique to the common practice of pulmonary vein isolation is feasible in persistent AF cases. More studies are required for further demonstrating clinical outcomes.

USE OF PEAK FREQUENCY AND EMPHASIS MAPPING IN IDENTIFYING GAPS ALONG PULMONARY VEIN ISOLATION LINE DURING ATRIAL FIBRILLATION PULSE FIELD ABLATION

**HUI TING STEPHANIE LOH** ^1^, XUANMING PUNG^1^, CHEONG KIAT JULIAN TAY^1^, WEI SHENG JONATHAN ONG^1^, JIE MIN GERMAINE LOO^1^, TIEN SIANG ERIC LIM^1^, CHUN YIH PAUL LIM^1,2^, CHI MING KELVIN CHUA^1,3^, KAH LENG HO^1^, THUAN TEE DANIEL CHONG^1^, WEE SIONG TEO^1,2^, CHI KEONG CHING^1^;

^1^National Heart Centre Singapore, Singapore, Singapore,^2^Mount Elizabeth Hospital and Parkway East Hospital, Singapore, Singapore,^3^Mount Elizabeth Hospital and Gleneagles Hospital, Singapore, Singapore.

**Introduction:** Peak frequency mapping serves to differentiate near field from far field electrograms in substrate characterisation, but its use in atrial fibrillation (AF) ablation has not been much described. In 2004, Pachon et al applied a similar approach using spectral analysis through the fast Fourier transforms (FFT) that went beyond time domain to frequency domain of the atrial potentials to target AF nests. In this case, intraprocedural peak frequency derived emphasis mapping was particularly useful in delineating gaps through local conductivity across pulmonary vein isolation line after initial ablation.

**Methods:** N/A

**Results:** A 77‐year‐old lady with a background of prior myocardial infarction, atrial septal defect status post surgical closure, and paroxysmal atrial fibrillation (AF) with tachy‐brady syndrome had recurrent hospital admissions related to worsening AF control despite treatment with amiodarone. She underwent an invasive electrophysiology study (EPS) and AF catheter ablation. Intraprocedural, left atrial omnipolar voltage map was generated using the EnSite Precision Mapping System and HD‐Grid catheter. When mapping the right superior pulmonary vein (RSPV), spontaneous and early activity leading to atrial fibrillation was noted. This was likely the arrhythmogenic vein. Pulsed field ablation with a Farawave 31mm catheter was performed. During isolation of RSPV, first application of PFA terminated her atrial fibrillation following this application. No further AF runs were seen after isolation of all 4 veins. All veins were remapped. On peak frequency and emphasis mapping, left superior pulmonary vein (LSPV) appears to have a connection over both posterior carina (Gap 1) and roof (Gap 2) areas. Targeted ablation was done and LSPV was then isolated.

**Conclusions:** Peak frequency and emphasis mapping may identify gaps along pulmonary vein isolation line.

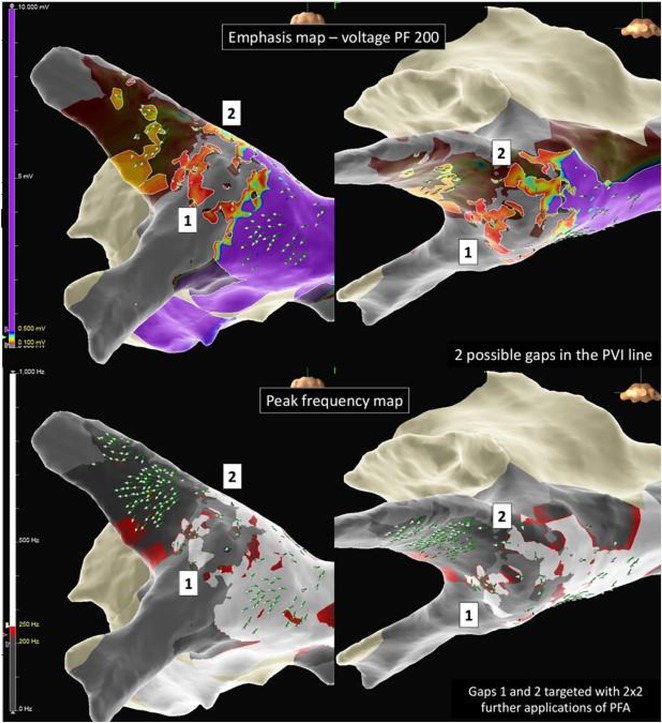

## PROGNOSTIC VALUE OF THE MODIFIED MELD SCORE IN PATIENTS TREATED WITH CARDIAC RESYNCHRONIZATION THERAPY

### **TIANXIN LONG**, YU YU, WEI HUA

#### Fuwai hospital, Chinese Academy of Medical Sciences, Beijing, China

**Introduction:** We aimed to assess the prognostic value of the Modified Model for End‐Stage Liver Disease (MELD‐XI and MELD‐albumin) score, assessing hepatorenal function, in patients undergoing cardiac resynchronization therapy (CRT).

**Methods:** We retrospectively evaluated 365 patients (mean age 58.7±11.1 years; 64.9% men) undergoing CRT implantation between 2007 and 2019. Patients were divided into four groups based on the MELD‐XI and MELD‐Albumin score quartiles before CRT. The primary endpoint was the combination of all‐cause mortality and HF hospitalization, whereas the secondary endpoint was CRT response at 6 months.

**Results:** During mean follow‐up of 3.3 (IQR: 1.9‐5.2) years, 168 patients reached the primary endpoint. Logistic regression revealed the MELD‐Albumin score were independently associated with CRT response, even after adjusting for covariates (adjusted OR 1.10; 95% CI, 1.02‐1.19; P= 0.013). Kaplan‐Meier analysis revealed patients with a higher MELD‐XI and MELD‐Albumin score had a greater risk of adverse outcomes (log‐rank test: P < 0.001). A Cox proportional hazard analysis showed that the modified MELD score remained significantly associated with the combined endpoint after adjusting for clinical and echocardiographic factors (MELD‐XI: HR 1.06; 95% CI 1.02‐1.11; P=0.006; MELD‐Albumin: HR 1.10; 95%CI 1.05‐1.16; P<0.001). Furthermore, ROC analysis indicated that the MELD‐Albumin score provided a stronger prognostic value for long‐term clinical outcomes in patients undergoing CRT than MELD‐XI (MELD‐Albumin: AUC 0.692, 95%CI 0.644‐0.742, MELD‐XI: AUC 0.659, 95%CI 0.608‐0.715, P=0.008).

**Conclusions:** The MELD‐Albumin score may be useful for stratifying patients at risk for CRT response and adverse outcomes in those undergoing CRT for HF.

## CATHETER ABLATION WITH HIGH DENSITY ELECTROANATOMICAL MAPPING FOR ATYPICAL ATRIAL FLUTTER ‐ AN ASIAN EXPERIENCE

### **GERMAINE, JIE MIN LOO**, XUANMING PUNG, JULIAN, CHEONG KIAT TAY, HOOI KHEE TEO, KAH LENG HO, ERIC, TIEN SIANG LIM, DANIEL, THUAN TEE CHONG, CHI KEONG CHING

#### National Heart Center Singapore, Singapore, Singapore

**Introduction:** Atypical atrial flutter (AFL) ablation is often technically challenging. The utilization of novel high‐density three dimensional mapping systems may improve procedural success. We sought to describe the clinical and procedural characteristics of patients who underwent atypical AFL ablation using high density electroanatomical mapping system.

**Methods:** This was a retrospective analysis on consecutive patients with atypical AFL who underwent radiofrequency ablation using the EnSite Precision^TM^ system and HD Grid^TM^ mapping catheter (Abbott, Chicago, IL) between March 2022 to January 2024 in a tertiary hospital in Asia. The clinical characteristics, procedural details and follow up data were collected.

**Results:** A total of 21 patients underwent atypical AFL ablation using high density mapping. The mean age was 59.6 ± 15.5 years, and patients were predominantly male (61.9%). Ten patients (47.6%) had prior cardiac surgery and 15 patients (71.4%) had previous ablation for atrial fibrillation or AFL. The median duration from onset of AFL to ablation was 15 months (IQR 4‐33). A total of 31 AFL circuits were induced. The most common atypical AFL location was scar/incisional related circuits (36.4%), followed by upper loop re‐entry (19%) and perimitral flutters (19%) (Table 1). Acute procedural success, defined as non‐inducibility, was achieved in 20 patients (95.2%). One patient (4.8%) suffered from vascular complication requiring angioembolisation one week post ablation. After a median follow up duration of 10 months (IQR 5‐20), AFL recurred in 2 patients (9.5%).

**Conclusions:** Catheter ablation with high density electroanatomic mapping systems for atypical AFL showed good acute procedural success rate with low complication and short term recurrence rates.

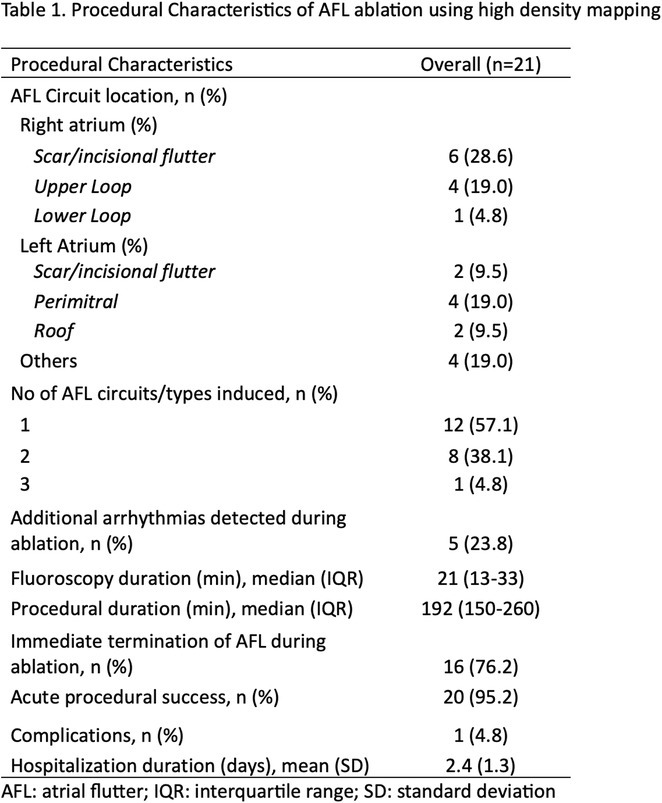

## FIRST EXPERIENCE WITH THE STEERABLE MOTORIZED XTRACTOR MECHANICAL LEAD EXTRACTION TOOL FOR REMOVAL OF PACEMAKERS AND DEFIBRILLATOR LEADS

### **CHARLES LOVE** ^1^, RAYMOND SCHAERF^2^, THOMAS CALLAHAN^3^, VIVEK REDDY^4^, BENNY RUSSO^5^, NAAMA WEINTRAUB^6^

#### ^1^Johns Hopkins Hospital and School of Medicine, Baltimore, MD,^2^Cedars Sinai Medical Center, Los Angeles, CA,^3^Cleveland Clinic Foundation, Cleveland, OH,^4^Mount Sinai School of Medicine, New York, NY,^5^Xcardia Inovation Ltd, Rehovot, Israel,^6^Xcardia Innovations Ltd, Rehovot, Israel

**Introduction:** Conventional lead extraction procedures for cardiovascular implanted electronic devices face challenges due to fibrotic and calcified tissue adherence. The Xtractor™ device (Xcardia) is a novel, motorized mechanical tool designed to address some of the existing challenges. It features a flexible distal segment with steering capabilities, an advanced mechanical tip mechanism and motorized activation, offering feedback and enhanced control without hand fatigue.

**Methods:** Procedures were performed at 4 medical centers experienced in lead extraction, in hybrid operating rooms, under fluoroscopic guidance. A standard, commercially available locking stylet was used during lead preparation. The Xczrdia device was then advanced over the lead along the venous path towards the heart, carefully navigated from the pocket entry. A stepwise approach for switching between extraction tools was used in some cases per the operator's discretion. Technical and procedural information from these procedures was collected.

**Results:** 60 leads were attempted in 34 procedures, 25% indicated for infection. Five cases were performed from the right side. Mean lead age was 13.4 years (up to 26 years old). There were 39 pacing leads, 7 single‐coil and 14 dual‐coil ICD leads, 22 leads were located in the RA, 33 in RV and 5 in LV. 97% of the cases were successful. There was 1 major complication that required an unplanned surgical intervention. In 5 cases a femoral snare or pocket entry tools were first used. Xtractor was the first full length sheath attempted in 51 of the 60 leads. A mean of 1.9 tools (ranging 1‐5) were used per procedure. When more than one tool was needed, a mean of 3.0 tools were used. For leads up to 12 years old (mean age 8.0 y), 25 leads were attempted to be extracted with Xtractor, with 84% of them extracted by Xtractor alone. Mean estimated fluoroscopy time per lead: 6.5 min.

**Conclusions:** The Xtractor device is an effective electrically powered mechanical tool for the removal of chronically implanted leads. Results are similar to those when other extraction devices are used.

## DUAL CHAMBER PACEMAKER IMPLANTATION IN A PATIENT WITH PERSISTENT LEFT SUPERIOR VENA CAVA POST‐MODIFIED WARDEN PROCEDURE FOR PARTIAL ANOMALOUS PULMONARY VENOUS DRAINAGE: A CASE REPORT

### **MING YOONG LOW**, SURINDER KAUR KHALAE, SURAYA HANI KAMSANI, ROHITH STANISLAUS, AZLAN HUSSIN

#### National Heart Institute, Kuala Lumpur, Malaysia

**Introduction:** Traditional and modified Warden procedures are performed in patients with partial anomalous pulmonary venous drainage (PAPVD), with a 20% rate of sinus node dysfunction. The incidence of persistent left superior vena cava (PLSVC) in patients with congenital heart disease is approximately 10%. Owing to the complex venous anatomy, implantation of a left‐sided dual chamber pacemaker in these patients can be challenging. An alternate approach is a right‐sided implant; however, this should be done with caution in patients post‐modified Warden's as the superior vena cava is anastomosed to the right atrial appendage.

**Methods:** N/A

**Results:** We report the case of a 48‐year old who was referred to our unit for tachycardia‐bradycardia syndrome. She has a PAPVD detected incidentally on CT Thorax during workup for tuberculosis. Her right pulmonary vein inserts into the lower superior vena cava near the junction with the right atrium and she also had a sinus venosus atrial septal defect with left to right shunt. A modified Warden procedure and ASD closure was performed. During surgery, she was incidentally noted to have PLSVC. Post‐surgery, she unfortunately developed tachycardia‐bradycardia with significant pauses, necessitating dual chamber pacemaker implantation. We initially performed a right‐sided venogram to assess the venous anatomy of the right upper limb, which revealed stenosis at the anastomosis site. A left upper limb venogram confirmed a PLSVC. Fearing the risk of causing damage to the anastomotic site, the decision to implant a left sided pacemaker, through the PLSVC, was made. The right ventricular lead was looped in the right atrium, passed into the right ventricle and placed at the RV apical region. The atrial lead was placed at the right atrium free wall region. We used active fixation for both her leads. Her parameters were unremarkable and she was discharged well the next day.

**Conclusions:** This case illustrates the difficulty faced during implantation of a dual chamber pacemaker in a patient with PLSVC after modified Warden procedure for PAPVD.

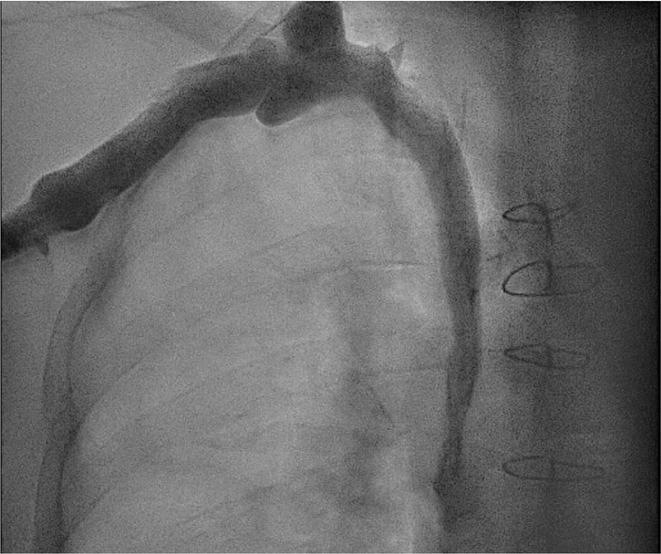

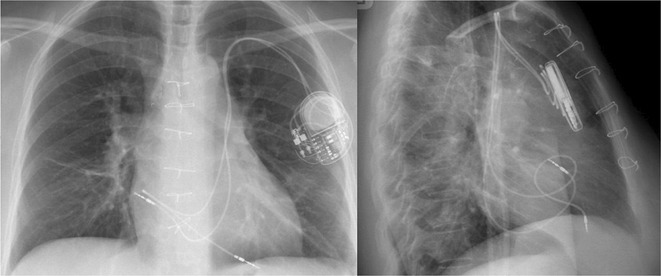

## PULSED FIELD ABLATION IN A DIFFICULT AND SMALL RIGHT INFERIOR PULMONARY VEIN: A CASE REPORT

### **MING YOONG LOW**, SURINDER KAUR KHALAE, SURAYA HANI KAMSANI, ROHITH STANISLAUS, AZLAN HUSSIN

#### National Heart Institute, Kuala Lumpur, Malaysia

**Introduction:** Pulsed field ablation is a relatively novel form of energy used in isolating the pulmonary veins in the treatment of atrial fibrillation. Compared to its predecessors, it has relatively fewer complication rates and shorter procedural times, with similar efficacy rates^1^.

**Methods:** N/A

**Results:** We report a 62‐year old lady, who presented to us with tachycardia‐induced cardiomyopathy and subsequently underwent pulsed field ablation using the FARAPULSE system. Ablation of the left upper and lower, as well as the right upper pulmonary vein was uneventful using the FARAWAVE^TM^ catheter through a FARADRIVE sheath, in both the flower and basket configurations. However, ablation of the right lower pulmonary vein proved to be difficult, owing to its small caliber. There was difficulty achieving a good seal with the FARAWAVE^TM^ catheter, in both ball and flower configurations when the conventional method was used, and this persisted even after we re‐punctured trans‐septally to obtain a more anterior location. With the catheter in a more anterior location, we decided to pass a guidewire deep into the pulmonary vein use this as a hinge to deliver first the FARADRIVE sheath in a “reverse‐hockey” configuration, and then the FARAWAVE^TM^ catheter. The right lower pulmonary vein was eventually ablated successfully via this method. All four pulmonary veins demonstrated entrance and exit blocks. Patient did not suffer any additional complications and was discharged the following day.

**Conclusions:** This case illustrates the difficulty faced during pulsed field ablation of atrial fibrillation in patients with small caliber pulmonary veins, as well as one of the methods employed to overcome this.

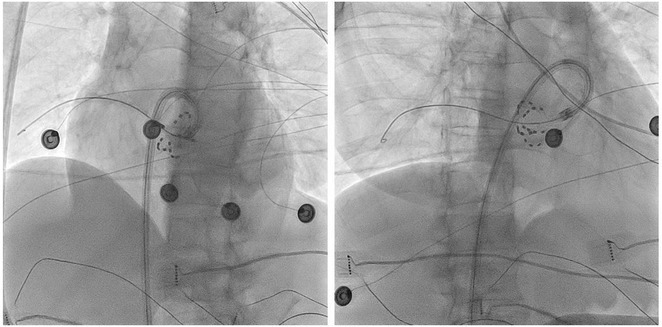

## NT‐PRO BNP IMPROVEMENT AFTER CONDUCTION SYSTEM PACING IMPLANTATION IN PATIENTS WITH ATRIAL FIBRILLATION AND SYMPTOMATIC HEART FAILURE. A CASE SERIES

### **ANGGIA LUBIS** ^1,2^, AHMAD HANDAYANI^3^, MIRHANSYAH HUTASUHUT^1,2^, HANA FAUZIYAH^1^, ALDY RAMBE^1^, YUNITA PANE^1^, MUHAMMAD MUNAWAR^4^, SUNU RAHARJO^4^, MUHAMMAD RUSDA^1^, MUSTAFA MAHMUD AMIN^1^

#### ^1^Faculty of Medicine, Universitas Sumatera Utara, Medan, Indonesia,^2^Adam Malik Hospital, Medan, Indonesia,^3^Universitas Muhammadiyah Sumatera Utara, Medan, Indonesia,^4^Universitas Indonesia, Jakarta, Indonesia

**Introduction:** Atrial fibrillation (AF) and Heart Failure (HF) are commonly co‐exist as they share mutual risk factors. However, management strategy in this situation is challenging. Current HF guidelines did not provide any class I recommendation in patients who remained symptomatic after HF therapies optimization. Pace and ablate strategy are one of the options recommended in the latest guideline with class IIb recommendation, however on the heels of conduction system pacing evolution renewed the enthusiasm for pace and ablate strategy in this setting. We presented 3 cases with AF and HF managed with pace and ablate strategy and tried to evaluate NT‐pro BNP improvement in all cases.

**Methods:** N/A

**Results: Case 1** A 71‐year‐old male with chronic HF and AF remained symptomatic despite medication. A single‐chamber pacemaker with left‐bundle branch pacing (LBBP) was implanted, leading to significant clinical, echocardiogram and NT‐pro BNP improvement at the 3‐month follow‐up. **Case 2** A 57‐year‐old male with permanent AF and heart failure with reduced ejection fraction remained symptomatic despite multiple hospitalizations, including one episode with cardiogenic shock, even after optimizing heart failure medication. An LBBP single‐chamber pacemaker was implanted, leading to clinical improvement.**Case 3**A A 78‐year‐old woman with known coronary artery disease, AF, and symptomatic heart failure came in with slow AF and unstable hemodynamics. After ruling out any reversible causes, it was decided to implant a pacemaker. A single chamber left bundle branch pacing approach was successfully implanted without any complications, leading to a significant clinical improvement and a marked decrease in NT‐pro BNP levels.

**Conclusions:** The emergence of conduction system pacing brings the pace and ablate strategy back into consideration in patients with AF and HF, as proven by the NT‐pro BNP improvement in all three cases with different scenarios. Moreover, this improvement also translated to better clinical conditions in this series.

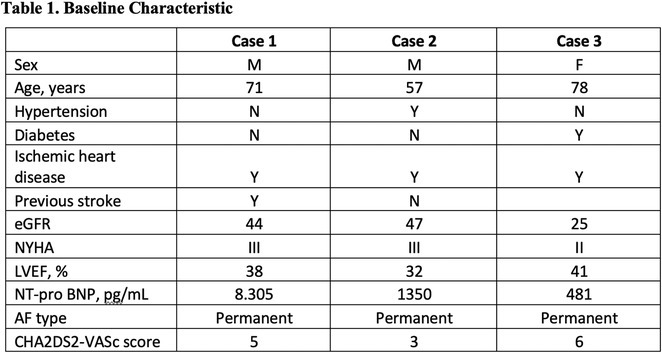

## DUAL DEVICE (S‐ICD AND PACING DEVICE) MANAGEMENT IN OUR INSTITUTE

### **KEI MABUCHI**, YOSUKE HAYASHI, SO ASANO, HIROSHI FUKUNAGA, KANKI INOUE, YUKIO SEKIGUCHI, JUNICHI NITTA, MITSUAKI ISOBE

#### Sakakibara Heart Institute, Tokyo, Japan

**Introduction:** Since the subcutaneous implantable cardioverter‐defibrillator (S‐ICD) has been available in Japan since February 2016, the consideration of managing it in combination with other devices has emerged. Subsequently, the term "dual device" is defined as two devices implanted in the same patient.

**Methods:** We retrospectively identified 161 consecutive patients who received S‐ICD implantation in our institute between February 2016 and December 2021. We analyzed 32 patients (20%) who were implanted not only S‐ICD but also transvenous device.

**Results:** The 32 patients (75% male) were aged 54.5 ± 20.0 years. When it would be appropriate to consider dual devices, it could be categorized into two scenarios. First, when an existing transvenous device is already implanted and further consideration of S‐ICD is needed. Secondary, when transvenous pacing devices become necessary after S‐ICD implantation. The most common reason of dual device management was lead breakage of a shock lead (13 cases, 41%). These patients required pacing function, so the transvenous ICD (TV‐ICD) and CRT‐D (cardiac resynchronization therapy‐ defibrillator) was downgraded to pacemaker (PM) and CRT‐P (CRT‐ pacemaker). In nine cases (28%), ventricular arrhythmia was detected after PM implantation, and S‐ICD was selected based on discussions. There were six cases (28%) in which pacing device became necessary after S‐ICD implantation: a leadless pacemaker was implanted in one case. Inappropriate shock occurred in eight cases (25%) because of noise due to over‐sensing of myopotential and T wave over‐sensing. The problem was solved by changing to sensing vector. Inappropriate shocks due to interactions between the pacing device and the S‐ICD were not observed. We avoid using pacing leads in unipolar and keep the settings for functions that measure thoracic impedance turned off, thereby preventing shocks due to interactions between the devices.

**Conclusions:** The basic principle of managing with a single device remains unchanged even after the S‐ICD became available. However, the concept of dual device management is a new approach that offers an additional option for providing optimal device therapy to patients.

## IDENTIFICATION OF VENTRICULAR DYSFUNCTION MARKERS IN ELECTROCARDIOGRAMS VIA AI‐DRIVEN ANALYSIS

### **HISAKI MAKIMOTO** ^1,2^, TAKAYUKI OKATANI^3^, MASANORI SUGANUMA^3^, TOMOYUKI KABUTOYA^1^, TAKAHIDE KOHRO^1^, ALEXANDRU BEJINARIU^2^, OBAIDA RANA^2^, ASUKA MAKIMOTO^1^, MALTE KELM^2^, KAZUOMI KARIO^1^

#### ^1^Jichi Medical University, Shimotsuke, Japan,^2^Heinrich‐Heine University, Düsseldorf, Germany,^3^Tohoku University, Sendai, Japan

**Introduction:** Recent studies have demonstrated that artificial intelligence can efficiently identify ventricular dysfunctions using electrocardiograms (ECGs). However, it remains unclear which specific ECG waveforms indicate ventricular dysfunction. This study seeks to address this gap by identifying the ECG segments that most accurately signal ventricular dysfunction.

**Methods:** We analyzed paired ECG and echocardiography datasets comprising 17,422 cases from Japan and Germany. A convolutional neural network with 10 layers was developed to detect left ventricular ejection fractions (LVEF) under 50%. The models were trained and validated using 4‐fold cross‐validation using the Japanese data. We explored the effectiveness of various lead configurations and ECG segments (P, PQRS, QRS‐only, QRST), assessing metrics such as AUC, accuracy, sensitivity, and specificity. The test of the models was performed using the internal test data from Japan and the external test data from Germany.

**Results:** The models utilizing single‐beat ECGs significantly outperformed those employing 3‐second multi‐beat ECGs in classifying the LVEF as less than or greater than 50%, during both internal and external validation (AUC 0.87±0.01 vs 0.86±0.01, P=0.019; 0.86±0.00 vs 0.83±0.02, P=0.004, respectively). This indicated that a single cardiac cycle provides more efficient data for detecting ventricular dysfunction. The analysis of lead configurations revealed that limb‐leads, particularly leads I and aVR, were more indicative of ventricular dysfunction. Further detailed analysis within the single‐beat ECGs, using both internal and external validation datasets, identified that the segments from QRS to T‐wave were most indicative of ventricular dysfunction (**Figure**).

**Conclusions:** Our study confirms that a single cardiac cycle is adequately informative for detecting ventricular dysfunction and its specific signals are predominantly located within the QRS to T‐wave segments of single‐beat ECGs. Further investigation is warranted to identify the specific ECG signals or waveforms for ECG‐based diagnostics for ventricular dysfunction.

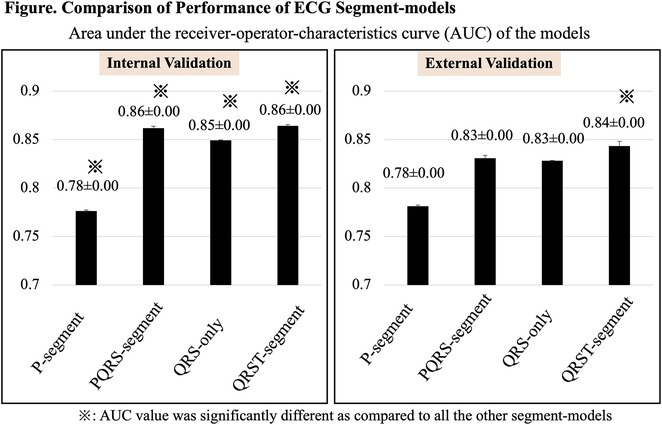

## CLINICAL PROFILE AND OUTCOMES OF PATIENTS UNDERGOING ATRIAL FLUTTER ABLATION AT A TERTIARY CARE CENTER IN INDIA: A RETROSPECTIVE COHORT STUDY

### **NAYANI MAKKAR** ^1^, KK MOHANAN NAIR^1^, ABHILASH SREEVILASAM P.^1^, MUKUND A PRABHU^2^, JYOTHI VIJAY^1^, VALAPARAMBIL KUMAR AJIT^3^, NARAYANAN NAMBOODIRI^1^

#### ^1^Sree Chitra Tirunal Institute of Medical Sciences and Technology, Thiruvanathapuram, Kerala, India,^2^Kasturba Medical College Manipal, Manipal Academy of Higher Education, Manipal, Karnataka, India,^3^Kerala Institute of Medical Sciences, Thiruvanathapuram, Kerala, India

**Introduction:** A higher prevalence of operated structural heart disease in Indian patients with atrial flutter (AFl) may impact the clinical demographics and procedural outcomes of radiofrequency (RF) ablation.

**Methods:** We retrospectively analyzed clinical, procedural and outcome data from a cohort of patients who had undergone RF ablation for macro re‐entrant atrial tachycardia.

**Results:** 100 patients underwent ablation for a macro re‐entrant atrial tachycardia from 2016 to 2023. These patients were predominantly male (66%) with a mean age at procedure of 50.3 ± 28.4 years, 41% patients had undergone cardiac surgery prior to AFl ablation. The patients were followed up for a median of 2 years. Non‐cardiac comorbidities were common in these patients with diabetes in 14%, hypertension in 15%, dyslipidemia in 10% and sleep apnea in 4%. 60% of patients had co‐morbid structural heart disease, with the commonest being a repaired secundum atrial septal defect (23 patients), 17 patients had coronary artery disease, 20 patients had left ventricular systolic dysfunction, 13% patients had atrial fibrillation (AF) prior to ablation and 19% patients developed AF subsequently. 77% of patients had cavo‐tricuspid isthmus dependent AFl. Of the remainder, 20 (83%) had macro re‐entrant right atrial tachycardia with half of these patients having scar related re‐entry from a right atriotomy. Acute success (the presence of bidirectional isthmus block and non‐inducibility of tachycardia) was obtained in 95% of patients, with significantly (p=0.08) more acute failures in patients with atypical (4 patients, 12.5%) than those with typical (1 patient, 1.3%) AFl. 8 patients had recurrent atrial flutter and 13 patients developed symptomatic bradycardia on follow up.

**Conclusions:** The presence of atypical flutter is the single most important predictor for procedural failure. The clinical profile of Indian patients undergoing ablation for AFl differs from data from prior western studies, including a younger age at onset, lower rates of comorbid AF, and a higher propensity for co‐morbid structural heart disease.

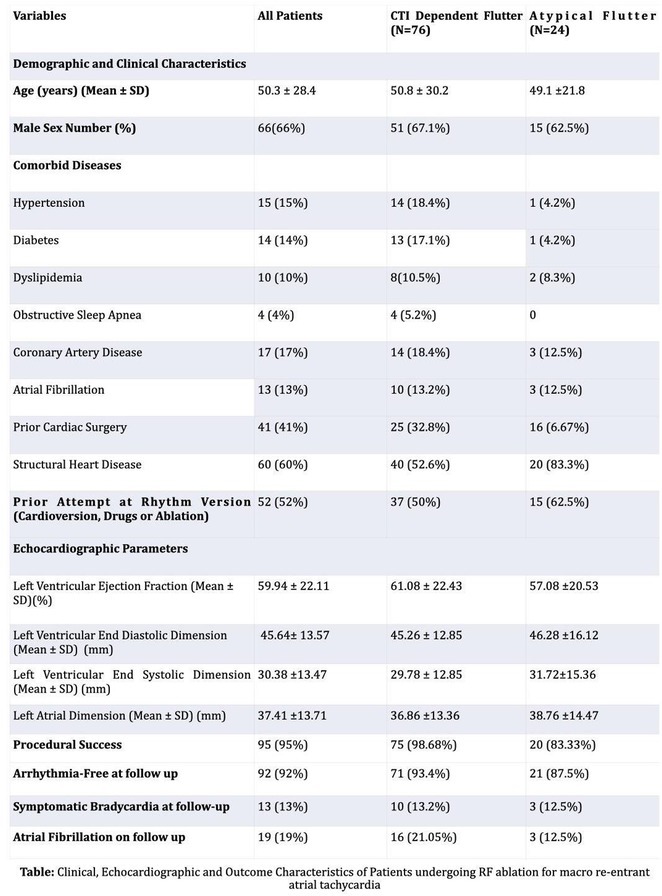

## MITRAL ANNULAR DISJUNCTION AND AN UNUSUAL ATRIAL TACHYARRHYTHMIA

### **NAYANI MAKKAR**, ABHILASH SREEVILASAM P., JYOTHI VIJAY, NARAYANAN NAMBOODIRI

#### Sree Chitra Tirunal Institute of Medical Sciences and Technology, Thiruvanathapuram, Kerala, India

**Introduction:** The risk of malignant ventricular arrhythmias in patients with mitral annular disjunction (MAD) in the mitral valve prolapse syndrome has received considerable interest. A similar pathophysiology of fibrosis and cellular uncoupling may also predispose the atria of these patients to the development of tachyarrhythmia.

**Methods:** N/A

**Results:** A lady in her thirties presented with episodes of abrupt onset, short‐lasting palpitations. The patient was in sinus rhythm with frequent runs of spontaneously induced, ill‐sustained tachycardia. The electrocardiogram demonstrated positive P waves in lead V1, negative deflections in leads II, III and aVF, and isoelectric/positive deflections in leads I and aVL. Electrograms recorded a regular narrow complex tachycardia with a tachycardia cycle length of 448 ms and a ventriculo‐atrial (VA) interval of 332 ms. There was spontaneous variability in the VA interval and attempted ventricular entrainment demonstrated VA de‐linking consistent with the diagnosis of an atrial tachycardia. Earliest atrial activation was seen in CS electrodes 7,8. Signals from the mapping catheter during tachycardia at the site of successful ablation on the posterior mitral annulus demonstrated a low amplitude fragmented signal 30 ms earlier than the surface P wave. These electrograms were characterized by the presence of both atrial and ventricular signals consistent with an annular location. Three‐dimensional electroanatomical maps of the left atrium in left anterior oblique and postero‐anterior projections exhibit a centrifugal pattern of left atrial activation with the earliest activation at a six o’ clock position on the mitral annulus. Delivery of radiofrequency energy at this site led to tachycardia termination within four seconds. Voltage‐mapping of the left atrium demonstrated no low voltage areas. Following ablation, tachycardia could not be induced despite aggressive pharmacological provocation and programmed stimulation.

**Conclusions:** Barring arrhythmias originating from the coronary sinus body, atrial tachycardia origin from the posterior mitral annulus is decidedly rare and may represent a pathophysiological correlate of MAD.

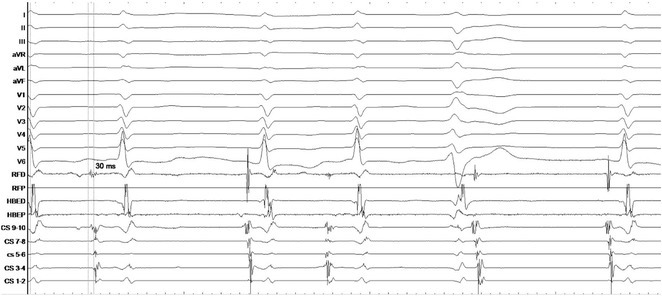

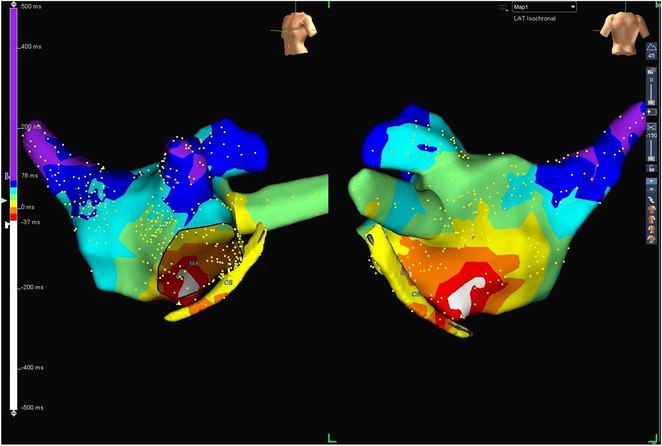

## WIDE COMPLEX TACHYCARDIA IN THE EBSTEIN HEART: AN UNUSUAL ABLATION TARGET

### **NAYANI MAKKAR**, JYOTHI VIJAY, ABHILASH SREEVILASAM P., NARAYANAN NAMBOODIRI

#### Sree Chitra Tirunal Institute of Medical Sciences and Technology, Thiruvanathapuram, Kerala, India

**Introduction:** The site of distal insertion of long decrementally conducting accessory pathways in the Ebstein heart is controversial.

**Methods:** N/A

**Results:** A lady presented with episodic wide complex tachycardia. Echocardiography demonstrated Ebstein's anomaly with moderate tricuspid regurgitation. Electrocardiography demonstrated a normal PR interval, absent right bundle branch block, absent septal forces and a rS pattern in lead III. Electrophysiology study demonstrated manifest pre‐excitation mediated by a decrementally conducting accessory pathway with only antegrade conduction. Burst atrial pacing induced a tachycardia with left bundle branch block morphology, a QRS axis of ‐30, and a late QRS transition. This tachycardia had a 1:1 VA relationship with a tachycardia cycle length of 354 ms, a VA interval of 144 ms, and an HV interval of ‐ 38 ms. An atrial premature complex during septal refractoriness advanced ventricular activation and reset the tachycardia. All features were consistent with an antidromic re‐entrant tachycardia via a ‘Mahaim’‐type accessory pathway. Initial mapping at the tricuspid annulus did not record a pathway potential. Attempted ablation at the site of shortest atrial stimulus to delta interval failed to abolish conduction. With the mapping catheter in the mid‐right ventricular cavity along the moderator band, sharp near field potentials were noted (Arrow). These potentials preceded ventricular activation (Interval B) and the His bundle potential (Interval A) reflecting a distal to proximal pattern of activation. Ablation at this site led to abolition of pathway conduction with the manifestation of complete right bundle branch block and restoration of proximal to distal activation of the right ventricular conduction system. This suggests that the distal insertion site of the accessory pathway was at the site of the right bundle traversing the mid‐moderator band confirming its atrio‐fascicular nature.

**Conclusions:** Where an anomalous tricuspid annulus makes mapping of the atrial insertion difficult, ablation at the distal insertion of an atriofascicular bypass tract may be a feasible alternative.

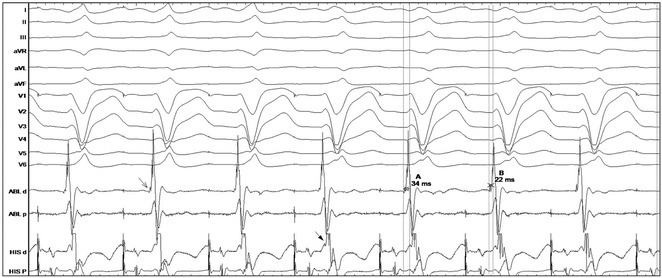

## ANTIBACTERIAL CIED ENVELOPE (TYRX)‐ SINGLE CENTRE EXPERIENCE

### AMIT KUMAR MALIK

#### MAX Superspeciality Hospital VAISHALI, Ghaziabad, India

**Introduction:** Cardiovascular implantable electronic devices (CIEDs) are implanted in an increasing number of patients each year, which has led to an increase in the risk of CIED infection. Infection is the most feared complication in patients with cardiac devices, with an incidence of 1‐3% during the lifetime. Upgradation of device and lead revision in background of previous multiple pulse generator replacements increase the risk many folds. Antibacterial CIED envelopes locally deliver antibiotics to the implant site over a short‐term period and have been shown to reduce the risk of implant site infection. We evaluate risk profiles and outcomes of patients who underwent CIED procedures with an antibacterial envelope.

**Methods:** Patients who underwent a CIED implantation between Nov 2022 and March 2024 were retrospectively reviewed. A total of 116 patients within this period were identified and reviewed. Total 10 CIED procedures used envelope (Tyrx™, Medtronic) that was coated with a resorbable polymer containing the drug substances rifampin and minocycline by the manufacturer.

**Results:** Average age of patient receiving envelope was 64.9 years, average PADIT score was 4.5, and 40% were reoperative procedures and one case with pocket infection. All cases in follow up and did not show any infection.

**Conclusions:** Antibacterial envelopes may benefit patients who are higher risk for infection, however additional studies are warranted to confirm this.

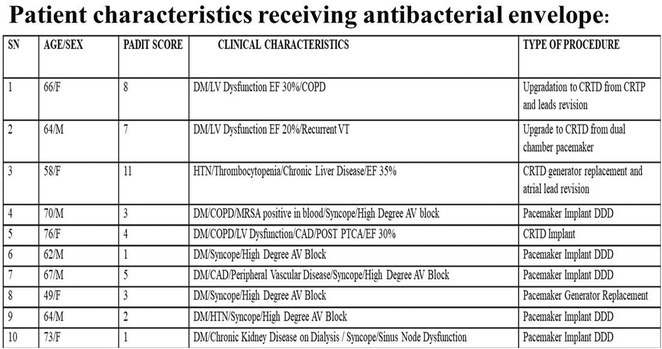

## DOUBLE THE TROUBLE, COMPLETE HEART BLOCK AND DILATED CARDIOMYOPATHY IN PROXIMAL MUSCLE WEAKNESS. HOW TO APPROACH IT?

### **SHADTHARCHARAN MANIAM**, FOO JHI HUI

#### Queen Elizabeth Hospital, KOTA KINABALU, Malaysia

**Introduction:** Neuromuscular diseases (NMDs) encompass a broad spectrum of diagnoses with overlapping but distinct phenotypes. Common to many NMDs is cardiac involvement. There are significant gaps in knowledge on how to approach cardiac care in these group of patients.

**Methods:** This is a 36 year old young gentleman on wheelchair bound for the past 10 years. He is known to have proximal muscle weakness but patient defaulted neurology follow up. This time around he presented to us with one history of syncope. ECG showed complete heart block, wide QRS complex and RBBB. Formal Echo showed EF around 35‐40% with a dilated LV and global hypokinesia. While Cardiac MRI confirmed a Non‐Ischemic DCM, evidenced by dilated LV, reduced LVEF (40%) and fibrosis over apical subepicardial, lateral subepicardial and midwall septal regions respectively. Patient's younger cousin has similar disease so we decided to send genetic test and muscle biopsy for the patient.

**Results:** Common cardiac manifestations in NMDs includes conductions system abnormalities, ventricular arrhythmias and dilated cardiomyopathy. For our patient, the first clinical presentation is complete heart block and cardiac pacing is needed to prevent sudden cardiac death. A conventional dual chamber pacemaker is mostly sufficient, but our patient had co‐existing dilated cardiomyopathy. Comparing to LOT‐CRTD and LOT‐ICD, Our team decided to implant a LOT‐CRTD, reason being, this patient is young and is having LV dysfunction with LV dysnchrony as well. In this terms, the LOT‐CRTD can achieve further resynchronization compare to LOT‐ICD. The defibrillator option is to prevent sudden cardiac death in view of the high prevalence in certain phenotype of NMDs. Apart from that, for this patient, the CRTD optimisation was done with EP guided method to achieve a more physiological PR interval.

**Conclusions:** In conclusion, managing patient with NMDs should be an individualised care. In a desire to avoid the adverse hemodynamic effects of RV pacing, we opted for conduction system pacing, namely LOT‐CRTD.

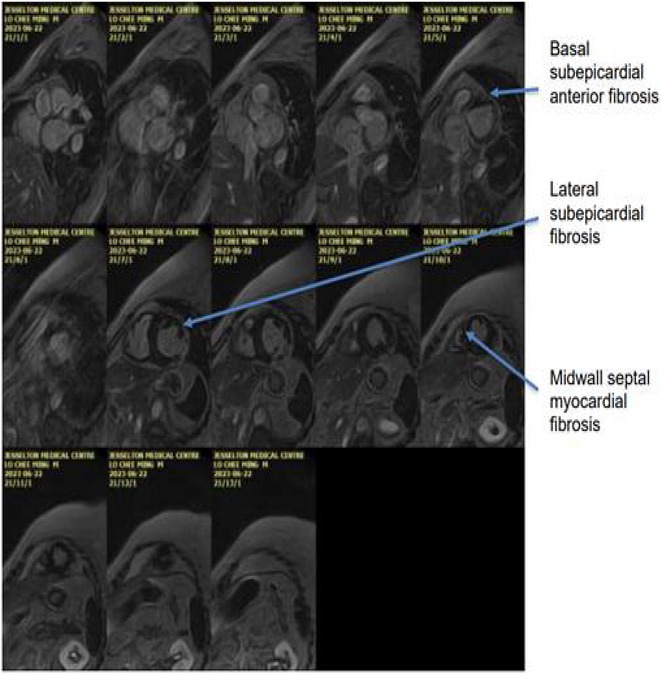

## PREPARATION KEY TO SUCCESS, AN UPFRONT CARDIONEUROABLATION STRATEGY IN ATRIAL FIBRILLATION AND MULTIFOCAL ATRIAL TACYCARDIA

### **SHADTHARCHARAN MANIAM**, FOO JHI HUI

#### Queen Elizabeth Hospital, KOTA KINABALU, Malaysia

**Introduction:** Cardioneuroablation, have emerged as a potential strategy for modulating autonomic function. Some studies have shown this method can help in treating vasovagal syncope, functional atrioventricular block, and sinus node dysfunction. Combining this technique with the conventional Atrial fibrillation and Atrial tachycardia ablation, may aid in treating patients who have both brady and tachyarrhythmia together.

**Methods:** This is a 47 year old female, with underlying hypothyroidism post RAI on maintainence thyroxine. Currently she is euthyroid. She has been diagnosed with paroxysmal Atrial fibrillation since 2021 and is on Vitamin K antagonist. She presented to us with recurrent palpitation 3 times this year since May 2023. The ECG showed Atrial Tachycardia for each presentation, and patient required electrical cardioversion to achieve sinus rhythm. Echo was done for her with LV function is preserved. Our working diagnosis was Paroxysmal Atrial Tachycardia and Paroxsymal Atrial fibrillation.

**Results:** Our team approach for this patient will be AF and AT ablation. The ECG for this patient prior to ablation was sinus beat and junctional escape beat, we anticipated possible underlying bradyarrhythmia due to likely sinus node dysfunction, verapamil or propranolol side effects. Expecting post ablation, the bradycardia component might become more dominant, we decided to do a cardioneuroablation strategy upfront followed by the ablation for the Atrial fibrillation and Atrial tachycardia. True enough, post procedure, patient developed junctional bradycardia and sinus pause requiring IVI isoprenaline temporarily in the ward. The bradycardia was only transient. Patient had a good chronotropic response later and was discharged with theophylline. Her heart rate on discharge was around 70‐80 in sinus rhythm.

**Conclusions:** As a whole, managing patient with tachy and brady arrhythmia should be an individualised care. In a desire to avoid the adverse effect of bradyarrhytmia post procedure, we opted for an upfront cardioneuroablation strategy followed by Atrial fibrillation and Atrial Tachycardia ablation in our patient.

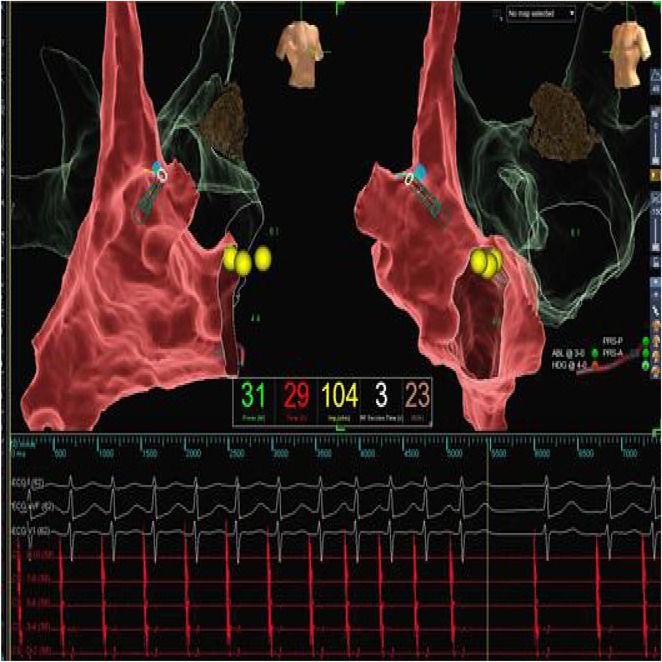

## THE HURDLE IN TREATING A PATIENT WITH BELHASSEN SYNDROME AND THE BOON OF BARTS SENSE PROTOCOL

### **SHADTHARCHARAN MANIAM**, FOO JHI HUI

#### Queen Elizabeth Hospital, KOTA KINABALU, Malaysia

**Introduction:** The occurrence of Idiopathic Fascicular VT is rare, and more uncommon a left anterior fascicular in origin. This arrhythmia has been reported to respond to intravenous verapamil, but some patient might have recurrence despite optimal medical therapy whom will need catheter ablation. Using the correct method to induce the VT substrate is essential. Sometimes this arrhythmia can be mistakenly diagnosed as AVNRT which occurred in our patient and to worse, a slow pathway modification was done prior to this, which made our procedure more challenging.

**Methods:** This is a 20 year old female, presented with palpitation, ECG showed RBBB, right axis deviation and AV dissociation, which was suggestive of left anterior fascicular VT. Fortunately patient was hemodynamically stable. The echo done in our side showed structurally normal heart. Patient was refractory to high doses of verapamil, hence we decided for catheter ablation. Further history revealed she had a slow pathway modification done 3 months ago for the similar presentation in a different centre. During the EP study, they were unable to induce VT.

**Results:** In view patient had a slow pathway modification done before, we anticipated a pre‐existing infra‐hisian block prior to the procedure. Well inducing VT was a challenge in the previous centre, hence our strategy was to use the Barts Sense Protocol to induce the VT, and map the substrate during sinus rhythm and VT, in view hemodynamically stable. Subsequently to ablate with a red curve biotronic catheter due to the anticipated anatomical difficulties. During the procedure, the baseline HV was prolonged 69ms. VT was inducible, VT stimulation was done upto V3, mapping done and ablation was successful.

**Conclusions:** As a whole, this case demonstrated that choosing the correct technique to induce VT is important, which in this case the Barts Sense protocol to map the VT substrate. Besides that, the choice of catheter also plays a role in ablation, especially to overcome the anatomical difficulties and pre‐existing infrahisian block. Lastly,the case taught us also on the importance of pre‐procedure ECG diagnosis in differentiating Left anterior fascicular VT and AVNRT.

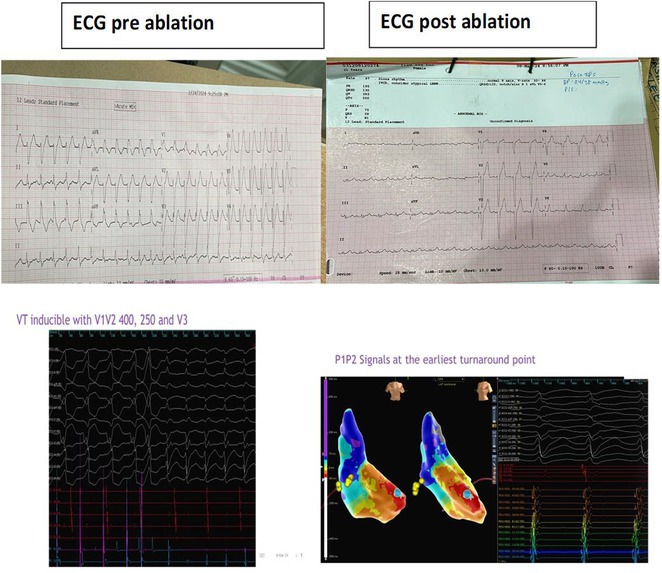

## CLINICAL PROFILE AND OUTCOMES OF ADULT PATIENTS DIAGNOSED WITH SUPRAVENTRICULAR TACHYCARDIA IN A PUBLIC TERTIARY HOSPITAL IN THE PHILIPPINES

### **MARIE KIRK PATRICH MARAMARA**, BIANCA VELANDO, RONALD ALLAN RODEROS, GISELLE GERVACIO

#### Philippine General Hospital, Metro Manila, Philippines

**Introduction:** Supraventricular tachycardia is now readily identified because of improved health‐seeking behavior of patients. In the Philippines, the incidence of SVT remains undocumented.Determining the characteristics of these patients may be helpful in providing appropriate management.

**Methods:** This is a retrospective cohort study involving adult patients with supraventricular referred to Cardiology service in Philippine General Hospital (PGH). Demographic, clinical, electrocardiographic, and echocardiographic profiles and the outcomes of patients with supraventricular tachycardia were gathered and analyzed.

**Results:** 49 patients diagnosed with SVT from June 2022 to June 2023 were included, the mean age was 45 years old with 53.06% being females. The most common comorbidity was hypertension (44.09%), diabetes mellitus (16.33%), and heart failure (16.33%). Majority of patients (57.14%) were diagnosed with AVNRT, with 73.47% presenting as palpitations. During SVT, 53.06% showed pseudo S and R’ in the ECG, while 30.61% with pre‐excitation. Echocardiogram findings such as LV dysfunction (8.16%), RV dysfunction (4.08%), valvular abnormality (6.12%), and congenital anomaly (6.12%) were seen in few patients.63.27% patients underwent RFA with high initial success rate of 96.77% and low complication rate of 3.23%. Among those managed medically, no pharmacologic side effects were reported. Forty‐eight (97.96%) of the patients were discharged stable, with a median hospital stay of 3 days.

**Conclusions:** This study demonstrated that AVNRT was the most common mechanism for SVT, with most patients being female. Hypertension, diabetes mellitus, and heart failure were the most common comorbidity among the patients. More than half of the patients underwent RFA with a high success rate. Meanwhile, those who were managed medically did not report pharmacologic side effects. To the best of our knowledge, this is the only local study done to identify the clinical profile and outcomes of SVT patients in general.

## A CASE OF PRE‐EXCITATION WITH REVERSE PATTERN BREAK ECG AS A PREDICTOR OF POSTEROSEPTAL ACCESSORY PATHWAY ABLATABLE FROM PROXIMAL CORONARY SINUS

### AIMADUDDIN MAT DAUD

#### Hospital Sultan Idris Shah, Serdang, Malaysia

**Introduction:** We present a case of Wolf‐Pakinson‐White (WPW) syndrome whom we successfully ablated the accessory pathway from the proximal coronary sinus.

**Methods:** none

**Results:** A middle age lady was referred to our centre for symptomatic supraventricular tachycardia. Her baseline ECG showed pre‐excitation with reversed pattern break (monophasic tall R wave in V2 larger than V1 and V3, along with R/S <1 in V3 and S wave V3 >4mV). She underwent a 2D EP study which induced orthodromic AVRT on table. Ablation was done to the right posteroseptal region (5 o’clock in LAO). The EGM showed separation of V‐A signal on distal ablation catheter and the tachycardia was terminated.

After a month she came back with incessant narrow complex tachycardia (NCT). She underwent for a second EP study with 3D Ensite mapping system. Orthodromic AVRT was induced again with earliest A seen at CS 5,6. Open window mapping system using HD Grid showed fused V‐A signal at CS OS with AP potential. Propagation mapping also showed AP breakout at CS OS. Ablation done inside the CS to the area and the tachycardia terminated within a few seconds. Consolidation ablation was done.

A 30 minutes waiting period showed persistent loss of delta waves, and NCT was no longer inducible with multiple manoeuvres and IV isoprenaline boluses. She was discharged well. During clinic follow up she no longer had symptoms of palpitation and her ECG remained without delta waves.

**Conclusions:** Posteroseptal accessory pathways (PSAPs) occasionally lie within the coronary sinus (CS) or its tributaries, in a CS diverticulum or along the epicardial surface. This makes ablation of the accessory pathway more challenging, and may lead to insufficient ablation. The baseline ECG in sinus rhythm is an important investigation to decide the strategy for ablation.

Reversed pattern break in a patient with delta waves ECG is predictor of possible posteroseptal accessory pathway ablatable from the proximal CS This may indicate the need for the physician to map within the CS, CS diverticulum or epicardium. Recognising this pattern will help the physician to achieve durable and successful ablation.

## UNIPOLAR VOLTAGE MAP GUIDED HIGH VOLTAGE AREA TARGETED ABLATION IS AN EFFICIENT APPROACH IN PULMONARY VEIN ISOLATION

### **TAKUMI MATSUBARA**, SADAHARU MATSUMOTO, YOKO TAIRA, HIROMI OKAZAKI, KEIKO NISHIYAMA, HIRONOBU KIKUCHI, MASAHIRO MYOJO, YASUYUKI SUGISHITA, NOBUHIKO ITO, FUMIKO TABEI

#### Kanto Central Hospital, Setagaya, Tokyo, Japan

**Introduction:** Voltage mapping of the left atrium is one of the general approach for catheter ablation of atrial fibrillation (AF). Recently, relationship between unipolar voltage and atrial wall thickness in AF cases has reported. Although, how to utilize unipolar voltage map in pulmonary vein isolation (PVI) has not reported.

**Methods:** The present study is single‐center, retrospective study. Cases with unipolar voltage map guided PVI were compared to cases with conventional PVI. Cases with first‐session PVI since May 2023 to March 2024 were included. Total ablation points, total ablation time, total procedure time, lesion distances were assessed. Left atrial unipolar voltage ranges were also assessed to identify relatively high voltage area in the left atrium. The upper limit of the bipolar voltage was set to the lower limit of the unipolar voltage, and the upper limit of the unipolar voltage was set to be two to three times the lower limit. The areas which above the unipolar voltage upper limit were defined as high voltage area in unipolar voltage map.

**Results:** There were 29 cases for conventional PVI and 12 cases in unipolar voltage map guided PVI. PVI were accomplished in all cases. Total ablation points, ablation time were decreased in unipolar voltage map guided ablation group (78 ± 30 points vs 137 ± 72 points, p=0.0025. 1307 ± 519 sec vs 1928 ± 512 sec, p=0.002). Lesion distances were increased in unipolar voltage map guided ablation group (left pulmonary vein : 4.8 ± 1.1 mm vs 3.3 ± 0.9 mm, p<0.0001. right pulmonary vein : 5.4 ± 1.7 mm vs 3.2 ± 0.7 mm, p<0.0001). High voltage areas were mostly determined in roof of left superior pulmonary vein, anterior area of right pulmonary vein and bilateral posterior carina.

**Conclusions:** The present method has as high acute success rate as conventional method and reduction of number of radio‐frequency application was possible. Unipolar voltage map guided PVI seemed to be an efficient approach to accomplish PVI.

## PREVALENCE OF SLEEP DISORDERED BREATHING AND CHEYNE‐STOKES RESPIRATION IN PATIENTS WITH ATRIAL FIBRILLATION

### HIROKI MATSUMOTO

#### Juntendo University, Tokyo, Japan

**Introduction:** Sleep disordered breathing (SDB) is prevalent in patients with atrial fibrillation (AF). Obstructive sleep apnea (OSA) is associated with the recurrence of AF and treatable with continuous positive airway pressure (CPAP). Central sleep apnea (CSA) is also present but underdiagnosed with conventional respiratory polygraphy in patients with AF. Cheyne‐Stokes respiration (CSR), a specific form of CSA, plays an important role in the progression of heart failure (HF). Limited data is available regarding the prevalence of CSR‐CSA in Japanese patients with AF.

**Methods:** Patients with paroxysmal or persistent AF underwent echocardiography and cardiorespiratory polygraphy (ApneaLink Air; ResMed, Australia). Multiple linear regression analysis was performed treating logarithmically transformed apnea‐hypopnea index (AHI) and the percentage of CSR as dependent variables.

**Results:** Overall, 462 patients were enrolled. 242 patients (52.4%) were diagnosed as SDB (AHI ≥10/hour) and the median AHI was 22.9/hour. CSR was present in 101 patients (21.9%). Multiple linear regression analysis showed age, gender, body mass index (BMI), and hypertension were independent correlates of AHI (p=0.0188, 0.0002, &lt;0.0001, and 0.0457). On the other hand, age and plasma NT‐proBNP were independent correlates of the percentage of CSR (p=0.0026 and 0.0003).

**Conclusions:** OSA and CSR‐CSA were prevalent in Japanese patients with AF. CSR was associated with age and plasma NT‐proBNP significantly.

## FEASIBILITY AND EFFICACY OF 50W ABLATION WITH TACTIFLEX CATHETER IN INITIAL PULMONARY VEIN ISOLATION FOR ATRIAL FIBRILLATION

### **KAZUHISA MATSUMOTO**, HITOSHI MORI, NAOMICHI TANAKA, TSUKASA NAGANUMA, WATARU SASAKI, MASATAKA NARITA, YOSHIFUMI IKEDA, TAKAHIDE ARAI, SHINTARO NAKANO, RITSUSHI KATO

#### Saitama Medical University International Medical Center, Hidaka, Japan

**Introduction:** A novel contact force (CF) sensing catheter with a mesh‐shaped irrigation tip (TactiFlexTM SE, Abbott), was invented and expected to be safe and effective radiofrequency ablation. Our previous ex‐vivo study demonstrated the efficacy of 50W ablation using TactiFlex catheter in creating transmural lesions in pulmonary vein. This study examined the feasibility and safety of 50W ablation with TactiFlex in the initial ablation of atrial fibrillation (AF).

**Methods:** 100 AF patients who underwent ablation with TactiFlex were studied. Pulmonary vein isolation (PVI) was performed with a 50W setting, a 5‐20g contact force (CF), and a 15‐20 second energy delivery. Primary outcomes included the success of PVI, first‐pass isolation (FPI), and the presence of PV conduction gaps. Patients were followed up for 12 months after PVI and underwent mandatory ECG recordings at 1, 3, 6, and 12 months post‐PVI.

**Results:** PVI was achieved in all cases. FPI was observed in 82/100 (82%) for right pulmonary vein (RPV) and 87/100 (87%) for left pulmonary vein (LPV). Among unsuccessful FPIs in RPV, residual carina potential was noted in 16/18 cases (89%), PV gap in 1/18 cases (5.5%), and both carina and PV gap in 1/18 cases (5.5%). Similarly, among unsuccessful FPIs in LPV, residual carina potential was observed in 11/13 cases (84.6%), PV gap in 1/13 cases (7.7%), and both carina and PV gap in 1/13 cases (7.7%). Periesophageal nerve injury occurred in 1/100 cases (1%), and no cardiac tamponade occurred. Impedance drop was significantly greater in the FPI group for both RPV and LPV (FPI group: 16.1±6.6 ohm vs. non‐FPI group: 14.4±5.2 ohm in RPV (p<0.01), and FPI group: 15.9±6.5 ohm vs. non‐FPI group: 13.5±5.1 ohm in LPV (p<0.01)). AF free rate at one year was 86.0% for PAF and 76.3% for non‐PAF.

**Conclusions:** The 50W ablation with TactiFlex demonstrated a high rate of FPI, low incidence of PV gaps, and low AF recurrence rate at one year for PAF. This method proved to be a safe and effective approach in the initial PVI for AF.

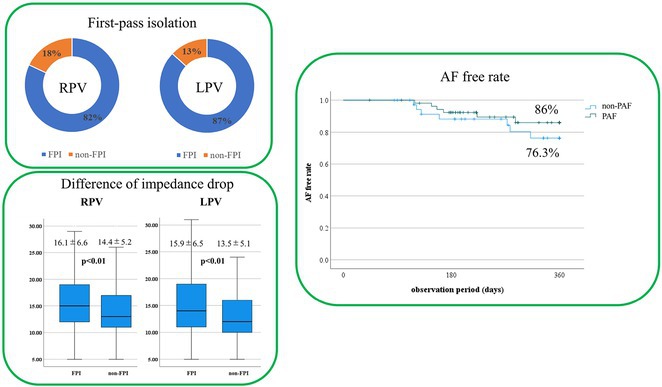

## TREATMENT OUTCOMES FOR CATHETER ABLATION IN ATRIAL FIBRILLATION OF ADULT CONGENITAL HEART DISEASE

### YU MATSUMURA

#### Sakakibara Heart Institution, Tokyo, Japan

**Introduction:** The population of adult congenital heart disease (ACHD) patients is increasing. These patients often have atrial arrythmia and require hospitalization. Number of cather ablation for ACHD patients is on the rise. However, the report on catheter ablation results is limited.

**Methods:** A single center retrospective analysis was performed of ACHD patients undergoing atrial arrythmia ablation. Clinical data were collected, including type of arrythmia, CHD type, and outcomes. One‐year procedural success was defined as freedom from arrythmia.

**Results:** Overall, 21 ACHD patients ( mean age 43years, 71.4% male; 47.6% with atrial flutter) undergoing cather ablation. Pulmonary vein isolation was perfoemd in 8 (38%) procedures. One year success rate was 88%. (15/17) Four patients had ablation less than a year after the procedure. There were no major complications. Four of the 15 patients had arrhythmia event after one year.

**Conclusions:** In this study, the result of ablations for atrial arrythmia for ACHD patients was satisfactory. Since recurrence increases in the long term, careful follow‐up is necessary.

## ENHANCING ICM PAUSE EPISODE DISCRIMINATION AI/ML MODELS THROUGH DATA AUGMENTATION TECHNIQUES

### ELNAZ LASHGARI, DANIEL MONJE, FADY DAWOUD, FUJIAN QU, ADITYA GOIL, **LUKE MCSPADDEN**

#### Abbott, Sylmar, CA

**Introduction:** Remote monitoring of patients with insertable cardiac monitors (ICMs) has improved timely diagnosis of cardiac arrhythmias, but challenges remain in managing and adjudicating ICM electrograms. AI models can provide accurate rhythm classification from device electrocardiograms (EGMs). Training AI model using data augmentation (DA) techniques allows enriching datasets to achieve larger training sample size with more diversity resulting in robust models resistant to data excursions and reduced overfitting. This study evaluated the impact of DA on the performance of three distinct machine learning models tailored for the specific task of rhythm classification in pause episodes detected by ICMs.

**Methods:** Three custom designed models, a dense neural network (NN), a convolutional neural network with a dense layer (CNN‐Dense), and a convolutional neural network with a global average pooling layer (CNN‐GAP) for pause rhythm classification were evaluated. The training data consisted of ICM‐detected pause episodes from 1,173 patients. The validation dataset includes 1,095 detected Pause episodes from 256 patients for hyperparameter tuning. All episodes were manually adjudicated. Device EGMs were augmented by reversing polarity thereby doubling the size of the training dataset. Additional augmentation was performed by adding gaussian noise (mean = 0, std 0.05) to the training dataset. To confirm the clinical accuracy of data augmentation, a subset of the augmented EGMs was manually reviewed, and previous labels reconfirmed. The 3 models were trained with and without data augmentation. All 6 trained models were assessed against an unseen test set for performance evaluation.

**Results:** The test data consisted of 1,986 episodes (744 true episodes from 382 patients and 1,242 false episodes from 485 patients). The models trained with DA showed similar or better performance, compared to without DA, in sensitivity, specificity, accuracy and F1 score metrics as shown in the table.

**Conclusions:** Clinically appropriate data augmentation applied to neural networks shows similar or better model performance when assessed in an unseen test dataset.

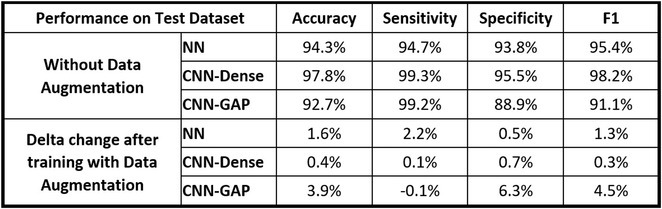

## PERFORMANCE OF AUTOMATIC CAPTURE CONFIRMATION ALGORITHM IN A LARGE COHORT OF PACEMAKER PATIENTS WITH LEFT BUNDLE BRANCH AREA PACING

### MATTHEW BERNABEI^1^, SANDEEP BANSAL^1^, R. WARD PULLIAM^1^, FADY DAWOUD^2^, WENWEN LI^2^, **LUKE MCSPADDEN** ^2^, LEYLA SABET^2^, KYUNGMOO RYU^2^, JEFFREY ARKLES^1^

#### ^1^Lancaster General Health, Lancaster, PA,^2^Abbott, Sylmar, CA

**Introduction:** The automatic capture confirmation algorithm has been frequently used in implantable pulse generators to automatically measure ventricular pacing capture thresholds (PCTs) and to ensure beat‐by‐beat capture while optimizing battery usage. We aimed to evaluate the performance of the AutoCapture™ (Abbott, IL, USA) algorithm in a large cohort of pacemaker patients with left bundle branch area pacing (LBBAP).

**Methods:** De‐identified device data were analyzed from patients who received LBBAP and Abbott pacemaker implant from June 2021 to August 2023. AutoCapture™ measured PCTs at implant were compared to the manually measured PCTs. A difference within ±0.25 V was deemed equivalent. AutoCapture™ PCTs in all available follow‐up periods were also obtained from Merlin.net remote transmissions to characterize the long‐term stability of PCTs.

**Results:** A total of 619 patients implanted with Abbott Assurity MRI single chamber (Model 1272, n= 89) or dual‐chamber (Model 2272, n= 530) pacemakers in a single center were included in the analysis. Pacing indications included (some with multiple indications): sinus node dysfunction (15%), AV block (46%), atrial fibrillation (37%), and AVN ablation (28%). AutoCapture™ and the manually measured PCTs at implant were equivalent in 600/615 (97.6%) patients with average PCTs of 0.76±0.28 and 0.80±0.25 V, respectively, at pulse width of 0.5 ms. The difference in PCTs by AutoCapture™ setup test and manual test is shown in Figure 1A. At 1‐month, 3‐month, 6‐month, 12‐month, and 24‐month remote follow‐up, AutoCapture™ measured PCTs were 0.67±0.29 (n=594), 0.66±0.25 (n=560), 0.71±0.29 (n=543), 0.77±0.29 (n=447) and 0.81±0.28 (n=112), respectively (Figure 1B). At the most recent remote follow‐up (median 521 [IQR: 347‐693] days), bipolar pacing (612/619, 99%) impedance was 534±60 ohms and sensed R‐wave amplitude was 11±3 mV.

**Conclusions:** The AutoCapture™ algorithm measured accurate PCTs at implant and showed a stable trend during long‐term follow‐up in pacemaker patients receiving LBBAP.

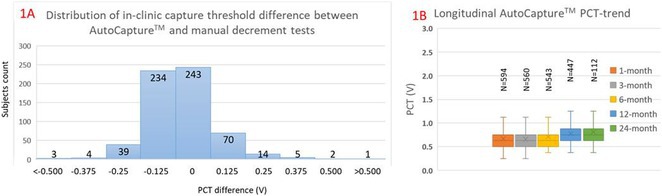

## EFFECT OF VENTRICULAR PACING THRESHOLD IN PATIENTS WITH PACEMAKERS AFTER COVID‐19 INFECTION

### KOMSING METHAVIGUL

#### Central Chest Institute of Thailand, Nonthaburi, Thailand

**Introduction:** Patients recovered from COVID‐19 infection had several cardiovascular consequences. Previous trials showed that those patients have found evidences of myocarditis and late gadolinium enhancement reflecting myocardial scar after recovery of COVID‐19 infection. However, data about effect of ventricular pacing threshold after recovered COVID‐19 infection are lacking. This trial was conducted to study the effect of ventricular pacing threshold in patients with pacemakers after COVID‐19 infection.

**Methods:** Patients with pacemakers were retrospectively recruited at device clinic, Central Chest Institute of Thailand among January 2022 to September 2023. Those patients were classified to 2 groups according to a history of previous COVID‐19 infection. The primary outcome was the high ventricular pacing threshold in each group. The secondary outcome was the mean ventricular pacing threshold in each group. The chi‐square test was used to compare the primary outcome between both groups. The independent t test was used to compare the secondary outcome between both groups.

**Results:** A total of 122 patients were enrolled. An average age was 69.4 years. An average follow‐up time was 10.7 months. There were 54 patients in COVID‐19 group and 68 patients in non‐COVID‐19 group. More patients in COVID‐19 group had high pacing threshold than those in non‐COVID‐19 group with no statistical significance (odds ratio 1.27; 95% confidence interval 0.17‐9.31; *P* > 0.99). There was comparable mean ventricular pacing threshold between both groups (mean absolute difference ‐0.03; 95%CI ‐0.14 to 0.09; *P* = 0.65).

**Conclusions:** Patients with pacemakers had no significantly increased ventricular pacing threshold after recovered COVID‐19 infection.

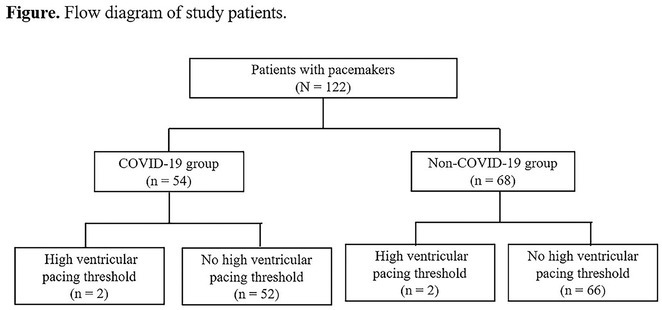

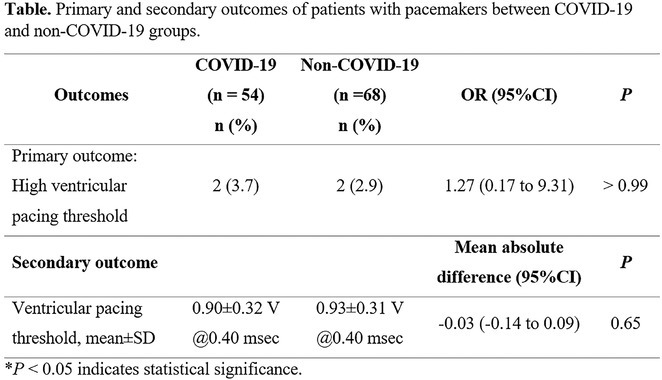

## THE FIRST ICD SHOCK OCCURRED 34 YEARS AFTER THE LAST SYNCOPE EPISODE IN A SYMPTOMATIC BRUGADA PATIENT

### **ASUKA MINAMI‐TAKANO** ^1^, MASATAKA SUMIYOSHI^2^, ATSUSHI KIMURA^2^, MITSUHIRO KUNIMOTO^2^, HARUNA TABUCHI^2^, GAKU SEKITA^2^, KIKUO ISODA^2^

#### ^1^Juntendo University, Chiba, Japan,^2^Juntendo University Nerima Hospital, Tokyo, Japan

**Introduction:** Atrial fibrillation (AF) is the most common arrhythmia other than ventricular fibrillation (VF) in Brugada syndrome (BrS), and AF is well known as a major cause of inappropriate shock from an implantable cardioverter defibrillator (ICD).

**Methods:** “N/A”

**Results:** “Case” A 70s male underwent pacemaker implantation (VVI) for sick sinus syndrome in 19XX (age at 40s). One year later, he experienced syncope due to VF with incomplete right bundle branch block and persistent ST elevation, which was later diagnosed as BrS. The patient underwent an ICD upgrade in 200X (at age 50s) and an ICD replacement in 201X, with no prior ICD treatment due to arrhythmias and syncope. However, in 202X, 34 years after the last syncope (at age 70s), the ICD delivered 4 shocks during the daytime. Device analysis revealed that the ICD shocks were caused by paroxysmal AF, which was subsequently treated with pulmonary vein isolation and no recurrence was observed. AF is the most common atrial arrhythmia in BrS, and enhanced duration of atrial action potential and increased intra‐atrial conduction time may contribute to the genesis of atrial arrhythmias. In this case, although AF was rarely observed during device follow‐up, the frequency of AF burden may have increased due to aging. In addition, the single‐lead ICD may be attributed to the ICD malfunction.

**Conclusions:** During long‐term follow‐up, BrS patients with single‐lead ICDs have a potential risk for ICD malfunction due to age‐related AF although no AF episodes were observed during ICD follow‐up.

## ABSORBABLE ANTIBACTERIAL ENVELOPE PROMOTES DEVELOPMENT OF A HEALTHY DEVICE POCKET

### **SUNEET MITTAL** ^1^, FRANCOIS PHILIPPON^2^, ELENA LADICH^3^, RENU VIRMANI^3^, JAMES IP^4^, JAY WRIGHT^5^, ANDREW HAZLITT^6^, MAURO BIFFI^7^, ERIC JOHNSON^8^, ANNA JOKINEN^9^, JEFF LANDE^9^, ALOKE FINN^10^, CHIARA BALDOVINI^11^, CHRISTOPHER ELLIS^12^

#### ^1^Valley Health System, Paramus, NJ,^2^Institu Universitaire de Cardiologie et de Pneumologic de Quebec, Quebec, QC, Canada,^3^CV Path Institute, Gaithersburg, MD,^4^Weill Cornell Medicine ‐New York Presbyterian Hospital, New York, NY,^5^Liverpool Heart and Chest Hospital, Liverpool, United Kingdom,^6^Clearwater Cardiovascular Consultants, Clearwater, FL,^7^Policlinico Sant Orsola, Malpighi, Italy,^8^The Stern Cardiovascular Foundation, Germantown, TN,^9^Medtronic, Mounds View, MN,^10^2Institut Universitaire de Cardiologie et de Pneumologie de Québec, Quebec, QC, Canada,^11^IRCCS Azienda Ospedaliero‐Universitaria di Bologna, Bologna, Italy,^12^Vanderbilt University Medical Center, Nashville, TN

**Introduction:** Cardiac implantable electronic device (CIED) implantation involves creating a subcutaneous pocket that results in an inflammatory response which can impact healing within the pocket. An absorbable antibacterial envelope (TYRX™, Medtronic Inc.) has been developed to stabilize CIEDs and reduce infection. While the envelope has proven safe and effective at reducing infection in the WRAP‐IT study (NCT02277990), characterization of its impact on CIED capsule formation in humans is limited. The objective of this study was to characterize histological and morphometric features of device capsules in patients undergoing secondary procedures after a procedure with an envelope.

**Methods:** The TYRX™ Pocket Health Study (NCT05356546) was conducted June 2022 to July 2023 at 9 sites. The study characterized pocket conditions of patients with an existing CIED previously implanted with an absorbable antibacterial envelope returning for a replacement procedure. Tissue sections were obtained, stained with H&E, Masson's Trichrome and Movat Pentachrome and evaluated for adhesions, capsule thickness, inflammation, neovascularization, and calcification.

**Results:** From 31 eligible patients, 28 anterior tissue samples were obtained. The initial envelope had been used 6.8±1.3 years prior to the current procedure. Most patients presented with absent or only mild lead wrap or lead connector adhesions (67.7%). Mean capsule wall thickness was 0.33±0.16 mm and was comprised of predominantly mature collagen (Figure). Cellular markers of chronic inflammation were low. Twenty‐six out of 28 patients had no clinically apparent calcification. Neovascularization of tissue was minimal (n=19) or mild (n=9).

**Conclusions:** In this prospective evaluation of long‐term pocket healing with an absorbable envelope, low adhesions, low inflammation, and well‐formed capsules were observed. This suggests that absorbable antibacterial envelopes may have benefits beyond infection, which is desirable during secondary procedures.

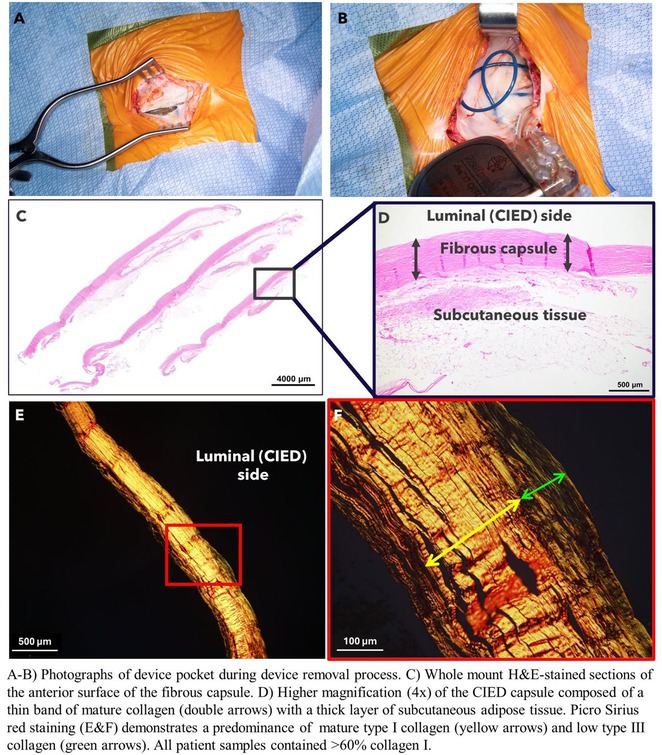

## A CASE OF MITRAL ISTHMUS BLOCK THAT COULD NOT BE CREATED WITH RADIOFREQUENCY ABLATION BUT COULD BE CREATED WITH CRYOBALLOON ABLATION

### **MARIE MIURA**, ATSUSHI SUZUKI, RENA NAKAMURA, MICHIO USUI

#### JCHO Tokyo Yamate Medical Centre, Shibuya, Tokyo, Japan

**Introduction:** Mitral isthmus block creation for peri mitral atrial tachycardia is still challanging with radiofrequency catheter ablation.

**Methods:** A 73‐year‐old man had recurrence of atrial fibrillation after pulmonary vein isolation and underwent a second session. After successful left atrial roof line, peri‐mitral atrial tachycardia(PMAT) was induced. Mitral isthmus block creation with radiofrequency catheter ablation(RFA) were performed from endocardial and epicardial approach, resulting in unsuccessful. Four years later, an atrial tachycardia(AT) developed frequently, necessitating a third session.

**Results:** Activation mapping revealed the mechanism of AT as PMAT. Meanwhile, entrainment pacing from a distal electrode of coronary sinus revealed an epicardial conduction block. Mitral isthmus ablation was performed using cryoballoon because of the difficulty of creating endocardial conduction block with RFA in the previous session. First, when the cryoballoon ablation was started from the LIPV antrum side with LIPV anchoring, the tachycardia cycle length was prolonged from 280ms to 290ms. Further extended to 300ms with the next freezing. However, the balloon could not reach the annulus side of the mitral isthmus with LIPV anchoring. Then, cryoballoon ablation were performed to the annulus side of mitral isthmus without anchoring, then the tachycardia cycle length rapidly prolonged and finally the tachycardia stopped. Finally bidirectional mitral isthmus block was obtained.

It is common that epicardial conduction across the roof line after roof block creation using radiofrequency catheter ablation. On the other hand, there are no report with epicardial conduction after roof line creation using a cryoballoon. Mitral isthmus ablation using cryoballoon can be expected to produce a transmural lesion similar to roof line, and generally band‐like lesions are obtained, making it easy to see the gap.

**Conclusions:** Mitral isthmus block could be achieved effectively by using the cryoballoon.

## PREVALANCE OF SUPERIOR VENA CAVA (SVC) FIRING: SIMPLIFIED SVC AND SINUS NODE MAPPING(SSS MAP) TO AVOID SINUS NODE INJURY

### **TOMOHIRO MIYATA** ^1^, ATSUSHI SUZUKI^2^, RYUDO FUJIWARA^3^, YUSUKE SONODA^3^, JUN SAKAI^3^, DAISUKE MATSUMOTO^2^, KUNIHIKO KIUCHI^2^, YOICHI KIJIMA^3^, EIJI OGURA^1^, HIROSHI TAKAISHI^2^, JUNYA SHITE^3^

#### ^1^Biwako Ohashi Hospital, Otsu City, Japan,^2^Yodogawa Christian Hospital, Osaka, Japan,^3^Osaka Saiseikai Nakatsu Hospital, Osaka, Japan

**Introduction:** The superior vena cava (SVC) is known as a major source of non‐pulmonary vein AF foci (NPVAF). In some cases, an incessant form of AF (iAF) by SVC firing prevents obtaining the sinus node location, and anatomical SVC isolation has a risk of sinus node injury (SNI).This study was aimed to explore the frequency of iAF by SVC firing and the efficacy of the simplified mapping strategy to avoid SNI.

**Methods:** Consecutive AF ablation procedures were retrospectively investigated. Simplified SVC/SN mapping (SSS‐map: Obtaining the top‐end of the SVC potential and >4points with 5‐10mm spacing in the upper lateral side of the right atria (RA) for roughly locating the earliest activation site in sinus rhythm) was performed before PV isolation in the last 138 patients. In case of the initial rhythm was AF, cardioversion was performed. In the cases the high‐density mapping catheters were available, the high‐density activation maps of the RA in sinus rhythm were obtained to investigate the dislocation of the earliest activation site of the SSS map. When SVC isolation was required due to iAF, SVC isolation line was created to keep the distance of 10 mm above the second earliest activation site.

**Results:** 1160 procedures (male: 736, age: 69.9, paroxysmal AF: 544, first session: 997) were investigated. NPVAF were observed in 160 procedures (13.7%) (SVC: 64, posterior wall of the left atria: 24, coronary sinus: 19, and others: 53). 16 patients developed to iAF by SVC firing. The SSS‐map was successfully obtained in all of the last 138 patients (Mapping time: 1.3±0.6 min, number of obtained points: 8.4±1.3). In 21 patients in which the high‐density RA map was obtained, the SN dislocation distance was 5.1±2.2mm between the SSS map and the high‐density RA map. 5 of 138 developed to iAF by SVC firing, and SVC isolation based on the SSS‐map was successfully achieved without SNI in the cases.

**Conclusions:** This study indicated iAF by SVC firing after PV isolation occurred in certain number of patients. The SSS‐map before PV isolation was practical and showed the potential to be the mapping strategy avoid the SN injury in such cases.

## LONG RP TACHYCARDIA INDUCED BY VENTRICULAR PACING AT HIS‐BUNDLE REFRACTORY TIMING

### **NAOKO MIYAZAKI** ^1^, ATSUSHI DOI^2^, KOUHEI IWASA^1^, MASATO OKADA^1^, YUKO HIRAO^1^, KOJI TANAKA^1^, NOBUAKI TANAKA^1^

#### ^1^Sakurabashi Watanabe Hospital, Osaka, Japan,^2^Tane general Hospital, Osaka, Japan

**Introduction:** The diagnosis of atrioventricular nodal reentrant tachycardia (AVNRT) with a bystander nodofascicular/nodoventricular bypass tract (NF/NV‐BT) is challenging and requires precise interpretation of the intracardiac electrograms.

**Methods:** N/A

**Results:** A 51‐year‐old female with frequent palpitations was referred for radiofrequency ablation. The dual retrograde conduction pathways exhibited decremental conduction properties, with the earliest atrial activation recorded in the His‐bundle and coronary sinus (CS) ostium region. A supraventricular tachycardia (SVT) with a long RP interval was induced through a ventricular‐atrial‐ventricular (VAV) response by ventricular pacing during the His‐bundle refractoriness. The earliest atrial activation during the SVT was identical to the CS ostium site during ventricular pacing. The atrial overdrive pacing with varying numbers of beats applied from the CS ostium during SVT did not result in a constant VA interval following the cessation of pacing. During the SVT, His‐bundle refractory ventricular extrastimulus delayed the subsequent atrial activation without altering the activation sequence, thus resetting the tachycardia. This finding suggests the presence of a concealed atrioventricular accessory pathway or NF/NV‐BT. Disappearance of the tachycardia reset phenomenon was noted upon the delivery of an earlier ventricular extrastimulus, excluding the possibility of orthodromic reentrant tachycardia. Moreover, administration of an even more premature ventricular extrastimulus resulted in a delay in the subsequent atrial activation without altering the activation sequence, resetting the tachycardia once again. Consequently, we diagnosed the SVT as fast/slow AVNRT with a bystander NF/NV‐BT inserting the retrograde slow pathway.

**Conclusions:** It is possible to identify the presence of a concealed NF/NV‐BT when AVNRT is induced by His‐bundle refractory ventricular stimulation. Therefore, meticulous attention to the manner of the tachycardia induction is imperative for an accurate diagnosis of the tachycardia.

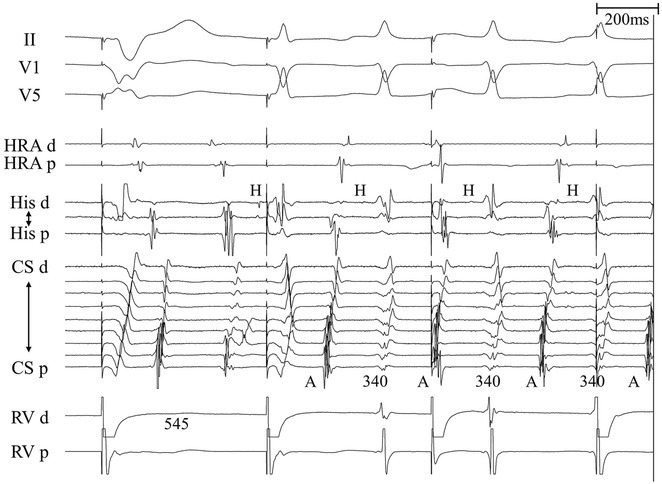

## SLOWLY PROGRESSIVE CARDIAC TAMPONADE AFTER CATHETER ABLATION FOR PERSISTENT ATRIAL FIBRILLATION INDUCED BY BOTH OOZING RUPTURE AND PERICARDITIS

### **NAOKO MIYAZAKI**, NOBUAKI TANAKA, KOUHEI IWASA, MASATO OKADA, YUKO HIRAO, KOJI TANAKA

#### Sakurabashi Watanabe Hospital, Osaka, Japan

**Introduction:** A delayed cardiac tamponade is a rare complication of atrial fibrillation ablation. Here we report a case of a delayed tamponade that was incidentally identified on cardiac CT 3 months post‐catheter ablation (CA).

**Methods:** N/A

**Results:** A 67‐year‐old woman was referred for radiofrequency ablation of persistent atrial fibrillation. She underwent a bilateral extensive encircling pulmonary vein isolation. During the procedure, her blood pressure dropped several times, requiring catecholamines administration. However, a postoperative echocardiogram showed no significant pericardial effusion. A chest x‐ray on the third postoperative day showed no enlargement of the cardiac silhouette, and the patient was discharged on the fourth day. On the 30^th^‐day visit post‐discharge, she exhibited no significant symptoms. The blood tests showed no anemia progression, and the ECG was normal sinus rhythm with a heart rate of 80 bpm. Starting on 70 days post‐discharge, she experienced chest discomfort when leaning forward, and by 80 days she began to develop a cough. A follow‐up cardiac CT scan on 90 days post‐discharge revealed a circumferential pericardial effusion. Her blood tests showed no anemia progression, but an electrocardiogram showed sinus tachycardia with a heart rate of 105 bpm and low‐amplitude potentials. Echocardiography revealed a circumferential pericardial effusion of up to 32 mm and the right heart system collapse during early diastole. An emergency pericardiocentesis was performed the same day, yielding 1400 ml of clear red pericardial fluid. The hemoglobin level of the pericardial fluid was low at 5.5 g/dL. After the pericardiocentesis, a small amount of serous pericardial fluid was observed, which was within the physiological range, and the patient was discharged on the 8th day without any symptom exacerbations.

**Conclusions:** This was a rare case of a very slowly progressive pericardial effusion 3 months post‐CA. The clinical presentation and pericardial fluid findings suggested that both an oozing rupture and pericarditis mechanisms were involved.

## SELECTIVE COMPLEX ATRIAL FRACTIONATED ELECTROGRAM ABLATION BASED ON THE NUMBER OF THE FRACTIONATIONS FOR REFRACTORY PERSISTENT ATRIAL FIBRILLATION

### **MASAHIRO MIZOBUCHI**, TOMOKI YAMASHITA, JUNYA MATSUMOTO, ATSUSHI FUNATSU, TOMOKO KOBAYASHI, SHIGERU NAKAMURA

#### Kyoto‐Katsura Hospital, Kyoto, Japan

**Introduction:** Previous studies have suggested that the prolonged or highly fractionated electrograms during atrial fibrillation (AF) are closely related to the reentrant driver regions. We hypothesized that exploration and ablation of these critical complex atrial fractionated electrograms (CFAE) may improve the outcome of persistent AF (PeAF) refractory to conventional PVI.

**Methods:** A total of 73 PeAF patients with residual inducibility or failed cardioversions of AF after PVI were enrolled and underwent number –of –fractionation mapping (NFM) by counting the number of fractionations in 2.5 seconds at each of the points using the CARTO3 (ICL mode) and EnSite (fractionation map) systems. After NFM, selective CFAE ablation (NFMCA) was performed as an additional procedure for refractory PeAF. We investigated the prognosis of these patients within 24 months after the index ablation procedure and the relationship between changes in activation patterns during the ablation procedure and their prognosis. We also performed a propensity score matched analysis comparing these patients with historical controls (HC) to identify the optimal indications for NFMCA.

**Results:** The AF/AT free survival rate was 79.1% at 12 months and 56.7% at 24 months. Patients with AF termination or AF cycle length prolongation ≥21ms during the procedure had significantly better AF/AT free survival rates than those without notable activation changes (87.7% vs. 69.0%, logrank p=0.028). After propensity matched analysis, AF/AT free survival showed comparable results between the two groups (1yr; NFM 72.1% vs. HC 77.1%, logrank p=0.649).

**Conclusions:** NFMCA is a versatile and less invasive adjunctive procedure for patients with PVI refractory PeAF who showed a comparable prognosis to patients with PVI compliant PeAF. In particular, remarkable activation changes during the procedure (AFCL prolongation ≥21ms or acute termination) suggest a favorable prognosis.

## LONG‐TERM OUTCOMES OF WHITE‐LINE APPROACH OF SUPERIOR VENA CAVA ISOLATION

### **YOSHIAKI MIZUNUMA**, MASAO TAKAHASHI, FUMIYA YOKOZEKI, MASANAO HOMMA, MASATAKA SUNAGAWA, WATARU TSUNO, TAKAFUMI SASAKI, KOICHIRO YAMAOKA, HIROFUMI KUJIRAOKA, TOMOYUKI ARAI, KIYOTAKA YOSHIDA, KENSUKE KASANO, RINTARO HOJO, TAKAAKI TSUCHIYAMA, SEIJI FUKAMIZU

#### Tokyo Metropolitan Hiroo Hospital, Tokyo, Japan

**Introduction:** A spontaneous right atrium (RA)‐superior vena cava (SVC) conduction block exists in some patients. We showed a novel approach for SVC isolation that uses the visualization of the RA‐ SVC conduction block as a white line with the extended early meets‐ late (EEML) tool of the CARTO system. This approach may reduce the procedure time with fewer complications. However, long‐term outcomes and the durability of SVC isolation using a white‐line approach is un‐researched.

**Methods:** Overall, 203 patients who underwent SVC isolation as the first therapy for AF or additional procedures between May 2015 and‐ April 2024 were included in this study. We created an activation map in sinus rhythm, adjusted the EEML settings, and confirmed the white line. When there was a white line in the EEML tools, we performed SVC isolation using the white line (block group, n = 145 [71.4%]). When there was no white line in the EEML tools, we performed encircling SVC isolation (non‐block group, n = 58 [28.6%]). We counted patients with AT/AF recurrence at 1‐year follow‐up. If additional procedures were needed at follow‐up, repeat sessions were performed to identify targets for treatment, including reconduction of the SVC. Then, SVC durability was compared between the two groups.

**Results:** Thirty‐three patients had AT/AF recurrence in 1 year (20/145 [13.8%] vs. 13/58 [22.4%], p = 0.098). AT/AF recurrence tended to be lower in the block group than in the non‐block group. Thirty‐six patients underwent additional procedures, and SVC isolation durability was confirmed. There was SVC re‐conduction in 11 patients (30.6%; 2/16 [12.5%] vs. 9/20 [45.0%], p = 0.067). The SVC isolation durability tended to be higher in the block group than in the non‐block group.

**Conclusions:** Our white‐line approach for SVC isolation tended to decrease AT/AF recurrence and maintain SVC isolation durability.

## PALPITATIONS IN A 10 YEARS OLD

### **IZYAN MOHAMMAD**, SOFIAN JOHAR

#### RIPAS Hospital, Bandar Seri Begawan, Brunei Darussalam

**Introduction:** Andersen‐Tawil Syndrome or Long QT syndrome (LQTS) Type 7 is a rare arrhythmogenic condition that typically presents with neurological symptoms or dysmorphism. Here we present a case of a 10 year old female diagnosed with Andersen‐Tawil syndrome whose main presentation was palpitations.

**Methods:** N/A

**Results:** Our patient presented through emergency department with 1 month history of palpitations and occasional chest pain. She denied any syncope or neuromuscular weakness. Her admission ECG revealed frequent premature ventricular complexes (PVC) in bigeminy pattern with U waves and QTc of 526ms. Subsequent echocardiogram was normal and exercise stress test did not show worsening arrhythmia. Holter study revealed 29% PVC burden with frequent non‐sustained ventricular tachycardia episodes. Her physical examination and initial blood test investigations were unremarkable. Further history revealed that her mother had received ablations for frequent PVC and clinodactyly. Genetic studies later revealed KCNJ2 gene pathogenic variant which confirmed her diagnosis for Andersen‐Tawil Syndrome. She was started on propranolol which settled her symptomatically. Subsequent ECG on follow‐up showed improvement of QTc to 440ms.

**Conclusions:** Our case illustrates challenges in diagnosing patient with Andersen‐Tawil Syndrome given absence of dysmorphia and neuromuscular symptoms. Presence of family history supported this diagnosis which was subsequently confirmed with genetic study.

## CURRENT OF INJURY DETECTED ON THE RIGHT VENTRICULAR SEPTAL SURFACE BEFORE SUCCESSFUL LEAD DEPLOYMENT FOR LEFT BUNDLE BRANCH AREA PACING

### **TAKATO MOHRI**, TOSHIAKI SATO, HIROTSUGU IKEWAKI, YUMI KATSUME, MIKA TASHIRO, NORIKO NONOGUCHI, KYOKO HOSHIDA, IKUKO TOGASHI, SATORU NISHIO, AKIKO UEDA, SEIICHIRO MATSUO, KYOKO SOEJIMA

#### Kyorin University Hospital, Tokyo, Japan

**Introduction:** Left bundle branch area pacing (LBBAP) captures the left ventricle (LV) by transeptal lead deployment. This study aimed to identify an initial site on right ventricular (RV) septum for successful transeptal deployment by evaluating EP parameters.

**Methods:** Ninety‐four patients with atrioventricular block underwent successful LBBAP. During deployment of the lumenless lead, unipolar impedance, amplitude of current of injury (COI) of the unfiltered ventricular electrogram and other EP parameters were evaluated. The lead tip location was identified by repeated injections of contrast material or judged by the appearance of a late R‐wave in lead V6.

**Results:** In total, 206 attempts were made to deploy the LBBAP lead in 94 patients (infranodal block, 59%; QRSd, 120 ± 27 ms; LVEF, 64 ± 7%). Following 94 successful trials, LBBAP was achieved with a stimulus‐to‐peak of R‐wave (RWPT) in V6 of 76 ± 8 ms. The COI amplitude increased from 0.44 ± 0.21 mV on the RV surface before deployment to 0.71 ± 0.20 mV in LV after that (p<0.001). After 91 attempts, a lead tip did not reach LV with no late R‐wave appearing in V1. The helix was trapped on the RV surface after 46 attempts or remained in the RV septum after 45 attempts. Before these failed attempts, on the RV surface, the COI amplitude (0.28 ± 0.24 mV) was significantly lower than that of successful trials (p <0.01). Unipolar impedance before failed attempts was lower than that before successful trials (736 ± 189 and 830 ± 160 Ω, respectively, p <0.01) and RWPT before failed attempts was longer than that before successful trials (101 ± 14 and 95 ± 14 ms, respectively, p<0.01). Multivariate analysis showed that the COI amplitude was related to the successful trial (OR,1.05; 95%CI,1.02‐1.08; p<0.01). Analysis of the ROC curve determined the COI amplitude of 0.28 mV to be an optimal value for identifying the successful trial (specificity,0.78; sensitivity,0.68; AUC,0.77).

**Conclusions:** Elevated COI was detected on the RV surface before the successful placement of LBBAP lead. Intracardiac ventricular electrogram should be evaluated as a marker of the initial site of LBBAP lead deployment.

## OPTIMAL STRATEGY AND RISK FACTORS OF CATHETER ABLATION FOR ATRIAL FIBRILLATION IN PATIENTS WITH HISTORY OF ACUTE DECOMPENSATED HEART FAILURE ADMISSION

### **KAZUKI MORI**, AYAKA YOSHIHARA, TOMOARI KURIYAMA, YUKIKO SHIMIZU, KAZUTO KUJIRA, YUKIHITO SATO

#### Hyogo prefectural Amagasaki General Medical Center, Amagasaki city, Japan

**Introduction:** Recent clinical trials have demonstrated catheter ablation for atrial fibrillation(AF) patients with heart failure(HF) improved prognosis. However, the clinical efficacy and optimal ablation strategy in unstable patients with history of admission with decompensated HF still remains unclear.

**Methods:**

From January 2019 to March 2022, consecutive 693 patients with HF and AF who were admitted to our institution due to acute decompensated heart failure were enrolled. 73 patients (47 male sex, mean age 69.9+/‐10.8 years old and mean 12.0 days of hospitalization) were performed initial catheter ablation for AF after HF admission were analyzed. Patients with congenital heart diseases or who had ablation for other arrhythmias were excluded.

**Results:** All patients were performed pulmonary vein(PV) isolation and cavotricuspid isthmus block, 30 patients were performed extra PV trigger and adjunctive linear ablation each physician's discretion. Twenty eight of 73 (38.4%) patients had atrial arrhythmia recurrence and 8(11.0%) patients had re‐admission for HF. Arrhythmia recurrence was associated with re‐admission (odds ratio 5.8, p=0.048). Multivariate analysis showed that high left atrial pressure(e/e’>14) was significantly associated with AF recurrence (odds ratio 3.44(1.16‐11.6,95%CI), p=0.002), but ablation procedure was not correlated with clinical outcomes.

**Conclusions:** Among patients with history of decompensated HF admission, arrhythmia recurrence was associated with HF re‐admission and high left atrial pressure was a risk factor of recurrence. An optimal ablation strategy was not suggested in this study.

## SUPERIOR VENA CAVA ISOLATION WITH A 51W‐HIGH POWER 4‐SECOND INTELLIGENT TEMPERATURE‐CONTROLLED ABLATION USING THE QMODE PLUS ALGORITHM: SAFETY AND FEASIBILITY

### **ITSURO MORISHIMA**, YASUNORI KANZAKI, HIROYUKI MIYAZAWA, KAZUKI SHIMOJO, YASUHIRO MORITA, NAOKI WATANABE, NAOKI YOSHIOKA, NAOKI SHIBATA, YOSHIHITO ARAO, TAKUMA OHI, HIROKI GOTO, HOSHITO KARASAWA, YUTA NAKAGAWA, TATSUKI YOSHIE, YUKI KAWASAKI

#### Ogaki Municipal Hospital, Ogaki, Japan

**Introduction:** Because a superior vena cava (SVC) isolation can be challenging due to the lesion's proximity to the phrenic nerve (PN) and sinus node (SN), the ablation strategy remains unsettled. The 51W 4‐second temperature‐controlled (51W/4S‐TC) ablation using the QMode plus algorithm may simplify the procedure and provide an optimal SVC isolation.

**Methods:** We studied 50 consecutive patients who underwent an SVC isolation with a QMode plus 51W/4S‐TC ablation (age, 70±9 years old; male, 78%; paroxysmal atrial fibrillation, 40%). The ablation strategy included 3 steps. First, a local activation time map of the SVC and right atrium (RA) was created to identify the SN breakout site. Second, high current pacing along the lateral portion of the RA‐SVC junction was delivered to locate the area of the PN stimulation (PNS). Finally, a circumferential 51W/4S‐TC ablation with a 4mm inter‐lesion distance was performed starting 10mm above the SN breakout site (Figure).

**Results:** The SVCs were isolated in all patients with a first‐pass success in 46 patients (92%). The average procedure time was 9.2±5.3 minutes. The average length of the circumferential ablation line was 58.0±13.7mm. PNS was observed in 38 (76%) patients with a PNS length of 9.0±4.2mm, which was commonly located on the posterolateral wall. The total number of radiofrequency applications was 20.2±4.3, of which 2.9±1.7 applications were given in the PN capture region. The average contact force was 10.8±1.5g. No SN injury or PN palsy was observed. Asymptomatic transient mild PN injury occurred in one patient as demonstrated by a right diaphragmatic elevation of more than half the height of one vertebra on the chest X‐ray one day post‐procedure.

**Conclusions:** The 51W/4S‐TC ablation using the QMode plus algorithm may be a simple, safe, and efficient procedure for an SVC isolation.

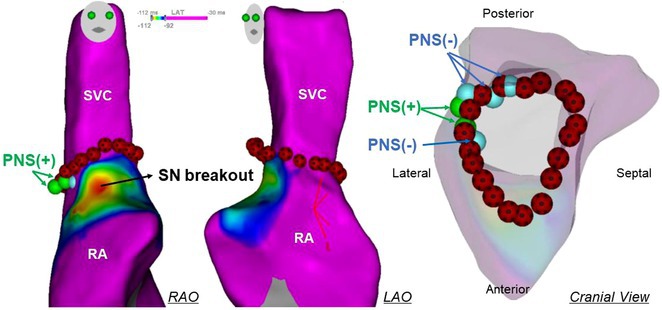

## SINUS NODE DYSFUNCTION WITH ATRIOVENTRICULAR BLOCK POST‐PACEMAKER IMPLANTATION

### **JUNJI MORITA**, YUHEI KASAI, TAKAYUKI KITAI

#### Sapporo Cardio Vascular Clinic, Sapporo City, Japan

**Introduction:** There are two types of leadless pacemakers suitable for atrioventricular block: Micra™ AV (Medtronic) and Aveir™ DR (Abbott). The difference between these two lies in their pacemaker settings: Micra™ AV functions as VDD, while Aveir™ DR operates as DDD. However, concurrent sinus node dysfunction (SND), especially during complete atrioventricular block, poses a diagnostic challenge. In this study, we investigated the predictors for concomitant SND in patients with atrioventricular block post‐pacemaker implantation.

**Methods:** We recruited patients who underwent pacemaker implantation for atrioventricular block with a DDD setting and a lower rate of 60. The atrial pacing rate was measured the next day and 1 year later. An atrial pacing percentage ≥40% was defined as the SND group.

**Results:** Precisely 86 patients completed the protocol. The median atrial pacing percentage was 6.5% on the day after implantation and 23.6% 1 year later (p=0.089). The prevalence of SND was 33.7% (29 patients) at 1 day post‐implantation and 31.4% (27 patients) at the 1‐year follow‐up (p=0.762). A multivariate analysis confirmed that the presence of paroxysmal atrial fibrillation at the time of implantation was an independent predictor of SND concomitance after 1 year (p=0.017, OR 5.04).

**Conclusions:** At the 1‐year follow‐up after post‐pacemaker implantation for atrioventricular block, the atrial pacing percentage was not rare and nearly one‐third of the patients concurrently had SND. Paroxysmal atrial fibrillation at the time of implantation was an independent predictor for the occurrence of SND post‐implantation.

## EFFICACY OF THE INTRACARDIAC ECHOCARDIOGRAPHY FOR CAVO‐TRICUSPID ISTHMUS ABLATION

### **TSUKASA MOTOYOSHI**, SHINTARO YAMAGAMI, TOMOHIRO SATO, TOYOKI OKUDA, MASAYA AKIYAMA, KAZUSHIGE SHIMIZU, SADANORI SHIMIZU, SOSUKE SUGIMURA, YUTA NAKANO, MASANORI MURAYAMA, HIROKAZU KONDO, TOSHIHIRO TAMURA

#### Tenri Hospital, Nara, Japan

**Introduction:** Radiofrequency ablation (RFA) of the cavo‐tricuspid isthmus (CTI) is one of the most frequently performed procedures with a high success rate. However, to determine the optimal site of the block line for CTI ablation could be unusually challenging in some cases due to the abnormal rotation of the heart. We hypothesized that intracardiac echocardiography (ICE)‐guided CTI ablation could be performed over a shorter distance compared to the conventional fluoroscopy‐only CTI ablation.

**Methods:** We analyzed 40 consecutive cases who underwent CTI ablation between July 2023 and May 2024 at Tenri hospital (20 ICE‐guided CTI ablation cases [ICE‐guided group]and 20 conventional fluoroscopy‐only CTI ablation cases [conventional group]).

**Results:** No significant differences in characteristics were found between the ICE‐guided group and the conventional group. The length (mm) of the block line was significantly shorter in the ICE‐guided group (30.0±2.20 vs 39.6±1.41 P=0.0013). Total number of RF applications, total ablation times (ms) and procedure times (ms) were comparable between the 2 groups (16.3±5.62 vs. 15.9±5.86, p=0.88,553±190 vs. 539±222, p=0.89 and 835±353 vs. 916±558, p=0.76, respectively).

**Conclusions:** ICE‐guided CTI ablation significantly shortens the length of the block line compared to the conventional fluoroscopy‐only CTI ablation.

## HIGH‐RATE ATRIAL PACING DURING RADIO FREQUENCY CATHETER ABLATION OF TYPICAL ATRIOVENTRICULAR NODAL RE‐ENTRANT TACHYCARDIA: IS IT A PREDICTOR OF SUCCESSFUL AND SAFE ABLATION?

### AHMED MOUSTAFA

#### cairo university, 99 ‐ Etranger, Egypt

**Introduction:** Radiofrequency (RF) ablation of slow pathway (SP) is usually performed in sinus rhythm while monitoring the occurrence of a slow junctional rhythm (JR). JR although sensitive, is not specific for elimination of SP conduction. Our objective was to prospectively evaluate feasibility and safety of SP elimination using fast atrial rate pacing (FAP) during RF delivery.

**Methods:** Consecutive patients admitted for atrioventricular nodal re‐rentrant tachycardia (AVNRT) ablation were included. The rate of proximal coronary sinus (CS) pacing was set to a value constantly yielding antegrade SP conduction, while carefully monitoring the AH interval. RF delivery (at the lower part of Koch's triangle) was considered successful if there were AH shortening or if transition from Wenckebach (WK) periods to a 1:1 conduction occurred.

**Results:** 40 patients were included (38 ± 7 y) divided equally into two groups. Typical AVNRT was induced in all. Regarding group I RF delivery during CS pacing (300 ± 40 ms) led to AH shortening by 88 ± 21 ms. Waiting time,Fluorouscopy time and procedure time were less in group I. In one patient a femoral hematoma during sheath insertion has occurred in both groups. Non‐inducibility and SP conduction disappearance was obtained in all patients. No patient developed AV block. After a follow‐up of 12 ± 3 months, no recurrences were observed in both groups.

**Conclusions:** The new method for ablation of the slow pathway during high rate atrial pacing isn't inferior to the traditional conventional method and allows direct visualization of its disappearance. The procedure time and the waiting time have been reduced in patients undergone ablation by the new method significantly

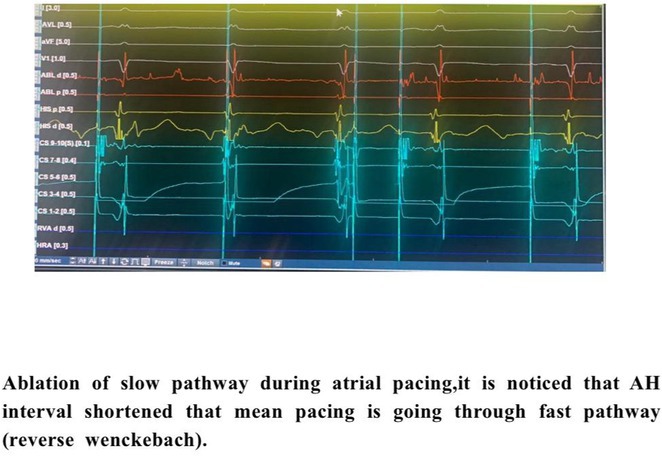

## REFRACTORY ATRIAL TACHYCARDIA MIMICKING ATYPICAL ATRIAL FLUTTER WITH VARIABLE BLOCK ORIGINATING FROM LEFT SUPERIOR PULMONARY VEIN

### **RADITYA RIZKI MUHAMMAD**, ADRA ACHIRULTAN RAMAINALDO SUGIARTO, RERDIN JULARIO, SHINTA DEWI RASTI, BUDI BAKTIJASA DHARMAJATI, RAGIL NUR ROSYADI, MAKHYAN JIBRIL AL FARABI, RUTH IRENA GUNADI, YOSUA HENDRIKO MANURUNG, MOHD. ZULKHAIRI RHIZA Z. TALA

#### Universitas Airlangga ‐ Dr Soetomo General Hospital, Surabaya, Indonesia

**Introduction:** Atrial Tachycardia (AT) have been demonstrated to arise from both atria and other surrounding structures. Its origin from left side atria, particularly in pulmonary vein (PV), is less frequent.

**Methods:** N/A

**Results:** A 41‐year‐old woman was referred to our emergency department with refractory and recurrent supraventricular tachycardia (SVT). ECG revealed narrow complex tachycardia with a rate of 166 beats per minutes, recognized as atrial flutter (AFL) with variable block (1:1‐2:1). The patient was treated with amiodaron, beta blocker, and synchronized cardioversion. After recalcitrant SVT, she was implanted with a temporary pacemaker as atrial overdrive pacing. On physical examination, the patient was classified as overweight (BMI 25.3 kg/m2). The vital signs were normal, except for the increased heart rate. Laboratory and echocardiography results within normal ranges. The woman has had intermittent palpitations for the past 12 years, intensified over the last 3 years particularly during heavy activity. The patient underwent an Electrophysiology study (EPS) which revealed non‐inducible tachyarrhythmia and normal function of the SA and AV nodes. However, complaints of palpitations still continue to recur. Hence, EPS with 3D activation mapping was performed. The P‐wave from surface ECG showed positive at V1 and bifid at lead II, suggestive of AT locating at left PV or left atrial appendage. Orion Cathether was placed in both atria for 3D activation mapping. The earliest activation was at Left Superior Pulmonary Vein (LSPV), thus radiofrequency ablation was applied there and the rhythm converted into sinus during ablation. AT often appeared with variable conduction. In such case, the variable conduction may occur because of the Wenckebach periodicity and the effect of anti‐arrhythmic drugs as AV Node blocker.

**Conclusions:** AT with variable conduction can be challenging to be differentiated from AFL. Besides, it is often refractory with anti‐arrhythmic drugs. Cathether ablation with 3D activation mapping can effectively revealed origin and offers a high acute as well as long‐term success rate.

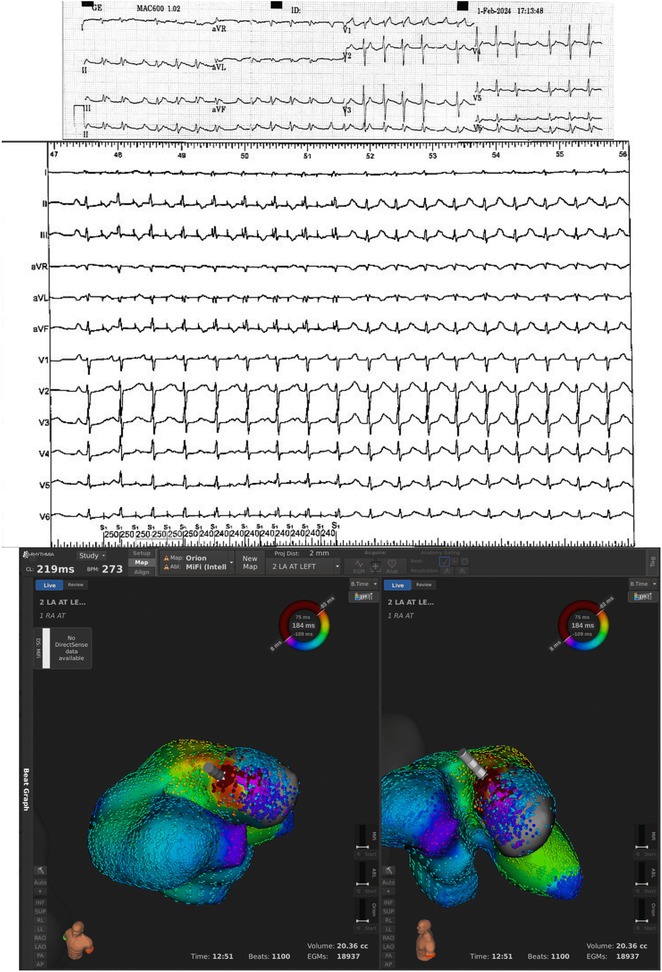

## UNEXPECTED PACEMAKER IMPLANTATION FOR SINUS NODE DYSFUNCTION AFTER ATRIAL ARRHYTHMIA ABLATION IN AN ADULT WITH REPAIRED TETRALOGY OF FALLOT

### **RADITYA RIZKI MUHAMMAD**, ADRA ACHIRULTAN RAMAINALDO SUGIARTO, KEN CHRISTIAN KAWILARANG, MOHD. ZULKHAIRI RHIZA Z TALA, RERDIN JULARIO, BUDI BAKTIJASA DHARMAJATI, RAGIL NUR ROSYADI, MAKHYAN JIBRIL AL FARABI, RUTH IRENA GUNADI, SHINTA DEWI RASTI

#### Universitas Airlangga ‐ Dr Soetomo General Hospital, Surabaya, Indonesia

**Introduction:** Arrhythmias remain a major cause of morbidity and mortality for adults with repaired Tetralogy of Fallot (TOF). Atrial arrhythmias (AA) were presented in 34% of those cases. Sinus node dysfunction (SND) and AA frequently occur together and may perpetuate each other.

**Methods:** N/A

**Results:** A 30‐year‐old female was referred with increasing shortness of breath over the past week and palpitations, which both was exacerbated by physical activity. The patient had a history of TOF that was surgically repaired when she was 3.5 years old and there had been no significant complaints thenceforward. On physical examination and vital sign, all findings were within normal limits, except for grade 1 obesity (BMI 33.3 kg/m2) and elevated heart rate of 136 beats per minute. ECG revealed a sinus tachycardia rhythm with complete RBBB. Echocardiography revealed dilation of the right atrium and right ventricle, with normal ejection fraction. Supraventricular tachycardia were the most notable finding on Holter monitoring, specifically atrial tachycardia, or possibly atrial flutter (AFL) as a differential diagnosis. Electrophysiology study (EPS) with 3D activation mapping was performed. Atrial burst pacing was given, then AA was induced with 3:1 conduction and the P‐wave showed negative at inferior lead but positive at V1, suggesting for typical AFL. Radiofrequency ablation was applied at Cavo Tricuspid Isthmus (CTI) area and the rhythm converted into sinus. A short time later, junctional rhythm suddenly observed, hence SND were suspected and single‐chamber atrial permanent pacemaker was implanted. AA and cardiac conduction disorder system may occur attributable to atrial scarring, structural and electrophysiological remodelling, or surgical injury from the TOF repair.

**Conclusions:** In such case, recognition of the underlying pathology is important to enhance our ability to design better therapeutic strategies. Catheter ablation for AFL in adult patients with surgically repaired TOF had a high success rate, besides using atrial pacemakers to treat SND is the key recommendation, henceforth could improve symptoms.

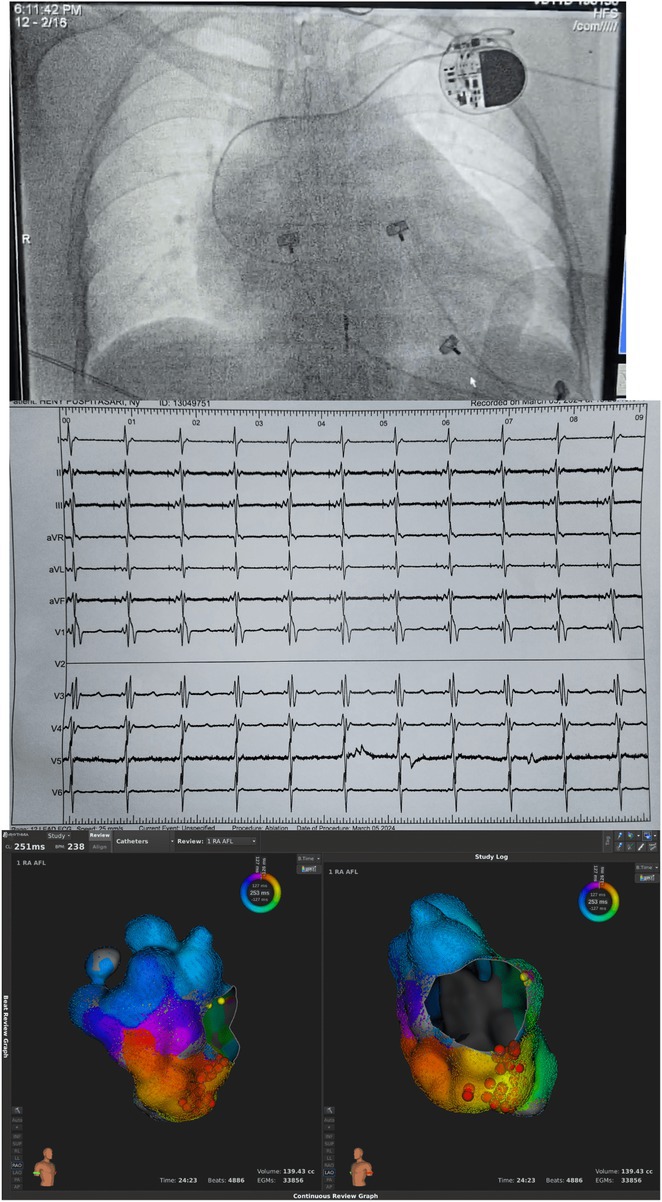

## CHARACTERISTICS AND ABLATION OUTCOMES OF RECURRENT ATRIAL TACHYARRHYTHMIA AFTER MAZE OPERATION ‐A MULTICENTER STUDY IN FUKUOKA

### **YASUSHI MUKAI**, SHUNSUKE KAWAI, HIROHIDE MATSUURA, ARIHIDE OKAHARA, MASAKI TOKUTOME, KIYOHIRO OGAWA, RYUICHI MATSUKAWA

#### Japanese Red‐Cross Fukuoka Hospital, Fukuoka, Japan

**Introduction:** Catheter ablation of atrial tachyarrhythmia after Maze operation is challenging. Purpose of the present study was to clarify the characteristics and ablation outcomes of recurrent atrial tachyarrhythmia after Maze operation

**Methods:** Twenty‐eight cases who underwent catheter ablation of post‐Maze procedure atrial tachyarrhythmia (42 sessions; 1.5 per patient) in our 5 teaching affiliate hospitals were retrospectively analyzed.

**Results:** Cox‐IV Maze procedure and left atrial Maze were performed in 19 cases and 5 cases, respectively. Median interval from surgery to index ablation procedure was 35 months. In total, 46 atrial tachyarrhythmias were studied. Atrial tachycardia (AT) was the most common (n=36), whereas 4 atrial fibrillation (AF) and 2 focal AT were also observed. Identified tachyarrhythmia circuits were as follows; 16 mitral AT, 9 left atrial localized reentry (4 septal, 3 posterior, 1 left atrial appendage, 1 anterior), 6 right atrial lateral incision‐related AT, 5 cavo‐tricuspid isthmus (CTI)‐dependent AT, 3 roof‐dependent AT, 2 right atrial localized reentry (1 coronary sinus (CS), 1 CTI), 2 focal AT (1 para His, 1 CS), 1 bi‐atrial AT, 1 pulmonary vein tachycardia, and 1 slow‐fast AVNRT. Termination of targeted tachyarrhythmia was achieved in 34 sessions (81%). AT/AF recurrence free rate at 12, 24, 36 months of follow‐up were 91.8%, 81.6%, and 65.3%, respectively. Seven cases underwent multiple sessions (2 2^nd^ sessions, 3 3^rd^ sessions, 2 4^th^ sessions). In these cases, de‐novo atrial tachyarrhythmias were detected.

**Conclusions:** Most of the tachyarrhythmias after Maze operation were incision/gap‐related ATs, among which mitral AT and LA localized reentry were the most prevalent. Although these challenging tachyarrhythmias can be treated with the contemporary mapping and ablation techniques, de‐novo tachyarrhythmias can emerge in a remote period.

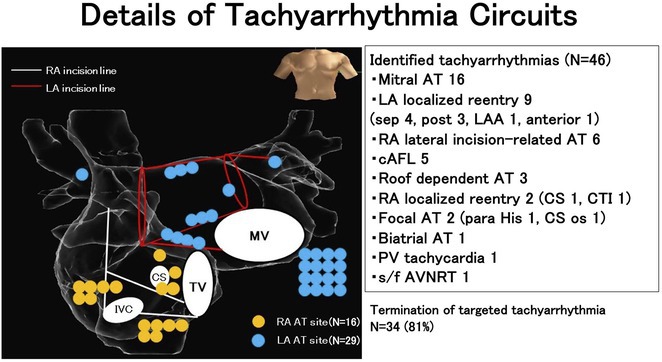

## A PARAHISIAN TACHYCARDIA ABLATION DONE VIA NON CORONARY CUSPS USING ZERO FLUROSCOPY

### **ABHILASHA MUNISINGH**, MURALIDHARAN THODDI RAMAMURTHY, PREETAM KRISHNAMURTHY, PRASANNA SUBBARAJU, R. SWAMINATHAN VEERASAMY, VASUNDHARA PONNAGANTI, LOGAVENGATESH KAVINDAPADI VAJRAVELUSWAMI

#### SRM Medical College Hospital and Reasearch Center, Chennai, India

**Introduction:** A zero fluro 3D mapping and ablation done for a challenging parahisian tachycardia from the non coronary cusp focus, in a patient presenting with a narrow complex tachycardia.

**Methods:** N/A

**Results:** A 61 year female presenting with recurrent episodes of palpitations lasting for a duration of 10 mins to 30 mins. A hemodynamically stable patient, ECG recorded narrow complex tachycardia ‐short RP tachycardia, which responded to 2 doses of IV adenosine. Aafter baseline investigations and an informed consent, patient was taken up for electrophysiological study and ablation using zero fluro and ensite 3D NAV X system. Mapping was done placing a decapolar catheter in coronary sinus and a quadripolar catheter in his position, baseline intervals were normal. Programmed electrical stimulation induced tachycardia with no AH jump, placing the quadripolar catheter placed in RV pes showed no VA conduction. Tachycardia induced with programmed electrical stimulation s1:350 ms; s2:300 ms on isoprenaline a long RP tachycardia with AHV pattern noted. Septal VA 230 ms, tachycardia cycle length 340 ms, AH 86 ms HV 50 ms. entrainment of right ventricle showed dissociated va and entraiment of right atrium showed no VA linking during tachycardia.

**Conclusions:** An ectopic atrial tachycardia was contemplated, using second decapolar catheter placed in the right atrium mapping of the tachycardia. Earliest a signal was noted to be close to the parahisian region, mapping of the non coronary cusp was contemplated, using a 4 mm ablation catheter and mapping done. The earliest a in NCC was found on the time with a in his bundle, ablation done 30 watts 60 degree celsius 120 ms. Tachycardia terminated with in 7 seconds of ablation initiation. none inducibiltiy of tachycardia was demonstrated. Post RFA were within normal limits in a parahisian subgroup of tachycardia its close proximity to the conduction system is a challenge for any electrophysiologist to tackle, which in this case was done effectively by using zero fluro 3D ensite mapping and ablation.

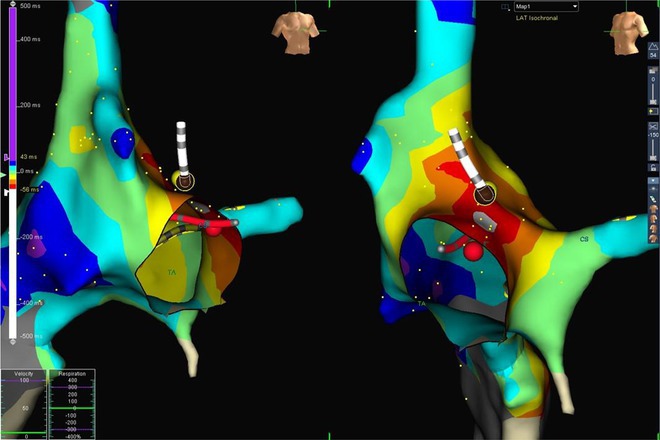

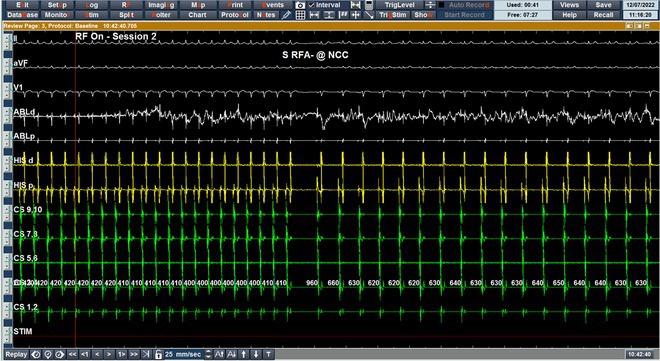

## MAPPING AND ABLATION OF COMPLEX RE‐RENTRY TACHYCARDIA IN PATIENTS RECEIVING THE SENNING PROCEDURE

### **MATHAN MUNUSAMY**, ROHITH STANISLAUS, SURAYA HANI KAMSANI, AZLAN HUSSIN

#### National Heart Institute, Kuala Lumpur, Malaysia

**Introduction:** The Senning procedure was introduced as palliative strategy for patients with transposition of great arteries (TGA). Now, these procedures are part of a complex surgery aimed to repair congenitally corrected TGA (CCTGA). These patients frequently present later with atrial tachyarrythmias which may prove difficult to control with medications. We present our experience of electrophysiology study, transbaffle puncture and radiofrequency ablation (EPS/RFA) in a cohort of 4 patients, all of whom received Senning procedure.

**Methods:** N/A

**Results:** In all 4 of the patients, the common tachyarrhythmia was atrial flutter. Senning procedure provides a technical complexity to the conduct of EPS/RFA as the systemic veins are redirected towards the anatomical left atrium. As a result, the anatomical right atrium which is now the pulmonary venous atrium must be accessed by trans‐baffle puncture. In the first two patients, we performed CT scan and 3D print of the heart model to achieve familiarity with the complex anatomy. Using the 3D printed model, we could simulate catheter behaviour inside the cardiac chambers as well as guiding trans‐baffle puncture. Mapping of the atrium was performed using the Advisor HD Grid Mapping Catheter with 3D electroanatomical mapping using the Abbott Ensite X system. Transbaffle puncture was performed using the BRK‐1 transeptal puncture needle. The use of 3D electroanatomical map accurately predicted the critical isthmus of the atrial flutter in all 4 patients and targeted ablation proved successful.

**Conclusions:** We have demonstrated effective usage of the 3D electroanatomical mapping technology in the successful ablation of atrial arrhythmia in this complex anatomy. These patients are at a high risk to develop recurrent arrhythmias, it is imperative to embrace new technologies that increase the success of ablation procedures.

## OPEN WINDOW MAPPING (OWM) IN PAEDIATRIC ATRIOVENTRICULAR RECIPROCATING TACHYCARDIA (AVRT) MAPPING

### **MATHAN MUNUSAMY**, ROHITH STANISLAUS, SURAYA HANI KAMSANI, AZLAN HUSSIN

#### National Heart Institute, Kuala Lumpur, Malaysia

**Introduction:** Catheter ablation is the treatment of choice for patients with symptomatic accessory pathway (AP). Point‐by‐point mapping of AP to identify local EGM and its origin may prove inefficient or yield inadequate information. Recently, open window mapping (OWM) offers a novel and quick technique to locate these AP, although data on paediatric ablations remains scarce. We report our experience using OWM for ablation of paediatric AVRT.

**Methods:** Between 2021 to March 2024, we have performed 23 OWM for 20 paediatric patients with AVRT. Focused high density maps were created using multielectrode mapping catheter and EnSite Precision (Abbott) 3D mapping system. OWM protocol was used to annotate the sharpest local signal at each point and to delineate the location of AP.

**Results:** The average age of patients was 10.05 with 50% being males. 16 patients had right sided AP (80%), 2 had left sided pathways (10%) and 2 had both right and left pathways (10%). 4 patients had underlying Ebstein malformation (20%), with all of them having right sided AP. Average procedure time for left AP was 108.75 mins vs 83.16 mins for right AP( *p*=0.204). Average fluoroscopy time for left AP was 31.06 vs 19.03 mins for right AP ( *p*=0.025). All patients had the AP successfully mapped using the OWM. Overall, APs were successfully ablated in 95.0% (n=19) with three cases not attempted due to the AP's paraHisian location. Three patients experienced recurrence of the delta wave, with two successfully abolished in repeat procedure.

**Conclusions:** To our knowledge, this is the largest cohort to investigate utility of OWM in ablation of paediatric AVRT. Our data supports the utility of OWM for efficient AP ablation procedures in paediatric population with a success rate comparable with previous published data.

## PROGRAMMED VENTRICULAR STIMULATION (PVS) IN PATIENTS WITH REPAIRED TETRALOGY OF FALLOT (TOF) BEFORE PERCUTANEOUS PULMONARY VALVE IMPLANTATION (PPVI)

### **MATHAN MUNUSAMY**, ROHITH STANISLAUS, SURAYA HANI KAMSANI, AZLAN HUSSIN

#### National Heart Institute, Kuala Lumpur, Malaysia

**Introduction:** Adults with repaired Tetralogy of Fallot (TOF) are at risk of ventricular arrhythmias (VA) and pulmonary regurgitation (PR) mandating a pulmonary valve replacement (PVR). Many centers recommend routine electrophysiological study (EPS) with programmed ventricular stimulation (PVS) to identify at risk patients undergoing percutaneous PVR. The rationale for this is that the pulmonary prosthesis may cover the infundibular septum and interfere with later attempts for ablation.

**Methods:** N/A

**Results:** The 1^st^ patient is a 21‐year‐old male who received TOF repair at age of 6 and complicated by complete heart block requiring a dual chamber pacemaker. His pacemaker interrogation revealed non‐sustained ventricular tachycardia (NSVT). We subjected him for PVS and performed substrate mapping using Ensite Precision (Abbott) high density 3D electroanatomical mapping and identified deceleration zones at the posteroseptal RVOT. Post ablation, the conduction speeds were more homogenized with wave front collisions. The second patient is a 19‐year‐old gentleman who had TOF repair at age of 3 and presented with palpitations. He had a prior anterolateral RVOT VT ablation done in 2016. Prior to PVR, we performed another EPS for him and easily induced VT. RVOT mapping identified slowly conducting isthmus over a large area from anterior to posterior RVOT. We created a line of ablation across these isthmuses and no further tachycardia was inducible.

**Conclusions:** These result supports performing EPS and PVS in at‐risk patients prior to PPVI. In our center, we offer targeted PVS for candidates deemed at higher risk of developing VA. These include symptomatic palpitations, evidence of VT or NSVT on follow up, poor ventricular function, QRS duration > 180 msec and/or fibrosis on magnetic resonance imaging.

## VENTRICULAR TACHYCARDIA ASSOCIATED WITH CARDIAC FIBROMA AS AN INHERITED MANIFESTATION OF GORLIN SYNDROME

### **HIROSHIGE MURATA** ^1^, TAKASHI NITTA^1^, SHINOBU KUNUGI^1^, HIDEMORI HAYASHI^2^, WATARU SHIMIZU^1^, KUNIYA ASAI^1^, YU‐KI IWASAKI^1^

#### ^1^Nippon Medical School, Tokyo, Japan,^2^Juntendo University, Tokyo, Japan

**Introduction:** Nevoid basal cell carcinoma syndrome, also referred to as Gorlin syndrome, is a rare autosomal dominant disease characterized by odontogenic keratocysts, basal cell carcinoma and predisposed to cardiac fibromas. Here, we show a rare case of ventricular tachycardia (VT) associated with inherited cardiac fibroma (CF).

**Methods:** N/A

**Results:** A 15‐year‐old male patient presented with syncopal attacks caused by VT storm associated with 27×25‐mm large CF, revealed by cardiac MRI. He was transferred to our hospital for surgical treatment of the VT storm that was refractory to amiodarone and repetitive endocardial catheter ablations. Preoperative endocardial mapping showed an extensive low voltage zone (LVZ) in the posterior wall of the left ventricle, suggesting the infiltration of CF into the myocardium. Two different morphologies of VT were induced by the programmed ventricular stimuli: VT#1 (RBBB, left axis deviation, CL 300 ms) originating from the postero‐septal side of the LVZ and VT#2 (RBBB, right axis deviation, CL 300 ms) originating from the basal‐lateral side of the LVZ. Intraoperative electroanatomical mapping during induced VTs showed a centrifugal activation pattern originating from the border between the tumor and normal myocardium, where fractionated potentials were observed during sinus rhythm. Because a complete resection of the tumor was unfeasible, encircling surgical cryoablation was performed to isolate the tumor that resulted in suppression of the VT storms. Histopathological studies of specimens acquired from the earliest activation sites of VTs revealed tumor infiltration into the surrounding myocardium with cell disorganization, exhibiting myocardial disarray.

**Conclusions:** The mechanism underlying CF‐related VT is a localized re‐entry, wherein the circuits are located at the tumor boundaries, and myocardial disarray associated with tumor infiltration is a substrate of the VT. Map‐guided encircling cryoablation to isolate the tumor is a therapeutic option for this form of VT.

## AN EXPERIMENTAL STUDY COMPARING LESION FORMATION BY NONOCCLUSIVE CRYOBALLOON ABLATION BETWEEN POLARX AND ARCTIC FRONT ADVANCE PRO AND THE DURABILITY OF THE LESIONS CREATED BY LEFT ATRIAL ROOF LINE

### **KAZUYA MURATA**, YUMI YASUI, ATSUHITO ODA, HIROHUMI ARAI, YUICHIRO SAGAWA, YASUTERU YAMAUCHI

#### Yokohama City Minato Red Cross Hospital, Yokohama, Kanagawa, Japan

**Introduction:** Pulmonary vein (PV) isolation and linear ablation with a left atrial (LA) roof line can be performed using a cryoballoon (CB) in atrial fibrillation (AF). The differences between the two CBs, Arctic Front Advance‐pro (AFA) and POLARx, are unclear. This study investigated the long‐term durability of LA roof‐line lesions and the differences between the two CBs.

**Methods:** We performed an ex vivo experiment using ablation novel models made of konjac, which turn red at ‐20°C, allowing lesion visualization after CB ablation. We performed cryoablation for 3 minutes by pressing the northern hemisphere of the CB against the model in a water tank. In clinical research, 67 consecutive patients (mean age 65.3 ± 10.9 years, 64.2% male, 10.4% paroxysmal AF, 41.8% longstanding persistent AF (L‐PeAF), 10.3% POLARx) who underwent first‐time ablation with PV isolation and LA roofline with CB were retrospectively analyzed after recurrence and second‐time ablation.

**Results:** In the ex vivo experiment, the lesion depth was significantly greater with POLARx (POLARx: 5.80 ± 0.29mm vs. AFA: 4.34 ± 0.32mm, P < 0.001). In the clinical research, 20 patients (29.9%) showed LA roofline reconnection, and 39 patients (57.4%) showed PV reconnection. Additionally, 17 patients (25.4%) had recurrent atrial tachycardia, of which 4 patients had roof‐dependent atrial tachycardia. LA roofline reconnection occurrence was associated with LA volume by CT (190.1 ± 66.5 cc vs. 147.4 ± 35.5 cc, P = 0.02), and with roof touch‐up radiofrequency ablation (5/20 (25.0%) vs. 2/47 (4.3%), P = 0.01). Also, L‐PeAF resulted in significantly more reconnection (13/28 (46.4%) vs. 7/39 (18.0%), P = 0.01). The POLARx had a higher percentage of roofline durability, but the difference was not significant (AFA 70.6%, POLARx 85.7%, P = 0.396).

**Conclusions:** Non‐occlusive cryoballoon ablation with POLARx can create deeper lesions, which may be advantageous in LA roof ablation for persistent AF. LA roof durability with CB was relatively high, and the probability of roof‐dependent atrial tachycardia was relatively low.

## LEFT ATRIAL APPENDAGE SIZE AND MORPHOLOGY TYPE IN ASIAN PATIENTS

### **TSUKASA NAGANUMA**, WATARU SASAKI, MASATAKA NARITA, KAZUHISA MATSUMOTO, NAOMICHI TANAKA, HITOSHI MORI, DAISUKE KUDO, YOSHIFUMI IKEDA, TAKAHIDE ARAI, SHINTAROU NAKANO, KAZUO MATSUMOTO, RITSUSHI KATOU

#### Saitama Medical University International Medical Center, Saitama, Japan

**Introduction:** Left atrial appendage (LAA) has been regarded as an important source of cardiac thrombus formation and appears important in the contribution to thromboembolism in patients with atrial fibrillation (AF). The evaluation of LAA size and morphology type is important to select the optimal LAA closure device for patients with AF. However, the LAA characteristics of Asian patients remain uninvestigated. The aim of this study was to investigate the morphology and size of LAA in Asian patients.We used cardiac multidetector computed tomography (MDCT) performed before catheter ablation to evaluate the size and morphology type of LAA in Japanese patients. And then, our aim is to evaluate to contribute to select the optimal LAA closure device.

**Methods:** We retrospectively analyzed the cardiac multidetector computed tomography (MDCT) which was performed before the catheter ablation for AF from August 26, 2022, to November 30, 2023. We analyzed the LAA morphology types and measured the LAA ostial dimension and depth based on the measurement algorithm of the WATCHMAN system. The LAA was classified as chicken wing, swan, cauliflower, or windsock, based on predefined morphology classification criteria.

**Results:** A total of 221 patients (mean age 67.4±10.5 years, 21.7% female) were included in this analysis (205 patients with AF and 16 patients without known AF). Windsock was the most prevalent LAA morphology (90 patients, 40.7%) in the overall study population, followed by cauliflower (57 patients, 25.8 %), chickenwing (45 patients, 20.4%), and swan LAA morphology (28 patients 12.7%). Thrombi were observed in 8 out of 9 patients (89%) with chickenwing type, indicating a higher tendency for thrombus risk compared to other morphologies. The maximum diameter of the orifice was 20.0 ±5.0mm and the depth was 26.2 ±5.9mm. There were 20 patients (9.8%) where the WATCHMAN FLEX was deemed unsuitable due to a maximum diameter exceeding 31.5mm.

**Conclusions:** The chickenwing type LAA morphology was relatively common in Japanese patients and 20% of them had thrombosis. In approximately 10% of cases, the WATCHMAN FLEX was unsuitable.

## FEASIBILITY OF THE RHYTHMIA™ MAPPING SYSTEM IN PEDIATRIC PATIENTS UNDER 20 KG

### **KOTA NAGAOKA** ^1^, TAISUKE NABESHIMA^1^, WATARU SASAKI^2^, HITOSHI MORI^2^, NAOKATA SUMITOMO^1^

#### ^1^Department of Pediatric Cardiology, Saitama Medical University International Medical Center, Saitama, Japan,^2^Department of Cardiology, Saitama Medical University International Medical Center, Saitama, Japan

**Introduction:** The Rhythmia™ mapping system enables rapid automatic acquisition of high‐resolution electroanatomic and activation mapping with minimal manual annotation. We previously reported the utility and accuracy of this system in pediatric patients whose body weight was > 20 kg. There have been no reports about the usefulness of this system in children whose body weight was less than 20 kg.

**Methods:** We retrospectively analyzed the data of catheter ablation using the Rhythmia™ system and Orion™ catheter in patients younger than 20 years old between February 2017 and May 2022. The patients were divided into two groups, a body weight over 20 kg group (> 20 kgG) and under 20 kg group (≤ 20kgG). The mapping time, number of mapped beats, and number of mapping electrodes were compared for the left and right atria. The number of manual re‐annotation points and incidence of complications were also compared.

**Results:** This study included 146 consecutive patients (>20 kgG; range 20.1‐96.0 kg, n=132; ≤ 20 kgG; range 15.7‐19.3 kg, n=14). There were no significant differences in the mapping time, accepted beats, and electrograms (RA, > 20kgG [n=104] vs. ≤ 20 kgG [n=11]; mapping time, 14.1 minutes [interquartile range (IQR) 10.2‐18.4] vs. 20.1 minutes [IQR 9.4‐25.3], p=0.30; accepted beats, 749 beats [IQR 573‐1098] vs. 953 beats [IQR 654‐1079], p=0.87; points of electrograms, 6928 points [IQR 5132‐10927] vs. 6650 points [IQR 4474‐9527], p=0.45) (LA, > 20 kgG [n=62] vs. ≤ 20kgG [n=7]; mapping time, 11.5 minutes [IQR 8.1‐18.3] vs. 14.5 minutes [IQR 8.5‐15.4], p=0.61; accepted beats, 619 beats [IQR 354‐1007] vs. 512 beats [IQR 316‐810], p=0.55; points of electrograms, 6809 points [IQR 4025‐10108] vs. 4338 points [IQR 3369‐8693], p=0.38). There were no significant differences in the number of manual re‐annotation points. There were no cardiac tamponades or embolic stroke events.

**Conclusions:** Our study concluded that the Rhythmia™ system could be safely used for mapping even in pediatric patients whose body weight was more than 15 kg. There was no need for an additional manual re‐annotation even in small children.

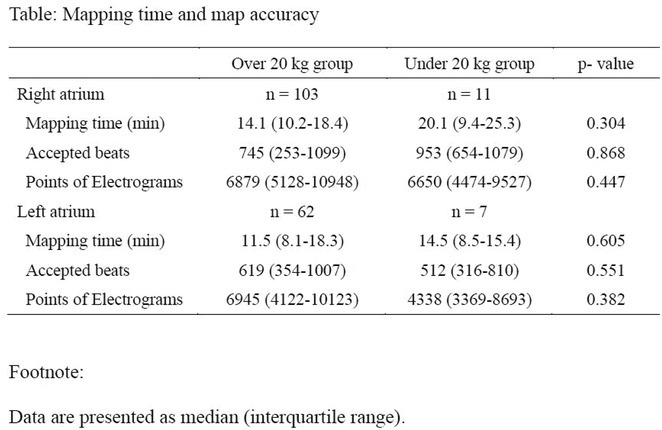

## THE EFFECTIVENESS OF CARDIOVASCULAR IMPLANTABLE ELECTRONIC DEVICES LEAD EXTRACTION FOR NON INFECTIOUS PATIENTS

### **MICHIO NAGASHIMA**, REI KUJI, KOMEI ONUKI, TOMONORI KATSUKI, HIROYUKI KONO, KENICHI HIROSHIMA, KENJI ANDO

#### Kokura Memorial Hospital, Kitakyushu, Japan

**Introduction:** Although the indication for transvenous lead extraction (TLE) to clear a cardiac device‐related infection is relatively uncontroversial, the decision‐making process regarding lead extraction for noninfectious indications is frequently less straightforward. Not only are there no randomized data to guide treatment, but it is unclear in many cases whether the risk of extraction would outweigh the benefit of having the leads removed. Authors provide the retrospective analysis of the effectiveness and safety of TLE for non‐infectious lead.

**Methods:** Between 2009 September and 2023 August, we performed TLE in 656 patients (pts). Out of these, 152 (23.2%) pts had been performed TLE because of non ‐ infectious indications. The clinical characteristics including indication and complication were summarized.

**Results:** There were 152 patients (Male 112pts, Mean Age 60.1 &plusmn;16.2 years) in non‐infectious group. Most common indication for lead extraction was lead failure in 119pts (78.2%). All patients underwent successful transvenous removal of endocardial leads. Two of 152 patients (1.3%) had evidence of major complication in non‐infectious patient (1hemothorax, who recovered by emergent surgery and one cardiac tamponade with drainage). No significant difference of major complication was found in the non ‐ infectious lead group compared to infectious lead group (1.3vs.1.6%, P=0.65).

**Conclusions:** In non‐infectious group, percutaneous transvenous lead extraction could be performed safely. In assessing an individual's indication for transvenous lead extraction, the risks of extraction must be compared with the risk of lead abandonment.

## ELECTROCARDIOGRAPHIC CHARACTERISTICS AND ABLATION OUTCOMES ASSOCIATED WITH VENTRICULAR ARRHYTHMIAS ORIGINATING FROM THE PARA‐HISIAN REGION

### **ANUGRAH NAIR**, JENISH SHROFF, LUKAH TUAN, LORI BELL, ADRIANA TOKICH, RAJEEV PATHAK

#### Canberra Heart Rhythm, Canberra, Australia

**Introduction: Objective:** we investigated the electrocardiographic and electrophysiological characteristics of ventricular arrhythmias (VA) originating from the para‐hisian (PH) area.

**Methods: Methods:** 26 out of 182 patients with VAs between 2018‐2024 were studied. Electroanatomic mapping was used to define the PH region. PH‐VAs were compared to RVOT VAs. Features, differentiating left/right and supra/infrahisian PH‐VAs were identified.

**Results: Results:** Out of the 182 patients, 26 patients (14.28%) had PH VA, 13 were mapped to right PH site, 13 were mapped to have left PH site. 11 patients had a suprahisian and 15 patients had Infrahisian origin. The common features were a Left bundle morphology (rS, QS complex) in lead V1 (80.76%), mean QRS duration of 134.08±19.6ms, Inferior lead discordance (69.23%), R lead II>III (84.6%) and Lead III more negative than lead II (96.15%). 5 patients with myocardial scar had QRS >150ms. When compared to VAs originating from the RVOT, PH VAs had narrower QRS (134 ±19.6 ms vs 169 ± 24 ms, p<0.05), early transition, greater R lead II/III, and greater R/S V1 (p<0.05). Left PH VAs had greater Lead III, aVL, V1 R/S ratio (p<0.05). Right‐sided PH VAs had a greater Lead I R/S ratio, greater R lead II, and greater S V1 (p<0.05). The ratio of R wave in aVL/aVR of the PVC > sinus beat was present in the majority of right PH VAs (84.6%), irrespective of the supra and infrahisian location (p<0.05). However, this feature was absent in all left suprahisian VAs and present in only 57.14% of left infrahisian VAs. Post ablation mean improvement of LVEF was 30.41± 9.32% (p<0.05) and the mean reduction of VA burden was 82.13 ±15.2% (p<0.05). 2 patients required a redo procedure and none of the patients developed AV block.

**Conclusions: Conclusion:** Para‐Hisian VAs have distinctive electrocardiographic features. These VAs can be successfully eliminated without disturbance of the atrioventricular conduction.

## EARLY IMPLANT AND CLINICAL EXPERIENCE OF A NEW INSERTABLE CARDIAC MONITOR

### **DEVI NAIR** ^1^, DALE YOO^2^, ULRIKA BIRGERSDOTTER‐GREEN^3^, HARISH MANYAM^4^, BABAK YASMEH^5^, RAKESH GOPINATHANNAIR^6^, FUJIAN QU^7^, NIMA BADIE^7^, KYUNGMOO RYU^7^, DHANUNJAYA LAKKIREDDY^6^

#### ^1^St. Bernard's Medical Center, Jonesboro, AR,^2^Heart Rhythm Specialists, Dallas, TX,^3^UC San Diego Health, San Diego, CA,^4^Erlanger Health System, Chattanooga, TN,^5^Aurora Medical Center, Oshkosh, WI,^6^Kansas City Heart Rhythm Institute, Overland Park, KS,^7^Abbott, Sylmar, CA

**Introduction:** Insertable cardiac monitors (ICMs) have been increasingly used to diagnose and monitor a variety of heart conditions and guide clinical management in patients with known atrial fibrillation (AF). A new ICM system with 3‐ and 6‐year longevity was recently introduced with a set of new features, including body posture evaluation, 3D activity trends (for total activity, elevated heart rate with/without activity), and daily premature ventricular contraction (PVC) burden trends. The aim of this analysis was to assess implant indications, R‐wave sensing amplitude, and utilization of new device features in Assert‐IQ™ ICM models.

**Methods:** Randomly selected Assert‐IQ™ ICM devices (n=600; 300 each for the 3‐ and 6‐yr model) with remote transmissions sent to the Merlin™ Patient Care Network were included in the analysis. Reason for monitoring, R‐wave sensing amplitude, and status of monitoring features for each device were extracted. Device triggered atrial fibrillation (AF), bradycardia, pause, and tachycardia episodes were manually adjudicated.

**Results:** The distribution of implant indications in this randomly selected patient cohort is shown in the figure. The 6‐yr model was implanted twice as likely in patients indicated for AF management, post‐AF ablation, and ventricular tachycardia; whereas the 3‐yr model was implanted 1.6 times more likely in patients for syncope and palpitations. Patients implanted with the 3‐yr model were older than those with the 6‐yr model (67.2 ± 14.9 yrs vs. 64.5 ± 14.8 yrs, p<0.05). R‐wave sensing amplitudes at implant were similar in 3‐ and 6‐yr devices (0.5 ± 0.2 mV vs. 0.5 ± 0.2 mV). The new body posture, 3D activity trend, and PVC burden trend features were activated in 54%, 72%, and 74% of patients, respectively. By 3 months of monitoring, AF, bradycardia, pause, and tachycardia were diagnosed in 12%, 10%, 8%, and 22% of patients, respectively.

**Conclusions:** Adequate sensing amplitudes were confirmed by the new ICM system in both device models. The 6‐yr model was implanted in more patients needing long‐term monitoring and disease management beyond 3 yrs. New features were activated in more than half of the patients.

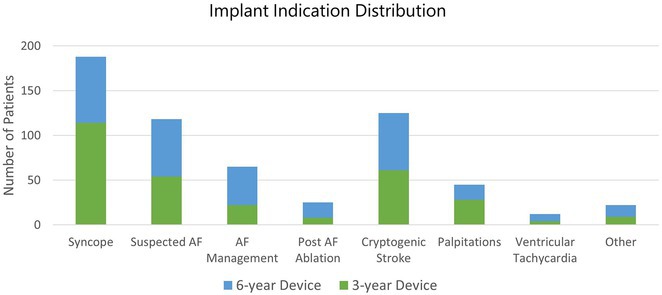

## COMPARISON OF LEFT ATRIAL POSTERIOR WALL ISOLATION AREAS BETWEEN CRYOBALLOON SYSTEMS

### **TOMOFUMI NAKAMURA**, YUTO TESHIMA, KOYO SATO

#### Nagoya Heart Center, Nagoya, Japan

**Introduction:** Cryoballoon‐based pulmonary vein isolation is a fundamental strategy for treating atrial fibrillation. Two types of cryoballoon systems are available, and with POLARx, it's possible to adjust the size. In PVI, the left atrial antrum is also targeted. This study aimed to investigate whether there are differences in the ablation area of the left atrial antrum depending on the type of balloon used.

**Methods:** We evaluated 139 patients (69.3 ± 12.4 years, 82 male) who underwent their first‐time cryoballoon ablation for paroxysmal or persistent AF. Following electrical isolation of the pulmonary veins, mapping of the LAPW was performed under constant pacing from the distal coronary sinus or higher right atrial wall to measure the extent of isolation. The isolation area was defined as the area <0.5mV. The LA posterior wall (LAPW) was defined as the area bounded by the roof line, bottom line, and the pulmonary vein orifices. The percentage of isolation area relative to the entire LAPW was defined as %IA. Patients were divided into three groups by used devices: Arctic Front Advance Pro(AFA) group, POLARx group, and POLARxFIT group.

**Results:** An Arctic Front Advance (AFA) was used in 71 patients, POLARx in 37 patients, and POLARx Fit in 31 patients. PVI was successfully achieved solely through cryoablation in 99.3% of the cases (5.0 ± 1.4 cryo‐application in AFA, 5.4 ± 1.6 POLARx, and 5.1 ± 1.2 in POLARx Fit). The average %IA was 54.3±11.3% in the AFA group, 60.2±10.5% in the POLARx group, and 62.9±11.0% in the POLARxFIT group. The POLARxFIT group demonstrated a significantly wider isolation area (p<0.0001). During the observation period after a 3‐month blanking period, recurrent cases were observed in 12 out of 100 patients during an average follow‐up period of 247 days. However, no significant difference in recurrence rates was observed between the groups (p=0.279).

**Conclusions:** The isolated area extending to the LAPW was the largest in the POLARxFIT group. Since the LAPW can be an arrhythmogenic source, this difference can associate with ablation outcomes.

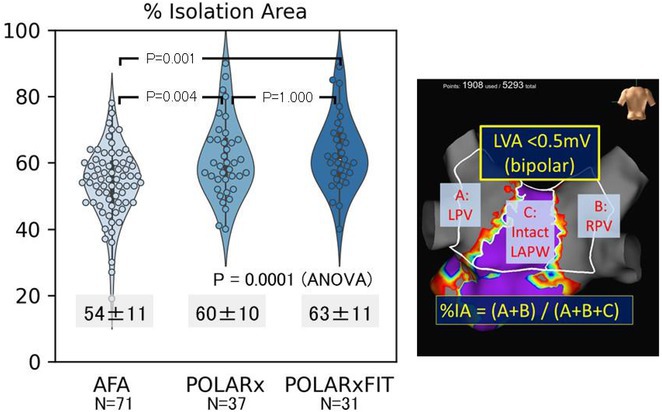

## EFFICACY AND SAFETY OF SIZE‐ADJUSTABLE CRYOBALLOON COMPARED TO CONVENTIONAL CRYOBALLOON FOR PAROXYSMAL ATRIAL FIBRILLATION

### **KENTARO NAKATA**, YUKIHIRO INAMURA, SHIN MEGURO, TOSHIKI MICHISHITA, YUHEI ISONAGA, SHINICHI TACHIBANA, HIROAKI OHYA, TAKAMITSU TAKAGI, AKIRA SATO, OSAMU INABA, YUTAKA MATSUMURA

#### Japanese Red Cross Saitama Hospital, Saitama prefecture, Japan

**Introduction:** Pulmonary vein (PV) isolation with cryoballoon (CB) technology is a well‐established therapy for atrial fibrillation (AF) patients. Most recently, a size‐adjustable CB (POLARx FIT) that enables delivery as a standard 28mm or expanded 31mm size was introduced. The efficacy and safety of size‐adjustable CB remains unclear.

**Methods:** Seventy paroxysmal atrial fibrillation patients who underwent initial cryoballoon ablation (CBA) were included. We divided these patients into the following two groups, who underwent CBA with POLARx FIT (FIT group; n=40), and 28mm conventional CB (28mm group; n=30). In FIT group, first freezing was performed with 31mm CB in each PVs. If occlusion or isolation was not possible, a 28mm CB was used. We compared ablation outcomes and complication rate between these groups.

**Results:** Of the 160 PVs targeted in the FIT group, 149 (93.1%) PVs were successfully isolated with 31mm cryoballoon, and all other PVs were isolated with 28mm CB. Total procedure time, number of CBA applications per patients, total freezing time, time to isolation of each PVs, and the number of patients whose esophageal temperature reached 15°C, were not significantly different between FIT and 28mm groups. The ratio who required touch‐up radiofrequency ablation of PVs were significantly less in FIT group (FIT=0.0%, 28mm=13.3%, p=0.03). Isolated area of left atrium after CBA was greater in FIT group (FIT=22.6±5.3 cm2, 28‐mm=17.3±2.8 cm2, p<0.001). The incidence of transient phrenic nerve palsy (PNP) was less in FIT group (FIT=0%, 28‐mm=16.7%, p=0.006), and the other major complication was not significantly different between the two groups. After a mean follow‐up of 294±48 days, the Kaplan‐Meier estimated freedom from atrial tachyarrhythmia recurrence without any antiarrhythmic drugs after single procedure was 92.5% in FIT group, and 93.3% in 28‐mm group (p=0.33).

**Conclusions:** The size‐adjustable CB was associated with less frequent touch‐up radiofrequency ablation of PVs, greater isolated area of left atrium and less frequent PNP compared to conventional CB.

## A CASE OF SUCCESSFUL CRYOABLATION OF PARA‐HISIAN ACCESSORY PATHWAY RECURRENCE AFTER PREVIOUS RADIOFREQUENCY ABLATION

### **JUNE NAMGUNG**, JAE‐JIN KWAK

#### Inje University Ilsan Paik Hospital, Goyang‐si, Korea, Republic of

**Introduction:** Radiofrequency catheter ablation of supraventricular tachycardia ablation with earliest activation site close to His‐bundle is a challenge due to the risk of complete AV block by its proximity to His‐Purkinje system. An alternative to minimize this risk is cryoablation of accessory pathway.

**Methods:** N/A

**Results:** We report a case of 18‐year‐old male with a history of WPW syndrome who had undergone radiofrequency catheter ablation of orthodromic atrioventricular reentrant tachycardia (AVRT), documenting a right anteroseptal accessory pathway near the His bundle. A 12‐lead electrocardiogram performed a few days later showed delta waves, and the patient complained of recurrent palpitations. The decision was made to proceed with cryoablation for the accessory pathway.

Cryoablation was performed in a position slightly anterior to the His bundle, which successfully resolved the accessory pathway conduction. There was no recurrence of accessory pathway conduction on follow‐up ECG one‐month post‐cryoablation.

**Conclusions:** Cryoablation is an option for the ablation of accessory pathways that are close to the normal conduction pathways.

## SUCCESSFUL CATHETER ABLATION OF A PARA‐HISIAN ACCESSORY PATHWAY FROM THE AORTIC CUSP

### **JUNE NAMGUNG**, JAE‐JIN KWAK

#### Inje University Ilsan Paik Hospital, Goyang‐si, Korea, Republic of

**Introduction:** The para‐Hisian accessory pathway is occasionally encountered in WPW syndrome. However, radiofrequency catheter ablation can be challenging because of the risk of complete atrioventricular block.

**Methods:** N/A

**Results:** We describe a case of 19‐year‐old male with recurrent palpitation with a Wolff‐Parkinson‐White electrocardiographic pattern and highly possible para‐Hisian accessory pathway localization. The electrophysiological study revealed that the accessory pathway was a right anteroseptal accessory pathway, located very close to the His bundle. This proximity posed a high risk of complete atrioventricular block with conventional ablation approaches. Complete elimination of an accessory pathway conduction was achieved with a radiofrequency energy application from the from the aortic cusp.

**Conclusions:** The para‐Hisian accessory pathway has a high risk of complete atrioventricular block during catheter ablation. However, ablation from the aortic cusp can reduce this risk and may be considered as an alternative approach.

## COMPARISON OF CLINICAL OUTCOMES AFTER ATRIAL FIBRILLATION ABLATION USING INTELLANAV STABLEPOINT AND OTHER TYPES OF CATHETERS

### **MASATAKA NARITA**, H. MORI, DAISUKE KAWANO, NAOMICHI TANAKA, TSUKASA NAGANUMA, WATARU SASAKI, KAZUHISA MATSUMOTO, YOSHIFUMI IKEDA, RITSUSHI KATO

#### Saitama Medical University International Medical Center, Hidaka, Japan

**Introduction:** Some studies reported that ablation catheters with the Rhythmia system may lose more energy into the bloodstream because of the longer tip length (4.0 mm) of the StablePoint used in the Rhythmia system. It was not clear what the results were in similar radiofrequency ablation settings with the Rhythmia system and the 3.5mm tip of catheters. This study aimed to examine the clinical outcome of ablation with the StablePoint versus other ablation catheters.

**Methods:** This study was a single‐center, retrospective analysis. 337 patients who underwent radiofrequency catheter ablation for atrial fibrillation between February 1, 2021, and March 31, 2022, have been included. Ablation catheters with a 4.0mm tip, such as the StablePoint, were divided into group 1 (98 patients), while catheters with a 3.5mm tip, such as the ThermoCool, the SmartTouchTM and the Tacticath SE, were divided into group 2 (239 patients). Group 1 was compared to group 2, and their clinical outcomes were evaluated. Catheter ablation was set at 35‐40 W for about 20‐30 seconds with pulmonary vein isolation and I performed tags analysis.

**Results:** There was no significant difference in atrial fibrillation recurrence after catheter ablation (14.3% vs. 15.9%, p=0.868). However, the 4mm tip catheter showed a significantly lower success rate than the 3.5mm tip catheter for the first pass (66.3% vs. 85.8%, p=0.0001). A propensity score match was performed with similar results (66.3% vs 84.9%, p=0.007). We excluded gaps due to carina in PVI, extracted unsuccess tags, and compared them to success tags. The LI drop was significantly larger when the tag was analyzed in group 1, and the success tags were compared to the unsuccess tags (success tag; 24 ohms [17‐32] vs. unsuccess tag; 21 ohms [14‐29], p=0.0175).

**Conclusions:** In practice, there was no difference in recurrent atrial fibrillation between the 4mm tip catheter and the 3.5mm tip catheter, but the 4mm tip catheter had a lower success rate in the first pass. When performing catheter ablation using a StablePoint catheter, it seemed important to check LI drop to ensure the first pass.

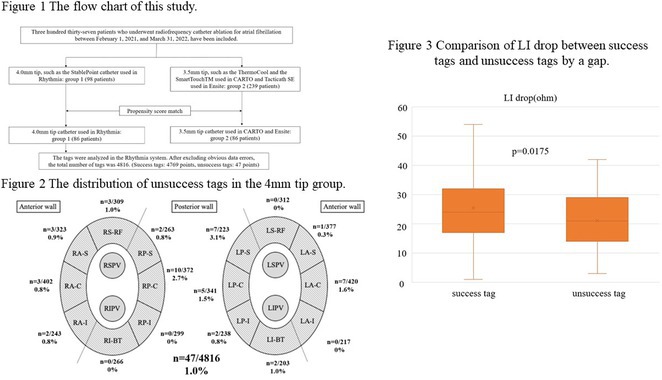

## TECHNIQUES OF PACING AND ABLATION WITH MICROELECTRODES FOR ISOLATING PULMONARY VEINS VIA LOCAL IMPEDANCE‐GUIDED CATHETERIZATION

### **MASATAKA NARITA** ^1^, HITOSHI MORI^1^, HIDEHIRA FUKAYA^2^, KAZUHISA MATSUMOTO^1^, TSUKASA NAGANUMA^1^, WATARU SASAKI^1^, NAOMICHI TANAKA^1^, DAISUKE KAWANO^1^, YOSHIFUMI IKEDA^1^, RITSUSHI KATO^1^

#### ^1^saitama Medical University International Medical Center, Hidaka, Japan,^2^kitasato University Hospital, Sagamihara, Japan

**Introduction:** The IntellaNav MiFi OI catheter (MiFi) is equipped with a sensor for local impedance (LI) monitoring and three mini‐electrodes. These mini electrodes, separate from the ablation electrodes, enable ablation while conducting local pacing, indicating its potential for signaling local electrical conduction interruption. In this study, we investigated the target LI values for a successful pulmonary vein isolation (PVI) under the pacing and ablation technique using the MiFi catheter.

**Methods:** A cohort of twenty‐seven patients was subjected to PVI employing the MiFi catheter, where pacing was conducted through its mini electrodes. Effective RF applications were defined as the loss of pacing capture, and an additional 10 seconds of ablation after capture loss was set as the endpoint for each ablation site. The local impedance (LI) changes, generator impedance (GI) changes, and the time to capture loss were evaluated.

**Results:** First‐pass isolations were obtained in 15 patients (57.7 %) for right PVs and 22 (84.6 %) for left PVs. Both the LI and GI decreased over the course of ablation, with the LI showing a greater rate of change than the GI. At Gap sites, the impedance decrease was smaller than at Non‐gap sites (Non‐gap sites vs. Gap sites; LI drop, 23.2 [±10.3] vs. 15.6 [±7.7] ohms, p<0.0001; GI drop, 4.8 [±4.1] vs. 2.7 [3.9] ohms, p=0.0026; % LI drop, ‐19.3 [±7.4] vs. ‐13.1 [±6.1] %, p<0.0001; % GI drop, ‐5.1 [±4.2] vs. ‐2.9 [±4.2] %, p=0.0020), suggesting that changes in impedance could be useful for predicting gaps. The LI was more effective than GI in predicting gaps. The cutoff values for predicting no gaps were identified as 15.0 ohms for the LI drop and ‐13.74% for the %LI drop, respectively.

**Conclusions:** The MiFi catheter's pacing and ablation approaches have shown efficacy in PVI. LI exhibited more significant variations than GI and proved valuable in gap prediction. Conduction gaps could be anticipated with LI drop cutoffs set at 15.0 ohms and ‐13.74% for the %LI drop.

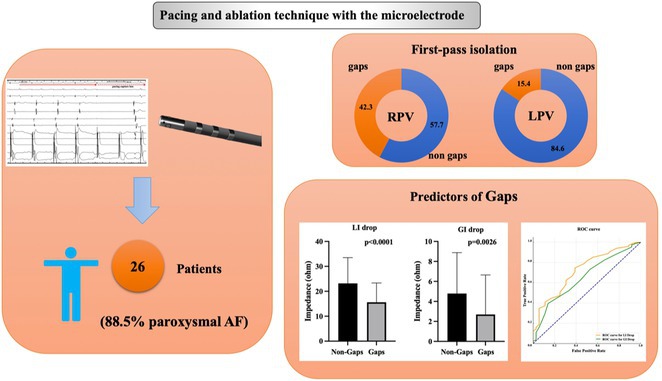

## DIVERSITY IN ATRIAL FIBRILLATION MANAGEMENT ACROSS MEDICAL SPECIALTIES : A SURVEY‐BASED INSIGHTS FROM A TERTIARY CARE HOSPITAL IN PAKISTAN

### **AIYSHA NASIR**, UMAIR JAVED, ARMUGHAN TAUHEED FAROOQI, FARYAL SUBHANI, YAWER SAEED

#### Aga Khan University Hospital, Karachi, Pakistan

**Introduction:** Atrial Fibrillation(AF) represents the most common arrhythmia diagnosed in contemporary clinical practice. There is limited knowledge about epidemiological datasets concerning AF, from the South‐Asian population. This study analyzes the trends associated with the diagnosis and treatment of AF by multiple specialists across a tertiary care institution in Karachi, Pakistan.

**Methods:** In this quantitative, observational cross‐sectional research, an online survey was circulated via email with all faculty members/consultants and residents in the Department of Medicine, and Allied at a tertiary care hospital, Karachi, Pakistan, in March 2021.

**Results:** In this survey of 104 participants, including 27 consultants (C) and 77 residents (R), most(85.6%) were aware of different atrial fibrillation (AF) types, with consultants slightly more informed(88.9%) than residents(84.4%). Diagnostic methods varied: residents favored 12‐lead ECG(49.4%), while consultants preferred it combined with a pulse check(63.0%). Treatment preferences also differed; residents favored rate control(38.9%), while consultants preferred rhythm control(65.2%) for new‐onset AF. Beta‐blockers were the preferred rate control agents(83.3% C, 76.3% R), and amiodarone was favored for rhythm control(92.0% C, 90.3% R). Risk score evaluations, particularly CHA2DS2VASC, were commonly used for anticoagulation initiation(85.2% C, 87.0% R), with rivaroxaban being the most frequently chosen agent(63.0% C, 55.3% R). Consultants were more aware of NOAC contraindications in specific AF cases(48.2%), and both groups generally agreed on the safety of triple therapy post‐PCI(55.6% C, 51.9% R), with most continuing it for three months.

**Conclusions:** Our study portrays that the management of patients with AF and its subtypes is variable, due to a lack of observational research and preferred reliance on clinical experience than ever‐changing updated guidelines. Optimizing management could lead to better outcomes and avoidance of unnecessary interventions.

## PREVALENCE OF ARRHYTHMIAS AND ELECTROCARDIOGRAPHIC ABNORMALITIES IN PAKISTAN: AN OUTPATIENT ECG‐BASED STUDY

### **AIYSHA NASIR**, ARMUGHAN TAUHEED FAROOQI, SAADIA SATTAR, YAWER SAEED

#### Aga Khan University Hospital, Karachi, Pakistan

**Introduction:** Little is known about the arrhythmia prevalence in Pakistan. This study is performed to assess the frequency of arrhythmias and ECG abnormalities.

**Methods:** All ECGs performed at a tertiary care hospital in 2022, were analyzed by a Cardiologist per Minnesota coding. A comparison was performed between men, women, age groups (&lt;18 years, 18‐50, 51‐65, &gt;65 years), and provinces of Pakistan [Sindh, Punjab, Baluchistan, Khyber‐Pakhtunkhwa (KPK) and Gilgit‐Baltistan (GB)].

**Results:** 8746 ECGs were analyzed, 56.86% were men. Common arrhythmia includes atrial fibrillation (AF) (1.2%), atrial flutter (AFL) (0.2%), sinus arrhythmia (0.8%), 2^nd^ degree/complete heart block(0.1%) and supraventricular tachycardia(SVT)(0.1%). Significant results with p values of less than 0.01 included: Women had more AF (1.6%). More than 65years had more AF (3.3%), AFL(0.6%), SVT(0.4%), sinus bradycardia(10%). Common ECG abnormalities noted were left anterior fascicular block (LAFB) (7%), poor‐R‐wave progression (5%), early repolarization pattern (4%), right bundle‐branch block(RBBB)(3%), pathological Q‐waves(3%), left ventricular hypertrophy(LVH) (2%) and 1^st^ degree AV block(1.3%). Significant results with p values of less than 0.01 included: Women has more poor R‐wave progression (6.4%) while men had more LAFB(8%), early repolarization pattern(5%), RBBB(4%), pathological Q waves(4%) and LVH(2%). More than 65 years had significantly more LAFB(13%), poor R‐wave progression(9%), pathological Q waves(6%), 1^st^ degree AV block(4%) and left bundle branch block(LBBB)(2%). Less than 18 years has higher prevalence of RAD(11%), RBBB(10%) and long QT(3.7%). Regional differences show that patients from KPK more AF (3.8%, p &lt;0.05) and LVH (7.7%, p&lt;0.01), whereas patients from GB has higher sinus bradycardia (28%, p&lt;0.01).

**Conclusions:** This study details important information about arrhythmia prevalence in a large sample of the South‐East‐Asian population. AF is prevalent in women, >65 years, and people from KPK. >65 years has more arrhythmias including AF, conduction system diseases (LAFB, LBBB) and pathological Q waves.

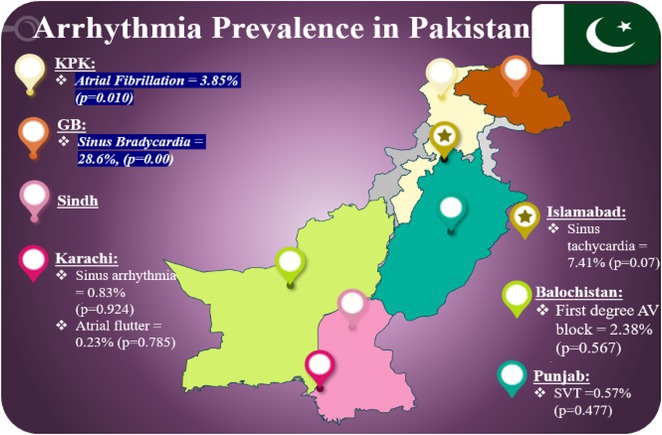

## HIS‐OPTIMIZED CARDIAC RESYNCHRONIZATION THERAPY COMBINED WITH ATRIOVENTRICULAR NODAL ABLATION IN A PATIENT WITH HEART FAILURE AND PERMANENT ATRIAL FIBRILLATION: A CASE REPORT

### **MUHAMMAD RAIHAN RAMADHAN NATADIKARTA**, GIKY KARWIKY, CHAERUL ACHMAD, HAWANI SASMAYA PRAMESWARI, MOHAMMAD IQBAL

#### Universitas Padjadjaran/Dr. Hasan Sadikin Hospital, Bandung, Indonesia

**Introduction:** Cardiac resynchronization therapy (CRT) is a treatment option for chronic heart failure (HF) patients who are accompanied by left bundle branch block (LBBB). The role of CRT is less well established in permanent atrial fibrillation (AF) patients with coexistent heart failure compared to sinus rhythm. Atrial fibrillation with an uncontrolled rapid ventricular response rate results in a necessity of atrioventricular nodal ablation (AVNA). His‐optimized cardiac resynchronization therapy (HOT‐CRT) was preferred due to narrower QRS duration and combination with AVNA may enhance the clinical outcomes in those specific patients. We are presenting a clinical case with preexisting HOT‐CRT who underwent AVNA with satisfactory results in follow‐up period.

**Methods:** N/A

**Results:** We report the case of 65‐year‐old man who has been experiencing heart failure for the past 3 years with frequent hospitalization due to decompensated heart failure. He also has permanent atrial fibrillation with bundle branch block. The left ventricular ejection fraction (LVEF) was 17% and the QRS complex duration was 180 ms, with a morphology indicating left bundle branch block (LBBB). The patient received HOT‐CRT in late 2022, but the symptoms were not significantly improved and the biventricular pacing percentage was only 64%. We successfully performed atrioventricular nodal ablation (AVNA) with left sided approach. We observed that 40 ms His to LV delay resulted highest stroke volume based on echocardiography and narrowest QRS complex duration. Consequently, the biventricular pacing percentage improved to 99%, QRS complex duration was significantly reduced to 80 ms, LVEF increased to 26%, and there was a significant improvement in clinical condition, assessed by the Minnesota Living with Heart Failure Questionnaire, during a 1‐month and 3‐month follow‐up period.

**Conclusions:** There is a notable improvement in the function of the left ventricle, symptoms, and quality of life in specific heart failure patients with persistent atrial fibrillation coexist with LBBB who had HOT‐CRT and AVNA.

## MULTIFACETED IMPACT OF THERAPEUTIC INTERVENTIONS ON ECTOPIC BURDEN AND LEFT VENTRICULAR EJECTION FRACTION IN A PATIENT WITH AORTIC STENOSIS UNDERGOING TAVI: A CASE REPORT

### **ANNA NATHER**, CHARIS COSTOPOULOS, SHARAD AGARWAL

#### Royal Papworth Hospital NHS Trust, Cambridge, United Kingdom

**Introduction:** In patients with aortic stenosis, reduced LVEF often presents a complex interplay of various factors, making it challenging to pinpoint the primary driver of compromised ejection fraction (EF). The intricate relationship between physiological and anatomical changes along with alterations in electrophysiological properties add to the complexity of understanding the causative mechanisms.

**Methods:** NA

**Results:** A 74‐year‐old male with symptoms of heart failure was diagnosed to have severe AS, RBBB, impaired LVEF and frequent PVC. His recovery involved a Transcatheter Aortic Valve Implantation (TAVI) procedure for AS, Cardiac Resynchronization Therapy (CRT) and ablation for premature ventricular contractions (PVC). After TAVI, the LV function improved to 40% and ectopic burden reduced to 5%. He had LBBB area pacemaker for tri‐fascicular block and syncope and the impaired LV function. His heart failure medications were optimised with beta blockers, Entresto, spironolactone and SGLT2 inhibitors. He had further recurrence of PVC and the CRT pacing was inadequate. The LV function again deteriorated to 35%. Ablation of the PVC, originating from LVOT, just below the TAVI valve was successfully carried out, transseptally. The patient continued to improve, with a subsequent echocardiogram five months later revealing an improved LVEF of 45‐50%. A follow‐up 12 months later confirmed sustained improvement in ectopic burden, left ventricular ejection fraction and symptoms. (fig 1).

**Conclusions:** This case study highlights AS's physiological effects on LV function, conduction system, and ventricular arrhythmias. Although each intervention contributed to systolic function enhancement, substantial long‐term improvement occurred only after ectopic rhythm ablation, increasing EF from 35% to a sustained 50%. This provides insights into cellular remodelling's cumulative effect on cardiac muscle electrical activity and effective LV function disruption, possibly by ventricular ectopic rhythms, revealing a novel perspective on ectopic‐induced cardiomyopathy.

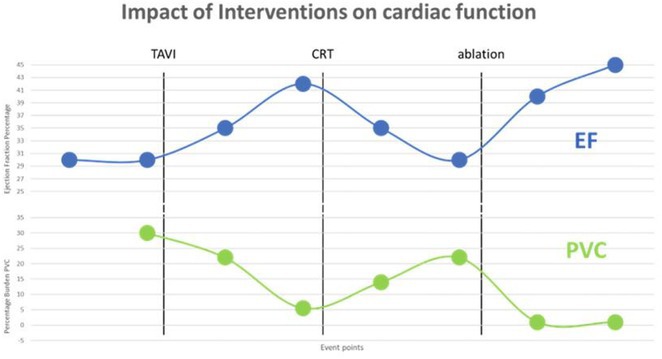

## FEASIBILITY OF REMOTE SOTALOL AND DOFETILIDE INITIATION IN PATIENTS WITH ATRIAL FIBRILLATION: A SYSTEMATIC REVIEW

### **RACHITA NAVARA** ^1^, MAITREYI BHARATH^2^, MARIA DOPIERALA^3^, GABRIEL KOO^4^, AZEEM RATHORE^5^, ADITI PALLOD^6^, TIARA PAUL^7^, BISHOW PAUDEL^8^, SHIYONA DE^2^, KUNJ PATEL^9^

#### ^1^University of California San Francisco, Manteca, CA,^2^Safebeat Rx, San Francisco, CA,^3^University Hospital, Poznan, Poland,^4^Plymouth Highschool, Canton, MI,^5^University of Florida, Gainesville, FL,^6^Pennsylvania State University, State Colelge, PA,^7^Sidney Kimmel College at Thomas Jefferson University, Philadelphia, PA,^8^University of Alabama at Birmingham, Birmingham, AL,^9^St. Mary's Hospital, San Francisco, CA

**Introduction:** Atrial fibrillation (AF) is the most common arrhythmia worldwide. Some antiarrhythmic drugs (AADs), such as sotalol and dofetilide, are initiated in the hospital setting to monitor for proarrhythmic effects. Hospitalization poses challenges to both patients and physicians, making remote AAD initiation an attractive alternative. Growing developments in cardiac monitoring promote safe AAD initiation in remote settings.

**Methods:** We comprehensively searched PubMed and Google Scholar databases to identify studies on outpatient sotalol and dofetilide initiation in AF patients. Data regarding drug initiation success was also extracted.

**Results:** Five studies were identified (n = 204) that met our inclusion criteria. Sotalol was the loading drug in four studies (174 total patients) and dofetilide in one study (30 total patients). Among all patients across the studies, the most popular cardiac monitors were implantable loop recorders (n = 87, 42%) and continuous loop event recorders (n = 57, 28%). Regarding monitoring frequency, 87 individuals (43%) underwent continuous monitoring for up to 8 hours, while 117 individuals (57%) were monitored intermittently, with a scheduled ECG transmission in the second hour following a dose. Successful initiation of AAD, defined as the absence of adverse events leading to AAD discontinuation or failure to convert to or maintain sinus rhythm, was achieved in 78% of patients (n = 159). Additionally, no ventricular arrhythmias were observed.

**Conclusions:** Our findings indicate that outpatient initiation of AADs is feasible with the utilization of commercially available monitoring devices. The 78% success rate underscores the potential for outpatient AAD initiation to effectively and safely manage AF, thus minimizing the need for costly hospitalizations. Further prospective studies are needed to evaluate the use of cardiac devices in outpatient settings to optimize AF management strategies as a means to reduce overall healthcare costs.

## "UNMAPPABLE" WAS ACTUALLY THE CLUE FOR SUCCESSFUL SITE FOR PVC ABLATION

### **IGNATIUS YANSEN NG** ^1,2^, SIMON SALIM^1,3^, MUHAMMAD YAMIN^1,3^, DANIEL TANUBUDI^1^

#### ^1^MyCardia, Eka Hospital BSD, South Tangerang, Banten, Indonesia,^2^Tangerang District Hospital, Tangerang, Banten, Indonesia,^3^Cipto Mangunkusumo Hospital, Jakarta, Indonesia

**Introduction:** LVOT PVC usually need 3D mapping to help pinpoint the location. In specific instances, mechanically suppressing PVCs with a catheter can provide valuable information regarding the optimal location for ablation. (1)

**Objective:** To document a case of effective ablation of PVC in the GCV/AIV by utilizing clues obtained from mechanical suppression of PVC.

**Methods:** N/A

**Results:** A 57yo male patient with dizziness and presyncope symptoms was treated with radiofrequency ablation due to its resistance to verapamil. The electrocardiogram (ECG) revealed monomorphic premature ventricular contractions (PVCs) with an inferior axis and positive concordance in all precordial leads, exhibiting a break pattern specifically in leads V2‐3 (Figure 1A). The PVCs were initially spontaneous and frequent during treatment, but after a decapolar catheter was inserted into the coronary sinus, they ceased. The process was terminated due to the PVCs being deemed "unmappable." However, the PVC resurfaced immediately following the removal of the decapolar catheter, indicating mechanical inhibition by the cs catheter. A local activation time measurement of ‐30 ms was determined. (Figure 1B) As a precautionary measure, nearby structures were assessed, which only exhibit an earlier of 10 to 20 milliseconds. The ablation procedure was performed using a 25‐watt irrigating catheter in the coronary sinus distal (Figure 1C), with each effort lasting only 5 seconds due to a dramatic increase in impedance. The PVC were successfully eradicated on the first attempt, and subsequent ECG monitoring for 24 hours showed no occurrence of any PVC.

**Conclusions:** Physicians should be cognizant of changes in procedure stages, as mechanical suppression in our case led to successful ablation, even without 3D imaging. PVC can be repressed mechanically, indicating superficial origin, which was confirmed with short ablation time.

**Reference**: Jain R, Barmeda M, Jain R, Shirazi J, Miller JM. Mechanical suppression of premature ventricular complexes during catheter ablation procedures. Indian Pacing Electrophysiol J. 2021 Jan‐Feb; 21(1): 29‐35.

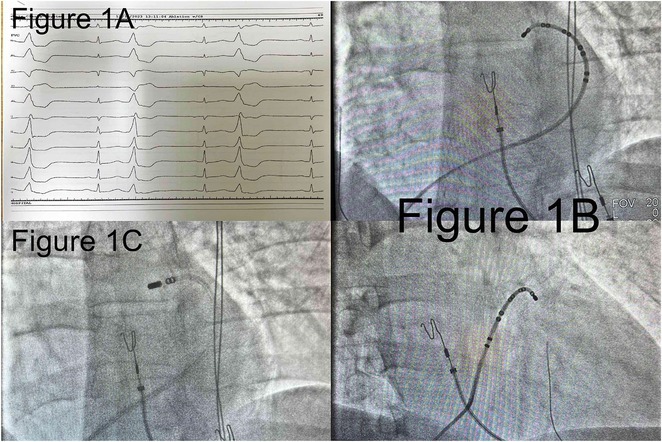

## EARLIER IS NOT ALWAYS BETTER : PARAHISIAN ACCESSORY PATHWAY ABLATED FROM AORTIC CUSP

### **IGNATIUS YANSEN NG** ^1,2^, SIMON SALIM^2,3^, MUHAMMAD YAMIN^2,3^, DANIEL TANUBUDI^2^

#### ^1^Tangerang District Hospital, Tangerang, Banten, Indonesia,^2^MyCardia, Eka Hospital BSD, South Tangerang, Banten, Indonesia,^3^Cipto Mangunkusumo Hospital, Jakarta, Indonesia

**Introduction:** Catheter ablation is a Class I indication therapy for symptomatic supraventricular tachycardia caused by atrioventricular re‐entry via an accessory pathway (AP). This method has a success rate of more than 95% and a low risk of complication. [1] However, the proximity of the Parahisian Acessory Pathway to the regular atrioventricular conduction system makes the procedure technically difficult and high risk for complication.

**Objective:** To report on a successful redo ablation of a parahisian AP ablated from the Non Coronary Aortic Cusp.

**Methods:** N/A

**Results:** A 40‐year‐old female with a history of unsuccessful parahisian AP ablation presented to our facility with highly recurrent SVT that was resistant to beta blockers. Her previous attempt was 9 years ago, and it was a lengthy procedure (more than 4 hours). We use Ensite precision in conjunction with an open window mapping technique to display the earliest V during A pacing and earliest A during A pacing. The breakthrough signal was shown in 3D with the proximity to His signal (Figure 1A). Antidromically, SVT was easily induced. Even though we performed ablation from both the atrial and ventricular sides, we failed to eliminate the pathway. We moved on to the non‐coronary cusp area, and the pathway was eliminated in 10 seconds utilizing an open irrigating catheter with a 25‐watt power supply. (Figure 1B).

**Conclusions:** Because it is further away from the AV node, the non‐coronary aortic cusp could be an alternative place for the parahisian accessory pathway ablation, especially after multiple attempts from the right sided area failed to provide any effect.

**Reference:** Chokr MO, De Moura LG, Aiello VD, Lopes HB, et al. Catheter ablation of the parahisian accessory pathways from the aortic cusps ‐ Experience of 20 cases ‐ Improving the mapping strategy for better results. Journal of Cardiovascular Electrophysiology, 10.1111/jce.14499, 31, 6, (1413‐1419), (2020).

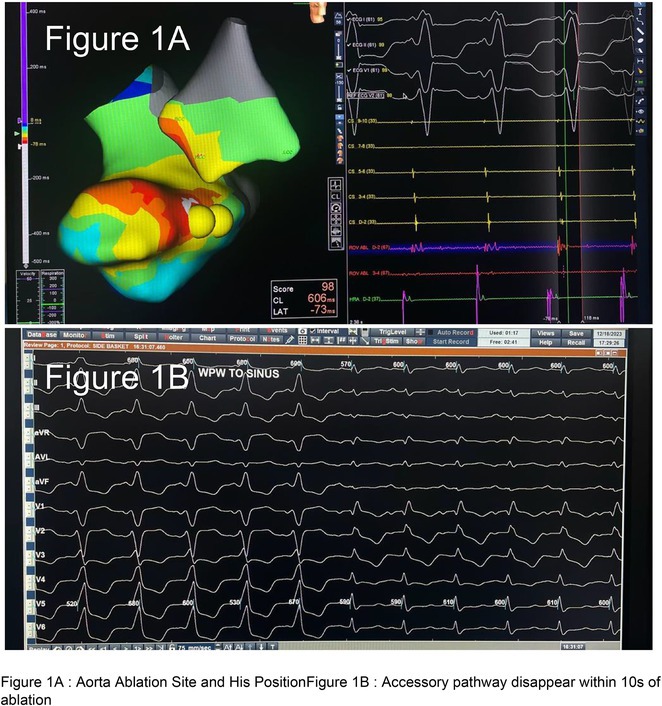

Figure 1A : Aorta Ablation Site and His PositionFigure 1B : Accessory pathway disappear within 10s of ablation

## TO OCCLUDE OR NOT TO OCCLUDE : HIGH‐RISK PFO IN ATRIAL FIBRILLATION

### **IGNATIUS YANSEN NG** ^1,2^, SIMON SALIM^1,3^, MUHAMMAD YAMIN^1,3^, DANIEL TANUBUDI^1^, GUIDDO ILYASA PURBA^1^

#### ^1^MyCardia, Eka Hospital BSD, South Tangerang, Banten, Indonesia,^2^Tangerang District Hospital, Tangerang, Banten, Indonesia,^3^Cipto Mangunkusumo Hospital, Jakarta, Indonesia

**Introduction:** One of the processes that typically lead to an ischemic stroke is embolism. Among the several causes of embolism are a patent foramen ovale (PFO) and atrial fibrilation (AF).This case study presents a 48‐year‐old man with a history of stroke, AF, a large chiari network, and a PFO.

**Methods:** N/A

**Results:** A 48‐year‐old man was referred to our facility with a history of ischemic stroke and type 2 diabetes. A symptomatic atrial fibrilation was discovered during the examination, and cryoablation with transesophageal echocardiography (TEE) was scheduled to mitigate the palpitations. TEE revealed a patent foramen ovale (PFO) and a large chiari network (Figure 1). These recent findings have revealed a significant risk factor for stroke. The Risk of Paradoxical Embolism (RoPE) Score and the CHA2DS2‐VASc Score were used as tools for assessing the risk for stroke, with a score of four and six, respectively. Due to the high CHA2DS2‐VASc Score, anticoagulation treatment was started. Following that, the cryoablation surgery was done, and the postoperative electrocardiogram (ECG) showed no reappearance of atrial fibrillation. After the ablation, two options are being contemplated. The use of long‐term novel oral anticoagulants (NOACs) or the adoption of PFO closure along with long‐term NOACs. There is currently no substantial evidence indicating that PFO closure offers any additional benefits on top of NOACs in individuals with AF.(1) In the absence of evidence, we decided to employ life‐long NOACs. If stroke recur or contraindications arise, the need for PFO closure should be reassessed.

**Conclusions:** Our case underscores the significance of comprehensive clinical evaluation and making decisions based on solid data. Given the CHA2DS2‐VASc Score of six, it is necessary to prioritize NOACs. In the absence of evidence, we postponed the PFO closure, and AF ablation was done for symptoms relieve. Reference:1. Kavinsky C.J., et al. SCAI Guidelines for the Management of Patent Foramen Ovale. *J. Soc. Cardiovasc. Angiogr. Interv*. 2022;1:100039. doi: 10.1016/j.jscai.2022.100039.

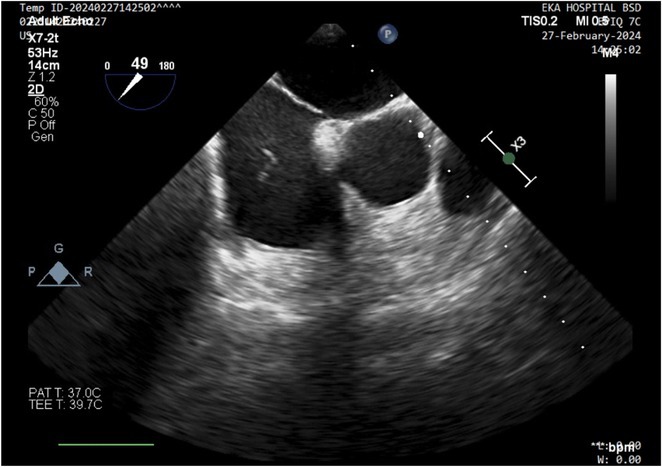

## ATRIAL FIBRILLATION SELF‐MANAGEMENT QUESTIONNAIRE (AFSM): DEVELOPMENT AND QUALITATIVE VALIDATION OF AN AF‐SPECIFIC PROM EVALUATING THE INDIVIDUAL’S ENGAGEMENT IN MANAGING THEIR AF

### **ANH HONG NGUYEN** ^1^, LYNETTE CUSACK^2^, PHILLIP TULLY^3^, EMILY BRINDAL^4^, RAKESH AGARWAL^1^, GAI MCMICHAEL^1^, MARK BOYD^1^, LYLE J. PALMER^5^, RAJIV MAHAJAN^1^

#### ^1^School of Medicine, University of Adelaide and Lyell McEwin Hospital, Adelaide, Australia,^2^Adelaide Nursing School, University of Adelaide and Lyell McEwin Hospital, Adelaide, Adelaide, Australia,^3^School of Psychology, University of New England, Armidale, Australia,^4^Public Health and Wellbeing, CSIRO, Adelaide, Australia,^5^School of Public Health, University of Adelaide, Adelaide, Australia

**Introduction:** The optimal management of Atrial Fibrillation (AF) requires management of stroke risk, heart rate and rhythm control, and management of risk factors. Many AF risk factors are lifestyle related and require patients to engage in health behaviour change. According to the COM‐B model, interventions involving health behaviour change require consideration of patient capabilities, opportunities, and motivational factors. Currently we lack an AF‐specific patient reported outcome measure (PROM) to measure patient engagement and readiness to manage their condition.

**Methods:** The development of this PROM followed COSMIN guidelines involving 1) a literature review; 2) item generation covering the COM‐B domains with input of an Electrophysiologist, two registered research nurses with expertise in PROM development and two health behavioural scientists; 3) Content validity was tested through experts in the management for AF [n=10] and consumers with lived experience for AF [n=8]; 4) Item refinement of the final PROM was informed by a qualitative analysis.

**Results:** Item generation identified 48 items. Feedback from the expert focus group reduced the instrument to 14 items, which were presented to the broader expert and consumer groups. An item was removed and three items were added for improved comprehensiveness. The final version of the AFSM consists of two sections and 17 items. Section A explores general management of AF including medication adherence, rate and rhythm control and having access to monitoring devices. Section B explores the ability to manage their AF risk factors.

**Conclusions:** The AFSM is a 16‐item PROM developed and qualitatively validated as a tool to measure a patient's readiness and engagement in their AF management. The AFSM could be a valuable tool in clinic and in research to measure the effectiveness of interventions for improving care of individuals with AF. Furthermore the AFSM may identify specific factors to improve an individual's ability to manage their AF in the long‐term. The AFSM is undergoing quantitative validation in 200 individuals.

## EFFICACY AND SAFETY OF THE FIRST STEP TO APPLY CRYOBALLOON ABLATION IN VIETNAM TO ISOLATE PULMONARY VEINS IN THE TREATMENT OF PAROXYSMAL ATRIAL FIBRILLATION

### **DUY LINH NGUYEN** ^1^, DINH PHONG PHAN^1^, VO KIEN LE^1^, TUAN VIET TRAN^1^, TUAN VIET NGUYEN^2^

#### ^1^Vietnam National Heart Institute, Ha Noi, Viet Nam,^2^Thanh Hoa Province General Hospital, Thanh Hoa, Viet Nam

**Introduction:** Treatment of atrial fibrillation (AF) may be medical or surgical, depending on patient characteristics, duration of disease, symptoms, and patient preference. The two most frequently used energy sources used for ablation are radio‐frequency (RF) and cryo‐energy; although other energy sources are being actively investigated for their efficacy and safety. Our research focuses on evaluating the safety and effectiveness of cryoablation in the initial treatment of paroxysmal atrial fibrillation (PAF) in Vietnam

**Methods:** 15 random PAF patients underwent cryo‐balloon ablation to isolate the pulmonary veins (PV) according to the following procedure: (1) Open blood vessel access; (2) Placement of the balloon inside the root of PVs. Isolation of the PVs using cryo balloon and testing to evaluate the degree of PVs isolation. The safety of the procedure was determined based on the occurrence of severe complications (including pericardial effusion; aortic perforation; embolism, atrial septal dissection, access complications, atrioventricular block, phrenic nerve injury and left atrio‐esophageal communication). Meanwhile, the effectiveness was evaluated based on successful PVs isolation

**Results:** The average duration of PAF was 10 ± 12 months with an average frequency of 6.1 ± 7.1 episodes per month. In terms of PVs anatomy, MSCT noted 1 case of variation with 3 right pulmonary veins. All patients had completely isolated pulmonary veins, with radiation time and total ablation time being 14 ± 8 minutes and 125 ± 32 minutes, respectively. No complications related to the procedure were recorded.

**Conclusions:** Our reseach results show the safety of cryoablation technique to isolate pulmonary veins in patients with paroxysmal atrial fibrillation, when no complications related to the procedure were recorded. Cryo ablation holds great promise as an effective method for treating PAF in addition to RF. It is necessary to continue to expand the scope of research with a larger sample size and longer follow‐up time, so that we can accurately evaluate the effectiveness of reducing atrial arrhythmia recurrence

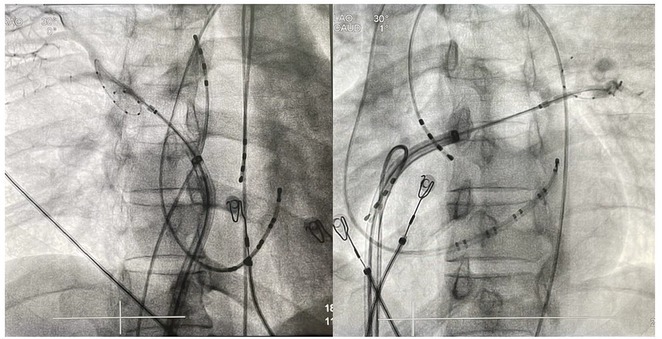

## SURFACE ECG ANALYSIS COMBINED WITH HD GRID CATHETER IN THE ABLATION OF COMPLEX VENTRICULAR ARRHYTHMIAS

### **DUY LINH NGUYEN** ^1^, DINH PHONG PHAN^1^, VO KIEN LE^1^, TUAN VIET TRAN^1^, TUAN VIET NGUYEN^2^

#### ^1^Vietnam National Heart Institute, Ha Noi, Viet Nam,^2^Thanh Hoa Province General Hospital, Thanh Hoa, Viet Nam

**Introduction:** We present a case to evaluate the usefulness of a combination surface ECG and the Advisor HD Grid Mapping Catheter in diagnosing and localizing complex arrhythmias that originate in the left ventricular

**Methods:** N/A

**Results:** A 67‐year‐old man with a history of hypertension but no structural or ischemic heart disease. The patient complained of anxiety and palpitations regularly. Further, the patient's surface ECG revealed double and triple PVCs, and the overall PVC burden in a 24‐hour Holter was 21%. Analyzing the morphology of PVC with a left posterior branch block in the surface ECG (right axis deviation, rS type in leads I and aVL) could help identify the location of arrhythmia initiation in the left ventricular and guide us to approach papillary muscle retrogradely through the descending aorta and the femoral artery. Combined with the Advisor HD Grid Mapping capability, which utilizes high density points in modeling, we try to find the potential electrograms of the left anterior bundle brach (P1 and P2). Based on the data presented above, we can precisely find that the origin of PVC was the left anterior bundle branch area. Finally, a successful PVC ablation was performed in the left ventricle at the left posterior bundle branch area with EAT ‐29 ms

**Conclusions:** The clinical case demonstrates the importance of carefully analyzing the surface ECG and using the Advisor HD Grid Mapping Catheter to locate the ventricular arrhythmia and orient the vascular access. In cases where ultrasound intra‐cardiac echo (ICE) is still not widely available, this combination increases the likelihood of successful ablation of dangerous arrhythmias originating in complex locations such as the left ventricle. In addition, this is the basis for using the HD grid catheter to treat complex arrhythmias from the left ventricle, increasing the likelihood of success and minimizing procedure time, as well as radiation exposure time when using the 3D mapping system.

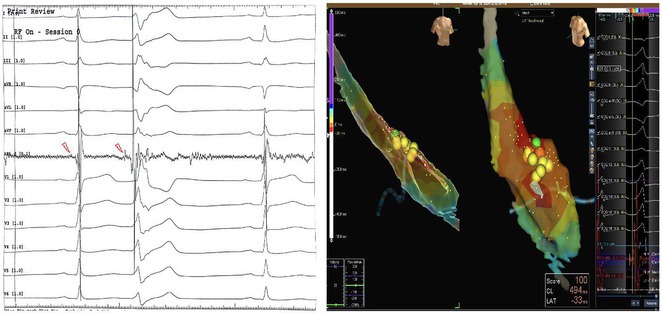

## COEXISTENT IDIOPATHIC LEFT VENTRICULAR TACHYCARDIA AND ATRIOVENTRICULAR NODAL REENTRANT TACHYCARDIA

### **NGOC NGUYEN DINH SON**, NHUT NGUYEN MINH, SON LUONG CAO

#### University Medical Center, HCM city, Ho Chi Minh city, Viet Nam

**Introduction:** We presented a 26‐year‐old male with double tachycardias, including Idiopathic Left Ventricular Tachycardia (ILVT) and Atrioventricular Nodal Reentrant Tachycardia (AVNRT).

**Methods:** N/A

**Results:** This patient had many episodes of palpitation and dizziness, and his ECG showed wide QRS tachycardia with a right bundle branch block and northwest axis QRS morphology at a heart rate of 153 bpm (Figure 1), hypotension was noted, and cardioversion was performed, which successfully reverted to sinus rhythm so idiopathic ventricular tachycardia was suspected. Despite being under oral verapamil (120 mg/day), symptomatic episodes occurred during follow‐up. The standard examinations showed no evidence of structural heart disease, myocarditis, long QT syndrome, or electrolyte imbalance. EPS was performed in November 2023. His ECG showed sinus rhythm without preexcitation. A typical slow‐fast AVNRT was induced by premature atrial complexes with a TCL of 380 ms and VA interval of 20 ms (Figures 2 and 3), then changed to the sustained wide QRS tachycardia with more V than A (Figures 4, 5, 6 and 7) and QRS morphology identical to the clinically documented tachycardia. His is recorded after QRS onset, and the second tachycardia characteristics, including TCL of 322 ms, QRS 146 ms, VA interval of 90ms and HV interval ‐ 44ms favoured VT (Figure 8). We used Navista to map the inferior‐basal septum of the left ventricle by retrograde transaortic approach. Purkinje potentials and diastolic potentials were observed at the mid portion of the left ventricular posterior septum (Figures 9, 10 and 11), and radiofrequency energy was applied, followed by tachycardia termination. After ablation of VT, we induced the slow‐fast form of AVNRT again (Figure 12). Three pulses of radiofrequency energy were successfully delivered to eliminate the slow pathway. After these ablations, neither VT nor AVNRT was induced. Anti‐arrhythmic drugs were then discontinued, and he did not have any symptoms.

**Conclusions:** The coexistence of supraventricular tachycardia and VT is a rare condition. Successful ablation of ILVT and the slow pathway resulted in the cure of double tachycardias.

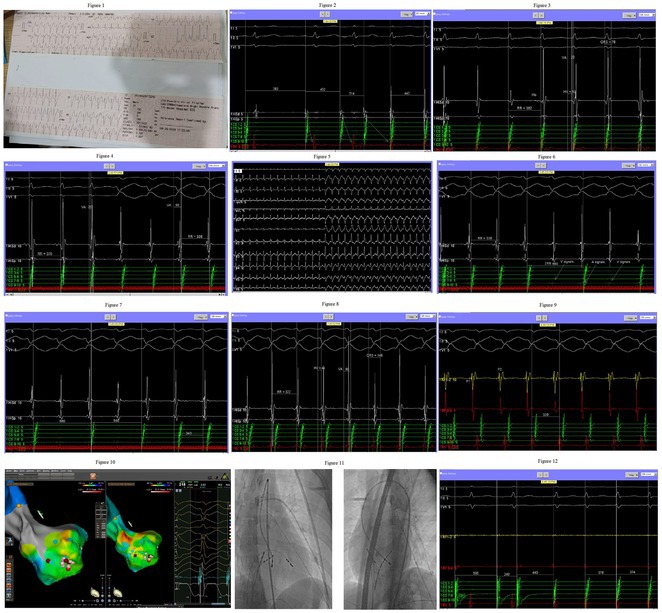

## RADIOFREQUENCY ABLATION OF ATRIAL TACHYCARDIA WITH 3D MAPPING SYSTEM: A CASE REPORT OF TACHYCARDIA‐INDUCED CARDIOMYOPATHY

### **TUAN NGUYEN DUY**, LINH PHAM TRAN, LONG VIEN HOANG, THUY NGUYEN THI LE, THANH VU HUY

#### Vietnam National Heart Institue, Bach Mai Hospital, Hanoi, Viet Nam

**Introduction:** Tachycardia‐induced cardiomyopathy, which is a rare cause of dilated cardiomyopathy, usually results from long‐standing supraventricular or ventricular tachycardias. Tachycardia‐induced cardiomyopathy can be reversible by appropriate management of tachycardias and heart failure.

**Methods:** N/A

**Results:** A 60‐year‐old man with a history of cigarette smoking and some periods of palpitations, presented with palpitations and progressive shortness of breath for the last 1 week and reported orthopnea at admission. A physical examination found a heart rate of approximately 230 beats/min, bilateral lung crackles, no hepatomegaly and pitting leg edema. A full cardiac workup was performed with electrocardiography showed atrial tachycardia, transthoracic echocardiography indicated left ventricular ejection fraction of 22%, and laboratory test includes high‐sensitive troponin T level of 136 ng/L and NT‐proBNP of 4724 pg/mL. The patient was unsuccessfully cardioversion with intravenous amiodarone and subsequently cardioverted to sinus rhythm by synchronized cardioversion. After management of tachycardia, he was started with guideline‐directed medical therapy for heart failure management, and underwent coronary angiography with stenosis of 50% at segment 1 of the diagonal branch. The patient was discharged 1 week later with an ejection fraction of 32% on echocardiography. After 1 month, he was admitted several times due to recurrence of atrial tachycardia and acute heart failure. The patient underwent an electrophysiology test, which indicated a macro reentry atrial tachycardia and successfully ablated by radiofrequency energy with the 3D mapping system. A repeat transthoracic echocardiography 1 day after ablation indicated the ejection fraction was improved from 20% to 32%. He was followed up every month, the ejection fraction was 41% at 3 months 52% at 6 months after ablation.

**Conclusions:** Tachycardia‐induced cardiomyopathy should be suspected in patients with prolonged tachycardias and unexplained heart failure. The earlier patients get appropriate management, the better prognosis they get.

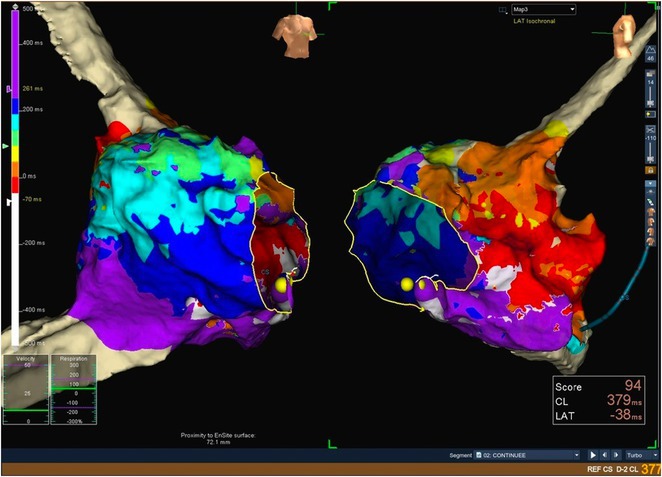

## SIGNIFICANCE OF 12‐LEAD ELECTROCARDIOGRAM MONITORING DURING WOLFF‐PARKINSON‐WHITE SYNDROME ABLATION

### **HUY NGUYEN THE NAM** ^1^, HUNG PHAM NHU^1^, MINH NGO HUNG QUANG^2^, LOC LAM MINH^2^, THAI NGUYEN HUU^2^, TUAN NGUYEN XUAN^1^, DAN NGUYEN VAN^1^, TUNG PHAM VAN^1^

#### ^1^Hanoi Heart Hospital, Ha Noi, Viet Nam,^2^Hoan My Cuu Long Hospital, Can Tho, Viet Nam

**Introduction:** Ablation of the right‐sided pathway in WPW syndrome typically presents challenges. In rare instances, alterations occur in characteristic conduction post ablation, leading to changes in some leads on the electrocardiogram (ECG), while the accessory pathway remains. We present a case study demonstrating gradual ECG changes during the ablation procedure

**Methods:** N/A

**Results:** A 60‐year‐old male patient presented to the physician with intermittent palpitations and tachycardia. An ECG confirmed the diagnosis of WPW syndrome type B. Subsequently, the patient underwent electrophysiological study using conventional methods.

Localization of the accessory pathway on the ECG suggests a paraseptal location (Figure A). Ventricular burst pacing, utilizing a stimulation train with a cycle length (CL) of 300 ms, initially demonstrates 1:1 ventricular‐atrial (VA) conduction, followed by tachycardia. Subsequent to tachycardia termination, the ablation catheter is positioned in the paraseptal region. Post‐ablation, the WPW image disappears when observing leads displayed on the screen (V1, DII). Burst pacing using the same CL reveals that ventricles and atria no longer exhibit 1:1 conduction. Notably, when observed on a 12‐lead ECG, alterations are evident in the PR segment and QRS complex in leads V1, DI, II, III, and aVF, while the delta wave remains clear in leads V3, V4, and V5 (Figure B). Subsequent catheter movement counterclockwise from the paraseptal position to the posterior of the tricuspid annulus involves searching for suspicious electrical signals and performing ablation. Following ablation of the posterior tricuspid annulus, the 12‐lead ECG exhibits only slight changes in the QRS complex in leads V2, V3, and V4, while the delta wave persists (Figure C). The catheter is further moved to the lateral tricuspid annulus for the final ablation position. Post‐ablation, burst pacing reveals Wenckebach phenomenon with a CL of 600 ms, accompanied by complete PR and QRS complex changes across all leads (Figure D)

**Conclusions:** In cases of WPW syndrome type B, meticulous monitoring of the 12‐lead ECG during ablation procedures is important.

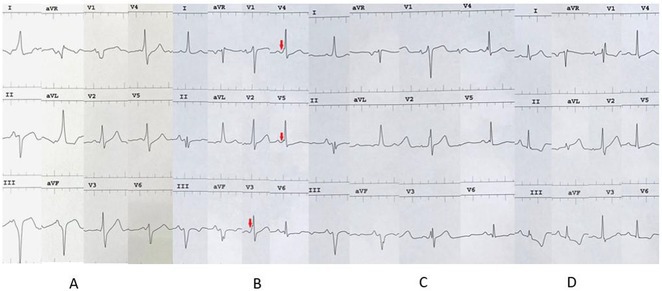

## WEARABLE RING‐TYPE BIOMEDICAL EDDY CURRENT SENSOR FOR CARDIAC ARRHYTHMIA DETECTION

### **SHIH‐HUA NI**, YEN‐LING SUNG, DONG‐YU HSU, CHIU‐YUN HUANG, DING‐NAN LAI, TING‐WEI WANG

#### National Tsing Hua University, Hsinchu, Taiwan

**Introduction:** Cardiac arrhythmias, characterized by unpredictable and random abnormal heartbeats, present significant diagnostic challenges due to their transient nature during routine clinical evaluations. The development of wearable cardiovascular monitoring technologies becomes crucial, offering continuous and long‐term monitoring capabilities. Presently, most wearable sensors rely on photoplethysmography (PPG), which must be sensitive to variations in skin tone.

**Methods:** We propose a novel sensing method using a ring‐type sensor based on biomedical eddy current sensing technology. The proposed ring sensor, measuring 2.42 cm in diameter, targets the digital artery using alternating current (AC) magnetic fields and records resonance frequency signals. It features an application‐specific integrated circuit (ASIC), an LC tank, and a wireless microcontroller unit (MCU) supporting Bluetooth data transmission.

**Results:** To validate the pulse signals from the ring‐type biomedical eddy current sensor, synchronous measurements were conducted using a gold‐standard electrocardiogram (ECG) system (MP36, Biopac). Fast Fourier transform (FFT) analysis revealed an identical frequency peak at 1.06 Hz, corresponding to 64 beats per minute (bpm), thereby confirming the sensor's reliability. To evaluate the feasibility of the ring sensor for multi‐finger application, synchronous testing was performed on the index finger, middle finger, ring finger, and pinky. FFT analysis results revealed variations across the fingers, attributed to differential blood flow patterns. Synchronous measurements with the ECG system for arrhythmia diagnosis used continuous wavelet transform (CWT), providing a detailed analysis of energy distribution and dynamic fluctuations across time, enhancing diagnostic accuracy.

**Conclusions:** This study introduces a novel ring‐type biomedical eddy current sensor, employing electromagnetic induction for cardiac arrhythmia monitoring. It offers a viable alternative to traditional optical methods, thereby advancing innovations in ring sensor technology.

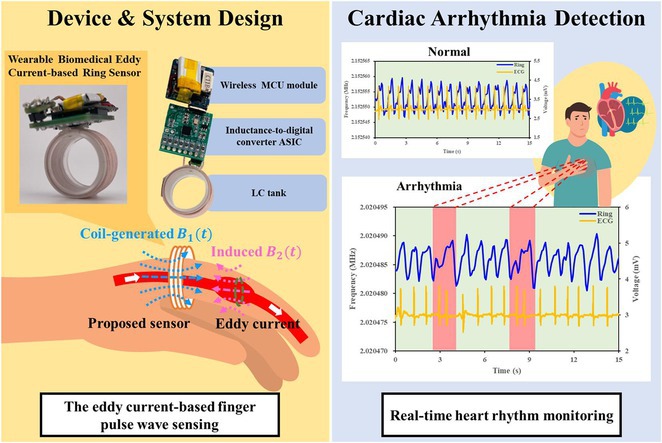

## DIFFERENCES IN OUTCOMES OF LEAD EXTRACTION BETWEEN INFECTED AND NON‐INFECTED INDICATION

### **NOBUHIRO NISHII**, TAKURO MASUDA, AKIRA UEOKA, SAORI ASADA, MASAKAZU MIYAMOTO, KOJI NAKAGAWA, HIROSHI MORITA, SHINSUKE YUASA

#### Okayama University, Okayama, Japan

**Introduction:** Background: Lead extraction plays an important role in the follow‐up of patients with cardiac implantable electric devices in the event of device infection, lead injury, or device upgrade. Lead extraction has been performed mainly for infected patients, but lead extraction for non‐infected leads is gradually increasing. We investigated the differences in outcomes and complications between the two groups.

**Methods:** We retrospectively evaluated 278 patients who underwent lead extraction at our hospital between 2010 and 2023.

**Results:** There were 175 patients in the infected group (368 leads, mean time of implantation 8.2 years) and 103 patients in the uninfected group (210 leads, mean time of implantation 7.3 years). Compared to the non‐infected group, the infected group was older, had lower eGFR and hemoglobin, and had a higher left ventricular ejection fraction. The complete success rate was 94.3% in the infected group and 84.5% in the uninfected group (p = 0.010), and the clinical success rate was 98.3% in the infected group and 86.4% in the uninfected group (p < 0.001). The major complication rate was 2.9% in the infected group and 0% in the uninfected group (p = 0.161). No distant complications related to residual leads were observed in either group.

**Conclusions:** Due to differences in strategy, the success rate of lead extraction was higher in the infected group. However, there were no major complications in the non‐infected group, and in all cases, the intended lead addition was possible. In all cases, the lead extraction strategy for non‐infected patients was considered to be within the acceptable range.

## CRYOABLATION FOR PAROXYSMAL ATRIAL FIBRILLATION IN THE POLAR SMART STUDY WITH NEWLY AVAILABLE BALLOON CATHETERS IN JAPANESE AND KOREAN CENTERS

### **JUNICHI NITTA** ^1^, YOUNG KEUN ON^2^, SHINSUKE MIYAZAKI^3^, KEIICHI ASHIKAGA^4^, OSAMU INABA^5^, TAKESHI SASAKI^6^, YUKIHIKO YOSHIDA^7^, TORSTEN KAYSER^8^, JONATHAN RAYBUCK^8^, JAMIE KWEK^8^, MASAOMI KIMURA^9^, YASUTERU YAMAUCHI^10^

#### ^1^Sakakibara Heart Institute, Fuchu, Tokyo, Japan,^2^Department of Internal Medicine, Heart Vascular and Stroke Institute, Samsung Medical Center, Sungkyunkwan University School of Medicine, Seoul, Korea, Republic of,^3^Tokyo Medical and Dental University, Tokyo, Japan,^4^Miyazaki Medical Association Hospital, Miyazaki, Japan,^5^Japanese Red Cross Saitama Hospital, Saitama, Japan,^6^National Hospital Organization Disaster Medical Center, Tokyo, Japan,^7^Japanese Red Cross Aichi Medical Center Nagoya Daini Hospital, Aichi, Japan,^8^Boston Scientific Corporation, St Paul, MN,^9^Hirosaki University Hospital, Aomori, Japan,^10^Yokohama City Minato Red Cross Hospital, Kanagawa, Japan

**Introduction:** Catheter ablation of atrial fibrillation (AF) is becoming increasingly common in Japan and South Korea. Limited data is available on the novel single‐shot cryoablation catheters POLARx (28mm) and the variable size POLARx FIT (28/31mm) (Boston Scientific), especially in Japanese and Korean populations. The POLAR SMART study is designed to provide insight on the safety, efficacy and use of these cryoballoons.

**Methods:** This is a multi‐center study (19 centers; 17 Japan, 2 South Korea) of patients who received pulmonary vein isolation (PVI) for treatment of paroxysmal AF. Ablation were conducted based on center standard of care, data were collected on 2 treatment cohorts: PVI with no additional locations, and PVI combined with RF ablation of additional sites (i.e CTI, roof, PW etc.; PVI+). Patients will be followed for 12 months with blanking period of 3 months post procedure after which AF recurrence, re‐do procedures, adverse events, and ongoing anti‐arrhythmic medications were collected. The rate of acute procedural success, defined by electrical isolation of all pulmonary veins (PV) using the cryoablation catheter, was collected.

**Results:** 293 patients were enrolled; 202 (68.9%) treated with POLARx and 91 (31%) with dual‐diameter POLARx FIT. 223 patients received just PVI and 70 received PVI+. Mean age was 65±12 years, and 34.1% were female. In patients that received just PVI, patients required an average of 5.5±2.1 applications/patient or 1.4±0.1 application/vein. Average time‐to‐isolation was 44±32 sec with average Nadir temperature of ‐57±6.4°C and thaw time of 25±15 sec. Median procedure time was 75 mins (IQR, 60‐99) and median LA dwell‐time was 41 mins (IQR, 33‐55). In these patients, acute success by vein was 98.3% (1249/1270 veins) and acute success by patient was 96.4% (215/223 patients). At present there are 3 (1%) ongoing cases of phrenic nerve impairment.

**Conclusions:** These acute data suggest that the POLARx cryoablation system is safe and effective for treatment of AF with a low PNI rate, and 1.4 applications per vein.

## TRANSIENT ISCHEMIC ATTACK CAUSED BY INCIDENTAL LATE ELECTRICAL ISOLATION OF LEFT ATRIAL APPENDAGE; A CASE REPORT

### **MASATSUGU NOZOE**, SAYANA KURAOKA, TORU KUBOTA

#### Saisekai Fukuoka General Hospital, Fukulka, Japan

**Introduction:** Radiofrequency catheter ablation (RFCA) is effective for antiarrhythmic drug refractory atrial fibrillation (AF) and several retrospective analyses have also shown that RFCA reduces the risk of ischemic stroke in patients with AF. However, mechanical stasis imparted by an electrically disconnected left atrial appendage (LAA) may predispose to thromboembolism.

**Methods:** N/A

**Results:** A 69‐years old woman with CHADS2 score 0 point had a history of repeated catheter ablation for long standing persistent AF. Although she maintained sinus rhythm, she suddenly developed transient ischemic attack (TIA). She was treated as cryptogenic brain infarction with Dabigatran 300mg daily, and discharged without any symptoms.At the first and the second session of AF ablation, liner ablation at left atrial (LA) anterior was performed, however incomplete block line resulted in a recurrence of mitral flutter. At the third session (5 years prior to TIA) mitral flutter did not cease by energy application at anterior mitral line (AML), but liner ablation at left mitral Isthmus (LMI) terminated this tachycardia. Because she maintained sinus rhythm, we discontinued anticoagulation 1 year after the third session (4 years prior to TIA).She complained palpitation and presyncope again 4 years after TIA. Voltage mapping at the fourth session revealed large scar at the antero‐lateral segment of LA including LAA. The induced tachycardia was diagnosed as localized reentrant tachycardia at inferior LA, and energy application at this point successfully terminated this tachycardia. We speculated that incidental late electrical isolation of LAA caused TIA without recurrence of AF at that time.

**Conclusions:** Incomplete electrical block line at AML and complete block line at LMI caused late incidental isolation of LAA due to age‐related degeneration. Incidental isolation of LAA resulted in subsequent TIA without AF recurrence. Two liner ablations that sandwich the LAA should be avoided even if one of these were incomplete.

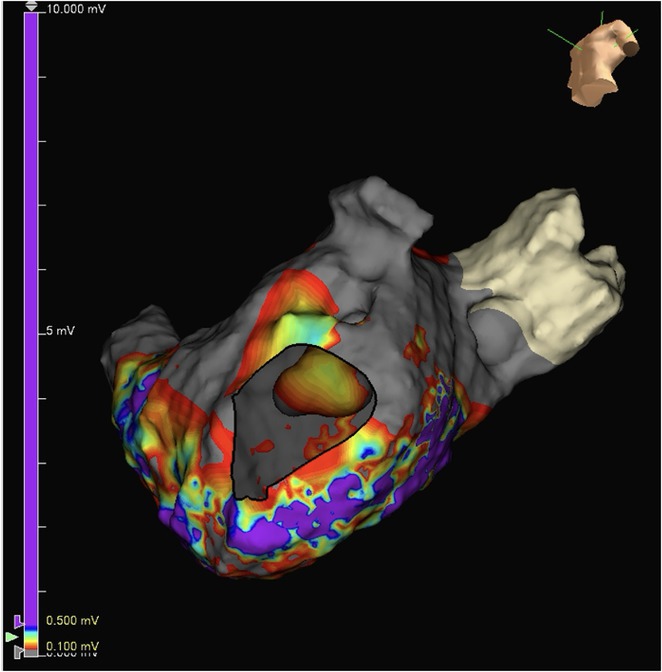

## VENOPLASTY DURING LEFT‐BUNDLE PACING UPGRADE IN PATIENT WITH PACEMAKER‐INDUCED CARDIOMYOPATHY

### **ADITYA NURTANTIJO**, GIKY KARWIKY, MOHAMMAD IQBAL, CHAERUL ACHMAD

#### Hasan Sadikin General Hospital, Bandung, Indonesia

**Introduction:** Venous stenosis is a known complication after intracardiac device implantation. Multiple lead and procedures were found to increase incidence of venous stenosis. Pacemaker‐Induced Cardiomyopathy (PICM) commonly defined as reduction in left ventricular (LV) function in the setting of right ventricular (RV) pacing. PICM may be associated with the onset of clinical heart failure but may be avoid by newer pacing options such as Left Bundle Pacing (LBP).

**Methods:** N/A

**Results:** A male 73‐year‐old patient was referred to tertiary hospital for PPM reimplantation. Patient was diagnosed with total AV block and was implanted Single Chamber RV Pacing PPM in 2017. During 2 years evaluation after PPM implantation, patient was having dyspnea on effort complain and echocardiography showed reduced Left Ventricular Ejection Fraction (LVEF) 43%. Patient was diagnosed with PICM and was on medical therapy for heart failure. Patient was scheduled to have upgrade to LBP. Access was made from cephalic vein to superior vena cava using 7F sheath. Resistance was found during wire introduction; hence venography was done. Venography showed severe stenosis at the left innominate vein. Intervention was done using hydrophilic guide wire and using Armada Balloon 35 x 80 mm, inflated to 6 atm. Post balloon inflation, stenosis was improved into mild stenosis. After venoplasty, bipolar lead was positioned at the left bundle branch area and lead was connected to PPM. During 1 week evaluation, LVEF was improved to 54%.

**Conclusions:** Venous stenosis was recognized complication in cardiac device implantation. In this patient, we performed venography before making decision considering history of cardiac device implantation. The equipment was typically used in peripheral artery angioplasty. Balloon venoplasty is considered simpler approach to manage venous obstruction compared to other alternative such as contralateral implant, right‐sided implantation, or jugular access considering the increased risk of complication and patient's comfort.

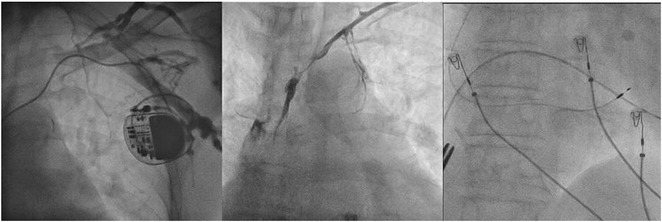

## PREVALENCE OF GASTRICHYPOMTILITY AFTER NOVEL CRYOBALLOON ABLATION

### **ATSUHITO ODA**, YUMI YASUI, HIROFUMI ARAI, KAZUYA MURATA, YUICHIRO SAGAWA, YASUTERU YAMAUCHI

#### Minato Red Cross Hospital, Yokohama, Kanagawa, Japan

**Introduction:** Conventional cryoballoon ablation (CBA) catheters have been widely used for pulmonary vein isolation (PVI). Although cryoballoon ablation is a safe and effective therapy, the enhanced cooling properties may cause collateral damage to another organ. Gastric hypomotility (GH) has been reported to be caused by damage to the vagal nerve, which may lead to gastrointestinal symptoms. The POLARx cardiac cryoablation system was recently introduced. It has been reported that the nadir cryoballoon temperature with the POLARx is lower than with the AFA‐Pro. However, there are no reports on GH after PVI and CB roof using the novel POLARx cryoballoon. We investigate the prevalence and predictors of GH when using the POLARx.

**Methods:** A total of 41 patients with paroxysmal AF(PAF) undergoing CBA with POLARx were included. All patients underwent esophagogastroscopy 2 days after ablation under overnight fasting conditions; GH was defined as the retention of food in the stomach. All patients underwent three‐dimensional computed tomography (3DCT) prior to ablation. Anatomical factors including distance between PV and esophagus were analysed by 3DCT using a computer workstation.

**Results:** Baseline patient characteristics are shown in Table. GH was confirmed in 12 patients. As shown in the table, patients with a shorter distance from RIPV to esophagus, lower balloon and esophageal temperature during right inferior PVI were more likely to be at high risk of GH

**Conclusions:** The prevalence of GH with POLARx is 29.3%. The esophageal location obtained with 3DCT helps to identify high‐risk populations for GH.

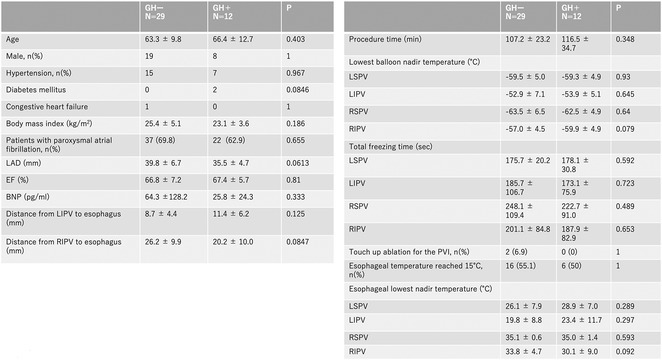

## COMPARATIVE IMPACT OF DIFFERENT ENERGY SOURCES ON ATRIAL FUNCTION AFTER PULMONARY VEIN ISOLATION: A SINGLE‐CENTER RETROSPECTIVE ANALYSIS

### **KOJIRO OGAWA** ^1^, HIRO YAMASAKI^2^, TOMOFUMI NAKATSUKASA^3^, YUICHI HANAKI^1^, AKIRA KIMATA^2^, YUKI KOMATSU^1^, MIYAKO IGARASHI^2^, TOMOKO ISHIZU^2^

#### ^1^University of Tsukuba Hospital, Cardiology, Tsukuba, Japan,^2^University of Tsukuba, Department of cardiology, Tsukuba, Japan,^3^The Sakakibara Heart Institute of Okayama, Department of Cardiology, Okayama, Japan

**Introduction:** Pulmonary vein isolation (PVI) procedures for atrial fibrillation (AF) are widely performed using various energy sources including radiofrequency and balloon ablation. The impact of different energy sources on postoperative atrial function remains unclear.

**Methods:** In a single‐center retrospective observational study, we analyzed 147 patients with non‐valvular paroxysmal AF who underwent transthoracic echocardiography, including strain analysis of left atrium (LA), under sinus rhythm within one month before and six months after the procedure. Only patients treated with PVI for the LA were included in the analysis. Patients were grouped by energy source into radiofrequency ablation(RF, n=64), cryoballoon ablation (CB, n=42), and hot balloon ablation(HB, n=41).

**Results:** No significant differences were observed in baseline characteristics before the procedure; however, significant differences were found the day after the procedure in CKMB and CRP among the three groups (RF vs. CB vs. HB; CKMB: 4.2±3.1 vs. 26.5±16.0 vs. 10.3±6.0, p<0.001; CRP: 0.58±0.39 vs. 0.95±0.76 vs. 0.89±0.77, p=0.005). There were no significant differences in atrial strain indicators before and after the procedure, but significant differences were observed in LA reservoir strain (LASr) and contractile strain (LASct) in patients aged 75 and over (RF vs. CB vs. HB; LASr: 4.4±6.4 vs. ‐0.8±7.2 vs. ‐3.2±4.4, p=0.044; LASct: 2.6±4.0 vs. ‐1.9±4.7 vs. ‐3.0±3.6, p=0.014).

**Conclusions:** Within six months post‐procedure, the impact of different energy sources on left atrial function appears minor with PVI alone. However, in older elderly patients, variations in energy sources may be associated with significant post‐procedural declines in atrial function, suggesting that baseline functional deterioration could predispose to more evident atrial myocardial damage from the ablation.

## SUCCESSFUL EPICARDIAL ABLATION OF PERI‐MITRAL FLUTTER INVOLVING THE VEIN OF MARSHALL

### **SHO OGISO**, SHUHEI KOBAYASHI, HIDEHIRA FUKAYA, YUKI ARAKAWA, HIRONORI NAKAMURA, NARUYA ISHIZUE, JUN KISHIHARA, SHINICHI NIWANO, JUN OIKAWA, JUNYA AKO

#### Kitasato University School of Medicine, Sagamihara, Japan

**Introduction:** N/A

**Methods:** N/A

**Results:** The case report describes a 73‐year‐old male who experienced recurrent atrial tachycardia (AT) after undergoing three radiofrequency catheter ablation (RFCA) procedures for atrial fibrillation. Prior interventions included pulmonary vein isolation, cavo‐tricuspid isthmus ablation, and mitral isthmus (MI) ablation. The intracardiac electrograms recorded during the AT with a tachycardia cycle length (TCL) of 310 msec showed proximal to distal activation sequence in the coronary sinus (CS) catheter. The AT activation map of the left atrium revealed a peri‐mitral flutter pattern, with a conduction gap in the MI line and a centrifugal activation pattern from the 1 o’clock position of the mitral valve annulus; however local activation time recorded was only 250 msec of TCL (80%). This suggests that there is an epicardial pathway bridging the MI line. Following CS angiography, a 1.8‐Fr electrode catheter was inserted into the vein of Marshall (VOM), where additional epicardial activation mapping was performed, revealing that the total activation time matched the AT cycle length. The post‐pacing interval (PPI) minus TCL was identical at the mid VOM, indicating a peri‐mitral flutter mediated through the VOM. RFCA at site A (right bottom, Figure), the endocardial breakthrough site via VOM, terminated the tachycardia; however, the AT recurred immediately. Additional RFCA procedures were performed from the proximal part of great cardiac vein to the ostium of the VOM (site B, right bottom, Figure), which were the successful ablation sites. This case illustrates that the electrode catheter inserted into the VOM was effective for diagnosing the AT circuit, and RFCA from the epicardial site was necessary for the complete termination of the AT.

**Conclusions:** N/A

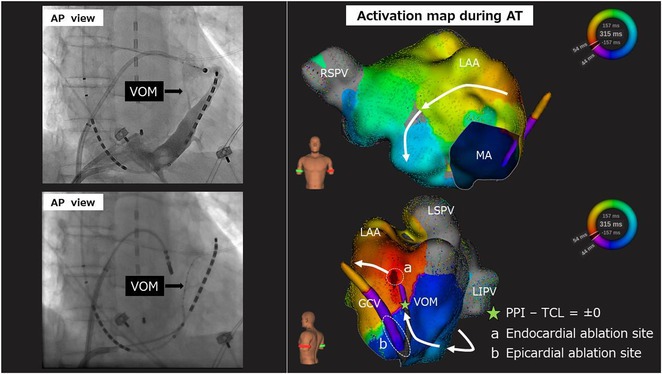

## LEFT BUNDLE BRANCH OPTIMIZED CARDIAC RESYNCHRONIZATION THERAPY ACHIEVES A HIGH RESPONDER RATE WITH QRS NARROWING

### **AKIHIRO OKA**, MASATOMO OZAKI, YUYA SUDO, MASAHIRO SOGO, MASAHIKO TAKAHASHI, KEISUKE OKAWA

#### Kagawa Prefectural Central Hospital, Takamatsu, Japan

**Introduction:** The necessity of a left ventricular (LV) pacing lead implantation for cardiac resynchronization therapy (CRT) has been discussed because left bundle branch area pacing (LBBAP) CRT alone has been expected to be more effective than conventional biventricular pacing CRT. However, the effect of LBBAP with LV pacing (LBB‐optimized CRT [LOT‐CRT]) as compared to LBBAP‐CRT remains unclear. We investigated the effect of LOT‐CRT on the QRS width as compared to LBBB‐CRT and on the LV function 6 months after the operation.

**Methods:** We retrospectively studied 20 consecutive patients who underwent LOT‐CRT. We compared the QRS width between that during LOT‐CRT and LBBAP‐CRT during the acute operative period and evaluated the responder rate of the LOT‐CRT based on the changes in the LV parameters assessed by echocardiography before and 6 months after the operation. We defined a super responder to CRT as an LV ejection fraction (LVEF) increase of ≥10% and/or LV endo‐systolic volume (LVESV) decrease of ≥30%.

**Results:** Among the 20 patients, 16 (80%) exhibited sinus rhythm and 19 (95%) LBB block. The QRS width was 164±17msec before the operation and significantly shortened after both the LOT‐CRT (115±14msec) and LBBAP‐CRT (121±13msec), respectively (p<0.001). The QRS width during the LOT‐CRT was significantly shorter than that during the LBBAP‐CRT (p=0.015). Six months after the LOT‐CRT operation, 16 patients (80%) had an LVEF increase of ≥10% and 15 (75%) an LVEDV decrease of ≥30%. The super responder rate was 85%.

**Conclusions:** LOT‐CRT achieved a greater QRS narrowing than the LBBAP‐CRT and a high responder rate at 6 months after the operation, suggesting the necessity for an LV pacing lead even in this conduction system pacing era.

## OPTIMIZING ABLATION SITES FOR RE‐CONDUCTION OF PULMONARY VEIN IN PATIENTS WITH ATRIAL FIBRILLATION: A NOVEL APPROACH USING EMPHASIZE SETTINGS

### **SHUHEI OKAJIMA**, YUHI FUJIMOTO, HIROAKI HIRAYAMA, MAKOTO KOBAYASHI, NOBUAKI ITO, MASATO HACHISUKA, HIROSHIGE MURATA, YOSHIYASU AIZAWA, KENJI YODOGAWA, WATARU SHIMIZU, KUNIYA ASAI, YU‐KI IWASAKI

#### Nippon Medical School, Tokyo, Japan

**Introduction:** Although catheter ablation techniques for atrial fibrillation (AF) have advanced, a certain number of cases of re‐conduction after pulmonary vein isolation (PVI) still exist and are the most common cause of recurrence of atrial tachyarrhythmias. In some cases, the diversity of anatomical wall thickness and fiber orientation around the PVs might contribute to re‐conduction. Identifying the optimal target ablation site of re‐conduction could reduce unnecessary ablation and decrease the risk of complications. We investigated the utility of using the Emphasize settings in the emphasis map obtained by combining activation mapping and peak frequency mapping in the EnSite X system to determine the optimal ablation sites compared to the conventional map based on the activation mapping.

**Methods:** Patients undergoing AF ablation of PVI with the EnSite X system who had recurrent cases or had PVI re‐conduction at the initial ablation were included. We retrospectively analyzed the cases from October 2021 to April 2024. In all cases, bipolar voltage and activation maps for the left atrium and PVs were obtained by a 16‐pole grid catheter (Advisor HD Grid) during atrial pacing or sinus rhythm. The Emphasis map (a combination of local activation timing and peak frequency maps) was performed to obtain the Emphasize settings indicating the optimal ablation sites (E‐group: Figure). Another group determined the ablation sites based on the activation mapping (A‐group).

**Results:** Sixty‐four patients (67 ± 9 years, 50 males) and 110 PVs were analyzed. The gaps of 55 and 66 points of PVI were observed in the E‐group and A‐group, respectively. The number of needs for the ablation points to successfully eliminate gaps and total energies in each PV was markedly smaller in the E‐group (1.8 ± 1.2 vs. 7.2 ± 4.4 points, p<0.001, 3428 ± 1828 vs. 5670 ± 3826J, p<0.001). The optimal Emphasize setting was 349 ± 69 Hz in the E‐group.

**Conclusions:** The optimal ablation site was visualized by adjusting the Emphasize settings, and the number of ablation points to re‐isolation of PV might be markedly reduced.

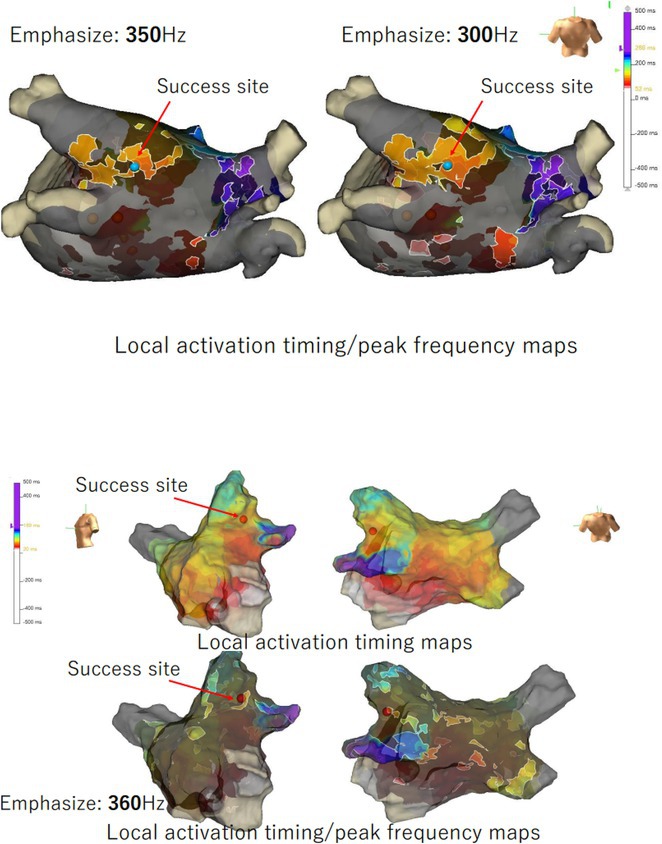

## ENDOTHELIAL FUNCTIONAL IMPROVEMENT AND THE OUTCOMES AFTER CATHETER ABLATION OF PERSISTENT ATRIAL FIBRILLATION

### **KEISUKE OKAWA**, YUYA SUDO, AKIHIRO OKA, MASAHIRO SOGO, MASATOMO OZAKI, MASAHIKO TAKAHASHI

#### Kagawa Prefectural Central Hospital, Takamatu, Japan

**Introduction:** Endothelial function (EF) could be damaged by atrial fibrillation (AF) persistence and could be reversed by sinus rhythm restoration with AF ablation. However, the impact of the improvement in the EF post‐ablation on the outcomes is unknown. This study aimed to investigate the relationship between the changes in the EF and cardiovascular events after catheter ablation of persistent AF.

**Methods:** We conducted a prospective cohort study of patients with persistent AF who underwent a first‐time ablation and for whom the EF was assessed by the peripheral vascular reactive hyperemia index (RHI) before and 6 months after the ablation. Cardiovascular events included strokes, heart failure, arteriosclerotic disease, venous thromboses, and ventricular arrhythmias and/or sudden cardiac death. We determined the cutoff value of the increase in the RHI for cardiovascular events and divided the patients into two groups according to whether they were above and below that value: improved EF and non‐improved EF groups. We compared the 4‐year incidence of cardiovascular events post‐AF ablation between the groups.

**Results:** The cutoff value was determined as a 1.25‐fold increase in the RHI. Among the 537 enrolled patients, 149 (27.7%) had an improved EF. The 4‐year cumulative incidence of cardiovascular events was significantly lower in the improved EF group than non‐improved EF group (5 [6.8%] vs. 35 [14.8%], log‐rank P=0.033). An increase in the RHI ≥ 1.25‐fold was determined as an independent predictor of cardiovascular events (hazard ratio 0.36, 95% confidence interval 0.14‐0.92, P=0.032).

**Conclusions:** An improvement in the EF post‐AF ablation in patients with persistent AF was associated with a lower long‐term incidence of cardiovascular events.

## OPTIMIZATION OF AV AND VV DELAY IN PATIENT HFREF WITH IVCD IMPLANTED LOT‐CRT

### **ANDRIANUS OKTOVIANTO** ^1^, GIKY KARWIKY^2^

#### ^1^Dr. M. Soewandhie General Hospital, Kota Surabaya, Indonesia,^2^Dr. Hasan Sadikin Central General Hospital, Kota Bandung, Indonesia

**Introduction:** Optimal management of the AV and VV delay has become important method to improve left intraventricular synchrony of LOT‐CRT therapy. We present a case of the greater electrical resynchronization through AV and VV delay optimization in male receiving LOT‐CRT for HFrEF with IVCD.

**Methods:** N/A

**Results:** A 40 yo male was hospitalized for intractable NYHA FC III of HF of a non‐ischaemic aetiology. The ECG showed 1^st^ degree AVB and IVCD with QRSd of 215 ms. He had reduced LVEF of 20%, dyskinetic base to mid IVS. The patient underwent LOT‐CRT. Optimal LV quadripolar lead pacing configuration, AV delay and VV delay was determined during implantation in search of the narrowest QRSd. The LV1 to LV4 gave the satisfactory pacing parameter without resulting high capture threshold and phrenic nerve stimulation. Due to the challenges in achieving LBB capture, LVSP was adopted as a bailout strategy for LBBP. The bipolar‐LVSP mode alone paced QRSd and LVAT were 182ms and 120ms. The LVSP capture threshold at implant was 0.5V at 0.4ms and Rwave amplitude >20mV. Acute response was measured by QRSd immediately after implantation. Empirically program device to a fixed AV delay (SAV of 120ms;PAV of 150ms) was used. The VV delay (LBBB pre‐excitation) of 70ms had shown to be the most effective resynchronization resulting narrowest QRSd of 120ms. At 12‐weeks follow up, patient underwent optimization reprogramming. The patient was symptomatically much improved in NYHA FC I yet his LVEF remained 20%. Repeat echocardiography showed more synchronous activation of LV. The narrowest QRSd was still seen at a same value of programmed AV and VV delay during acute programming. His latest pacing check showed that both the threshold of the LV lead and LBBAP remained stable. Overall, the percent of atrial pacing and LOT‐CRT ventricular pacing rate was 13.0% and 99.6%, respectively.

**Conclusions:** Individualized adjustments of the AV and VV delay result in sequential LOT‐CRT pacing and directly modify the sequence of atrioventricular and intraventricular activation, which may achieve narrower QRSd and better LV electrical synchrony.

## ASSESSMENT OF SUCCESSFUL RADIO FREQUENT APPLICATION WITH IMPEDANCE DROP AND ABLATION INDEX IN PULMONARY VEIN ISOLATION FOR ATRIAL FIBRILLATION

### **YUSUKE OKUYAMA** ^1^, TOSHIKI KAZUMA^2^, KOUHEI UEDA^1^, DAISUKE HATTORI^1^, MIYU SUGIMOTO^1^, CHISAKI TAKAHARA^1^, KEIKOKU KASHO^1^, ATSUSHI TAMURA^1^, MASAHIKO JIKAN^1^, KANTA NAKAJIMA^1^, KAZUNORI MASUDA^1^, SHUNZO MATSUOKA^1^, YOSHIHISA NAKAGAWA^3^

#### ^1^Uji‐Tokushukai Medical Center, Uji, Japan,^2^Nagoya Heart Center, Nagoya, Japan,^3^Shiga University of Medical Science, Otsu, Japan

**Introduction:** Ablation index (AI) has been reported to be important to form durable lesion in catheter ablation (CA) for atrial fibrillation (AF). Impedance drop (ID) had also been thought to be important for adequate lesion formation with radio frequent (RF) application. However, relationship of ID and AI is unclear because AI does not consist of ID. In addition, there are some modes of ID and it is unclear that what ways of dropping impedance is effective. We investigated association of parameters of ablation lesion with RF including AI and ID. What ways of ID was effective for acute successful lesion formation was also evaluated.

**Methods:** Ablation lesions made by RF application at power of 35 watts during CA for AF were studied. Only lesions created for the first PVI line were analyzed. These points were classified into 5 groups according to the ways of ID. Points with gradual ID, 2‐phased ID, and steep ID were respectively classified as group 1, 2, and 3. Points with no ID, increase in impedance, or RF application time (T_RF_) of less than 5 seconds were excluded. Acute success rate, decrease in impedance, T_RF_, contact force (CF) and value of AI were investigated, as well as the relationship with AI value and acute success rate, for each group.

**Results:** From 24 consecutive cases, 1579 lesions by RF application at 35 and 50 watts were obtained, of which 1084 were investigated. Group 1, 2, and 3 consisted of 303, 718, and 63 points, respectively. Acute success rate for each group were 92.4, 98.3, and 95.2%, and significantly higher in groups 2 and 3 (p<0.05). AI value in group 2 was the highest (462±44 vs 471±48 vs 455±66, p<0.05). ID and CF were largest in Group 3 and smallest in Group 1 (7.9±3.5 vs 12.7±5.5 vs 14.3±6.3 ohm, p<0.05. 12.5±4.9 vs 14.8±6.0 vs 16.9±6.4 g, p<0.05) T_RF_ was longest in Group 1 and shortest in Group 3 (24.5±4.6 vs 23.0±4.5 vs 19.7±6.1, p<0.05).

**Conclusions:** Acute success rate and AI values were significantly higher in group 2 than the others. It is important to obtain 2‐phased ID as well as AI values to create adequate lesions with RF at power of 35 watts.

## DETAILED PREOPERATIVE ASSESSMENT FOR SUCCESSFUL CATHETER ABLATION IN AN OCTOGENARIAN WITH PERSISTENT ATRIAL FIBRILLATION COMPLIED BY COR TRIATRIATUM SINISTER

### **YUSUKE OKUYAMA** ^1^, ATSUSHI TAMURA^1^, KOUHEI UEDA^1^, MIYU SUGIMOTO^1^, KEIKOKU KASHO^1^, DAISUKE HATTORI^1^, CHISAKI TAKAHARA^1^, MASAHIKO JIKAN^1^, KANTA NAKAJIMA^1^, KAZUNORI MASUDA^1^, SHUNZO MATSUOKA^1^, YOSHIHISA NAKAGAWA^2^

#### ^1^Uji‐Tokushukai Medical Center, Uji, Japan,^2^Shiga University of Medical Science, Otsu, Japan

**Introduction:** Cor Triatriatum Sinister (CTS) is a rare but sometimes complicates atrial fibrillation (AF). AF causes symptoms to be relieved by catheter ablation (CA), but CA for AF with CTS has been scarcely reported. Because CTS can be associated with other congenital heart disease, detailed preoperative assessment is important.

**Methods:** N/A

**Results:** An 80‐year‐old male referred our institution for shortness of breath which had persisted for two months when he was first diagnosis with AF. Transthoracic echocardiography revealed an enlarged LA and a membrane dividing the LA into two chambers. Transesophageal echocardiography demonstrated the membrane crossing from fossa ovalis (FO) to the Coumadin ridge and the accessory (dorsal) chamber (AC) was more in contact with FO. Computed tomography showed that all pulmonary veins (PVs) flowed into AC, and no PV anomaly. No other heart anomaly was revealed and there was no thrombus in LA. Based on these assessment, pulmonary vein isolation (PVI) with CA was thought to be performed safely. Transseptal puncture was conducted with intracardiac echocardiography for precise catheterization to AC. PVI was performed successfully. The patient was discharged 4 days after procedure without any complications. His symptoms improved after this procedure and sinus rhythm had been maintained without antiarrhythmic drug during 18‐month follow up period.

**Conclusions:** CTS is a rare anomaly accounting for 0.1% of congenital heart disease but complicates symptomatic AF. Detailed preoperative anatomical assessment with many imaging modalities helps us accomplish safe and effective CA for AF with CTS, even in elder patients.

## ASSOCIATION BETWEEN LEFT ATRIAL WALL SHEAR STRESS MEASURED USING 4D FLOW MRI AND THE CARDIOEMBOLIC STROKE RISK IN PATIENTS WITH PAROXYSMAL ATRIAL FIBRILLATION

### **TAKUYA OMURO** ^1^, YASUHIRO YOSHIGA^1^, MASAKAZU FUKUDA^1^, HIRONORI ISHIGUCHI^1^, SYOHEI FUJII^1^, MASAHIRO HISAOKA^1^, SHINTARO HASHIMOTO^1^, MAKOTO ONO^1^, NORIKO FUKUE^2^, AKIHIKO SHIMIZU^3^, MOTOAKI SANO^1^

#### ^1^Yamaguchi University Graduate School of Medicine, Ube, Japan,^2^Yamaguchi University Health Science Center, Ube, Japan,^3^Ube‐kohsan Central Hospital, Ube, Japan

**Introduction:** Atrial fibrillation (AF) induces structural and electrical remodeling of the left atrium (LA), thus altering LA function and intracardiac blood flow. Associations between various hydrodynamic indicators and cardioembolic stroke risk have been identified in intracardiac blood flow analysis in recent years. Although wall shear stress (WSS) has been implicated in thrombus formation, the relationship between LA‐WSS and cardioembolic stroke risk remains unclear. In this study, we measured the LA‐WSS in patients with paroxysmal AF (PAF) using 4D‐flow magnetic resonance imaging (MRI) and investigated its association with the cardioembolic stroke risk score and history of stroke.

**Methods:** Four‐dimensional flow MRI was performed on 17 patients with PAF before catheter ablation (in the sinus rhythm during MRI scan, 11 males, 69 ± 9 years old). The mean LA‐WSS over the cardiac cycle was quantified, and its correlation with CHADS_2_, CHA_2_DS_2_‐VASc scores, and LA low voltage areas (LVAs) was investigated. The LA‐WSS was compared between patients with and without a history of stroke.

**Results:** The median CHADS_2_ and CHA_2_DS_2_‐VASc scores were 1 and 2, respectively. The mean LA‐WSS was 1.93 ± 0.37 Pa. LA‐WSS showed a significant negative correlation with CHADS_2_ score (r = ‐0.76, p < 0.001), CHA_2_DS_2_‐VASc score (r = ‐0.67, p = 0.003), and LVAs (r=‐0.53, p=0.029). Furthermore, LA‐WSS was significantly lower in patients with a history of stroke (n=2) compared to those without a history of stroke (1.24 ± 0.19 Pa vs. 2.02 ± 0.07 Pa, p = 0.002).

**Conclusions:** Patients with PAF at a high risk of cardioembolic stroke demonstrated decreased LA‐WSS. The LA‐WSS may, therefore, serve as a new marker of cardioembolic stroke risk.

## THE DIFFERENT ARRHYTHMIC EVENTS AND CARDIOVASCULAR OUTCOMES IN PATIENTS HOSPITALIZED WITH COVID‐19 INFECTION DURING THE PANDEMIC OF 2021 AND 2022 IN TAIWAN: A SINGLE CENTER EXPERIENCE

### JIE YWI ONG

#### Taipei Wanfang Municipal Hospital, Taipei City, Taiwan

**Introduction:** Coronavirus disease 2019 (COVID‐19) was associated with cardiovascular (CV) complications, including myocardial infarction (MI), thromboembolism, heart failure (HF), and cardiac arrhythmias. This study aims to compare the arrhythmic events and CV outcomes of hospitalized COVID‐19 infection patients in 2021 and 2022 in Taiwan.

**Methods:** A retrospective study was conducted on a cohort of 186 patients hospitalized between May, 2021 and October, 2021, and another cohort of 308 patients hospitalized between March, 2022 and August, 2022 with confirmed COVID‐19 infection. We compared the arrhythmic events during hospitalization, treatment administered, mortality rates, and major adverse cardiovascular events (MACE), including non‐fatal ischemic stroke or systemic embolism, non‐fatal MI, CV death, HF in 2021 and 2022.

**Results:** There was a non‐significant increase in arrhythmic events among patients hospitalized in 2022 compared to 2021 (44.2% vs 36.6%, p = 0.097). The most frequently recorded arrhythmias were sinus tachycardia (24.7% and 22.0% in 2022 and 2021 respectively, p=0.505), atrial premature complexes (11.0% and 6.5%, p=0.089) and atrial fibrillation (AF) (9.4% and 7.0%, p=0.349). There were 8 (2.6%) and 10 (5.4%) cases of new onset AF during hospitalization in 2022 and 2021 cohorts respectively. Mortality rates were comparable between the two years (20.3% vs 19.9%, p = 0.949), but the incidence of MACE was lower among patients hospitalized in 2022 compared to 2021 (4.6% vs 9.1%, p = 0.041). Logistic regression revealed that arrhythmic events during hospitalization was a significant predictor of mortality in both 2021 (age‐adjusted OR: 4.45, 95% CI: 1.94‐10.62) and 2022 (age‐adjusted OR: 2.81, 95% CI: 1.58‐5.12) cohorts.

**Conclusions:** There are some discrepancies between the arrhythmic events and CV outcomes of COVID‐19 infection patients during the pandemic of 2021 and 2022 in Taiwan. Frequent arrhythmic events remained a significant concern and an important predictor of mortality.

## CHANGES IN TERMINATION MODE ACCORDING TO THE TIMING OF PVC DELIVERY DURINGAVNRT WITH A BYSTANDER NODOVENTRICULAR PATHWAY CONNECTING TO THE FAST PATHWAY

### **KOUMEI ONUKI** ^1^, KOICHI NAGASHIMA^2^, YASUHARU MATSUNAGA‐LEE^3^, MASATO FUKUNAGA^1^, KEIGO MISONOU^1^, MAIKO KURODA^1^, HIROYUKI KONO^1^, TOMONORI KATSUKI^1^, REI KUJI^1^, KENGO KORAI^1^, MICHIO NAGASHIMA^1^, KENICHI HIROSHIMA^1^, KENJI ANDO^1^

#### ^1^Kokura Memorial Hospital, Kitakyushu city, Japan,^2^Nihon University School of Medicine, Ibaraki, Japan,^3^Osaka Rosai Hospital, Sakai, Japan

**Introduction:** Diagnosing atrioventricular nodal reentrant tachycardia (AVNRT) with a bystander concealed nodoventricular pathway (cNVP) is challenging and requires careful interpretation. Furthermore, it is unclear whether the cNVP itself has decremental properties.

**Methods:** N/A

**Results:** A 76‐year‐old man underwent an electrophysiological study for narrow QRS tachycardia. At baseline with the standard catheter position, the AH and HV intervals were 136 ms and 58 ms, respectively. Retrograde conduction showed decremental properties with the earliest atrial activation (EAA) seen in the His region. The clinical tachycardia, with a cycle length of 448 ms and a VA interval of 68 ms, initiated spontaneously without an AH jump, exhibiting an identical atrial sequence. A premature ventricular contraction (PVC) during His refractoriness consistently terminated the tachycardia without atrial capture (Picture A). Furthermore, a slightly earlier PVC, still within His refractoriness, failed to reset the subsequent His electrogram but terminated the tachycardia without atrial capture one cycle later (Picture B). Ventricular overdrive pacing terminated the tachycardia with VA block within the transition zone. Given these findings, slow‐fast AVNRT with a bystander cNVP was diagnosed. The tachycardia termination was caused by HA block, which indicated that the cNVP was connecting to the fast pathway. The most plausible mechanism for the tachycardia termination by a His‐refractory PVC is that the PVC conducted over the cNVP and caused a conduction block in the retrograde fast pathway. Similarly, a slightly earlier PVC terminated the tachycardia by penetrating the fast pathway. However, the prematurity of the earlier PVC may have led to decremental conduction over the cNVP, resulting in tachycardia termination one cycle later. Cryoablation at the slow pathway region rendered the tachycardia non‐inducible.

**Conclusions:** The termination mode of AVNRT by His‐refractory PVC at various coupling intervals might be useful for delineating the cNVP and assessing its decremental properties.

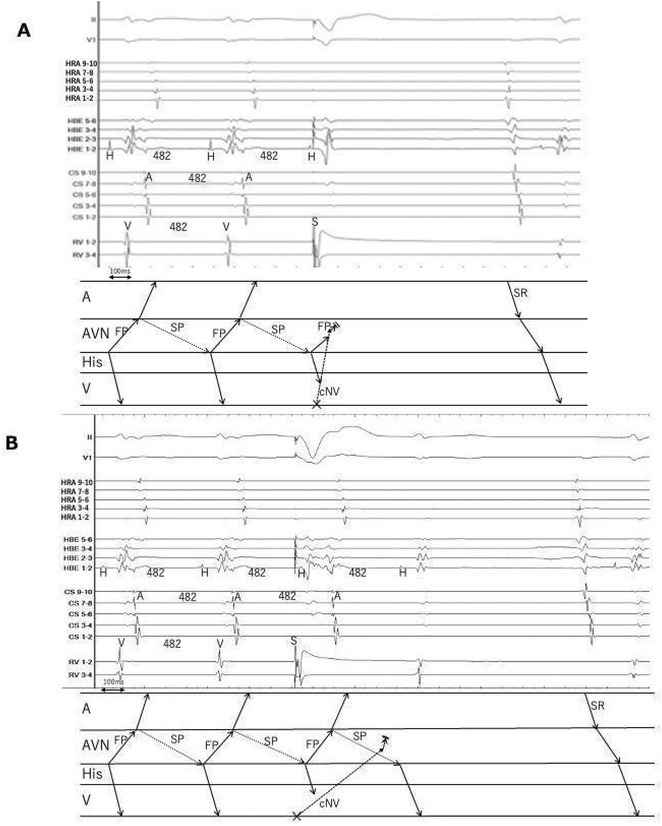

## CLINICAL EFFECTIVENESS OF NOVEL LARGER CRYOBALLOON FOR PULMONARY VEIN ISOLATION

### **JUNICHI OOKA**, ATSUSHI KOBORI, YASUHIRO SASAKI, YUTAKA FURUKAWA

#### Kobe City Medical Center General Hospital, Kobe, Japan

**Introduction:** A novel expandable cryoballoon catheter is expected to improve the effectiveness of pulmonary vein isolation (PVI) in patients with atrial fibrillation. We evaluated the efficacy and safety of a large cryoballoon compared with a standard‐size cryoballoon.

**Methods:** A total of 207 consecutive patients undergoing PVI with the POLARx ablation system were enrolled. The acute characteristics of PVI were assessed between patients using only 31mm mode (CB31; 83 patients) and 28mm cryoballoon (CB28, 124 patients).

**Results:** In baseline characteristics, each PV diameter of CB31 is larger than CB28. The success rates of PVI using the cryoballoon alone (CB31: 97.6% vs. CB28: 97.6%, ns) and the success rates of PVI by initial application were similar between groups. The time to PVI were equal between groups. The balloon temperature at PVI success and at the end of freezing were comparable between groups. There were no differences in complications and in the rate of discontinuation of freezing between groups. On the other hand, CB31 required considerably less additional freezing to the carina area of right superior PV (RSPV) and the antrum of left superior PV (LSPV) than the CB28 (CB31: 40.1%, 35.7% vs. CB28: 59.9%, 64.3%, p &lt;0.05 respectively). Consequently, the total number and freezing time of applications of CB31 were significantly less and shorter for LSPV and RSPV (CB31: 1.08, 1.55 vs. CB28: 1.74, 1.93, CB31: 243.8sec, 245.9sec vs. CB28 270.7sec, 280.6sec, p &lt;0.05 respectively).

**Conclusions:** The novel large cryoballoon ablation can provide effective treatment with less and shorter applications.

## CLINICAL OUTCOME AFTER CATHETER ABLATION FOR IMMEDIATE RECURRENCE OF ATRIAL FIBRILLATION IN ATRIAL FIBRILLATION PATIENTS

### **HIROTSUNA OSETO** ^1^, SEIGO YAMASHITA^2^, RYUTARO SAKURAI^2^, SATOKO SHIOMI^2^, HIDENORI SATO^2^, KENICHI TOKUTAKE^2^, MICHIFUMI TOKUDA^2^, MICHIHIRO YOSHIMURA^2^, TEIICHI YAMANE^2^

#### ^1^jikei University School of Medicine Hospital, Tokyo, Japan,^2^Jikei University School of Medicine, Division of Cardiology, Department of Internal Medicine, Tokyo, Japan

**Introduction:** Immediate recurrence of atrial fibrillation (IRAF) signifies the rapid return of AF following sinus rhythm (SR) restoration. It is uncertain whether elimination of IRAF relates to the clinical success, therefore we investigated the clinical outcome after catheter ablation (CA) for IRAF.

**Methods:** This study included 82 AF patients with IRAF (paroxysmal PAF (PAF) in 50 and persistent AF (PsAF) in 32). IRAF was defined as immediate AF recurrence within 90 seconds just after SR restoration. We tried to identify the origin of IRAF by using high‐density mapping catheters, and pulmonary vein (PV) isolation and/or non‐PV origin were targeted according to the IRAF origin.

**Results:** In total, IRAF from PV and non‐PV trigger was found in 22(27%) and 66(80%) patients. IRAF from multiple triggers appeared in 39(48%) patients. There was no significant difference in the occurrence of non‐PV IRAF between PAF and PsAF (80% vs. 78%, p=NS), while IRAF from PV trigger was more frequently observed in PsAF than PAF (42% vs. 16%, p=0.03). We eliminated IRAF by CA in 49(60%) patients, but IRAF remained in 38(46%) patients. Pilsicainide was used in 50(61%) patients, and IRAF disappeared at the end of the procedure in all patients. There was no significant difference in the distribution of non‐PV foci between PAF and PsAF. During 20±11months follow‐up, AF recurrence rate was significantly higher in PsAF compared to PAF (44% vs 18%, p=0.02), but the occurrence of non‐PV IRAF and elimination of IRAF by CA were not associated with clinical success in PAF and PsAF.

**Conclusions:** Distribution of IRAF was different according to AF type. Aggressive CA targeting IRAF triggers may not lead to better clinical outcome.

## REELED AND TWIDDLED; A CASE OF LEADLESS PACEMAKER IMPLANTATION AFTER TWICE DEVICE MANIPULATION RELATED COMPLICATIONS IN AN AGED PATIENT

### **YUJI OTA**, MASATERU KONDO, KAZUYA ARASAWA, SOICHIRO TANAKA, AZUSA OYAMA, YUTA KAGAYA, HIROKI SAITO, KENJIRO SATO, MASANORI KANAZAWA, MASANOBU MIURA, HIDEHARU ENDO, AKIHIRO NAKAMURA

#### Iwate Prefectural Central Hospital, Morioka, Iwate, Japan

**Introduction:** While uncommon (occurring in 0.1‐3% of pacemaker patients), lead dislodgement due to device manipulation is a severe complication that can have significant consequences. Proposed risk factors include female gender, older age, obesity, cognitive impairment, and inadequate generator pocket size. Based on the external force applied, the mechanism of lead dislodgement can be categorized into Reel, Ratchet, and Twiddler syndrome.

**Methods:** N/A

**Results:** A male patient over 90 years old with a history of dementia and hypertension presented with transient loss of consciousness and was transferred to our hospital. Upon evaluation, an electrocardiogram revealed bradycardic atrial fibrillation, prompting the implantation of a single‐chamber ventricular pacemaker. Nine months later, a routine follow‐up revealed ventricular lead dislodgement. A chest X‐ray demonstrated the clockwise rotation of the generator. Subsequently, surgical revision of the ventricular lead was performed. Seven months later, a second pacing failure occurred. A chest x‐ray revealed tangled lead and generator malrotation. Attributing these failures to generator manipulation, we decided to replace the transvenous pacemaker system with a leadless pacemaker. A Micra VR pacemaker was successfully implanted in the lower ventricular septum. Upon opening the delivery capsule, there was only a tiny space between the capsule and the generator, and the leads were found to be twisted. The generator was rotated nine times to achieve proper lead orientation. An active fixation screw was pre‐mounted on the lead, and the lead was extracted smoothly. Following the second revision, the patient remains in good health without further pacing failures.

**Conclusions:** The susceptibility to device manipulation‐related pacemaker failure risks is another compelling argument in favor of leadless pacemakers.

## A REVIEW OF ELECTROPHYSIOLOGICAL STUDY PROCEDURE USING A PORTABLE 3D MAPPING SYSTEM IN A TEACHING UNIVERSITY HOSPITAL: HOSPITAL UNIVERSITI SAINS MALAYSIA EXPERIENCE

### **MOHD KHAIRI OTHMAN**, ZURKURNAI YUSOF, SARAVANAN KRISHINAN, W YUS HANIFF W ISA

#### Hospital Universiti Sains Malaysia, Kota Bharu, Malaysia

**Introduction:** Electrophysiology study and radiofrequency ablation are common procedures for treating cardiac arrhythmias. Since 2021, our center had performed electrophysiology studies using a portable 3D mapping system, as our center does not have dedicated electrophysiology study facilities. The aim of this study is to describe procedures done using portable 3D mapping system in Hospital Universiti Sains Malaysia.

**Methods:** This is a cross‐sectional retrospective analysis of patients who underwent electrophysiology study and radiofrequency ablation from 2021 until 2023. Data collected include demography, symptoms, diagnosis, and procedure outcome among patients who underwent an electrophysiology study procedure and radiofrequency ablation. The data was tabled in SPSS version 27 and analyzed accordingly.

**Results:** 19 patients underwent an electrophysiology study in our center from 2021 until 2023. Mean age was 39.2±15.9 years and 52.6% were female. 15.8% had type 2 diabetes mellitus and hypertension respectively, 21.1% had dyslipidemia, and 15.8% had heart failure reduced ejection fraction. The initial symptom was varied; palpitation (52.6%) was the main symptom, followed by chest pain (15.8%) and shortness of breath (15.8%). Other symptoms include syncope (10.6%) and dizziness (5.3%). The main diagnosis were PVC (47.4%), WPW (21.1%), atrial flutter (15.8%), supraventricular tachycardia (10.5%), atrial fibrillation (5.35) and ventricular tachycardia (5.3%). Both EnSite Precision (52.6%) and Carto 3 (47.4%) 3D mapping system were used. All patients underwent radiofrequency ablation. The immediate success rate of radiofrequency ablation was 94.7%, and 21.1% had recurrence before discharge from the hospital.

**Conclusions:** Our experience of performing electrophysiology study and radiofrequency ablation with a portable 3D mapping system showed an excellent success rate. Apart from that, the study showed portable 3D mapping system can be utilised for various cardiac electrophysiology procedures in a limited resources centre safely and effectively.

## LEFT ATRIAL APPENDAGE THROMBUS IN A PATIENT WITH ATRIAL FIBRILLATION WITH LOW CHA2DS2VASC SCORE ‐ A CASE REPORT

### **MOHD KHAIRI OTHMAN** ^1^, EZELEA SANDOSAM^1^, SARAVANAN KRISHINAN^2^, ZURKURNAI YUSOF^1^, W YUS HANIFF W ISA^1^

#### ^1^Hospital Universiti Sains Malaysia, Kota Bharu, Malaysia,^2^Hospital Sultanah Bahiyah, Alor Setar, Malaysia

**Introduction:** Atrial fibrillation is a commonly encountered arrhythmia. The worldwide prevalence of AF is approximately 1%, however it is found in approximately 9% of individuals over the age of 75. Atrial fibrillation is the major cause of cardioembolic stroke. The most common site for this embolus is the left atrial appendage and has been seen in up to 90% of strokes caused by atrial fibrillation. CHA2DS2VASc score is the current scoring system that is used to risk stratify patients with atrial fibrillation on the risk of developing a stroke. Current guidelines recommend oral anticoagulation for patients who have a CHA2DS2VASc score of 2 or more.

**Methods:** N/A.

**Results:** We report a case of a 51‐year‐old male who was diagnosed with atrial fibrillation. He had a left atrial appendage thrombus on transesophageal echocardiography despite having a low CHA2DS2VASc score of 1 (Figure 1). Magnetic resonance imaging (MRI) of the brain also showed an old infarct of the right basal ganglia. He was started on oral anticoagulation and underwent radiofrequency ablation. Patients with low CHA2DS2VASc score that are considered to be at low risk can still develop left atrial appendage thrombus which could lead to a cardioembolic stroke.

**Conclusions:** This case suggests that combining clinical and echocardiographic parameters will help in predicting the risk of left atrial thrombus and hence the need for anticoagulation. Transesophageal echocardiography should be considered in AF patients with low CHA2DS2VASc score before radiofrequency ablation or cardioversion.

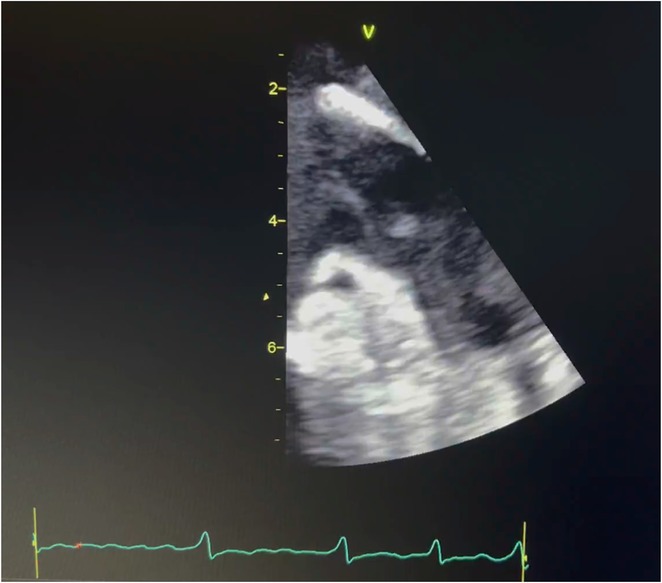

## ELECTROPHYSIOLOGICAL AND CLINICAL FEATURES OF CARDIAC AMYLOIDOSIS IDENTIFIED BY ATRIAL BIOPSY DURING ATRIAL FIBRILLATION ABLATION

### **TOYOKAZU OTSUBO** ^1^, TAKANORI YAMAGUCHI^2^, YUYA TAKAHASHI^2^, KANA NAKASHIMA^2^, KODAI SINZATO^2^, RYOSUKE OSAKO^2^, SHIGEKI SHICHIDA^2^, YUKI KAWANO^3^, AKIRA FUKUI^4^, NAOHIKO TAKAHASHI^4^, KOICHI NODE^2^

#### ^1^Saga‐Ken Medical Center Koseikan, Saga, Japan,^2^Saga University, Saga, Japan,^3^saiseikai Futsukaichi Hospital, Chikushino, Japan,^4^Oita University, Yufu, Japan

**Introduction:** Previous studies have shown that atrial bipolar voltage is lower in atrial fibrillation (AF) patients with cardiac amyloidosis (CA) than in non‐CA patients. The purpose of this study is to investigate the electrophysiological and clinical features that can differentiate CA from non‐CA in patients undergoing AF ablation.

**Methods:** Right atrial biopsy was performed in 577 patients who underwent AF ablation. Electroanatomic mapping was performed during high RA pacing using EnSite system (Abbott). Global LA voltage (V_GLA_) was evaluated with the mean of the highest voltage at a sampling density of 1 cm^2^ and patients were categorized into quartiles (Q1 to Q4). Fractionated electrograms (EGMs) were evaluated and were defined as ≥5 deflections in each EGM in 284 patients. The proportion of points with fractionated EGM was calculated as %fractionated EGM. Peak frequency was also evaluated in 52 patients. Amyloid deposition was assessed by Congo red staining and apple‐green birefringence under a polarizing microscope.

**Results:** Amyloid deposition was identified in 41 patients (7%), among which 24 patients were classified in the Q1 group. The prevalence decreased as the quartile moved up (p<.001). There were no significant differences in %Fractionated EGM and median peak frequency between the CA and non‐CA groups. There were significant differences in Troponin T level and left ventricular posterior wall thickness (PWD) between the CA and non‐CA groups (32.7±28.5 vs 28.3±38.7, p=0.009, 11.3±2.2 vs 9.6±1.4, p<.0001 respectively). ROC analyses were performed in each parameter (AUC=0.70, p=0.01, cutoff value=15.3pg/ml for Troponin T, AUC=0.72, p<.0001, cutoff value=12mm for PWD).

**Conclusions:** The prevalence of CA increased as left atrial voltage decreased. Troponin T levels and PWD may be useful in distinguishing CA, however, these markers have relatively poor discriminatory power individually.

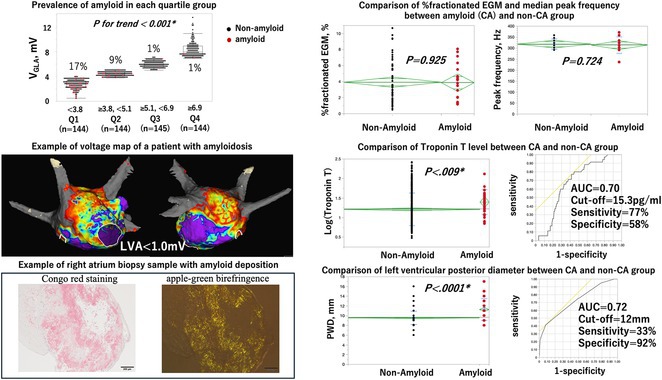

A CASE OF EPICARDIAL CATHETER ABLATION FOR BRUGADA SYNDROME WITH TRANSIENT HIGH‐DEGREE RIGHT BUNDLE BRANCH BLOCK MASKING THE TYPE 1 BRUGADA ECG PATTERN

**HIYORI OYAMA**, KENTARO MINAMI, IKUO ATAGI, KOHKI INOUE, KEITARO IIDA, IKUTA SAITO, YOSHIYUKI KITAGAWA, TOSHIAKI NAKAJIMA, SHIGERU TOYODA

Dokkyo Medical University, Tochigi, Japan

**Introduction:** The characteristic ECG of Brugada syndrome (BS) can be masked by complete right bundle‐branch block (CRBBB). We report a case of drug‐resistant Brugada syndrome treated by epicardial ablation, which was difficult to diagnose due to a complete right bundle branch block.

**Methods:** NA

**Results:** A 34‐year‐old man experienced repeated transient loss of consciousness during the night and was rushed to the hospital. Transient ventricular fibrillation (VF) frequently appeared at the emergency department. Echocardiography, coronary angiography, contrast‐enhanced MRI, and myocardial biopsy revealed no organic heart disease. VF was suppressed by continuous administration of isoproterenol. The 12‐lead ECG showed complete right bundle branch block, and the pilsicainide stress test did not show the typical Type 1 Brugadada‐type ECG. However, during hospitalization, complete right bundle branch block spontaneously resolved, followed by Coved‐type ECG waveform, leading to the clinical diagnosis of Brugada syndrome. Genetic testing revealed a mutation in SCN5A. Even after drug treatment, repetitive ICD Shock Therapy occurred due to VF, so epicardial ablation was performed. Extensive delayed potentials were observed in the anterior surface of the epicardial right ventricular outflow tract.

**Conclusions:** BS can coexist behind CRBBB, and CRBBB can completely mask BS. BS might be demonstrated by relief of CRBBB or by spontaneous or drug‐induced ST‐segment elevation.

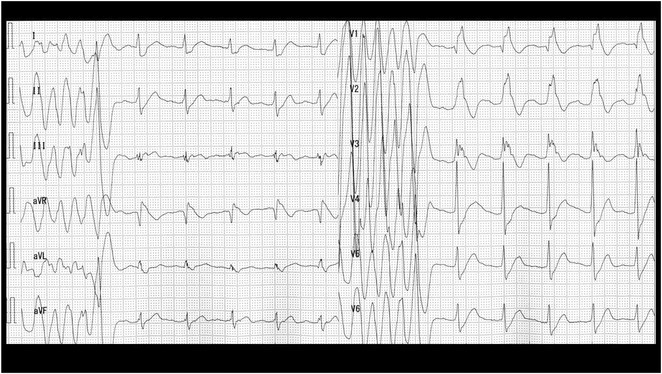

## SAFETY AND EFFICACY OF HELIX‐FIXACTION LEADLESS PACEMAKER: A COMPARISON WITH TINE BASED LEADLESS PACEMAKER

### **SANAMI OZAKI**, AKIRA KASAGAWA, IKUTARO NAKAJIMA, KENICHI SASAKI, TOMOO HARADA, J. YOSHIHIRO AKASHI

#### St. Marianna University, Kawasaki, Japan

**Introduction:** Leadless pacemaker have been developed to solve complications related to pockets and leads in patients with conventional pacemaker. Recently, helix‐fixaction leadless pacemaker (Aveir; Abbott) have become available, offering a choice between tine‐based (Micra; Medtronic) and heilx‐fixaction implantations. This study aimed to demonstrate the safety and efficacy of helix‐fixaction leadless pacemaker compared with tine‐based leadless pacemaker.

**Methods:** 31 patients with leadless pacemaker were enrolled in this study. 16 cases were selected for Aveir and 15 cases were selected for Micra (Micra AV: 13 caces, Micra VR: 2 cases). We compared pericardial effusion risk score, pacemaker implantation data (deployment times, absorbed dose, and complications), and pacemaker information at the implantation and a month after implantation for both devices.

**Results:** All the implantations were successful. Single attempt deployment was achieved in 87.5% and 66.7% of the patients in Aveir and Micra groups, respectively (p=0.002). Although the absorbed dose of Micra was higher than that of Aveir (238±176 vs 58.2±49.0 (mGy), p<0.001), there was no difference in the absorbed dose focusing single deployment groups (57.0±54.3 vs 97.7±69.0 (mGy), p=0.14). Patients of each device had a relatively high pericardial effusion score (Aveir: 2.18 (IQR 1‐3) vs Micra: 2.6 (IQR 1‐4), p=0.67); fortunately, no complications, including pericardial effusion, were observed. There was no change in the threshold for each device. (Aveir: 0.75±0.49/0.4 vs 0.5±0.61/0.4 (V/msec), p=0.84, Micra: 0.75±0.41/0.24 vs 0.50±0.69/0.24 (V/msec), p=0.19).

**Conclusions:** In this study, the deployment times of Aveir were less than those of Micra, which could affect the lower absorbed dose of Aveir compared with Micra. Additonaly, Aveir can be implanted as safely as Micra even in the high‐risk group of pericardial effusion.

## ACCESSORY PATHWAY ABLATION FROM A CHALLENGING UNUSUAL LOCATION

### JAYAPANDIAN PANDIAN

#### Meenakshi Mission Hospital and Research Centre, Madurai, India

**Introduction:** Posteroseptal accessory pathways location usually presented at Right / Left Septal, Diverticulum, Middle cardiac Vein. We report a case of Pathway location at SMALL CARDIAC VEIN (SCV ).

**Methods:** A 40 year old Female Reported with Frequent palpitation and documented Regular Narrow QRS Tachycardia with Long RP Interval suggestive of likely Orthodromic AVRT and ECG at rest revealed Preexcitation. During EP Study, Noticed Negative HV interval confirmed the presence of pre excitation, Easily Inducible narrow QRS tachycardia with activation pattern CS proximal to distal. RV Entrainment with PPI‐TCL < 60 ms and VAV response proved its ORT. Mapping performed during Sinus pre ex and during Tachycardia. During Sinus with pre Ex, Site 1‐ Right Postero septal, Site 2 ‐Middle cardiac Vein, Site 3‐Right Mid septal was mapped. Ventricular activity is Late compared to Onset of Pre Ex QRS at those areas. Levophase of coronary angio revealed a Large Middle cardiac vein and NO Diverticulum was present. Transeptal puncture performed and mapped left postero septal and MA, No good signals obtained. When Reviewing the CS levophase angiogram closely before giving up the case, Noticed another small Vein inside CS OS posteriorly located, 7F Irrigation Ablation catheter can’t enter into the SCV. Using 5F non Irrgn Ablation catheter, SCV accessed and got Earliest V during Sinus pre ex and Earliest A during Tachycardia. RF energy delivery at this site ( SCV) terminated the Tachycardia, and post termination the basal pre ex also vanished.

**Results:** Successful Accessory pathway ablation performed at a site which is unusual and challenging to reach.

**Conclusions:** SCV location for accessory pathway is very rare, but reported earlier. Here we report one of such case and insist the importance for looking at SCV also, along with usual Diverticulum or MCV, while mapping difficult postero septal accessory pathways.

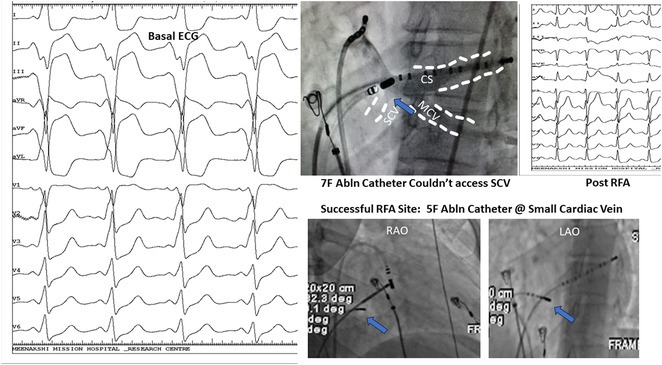

## OUTCOME OF PACEMAKER INDUCED CARDIOMYOPATHY UPGRADE TO CONDUCTION SYSTEM PACING A SERIAL CASE

### **RAHADIAN PANGESTU** ^1^, GIKY KARWIKY^2^, MOHAMMAD IQBAL^2^, CHAERUL ACHMAD^2^

#### ^1^Cardiology Resident, Department of Cardiology and Vascular Medicine, Universitas Padjadjaran, Bandung, Indonesia,^2^Electrophysiology and Arrhytmia Division, Department of Cardiology and Vascular Medicine, Universitas Padjadjaran, Bandung, Indonesia

**Introduction:** The incidence of Pacemaker Induced Cardiomyopathy (PICM) depends on the definition used, but in general it occurs in 10‐20% of patients after 3‐4 years of implantation. In recent years, the use of conduction system pacing (CSP), whether through His bundle pacing (HBP) or left bundle branch area pacing (LBBAP), has shown promising results in the management of PICM as a resynchronization therapy. However, there is still limited data presenting the success of conduction system pacing in the management of PICM.

**Methods:** N/A

**Results:** We report 8 cases of patients diagnosed with PICM who underwent conduction system pacing (CSP) upgrade, mostly male (62.5%). The average age of the patients was 61.3 years, with an age range from 35 to 78 years. Patients underwent CSP upgrade due to the diagnosis of PICM and experienced heart failure symptoms such as fatigue, weakness, or dyspnea. Before undergoing CSP upgrade, patients underwent echocardiography with an average LVEF of 39.5%, with the lowest LVEF reaching 18% and the highest LVEF reaching 46%. About 3 patients (37.5%) had coronary artery disease, underwent percutaneous coronary intervention and completely revascularization. Six patients (75%) underwent LBBAP and two patients implanted His bundle pacing. All patient underwent echocardiography evaluation with an average follow‐up of 6 months. Echocardiography showed an improvement in LVEF, with an average increase to 51.5%, representing an average increase in LVEF of 11.3% compared to before. Almost half patient had superresponder and normalization of LVEF. QRS duration before upgrade was 172 ms then become 131 ms after upgrade to conduction system pacing.

**Conclusions:** Conduction system pacing, whether with HBP or LBBAP, is a new physiological pacing modality that can be considered as one of the therapy options providing resynchronization in cases of PICM. HBP and LBBAP have been proven safe and effective with a physiological pacing strategy. Thus, HBP and LBBP can lead to improved LV function and reduced patient mortality. However, further research is still needed to determine whether CSP‐based CRT is non inferior than conventional CRT.

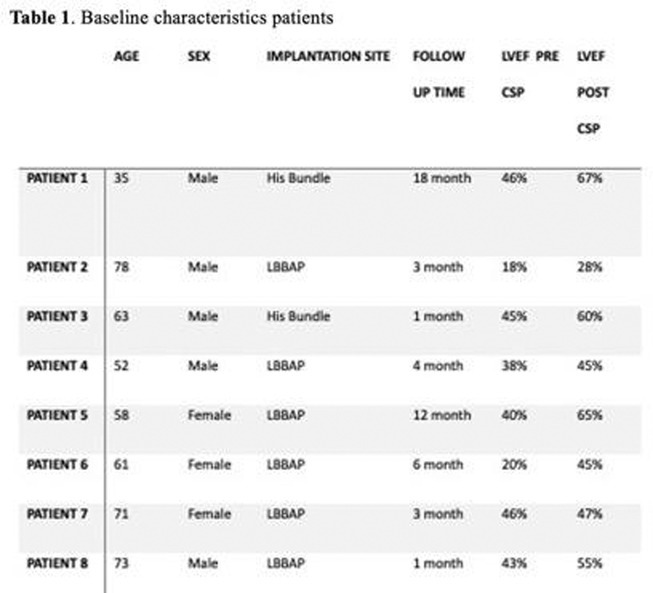

## A TALE OF TWO PATHWAYS ‐ ATYPICAL ATRIOVENTRICULAR NODAL REENTRANT TACHYCARDIA WITH MULTIPLE SLOW PATHWAYS AND ABSENT RETROGRADE FAST PATHWAY CONDUCTION

### **CHINMAY PARALE** ^1^, RAJESH DHOPESHWARKAR^1^, RAJA SELVARAJ^2^

#### ^1^Deenanath Mangeshkar Hospital and Research Centre, Pune, India,^2^Jawaharlal Institute of Postgraduate Medical Education and Research (JIPMER), Puducherry, India

**Introduction:** N/A

**Methods:** N/A

**Results:** A 67‐year‐old male was admitted with palpitations and breathlessness. His electrocardiogram showed a narrow complex, regular, long RP supraventricular tachycardia (SVT) which was adenosine responsive. In view of recurrent symptoms, invasive electrophysiologic evaluation was planned. Catheters were placed in the high right atrium (RA), right ventricular (RV) apex and coronary sinus (CS). Baseline rhythm was sinus with a cycle length (CL) of 890 ms, PR interval of 130 ms, QRS duration of 87 ms an AH interval of 42 ms and HV interval of 36 ms. Incremental and programmed RV pacing consistently induced an SVT with a cycle length of 330 ms, AH interval of 106 ms, HA interval of 212 ms, septal VA interval of 174 ms and central atrial activation pattern (Figure 1, Panel A). Ventricular overdrive pacing showed a VAV response. PPI‐TCL was 140 ms from the RV apex and 170 ms from the RV base. All the above findings were consistent with a diagnosis of fast‐slow AVNRT. Catheter induced premature atrial beats led to an abrupt change in VA interval to 100 ms. AH interval in this tachycardia was 222 ms and HA interval was 112 ms, consistent with a diagnosis of slow‐slow AVNRT (Figure 1, Panel B). Earliest atrial activation during both the tachycardias was found to be in the conventional slow pathway region. Ablation in this region led to junctional automaticity with absence of 1:1 VA conduction (Figure 2, Panel B). Ablation was continued in this region with careful monitoring of AV conduction. Post‐ablation, there was absence of VA conduction and no tachycardia was inducible. Atypical AVNRT with absent retrograde fast pathway conduction is a rare and distinct entity. In these cases, absence of 1:1 VA conduction during junctional automaticity during ablation does not represent fast pathway injury and ablation can be safely continued, provided antegrade AV conduction is monitored with atrial pacing.

**Conclusions:** N/A

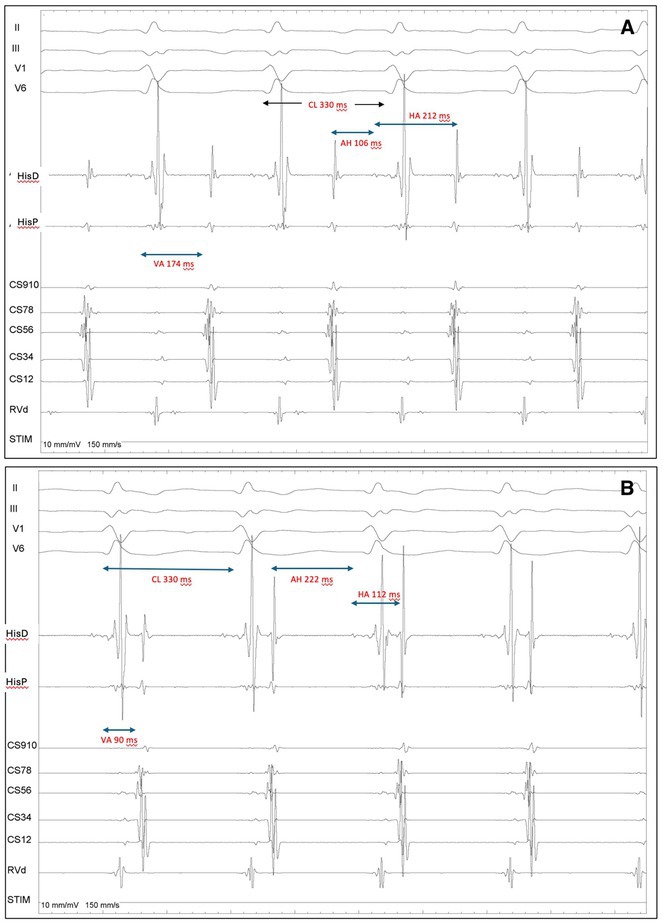

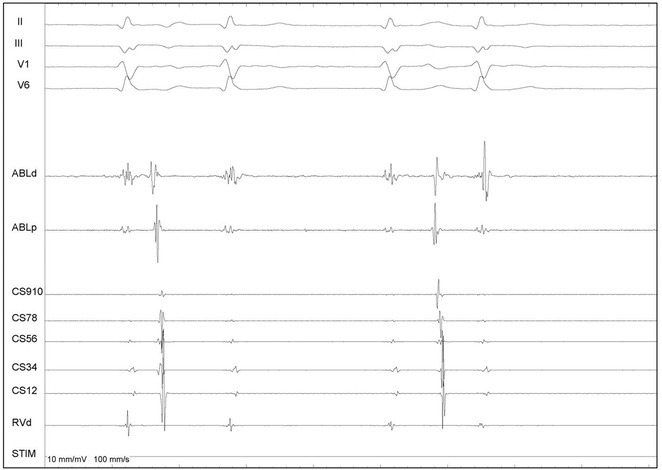

## DIAGNOSIS OF ISCHEMIC HEART DISEASE AND LEFT VENTRICULAR DYSFUNCTION IN PATIENTS WITH LEFT BUNDLE BRANCH BLOCK USING ARTIFICIAL INTELLIGENCE ENABLED ELECTROCARDIOGRAPHY

### **GURUNATH PARALE** ^1^, CHINMAY PARALE^1^, HRISHIKESH PARALE^2^

#### ^1^Spandan, Solapur, India,^2^Spandan, Solapur, India

**Introduction:** Left bundle branch block (LBBB) is a common condition with prevalence ranging from 0.06% to 0.1% in the general population. The prognosis in persons with LBBB is determined by the presence of underlying heart condition. Surface electrocardiography (ECG) by itself is unable to separate the patients of LBBB with structural heart disease from those without.

**Methods:** We aimed to develop and validate an artificial intelligence (AI) algorithm using a convolutional neural network (CNN) to identify the presence of ischemic heart disease and/or left ventricular dysfunction in patients with LBBB on the 12‐lead surface ECG.This was a retrospective single centre study done at a tertiary care centre. Electronic health record (EHR) data was screened and patients with LBBB on the surface ECG were identified. Out of these patients, patients who had undergone echocardiography and coronary angiography were used to develop the AI model. The deep learning framework was built using Keras 3 and Softmax, ReLu activation functions were used. The Loss function used was binary‐crossentropy and optimization was done using the Adam algorithm.

**Results:** Using electronic health record data, a total of 1,62,484 ECGs from 56,455 patients were screened and 317 eligible patients were identified. Out of these, 214 patients had structural and/or ischemic heart disease in the form of obstructive coronary artery disease (117 patients; 54.6%) and left ventricular dysfunction (97 patients; 45.4%). Data from 221 patients was used for training the CNN and 96 patients’ data was used for testing. The AI model thus generated was able to identify the presence of obstructive CAD and/or left ventricular dysfunction heart disease in patients with LBBB with the accuracy of 83%. ROC curve showed a good performance of the model with an area under the curve (AUC) of 0.828.

**Conclusions:** In patients with LBBB, AI enabled electrocardiography can identify patients with coronary artery disease and/or left ventricular dysfunction with reasonable accuracy.

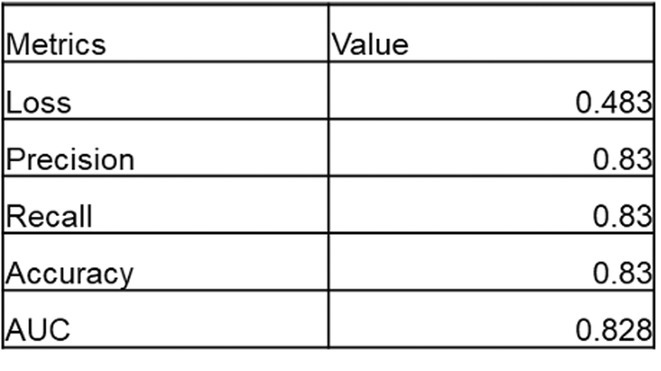

## CHANGE IN PACED QRS MORPHOLOGY IN A CASE OF EBSTEIN ANOMALY. WHAT IS THE MECHANISM?

### **NITIN KUMAR PARASHAR**, KRISHNA KUMAR MOHANAN NAIR, ABHILASH SP, NARAYANAN NAMBOODIRI

#### Sree Chitra Tirunal Institute for Medical Sciences and Technology, Trivandrum, India

**Introduction:** A 40‐year‐old female, known case of Ebstein Anomaly, presented with episodic palpitations. Electrocardiogram demonstrated manifest preexcitation. Patient was taken up for electrophysiologic study with an intent of radiofrequency (RF) ablation.

**Methods:** Atrial pacing showed incremental preexcitation. During atrial pacing at 280 ms cycle length, a transition with change in QRS morphology was noted. An increment in PR interval (from 82 ms to 146 ms) was also noted along with this transition. This increment in PR interval suggests a decremental antegrade conduction which can occur over either atrioventricular (AV) node or a decrementally conducting accessory pathway (AP).Transition from AP to AV node and mixed conduction over AV node and AP were excluded on the basis of increased pre‐excitation and negative HV interval. Another possibility which remains is the presence of a decrementally conducting AP. The anterograde decremental conduction is a characteristic feature of Mahaim's atriofascicular (AF) pathway.

**Results:** This patient was found to have two APs. First AP was mapped at 6 o’clock position of tricuspid annulus and was successfully ablated. However, a wide complex tachycardia (antidromic atrioventricular reciprocating tachycardia) with a late QRS transition pattern was still inducible. Second AP showed anterograde decremental conduction and absent retrograde conduction. Earliest ventricular activation was noted at right ventricular apex. This AP was mapped at 8 o’clock position of tricuspid annulus which is the most common location of Mahaim's AF pathway. RF ablation produced Mahaim's automaticity and led to successful ablation of Mahaim's pathway. Post RF ablation, there was no manifest preexcitation and pacing maneuvers did not induce any tachycardia.

**Conclusions:** Ebstein anomaly can be associated with multiple APs as seen in our case. Atrial pacing showed a transiton with change in QRS morphology with prologation in PR interval. This transition happened because of transfer of conduction from a typical accessory pathway to another atypical atriofascicular pathway.

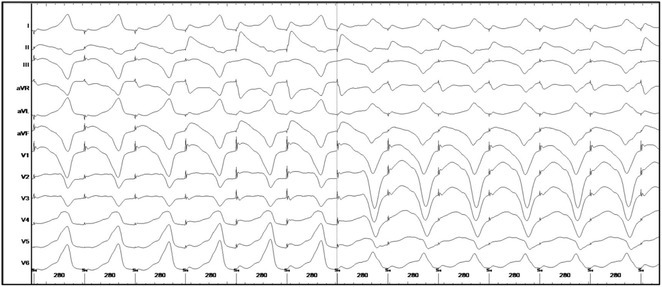

## DISTAL RIGHT CORONARY ARTERY OCCLUSION FOLLOWING RADIOFREQUENCY ABLATION OF PERMANENT JUNCTIONAL RECIPROCATING TACHYCARDIA

### **NITIN KUMAR PARASHAR**, ABHILASH SP, JYOTHI VIJAY, KRISHNA KUMAR MOHANAN NAIR, NARAYANAN NAMBOODIRI

#### Sree Chitra Tirunal Institute for Medical Sciences and Technology, Trivandrum, India

**Introduction:** A 45‐year‐old lady underwent an electrophysiological (EP) study for long RP tachycardia. Pacing maneuvers performed during tachycardia and at baseline suggested permanent junctional reciprocating tachycardia (PJRT) associated with a slow‐conducting concealed accessory pathway.

**Methods:** Activation mapping showed the earliest atrial activation at the inferior lip of coronary sinus‐os (CS‐os) (Panel A). Radiofrequency ablation (RFA) was performed at this site (Panel B) for 120 seconds in pulses of 20 seconds each with FlexAbilityTM bidirectional irrigated ablation catheter (Abbott healthcare, USA) at 35 W, 43 degrees Celsius and 17 ml/min flow of saline which rendered tachycardia non‐inducible and VA dissociation was documented following ablation.

**Results:** When EP catheters were being removed, ST‐segment changes were noticed in electrocardiogram, and simultaneously, the patient complained of a burning sensation in the chest. Diagnostic coronary angiogram revealed a total occlusion of distal right coronary artery (RCA) just proximal to crux (Panel C). Since the attempt to open the vessel with balloon angioplasty was unsuccessful, a drug‐eluting stent was deployed in RCA which reestablished the blood flow (Panel D).

**Conclusions:** The crux of RCA and the proximal part of PLB lie in close association with the inferior or ventricular end of the CS‐os. In a left dominant circulation, distal left circumflex and its division will be at this location.

We need to have a high index of suspicion for acute coronary occlusion during RFA as immediate management helps achieving favorable outcomes. If multiple ablations are planned near CS‐os, a concomitant coronary angiogram to delineate the anatomy would be desirable.

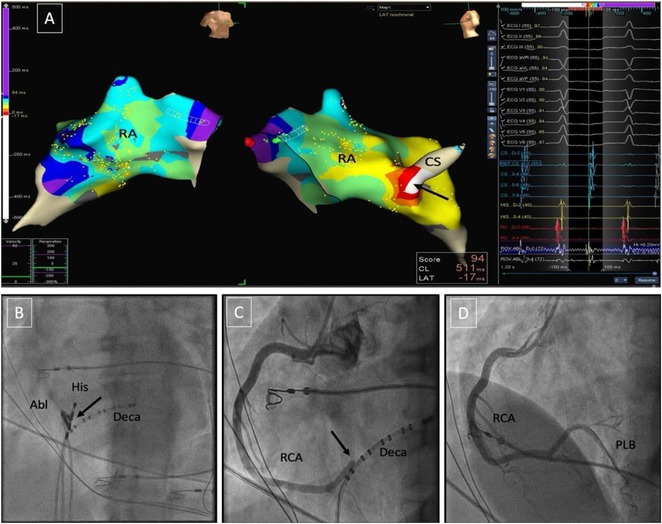

## POINT DENSITY EXCLUSION MAPPING FOR ABLATION OF PREMATURE VENTRICULAR COMPLEXES ORIGINATING FROM RIGHT VENTRICULAR PAPILLARY MUSCLE

### **NITIN KUMAR PARASHAR**, NARAYANAN NAMBOODIRI, KRISHNA KUMAR MOHANAN NAIR, ABHILASH SP

#### Sree Chitra Tirunal Institute for Medical Sciences and Technology, Trivandrum, India

**Introduction:** A 32‐year‐old female presented with episodic palpitations associated with dizziness. A 24‐hour Holter study demonstrated frequent premature ventricular complexes (PVC) (burden of 35%) with runs of non‐sustained ventricular tachycardia. QRS Morphology suggested right ventricle (RV) inflow focus for the PVCs. Cardiac MRI did not show any area of late gadolinium enhancement suggesting fibrosis.

**Methods:** RV geometry creation and activation mapping for PVCs was performed with Advisor high‐density grid catheter under Ensite Precision 3D electroanatomic mapping system. Initially PVCs were localized at the lateral wall of the RV (preceded the surface QRS by 30 ms), however, ablation at the earliest point was unsuccessful. Point density exclusion (PDx) mapping which helps to visualize endocavitary structures was performed.

**Results:** PDx mapping which takes inner points into consideration for delineating endocavitary structures found the origin of PVCs from the right ventricular papillary muscle (preceded the surface QRS by 39 ms). Ablation was performed near the tip of papillary muscle which showed acceleration of PVCs followed by complete subsidence of PVCs.

**Conclusions:** Sometimes activation mapping can misguide us if we are not able to reach the earliest point or the exit site. PDx mapping can be helpful in some of these cases when the origin of PVC is from endocavitary structures such as papillary muscles.

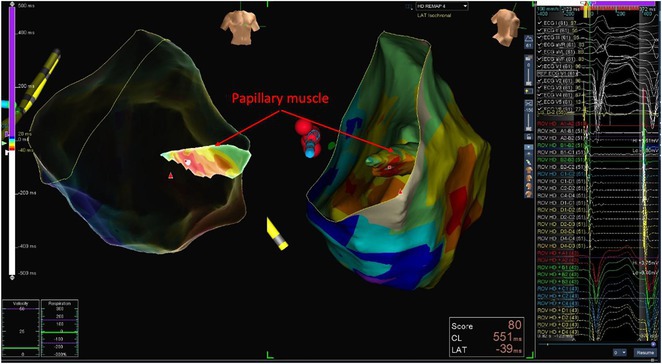

## WOLF LIVING WITH A SHEEP

### **NITIN KUMAR PARASHAR**, NITISH NAIK, GAUTAM SHARMA, SIDDHARTHAN DEEPTI

#### All India Institute of Medical Sciences, New Delhi, NEW DELHI, India

**Introduction:** A 43‐year‐old gentleman presented a history of episodic palpitations. Electrocardiogram showed manifest preexcitation with positive delta wave in V1 suggesting a left sided accessory pathway (AP). He was taken up for electrophysiologic study with the intent of radiofrequency ablation (RFA).

**Methods:** Orthrodomic AVRT was induced on atrial extrastimulus pacing protocol. AP was mapped at 3 O’ clock of mitral annulus and was successfully ablated in sinus rhythm. Local AV separation was documented on RFA.

**Results:** Post RFA, minimal preexcitation with positive delta wave in V1 was persistent and baseline HV interval was 26 ms which further confirmed presence of AP. Possibility of the same pathway with persistent partial anterograde conduction vs another AP was considered. Pacing maneuvers were repeated. This time incremental preexcitation was not present and the HV interval remained constant on atrial pacing. Junctional beats were found to be preexcited. Adenosine produced AV block and blocked P waves did not conduct through accessory pathway. No tachycardia was inducible on intensive pacing protocol. Based on these findings, the diagnosis of left‐sided fasciculoventricular (FV) pathway was confirmed.

**Conclusions:** In this case, after ablation of the typical accessory pathway (Wolf), persistent preexcitation was found to be related to the FV pathway (sheep). FV pathways are mostly right sided but rarely they can be on the left side. Pacing maneuvers should be performed to confirm this diagnosis to prevent inadvertent ablation attempts. Ablation attempts for the FV pathway will increase the risk of AV block without any clinical benefit to the patient.

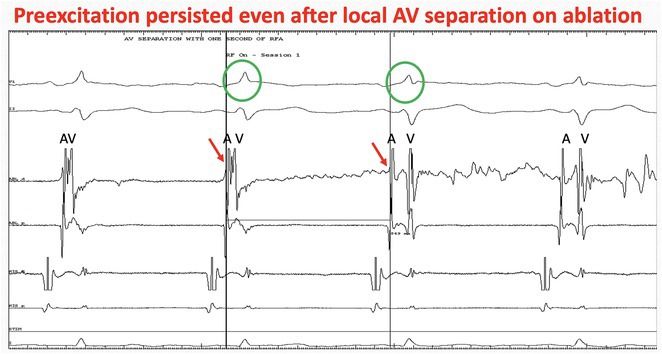

## THERAPEUTIC EFFECTS OF SGLT2 INHIBITOR ON ATRIAL FIBRILLATION

### **HYEWON PARK** ^1^, BOKYEONG PARK^1^, HYELIM PARK^2^, JUNBEOM PARK^1^

#### ^1^Ewha Womans University, Seoul, Korea, Republic of,^2^Inha University School of Medicine, Incheon, Korea, Republic of

**Introduction:** Atrial fibrillation (AF) is the most common persistent arrhythmia in clinical practice. AF is highly correlated with multiple risk factors including diabetes, obesity and heart failure. Sodium‐glucose co‐transporter 2 inhibitor (SGLT2i) is a new type of hypoglycemic drug, which can regulate blood glucose level safely and effectively. Recently, it was reported that SGLT2 inhibitors reduced the incidence of Cardiovascular disease. The purpose of this study is to find the relationship between AF and SGLT2 inhibitor and investigate its potential as a therapeutic target.

**Methods:** The experiment used HL‐1 Cell, and each cells were classified contol, AF induced group by treated angiotensin II (Ang II), and Ang II + SGLT2i group. The animal experiment compared four groups: control, AF model injected with acetylcholine + calcium chloride mixture, and pretreatment and post‐treatment of SGLT2i in the AF model. The expression patterns of fibrosis marker, calcium‐handling proteins and Connexin 40 and 43 were confirmed by quantitative real‐time PCR, respectively.

**Results:** In AF+SGLT2i goup, the fibrosis marker (Col Iα, Col IIIα, TGF‐ β1) decreased, and CaMKII and RyR2 decreased whereas SERCA2a increased. In calcium Release image, it was observed that the AF+SGLT2i group had a low regular intensity. Connexin mRNA expression went down in the Ang II group compared to the control group and recovered in the AF+SGLT2i group.

**Conclusions:** The results of this study confirmed the effectiveness of SGLT2i and its potential as a treatment in fibrosis‐induced atrial fibrillation.

## THE EFFICACY OF LEFT ATRIAL APPENDAGE OCCLUSION DURING TOTAL THORACOSCOPIC ABLATION OF ATRIAL FIBRILLATION ON RHYTHM OUTCOME IN PATIENTS WITH HEART FAILURE

### **SEONGJIN PARK**, MYOUNG JUNG KIM, HYO JIN LEE, JU YOUN KIM, JUWON KIM, DONG SEOP JEONG, SEUNG‐JUNG PARK, KYOUNG‐MIN PARK, YOUNG KEUN ON

#### Samsung Medical Center, Seoul, Korea, Republic of

**Introduction:** The left atrial appendage (LAA) represents the main source of thrombus formation in patients with atrial fibrillation (AF). Also, LAA plays an important role in LA reservoir function especially in patients with heart failure (HF). Limited data are available regarding the efficacy for rhythm outcome of surgical thoracoscopic LAA occlusion during total thoracoscopic ablation (TTA) of AF. We evaluated the rhythm outcome of surgical thoracoscopic LAA occlusion during TTA of AF among the patients who had AF combined with HF.

**Methods:** Patients who underwent TTA for AF combined with HF from February 2012 to April 2023, were included. We evaluated the development of AF recurrence in these patients. Furthermore, the electrophysiological characteristics of patients who underwent redo catheter ablation were analyzed.

**Results:** A total of 129 patients (mean age, 55.6 ± 9.6 years; 118 [91.5%] males) who received thoracoscopic ablation of AF and history of HF was included. Among the patients, 14 (10.9%) patients did not receive LAA occlusion according to the physician's discretion. The recurrence rate of atrial tachyarrhythmia showed tendency to increase in no‐LAA occlusion group (HR 1.26, 95% CI 0.65‐2.47). Among the AF recurrence patients, 36 underwent electrophysiology study and endocardial catheter ablation. The reconnection rate of left pulmonary vein (LPV) showed significantly higher in no‐LAA occlusion group (OR 27.0, 95% CI 2.38‐306.66).

**Conclusions:** Thoracoscopic LAA occlusion during total thoracoscopic ablation of AF was effective to maintain durable LPV isolation in patients with HF.

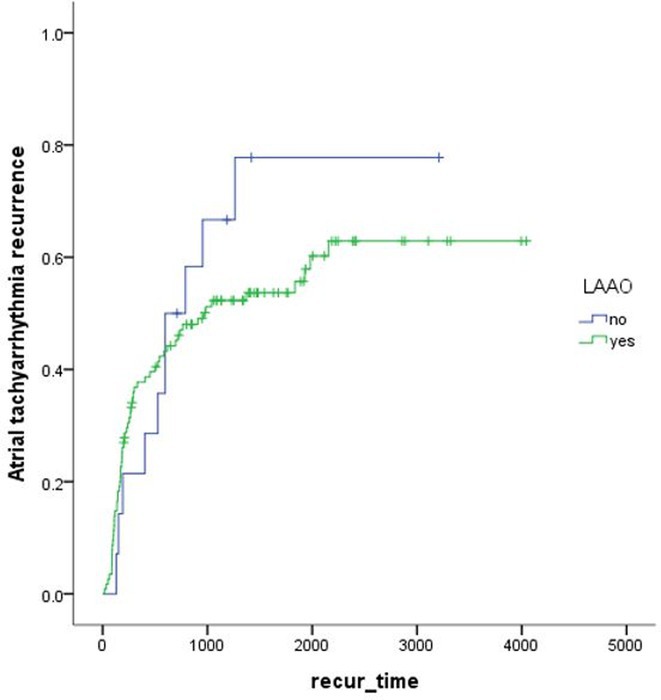

## ASSOCIATIONS OF ADDING SALT TO FOODS WITH INCIDENT ATRIAL FIBRILLATION IN THE UK BIOBANK STUDY

### **YOON JUNG PARK** ^1^, MYUNG HWAN BAE^2^, PIL‐SUNG YANG^3^, EUNSUN JANG^4^, HEE TAE YU^4^, TAE‐HOON KIM^4^, HUI‐NAM PAK^4^, MOON‐HYOUNG LEE^4^, BOYOUNG JOUNG^4^

#### ^1^Kyungpook National University Chilgok Hospital, Daegu, Korea, Republic of,^2^Kyungpook National University Hospital, Daegu, Korea, Republic of,^3^CHA Bundang Medical Center, Seongnam, Korea, Republic of,^4^Severance Cardiovascular Hospital, Seoul, Korea, Republic of

**Introduction:** High sodium and low potassium intake were associated with increased risk of hypertension and cardiovascular disease. We aimed to investigate the relationship between frequency of adding salt to food and incident atrial fibrillation (AF) and effect of potassium intake on changes in new‐onset AF risk according to the frequency of salt addition.

**Methods:** The UK Biobank cohort study enrolled more than 500,000 participants aged 40 to 70 years across the United Kingdom from 2006 to 2010. This study included 416,868 participants who completed the questionnaire of the frequency of adding salt to foods at baseline.

**Results:** During follow‐up, 19164 (4.6%) developed AF. The incidence of new‐onset AF was increased according to the frequency of salt addition (never/rarely 3.83; always 4.72 per 1,000 person‐years). Compared with the group of adding salt never/rarely, those adding salt always was significantly associated with higher risk of incident AF after multivariable adjustment (Hazard ratio [HR] 1.15, 95% confidence interval [CI] 1.06‐1.24), and even additional adjustment of dietary and total energy intake (HR 1.37, 95% CI 1.08‐1.73). In subgroup analysis, risk of incident AF according to frequency of salt addition significantly increased in low urine potassium level compared to high urine potassium level (p for interaction=0.046). In AF patients, incidence of all‐cause mortality increased dramatically with increasing frequency of salt addition.

**Conclusions:** Our study indicates that higher frequency of adding salt to foods was associated with higher risk of incident AF, even after the adjustment of dietary and total energy intake. Moreover, in the high urine potassium group, the effect of high sodium intake on the risk of incident AF was observed to be attenuated.

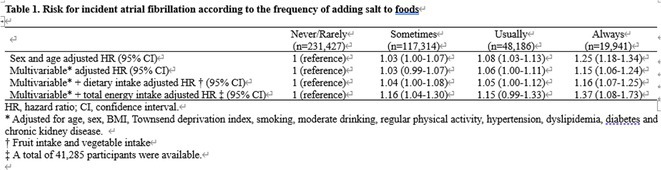

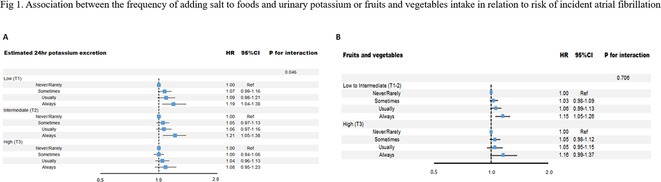

## ARTIFICIAL INTELLIGENCE‐BASEDELECTROCARDIOGRAM PREDICTION FOR NEW ONSET ATRIAL FIBRILLATION

### YOUNG JUN PARK

#### Wonju Severance Christian Hospital, Wonju, Korea, Republic of

**Introduction:** Atrial fibrillation (AF) is the most common sustained arrhythmia and is associated with poor clinical outcomes, including stroke, acute coronary events, and heart failure. Recent studies have shown that early rhythm treatment of new‐onset atrial fibrillation improves the patient's prognosis. However, atrial fibrillation is often asymptomatic, and it is difficult to determine its duration accurately. Recently, AI‐based ECG has been studied for various cardiovascular diseases.

We sought to develop and validate a predictive model of the ECG for new onset atrial fibrillation.

**Methods:** All patients aged 18 years older with at least one ECG were included in the study. Only patients with sinus rhythm with ECG prior to atrial fibrillation with ECG were selected. An ECG within 1 year from AF was first documented in ECG was defined as new onset AF. After dividing our datasets into training (and test sets, we developed an end‐to‐end deep neural network to predict for the duration of AF. Performance evaluation was conducted using various metrics, including AUROC, AUPRC, sensitivity, specificity, F1 score.

**Results:** The dataset consisted of 83,525 ECGs from 16,193 patients from two hospitals. The AUROC for discriminating old AF and new‐onset AF is 0.8186 (0.8181 ‐ 0.8190) on internal validation set and 0.7967 (0.7966 ‐ 0.7969) on external validation set. Sensitivity, Specificity, and F1 score are 0.7126((0.7118‐0.7134), 0.7697 (0.7693‐0.7701) and 0.5751 (0.5745‐0.5757) on internal validation set and 0.7309 (0.7307‐0.7311), 0.7225 (0.7224‐0.7227) and 0.6354 (0.6352‐0.6356)on external validation set.

**Conclusions:** Our deep learning model can be used to predict for new onset atrial fibrillation. Additional studies are ongoing to understand the relative importance of ECG features.

## ACUTE MARIJUANA INTOXICATION INDUCED BRUGADA ELECTROCARDIOGRAM PATTERN AND VENTRICULAR FIBRILLATION

### **ANAND YADAV PASULA** ^1^, HYNDAVI BELLAGANTI^2^, ULHAS M PANDURANGI^3^

#### ^1^KIMS Hospital, ongole, India,^2^KIMS Hospital, HYDERABAD, India,^3^Arrhythmia‐Heart Failure‐Academy, The Madras Medical Mission Hospital, Chennai, Tamil nadu, India

**Introduction:** Marijuana is one of the most commonly used illicit substances in the world. It interacts with endocannabinoid system in the human body. Exposure to cannabis produces different cardiovascular effects in dose‐dependent manner.

**Methods:** N/A

**Results:** A 21 year old woman with no significant medical history presented to the hospital with abdominal pain, vomiting, and syncopal episode. She gave a history of smoking of cannabis and alcohol consumption. Electrocardiogram showed sinus tachycardia coved ST elevation pattern in lead V1, V2 consistent with a Brugada Type I pattern. Patient 2D echocardiogram showed normal heart. Urine toxicological analysis revealed elevated tetra‐hydrocannbinol 70ng/ml. Blood investigations showed normal complete blood count, troponin levels, and electrolytes. In hospital patient developed ventricular fibrillation and collapsed. Brugada electrocardiogram pattern can be caused by abusive substances and various medications. In brugada syndrome patients the ST segment and T wave inversions were due to heterogeneity of depolarization and repolarization between epicardial and endocardial cells. Tetrahydrocannabinol (THC) is one of the important active chemical ingredients of marijuana. It acts on cannabinoid receptors. Previous case reports mentioned that the marijuana induced brugada ECG pattern as transient phenomenon and associated with benign outcome. In our patient consumption of heavier doses of marijuana and alcohol might have caused toxic levels of THC. Arrhythmias are triggered by excessive catecholamine release mediated by THC and that results in refractory ventricular arrhythmia and death.

**Conclusions:** Our case raises suspicion that the marijuana‐induced type 1 Brugada pattern is not always associated with benign outcomes and has the potential to provoke malignant arrhythmias in heavily intoxicated patients.

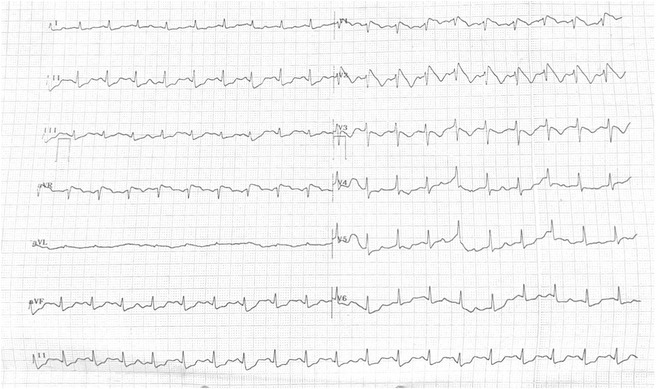

## DUAL LOOP RIGHT ATRIAL FLUTTER‐ IMPORTANCE OF REVISITING THE SURGICAL NOTES

### **SANJAI PATTU VALAPPIL**, SUBRAMANYAN K, PRAMOD KUMAR JAISWAL, PRASAD GN, JAYANTHI K, SRINATH TS, SHARAN SHREEDHAR

#### SIMS, Chennai, Chennai, India

**Introduction:** The surgical site incision has a profound implication on the ablation strategies. A 58 year old gentleman with history of coronary artery bypass graft and mitral valve repair presented with drug resistant atrial flutter and tachycardiomyopathy 3 months after the surgery. He was taken up for electrophysiology study and radio frequency ablation (EPS /RFA)

**Methods:** N/A

**Results:** The tachycardia cycle length (TCL) was 255 ms with a distal to proximal activation of the coronary sinus (CS). However, the left atrium (LA) appeared to be outside the circuit by entrainment mapping. Voltage map of the right atrium (RA) showed areas of low voltage signals in the anterolateral right atrium parallel to the right atrioventricular (AV) groove, corresponding to the surgical incision for the right superior transeptal access. The propagation map showed the possibility of 2 loops in the RA. Entrainment from the lower anterior RA scar (probable isthmus of the lower loop) showed concealed fusion with short post pacing interval (PPI) ‐ TCL (252‐252). RFA at this site resulted in prolongation of the TCL to 323 ms without change in the activation pattern. The ablation sets were anchored to the IVC. Entrainment repeated from the lower anterior RA after ablation now showed manifest entrainment with long PPI‐ TCL.The flutter changed to the dominant loop with critical Isthmus at the upper anterolateral RA. Entrainment from the upper common isthmus showed concealed fusion with PPI‐TCL (324‐324). RFA done along the fragmented signals in the upper anterolateral RA scar resulted in termination of the tachycardia.Patient developed prolonged sinus pause and slow junctional rhythm. The ablation line was anchored to the lower RA ablation sets. No flutters were reinducible and patient subsequently required permanent pacemaker for symptomatic sinus bradycardia.

**Conclusions:** The right superior transeptal approach for mitral valve surgeries often result in atrial flutters with the critical isthmus at the large incision in the anterolateral RA and coexistence of significant sinus bradycardia. The dual loop RA flutter was demonstrated by careful entrainment mapping.

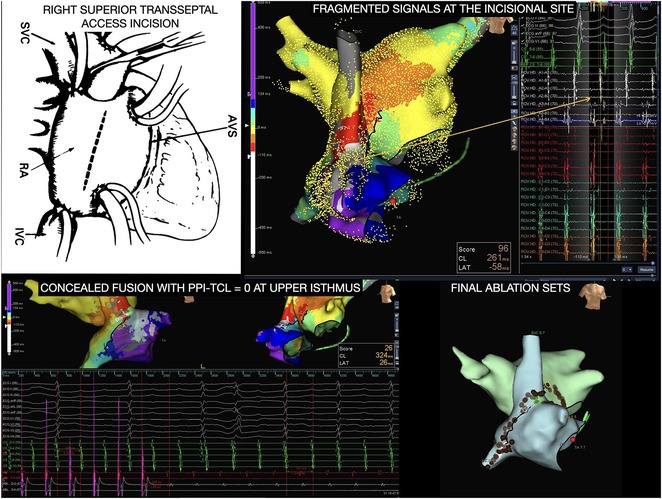

## ISOCHRONAL LATE ACTIVATION MAP (ILAM) GUIDED EPICARDIAL VT ABLATION IN AN ACUTE CORONARY SYNDROME (ACS) VT STORM

### **SANJAI PATTU VALAPPIL**, SUBRAMANYAN KRISHNASWAMY, PRAMOD KUMAR JAISWAL, PRASAD GOLLA, JAYANTHI K, SRINATH TS, SHARAN SREEDHARAN, RAJKAMAL VISHNU

#### SIMS, Chennai, Chennai, India

**Introduction:** 65 yr old gentleman with recent coronary angioplasty and moderate left ventricular (LV) systolic dysfunction was referred for management of ventricular tachycardia (VT) storm.

**Methods:** N/A

**Results:** Patient was intubated, ventilated and was taken up emergency radio frequency ablation (RFA) under 3D Ensite Navx system. VT had RBBB morphology, right axis, maximum deflection index (MDI>0.55), Q waves in lead 1 and aVL. The bipolar voltage map (0.2 ‐1.5mv) revealed no scar in the LV endocardium, whereas the unipolar endocardial voltage map (4.0‐7.5 mv) revealed a posterolateral epicardial scar. The LV activation map during VT, showed a wide area of early activation in the posterolateral LV. However the LV endocardial voltage map showed no mid diastolic potentials (MDP) in this site. Hence the isthmus of the VT was postulated to be epicardial. As patient was on dual antiplatelets, the epicardium was accessed by a subxiphoid surgical epicardial window. The LV epicardial surface was mapped with the HD grid catheter. The epicardial ILAM showed deceleration zone around the posterobasal LV. The HD grid was placed at the putative epicardial isthmus and VT was induced, which demonstrated consistent MDP at these sites. RF Ablation at this region at 40 W, 43 degrees, 17 ml/ min resulted in termination of VT within 4 seconds. The ablation sets were given upto the inferior border zone of the epicardial scar. VT reinduction resulted in another VT with RBBB morphology and leftward axis, suggestive of a superior epicardial exit. RF ablation were now given targeting the late potentials (LP) at the lateral epicardial scar border zone and anchored to the anterolateral mitral annulus. Patient was subjected to aggressive reinduction and no VTs were inducible. Patient underwent successful implantable cardioverter defibrillator (ICD) implantation predischarge. PET CT done after 4 months was suggestive of infiltrative cardiomyopathy (IFCM). Patient had no recurrence of VT.

**Conclusions:** The case demonstrates the feasibility of ILAM guided epicardial VT ablation using a limited subxiphoid epicardial window in a patient with recent PTCA and VT storm

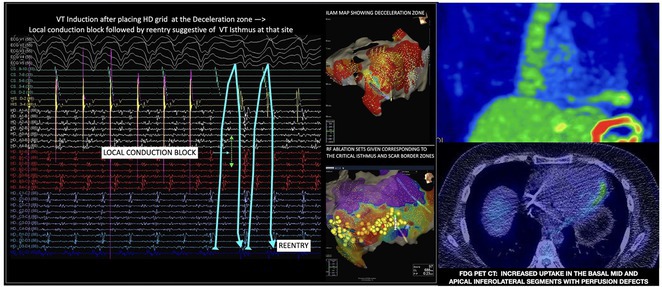

## A CASE OF ILVAH (ISOLATED LEFT VENTRICULAR APICAL HYPOPLASIA) WITH NON‐ISCHEMIC DILATED CARDIOMYOPATHY IN A MIDDLE AGE GENTLEMEN ‐ A FOUR‐LEAF CLOVER

### **KUNARAJ PERUMALU** ^1^, CHIN WEE LEE^1^, SARAVANAN KRISHINAN^2^, BARAKATH BADUSHA ABDUL KAREEM^3^, MOHAMED JAHANGIR ABDUL WAHAB^1^

#### ^1^Hospital Pulau Pinang, Penang, Malaysia,^2^Hospital Sultanah Bahiyah Alor Setar, Kedah, Malaysia,^3^Hospital Lam Wah Ee, Penang, Malaysia

**Introduction:** Isolated left ventricular apical hypoplasia is extremely rare and unclassified cardiomyopathy. ILVAH also known as truncated left ventricle is characterized by truncated, spherical, non‐apex forming LV with systolic dysfunction. The true apex is occupied by right ventricle which wraps around distal LV.

**Methods:** N/A

**Results:** 54‐year‐old gentlemen whom presented with reduced effort tolerance since age of 13 years old. Patient was admitted for supraventricular arrhythmia year 2003 and was treated with verapamil 40mg TDS. He was diagnosed with dilated cardiomyopathy concurrently with a LVEF of 40% and global hypokinesia with aneurysmal septum with akinetic anterior wall. He also had electrophysiology study and radiofrequency catheter ablation done year 2015 for concealed orthodromic AVRT and a diagnosis of left posteroseptal accessory pathway was made. Despite under the follow up of heart failure clinic with optimization of medications he still had recurrent heart failure symptoms. A TTE and cardiac MRI were done in January 2024 and confirmed the diagnosis of isolated left ventricular apical hypoplasia (ILVAH). Coronary angiogram showed minor coronary artery disease. He had a CRT‐D implanted in January 2024 for NYHA class II non‐ischemic dilated cardiomyopathy with LBBB morphology electrocardiogram despite optimal medical therapy. He showed good clinical response post CRT‐D implantation to NYHA class I. His device programming and heart failure medications was further optimized on his latest follow‐up.

**Conclusions:** 1. Importance of clinical presentations and non‐invasive tools in clinching the diagnosis for an extremely rare congenital cardiac abnormality 2. The role of EPS for prophylactic AF ablation and PVI in patients with ILVAH 3. What are the important morphologic criteria for diagnosis of ILVAH? a) A truncated and spherical LV with abnormal diastolic or systolic function b) Invagination of fatty material into the myocardium of the defective LV apex c) Abnormal origin of a complex papillary network from the flattened apical LV d) An elongated RV wrapping around the deficient apex

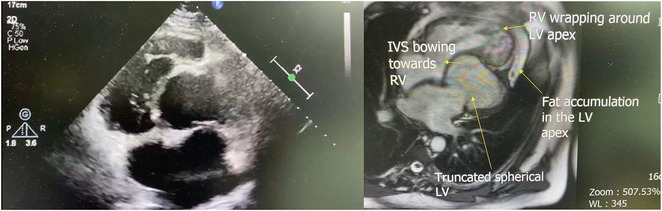

## A CASE OF LEFT SUPERIOR PULMONARY VEIN ATRIAL TACHYCARDIA IN A YOUNG LADY WITH PRIMARY MEDIASTINAL B‐CELL LYMPHOMA

### **KUNARAJ PERUMALU** ^1^, SARAVANAN KRISHINAN^2^, MOHAMED JAHANGIR ABDUL WAHAB^1^

#### ^1^Hospital Pulau Pinang, Penang, Malaysia,^2^Hospital Sultanah Bahiyah Alor Setar, Kedah, Malaysia

**Introduction:** Focal atrial tachycardia accounts up to 15% of patients presenting with supraventricular tachycardia. Common anatomic locations are in the right atrium, especially the crista terminalis, tricuspid annulus, coronary sinus ostium, right atrial appendage and peri‐nodal region. The most frequent site of origin in the left atrium is at the pulmonary vein ostium.

**Methods:** N/A

**Results:** A 32‐year‐old lady with primary mediastinal B‐cell lymphoma in remission presented with palpitation for 3 days. She was diagnosed with primary mediastinal B‐cell lymphoma in November 2020. She has completed 6 cycles of chemotherapy (R‐DAEPOCH) from (19/11/2020 ‐ 22/03/2021). She has also completed 15 cycles of radiotherapy from (09/06/2021 ‐ 30/06/2021). Her PET scan done on 23/09/2021 showed complete metabolic response to treatment. Her current presentation was intermittent palpitation associated with chest discomfort during the palpitation. The palpitation is of sudden onset with no specific triggers and lasted 5 minutes with self‐termination. The frequency of palpitation is around 5‐6 times per day. She had one previous history of palpitation associated with giddiness in October 2023. Her echocardiogram findings showed LVEF 61% with all chambers normal size and no RWMA. Her heart rate was at 180/min on current presentation and she was treated with IV Esmolol 5mg x 2 at 15 minutes apart and given loading dose of IVI Amiodarone 300mg for atrial fibrillation. Her ECG showed positive P wave in Lead V1, II, III, aVF, with negative P wave in Lead I, aVL. The P waves in Lead II was bifid in pattern. She is planned for electrophysiology study and left superior pulmonary vein isolation under 3D electroanatomical mapping. She was kept on bisoprolol 2.5mg OD.

**Conclusions:** The important ECG features to recognize in a patient presenting with narrow complex tachycardia and pattern recognition for SVT's

The role of all 4 pulmonary vein isolation vs left superior pulmonary vein isolation alone in left superior pulmonary vein tachycardia

The role of pulse field ablation in left superior pulmonary vein atrial tachycardia vs radiofrequency catheter ablation.

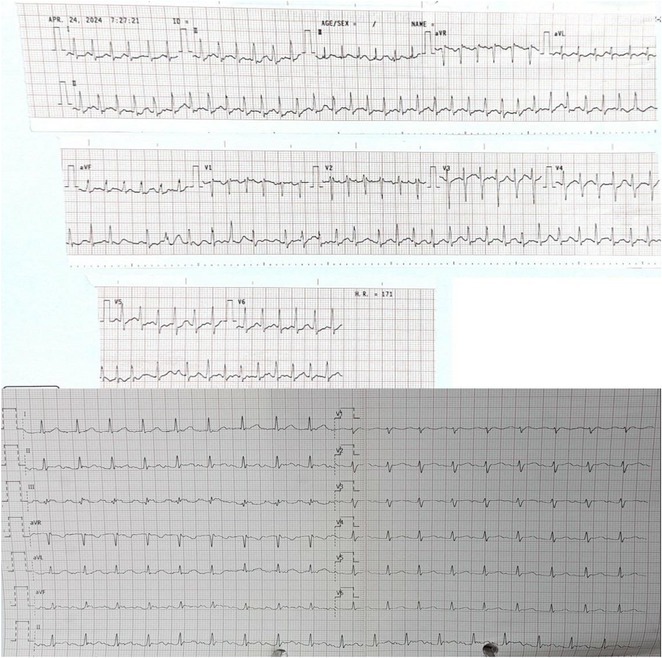

## A CHALLENGING CASE OF FASCICULAR VENTRICULAR TACHYCARDIA IN A YOUNG FEMALE PATIENT ‐ A CLASSICAL BELHASSEN TYPE VT

### **KUNARAJ PERUMALU** ^1^, SARAVANAN KRISHINAN^2^, CHIN WEE LEE^1^, MOHAMED JAHANGIR ABDUL WAHAB^1^

#### ^1^Hospital Pulau Pinang, Penang, Malaysia,^2^Hospital Sultanah Bahiyah Alor Setar, Kedah, Malaysia

**Introduction:** Idiopathic posterior fascicular VT (P‐IFLVT) is a verapamil sensitive tachycardia originating from left ventricle. The mechanism is reentry and occurs in structurally normal heart individuals. It's predominantly present in young adults aged 15 to 40 years old and mainly affects males in 60% to 80%. ECG features include right bundle branch block (RBBB) morphology and left axis indicating circuit exit is located at infero‐posterior septum.

**Methods:** N/A

**Results:** A 23‐year‐old lady presented with recurrent palpitations with a heart rate of 160 beats per minute. Her 12 lead ECG showed presence of narrow complex tachycardia with right bundle branch block morphology with capture beats and presence of VA dissociation. Despite being given adenosine and digoxin in emergency department her symptoms and tachycardia did not resolute. She was eventually given intravenous verapamil 5mg and it reverted to sinus rhythm. She was started on oral verapamil 40mg TDS subsequently. Her echocardiogram showed all chambers normal size, LVEF ‐ 67%, all valves normal morphology and a probable scar lesion at mid septal, TAPSE ‐ 2.0cm with RVS’ ‐ 0.12m/s. Her MSCT coronaries was normal with no coronary anomalies or coronary artery disease. Electrophysiology study showed AVWCB CL of 280ms, VAWCB CL of 300ms. AH jump noted with S2 at 250ms and ECHO beat with Isuprel during S3 at 190ms. Pentaray mapping catheter was used to tag His cloud and P1, P2 signals were seen during the mapping at left posterior fascicle. Thermocool SmartTouch SF ablation catheter was used. However due to difficulty in approaching the target ablation site, hence a transeptal puncture was done successfully with heparin protocol. Episodes of clinical VT was induced during the study which was terminated with successful step‐up ablation at 40W to 45W for 60 seconds. No further VT induced on repeat EPS.

**Conclusions:** The important ECG features to recognize in a patient presenting with narrow complex tachycardia that can be misdiagnosed as SVT with aberrancy The challenges associated with a fascicular VT ablation and the strategies and outcome from a successful mapping and ablation

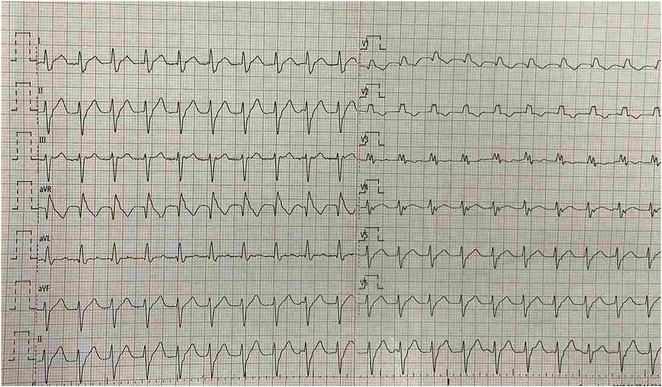

A CLASSICAL CASE OF PARAHISIAN PVC WITH PATHOGNOMONIC ECG SIGNS

**KUNARAJ PERUMALU** ^1^, CHIN WEE LEE^1^, SARAVANAN KRISHINAN^2^, MOHAMED JAHANGIR ABDUL WAHAB^1^;

^1^Hospital Pulau Pinang, Penang, Malaysia,^2^Hospital Sultanah Bahiyah Alor Setar, Kedah, Malaysia.

**Introduction:** Radiofrequency catheter ablation for parahisian premature ventricular contractions can be technically challenging and associated with increased risk of atrioventricular block.

**Methods:** N/A

**Results:** 67‐year‐old gentlemen with background history of hypertension and thyroidectomy done year 2008. He had history of admission in year 2020 for heart failure. He was diagnosed with dilated cardiomyopathy and heart failure with reduced ejection fraction with a LVEF of 15%. He had multiple admissions for recurrent chest pain with NYHA class I. A coronary angiogram done year 2020 showed presence of two vessel disease involving left circumflex artery and right coronary artery and both vessels were intervened with drug eluting stent and drug coated balloon. Despite coronary intervention, patient had recurrent episodes of palpitations and chest discomfort. His 12 lead ECG showed presence of multifocal PVC's. Hence, HOLTER done year 2021 showed PVC Burden 5.94% which increased to 24.89% the subsequent year. PVC ablation via Ensite system done year 2024, which showed frequent dominant multifocal PVC's with LBBB morphology in V1 with discordant Lead II, III aVR, aVL. Hence suspected mid to posterior TV origin. Thus, the RV (apex, RVOT) + TV annular area was mapped with PVC induced by Isuprel. Earliest breakthrough site noted at Parahisian region. His Cloud was tagged. Posterior mid‐site targeted for RFCA with unipolar QS signal earliest by 30ms. Successful ablation at 30W with irrigated tacticath. This caused transient RBBB and abolished PVC's completely. A repeated 12 lead ECG post ablation 24 hours showed no presence of further PVC's.

**Conclusions:** 1. Pathognomonic features of a 12 lead ECG that points the correct landmark for ablation and the successful outcome2. The challenges and high‐risk features associated with parahisian PVC ablation and strategies for a good clinical outcome.

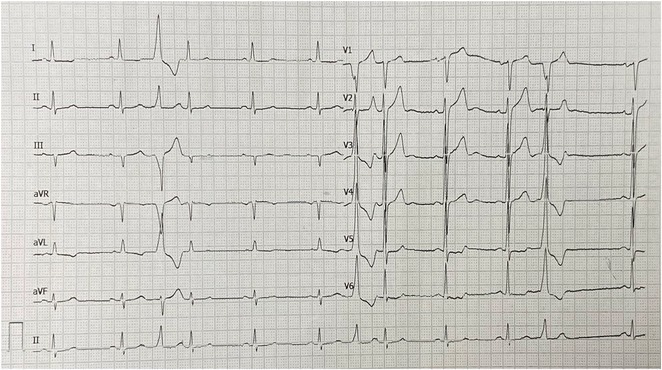

**A COMPLEX CARDIAC RESYNCHRONIZATION THERAPY DEFIBRILLATOR IMPLANTATION IN A CHALLENGING PATIENT**

**KUNARAJ PERUMALU**, TAN NEE HOOI, MOHAMED JAHANGIR ABDUL WAHAB;

Hospital Pulau Pinang, Penang, Malaysia.

**Introduction:** Cardiac resynchronization therapy device in general improves cardiac function, general well‐being with reduction of morbidity & mortality in the correctly chosen group of patients.

**Methods:** N/A

**Results:** 65‐year‐old Indian gentlemen was scheduled for CRT‐D implantation. He had recurrent admissions for heart failure with ventricular tachycardia episodes despite optimal medical therapy. The RV DF‐4 ICD lead was secured at apical RV septum with R wave > 5.0 mV. Intra‐procedural PVC's triggered sustained VT and attempt made to terminate VT with overdrive pacing from device programmer but failed after a few attempts. DC shocks (synchronized) were delivered at 200J (biphasic) and successfully reverted to sinus rhythm. Post DC shock, RV DF‐4 ICD lead R wave dropped to < 2.0 mV and high capture threshold. RV DF‐4 ICD lead unscrewed and removed. Noted reduced contractility of cardiac silhouette via fluoroscopy with gradual reduction in patient's blood pressure. Urgent ECHO showed expanding pericardial effusion due to RV DF‐4 ICD lead perforation during cardioversion. Urgent pericardiocentesis was performed. Remapping with RV DF‐4 ICD lead noted low voltage areas and scars at RV apex, apical septum and low RV septum (all R wave < 1.0 mV) keep leading to VT storm (thin and scarred septum on echocardiography). Patient went into VT storm during procedure, therefore multiple (synchronized) DC shocks (> 10 shocks) were delivered at 200J (biphasic) together with anti‐arrhythmics. Decision made to map higher at mid RV septum and high RV septum. Finally, the RV DF‐4 ICD lead was placed and screwed in mid postero‐septum of RV (R wave > 8.0 mV). Attain stability Quad LV active fixation lead was placed and screwed in at lateral branch vein of coronary sinus. The RA helix lead to right atrial appendage.

**Conclusions:** Learning points: 1. Review of cardiac MRI pre‐procedure. 2. Conduction system pacing (CSP) not a good choice due thin septum. 3. Choose to use DF‐1 device Place ICD lead at low septum for shocking purpose. Place IS‐1 lead at high septum or mid septum for sensing and pacing. 4. Can consider 3D electro‐anatomical mapping of RV cavity and coronary sinus before procedure

## CONDUCTION SYSTEM PACING MIRACLE IN A YOUNG PATIENT WITH EPICARDIAL LV PACING HEART FAILURE ‐ THE PERFECT ANTIDOTE

### **KUNARAJ PERUMALU** ^1^, SARAVANAN KRISHINAN^2^, SOTHEENATHAN KRISHINAN^1^, MOHAMED JAHANGIR ABDUL WAHAB^1^

#### ^1^Hospital Pulau Pinang, Penang, Malaysia,^2^Hospital Sultanah Bahiyah Alor Setar, Kedah, Malaysia

**Introduction:** Conduction system pacing plays a major emerging role for cardiac resynchronization in patients with heart failure reduced ejection fraction and inter‐ventricular dyssynchrony.

**Methods:** N/A

**Results:** A 16‐year‐old girl with congenital complete heart block had an epicardial LV lead (VVIR) implanted on 30/08/2008 and pulse generator change done year 2018. Her echocardiogram year 2009 showed LVEF of 72%. Her mother is anti‐Ro/SSA antibody positive. She had an eventful birth with diagnosis of persistent pulmonary hypertension of newborn and intraabdominal sepsis that improved with nitric oxide ventilation and antibiotic therapy. She was admitted for heart failure with NYHA class III in December 2023. Her heart failure medications were optimized prior to conduction system device implantation. Her echocardiogram in December 2023 showed LVEF of 15% with global hypokinesia and poor RV systolic function (TAPSE 1.20cm; RVs”= 4.3cm/s), severe TR (ePASP = 48mmHg) and moderate MR. Coronary angiogram showed smooth coronaries. Dual chamber pacemaker RV lead helix was screwed at low septal and extended to LBB area with LVAT of 84ms and V1p ‐ V6p = 60ms. RA lead helix to right atrial appendage with P wave > 4.0mV. Her native ECG showed LBBB morphology and post conduction system implantation showed narrow complex RBBB morphology. Her follow‐up in device clinic showed remarkable improvement in her LVEF and activities of daily living to NYHA I with medication compliance.

**Conclusions:** 1. The effective outcome of conduction system pacing in a young patient with LV dyssynchrony heart failure with reduced ejection fraction. 2. The options of choosing between conduction system pacing vs CRT‐P(conventional pacing) in patients with HFrEF

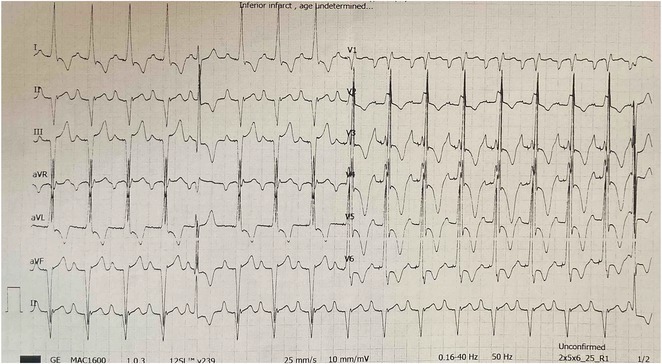

## WHEN THE CURE CAUSES THE PAIN: POST‐CARDIAC INJURY SYNDROME FOLLOWING VENTRICULAR TACHYCARDIA ABLATION

### **DAVID JONATHAN PESIRERON**, DANIEL NUGRAHA, SAFIR SAFIR, ARUMAN YUDANTO ARIBOWO BINARSO, PIPIN ARDHIANTO

#### Kariadi General Hospital, Semarang, Indonesia

**Introduction:** Post Cardiac Injury Syndrome (PCIS) is an inflammatory syndrome secondary to cardiac injury that develops after percutaneous cardiac intervention such as catheter ablation. Acute post‐ablation pericardial effusion as a manifestation of PCIS is a common complication of atrial fibrillation ablation but its occurrence following an uneventful ventricular tachycardia (VT) catheter ablation is rare.

**Methods:** N/A

**Results:** A 47‐year‐old woman [MS1] diagnosed with idiopathic right ventricular outflow tract (RVOT) ventricular tachycardia was referred for an electrophysiological study and catheter ablation. During the procedure, a total of four radiofrequency (RF) burns were applied to the anterior and posterior‐mid septal region of RVOT. In the following day, the patient had a severe chest discomfort with a slight dyspnea, relieved when she's leaning forward. 12 lead ECG examinations showed ST elevation in almost all lead (lead I, II, II, avF, avL, V4‐V6), with PR depression. Laboratory findings showed elevated CRP and Troponin, Transthoracic echocardiography revealed circumferential pericardial effusion. The patient was diagnosed with PCIS and initiated on NSAID, steroid and low‐dose colchicine. At a 5‐day follow‐up, the patient was free of symptoms and the pericardial effusion was diminished.

**Conclusions:** Pericarditis as a manifestation of Post Cardiac Injury Syndrome (PCIS) although rarely occurred following a VT catheter ablation, can still happen. Early identification and prompt treatment is vital to mitigate the complication.

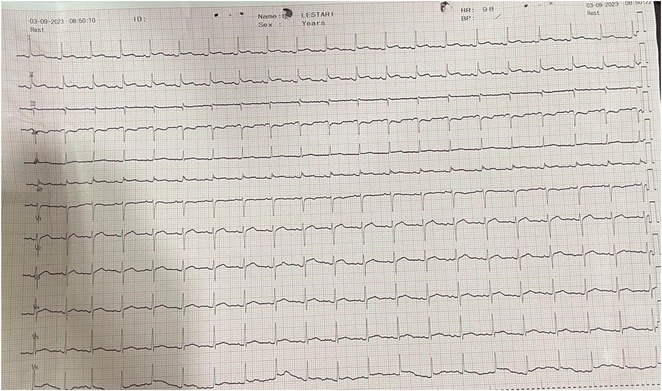

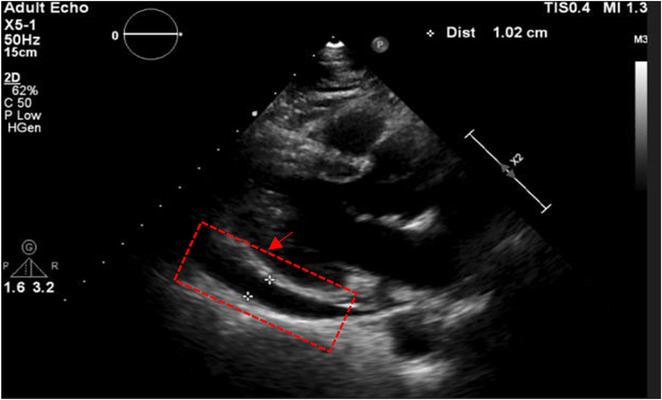

## A NOVEL DEEP ENSEMBLE METHOD FOR IMAGE‐BASED ELECTROCARDIOGRAPHIC DIAGNOSIS OF ATRIAL FIBRILLATION

### **JUSTIN PHAN** ^1,2,3^, GEORGE PARKER^1^, ADAM HILL^1,3^, JAMIE VANDENBERG^1,3^, REZA ARGHA^3^

#### ^1^Victor Chang Cardiac Research Institute, Darlinghurst, Australia,^2^St Vincent's Hospital Sydney, Darlinghurst, Australia,^3^University of New South Wales, Sydney, Australia

**Introduction:** Image‐based machine learning is an increasingly popular method for performing electrocardiographic (ECG) classification and allows for leverage of large historical databases. We describe a method for training a two‐dimensional convolutional neural network (CNN) to detect atrial fibrillation (AF) on images of 12‐lead ECGs utilising an ensemble of pre‐trained neural networks. We provide benchmark testing compared to a previously validated approach using digital ECG signals.

**Methods:** EfficientNet B3 is a pretrained CNN for image classification. We retrained EfficientNET B3 to perform ECG classification. The features extracted from different layers of the pretrained model were used to train an ensemble of neural networks. Two ensemble CNNs were studied. The first ensemble set (truncNETs) were trained upon 3 truncated forms of the EfficientNet B3, each with different splits of frozen and unfrozen layers. The second ensemble set (retrainNETs) comprised the unfrozen truncated networks from truncNETs.The PTB‐XL ECG database (Wagner et al., 2020) was used to produce 12 lead ECG images using ECG_plot (dy1901, 2022). These images were used to develop the ensemble classifiers to perform AF classification. Binary classification was performed (AF vs not AF). The data was split into an 80% training cohort and a 20% validation cohort. The performance of the neural net was compared to a validated high‐performing 1‐dimensional (1D) CNN using digital signals (DigitalNet; Cai et al., 2020).

**Results:** The performance metrics for a single EfficientNet B3, truncNETs, retrainNETs and DigitalNet are shown. The retrainNETs ensemble network offered the best performance of all image‐based ECG classification networks, with an F‐1 score of 0.86 (AUC 0.99).

**Conclusions:** An ensemble of neural net classifiers trained on features from 2‐dimensional CNNs to classify ECG images offers similar performance to 1‐D CNNs utilising 1‐D digital ECG data for the classification of atrial fibrillation. This approach may allow harnessing of pre‐existing libraries of ECG images to train neural networks for new applications.

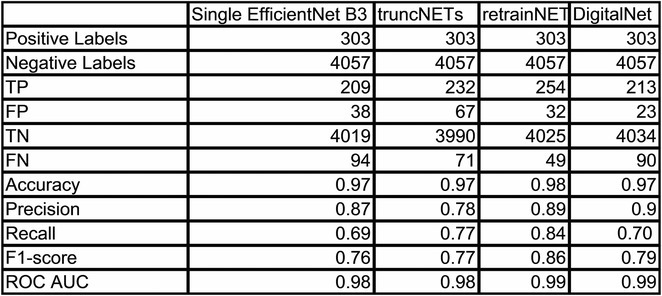

## ELECTRICAL PERFORMANCE OF SUTURE‐ON AND SCREW‐IN EPICARDIAL PACING LEADS

### **JUSTIN PHAN** ^1,2,3^, CALLUM CHERRETT^1^, ALASDAIR WATSON^1^, BRUCE WALKER^1^, WILLIAM LEE^1^, RAJESH SUBBIAH^1,2,3^

#### ^1^St Vincent's Hospital Sydney, Darlinghurst, Australia,^2^Victor Chang Cardiac Research Institute, Darlinghurst, Australia,^3^University of New South Wales, Sydney, Australia

**Introduction:** There is limited data on the performance of suture‐on (SO) compared to screw‐in (SI) permanent epicardial pacing leads. We sought to compare the electrical performance of the SO Medtronic CapSure Epi (4968) lead and the SI Enpath MyoPore (511212) leads.

**Methods:** We reviewed epicardial lead implants in our institution between January 2020 and December 2023. Demographic data, indication for pacing and lead location were recorded. Time of implant (acute) and early (6 month) electrical parameters were compared.

**Results:** Eighty eight patients were included in the analysis. Thirty‐eight screw‐in leads were implanted in 35 patients and 78 suture‐on leads were implanted in 53 patients. The indication for pacing was cardiac resynchronisation in 44 patients and atrioventricular block in 44 patients.

SO epicardial leads had higher acute pacing threshold (TH) [V@0.4ms; 1.1 vs 0.85, p <0.029] but lower early TH (1 vs 1.9, p <0.029). This relationship was similar for both right ventricular (RV) and left ventricular (LV) positions. The SO lead group had higher pacing impedance (IMP) compared to SI leads (acute: 707 vs 399 ohms, p <0.0001 and early: 602 vs 399 ohms, p <0.0001). In the RV position, SO leads had higher R wave sensing (acute: 10 vs 6mV, p=0.002 and early: 11.1 vs 5.5mV, p=0.005). Nine patients with SI leads had elevated TH (>2.5V@0.4ms), including 2 patients with loss of capture on follow up, compared to 1 patient in the SO lead group. No patients in the SO lead group had loss of capture at early follow up.

**Conclusions:** Suture‐on epicardial leads exhibited superior electrical performance compared to screw‐in epicardial leads.

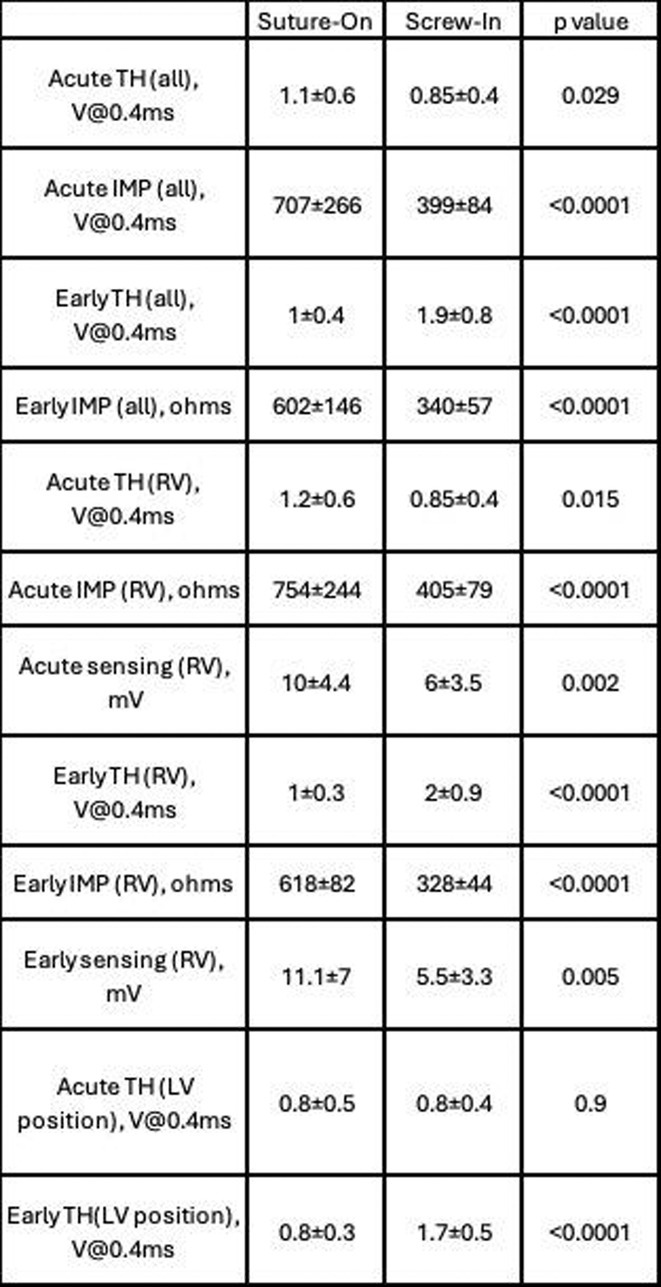

## HYBRID VERSUS CATHETER ABLATION: 24 MONTH OUTCOMES OF THE RANDOMIZED CEASE‐AF TRIAL

### **ADRIAN PICK** ^1^, NICOLAS DOLL^2^, TIMO WEIMAR^3^, DARIUSZ KOSIOR^4^, ALAN BULAVA^5^, ALES MOKRACEK^5^, GEROLD MÖNNIG^6^, JONATHAN SAHU^7^, STEVEN HUNTER^8^, MAURITS WIJFFELS^9^, BART VAN PUTTE^10^, NORMAN RÜB^11^, PETR NEMEC^12^, TOMAS OSTRIZEK^12^, ERIK FRANSEN^13^, PIOTR SUWALSKI^14^

#### ^1^Victorian Heart Hospital, Melbourne, Australia,^2^Schüchterman Klinik, Bad Rothenfelde, Germany,^3^University of Tübingen, Tübingen, Germany,^4^Central Clinical Hospital of the Ministry of Interior and Administration, Warsaw, Poland,^5^Ceske Budejovice Hospital, Ceske Budejovice, Czech Republic,^6^Schüchtermann‐Klinik, Bad Rothenfelde, Germany,^7^Northern General Hospital, Sheffield, United Kingdom,^8^Northern Genral Hospital, Sheffield, United Kingdom,^9^St. Antonius Hospital, Nieuvegein, Netherlands,^10^St. Antonius Hospital, Neuvegein, Netherlands,^11^RKH Klinikum Ludwigsburg, Ludwigsburg, Germany,^12^Center of Cardiovascular Surgery and Transplantation, Brno, Czech Republic,^13^AtriCure Inc., Amsterdam, Netherlands,^14^National Medical Institute of the Ministry of Interior and Administration, Warsaw, Poland

**Introduction:** CEASE‐AF revealed superior rhythm outcome for epi‐/endocardial hybrid ablation compared to catheter ablation in non‐paroxysmal atrial fibrillation (AF). We report if the benefit could be maintained mid‐term and the effect on quality of life (QoL).

**Methods:** CEASE‐AF (NCT02695277) is a multi‐center, 2:1 randomized controlled trial. Eligible patients were 18‐75yrs with symptomatic, drug‐refractory persistent AF with enlarged left atrium (LA, diameter 4‐6cm) or longstanding persistent AF (LSPAF) and no prior ablation. The Hybrid arm (HA) included thoracoscopic epicardial radiofrequency (RF) ablation for isolation of pulmonary veins (PVI) and left atrial posterior wall with epicardial left atrial appendage exclusion followed by sequential endocardial ablation 91‐180d later. The Catheter arm (CA) consisted of a minimum of RF PVI, allowing repeat ablation from 91‐180d postprocedurally. Both arms allowed additional lesions. Effectiveness was freedom from AF/atrial flutter/atrial tachycardia episodes >30sec through 24mo, without Class I/III anti‐arrhythmic drugs except those that had failed at same or lower dosage previously. Safety was composite major complication rate, adjudicated by an independent Clinical Events Committee. QoL was assessed by Short Form‐12 (SF‐12) from baseline to 24mo.

**Results:** Of 102 HA and 52 CA patients, 26% were female, mean age 60.7±7.9yrs, mean LA diameter 4.7±0.4cm, and 19% LSPAF with no differences between arms. Through 24mo, effectiveness was 66.3% (63/95) in HA and 33.3% (17/51) in CA (Δ 33.0% [95% CI 14.3%, 48.3%; p<0.001]). Safety rates through 24mo were similar at 10.8% (11/102) in HA and 9.6% (5/52) in CA (p=1.0). Mean improvement from baseline to 24mo in SF‐12 physical component score was significant in HA (5.7±10.49; p<0.0001) and CA (5.1±9.0; p=0.0003). However, only HA had a significant improvement in SF‐12 mental component score (4.4±9.36; p<0.0001).

**Conclusions:** Hybrid ablation has maintained superior mid‐term rhythm outcomes that were reflected in improved physical and mental QoL scores. This was achieved without significantly increased complications compared to catheter ablation.

## SIMPLE DIAGNOSTIC SCORE IN PATIENTS PRESENTING WITH SUSPECTED ACUTE STEMI

### **NATNICHA PONGBANGLI** ^1^, THANAPORN ANUCHAI^1^, WANWARANG WONGCHAROEN^2^

#### ^1^Chiangrai Prachanukroh Hospital, Chiangrai, Thailand,^2^Chiang Mai University, Chiang Mai, Thailand

**Introduction:** Patients with suspected acute ST elevation myocardial infarction (STEMI) requires immediate attention. However, not all patients actually have myocardial infarction. Our aim was to develop a diagnostic score to predict false activation of STEMI.

**Methods:** This was a prospective observational study. We recorded data through alerts from the STEMI line application. To determine the independent predictors for false‐ STEMI activation, a multivariable logistic regression analysis was conducted. Regression coefficients were used to generate a diagnostic score to predict false STEMI activation.

**Results:** A total of 301 patients were included. The incidence of false STEMI activation was 38.21%. Left ventricular hypertrophy emerged as the most common misinterpretation leading to STEMI activation. Multivariable analysis revealed independent predictors for false STEMI activation, including non‐typical chest pain (odds ratio [OR] 260.73; 95% confidence interval [CI] 37.36‐1819.48), absence of reciprocal changes (OR 180.87; 95% CI 18.67‐1752.35), and localized ST elevation at V1‐V3 or V1‐V4 (OR 101.43; 95% CI 13.46‐764.16). We devised a simple diagnostic score using regression coefficients: assigning 1 point for non‐typical chest pain, absence of reciprocal change, and localized STE at V1‐V3 or V1‐V4. If the score reaches at least 2 points, it indicates a potential false STEMI activation. The receiver operating characteristic curves for our risk score yielded impressive areas under the curve at 0.983. Sensitivity raised at 96.5% while specificity reached 95.7%.

**Conclusions:** We proposed a simple diagnostic score to predict false‐STEMI activation. Larger‐scale validation studies are required to validate our score and demonstrate its potential in decreasing false activation of STEMI in clinical practice.

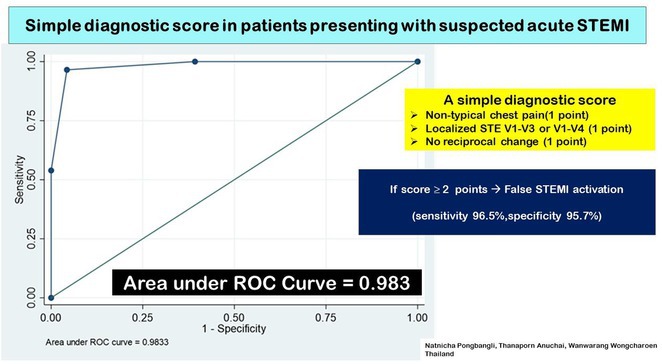

## REVEALING BRUGADA TYPE I PATTERN IN RURAL SETTING WITH LIMITED RESOURCES : COMBINING HIGH RIGHT PRECORDIAL LEAD ELECTROCARDIOGRAM AND FULL STOMACH TEST METHOD

### **CHIQUITA FEBBY PRAGITARA** ^1^, MUAMMAR EMIR ANANTA^2^, BILLY DOHOTAN DOHAR SITUMORANG^1^, ISNA NISRINA HARDANI^1^, ERAGRADITYA HARDIANTO^1^, BAYUSHI EKA PUTRA^1^, JERRY MARISI HASIHOLAN MARBUN^1^

#### ^1^Berkah General Hospital, Pandeglang, Indonesia,^2^Cempaka Putih Jakarta Islamic Hospital, Central Jakarta, Indonesia

**Introduction:** Southeast Asia has the highest prevalence of Brugada Syndrome (BrS), an inherited arrhythmic disorder leading to syncope and sudden cardiac death (SCD). Diagnosing BrS is challenging and often requires a sodium channel blocker drug‐provocation test, which poses risks and not always available, making it impractical in rural areas. We present a case of unmasking type 1 Brugada ECG pattern using simple diagnostic tests suitable for rural settings in a patient without a family history of syncope or SCD.

**Methods:** N/A

**Results:** A 64‐year‐old male with a history of smoking presented with atypical chest pain, nocturnal agonal respiration, and frequent lightheadedness but no episodes of syncope. He denied any family history of sudden death, cardiac arrest, or drug use. Physical examination, lab tests including renal function, electrolytes, troponin, as well as echocardiography were all normal. Basal ECG showed a ≥2 mm J‐point elevation, ≥1 mm ST segment elevation, and a saddleback appearance with a positive T wave in V2, suggesting a type II Brugada pattern. The beta angle was 44 degrees and the length of the base of the r’ wave triangle 5 mm below the maximum rise point was >4mm. The Shanghai score was ≥3.5, and the nomogram predicted a >95% chance of a positive provocation test. Another known procedure, the full stomach test, also showed significant ST elevation changes in V1‐V2. This was due to increased vagal tone induced by rapidly filled stomach from a full meal and soda. Furthermore, repositioning the right precordial leads (V1 and V2) to the third intercostal space revealed a more prominent coved ST‐segment elevation with a J‐point amplitude ≥2 mm and a negative T wave. More cranial positioning of high right precordial leads (HRPL) enhances V1 and V2 sensitivity for detecting the type 1 Brugada pattern due to closer apposition to right ventricular outflow tract.

**Conclusions:** HRPL placement combined with the full stomach test is more sensitive than the full stomach test alone in revealing the Brugada type I pattern. This simple combined method can aid in BrS detection in rural areas.

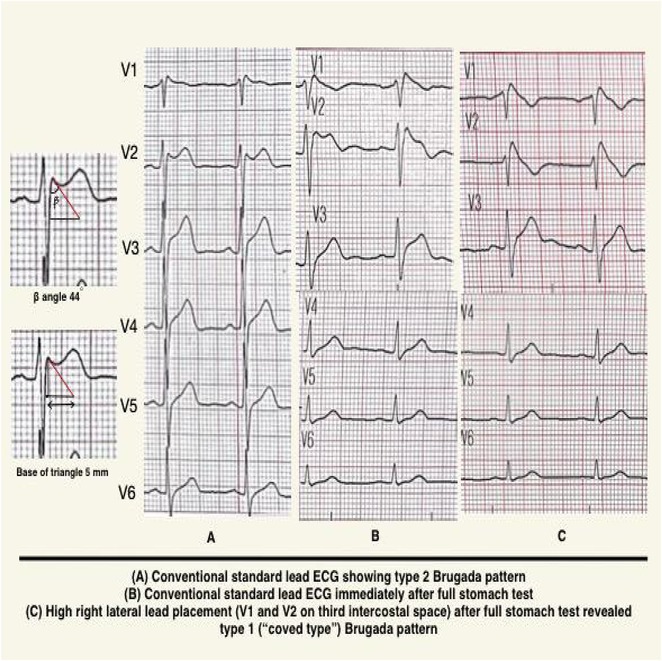

## HIGH‐RISK ABLATION OF ANTEROSEPTAL AND PARA‐HISIAN ACCESSORY PATHWAYS UTILIZING 3D MAPPING FOR WPW SYNDROME: A CASE REPORT

### **RICKY PRATAMA**, MUZAKKIR AMIR

#### Hasanuddin University, Makassar, Indonesia

**Introduction:** Anteroseptal and para‐hisian accessory pathways (AP) are rare variants in Wolf Parkinson White (WPW) syndrome, typically diagnosed with an ECG algorithm and treated with ablation therapy. While radiofrequency ablation (RFA) is effective for terminating these APs, it carries a high risk of complete atrioventricular block. A 50‐year‐old woman with sudden self‐limiting palpitation, ECG showed WPW suggestive anteroseptal AP. This case emphasizes the importance of precise AP mapping using 3D mapping technology.

**Methods:** N/A

**Results:** Anteroseptal and para‐hisian APs are located close to His Bundle, direct contact of RFA poses a high risk of complete AV block, necessitating a permanent pacemaker. This risk is mitigated by using 3D mapping, which helps to confirm the precise location of the anteroseptal AP. An electrophysiology study (EPS) with 3D mapping of this patient confirmed the anteroseptal AP. Initial multiple RFA caused intermittent junctional rhythm with a delta wave, leading to ablation cessation. Re‐evaluation with a CS catheter identified AV fusion at CS 9‐10, 3D mapping indicating para‐hisian AP. Multiple RFA with 30 watts, 60°C for 120 seconds successfully separated the AP. The final ECG showed sinus rhythm with a left bundle branch block (LBBB) pattern.

**Conclusions:** Despite the high risk of complete AV block associated with RFA, 3D mapping allows for accurate targeting and effective treatment; highlights the efficacy and safety of this approach, achieving a high success rate while minimizing the risk of complications. With precise ablation, the treatment success rate is expected to be 95% to 99%.

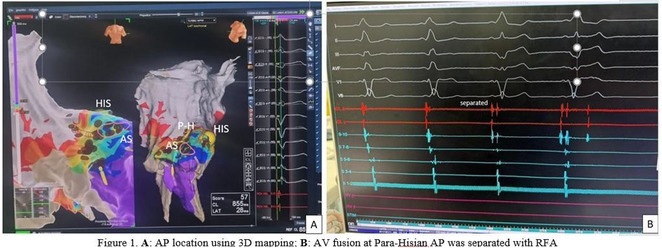

## NEW ONSET ARRHYTHMIA IN ACUTE CORONARY SYNDROME (ACS) PATIENT ACCORDING TO REVASCULARIZATION STATUS

### **WULAN PRISILLA**, HAUDA EL RASYID, TOMMY DAINDES

#### Department of Cardiology and Vascular Medicine, Andalas University/ DR. M. Djamil Hospital, Padang, Indonesia

**Introduction:** Arrhythmia in ACS is a common clinical problem requiring prompt recognition and treatment. Acute and evolving myocardial ischemia is well described to be highly arrhythmogenic due to severe metabolic and electrophysiological changes. The management goal of ACS is complete revascularization (CR). Studies showed that an increased risk of ventricular tachyarrhythmias and mortality in patients with incomplete revascularization (ICR). This study aims to identify in hospitalization / short time risk of new onset arrhythmia based on revascularization status in ACS patient.

**Methods:** This is a retrospective, single center study of patient diagnosed with ACS that admitted to Dr. M. Djamil Hospital Padang, West Sumatera, Indonesia. The inclusion criteria are all of ACS patients that performed coronary angiography (CAG) during July until December 2023. The exclusion criteria are patients with known arrhythmia and earlier percutaneous coronary intervention (PCI) or coronary artery bypass graft (CABG) surgery before admission.

**Results:** Total sample are 98 patients, 32 (33%) of them were performed CR and 68 (67%) ICR. The PCI procedure are more in men compare than women, but no differences between ICR or CR status, neither age of the patients. Based on revascularization status analysis there are no significant differences between comorbidities. Meanwhile there is a significant difference between ICR and CR status in ACS patients (p<0.05). New onset arrhythmia was found in 7 (21%) patients with CR and 23 (34%) in patient with ICR. All‐cause mortality found in 6 (9%) patients with ICR but not find in CR status. There are no differences between new onset arrhythmia events and revascularization status (p>0,05).

**Conclusions:** The results of this study did not find a significant correlation of the onset of new onset arrythmia and mortality between patients undergoing CR and ICR status. However, all types of arrhythmias were found more in the ICR group.

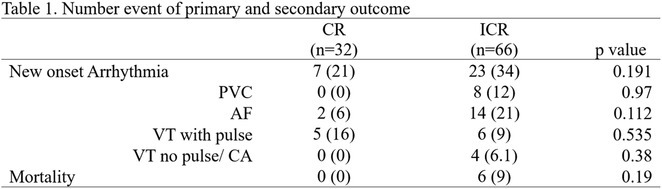

## THE ROLE OF HOXA5 IN ATRIAL ELECTROPHYSIOLOGY ALTERNATION AND ATRIAL FIBRILLATION VULNERABILITY IN T2DM MICE

### **SIJIA PU**, LU FU, FANG RAO, YUMEI XUE

#### Gangdong Cardiovascular Institute, Guangdong Provincial People's Hospital (Guangdong Academy of Medical Sciences), Guangzhou, China

**Introduction:** HOXA5, a metabolism‐related transcription factor, plays an important role in metabolic diseases and cardiac hypertrophy. This project aims to investigate whether HOXA5 is involved in diabetes mellitus (DM)‐related atrial conduction disorders and susceptibility to atrial fibrillation (AF), as well as its pathology alteration.

**Methods:** We conducted experiments on *db/db* mice and wild‐type mice at the age of 12 weeks. Adeno‐associated viruses serotype 9 vectors (AAV9) containing a cardiomyocyte‐specific cTnT promoter carrying the shRNA against HOXA5 or a non‐target shRNA were used to establish a heart‐specific HOXA5‐knockdown mouse model. The alternation in atrial electrophysiology and atrial excitation patterns was tested by multi‐channel electrophysiological mapping, programming electrical stimulation, and induction of AF in the whole Langendorff working hearts. The functional and pathologic changes in mouse hearts were detected by cardiac ultrasound, Masson staining, and oil‐red staining. Western blot and immunofluorescence were used to detect the expression of HOXA5.

**Results:** The expression of HOXA5 in the atrial tissue of *db/db* mice is significantly higher than in wild‐type mice, proven by western blot and IF. In comparison with wild‐type mice, *db/db* mice showed decreased atrial conduction, increased dispersion, and increased susceptibility of AF along with a longer atrial effective refractory period. These can be alleviated by heart‐selective knocking down HOXA5 *in vivo*. Compared with wild‐type mice, *db/db* mice demonstrated mild fibrosis and lipid accumulation in atrial tissue, which can also be improved by cardiac‐specific down‐regulating of HOXA5.

**Conclusions:** We preliminarily prove that expression of HOXA5 is positively associated with atrial electrophysiology abnormality and increased AF vulnerability in type 2 DM mice, which can be ameliorated by knocking it down. This study reveals for the first time that HOXA5 is involved in DM‐related atrial electrical conduction abnormalities, providing a new potential target for DM‐related atrial fibrillation.

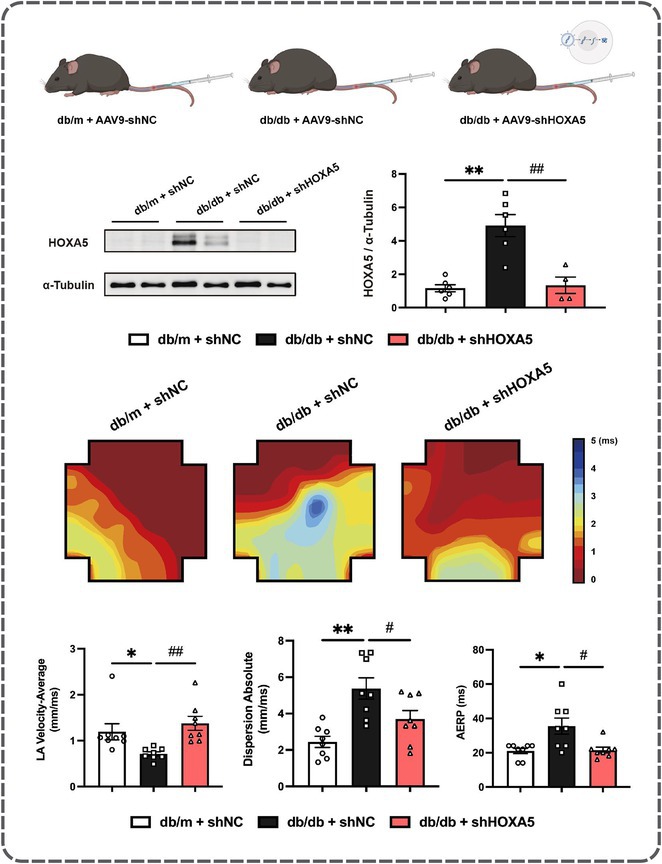

## ASSESSING TRICUSPID REGURGITATION FOLLOWING HIS AND LEFT BUNDLE BRANCH PACING WITH STYLET‐DRIVEN LEADS: A SHORT‐TERM COHORT STUDY

### **ANDRIANY QANITHA**, MUZAKKIR AMIR, ARMANDO TITO, AUSSIE F GHAZNAWIE

#### Hasanuddin University, Makassar, Indonesia

**Introduction:** The stylet‐driven delivery system was substituted with a pre‐shaped catheter delivery system for conduction system pacing several years ago, primarily due to its advantage of facilitating easier access to the pacing location. However, in several low‐ and middle‐income countries, including Indonesia, the availability of catheter systems remains limited. This study aimed to assess the incidence and alterations in the severity of tricuspid regurgitation (TR) when employing the stylet‐driven lead delivery system in patients undergoing His bundle (HBP) and Left Bundle Branch pacing (LBBP).

**Methods:** This retrospective cohort study was conducted on patients who underwent HBP and LBBP utilizing a stylet‐driven delivery system between January and December 2022, drawn from the Makassar Permanent Pacemaker (PPM) Registry. TR was evaluated through echocardiography before implantation and at the six‐month after PPM implantation. For patients who exhibited no TR post‐implantation, the lead position was assessed utilizing 3D echocardiography.

**Results:** Among the 42 participants (14 males and 28 females), the mean age was 65 ± 11.7 years. The primary reasons for PPM implantation were total atrioventricular block (TAVB) in 25 (59.5%) participants and sinus node dysfunction (SND) in 17 (40.5%) participants. HBP was performed on 21 (50%) participants, while the remaining 21 (50%) underwent LBBP. Tricuspid regurgitation was present in 19 (33.5%) patients before implantation. Following implantation, improvements in TR were observed in four (9.5%) patients. However, no significant association was found between HBP and LBBP using the stylet‐driven lead delivery system and the occurrence or improvement of TR after implantation.

**Conclusions:** The stylet‐driven system proves to be efficient and could be considered an acceptable option for HBP and LBBP procedures.

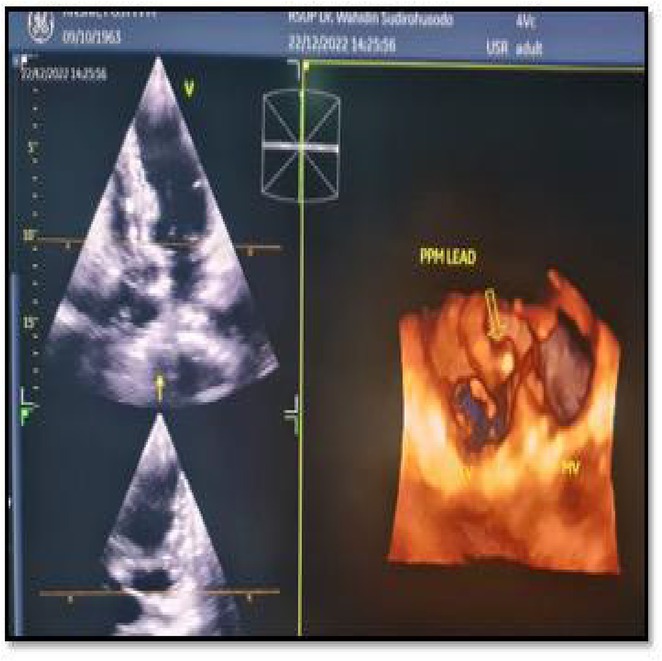

## TYPICAL ATRIOVENTRICULAR NODE REENTRANT TACHYCARDIA IN A 34‐YEAR‐OLD MALE WITH SUBCLINICAL HYPOTHYROIDISM: A CASE REPORT

### **ANDRIANY QANITHA**, MUZAKKIR AMIR, RIFNA FEBRAINI ASNAWI

#### Hasanuddin University, Makassar, Indonesia

**Introduction:** Supraventricular arrhythmias are prevalent and often symptomatic, necessitating management through pharmacological and electrophysiological interventions. Several studies have demonstrated a correlation between hypothyroidism and vascular abnormalities, including endothelial dysfunction, arterial stiffness, and atherosclerosis. We report a case of typical Atrioventricular Node Reentrant Tachycardia (AVNRT) and subclinical hypothyroidism.

**Methods:** N/A

**Results:** A 34‐year‐old male presented to the Emergency Department of Dr. Wahidin Sudirohusodo Hospital with palpitations that commenced three hours prior to admission. Accompanying symptoms included dyspnea, dizziness, and generalized weakness. The patient had no previous history of palpitations but reported exertional dyspnea and orthopnea. His medical history included hypertension and hypercholesterolemia.Physical examination revealed a severely ill but alert and oriented patient. Vital signs were: blood pressure 119/92 mmHg, heart rate 166 bpm (regular), respiratory rate 24 breaths per minute, and oxygen saturation 98% on room air. The ECG showed a supraventricular rhythm consistent with AVNRT, with a heart rate of 166 bpm, regular rhythm, normoaxis, visible P waves, and a P rate greater than the QRS rate. A Modified Vagal Maneuver was ineffective in correcting the heart rhythm. Subsequent synchronized cardioversion at 50 Joules, successfully restored sinus rhythm (heart rate 65 bpm, regular, normoaxis), as confirmed by ECG. Thyroid function tests indicated subclinical hypothyroidism with decreased TSH levels. Invasive coronary angiography revealed three‐vessel coronary artery disease, prompting a recommendation for revascularization following a surgical conference. After a 10‐day hospitalization, the patient's condition stabilized, and he was discharged.

**Conclusions:** This case highlights the importance of integrated medical approaches in managing complex conditions that involve both endocrine and cardiovascular systems.

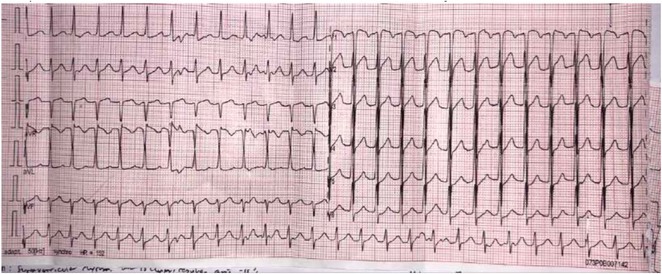

## ACUTE AND THREE‐MONTH FOLLOW‐UP EVALUATION OF ECHOCARDIOGRAPHY‐GUIDED PERCUTANEOUS TRANSAPCIAL INTRAVENTRICULAR SEPTUM PACING IN A CANINE MODEL

### **ZHAOHUI QIU** ^1^, XIANHAO WU^1^, KE DU^1^, HAIYAN CHEN^2^, ZHENG LI^2^, YUHUA TANG^1^, WEI HU^1^, RUIHAO WU^1^, JIAYIN ZHONG^1^, WANLAN CHEN^1^, LULU CHEN^1^, MINGYUE SUN^1^, JING NI^1^, LIHONG ZHOU^1^, ZIMING OU^1^, ZHONGCHENG XU^1^

#### ^1^Tong Ren Hospital Shanghai Jiao Tong University School of Medicine, shanghai, China,^2^Zhongshan Hospital Fudan University, shanghai, China

**Introduction:** Transvenous lead implantation is prone to venous access issues and lead‐related complications. This study aimed to evaluate the technical feasibility and procedural safety of a novel pacing method, echocardiography‐guided percutaneous transapical intraventricular septum (PTAIVS) pacing.

**Methods:** Twelve adult dogs (20.17±1.80 kg) underwent PTAIVS pacing. Transthoracic echocardiogram was employed to locate the apical region for insertion. Then, under real‐time transesophageal echocardiography guidance, a coaxial introducer needle was inserted obliquely from the designated region into the IVS. Upon reaching the basal segment of the IVS, the inner stylet was retracted and a pacing lead was deployed through the needle cannula for fixation. Implantation for the initial six animals used a 17G*10cm coaxial introducer needle (CareFusion, USA) and a Model 3830 pacing electrode (Medtronic, USA). Subsequently, a revised pacing set comprising a 15G*10cm coaxial introducer needle (CareFusion, USA) and a pacing lead with an extended helix of 10.0mm (SMCSP2023F, Singular Medical, China) was used for the remaining six experiments.

**Results:** All animals had successful lead placements without any acute procedural complications. The mean procedure time, from insertion to suture closure, was 21 (19‐24) minutes. The mean paced QRS duration was 67.00±8.93ms, the capture threshold at 0.5ms was 0.80±0.12V, the impedance was 693.67±254.07Ω, R‐wave amplitude was 13.88±7.26 mV. Lead dislodgements occurred in 6 animals (100.0%) at 1‐month follow‐up using the initial pacing set. Subsequent modifications led to an 83.3% lead stability rate in 5 out of 6 animals at the 3‐month follow‐up. Histopathology of these 5 animals showed no identifiable damage to the aortic valve or ventricles.

**Conclusions:** Our study demonstrated the technical feasibility of echocardiography‐guided PTAIVS pacing in a canine model, characterized by brief procedural duration and relative safety. PTAIVS pacing has the potential to be an alternative to transvenous pacing.

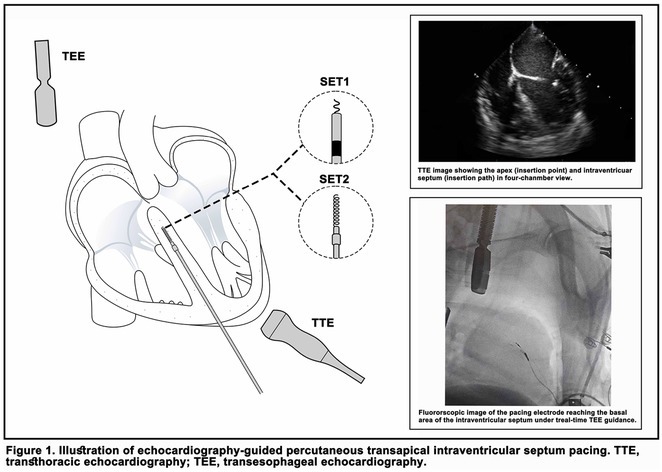

## ABLATION OF ACCESSORY PATHWAY FROM AN UNSUAL SITE WITH 3D MAPPING & ZERO FLUORO

### VASUNDHARA PONNAGANTI, MURALIDHARAN THODDI RAMAMURTHY, PREETAM KRISHNAMURTHY, **SWAMINATHAN VEERASAMY R**, ABHILASHA MUNISINGH, ARUN SHRIRAM AMUDHAN, PRASANNA SUBBARAJU

#### SRM Medical College and General Hospital, Chennai, India

**Introduction:** supraventricular tachycardia affects mainly young and middle aged adults.an electrophysiology study is definitive to diagnose and differentiate from other arrhythmia. catheter ablation is currently the best therapeutic option for symptomatic patients, and while not without risk, and is generally very well‐tolerated.

**methods:** n/a

**results:** a 38 yr old female presented with complaints of palpitations to er. Ecg showed narrow complex tachycardia which was reverted with adenosine. 2d echo showed no rwma normal lv function ef 64%. She underwent baseline inbestigations which turned out normal, patient was taken up for electrophysiology study. A decapolar catheter was used for anatomical 3d mapping, quadipolar catheter placed at his bundle. Baseline ep study revealed atrial tachycardia. There is no evidence av nodal accessory pathway. On further mapping origin of pathway was localised in superior vena cava (svc). Catheter taken to svc and careful ablation done without any injury to adjacent structures as shown in the image(1).non inducibility of the tachycardia noted with programmed electrical stimulation. Successful ablation was done without any complications with 3d mapping and zero fluoro.

**Conclusions:** superior vena cava as a source of narrow complex tachycardia is rare. limiting the use of x‐rays is necessary, especially using 3d mapping allow a zero‐fluoro (zf) approach. A lower radiation exposure may be reached, reducing fluoroscopy usage whenever possible during cardiac ablation procedures with high safety, full feasibility, and efficacy as in our case

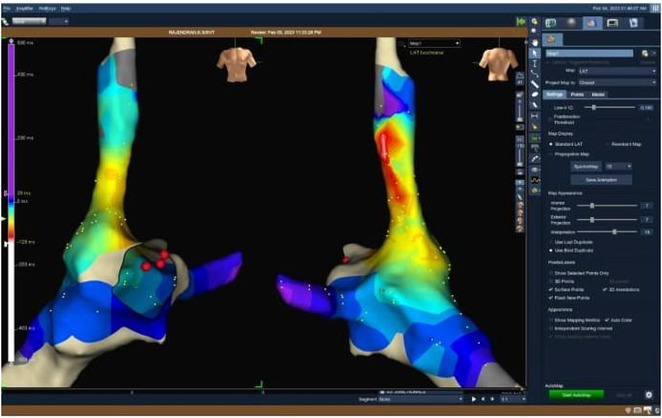

## IMPROVEMENT OF LEFT ATRIAL STRAIN IN PATIENT WITH HIGH VENTRICULAR PACING BURDEN AFTER DUAL CHAMBER CARDIAC PACEMAKER IMPLANTATION

### **ADELIA ULYA RACHMAN**, AGUNG PRASETYO WICAKSONO, FERA HIDAYATI, ERIKA MAHARANI, LUCIA KRIS DINARTI

#### RSUP Dr. Sardjito / Faculty of Medicine, Public Health and Nursing Universitas Gadjah Mada, Sleman, Indonesia

**Introduction:** Dual‐chamber cardiac pacemaker implantationremains as the first choice for the treatment of atrioventricular nodedysfunction (AVND) and sinus node dysfunction (SND). Although dual‐chamberpacing was thought to be more physiological, pacing in general has been shownto have a detrimental effect on cardiac function mainly through atrioventricular(AV) dyssynchrony. This study aims to evaluate the correlation between highventricular (V) pacing burden with atrial function measured by left atrialstrain (LAS) in patients with dual‐chamber pacemaker.

**Methods:** This study analysed 37 patients underwentdual‐chamber pacemaker implantation using ALEKA registry data at Dr. SardjitoHospital Yogyakarta from November 2022 to July 2023. Left atrial strainreservoir (LASr), conduit (LAScd), and contractile (LASct) were assessed beforeand after implantation. Patients with pacing burden ≥40% were classifiedas having high V.

**Results:** Among the observations, indicationfor pacemaker implantation was due to SND (n=10, 27%), AVND (n=22, 59.5%), andboth SND and AVND (n=5, 13.5%). Baseline LAS showed lower atrial functionbefore pacemaker implantation for high V pacing burden group (LASr 20.09±7.49%vs. 28.38±6.64%, *p*=0.008; LAScd 11.88±4.80% vs. 14.94±7.09%, *p*=0.161;LASct 8.26±4.74% vs. 13.19±3.82%, *p*=0.011), 12 patients (32.4%) of whichwere on temporary pacemaker (TPM). These differences between high V and low Vgroup were diminished in the evaluation of LAS after implantation, with high Vgroup showing significant improvement in LASr (Δ4.63±2.08%, *p*=0.029) andLASct (Δ3.71±5.84%, *p*=0.002). Difference‐in‐differences (DID) analyseswere performed to adjust for differences at baseline found that the averagetreatment effect (ATE) of the improvement of LASr (ATE 8.200, p=0.068) andLAScd (ATE 5.744, p=0.061) in high V group after implantation still holds.

**Conclusions:** Patients with high ventricular pacing burdenafter dual‐chamber pacemaker showed significant improvement of LAS afterimplantation. This suggest that ventricular pacing in dual‐chamber pacemakermight improve atrial and subsequently diastolic function through better AVsynchrony.

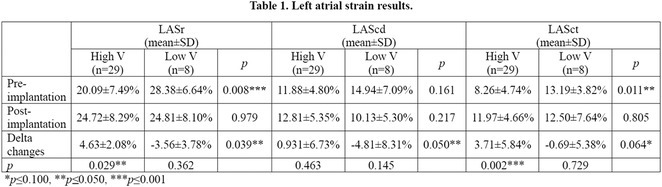

## FAMILIAL HYPERTROPHIC CARDIOMYOPATHY WITH GENETIC MUTATION CAUSING DIFFERENT CLINICAL MANIFESTATION

### **RAISSA RAFIDHINAR** ^1^, GIKY KARWIKY^2^, MOHAMMAD IQBAL^2^, CHAERUL ACHMAD^2^, HAWANI SASMAYA PRAMESWARI^3^

#### ^1^Cardiology Resident, Department of Cardiology and Vascular Medicine, Universitas Padjadjaran / Dr. Hasan Sadikin General Hospital, Bandung, Indonesia,^2^Electrophysiology and Arrhythmia Division, Department of Cardiology and Vascular Medicine, Universitas Padjadjaran / Dr. Hasan Sadikin General Hospital, Bandung, Indonesia,^3^Heart Failure Division, Department of Cardiology and Vascular Medicine, Universitas Padjadjaran / Dr. Hasan Sadikin General Hospital, Bandung, Indonesia

**Introduction:** Familial hypertrophic cardiomyopathy (HCM) is an autosomal dominant disease that could affect as many as 1 in 500 individuals, showcasing variability among patients regarding the timing of onset, phenotype, and clinical progression. The two genes in which most mutations have been described are the B‐myosin heavy chain (*MYH7*) and the myosin binding protein C (*MYBPC3*).

**Methods:** N/A

**Results:** The patient a 60‐year‐old mother whose genetic status was unknown, electrophysiology study revealed sick sinus syndrome included atrial fibrillation and bradyarrhythmia. Echocardiography revealed a dilated LA and concentric LVH with a borderline LV ejection fraction (EF) (53%). Late gadolinium enhancement (LGE) indicated transmural enhancement at the apicoinferoseptal region, patchy enhancement at the mid inferoseptal and anteroseptal areas. She passed away suddenly with an undocumented malignant arrhythmia suspected as the cause of death. Her 39‐year‐old son was found to have the MYH7 mutation. Echocardiography showed biatrial dilatation with a reduced EF (40%). LGE revealed prominent focal patchy mid‐wall enhancement at both right ventricular (RV), particularly at the anterior extending to the basal anterior, basal to mid anteroseptal, and apical anterior RV. The QRS complex initially appeared narrow but gradually widened over time, indicating significant progression compared to his mother. After recurrent episode of ventricular tachycardia (VT), an implantable cardioverter‐defibrillator (ICD) was implanted. However, he did not survive thereafter due to a VT storm followed by pulseless electrical activity. Both patients were diagnosed with HCM.

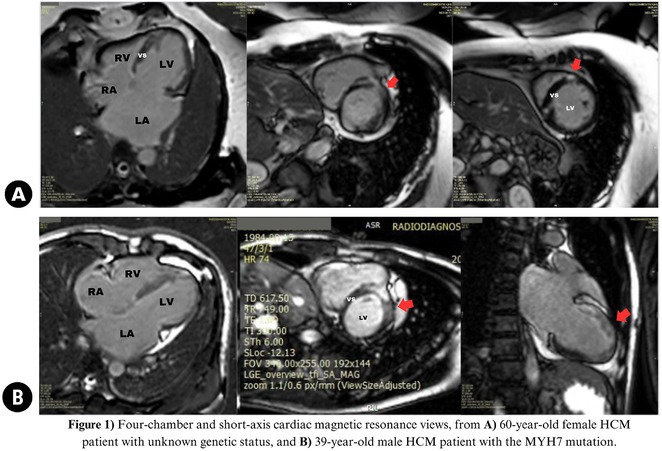

**Conclusions:** The life‐long process of LV remodeling and progressive dysfunction that occur in a substantial proportion of HCM patients and culminates in the rare but dramatic clinical evolution termed as *end‐stage* or *burned‐out* phase characterized by severe functional deterioration of the LV. It is clinically important to distinguish between the various stages of HCM. A key mechanism for adverse outcomes is believed to be myocardial fibrosis, a pathological hallmark of the condition.

## SUBCUTANEOUS ICDS IN RURAL AUSTRALIA: A FIVE‐YEAR REVIEW OF SAFETY AND EFFICACY

### **RAKIN RAHMAN** ^1^, VISHWA PAKEERATHAN^2^, ABDUL RAHMAN MOHAMMED^3^, AMARDEEP AMARDEEP^3^, SONIAH MOLOI^3^, DAVID HINDS^1^, AYRA KASSAM^1^, BOBBY JOHN^3^

#### ^1^Logan Hospital, Logan, Australia,^2^Princess Alexandra Hospital, Brisbane, Australia,^3^Townsville University Hospital, Townsville, Australia

**Introduction:** This study explores the clinical outcomes of Subcutaneous ICD (S‐ICD) implants in Rural Australia. The growing global use of S‐ICDs for preventing Sudden Cardiac Death (SCD) across various cardiac pathologies is due to its design that avoids long‐term complications of intravascular leads.

**Methods:** A retrospective analysis was conducted on a cohort of 26 patients who underwent S‐ICD implantation or generator replacement from November 2019 to January 2024 at Townsville University Hospital, Queensland, Australia.

**Results:** Our cohort of 73 patients (mean age 44.7, 13 males) had various indications for the procedure, including Hypertrophic cardiomyopathy HCM (6), Non ischemic Cardiomyopathy NICM (6), Ischemic cardiomyopathy ICM (4), Out of hospital cardiac arrest OOHCA (4) without any discernible cause, Mitral Annular Disjunction MAD (2), Long QT Syndrome LQTS (2), Arrhythmogenic Right Ventricular Dysplasia ARVD (1), Brugada Syndrome (1). Primary prevention was the indication in 10 patients and secondary prevention in 16. All procedures were performed under general anaesthesia. No acute complications were observed post‐procedure. Over a mean follow‐up of 1447.7 days, two patients received appropriate therapies. Three faced premature battery depletion, requiring generator replacement. Two received inappropriate shocks due to oversensing and lead issues. One needed a pocket revision and reimplantation after 6 years due to lead noise and inappropriate shock.

**Conclusions:** S‐ICDs are a safe and effective alternative to transvenous ICDs, especially in young patients who do not require pacing. This approach can help circumvent long‐term issues associated with transvenous leads. Our findings align with those reported in global studies.

## ASSESSMENT OF QRS COMPLEX FEATURES IN REAL WORLD ILR PATIENTS WITH PRIOR HEART FAILURE DIAGNOSIS

### NEETHU VASUDEVAN, SHANTANU SARKAR, **GAUTHAM RAJAGOPAL**

#### Medtronic, Mounds View, MN

**Introduction:** Implantable Loop Recorders (ILR) can continuously monitor electrocardiogram (ECG) in an ambulatory setting. This allows the chance to identify markers in routine cardiac ECG associated with increased risk of heart failure events (HFE). Our study evaluates the difference in QRS features among ILR patients with HFE and without HFE.

**Methods:** ILR patients with history of HFE were included from Optum® de‐identified Electronic Health Record dataset (Optum® EHR). ILR devices collected ECG data were merged with the Optum® EHR data to create a de‐identified database of real‐world patients. HFEs were defined as heart failure related inpatient or observation unit, or emergency department stay with IV (Intravenous) diuresis administration. An event and control group were created from ILR patients post implant with HFE and no HFE event, respectively. Control group included 90 days of baseline ECG and event group included 90 days of ECG prior to HFE. QRS complex was extracted from ECG signal and amplitude was normalized w.r.t to individual patient. Features such as QRS width and QRS amplitude were calculated. Mood's median test was performed to compare the QRS features among control and event group.

**Results:** A total of 900 ILR patients (Control N=767; Event N= 124) with 2769 follow up months and 156 HFE were included. Event group had wider QRS compared to control group (p value:<0.001,95% Confidence Interval for median difference: [‐10, ‐5]). No significant difference in QRS amplitude observed between the groups (p value:0.91,95% Confidence Interval for median difference: [‐0.00108618,0.000969728]).

**Conclusions:** In a real‐world population of ILR patients, we found that patients with increased risk of heart failure event has wider QRS compared to patients with reduced risk of heart failure event.

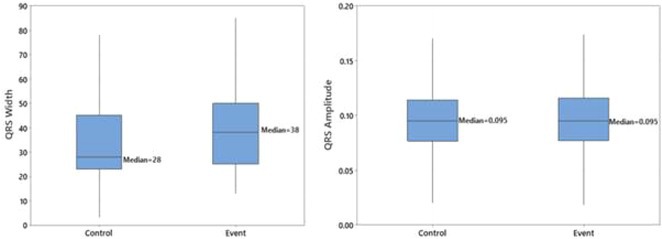

## CONTINUOUS INDEX OF CARDIAC ELECTROPHYSIOLOGICAL BALANCE TRENDS OBSERVED IN PATIENTS USING INSERTABLE CARDIAC MONITORS DURING ANTIARRHYTHMIC LOADING HOSPITALIZATION

### ANTONY CHU^1^, ANISH AMIN^2^, **GAUTHAM RAJAGOPAL** ^3^, KAJA PEDERSON^3^, BRANDON STOICK^3^, SHANTANU SARKAR^3^, AMY LAUTENBACH^3^, VENKATA SAGI^4^

#### ^1^Brown University, Providence, RI,^2^Riverside Methodist Hospital, Columbus, OH,^3^Medtronic Inc., Mounds View, MN,^4^Baptist Medical Center, Jacksonville, FL

**Introduction:** Index of cardiac electrophysiological balance (iCEB) has been described as a novel risk marker for predicting malignant ventricular arrhythmia. An implantable cardiac monitor (ICM) capable of longitudinal QT monitoring allows for automated remote monitoring of dynamic iCEB changes.

**Methods:** The developed QT algorithm for an ICM computes the QTc for every beat. QTc detected from continuously collected ICM ECG from patients undergoing antiarrhythmic drug (AAD) loading in an inpatient setting and enrolled in a clinical study at 3 sites were analyzed. The ICM ECG was processed through the QT algorithm. The corrected iCEB (iCEBc) is measured as ratio of QTc to QRS duration for every beat. The iCEBc was computed for every beat from ICM during the AAD hospitalization period and iCEBc trends for 3 hours after the first four AAD dosages were analyzed. Metrics including mean iCEBc after each dosage and max. and min. iCEBc for enrolled patients during the hospitalization period were studied.

**Results:** Continuous telemetered ICM ECG data during the index AAD hospitalization period was available in 6 out of 21 patients (avg. age 69.5) enrolled in the QT clinical study and were included in this analysis. For this analysis, QTc, QRS widths, and iCEBc were computed for over 965,000 ICM beats (avg. 161,000 beats/patient) during the AAD loading hospitalization period. Figure 1 shows the ensemble average trends of iCEBc values from patients for the first four AAD dosages. The iCEBc after the first dose was 3.2±0.4 which increased to 3.7±0.8 after the second dose. The iCEBc after the third and fourth AAD dosages were 3.5±0.8 and 3.2±0.6. The max. and min. iCEBc during the hospitalization period were 4.1±1.1 and 2.9±0.5 with the magnitude of change in iCEBc being 1.1±0.8.

**Conclusions:** ICM monitoring performed utilizing a novel automated QT algorithm provides longitudinal and long‐term dynamic iCEBc trends in patients with ICM. Drug related dynamic changes in iCEBc using an automated ICM algorithm warrants further investigation as a longitudinal marker for cardiotoxicity and cardiac death.

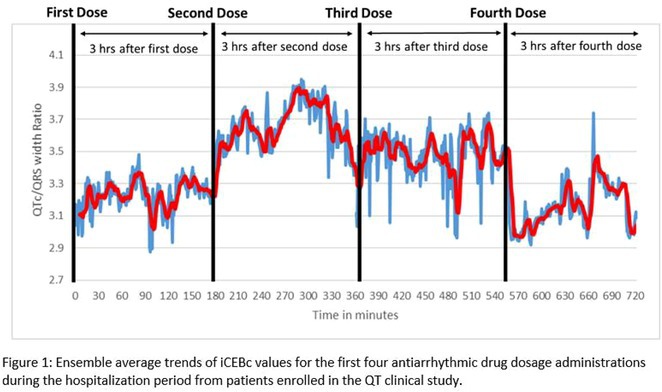

## LONG‐TERM QT INTERVAL TRENDS OBSERVED BY CONTINUOUS QT INTERVAL MONITORING USING INSERTABLE CARDIAC MONITORS DURING FOLLOW UP PERIOD AFTER ANTIARRHYTHMIC LOADING HOSPITALIZATION: RESULTS FROM THE LINQ QT STUDY

### ANTONY CHU^1^, ANISH AMIN^2^, **GAUTHAM RAJAGOPAL** ^3^, KAJA PEDERSON^3^, SHANTANU SARKAR^3^, AMY LAUTENBACH^3^, VENKATA SAGI^4^

#### ^1^Brown University, Providence, RI,^2^Riverside Methodist Hospital, Columbus, OH,^3^Medtronic Inc., Mounds View, MN,^4^Baptist Medical Center, Jacksonville, FL

**Introduction:** Pharmacotherapy including antiarrhythmic drugs (AAD) are known to prolong QT interval. An insertable cardiac monitor (ICM) capable of continuously monitoring QT interval longitudinally may be a useful tool for managing patients taking QT prolonging drugs.

**Methods:** The developed QT algorithm detects T‐wave and determines QTc for every beat. QT intervals detected from continuously collected ICM ECG data from patients enrolled in a prospective clinical study from 3 sites were analyzed with the objective of studying long‐term QT trends in patients during follow up of 90 days after Class III AAD loading hospitalization. The ICM ECG was processed through the QT algorithm. Ensemble average QTc trends were analyzed over all patients during the 90 day follow up period after discharge and metrics including QTc interval during the first and last day of follow up period, maximum and minimum QTc, and number of days between max. and min. QTc intervals during the follow up period were studied.

**Results:** ICM ECG data during the study follow up period was available in 16 out of 21 patients (avg. age 73.6 years) enrolled in the QT clinical study with completed follow up and were included in this analysis. Figure 1 shows the overall ensemble average QTc trends over all patients during the 90‐day follow up period after hospitalization. The long‐term ensemble average trend showed longer QTc immediately following AAD hospitalization discharge which progressively decreased over the 90‐day follow up. The QTc interval observed during the first day of follow up after hospitalization discharge was (median[IQR] = 413[332,454] msec) and during the 90^th^ day of follow up was (median[IQR] = 391[319,438] msec). The maximum mean QTc interval of 405 msec was observed on day 3 after hospitalization discharge and minimum QTc of 369 msec was observed on day 88 during the follow up.

**Conclusions:** ICM QT monitoring performed utilizing a unique QT algorithm provides longitudinal and long‐term QT trends in patients with ICM allowing for early intervention and preventing arrhythmias.

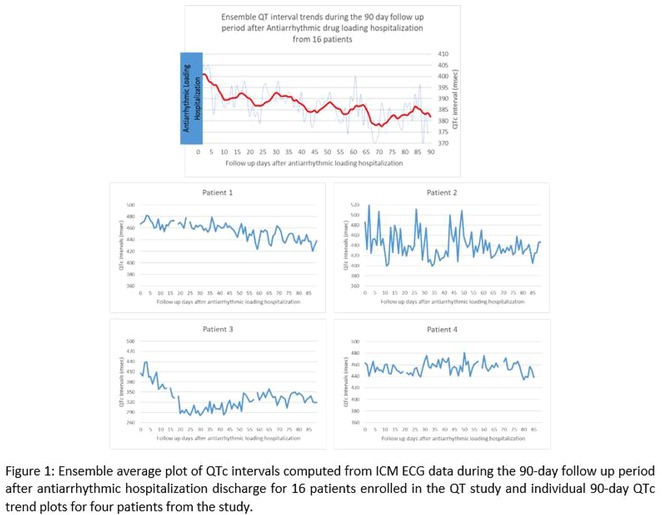

## CORRELATION BETWEEN LEFT ATRIAL WALL THICKNESS WITH RECURRENCE OF ATRIAL ARRHYTMIA IN POST ATRIAL FIBRRILLATION ABLATION

### **FRIDYAN RATNASARI** ^1^, CELLY A. ATMADIKOESOEMAH^2^, SUNU BUDHI RAHARJO^2^

#### ^1^Universitas Indonesia, Jakarta, Indonesia,^2^Departemen Kardiologi dan Kedokteran Vaskular FK Universitas Indonesia/RS Jantung dan Pembuluh Darah Harapan Kita, Jakarta, Indonesia

**Introduction:** Atrial fibrillation (AF) is the most common arrhythmia and its prevalence increases with age. Management of catheter ablation is effective in reducing symptom burden but its recurrence rate is still considered high (10‐30%) with unclear mechanism. Atrial remodeling plays important role in AF progression. The left atrial wall thickness (LAWT) is heterogenous which cause different propagation and penetration of ablation power. Recurrence of arrhythmia atrial after catheter ablation for atrial fibrillation (AF) has been considered as a common phenomenon but its mechanism and implication in long‐term outcome has not been fully understood. We aimed to clarify the relation between left atrial wall thickness and arrhythmia atrial recurences after ablation.

**Methods:** A total of 127 patients with history of catheter ablation for AF were consecutively recruited from period of January 2018‐ January 2023. Recurrences was defined as recurrence of atrial tachyarrhythmia using surface electrocardiogram and Holter monitoring. LAWT was assessed using cardiac CT scan. The statistical analysis was performed to find the relationship between the left atrial wall thickness and arrhythmia atrial recurrences.

**Results:** From 127 patients post catheter ablation for AF, patients included mean age of 55 years old and 64% patients are male. Based on type of AF, most of patients (65%) are paroxysmal AF. The mean left atrial wall thickness (OR 1,56; IK 95% 1,20‐2,03; p value &lt 0,001) and diameter of left atrium from echocardiography (OR 1,07; IK 95% 1,00‐1,14; p value 0,0038) were significantly associated with arrhythmia atrial post catheter ablation.

**Conclusions:** The left atrial wall thickness and diameter assessed with CT scan could predict atrial arrhythmias recurrences post AF ablation.

## THE DEATHLY 'HEART'QUAKE

### MOHD HAFIZ IZZUDDIN RIDZUAN

#### Hospital Sultanah Bahiyah, Alor Setar, Kedah, Malaysia

**Introduction:** 40 years old gentleman with no underlying co‐morbid. Presented with palpitations, shortness of breath with worsening failure symptoms.

ECG noted bigeminy PVC's with couplets for most of the time and the echocardiography(ECHO) showed reduced ejection fraction about 20 %, with biventricular dyssynchrony. Cardiac MRI showed, all myocardial segments are viable.

After reviewing the results, we concluded this patient is likely to suffer from tachycardia induced cardiomyopathy.

A appointment date were given to the patient for Coronary Angiogram and Electrophysiology Study (EPS) and proceed with radiofrequency ablation with 3D mapping.

**Methods:** Coronary angiogram showed normal coronaries. Hence electrophysiology study (EPS) with 3D mapping system was conducted. Using the 3D mapping system, we were able to locate the origin of the PVC's. After we get enough data, we finally concluded the area of interest is around anterior septal of RVOT. Hence ablation started using Radio Frequency Ablation (RFA). After a few times ablation, the PVC's was completely gone. Hence we ended the procedure. Post procedure no any tachycardia was inducible.

**Results:** Patient claimed the symptoms was improved right after the procedure was done. The echocardiogram and ECG was done the next day after the procedures and the ECG showed normal sinus rhythm. The echocardiogram results showed that the LV contraction had become synchronize despite poor LVEF (20 %). Patient was discharged home without any medication and were given six weeks appointment to assess symptoms and repeat ECHO.

After six weeks, the patient came to our centre. The ECG showed normal sinus rhythm and the LVEF has improved to 50% with normal chambers size and the symptoms markedly improved.After six months, the patient asymptomatic and the LVEF improved to 70 % and the ECG remained in sinus rhythm. Hence the patient was discharged from our centre.

**Conclusions:** Based on this case, we proved that frequent PVC's can lead to non ischemic dilated cardiomyopathy; by ablating the PVC's, the LVEF can return to near normal. As a conclusion, this a classical case of tachycardia induced cardiomyopathy, with successful ablation, patient LVEF can return to normal without any anti‐failure medications.

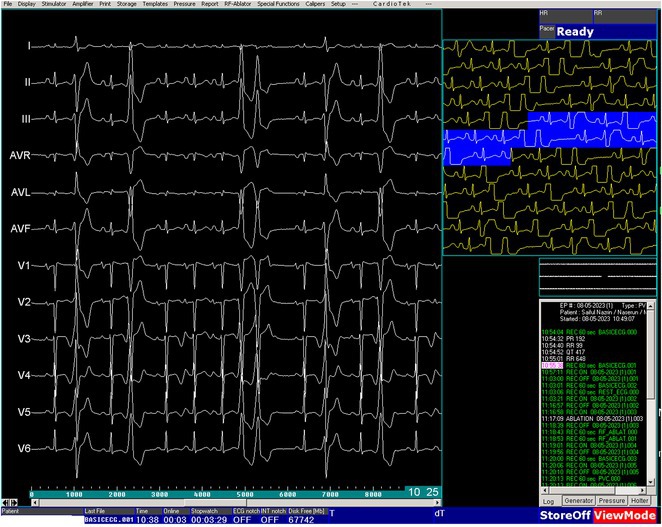

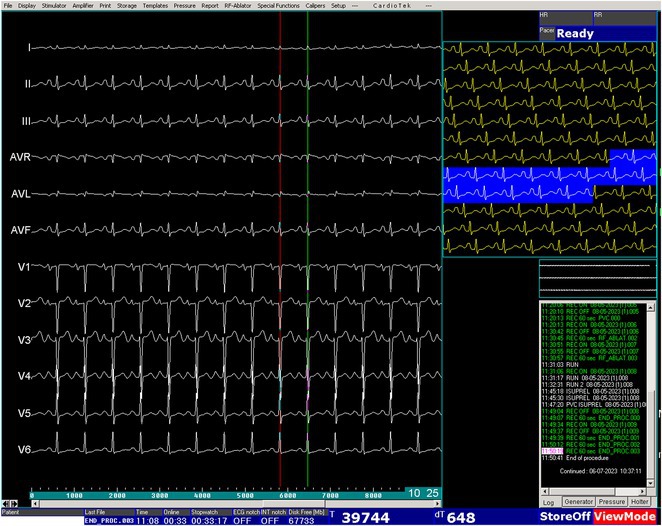

## ELECTROPHYSIOLOGIC CHARACTERISTICS OF PATIENT WITH ACCESSORY PATHWAY & ATRIAL FIBRILLATION

### CHANDARA RITH

#### Calmette hospital, Phnom Penh, Cambodia

**Introduction:** To compare the electrophysiology characteristics in patients with accessory pathway (AP) & AF

**Methods:** All patients who 1‐ encountered successful radiofrequency ablation of the accessory pathway, 2‐ age between 30‐60 years old, 3‐ sexes, 4‐ location of accessory pathway were included in the study. Exclusion criteria included 1‐ Benign WPW pattern, 2‐ Bystander AP, 3‐ inducible tachycardia after ablation.

**Results:** The correlation between AVRT with AF & patient characteristics.The correlation AVRT with AF & AP location. Manifest & concealed accessory pathway.Cross sectional analysis

**Conclusions:** In our cases study, showed that spontaneous AF occurs in approximately 24.6% of patients with AVRT. The location of accessory pathways was not critical in the pathogenesis of AF. However, middle‐aged, male sex & manifest accessory pathways were associated with an increased risk of clinical AF. For prevention of recurrent arrhythmias, the definitive treatment for preexcited AF in WPW syndrome is radiofrequency ablation.

## GETTING HEADACHE WHEN DIAGNOSING PALPITATION, HOW WE BOUNCE BACK?

### **RIZQON ROHMATUSSADELI**, PIPIN ARDHIANTO, ARUMAN YUDANTO ARIBOWO BINARSO MOCHTAR, EFFENDI TAN, DAVID JONATHAN PASIRERON

#### Diponegoro University, Semarang, Indonesia

**Introduction:** Paroxysmal SVT with sudden onset and termination is relatively common. In a large cohort of patients with symptomatic paroxysmal SVT referred for ablation, AVNRT was the most common mechanism. Initial screening using wave patterns and algorithms by ECG can help to aim a specific diagnosis of SVT before an EP Study (EPS) is carried out. Revealing diagnosis of atypical AVNRT, orthodromic AVRT through a concealed AP or AT is challenging due to similar findings during RV pacing maneuver. Here we were presenting a successfully management using 2D ablation in patient with fast‐slow AVNRT.

**Methods:** N/A

**Results:** A 57‐year‐old female patient presented with palpitations and had a documented SVT during visit to the EMS. The ECG showed narrow complex tachycardia with long R‐P interval and inverted P waves in the inferior leads. This initial finding put AVRT as a diagnosis with differential diagnosis of atypical AVNRT and AT. An EPS was performed, revealing normal basic intervals. During the retrograde maneuver, we found a retrograde block at 510 ms, indicating poor retrograde conduction. We administered infusion of isoproterenol to the patient. An atrial extra stimulation maneuver at 320 ms was performed, successfully inducing tachycardia with no AH Jump. We successfully excluded the possibility of orthodromic AVRT with findings of more than *one beat to follow*, *no reset phenomenon*, long period of PPI ‐ TCL and pseudo VAAV. We positioned the catheter in the slow pathway and measured the earliest activation of the atrium, obtaining a result of ‐64 ms. Based on the results of several EPS maneuvers we performed, we concluded that the patient's tachycardia is more likely to be atypical AVNRT rather than AT. We performed ablation on the slow pathway. After observation, programmed and burst atrial pacing could not induce any form of tachycardia. A follow‐up after a month showed no episodes of palpitations.

**Conclusions:** By applying the EPS maneuver carefully, it has been proven to be effective in helping to lead to the correct diagnosis of paroxysmal SVT. This certainly helps us in determining the appropriate ablation location to reduce morbidity and improve the patient's quality of life, measured twice cut once.

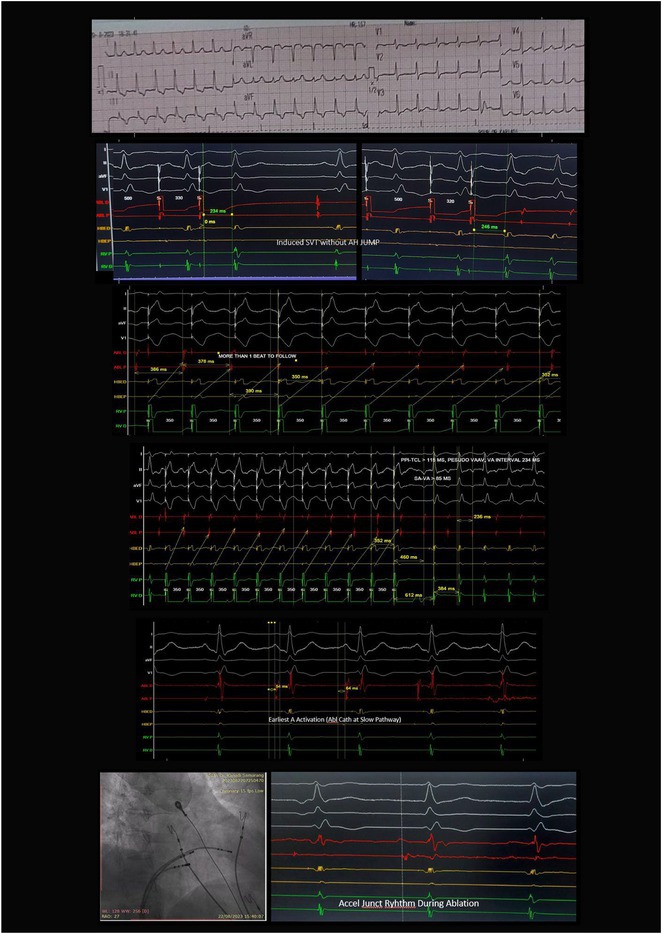

## PULSED FIELD ABALTION PREVENTS LEFT ATRIAL RESTRICTIVE PHYSIOLOGY AFTER POSTERIOR WALL ISOLATION IN PATIENTS WITH PERSISTENT ATRIAL FIBRILLATION

### ARIEL BANAI, EHUD CHORIN, ARIE LORIN SCHWARTZ, YUVAL LEVI, HEND SLIMAN, OMRI FEDER, DANA VISKIN, SAMI VISKIN, SHMUEL BANAI, **RAPHAEL ROSSO**

#### Tel Aviv Medical Center, Tel Aviv, Israel

**Introduction:** Patients undergoing radiofrequency ablation (RF) for persistent atrial fibrillation (AF) have higher risks of recurrence when the procedure consists only of pulmonary vein isolation (PVI). More extensive ablation, including posterior wall isolation (PWI) of the left atrium (LA) is often advocated. However, extensive thermal ablation of the posterior wall may cause fibrosis and “stiff‐left‐atrium” syndrome.

**Methods:** We studied 32 consecutive patients (73.5±9.9 years old, 20 males) undergoing PVI and PWI using pulsed filed ablation (PFA) for persistent AF. Echocardiographic assessment of LA strain was performed before the procedure and 1 day and 3 months later. The ablation procedure was performed using the FARAPULSE PFA system (Boston Scientific).

**Results:** PVI and PWI were accomplished in all patients without complications (Figure 1A,B). Among 15 patients in sinus rhythm at the time of ablation, LA strain, active emptying and expansion indexes significantly declined at day 1 after the ablation but returned to baseline values by 3 months. For the entire study‐group, all measurements of LA function significantly improved after 3 months (Figure 1C‐H). At 3 months, 26 (81.8%) patients were free of AF recurrence; 3 patients underwent redo‐ablation after 4‐6 months and persistent PVI and PWI were confirmed in all.

**Conclusions:** Contrary to findings in existing literature that report a permanent diminishment in LA function and compliance following RF ablation, our study presents novel evidence indicating that the impairments in LA compliance and mechanical performance following PFA with PWI are temporary.

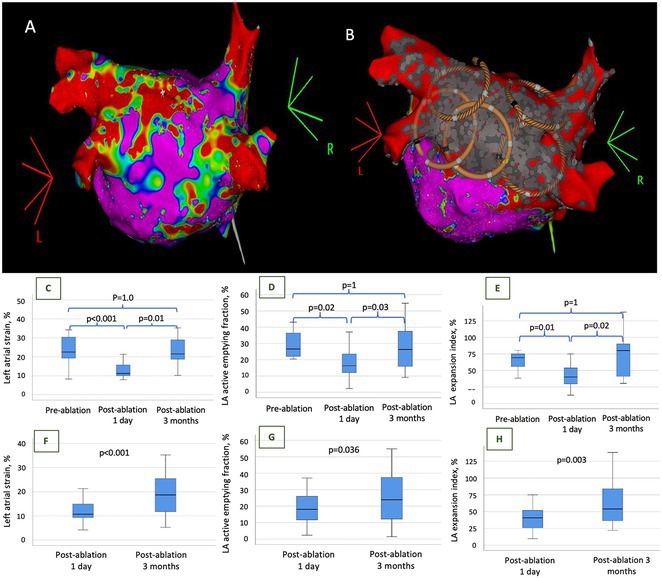

## EFFECTS OF SPINAL METAL RODS ON PULSED FIELD ENERGY DELIVERY IN A PATIENT UNDERGOING AF ABLATION: INSIGHTS FROM FINITE ELEMENT MODELING

### **TIMOTHY RYAN** ^1^, WAHEED AHMAD^1^, ARASH ARYANA^2^, DORIN PANESCU^3^, PAUL GOULD^1^

#### ^1^Princess Alexandra Hospital, Brisbane, Australia,^2^Cardiac Arrhythmia Service, Mercy General Hospital and Dignity Health Heart and Vascular Institute, Sacramento, CA,^3^CRC EP, Inc., Tustin, CA

**Introduction:** Pulsed field ablation (PFA) is a non‐thermal ablative strategy that achieves cell death via electroporation. The aim of RESET‐AF, a prospective, first‐in‐human clinical study, was to evaluate the efficacy and safety of PVI in patients with AF using a novel, QRS‐gated PFA system (CRC EP) delivering bipolar, biphasic electric fields (&gt;2kV) through a spiral, 8.4‐Fr, 16‐electrode mapping/ablation catheter (ElePulse, CRC EP).

**Methods:** A female subject with previously implanted spinal metal rods (age: 63 y, LA diameter: 45 mm) was enrolled in RESET‐AF. She underwent conventional PVI using the ElePulse PFA system as per study protocol.

**Results:** Acute PVI was achieved, and the procedure was unremarkable. However, 3D mapping post‐PFA revealed unusually wide‐area PVI that included much of the posterior wall (PW) acutely (Panels A, B) and again at a 3‐month re‐mapping study (Panel C). Thus, ‘acute stunning’ was ruled out, while other causes for persistence of the unexpected PW isolation were explored through finite element modeling. The latter suggested that the stray capacitive coupling between the metal rods and the output of the PFA generator likely contributed synergistically to an overexpanded ablation field (Panel D). It is postulated that in this case the metal rods acted as a virtual ground due to the stray capacitive coupling. Consequently, the bipolar PFA delivery received a monopolar contribution that further expanded the ablation field. Meanwhile, the patient has remained stable and free from AF at 6‐month ECG and Holter follow‐up.

**Conclusions:** Stray capacitive coupling of metallic implants that have conductive surfaces to the PFA energy source may lead to more extensive ablation fields.

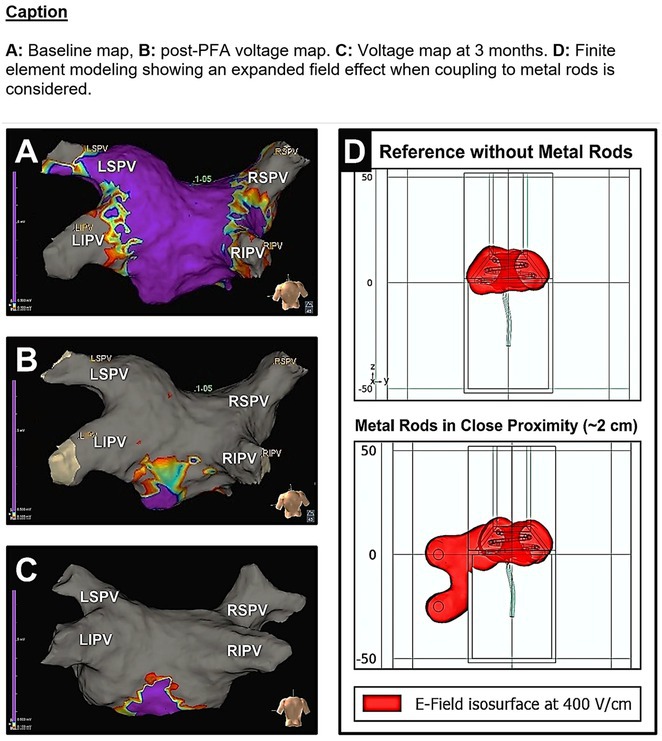

## FEASIBILITY OF PVI IN A PATIENT WITH SURGICALLY‐LIGATED PULMONARY VEINS USING A NOVEL PULSED FIELD ABLATION SYSTEM

### **TIMOTHY RYAN** ^1^, WAHEED AHMAD^1^, ARASH ARYANA^2^, DORIN PANESCU^3^, PAUL GOULD^1^

#### ^1^Princess Alexandra Hospital, Brisbane, Australia,^2^Cardiac Arrhythmia Service, Mercy General Hospital and Dignity Health Heart and Vascular Institute, Sacramento, CA,^3^CRC EP, Inc., Tustin, CA

**Introduction:** Pulsed field ablation (PFA) is a non‐thermal form of ablation that uses electrical fields to ablate cardiac tissue. The first‐in‐human RESET‐AF clinical trial evaluated the efficacy and safety of PVI in patients with AF using a novel, single‐shot (i.e. without catheter repositioning), QRS‐gated PFA system (CRC EP) delivering bipolar, biphasic electric fields (@gt; 2kV) through a spiral, 8.4‐Fr, 16‐electrode mapping/ablation catheter (ElePulse, CRC EP) which does not require positioning/delivery over a guide wire.

**Methods:** A female subject (age: 45 y, LA diameter: 38 mm) with paroxysmal AF and surgically‐ligated left pulmonary veins (PV), due to a prior lobectomy, was enrolled in RESET‐AF. She underwent a routine PVI using the ElePulse PFA catheter system, as per the study protocol.

**Results:** The procedure was uncomplicated. PVI was achieved with ease including at the ligated PVs which were deemed ‘active’. Of note, the spiral design of the ElePulse PFA catheter, which does not require positioning/delivery over a guide wire, made it particularly suitable for ablation of the ligated PV ‘stumps’. Each PV, including the ligated PVs, was ablated using 2 sequential applications without catheter positioning (ablation time: 6 min, total procedure time: 106 min). Voltage mapping pre‐ versus post‐PFA revealed wide‐area PVI acutely (Panels A, B) and long‐term at a 3‐month mandatory, repeat mapping study (Panel D). The pt did well and has remained free from recurrent atrial arrhythmias at 6‐month ECG and Holter follow‐up.

**Conclusions:** Due to its spiral design and without the need for delivery over a guide wire, PFA using the ElePulse PFA catheter proved highly effective and simple at achieving acute and durable PVI in a patient with surgically‐ligated PVs.

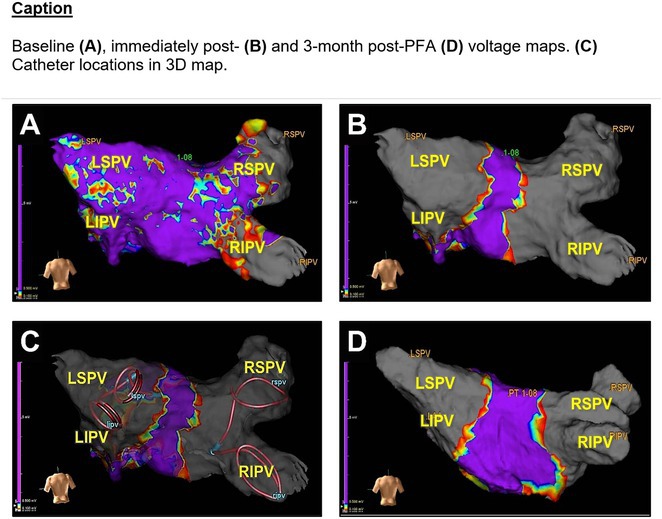

## PERCUTANEOUS IMPLANTATION OF VDD LEADLESS PACEMAKER FOLLOWING TRICUSPID VALVE REPLACEMENT: A CASE REPORT

### **TIMOTHY RYAN**, WAHEED AHMAD, KIERAN DAUBER, PAUL GOULD, JORDAN ROSS, NEELABH SHARMA, REZA REYALDEEN

#### Princess Alexandra Hospital, Brisbane, Australia

**Introduction:** Leadless pacemakers offer advantages over conventional transvenous systems including significantly lower rates of cardiac implantable electronic device infection and the avoidance of leads traversing the tricuspid valve. These attributes make them appealing pacing solutions, particularly for patients with tricuspid valve replacement (TVR). However, literature regarding the placement of leadless pacemakers in patients with TVR remains limited.

**Methods:** A female patient (age 23) presented with pocket infection of her left‐sided infra‐clavicular permanent pacemaker (right atrial transvenous lead, left ventricular epicardial surgically placed lead) implanted 12 months prior, for the management of complete atrioventricular block. The patient's conduction disease occurred as a consequence of methicillin‐sensitive Staphylococcus aureus infective endocarditis of the native tricuspid valve, necessitating surgical intervention with bioprosthetic TVR. Extraction of the existing system was planned and prior to this a VDD leadless pacemaker (Micra AV; Medtronic, Minneapolis) was successfully implanted in the right ventricle, guided by transesophageal echocardiography and fluoroscopy.

**Results:** The was no procedural complication. Post‐procedure transthoracic echocardiography demonstrated no exacerbation of tricuspid regurgitation. There was adequate sensing of atrial activity by the leadless pacemaker. To our knowledge, this marks the smallest TVR to successfully crossed with a leadless pacemaker.

**Conclusions:** Implantation of a VDD leadless pacemaker across a 27mm bioprosthetic TVR is feasible without compromising valve function.

## AN EARLY APPROACH TO MONITORED ANAESTHESIA CARE PROTOCOL WITH INTRAVENOUS PROPOFOL AS AN ALTERNATIVE TO GA FOR PULSE FIELD ABLATION OF ATRIAL FIBRILLATION

### **NIRPAL SACHDEV**, SURAYA HANI K, ROHITH STANISLAUS, MATHAN MOHAN, LOW MY, SURINDER K, AZLAN HUSSIN

#### National Heart Institute, Kuala Lumpur, Malaysia

**Introduction:** Pulsed‐field ablation (PFA) for Atrial Fibrillation (AF) has been gaining popularity. The majority of these procedures in our centre were performed using general anesthesia (GA). However, due to the risks associated with GA; we report our early experience of deep sedation with propofol in monitored anaesthesia care (MAC) as an alternative. All cases were performed using the Boston Scientific Farapulse ablation system.

**Methods:** Our initial sedation protocol involves the intravenous administration of fentanyl (1‐1.5 mcg/kg) and midazolam (2 mg) at low doses before local anesthesia with lignocaine. Subsequent, maintainance of MAC was done using propofol infusion and titrated with targeted control infusion (TCI) module. Additional doses of fentanyl 25mcg was given during each ablation of pulmonary vein while oxygen was administered via a nasal cannula of 3‐5L/min throughout the procedure.

**Results:** 5 patients were enrolled in this study (mean age = 64.2 [58‐74]). Our patients were all male, with mean body mass index = 29.05 ± 5 kg/m2. Patients enrolled had an EF greater than 55%. Mean sedation time was 50±5 min. Our finding demonstrates a mean fluoroscopy time of 17.54[10‐25] min, while skin‐to‐skin time of 42[35‐60] min which was comparable to that done under GA. A satisfactory Ramsey Sedation Scale with TCI propofol was achieved in all patients. However, two patients developed cough or hiccup during ablation. Transient hypotension was seen in two patients as well and was corrected by fluids and small boluses of vasopressor. Oxygen saturation was maintained in all patients during procedure. Any mild hypoxemia with saturation of less than 90% was easily corrected by jaw thrust. Non‐invasive ventilation or tracheal intubation was not required for any of the recruited patients. Both patient and operator were satisfied with the level of anaesthesia provided. No major procedure nor anesthesia‐related complications were reported.

**Conclusions:** In conclusion, our study protocol with TCI propofol is safe and as effective as GA with optimal patients’ and operators’ satisfaction.

## LONG‐TERM FOLLOW‐UP OF PATIENTS WITH NON‐PAROXYSMAL ATRIAL FIBRILLATION UNDERGOING CRYOBALLOON‐BASED ABLATION:A PROPENSITY SCORE‐MATCHED COMPARISON BETWEEN PULMONARY VEIN ISOLATION COMBINED WITH EITHER LEFT ATRIAL ROOF LINE ABLATION OR LEFT ATRIAL POSTERIOR WALL ISOLATION

### **YUICHIRO SAGAWA** ^1^, YASUTERU YAMAUCHI^1^, KAZUYA MURATA^1^, HIROFUMI ARAI^1^, ATSUHITO ODA^1^, YUMI YASUI^1^, TETSUO SASANO^2^

#### ^1^Japan Red Cross Yokohama City Bay Hospital, Yokohama, Japan,^2^Tokyo Medical and Dental University Hospital, Tokyo, Japan

**Introduction:** It is unknown whether LA posterior wall isolation (LAPWI) combined with pulmonary vein isolation (PVI) using a cryoballoon provides better outcomes than left atrial roof ablation (LARA) combined with PVI in patients with non‐paroxysmal atrial fibrillation (non‐PAF). The purpose of this study was to compare clinical outcomes after PVI+LARA versus PVI+LAPWI.

**Methods:** 206 patients [68 (58‐75) years; 50 female] with non‐PAF undergoing cryoballoon‐based ablation were included. In this study, the cryoapplications were delivered at pulmonary veins and LA‐roof area in the both groups. LA‐bottom block line was created by radiofrequency ablation in PVI+LAPWI group. The AF‐free survival rate was compared betweenPVI+LARA and PVI+LAPWI groups using propensity score analyses.

**Results:** We selected 52 patients treated with PVI+LARA and 52 patients treated with PVI+LAPWI using one‐to‐one propensity score matching. AF‐free survival rate was comparable in the both groups during a median follow‐up of 455 days (PVI+LARA group; 82% at 12 months, PVI+LAPWI group; 84% at 12 months, log rank P = 0.822). In multivariate analysis before propensity score matching, female sex (hazard ratio [HR], 1.97; 95% CI, 1.05‐3.71; P = 0.035), and AF duration (HR, 1.01; 95% CI, 1.00‐1.02; P = 0.020) persisted as significant predictors of ablation outcome.

**Conclusions:** In non‐PAF patients, LARA combined with PVI using a cryoballoon might provide outcomes comparable to LAPWI combined with PVI after cryoballoon‐based ablation.

## FAST BROAD IRREGULAR TACHYCARDIA: NOT ALWAYS PRE‐EXCITED ATRIAL FIBRILLATION

### DALJEET SAGGU

#### AIG Hospitals, Hyderabad, India

**Introduction:** Any electrocardiogram (ECG) displaying fast broad irregular tachycardia (FBIT) the diagnosis considered is atrial fibrillation (AF) with aberrancy or pre‐excited AF. Ventricular tachycardia (VT) has been classically described as wide complex regular tachycardia. We report a series of 7 cases of FBIT, which were proven to be VT due to myocardial inflammation.

**Methods:** Seven patients of inflammatory cardiomyopathy with proven VT presented with FBIT. Tachycardia cycle length (TCL) variation was defined as difference between longest R‐R interval and shortest R‐R interval of more than 50 ms.

**Results:** Mean age was 39.85 ± 9.8 years and 3 were male. These patients presented with clinically stable FBIT (Figure 1). None of them had evidence of pre‐excitation on baseline ECG or VT morphology mimicking baseline ECG. All of them were confirmed to have an underlying myocardial inflammation (Cardiac sarcoidosis= 5; Cardiac Tuberculosis—1, Inflammatory myocarditis‐1). Out of 7, 4 cases underwent catheter ablation for VT storm. In all these cases, FBIT was confirmed to be VT fulfilling standard criteria during electrophysiology study. In none of these cases, AF was induced in the EP lab.

**Conclusions:** VT should also be considered in the differential diagnosis of FBIT and clinicians should maintain a high index of suspicion for irregular VT in patients with wide complex irregular tachycardias, especially when typical AF is not evident. Timely diagnosis and appropriate management are essential for improving patient outcomes in such cases.

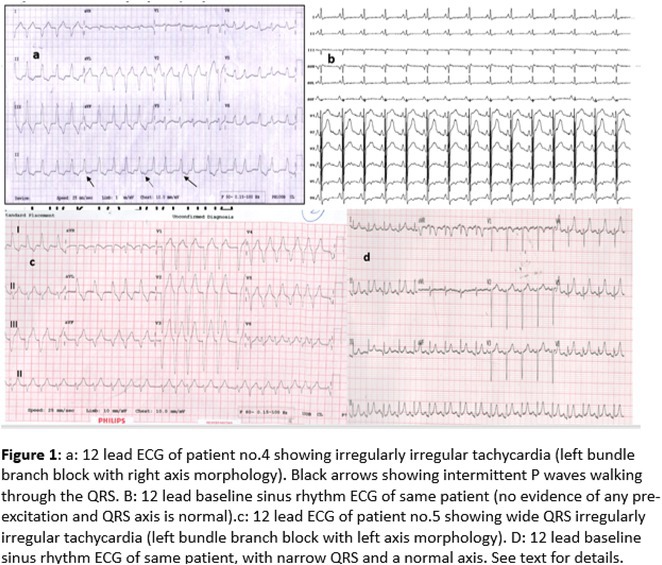

## OMNIPOLAR MAPPING TECHNOLOGY FOR CATHETER ABLATION OF ATRIOVENTRICULAR ACCESSORY PATHWAY

### **IKUTA SAITO**, KENTARO MINAMI

#### Dokkyo Medical University, Mibu, Tochigi, Japan

**Introduction:** The conventional mapping approach for the atrioventricular accessory pathway (AP) involves point‐by‐point mapping to identify the AP's connection sites to the atria or ventricle. However, this approach requires an accurate interpretation of local electrograms. Omnipolar mapping technology (OMT) explains how vector and wave speed are produced using unipolar and bipolar signals to obtain omnipolar signals, directions, and conduction velocity. This study aims to verify the effectiveness of OMT for catheter ablation of AP.

**Methods:** From January 2018 to December 2023, 68 patients who underwent catheter ablation of APs were enrolled. 35 patients (OMT group) received high‐resolution omnipolar mapping, while 33 patients underwent radiofrequency ablation with a conventional approach (conventional group). The background characteristics and procedural details of the two groups were compared.

**Results:** Acute success was obtained in all patients. Any arrhythmia recurrence was observed in one patient in the OM group and three in the conventional group (p = 0.0501). In the OM group, AP elimination by the first RF applications (77.1% vs 48.4%, p = 0.0143), the number of RF applications for eliminating AP (median [IQR]; 1.1 [1.0‐3.0] vs 4.4 [1.0‐7.0], p = 0.0012), procedure time (median [IQR], min; 80.1 [72.2‐92.7] vs 112.0 [95.1‐125.4], p < 0.01), fluoroscopy time (median [IQR], min; 12.0 [9.5‐15.2] vs 19.8 [13.6‐28.1], p < 0.01), and fluoroscopy dose (median [IQR], mGy; 60.9 [45.0‐83.5] vs 129.0 [80.5‐360.2], p < 0.01) were significantly lower than in the conventional group.

**Conclusions:** The Omnipolar mapping technology was useful for ablating accessory pathways and reducing the number of RF applications and radiation exposure compared to conventional mapping approaches.

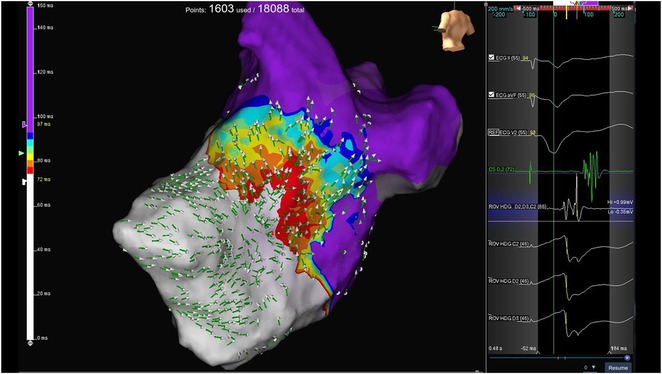

## TPEAK TO TEND INTERVAL TO QT INTERVAL RATIO MAY BE A USEFUL INDICATOR OF CHANGES IN VENTRICULAR SYMPATHOEXCITATION IN HUMAN WITHOUT ORGANIC HEART DISEASE

### **SHOTA SAITO**, TARO TEMMA, KEI KAWAKAMI, MASAHIRO KAWASAKI, KINTARO SHIMANO, JIRO KOYA, DAISHIRO TATSUTA, KOTARO NISHINO, HIROYUKI NATSUI, TAKAHIDE KADOSAKA, MOTOKI NAKAO, TOSHIHISA ANZAI

#### Department of Cardiovascular Medicine, Faculty of Medicine and Graduate School of Medicine, Hokkaido University, Sapporo, Japan

**Introduction:** Transmural dispersion of repolarization (TDR) in the myocardium is reflected by Tpeak to Tend interval (Tp‐Te). Sympathetic innervation of the myocardium extends from the epicardium to the endocardium, and this may contribute to TDR. We hypothesized that Tp‐Te may be a useful marker of cardiac sympathoexcitation if TDR variability is caused by differences in myocardial sympathetic distribution and associated activity.

**Methods:** We analyzed patients who underwent cardiopulmonary exercise testing (CPET) between 2013 and 2023, and patients treated premature ventricular contractions (PVCs) and paroxysmal supraventricular tachycardias (PSVTs) by catheter ablation (ABL) between 2017 and 2022. We measured ECG parameters before and 1 minute after maximal exercise (1‐min recovery) in CPET, and pre and post isoproterenol (ISO) injection in ABL. ECG parameters were analyzed on V5.

**Results:** In CPET study, 45 cases without organic heart disease were included (female: 35.6%; baseline age: 51 [IQR 42‐66]). Tp‐Te, Tp‐Te to QT interval ratio (Tp‐Te/QT), heart rate (HR), activation recovery interval (ARI), ARI to QT interval ratio (ARI/QT) and QT interval (QT) were different between pre and 1‐min recovery (all p< 0.05). The degree of difference of Tp‐Te/QT between pre and 1‐minute recovery was highest compared to other parameters (p< 0.001). In ABL study, 55 cases without organic heart disease were included (female: 63.6%; baseline age: 55 [IQR 42‐68]). Tp‐Te, Tp‐Te/QT, HR, ARI and QT were different between pre and post ISO infusion (all p< 0.05). The degree of difference of Tp‐Te/QT was also highest compared to other parameters (p< 0.001). In both exercise and ISO infusion, the degree of difference of Tp‐Te / QT was highest even considering sex compared to other parameters (p< 0.001), and there was no correlation between left ventricular wall thickness and Tp‐Te/QT.

**Conclusions:** Tp‐Te/QT varies during sympathoexcitation by exercise and ISO infusion independent of sex and left ventricular wall thickness. Tp‐Te/QT may be a useful marker of ventricular sympathoexcitation without organic heart disease.

## ACCESSORY PATHWAYS ARE NOT ALWAYS SINGULAR

### **YUJI SAITO**, KOICHI NAGASHIMA, SHU HIRATA, RYUTA WATANABE, YUJI WAKAMATSU, MOYURU HIRATA, MASANARU SAWADA, SAYAKA KUROKAWA, YASUO OKUMURA

#### Nihon University School of Medicine, Itabashi‐ku, Tokyo, Japan

**Introduction:** Orthodromic reciprocating tachycardia (ORT) in the setting of the 2 accessory pathways (AP) are rare but needs careful interpretation.

**Methods:** N/A

**Results:** A 52‐year‐old woman underwent the electrophysiologic study for supraventricular tachycardia. After positioning the multielectrode catheters into the high right atrium, His bundle region (HBE), coronary sinus (CS), and right ventricle (RV), we initiated the electrophysiological study. During RV overdrive pacing, the earliest activation site (EAAS) was identified at the proximal CS (pCS). By decreasing the coupling interval of the RV extrastimulus from 370 ms to 270 ms, with a basic pacing cycle length of 600 ms, a double atrial response was seen; the EAAS in the first atrial sequence was identical to that during RV overdrive pacing and lacked decremental property, and the EAAS in the second sequence was seen in HBE (Figure A). By further reduction of the coupling interval of the RV extrastimulus (260 ms), the EAAS was shift to the distal CS (dCS). A narrow QRS tachycardia with the tachycardia cycle length (TCL) of 242 ms, and the ventriculo‐atrial interval of 62 ms was induced. The atrial sequence during tachycardia exhibited the reverse chevron type atrial sequence with the EAAS at both the pCS and dCS. His‐refractory LV extrastimulus advanced the next atrial electrogram and terminated the tachycardia, which was diagnostic of ORT. A scanned single atrial extrastimulus from the EAAS in left lateral atrium failed to reset the tachycardia, indicating the left lateral AP was bystander (Figure B). Further decreasing of coupling interval of the scanned single atrial extrastimulus advanced the EAAS but failed to reset the tachycardia, indicating the atrium was not involved in the tachycardia circuit. Given those findings, we diagnosed ORT via a nodoventricular pathway connecting to the left inferior extension with a bystander left lateral AP.

**Conclusions:** Reverse chevron atrial sequence during tachycardia may be first step toward recognizing multiple AP.

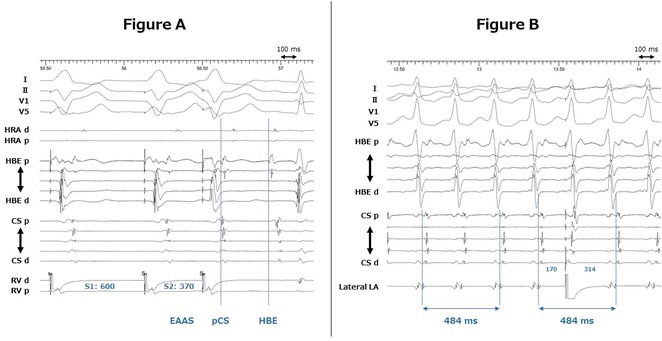

## EXCITATION RECOVERABLE MYOCARDIUM AROUND DURABLE RADIOFREQUENCY (RF) LESIONS; CORRELATION BETWEEN THE DISTRIBUTION OF PACING THRESHOLD AND GROSS MYOCARDIAL COLOR CHANGES

### **OSAMU SAITOH** ^1^, TAKUMI KASAI^1^, AYAKA OIKAWA^1^, HIROKO KOBAYASHI^1^, YASUHIRO IKAMI^2^, YUKI HASEGAWA^2^, SOU OTSUKI^2^, HIROSHI FURUSHIMA^1^, TAKAYUKI INOMATA^2^, MASAOMI CHINUSHI^1^

#### ^1^School of Health Sciences Faculty of Medicine, Niigata University, Niigata, Japan,^2^Department of Cardiovascular Medicine, Niigata University Graduate School of Medical and Dental Sciences, Niigata, Japan

**Introduction:** Around durable lesions, RF catheter ablation induces excitation recoverable myocardium, which can be a substrate of arrhythmia recurrence. On the other hand, volume of RF lesion is measured by gross color changes of the myocardium, but this method may include excitation recoverable myocardium surrounding durable lesions.

**Methods:** In coronary perfusing porcine hearts, pacing threshold through the ventricle was measured using eight‐pole (1 mm distance) needle electrodes vertically inserted into myocardium before, 3 and 40 min after 40 W ablation with 10‐g contact; irrigation catheter for 15 sec (G1) and for 40 sec (G2), non‐irrigation catheter for 15 sec (G3) and for 40 sec (G4), all with 12 applications each. Change in color tone of RF lesion was evaluated using Image‐J application with 256 shades of gray.

**Results:** 3 min after ablation, pacing uncaptured electrodes were distributed from the surface into the myocardium in the following order; G2=G4 (around 4.2 mm and 5 electrodes) > G1=G3 (around 2.3 mm and 3 electrodes). 40 min after ablation, excitation recovered electrodes (ERE) developed above (G1: 1.7 electrodes) and below (all groups: around 0.6 electrodes) the durable lesion. Because of the surface excitation recovery, total numbers of ERE were greater in G1 followed by the other three groups. Image‐J analysis revealed that gray scale values in the excitation recovered lesions (122±20) were in the middle between those in the durable lesions (146±18) and normal myocardium (102±15). Accordingly, volume estimated based on the gross color changes overestimated durable lesion size.

**Conclusions:** Regardless of the ablation methods, excitation recoverable myocardium was present similarly below but varied depending on the application time and irrigation flow volume above the durable lesions. Gross color changes basis assessment would include excitation recoverable myocardium and overestimated durable lesion size.

## EVALUATION OF UNUSUAL VARIANTS IN SLOW/FAST ATRIOVENTRICULAR NODAL REENTRANT TACHYCARDIA: EFFICACY AND PRACTICALITY OF A SINGLE LATE ATRIAL EXTRASTIMULUS

### **YUICHIRO SAKAMOTO**, YUKOH UEMURA, RYO YAMAGUCHI, HIROKAZU NAGANAWA, DAISUKE YOSHIMOTO, TAKAHIKO SUZUKI

#### Toyohashi Heart Center, Toyohashi, Japan

**Introduction:** We sought to determine the frequency of unusual variants of atrioventricular nodal reentrant tachycardia (AVNRT) and the effectiveness and practicality of a single late atrial extrastimulus (AES) for diagnosing them.

**Methods:** We reviewed 101 consecutive cases of slow/fast AVNRT. A single late AES was delivered at the inferior triangle Koch (Rightward inferior extension [RIE]) and 2‐3 cm distal from coronary sinus ostium (Leftward inferior extension [LIE]) during tachycardia. The site where the later AES resets tachycardia was considered to be in proximity to the slow pathway input and ablated.

**Results:** The AES study was attempted in 93 of 101 cases, with successful execution in only 58 cases, primarily due to mainly difficulty in induction and stability of tachycardia. 56 cases showed longer His‐AES interval in the RIE compared to the LIE (52±27ms vs. 21±27ms, P<0.05) and underwent ablation there. In 2 cases (1.9%), longer His‐AES interval in the LIE (19ms v.s 33ms) suggested LIE involvement, prompting an approach from left atrium. In one case, misinterpretation of the study led to unnecessary mapping and ablation from left atria. In the remaining 43 cases, empirical ablation targeting the RIE was performed. Tachycardia was eliminated in all cases without atrioventricular block or postoperative recurrence.

**Conclusions:** The unusual variant of slow/fast AVNRT was 1.9%. A single late atrial extrastimulus was effective for diagnosis; however, there were many cases in which it could not be undergone. Misinterpretation of results led to unnecessary left atrial mapping and ablation in one case.

## A CASE OF REMOVAL OF A DANCING AVEIR

### **ATSUSHI SAKATA**, HIRONOBU SUMIYOSHI, KENTA YOSHIDA, RYUKI CHATANI, MITSURU YOSHINO, HIROSHI TASAKA

#### Kurashiki Central Hospital, Kurashiki city, Japan

**Introduction:** In April 2023, Aveir VR, a new leadless spacemaker with a fixed helix and pre‐mapping function, became available in Japan. In addition, Aveir VR has a dedicated retrieval catheter using a screw‐in mechanism, and has shown an 80% device removal success rate over 7 years after implantation.

**Methods:** N/A Fifteen years ago, a DDD pacemaker was implanted on the left side for sick sinus syndrome type III. Six years ago, the patient underwent generator replacement surgery for battery depletion and was followed up as an outpatient. Lead failure was observed during outpatient follow‐up, and subclavian venography revealed occlusion of the implanted vein. We considered adding a lead from the contralateral side or a leadless pacemaker, but due to bradycardia atrial fibrillation and the risk of wire breakage with the addition of a lead, we implanted a leadless pacemaker (Aveir) and removed the generator.

**Results:** A 7Fr sheath was inserted through the right femoral vein, and right ventriculography was performed. Next, the sheath was changed to an Aveir introducer, and the Aveir catheter was used to screw in the lower septum of the right ventricle. The current of Injuly was good, impedance was 350‐380 ohms, and the threshold improved to 1.25V/0.4ms after 30 minutes, so we released the device. Although no obvious dislodgement of the device body was observed, the device body started dancing immediately after that, and non‐sustained ventricular tachycardia occurred frequently and was difficult to control, so a retrieval tool was used to remove it.Aveir was deployed apex successfully during the same procedure with optimal electrical values.

**Conclusions:** We report a case in which a leadless pacemaker, Aveir, was removed and redeployed due to the apperance of NSVT after insertion.

## VENTRICULAR TACHYCARDIA AND BETA 2 AGONIST‐ASSOCIATION: UNDERSTANDING THE RISK

### **DEWI SAKINAH**, MUZAKKIR AMIR, ARISAL ARISAL

#### Hasanuddin University, Makassar, Indonesia

**Introduction:** The occurrence of ventricular tachycardia is believed to be associated with the use of Beta‐2 agonist drugs. In the following case report, we describe a patient with electrocardiogram (ECG)‐confirmed Ventricular Tachycardia who has a history of Beta‐2 agonist drug use.

**Methods:** N/A

**Results:** A 68‐year‐old male presented with chest discomfort lasting for 5 days, characterized by a heavy, pressing sensation lasting over 20 minutes, cold sweats, and pain radiating to the left arm and shoulder (VAS 9/10). He had a history of intermittent chest pain over the past week, particularly during physical activity, and continuous palpitations for the past 2 weeks, with no previous history of palpitations.Vital signs included a blood pressure of 140/90 mmHg, heart rate of 152 beats per minute, respiratory rate of 20 breaths per minute, and oxygen saturation of 85%, which improved to 97% with nasal cannula oxygen. His temperature was 36.5°C. Electrocardiography (ECG) showed tachycardia with a wide QRS complex, extreme axis deviation, rSR' pattern in V1 with R/S >1, a wide slurred S wave in lateral leads (I, aVL, V5‐6), and AV dissociation. The ECG showed monomorphic VT with RBBB morphology. The patient has a history of coronary artery disease with three‐vessel disease (CAD 3 VD) and has undergone coronary artery bypass grafting. He is also known to suffer from bronchial asthma and is using a Beta‐2 agonist inhaler, specifically salbutamol. The ECG examination revealed monomorphic ventricular tachycardia. During his hospital stay, the patient underwent ablation and implantable cardioverter‐defibrillator (ICD) implantation as therapeutic measures for ventricular tachycardia.

**Conclusions:** This case of ventricular tachycardia is likely due to myocardial scarring from previous coronary artery disease, as confirmed by the patient's history of coronary angiography and CABG surgery. The condition may have been worsened by the use of Beta‐2 agonist medications for asthma control. These medications can have pro‐arrhythmic side effects due to their effects on heart rate, electrical activity of the heart, and cellular potassium distribution mediated through β‐adrenergic receptors.

## BRUGADA SYNDROME : MASTER OF MULTIPLE FACIES

### DOREEN SUMPAT^1^, IMRAN ZAINAL ABIDIN^2^, MEXMOLLEN MARCUS^1^, **SIMON SALIM** ^3^

#### ^1^Universiti Malaysia Sabah, Kota Kinabalu, Malaysia,^2^Universiti Malaya, Kuala Lumpur, Malaysia,^3^Division of Cardiology, Department of Internal Medicine, Faculty of Medicine, Universitas Indonesia, Jakarta, Malaysia

**Introduction:** Brugada Syndrome (BrS) exhibits higher prevalence in Southeast Asia, presenting challenges in genetic characterization and displaying a wide spectrum of clinical manifestations. Hence underscoring the importance of risk stratification utilizing electrocardiogram (ECG) features. Here, we present two distinct cases, each illustrating variations in clinical presentation.

**Methods:** NA

**Results:** Mr. A, a 20‐year‐old student, came with chest pain of three days. He denied other symptoms and had no family history of sudden cardiac death (SCD). His initial ECG (a) revealed Type 1 Brugada pattern. Further investigations yielded normal results. Mr. B, a 35‐year‐old swim coach, presented with dyspnea and chest tightness for one week. He denied other symptoms and had no family history of SCD. Despite hemodynamically stable, his initial ECG (b) displayed ventricular tachycardia, necessitating synchronized cardioversion. ECG (c) post‐cardioversion revealed Type 1 Brugada pattern. Other investigations were normal.Type 1 Brugada ECG pattern is linked with a three‐to‐fourfold higher risk of cardiac events. Family history of SCD at any age does not independently predict cardiac events. Depolarization ECG markers include QRS‐fragmentation, aVR sign and a wide or large S‐wave in lead I. Repolarization ECG markers are inferolateral early repolarization patterns and prolonged QTc intervals. These markers are potent indicators for ventricular arrhythmias. In both patients, only depolarization ECG markers were observed. Mr. B's ECG displayed all the aforementioned depolarization markers.

**Conclusions:** Larger prospective studies incorporating clinical presentations, ECG findings, genetic studies, and electrophysiology mapping will provide accurate and detailed risk stratification. Such studies offer valuable insights into the interplay of various factors contributing to the risk of cardiac events in this syndrome, thereby improving clinical management strategies and outcomes.

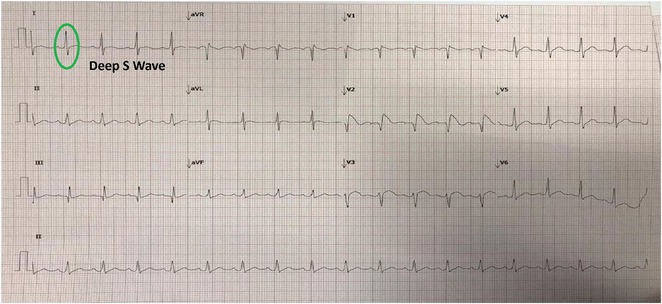

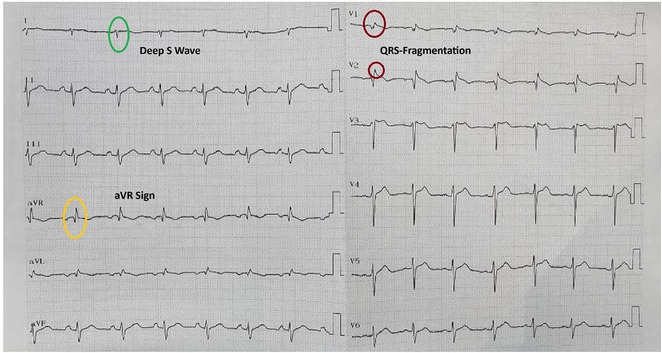

## SF36 SCORE TO PREDICT AFEQT SCORE IN INDONESIAN CULTURE

### **SIMON SALIM** ^1,2^, PUTRI ZULMIYUSRINI^1^, SUDARTO RONOATMODJO^3^, BESRAL BESRAL^3^, MUHAMMAD YAMIN^1^

#### ^1^Cipto Mangunkusumo Hospital, Jakarta, Indonesia,^2^Doctorate Candidate in Clinical Epidemiology, Faculty of Public Health, University of Indonesia, Indonesia,^3^Faculty of Public Health, University of Indonesia, Jakarta, Indonesia

**Introduction:** Health related quality of life (HRQoL) is one of the main targets in atrial fibrillation ablation. Previous study showed AFEQT <70 gain the most benefit to ablation (1). Our group had successfully translated and validated SF36 and AFEQT in Indonesian culture (2,3). SF36 had broader distribution than AFEQT and widely used in atrial fibrillation patients.

**Objective:** To assess what cut off can be used in SF‐36 to predict lower AFEQT score (<70).

**Methods:** Atrial fibrillation patients from January to March 2023 were interviewed using SF36 and AFEQT in Integrated Cardiovascular Service unit in Cipto Mangunkusumo Hospital, Jakarta, Indonesia. SF‐36 were evaluated for its sensitivity and specificity to predict lower AFEQT overall score (<70).

**Results:** A total of 102 patients were included in the data analysis. Physical Component Summary (PCS) and Mental Component Summary (MCS) scores of SF‐36 had a discriminatory ability based on Area Under the Curve (AUC) of 0.870 and 0.856 respectively to AFEQT, with the optimal cut‐off point to differentiate AFEQT overall score <70 was 54.52 for PCS (sensitivity 0.804 and specificity 0.826) and 60.71 for MCS (sensitivity 0.875 and specificity 0.674).

**Conclusions:** Based on AUC and ROC, SF‐36 has good discriminatory ability to overall score AFEQT (<70).

**Reference: (1)** Zeitler EP, Li Y, Silverstein AP, Russo AM, Poole JE, Daniels MR, et al. Effects of Ablation Versus Drug Therapy on Quality of Life by Sex in Atrial Fibrillation: Results From the CABANA Trial. Journal of the American Heart Association. 2023 Feb 7;12(3):e027871.(2) Salim S, Yamin M, Alwi I, Setiati S. Validity and reliability of the Indonesian version of SF‐36 quality of life questionnaire on patients with permanent pacemakers. Acta Med Indones. 2017 Jan 1;49(1):10‐6.(3) Zulmiyusrini P, Yamin M, Muhadi M, Kurniawan J, Salim S. The validity and reliability of Indonesian version of atrial fibrillation effect on quality of life (AFEQT) questionnaire for atrial fibrillation patients. Journal of Patient‐Reported Outcomes. 2023 Dec;7(1):1‐0.

## REAL‐WORLD ECONOMIC IMPACT OF IMPROVED EFFICIENCY OF TACTIFLEX SE ABLATION CATHETER COMPARED TO TACTICATH SE IN THE TREATMENT OF PAROXYSMAL ATRIAL FIBRILLATION IN ASIA‐PACIFIC AND EUROPE

### **PRASHANTHAN SANDERS** ^1^, THADCHAIGENI PANCHALINGAM^2^, VERONICA SANSING‐FOSTER^2^, DANIEL STEVEN^3^, ARIAN SULTAN^3^, TAKANORI YAMAGUCHI^4^, JEFFREY WINTERFIELD^5^, MOUSSA MANSOUR^6^, ISABELLE DEISENHOFER^7^, YASUTERU YAMAUCHI^8^

#### ^1^The University of Adelaide, Royal Adelaide Hospital, Adelaide, Australia,^2^Abbott Laboratories, Los Angeles, CA,^3^Heart Center at the University of Cologne, Department of Electrophysiology, Cologne, Germany,^4^Department of Cardiovascular Medicine, Saga University, Saga, Japan,^5^Medical University of South Carolina, Division of Cardiology, Charleston, SC,^6^Massachusetts General Hospital, Harvard Medical School, Boston, MA,^7^German Heart Center Muenchen Technical University, Munich, Germany,^8^Yokohama City Minato Red Cross Hospital, Yokohama, Japan

**Introduction:** Clinical trial data show that use of the TactiFlex (TF) Ablation Catheter Sensor Enabled (SE) for the treatment of paroxysmal atrial fibrillation (PAF) ablation results in shorter procedure and ablation times compared to use of TactiCath (TC) Ablation Catheter SE. However, the time savings and resulting economic benefits of TF use in the real‐world setting are unclear. In this study, we evaluate the real‐world time and economic benefits of TF compared to TC ablation catheters for the treatment of de novo PAF.

**Methods:** We retrospectively analyzed deidentified PAF ablation procedural data from hospitals in Asia‐Pacific (Japan and Australia) and Europe (Germany, France, Italy, United Kingdom, Portugal, and Belgium) between 11/16/2021‐06/09/2023. Median ablation times (start to end of an ablation) and procedure times (skin‐to‐skin) were compared between TF and TC within each region. Net monetary benefits were quantified in Euros (2022 €) using the difference in procedure times and corresponding physician‐operator, medical technician, and Cath lab costs. The cost structures used to quantify the net benefits differed between the two regions; European analyses were based on costs from Germany, while Asia‐Pacific analyses were based on an adaptation of U.S. costs.

**Results:** Study included 118 TF/53 TC records from Asia‐Pacific (90% from Japan), and 42 TF/96 TC records from Europe (71% from Germany). Median ablation and procedure times with TF were significantly lower compared to TC by 15.0 and 29.5 minutes per procedure (p < 0.001) in Asia‐Pacific, and by 11.5 (p <0.01) and 30.5 minutes per procedure (p <0.001) in Europe. The reduced procedure time with TF yielded a median total net benefit of € 889 per procedure in Asia‐Pacific and € 406 in Europe.

**Conclusions:** In a real‐world study of PAF ablations performed in Asia‐Pacific and European hospitals, TF led to reduced procedure and ablation times compared to the previous generation catheter. This resulted in significant economic benefits.

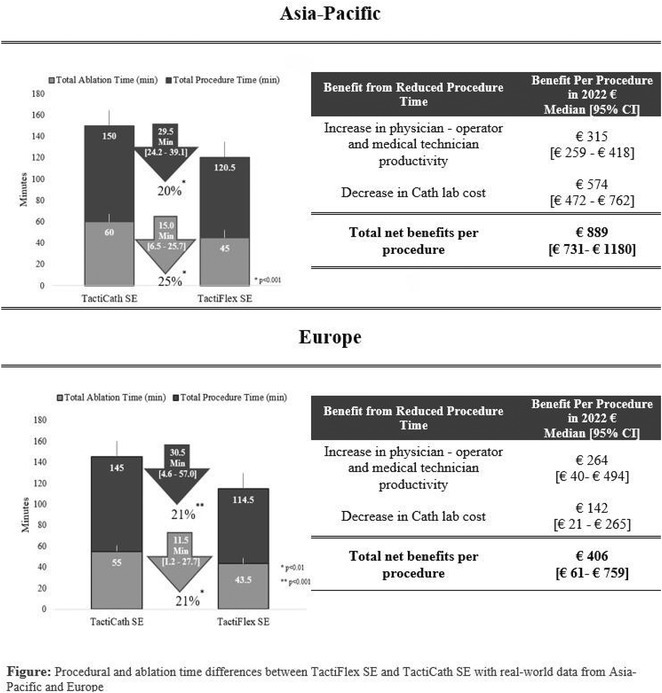

## FREQUENCY AND POSTOPERATIVE COURSE OF CARDIAC RESYNCHRONIZATION THERAPY IN THE INDICATION AS THE BLOCK HF TRIAL

### **HARUKA SASAJIMA**, SHUN NAKAMURA, MASAHIRO MORISE, NOBUHIRO NISHIYAMA, TAKAHIDE KODAMA

#### Toranomon Hospital, Minato City, Japan

**Introduction:** The results of the Block HF trial shows that biventricular pacing was superior to right ventricular (RV) pacing in the patients with left ventricular (LV) systolic dysfunction with NYHA class I‐III. The frequency of cases with Block HF indications remains unclear.

**Methods:** Screening of eligible cases was performed using magnetic resonance imaging (MRI), radio isotope (RI) as well as echocardiography. First we examine its frequency and patient backgrounds in Asian populations, and then the place of LV lead is determined with reference to interventricular delay. Pre‐ and postoperative ECG and echocardiographic parameters were evaluated and clinical follow‐up was observed.

**Results:** 19 patients received cardiac resynchronization therapy (CRT) by Block HF indication from January 2019 to March 2023 in our hospital. 12 of 109 (11%) patients with AV block, and 7 of 26 (27%) patients with bradycardic atrial fibrillation were eligible for CRT, respectively. The average age was 68.8±11.2 years, and 16 were male. 8 of the patients had an ischemic heart disease, and 4 cases were postoperative for valvular disease. LV lead was implanted at electrical delay site after seeing (RV‐paced to LV‐sensed (RVp‐LVs)). Before device implantation, the average QRS width was 137.9±28.3 msec, and the average QRS width at RV pace (QRS@RVp) was 173.8±22.6 msec. It was able to implant LV lead at the position where the time from RVp‐ LVs was 89.0±6.3% of QRS@RVp. The average QRS width after device implantation was 135.6±12.1 msec. Patients were followed for a mean of 29.4±14.3 months. There were no deaths, and only one patient presented with heart failure and was indicated for transcatheter mitral valve repair.

**Conclusions:** In Asian populations, CRT may be indicated in about 10% of AV block and 25% of bradycardic atrial fibrillation. Especially in cases with ischemic heart disease or a history of valvular disease, a close examination may be desirable. And LV lead placement with interventricular delay (RVp‐LVs) may be useful in CRT for Block HF indication.

## A CASE OF CRYOBALLOON ABLATION TO THE MITRAL ISTHMUS FOR PERI MITRAL FLUTTER RESISTANT TO RADIOFREQUENCY ABLATION

### **TAKAFUMI SASAKI**, MASAO TAKAHASHI, FUMIYA YOKOZEKI, MASANAO HONMA, MASATAKA SUNAGAWA, WATARU TSUNO, YOSHIAKI MIZUNUMA, KOICHIRO YAMAOKA, HIROFUMI KUJIRAOKA, TOMOYUKI ARAI, KIYOTAKA YOSHIDA, KENSUKE KASANO, RINTARO HOJO, TAKAAKI TSUCHIYAMA, SEIJI FUKAMIZU

#### Tokyo Metropolitan Hiroo Hospital, Shibuya, Tokyo, Japan

**Introduction:** The vein and ligament of coronary sinus are associated with arrhythmogenic roles in peri mitral flutter. It is now well known that termination peri mitral flutter and creating a complete line block in the mitral isthmus are difficult due to myocardial thickness, conductive cooling due to coronary sinus (CS) blood flow and epicardial connections. It can be technically difficult to perform bidirectional block in the mitral isthmus by only radiofrequency ablation at the endocardium and CS.

**Methods:** N/A

**Results:** The patient was a 60‐year‐old man who had undergone pulmonary vein isolation and left atrial roof line/incomplete mitral isthmus (MI) line/complex fractionated atrial electrogram ablation for persistent atrial fibrillation. His other medical history was only bronchial asthma. After ablation uncommon atrial flutter was documented, so another catheter ablation was performed. A counterclockwise peri mitral flutter (cycle lengths:310ms) through the MI and partially bridging the CS was assumed. An additional line along the previous line was performed, however, the tachycardia did not terminate, and an extra ablation of intra‐CS was ineffective. The CS angiogram showed that the Marshall vein was too small to undergo chemical ablation, so we performed cryoballoon ablation. Two cryoapplications delivered to the MI resulted in cycle length prolongation and tachycardia terminated. An additional three cryoapplications caused to achieve conduction block. Bidirectional block line of the MI was demonstrated by pacing from the left atrial appendage and CS. Compared with radiofrequency ablation, cryoballoon ablation provides better catheter stability and a more uniform lesion, and may be an option for block line creation in the MI.

**Conclusions:** We have experienced peri mitral atrial flutter seen after catheter ablation for persistent atrial fibrillation. Cryoballoon ablation can be considered when MI ablation with conventional approaches is difficult.

## FEASIBILITY AND SAFETY OF LEFT ATRIAL APPENDAGE OCCLUSIONS EARLY AFTER ATRIAL FIBRILLATION ABLATION PROCEDURES

### **TAKESHI SASAKI** ^1^, AYAKA USUI^1^, RYOTARO FUNAYAMA^1^, KYOJI HAYASHI^1^, SAYUMI NOZAKI^1^, JUNICHI DOI^1^, HAYASAKA KAZUTO^1^, SHU YAMASHITA^1^, YASUHIRO SHIRAI^2^, SHINSUKE MIYAZAKI^3^, TETSUO SASANO^3^

#### ^1^National Hospital Organization Disaster Medical Center, Tokyo, Japan,^2^AOI international Hospital, Herth Rhythm Center, Kawasaki, Japan,^3^Tokyo Science University, Cardiovasular Mdicine, Tokyo, Japan

**Introduction:** Percutaneous left atrial appendage occlusion (LAAO) is available to prevent thromboembolisms in patients with atrial fibrillation (AF), especially in patients at high‐risk of thromboembolic strokes or life‐threatening bleeding events. However, efficacy and safety of LAAO early after AF ablation remain unclear. This study aims to investigate the clinical outcomes of LAAO early after AF ablation.

**Methods:** This study consists of 96 patients (77±6 years‐old) who underwent LAAO with WATCHMAN devices after AF ablation. LAAO early after AF ablation were defined as LAAO within 30 days after ablation procedures. Patient characteristics, procedure outcomes including procedure‐relate complications and long‐term outcomes of LAAO early after AF ablation were investigated.

**Results:** A total of 45 patients (46.9%) received LAAO in 6.9±4.4 [3‐29] days after AF ablation procedures. LAAO devices (WATCHMAN 2.5; 8 patients, FLX 37 patients) were successfully implanted in all patients without complications after 1.8±1.2 ablation procedures. The mean procedure time was 51±13 minutes. Previous stroke (28.8%), bleeding events (33.3%), chronic kidney disease including 4 patients with hemodialysis (55.5%), and implanted cardiac devices (21.2%) were identified as patient characteristics. Sixteen patients (35.5%) required LAAO for the electrical isolations of the LAA resulting from complete blocks in both mitral isthmus and LA anterior wall. During a follow‐up of 750±298 days, 41 patients (91.1%) were in sinus or pacing rhythm. No significant adverse events such as device‐related thrombus, ischemic strokes, or major bleedings were observed except for 1 patient with hemodialysis who died 60 days after LAAO due to cerebral hemorrhage under dual antiplatelet therapy.

**Conclusions:** Percutaneous LAAO early after AF ablation procedures were safe and feasible, and no significant adverse events due to the early implantation of WATCHMAN devices were observed. This study result suggests LAAO early after AF ablation may be acceptable especially in patients who require semi‐urgent LAAO due to the electrical isolation of the LAA during AF ablations.

## VALIDATION OF ABLATION SITE CLASSIFICATION ACCULACY FOR ATRIAL FIBRILLATION USING THE CARTONET R 12.1 MODEL

### **WATARU SASAKI** ^1^, DAISUKE KUDO^1^, NAOMICHI TANAKA^1^, TSUKASA NAGANUMA^1^, MASATAKA NARITA^1^, HITOSHI MORI^1^, KAZUHISA MATSUMOTO^1^, KENTA TSUTSUI^2^, YOSHIFUMI IKEDA^1^, HIDETAKA ARAI^1^, SHINTARO NAKANO^1^, KAZUO MATSUMOTO^3^, RITSUSHI KATO^1^

#### ^1^Saitama Medical University International Medical Center, hidaka, Japan,^2^Teikyo university hospital, itabashi, Japan,^3^Higashimatsuyama Medical Association Hospital, higashimatsuyama, Japan

**Introduction:** CARTONET**®** enables automatic ablation site classification and reconnection site prediction using machine learning. However, the accuracy of site classification model is uncertain.

**Methods:** We studied a total of 396 cases. 313 patients underwent pulmonary vein isolation (PVI), including a cavotricuspid isthmus (CTI) ablation (PVI group) and 83 underwent PVI and additional ablation (i.e box isolation, superior vena cava isolation) (PVI+ group). To examine the validity of the learning model, a comparison was performed between the anatomical accuracy of each point automatically classified by machine learning and the anatomical positions as identified by electrophysiologists. The analysis of the ablation lines was conducted by a total of eight electrophysiologists. For each case, a double‐check was performed by two electrophysiologists and in cases where their opinions differed, a discussion was conducted to determine the location. We investigated the sensitivity and positive predictive value (PPV) for automatic site classification in the total cohort and compared PVI group with PVI+ group for subgroup analysis.

**Results:** A total of 29,422 points were analyzed. In the right PV, the sensitivity and PPV were as follows: inferior (75.3%, 96.8%); anterior (98.1%, 81.2%); roof: (89.0%, 83.7%); posterior (91.7%, 86.6%); Carina (30.9%,43.9%). In the left PV, inferior (82.1%, 96.7%); anterior (92.6%,86.8%); ridge (98.4%,91.9%); roof (93.4%,87.4%); posterior (92.5%, 83.3%); Carina (35.9%, 35.7%). In the roofline (22.4%, 33.3%), In the posterior line (18.1%, 44.0%). CTI and superior vena cava could not be recognized or analyzed.

**Conclusions:** The automatic site Classification of CARTONET**®**R12.1 model demonstrates relatively high accuracy in pulmonary veins excluding the carina.

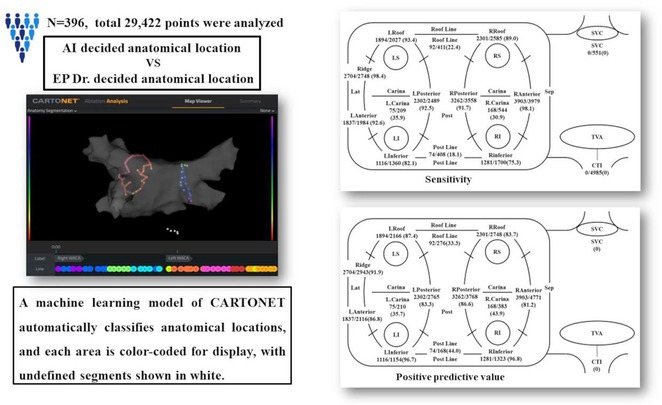

## OUTCOME AND SAFETY OF PULMONARY VEIN ISOLATION WITH VERY HIGH POWER SHORT DURATION ABLATION

### **YASUHIRO SASAKI**, JUNICHI OOKA, ATSUSHI KOBORI, YUTAKA FURUKAWA

#### Kobe City Medical Center General Hospital, Kobe, Japan

**Introduction:** At a previous conference, we reported on the acute results of pulmonary vein isolation (PVI) using very high‐power short‐duration ablation (vHPSD) at 90 w/4 sec. We found that acute re‐conduction of the anterior carina of both pulmonary veins was frequent, which led us to use 50w in the anterior wall. There are few data comparing it to combination ablation at 90w and 50w. In this study, we examined the efficacy and safety of PVI using two methods of ablation.

**Methods:** Forty patients (69±11 years, 27 male, 31 non‐paroxysmal) with AF undergoing PVI with vHPSD alone (90w group, 22 patients) or combination ablation (50/90w group, 18 patients) at our hospital. In the 90w group, 90w was used circumferentially for PVI, while in the 50/90w group, 50w using Ablation index=500‐550 was used in the anterior wall and 90w in other areas. Additional therapy other than PVI and cavotricuspid isthmus line was left to the operator's discretion. Acute re‐connection rates during the procedure, complications, and AF/Atrial tachycardia (AT) recurrence rates after 90 days postoperatively were examined.

**Results:** The rate of achieving first‐pass PVI in both groups was not significantly different in the left PV (82% vs. 94%, p=0.3555), but was lower in the 90w group in the right PV (64% vs. 100%, p=0.0047). There was no difference in acute re‐connection rates in both PVs (left PV: 27% vs. 11%, p=0.2579, and right PV: 16% vs. 11%, p=1.0000). PVI procedure time was significantly longer in the 90w group (64±22 vs. 44±15 minutes, p=0.003). Perioperative complications included 2 cases of PV‐stenosis in the 90w group, but the difference was not significant (p=0.4923). During a mean follow‐up period of 310±83 days, 3 (14%) and 3 (17%) recurrent AF/AT were observed in both groups, respectively, with no significant difference in the time‐to‐event analysis (p=0.5664).

**Conclusions:** Compared to vHPSD ablation alone, combination ablation of 50w and 90w allows for more efficient PVI, but care should be taken to avoid PV stenosis when using 90w.

## COMPARISON OF THE CRYOBALLOON PULMONARY VEIN ISOLATION USING ARCTIC FRONT ADVANCE AND POLARX

### **HIDENORI SATO**, MICHIFUMI TOKUDA, UI TAKATO, YOSHITO YAMAZAKI, RYUUTAROU SAKURAI, SATOKO SHIOMI, TAKUYA MATSUMOTO, OSATO HIROTSUNA, MASAAKI YOKOYAMA, KENICHI TOKUTAKE, SEIGO YAMASHITA, TEIICHI YAMANE

#### The Jikei University School of Medicine, Tokyo, Japan

**Introduction:** Various balloon ablation devices have been reported effective for pulmonary vein isolation (PVI) in patients with atrial fibrillation (AF). Arctic Front Advance (AFA‐pro, Medtronic) and POLARx (Boston Scientific) can now be used as cryoballoon devices.

**Methods:** Two hundred paroxysmal AF patients who underwent the initial PVI by the AFA‐pro (Group A, n=140) or POLARx (Group P, n=60) were included. Details of the procedure and follow‐up ambulatory monitoring after the procedure were compared.

**Results:** Total procedure and fluoroscopic time were similar between the two groups. The nadir temperature was significantly lower for POLARx for all PVs. In LIPV, time to isolation (29.1 vs. 39.5 sec, p=0.01) and total application time (177.1 vs. 201.8 sec, p<0.005) was significantly shorter in group P than in group A. A voltage map after PVI showed no significant difference in the isolated area of each PV. Regarding the necessity of touch‐ups, group P was significantly lower in RIPV(P<0.005) than group A. There was no significant difference in complications. One month after the procedure, ambulatory monitoring showed that the number of premature atrial contractions (PACs) in group P was significantly higher than in group A. However, there were no significant differences in PACs in ambulatory monitoring three months after the procedure.

**Conclusions:** POLARx had significantly fewer touch‐ups in RIPV than Arctic Front Advance. There was no significant difference in safety between the two groups.

## A CASE IN WHICH THE NARROW ISTHMUS OF ATRIAL TACHYCARDIA COULD BE IDENTIFIED BY CHECKING THE PEAK FREQUENCY MAP IN SINUS RHYTHM PREVIOUSLY

### **KOYO SATO**, TOMOFUMI NAKAMURA, YUUTO TESHIMA, TOSHIKI KAZUMA, YASUHIDE OOKAWA

#### Nagoya Heart Center, Nagoya, Japan

**Introduction:** N/A

**Methods:** N/A

**Results:** The patient was a 80‐year‐old man who underwent catheter ablation for repeated atrial fibrillation (AF) and atrial tachycardia (AT). He underwent pulmonary vein isolation and CTI ablation because AT couldn’t be induced by induction test. Six months after the procedure, he underwent a second session because of recurrent AF and AT. Before second session, we analyzed the sinus map of the first session with EnSite™ OT Near‐Field Software. And we detected the small low voltage area with high frequency (≧300Hz) in the left atrial anterior wall. In the second session we could induced AT with atrial burst pacing. The AT was the figure 8 reentry and the narrow isthmus of the AT was in the left atrial anterior wall, and the position of the narrow isthmus corresponded with the low voltage and high‐frequency area of sinus rhythm in the first session. Therefore, we performed RF applications to the narrow isthmus site in left atrial anterior wall, and successfully the AT was terminated. After that we perform the liner ablation from the narrow isthmus to mitral annulus. After the procedure,the patient was confirmed to have no inducible AT and AF. We report this case because we were able to estimate the circuit of AT with EnSite™ OT Near‐Field Software preoperatively.

**Conclusions:** N/A

## A CASE OF DILATED CARDIOMYOPATHY WITH MULTIPLE VENTRICULAR TACHYCARDIA CIRCUITS, IN WHICH THE INTRINSIC ANTITACHYCARDIA PACING SLGORITHM PROVED TO BE EFFECTIVE

### **TOMOHIRO SATO** ^1^, SUGURU NISHIUCHI^2^, YODO TAMAKI^1^, SHINTARO YAMAGAMI^1^, TOYOKI OKUDA^1^, TSUKASA MOTOYOSHI^1^, HIROKAZU KONDO^1^, TOSHIHIRO TAMURA^1^

#### ^1^Tenri Hospital, Tenri, Nara, Japan,^2^Gunma Prefectural Cardiovascular Center, Maebashi Gunma, Japan

**Introduction:** Intrinsic antitachycardia pacing (iATP) is a novel automated antitachycardia pacing that provides individual treatment to terminate ventricular tachycardia (VT). The iATP sequence consists of multiple S1 pulses and a final S2 pulse, and the algorithm automatically adjusts the number of pulses or shortens the S1‐S2 interval depending on the post pacing interval (PPI) of sequence 1.

**Methods:** N/A

**Results:** A 62‐year‐old woman with a history of hospitalisation for heart failure was referred to our department for further investigation and who was diagnosed with dilated cardiomyopathy on myocardial biopsy. An implantable cardioverter defibrillator (ICD) was implanted because of VT‐induced syncope. Clinical VT episodes were observed by a remote monitoring system. VT1 with a cycle length (CL) of 370 ms had a PPI of 800 ms after the first sequence and was terminated by increasing the number of S1 pulses in the second sequence; VT2 with a CL of 350 ms had a PPI of 490 ms after the first sequence; reducing the number of S1 pulses and then shortening the S1‐S2 interval was interval in the second sequence successfully terminated the tachycardia. In VT1, the PPI was longer than the PPI calculated with the estimated propagation time, and sequence 1 did not achieve the circuit and VT could not be terminated. iATP extended the estimated propagation time and increased the appropriate number of S1 pulses required to reach the VT circuit. As a result, sequence 2 was able to reach the circuit and terminate the tachycardia. In contrast, VT2 had a shorter PPI and Sequence 1 reached the VT circuit but failed to terminate the VT; the appropriate number of S1 pulses required to reach the VT circuit was automatically calculated. Furthermore, by shortening the S1‐S2 interval, the refractory period of the tachycardia was used to terminate the tachycardia. The VT episode of the ICD showed that these two VTs have different circuits causing the different waveforms and propagation times to the circuit.

**Conclusions:** This case is the first to report that iATP was effective for multiple VT circuits.

## IN‐VITRO ELECTROPORATION VEGETABLE MODEL AND STAINING PROTOCOLS WITH VARIABLE VOLTAGE AND REPETITIVE PFA APPLICATIONS

### NATHANIEL STEIGER, LOGAN MCCLENNEN, JUSTIN BILENKER, CARLOS PATINO, CARLOS MATOS, CAROLINA HOYAS, DANIEL CAMPOS, CHIEKO IMAMURA, KATHERINE M. SAUER, USHA TEDROW, PAUL C. ZEI, JORGE E. ROMERO, **WILLIAM SAUER**

#### Brigham and Women's Hospital, Boston, MA

**Introduction:** Unlike benchtop models evaluating radiofrequency (RF) lesions, living tissue is required to assess pulsed field ablation (PFA) lesion characteristics. An in vitro vegetable model could allow extensive evaluation of PFA strategies on lesion formation without using animals. We sought to demonstrate the potential utility of an *in vitro* electroporation vegetable model.

**Methods:** Eggplant, sweet potato, beets, and Russet potatoes underwent unipolar PFA in a saline bath using a 3.5 mm electrode catheter and a submersed grounding pad connected to a custom pulsed‐field generator. PFA sequences were delivered on the vegetable models with amplitudes ranging from 480V to 5000V delivered over repeated applications. The slabs were sliced and stained in 0.5% 2,3,5‐triphenyltetrazolium chloride (TTC), 0.5% triarylmethane blue, and 0.5% potassium iodine solutions.

**Results:** The Russet potato, using the TTC staining protocol, yielded consistent and visible lesions. Non‐potato vegetable models and non‐TTC stains produced inferior and inconsistent results. For the TTC‐stained potatoes, 3 distinct staining zones were identified (Figure 1A) corresponding to necrostic cells, electroporated cells with intact organelles, and unaffected tissue. As expected, increasing voltage demonstrated a linear increase in radius, depth, and volume. Increasing the delivered application number from 1 to 3 demonstrated an increase in depth and plateaued beyond three applications (Figure 1B and C).

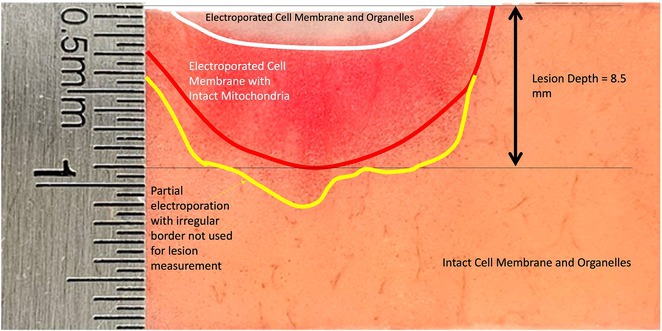

**Conclusions:** An *in vitro* electroporation potato model can be used to assess cellular injury induced by surface PFA and allow for further investigation of PFA biophysics prior to *in vivo* testing. In this model, adjusting voltage output and repeated number of applications leads to altered lesion characteristics that are comparable with animal studies using viable cardiomyocytes.

## BIZARRE AND MALIGNANT ARRHYTHMIA IN DILATED CARDIOMYOPATHY

### OUNG SAVLY

#### Kantha Bopha Children's Hospital, Phnom Penh, Cambodia

**Introduction:** Patients with dilated cardiomyopathy (DCM) can develop a broad range of brady‐arrhythmias and tachy‐arrhythmias including sinus node dysfunction, various degrees of atrioventricular block, interventricular conduction delay, and atrial and ventricular arrhythmias.

**Methods:** N/A

**Results:** 13‐year‐old, girl, 27kg, diagnosed as dilated cardiomyopathy at the age of 11. She is ongoing with ACE inhibitor, digoxin, aspirin, diuretic and spironolactone. Two days prior her admission, she complains of fainting and chest discomfort. She's apyretic. During hospitalization, she lost consciousness brutally with seizure while she was eating.

She is certainly at risk for having VT/VF which explains her seizure. In our case, the arrhythmia has subsided with electrolyte correction. It becomes in sinus rhythm. She is doing well. She was discharged home in one week and we just continued the anti‐heart failure medications and potassium supplement and nothing else is needed other than to not let her electrolytes get that abnormal again.

**Conclusions:** In case of DCM with the routine use of diuretics, the maintain high potassium is very crucial. The lower the potassium, the higher risk of ventricular arrhythmia, sometimes lethal.

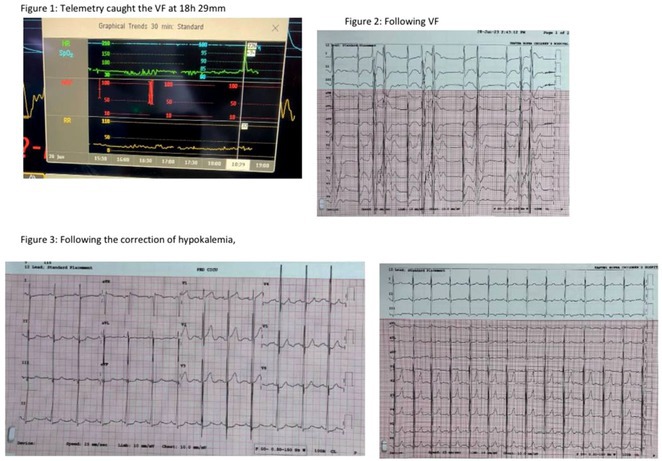

## UNCERTAIN EPICARDIAL PACEMAKER LEAD STATUS DUE TO SEVERE BATTERY DEPLETION

### **OUNG SAVLY** ^1^, JEFFREY M VINOCUR^2^

#### ^1^Kantha Bopha Children's Hospital, Phnom Penh, Cambodia,^2^Yale School of Medicine, New Haven, CT

**Introduction:** Epicardial pacing leads have elevated failure rates and require close surveillance. Patients who become lost to follow up may present at RRT/ERI with no recent lead assessment leading to clinical complexity.

**Methods:** N/A

**Results:** The first patient is 21 years old with complex single ventricle with L‐looping and complete heart block, prior Fontan and epicardial dual‐chamber pacemaker. He presented with symptomatic bradycardia (Figure 1A, VVI 65 pacing without capture). When last seen at another hospital 3.5 years earlier, leads were functioning normally and battery was predicted 2.5 years. The Medtronic Adapta generator was at RRT status with ventricular lead impedance < 100 ohms (Figure 1B) vs prior 892 ohms, and atrial lead unable to assess in fixed VVI mode. CXR showed no radiographic fracture. The pocket was opened with preparations for possible sternotomy; the leads functioned normally (impedances 703 and 722 ohms) and new generator implanted.

The second patient is 11 years old with ventricular septal defect repaired at 11 months of age, surgical heart block with epicardial dual‐chamber pacemaker placed at that time. AV conduction apparently recovered at some point but the timeline of this was uncertain given limited follow up. No pacemaker records were available. The Medtronic Adapta generator was found to be past RRT with inability to clear alert status (Figure 1C) and no way to assess lead function. CXR showed no radiographic fracture. Given risk for recurrent AV block, and subtle sinus node dysfunction (Figure 1D), the pocket was opened with preparations for possible sternotomy; the leads functioned adequately and new generator implanted.

**Conclusions:** When pacemaker patients present after reaching RRT, lead assessment may be limited by design (many Medtronic devices allow only ventricular lead testing after RRT) or by profound battery depletion (when any device can behave erratically, as seen here). While most leads will turn out to be functional, ideally generator change would be performed in a setting where new leads could be placed if required, to avoid delays in reestablishing pacemaker function and the infection risk associated with early pocket reopening.

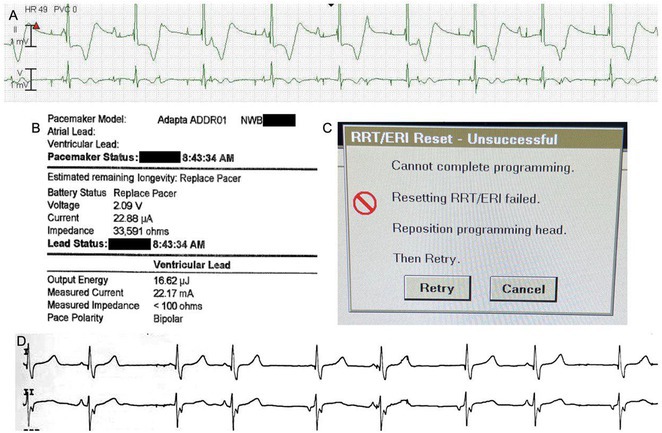

## PROTEINURIA IS ASSOCIATED WITH AN INCREASED RISK OF SUDDEN CARDIAC ARREST IN THE YOUNG POPULATION

### **CHANG‐OK SEO** ^1^, YEJI KIM^1^, JOO HEE JEONG^1^, KYUNG‐DO HAN^2^, SEUNG‐YOUNG ROH^3^, HYOUNG SEOK LEE^1^, YUN YOUNG CHOI^1^, JAEMIN SHIM^1^, YOUNG‐HOON KIM^1^, JONG‐IL CHOI^1^

#### ^1^Korea University College of Medicine and Korea University Anam Hospital, Seoul, Korea, Republic of,^2^Soongsil University, Seoul, Korea, Republic of,^3^Korea University College of Medicine and Korea University Guro Hospital, Seoul, Korea, Republic of

**Introduction:** Proteinuria is a risk factor for cardiovascular events, but its prognostic value for sudden cardiac arrest (SCA) in healthy young individuals remains unproven. We aimed to evaluate whether proteinuria in young people is associated with an increased risk of SCA.

**Methods:** This retrospective study used nationwide healthcare claims data of South Korea. Individuals aged between 20‐39 years who underwent health screening between 2009 and 2012 were included (n=6,891,400). Urine protein was measured using the spot urine dipstick method. Main outcome was aborted or unaborted SCA identified by International Classification of Disease, 10^th^ edition codes.

**Results:** The mean age was 30.9±5.0 years. Participants with proteinuria had a higher prevalence of hypertension, diabetes mellitus, dyslipidemia, chronic kidney disease, and heart failure. During follow‐up, SCA occurred in 5,352 individuals. Participants with proteinuria had a higher incidence of SCA (0.19) than those without proteinuria (0.09). Adjustment of confounders resulted in an 83% increase in the risk of SCA in participants with proteinuria (adjusted hazard ratio 1.83, 95% confidence interval 1.58‐2.12, p<0.001). Participants with proteinuria +3‐4 showed a significant increase in the risk of SCA (3.43, 2.29‐5.13, p<0.001). The proteinuria with chronic kidney disease subgroup had the highest risk of SCA (5.52, 3.80‐8.03, p<0.001).

**Conclusions:** Proteinuria was significantly associated with an increased risk of SCA in healthy young people. Individuals with pre‐existing chronic kidney disease showed a stronger association between proteinuria and the risk of SCA.

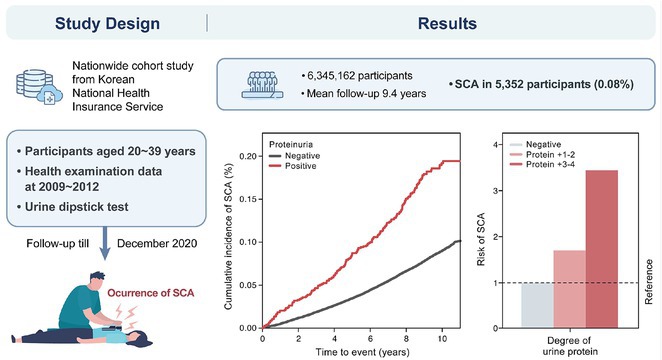

WOLFF PARKINSON WHITE SYNDROME CASE SERIES :PERSONALIZED APPROACH FOR EACH PATIENT’S ACCESSORY PATHWAY

**NUR SEPDYANTI**, MUZAKKIR AMIR, SUMARNI WAHYUDI;

Hasanuddin University, Makassar, Indonesia.

**Introduction:** Wolf‐Parkinson‐White (WPW) syndrome involves abnormal pathways increasing the risk of dangerous heart rhythms, ranking as the second most common cause of certain heart rhythm disorders worldwide. Management options range from monitoring to medications or ablation therapy.

**Methods:** N/A

**Results:** We present three cases of WPW syndrome, each with unique clinical backgrounds, presentations, ECG findings, and accessory pathway (AP) locations. Treatment decisions were tailored to individual risk assessments. The first case involved a 39‐year‐old man experiencing symptomatic tachyarrhythmia without structural heart disease. His ECG revealed atrial fibrillation (AF) with pre‐excitation, spontaneously converting to sinus rhythm. WPW syndrome type A was diagnosed, and based on ECG findings, the left lateral AP was suspected. Following American Heart Association (AHA) guidelines, catheter ablation was performed, confirming the site of origin at the Left Lateral AP. The second case featured a 48‐year‐old woman with symptomatic but undocumented tachyarrhythmia and no structural heart disease. Hospital ECG showed sinus rhythm with WPW syndrome type B, suggesting the right septal annulus as the AP site. An electrophysiology (EP) study confirmed the Right Anteroseptal AP location. The third case involved a 19‐year‐old male with asymptomatic WPW syndrome discovered incidentally during a medical checkup. His ECG indicated WPW syndrome type A, with suspicion of the Left Lateral AP site. Following European Society of Cardiology (ESC) guidelines, an EP study was conducted due to his high‐risk occupation, revealing the Left Lateral AP site. High‐risk features of sudden death were identified, prompting catheter ablation for management.

**Conclusions:** Management of WPW syndrome should be individualized. Patients' symptoms, location of the accessory pathway, and risk stratification are useful for determining management and prognosis. Understanding WPW is very helpful in educating patients about further treatment.

## VENTRICULAR ARRHYTHMIA IN CARDIAC SARCOIDOSIS: IS THIS A SIGN WE SHOULD BE MORE AGGRESSIVE?

### **CYNTIA SEPTRIYANTI** ^1^, MOHAMMAD IQBAL^2^, GIKY KARWIKY^2^, CHAERUL ACHMAD^2^, HAWANI SASMAYA PRAMESWARI^3^, ASTRI ASTUTI^4^, NURAINI YASMIN KUSUMAWARDHANI^4^

#### ^1^University of padjajaran, Bandung, Indonesia,^2^Electrophysiologist University of padjajaran, Bandung, Indonesia,^3^Division of Heart Failure University of padjajaran, Bandung, Indonesia,^4^Division of Imaging University of padjajaran, Bandung, Indonesia

**Introduction:** The incidence rate of ventricular arrhythmias (VAs) in patients with cardiac sarcoidosis (CS) is unclear, however it relates to poor outcome. This case report reviews the knowledge of CS complicated with VAs.

**Methods:** N/A

**Results:** A 27 years old woman was referred to our center complaining of intermittent palpitations over the past one week. Elevated cardiac Troponin I. Electrocardiogram (ECG) demonstrated sinus tachycardia with frequently multifocal premature ventricular contraction and prolonged QTc interval (470msec) with no significant hypokalemia. Echocardiography showed dilated LA, LV, eccentric LV hypertrophy with reduced LV systolic function and global hypokinetic. CMR findings were in keeping with sarcoidosis with myocardial and pericardial inflammation. The endomyocardial biopsy (EMB) result favored myocarditis without signs of sarcoidosis considering patchy involvement of myocardial part with lack success rate in EMB. The patient was diagnosed with acute fulminant myocarditis related to cardiac sarcoidosis, meeting the 4 major points of The Japanese Circulation Society criteria. During hospitalization, she had 2 episodes of VT and VF while the ECG showed normal QTc interval. She was treated with prednisone 1x40 mg, colchicine 2x0.5 mg, ibuprofen 3x600 mg, and lidocaine 0.5 mg/hour. She was also given methylprednisolone 1x500 mg iv for 3 days continued with methylprednisolone 24‐0‐12 mg and azathioprine 1x50 mg orally. Unfortunately, despite optimal management she deceased after an episode of VT.

**Conclusions:** VAs is a significant predictor of mortality in patients with CS. The role of immunosuppressants in treating electric instability in CS remains unclear. We believe that prompt initiation of corticosteroid therapy in CS patients may improve outcomes. Therefore, aggressive therapy to manage inflammation in CS is needed especially in patients with VAs as these cases have a high mortality rate.

## CATHETER ABLATION OF PERSISTENT ATRIAL FIBRILLATION USING A COMBINATION OF PULMONARY VEIN ISOLATION, POSTERIOR BOX ISOLATION, AND COMPLEX FRACTIONATED ATRIAL ELECTROGRAMS IN A PATIENT WITH A HISTORY OF MITRAL VALVE REPAIR

### **TIYA SETIADI** ^1,2^, S. BUDHI RAHARJO^1,3^, D. YUGO HERMANTO^1^, D. ARMEIN HANAFY^1^, YOGA YUNIADI^1^

#### ^1^Division of Arrhythmia, Department of Cardiology and Vascular Medicine, Faculty of Medicine, Universitas Indonesia / National Cardiovascular Center Harapan Kita, Jakarta, Indonesia,^2^Cilandak Marine Hospital, Jakarta, Indonesia,^3^Heartology Cardiovascular Center, Jakarta, Indonesia

**Introduction:** A strategy of substrate based ablation targeting areas of complex fractionated atrial electrograms (CFAE) for AF ablation remains controversial. We report an ablation case of persistent AF in a patient with post mitral valve repair.

**Methods:** N/A

**Results:** A 56 year old man with a history of prior coronary artery bypass graft and mitral valve repair 5 years prior was referred for management of persistent AF. Echocardiography data showed an LA diameter of 52 mm, an LA volume index of 59 ml/m^2^, and normal LV function. The patient underwent 3D ablation using the EnsiteX EP system under general anesthesia. Wide‐area circumferential ablation was performed to isolate the pulmonary veins completely. Following this, repeat cardioversion failed to convert to sinus rhythm. We decided to do posterior box isolation, but the cardioversion still failed. A complex fractionated atrial electrograms (CFAE) map was performed and continued with ablation of the CFAE at the anterior left inferior pulmonary vein (PV), roof, anterolateral, and anteroseptal mitral annulus. Post CFAE ablation, single cardioversion was successfully converted to sinus rhythm.

**Conclusions:** A combination of pulmonary vein isolation, posterior box isolation, and CFAE ablation was useful in a persistent AF patient with a history of mitral valve repair.

## MULTIPLE ACCESSORY PATHWAY CATHETER ABLATION IN EBSTEIN ANOMALY : FROM ABLATE TO PACE

### **TIYA SETIADI** ^1,2^, R. AULIA FANANI^1^, D. ARMEIN HANAFY^1^, D. YUGO HERMANTO^1^, S. BUDHI RAHARJO^1^, YOGA YUNIADI^1^

#### ^1^Division of Arrhythmia, Department of Cardiology and Vascular Medicine, Faculty of Medicine, Universitas Indonesia / National Cardiovascular Center Harapan Kita, Jakarta, Indonesia,^2^Cilandak Marine Hospital, Jakarta, Indonesia

**Introduction:** Catheter ablation of accessory pathways in Ebstein's anomaly is a challenging and demanding procedure with a generally low success rate fraught with recurrence and repeat procedures in almost up to half of the patients. Structural abnormalities of the atrioventricular conduction system may observed with the Ebstein malformation. We report an ablation case in an Eibstein patient with multiple accessory pathways who underwent pacemaker implantation one month after secondary ablation.

**Methods:** N/A

**Results:** A 51 year old male patient with Eibstein anomaly was referred to our centre due to recurrent palpitations associated with chest discomfort. A 12 lead ECG during sinus rhythm showed ventricular pre excitation suggesting from right side AP. He underwent successful septal AP ablation with residual bystander right lateral AP. He was taken up for a repeat radiofrequency ablation (RFA) due to recurrent palpitation six months later after his first ablation. However, we decided to defer it because of episodes of total atrioventricular (AV) block during ablation. After one minute of observation, AV dissociation subsided with documented AV fusion in the posteroseptal tricuspid annulus. Ventricular pacing still showed retrograde block. We performed pacemaker implantation one month after the last ablation due to an episode of syncope and total AV block.

**Conclusions:** Multiple AP catheter ablation in Ebstein patients remains challenging due to its high recurrence and this case report demonstrates the extremely rare association of Ebstein's anomaly with complete AV block after AP ablation that was successfully managed by permanent pacemaker implantation

## T‐WAVE INVERSION AFTER SUCCESSFUL WPW ABLATION: A DEFINITE PATHOLOGICAL PHENOMENON?

### **EKIY SETIYAWAN** ^1^, ARSHA PRAMUDYA^1^, IRNIZARIFKA IRNIZARIFKA^1,2^, CHAERUL ACHMAD^1,3^

#### ^1^Hasna Medika Cirebon Hospital, Cirebon, Indonesia,^2^Department Cardiology and Vascular Medicine, Universitas Sebelas Maret, Surakarta, Indonesia,^3^Department Cardiology and Vascular Medicine, Universitas Padjadjaran, Bandung, Indonesia

**Introduction:** Wolff‐Parkinson‐White (WPW) syndrome is a cardiac pre‐excitation syndrome due to abnormal electrical conduction through accessory pathway (AP) characterized by short PR intervals, wide QRS duration and delta waves. T‐wave inversion is most commonly associated with ischemic conditions. However, it can also occur because of ventricular depolarization and repolarization vectors changes, known as T‐Wave memory. We report a case of T‐Wave Inversion post right posteroseptal (RPS) WPW Ablation due to T‐Wave Memory.

**Methods:** N/A

**Results:** A 52‐year‐old female, no CAD risk factors, were admitted due to recurrent palpitations. The surface ECG evinced manifest WPW (RPS AP) (Figure 1‐A). EP Study was decided and found malignant parameters of the AP with AV Fusion at right posteroseptal tricuspid annulus. Ventricular pacing showed an earliest retrograde A at the same point. Ablation was successfully performed using a 7F non‐irrigating ablation catheter. Ventricular pacing evaluation showed VA dissociation, and antegrade conduction showed earliest V at His catheter. Aggressive pacing from the atria and ventricle did not induces further tachyarrhythmia. Despite no delta wave evinced after ablation, there was T wave inversion at inferior lead (Figure 1‐B) without evidence of chest pain. After 5 months post ablation, no palpitation complained by the patient. ECG showed no delta wave and alleviation of the T wave inversion (Figure 1‐C).

**Conclusions:** We report a case of T‐Wave inversion post successful RPS AP ablation. Since no clinical symptom and the T‐wave inversion alleviates after 5 months, we assessed this T‐Wave inversion occurred due to T‐Wave memory. This is due to changes in ventricular depolarization and repolarization vectors which previously fused through the AP and AV Node to only AV Node conduction which manifests as changes in ventricular T waves and polarity after ablation of AP. Normalization of the T waves in our case was observed 5 months after the procedure. Based on our case, we conclude that the T‐Wave inversion in patients with post ablation WPW is not a definite pathological phenomenon, that might be misinterpreted as ischemia.

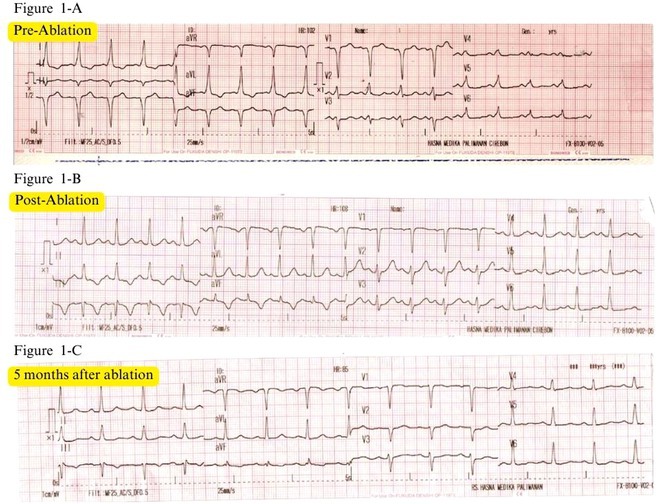

## FAILURE OF ICD SHOCK OBSERVED IN CARDIAC SARCOIDOSIS PATIENT PRESENTING WITH POLYMORPHIC VT

### **NADINE NURANI SHABRINA** ^1^, GIKY KARWIKY^2^, MOHAMMAD IQBAL^2^, CHAERUL ACHMAD^2^, HAWANI SASMAYA PRAMESWARI^3^

#### ^1^Department of Cardiology and Vascular Medicine, Padjadjaran University, Bandung, Indonesia,^2^Electrophysiology and Arrhythmia Division, Department of Cardiology and Vascular Medicine, Padjadjaran University, Bandung, Indonesia,^3^Heart Failure Division, Department of Cardiology and Vascular Medicine, Padjadjaran University, Bandung, Indonesia

**Introduction:** Implantable cardioverter‐defibrillators (ICDs) are utilized in patients who survive sustained VT or VF that is either transitory or at high risk for recurrent arrhythmia. Ventricular arrhythmias in cardiac sarcoidosis (CS) patients may be treated with immunosuppressant therapy, antiarrhythmic drugs, catheter ablation, or ICD placement. We report a failed ICD shock case of CS presenting with polymorphic VT, along with strategy in such patients

**Methods:** N/A

**Results:** A 60‐year‐old male with a known case of CS and a history of monomorphic VT. A single chamber ICD was implanted six months prior to the current event for secondary prevention of sudden cardiac death. In December 2023, patient was admitted to the emergency room as a result of an abrupt complaint of palpitation. ECG showed polymorphic VT with a heart rate of 166‐188 beats per minute (bpm). The ICD failed to deliver the shock, leading to the inability to terminate the VT and need for external defibrillation. The examination of the ICD revealed abnormal sensing parameters. The ventricular autosensing histograms detected that all identified polymorphic waves; a fast VT zone, were below the sensitivity threshold. The ICD was reprogrammed to deliver fast VT therapy, switching from VT to VF. The treatment protocol now consists of 4x ATP and 2x 35 J shocks, which has been adjusted to 3x ATP and 3x 35 J shocks. The transition from monomorphic to polymorphic VT was thought to be the result of persistence inflammation in CS. As the PET scan was inaccessible, the patient underwent a cardiac MRI. The results were not interpretable due to substantial artifacts. The decision remains to escalate the immunosuppressants with azathioprine and corticosteroids. During the three‐month follow‐up, the patient remained to show no signs or symptoms.

**Conclusions:** Changes in arrhythmia morphology may be attributable to the presence of peculiar timing cycles or algorithms programmed in the device. Collaboration within a multidisciplinary team is required to prevent and manage this condition.

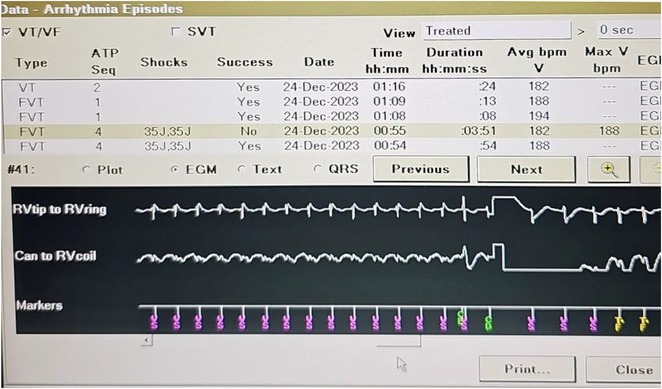

## SEX DIFFERENCES IN SYMPTOMS AND QUALITY OF LIFE, ROLE OF EDUCATION

### **ELNAZ SHAHMOHAMADI** ^1^, JEROEN M. HENDRIKS^2^, DEBRA ROWETT^3^, PRASHANTHAN SANDERS^1^, MELISSA E. MIDDELDORP^1^

#### ^1^Centre for Heart Rhythm Disorders, University of Adelaide, Adelaide, Australia,^2^Caring Futures Institute, College of Nursing and Health Sciences, Flinders University, Adelaide, Australia,^3^School of Pharmacy and Medical Sciences, University of South Australia, Adelaide, Australia

**Introduction:** There is a significant paucity of data regarding the role of sex differences in atrial fibrillation (AF). Females are often under‐represented in trials, with more symptoms of AF and greater impairment in quality of life. We aimed to assess the difference in role of a dedicated education program between males and females.

**Methods:** In a multicentred randomised controlled trial, patients who presented to the emergency department with a primary diagnosis of AF were recruited. Patients were randomised 1:1 to education group (EG) or usual care and followed up for 24 months. Education was undertaken in the form of two home‐visits with delivered by a nurse or pharmacist, with focus on medications, lifestyle changes and application of a person AF action plan in the event of an episode. Quality of life was measured through general health‐related quality‐of‐life questionnaires (AFEQT and SF‐36) and AF symptom burden and severity was assessed by the Atrial Fibrillation Severity Scale (AFSS).

**Results:** A total of 627 patients were randomised to EG (n=314) and standard of care (n=313), 42% female. Females had a higher CHADSVASC (3 [IQR: 2‐5] vs 2 [IQR: 1‐3], p<0.001), and higher proportion of obese BMI >30, (50% vs 37%, p=0.002). A higher proportion of females lived alone (38% vs 19% in men). Females presented with greater symptoms of palpitations (p=0.003), syncope (p=0.005), fatigue (p<0.001), and nausea (p<0.001). Females considered their AF less serious compared to a heart attack (63% vs 74%, p=0.036). At enrolment, females reported lower adjusted AFEQT scores [66 (64‐68) vs 70(68‐71)]. At 24 months, the adjusted AFEQT scores among males were significantly higher in in EG group compared to control group, with no significant difference observed among females in different groups. There were no significant differences in Global Well Being subscale of the AF‐Severity scores between sexes at 24 months (p=0.723).

**Conclusions:** Females presented with greater symptoms however viewed their AF not as seriously as what males perceived. Home‐based structured education, improved quality of life in both females and males, with more difference reported among males.

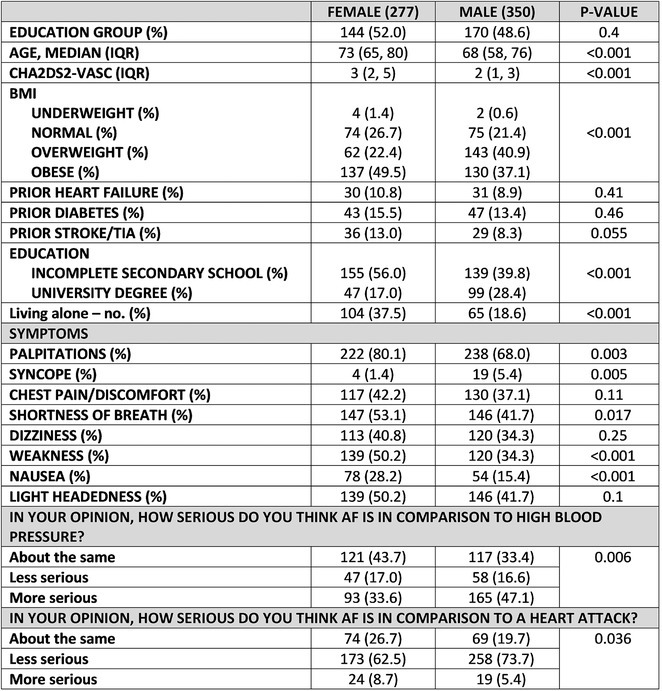

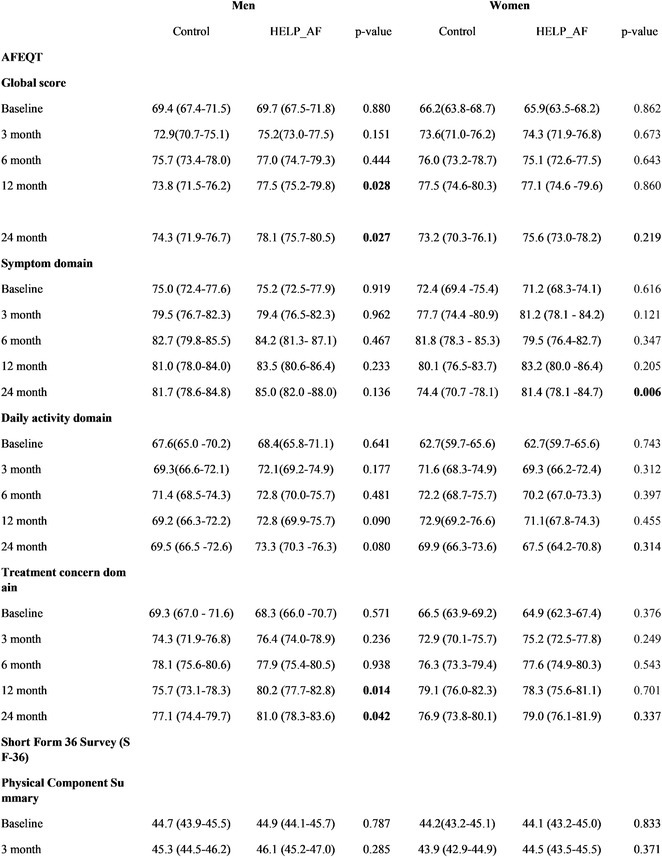

## SYNCOPE AFTER ICD IMPLANTATION IN A PATIENT WITH COMPLEX CONGENITAL HEART DISEASE ‐ WHAT IS THE MECHANISM?

### **MICHAEL SHEHATA**, SABINA HADZIABDULAOHVIC, ASHKAN EHDAIE

#### Cedars Sinai Heart Institute, Los Angeles, CA

**Introduction:** The mechanisms of periodic ventricular oversensing in pacemaker‐dependent patients may be obscure and difficult to reproduce.

**Methods:** N/A

**Results:** Case: A 41‐year‐old female with a history of repaired CCHD (pulmonary atresia and Ebstein anomaly), prior ablations for atrial arrhythmias, and high‐burden NSVT presented with syncope during exercise and intermittent CHB. A dual chamber ICD was implanted with an integrated bipolar RV sensing vector (tip‐coil) due to large T waves observed in a dedicated bipolar configuration (tip‐ring) and concern for potential T‐wave oversensing. She presented two months later with episodes of profound unprovoked syncope. ICD interrogation showed pacemaker dependence with normal lead parameters, no active oversensing, and no tachyarrhythmias. Neurological evaluation was normal. Monitoring is shown during transient loss of consciousness in Fig 1A. Maneuvers to provoke myopotentials or unmask any source of oversensing were attempted but not reproducible. A Cardiac CT scan is shown in Fig 1B. Suspicion was high for far‐field atrial oversensing on the ventricular channel due to 1.) RV coil in proximity to atrial tissue due to Ebstein's anomaly, and 2.) delayed far‐field atrial signal due to prior ablation potentially occurring after the atrial blanking period on the ventricular channel. Sensing was changed to dedicated bipolar and RV sensitivity was decreased (0.3 to 0.45 mV) empirically to address this possibility. The patient was observed on telemetry for 24 hours, and a 2‐week ambulatory monitor showed no evidence of asystole/failure to pace. Syncope did not recur.

**Conclusions:** Our case highlights the potential for intermittent far‐field atrial oversensing in a dual‐chamber ICD in a patient with Ebstein anomaly and integrated bipolar RV sensing. Clinicians should be aware of this potential issue, especially in Ebstein anomaly and preferentially use a dedicated bipolar RV lead and configuration if possible.

Figure 1. A ‐ Telemetry showing asystole due to failure to pace. B ‐ Cardiac CT showing the proximal portion of the defibrillator coil (red arrow) in the right.

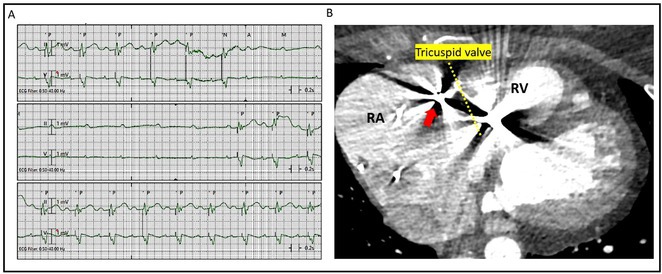

## UNUSUAL CASE OF WOLF‐PARKINSON‐WHITE: ENDOCARDIAL ABLATION OF PATHWAY CONDUCTION VIA THE RIGHT VENTRICULAR OUTFLOW TRACT

### NATASHA CUK, ERIC BRAUNSTEIN, ARCHANA RAMIREDDY, ASHKAN EHDAIE, XUNZHANG WANG, **MICHAEL SHEHATA**

#### Cedars‐Sinai Medical Center, Los Angeles, CA

**Introduction:** Accessory pathways (AP) are muscular bundles which typically connect the atrium and ventricle at the level of the AV valves. Endocardial catheter ablation is highly effective and has become the standard for curative treatment of Wolf‐Parkinson‐White syndrome. Rare non‐annular pathways such as those arising from the right atrial appendage (RAA) to the right ventricle (RV) have been described and account for <0.5% of all AV connections. Catheter ablation of an unusual AP from the right ventricular outflow tract (RVOT), however, has not been previously described.

**Methods:** N/A

**Results:** A 41 year‐old male with a manifest right‐sided accessory pathway participating in recurrent orthodromic and antidromic AVRT and AF with high risk features presented for a repeat EP study after his third failed ablation.

Baseline delta wave had an inferior axis with V1 rS pattern suggestive of right anterior free wall accessory pathway (surface ECG leads Figure 1A‐B). VA conduction was nodal. Activation mapping was performed in sinus rhythm. Earliest ventricular activation was mapped along the tricuspid annulus with a 4mm nonirrigated ablation catheter and measured ‐35ms pre‐delta along the anterior RA free wall (Figure 1A) but a 25 second lesion (max 30W/60°C) failed to eliminate pathway conduction. The RAA demonstrated only atrial electrograms. Within the RVOT, no atrial signal was seen but the earliest ventricular activation was ‐55ms pre‐delta (Figure 1B) along the anterior/free wall RVOT (Figure 1C). Pathway conduction was eliminated after 5.5 seconds of RF at this location (Figure 1D) and the lesion was continued for 60 seconds. After a 30‐minute waiting period, no return of pathway conduction was seen, and the patient had no recurrence of palpitations or tachycardia in follow up.

**Conclusions:** Non‐annular APs with RAA to RV connections are rare. Of these, only endocardial ablation within the RAA has been described and a single case of RAA‐RVOT connection has been reported. We present a case of successful pathway elimination via ablation within the RVOT, a novel approach for rare epicardial right‐sided pathway connections.

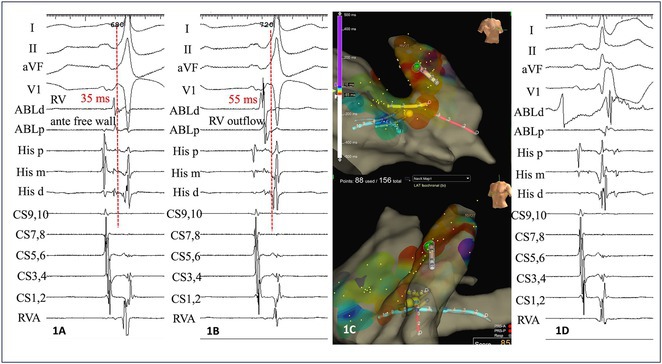

## NON SUSTAINED WIDE AND NARROW QRS COMPLEX TACHYCARDIA‐ WHAT IS THE MECHANISM?

### VIJAY SHEKAR

#### Apollo Hospitals, Tiruchirapalli, India

**Introduction:** N/A

**Methods:** N/A

**Results:** A 36 year old female presented with recurrent palpitations and presyncope. 24 hour Holter demonstrated multiple self‐terminating episodes of narrow and wide complex tachycardia. In view of tachycardias of varying morphology, patient was evaluated for myocarditis and found to be negative.During EP study, VA conduction was concentric with VAWB at 450 ms. On atrial pacing and isoprenaline facilitation, narrow complex tachycardia with HVA sequence and eccentric atrial activation pattern was induced. Entrainment could not be performed due to self‐terminating tachycardia. Spontaneous termination with V and intermittent VA dissociation were observed, suggesting atrial tachycardia(AT) as the possible mechanism. 12 lead ECG during tachycardia showed P wave positivity in V1 suggestive of left atrial focus.Another tachycardia ‐ wide complex tachycardia (WCT) of LBBB morphology was inducible with positive HV interval, 1:1 VA relationship and similar atrial activation pattern. Diagnostic maneuvers could not be performed as tachycardia was ill sustained. in view of similar atrial activation pattern and AA intervals preceding cycle length changes in HH intervals, AT with aberrancy was considered.Transeptal puncture was done and left atrium was mapped under 3D EAM guidance ( Navistar, Carto 3). Atrial activation mapping demonstrated earliest A (46 ms)along the lateral aspect of mitral annulus. The observations were consistent with focal AT arising from the mitral annulus. RF energy ( 50W/55C) was delivered, which terminated the tachycardia within 5 seconds and lesions were further consolidated.Both tachycardias were non inducible after the RF application, thus confirming focal AT from mitral annulus as the common underlying mechanism. Spontaneous initiation and termination, varying cycle length and failure to entrain suggest enhanced automaticity as the arrhythmogenic mechanism.Our case highlights an uncommon presentation and mechanism of mitral annular AT. Patient presented with tachycardias of varying morphology and enhanced automaticity as the possible underlying mechanism.

**Conclusions:** N/A

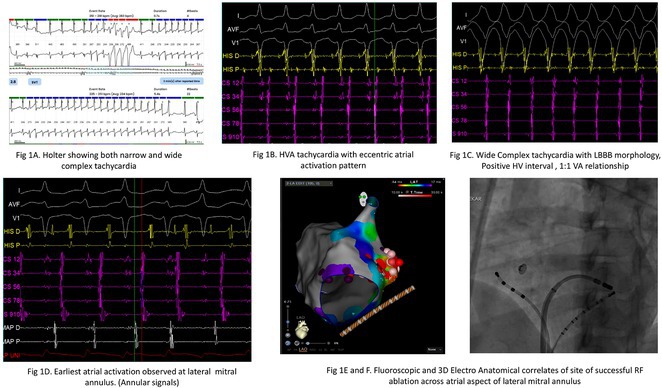

## RA ‐ LV PACING FOR COMPLETE HEART BLOCK POST TRICUSPID VALVE REPLACEMENT ‐ A CASE REPORT OF TWO PATIENTS

### **VIJAY SHEKAR** ^1^, AURAS ATREYA^2^

#### ^1^Apollo Hospitals, Tiruchirapalli, India,^2^AIG Hospitals, Hyderabad, India

**Introduction:** N/A

**Methods:** N/A

**Results:**

**Case Reports:CASE 1:** A 32 year old female, Post Tetralogy of Fallot(TOF) repair 26 years back presented with Class III dyspnea for the past 3 months. She was evaluated to have severe pulmonary and tricuspid valve regurgitation. Patient underwent successful replacement of pulmonary (23 mm Epic Plus, Abbott) and tricuspid valve( 29 mm Epic Plus, Abbott) replacement via re do thoracotomy. Post operatively, patient developed complete heart block, requiring permanent pacing. RA ‐ LV pacing was accomplished with an active fixation LV quadripolar lead (Attain Stability Quad, Medtronic) in the posterolateral vein and additional LV bipolar lead ( Attain Ability Plus, Medtronic)in the posterior vein. **CASE 2 :** A 61 year old female with restrictive cardiomyopathy, preserved EF and severe TR was evaluated for liver transplant due to HCV related chronic liver disease. To improve graft survival, patient underwent bioprosthetic tricuspid valve replacement Post operatively patient developed complete heart block requiring permanent pacemaker implantation. RA ‐ LV pacing was performed using two coronary sinus leads (Quadripolar Active fixation‐ (Attain Stability Quad, Medtronic) lead in posterolateral vein and Bipolar Lead (Attain Ability, Medtronic) in anterior interventricular vein). Both patients have stable pacing parameters during follow up. Presence of prosthesis in tricuspid position poses significant challenges to conventional pacemaker implantation. Though conventional pacing (RV leads) have been positioned across tricuspid valve, the procedure carries significant risk of damage to the prosthetic valve during manipulation, risk of infective endocarditis and tricuspid regurgitation.Alternate techniques ‐ RA ‐ LV pacing using coronary sinus leads, epicardial pacing and leadless pacemakers have been described to overcome these challenges. RA ‐ LV pacing is an effective alternative pacing strategy for post tricuspid valve replacement patients with pacing indications. Use of 2 coronary sinus leads may be needed to ensure stable pacing parameters and ventricular synchrony.

**Conclusions:** N/A

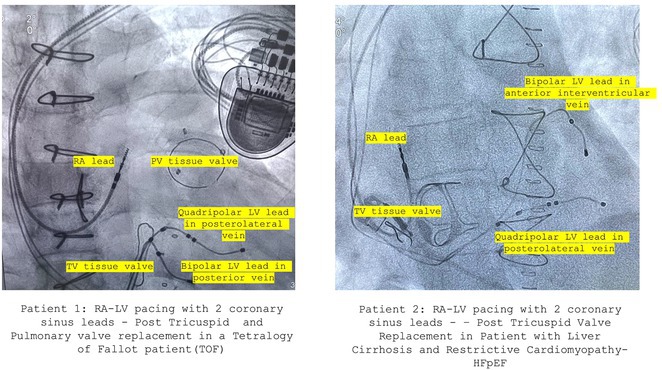

## EXPLORING PREDICTIVE FACTORS FOR SURVIVAL OUTCOME IN CARDIAC ARREST PATIENTS UNDERGOING EXTRACORPOREAL MEMBRANE OXYGENATION: A RETROSPECTIVE ANALYSIS

### CHUN‐TING SHIH

#### Kaohsiung Chang Gung Memorial Hospital, Kaohsiung City, Taiwan

**Introduction:** Extracorporeal membrane oxygenation (ECMO) has been established as a last‐resort therapy for patients experiencing cardiac arrest. Despite its efficacy, the predictive factors influencing final survival remain unexplored. Therefore, our study aimed to investigate the survival outcomes of patients with cardiac arrest who underwent ECMO rescue therapy.

**Methods:** A retrospective single‐center evaluation was conducted on all patients who experienced in‐hospital and out‐of‐hospital cardiac arrest and received ECMO support between 2021 and 2023. The primary outcomes assessed were 30‐day and 1‐year survival rates following cardiac arrest, with secondary endpoints focusing on neurological and functional disability outcomes.

**Results:** Ninety‐nine patients were included in the analysis. The final mortality rate was 76.8% (n=76 deceased). Survivors exhibited significantly higher Glasgow Coma Scale (GCS) scores (5.5 [3.0‐8.0] vs. 3.0, P < 0.001), a shorter duration of ECMO support (3.0 [1.0‐5.0] vs. 3.5 [2.0‐5.0], P < 0.001), a lower incidence of cardiogenic shock (60.9% vs. 88.2%, P = 0.003), better cardiac function at the time of ECMO placement, fewer instances of coagulopathy, and improved perfusion. Additionally, survivors exhibited lower levels of creatinine (1.46 [0.79‐2.69] vs. 2.11 [1.19‐3.35], P = 0.027), reduced lactate levels (79.30 [25.1‐126.9] vs. 102.1 [77.25‐121.95], P = 0.037), and less severe acidosis (pH values 7.40 ± 0.13 vs. 7.28 ± 0.19, P = 0.033). Among survivors, 10 (43.5%) achieved favorable neurological outcomes.

**Conclusions:** The survival rate of patients who received ECMO following cardiac arrest was 23.2%. Various hemodynamic, laboratory, and echocardiographic parameters associated with the outcomes of ECMO‐treated patients post‐cardiac arrest can be evaluated. The development of a predictive scoring model for survival in this population is warranted for future research.

## IMPACT OF SUPERIOR VENA CAVA ISOLATION DEMARCATION BASED ON ANATOMICAL INDICATORS

### **TAKAYUKI SHIMIZU**, JUMPEI YAMAMOTO, MASAKO ASAMI, KEIJIRO NAKAMURA, HIDEHIKO HARA

#### Toho University Ohasi Medical Center, Tokyo, Japan

**Introduction:** Traditional isolation of the Superior Vena Cava (SVC) involves separation at the SVC‐Right Atrium (RA) junctions, demanding careful attention due to the proximity of the sinus node and phrenic nerve. The common practice is to target the SVC above the roof of the Right Superior Pulmonary Vein (RSPV) as an anatomical reference, yet uncertainty persists regarding the appropriateness of this site.

**Methods:** From January 2021 to December 2023, an extensive retrospective analysis examined 70 cases of SVC isolation within a cohort of 640 undergoing ablation for atrial fibrillation. The assessment included measuring the distance from the SVC‐RA junction site to the RSPV roof using preoperative cardiac CT scans.

**Results:** With a mean age of 65 ± 1.2 years, male (69%), the average RSPV to SVC‐RA junction distance was 11.3 ± 0.8 mm. In 94% of cases, the RSPV was superior to the SVC‐RA junction (average distance: 12 ± 0.7 mm), while in 6%, it was below (distance: ‐4 ± 0.8 mm). Notably, 57% of cases positioned the RSPV more than 10 mm above the SVC‐RA junction, a standard region for SVC isolation. Remarkably, no incidents of phrenic nerve paralysis or sinus node injury were reported. Among isolated SVC cases, atrial fibrillation (AF) firing occurred in 19 cases, with seven (36%) effectively isolated within 10 mm from the SVC‐RA junction.

**Conclusions:** Utilizing the RSPV as an anatomical landmark for SVC isolation is notably secure. However, the designated isolation site may extend beyond the initial demarcation line.

## MYOCARDIAL INJURY AND INCIDENCE OF EARLY RECURRENCE OF ATRIAL FIBRILLATION AFTER CATHETER ABLATION BETWEEN TWO TYPES OF CRYOBALLOON SYSTEMS

### **SATOKO SHIOMI**, MICHIFUMI TOKUDA, UI TAKATO, RYUTARO SAKURAI, YOSHITO YAMAZAKI, TAKUYA MATSUMOTO, HIDENORI SATO, HIROTSUNA OSETO, MASAAKI YOKOYAMA, KENICHI TOKUTAKE, MIKA KATO, SEIGO YAMASHITA, SATORU MIYANAGA, MICHIHIRO YOSHIMURA, TEIICHI YAMANE

#### The Jikei University School of Medicine, Tokyo, Japan

**Introduction:** There are currently two types of cryoballoon systems for catheter ablation of atrial fibrillation (AF). It is not clear how the difference between POLARx (Boston Scientific) and AFA‐Pro (Medtronic) relates to myocardial injury and the incidence of early recurrence of fibrillation (ERAF). ERAF is defined within 90 days of AF recurrence after the procedure.

**Methods:** Consecutive 137 patients who underwent catheter ablation for paroxysmal AF by two cryoballoon devices were included (AFA‐Pro in 87; POLARx in 50). We assessed CKMB pre and post‐procedure, ERAF and the number of atrial premature contractions (APCs) at Holter monitoring one month and three months after procedure. The CKMB change ratio was defined by the following formula: (CKMBpost / CKMBpre).

**Results:** There was no significant difference in the number of CB applications and the percentage of touch‐up applications by radiofrequency catheter. CKMB change ratio was significantly higher in POLARx (19±22 vs. 27±21; p=0.04). The incidence of ERAF was similar (16% vs. 24%; p=0.28). There was no difference in the number of APCs (Table).

**Conclusions:** POLARx causes stronger myocardial injury than AFA‐Pro but does not increase ERAF and APC numbers.

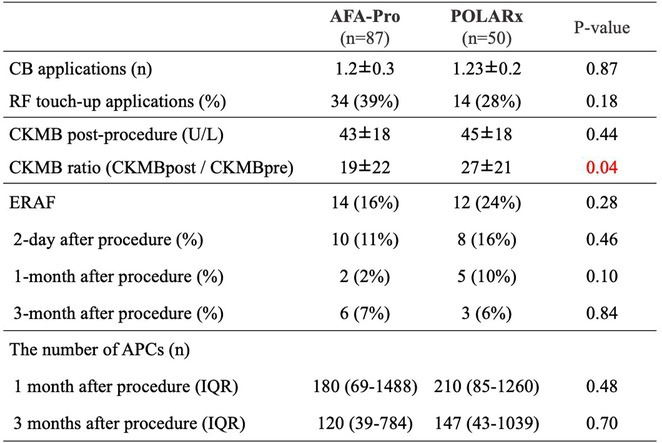

## OUTCOME OF CHEMICAL ABLATION FOR OUTFLOW TRACT VENTRICULAR ARRHYTHMIA ARISING FROM THE VICINITY OF THE LEFT VENTRICULAR SUMMIT

### **YASUHIRO SHIRAI** ^1^, KENZO HIRAO^1^, TAKESHI SASAKI^2^, KAZUTO HAYASAKA^2^, SHU YAMASHITA^2^, SHINSUKE MIYAZAKI^3^, TETSUO SASANO^3^

#### ^1^AOI Universal Hospital, Kanagawa, Japan,^2^NHO Disaster Medical Center, Tokyo, Japan,^3^Tokyo Medical and Dental University, Tokyo, Japan

**Introduction:** Catheter ablation of outflow tract ventricular arrhythmias (OTVAs) with epicardial or intramural site of origin is challenging.This study was aimed to investigate the outcome of chemical ablation for OTVAs arising from the vicinity of the left ventricular (LV) summit after failed conventional radiofrequency (RF) catheter ablation.

**Methods:** We retrospectively analyzed procedural outcomes for 8 patients in whom chemical ablation was considered after unsuccessful conventional radiofrequency catheter ablation of OTVAs.

**Results:** The coronary venous system (CVS) was mapped by the 2.7Fr size over‐the‐wire microelectrodes catheter (Japan Lifeline, Tokyo, Japan) guided by 0.014‐inch wire, and the earliest activation was recorded within the CVS in all 8 patients. The earliest activation was recorded in the LV 1^st^ septal perforator in 4, the LV annular branch in 2, the great cardiac vein (GCV) in 1, and the anterior interventricular vein (AIV) in 1 patient. The targeted branch for ethanol infusion was the LV 1^st^ septal perforator in 4 and the LV annular branch in 4 patients, respectively. In 2 patients in whom the earliest activation was recorded within the GCV and the AIV, the earliest activation site was targeted via collateral flows from the CVS branches. Targeted OTVA was suppressed in all patients by total of 5.0±1.6 ml ethanol infusion without any complication during the procedure. During follow‐up period, targeted VA recurred in 3 patients.

**Conclusions:** Chemical ablation by ethanol infusion was effective alternative therapy for OTVAs with epicardial or intramural site of origin in the vicinity of the LV summit refractory to conventional RF ablation.

## INFECTION RISK WITH IPSILATERAL OR CONTRALATERAL VASCULAR ACCESS IN BREAST CANCER PATIENTS: IMPLICATIONS FOR CIED DECISION MAKING

### DEENA SUKHON^1^, KALDEEP SHAH^2^, KETAN TAMIRISA^3^, ISHMAEL JAIYESIMI^4^, MEHTA NISHAKI^5^, **NOLAN SHOUKRI** ^1^

#### ^1^Oakland University William Beaumont School of Medicine, Auburn Hills, MI,^2^MercyOne Siouxland Heart and Vascular Center, Sioux City, IA,^3^Caroll Independent School District, Southlake, TX,^4^Corewell Health William Beaumont University Hospital, Cancer Center, Royal Oak, MI,^5^Corewell Health William Beaumont University Hospital, Department of Cardiovascular Medicine, Royal Oak, MI

**Introduction:** Breast cancer survivors face an elevated risk of both surgical site infections (SSI) after mastectomy. Lymph node disruption can impact CIED placement and most electrophysiologists avoid ipsilateral CIED placement for theoretical concern of increased infection risk. This meta‐analysis seeks to improve understanding of infection risk in this patient cohort.

**Methods:** We conducted a systematic review and meta‐analysis of studies reporting infectious complications following ipsilateral or contralateral vascular access in breast cancer patients undergoing treatment. Relevant articles were identified through comprehensive searches of PubMed and other databases, and screened based on predefined eligibility criteria.

**Results:** The meta‐analysis included seven studies that analyzed a total of 8,974 intravenous (IV) placements for breast cancer patients over various time frames ranging from 2000 to 2018. The overall heterogeneity of the studies was 98%. The meta‐analysis found no statistically significant difference in the risk of infectious complications between ipsilateral and contralateral vascular access sites.The types of vascular access included central venous ports, intravenous lines (IVs), subcutaneous port catheters (SPCs), needle punctures or intravenous injections, chest wall ports, and subclavian port catheter implantations. The pooled incidence of complications was similar with ipsilateral access: 4.36% (95% CI: 0.07‐12.58) and with contralateral access: 6.47% (95% CI: 0‐42.08) (Figure 1).

**Conclusions:** This analysis suggests that the risk of infection may be similar in ipsilateral and contralateral access. While none of the patients had CIED, the signals from this study could potentially be further investigated in a selected CIED cohort.

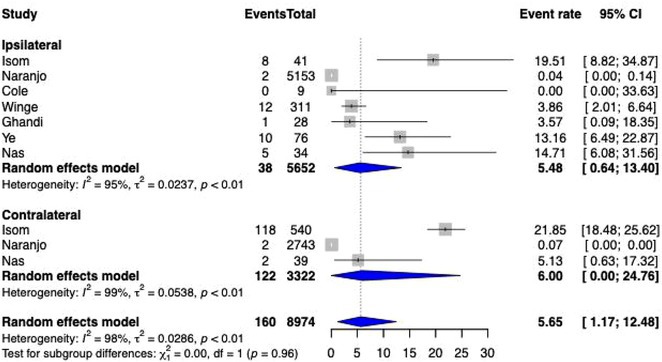

## NATURAL HISTORY OF PERI‐DEVICE LEAKS IN PATIENTS UNDERGOING PERCUTANEOUS LEFT ATRIAL APPENDAGE OCCLUSION THERAPIES

### KARAN CHHABRA^1^, KATERYNA STRUBCHEVSKA^1^, ELVIS CAMI^1^, NISHAKI MEHTA^1^, **NOLAN SHOUKRI** ^2^

#### ^1^Corewell Health East William Beaumont University Hospital, Royal Oak, MI,^2^Oakland University William Beaumont School of Medicine, Royal Oak, MI

**Introduction:** Left atrial appendage occlusion is an alternative approach to reduce cardioembolic risk in patients with nonvalvular atrial fibrillation. The incidence of peri‐device leaks within 6 months of introduction of the Amulet device in the US was compared to Watchman FLX.

**Methods:** We conducted a retrospective analysis of a database comprising of patients undergoing percutaneous Left atrial appendage occlusion procedures from January 2022 to December 2022. All patients had atrial fibrillation with a CHADS2 Vasc score of at least 2. A total of 131 patients were included in the analysis.

**Results:** Out of the 131 patients, 32 (24.4%) had peri‐device leaks on follow up. Amongst the patients with leaks, 20 (62.5%) of them had the Watchman implant and 12 (37.5%) received the Amulet device. There was no correlation between the presence or absence of leak with the type of device that was used for left atrial appendage occlusion (p‐value was 0.69). Mean size of leaks was 2.26±0.385. None of the 32 patients with the leaks had ischemic stroke on follow up. This may be because all the leaks were sized under 5 mm and leaks smaller than 5 mm have not been associated with higher incidence of stroke. Amongst the 32 patients with peri‐device leaks, only 2 (6.25%) had extension of anticoagulation/anti‐platelet therapy due to leaks. In both the cases, size of the leak was between 3‐5 mm.

**Conclusions:** Peri‐device leaks less than 3 mm do not increase the risk of ischemic stroke in patients undergoing percutaneous left atrial appendage occlusion procedure. For leaks ranging from 3‐5 mm, duration of anti‐platelet or anticoagulation may be extended based on a case‐to‐case basis.

## NOVEL HEMATOMA PREVENTION DEVICE

### **NOLAN SHOUKRI** ^1^, NISHAKI MEHTA^2^

#### ^1^Oakland University William Beaumont School of Medicine, Rochester, MI,^2^Corewell Health William Beaumont University Hospital, Royal Oak, MI

**Introduction:** Pocket hematomas are a complication of cardiac internal electronic device (CIED) placement, that has risen to a high frequency over the years, with articles citing it to be in the 20‐25% range of reoperations on CIEDs. PressRite is a novel compression device developed based on nursing technician and physician feedback. This project assessed initial volunteer testing performance of the PressRite prototype.

**Methods:** The prototypes were placed on two volunteers at three positions, left upper pectoral (LUP), left lower pectoral (LLP) and right upper pectoral (RUP), and inflated with a pressure sensor device underneath for two‐hour sessions. Images are taken and analyzed before and after the session to compare skin changes. A survey is given to collect feedback on device comfort, pain and how they felt during the session. Data for pressure over time, and ability to deliver adequate pressure.

**Results:** Device tests so far have shown good ability to maintain adequate pressure 100% of the time at >40 mmHg proven by external sensor. Volunteer feedback rates pain as 0/10 with no significant discomfort throughout the testing with on average 2/10 pain (Ranging from 0‐4) in device removal expressed to be similar to bandage removal. All volunteers expressed willingness to use the device if needed for their own health. Visual analysis showed no visible skin damage.

**Conclusions:** There were no complications with device application with reliable consistent pressure. Further volunteer trials are planned to gather more feedback for use in improving future device prototypes prior to trials with patients.

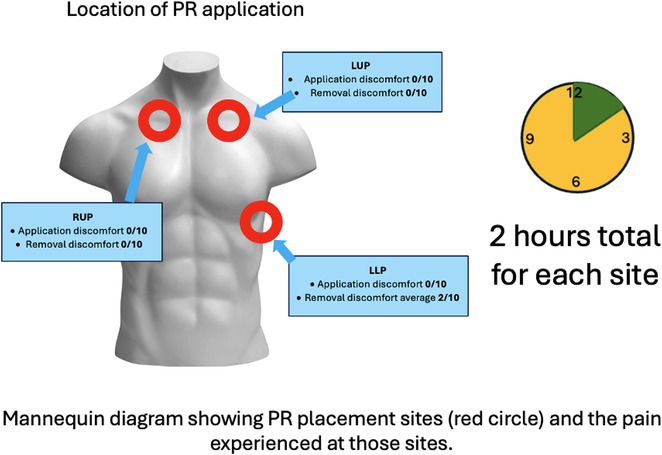

## PREDICTORS OF ISCHEMIC STROKES AFTER PERCUTANEOUS LEFT ATRIAL APPENDAGE OCCLUSION

### KARAN CHHABRA^1^, KATERYNA STRUBCHEVSKA^1^, ELVIS CAMI^1^, NISHAKI MEHTA^1^, **NOLAN SHOUKRI** ^2^

#### ^1^Corewell Health East William Beaumont University Hospital, Royal Oak, MI,^2^Oakland University William Beaumont School of Medicine, Royal Oak, MI

**Introduction:** Left atrial appendage occlusion is an alternative approach to reduce cardioembolic risk in patients with nonvalvular atrial fibrillation. In a real‐life scenario, spontaneous echo contrast (SEC), stroke risk was compared between Watchman FLX and Amulet devices.

**Methods:** We conducted a retrospective analysis of a database comprising of patients undergoing percutaneous left atrial appendage occlusion procedures from January 2022 to December 2022. All patients had atrial fibrillation with a CHADS2 Vasc score of at least 2. A total of 131 patients were included in the analysis. Of these, 78 (59.5%) had Watchman FLX implants and the rest 53 (40.5%) had Amulet devices.

**Results:** Of the 131 patients, 2 patients (1.52%) were found to have had ischemic stroke on follow up. 10 (7.6%) patients had presence of spontaneous echo contrast on initial transesophageal echocardiogram. Out of the 10 patients with spontaneous echo contrast (see image 1), one developed ischemic stroke (10%). One of the remaining 121 patients (0.8%) without spontaneous echo contrast developed ischemic stroke. There was a significant correlation between the presence of SEC and the development of stroke; p‐value = 0.023 (<0.05) and the chi‐square statistic being 5.17. Also, the mean CHADS2 Vasc score of patients with SEC was significantly greater than those without SEC on TEE (4.8±0.82 and 4.06±0.23 respectively; p‐value being 0.045).The mean CHADS2 Vasc score for patients without ischemic stroke was 4.05 ± 0.22.The mean CHADS2 Vasc score for patients with ischemic stroke was 7. This difference in mean CHADS2 Vasc score was considered significant when using the unpaired t‐test (two‐tailed p value was 0.0013). For the two patients with stroke, one of them did not have a history of prior stroke, although this patient did have prior history of transient ischemic attack (TIA). The second patient had a prior history of ischemic stroke.

**Conclusions:** The presence of SEC strongly portends stroke risk which can occur early or late. Defining SEC characteristics to risk stratify stroke probability needs concerted effort with our imaging colleagues.

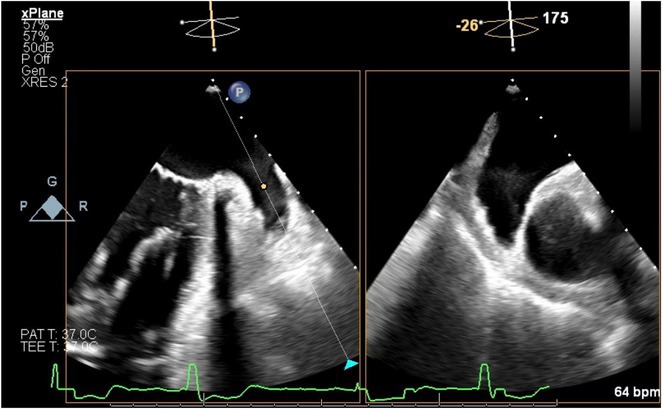

## QUALITY OF LIFE SURVEY OF PATIENTS LIVING WITH CARDIAC IMPLANTABLE ELECTRONIC DEVICES

### RIYA CHHABRA^1^, DIEDRE BRUNK^2^, ILANA KUTINSKY^2^, BRIAN WILLIAMSON^2^, DAVID CRAGG^3^, MICHAEL GALLAGHER^2^, AKHIL GULATI^2^, WILLIAM DEVLIN^2^, DAVID HAINES^2^, NISHAKI MEHTA^2^, **NOLAN SHOUKRI** ^1^

#### ^1^Oakland University William Beaumont School of Medicine, Rochester, MI,^2^Corewell Health William Beaumont University Hospital, Royal Oak, MI,^3^Michigan Heart Group, Troy, MI

**Introduction:** The number of pacemakers and defibrillators (ICD) is increasing every year. However, the long‐term quality of life impact of indwelling cardiac implantable electronic devices (CIED) has not been systematically studied. The goal of this study is to look at lifestyle perception among patients after acute and chronic implants.

**Methods:** As a quality improvement initiative, we conducted interviews over 6 months (July‐December 2023) in patients with transvenous (TV) and subcutaneous (SICD) devices in 2 ambulatory settings. Recent implants were defined as within 2 years of implant. The survey was categorized into 6 categories: 1. cosmetic appearance, 2. comfort level, 3. ability to move, 4. quality of sleep, 5. healing and scar process, and 6. restriction with clothing choices. Patients were asked to rate each of these questions on a scale from 1‐5 (1= very unsatisfied to 5= very satisfied).

**Results:** One hundred and two interviews were completed in person. Sixty‐four pacemakers and 38 ICDs (33 TV ICD and 5 SICD) were included. There were 56 men (55%) and 46 women. The median age of men and women were similar (74.6 and 76.6 years, respectively (p=‐NS)). The average time since implant was 4.3 years (1mo‐12 years). There was equal distribution of recent and remote implants in men and women. The overall score across all categories was 4.64. (men 4.70 and women 4.56 (p=‐NS)). There was no difference reported between men and women for cosmetic appearance (4.50 vs 4.35), comfort level (4.54 vs 4.57), ability to move (4.80 vs 4.93), and quality of sleep (4.77 vs 4.61). However, women reported higher discontentment with the healing process (4.41 vs 4.82, p= 0.0029) and greater restriction with clothing choices (4.50 vs 4.75, p=0.038).

**Conclusions:** There was minimal impact on overall QOL in this study. However, women perceived poor healing and expressed more limitations for clothing choices following CIED implants. This survey may improve focused pre‐device counseling and post‐procedure adjustment to living with CIED.

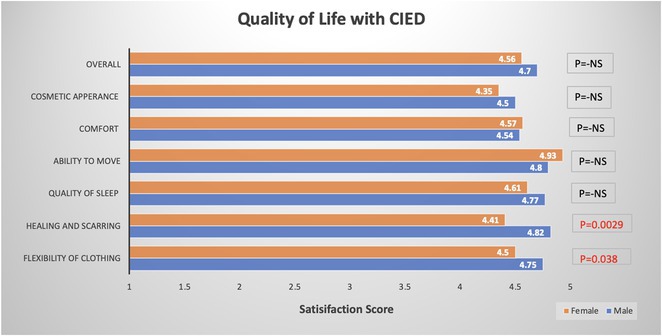

## RADIATION USE IN LEFT ATRIAL APPENDAGE OCCLUSION PROCEDURES: A COMPARISON BETWEEN ELECTROPHYSIOLOGY AND STRUCTURAL CARDIOLOGY OPERATORS

### KARAN CHHABRA^1^, ZACHARY DEMERTZIS^1^, ELA AHMAD^1^, **NOLAN SHOUKRI** ^2^, DAVID HAINES^1^, NISHAKI MEHTA^1^

#### ^1^Corewell Health William Beaumont University Hospital, Royal Oak, MI,^2^Oakland University William Beaumont School of Medicine, Auburn Hills, MI

**Introduction:** Radiation exposure in the cardiac procedure laboratory is a hazard to the patient and the health care team. There is a large focus on radiation reduction in interventional and structural cardiology (SC) societies in contrast to electrophysiology (EP). While most procedures use minimal radiation in EP, atrial appendage occlusion (LAAO) devices require use of fluoroscopy. We sought to understand radiation usage among EP and SC for LAAO devices given lower focus on radiation protection in EP.

**Methods:** Single‐center, retrospective analysis of all LAAO procedures since 2017, averaging procedure duration, fluoroscopy time, air kerma dosing, and dose area product by operator specialty. We excluded cases that had additional procedures done during LAAO.

**Results:** Total of 476 LAAO cases analyzed, 276 performed by EP and 200 by SC. Patient demographics (age, sex, and BMI) were similar in both groups. Procedure duration was similar between operator specialties (60 vs 62 min). On average, EP had less fluoro time (11 vs 15 min), air kerma dosing (351 vs 537 mGy), and dose area product (4365.51 vs 7602.13 uGy‐m2). Complications rates (para‐device leak) were similar between both groups. On multivariate analysis, operator specialty (SC, (OR: 3.16, 95% CI:2.03‐5.01, p<0.001)), male gender (OR: 7.5, 95% CI:4.69‐ 12.4, p<0.001), BMI (OR: 1.17, 95% CI: 1.13‐1.23, p<0.001) were independent predictors of higher DAP during the procedure.

**Conclusions:** Radiation usage was found to be significantly less amongst EP compared to structural cardiologists after adjusting for other variables. This may be from transseptal access familiarity and may represent a fertile area for collaboration to lower patient and team radiation.

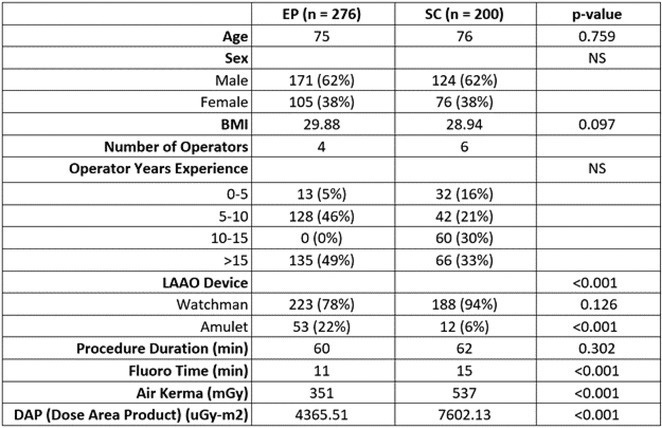

## ECG PREDICTORS OF CLINICAL OUTCOMES IN HEART FAILURE PATIENTS AFTER LEFT BUNDLE BRANCH AREA PACING

### **JENISH SHROFF** ^1^, ANUGRAH NAIR^1^, LUKAH Q TUAN^1^, ADRIANA TOKICH^1^, NATASHA JONES‐LEWIS^2^, LORI BELL^2^, LARA HEDLEY^1^, RAJEEV K PATHAK^1^

#### ^1^Canberra Heart Rhythm, Canberra Heart Rhythm Foundation, Australian National University, Canberra, Australia,^2^Canberra Heart Rhythm, Canberra Heart Rhythm Foundation, Canberra, Australia

**Introduction:** Left‐bundle branch area pacing (LBBAP) has been evaluated as an alternative means of effecting cardiac resynchronization therapy (CRT). Paced QRS morphology may vary depending upon the pacing location. Association between paced QRS morphology and clinical outcomes is unclear. We aimed to evaluate ECG predictors of clinical outcomes in heart failure patients with class I CRT indication.

**Methods:** 59 of 79 LBBAP recipients were analysed after exclusions. LBBAP was performed using Medtronic 3830 lead. Patients were categorized into various groups based on paced QRS in lead V1 (qR and Qr) and paced QRS axis (normal, left or right) to compare clinical outcomes.

**Results:** Improvement in left ventricular ejection fraction (LVEF) was significantly greater in qR group (21.4±6.4 vs 16.4±8.3%, P=0.01) at 12‐months compared to Qr group. Paced R‐wave peak time (RWPT) was significantly longer in Qr arm (85.3±11.3 vs 75.7±17.5ms, P=0.014) and transition during threshold test was observed less frequently (28% vs 55%, P=0.036). V6RWPT, LVEF or left ventricular strain improvement did not differ in patients with different paced QRS axis (all P>0.05). Non‐responders had grater incidence of loss of ‘R’ prime (54.5% vs 10.5%, P=0.01) and prolongation of V6RWPT (11.8±11.3 vs ‐0.3±6.8ms, P=0.001) on follow up compared to super‐responders.

**Conclusions:** Paced ‘qR’ in lead V1 predicted greater improvement in LVEF as compared to ‘Qr’ which was more often associated with left ventricular septal pacing. Paced QRS axis did not determine clinical outcomes. Loss of terminal ‘R’ in lead V1 and prolongation of RWPT prognosticated non‐response to LBBAP.

## ELECTROCARDIOGRAPHIC AND ECHOCARDIOGRAPHIC RESPONSE TO LEFT BUNDLE BRANCH AREA PACING IN PATIENTS WITH CARDIAC MAGNETIC RESONANCE DOCUMENTED SCAR

### **JENISH SHROFF** ^1^, ANUGRAH NAIR^1^, LUKAH Q TUAN^1^, ADRIANA TOKICH^1^, NATASHA LEWIS‐JONES^2^, LORI BELL^2^, LARA HEDLEY^1^, RAJEEV K PATHAK^1^

#### ^1^Canberra Heart Rhythm, Canberra Heart Rhythm Foundation, Australian National University, Canberra, Australia,^2^Canberra Heart Rhythm, Canberra Heart Rhythm Foundation, Canberra, Australia

**Introduction:** Left bundle branch area pacing (LBBAP) has been evaluated for cardiac resynchronization therapy. However, impact of myocardial scar on clinical outcomes is still debated. This study aims at evaluating the association between scar burden on cardiac magnetic resonance (CMR) and response to LBBAP‐CRT in heart failure patients with left ventricular ejection fraction (LVEF) ≤35%.

**Methods:** 60 patients who underwent CMR followed by LBBAP were included. Patients were categorized into ≥10% (n=23) vs <10% late Gadolinium enhancement (LGE) (n=37) groups. The location of LGE was classified as septal, left ventricular (LV) free wall, or occurring in both locations. Post‐implant electrocardiographic parameters and change in echocardiographic parameters at 12‐months were analysed.

**Results:** Mean follow‐up was 27±12 months. Baseline characteristics including type of dyssynchrony, and cardiomyopathy were not significantly different (all P>0.05). 43.5% had septal + LV free wall, 39% had septal and 17.5% had LV free wall scar in ≥10% LGE group. 6 patients with septal scar in ≥10% LGE group crossed over from biventricular CRT. Baseline (157.3±20.9 vs 160.6±25.9ms, P=0.9) and post‐implant QRS duration (131.2±11.5 vs 129.9±17.3ms, P=0.8) and R‐wave peak time (78.6±15.1 vs 77.2±17.9ms, P=0.8) were not significantly different in ≥10% vs <10% LGE cohorts, respectively. While baseline LVEF (29.3±5.9 vs 30.4±5.4%, P=0.5) was comparable, 12‐month LVEF was significantly better in <10% LGE group (50.7±7.2 vs 45±7.8%, P<0.001).

**Conclusions:** Lower scar burden on CMR was associated with greater improvement in LVEF. CMR facilitated assessment of potential responders to LBBAP‐CRT. Furthermore, septal scar may not necessarily preclude LBBAP.

## OUTCOMES OF LEFT BUNDLE BRANCH AREA PACING IN HEART FAILURE PATIENTS WITH CARDIAC RESYNCHRONIZATION THERAPY

### **JENISH SHROFF** ^1^, ANUGRAH@CHRC.NET.AUUGRAH NAIR^1^, LUKAH Q TUAN^1^, ADRIANA TOKICH^1^, NATASHA JONES‐LEWIS^2^, LORI BELL^2^, LARA HEDLEY^1^, RAJEEV K PATHAK^1^

#### ^1^Canberra Heart Rhythm, Canberra Heart Rhythm Foundation, Australian National University, Canberra, Australia,^2^Canberra Heart Rhythm, Canberra Heart Rhythm Foundation, Canberra, Australia

**Introduction:** Left bundle branch area pacing (LBBAP) is rapidly emerging as an alternative to biventricular pacing for cardiac resynchronization therapy (CRT). Data regarding outcomes in long‐term is scarce. We aim to report long‐term outcome data of LBBAP for CRT.

**Methods:** 60 patients with left ventricular ejection fraction (LVEF) <35% and QRS >130ms were included in this prospective registry. LBBAP was performed using Medtronic's 3830 lead. Change in QRS duration (QRSd), echocardiographic parameters, NYHA class and quality of life (QoL) were analysed.

**Results:** Mean follow‐up was 26±8 months. 6 (10%) patients had ischemic cardiomyopathy. 55 (92%) patients had left bundle branch block. Baseline QRSd was 161.3±24.3ms which narrowed to 130.4±15.4ms post‐implant (P<0.001). Significant improvement in LVEF (29.5±5.2% to 50.4 ±10.3%, P<0.001), NYHA class (2.8±0.7 to 1.4±0.6, P<0.001) and EQ‐5D‐5L QoL index (41.7±18.2 to 73.1±18.1, P<0.001) was observed. Left ventricular end‐systolic volume and left atrial volume reduced from 113.6±45.4ml to 68±26.6ml (P<0.001) and 102.8±41.8ml to 87.2±38.2ml (P=0.03), respectively. 20 (33%) were super‐responders and 11 (18.3%) were non‐responders based on LVEF improvement of ≥20% and <10%, respectively. Mean pacing threshold was stable at 2‐years (0.75±0.4V vs 0.65±0.3V at baseline, P=0.1). 8 (13%) patients had loss of LBBAP at final follow‐up evident by loss of terminal ‘R’ in lead V1 and prolongation of R wave peak time.

**Conclusions:** LBBAP was associated with remarkable improvement in LVEF, symptoms and QoL in heart failure patients. While one‐third of patients had super‐response, loss of CRT and non‐response were also observed in small subset of patients.

## OUTCOMES OF LEFT BUNDLE BRANCH AREA PACING IN PATIENTS WITH MILD‐MODERATELY REDUCED EJECTION FRACTION

### **JENISH SHROFF** ^1^, ANUGRAH@CHRC.NET.AUUGRAH NAIR^1^, LUKAH Q TUAN^1^, ADRIANA TOKICH^1^, NATASHA JONES‐LEWIS^2^, LORI BELL^2^, LARA HEDLEY^1^, RAJEEV K PATHAK^1^

#### ^1^Canberra Heart Rhythm, Canberra Heart Rhythm Foundation, Australian National University, Canberra, Australia,^2^Canberra Heart Rhythm, Canberra Heart Rhythm Foundation, Canberra, Australia

**Introduction:** Left bundle branch area pacing (LBBAP) has become an alternative for cardiac resynchronization therapy. Its role in heart failure patients with mid‐moderately reduced left ventricular ejection fraction (LVEF) is still being explored. We aim to report the clinical outcomes of LBBAP in this subset of patients.

**Methods:** 51 patients with LVEF of 36‐50% were included in this prospective registry after 4 exclusions who moved interstate/overseas. LBBAP was performed using Medtronic's 3830 lumenless lead with C315HIS delivery sheath. Change in QRS duration (QRSd), echocardiographic parameters, NYHA class and quality of life (QoL) were analysed.

**Results:** Mean follow‐up was 23±8 months. 9 (17.6%) patients had ischemic cardiomyopathy. Sick sinus syndrome, symptomatic bradycardia, heart block were the major indications for permanent pacemaker. 4 patients had permanent atrial fibrillation requiring AV node ablation. 29 (57%) patients had narrow QRS complex while 22 patients had dyssynchrony [17 (33%) with left and 5 (10%) with right bundle branch block]. Post‐pacing QRSd was not significantly different compared to baseline (133.9±25.9 vs 131.5±32.5ms, P=0.7). Mean pacing was 83.5±30.2%. LVEF improved from 42.6±5.4% to 51.7±.6% (P<0.001) and indexed left ventricular end‐systolic volume (LVESVi) reduced from 34±11 to 27±12ml (P=0.003). Left atrial volume did not change significantly (44.6±16.6 vs 43.9±5.5ml, P=0.7). NYHA class improved from 2±0.7 to 1.5±.7 (P<0.001). EQ‐5D‐5L QoL index improved from 63±12 to 74±14.

**Conclusions:** LBBAP was associated with significant improvement in LVEF and reduction in LVESVi in patients with mild‐moderately reduced LVEF which resulted into improvement in symptoms and meaningful gain in QoL.

## RISK OF ARRHYTHMIA IN BIVENTRICULAR PACING VS LEFT BUNDLE BRANCH AREA PACING

### **JENISH P SHROFF** ^1^, ANUGRAH NAIR^1^, LUKAH Q TUAN^1^, ADRIANA TOKICH^1^, NATASHA LEWIS‐JONES^2^, LORI BELL^2^, LARA HEDLEY^1^, RAJEEV K PATHAK^1^

#### ^1^Canberra Heart Rhythm, Canberra Heart Rhythm Foundation, Australian National University, Canberra, Australia,^2^Canberra Heart Rhythm, Canberra Heart Rhythm Foundation, Canberra, Australia

**Introduction:** Arrhythmia is associated with significant morbidity and mortality in HF patients. Left bundle branch area pacing (LBBAP) has emerged as an alternative to biventricular pacing (BVP). However, data around arrhythmia occurrence in patients with LBBAP vs BVP is scarce. This study aims at evaluating the occurrence of atrial and ventricular arrhythmia in HF patients with LBBAP in comparison to BVP.

**Methods:** 62 patients with LBBAP and 51 with BVP‐CRT were included in this prospective study after excluding patients with pre‐existing atrial and ventricular arrhythmia. Incidence of atrial fibrillation (AF), atrial flutter, atrial tachycardia (AT), non‐sustained and sustained ventricular tachycardia (VT), ventricular fibrillation (VF), >5% paroxysmal ventricular complexes (PVC), AF/atrial flutter ablation and PVC/VT ablation post‐CRT were recorded. Fisher's exact test was used to compare frequencies.

**Results:** Mean follow‐up was 33±11 months. Incidence of AF was significantly higher in BVP cohort (15% vs 4%, P=0.04). Incidence of atrial flutter (3.9% vs 3.2%, P=0.8), AT (6.4% vs 5.9%, P=0.4), sustained VT/VF (4.8% vs 11.5%, P=0.2), non‐sustained VT (19.4% vs 19.2%, P=0.8), >5% PVC (6.4% vs 11.5%, P=0.3), AF/atrial flutter ablation (1.6% vs 5.8%, P=0.2) was not significantly different in LBBAP vs BVP groups, respectively. Greater number of PVC/VT ablations was recorded in BVP cohort (23% vs 5%, P=0.02).

**Conclusions:** LBBAP was associated with lower incidence of new onset AF compared with BVP. Trend towards higher incidence of ventricular arrhythmia was seen in BVP cohort. Significantly greater number ablations for VT were recorded in BVP cohort.

## EVALUATION OF A SHORT DURATION HEAD UP TILT TABLE TEST PROTOCOL AND ITS ROLE IN CONTEMPORARY CLINICAL PRACTICE

### **IBRAHIM SHUGMAN** ^1,2^, TU HAO TRAN^1,2^, KENNETH CHO^1,2^, PHOEBE GANIS^3^, KAVIE SOOSAPILLA^1,2^, DANIEL AKARAWI^1,2^, JONATHAN HOOPER^1^, ZAIDON AL‐FALAHI^1,2^, KRISHNA K KADAPPU^1,2^, TAMER NAGUIB BADIE^1,2^, HASHIM KACHWALLA^1,2^, TUAN NGUYEN^1,2^, UPUL PREMAWARDHANA^1,2^

#### ^1^Cardiology Department, Campbelltown Hospital, Campbelltown, Sydney, NSW, Australia,^2^Western Sydney University, Campbelltown, Sydney, NSW, Australia,^3^Cardiology Department, Liverpool Hospital, Liverpool, Sydney, NSW, Australia

**Introduction:** The head‐up tilt‐table test (HUTT) is a valuable diagnostic tool for evaluating patients with orthostatic symptoms especially when clinical history fails to offer a definitive explanation for presyncope and syncope. This study aims to evaluate single‐centre experience using short‐duration HUTT.

**Methods:** All consecutive patients (n=104; 81% female) who underwent HUTT at Campbelltown Hospital from June‐2019 to March‐2024 were included in this study.

**Results:** The median age was 32 years (IQR: 23‐46). The referrals for HUTT included syncope (n=64 [61.5%]), presyncope (n=30 [28.8%]), and the evaluation of postural orthostatic tachycardia syndrome (POTS)(n=10 [9.6%]). The HUTT involved a 5‐minute supine equilibration phase, followed by a 20‐minute passive tilt phase at an angle of 70‐degrees. Subsequently, a provocation phase at 70‐degrees with the administration of sublingual Glyceryl trinitrate (GTN) if needed. HUTT was abnormal in 29(28%) patients, including orthostatic hypotension (n=10), POTS (n=6), vasovagal syncope (total=9;cardioinhibitory n=5]), and syncope without haemodynamic changes (n=4). Among the 64 patients referred for syncope evaluation, 21(33%) patients exhibited abnormal HUTT results. In cases of vasovagal syncope (n=9), GTN provocation was necessary for diagnosis in four patients. No adverse events occurred during HUTT. Recommendations, such as lifestyle adjustments, utilization of compression stockings, or initiation of drug therapy, were provided to all patients with abnormal HUTT results.

**Conclusions:** Short‐duration HUTT continues to be a valuable tool for evaluating patients with presyncope, syncope or POTS. Utilizing short‐duration HUTT with GTN provocation in syncope patients has the potential to enhance test sensitivity in detecting vasovagal syncope.

EVALUATING THE SAFETY AND EFFICACY OF THE AMPLATZER AMULET LAA OCCLUDER FOR STROKE PROPHYLAXIS WITHOUT PRE‐PROCEDURAL CHEST CT: A SINGLE‐CENTER’S EXPERIENCE

**USMAN SIDDIQUI** ^1^, AQEEL KHANANI^2^;

^1^AdventHealth Florida, Orlando, FL,^2^Lakeland Regional Health, Lakeland, FL.

**Introduction:** Left atrial appendage (LAA) closure has emerged as an alternative in stroke prophylaxis in patients with AF. The Amulet LAA Occluder is one such occlusion device. Pre‐procedural CT is routinely used prior to Amulet placement. Here, we share results of our 83 cases of Amulet implantation without pre‐procedural CT. Number of devices used per procedure, bleeding & procedural complications, and incidences of hospitalizations, stroke, and embolism were followed.

**Methods:** 83 AF patients underwent LAA closure with Amulet. Average age was 76.7 years, and 54.2% were male. The device was implanted with intraprocedural TEE, intracardiac left atrial echocardiography, and intraprocedural angiography. Pre‐procedural CT was not used. Patients were followed post‐procedure. Primary safety endpoints were rates of procedural/bleeding complications. Primary efficacy endpoints included rates of stroke and embolism, and number of devices used during the procedure.

**Results:** Patients had early ambulation with closure. There were no perioperative complications. Two patients experienced post‐procedure pericardial effusion. 1 was symptomatic 5‐days post‐procedure, while the other after 3.5‐weeks. In another case, the device shifted proximally and was eventually removed during a Mitral repair. There were no cases of stroke or embolism following implantation. 73 cases required only 1 device, whereas 9 cases required 2 devices; 1 case required 4 devices to fit. Average of 1.14 devices per case. 96.4% of cases were successful with no complications.

**Conclusions:** Amulet is an effective alternative to oral anticoagulation (OAC) for stroke prophylaxis in AF patients. There were no stroke or emboli in the peri‐ or post‐procedural period. In 83 cases, 2 had instances of pericardial effusion post‐procedure. In a 3^rd^ case, the device shifted and was removed. 96.4% of cases were successful with no peri‐ or post‐procedural complications. Findings suggest that the Amulet is a viable alternative to OAC and can be safely placed without pre‐procedural CT. Larger studies are recommended to further evaluate.

## A CHANGE OF HEART: FROM CARDIAC RESYNCHRONISATION THERAPY‐DEFIBRILLATOR (CRT‐D) TO DUAL CHAMBER IMPLANTABLE CARDIAC DEFIBRILLATOR (DC‐ICD) IMPLANTATION IN A GENTLEMAN WITH A BIG HEART

### **JIAN HAO SIM** ^1^, ABDUL RAQIB ABD GHANI^2^, GURUDEVAN MAHADEVAN^1^, CHIEH YEE KHER^1^, KIM FONG NG^1^

#### ^1^Hospital Sultanah Aminah, Johor Bahru, Malaysia,^2^Hospital Sultan Idris Shah, Serdang, Malaysia

**Introduction:** CRT is recommended for patients with LVEF<40%, regardless of NYHA class, with indication for ventricular pacing and high degree atrioventricular (AV) block.

**Methods:** N/A

**Results:** 71 years old gentleman with triple vessel coronary artery disease, who had CABG in 2016, presented with dizziness for 1 month. ECG: complete heart block, QRS 132ms. Echo: LVEF 30‐35%, global hypokinesia, dilated LA and LV, mild MR, mild AR, poor RV function, TAPSE 10mm. He was planned for CRT‐D. After left axillary vein puncture, we struggled to advance the pacing lead through a severe TR, encountered during the procedure, causing instability, and failed septal pacing. We had to change our strategy. We positioned the pacing lead at the Right Ventricular (RV) apex and cannulate the coronary sinus (CS) using steerable CS Electrophysiology catheter within Selectra extended hook outer sheath. However, coronary sinus balloon venography revealed unfavourable CS tributaries. We failed to subselect the very tortuous lateral vein using 0.014”ASAHI SION® Blue coronary guidewire. Left bundle branch area pacing attempted as bailout solution, but the conduction system pacing sheath Selectra 3D 55/39 failed to reach beyond the tricuspid annulus due to dilated heart; a lateral axillary vein puncture site also made it challenging for longer sheath to reach the septum. Eventually, we decided for Dual Chamber ICD. ECG during implantation: sinus rhythm, PR interval 357ms, QRS 140ms. AAI pacing showed 2:1 AV block at 70 bpm. RV pacing should be minimized. Nevertheless, for our patient with AV node disease and pacing QRS of 185ms, we set his ICD in VDD 50/100 instead of VVI or VDI as it is important to pace and maintain his AV synchrony. DDD mode was not selected due to intact sinus node.

**Conclusions:** CIEDs implantation can be challenging in heart failure patients, where anatomy is usually modified. CRT‐D may provide better outcomes for our patient. However, due to the unfavourable circumstances, we had a change of heart and implanted a Dual Chamber ICD, which we believe will be life saving for our patient.

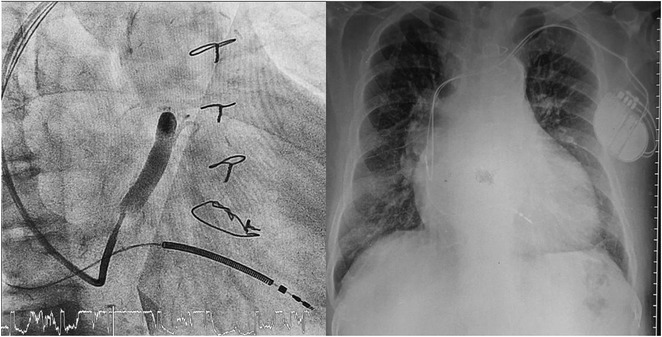

## EVALUATING THE PROGNOSTIC UTILITY OF CHA_2_DS_2_‐VASC SCORES IN PATIENTS WITHOUT ATRIAL FIBRILLATION FOR COGNITIVE DECLINE, CEREBROVASCULAR DISEASE PROGRESSION AND CLINICAL OUTCOMES

### **MING ANN SIM** ^1,2^, EUGENE TAN^1,3,4^, SIEW PANG CHAN^1,3^, SAIMA HILAL^1^, CHRISTOPHER CHEN^1^, LIENG‐HSI LING^1,3^

#### ^1^National University of Singapore, Singapore, Singapore,^2^National University Hospital, Singapore, Singapore,^3^National University Heart Centre, Singapore, Singapore,^4^Royal Melbourne Hospital, Victoria, Australia

**Introduction:** The prognostic utility of the CHA_2_DS_2_‐VASc score as a clinical biomarker for cognitive decline and cerebrovascular disease in patients without atrial fibrillation (AF) is unclear.

**Methods:** In a longitudinal prospective study of memory clinic subjects without AF, annual neurocognitive assessments and biennial brain magnetic resonance imaging (MRI) scans were performed. Associations of baseline CHA_2_DS_2_‐VASc score with progression of cerebrovascular disease (cortical infarcts, lacunes, cerebral microbleeds [CMB], white matter hyperintensities [ARWMC scores], intracranial stenosis), cognitive decline (Clinical dementia rating Global [CDR‐GS] score ≥0.5 increment from baseline, worsening of neuropsychological domain‐specific Z‐scores), and MACCE (major cardiac and cerebrovascular events defined as mortality, stroke, acute cardiac events) were evaluated.

**Results:** Of 648 subjects (age 72.8±8.0years, 42.9% male), incident MACCE occurred in 117/648 (18.1%), and 242/604 (40.1%) developed cognitive decline over 46.5±17.2 months. Higher CHA_2_DS_2_‐VASc scores associated significantly with progression of cortical infarcts (adjusted risk ratio [ARR] 1.24, 95% C.I. 1.05‐1.46, p=0.013), lacunes (ARR 1.27, 95% C.I. 1.17‐1.38, p<0.0001), CMBs (ARR 1.11, 95% C.I. 1.07‐1.15, p<0.0001) and intracranial stenosis (ARR 1.25, 95%C.I. 1.06‐1.48, p=0.010).Additionally, higher CHA_2_DS_2_‐VASc scores associated with faster cognitive decline in visuoconstruction (β ‐0.282, 95% C.I ‐0.47, ‐0.10, p=0.003), visuomotor (β ‐0.096, 95% C.I ‐0.18, ‐0.02, p=0.019) and memory (β ‐0.203, 95% C.I ‐0.34, ‐0.07, p=0.003) *Z*‐scores.Higher CHA_2_DS_2_‐VASC scores independently predicted increased risk of MACCE (adjusted hazards ratio [AHR] 1.64, 95% C.I. 1.12‐2.39, p=0.011, Figure 1).

**Conclusions:** In older subjects without AF, CHA_2_DS_2_‐VASC scores may be a clinically accessible prognostic biomarker for progression of cognitive decline, cerebrovascular disease and adverse clinical outcomes.

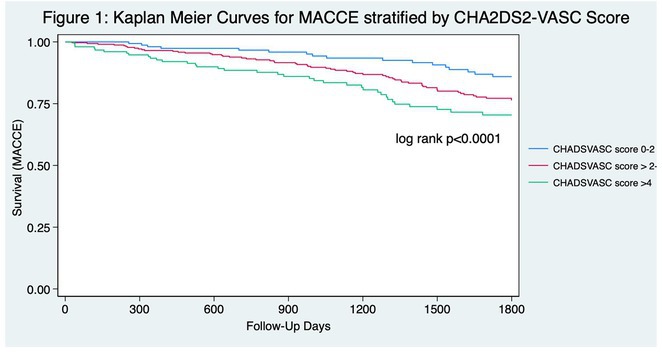

## RECURRENT ATRIAL ARRHYTHMIAS AFTER ATRIAL FIBRILLATION ABLATION USING ELECTROANATOMIC MAPPING SYSTEM AND CIRCUMFERENTIAL PULMONARY VEIN ISOLATION: MECHANISMS AND IMPLICATIONS

### RAHUL SINGHAL

#### Fortis Hospital Jaipur, Jaipur, India

**Introduction:** Atrial Fibrillation (AF) ablation is associated with recurrences whatever modality of ablation is used mainly in persistent AF.Aim was to investigate mechanisms of recurrent atrial tachyarrhythmias(ATs) after ablation using circumferential pulmonary vein isolation(CPVI) utilizing CARTO system and result of re‐ablation.

**Methods:** 16/78 patients after AF ablation using CPVI had recurrent ATs.LA was approached thru transseptal technique.2 lasso catheters were placed in ipsilateral(IL) pulmonary veins(PV).If there was still LA‐PV conduction in both or either ILPV mapped by catheter, LA and PV geometry created by CARTO system.Conduction gaps from previous ablation were mapped and ablated at LA 0.5 to 1.0 cm outside PV ostium.End point defined as absence of all PV spikes documented with lasso within ILPVs. If no PV‐LA conduction confirmed before ablation,isoproterenol administrated to find non‐PV foci.If typical atrial flutter was recorded before/during ablation,CTI line ablation was done to reach end point of bidirectional conduction block. Irrigated RF energy was delivered in all.

**Results:** During repeat procedures in 16,conduction gaps in previous continuous circumferential lesion (CCL) were found in both sides in4,right‐sided CCL in8, left‐sided in4. PV tachycardia via conduction gaps was demonstrated in6.All conduction gaps were successfully ablated by irrigated RF applications in11.In 3 with typical atrial flutter,bidirectional conduction block in RA isthmus was reached.In another 2 without PV‐LA conduction,non‐PV foci in SVC were defined and ablated.After second procedure,15/16 patients were free of ATs during 24 months of follow‐up.

**Conclusions:** Our study showed that following index AF ablation most frequent cause of atrial tachyarrhythmias recurrence was reversion of PV‐LA conduction and RA Atrial flutter was also the likely cause especially in females. Repeated ablation to reach the endpoint of PV isolation may improve the success rate of AF ablation. Also important is to identify and ablate the non‐PV foci to increase the success rate.

## CASE OF RECURRENT MODERATOR BAND PREMATURE VENTRICULAR COMPLEX TREATED WITH PULSE FIELD ABLATION

### **TAI CHUNG SO**, KAIMIN HUANG, SAURABH KUMAR

#### Westmead Hospital, Sydney, Australia

**Introduction:** Management of moderator band(MB) premature ventricular complex(PVC) can be challenging. It often presented as life‐threatening PVC triggered ventricular fibrillation(VF), radiofrequency ablation is difficult due to poor catheter contact and stability. The long MB with surrounding thick myocardial structures rendering ablation of intramural PVC origin ineffective. The PVC is also suppressed by sedation rendering activation map impossible. Thereby given the various exit sites it leads to multiple pace maps and extensive ablation.

**Methods:** N/A

**Results:** A 55 year old man presented with intracardiac defibrillator (ICD) shock. He had history of right ventricular(RV) dysplasia and VF arrest with ICD implanted.

He complained of increased palpation from PVC, which initiated VF and thereby ICD shock. He had MB PVC since 8 years ago and underwent 2 ablations including one epicardial approach, which ablated the lateral RV free wall exit but failed to eliminate the PVC. Antiarrhythmic drugs are neither tolerated or effective. His 12 lead Holter showed 10% PVC burden with left bundle morphology, late transition, left axis and inferior lead discordance.

Re‐do ablation was offered. The procedure was done under general anesthesia with 3D mapping and intracardiacechography. There were spare PVCs with multiple similar morphologies. The pace maps of PVCs were around 90%, located at the myocardial connection between MB and anterior RV free wall. Radiofrequency ablation there was unsuccessful.

Adjuvant pulse field ablation(PFA) was used. A 31mm multielectrode pentaspline catheter was advanced into RV in basket configuration. 28 applications of PFA, comprising a train of five pulses of 2.0 kV at a duration of 2.5 seconds, was performed in the previously ablated area and over the moderator band. At the follow up, there was no more PVC with symptoms improved.

**Conclusions:** This is the first reported case of ablation of moderator band PVC using PFA. In this case success could be due to deeper lesion formation and larger contact area using PFA. This case highlights the challenges of moderator band PVC ablation and potential use of PFA in treating these difficult cases.

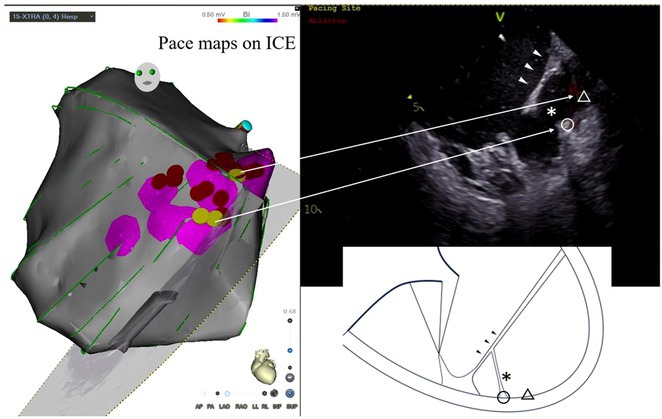

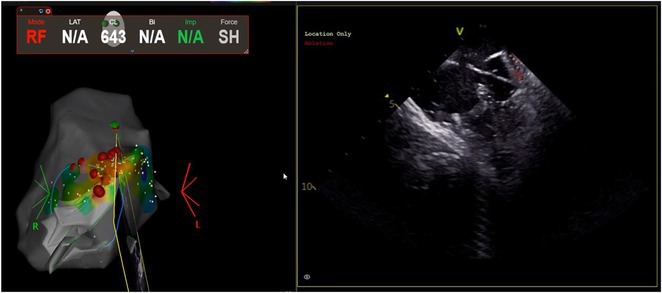

## DETECTION OF ATRIAL REMODELING SITES IN PERSISTENT ATRIAL FIBRILLATION USING ARTIFICIAL INTELLIGENCE

### **YOSHIHIRO SOBUE** ^1^, KAZUYA TAKEDA^1^, TAISHI FUKUSHIMA^1^, SYUN ITO^1^, SYUHEI TAKAHARA^1^, AKANE MIYAZAKI^1^, TAKEHIRO ITO^1^, MASATAKA YOSHINAGA^1^, WAKAYA FUJIWARA^1^, EIICHI WATANABE^1^, HIDEO IZAWA^2^

#### ^1^Fujita Health University Bantane Hospital, Nagoya, Japan,^2^Fujita Health University Hospital, Toyoake, Japan

**Introduction:** While atrial dilation attributed to atrial fibrillation is widely recognized, comprehensive understanding of the remodeling process remains elusive. Insight into the trajectory of remodeling in persistent atrial fibrillation holds potential to optimize ablation efficacy targeted at these sites. Artificial intelligence (AI) has recently emerged as a pivotal asset across various clinical domains. However, its diagnostic performance in distinguishing various types of atrial fibrillation (AF) remains inadequately explored. This study aimed to evaluate the diagnostic accuracy of AI utilizing cardiac computed tomography (CT) for AF type classification and assessment of remodeling progression.

**Methods:** Seventy patients who underwent catheter ablation for AF, comprising 30 with paroxysmal AF and 30 with long‐standing AF, were enrolled. Three‐dimensional reconstructed axial cardiac CT images were employed to train three deep convolutional neural networks (DCNN) using transfer learning for image‐based AF type classification. Subsequently, the diagnostic performance of these DCNN models and GradCAM class activation visualization were evaluated.

**Results:** Among the three DCNN models (ResNet50, ResNet101, ResNet152), ResNet 152 demonstrated superior classification performance, achieving an accuracy of 88.4% for AF type classification. GradCAM class activation visualization highlighted remodeling sites predominantly at the base of the left atrial appendage and anterior wall, with a sensitivity of 79% in persistent AF, while being absent in paroxysmal AF cases.

**Conclusions:** Our study underscores the potential of deep learning utilizing DCNN on cardiac CT images as a valuable tool for both classifying different types of AF and detecting atrial remodeling sites. This approach holds promise for refining ablation strategies and enhancing patient outcomes in persistent atrial fibrillation.

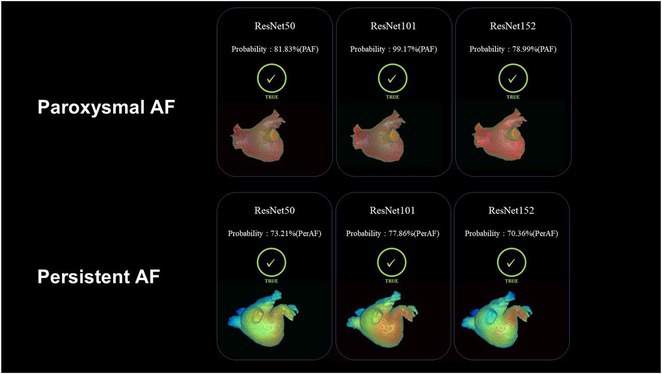

## MID‐TERM PROGNOSIS FOLLOWING ABLATION OF FOCAL AND ROTATIONAL ACTIVATION PATTERNS BEYOND PULMONARY VEIN ISOLATION FOR ATRIAL FIBRILLATION

### **YOSHIHIRO SOBUE** ^1^, TAISHI FUKUSHIMA^1^, SYUHEI TAKAHARA^1^, SYUN ITO^1^, AKANE MIYAZAKI^1^, TAKEHIRO ITO^1^, MASATAKA YOSHINAGA^1^, WAKAYA FUJIWARA^1^, EIICHI WATANABE^1^, HIDEO IZAWA^2^

#### ^1^Fujita Health University Bantane Hospital, Nagoya, Japan,^2^Fujita Health University, Toyoake, Japan

**Introduction:** Pulmonary vein isolation (PVI) stands as a cornerstone ablation technique for atrial fibrillation (AF), yet its efficacy remains constrained. In a bid to augment its effectiveness, we engaged in driver evaluation employing Unipolar electrograms with multipolar electrodes during AF (utilizing CARTO Finder) and delved into the supplementary ablation effects.

**Methods:** We undertook driver evaluation utilizing Unipolar electrograms from multipolar electrodes during AF in 106 cases of persistent atrial fibrillation (PerAF). We delineated Qs patterns manifesting at least twice as Focal patterns (F) and those encompassing 50% or more of the measured area in terms of cycle length as Rotational patterns (R). We targeted those demonstrating high reproducibility for ablation and executed additional ablation at the same site post‐PVI, followed by prognostic evaluation. The endpoint was defined as AF recurrence lasting over 30 seconds, and we prospectively compared its efficacy in cases of paroxysmal atrial fibrillation (PAF) where solely PVI was undertaken in 104 cases.

**Results:** The mean age stood at 69 years, with 74% comprising males. Left atrial diameter averaged at 44 mm, and the typical AF duration was 3 months. The AF non‐recurrence rate at a mean follow‐up of 13 months was 92.8% for PAF and 93.4% for PerAF, with a hazard ratio of 1.34 (95% confidence interval 0.48‐3.65, p=0.56), signifying no significant disparity.

**Conclusions:** Therapeutic interventions supplementary to PVI for PerAF employing the CARTO Finder function exhibit efficacy and harbor the potential to amplify ablation outcomes.

## EFFECT OF ROUTINE EXTERNAL LOOP RECORDER EVALUATION ON THE CARDIOVASCULAR EVENTS AFTER ATRIAL FIBRILLATION ABLATION

### **MASAHIRO SOGO**, KEISUKE OKAWA, YUYA SUDO, AKIHIRO OKA, MASATOMO OZAKI, MASAHIKO TAKAHASHI

#### Kagawa Prefectual Central Hospital, Takamatsu‐shi, Japan

**Introduction:** Asymptomatic recurrences frequently occur after atrial fibrillation (AF) ablation and may lead to serious AF‐related events. External loop recorders (ELRs), which can automatically detect asymptomatic arrhythmic episodes, have been recommended for assessing AF recurrences after ablation. However, the effect of this ELR evaluation on the long‐term outcomes after AF ablation is unknown. This study aimed to explore the prognostic impact of assessing AF recurrences after ablation using ELRs.

**Methods:** We conducted a retrospective cohort study of consecutive patients with paroxysmal AF who underwent a first‐time AF ablation. The patients were routinely evaluated using ELRs at 6 months post‐ablation from January 2015 to June 2018 but the evaluations were discontinued thereafter due to a clinical overload. We divided them into two groups according to the era in which the ELRs were routinely used or not, and compared the 2‐year incidence of the composite outcome of major adverse cardiovascular events (MACE), which included thromboembolisms, heart failure, acute coronary syndrome, major bleeding, and cardiovascular death, between the ELR and non‐ELR era groups.

**Results:** The ELR era group had 407 patients and non‐ELR era group 305. The baseline characteristics were almost the same, except for the estimated glomerular filtration rate, between the groups. The cumulative incidence of MACE was significantly lower in the ELR era group than non‐ ELR era group (3.3% vs. 6.6%, log‐rank test, P = 0.032). After adjusting for MACE‐related factors, a routine ELR evaluation after AF ablation was determined as an independent predictor of the 2‐year MACE (adjusted hazard ratio 0.48, 95% confidence interval: 0.23‐0.99, P = 0.049).

**Conclusions:** Routine evaluations of AF recurrence using ELRs at 6 months after ablation were associated with a lower incidence of the MACE in patients with paroxysmal AF.

## ECHOCARDIOGRAPHY STUDY ON THE IMPACT OF DUAL CHAMBER PACEMAKER INSERTION IN A SINGLE YEAR FROM MALAYSIAN HEART CENTRE

### **SI LING SOH**, AZLAN HUSSIN

#### Institute Jantung Negara, Kuala Lumpur, Malaysia

**Introduction:** Pacemaker insertion involves passing a lead through tricuspid valve and into the right ventricle. It is known that pacing can induced cardiomyopathy but the short‐term effect of pacemaker insertion on right and left ventricle function is less studied

**Methods:** We conducted a retrospective observational cohort study of patients who underwent pacemaker insertion in 2022 who have both pre and post implantation echocardiogram within 6 months of the procedure. We analyze the patient demographic and their left and right ventricle echocardiographic parameters

**Results:** Overall there are 57 patients with complete and good quality echocardiographic images. Their average age is 58.5 ± 22.2yrs and 32 (56.1%) are male. 39 (68.4%) patients have some tricuspid regurgitation pre and this increases to 52 (91.2%) after pacemaker insertion. Moderate tricuspid regurgitation increases from 6 (10.5%) to 10 (17.5%) patients, and severe tricuspid regurgitation increases from 3(5.3%) to 10 (17.5%) patients. In terms of RV function TAPSE and S’ are both numerically lower after pacemaker but it is not statistically significant (TAPSE; 1.98 ± 0.48 to 1.91 ± 0.46 p=0.297, S’; 11.46 ± 2.68cm/s to 10.62± 2.96cm/s p=0.059). Similarly, tricuspid regurgitation max velocity and right ventricle dimension changes are also not statistically significant. The only echocardiographic parameters with statistically significant changes is ejection fraction that come down from 59.2± 9.6% to 56.2±8.6%, p=0.016.

**Conclusions:** Right ventricle function is preserved 6 months after pacemaker insertion. However, the number of patients with TR and its severity increases and LV ejection fraction decreases after pacemaker insertion

## ENHANCING COMMUNITY DETECTION RATES OF CARDIAC CHANNELOPATHISE ‐ INSIGHTS FROM A PROSPECTIVE NATIONAL REGISTRY

### **MADHUSOODANAN PILLAI SREELEKSHMI**, VINODA THULASEEDHARAN JISSA, MADHUSOODANAN URULANGODI, SRINIVAS GOPALA, NARAYANAN NAMBOODIRI

#### Sree Chitra Tirunal Institute for Medical Sciences and Technology, Thiruvananthapuram, Thiruvananthapuram, India

**Introduction:** An early recognition of subjects affected with cardiac channelopathies in the community is relevant in view of the potential risk of sudden cardiac death at a young age in them.

**Methods:** We prospectively studied the probands (N=100) with a diagnosis of a major cardiac channelopathy enrolled in the South Indian Cohort of a national channelopathy registry initiated in May 2021. The factors that contributed to the delayed diagnosis (defined as the time interval from the first noticed clinical symptom to the consideration of channelopathy as a potential diagnosis exceeding one month), if any, in this cohort were looked for.

**Results:** This study involved 100 consecutive subjects (age 0.5‐65 years with a mean of 29.25±18.27 years)diagnosed with cardiac channelopathy disorders (long QT syndrome; LQTS (n=69), Brugada syndrome; BrS (n=27) or catecholaminergic polymorphic ventricular tachycardia; CPVT (n=4)). A significant delay in diagnosis was observed in 48.0%. A higher proportion of subjects in the following groups had delay in recognition of the correct diagnosis: a provisional diagnosis of epilepsy at the initial event (77.3% vs. 39.7%; p=0.002), age ≤18 years vs >18 years (61.9% vs. 37.9%; p=0.018), LQTS vs. non‐LQTS diagnosis (58.0% vs. 25.8%; p=0.003), and evaluation by a cardiology specialist within a month of the index event (32.4% vs.56.1 %, P=0.025). The adjusted analysis of binary logistic regression showed that consultation with a cardiology specialist within a month of the index event (adjusted OR=2.90; CI: 1.10‐7.63) and no prior diagnosis of epilepsy (adjusted OR=3.16; CI: 0.987‐10.17) had lesser chance of delayed diagnosis.

**Conclusions:** In this prospective national cardiac channelopathy registry, we identified age ≤ 18 years, misdiagnosis as epilepsy and a delay in a cardiology specialist consultation after the initial event as the main factors that contributed to the delayed detection of cardiac channelopathy. These findings call for better awareness of the clinical spectrum of channelopathies among medical practitioners.

## CLINICAL PROFILE AND ECHOCARDIOGRAPHIC PARAMETERS OF PATIENTS WITH ATRIAL FIBRILLATION UNDERGOING TEE BEFORE CARDIOVERSION–A RETROSPECTIVE STUDY

### **DIVYA SRIDHAR**, PREETAM KRISHNAMURTHY, MURALIDHARAN THODDI RAMAMURTHY, VINOD KUMAR BALAKRISHNAN, RAMESH SANKARAN

#### Sri Ramachandra Institute of Higher Education and Research, Chennai, India

**Introduction:** Atrial fibrillation (AF) is the most common sustained arrhythmia and is responsible for almost 3,00,000 hospital admissions annually. The Direct Current Cardioversion (DCC) is an important treatment method for AF. However, there is a significant risk of stroke and other systemic thromboembolism after DCC. Transoesophageal echocardiography (TEE) before Cardioversion might be as effective as conventional anticoagulation in preventing stroke while also expediting restoration to sinus rhythm.

**Methods:** This was a retrospective, single‐centre study. We collected the data of patients who underwent TEE before cardioversion, from the TEE registry and patient database software at Sri Ramachandra Institute of Higher Education and Research (SRIHER), from 2019 to 2023. We collected Baseline characteristics, diagnosis, comorbid conditions, Echocardiographic parameters, and follow‐up data and presented them as frequency and proportion.

**Results:** We collected data of 50 patients who underwent TEE prior to cardioversion. The mean age was 57 years (SD 11.5) and more than half of the patients were females (n=28). Nearly half of the patients had comorbid conditions (n=22). The majority of the patients had presented heart failure (n=34) and belonged to NYHA class 3 (n=27). Nearly two‐thirds of the patients had no AF recurrence (n=34) following cardioversion. Nearly one‐third of the patients improved to NYHA class 1 after treatment (n=15). 32 patients were cardioverted as they had no left atrial appendage clot, at the time of presentation with Atrial Fibrillation and half of them had windsock shape of Left atrial appendage (n=25). Majority of the patients maintained in sinus rhythm during follow up.

**Conclusions:** In areas, where majority patients have difficulty in access to centers that perform pulmonary vein isolation for Recurrent AF, performing DC cardioversion after assessment of Transesophageal echocardiographic parameters would help a significant proportion of patients to remain in sinus rhythm and will alleviate the symptoms of heart failure due to AF and to improve functional capacity.

## ASSOCIATION OF VERY SHORT INTERVAL HEART RATE MEAN ABSOLUTE DEVIATION WITH HEART FAILURE: FROM NHANES 2017‐ 2020

### NARATHORN KULTHAMRONGSRI^1^, TATCHAYA KANTHAJAN^2^, CHUTAWAT KOOKANOK^3^, **ADIVITCH SRIPUSANAPAN** ^4^, NICHA WAREESAWETSUWAN^5^, THANATHIP SUENGHATAIPORN^6^, THANABOON YINADSAWAPHAN^1^, VITCHAPONG PRASITSUMRIT^5^, POJSAKORN DANPANICHKUL^7^, PHUUWADITH WATTANACHAYAKUL^8^, PRACHAWANEE NUCHPRAMOOL^9^, PHATSAWUT SIRITONGTAWORN^5^, THITIPHAN SRIKULMONTRI^8^, SAMYAM BICKRAM PATHAK^10^, EKAMOL TANTISATTAMO^11^, PECHRODETH (JENNIFER) MAO^12^

#### ^1^University of Hawaii Internal Medicine Residency Program, Honolulu, HI,^2^Faculty of Medicine, Srinakharinwirot University, Bangkok, Thailand,^3^Phramongkutklao College of Medicine, Bangkok, Thailand,^4^Faculty of Medicine, Chiang Mai University, Chiang Mai, Thailand,^5^Faculty of Medicine, Siriraj Hospital, Mahidol University, Bangkok, Thailand,^6^Griffin Hospital, Griffin, CT,^7^Texas Tech University Health and Science Center, Lubbock, TX,^8^Albert Einstein Medical Center, Philadelphia, PA,^9^Department of Obstetrics and Gynecology, Ramathibodi Hospital of Mahidol University, Bangkok, Thailand,^10^KIST Medical College and Teaching Hospital, Mahalaxmi, Nepal,^11^University of California Irvine School of Medicine, Irvine, CA,^12^Edward Via College of Osteopathic Medicine, Monroe, LA

**Introduction:** Heart Rate Variability (HRV) has recently gained popularity as an indicator of cardiovascular health. However, the association between the Mean Absolute Deviation of Heart Rate (HR‐AMD) as HRV parameters, and Heart Failure (HF), especially in very short intervals, in a nationally representative population, remains unknown.This study aims to investigate the relationship between HR‐AMD and HF in the general population.

**Methods:** A cross‐sectional analysis of data from the 2017‐2020 NHANES was conducted to explore the association between HR‐AMD and HF. Participants had their heart rate measured 3 consecutive times, 60 seconds apart. HF data were collected through self‐reporting. HR‐AMD was calculated and categorized into quintiles. Logistic regression models, adjusting for various factors including demographics and comorbidities, were utilized for the analysis.

**Results:** The study included 9,194 adult participants with a mean HR‐AMD of 0.02 beats per minute. Among them, 359 (3.90%) experienced HF. In all populations, multivariate analysis revealed that the 2nd, 3rd, and 4th quintiles of HR‐AMD are negatively associated with HF, but significance was observed only in the 4th quintile. (OR 0.58) However, the 5th quintile is positively associated with HF, as shown in the table. Participants aged above 50 and 60, whose HR‐AMDs are in the 5th quintile, show statistically significant associations with 1.67 and 1.87 times greater risk of HF, respectively.

The race disparity also impacts the association differently: among White participants in both the 2nd and 4th quintiles of HR‐AMD, there was no negative association with HF. However, among Black participants, the odds ratios were significantly lower at 0.46 times in both quintiles.

**Conclusions:** A very short interval HR‐AMD shows an U‐shape association with HF in the general population. The greater positive associations among older age groups suggest that age may act as effect modifiers in the 5th quintile of HR‐AMD. Racial disparity between White and Black populations is also found across all five quintiles.Further studies are required for a deeper understanding of this relationship.

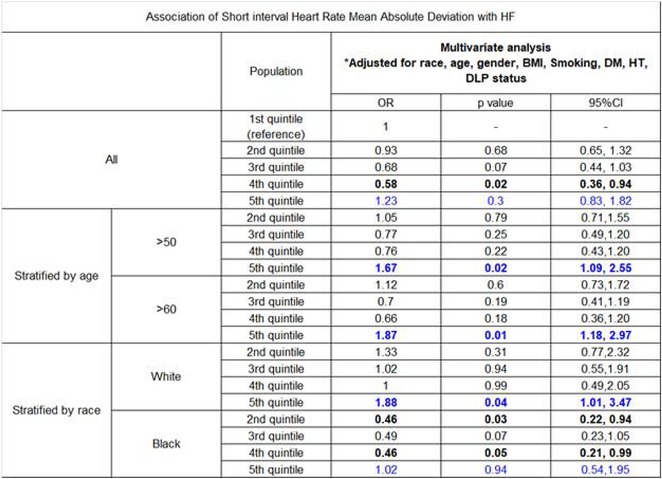

## CORONARY ARTERY CALCIUM SCORE AND RISKS OF ATRIAL FIBRILLATION RECURRENCE AFTER CATHETER ABLATION: A SYSTEMATIC REVIEW AND META‐ANALYSIS

### **ADIVITCH SRIPUSANAPAN** ^1^, NATEE DEEPAN^2^, PAKPOOM WONGYIKUL^3^, PANAT YANPISET^4^, POJSAKORN DANPANICHKUL^4^, NARATHORN KULTHAMRONGSRI^5^, NICHA WAREESAWETSUWAN^6^, JAKRIN KEWCHAROEN^7^, NITHI TOKAVANICH^8^, NARUT PRASITLUMKUM^9^, RONPICHAI CHOKESUWATTANASKUL^10^

#### ^1^Faculty of Medicine, Chiang Mai University, Chiang Mai, Thailand,^2^Faculty of Medicine, Chulalongkorn University, Bangkok, Thailand,^3^Center for Clinical Epidemiology and Clinical Statistics, Faculty of Medicine, Chiang Mai University, Chiang Mai, Thailand,^4^Internal Medicine Residency Program, Texas Tech Health Science Center, Lubbock, TX,^5^Internal Medicine Residency Program, University of Hawaii, Honolulu, HI,^6^Faculty of Medicine, Siriraj Hospital, Mahidol University, Bangkok, Thailand,^7^Division of Cardiology, University of California San Francisco, San Francisco, CA,^8^Division of Cardiovascular Medicine, Frankel Cardiovascular Center, University of Michigan Health, Ann Arbor, MI,^9^Department of Cardiovascular Medicine, Mayo Clinic College of Medicine, Rochester, MN,^10^Chulalongkorn University, Division of Cardiology, Department of Medicine, Bangkok, Thailand

**Introduction:** Studies have found that coronary artery calcium (CAC) score greater than 100 in patients with atrial fibrillation (AF) undergoing catheter ablation is associated an increased risks of AF recurrence. However, results were inconclusive. Hence, we conducted a systematic review and meta‐analysis to evaluate this association.

**Methods:** We searched MEDLINE, SCOPUS, EMBASE for relevant studies from inception until February 2024. Included studies were observational studies evaluating the risks of AF recurrence after catheter ablation between patients with CAC score greater than 100 and without. Baseline characteristics of study participants and outcomes of interests were extracted. Hartung‐Knapp‐Sidak‐Jonkman random effect model was used to calculate pool odd ratios (OR) with 95% confidence interval (95%CI). The statistical heterogeneity of pooled effect was assessed via the use of the Cochrane Q test. Statistical analysis was performed using STATA version 17.

**Results:** 5 studies involving total of 1,169 patients with documented CAC score undergoing AF catheter ablation were included (~66% men, mean age ~59±4.7 years, 28% had CAC score greater than 100, ~72% had paroxysmal AF, ~73% underwent RF, mean LAVI 42.06±9.81 ml). At the average follow‐up duration approximately 18.5 months (ranging from 12 to 34 months) after catheter ablation, patients with a CAC score greater than 100 had a higher risks of AF recurrence (OR= 2.02, 95% CI: 1.10 ‐ 3.72, I^2^=74.88%, Q test p‐value=0.02).

**Conclusions:** Our systematic review and meta‐analysis indicate that a CAC score greater than 100 is associated with an increased risk of AF recurrence after catheter ablation at 18.5‐month follow‐up. Further research is needed to elucidate the mechanisms underlying this association and to assess the utility of incorporating CAC scoring into the clinical decision‐making process for atrial fibrillation ablation therapy.

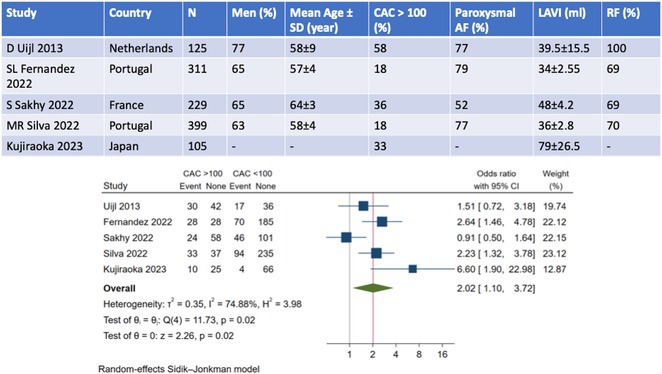

## THE UTILITY OF CONVERSATIONAL ARTIFICIAL INTELLIGENCE FOR PATIENT EDUCATION ON CARDIAC ELECTROPHYSIOLOGY PROCEDURES

### **HARI SRITHARAN** ^1,2^, JUSTIN CHIA^3^, KELSEY GARDINER^1^, NEILA LITKOUHI^1^, KEVIN HELLESTRAND^1^, DAVID WHALLEY^1^, LOGAN KANAGARATNAM^1,4^, KARIN CHIA^1^

#### ^1^Royal North Shore Hospital, St Leonards, Australia,^2^University of Sydney, Sydney, Australia,^3^Coffs Harbour Base Hospital, Coffs Harbour, Australia,^4^Ryde Hospital, Eastwood, Australia

**Introduction:** Conversational artificial intelligence (AI), driven by large language models, provides a new avenue for patient education. We aimed to evaluate the accuracy and readability of these models for educating patients on cardiac electrophysiology (EP) procedures.

**Methods:** We tested 3 freely available conversational AI models, OpenAI's ChatGPT 3.5, Google's Gemini and Microsoft's Copilot, with standardised questions regarding 6 cardiac EP procedures: atrial fibrillation (AF) ablation, cardiac catheter ablation, electrical cardioversion (DCCV), electrophysiology study (EPS), implantable cardiac defibrillator (ICD) insertion and pacemaker (PPM) insertion. Response accuracy was graded using a 5‐point Likert‐scale, compared with patient information sheets from Queensland Health. The readability of responses were graded using the Flesch Reading Ease (FRE) score and Flesch‐Kincaid Grade (FKG) Level. Differences were analysed by Friedman test and post‐hoc Wilcoxon signed‐rank tests with Bonferroni correction.

**Results:** There was no significant difference in response accuracy between models (χ^2^(2) = 4.457, p = 0.108). Most responses across models were graded as ‘acceptable’ to ‘very good’ accuracy for EPS (100%), PPM insertion (91.7%) and ICD insertion (91.7%), but less so for DCCV (58.3%), cardiac catheter ablation (58.3%) and AF ablation (75%). There was a significant difference between models in FRE scores (χ^2^(2) = 20.583, p < 0.001) and FKG levels (χ^2^(2) = 27.583, p < 0.001). There were significantly increased FRE scores and significantly decreased FKG levels with Copilot compared to ChatGPT 3.5 (Z = ‐4.114, p < 0.001 & Z = ‐4.286, p < 0.001 respectively) and Gemini (Z = ‐3.200, p = 0.001 & Z= ‐4.029, p < 0.001 respectively), with no significant difference between Gemini and ChatGPT 3.5; reflecting increased readability with Copilot.

**Conclusions:** Conversational AI models demonstrate consistently accurate responses overall, albeit variation in accuracy between cardiac EP procedures and varied response readability. Cautious use of current models is suggested, with refinement needed for reliable patient education.

## A SINGLE CENTRE EXPERIENCE INVOLVING PERI‐AORTIC VENTRICULAR TACHYCARDIA

### **ROHITH STANISLAUS**, SURAYA HANI K, LOW MY, MATHAN MOHAN, SURINDER K, AZLAN HUSSIN

#### National Heart Institute, Kuala Lumpur, Malaysia

**Introduction:** Peri‐aortic Ventricular Tachycardia is an arrhythmogenic foci for both idiopathic VT as well as scar‐related VT. Peri‐aortic VT was defined as low‐voltage and/or deceleration zones within 2 cm of the left ventriculo‐aortic junction with a corresponding critical site during VT. It is noted that scar‐related VT is mostly reported in older patients who tend to have inducibility of multiple monomorphic VT. However the mechanism for scare related peri‐aortic VT remains incompletely described. In the case of non‐ischemic peri‐aortic VT, it is often intramural and difficult to ablate.

**Methods:** Between September ‐ November 2023, 5 patients with structural heart disease underwent peri‐Aortic VT Ablation at our centre. These patients all had both arterial and venous access pre‐procedure to allow for both ante‐grade/retro‐grade approach for mapping/ablation. They were subjected to an electrophysiology study utilizing Voltage Mapping, Isochronal Late Activation Mapping (ILAM), Decrement‐evoked potential (DeEP) mapping and a Sense Protocol Mapping. Electro‐anatomical mapping was done using the Ensite X system with a HD‐grid catheter with omnipolar signals. Ablation was then performed to these areas of deceleration zones after careful analysis.

**Results:** During the study period, 5 patients in total had peri‐aortic VT. 60% were patients with non‐ischemic dilated cardiomyopathy. The lowest ejection fraction was 24% in a patient with PVC induced cardiomyopathy. Analysis after mapping noted that 80% of patients had a 3‐dimensional VT circuit. Radiofrequency ablation was performed to the Left Coronary Cusp (2 cases), Right Coronary Cusp (2 cases) and 1 to the LCC‐RCC commissure. Only 1 patient had a recurrence of VT at 6 months, he however refused to undergo a re‐do procedure.

**Conclusions:** Peri‐aortic VT can be mapped and successfully ablated with a low recurrence rate. The VT Circuit in our cohort of patients predominantly demonstrated a 3‐dimensional circuit.

## INITIAL EXPERIENCE WITH THE S3 PROTOCOL FOR VT ABLATION

### **ROHITH STANISLAUS**, SURAYA HANI K, LOW MY, MATHAN MOHAN, SURINDER K, AZLAN HUSSIN

#### National Heart Institute, Kuala Lumpur, Malaysia

**Introduction:** Ventricular Tachycardia in the setting of structural heart disease is commonly associated with a re‐entrant mechanism. The gold standard for identifying critical parts of this circuit has been entrainment and activation mapping. However, not all VT are hemodynamically tolerated and some are non‐sustained or non‐inducible. This led to the development of substrate mapping techniques in sinus rhythm. Substrate mapping differentiates between passive and active role of local signals as critical components of the VT. Decrement‐evoked potential (DeEP) mapping was developed to map the functional substrate by utilizing an extra‐stimulus added to a pacing drive train. The S3 protocol hypothesized that functional mapping after double extra‐stimulus would uncover more areas to target for ablation.

**Methods:** Between November 2023 till March 2024, 17 patients with structural heart disease underwent VT Ablation at our centre. These patients all had both arterial and venous access pre‐procedure to allow for both ante‐grade/retro‐grade approach for mapping/ablation. Patients had assessment of the ventricular effective refractory period (ERP) followed by the pacing protocol. The pacing protocol was right ventricular apex pacing with S1 x 6 (cycle length: 400‐500msec) followed by S2 (ERP +30msec) and S3 (ERP + 20msec). Electro‐anatomical mapping was done using the Ensite X system with a HD‐grid catheter with omnipolar signals.

**Results:** It was noted that majority of cases were of Ischemic Dilated Cardiomyopathy (70.5%). Out of the 17 cases performed, only 2 required Epicardial mapping/ablation. The first was a case of Cardiac Sarcoidosis and the second was a case of ARVD. The most common site of ablation was the apex of the left ventricle (29.5%). All cases used the S3 protocol as well as ILAM mapping to look for deceleration zones. 1 patient suffered from immediate complication post procedure in the form of cardiac tamponade. Freedom from VT was described as 76.5% at 6 months follow up.

**Conclusions:** VT ablation utilizing the S3 protocol for functional substrate mapping is safe and allows for better identification of targets for ablation and carries a good outcome with respect to freedom from VT.

## LEFT SIDED CARDIAC RESYNCHRONIZATION THERAPY‐DEFIBRILLATOR IMPLANT VIA A PERSISTENT LEFT SIDED SUPERIOR VENA CAVA

### **ROHITH STANISLAUS**, SURAYA HANI K, LOW MY, MATHAN MOHAN, SOH SL

#### National Heart Institute, Kuala Lumpur, Malaysia

**Introduction:** Persistent Left Superior Vena Cava (PLSVC) is the most common thoracic venous anomaly. The incidence of PLSVC in healthy population is 0.2% to 3% with no difference in prevalence between males and females. Patients with congenital heart disease, incidence increases to 1.3% ‐ 11%. It mostly drains into the right atrium (RA) via the coronary sinus (CS).

**Methods:** We describe a case of a 68 year old gentleman who was investigated for history of syncope. Initial investigation revealed a normal electrocardiogram, normal Holter and normal carotid doppler. His ejection fraction was reduced at 30%. In view of his risk factors, we proceeded with a coronary angiogram which revealed severe 3 vessel disease. Patient was referred to our cardiothoracic colleagues and a CABG was performed successfully. During his recovery period in the ward, patient developed episodes of AF followed by termination pause as well as episodes of bradycardia. A diagnosis of sick sinus syndrome with paroxysmal AF was made and patient was put up for a CRT‐D in view of his reduced ejection fraction. A left sided implant was planned and venogram was performed which revealed a PLSVC. We decided to attempt a left sided CRT‐D in view of the venogram showing an appropriate postero‐lateral cardiac vein branch. Each lead was implanted after individual Axillary vein access. The RV shocking coil was implanted first, a Terumo wire was used to cannulate the vein and progress into the right atrium and subsequently into the right ventricle. A long sheath was used to provide support within the CS and then the shocking coil was placed at the apex. The LV lead was then attempted, using an Attain Select 90^0^ to gain access into the selected vein. A 3830 Medtronic lead was then used for the RA to the lateral RA wall. All parameters were within limits and post CRT‐D electrocardiogram revealed a narrow complex QRS of 12msec.

**Results:** Left sided CRT‐D implanted via the Left Persistent SVC with good parameters and narrow QRS complex.

**Conclusions:** Left sided CRT‐D can be safely and effectively implanted in the presence of a PLSVC via the Left Subclavian route.

## RIGHT SIDED CRT UPGRADE IN AN ESRF PATIENT REQUIRING SUBCLAVIAN VENOPLASTY

### **ROHITH STANISLAUS**, SURAYA HANI K, LOW MY, MATHAN MOHAN, SURINDER K, AZLAN HUSSIN

#### National Heart Institute, Kuala Lumpur, Malaysia

**Introduction:** Incidence of SSS and AV node disease increases with age. Pacemaker implantation increases the heart rate and improves symptoms. Complications associated with long term use are pacemaker induced cardiomyopathy (PICM) and venous occlusions. Long‐term use of RV pacing is reported to induce ventricular dyssynchrony. PICM is estimated to occur in 5‐20% of patients on RV pacing for >2 years. The incidence of central vein stenosis in HD patients is 41%. The most common site is the junction of the subclavian and cephalic veins.

**Methods:** A 67 year old gentleman with underlying ESRF presented with intermittent complete heart block. He'd been on regular HD via Left BCF. Prior to fistula creation, he had undergone multiple central venous catheter placements. In view of vascular access concerns, a leadless pacemaker was implanted.

Patient was well for 3 years when his pacing burden was low, however over the past 1‐2 years he had become increasingly dependent on the pacemaker. Serial echocardiograms revealed reduction of EF as RV pacing increased. He was breathless during HD and thus a decision was made to upgrade his pacing system. His EF had dropped from 55% to 40%. Prior to incision, a venogram was performed which showed total occlusion of the right subclavian vein. An axillary approach was chosen as this has been shown to have more support in crossing total occlusions in our experience. The left brachial vein was punctured under fluoroscopy with an 18G needle and wired with a 0.018mm guidewire. A 6F sheath was the inserted. Attempts to cross the total subclavian occlusion were made using a 0.018mm guidewire with the support of a JR guiding catheter. Once the occlusion was crossed, the lesion was progressively dilated with peripheral angioplasty balloons. The pocket was then created, vein punctured and procedure continued as per usual.

**Results:** Successful implantation of a right sided CRT‐P in a patient with ESRF following venoplasty of the right subclavian vein.

**Conclusions:** Right subclavian venoplasty followed by Right sided Cardiac Re‐synchronization Therapy Pacemaker implantation is a feasible option for patients with ESRF who have limited vascular access requiring a device upgrade.

## VENTRICULAR TACHYCARDIA ABLATION UTILIZING SENSE PROTOCOL: A SINGLE CENTRE REVIEW OF OUTCOMES AT 1 YEAR

### **ROHITH STANISLAUS**, SURAYA HANI K, LOW MY, MATHAN MOHAN, SURINDER K, AZLAN HUSSIN

#### National Heart Institute, Kuala Lumpur, Malaysia

**Introduction:** Ventricular tachycardia (VT) is often poorly tolerated in patients with structural heart disease. Post infarct VT occurs due to re‐entry over the scared ventricular tissue. Some of the challenges faced during VT mapping and ablation are poorly tolerated VT, a non‐inducible and non‐sustained VT. Classically, activation mapping was used to define the critical VT isthmus but this is tolerated in around 30% of patients. A technique to overcome these hurdles in management was to do functional substrate mapping by using a single extra‐stimulus protocol. This mapping technique allowed for mapping in a more stable environment. It identified areas of delayed potentials which may play a critical role. Functional substrate mapping techniques, such as single extra‐stimulus protocol mapping, identify regions of unmasked delayed potentials, which, by nature of their dynamic and functional components, may play a critical role in sustaining VT. These methods may improve substrate mapping of VT, potentially making ablation safer and more reproducible, and thereby improving the outcomes.

**Methods:** Between 2021 and 2023, a total of 31 patients with VT were subjected to RFA after careful selection. These patients underwent EPS utilizing a Sense protocol as described by the team at Barts Heart Centre. Mapping was performed with a HD grid multipolar catheter and Ensite mapping system. A bipolar voltage map was created during sinus rhythm and during right ventricular pacing. A sense protocol map was then obtained to detect areas of late potentials. Ablation was then performed to these deceleration zones.

**Results:** A total of 31 patients underwent VT ablation using the sense protocol, 100% were males. The mean age was 61 years and 87% of them were cases of Ischemic Dilated Cardiomyopathy with an average Ejection Fraction of 30% and the lowest being 15%. 3 of these patients had undergone previous ablation. 19.4% of patients in this cohort had a recurrence of VT within the first year.

**Conclusions:** VT mapping and ablation utilizing a sense protocol approach is safe with good outcomes. Our data reveals a VT recurrence at 1 year of 19.4% with a low complication rate of 12.9%.

## ACTIVE FIXATION ATRIAL PACING LEAD PLACEMENT IN PERSISTENT LEFT SUPERIOR VENA CAVA WITH MODIFIED GUIDING CATHETER

### **JUNICHI SUGIURA** ^1^, TAKU NISHIDA^2^, TOMOKO KIKAWA^3^, YANG WANG^2^, KENICHI ISHIGAMI^1^, SHUNGO HIKOSO^2^

#### ^1^Nara City Hospital, Nara City, Japan,^2^Nara Medical University, Kashihara City, Japan,^3^Nara Medical University, Kashihar City, Japan

**Introduction:** Atrial pacemaker lead is generally placed in right atrium, but rarely in coronary sinus (CS).

**Methods:** “N/A”

**Results:** A 77‐year‐old woman, who had undergone pulmonary vein and persistent left superior vena cava (PLSVC) isolation for paroxysmal atrial fibrillation, admitted due to syncope. Ambulatory electrocardiogram monitoring detected ventricular tachycardia (VT) and we decided radiofrequency catheter ablation. However, we could not any intervention for VT because it was impossible to induce VT and identify any arrhythmogenic substrate. Thus, she underwent implantable cardioverter defibrillator (ICD) implantation on the right side due to the existence of PLSVC. After shock lead placement, active fixation atrial pacing lead (2088TC/46cm, Abbott, U.S) was placed to right atrial free wall, because of low P‐wave amplitude or high threshold in right atrial appendage (RAA) and right atrial septum (RAS). Echocardiography after ICD implantation revealed a significant amount of pericardial effusion and pericardial drainage was performed. After pericardial drainage, both atrial and shock lead failed pacing. ICD re‐implantation was conducted with cardiac surgical back‐up. Both leads were extracted without exacerbation of pericardial effusion. The same as the initial implantation, each parameter was inadequate in RAA and RAS. However, P‐wave amplitude was over 2mV without far field ventricular wave sensing in the enlarged CS and proximal PLSVC. Atrial lead fixation to CS and PLSVC with stylet‐driven system was difficult because the lead helix was attached in a nearly parallel direction to the wall of CS and PLSVC. Therefore, we delivered the atrial lead with a modified CS guiding catheter for left ventricular lead implantation (CPS Direct Wide curve, Abbott, U.S). The atrial lead was successfully implanted into the left atrial sided wall of proximal PLSVC. Each parameter of atrial lead was adequate and remained stable at 10‐month follow‐up with no complications.

**Conclusions:** This was the first case that active fixation atrial pacing lead was placed into PLSVC via retrograde approach with a modified guiding catheter.

## ACUTE CHANGES IN LEFT ATRIAL APPENDAGE FUNCTION WITH PREMATURE VENTRICUALAR CONTRACTIONS

### **SURESH KUMAR SUKUMARAN**, ANISH BHARGAV, SRIDHAR BALAGURU, AVINASH ANANTHARAJ, RAJA J SELVARAJ

#### Jawaharlal Institute of Postgraduate Medical Education and Research (JIPMER), Pondicherry, India

**Introduction:** Left atrial appendage (LAA) dysfunction is a well‐established risk factor for stroke in the setting of atrial fibrillation. Evidence shows that frequent premature ventricular complexes (PVCs) are associated with embolic stroke. Whether left atrial dysfunction is the bridging link between frequent PVCs and stroke is unknown. The objective is to study the acute changes in left atrial appendage function with premature ventricular contractions.

**Methods:** Patients with a structurally normal heart planned for elective electrophysiology study for supraventricular tachycardia were included in the study after informed consent. Transesophageal echo was used to measure LAA doppler flow velocities. To simulate PVCs in bigeminal rhythm, single paced beats were delivered through a quadripolar catheter in the right ventricle with a coupling interval of QT + 10 % RR interval after each sinus beat. LAA flow doppler velocities were acquired at baseline, after five minutes of pacing and again five minutes after cessation of pacing.

**Results:** Ten patients were included in the study. Late diastolic emptying velocity decreased significantly after five minutes of PVCs (55.68 ± 16.33 cm/s, p = 0.01) compared to baseline (68.01 ± 10.34 cm/s). This almost returned to baseline after a rest period of five minutes (63.13 ± 16.16 cm/s, p = 0.277). Left atrial appendage filling velocity also decreased after five minutes of PVCs (45.70 ± 10.85 cm/s, p = 0.129) compared to baseline (51.31 ± 14.11 cm/s) and almost returned to baseline after a rest period of five minutes (48.63 ± 6.79 cm/s, p = 0.332). Figure 1 shows the doppler flow velocities in a representative patient at the three time points.

**Conclusions:** Premature ventricular contractions in bigeminal pattern for five minutes resulted in an acute decrease in the late diastolic emptying and filling velocities. This LAA dysfunction induced by PVCs is a possible mechanism for the increased risk of thromboembolic strokes in patients with frequent PVCs.

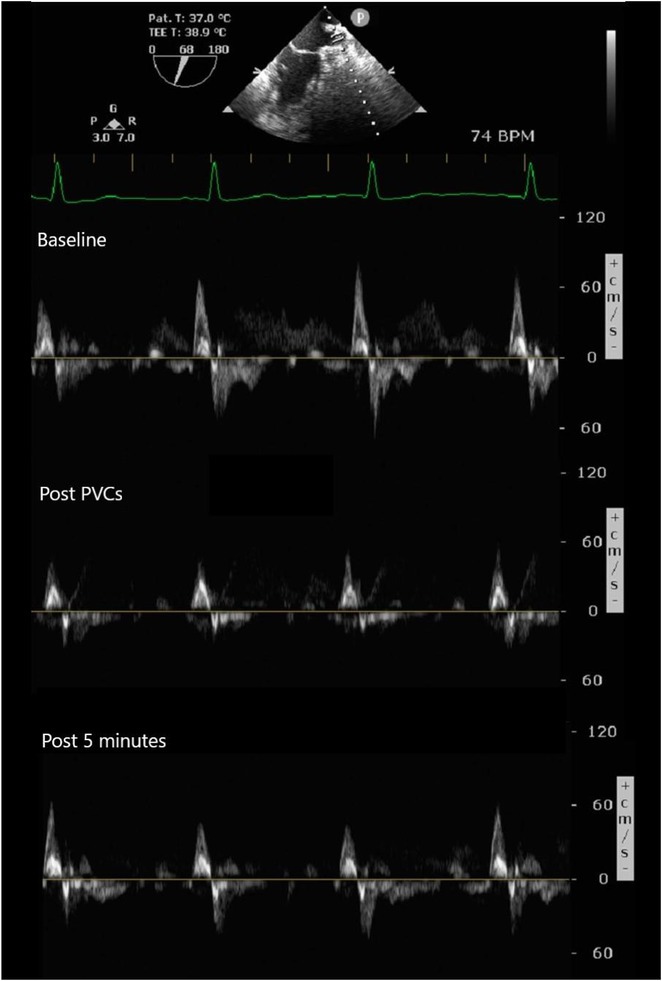

## THE NONLINEAR RELATIONSHIP BETWEEN EPICARDIAL ADIPOSE TISSUE AND ATRIAL FUNCTION IN PAROXYSMAL ATRIAL FIBRILLATION PATIENTS: INSIGHTS FROM CARDIAC MAGNETIC RESONANCE IMAGING

### **HUAN SUN**, YUQUAN HE, YANJING WANG

#### China‐Japan Union Hospital of Jilin University, Changchun, China

**Introduction:** Recent studies link epicardial adipose tissue (EAT), a distinctive visceral fat associated with local inflammation, to atrial remodeling in AF patients. However, the relationship between atrial function and EAT in the context of paroxysmal AF remains underexplored.

**Methods:** We conducted a retrospective analysis of the CMR image at China‐Japan Union Hospital of Jilin University from July,2020, to November,2023. Our study targeted 85 patients with PAF, while 46 control subjects without structural heart disease or AF history, who underwent CMR, were selected. Utilizing CVI42 software, CMR images were analyzed to extract Es,Ee,Ea. EAT area was calculated using CVI42 software in the four‐chamber view. The correlation between EAT area and atrial strain parameters in PAF patients was subsequently analyzed.

**Results:** After propensity score matching, baseline parameters showed no statistically significant differences between the PAF and control groups (n=36 in each group, p>0.05). In the matched data analysis, there were no significant differences in EAT area between the PAF and control groups [(7.05 ± 3.47) cm2 vs. (6.01± 2.34) cm2, p = 0.143]. LA strains, exhibited statistically significant differences between the two groups(Es;Ee;Ea). Further analysis within the PAF group (n=85) revealed that Es and Ee correlated with EAT (r= ‐0.3407 p = 0.0014, r=‐0.4183 p <0.0001), while Ea showed no correlation(p=0.2857). Subsequently, the PAF group was stratified into high EAT (≥median value of 6.18 cm2) and low EAT (<median value of 6.18 cm2) groups. Es and Ee correlated with EAT only in the low EAT group (r=‐0.3091 p = 0.0437, r=‐0.4842 p = 0.0010), and Ea showed no correlation. None of these strain parameters correlated with EAT in the high EAT group.

**Conclusions:** The relationship between epicardial adipose tissue (EAT) and left atrial (LA) function is notably nonlinear: while a linear correlation is evident in individuals with low EAT levels, no correlation is observed in those with high EAT levels.

## IS PACEMAKER ALWAYS THE ANSWERS IN LATE PRESENTING HIGH DEGREE HEART BLOCK AFTER MITRAL VALVE REPLACEMENT

### **HANS SUNARTO**, BENNY SETIADI, NANCY LAMPUS, DYLAN HADI, JASON SAMSUDIN

#### Prof R. D. Kandou Central Hospital, Manado, Indonesia

**Introduction:** Atrioventricular (AV) block is one of the complications that occur after Mitral Valve Replacement (MVR) surgery. Late‐presenting AV block is very rare after this procedure. Pacemaker placement is the definitive therapy for this kind of case.

**Methods:** N/A

**Results:** A 44‐year‐old woman presents with complaints of weakness and fever for the past week. The patient underwent MVR surgery 6 months ago with good results. Post‐operative ECG before discharge showed first degree AV block. Transesophageal echocardiography (TEE) examination 3 weeks ago revealed thrombus on the prosthetic mitral valve. ECG examination on the following days revealed intermittent High degree AV block. The patient received antibiotic therapy and underwent an electrophysiology (EP) study, revealing normal HV interval (50ms), prolonged AH interval (420ms) and suprahisian block at S1 500ms. High degree AV block is suspected to be caused by inflamation (myocarditis/pericarditis). ECG during the remainder of the treatment revealed first degree AV block after 1 week of antibiotic therapy. She had an uneventful recovery afterwards. Follow up ECG in cardiology clinic 6 month after discharge still showed first degree AV block without symtomps.

**Conclusions:** Late‐presenting AV block is rare occurrence in post‐MVR patients, but not all High degree AV block require pacemaker placement as definitive therapy. An EP study can be one of the procedures to assess the source of block (suprahisian, intrahisian or infrahisian), then determine whether pacemaker implantation is necessary or not.

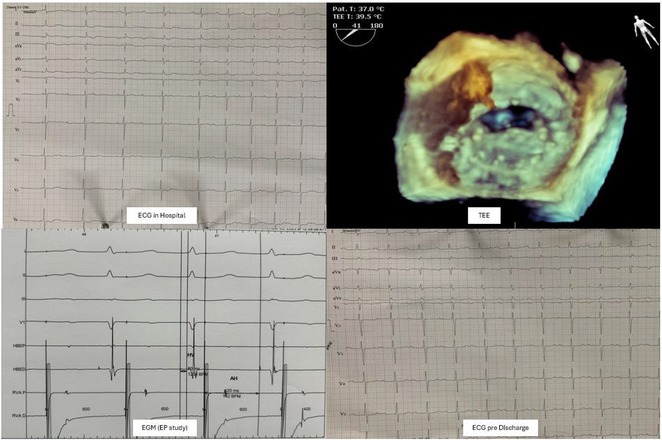

## A NOVEL REMOTE MONITORING SOLUTION FOR LEADLESS PACEMAKERS

### **SRI SUNDARAM** ^1^, NITIKA CHELLAPPA^2^, RAJESH BANKAR^3^

#### ^1^South Denver Heart Center, Colorado, US, Denver, CO,^2^Abbott Medical, Chicago, IL,^3^Hoag Memorial Hospital Presbyterian, Newport Beach, CA

**Introduction:** Remote monitoring has become standard of care for cardiac rhythm devicemanagement. **However, remote monitoring is not currently available for DR leadlesspacemakers**. The AVEIR™ single‐chamber (VR) and dual‐chamber (DR) leadlesspacemaker (LP) systems (Abbott, Abbott Park, IL) utilize a novel conductive telemetry communicationschema for in‐clinic device interrogation. A patient transmitter can bedesigned as an at‐home remote monitoring solution that interrogates AVEIRleadless pacemaker systems and transmits the patient data to Merlin.net™Patient Care Network (PCN).**Objective**: Demonstrate feasibility of a patienttransmitter to interrogate AVEIR DR LP system.

**Methods:** Patientsimplanted with an AVEIR DR system during IDE trial in the USA were enrolled ina non‐significant risk trial after IRB approval. The study protocol included 1)LP system interrogation with the patient transmitter in a controlled setting inhand‐held and wired modes of use; and 2) programmer interrogation to confirm LPparameters were unchanged by transmitter use. Patient transmitters wereconfigured for abbreviated interrogation of device memory and local storage ofdata on‐unit.

**Results:** N=19patients in 2 centers (37% female) completed interrogation of their AVEIR DRsystem using the patient transmitter in both modes. Patients’ pacing modesincluded DDD (N=14), AAI (N=3), and VVI (N=2) modes, both with and without rate‐responsivepacing enabled. In 100% of cases, patient transmitter interrogation wassuccessful. The remote monitoring interrogations ranged from 2 ‐ 31 seconds per LP device. The majority of theinterrogations were of maximum telemetry throughput and speed. Programmerinterrogation confirmed LP parameters were unchanged by remote monitoringinterrogation.

**Conclusions:** Theinitial, real‐world experience of utilizing a remote transmitter with AVEIR DRpatients, demonstrated feasibility of a remote monitoring solution for leadlesspacemaker patients via conductive telemetry.

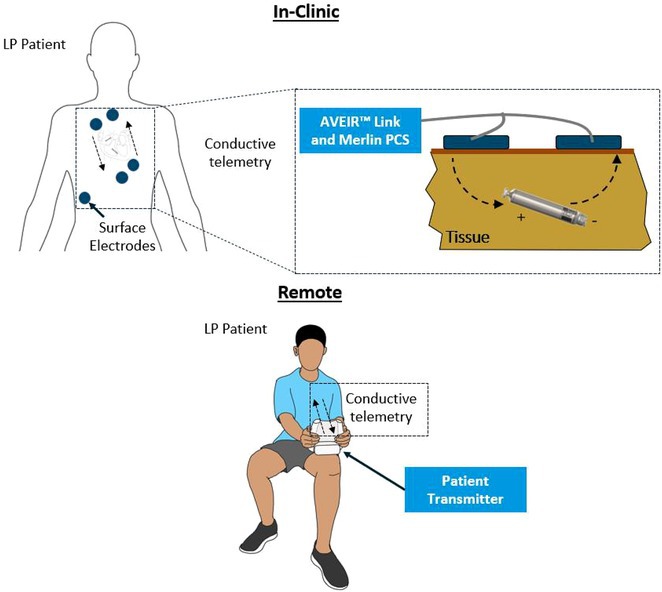

## CARDIOTOXIC IMPACT OF DEHP EXPOSURE: UNDERSTANDING EFFECTS ON CARDIAC ELECTROPHYSIOLOGY AND MYOCARDIAL CELLS

### TING‐TSE LIN^1^, DEBBY SUNG^2^, **YEN‐LING SUNG** ^1^

#### ^1^National Taiwan University Hospital, Taipei, Taiwan,^2^Taipei Medical University, Taipei, Taiwan

**Introduction:** Cardiovascular diseases (CVD) pose a significant global health threat, with millions of deaths attributed to them annually. Environmental pollutants, including the endocrine‐disrupting compound Di(2‐ethylhexyl) phthalate (DEHP), have emerged as potential contributors to CVD. This study investigates DEHP's impact on cardiac electrophysiology and myocardial cells, shedding light on its cardiotoxic effects.

**Methods:** We employed optical mapping techniques to assess cardiac electrophysiology in Langendorff‐perfused rat hearts exposed to DEHP, comparing them to a control group. Additionally, we evaluated the effect of DEHP on cardiac muscle cells using H9C2 rat cardiomyoblasts.

**Results:** Prolonged DEHP exposure resulted in elevated irregular heart rate **(1A)**, increased electrocardiographic variability, and higher low‐frequency values, indicative of sympathetic nervous system overactivity. Action potential duration at APD80 increased, leading to greater action potential triangulation. Electrical alternans observed in the DEHP‐treated group heightened the risk of arrhythmias. Single‐cell analysis revealed severe mitochondrial damage **(1B)**, myocardial cell apoptosis, and disrupted calcium ion balance.

**Conclusions:** This study highlights DEHP's cardiotoxicity, suggesting its potential role in arrhythmogenesis and cardiac dysfunction. Environmental pollutants like DEHP warrant consideration as contributors to cardiovascular diseases, emphasizing the need for further research on preventive strategies.

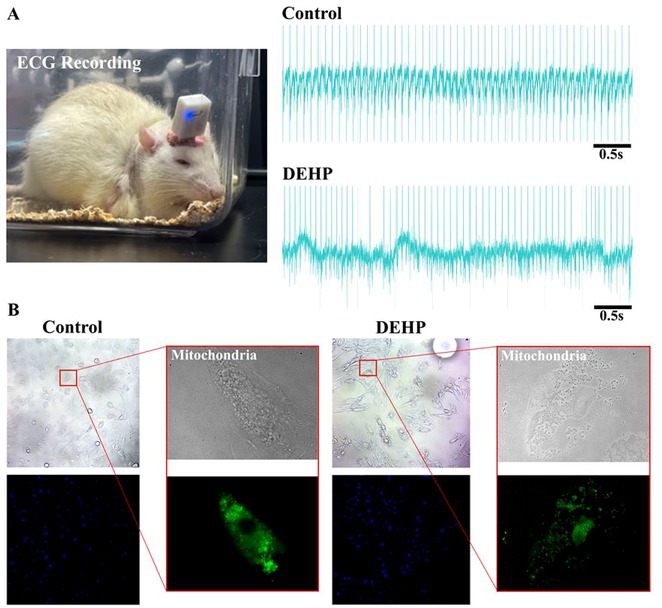

## A NOVEL HIGH‐COMPLIANCE CRYOBALLOON REDUCED BALLOON‐PULMONARY VEIN GAP COMPARED WITH CONVENTIONAL CRYOBALLOON

### **ATSUSHI SUZUKI**, KUNIHIKO KIUCHI, SATO NAMURA, MASAHIRO YAMADA, WAKO SUEHIRO, KEN KINUGAWA, RYO TAKESHIGE, NAO SHIBATA, SATORU SASAKI, DAISUKE MATSUMOTO, HIROSHI TAKAISHI

#### Yodogawa Christian Hospital, Osaka, Japan

**Introduction:** The utility of intracardiac echography (ICE) and the negative correlation between peri‐balloon leak flow velocity (LFV) and the diameter of pulmonary vein (PV) electrical gap have been reported. We aimed to assess the fitness to PV structure of high‐compliance cryoballoon (HCB; POLARx FIT™), with lower internal balloon pressure (IBP; 2.5psig when the balloon size is 28mm) than conventional cryoballoon (CCB; Arctic Front Advance™ Pro, IBP; 20psig), using ICE imaging.

**Methods:** Patients with atrial fibrillation underwent HCB or CCB PV isolation with the balloon size of 28mm. ICE leak flow evaluation followed by tip contrast injection (2‐3ml, 1‐1.5ml/s) for each PV was performed prior to freeze. When grade 3 or 4 PV occlusion achieved, 180s freeze (up to 240s when time to isolation >120s) was performed. The pull‐down maneuver was allowed when the right‐inferior PV was targeted.

**Results:** A total of 159 freezes to 140PVs, in 35 patients (HCB: n=10, CCB: n=25), were analyzed. Acute PV isolation was succeeded in all PVs. PV isolation with 1st freeze was achieved in 38/40PVs (95%) with HCB and in 86/100PVs (86%) with CCB (P=0.13). Figure shows ICE leak evaluation and comparison of LFV between groups. ICE leak was observed in 14 freezes (26.7%) with HCB and in 26 freezes (22.8%) with CCB (P=0.61), however, LFV was significantly higher in HCB than CCB (143.5±29.1 vs. 89.7±37.3 cm/s, P<0.01).

**Conclusions:** The present study showed the higher LFV in HCB ablation indicating the smaller diameter of pulmonary vein gap. A novel high‐compliance cryoballoon reduced balloon‐pulmonary vein gap compared with conventional cryoballoon.

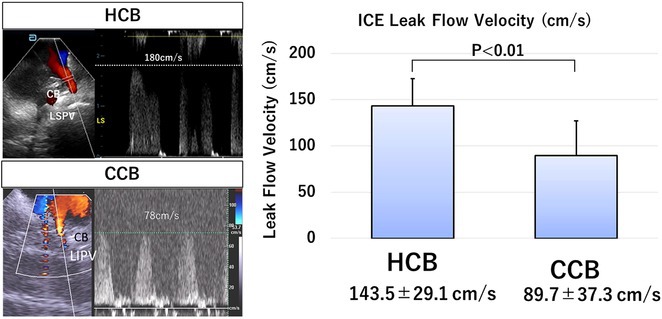

## A RETROSPECTIVE STUDY OF DEVICE‐RELATED INFECTIONS IN PATIENTS WITH CARDIAC RESYNCHRONIZATION THERAPY‐DEPENDENT HEART FAILURE

### **KEIGO SUZUKI**, HOKUTO YAMAGISHI, SHIGETOSHI SAKABE, KENICHI MAENO, ATSUNOBU KASAI

#### Ise Red Cross hospital, Ise City, Japan

**Introduction:** When heart failure cases dependent on cardiac resynchronization therapy (CRT) are complicated by device‐related infections, implantation of a new left ventricular lead (LVL) is often difficult. We aimed to retrospectively analyze the clinical course of infected cases and consider appropriate management.

**Methods:** The subjects were CRT‐dependent device‐related infection cases treated at our institution between 2014 and 2023, and their treatment and prognosis were analyzed.

**Results:** In 10 patients (mean age 74.8 years, nine men, 4 with CRT‐P, mean follow‐up 46.6 months after infection), there were no deaths due to infection; implantation of a new device, including LVL, was attempted in 8 patients and six were successful. However, 2 (an 82‐year‐old male and an 86‐year‐old male) underwent right ventricular septal pacing because a suitable LVL could not be implanted. These cases died of heart failure at 2 and 6 months, respectively. Two other cases (a 62‐year‐old male and an 84‐year‐old male) were considered difficult to reimplant LVL based on the findings at the time of initial implantation; both had local infections with identified pathogens (*Staphylococcus epidermidis and Cutibacterium acnes*), one underwent drainage, and one underwent new generator implantation at a remote site with preservation of the former LVL and continued oral antibacterial therapy. There was no re‐infection in these two cases during the observation periods of 20 months and 13 months, respectively.

**Conclusions:** In CRT device‐related infections in severe heart failure, the priority should be to continue CRT. Preservation of LVL may also be an option in some conditions.

## ECG‐AI AIDED ECHO REFERRAL OPTIMIZATION

### **ABHYUDAY SWAMY** ^1^, DEEPAK KRISHNAN^1^, PRANAY UMREDKAR^1^, PARAMITA GHORAI^2^, SANTHOSH RATHNAM PALANI^1^, VIVEK RAJAGOPAL^1^, PRADEEP NARAYAN^2^, DEEPAK PADMANABHAN^3^

#### ^1^Medha Analytics, Narayana Health, Bengaluru, India,^2^Rabindranath Tagore International Institute of Cardiac Sciences (RTIICS), Narayana Health, Kolkata, India,^3^Narayana Institute Of Cardiac Sciences (NICS),Narayana Health, Bengaluru, India

**Introduction:** The echocardiogram's (echo) efficacy is often mitigated by unnecessary referrals. Detecting abnormalities solely based on patient interaction challenges non‐specialists, necessitating a rapid, scalable, and cost‐effective screening tool integrated into existing workflows. Leveraging the 12‐lead Electrocardiogram (ECG) trace image, our study introduces a Deep learning algorithm ensemble aiding in screening for low ejection fraction (EF), valvular heart disease (VHD), and pulmonary arterial hypertension (PAH), streamlining echo referrals.

**Methods:** From our in‐house database, between January 2021 to November 2022, we identified 53,661 pairs of ECGs and echos (≥15 years of age) performed on the same day. From the echo reports, if one or more of EF ≤ 35%, any grade of stenosis (Aortic, Mitral, Tricuspid), moderate to severe regurgitation (Aortic, Mitral, Tricuspid), and or PAH were identified, the data point was assigned 1 (dysfunction) else 0 (non‐dysfunction). The data was split into train (n=40,796), validation (n=2,148) and test sets (n=10,717). The ECGs were then used to train an ensemble of 3 neural networks to predict the probability of dysfunction. The predictions on the test set (InV) were then classified into 1 vs. 0 by optimizing the probability threshold using the F1 score (F1) and Youden's Index (YI). Performance was assessed using the area under the receiver‐operating characteristic (ROC) and precision‐recall (PR) curves, and classification metrics. Independent validation was done on an external dataset (ExV; n =22,055) from another hospital about 1870 km away.

**Results:** The prevalence of dysfunction in InV was 19.24% vs. 16.35% in ExV. The sensitivity, specificity, positive predictive value, and negative predictive value (NPV) were, InV: F1 ‐ **0.68, 0.89, 0.59**, and **0.92**; YI ‐ **0.80, 0.80, 0.48**, and **0.94**. ExV: F1 ‐ **0.62, 0.87, 0.49**, and **0.92**; YI ‐ **0.82, 0.73, 0.37**, and **0.95**. Other metrics are given in Fig. 1.

**Conclusions:** Even with varying prevalence, the model shows high NPV. Assuming no other clinical context or indication, using the YI threshold predictions on ExV, 13,403 out of 18,449 non‐dysfunction echos could have been obviated, at the cost of missing 652 of the 3,606 dysfunction cases.

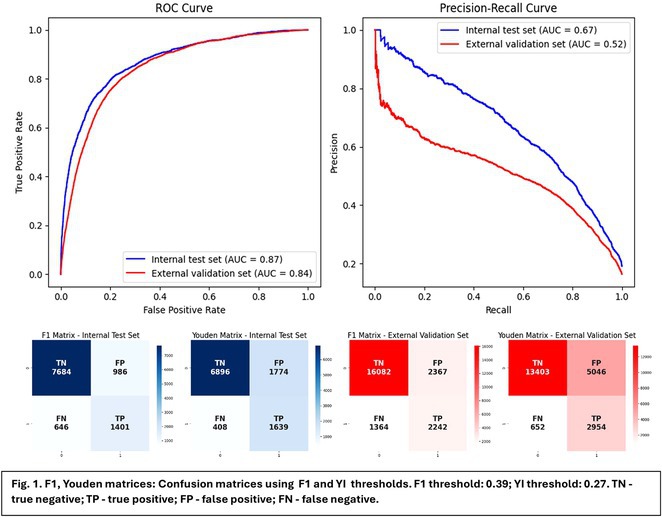

## SUCCESSFUL RADIOFREQUENCY CATHETER ABLATION OF THE PARA‐HISIAN ACCESSORY PATHWAY IN A YOUNG GIRL WITH WOLFF‐PARKINSON‐WHITE SYNDROME

### **ANDI NOVITA DEWI ARYANTI SYAFNI**, MUZAKKIR AMIR, PETER KABO

#### Hasanuddin University, Makassar, Indonesia

**Introduction:** Wolff‐Parkinson‐White (WPW) syndrome is a congenital heart condition causing ventricular pre‐excitation due to the presence of an accessory pathway. A rare variant involves para‐Hisian accessory pathways, close to the AV node and bundle of His, posing risks during ablation due to the potential complete AV block.

**Methods:** N/A

**Results:** A 9‐year‐old girl had been admitted to the Cardiac Center Wahidin Sudirohusodo Hospital outpatient clinic due to self‐limiting palpitations since she was 4 years old. We performed an ECG, which revealed sinus rhythm with a short PR interval, a delta wave, and a widened QRS complex. Physical examinations and echocardiography are within normal limits. She was planning to get an electrophysiology study (EPS) and radiofrequency catheter ablation. We positioned a quadripolar catheter 7F at the right ventricular apex. A duodecapolar coronary sinus (CS) catheter 6F was positioned across the tricuspid annulus from the right atrium to the right ventricle through the bundle of His. The burst ventricular pacing was performed, which induced atrioventricular re‐entrant tachycardia (AVRT). AV fusion was seen on the CS 7‐8 in the anteroseptal tricuspid annulus, followed by 3D mapping of the tricuspid annulus. The ablation catheter 6F was inserted and placed in the tricuspid annulus, and then we decided to perform ablation in the para‐Hisian region. The ablation of the para‐Hisian accessory pathway was performed with 30 watts of energy delivered for 60 seconds at 50°C. After several radiofrequency energy deliveries, the delta wave and AV fusion disappear. The final ECG after ablation showed sinus rhythm without the presence of delta waves. Several days after the procedure, the patient was discharged without any significant complaints.

**Conclusions:** Radiofrequency catheter ablation in children with relatively rare accessory pathways located close to the normal AV conduction system requires great care and caution due to the high risk of causing a complete AV block. Radiofrequency catheter ablation with 3D mapping can help make the procedure more effective and safe for children.

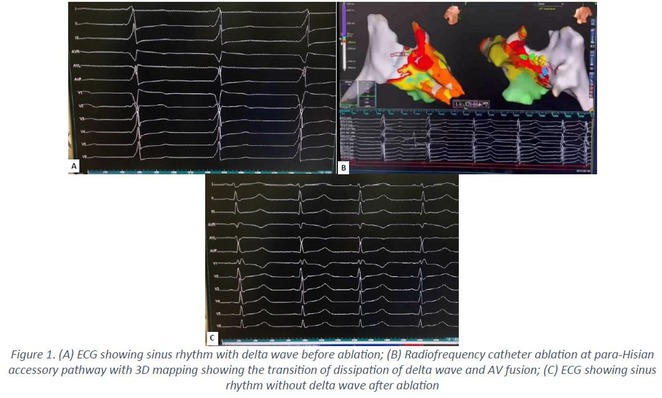

## THE ASSOCIATION BETWEEN LATE GADOLINIUM AMONG VENTRICULAR ARRHYTHMIA IN PATIENT WITH DEFINITE OF ARVC: A SEVEN‐YEAR HOSPITAL‐BASED CROSS‐SECTIONAL STUDY AT DR. SARDJITO HOSPITAL ‐ INDONESIA

### **MUHAMMAD SYAHRIZA**, FERA HIDAYATI, PUTRIKA PRASTUTI RATNA GHARINI, DYAH ADHI KUSUMASTUTI, ERIKA MAHARANI

#### Faculty of Medicine, Public Health and Nursing, Universitas Gadjah Mada, Yogyakarta, Indonesia

**Introduction:** Clinically significant ventricular arrhythmias pose a serious health risk, necessitating thorough investigation and treatment. Our study aims to examine the association between late gadolinium enhancement (LGE) and arrhythmogenic right ventricular cardiomyopathy (ARVC) in patients presenting with ventricular arrhythmias, offering insights into diagnostic approaches and therapeutic strategies.

**Methods:** A total of 343 patients diagnosed with ventricular arrhythmia were enrolled in a hospital‐based cross‐sectional study conducted over seven years, from January 2017 to December 2023. These patients were then diagnosed for definite ARVC according to the Padua criteria, confirmed through Holter electrocardiography, echocardiography, and CMR. Data on LGE were retrieved. Univariate analysis was performed and chi‐square tests was used to study how variables relate.

**Results:** The baseline data reveals that participants' ages ranged from 12 to 73 years old, with a mean age of 43.84 years (95% CI 42.43 to 45.25). The incidence of ventricular arrhythmia was notably higher among women, accounting for 230 participants (67.7%), compared to 113 (32.9%) among men. However, the incidence of ARVC within males was nearly one‐third compared to those within females with less than 15%, with a p‐value of 0.016. Out of the entire group, 37.6% (129 individuals) have tested positive for LGE, while 16.9% (58 individuals) have been diagnosed with definite ARVC. Among the definite ARVC patients, those who underwent CMR examinations showed evidence of LGE, indicative of myocardial fibrosis, with statistically significant results (p‐value of 0.00).

**Conclusions:** Our study highlights gender‐based disparities in the prevalence of ventricular arrhythmia and arrhythmogenic right ventricular cardiomyopathy (ARVC). Additionally, the presence of late gadolinium enhancement (LGE) strongly correlates with cases of ARVC. Keywords: late gadolinium enhancement, arrhythmogenic right ventricular cardiomyopathy, cardiac magnetic resonance, ventricular arrhythmia.

## THE EFFICACY OF THE 4 LINE METHOD IN LEFT ATRIAL POSTERIOR WALL ISOLATION

### **HARUNA TABUCHI** ^1^, GAKU SEKITA^2^, MASATAKA SUMIYOSHI^1^, HIDEMORI HAYASHI^2^, ASUKA TAKANO^3^, KIKUO ISODA^1^

#### ^1^Juntendo University Nerima Hospital, Tokyo, Japan,^2^Juntendo University graduate school of medicine, Tokyo, Japan,^3^Juntendo University Faculty of Medical Science, Tokyo, Japan

**Introduction:** Left atrial posterior wall isolation(PWI) is considered an effective procedure in the ablation of atrial fibrillation(AF). However, it presents challenges such as a high rate of reconnection,increase the incidence of atrial tachycardia(AT).

**Methods:** We retrospectively enrolled 70 consecutive AF patients who underwent pulmonary vein isolation(PVI) and PWI using the CARTO 3 System. In 40 cases(Group A), conventional PWI was performed by creating two lines connecting the left atrial roof line, bottom line. 30 cases(Group B) underwent posterior wall isolation by creating four lines on the anterior and posterior sides of the left atrial roofline, at the height of the left and right carina, and bottom line. We compared and examined the PWI first‐pass rate, chronic posterior wall BOX reconnection rate, and the recurrence rate of AT/AF between the two groups.

**Results:** The first‐pass PWI success rate was 47.5%(19/40) in Group A and 90.0%(27/30) in Group B. In Group A, gaps were observed in multiple areas, including the roof and bottom. However, in Group B, all three cases with gaps were limited to the esophageal proximity of the bottom line, with no gaps observed in the roof. The recurrence rate of AT/AF was 32.5%(13/40) in Group A and 13.3%(4/30) in Group B. In cases that experienced recurrence and underwent retreatment, the posterior wall reconnection rate was significantly higher in Group A at 81.8%(9/11) compared to Group B, where no reconnections were observed(0/3).

**Conclusions:** The 4‐line method is considered a useful strategy for maintaining higher durability for posterior wall isolation.

## LOCAL VENTRICULAR ACTIVATION IN THE DISTAL GREAT CARDIAC VEIN DETERMINES A RIGHT‐ OR LEFT‐ SIDED APPROACH FOR CATHETER ABLATION OF OUTFLOW TRACT ARRHYTHMIAS: A SIMPLE AND RAPID INDICATOR

### **HIROSHI TADA**, KANAE HASEGAWA, MACHIKO MIYOSHI, TOSHIHIRO TSUJI, MOE MUKAI

#### University of Fukui, Fukui, Japan

**Introduction:** When the precordial transition zone (TZ) of idiopathic outflow tract arrhythmias (OTAs) is present around lead V3, it may be difficult to precisely determine which outflow tract is responsible for the origin of the OTAs from the ECG characteristics. The purpose of this study is to determine whether activation mapping around the distal great cardiac vein (dGCV) during OTA is useful in identifying whether the origin of OTA is right‐sided or left‐sided.

**Methods:** Among 28 OTA patients who underwent dGCV mapping and successful radiofrequency catheter ablation at our institution from June 2021 to February 2024, 16 whose TZ during the OTAs was >lead V2 and <lead V4 were included in this study. In all patients, activation mapping around the dGCV with the novel 2.7 Fr over‐the‐wire (OTW)‐type decapolar catheter (EP star Fix AIV^TM^, Japan Lifeline, Tokyo, Japan) was performed during the OTAs. Ablation was performed using an irrigated catheter.

**Results:** In all patients, the mapping catheter could be easily placed in the dGCV. In patients whose OTAs were successfully ablated from the left‐sided OT (n=7), the local ventricular activation time in the dGCV during OTAs preceded the QRS onset (LAT‐dGCV) by ‐26.1±7.0 msec, which was significantly earlier than that of OTAs successfully ablated from the right‐sided OT (n=9; ‐4.0±5.7 msec; p<0.001). An LAT‐dGCV >‐18 msec indicated a left‐sided OTA, and right‐sided and left‐sided OTAs were completely distinct. Patients with an LAT‐dGCV >‐18 msec and mapping initiated first in the left‐sided OT had shorter procedure times than those with mapping initiated first in the right‐sided OT (p<0.001).

**Conclusions:** Mapping the dGCV with this OTW‐type catheter is easy and safe. The earliness of the local ventricular activation in the dGCV during OTAs could easily and precisely differentiate between right‐sided and left‐sided OTAs, resulting in a successful ablation with a shorter procedure time.

## RECURRENCE OF VENTRICULAR FIBRILLATION IN A PATIENT, ACCOMPANIED BY INTERMITTENT J WAVE MANIFESTATION DURING BRADYCARDIA

### **MINORU TAGAWA** ^1^, YUICHI NAKAMURA^1^, KOUICHI FUSE^2^, SEIKO OHNO^3^, MASAOMI CHINUSHI^4^

#### ^1^Nagaoka Chuo General Hospital, Nagaoka, Japan,^2^Tachikawa Medical Center, Nagaoka, Japan,^3^Department of Bioscience and Genetics, National Cerebral and Cardiovascular Center, Osaka, Japan,^4^Graduate School of Health Science, Niigata University School of Medicine, Niigata, Japan

**Introduction:** N/A

**Methods:** N/A

**Results:** A 59‐year old man suddenly fell unconscious in front of his family because of ventricular fibrillation (VF) around 5:10 a.m. VF was terminated by a single 150J shock administered in an ambulance. He had neither episodes of syncope nor a family history of sudden death. His electrocardiogram (ECG) upon admission to our hospital showed sinus rhythm with a heart rate of 53 bpm and mild ST‐depression in leads I, II, aVF, and V3‐V6. His serum potassium level was low at 3.2 mEq/L. During hypothermia therapy (34°C, 12 hours), his heart rate was intermittently decreased to about 40 bpm, and Osborn waves (I, II, aVL, and V2‐V6) and QT interval prolongation (600ms) were recorded. After his temperature was normalized, the QT interval was in normal range, but J waves were still sometimes recorded at the slower heart rates. Epinephrine infusion prolonged the QTc interval from 381 ms to 506 ms, but genetic screening for arrhythmia‐related genes was negative. Brugada‐type ST‐T changes were not induced with a pilsicainide provocation test. Cardiac MRI and coronary angiogram were normal and coronary vasospasm was not induced. VF was not induced by programmed electrical stimulation. While waiting for an implantable cardioverter‐defibrillator (ICD) procedure in the hospital, the patient lost consciousness again, because of a VF recurrence, following a sinus rhythm of 40 to 50 bpm and J wave elevation at rest. After starting 70 bpm pacing from an ICD, the J wave has not been observed, and VF has not recurred for six years.

**Conclusions:** In this patient, a slower heart rate and lower temperature, both of which were induced by hypothermia therapy,allowed for important observations to be made to reach a diagnosis of J wave‐related VF.

## FIRST IN ASIA: VT ABLATION IN PATIENTS WITH MECHANICAL AORTIC AND MITRAL VALVES USING RIGHT ATRIUM TO LEFT VENTRICLE APPROACH

### **BAI SITTI AMEERAH ASLEAH TAGO**, CHIN‐YU LIN, TING‐YUNG CHANG

#### Taipei Veterans General Hospital, Taipei, Taiwan

**Introduction:** Ablating ventricular arrhythmias in the presence of mechanical aortic and mitral valves presents significant challenges.

**Methods:** N/A

**Results:** A 59‐year‐old male with aortic and mitral mechanical valves was admitted for syncope and episodes of ventricular tachycardia (VT)/ventricular fibrillation (VF) with implantable cardioverter‐defibrillator device (ICD) shock were recorded.The procedure protocol consisted of Carto system, STSF ablation catheter, Decanav mapping catheter, Boston Scientific V‐18 Control wire 0.018x 300cm, Boston Scientific Mustang 10mmx40mmx135mm, ST‐01 catheter, Agilis sheath, 0.032mm wire, intracardiac echocardiography (ICE), Abbott, and TEE image. An Agilis sheath was inserted and placed at the right atrium(RA). The 0.032 wire was inserted into the Agilis and, guided by ICE, positioned at the inferior RA where the puncture was made using cautery (40 Watts) with the tail of the 0.032 wire (Figure 1A). The 0.032 wire and ST‐01 catheter were advanced into the puncture site (Figure 1B). The 0.032 wire was replaced with steel wire (Figure 1C) and was exchanged for a Boston Scientific Control wire (Figure 1D). The ST‐01 was changed with a Boston Scientific Mustang balloon catheter (Figure 1D). Once puncture site was dilated, the Agilis was inserted into the LV with the dilator. Substrate modification was performed at the mid to low basal LV septum and basal free wall. On repeat echocardiography one month after the procedure, a small residual shunt was still noted.Our patient has a similar approach to Dr. Santangeli's proposed technique.^1^ We modified the technique by using cautery‐guided puncture of the access site as the ablation wire was not available in Asia. This is the first reported case in Asia using this approach.

**Conclusions:** VT ablation with right atrium to left ventricle access is feasible and safe in patients with both mechanical aortic and mitral valve with the traditional equipment in electrophysiological laboratory. This is the first reported case in Asia using this approach.

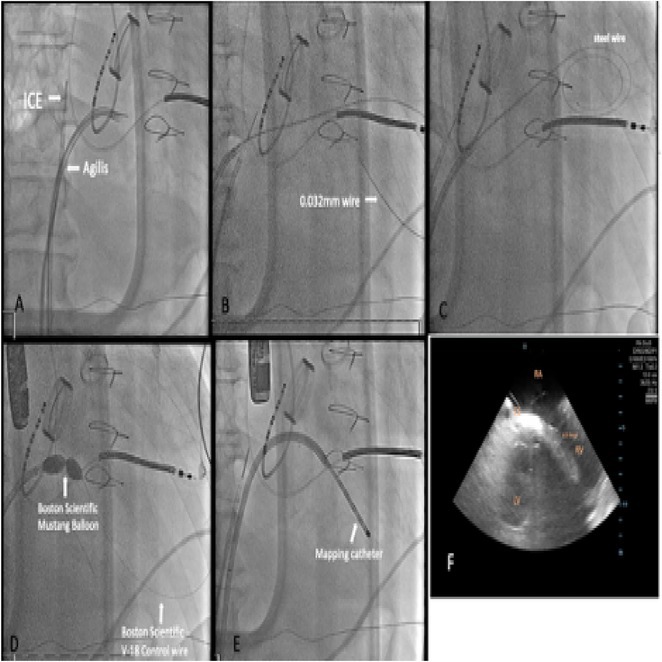

## NONFLUOROSCOPIC CATHETER ABLATION OF LONG‐STANDING PERSISTENT ATRIAL FIBRILLATION IN A YOUNG ADULT WITH SITUS INVERSUS CONGENITALLY CORRECTED TRANSPOSITION OF THE GREAT ARTERIES AND HEART FAILURE WITH REDUCED EJECTION FRACTION

### **I HSIN TAI** ^1^, CHIEH‐MAO CHUANG^2^, PI‐CHANG LEE^2^, CHENG‐HUNG LI^2^, YU‐CHENG HSIEH^2^, GUAN‐YI LI^2^, SHANG‐JU WU^2^, MING‐JEN KUO^2^, SHIH‐ANN CHEN^2^

#### ^1^China Medical University Children's Hospital, Taichung, Taiwan,^2^Taichung Veterans General Hospital, Taichung, Taiwan

**Introduction:** Congenitally corrected transposition of the great arteries (ccTGA) develops heart failure with reduced ejection fraction (HFrEF) in early decades of life because of using RV as systemic ventricle. Atrial fibrillation (AF) often occurs subsequently due to dilated pulmonary venous atriam (PVA) with severe tricuspid regurgitation. Catheter ablation is well‐established treatment for AF with HFrEF. However, there is limited report about AF ablation in severe form of ACHD with HF.

**Methods:** NA

**Results:** A 39‐year‐old male had situs inversus with ccTGA, VSD, and pulmonary atresia s/p Rastelli & mechanical TVR, HFrEF, and long‐standing persistent atrial fibrillation. After guideline‐directed medical therapy for HF and rhythm control with amiodarone and bisoprolol, exertional dyspnea with palpitation persisted with a high NT‐pro‐BNP level (2504 pg/ml). Pre‐ablation cMRI showed a sys. RVEF of 9.47% and dilated PVA (136 ml) with a moderate amount of late gadolinium enhancement. Due to previous atrial flutter history, cavomitral isthmus linear ablation was conducted. Transseptal puncture through thickened and fibrotic septum by RF needle with TEE surveillance. We used HD grid mapping catheter to create geometry under AF and a force‐sensing ablation catheter (Taciticath, Abbott) to perform PVI supported by a steerable long sheath (Agilis sheath; St. Jude Medical). Partial LIPV anterior wall ablation was hindered due to the transseptal site's proximity to LIPV. After four PVs isolated, AF persisted. A 100J synchronized cardioversion restored sinus rhythm, but profound sinus bradycardia with hypotension. Temporary atrial pacing using coronary sinus catheter was done. Despite an acute recurrence within 3 months, synchronized cardioversion was administered once, and the patient remained AF‐free for over 1 year and the sys. RV function improved (Post cMR sRVEF 23.9%, NT proBNP<500).

**Conclusions:** Catheter ablation of LsPAF in a complex CHD with HFrEF via the nonfluoroscopic method is safe and feasible.

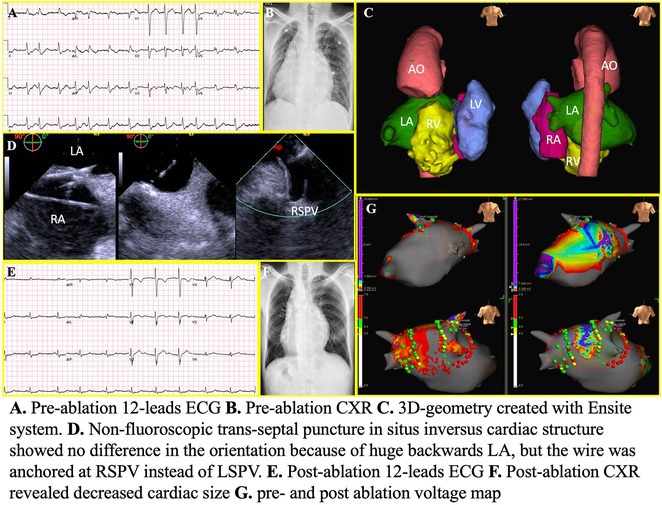

## RISK FACTORS FOR INTRAOPERATIVE INSTABILITY IN SEDATED PATIENTS UNDERGOING PULMONARY VEIN ISOLATION ABLATION

### **TOMOKO TAKAHASHI** ^1^, TAKESHI SOEKI^2^, TOMOMI MATSUURA^1^, KAZUKI TEZUKA^1^, AKIHIRO TANI^1^, RYO BANDO^1^, ROBERT ZHENG^1^, TOMONORI TAKAHASHI^1^, YOSHIHITO SAIJO^1^, TOMOYA HARA^1^, MUNEYUKI KADOTA^1^, YUTAKA KAWABATA^1^, RIE UENO^1^, TAKAYUKI ISE^1^, KOJI YAMAGUCHI^1^, SYUSUKE YAGI^3^, HIROTSUGU YAMADA^4^, TETSUZO WAKATSUKI^1^, MASATAKA SATA^1^

#### ^1^Department of Cardiovascular Medicine, Tokushima University Graduate School of Biomedical Sciences, Tokushima, Japan,^2^Department of Community Medicine and Medical Science, Tokushima University Graduate School of Biomedical Sciences, Tokushima, Japan,^3^Department of Community Medicine and Family medicine, Tokushima University Graduate School of Biomedical Sciences, Tokushima, Japan,^4^Department of Community Medicine for Cardiology, Tokushima University Graduate School of Biomedical Sciences, Tokushima, Japan

**Introduction:** Persistent or paroxysmal atrial fibrillation is typically treated with pulmonary vein isolation (PVI) ablation under deep sedation with propofol. Intraoperative hemodynamic or respiratory instability often interferes with the surgical procedure. Therefore, in the present study, we retrospectively investigated risk factors for intraoperative instability in 80 patients who underwent their first PVI ablation for atrial fibrillation at our hospital.

**Methods:** Background and echocardiography findings were collected from their electronic charts and the questionnaires they completed during hospitalization. Total intraoperative propofol dose and bolus injections (total number and volume) were defined as surrogate measures of patient instability. Single and stepwise multiple regression were performed using each measure as the dependent variable.

**Results:** When total propofol dose was employed as the dependent variable, significant associations were observed with drinking status (P <0.05 ) and body mass index (BMI) (P <0.05). When total number or volume of intravenous propofol boluses were each used as the dependent variable, significant associations were noted with age (P <0.05) and BMI (P <0.05). Separately, statistical analyses were conducted using total propofol dose or total number of bolus injections as the dependent variable and echocardiography parameters as independent variables. A significant association was detected between total dose and left atrial dimension (P <0.05).

**Conclusions:** These results suggested that younger age, higher BMI (obesity), and current drinking status adversely affect patient stability under deep sedation. To ensure safe ablation, physicians should pay attention to these risk factors when administering deep sedation for PVI.

## IMPACT OF METABOLIC DYSFUNCTION‐ASSOCIATED FATTY LIVER DISEASE ON ATRIAL FIBRILLATION RECURRENCE IN JAPANESE PATIENTS

### **AIKO TAKAMI** ^1,2^, MASARU KATO^1^, YASUHITO KOTAKE^1^, AKIHIRO OKAMURA^1^, TAKUYA TOMOMORI^1^, SHUNSUKE KAWATANI^1^, KAZUHIRO YAMAMOTO^1^

#### ^1^Tottori University, Yonago, Japan,^2^Tottori Prefectural Central Hospital, Tottori, Japan

**Introduction:** Metabolic derangements, including obesity and diabetes, are associated with incident and recurrent atrial fibrillation (AF), in addition to the development of metabolic dysfunction‐associated fatty liver disease (MAFLD). Recently, there was reported MAFLD was associated with significantly increased arrhythmia recurrence rates following AF ablation in Western patients. However, it has been reported that Asian people have relatively lower body mass index values compared with Western people, it was not clear whether MAFLD affects recurrence after AF ablation in Japanese patients.This study aimed to determine the impact of MAFLD on AF recurrence in Japanese patients.

**Methods:** We enrolled 872 patients with paroxysmal or persistent AF who received ablation procedure and assessed the relationship between MAFLD and the AF recurrence. Fifty‐four patients with other causes of chronic hepatitis or a history of drinking were excluded. Patients who had an abdominal computed tomography scan were calculated the liver/spleen (L/S) ratio. Liver fibrosis scores were also collected.

**Results:** Recurrent arrhythmia was observed in 59/154 (38.3%) patients with MAFLD and 149/664(22.4%) patients without MAFLD. Although L/S ratio was significantly lower among those with AF recurrent patients, liver fibrosis scores were not significantly different for AF recurrence. The multivariate Cox proportional hazards regression analysis identified MAFLD as independent predictor of AF recurrence (adjusted HR 1.78; 95% CI [1.26 ‐2.51]; p<0.05). We further performed subgroup analyses of risks of the recurrence of AF for MAFLD by dividing patients with the risk variables and the interaction effects of MAFLD with other risk variables. We found not confounding effect, but the significant interactions of MAFLD with Homeostatic model assessment for insulin resistance (p for interaction = 0.019).

**Conclusions:** MAFLD was an independent risk factor for recurrence after AF ablation, and necessary to taking account of the effects of insulin resistance.

## IMPACT OF CONTINUOUS RAPID‐MODE ABLATION WITH A THIRD‐GENERATION LASER BALLOON

### **YUKIKO TAKANASHI** ^1^, YUSUKE KONDO^1^, MASAHIRO NAKANO^2^, TAKATSUGU KAJIYAMA^3^, MIYO NAKANO^1^, TOSHINORI CHIBA^2^, SATOKO RYUZAKI^3^, YUTAKA YOSHINO^1^, YUYA KOMAI^1^, YOSHIO KOBAYASHI^1^

#### ^1^Chiba University Graduate School of Medicine Department of Cardiovascular Medicine, Chiba, Japan,^2^Chiba University Graduate School of Medicine Department of Advanced Cardiorhythm Therapeutics, Chiba, Japan,^3^Chiba University Graduate School of Medicine Department of Advanced Arrhythmia Bioengineering, Chiba, Japan

**Introduction:** The visually‐guided laser balloon (VGLB) is useful for pulmonary vein isolation (PVI) for paroxysmal atrial fibrillation (PAF) by delivering laser energy to the cardiac tissue under real‐time endoscopic visualization. Currently, the third generation of VGLB is available in Japan, following the first generation of VGLB. The third generation of VGLB is equipped with the “RAPID mode,” which enables automatic continuous energy irradiation, whereas the first generation is point by point manual irradiation. Compared to the first generation, the procedure using the third generation can be performed in a shorter time. However, there are few reports from Japan on its safety and efficacy. The aim of this study was to clarify the treatment outcomes of each generation of VGLB.

**Methods:** Eighty‐two consecutive patients (female, 25 (31%); mean age, 63.4±11.8 years) who underwent VGLB for PAF between February 2019 and November 2022 were included in this study. We retrospectively analyzed our ablation database and investigated patient background, treatment information, and outcomes.

**Results:** Sixty‐six patients (80.5%, group X1) were in the first generation of Heart Light and 16 patients (19.5%, group X3) were in the third generation. Between two groups, there was no significant difference in the patient baseline characteristics except for the median follow‐up period (745 days vs 390 days, p=0.001). All patients had a successful acute PVI procedure. Although there was no significant difference in the rate of PVI first attempt (59% vs. 62.5%, p=1), the procedure times were 118.9±30.3 minutes and 98.8±17.6 minutes, respectively (p=0.013). Between two groups, there was no significant difference in major complication rate (4.5% vs 12.5%, p=0.25), and the recurrence rate of PAF at one‐year follow‐up (6.2% vs 12.5%, p=0.338). Six patients (group X1: 5 patients, group X3: 1 patient, 24PVs) underwent a second procedure because of recurrent AF. There were 6 gaps; case 2 was successful PVI (Figure).

**Conclusions:** The third‐generation VGLB could help achieve the reduction of procedure time without compromising the safety in the clinical real‐world settings.

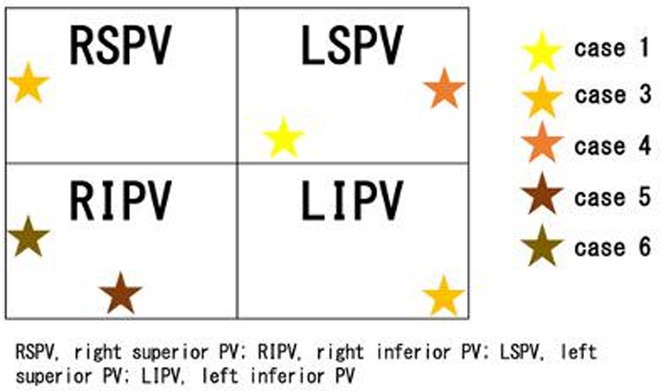

## IMPACT OF INTER‐LESION DISTANCE ON OUTCOMES OF PULMONARY VEIN ISOLATION USING VERY HIGH‐POWER SHORT‐DURATION ABLATION

### **TESTURO TAKASE**, YOSHIO FURUKAWA, AKIRA SHINODA, NOBUKIYO TANAKA, AKIFUMI MITSUSHIMA

#### Ichinomiya Nishi Hospital, Ichinomiya, Japan

**Introduction:** A novel contact‐force sensing temperature‐controlled radiofrequency ablation catheter allows for very high‐power short‐duration (vHPSD, 90 W/4s) ablation aiming a potentially safer, more effective and faster ablation. However, previous studies have shown that pulmonary vein isolation (PVI) with vHPSD is often associated with low first‐pass isolation rate with gaps most commonly found in the carina region owing to shallower lesions created. We thought to evaluate the impact of inter‐lesion distance(ILD) on first‐pass isolation rate during PVI using vHPSD.

**Methods:** We conducted a randomized trial to determine whether tightening ILD in carina region from 4mm to 2mm would improve first‐pass isolation rate in patients with atrial fibrillation solely utilizing vHPSD. ILD for non‐carina region was 4mm for both groups.

**Results:** A total of 90 patients (mean age 71 years old, 33% non‐paroxysmal) underwent randomization (39 assigned to ILD 2mm and 51 assigned to ILD 4mm group). The median(±SD) RF time was 32(±8) min in 2mm and 23(±10) min in 4mm‐group; p = 0.002. The median(±SD) procedural time was 103(±28) min in 2mm and 99(±22) min in 4mm‐group; p =0.37. First pass isolation rate was similar in both groups (84 % (2mm) and 88 % (4mm), p = 0.888). Number of Gaps located in carina sectors (2mm: 21/195, 11% vs. 4mm: 25/255, 9.8 %; p = 0.73) and non‐carina sectors (2mm: 8/351, 2.2% vs. 4mm: 17/459, 3.7%; p = 0.24) were not different. There were no severe adverse events in both groups and no differences for char formation (2mm: 4/39, 10% vs. 4mm: 4/51, 7.8 %; p = 0.69) or acute reconnection after adenosine challenge (2mm: 2/39, 9% vs. 4mm: 1/51, 2%; p = 0.69).

**Conclusions:** In this randomized trial, tightening ILD from 4mm to 2mm in carina region increase ablation time without improving first‐pass isolation rate during PVI using vHPSD.

## A CASE OF ATRIAL TACHYCARDIA ARISING FROM SUPERIOR VENA CAVA

### **SOU TAKENAKA**, AKIHIKO UENO, SOICHIRO OGURA, DAISUKE IIDA, NOBUHIRO HARA, TOMOHIRO OKUNO, MASAYOSHI SAKAKIBARA

#### IMS Katsushika Heart Center, Tokyo, Japan

**Introduction:** Atrial tachycardia of superior vena cava origin is rarely reported.

**Methods:** N/A

**Results:** A 68‐year‐old man was undergoing mitral valvuloplasty and was aware of palpitations preoperatively. A short time after mitral valvuloplasty, he became aware of palpitations and flashes and visited his local doctor, who performed an electrocardiogram that showed supraventricular tachycardia. The patient was referred to our hospital for emergency care, and after 20 mg of intravenous ATP was administered, 2:1 atrioventricular conduction was achieved, but the tachycardia did not stop, and a diagnosis of atrial tachycardia was made. The tachycardia was sensitive to verapamil and pilsicainide. Cardiac electrophysiologic studies were performed. Atrial tachycardia did not occur with programmed stimulation, but spontaneously occurred with isoproterenol loading. The earliest site was on the septal aspect of the superior vena cava. Atrial tachycardia did not stop with radiofrequency current at the earliest site, and a 20‐pole ring catheter was placed in the superior vena cava for superior vena cava isolation. After isolation, the patient was repeatedly electrical firing from the superior vena cava. More than 3 years have passed since the ablation without recurrence.

**Conclusions:** We report a case of atrial tachycardia of unusual superior vena cava origin treated with superior vena cava isolation.

## LAZER BALLOON ABLATION OF ATRIAL FIBRILLATION IN A PATIENT WITH A LARGE COMMON INFERIOR TRUNK

### TOMOHIRO TAKIGUCHI

#### Steel Memorial Yawata Hospital, Kitakyushu‐Shi Fukuoka‐Ken, Japan

**Introduction:** A balloon‐based visually guided laser balloon (LB) ablation (LBA) is as effective and safe as radiofrequency ablation and cryoballoon ablation in curing atrial fibrillation (AF) patients. The third generation LB is so compliant that it can be inflated to any pressure and size change of up to 41 mm with its maximal expansion, which enables maximum balloon/tissue contact regardless of the size or shape of each pulmonary vein (PV) ostium. A large common inferior trunk (CIT) with a structured, completely independent common ostium of both the right and left inferior PVs completely conjoined prior to the junction with the left atrium is an extremely rare anatomical variant and an important triggering focus in paroxysmal AF.

**Methods:** N/A

**Results:** We present a case of LB ablation of AF in a patient with a large CIT of 34 mm in diameter. The laser energy was individually deployed to the right‐ and left‐sided antrum of the large CIT with the LB positioned at the ostium of the CIT's right and left branch. The complete electrical isolation of the three pulmonary veins was achieved. The patient remained stable without any symptoms or AF recurrence 1‐year post‐ablation.

**Conclusions:** The LBA, which is individually deployed to the right‐ and left‐sided antra of the large CIT with the third generation‐LB positioned at the ostium of the right and left branches of the CIT without laser energy deployment to the posterior wall of the CIT, may be one of the effective strategies for patients with large CITs.

## FIRST STEPS OF A BURGEONING CATHLAB: A 6 YEAR MILESTONE OF PREMATURE VENTRICULAR COMPLEX ELECTROPHYSIOLOGICAL STUDY AND ABLATION

### **MOHAMMAD ZULKHAIRI RHIZA TALA**, ADRA ACHIRULTAN RAMAINALDO SUGIARTO, RERDIN JULARIO, BUDI BAKTIJASA DHARMADJATI, RAGIL NUR ROSYADI, MAKHYAN JIBRIL AL‐FARABI, RUTH IRENA GUNADI

#### Soetomo Regional General Hospital, Surabaya, Indonesia

**Introduction:** Premature ventricular complex (PVC) is a routinely encountered cardiac arrhythmia where a burden of >10% can lead to cardiomyopathy and heart failure. Studies have demonstrated that catheter ablation is more effective than pharmacotherapy for eliminating PVCs. There is a lack of study that examines the broad demographic picture of EP Study (EPS) & Ablation on PVCs in Indonesia. Therefore this study aim to assess the overall characteristics, success, and complication rate of EPS & ablation on PVCs in our Healthcare center in Surabaya, Indonesia from its very first attempt.

**Methods:** This Retrospective study uses cross sectional approach. The subjects of this study are PVC patients, whose inclusion criteria were diagnosed with PVC through ECG and/or Holter monitoring and underwent EPS and/or ablation in Dr. Soetomo Regional General Hospital from December 2018 to April 2024, with samples obtained through total sampling.

**Results:** There were 95 patients with PVC, mostly female (57.9%) that went through EPS and/or ablation. Main complaint being palpitation (87.4%), and almost half have no comorbidities (49.5%). The procedures netted a success rate of 58.9%. 7 (7.37%) patients underwent EPS only procedure, with the main excuse being the ablation procedure required a catheter upgrade. Bigeminy is the most prevalent pattern (61%) with 56.9% success rate. The typical origin of PVC is RVOT (55.8%) with 73.6% success rate. The morphology is primarily unifocal (87.4%) with 56.6% success rate. Most patients have frequent PVC (81%) with 58.4% success rate. Overall complication rate is miniscule (2.11%), highest being Cardiac tamponade.

**Conclusions:** In our study, patients with palpitation without comorbidities is the most common denominator. Moreover, unifocal & RVOT is the most common types of PVC. Despite the low complication rate, the learning curve of the procedures affected the general success rate. With further experience, improvements in success rate will be possible in future procedures in this center.

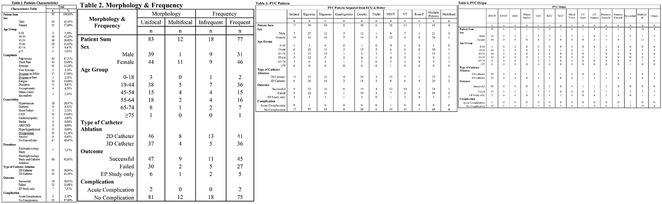

## CIED MONITORING TRENDS, MANAGEMENT AND OUTCOMES IN PATIENTS WITH HEART FAILURE HOSPITALISATION AT A TERTIARY CENTRE

### **JUSTIN TAN** ^1,2^, JOSEPH ASSAD^1,2^, HANY DIMITRI^1,2^

#### ^1^University of New South Wales, Sydney, Australia,^2^Liverpool Hospital, Sydney, Australia

**Introduction:** Heart failure (HF) is a significant and increasingly prevalent condition which involves costly hospitalisations with poor patient outcomes. Implantable cardioverter‐defibrillators (ICDs) and cardiac resynchronisation therapy defibrillators (CRT‐Ds) are devices that can be used to remotely monitor patient health. Algorithms such as HeartLogic incorporate multiple parameters recorded by these devices and can predict impeding HF decompensation. The aim of this study was to identify trends in clinical and device‐collected data prior to HF hospitalisation, and to evaluate pharmacological management during hospitalisation.

**Methods:** This study retrospectively collected clinical data from 177 patients with ICDs or CRT‐Ds admitted to Liverpool Hospital for HF between 2018 and 2021. Remote monitoring data was available for 73 patients for which device data was collected. Values were taken from 3 points in time ‐ 2 months (taken as a baseline), 1 month, and 1 week prior to the date of admission.

**Results:** The average number of HF ‘pillar’ medications increased from 2.00 to 2.17 during admission. HeartLogic was significantly greater and thoracic impedance significantly lower 1 week prior to admission than 2 months prior to admission. HeartLogic alerts were elevated above the alert threshold for 78% of admissions with a median alert time of 45 days and fall time of 36.5 days.

**Conclusions:** Pharmacological management of HF is suboptimal compared to the guideline standard of 4 HF ‘pillar’ medications. HeartLogic is successful in predicting HF hospitalisations and can be used to guide early treatment to improve outcomes. Remote monitoring is a valuable yet underutilised tool that can identify and prevent HF hospitalisations.

## OUTCOMES OF NON‐VITAMIN K ANTAGONIST ORAL ANTICOAGULANT (NOAC) IN PATIENTS WITH MITRAL STENOSIS IN CAMBODIA

### RITHY PONG TAN

#### Khmer‐Soviet Friendship Hospital, Phnom Penh, Cambodia

**Introduction:** Atrial fibrillation (AF) is the most common cardiac arrhythmia which remains one of the major causes of stroke, heart failure, sudden death, and cardiovascular morbidity in the world. For the prevention of stroke in patients with atrial fibrillation (AF) and mitral stenosis, anticoagulation is crucial. Patients suffering from moderate to severe mitral stenosis have not been included in any research conducted thus far on non‐vitamin K antagonist oral anticoagulants (NOACs).The aim of this study was to evaluate the efficacy of NOACs (Apixaban and Rivaroxaban) in patients with mitral stenosis for 3 months.

**Methods:** The study was enrolled from the patients of Khmer‐Soviet Friendship hospital in Cambodia, and it included patients who were diagnosed with moderate to severe mitral stenosis and AF and either were prescribed NOACs such as Apixaban and Rivaroxaban for off‐label use. The primary efficacy endpoint was ischemic strokes and intracranial hemorrhage.

**Results:** A total of 40 patients (mean age 53.1; 14 [35%] males; 26[65%] females) were included in the present study. Patients using Rivaroxaban were 22[55%] and 18[45%] under Apixaban. In the two NOAC groups, the rate of strokes was 2% for three months, the rate of cerebral bleeding was 0% for three months, and 98% of the groups experienced no problems. Within the Rivaroxaban group, the rate of strokes was 5%[1 patients], the rate of cerebral hemorrhages was 0%, and 95%[21 patients] of cases had no complications. In addition, 0% of the Apixaban group experienced strokes, 0% experienced no complications from cerebral hemorrhage, and 100%[18 patients] experienced no complications at all.

**Conclusions:** The rate of thromboembolism and intracranial hemorrhage is low for patients with mitral stenosis using NOAC. Even though thromboembolism events still occur but the effectiveness of NOAC is acceptable for developing countries. Furthermore, Apixaban is superior than Rivaroxaban in reducing thromboembolic and hemorrhagic events. Our data is limited, so further study is still being conducted and is going to be more in the future about the efficacy of NOAC in mitral stenosis and atrial fibrillation.

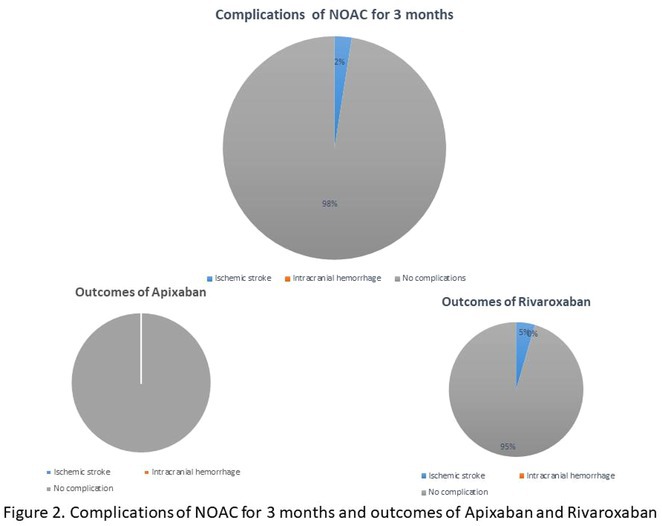

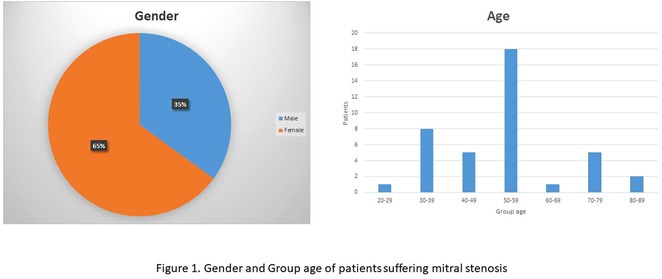

## PREDICTIVE FACTORS OF RECURRENT ATRIAL TACHYARRHYTHMIAS AFTER SIMPLE PULMONARY VEIN ISOLATION WITH CRYOBALLOON FOR ATRIAL FIBRILLATION

### **MARIKO TANAKA**, SHINYA HAYASHI, RYOTA KIDA, TOSHIHIKO TAYA, YOSHIAKI SHIMODA, TETSUYA TANAKA, MORIHIKO KONDO

#### JCHO Kobe Central Hospital, Kobe, Japan

**Introduction:** Recurrent atrial tachyarrhythmias(RATs) can occur after pulmonary vein isolation (PVI) with a cryoballoon(CB) for atrial fibrillation(AF), despite the device's excellent performance for PVI.

**Methods:** 93 consecutive new patients undergoing first‐time PVI with CB during the period from April 2020 to June 2023 were studied retrospectively. PVI was performed with a CB (Arctic Front Advance^TM^, Medtronic) and with a 3.5 mm irrigated catheter with contact force as a touch‐up if necessary. All patients received PVI only and did not receive linear ablation, defragmentation, or non‐PV focus ablation. In all cases, CARTO3 or EnSite NavX was used as the 3‐dimensional mapping system. Medical history, blood tests, echocardiography, pre‐catheter ablation (CA) computed tomography and ablation data were assessed. The primary endpoint was RATs >= 30 seconds after 90 days of CA.

**Results:** Mean age was 71.4 years old, 42 were men, mean follow‐up term was 2.0 years, and 33 patients had persistent AF(PeAF). A total of 22 patients (23.7% of the total) had RATs (paroxysmal AF 10, PeAF 12). Univariate logistic analysis showed that RATs was significantly more frequent in patients with PeAF, previous heart failure, left atrial (LA) diameter >40mm, LA volume index >40ml/m^2^, left superior PV(LSPV) major axis >20mm, and that LA low voltage area(LVA) defined as <0.5 mV on a 3D map calculated by CARTO3 or EnSite NavX >2cm^2^, and that tended to be more common in patients with eGFR<60 ml/min/1.73m^2^, BNP >100pg/ml, right superior PV(RSPV) major axis >20mm, lowest RSPV temperature >‐53°C at freezing, and lowest right inferior PV temperature >‐47°C. Multivariate logistic analysis showed that RSPV major axis>20mm and LVA>2cm^2^ were predictive factors for RATs after PVI with CB (RSPV: OR=14.6, 95%CI: 1.5‐140.1, p=0.0091, LVA: OR=6.3, 95%CI: 1.0 ‐ 40.8, p=0.0428).

**Conclusions:** LVA>2cm^2^ and RSPV major axis>20mm were predictive factors for RATs after CB ablation. In other words, LA tissue damage and PV morphology are strongly associated with RATs after PVI with CB.

## EVALUATION OF EPICARDIAL CONDUCTION IN PERI‐MITRAL ATRIAL TACHYCARDIA USING ULTRA‐HIGH‐DENSITY MAPPING

### **MUNEKAZU TANAKA**, SATOSHI SHIZUTA, TOMOYUKI INOUE, AKIFUMI MORINAGA, FUMIYA YONEDA, SHUSHI NISHIWAKI, REO HATA, HIROHIKO KOUJITANI, KOH ONO

#### Kyoto University Graduate School of Medicine, Kyoto‐shi, Japan

**Introduction:** Peri‐mitral atrial tachycardia (PMAT) often involves epicardial conduction through the Marshall bundle (MB). The aim of this study is to analyze the characteristics of the PMAT related to conduction through the MB using LUMIPOINT™.

**Methods:** We analyzed 80 patients with PMAT. When there was an obvious missing area of endocardial conduction at the site where the Marshall bundle crosses the posterior mitral isthmus (the Marshall area), PMAT was diagnosed to involve MB conduction in the circuit and was defined as MB‐PMAT. The length of the epicardial conduction, which was missing in the endocardial activation map, was measured. Furthermore, we assessed whether epicardial conduction through the MB could be visualized by reducing the strictness criteria of LUMIPOINT™. Subsequently, we analyzed whether the Marshall area could be highlighted using the peak slider algorithms of LUMIPOINT™. When the Marshall area was highlighted, the highlighted area was measured.

**Results:** MB‐PMAT was observed in 10 out of 80 patients (12.5%). Epicardial conduction through the MB was successfully visualized in 6 out of 10 patients (60%) by reducing the strictness criteria of LUMIPOINT™ from 85% to 20%. The length of epicardial conduction was longer in the failed visualization group compared to the successful visualization group (31.1± 0.9 mm vs. 19.0 ± 4.5 mm, P=0.001) (Figure A‐C). Additionally, in MB‐PMAT, the Marshall area highlighted by the peak slider algorithms was larger compared to non‐MB‐PMAT (0.59 ± 0.76 cm^2^ vs. 0.25 ± 0.45 cm^2^, P=0.048) (Figure D).

**Conclusions:** LUMIPOINT™ could delineate epicardial conduction through the MB in 60% of patients with MB‐PMAT. In MB‐PMAT, the Marshall area is more likely to be highlighted by using LUMIPOINT™.

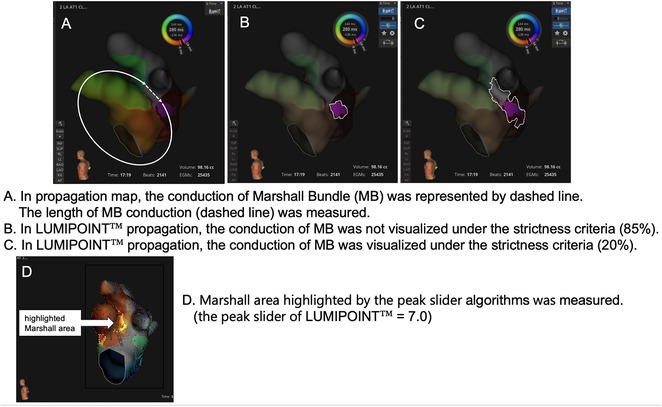

## EFFICACY OF VERY HIGH POWER SHORT DURATION FOR SVCI

### **NAOMICHI TANAKA**, DAISUKE KUDO, WATARU SASAKI, MASATAKA NARITA, TSUKASA NAGANUMA, KAZUHISA MATSUMOTO, HITOSHI MORI, YOSHIFUMI IKEDA, RITSUSHI KATO

#### Saitama Medical University International Medical Center, Hidaka‐City, Saitama‐Pref, Japan

**Introduction:** Previous studies have demonstrated the efficacy of Very High Power Short Duration (vHPSD) for Pulmonary Vein Isolation (PVI) in treating Atrial Fibrillation (AF). However, the efficacy and safety of vHPSD for Superior Vena Cava Isolation (SVCI) remain unclear.

**Methods:** We investigated 62 cases treated with Qdot Micro (QDM) QMODE+ for SVCI at our institution, focusing on the rate of 1st pass isolation, dormant conduction, and complications. With CARTO net, We also analyzed maximum temperature, impedance drop, and total energy of all ablation site during SVCI.

**Results:** The patients' ages averaged 68±10 years, with 35 cases (56.5%) had paroxysmal AF and 27 cases (43.5%) had chronic AF. And, 10 (16.1%) cases were in the second session of AF. 1st pass isolation was achieved in all 62 cases (100%), and no dormant conduction was observed in any case. The phrenic nerve injury or sinus node injury was not observed in any case. A total of 505 ablation points were analyzed, showing the impedance drop of 13.21±4.91Ω, total energy of 283.2±58.2J, and maximum temperature of 55.64±2.94°C.

**Conclusions:** The high rate of 1st pass isolation and low incidence of dormant conduction showed the efficacy of vHPSD mode in SVCI. Analysis of the ablation points indicates that adequate temperature rise and impedance drop were achieved for effective lesion formation. There were no specific complications like sinus node dysfunction or phrenic nerve paralysis that could be attributed to SVCI. Overall, SVCI using QDM QMODE+ might be as effective and safe as previously reported for PVI.

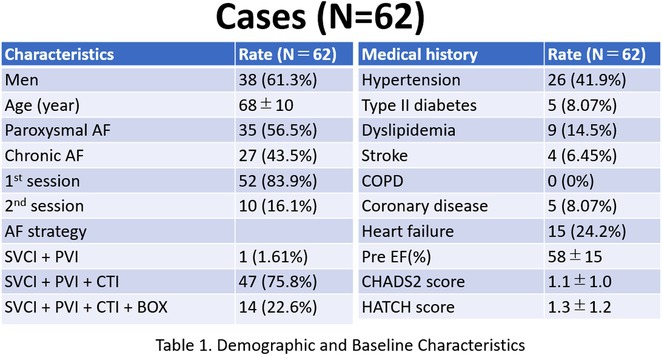

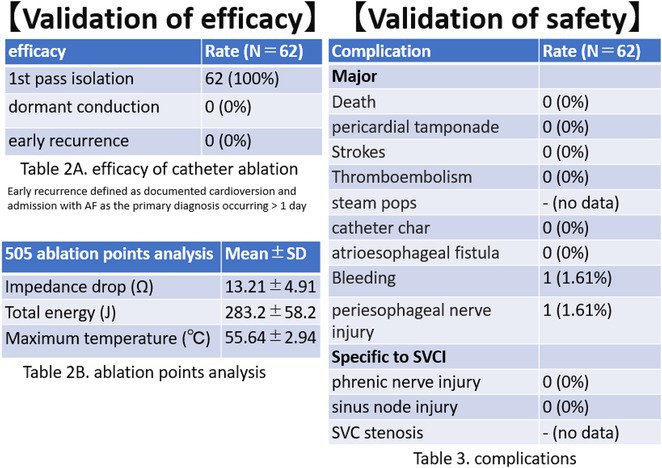

## ELECTRICAL STORM ABLATION : SALVAGE THERAPY OR WEATHERING THE STORM. A BRUGADA ‐ ELECTRICAL STORM CASE REPORT

### **YUDHIE TANTA** ^1,2^, NGUYEN VAN DANG^1^, TON NU KHANH AN^1^, TRAN CAO QUOC^1^, DO VAN BUU DAN^1^

#### ^1^Department of Electrophysiology, Tam Duc Cardiology Hospital, Ho Chi Minh City, Viet Nam,^2^Faculty of Medicine, Sriwijaya University, Palembang, Indonesia

**Introduction:** Electrical storm is a life threatening condition which has a wide range of etiologies including several primary electrical diseases such as Brugada syndrome. The presence of fragmented QRS in lead v1 to v3 signifies a worse prognosis in Brugada syndrome. Therapy options for clinically manifested Brugada syndrome are isoproterenol, quinidine, and radio frequency ablation. Here, we present a case of Brugada with electrical storm manifestation, treated with radio frequency ablation by endocardial and epicardial approach.

**Methods:** N/A

**Results:** The patient is a 55 years old male, with a history of ICD implantation in 2016 due to syncope and type 1 Brugada syndrome. He reported multiple episodes of syncope and appropriate ICD shocks, that is refractory with medications. Beside coved ST elevation, the ECG recording also showed fragmented QRS in lead v1 to v3. Radio frequency ablation become the chosen therapy since medication option for Brugada syndrome is limited in Vietnam. The strategy chosen is substrate modification guided by substrate mapping in endocardial and epicardial area. During epicardial mapping, substrates were predominantly found in free wall RVOT and RV. VF induction successfully performed pre ablation and can't be repeated post ablation. Partial normalization of Brugada ECG pattern achieved after ablation procedure. Patient developed well and discharged 5 days after procedure. There are no events reported by patient and from ICD interrogation during follow up.

**Conclusions:** Epicardial radio frequency ablation is a promising modalities for treating electrical storm or Brugada with electrical storm. The presence of fragmented QRS signifies a worse prognosis in Brugada syndrome.

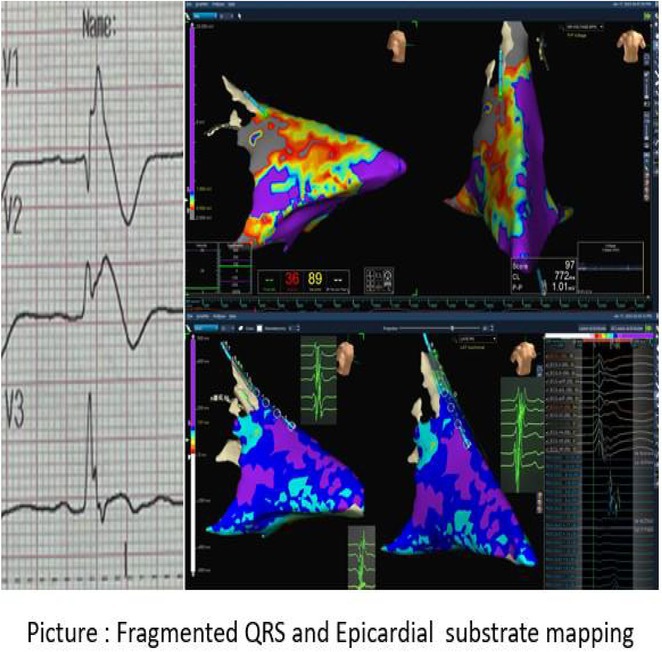

## ISOLATED RIGHT‐SIDED PERICARDIAL EFFUSION FOLLOWING HYBRID ABLATION FOR LONG‐STANDING ATRIAL FIBRILLATION

### YAHANG TAN, ZHE WANG, XINGPENG LIU, **YIRAO TAO**

#### Beijing Chaoyang Hospital,Capital Medical University, Beijing, China

**Introduction: Isolated right‐sided pericardial effusion following hybrid ablation for long‐standing atrial fibrillation**

**Methods:** N/A

**Results:** A 53‐year‐old man presented to our hospital with progressive dyspnea and a 4‐day history of nausea and vomiting. Thirty‐one days prior to his admission, the patient had undergone unilateral left‐sided hybrid ablation for long‐standing, persistent atrial fibrillation. The hybrid ablation procedure combines minimally invasive thoracoscopic epicardial ablation with transvenous endocardial electrophysiologic validation and touch‐up of incomplete epicardial lesions as needed. We initially diagnosed the patient with acute pulmonary embolism based on the following evidence: (1) the electrocardiogram showed a SIQIII‐type pattern and T wave inversion in leads V1‐V3 (Figure 1); (2) D‐dimer levels were significantly elevated; (3) arterial blood gas analysis demonstrated hypoxemia; and (4) the physical examination revealed mild bilateral lower limb edema and jugular vein distention. However, this diagnosis was challenged after we obtained a computed tomography pulmonary angiogram and an echocardiogram (Figure 2). Findings from both examinations suggested that the right ventricle was severely compressed by the pericardial effusion. We reached a final diagnosis of cardiac tamponade. Surprisingly, the pericardial effusion was limited to the right side of the heart. The patient underwent immediate pericardiocentesis guided by X‐ray fluoroscopy. In the left anterior oblique view of the heart silhouette, the right side was stationary, while the left side exhibited normal movement (Video 1). The symptoms of dyspnea and gastrointestinal discomfort were relieved after we drained 590 ml of dark red fluid. We speculate that the mechanisms underlying the regional pericardial effusion included: (1) post‐pericardiotomy syndrome; and (2) healing of the left pericardial incision before the buildup of the effusion.

**Conclusions:** N/A

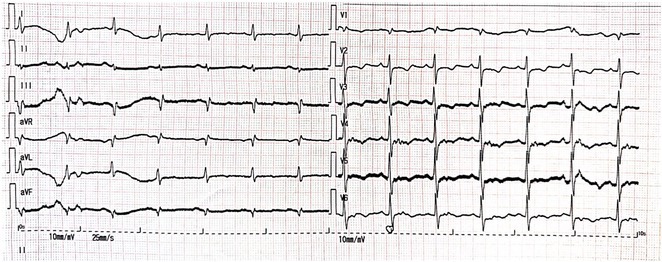

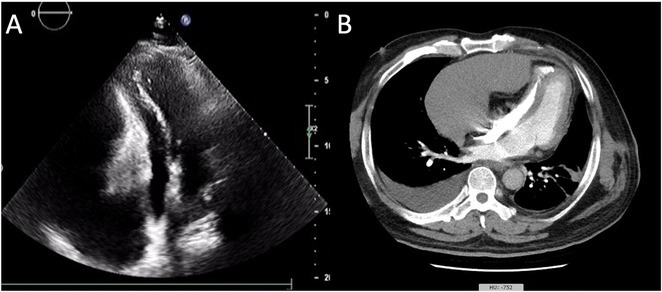

## IMPACT OF CONTACT ANGLE AND ACUTE MYOCARDIAL INJURY IN CATHETER ABLATION BY DUAL‐DIAMETER POLARX FIT CRYOBALLOON FOR PATIENTS WITH PAROXYSMAL ATRIAL FIBRILLATION

### HIROSHI TASAKA

#### Kurashiki Central Hospital, Kurashiki, Japan

**Introduction:** In pulmonary vein isolation (PVI) for paroxysmal atrial fibrillation (PAF), cryoballoon increases various biomarkers of myocardial injury. The POLARx FIT is a novel dual‐diameter cryoballoon which can be expanded to 31mm with one button. A 31mm POLARx FIT cryoballoon (Fit‐CB) can occlude the antrum of pulmonary veins (PVs) and is expected to isolate a wider area of PVs than the conventional POLARx cryoballoon (X‐CB).

**Methods:** A total of 103 PAF patients (mean age 68±11 years; men, 58%) undergoing PVI using Fit‐CB (37 patients) or X‐CB (66 patients) between August 2022 and December 2023 were investigated. In the Fit‐CB group, 31mm cryoballoon was used at least once. The balloon contact angle was measured as the sum of the angles of the upper and lower side from the equator line in each PV under fluoroscopic imaging (Figure). The procedural characteristics and the increase in creatine kinase‐MB (CK‐MB), one of biomarkers of myocardial injury were compared between the Fit‐CB and X‐CB groups.

**Results:** There was no significant difference in procedure time between the Fit‐CB and X‐CB groups (84±25 vs. 91±33 min, p 0.25), but fluoroscopy time tended to be shorter in the Fit‐CB group (22±11 vs. 26±13 min, p 0.07). In the Fit‐CB group, 31mm balloon was used for 95% of the superior PVs and 27% of the inferior PVs. The balloon contact angle in left superior PV (LSPV) and right superior PV (RSPV) were significantly larger in the Fit‐CB group than in the X‐CB group (LSPV: 95±10 vs. 85±13 degrees, p<0.01 and RSPV: 99±10 vs. 90±14 degrees, p<0.01), respectively. There were no significant differences in cryoablation application number, nadir temperature and time to isolation between the two groups. CK‐MB on the day after the procedure was significantly higher in the Fit‐CB group than in the X‐CB group (37±17 vs. 31±12 IU/L, p 0.035).

**Conclusions:** POLARx FIT cryoballoon enabled to expand contact surface area and cause greater thermal injury than POLARx cryoballoon during PVI in PAF patients.

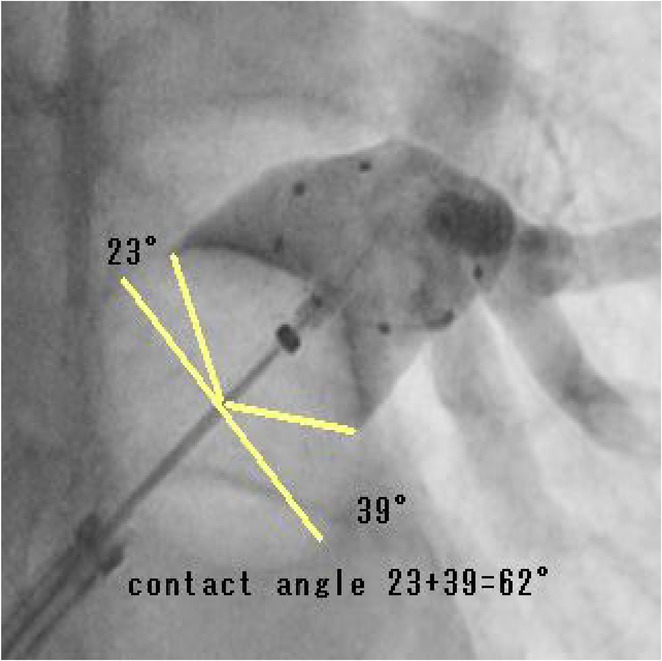

## PULSE FIELD ABLATION FOR ATRIAL FIBRILLATION WITH ELECTROANATOMICAL MAPPING ‐ ONE YEAR OUTCOMES

### **WEE SIONG TEO**, KELVIN CHEOK KENG WONG, PAUL CHUN YIH LIM

#### Mount Elizabeth Hospital Singapore, Singapore, Singapore

**Introduction:** Pulse field ablation (PFA) is a cardiomyocyte specific nonthermal energy delivery for atrial fibrillation ablation shown to be efficacious and safe. We report our initial Asian experience with pulse field ablation results at 1 year of follow up and discuss the merits of combining PFA with electroanatomic mapping (EAM).

**Methods:** 29 patients in our pilot study cohort undergoing PFA utilising the Farapulse (Boston Scientific, MA, USA) system with EAM have underwent at least 12 months follow up. Endpoints were acute ablation success, safety and freedom from atrial fibrillation 1 year post ablation.

**Results:** PFA was highly effective in achieving pulmonary vein isolation (PVI) for 28 of 29 patients (97%) with one patient requiring radiofrequency ablation due to unfamiliarity with sheath and catheter manipulation in our early experience. The mean procedural time for our cohort was 126.2 ± 31.4 mins. PFA time to PVI was 36.9 ± 18.1 minutes. Posterior wall isolation was 100% successful with a short ablation time of 7.7 ± 3.7 minutes. Only one patient suffered an air embolism during catheter exchange. EAM wise, pre‐PFA and post PFA mapping took 12.3 ± 5.7mins and 7.6 ± 3.9 mins respectively. EAM allows better visualisation of catheter positions to facilitate wide antral PVI. It aids to avoid creation of proarrhythmic narrow interlesional distances on the posterior wall and is essential for posterior wall isolation and mitral isthmus ablation. Limitations are increased cost and marginal increase in procedural time. The mean follow‐up duration was 15.2 ± 2.9 mths. 4 out of 29 patients had recurrence of atrial fibrillation beyond 3 months of blanking period translating to an 86.2% freedom of atrial arrhythmias.

**Conclusions:** Our initial Asian experience showed high rates of successful PVI with the Farawave PFA system. Intermediate term data appears promising with 86.2% freedom from atrial atrial arrhythmias. EAM appears to accord additional benefits.

## PREDICTORS OF DEVICE IMPLANTATION AND MORTALITY IN IMPLANTABLE LOOP RECORDER RECIPIENTS

### **NICHOLAY TEODOROVICH**, RAFAEL DIAMANTE, MUSTAFA JABER, JACOB GEORGE, MOSHE SWISSA

#### Kaplan Medical Center, Rehovot, Israel

**Introduction:** Implantable loop recorder (ILR) is frequently used for syncope evaluation. The purpose of this study is to identify predictors of cardiac implantable electronic device (CIED) implantation and mortality ILR recipients.

**Methods:** This was a retrospective study of 141 ILR recipients. We collected demographic, clinical and electrophysiolgic data of these patients.We analyzed the correlation between different baseline parameters and the CEID implantation as well as mortality in these patients.

**Results:** A total of 141 patients were included in the study, 53.9% were female, 96.5% underwent ILR implantation. Thirty seven (26.2%) patients underwent CEID implantation (34 (24.1%) pacemaker and 3 (2.1%) ICD). No demographic or clinical parameter predicted CIED implantation. The only ECG predictors were the PR interval and presence of RBBB. When pacemaker implantation only was assessed, the PR interval (203.3 vs 181.1 ms, p=0.033) and presence of RBBB (43.8% vs 19.6%, p=0.031) were also predictive of pacemaker implantation, as were older age (75.7 vs 70.2 years, p=0.017) and presence of pauses on ambulatory ECG monitoring (57.1% vs 23.2%, p=0.045).Ten (7.1%) patient died during the follow‐up. Predictors of mortality were heart failure, prolonged PR interval and complex ventricular ectopy on Holter monitoring, low LVEF, and previous pacemaker implantation.Cox regression analysis demonstrated that prolonged PR interval (HR 9.6, CI 1.7‐52.6, p=0.01) presence of LBBB ( HR 15.9, CI 3.4‐76.9, p<0.001) and status post pacemaker implantation (HR 5.1, CI 3.4‐20.0, p=0.02) were independent predictors of mortality.

**Conclusions:** Different conduction abnormalities have different prognostic significance in ILR recipients. While RBBB is predictive of pacemaker implantation, LBBB is predictive of mortality and prolonged PR interval is predictive of both. This can be used to better assess need for timely intervention in Patients considered for ILR implantation.

## ASSOCIATION BETWEEN PACED QRS WIDTH AND INCIDENT ATRIAL FIBRILLATION IN HEART BLOCK PATIENTS WITH PERMANENT PACEMAKER

### **THANAWAT THAMMACHART**, TREECHADA WISARATAPONG, WATCHARA LOHAWIJAN

#### Prince of Songkhla University, Hatyai, Thailand

**Introduction:** The paced QRS duration and burden of right ventricular pacing are associated with an increased risk of paced‐induced cardiomyopathy and HF hospitalization.The association between paced QRS width and the risk of developing atrial fibrillation(AF) is uncertain.

**Methods:** We collected retrospective data from 491 consecutive patients who underwent pacemaker implantation for an atrioventricular block in Songklanagarind hospital between January 2013 and December 2022.All patients were followed up at the device clinic for at least six months and had at least 90% ventricular pacing.Patients with a history of AF,implantable cardioverter‐defibrillator,cardiac resynchronization therapy implantation, and cardiac surgery within three months were excluded.Two independent cardiologists measured the paced QRS width and left ventricular activation time(LVAT).The primary outcome was time to first clinical AF or first longest atrial high rate events(AHREs) of more than 6 minutes.The secondary outcome was all‐cause mortality.

**Results:** A total of 491 patients were included. The mean age was 71.8±13.9 years, and 46.6% were male.The most common underlying disease was hypertension.The mean CHA_2_DS_2_‐Vasc score was 3±1.5.The ventricular lead location was at septum 86.4%, left bundle branch area 11.4%, and apex 2.2%,respectively.Incidence of AF/ AHREs were 123 (25.1%) patients with a mean follow‐up of 53.1±33.2 months.The median paced QRS width and LVAT were 160.0 (150.0, 170.0) and 108.5 (87.0, 151.3) milliseconds, respectively. The paced QRS width of more than 175 milliseconds was associated with the occurrence of AF/AHREs, adjusted with potential confounding factors (HR 1.670, 95% CI 1.087‐2.563, P=0.019).LVAT was not significantly associated with the occurrence of AF/AHREs or death.

**Conclusions:** The incidence of AF/AHREs in AV block patients is substantial.A paced QRS width of more than 175 ms significantly predicted the occurrence of AF/AHREs.LVAT was not associated with the risk of AF/AHREs. Additionally, neither paced QRS width nor LVAT was associated with all‐cause mortality.

## MD

### **ALVIN THENGKER**, BENNY SETIADI, FILIPUS MICHAEL YOFRIDO

#### Sam Ratulangi University, Manado, Indonesia

**Introduction:** Atrioventricular nodal reentrant tachycardia (AVNRT) ablation has a high success rate. Fully understanding the substrate for AVNRT remains a challenge. Various inputs to the AV node occur in less than 5% of cases. Persistent VA dissociation during RV pacing is uncommon. Right sided inferior nodal extensions could be the critical slow pathway in some cases and may require targeting for successful treatment.

**Methods:** N/A

**Results:** A 66 years old female referred with diagnosis of Supra Ventricular Tachycardia (SVT) for EP study (EPS) and Ablation. EPS has been done with 2 quadripolar catheter to His and RV apex, 1 duo‐decapolar catheter to CS and 1 ablation catheter. SVT consistently induced with extra stimuli. During SVT VA Interval 98 ms with earliest A in CS 1‐2 (located in mid CS). RV pacing during SVT consistently showed AV dissociation and earliest A switched between CS 1‐2 and 9‐10 without SVT termination. AVRT was excluded due to AV dissociation during RV pacing. SVT consistently induced by extra‐stimuli, sometimes followed with AV jump, which indicate AVNRT. Due to earliest A in mid septal which transitioned to CS 9‐10 without terminating SVT, we hypothesized that the mechanism was atypical slow‐slow AVNRT with Left Inferior Extension which sometimes switched to slow‐fast AVNRT, especially during RV pacing which penetrate the slow pathway. Slow Pathway at Right Inferior Extension were ablated using 35 W power for 60 seconds. After ablation, no SVT was induced with burst and extra‐stimuli pacing. Differentiating AVNRT from other SVT can be complex. Specific pacing maneuvers often needed during EPS. The presence of VA block rules the diagnosis of AVRT. The presence of AV jump indicates a dual nodal pathway. Uniquely, gradually decreased VA interval and switching of the earliest A were observed during SVT, which could differentiate AT and shifting pathway from slow‐slow to slow‐fast AVNRT. Successful slow pathway ablation at RIE area can confirm atypical AVNRT with shifting pathways.

**Conclusions:** Pacing maneuvers in conventional EPS of SVT can enhance the likelihood of identifying the pathway that should be ablated.

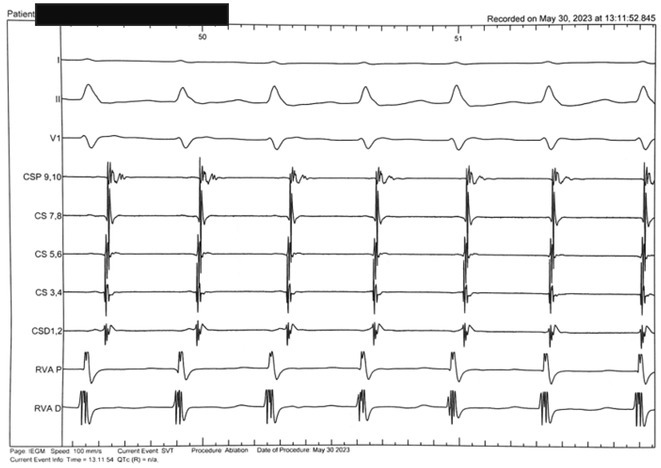

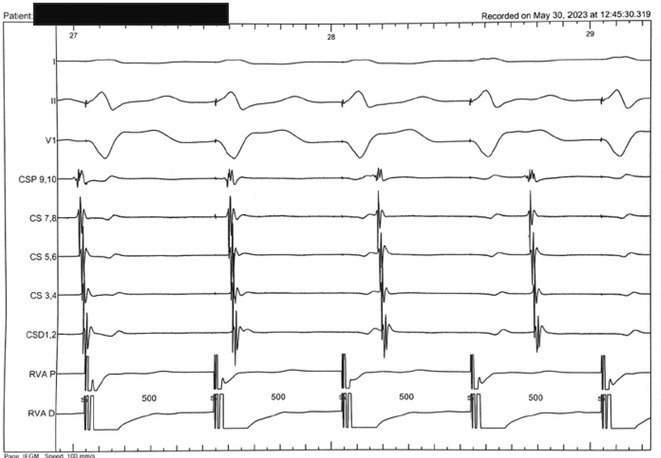

## SUCCESSFUL ABLATION OF RIGHT POSTERIOSEPTAL ACCESSORY PATHWAY USING THE CORONARY SINUS APPROACH

### **NGUYEN THI LE THUY**, PHAM TRAN LINH, VIEN HOANG LONG, NGUYEN DUY TUAN, VU HUY THANH, NGO THUY NGOC

#### Bach Mai Hospital, Ha Noi City, Viet Nam

**Introduction:** We present a case of successful radiofrequency ablation of the right posteroseptal accessory pathway (AP) using the coronary sinus approach. For epicardially located APs, the ablation success rate is relatively low. Approach through the coronary sinus using the 3D system enabled detailed mapping of the anatomical tricuspid annulus and coronary sinus, which then presents more precise ablation target for the AP.

**Methods:** N/A

**Results:** 39‐year‐old male patient has a history of multiple episodes of palpitation. 12‐lead electrocardiogram showed type B Wolff‐ Parkinson‐ White pattern with deep S wave in lead V1, negative delta wave in lead V1, II, III, aVF and positive delta wave in lead I and aVL. The patient underwent 3 unsuccessful ablations in other hospitals. We perfomed electrophysiology study with a decapolar catheter inserted into the coronary sinus and a quadripolar catheter into the right ventricular apex. The 3D map is constructed using a irrigated tip catheter. Programmed atrial stimulation induced an AVRT (cycle length 353ms) with earliest atrial signal at CS 7‐8. The ablation catheter was inserted in the coronary sinus, recording a large AP potential with earliest ventricular signal at CS 7‐8. Coronary sinus imaging showed that the target location was at the middle cardiac vein's ostium. The first ablation session of 20 watts terminated the tachycardia within 1.5 seconds. Then, programmed stimulation couldn’t induce any tachycardia. Several consolidation lesions were applied with power titrated up to 30 watts. No recurrence of preexcitation was observed in the following 30 minutes; even with the administration of adenosine. Ventricular stimulation showed retrograde conduction through the atrioventricular node. The patient was stable and discharged the next day. One‐month follow‐up showed normal electrocardiogram and no more sign of palpitation.

**Conclusions:** Ablation of epicardial accessory pathway using the coronary sinus approach is an effective and safe method, especially where endocardial ablation has failed.

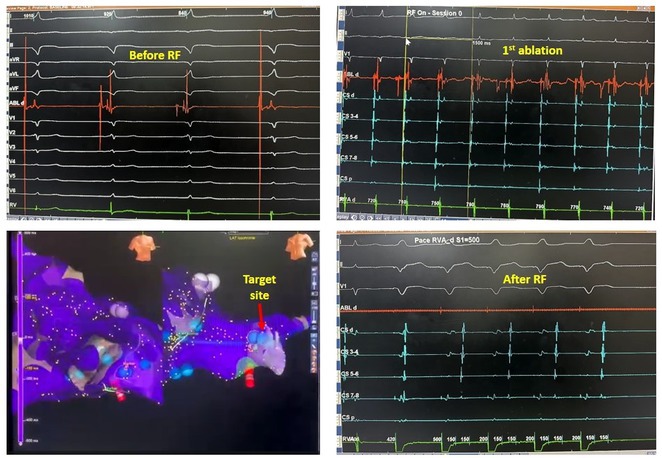

## BIDIRECTIONAL ASSOCIATION AMONG THE TYPE OF ATRIAL FIBRILLATION, SLEEP‐DISORDERED BREATHING SEVERITY, HEART FAILURE PROGRESSION, AND LEFT ATRIAL ENLARGEMENT, IN PATIENTS WITH ATRIAL FIBRILLATION

### SAKAI TOGO

#### Steel Memorial Yawata Hospital, Fukuoka, Japan

**Introduction:** This study aimed to clarify; 1) the association among the atrial fibrillation (AF) type, sleep‐disordered breathing (SDB), heart failure (HF), and left atrial (LA) enlargement and 2) the independent predictors of LA enlargement in patients with AF. The study's primary endpoint was LA enlargement (LA volume index [LAVI] ≥78 ml/m^2^).

**Methods:** Overall, 423 patients with non‐valvular AF were enrolled. We prospectively evaluated the role of the clinical parameters including the CHADS_2_ score, AF type, SDB severity, and HF in LA enlargement. Among them, 266 patients exhibiting a 3% oxygen desaturation index (ODI) of ≥10 events/hour underwent polysomnography to evaluate the SDB severity measured by the apnoea‐hypopnoea index (AHI). The LA enlargement and HF were characterized by the LA diameter/LAVI, an increase in the B‐type natriuretic peptide level, and a lower left ventricular ejection fraction.

**Results:** This study showed that non‐paroxysmal AF (NPAF) rather than paroxysmal AF (PAF), the SDB severity, LA enlargement, and HF progression had bidirectional associations and exacerbated each other, which generated a vicious cycle that contributed to the LA enlargement. NPAF (OR=4.56, p<0.001), an AHI of ≥25.00 events/hour (OR=1.66, p=0.004), a 3%ODI of ≥15.50 events/hour (OR=1.62, p=0.004), and a CHADS_2_ score of ≥4.00 points (OR=1.55, p=0.021) were proven to be independent predictors of an acceleration of the LA enlargement. AF ablation improved the HF and LA enlargement.

**Conclusions:** The NPAF in comparison to PAF, SDB severity, LA enlargement, and HF had a bidirectional association and exacerbated each other, which generated a vicious cycle that contributed to the LA enlargement. Because AF ablation could improve the HF and LA enlargement, it may overcome this vicious cycle and be the basis for suppressing the LA enlargement and HF progression subsequently eliminating the substrates for AF and SDB in patients with AF.

## ELECTROCARDIOGRAPHIC CHARACTERISTICS OF SWALLOWING‐INDUCED ATRIAL TACHYCARDIA

### **YUKO TOIDA**, YU‐KI IWASAKI, HIROAKI HIRAYAMA, MAKOTO KOBAYASHI, SHUHEI OKAJIMA, NOBUAKI ITO, TOSHIKI ARAI, SERINA KOBAYASHI, MASATO HACHISUKA, YUHI FUJIMOTO, KANAKO HAGIWARA, HIROSHIGE MURATA, YOSHIYASU AIZAWA, KENJI YODOGAWA, WATARU SHIMIZU, KUNIYA ASAI

#### Nippon Medical School, Bunkyo‐ku, Tokyo, Japan

**Introduction:** Swallowing‐induced atrial tachycardia (SIAT) is a relatively rare arrhythmia, and its mechanism remains unclear.

**Methods:** From August 2012 to December 2023, 4 consecutive patients (4 males, 54 ± 9.7 years) who underwent catheter ablation (CA) for SIAT examined the ECG characteristics.

**Results:** The P wave morphology of premature atrial contractions (PACs) initiating AT was positive in lead I in two cases, positive in lead II and V1 in all four cases. The 24‐hour Holter ECG prior to CA showed PACs constituting 6.4 ± 6.9% of total heartbeats (113,173 ± 16,724 beats/day), with a circadian variation of increased PACs post‐meals and a decreased at night. The coupling interval of PACs to the preceding sinus rhythm ranged from 50% to 100% of the sinus cycle length. Before CA, antiarrhythmic drugs were used in all cases, but none was effective for prevention of SIAT. In one patient, SIAT was completely suppressed with intravenous atropine. During CA, PAC provocation by swallowing was possible in only one case, and PACs originating from the left superior pulmonary vein were observed. The suggested origins of SIAT were the right superior pulmonary vein in two cases, the left pulmonary vein in one case, and unknown in one case. Pulmonary vein isolation (PVI) was performed in all cases, and superior vena cava isolation was performed in one case. Ectopic beats with exit block after PVI were observed in all cases: two in the right superior pulmonary vein, three in the left superior pulmonary vein, and one in the left inferior pulmonary vein. No recurrence of SIAT was observed in all four cases during follow‐up period (mean 12 ± 7.8 months). In the 24‐hour Holter ECG after CA, the heart rate variability analysis showed the suppression of the power spectrum density of high frequency component in all day, and the total heartbeats increased in all cases (mean 9.76 ± 6.08%).

**Conclusions:** PVI was effective for prevention of SIAT. Although the mechanism of SIAT remains controversial, this study suggested that cardiac parasympathetic nervous activity might be associated with the occurrence of SIAT.

## USE OF PERCUTANEOUS TEMPORARY MECHANICAL SUPPORT FOR VENTRICULAR TACHYCARDIA ABLATION

### **ADRIANA TOKICH** ^1,2,3^, LUKAH Q. TUAN^1,3^, NASTAHA JONES‐LEWIS^1,3^, LORI BELL^1,3^, JENISH SHROFF^1^, ANUGRAH NAIR^1,3^, RAJEEV K. PATHAK^1,3^

#### ^1^Canberra Heart Rhythm, Canberra, Australia,^2^Canberra Heart Rhythm Foundation, Canberra, Australia,^3^Australian National University, Canberra, Australia

**Introduction:** Catheter ablation is recommended for patients with recurrent ventricular tachycardia (VT) despite antiarrhythmic therapy, in the setting of prior myocardial infarction. Risk factors for haemodynamic compromise during VT ablation include age > 60 years and severe cardiomyopathy. Mechanical circulatory support can mitigate the risk of haemodynamic collapse and facilitate successful VT ablation.

**Methods:** A 61‐year‐old male with severe ischaemic cardiomyopathy (ejection fraction 15%), underwent catheter ablation following recurrent episodes of VT. Despite amiodarone and mexiletine, there were ongoing episodes of VT (LV origin, cycle length, CL, 460ms) requiring device therapies (88 episodes of anti‐tachycardia pacing and 14 shocks). His PAINESD score was 18, indicating a 22% chance of haemodynamic decompensation during VT ablation. A percutaneous left ventricular assist device (pLVAD) Impella (Abiomed) was placed to facilitate activation mapping. Three morphologies of VT were induced (CL 390‐470ms).

**Results:** During VT, additional inotropes were required but activation mapping was unhindered as no sustained hypotension was observed. Catheter manipulation during VT was difficult owing to the presence of the pLVAD. VT‐1 (CL 470ms ‐ clinical VT based on morphology and EGM) was stable and activation map and entrainment mapping was successfully performed with identification of entrance and isthmus area. Ablation terminated the VT‐1. VT‐2‐4 was unstable and therefore a substrate‐based ablation was subsequently performed targeting late potentials at the LV apex. At 12 months, there have been no sustained episodes of VT.

**Conclusions:** pLVAD was useful in maintaining haemodynamic stability during VT activation mapping and ablation. The pLVAD limited our ability to map around the left ventricular outflow tract and may have contributed to non‐clinical arrhythmias during the procedure.

## CATHETER ABLATION OF PAROXYSMAL ATRIAL FIBRILLATION IN ELDERLY PATIENTS USING RADIOFREQUENCY CATHETER, CRYOBALLOON, HOTBALLOON, AND LASERBALLOON

### **MICHIFUMI TOKUDA**, SEIGO YAMASHITA, UI TAKATO, YOSHITO YAMAZAKI, SATOKO SHIOMI, RYUTARO SAKURAI, TAKUYA MATSUMOTO, HIDENORI SATO, HIROTSUNA OSETO, KENICHI TOKUTAKE, MICHIHIRO YOSHIMURA, TEIICHI YAMANE

#### The Jikei University School of Medicine, Tokyo, Japan

**Introduction:** Recently, the radiofrequency catheter (RFC), the cryoballoon (CB), the hotballoon (HB), and the laserballoon (LB) have been employed to isolate the pulmonary veins (PVs) in patients with paroxysmal atrial fibrillation (Paf). However, the efficacy and safety of these devices in elderly patients have not been compared. This study aims to compare the efficacy and safety of catheter ablation in elderly (>70 yrs) Paf patients using RFC, CB, HB, or LB.

**Methods:** Of 1693 consecutive patients who underwent initial PV isolation using RFC, CB, HB, or LB in two university hospitals, 313 patients (RFC 107, CB 153, HB 18, and LB 35) were aged 70 or older (elderly).

**Results:** Baseline characteristics were similar among the groups. Total procedure time was shorter in the CB group than in the other three groups. Major complications similarly occurred among the groups (P=0.66). Compared to non‐elderly patients, major complications more frequently occurred in elderly patients in the RF and HB groups. The AF‐free rate after the ablation was similar among the four groups (Figure A). In the CB group, the AF‐free rate was higher in the elderly group than in the non‐elderly group (P=0.04, Figure B).

**Conclusions:** The safety and efficacy of each ablation device were similar for elderly patients with Paf. However, compared to the non‐elderly group, AF recurrence after CB ablation was less frequent in the elderly group.

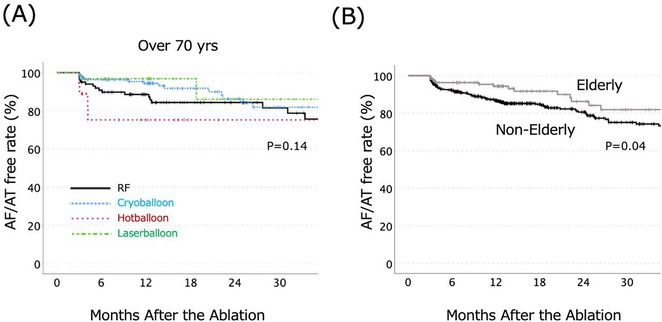

## SUCCESSFUL EXTRACTION OF AVEIR™ VR LEADLESS PACEMAKER FROM THE PULMONARY ARTERY USING TWO DIFFERENT RETRIEVAL DEVICES

### **YUSUKE TOKUDA**, KAZUYA SUGITATSU, HIROYUKI GIBO, RYO TSURUTA

#### Hakodate Municipal Hospital, Hakodate, Japan

**Introduction:** Leadless pacemakers (LLPMs) are now widly used as a safe and effective alternative to transvenous lead‐based devices. The Aveir™ VR (Abott, Abott Park, IL) is the LLPM that has a unique mapping capability and utilizes a helical flixation mechanism for implantation and chronic retrieval. While most dislodged LLPMs remain within the right ventricle (RV), there are several reported cases of dislodged LLPMs that have embolized to the pulmonary artery (PA), requiring complex retrievals. Special considerations during the device retrieval include avoiding injury to the pulmonary and tricuspid valves, adjacent vasculature, and cardiac chambers.

**Methods:** N/A

**Results:** A 93‐year‐old woman with sick sinus syndrome was treated with an Aveir™ implantation. The device was successfully implanted in the RV apical septum on the first attempt, with a capture threshold of 0.75 V at 0.4 ms, R‐wave amplitude of 5.0 mV, and pacing impedance of 350 ohms. However, two months after the implantation, complete pacing failure was observed. The chest X‐ray showed a pacemaker embolization in the right PA. Under local anesthesia, we tried to remove the device via femoral vein approach. Using fluoroscopy, the Indy OTW™ (COOK Medial Japan, Tokyo) snare was advanced over the LLPM and we caught the head of LLPM and drew it carefully to the right atrium (RA). In the RA, the LLPM was docked to the extraction sheath with re‐grasping its bottom by Aveir™ retrieval sheath (Figure).

**Conclusions:** This is the first case in whom successful removal of an Aveir™ from the PA using two different retrieval devices.

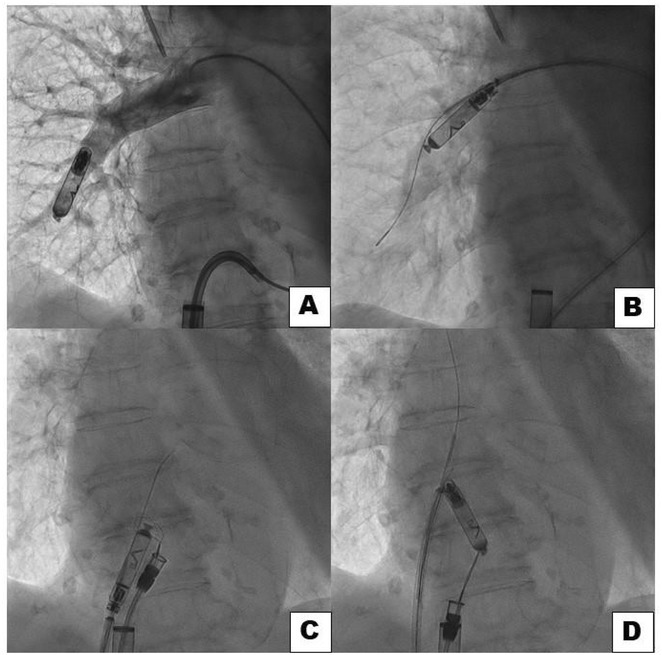

## IMPACT OF COMPOUND MOTOR ACTION POTENTIALS REDUCTION PATTERN ON LONG‐TERM PHRENIC NERVE INJURY AFTER CRYOBALLOON ABLATION FOR ATRIAL FIBRILLATION

### **KENICHI TOKUTAKE**, UI TAKATO, RYUTARO SAKURAI, YOSHITO YAMAZAKI, SATOKO SHIOMI, TAKUYA MATSUMOTO, HIDENORI SATO, HIROTSUNA OSETO, SEIGO YAMASHITA, MICHIFUMI TOKUDA, SATORU MIYANAGA, MICHIHIRO YOSHIMURA, TEIICHI YAMANE

#### The Jikei University School of Medicine, Tokyo, Japan

**Introduction:** Phrenic nerve injury (PNI) is one of the complications after cryoballoon ablation for atrial fibrillation (AF). While PNI often improves, it persists in some patients. The purpose of this study was to evaluate the relationship between the pattern of diaphragmatic compound motor action potential (CMAP) reduction during catheter ablation and long‐term phrenic nerve injury.

**Methods:** Cryoballoon ablation was performed on 870 consecutive patients with paroxysmal AF. During right pulmonary vein isolation, the phrenic nerve was paced and CMAP was monitored. PNI was defined as CMAP amplitude decrease more than 30 % from baseline or weakening movement of phrenic membrane (palpation or on X‐ray). If PNI was observed, the freeze was immediately aborted using a double‐stop technique.

**Results:** PNI occurred in 46 (5.3 %) patients. Among patients with, 9 patients (19.6%) persisted PNI the following day. Patients with persistent PNI (at least for one day or more) showed significantly earlier onset of CMAP reduction during catheter ablation compared with the patients with PNI improvement by the following day (80±30 days vs 124±27 seconds, P<0.01). In 9 patients with persistent PNI, seven patients experienced improvements (average duration was 99 days), while two patients had persistent PNI.

**Conclusions:** PNI occurred in 5.3 % of patients after Cryoballoon ablation for AF. In patients with persistent PNI, significantly earlier onset of CMAP reduction was observed. Cases with early CMAP reduction require more immediate stop to prevent PNI.

## THE IMPACT OF RESIDUAL UNIPOLAR VOLTAGE LESIONS IN PATIENTS WITH PAROXYSMAL ATRIAL FIBRILLATION AFTER CRYO‐BALLOON ABLATION

### **TAKUYA TOMOMORI** ^1^, YASUHITO KOTAKE^1^, SHUNSUKE KAWATANI^1^, AIKO TAKAMI^2^, MASARU KATO^1^, KAZUHIRO YAMAMOTO^1^

#### ^1^Tottori University Hospital, Yonago city, Japan,^2^Tottori Prefectural Central Hospital, Tottori city, Japan

**Introduction:** Pulmonary vein isolation (PVI) using cryo‐balloon ablation is the cornerstone strategy for paroxysmal atrial fibrillation (AF). As for PVI, creating a transmural block line of pulmonary vein (PV) from left atria is critically important, as an incomplete block line can pose a risk of AF recurrence. Based on the previous studies, endocardial unipolar voltage mapping had a higher sensitivity than bipolar voltage mapping for identifying intramural viable myocardium in the atria. The purpose of this study was to investigate the clinical significance of the residual unipolar voltage lesion following PVI in comparison with AF recurrence and other clinical parameters.

**Methods:** Data from patients presenting for AF ablation from April 2023 to January 2024 at Tottori University Hospital were reviewed. To assess the electrical isolation of PV, high‐resolution voltage mapping was performed after PVI procedure. Unipolar and Bipolar voltage maps were compared for cryo‐balloon ablation lesions.

**Results:** Fifty‐seven consecutive patients who underwent cryo‐balloon ablation for paroxysmal AF were included in this study (mean age 64.0, 21% female). Of these, residual unipolar voltage lesions after PVI were identified in 22 patients. During follow‐up periods, patients with residual unipolar voltage after PVI show higher early recurrence (P=0.01) and short‐term AF recurrence (P=0.05).

**Conclusions:** Patients with residual unipolar voltage following PVI tended to show a higher recurrence rate of AF. Residual unipolar voltage might be the predictor of AF recurrence, reflecting the non‐transmural lesion for PVI.

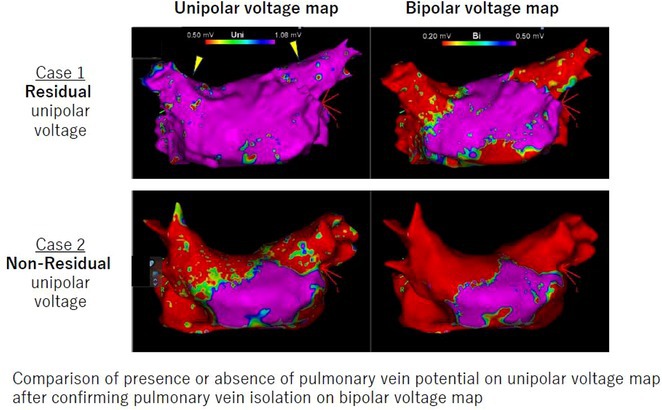

## POWER‐LAW DISTRIBUTION OF SPATIOTEMPORAL INTERMITTENCY IN ATRIAL FIBRILLATION

### **IVAYLO R. TONCHEV** ^1^, PAWEL KUKLIK^2^, DHANI DHARMAPRANI^3^, KATHRYN TIVER^1^, CAMPBELL STRONG^4^, SOBHAN S. SHAHRBABAKI^4^, DARIUS CHAPMAN^4^, LUKE O’LOUGHLIN^5^, LEWIS MITCHELL^5^, MATTHEW TUNG^6^, NIK STOYANOV^7^, ANAND GANESAN^1^

#### ^1^Department of Cardiovascular Medicine, Flinders Medical Centre, College of Medicine and Public Health, Flinders University, Adelaide, Australia,^2^Department of Cardiology, Asklepios Hospital St. Georg, Hamburg, Germany, Faculty of Medicine, Semmelweis University Campus Hamburg, Hamburg, Germany,^3^Australian Institute for Machine Learning, University of Adelaide; College of Medicine and Public Health, Flinders University, Adelaide, Australia,^4^College of Medicine and Public Health, Flinders University, Adelaide, Australia,^5^School of Mathematical Sciences, University of Adelaide, Adelaide, Australia,^6^Department of Cardiovascular Medicine, Sunshine Coast University Hospital, Sunshine coast, Birtinya, Australia,^7^Department of Cardiology, Fiona Stanley Hospital, Perth, Australia

**Introduction:** Spatiotemporal intermittency, characterized by alternating periods of laminarity (L) and turbulence (T), is commonly observed in clinical AF electrogram recordings. This study evaluates whether the temporal distribution of laminar and turbulent domains differs between paroxysmal (PAF) and persistent AF (perAF), and tests if Na+‐channel blockade terminates AF through spatiotemporal intermittency.

**Methods:** A prospective multicentre study involving pre‐ablation HD‐grid (16‐pole) one‐minute AF recordings taken at pre‐specified atrial sites. Periods of laminarity were identified by transforming bipolar electrograms into phase and computing mean phase coherence. Correlation lengths were determined and used to distinguish laminar and turbulent periods. Electrophysiological properties of L and T domains were quantified. Data from EGMs recorded before, during, and after IV flecainide infusion were analyzed for temporal durations of laminarity periods.

**Results:** A total of 1785 laminar windows were identified in 90 patients (43 PAF and 47 perAF). Dominant frequency (5.17±1.05 Hz vs 4.87±1.01 Hz, P<0.001), Shannon entropy (2.72±0.27 vs 2.41±0.41, P<0.001) were higher, and AF cycle length was faster (207±47ms vs 219±53ms, P<0.001) in periods of turbulence vs laminarity. Laminar events were more frequently observed (22.7±1.5 vs 17.2±1.8, P<0.001) among patients with PAF. A power‐law distribution of laminar event window lengths was observed among patients with PAF compared to exponential distribution in perAF patients. In 20/20 patients studied on Flecainide infusion, visible cycling oscillations between periods of laminarity and disorder were observed.

**Conclusions:** These observations suggest that criticality, identifiable through periods of intermittent laminarity, may be an important contributory mechanism in AF persistence or termination. Power‐law distribution is consistent with the concept that PAF could be a self‐organising critical state. The emergence of power‐law distributed temporal domains of laminarity in response to IV flecainide suggests a potential role for critical phase transitions.

## T‐WAVE ALTERNANS AS AN INDICATOR OF EARLY CARDIOTOXICITY IN BREAST CANCER PATIENTS ON ANTHRACYCLINE‐BASED CHEMOTHERAPY

### **ALEXANDER EDO TONDAS** ^1^, IRFANNUDDIN IRFANNUDDIN^2^, MUHAMMAD YAMIN^3^, TAUFIK INDRAJAYA^2^, ILARIA MARCANTONI^4^, LAURA BURATTINI^4^

#### ^1^Mohammad Hoesin General Hospital, Palembang, Indonesia,^2^Faculty of Medicine Universitas Sriwijaya, Palembang, Indonesia,^3^Faculty of Medicine Universitas Indonesia, Jakarta, Indonesia,^4^Università Politecnica delle Marche, Ancona, Italy

**Introduction:** Anthracycline‐class drugs, such as doxorubicin and its analogs, commonly used as the primary chemotherapy for breast cancer, have the potential to induce irreversible cardiotoxicity. Microvolt T‐wave alternans (TWA) is an established prognostic cardiac repolarization marker for cardiovascular or non‐cardiovascular diseases. However, its utility in the field of cardio‐oncology has not been explored.

**Methods:** This is an observational cohort study on 32 breast cancer patients completing 6 cycles of TAC (taxane‐anthracycline‐cyclophosphamide) regimen. Electrocardiograms (ECGs) signals were recorded before and after each cycle using the KardiaMobile 6L device, and then analyzed using the enhanced adaptive match filter (EAMF) method to quantify TWA. As a comparison, other subclinical cardiotoxicity markers, namely the global longitudinal strain (GLS) and hsTropI, were also assessed according to applicable standards.

**Results:** There was a significant increase in TWA when measured before and after drug administration, during the first to the third cycle, in both Lead I and Lead II. Baseline TWA in lead I ≥24.67 μV, with an AUC of 73.7% (p = 0.03), and in lead II ≥19.69 μV with an AUC of 77.2% (p = 0.012), could predict subsequent GLS decrement on the third cycle. Baseline TWA in lead I ≥36.185 μV with an AUC of 79% (p = 0.046) and in lead II ≥30.065 μV with an AUC of 77.3% (p = 0.004) could predict subsequent hsTropI elevation on the third cycle.

**Conclusions:** Microvolt TWA can indicate early cardiotoxicity in breast cancer patients undergoing anthracycline‐based chemotherapy.

## RIGHT VENTRICULAR OUTFLOW TRACT VENTRICULAR TACHYCARDIA WITH ABNORMAL ORIGIN OF CORONARY ARTERY

### LE UYEN PHUONG TRAN

#### Cho Ray Hospital, Ho Chi Minh City, Viet Nam

**Introduction:** We described 2 cases of the symptomatic right outflow tract (RVOT) ventricular tachycardia (VT) with anomalous origin of the coronary artery (AOCA) from the inappropriate sinus of Valsalva (SOV).

**Methods: *Case 1***: A 54‐year‐old female patient presented the monomorphic VT‐induced syncope. Despite of amiodarone IV infusion, ambulatory ECG still revealed non‐sustained and sustained VT with a burden of 49%. LVEF was reduced to 29% with normal LV diameter. AOCA was confirmed by computed tomography angiography (CTA) showing the left main emerged from the right coronary associated with an inter‐arterial course between the pulmonary artery and aorta. The origin of VT was located at the posterior wall of the RVOT (with earliest signal 54 ms before QRS onset). ***Case 2***: A 42‐year‐old male patient suffered several fasting‐related episodes of fainting, dizziness, and presyncope caused by multiples episodes of non‐sustained monomorphic VT with LBBB morphology and inferior axis with a burden of 8%. The CTA revealed right CA originated from the left SOV with an inter‐arterial course. The map revealed VT emerged from the septal wall of the RVOT (with earliest signal 58 ms before QRS onset).

**Results:** Non irrigation catheters were chosen for an effective RF ablation (35W‐56^o^C) for both two patients and prevent the underlying coronary from injury. The two patients remained asymptomatic post ablation without any ventricular arrhythmias on repetitive ambulatory ECG within six months and two years, respectively.

**Conclusions: Discussion & Conclusions** AOCA was uncommon in patients with RVOT VT. Whether the inter‐arterial course of AOCA from the opposite SOV causing vessel compression leading to transient ischemia and subsequent RVOT VT rests in doubt. The underlying inter‐arterial course of the coronary was subjected to injury when performing RFCA at the posterior wall of RVOT and therefore non‐irrigation catheter was a reasonable choice to ablate the VT preserving adjacent AOCA.

## VENTRICULAR ARRHYTHMIA INDUCED HEART FAILURE: SHORT TERM PROGNOSIS AFTER ABLATION

### LE UYEN PHUONG TRAN

#### Cho Ray Hospital, Ho Chi Minh City, Viet Nam

**Introduction:** Ventricular arrhythmias‐induced cardiomyopathy (VAICM) has been recognized as a reversible cause of heart failure. However, the symptoms in this group of patients might not be comparable to those in patients with PVCs but without heart failure.

**Methods:** We aimed to assess the clinical course of patients with VAICM referred for radio frequency ablation in our center by comparing the clinical characteristics between VA patients with and without heart failure regarding symptoms and the reverse remodeling process by echocardiography before and after ablation. Patients with idiopathic VA were divided into two groups: one with reduced left ventricular ejection fraction (LVEF) and the other with normal LVEF. The assessment of patients' HF condition were assessed by echocardiograms and PVC burden with 24‐hour Holter ECGs before the ablation and three months after.

**Results:** A total of 236 patients were included in our study, with 30 patients in the reduced LVEF group (female: 23.33%, average age: 52.32 ± 12.11) and 206 in the normal LVEF group (Female: 62%, Average age: 42.15 ± 9.21). Patients with reduced LVEF were more likely to experience effort dyspnea, while patients with normal LVEF usually complained of palpitations. Regarding the distribution of PVCs and VT throughout the daytime, patients with heart failure had more sustained VT than those without heart failure (62% vs. 23%, p = 0.023). As for the recovery of LVEF, patients with heart failure showed significant improvement after ablation (45.23 ± 9.15 vs. 53.19 ± 5.38%, p < 0.005). In this group of patients, only 5 were on optimal medical therapy after discharge, while 21 patients received only ARNI or ARB.

**Conclusions:** VAICM is a gradual process with effort dyspnea as principle symptoms while palpitations is major complaints of those without heart failure. The prognosis of VAICM appears to be more benign after resolution of ventricular arrhythmias by ablation which thereafter help reversing the remodeling process, and improving systolic function.

## ELECTROPHYSIOLOGY DETECTS SYNCOPE DUE TO SICK SINUS SYNDROME IN A PATIENT WITH HYPERTROPHIC CARDIOMYOPATHY

### **QUOC BAO TRAN**, ANH BINH HO, LE XUAN NGO, ANH KHOA PHAN

#### Hue Central Hospital, Hue City, Viet Nam

**Introduction:** Hypertrophic cardiomyopathy (HCM) is a hereditary disease with significant phenotypic heterogeneity in terms of hypertrophy, symptom presence and severity, and disease progression. Syncope in hypertrophic cardiomyopathy has two underlying mechanisms: arrhythmias and hemodynamic mechanisms Non‐invasive tests make it difficult to determine the underlying cause of syncope in an HCM patient. An electrophysiology study should be considered for unexplained syncope in these patients.

**Methods:** N/A

**Results:** A 54‐year‐old female patient with a history of HCM was admitted to the hospital because of episodes of syncope. Even after searching for potential causes of syncope, such as drug intoxication, electrolyte imbalance, and coronary ischemia, even with the 24‐hour Holter monitor, the patient's syncope remained unexplained. Therefore, an electrophysiology study was indicated, which showed a sinus node recovery time of 3462 ms and no supraventricular or ventricular tachycardia. A dual‐chamber pacemaker was implanted, and the patient did not suffer any episodes of syncope after that.

**Conclusions:** Electrophysiology study should be considered for patients with unexplained syncope in hypertrophic cardiomyopathy.

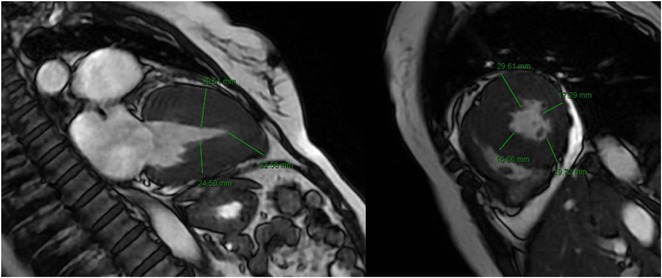

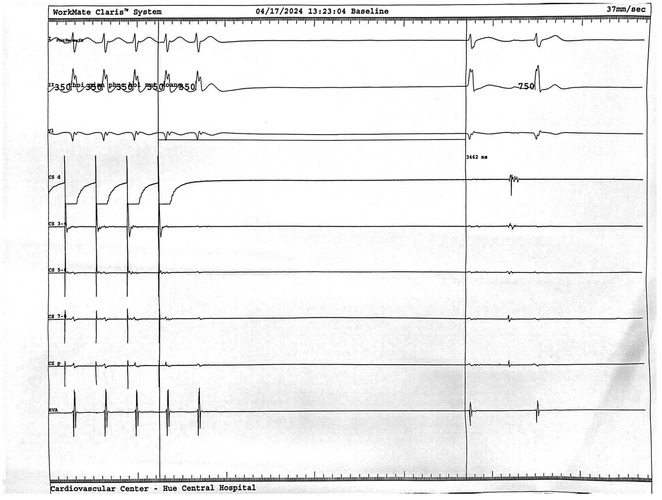

## SUCCESSFUL MANAGEMENT OF THE CO‐EXISTENCE OF THE COARCTATION OF THE AORTA AND WOLFF‐PARKINSON‐WHITE SYNDROME: A CASE REPORT

### **QUOC BAO TRAN**, LE XUAN NGO, ANH BINH HO

#### Hue Central Hospital, Hue City, Viet Nam

**Introduction:** Wolff‐Parkinson‐White (WPW) syndrome is a congenital pre‐excitation syndrome of the conduction system leading to the abnormality of cardiac electrical conduction via the accessory pathways (AP). Coarctation of the aorta (CoA) is a congenital narrowing of the aorta. To the best of our knowledge, there have been a few reports regarding the co‐existence of these two conditions as well as treatment guidelines. Therefore, we are pleased to share a rare clinical case of WPW syndrome co‐existing with CoA and our treatment approach in this case.

**Methods:** N/A

**Results:** A 19‐year‐old female patient was admitted to the hospital because of bilateral lower limb weakness on exertion. The blood pressure in the upper extremities was 160/90mmHg, the blood pressure in the lower limbs was 70/40mmHg, and the bilateral iliac arterial pulse was weak. The computed tomography (CT) of the thoracic aorta showed there was a narrow segment 16mm below the left internal carotid division. We deployed a thoracic aortic stenting intervention for the patient first. We measured pre‐intervention pressure: the ascending aorta (via the right radial artery) recorded 140/100/113 mmHg, and the abdominal aorta (via the right femoral artery) recorded 80/50/60 mmHg, the pressure gradient was 60 mmHg. We decided to deploy an 18 x 38 x 10 atm begraft stents. After that, the patient had palpitation with the ECG recorded 187 bpm regular tachycardia, and a narrow QRS complex. We used 6 mg adenosine intravenous infusion to terminate the tachycardia. The EP study and radiofrequency ablation indicated for the patient showed the AP was located at the right posterolateral site and was successfully eliminated. The patient was discharged from the hospital and did not receive any further antihypertensive and arrhythmic medications.

**Conclusions:** Wolff‐Parkinson‐White syndrome and the coarctation of the aorta are two distinctive congenital heart diseases. The co‐existence of two conditions is rare and should be treated based on the severity of each disease.

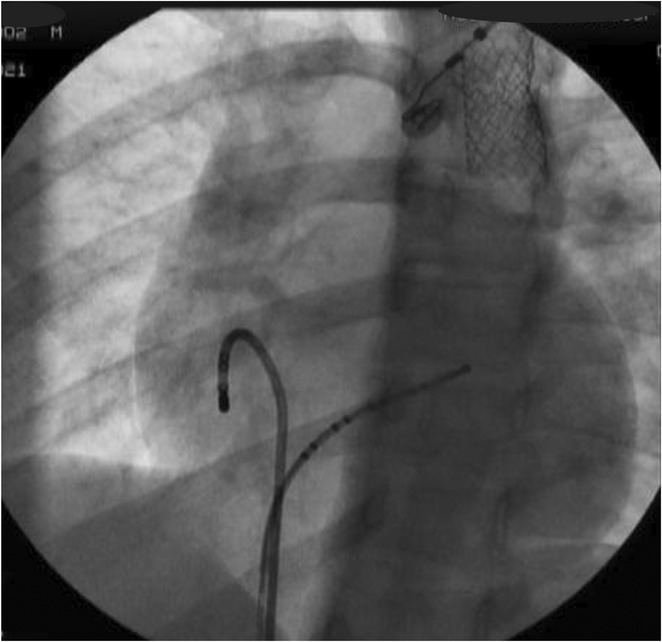

## CHA2DS2‐VASC SCORE AND PULMONARY HYPERTENSION PREDICT ADVERSE OUTCOMES IN ADULT CONGENITAL HEART DISEASE WITH ATRIAL FIBRILLATION

### **WEI‐CHIEH TSENG**, SHUENN‐NAN CHIU, MEI‐HWAN WU, CHUN‐WEI LU

#### National Taiwan University Hospital, Taipei, Taiwan

**Introduction:** The incidence of atrial fibrillation (AF) in adults with congenital heart disease (ACHD) was high. Factors predicting adverse outcomes among them remain unknown. We aimed to find out risks for stroke, major adverse cardiovascular events (MACE) and mortality in ACHD with AF through a large Asian cohort.

**Methods:** All patients aged >18 years with CHD from 2007 to 2018 were enrolled. The basic demographics, ECG/Holter results, and follow‐up results were collected. Patients with atrial flutter (AFL) and AF were categorized as AFL, paroxysmal AF, and non‐paroxysmal AF. CHD was categorized as simple, moderate, and complex. MACE was defined as the composite of total death, myocardial infarction, stroke, and heart failure admissions.

**Results:** From the cohort of 4403 patients, 293 (women, 54.6%) had AF/AFL. Among those with AF/AFL, 15.4% were moderate and 3.9% were complex CHD; 18.1% had AFL, 54.6% had paroxysmal AF, and 27% had non‐paroxysmal AF; the CHA2DS2‐VASc score was low to intermediate (score 0‐1) in 50.9%, and high (score ≥ 2) in 49.1%. Among ACHD with AF/AFL, 70‐year stroke‐free, MACE‐free and overall survival rate was 81.6%, 47.5%, and 75.5% respectively. The most significant risk factor for stroke, MACE, and mortality was the CHD complexity (p < .001); high CHA2DS2‐VASc score was associated with stroke (p = .011). Subgroup analysis according to CHD complexity revealed: in simple CHD, high CHA2DS2‐VASc score was associated with stroke (p = .033); no risk correlated with MACE; pulmonary hypertension significantly correlated with mortality (HR 1.6, p = 0.002), while high CHA2DS2‐VASc showed an inverse correlation (HR 0.53, p = 0.013). in moderate/complex CHD, no risk was associated with stroke; high CHA2DS2‐VASc correlated with MACE (HR 2.33, p = 0.026); pulmonary hypertension predicted mortality (HR 3.55, p = 0.045).

**Conclusions:** In ACHD with AF, the complexity of CHD was the most important risk for adverse outcomes. High CHA2DS2‐VASc score was associated with risk of stroke in simple CHD, and associated with MACE in moderate and complex CHD. Pulmonary hypertension predicted mortality in both CHD subgroups.

## 4 CASES OF PURKINJE DE‐NETWORKING FOR VENTRICULAR FIBRILLATION

### **WATARU TSUNO**, MASAO TAKAHASHI, FUMIYA YOKOZEKI, MASANAO HOMMA, MASATAKA SUNAGAWA, YOSHIAKI MIZUNUMA, TAKAFUMI SASAKI, KOICHIRO YAMAOKA, HIROFUMI KUJIRAOKA, TOMOYUKI ARAI, KIYOTAKA YOSHIDA, KENSUKE KASANO, RINTARO HOJO, TAKAAKI TSUCHIYAMA, SEIJI FUKAMIZU

#### Tokyo Metropolitan Hiroo Hospital, Tokyo, Japan

**Introduction:** Ventricular fibrillation (VF) appears in a variety of cardiomyopathies, is a lethal condition and may not be controlled even with drug therapy and deep sedation. Recently, Purkinje de‐networking has been reported to be effective for preventing VF. The method of Purkinje de‐networking was marking the left bundle branch (LBB) and Purkinje potentials. Thereafter, a virtual triangle was created with its apex formed by the proximal LBB and its base by the most distal Purkinje potentials. Linear ablation was performed along the distal part of the virtual triangle and within the body of the triangle. We report 4 cases of Purkinje de‐networking for VF.

**Methods:** N/A

**Results:** We performed Purkinje de‐networking to 4 cases (3 cases: anterior acute myocardial infarction, 1 case: Brugada syndrome). At first, we performed substrate mapping and identified the location of left anterior fascicle (LAF) and left posterior fascicle (LPF). Next, the Purkinje potential was tagged using a multi‐electrode catheter in the identified triangle, and radiofrequency application was performed using the tagged Purkinje potential as an indicator. Radiofrequency application (40W, 60sec) was performed for left ventricular Purkinje de‐networking (Fig: The distribution of LAF•LPF and Purkinje potential) and substrate modification. If VF was still easily induced, right ventricular Purkinje de‐networking was additionally performed. All cases have progressed without recurrence of VF after ablation.

**Conclusions:** We experienced 4 cases of successful Purkinje de‐networking for VF with no recurrence.

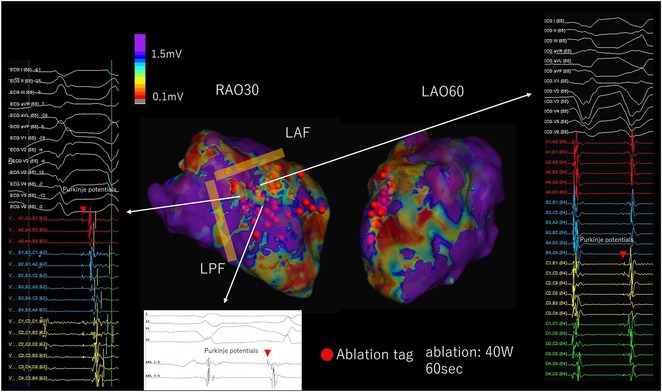

## VARIATIONS IN LOW‐POWER AND HIGH‐POWER SHORT‐DURATION LESION DIMENSIONS: A COMPARISON OF FIVE ABLATION CATHETERS

### **SAMUAL TURNBULL** ^1^, SAMUEL PACK^2^, MICHAEL BARRY^3^, KASUN DE SILVA^1^, ASHWIN BHASKARAN^1^, CLARA CHOW^1^, SAURABH KUMAR^1^

#### ^1^Westmead Hospital, Westmead Applied Research Centre, Sydney, Australia,^2^Westmead Hospital, Westmead, Australia,^3^Westmead Hospital, Sydney, Australia

**Introduction:** High‐power short‐duration radiofrequency ablations rapidly and robustly deliver lesions through resistive tissue heating with minimal conductive tissue heating, associated with thermal damage to adjacent structures. The use of high‐power ablations may still cause inadvertent local tissue damage. One ablation catheter, QDOT Micro ablation catheter (Biosense Webster) has been engineered to minimise risk of adverse events associated with high‐power ablations through automated power and irrigation regulation. We sought to compare the QDOT Micro to four other radiofrequency ablation catheters, each with different distal tip electrode irrigation design, across a spectrum of low‐to‐high powers.

**Methods:** In a validated gel‐tank phantom model, five ablation catheters (QDOT Micro; ThermoCool SmartTouch [ST; Biosense Webster]; ST SurroundFlow [STSF; Biosense Webster]; NaviStar Non‐Irrigated [Biosense Webster]; and TactiFlex SE [Abbott]) delivered repeated low‐power normal‐duration and high‐power short‐duration ablation lesions at power settings from 20‐90W, with contact angles of 0‐90 degrees, and 0.9% and 0.45% saline solution irrigation. Lesion depth, width, and volumes were captured every second to document lesion growth.

**Results:** A total of 639 stable contact ablation lesions were made. Compared to the QDOT at 90W, 4 seconds, 90° catheter angle, mean lesion volume difference for STSF was 3.5mm^3^ (95% CI: 1.87‐5.21mm^3^), *P&*It0.001 and 14.9mm^3^ (95% CI: 9.91‐19.95mm^3^), *P&*It0.001 for 0.9% and 0.45% saline, respectively. Mean volume difference at the same settings for ST were 23.6mm^3^ (95% CI: 21.88‐25.22mm^3^), *P&*It0.001 and 23.5mm^3^ (95% CI: 18.44‐28.48mm^3^), *P&*It0.001 No significant difference was observed in TF lesion volumes delivered at the same parameters.

**Conclusions:** High‐power short‐duration and low‐power normal‐duration lesions produced by different catheters under the same conditions demonstrated variation in lesion dimensions. Contact angle and irrigation choice affect lesion dimensions differently for different catheters.

## BAILOUT LEFT BUNDLE BRANCH AREA PACING IN FAILED CORONARY SINUS LEAD IMPLANTATION

### **YERLAN TURUBAYEV**, ABAY BAKYTZHANULY, SERIK BAGIBAYEV, ZHANDOS YESSILBAYEV, SARDOR YULDASHOV, OMIRBEK NURALINOV

#### University Medical Center CF, Astana, Kazakhstan

**Introduction:** Cardiac resynchronization therapy is a recognized treatment for heart failure with electrical dyssynchrony. CRT implantation can be complex, with challenges such as difficulties in coronary sinus lead placement due to anatomical and technical features. Several studies provide advisability for utilizing left bundle branch area pacing when effective CRT cannot be obtained with a CS lead due to anatomical or functional considerations. We present 2 cases of LBBAP‐CRT in order to conventional coronary sinus lead implantation due to technical challenges.

**Methods:** N/A

**Results:** This case study includes 2 patients, 72 years old woman and 62 years old man with non‐ischemic and ischemic cardiomyopathy. Both patients had symptomatic (NYHA III) heart failure with QRS duration >150ms, and ejection fraction <35% despite 3 month of GDMT. Initial strategy for implantation was conventional biventricular pacing with quadripolar LV lead. During the procedure one patient had CS dissection with following haemopericardium without signs of cardiac tamponade. In second patient, multiply dislodgement of LV electrode as a result of dilated CS branches were observed. In both patients, LBBAP was chosen as an alternative pacing modality. LBBAP was performed using C315HIS sheath with 3830 SelectSecure lead (Medtronic, Minneapolis, MN). Procedure duration and fluoroscopy times: 150/38 min and 240/90 min. Left bundle branch area capture was confirmed by measured peak left ventricular activation time (76 and 78 ms), V6‐V1 inter‐peak interval (46 and 48 ms) and transition from non‐selective to selective capture. LBBAP resulted in narrowing of QRS duration from 160 and 172 ms to 108 and 126 ms respectively. At 6 month follow‐up both patients presented with significant improvement in LV function and quality of life, LV EF increased to 44% and 50%, respectively. Pacing parameters remained stable with low threshlod and acceptable R‐wave amplitude

**Conclusions:** This cases highlights LBBAP as available and safe alternative to coronary sinus lead implantation, demonstrating significant efficacy and leading to improved clinical and echocardiographic outcomes comparable to conventional CRT.

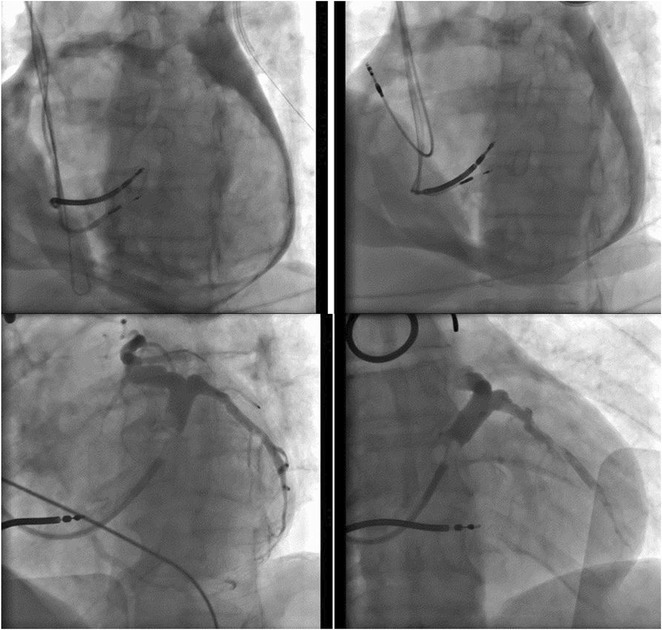

## CARDIAC RESYNCHRONIZATION THERAPY UTILIZING LBBAP OPTIMIZED DUAL CHAMBER ICD. PACING SITE MATTERS?

### **YERLAN TURUBAYEV**, ABAY BAKYTZHANULY, SERIK BAGIBAYEV, ZHANDOS YESSILBAYEV, SARDOR YULDASHOV, OMIRBEK NURALINOV

#### University Medical Center CF, Astana, Kazakhstan

**Introduction:** Cardiac resynchronization therapy (CRT) is a well established treatment option in patients with symptomatic heart failure and left bundle branch block. However, BVP is not enough physiologic pacing modality, which provide electrical resynchronizsation by fused stimulation of both ventricles. Suboptimal coronary venous anatomy and high capture thresholds leds to poor clinical responce. Left bundle branch area pacing is novel pacing modality which may lead to better clinical and echocardiographic response for resynchronization therapy. CRT performed by LBBAP results in lower pacing thresholds, greater narrowing of QRS complex and significantly improves left ventricular function. This prospective study aimed to evaluate efficacy and feasibility of dual‐chamber LBBAP‐optimized ICD therapy for patients with reduced LVEF and LBBB.

**Methods:** 32 patients with indication for conventional CRT underwent LBBAP‐CRT utilizing dual chamber ICD. In patients with suboptimal criteria of LBBA capture we implatned additional coronary sinus lead to obtain left bundle branch area pacing optimized CRT (LOT‐CRT). Baseline data, ECG and TTE characteristics, pacing parameters, procedural outcomes were collected. Follow‐up was performed at 3 and 6 months with monitoring of 12‐lead ECG and TTE with device telemetry.

**Results:** Total 32 patients was observed. 9 (28%) patients required additional CS lead. Fusion between LBBAP and native conduction resulted in significant narrowing of QRS from 171,8±16 ms to 116,6±9,6 ms. At 6 month follow‐up echocardiography showed significant improvement in LVEF (from 28,87±3,9% to 40,25±7,2%). Functional class was improved from 3,4± 1,2 to 1,9±0,9. LBBAP showed low and stable electrical parameters. There were no major lead related complications.

**Conclusions:** LOT‐ICD provides comparable electrical resynchronization to CRT. Implementation of this technology may be promising and cost‐effective for patients with heart failure. LOT‐ICD provides great improvement in heart failure symptoms and echocardiographic outcomes. Further investigations are necessary to confirm effectiveness and safety.

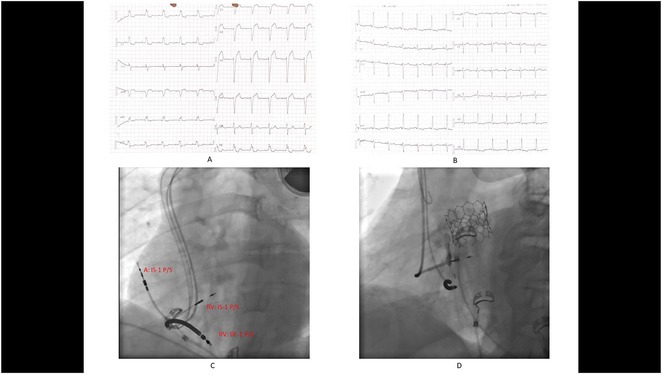

## SUPER RESPONCE FOR LEFT BUNDLE BRANCH AREA PACING OPTIMIZED CARDIAC RESYNCHRONIZATION THERAPY AFTER TRANSCATHETER AORTIC VALVE REPLACEMENT

### **YERLAN TURUBAYEV**, ABAY BAKYTZHANULY, ZHANDOS YESSILBAYEV, SERIK BAGIBAYEV, SARDOR YULDASHOV, OMIRBEK NURALINOV

#### University Medical Center CF, Astana, Kazakhstan

**Introduction:** Transcatheter aortic valve replacement is a feasible treatment option for patients with severe aortic stenosis and high surgical risk. Ventricular hemodynamic unloading by TAVR improves left ventricle function and heart faulire symptoms, decrease the mortality and reduce decompensations of pre‐excisting heart failure. We present the case of complete normalization of left ventricular function in heart failure patient with LBBB and severe aortic stenosis, who underwent transcatheter aortic valve replacement and LBBAP‐CRT implantation.

**Methods:** N/A

**Results:** 66‐year old male with severe aortic stenosis and heart failure with reduced LV EF presented to the clinic with evaluation of dyspnea, chest pain and gasp in horizontal plane. Previous medical history includes severe hypertension, paroxysmal atrial fibrillation, pulmonary embolism, type 2 diabetes mellitus, type B COPD. Coronary angiography showed absence of critical stenosis. Transthoracic echocardiography showed decreased LV ejection fraction (31%), EDD 48 mm, ESD 39 mm, EDV 161 ml, ESV 110 ml, moderate mitral regurgitation, PAP 50 mmHg, AVA (VTI) 0,36 cm2, AV peak gradient 71 mmHg, mean gradient 47 mmHg, moderate aortic regurgitation. ECG showed LBBB with QRS duration of 196 ms. Operative mortality risk assessed by the STS was 2.85%. The TAVR procedure was done utilizing MyVal 27,5 valve. LBBAP was performed using the SelectSecure pacing lead. Leads were connected to dual chamber ICD with DF‐1 configuration. Sensed AV delay was programmed to 70 ms, resulting fusion with native conduction and narrowing of QRS to 122 ms. After 6‐months follow‐up patient underwent TTE, which sowed significant increase of left ventricle ejection fraction (49%), decreasing of EDV (133 ml) and ESV (67%), improved NYHA functional class and quality of life.

**Conclusions:** LBBAP provides comparable electrical resynchronization to conventional CRT. Simultaneously performed TAVR and CRT provides improvement in heart failure related symptoms and echocardiographic responce.

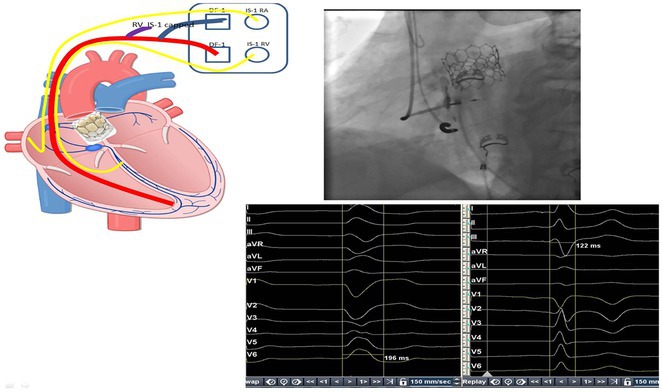

## EFFICACY OF CORTICOSTEROID THERAPY FOLLOWING INITIAL SUSTAINED VENTRICULAR ARRHYTHMIA ASSOCIATED WITH CARDIAC SARCOIDOSIS

### **AKIKO UEDA** ^1^, KYOKO SOEJIMA^1^, KOJI NAKAGAWA^2^, REINA TONEGAWA‐KUJI^3^, KOJI MIYAMOTO^3^, MIYAKO IGARASHI^4^, KENJI OKUBO^5^, AKIHIKO NOGAMI^4^, KENGO KUSANO^3^

#### ^1^Kyorin University, Tokyo, Japan,^2^Okayama University, Okayama, Japan,^3^National Cerebral and Cardiovascular Center, Osaka, Japan,^4^Tsukuba University, Tsukuba, Japan,^5^Yokosuka Kyosai Hospital, Yokosuka, Japan

**Introduction:** The impact of corticosteroid therapy (PSL) on sustained ventricular arrhythmias (sVA) associated with cardiac sarcoidosis (CS) is controversial; although inflammation suppression prior to the ablation is ideal, it is also known that VA transiently increases after initiation of PSL. The significance of early introduction of PSL has not been established. The aim of this study was to examine the impact of PSL use following initial sVA episodes in CS patients on subsequent sVA recurrence.

**Methods:** Of 137 patients enrolled in the registry which included CS patients with any kind of arrhythmia registry at 5 Japanese centers, 74 patients with the history of sustained VA were selected. The effect of PSL use status up to the initial 3 month following the first sVA on subsequent recurrence was examined.

**Results:** During the median 77 (Interquartile range; IQR 32 ‐ 115) month follow‐up period, 42 patients (57%) had sVA recurrence. sVA‐free survival rates at 24 and 60 months were 59% and 42%, respectively. Upon the initial sVA, 57patients were not on PSL and 17 on PSL. Twenty‐six patients who were newly started on PSL within 3 months had significantly fewer sVA recurrences up to 60 months than those who were already on or no PSL treatment initiated (adjusted hazard ratio; HR 0.392, 95% confidence interval; CI 0.175 ‐ 0.876, p = .022). Catheter ablation (CA) performed in 28 patients within 3 months was not associated with recurrence (HR 1.208, 95% CI 0.616 ‐ 2.370, p = .583).

**Conclusions:** In CS patients who developed sVA, early PSL initiation prevented subsequent recurrence. CA at the early timing did not prevent recurrence.

## ELECTROPHYSIOLOGICAL CHARACTERISTICS OF TACHYARRHYTHMIAS AND EFFECTS OF CATHETER ABLATION IN PATIENTS WITH FONTAN CIRCULATION

### **JAE‐SUN UHM**, JA‐KYOUNG YOON, HANJIN PARK, DAEHOON KIM, HEE TAE YU, TAE‐HOON KIM, BOYOUNG JOUNG, HUI‐NAM PAK, MOON‐HYOUNG LEE

#### Yonsei University Severance Hospital, Seoul, Korea, Republic of

**Introduction:** This study aimed to elucidate arrhythmia burden and effects of catheter ablation for tachyarrhythmias in patients who underwent Fontan operation for functional single ventricle.

**Methods:** The patients with Fontan circulation who had undergone catheter ablation for tachyarrhythmia were retrospectively consecutively included. Characteristics of congenital heart disease, mechanisms of tachyarrhythmia, how to approach to the heart, efficacy and safety of catheter ablation were analyzed.

**Results:** Total 20 patients (age, 22.8 ± 9.4 years, 9 men) with Fontan circulation who underwent catheter ablation for tachyarrhythmia were included. In these patients, 30 procedures were performed. Congenital heart diseases of the patients were as follows: double outlet right ventricle, 7; common atrioventricular septal defect, 7; pulmonary atresia, 4; transposition of the great arteries, 3; tricuspid atresia, 3; and total anomalous pulmonary venous return 1. Seven patients with heterotaxia syndrome were included; right isomerism, 6 and left isomerism, 1. Fontan types were as follows: lateral tunnel Fontan, 9 and extracardiac conduit Fontan 11. In 1 patient, no tachycardia was induced by programmed electrical stimuli. Fontan conduit puncture was successful in 18 of 19 patients. For Fontan conduit puncture, inferior vena cava and superior vena cava approach were used in 18 and 1 patients, respectively. In one patient, puncture of the Goretex extracardiac conduit was failed. For Fontan conduit puncture, the cut stylet was used. In 13, 10, and 7 patients, cut stylet, balloon, snare were used, respectively. Tachyarrhythmia Mechanisms were as follows: focal atrial tachycardia (AT), 9; intraatrial macroreentrant tachycardia (IART), 6; twin atrioventricular node reciprocating reentrant tachycardia, 4; AVNRT, 3; and unknown, 2. Acute success rate of catheter ablation, recurrence rate, and complication rate were 73.3%, 5.3%, and 0%, respectively.

**Conclusions:** Focal AT and IART are the most common tachycardia mechanisms in patients with Fontan circulation. Catheter ablation is effective and safe.

## ENHANCING HEALTHCARE EFFICIENCY: THE VALUE PROPOSITION OF VESSEL CLOSURE DEVICES IN CARDIAC ELECTROPHYSIOLOGY PROCEDURES

### **BHARAT PHANI VAIKUNTAM**, SHEBNEM ERDOL

#### Abbott Medical Australia Pty Ltd, Sydney, Australia

**Introduction:** Effective management of vascular access following cardiac ablation procedures is essential to mitigate potential complications, reduce recovery duration and minimise associated costs. While previous clinical studies globally have demonstrated the safety and efficacy of suture‐medicated Vessel closure devices (VCDs) (such as ProStyle^TM^) in procedures requiring cardiac catheterisation such as cardiac ablation interventions, their adoption rates vary across healthcare systems. A recent Australian study also revealed significant additional resource burden from bleeding‐related complications ranging between $4,250 ‐ $9,426 per patient. This analysis aims to assess the potential benefits of VCDs, focusing on cost savings, efficiency gains and reductions in clinical staff time within cardiac ablation procedures.

**Methods:** This preliminary analysis compares resource utilisation and costs of VCDs versus other closure techniques (manual compression/figure‐of‐8) in cardiac ablation procedures from the healthcare providers perspective in Australia. A detailed analysis of the patient clinical resource use from vessel closure till discharge was conducted using inputs from clinical experts, published clinical studies and publicly available resource costing information.

**Results:** The analysis demonstrated potential cost savings of more than $900 per patient across public and private hospital settings, alongside significant reductions in nursing staff time.

**Conclusions:** Vessel Closure Devices (VCDs) offer immediate and durable haemostasis, minimising rebleeding concerns and risk of access site complications and enhancing overall patient satisfaction. In resource‐constrained healthcare environments, the efficiency gains from expedited ambulation, reduced risk of potentially avoidable complications and readmissions, early discharge, and savings in nursing staff time, present significant value for healthcare systems across public and private hospital settings.

## POTENTIAL COST SAVINGS FROM EXTENDED BATTERY LONGEVITY OF AVEIR^TM^ VR

### **BHARAT PHANI VAIKUNTAM** ^1^, SHEBNEM ERDOL^1^, ARIF FAHIM^2^

#### ^1^Abbott Medical Australia Pty Ltd, Sydney, Australia,^2^Abbott Medical Singapore, Singapore, Singapore

**Introduction:** Aveir^TM^ VR is the latest generation leadless single chamber pacemaker (LP) technology in the world, indicated for patients with significant bradycardia and normal sinus rhythm with rare episodes of AV block or sinus arrest, chronic atrial fibrillation, and severe physical disability. Aveir VR LP with rate modulated pacing includes several novel features that are not present in conventional single‐chamber PMs, endocardial leads, or other LPs in the market including extended battery longevity, upgradability and chronic retrievability.

**Methods:** Two approaches were used to determine battery longevity: first using the pacing parameters [pacing threshold of ≤1 V (mean 0.60±0.38 V) at 0.24‐ms pulse duration] from Duray et al (2017) publication, the projected longevity of Aveir VR was estimated at 21.5 years, with an incremental longevity of 9.4 years over other LPs in the market and traditional pacemakers (12.1 years). Second approach based on Leadless II Phase 2 IDE study findings, the mean longevity of Aveir VR was 17.6 years (95% CI: 16.6‐18.6 years), with an incremental longevity of 5.5 years. A Cost‐consequence analysis was conducted to demonstrate the potential cost savings (weighted by patient age distribution) from extended battery longevity of Aveir VR (battery capacity of 243 mAh: 2x battery capacity than other LPs) from an Australian perspective. The analysis was limited to device‐related cost savings from device replacements avoided and did not include potential additional cost savings from avoided procedure costs, associated complication costs, and bed days saved.

**Results:** Based on incremental battery longevity of Aveir VR and the number of implants avoided per patient, the estimated potential device‐related cost savings ranged between AUD$5,551‐$7,767 per patient for an incremental longevity of 5.5 and 9.4 years respectively.

**Conclusions:** Aveir VR LP with extended battery longevity may result in significant cost savings to the payer and healthcare system. Fewer reinterventions, especially at advanced ages, significantly reduce patient mortality, complications risks associated with reintervention for device replacement, and quality‐of‐life impact.

## LEFT BUNDLE BRANCH AREA PACING IN PATIENTS WITH SEVERE LEFT VENTRICULAR SEPTAL HYPERTROPHY: A COMPARATIVE STUDY BETWEEN LUMEN LESS LEAD AND STYLET DRIVEN LEADS

### **SANJAI VALAPPIL**, SHAIMA HAFEEZ, SUBRAMANYAN KRISHNASWAMY, PRAMOD KUMAR JAISWA, PRASAD GOLLA, JAYANTHI K, SRINATH TS, SHARAN SHREEDHARAN, SHILPA MENON

#### SIMS, Chennai, Chennai, India

**Introduction:** There has been limited success in utilising left bundle branch area pacing (LBBAP) in patients with severe left ventricular septal hypertrophy (LVSH). This may be due to the additional length the pacing lead has to traverse in the hypertrophied inter ventricular septum (IVS). This study assesses the feasibility, safety, and outcomes of LBBAP in patients with severe LVSH using the Medtronic lumen less lead (MLLL) and the St Jude Abbott stylet‐driven lead (SSDL)

**Methods:** Adult patients who underwent LBBAP in our centre between August 2019 and February 2024 were enrolled. Severe LVSH was defined as IVS thickness > 16 mm in women and > 17 mm men. Baseline patient characteristics, procedural data and post procedural results were collected and analysed.

**Results:** Ten patients with severe LVSH and 75 patients with normal septal thickness (NST) underwent LBBAP during the study period. Implant success was achieved in 71 patients with NST (94.6%) and 7 patients with severe LVSH (70 %). Among the 10 patients with severe LVSH, 2 out of 4 patients (50 %) and 5 out of 6 patients (83.3%) underwent successful LBBAP with the MLLL and SSDL respectively (p:0.33). Mean septal thickness (20±0.81 mm vs 21±0.65 mm p:0.842) and lead penetration depth (17±0.5 mm vs 17.1±0.85 mm p:0.642) were comparable in both groups. Paced QRS duration (124±10 ms vs 126± 12 ms p:0.124) and left ventricular activation time (LVAT) (76±10 ms vs 74± 8 ms p:0.55) were similar in both the groups. The cause of failure of LBBAP in the MLLL group was the inability to advance the lead in the IVS, whereas the patient with the failed LBBAP in the SSDL group had high threshold despite capture of the conduction system. The lead thresholds ( 0.65 ±12 vs 0.85 ±11) remained stable over the mean follow up duration of 28.3 ± 16.8 months and there were no lead related complications.

**Conclusions:** The study indicates that LBBAP in patients with severe LVSH is safe and feasible and no lead‐related complications were observed. It may be prudent to use the SDL in this group of patients as they offer additional torquable force while advancing the lead into the stiff septum.

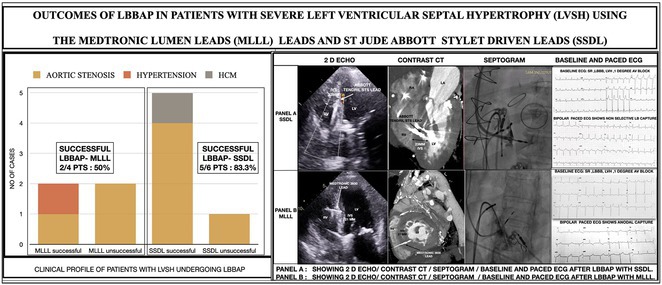

## NOVEL USE OF THE MEDTRONIC 3830 LEAD AS A BIOPTOME IN A CASE OF SUSPECTED INFILTRATIVE CARDIOMYOPATHY

### **SANJAI VALAPPIL**, SIRISHA PUSAPATI UMA, SUBRAMANYAN K, PRAMOD KUMAR JAISWAL, PRASAD GOLLA, JAYANTHI K, SRINATH TS, BANUMATHY RAMAKRISHNA

#### SIMS, Chennai, Chennai, India

**Introduction:** A 38‐year gentleman with left sided cardiac resynchronisation therapy ‐ defibrillator (CRT‐D) pocket infection underwent successful lead extraction. His subsequent management is outlined below

**Methods:** N/A

**Results:** The need for CRT‐D was critically reevaluated. His echocardiography showed moderate left ventricular (LV) dysfunction. Interrogation of the CRT‐D device showed one run of slow ventricular tachycardia (VT) and presence of mobitz type 2 atrioventricular (AV) block at atrial rates above 100 bpm. The options of right sided CRT‐D and left bundle branch area pacing (LBBaP) optimised ICD implantation were discussed with the patient. LBBaP was attempted after an informed consent. However despite consistent capture of the conduction system, left ventricular activation time (LVAT) was 90 ms at high and low output unipolar and bipolar pacing, whereas the LVAT during intrinsic rhythm was 70 ms. Pacing was attempted at multiple sites at the proximal and mid inter‐ventricular septum.There was a high suspicion of infiltrative cardiomyopathy in view of the young age of patient, combination of AV block, LV dysfunction, slow VT and the high normal paced LVAT ( 20 ms more than the intrinsic LVAT) despite conduction system capture. It was thus decided to send the septal tissues attached to the Medtronic 3830 lead for histopathology as an alternative to endomyocardial biopsy. A conscious decision was made to avoid LBBaP and subsequently successful right sided CRT‐D implantation was done. Histopathology report showed evidence of interstitial fibrosis without fatty infiltration, there was no evidence of lymphangiogenesis or epitheloid granulomas. He was discharged with guideline directed medical therapy for heart failure and antibiotics in a stable condition. FDG PET scan done after 3 months was positive for infiltrative cardiomyopathy. Patient is currently on immunomodulators for the same.

**Conclusions:** The Medtronic 3830 lead can be used as a bioptome for targeted endomyocardial biopsy. High normal paced LVAT longer than the intrinsic LVAT should alert us to the possibility of underlying infiltrative cardiomyopathy.

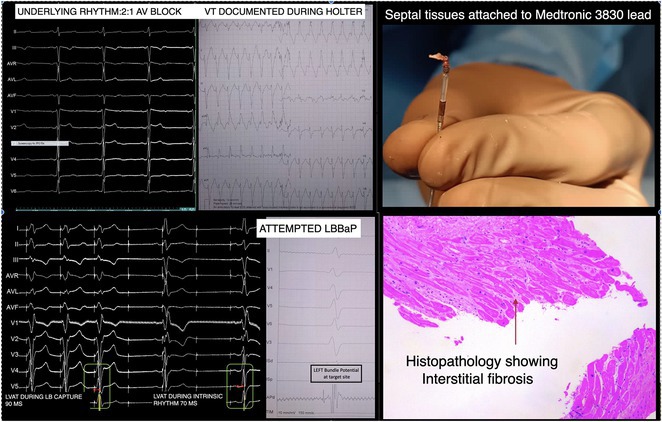

## SAVING ONE HEART AT A TIME: THE MHYH (MY HEART YOUR HEART) RANDOMIZED TRIAL OF NEW VS. REFURBISHED PACEMAKERS IN LOW‐ AND MIDDLE‐ INCOME COUNTRIES

### **DEEPTHY VARGHESE** ^1^, NIRAJ SHARMA^1^, ERIC PUROLL^2^, PATSY BRUENGER^2^, EVA KLINE‐ROGERS^2^, CHIH‐WEN PAI^2^, CONSTANTINA ALEXANDRIS‐SOUPHIS^2^, VICENTE FINIZOLA^3^, EMMANUEL EDAFE^4^, ADRIAN EBNER^5^, CONSTANTINE AKWANALO^6^, OLUWASEYE OLADIMEJI^7^, JAMES RUSSELL^8^, JORGE BAHENA^9^, KIM EAGLE^2^, ALBERTINO DAMASCENO^10^, THOMAS CRAWFORD^2^

#### ^1^Northside Hospital, Atlanta, GA,^2^University of Michigan, Ann Arbor, MI,^3^ASCARDIO, Barquisimeto, Venezuela, Bolivarian Republic of,^4^Port Harcourt Teaching Hospital, Port Harcourt, Nigeria,^5^FUNDACOR, Asuncion, Paraguay,^6^Moi Teaching and Referral Hospital, Eldoret, Kenya,^7^Lagos State University Teaching Hospital (LASUTH), Lagos, Nigeria,^8^University Sierra Leone Teaching Hospitals Complex, Freetown, Sierra Leone,^9^Hospital Universitario Universidad Autonoma de Nuevo Leon, Monterrey, Mexico,^10^Eduardo Mondlane University, Maputo, Mozambique

**Introduction:** Pacemaker implantation rates in low‐ and middle‐income countries are significantly lower than in the high‐income countries. High cost of pacemakers is one reason for this disparity. Project My Heart Your Heart (MHYH) has developed a reprocessing protocol for pacemaker reuse, which has been validated in terms of sterility and electrical functionality.

**Methods:** Pacemakers were sourced in the United States from patients undergoing device explants or upgrades in hospitals (life donor), and from the funeral industry (postmortem donor). Pacemakers were reprocessed according to a standardized protocol and the protocols have been published. Health authorities and institutional review boards of each country and site approved the trial. Eligibility criteria were: 1) class I indication for pacing, 2) inability to afford a new pacemaker, and 3, informed consent to being randomized to receive a reconditioned or new pacemaker. This is a multi‐center, single‐blind non‐inferiority study of refurbished vs. new pacemakers (ClinicalTrials.gov NCT 04016870). The primary endpoint is freedom from procedure‐related infection at 12 months. The secondary endpoint is freedom from pacemaker software or hardware malfunction (premature battery depletion, device or electrode malfunction, or unexplained death) at 12 months follow‐up.

**Results:** Eight centers have joined the trial from the following seven countries: Venezuela, Nigeria, Paraguay, Kenya, Sierra Leone, Mexico, and Mozambique. A total of 273 patients have been enrolled. Clinical follow‐up is on ongoing.

**Conclusions:** In this prospective randomized trial, we hope to demonstrate non‐inferiority of reconditioned pacemakers vs. new pacemakers in terms of freedom from procedure related infection or device malfunction. This study has a great potential to make pacemaker reuse recognized as an ethical and safe option for patients in low‐ and middle‐income countries who are not able to receive a new pacemaker due to its cost.

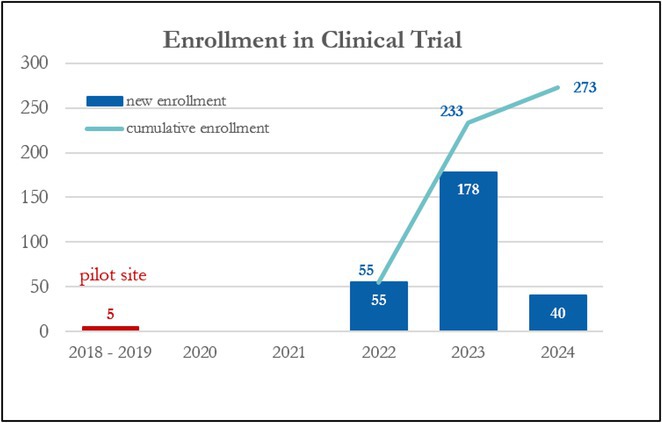

## UNDELIVERED SHOCK IN UNSTABLE HEMODYNAMIC VT: THE HURDLES OF DISCRIMINATION OF ICD MORPHOLOGY CRITERION

### **ZHAFRAN VELIAWAN**, ARDIAN RIZAL, ADHIKA PRASTYA, MOHAMMAD AZHAR ROSYIDI, ANASTASIA CHRISTINE

#### Faculty of Medicine, Universitas Brawijaya, Malang, Indonesia

**Introduction:** Implanted cardioverter defibrillators (ICDs) can sometimes administer inappropriate therapy, leading to unnecessary or undelivered shocks. This often occurs when the device misclassifies sinus tachycardia or atrial fibrillation/flutter with rapid atrioventricular conduction. Modern ICDs use multiple discriminators to distinguish these rhythms. However, errors can occur, as illustrated by a case where a single chamber discriminator ICD with morphology criterion misclassified ventricular tachycardia as supraventricular tachycardia, causing a patient to suffer a seizure and cardiac arrest without receiving shock therapy.

**Methods:** N/A

**Results:** The 40‐year‐old patient, with a history of recurrent ventricular tachycardia, coronary artery disease, and complete revascularization, experienced seizures following cardiac arrest. Immediate CPR in the ER led to a return of spontaneous circulation. ICD interrogation revealed non‐sustained VT and SVT episodes, but no recorded VT episode or shock therapy. Electrophysiology studies showed no induced supraventricular tachycardia and normal AV and SA node function. Coronary angiography confirmed patent stents in all coronary vessels.

**Conclusions:** The patient's seizures and cardiac arrest were due to the ICD misclassifying ventricular tachycardia episodes as SVT, leading to undelivered shock therapy. Morphology discriminating criterion errors in single chamber ICDs might happen due to some possible causes. It was highly recommended to increase the duration of the detection period to avoid misclassifying ICD.

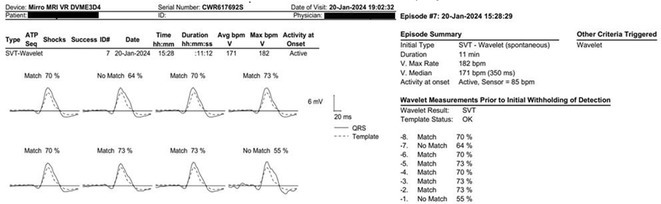

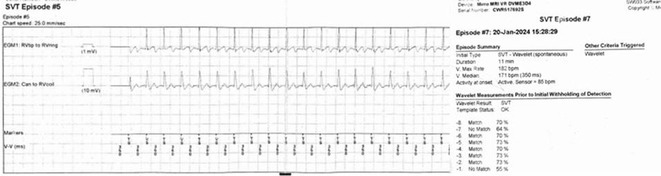

## LONG‐TERM DURABILITY OF A STYLET‐DRIVEN LEAD DESIGN FOR LEFT BUNDLE BRANCH AREA PACING

### HAO LIU, **KEITH VICTORINE**, WENWEN LI, WESLEY ALLEMAN, LEONARD GANZ

#### Abbott, Sylmar, CA

**Introduction:** Left bundle branch area pacing (LBBAP) has emerged as a promising approach to physiologic pacing. This study aimed to evaluate the long‐term durability of a stylet‐driven lead for LBBAP.

**Methods:** A human beating heart model ‐ Living Heart Model (Dassault Systemes, RI) was utilized for the lead durability analysis. The curvature change of lead body and stress on the lead conductor was calculated for various implant locations (RA, RV, His Bundle, LBBAP). **In the first analysis**, LBBAP implants with various insertion angles, lead depths, locations on the septum, and lead slack were studied in a heart model (RV basal diameter: 48.0 mm). **The second analysis** used the worst‐case scenario from the first analysis in a variety of heart sizes (RV basal diameter range: 25.7 to 64.4 mm). Safety factor was determined using stress and the mechanical properties of the conductor (Figure 1A). A higher safety factor indicates less stress on the lead and higher conductor durability. The survivability of Abbott stylet driven leads were also evaluated in a bench‐top fatigue test at higher stress states, thus at a lower safety factors, than the LBBAP modeling analysis cohort.

**Results:** A total of 8 implant scenarios for LBBAP, two RV (apex and apical septum), one HBP and one RA implant were modeled in the **first analysis**. The safety factors of LBBAP implants were comparable to the other implant locations (Figure 1B). Seven LBBAP implants were modeled in the **second analysis**. Normal hearts had lower safety factors compared to the enlarged hearts (Figure 1C). The durability of lead conductor was verified through successful lead fatigue testing at conservative safety factors of 2.00 ± 0.12 which had higher stress than all the LBBAP cases in both normal and enlarged hearts.

**Conclusions:** Implanting Abbott leads at the LBBA did not introduce any further concern on conductor's survival rate compared to the HBP or traditional RA/RV implant locations in a computational model. Successful bench testing at extreme conditions beyond expected in‐vivo conditions demonstrates the long‐term durability of Abbott leads implanted in the LBBA.

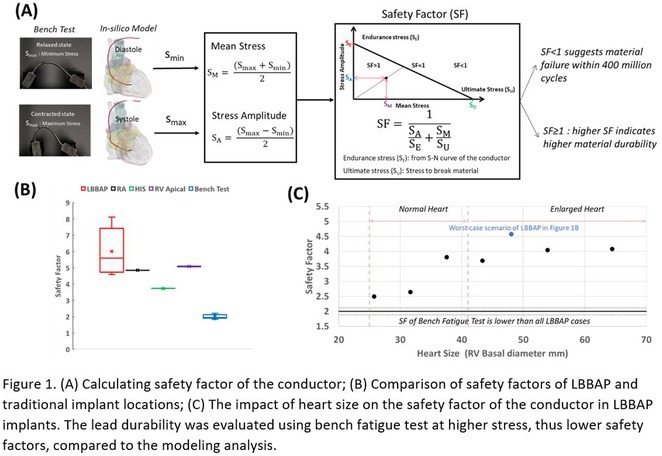

## NOVEL LEAD FIXATION TOOL PROVIDES CONTROL OVER THE EXTENSION AND RETRACTION OF THE HELIX FOR CONDUCTION SYSTEM PACING

### STEVE CHANTASIRIVISAL, CHRISTIAN MEDINA, PETER FONG, **KEITH VICTORINE**

#### Abbott, Sylmar, CA

**Introduction:** The Abbott Helix Locking Tool (HLT) is an accessory component to the cardiac active fixation IS‐1 implantable lead, that was designed to aid with lead fixation by providing the user with control over the extension and retraction of the helix.

**Methods:** Engineering characterization studies were performed to assess the maximum implant torque and percent change of helix electrode retraction with and without the HLT. Measurements of the helix length were performed before and after burrowing the distal end of the lead 1cm into porcine septal tissue and surrogate tissue to confirm suitability of the surrogate tissue. Helix retraction comparison with other leads and their associated implant tools were repeated in surrogate tissue.

**Results:** Torque‐in values were used to determine suitability between porcine septal and surrogate tissue. Results demonstrated surrogate tissue (avg 0.39 in‐oz) representing a slightly more challenging tissue substrate than porcine septal tissue (avg 0.24 in‐oz). Percent change of helix electrode retraction in porcine vs surrogate demonstrated 0% retraction with the HLT compared to 23% retraction when no HLT was used. Comparison of the helix retraction between the Abbott HLT and other leads demonstrated an average of 18% and 42% retraction of other leads while Abbott HLT showed 0% retraction.

**Conclusions:** The Abbott Helix Locking Tool successfully fixates the helix in the forward position with no retraction during deep septal placement of the lead; whereas other leads with their associated implant tools demonstrate helix retraction. The developed surrogate tissue serves as an appropriate tissue substrate for use during testing to obtain more repeatable results.

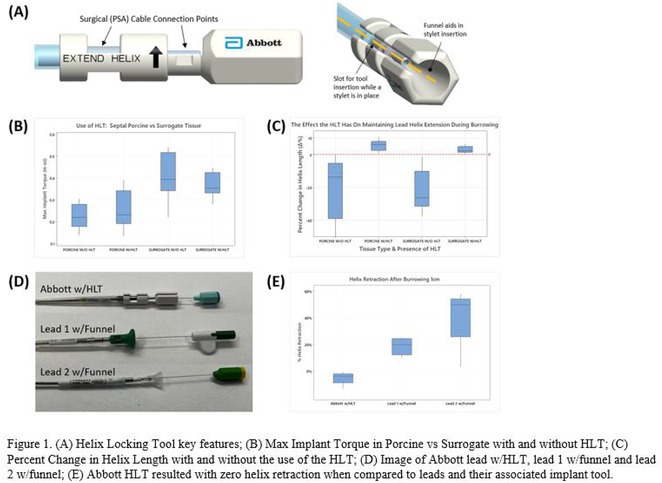

## REPLACE NEW POSITION OF LEFT VENTRICULAR LEAD FOR PATIENT WITH SEVERE HEART FAILURE WHOM NON RESPONDER TO CARDIAC RESYNCHRONIZATION THERAPY. (CRT)

### **LONG VIEN HOANG**, LINH PHAM TRAN, THUY NGUYEN THI LE, TUAN NGUYEN DUY

#### Vietnam National Heart Institute ‐ Bachmai Hospital, Hanoi, Viet Nam

**Introduction:** A 60‐year‐old male patient with a history of hypertension, diabetes mellitus, and anterior myocardial infarction underwent intervention for the left main (LM) and left anterior descending (LAD) arteries in 2017. After the intervention, there was no recovery of biventricular function, with an ejection fraction (EF) of 35%. The patient was indicated for cardiac resynchronization therapy (CRT) device implantation. He has been compliant with medication, currently taking Sacubitril/Valsartan 100 mg twice daily, Clopidogrel 75 mg once daily, Rosuvastatin 10 mg once daily, Bisoprolol 5 mg once daily, Furosemide 40 mg once daily, Verospirone 50 mg once daily, Empagliflozin 10 mg once daily, and Metformin 1000 mg once daily. However, the patient's cardiac function did not improve, with an ejection fraction of 23% upon admission.

**Methods:** N/A

**Results:** The patient returned for follow‐up when it was time to replace the CRT device. On chest X‐ray, it was noted that the left ventricular lead was positioned in the great cardiac vein, which could be one of the reasons for the lack of response to CRT and lack of improvement in cardiac function. Venography with contrast injection showed that the left subclavian vein remained patent. We decided that during the CRT device replacement, a new left ventricular lead would be placed in the posterior lateral branch of the coronary vein. After successfully implanting the new left ventricular branch lead, the patient continued the same medication regimen. After 3 months of follow‐up, the patient's cardiac function significantly improved with an ejection fraction of 50%.

**Conclusions:** There are many causes for non‐response to cardiac resynchronization therapy, and one of the modifiable causes is the selection of the left ventricular lead position. Cases where the left ventricular lead position is suboptimal should have the lead replaced to increase the chances of responding to cardiac resynchronization therapy.

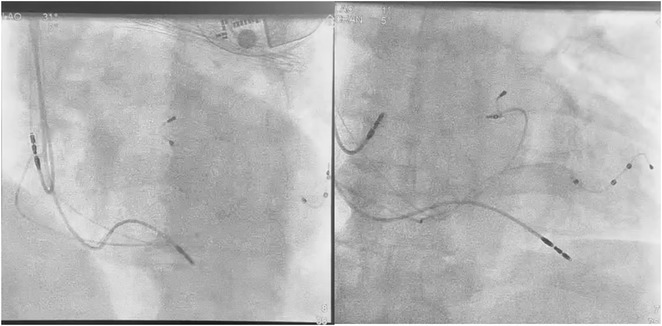

## A CASE OF TACHYCARDIOMYOPATHY ABLATED USING SPARKLE MAP

### **JYOTHI VIJAY**, ABHILASH SP, NARAYANAN NAMBOODIRI

#### Sree Chitra Tirunal Institute for Medical Sciences and Technology, Trivandrum, India

**Introduction:** N/A

**Methods:** N/A

**Results:** A 37‐year‐old male presented with palpitation of 12 months and dyspnea for 1 month. He occasionally consumes alcohol. No other comorbidities. On examination, Tachycardia, HR ‐130 bpm, BP 100/76 mm Hg. The cardiovascular exam showed an S1 soft, pan‐systolic murmur at the apex. ECG showed atrial flutter with intermittent ectopic of outflow tract morphology. The echocardiogram showed moderate LV dysfunction, LVEF 36%,MVP of AML with moderate MR, no PAH, and LV dilated with a diagnosis of likely tachycardiomyopathy.The patient was taken up for an EP study. The entire RA was mapped with an advisor HD grid catheter, which showed a large area of early activation in the upper interatrial septum. Tricuspid annulus and CTI region was excluded from the tachycardia circuit by entrainment.LA was mapped, and the scar was delineated. Sparklemap technology available in the 3d mapping system was used to visualize the local activation sequence upon the substrate map. It allows each electrogram to be visualized as a fluorescent dot lighting up corresponding to activation time. This helped to give targeted ablation and tachycardia terminated. Ablation was completed with a roof line.PVC was subsequently mapped in RVOT. This patient had Denovo scar‐related left atrial flutter, MVP with moderate MR, moderate LV dysfunction, likely tachycardiomyopathy, EPS showed Denovo scar‐related LA flutter, successful roof line done guided ny sparkle map and VPC from outflow tract. On follow up his LV function improved and free of symptoms.

**Conclusions:** Entrainment mapping with addition of newer 3D mapping technologies for scar delineation helps in identifying the circuits and planning ablation.

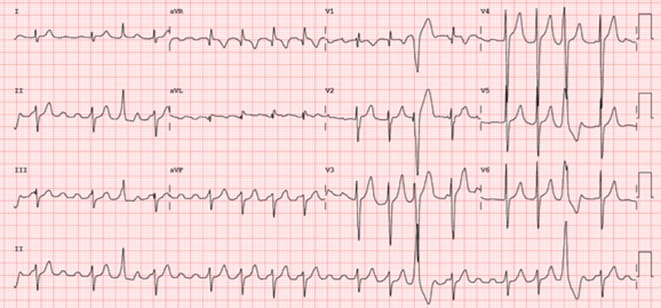

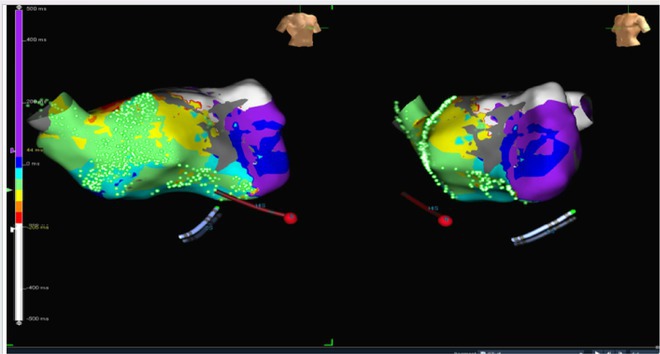

CORRELATION OF ARRHYTHMIAS WITH PEAK AND TROUGH DIGOXIN LEVELS USING AMBULATORY ELECTROCARDIOGRAPHY

**JYOTHI VIJAY** ^1^, HIMANSHU GUPTA^2^, NEELAM DAHIYA^2^, AJAY BAHL^2^

^1^Sree Chitra Tirunal Institute for Medical Sciences and Technology, Trivandrum, India,^2^Postgraduate Institute of Medical Education and Research, Chandigarh, India

**Introduction:** Digoxin was one of the earliest drugs to be used in the management of heart failure and rate control of arrhythmias. Fear of arrhythmic side effects limits the use of digoxin. The relationship between arrhythmias and the varying digoxin blood levels throughout the day is not known. We hypothesized that arrhythmias might increase at the time of peak digoxin blood levels and reduce at the time of trough levels. We aimed to study the relationship of ventricular and supraventricular ectopic with the timing of digoxin dose using ambulatory electrocardiographic monitoring.

**Methods:** This was a prospective observational study on 50 patients who were on digoxin therapy. 24‐hour Holter was carried out, and a 0.25 mg tablet of digoxin was administered 4 hours after connecting Holter. The 24‐hour period was divided into 6 intervals of 4 hours each. Incidence of supraventricular and ventricular arrhythmias among these 6 intervals was compared in between and with timing of peak levels. The pre‐specified time interval of 2‐6 hours after digoxin intake was assumed to be the period with peak digoxin levels and was compared with other time intervals. Post‐hoc pairwise tests for the Friedman test were performed using the Nemenyi test method and analyzed.

**Results:** 28 (56.0%) participants had severe LV dysfunction and non‐ischemic dilated cardiomyopathy, 7 (14.0%) had ischemic cardiomyopathy, and 15 (30.0%) had rheumatic heart disease. There was no correlation between supraventricular and ventricular ectopics with respect to timing from the digoxin dose (p>0.05). A subgroup comparison of eight patients with high (10‐100/hour) and three with very high ventricular ectopic burden (>100/hour) also did not show any significant correlation of ectopics with timing after digoxin dose.

**Conclusions:** Supraventricular and ventricular ectopics in patients on digoxin therapy were unrelated to peak or trough digoxin levels. This finding is important if confirmed in larger studies because digoxin is a low‐cost and readily available drug and can be a very good tool in armamentarium for managing heart failure and atrial fibrillation.

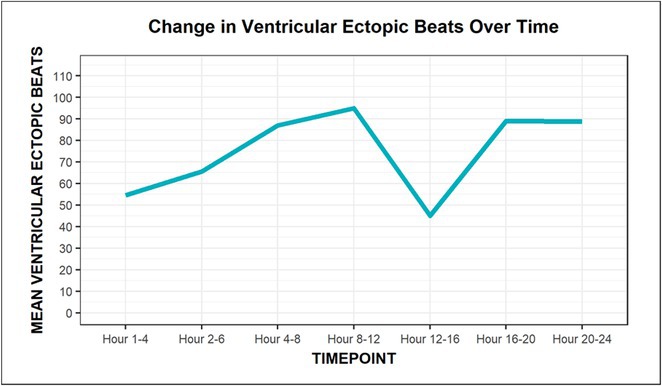

## EFFECT OF LEFT BUNDLE BRANCH AREA PACING ON ELECTROCARDIOGRAPHIC INDICES OF REPOLARISATION

### **JYOTHI VIJAY**, ABHILASH SP, AJIT KUMAR VK, NARAYANAN NAMBOODIRI

#### Sree Chitra Tirunal Institute for Medical Sciences and Technology, Trivandrum, India

**Introduction:** There has been wider applicability of left bundle branch area pacing in the last few years. But there is meagre data on long‐term outcomes of this technique from this region. Also, the impact of the left bundle pacing on parameters on indices of cardiac repolarisation is also unknown.

**Methods:** This is a single‐centre experience on the effect of the left bundle branch area of pacing on repolarization parameters left bundle pacing and its short‐term and long‐term outcomes.

**Results:** Of a total of 70 subjects, 67 who underwent successful left bundle pacing were enrolled in the study. 49 patients underwent a left bundle branch area optimized pacemaker compared with 18 cases who underwent a left bundle branch area optimized cardiac resynchronization therapy (LOT CRT). The mean left bundle capture threshold at implant was 0.7 +/‐ 0.3 volts for the LBP group and 0.7 +/‐ 0.2 volts for the LOT CRT group. Mean LV activation time in milliseconds was 67+/‐ 13.6 milliseconds for the LBP group and 79.2 +/‐11.9 milliseconds for the LOT CRT group. There was no difference in final paced QRS duration at implant LOT CRT group that LBP group.116 vs 110.2 milliseconds. Selective LB pacing was associated with a lower‐paced QRS duration at implant. (108.9 vs 120.88 milliseconds). There was a significant reduction in QT interval, both corrected and uncorrected, QT dispersion and T peak‐T end interval post‐procedure in comparison to pre‐procedure values. The maximum follow‐up period was 10.7 +/‐8.3 months

**Conclusions:** Left bundle branch area pacing improves the electrocardiographic parameters of repolarization and provides narrow QRS, which is sustained on short term and long‐term follow‐up. Short‐term and long‐term pacing outcomes of left bundle branch area pacing from this single‐center study appear promising and comparable to results previously reported in the literature.

## INCESSANT ATRIAL TACHYCARDIA FROM TRICUSPID ANNULUS IN ENDOMYOCARDIAL FIBROSIS

### **JYOTHI VIJAY**, NARAYANAN NAMBOODIRI, ABHILASH SP

#### Sree Chitra Tirunal Institute for Medical Sciences and Technology, Trivandrum, India

**Introduction:** N/A

**Methods:** N/A

**Results:** A 52‐year‐old lady presented with recurrent episodic palpitations for past 12 months. She had two episodes of palpitations requiring hospital admissions. Upon physical exam, she was conscious, oriented.No pallor icterus cyanosis or clubbing, tachycardic. A cardiovascular exam showed soft S1, no murmurs. ECG showed narrow QRS tachycardia. A transthoracic echocardiogram was done, which showed obliteration of RV apex versus prominent moderator band with moderate TR and Good LV function. Tachycardia was mapped using an advisor HD grid, and the earliest ectopic atrial activation was found from the tricuspid annulus at 6’o'clock location. Upon a single burn, tachycardia was successfully terminated with RFA. A simultaneous RV ventriculography showed a right ventricular silhouette with an obliterated apex and linear calcific shadows at the “amputated” apex suggestive of endomyocardial fibrosis(EMF). Later, cardiac CT was done, confirming the diagnosis of RV endomyocardial fibrosis. A final diagnosis of focal atrial tachycardia from tricuspid annulus (6 ‘o'clock position) with underlying endomyocardial fibrosis was made, and successful RF ablation was done.

**Conclusions:** We need to have a high index of suspicion for structural heart disease in uncommon locations of AT. EMF is rarely encountered in EP labs, but making a correct diagnosis is paramount.

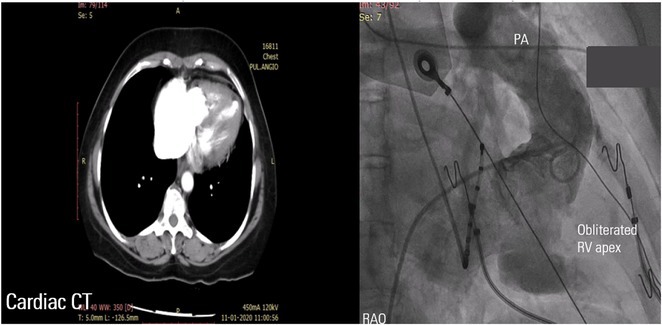

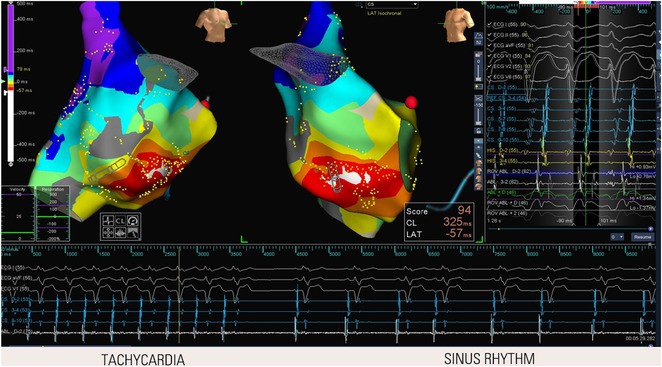

## SYNCOPE

### OAI VU

#### Vinmec International Hospital, Hanoi, Viet Nam

**Introduction:** Our hospital (Vinmec Times City Hospital) is one of two facilities in Viet nam with equipment to perform tilt table testing in diagnosis and directions for treatment of syncope. These are some special clinical cases.

**Methods:** N/A

**Results:** 1. A 20‐year‐old female patient was treated for epilepsy with medication for about 10 years, but fainting spells still occurred. The patient was referred to our hospital for evaluation and a tilt table test. The table test was positive for reflex syncope and during the syncope the patient showed signs of convulsions and shaking of the limbs similar to a syncope at home. The patient was advised to change lifestyle to control syncope and stop epilepsy medication treatment. After about 2 years, the patient no longer had any fainting spells. 2. A 56 year old male patient with a history of 3 syncopes, electrocardiogram result was type 2 Brugada and The patient had an EPS test that caused ventricular fibrillation when stimulated right ventricle with 4 pulses (S1: 400ms, S2: 240ms, S3: 220ms, S4: 220ms) patients were implanted with an ICD to prevent sudden death. After implantation, the patient still had fainting spells. The patient came to our clinic for examination. After programming the ICD, no arrhythmia was recorded. The patient had a tilt table test performed and was positive for reflex syncope with pre‐syncopal symptoms similar to the patient's previous syncope episodes. The patient was advised to change his lifestyle to control the fainting spells and after 1 year, no fainting spells occurred again. 3. A 59‐year‐old female patient has a history of repeated syncope. The patient went to many doctors and had most of the tests and assessments done but still could not find the cause. The patient came to our hospital for examination through a history of fainting episodes. The patient underwent a tilt table test and the results were positive for reflex syncope (Cardioinhibitory syncope with asystole) with an asystole lasting 5 seconds. The patient was implanted with a permanent pacemaker as well as advised on lifestyle changes to control syncope. Following the machine implantation, the patient's frequency of fainting spells was significantly reduced and the severity of symptoms was also milder.

**Conclusions:** N/a

## THE INJECTION SPEED AND VISIBILITY OF THE BRIDGE OCCLUSION BALLOON BASED ON CONTRAST AGENT DILUTION RATIOS

### AYUMI WAKITA

#### Ichinomiya Municipal Hospital, Ichinomiya, Japan

**Introduction:** Superior vena cava injury during lead extraction is an extremely rare but fatal complication. During the surgical intervention, a bridge occlusion balloon is an essential instrument for stabilizing the hemodynamic status. For safe and sure hemostasis, good visibility and rapid dilation of the bridge occlusion balloon at the bleeding site are required.The rapid dilation of the bridge occlusion balloon and its visibility under fluoroscopy are inversely related to the dilution ratio of the contrast agent. However, the optimal dilution ratio has yet to be fully determined. In this study, we investigated how contrast agent dilution ratios influence the injection speed and visibility of the bridge occlusion balloon.

**Methods:** Iopamidol 370 was used as a contrast agent with dilution ratios of 9.5:0.5, 9:1, 8:2, 7:3, 6:4, and 5:5 for *in‐vitro* studies using a cardiac model. For each dilution ratio, the injection speed and visibility were assessed five times. Imaging was conducted at 7.5 frames per second, and visibility was assessed by contrast‐to‐noise ratio (CNR).

**Results:** The average injection times from start to finish were 18.5 seconds, 20 seconds, 25.5 seconds, 25 seconds, 33 seconds, and 45 seconds for the dilution ratios of 9.5:0.5, 9:1, 8:2, 7:3, 6:4, and 5:5, respectively. For these same ratios, the respective CNR values were 2.9, 4.2, 10.5, 13.6, 16.3, and 18.0. As figure 1 showed, for this contrast agent, the optimal dilution ratio was 8:2.

**Conclusions:** The optimal dilution ratio for Iopamidol 370, when used in a bridge occlusion balloon, was found to be 8:2 when taking the balance between fluoroscopic visibility and injection speed into account. These findings correspond with the manufacturer's recommended dilution ratio.

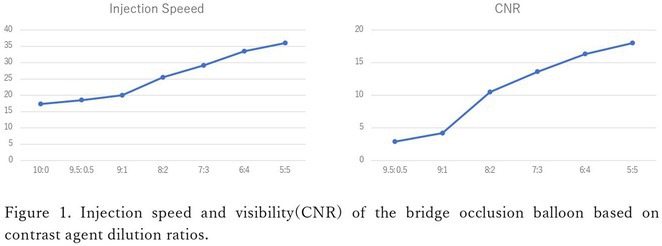

## SUBGROUP ANALYSIS OF CHADS_2_, HAS‐BLED, CHA_2_DS_2_‐VASC, AND CHA_2_DS_2_‐VA RISK SCORES ON CLINICAL OUTCOMES OF EDOXABAN THERAPY IN ATRIAL FIBRILLATION PATIENTS IN THE ETNA‐AF REGISTRY

### CHUN‐CHIEH WANG

#### Taipei and Linkou Chang Gung Memorial Hospital, On behalf of the Global ETNA Study Group, Taipei, Taiwan

**Introduction:** Current guidelines recommend oral anticoagulation (OAC) based on the CHA_2_DS_2_‐VASc score in atrial fibrillation (AF) patients with moderate or high stroke risk. Local cardiac societies also recommend some other thromboembolic risk scores.

**Methods:** The purpose of this study is to determine the impact of risk scores on clinical outcomes in AF patients treated with edoxaban using 2‐year data from global ETNA‐AF (group 1) and 4‐Asian region (Korea, Taiwan, Hong Kong, and Thailand) (group 2) cohorts. This post hoc study analyzed patient distribution based on CHADS_2_, modified HAS‐BLED, CHA_2_DS_2_‐VASc, and CHA_2_DS_2_‐VA risk scores in AF patients treated with edoxaban for stroke prevention. Also, 2‐year clinical outcomes were compared between group 1 (N=26,805) and group 2 (N=3,299) based on risk categories of different scores.

**Results:** The percentages of low (0), moderate (1‐2), and high (≥3) risks in groups 1 vs. 2 were 9.3, 61.6, and 29.1% vs. 9.9, 62.0, and 28.1% by CHADS_2_ categories, and 2.6, 36.2, 61.2% vs. 2.7, 37.8, and 59.5% by modified HAS‐BLED categories. The percentages of low (0‐1), moderate (2‐3), and high (≥4) risks in groups 1 vs. 2 were 11.4, 45.9, 42.8% vs. 11.2, 50.4, and 38.4% by CHA_2_DS_2_‐VASc categories, and 16.1, 54.8, and 29.2% vs. 17.2, 56.4, and 26.4% by CHA_2_DS_2_‐VA categories. Comparison of the 2‐year effectiveness and safety outcomes between groups 1 and 2 by CHADS_2_, HAS‐BLED, CHA_2_DS_2_‐VASc, and CHA_2_DS_2_‐VA categories showed that all‐cause mortality, cardiovascular mortality, and clinically relevant non‐major bleeding were higher in group 1 cohort. In contrast, any stroke and ischemic stroke were higher in the group 2 cohort.

**Conclusions:** According to the ETNA‐AF registry post hoc study, CHA_2_DS_2_‐VASc and CHA_2_DS_2_‐VA scores can help identify low thromboembolic risk patients under Edoxaban treatment. All four risk scores provide practical tools to assess the individual bleeding risk in real‐world AF patients treated with edoxaban.

## A NEW STRATEGY OF ONE‐STAGE LEFT UNILATERAL THORACOSCOPIC EPICARDIAL AND TRANSCATHETER ENDOCARDIAL ABLATION FOR PERSISTENT ATRIAL FIBRILLATION

### SHENGWEN YANG, **YANJING WANG**

#### Heart Center & Beijing Key Laboratory of Hypertension, Beijing Chaoyang Hospital, Capital Medical University, Beijing, China

**Introduction:** Long‐term success rates for catheter ablation (CA) in treating long‐standing persistent atrial fibrillation (LSPAF) are not satisfactory, demanding improved ablation methods to enhance treatment outcomes.This study aimed to compare the safety and effectiveness of a new single‐chest approach with the traditional bilateral chest wall approach for patients undergoing combined endo‐epicardial atrial fibrillation ablation surgery.

**Methods:** The study enrolled patients planned for combined ablation surgery at Beijing Chaoyang Hospital from July 2016 to August 2022. Two approaches were compared: the traditional bilateral thoracoscopic approach used from July 2016 to November 2018, and a new single left‐sided thoracoscopic approach used from March 2021 to August 2022.

**Results:** The study reported outcomes for 41 patients in the single left‐sided thoracic approach group and 15 in the bilateral thoracic approach group. The single‐access approach significantly reduced total surgery time, hospital stay, and ICU duration. At a follow‐up of 24.0±4.1 months, the single‐access group maintained a 78.0% sinus rhythm, while the double‐access group had an 80.0% rate after 57.1±10.7 months.

**Conclusions:** The novel unilateral thoracic approach for combined ablation significantly reduces surgery time, hospital stay, and complications, maintaining a similar success rate to the traditional bilateral approach. Despite its single‐center nature and other limitations, this approach simplifies the complexity of surgical ablation and may facilitate broader adoption in more hospitals.

## MULTI‐DIMENSIONAL VALUE OF THE SMARTTOUCH SURROUNDFLOW (STSF) CATHETER: A HEALTH TECHNOLOGY ASSESSMENT IN CHINA

### ZIDUN WANG

#### The First Affiliated Hospital with Nanjing Medical University, Nanjing, China

**Introduction:** The aim of this study was to assess the multi‐dimensional value of the SmartTouch SurroundFlow (STSF) catheter, an advanced iteration of the SmartTouch (ST) catheter, designed for radiofrequency catheter ablation (RFCA) in patients diagnosed with atrial fibrillation (AF), from a perspective of the healthcare system in China.

**Methods:** The present study enrolled 47 patients with AF who underwent RFCA between May and September 2023 in two hospitals in China. The clinical value of two catheters was assessed from safety and efficiency of procedures. The Visual Analog Scale (VAS) score was used to assess the level of intraoperative pain for patients. The Ablation Index Follow‐up Viewer (AIFV) was applied to evaluate the quality of operation by analyzing the raw data obtained from tracking the procedural path of physicians. Additionally, a decision tree model was developed to evaluate the cost‐effectiveness of STSF catheter. The incremental cost‐effectiveness ratio (ICER) was used as the result index of economic analysis.

**Results:** The findings indicated that the utilization of STSF catheter can optimize procedural efficiency for physicians while minimizing patients' pain perception. The incremental cost US$ 682.90 of the STSF catheter resulted in a gain of 0.019 quality‐adjusted life years (QALYs), leading to an ICER of US$ 36,466.44 per QALY.

**Conclusions:** The findings of this study suggests that STSF is probably a preferred choice for physicians and AF patients. Our studyalso concludes that a 5% discount on the existing cost of STSF would be more cost‐effective for decision‐makers in the price setting.

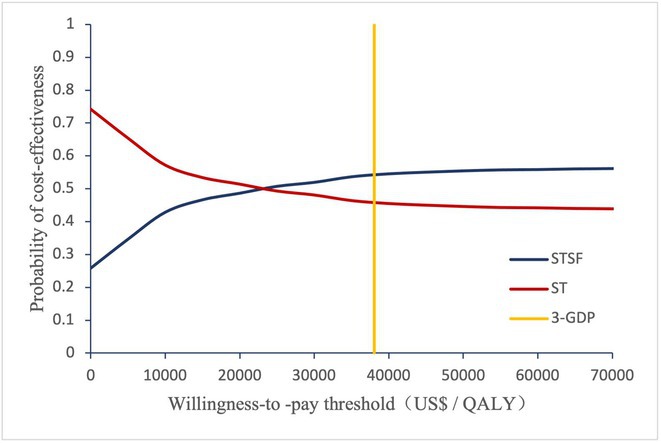

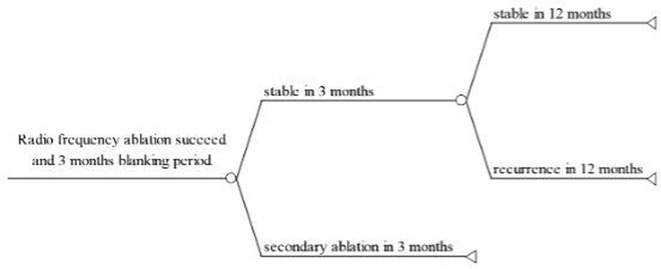

## CLINICAL IMPLICATION OF CARTO‐FINDER IN ATRIAL FIBRILLATION PATIENTS WITH PULMONARY ISOLATION

### **ATSUYUKI WATANABE**, TOMONARI KIMURA

#### Okayama Medical Center, Okayama, Japan

**Introduction:** Pulmonary vein isolation (PVI) is effective and well‐established method of ablation in atrial fibrillation (AF) patients. The CARTO‐FINDER&reg; (CF) may suggest the mechanism of AF, But the clinical implication of CF is unknown in AF patients underwent PVI.

**Methods:** We evaluated the prognosis of AF ablation using CF during PVI. The total 110 patients were attempted the PVI using CF. (paroxysmal AF(PAF): 44 patients, persistent AF(PeAF): 66 patients). We performed evaluation of the CF at bilateral PV antrum during induced or sustained AF and performed CF‐guided PVI.

**Results:** After 1 year follow up, 94 of 110 (85.4%) patients performed CF‐guided PVI were free from AF recurrence. Figure 1 shows the Kaplan‐Meier for freedom from AF recurrence (non CF vs CF). No CF findings at PV antrum suggested the excellent prognosis in PAF patients, on the other hand, the very poor prognosis in persistent AF patients.

**Conclusions:** CARTO‐FINDER&reg; during AF may be strong predictor of prognosis in AF patients performed PVI.

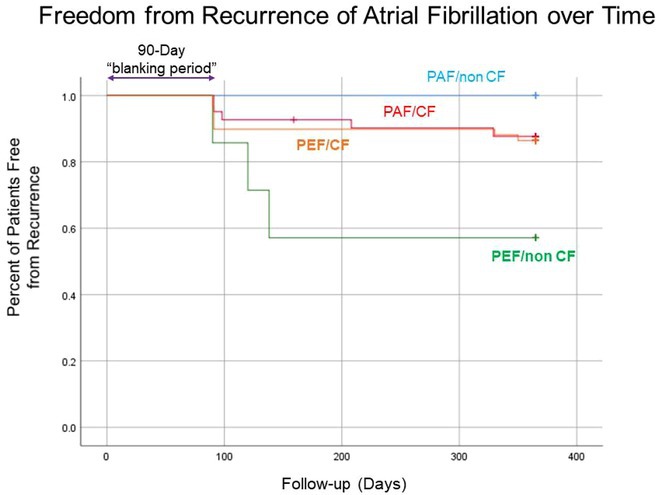

## CARDIAC RHYTHM CLASSIFICATION IN 12‐LEAD ELECTROCARDIOGRAMS USING A DEEP NEURAL NETWORK

### **EIICHI WATANABE** ^1^, YOSHIHIRO SOBUE^1^, KAZUYA TAKEDA^2^

#### ^1^Fujita Health University School of Medicine, Nagoya, Japan,^2^Fujita Health University Bantane Hospital, Nagoya, Japan

**Introduction:** Conventional, algorithm‐based automated ECG interpretation has limited diagnostic accuracy. The widespread availability of digital ECG data, combined with advances in deep neural networks, offers a significant opportunity to improve the accuracy of automated ECG analysis.

**Methods:** We developed a deep neural network (DNN) to classify six rhythm classes: sinus rhythm, atrial fibrillation (AF), cardiac pacemaker (PM), atrial premature contraction (APC), premature ventricular contraction (PVC), and paroxysmal supraventricular tachycardia (PSVT). The model was trained using 540 12‐lead ECGs (sampling 500 Hz,10 sec) and externally validated with 11,817 12‐lead ECGs from the PhysioNet database.

**Results:** We created a recurrence plot of 5000×5000 and down sampled it to 512×512. The following metrics were calculated (macro precision and macro recall, macro F1, accuracy) and results of external validation were presented in Table. The recall and precision of PM in internal validation were 0.790 and 0.822.

**Conclusions:** Our DNN demonstrates high diagnostic performance in sinus rhythm, PSVT, PM, and AF, but shows lower accuracy in detecting APC and PVC. Further studies are needed to determine if combining our DNN with algorithm‐based ECG interpretation can reduce the misdiagnosis rate.

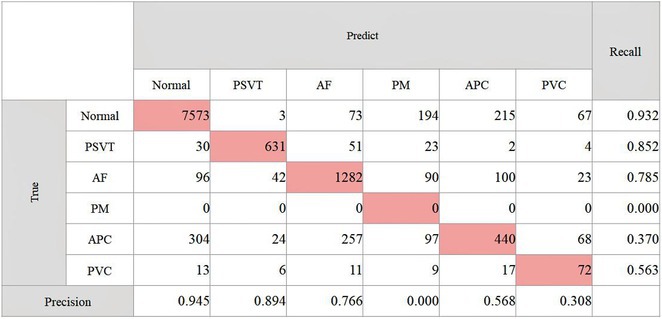

## INTRAVENOUS VAGAL STIMULATION CATHETER INDUCES RAPID HEART RATE REDUCTION WITHOUT HEMODYNAMIC COMPROMISE IN A DOG MODEL OF ATRIAL FIBRILLATION WITH SEVERE TACHYCARDIA

### **TSUKASA WATANABE** ^1^, KAZUO SAKAMOTO^1^, HIDETAKA MORITA^2^, HIROTAKE YOKOYAMA^1^, TOMOMI NAGAYAMA^1^, SUSUMU TAKASE^1^, KEITA SAKU^2^, KOHTARO ABE^1^

#### ^1^Department of Cardiovascular Medicine, Kyushu University, Fukuoka, Japan,^2^Department of Cardiovascular Dynamics, National Cerebral and Cardiovascular Center, Osaka, Japan

**Introduction:** Background:In patients with heart failure, the occurrence of atrial fibrillation (AF) with tachycardia often worsens their condition. While appropriate heart rate (HR) control is crucial for these patients, commonly used bradycardic agents in clinical practice can exacerbate heart failure due to their negative inotropic effects. In a normal physiology, the vagal nerve strictly regulates HR to maintain hemodynamic homeostasis. Furthermore, vagal nerve stimulation is known to rapidly lower HR. We have recently developed a basket‐shaped intravenous catheter device to stimulate the vagal nerve in the thoracic region. This study investigated the feasibility of the intravenous vagal nerve stimulation (iVNS) catheter to control HR in a dog model of AF tachycardia. Purpose:We aimed to assess the impact of the iVNS catheter on HR and hemodynamics during AF tachycardia.

**Methods:** In three beagle dogs (10.3±0.5 kg), we inserted the basket‐shaped iVNS catheter from the right femoral vein, positioning it at the level of the superior vena cava. Stimulation intensity was adjusted to 10‐20 V (20 Hz). Electrocardiogram and arterial pressure (AP) were continuously recorded. Atrial fibrillation was then induced by atrial high‐frequency stimulation(cycle length:50 msec) under ISP administration. In two of the three dogs, we also recorded ascending aortic flow. We compared HR and hemodynamics between baseline and one minutes after iVNS during the AF condition.

**Results:** iVNS rapidly altered HR, reaching a steady state within seconds of stimulation. iVNS significantly reduced HR (baseline: 244±5.2 vs. stimulation: 215±24 bpm, p=0.04) without significant changes in AP (40±7 vs 47±14 mmHg, p= 0.50) or cardiac output (1.97±0.03 vs 1.89±0.06 ml/min, p=0.12). HR promptly returned to baseline after iVNS cessation in all dogs.

**Conclusions:** Vagal nerve stimulation induced a reduction in HR during the AF condition. Our minimally invasive iVNS catheter offers immediate and adjustable bradycardia, making it a potential therapeutic option for patients of AF with severe tachycardia.

## IMPLANTABLE CARDIOVERTER DEFIBRILLATOR IMPLANTATION IN HYPERTROPIC CARDIOMYOPATHY PATIENT : DO CLINICAL FEATURES NEED TO BE CONSIDERED?

### SETIAWAN WIDODO

#### RSUD Karawang, Karawang, Indonesia

**Introduction:** Hypertrophic cardiomyopathy (HCM) is a common genetic condition with various complications such as SCD, atrial fibrillation (AF) and heart failure (HF). Early detection and ICD intervention has reduced mortality rate. Risk stratification and targeting candidates ICD can be complex due to clinical scenarios, arrhythmogenic substrate and a risk factor.

**Methods:** N/A

**Results:** A young man with HF was visited to clinic with history of cardioversion from VA. ECG showed atrial flutter and poor R wave progression. Echocardiography showed suggestive HCM and reduced EF. An MRI examination showed prominent focal patcy and fibrosis consistent of HCM. Scar burden 44,5%. Genetic testing revealed positive pathogenic variant identified in MYH7. The ICD was implanted. During procedur, patient suffered VA, ICD overdrive pacing successfully convert into sinus. Amiodarone drip was given. During hospitalization, he got a shock. Interogate showed episodic VA that recognize as ventricular fibrillation. A shock was delivered without prior ATP. Tram tracking sign was observed. The day after, he was urgently transferred to Intensive Care because unstable hemodinamik. Norephinephrine was administered. Patient experienced PEA and was declared dead. The earlier onset,and worse outcomes common in sarcomeric mutation patients. HCM with reduced EF will develop into refractory HF and myocardial scar. Fibrosis greater than 15% was associated with risk of SCD. Patient with AF also had a higher risk of mortality.

**Conclusions:** Patient selection, clinical features, arrhytmia, high electrical voltage to be considered in ICD management.

## LEADLESS PACEMAKER IMPLANTATION IN HAEMODIALYSIS PATIENTS: A SINGLE TERTIARY CENTRE'S EXPERIENCE IN REGIONAL NORTH QUEENSLAND AUSTRALIA

### **NETHMI WIJESEKERA**, AMARDEEP BISHNOI, YUNN YIING NG, BOBBY JOHN

#### Townsville University Hospital, Townsville, Australia

**Introduction:** Haemodialysis patients are a high‐risk group for conventional transvenous pacing; due to the predilection for lead‐related central venous stenosis and systemic infection. Leadless pacemakers have been proposed as a viable and attractive alternative for this group of patients.

**Methods:** A retrospective analysis was conducted of haemodialysis patients who had undergone leadless pacemaker (Micra) implantation at a tertiary centre in regional North Queensland in Australia between January 2019 and February 2024. The objective was to report on periprocedural and long‐term outcomes post‐implantation.

**Results:** Of a total of 72 implantations, 21 were implanted in haemodialysis patients. The age of patients varied from 51 to 87 years of age with a median age of 68 and male predominance (63%). Two were renal transplant patients with graft failure. Five patients were post cardiothoracic surgery. Ten patients had a history of or had a concurrent severe infection at the time of implantation. Concomitant atrial fibrillation and type II diabetes were present in fourteen and thirteen patients respectively. At implantation, the median R wave was 9.8mV, the median threshold was 0.6mV@0.24ms; and the median impedence was 880ohms. There were no periprocedural complications including procedure‐related deaths, vascular access site complications, rupture or pericardial effusion. One patient died 48 hours post‐device following a myocardial infarction. No device‐related infections were evident. Twelve patients were discharged within 48 hours of procedure, whilst three patients had prolonged hospital admissions (>1 month, not procedure‐related). Four patients were rehospitalized within 1 month due to non‐procedure related indications. The 1‐year mortality rate was 23.8%.

**Conclusions:** Our experience demonstrates that leadless pacemakers are an effective pacing option with a good safety profile in haemodialysis patients. These patients inherently have a guarded prognosis, and judicious implantation of devices is warranted in this group.

## SUCCESSFUL BAIL‐OUT OF A MASSIVE AIR EMBOLISM COMPLICATING CRYOBALLOON AF ABLATION

### **NETHMI WIJESEKERA**, BOBBY JOHN

#### Townsville University Hospital, Townsville, Australia

**Introduction:** Atrial fibrillation (AF) is the most common cardiac arrhythmia. Pulmonary vein isolation has been deemed appropriate for those intolerant or refractory to drug therapy. There are several rare, but serious complications including air embolism associated with AF ablation. We describe a case of successful bail‐out of a massive air embolism with urgent hyperbaric oxygen therapy without any neurological sequelae.

**Methods:** NA

**Results:** A 74‐year‐old man with a background of longstanding persistent atrial fibrillation, presented for a cryoballoon ablation procedure. He had failed medical therapy with flecainide: with ongoing symptoms of fatigue and lethargy. Routine trans‐septal puncture was performed with fluoroscopic, pressure and intracardiac echocardiographic guidance. A 28mm *cryoballoon* was advanced over 20mm *Achieve* mapping catheter through the *Flexcath* catheter into left superior pulmonary vein. *ACIST* automated power injection system was used for delivery of contrast to assess the position of the balloon. Malfunction of the system resulted in inadvertent injection of approximately 5mL of air into the left atrium. Immediate aspiration was attempted with autologous transfusion via femoral vein access and Trendelenburg position was assumed. Subsequently the patient developed haemodynamically unstable ventricular tachycardia, which reverted to sinus rhythm with a single 200J shock. There were no ischemic changes noted on ECG. The patient was ventilated with 100% FiO2 and underwent emergent hyperbaric oxygen therapy for 8 hours. CT brain did not demonstrate serpiginous hypodensities in the cerebral vasculature. He was monitored in ICU and did not incur any cerebral, coronary, gut, or limb ischaemic injury. He was followed up at 6 weeks with no neurological sequelae and absence of barotrauma to tympanic membrane. Echocardiogram confirmed preserved left ventricular systolic function without regional wall motion changes.

**Conclusions:** This case highlights the role of early recognition and emergent hyperbaric oxygen therapy for the successful treatment of massive air embolism incurred during AF ablation.

## CLINICAL IMPACT OF POST‐OPERATIVE ATRIAL FIBRILLATION FOLLOWING CARDIAC SURGERY: A 14 YEAR SINGLE‐CENTER EXPERIENCE

### **JEREMY WILLIAM**, JOSEPH HOGARTY, SANDEEP PRABHU, SILVANA MARASCO, PETER KISTLER, ALEKSANDR VOSKOBOINIK

#### Alfred Health, Melbourne, Australia

**Introduction:** Post‐operative atrial fibrillation (POAF) is common after cardiac surgery. We sought to investigate the relationship between POAF and subsequent clinical outcomes in patients undergoing cardiac surgery.

**Methods:** We performed a single center retrospective review of all patients undergoing cardiac surgery between 2010‐2023. We analyzed patients with early POAF, defined as new‐onset AF occurring within 30 days of surgery. Patients with a history of paroxysmal or persistent AF pre‐dating surgery were excluded.

**Results:** Of 7310 patients evaluated, POAF occurred in 2257 (30.9%). Patients experiencing POAF were older (66.7±13.6 vs 61.0±11.0 years, p<0.001) and more likely to be male (80.3% vs 76.4%, p=0.02). POAF was more frequently encountered in patients undergoing valve surgery compared to those undergoing coronary artery bypass graft (CABG) surgery (36.5% vs 31.4%, p=0.01). POAF resulted in longer ICU length of stay (126.2 hours vs 100.7 hours, p<0.001) and total post‐operative length of stay (16.0 days vs 13.9 days, p<0.001). POAF was associated with higher risk of peri‐operative stroke, major hemorrhage, new severe renal impairment, ICU re‐admission and re‐intubation (Figure). Overall rates of mortality and hospital re‐admission at 30‐days post‐operatively were not significantly higher in patients with POAF.

**Conclusions:** Post‐operative AF significantly increases length of stay and rates of in‐hospital complications after cardiac surgery. Interventions aimed at preventing peri‐operative AF may reduce these adverse outcomes and require further study.

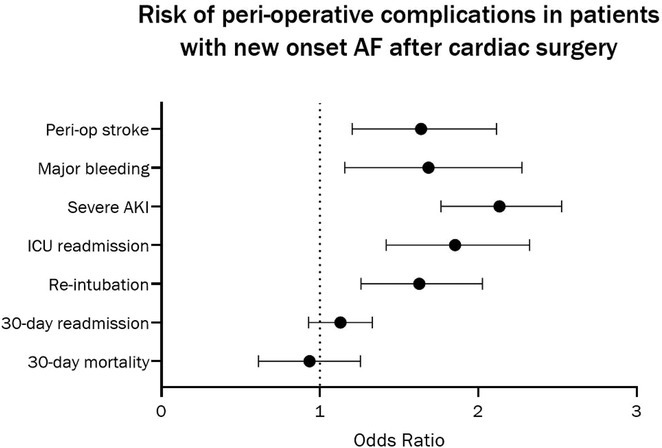

## INCIDENCE AND PREDICTORS OF LATE ATRIAL FIBRILLATION RECURRENCE AFTER CARDIAC SURGERY

### **JEREMY WILLIAM**, JOSEPH HOGARTY, KATE ROWE, XIAOMAN XIAO, SANDEEP PRABHU, SILVANA MARASCO, PETER KISTLER, ALEKSANDR VOSKOBOINIK

#### Alfred Health, Alfred Health, Australia

**Introduction:** While post‐operative atrial fibrillation (POAF) frequently occurs early after cardiac surgery, there is a paucity of data evaluating predictors and timing of late AF recurrence.

**Methods:** We performed a retrospective cohort study of all patients who underwent cardiac surgery at our institution from 2010‐2018. We included patients without a history of AF pre‐dating cardiac surgery who had at least 2 years of clinical follow‐up post‐surgery. We recorded the incidence and predictors of late AF recurrence, defined as occurring ≥12 months following surgery.

**Results:** 1017 patients were included (mean age at surgery 64±12 years, 74% male). Early POAF was recorded in 445 patients (43%). POAF was usually transient, with total AF duration <48 hours in 72%. At 4.7±2.4 years follow‐up, late AF occurred in 139 patients (14%). Median time to AF recurrence was 4.4 years post‐surgery (IQR 2.6‐6.2 years). Late AF was significantly more likely amongst patients with early POAF than those without (23% vs 6%, p<0.001), with greatest risk in those with POAF duration >48 hours (graphic). In a multivariable analysis, early POAF duration >48h was a significant predictor of late AF recurrence (HR 5.9). Surgery type, LV ejection fraction, left atrial size and CHADS‐VASc score were not predictive of late AF recurrence.

**Conclusions:** Post‐operative AF episodes of duration ≥48 hours predict recurrent AF episodes over long‐term follow‐up after cardiac surgery. Implications for arrhythmia surveillance and anticoagulation in patients with longer duration POAF episodes requires further study.

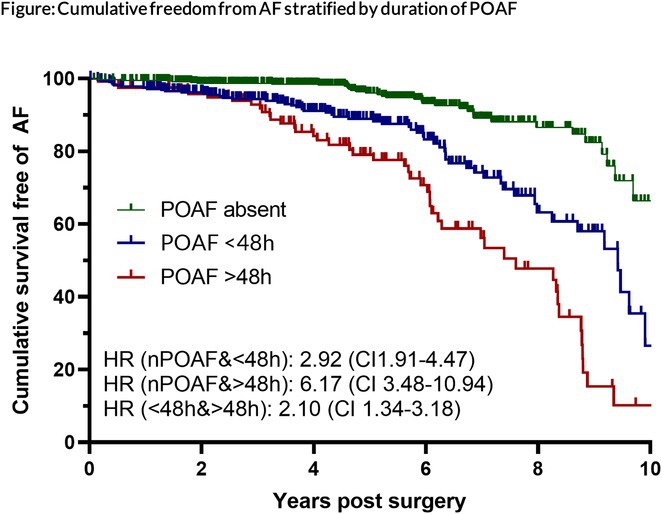

## UNMASKING THE RARE SLOW‐SLOW AVNRT: A CASE REPORT OF DIAGNOSTIC CHALLENGES AND SUCCESSFUL ABLATION

### **SETIAWAN WINARSO**, MUZAKKIR AMIR, SUMARNI WAHYUDI

#### Hasanuddin University, Makassar, Indonesia

**Introduction:** Slow‐slow AVNRT, a variant of the rarest AVNRTsubtypes, occurs by retrograde conduction via slow atrial fibers andanterograde conduction via slow AV nodal pathways. Certain characteristics ofthis mechanism can make diagnosis difficult because they resemble other typesof tachycardia on the ECG. Nonetheless, slow‐slow AVNRT can be reliablyidentified by sophisticated electrocardiography (ECG) analysis andelectrophysiological study (EPS) testing, offering vital information for bothdiagnosis and treatment.

**Methods:** N/A

**Results:** A 40‐year‐oldwoman presented with a seven‐year history of palpitations as her only symptom.A Holter monitor revealed a low burden of premature atrial contractions (PAC) and premature ventricular contractions (PVC). Subsequently, anelectrophysiology (EP) study was performed. Atrial stimulation showed an AHjump and induced a narrow complex tachycardia (TCL 390 ms, VA 129 ms, AV 216ms) with 1:1 AV conduction. The his‐refractory premature ventricular beat (HRPVB) maneuver failed to reset the tachycardia, while right ventricular overdrivepacing (RVOP) revealed a V‐A‐V response (PPI ‐ TCL difference 147 ms). Thefindings indicated atypical slow‐slow AVNRT, and successful radiofrequencyablation (RFA) followed after slow pathway mapping.

**Conclusions:** The challenge in identifying atypical AVNRT aredemonstrated by this example. Although preliminary results seemed normal,electrophysiological investigations validated the diagnosis and provideperspective on the arrhythmia mechanism. It emphasizes how crucial a thoroughelectrophysiological assessment is to the management of supraventriculararrhythmias.

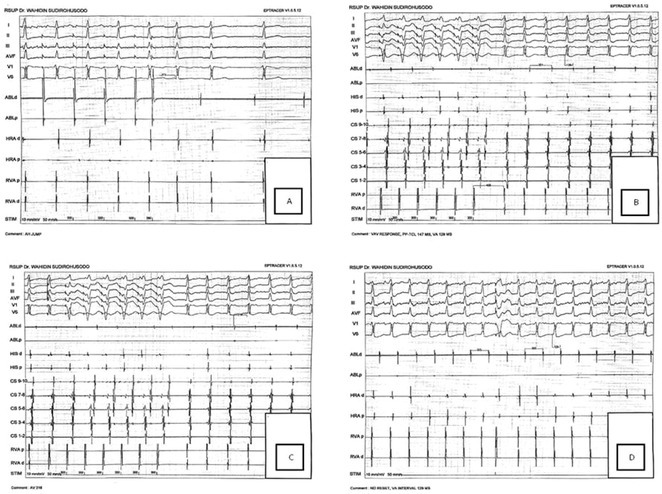

REAL WORLD SAFETY PROFILE OF EDOXABAN WITH CONCOMITANT DRUG INTERACTIONS

**CHUN KA WONG**, YUEN‐KWUN WONG, MINQING LIN, ALI CHOO, HUNG‐FAT TSE

The University of Hong Kong, Hong Kong, Hong Kong

**Introduction:** Real‐world data are needed on the safety profile of direct oral anticoagulant (DOAC), such as edoxaban, when used with interacting medications.

**Methods:** A retrospective cohort study was conducted involving all patients who received edoxaban in public hospitals in Hong Kong to assess bleeding outcomes. Medication with potential drug‐drug interaction were analyzed, including antiarrhythmics (amiodarone, digoxin, diltiazem, dronedarone, and verapamil), antidepressants, antimicrobials, and statin. The primary outcome was hospitalization for major bleeding, which included intracranial hemorrhage and gastrointestinal bleeding.

**Results:** From January 2017 to December 2020, 774 patients (age 75.6±11.0 years; 48.0% female) were prescribed edoxaban for atrial fibrillation. The mean CHA2DS2‐VASc score was 2.8±1.7. Among the medications with potential drug‐drug interactions analyzed, atorvastatin (29%), diltiazem (16%), digoxin (15%), amiodarone (9.0%), and antidepressants (7.0%) were most commonly prescribed. At one year, hospitalization for major bleeding occurred in 35 patients (5.0%), including intracranial hemorrhage in 7 (1.0%) and hospitalization for gastrointestinal bleeding in 29 (4.0%). Among all medications analyzed, only clarithromycin was associated with a higher risk of hospitalization for bleeding (adjusted hazard ratio 37.1, p‐value 0.001*) and hospitalization for gastrointestinal bleeding (adjusted hazard ratio 49.6, p‐value 0.001*). No other analyzed medication was associated with an increased risk of bleeding.

**Conclusions:** Concomitant use of edoxaban with clarithromycin was associated with a higher bleeding risk. Otherwise, no increased bleeding risk was observed in a real‐world setting when edoxaban was used concurrently with other potentially interacting medications.

## ASSOCIATION OF SERUM TNFSF14/LIGHT CONCENTRATIONS WITH ATRIAL FIBRILLATION

### **YIRONG WU**, MINGRUI SUN

#### Affiliated Hangzhou First People's Hospital, WestLake University School of Medicine, Hangzhou, China

**Introduction:** Tumour necrosis factor superfamily protein 14 (TNFSF14), also called LIGHT, is an important regulator of immunological and fibrosis diseases. Our study was aimed at clarifying whether serum LIGHT concentration correlate with atrial fibrillation(AF).

**Methods:** 161 individuals were recruited at the Affiliated Hangzhou First People's Hospital, Westlake University School of Medicine, including 110 AF patients and 51 healthy controls matched by body mass index and gender.The serum LIGHT levels of the two groups were measured, and relevant clinical data were collected for statistical analysis.

**Results:** Compared with the healthy controls, the serum LIGHT concentration was significantly higher in AF patients (P < 0.001) and the serum LIGHT concentration was further elevated in persistent AF patients compared to proxymal AF (P < 0.001). Serum LIGHT concentration were positively correlated with AF types (P < 0.001) and the severity of AF (CCS‐SAF) (P < 0.05) according to linear regression. Dichotomous logistic regression analysis found serum LIGHT concentration and age to be independent risk factors for AF (OR = 9.245, 95% CI: 2.648‐32.274; OR = 1.150, 95% CI:1.004‐1.665, respectively). The area under the curve(AUC) of LIGHT in AF was 0.844 (95% CI: 1.090‐1.213).The cut‐off value of LIGHT is was 29.548 ng/ml,with a sensitivity of 69.6%,and specificity of 86.3%. Further Diagnostic evaluation based on AF types revealed that serum LIGHT concentration present more superior diagnostic value in persistent AF with AUC was 0.737(95%CI:0.630‐0.844) than proxymal AF with AUC was 0.917(95%CI:0.867‐0.970).

**Conclusions:** In this study, we provide evidence that serum LIGHT concentration are elevated in patients with AF and correlate with symptom severity and AF types. In addition, LIGHT demonstrated satisfactory diagnostic performance in recognizing AF typically in persistent AF. These findings suggest that LIGHT may serve as a potential biomarker for AF.

## LEFT BUNDLE BRANCH AREA PACING FROM THE ILIAC APPROACH IN A PATIENT WITHOUT SUPERIOR ACCESS

### **LEI XU** ^1^, SHENGMEI QIN^2^, YANGANG SU^2^

#### ^1^Shanghai Institute of Cardiovascular Diseases, Zhongshan Hospital, Fudan University, Shanghai, China,^2^Shanghai Institute of Cardiovascular Diseases, Shanghai, China

**Introduction:** Left bundle branch area pacing (LBBAP), showing better effect on ventricular electrical and mechanical synchrony compared with right ventricular pacing, has been demonstrated the safety and feasibility of operation through superior access. In this report, we detail a case of LBBAP through the iliac approach.

**Methods:** An 89‐year‐old female, with a history of sinus arrest after AF, was referred to our hospital for management. The patient underwent pacemaker removal due to a pocket infection in the right subclavian area, necessitating tricuspid valvuloplasty for infective endocarditis. Venography revealed occlusion in the left brachiocephalic vein. Subsequently, LBBAP pacemaker implantation via the iliac vein was performed. Attempting Medtronic Attain Select II 6248V‐130p as the inner sheath, we guided the outer sheath (Medtronic Attain Command 6250V‐MB2X) into the right ventricle. The inner sheath was then positioned anterior and inferior to the His bundle, perpendicular to the ventricular septum. The 3830 lead was inserted along the inner sheath, and for increased usable lead length, the hemostatic valve was removed, and 12 cm of the proximal end of the inner sheath was cut. The lead was screwed into the right ventricular septum until the unipolar QRS complex morphology exhibited an incomplete right bundle branch block pattern in V1, with a width of 103 ms and an LV activation time of 69 ms in V6 at a low output.

**Results:** The pacing threshold was 1.0V at 0.4 ms, sensing was 11 mV, and impedance was 880 Ω. The generator was implanted in the lower‐right abdominal pocket. The post‐operative ECG revealed a QRS duration of 110 ms. During the six‐month follow‐up after the pacemaker implantation, the patient exhibited mobility without lower limb symptoms or edema. The pacing system continued to function properly.

**Conclusions:** The feasibility of iliac vein access for LBBAP implantation is highlighted, demonstrating good stability and offering a practical alternative. This approach mitigates unnecessary risks associated with thoracotomy and epicardial lead placement, providing a safer and effective option for cardiac pacing.

## INTRAVENOUS LIPID EMULSION THERAPY FOR CARDIAC ARREST FROM DRUG OVERDOSE IN A NEWLY DIAGNOSED BRUGADA SYNDROME

### FAROOK AHMAD^1^, **EMMA YAAKOP** ^2^, MUHAMMAD ATHAR SADDIQ^3^, STEPHEN COX^4^

#### ^1^Colchester General Hospital, Colchester, United Kingdom,^2^Hospital Sultan Idris Shah, Kajang, Malaysia,^3^Sultan Qaboos University Hospital, Muscat, Oman,^4^HeartCare Partners, Department of Cardiology, Brisbane, Australia

**Introduction:** Antidepressants, antipsychotics and antihypertensives overdose requiring resuscitation, stabilization and subsequent treatment are common presentation to the Intensive Care Unit. Current treatment options are limited and no antidote available. ILE is an effective treatment cardiorespiratory collapse associated with lipid soluble antidepressants, antipsychotics and antihypertensives, as they may have the capacity to bind lipophilic drugs present at toxic concentrations. In this case study, we described the successful resuscitation via ILE of a patient with a newly diagnosed BrS in cardiac arrest from polypharmacy overdose.

**Methods:** N/A

**Results:** We describe the initial management, resuscitation and subsequent recovery of a newly diagnosed Brugada syndrome (BrS) in a 29‐year‐old male following a potentially fatal overdose of amitriptyline, quetiapine, propranolol, nifedipine and pregabalin. Intravenous Intralipid® (ILE) was given as soon as after initiation of basic resuscitation as he remains in cardiogenic shock and refractory ventricular fibrillation. There was a rapid improvement in the patient's hemodynamic status after ILE administration. In the intensive Care unit, he suffered another episode of refractory ventricular fibrillation after extubation and he was administered ILE again. He recovered well and had Implantable cardioverter defibrillator (ICD) implanted prior to discharge. This adds further evidence for a genuine effect and efficacy of ILE. In this case, the lipid sink theory best explain the significant improvement in clinical and hemodynamic status as well as relapse in symptoms likely of amitriptyline, propranolol and nifedipine overdose.

**Conclusions:**

We believe this is the first case of management of electrical storm with ILE in a newly diagnosed Brugada syndrome. While the mechanism of action of ILE in treating lipophilic and cardiac drug toxicity remains unclear, it was effective in the treatment of his electrical storm and thus, unmasking the underlying Brugada syndrome.

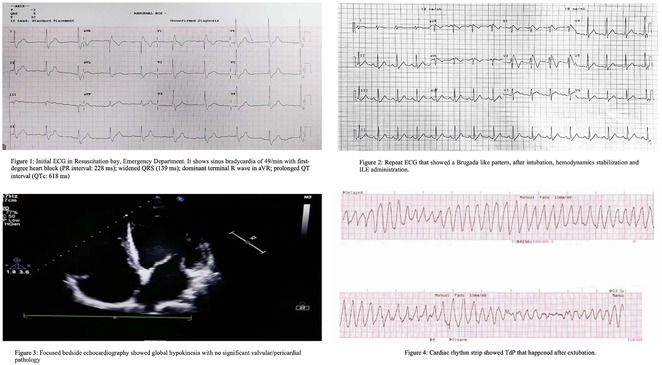

## SERUM CHITOTRIOSIDASE IN CARDIAC SARCOIDOSIS

### **VIJAY YADAV** ^1^, SANTOSH TIMALSINA^2^, MUTHIAH SUBRAMANIAN^1^, GUJJARLAPUDI DEEPIKA^1^, JUGAL KISHORE^1^, CALAMBUR NARASIMHAN^1^

#### ^1^AIG Hospitals, Hyderabad, India,^2^Chitwan Medical College, Chitwan, Nepal

**Introduction:** Cardiac sarcoidosis (CS) is a disease of granulomatous myocardial inflammation with progressive fibrotic changes. It can present as unexplained AV blocks, heart failure, ventricular tachycardia and sudden cardiac death. We evaluated the role of serum Chitotriosidase (CHT) to diagnose cardiac sarcoidosis.

**Methods:** Sixty‐five subject‐patients with suspected CS were prospectively recruited in the study. Demographic data, baseline CHT level, lymph node (LN), and myocardial uptake including SUVmax were collected from the Ga‐68 DOTA‐NOC PET‐CT scan. Spearman's rank correlation was performed to identify the correlation between variables. Comparison of medians between groups was performed by Mann Whitney U test or Kruskal Wallis H test as appropriate. P<0.05 was considered statistically significant.

**Results:** The mean age of the study population with CS (n = 65) was 50.2 ± 15.5 years (26 males and 39 females). The median baseline CHT level was 456.6 pg/ml (normal value is < 80 pg/ml). Both the LN and the myocardial uptake were observed in 25 (38.5%) patients. In this group of patients, there was a moderate correlation (Spearman's rho = 0.64, P=0.001) between lymph node SUVmax and myocardial SUVmax with their median values 3.2 and 3.4, respectively. Baseline CHT level was found to be significantly correlated with myocardial SUVmax (rho = 0.89, P<0.001), but not so much with lymph node SUVmax (rho = 0.43, P=0.03). Interventricular septum was the most commonly involved (69.2%) area of the heart. The entire myocardium was involved in 21.5% of the patients. Uptake in the basal myocardial wall was observed in 8 (12.3%) patients.

**Conclusions:** Baseline CHT levels were significantly correlated with myocardial SUVmax and the CHT levels were higher in patients with involvement of entire myocardium and in patients who had both myocardial and LN involvement.

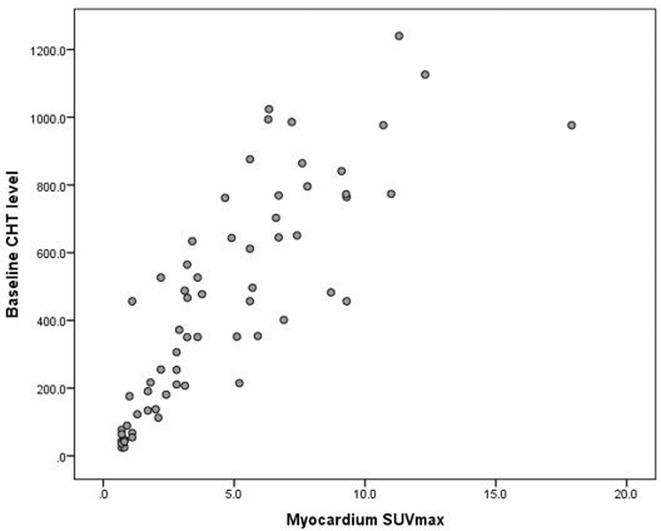

## TWO IN ONE: SUCCESSFUL TREATMENT OF TORSADES DE POINTES AND RAYNAUD’S PHENOMENON BY LEFT STELLATE GANGLION ABLATION

### **VIJAY YADAV**, JASVINDER SINGH, SIDDHARTH CHAVALI, MUTHIAH SUBRAMANIAN, DALJEET SAGGU, CALAMBUR NARASIMHAN

#### AIG Hospitals, Hyderabad, India

**Introduction:** Although drug refractory ventricular tachycardia (VT) is amenable to catheter ablation and surgical sympathectomy, both of them may not be immediately feasible in critically ill and hemodynamically unstable patients. The vasospastic spells of Raynaud's phenomenon (RP) is a sympathetically mediated process. Stellate ganglion blockage (SGB) or ablation is an emerging minimally invasive technique for treating both refractory VT and RP via temporary sympathetic interruption.

**Methods:** N/A

**Results:** A 32‐year‐old‐female with Sjogren's syndrome presented with palpitations. A 12‐lead electrocardiography (ECG) showed QTc interval of 617 ms with R‐on‐T phenomenon degenerating into polymorphic VT with rates of 214 bpm. She had persistent hypokalemia, hypomagnesemia, and hypocalcemia owing to renal loss secondary to tubulointerstitial nephritis. She also developed sharp pains, numbness, and bluish discoloration of bilateral upper distal limbs. In view of drug refractory polymorphic VT and RP, a left stellate ganglion block was performed by injecting 10 ml of 0.5% bupivacaine between the prevertebral fascia and the ventral surface of longus colli muscle under ultrasonographical assistance. Eventually, a pulsed radiofrequency ablation of the left stellate ganglion was also performed for a total of two cycles at 42 Degree Celsius for 180 seconds each. Post procedure, there was a significant improvement in the color of limbs with decrement of QTc to 383 ms, development of Horner's syndrome, and the pulse oximeter tracing showed a high Perfusion Index.

**Conclusions:** We present a rare case of simultaneous occurrence of acquired long QTc with refractory polymorphic VT and vasospastic episodes of RP where the left sided stellate ganglion block followed by its ablation can be a promising therapy to eradicate both the ailments.

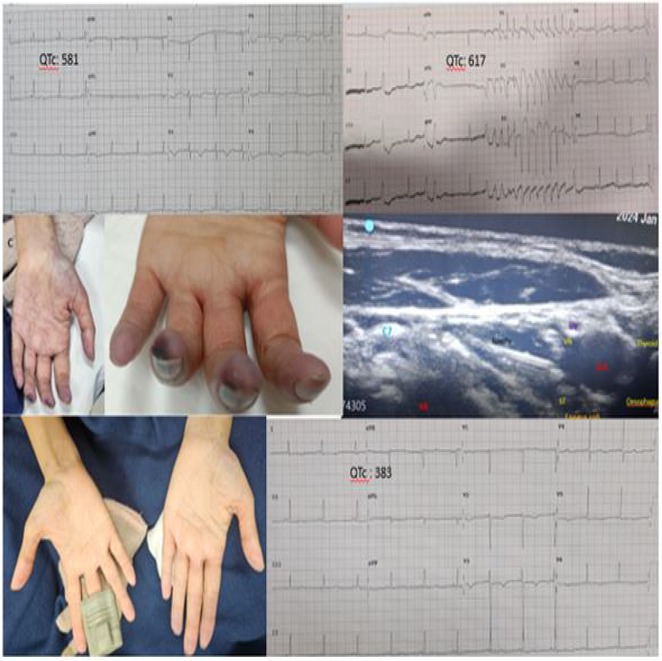

## AVNRT WITH 2:1 CONDUCTION WITH RBBB AND LBBB ABERATION

### RAMDEO YADAVE

#### Batra Hospital, Delhi, India

**Introduction:** Eight year old female presented with paroxysmal palpitations. ECG during palpitations showed PSVT. Echo showed structurally and functionally normal heart. Coronary angiography showed normal coronaries. She was taken up for EPS nd RF ablation of PSVT.

**Methods:** Four cathters put in the CS, HRA, RVA and His. On V extra retrograde conduction was concentric and decremental suggestive of retrograde conduction through the AV node.On A‐pacing dual AV nodal physiology seen with frequently inducible AVNRT with 2:1 conduction which become 1:1 on V extra and due to faster rate RBBB aberration. This aberration disappear and become narrow QRS tachycardia which spontaneously changed to LBBB aberration. Maneuver done to exclude other mechanism of tachycardia.

**Results:** Successful slow pathway ablation done during tachycardia with loss of dual AV nodal physiology. Over 6 months of follow up there was no recurrence of tachycardia.

**Conclusions:** AVNRT can present with 2:1 conduction particularly at onset and can become 1:1 on v extra due to peeling back of refractoriness. AVNRT can develop RBBB or LBBB aberration in same patient.

## FOURTEEN YEAR OLD BOY WITH TWO TYPES OF NARROW QRS TACHYCARDIA

### RAMDEO YADAVE

#### Batra Hospital, Faridabad, India

**Introduction:** •Fourteen year old male child presented with paroxysmal palpitations for two years. •Baseline ECG showed no preexcitation. •ECG during tachycardia showed narrow QRS. •Echo showed structurally and functionally normal heart. •He was taken up for EPS and RF Ablation.

**Methods:** On EP study we found presence of dual physiology on A‐ Pacing. On V‐pacing from RV apex VA conduction was concentric and incremental suggestive of retrograde conduction through the AV node. On atrial extra Regular narrow QRS tachycardia was induced with VA <70 msec which excludes bypass tract. RV apical Burst pacing during tachycardia also showed concentric retrograde A but on stopping leads to ORT with earliest A at distal CS suggest presence of left lateral AP.Maneuver done to exclude other mechanism of tachycardia.By trans‐septal route and on LV pacing eccentricity demonstrated and mapped in left posterolateral mitral annulus.

**Results:** First Iablated the slow pathway with loss of dual AV nodal physiology.Then paced LV lateral wall which showed eccentric retrograde pathway conduction. By single RF application left lateral AP was ablated with retrograde conduction block in the pathway. Over 2 months of follow up no recurrence of any tachycardia or palpitations.

**Conclusions:** This fourteen year boy having both AVNRT and ORT through left lateral AP. Both tachycardia was ablated successfully.No complications.

## MODERATOR BAND VT AND VF

### RAMDEO YADAVE

#### Batra Hospital, Delhi, India

**Introduction:** Thirty two year old male presented with palpitaions and syncope. ECG showed frequent VPCs in bigeminy and couplets and salvos. 24hour holter showed 42 % VPC. Echo showed structurally normal heart.

**Methods:** Patient underwent 24 hour holter after recurrence of syncope which showed self terminating VF.He was recommended for ICD implantation and put on Tab Amiodarone 200 mg once daily and Cap Maxilletine 150 mg twice daily.

**Results:** Moderator Band VT can present with VF and needs ICD even after ablation.

**Conclusions:** Moderator band VT can degenerate into VF and needs ICD implantation.

## ORTHODROMIC TACHYCARDIA FROM RIGHT POSTEROLATERAL CONCEALED ACCESSORY PATHWAY

### RAMDEO YADAVE

#### Batra Hospital, Faridabad, India

**Introduction:** Twenty seven year old male presented with paroxysmal palpitation. ECG durig palpitations showed PSVT.Baseline ECG showed no preexcitation. Echo showed structurally and functionally normal heart.He was taken up for EPS and RF ablation.

**Methods:** Four catheter put in. 6F decapolar in CS, 6F quadripolar in HRA and RVA and 7F ablation catheter St.jude at His and Right posteolateral area. On V ‐pacing earliest A is eccentric at HRA suggest Right lateral AP. On A‐pacing and spontaneously with atrial ectopics PSVT induced with earliest A in HRA suggest ORT through right lateral AP. V ‐Extra during His refractory PVC resets tachycardia confirming ORT. VOD pacing showed VAVA which excludes AT and PPI‐TCL and SA‐VA suggestive of ORT.

**Results:** Successful RF ablation done by single RF application with VA block. No complication occured.

**Conclusions:** Right posterolateral concealed accessory pathway is relatively rare cause of SVT and is curable by RF ablation. Over 6 months of follow up he is asymptomatic.

## RF ABLATION OF WPW SYNDROME IN AF

### RAMDEO YADAVE

#### Batra Hospital, Faridabad, India

**Introduction:** Fifty five year old male with recurrent palpitationwith ECG showing WPW syndrome with Rt.lateral free wall AP. Took him for EPS and RF ablation but every times goes in to AF.

**Methods:** Due to recurrent or almost incessant AF we could not able to map the pathway in sinus Rhythm or V pacing or A‐pacing. Therefore mapped the WPW in AF with earliest deep negative of Unipolar electrode and ablated with loss of preexcitation. But needed Cardioversion to check the retrograde conduction of AP which was blocked.

**Results:** Successful Rf Ablation of AP in AF done. No recurrence of pathway for last 10 years of follow up.

**Conclusions:** WPW can be ablated in AF by seeing the unipolar EGM.

## RFA OF LA FLUTTER POST DVR ROOF DEPENDENT

### RAMDEO YADAVE

#### Batra Hospital, Faridabad, India

**Introduction:** •Fifty eight year old male with RHD having severe MR and AR underwent DVR. •15 days after DVR he developed Regular Wide QRS Tachycardia with RBBB and RAD. •Baseline ECG showed Atrial flutter with positive P wave in V1 and leads II,III,and aVF suggestive of LA flutter after they treated with Diltiazem injection. •LVEF was 40% with global hypokinesia may be due to tachycardiomyopathy. Taken up for 3D mapping of LA flutter

**Methods:** On 3D mapping showed LA flutter roof dependent anteriorly is conducting channel which was on entrainment confirmed in the circuit with EGM lon fractionated suggest slow conducting channel.

**Results:** Single RF application terminated Atrial flutter but we make a roof line. On 2 years of follow up no recurrence of tachycardia.He had also Atrial tachycardia from posterior part of CS Os which was focal was also ablated.

**Conclusions:** Successfull RF ablation of Atrial flutter from LA roof was ablated without recurrence. Successful RF Ablation of AT from CS os was also ablated without recurrence.

## WPW SYNDROME WITH CORONARY SINUS ASSOCIATED ACCESSORY PATHWAY PRESENTED WITH AF

### RAMDEO YADAVE

#### Batra Hospital, Faridabad, India

**Introduction:** WPW syndrome associated with coronary sinus presented with Preexcited AF.Patient had structurally and functionally normal heart.

**Methods:** During EP study confirmed that Accessory pathway was associated with Coronary sinus floor. There was LBBB aberration during tachycardia with 20 msec of CL difference on narrowing of tachycardia suggest pathway was associated with RA to LV connection. V extra during His refractory sowed resetting of tachycardia suggest pathway was part of Orthodromic tachycardia.VOP also confirmed ORT.

**Results:** By using cool tip catheter and single RF application at the floor of Coronary sinus successful RF ablation done with block in both antegrade and retrograde conduction of pathway. No complications. On V pacing there was VA block.

**Conclusions:** WPW syndrome with CS associated pathway are dangerous due AF and impending VF as in our patient. RF Ablation of this pathway is cure from the risk of Sudden cardiac death.Over 6 months of follow up there is no recurrence of pathway conduction.

## CONTINUOUS POSITIVE AIRWAY THERAPY IMPROVES AUTONOMIC NERVE DYSFUNCTION ASSOCIATED WITH SLEEP‐DISORDERED BREATHING IN PATIENTS AFTER ABLATION OF ATRIAL FIBRILLATION

### ASAMI YAMADA

#### Steel Memorial Yawata Hospital, Kitakyushu‐shi,Fukuoka, Japan

**Introduction:** Sleep‐disordered breathing (SDB) is an independent risk factor for new‐onset of atrial fibrillation (AF) and recurrence of AF after ablation. Dysfunction of the autonomic nervous system may be a potential mechanism whereby SDB is linked to AF. Repetitive sympathetic activation during apneic episodes may impair cardiovascular reflex function, and increased sympathetic activity can stimulate renin release contributing to new‐onset and recurrence of AF. Given that patients with SDB may have reduced autonomic function, the purpose of this study was to determine whether treatment with continuous positive airway pressure (CPAP) for 1‐3 months would improve autonomic function.

**Methods:** Forty participants after ablation of AF with a diagnosis of SDB (apnea‐hypopnea index ≥ 20 events per hour) were prospectively evaluated for autonomic nerve function at baseline and post CPAP therapy for 1‐3 months using coefficient of variation of R‐R interval (CVRR) and the recurrence rate of AF after ablation. Ten participants discontinued CPAP therapy.

**Results:** Participants who continued CPAP therapy (n = 30) in this study showed improved CVRR after 1‐3 months (average 2 ± 1 months) of treatment (baseline vs follow‐up) as assessed by the mean (± SD) CVRR (1.56 ± 0.51 vs 2.19 ± 0.42; p < 0.05). On the other hand, participants who discontinued CPAP therapy (n = 10) did not improve CVRR after treatment (1.46 ± 0.53 vs 1.67 ± 0.48; p = 0.235). There was no statistically significant difference of the recurrence rate of AF between the participants who continued and discontinued CPAP therapy (3% vs 2%; p = 0.788).

**Conclusions:** Treatment of SDB with CPAP in patients after ablation of AF for 1‐3 months improved CVRR and may be beneficial in reducing the risk of autonomic nerve dysfunction.

## CONSIDERATIONS TO PREVENT DEVICE INFECTION IN CASES OF SIMULTANEOUS LEAD EXTRACTION AND IMPLANTATION

### TAKASHI YAMADA

#### Takaishi‐Fujii Hospital, Takaishi, Japan

**Introduction:** During cardiac implantable electronic device (CIED) implantation procedures, procedure time is a crucial factor affecting the occurrence of post‐implantation infections.

**Methods:** Among 491 CIEDs implanted between October 2016 and April 2023, 356 were newly implanted, including simultaneous implantations following non‐infectious lead extractions, while excluding battery replacements using transvenous leads, leadless pacemakers, and subcutaneous implantable cardioverter defibrillators.

**Results:** The mean age was 76.4 ± 11 years, 58.4% were male, 30.6% received cardiac resynchronization therapy (CRT) implantations, 41.3% underwent re‐implantations after removals, and 23.0% had simultaneous lead implantations following non‐infectious reasons (such as lead failures or upgrades). The mean operative time (from skin‐to‐skin) was 50.6 ± 29 min, and the mean fluoroscopy time was 7.8 ± 8.7 min. The procedure time differed significantly between de novo implantations and simultaneous implantations after removal (41.0 ± 17 min vs. 82.7 ± 37 min, respectively; P < 0.001). Procedure times for CRT and non‐CRT were 76.1 ± 34 min and 39.4 ± 17 min, respectively (P < 0.001). Furthermore, procedure times for de novo CRT and simultaneous CRT implantations after lead removals were 62.2 ± 19 min and 97.4 ± 41 min, respectively (P < 0.001). Procedure times exceeding 2 hours occurred in 14 (3.9%) cases, of which 13 (92.9%) were CRT implantations, 13 (92.9%) were simultaneous implantation cases, and 12 (85.7%) were CRT cases with simultaneous implantations after lead removals. In multivariate analysis, predictors of procedure times of 2 hours or longer included male gender (P = 0.039; OR 0.052‐0.925), CRT (P = 0.004; OR 0.005‐0.365), subclavian vein occlusion or severe stenosis (P = 0.036; OR 0.036‐0.895), and simultaneous implantation after removal (P = 0.037; OR 1.174‐145.6).

**Conclusions:** Simultaneous CRT implantations following transvenous lead extractions prolong procedure times compared to other CIED implantation procedures. Therefore, attention to post‐implantation infections may be warranted.

## THE EFFECT OF NON‐INFECTIOUS INDICATIONS ON TRANSVENOUS LEAD EXTRACTION TECHNIQUES

### TAKASHI YAMADA

#### Takaishi‐Fujii Hospital, Takaishi, Japan

**Introduction:** In recent years, as transvenous lead extraction (TLE) has become more widespread, it has been increasingly indicated not only for infectious cases but also for non‐infectious cases. In this study, we investigated to elucidate the differences in clinical and procedural aspects of the TLE procedure between infectious and non‐infectious cases.

**Methods:** A total of 484 consecutive TLE cases performed by our extraction team between 2010 and 2023 were analyzed. These cases were divided into two groups based on whether the indication for TLE was infectious or non‐infectious. We utilized a laser sheath as the initial powered sheath in all TLE cases, and additional devices included rotational dilator sheaths, Byrd dilator sheaths, and a femoral workstation.

**Results:** The number of cases with infectious indications (Group I) and non‐infectious indications (Group NI) were 347 and 139, respectively. There was a significant difference in patient age between the two groups (Group I: 74.2 vs. Group NI: 68.8; P < 0.001), while the duration of lead dwell time (years) showed non‐significant differences between the two groups (Group I: 9.1 vs. Group NI: 7.7; P = 0.067). The complete removal rate (Group I: 94.5% vs. Group NI: 95.0%; P = 0.846) and major complication‐free rate (Group I: 98.6% vs. Group NI: 97.1%; P = 0.288) did not show statistically significant differences between the two groups. The number of extracted leads differed between the groups (Group I: 2.3 vs. Group NI: 1.8; P < 0.001), as did the number of additional devices per patient (Group I: 0.25 vs. Group NI: 0.35; P = 0.049). The total laser pulse burden per case was similar between the groups (Group I: 856.1 vs. Group NI: 770.7; P = 0.526), but the total procedure time (Group I: 70.5 min. vs. Group NI: 86.3 min.; P < 0.001) and total fluoroscopy time (Group I: 11.4 min. vs. Group NI: 15.9 min.; P < 0.001) were longer in Group NI.

**Conclusions:** This study suggests that the difficulty of transvenous lead removal procedures may be higher in non‐infectious cases compared to infectious cases, as evidenced by longer procedure times and a greater number of devices used, even when the duration of lead implantation is similar.

## COMPARISON OF ABLATION SAFETY AND ISOLATION AREA SIZE USING NOVEL 31 MM EXPANDABLE CRYOBALLOON AND CONVENTIONAL 28 MM CRYOBALLOON CATHETERS

### **HOKUTO YAMAGISHI**, SHIGETOSHI SAKABE, KENICHI MAENO, ATSUNOBU KASAI

#### Japanese Red Cross Ise Hospital, Ise, Japan

**Introduction:** The POLARx^TM^FIT (PXF) is a variable cryoballoon for pulmonary vein isolation (PVI) that can expand balloon size from 28mm to 31mm. The purppose is compare the safety of the PFX with a conventional 28mm balloon and the size of the electrical isolate area (EIA) immediately after treatment.

**Methods:** The group treated with PXF (G31) was compared with those treated with a conventional 28mm balloon (G28). The subjects were consecutive randomized patients treated at our institution between June 2023 and February 2024. Common treatment strategies were guided by the EnSite Precision™ Cardiac Mapping System (EnSite) 3D‐mapping system. After successful isolation, no left atrial (LA) roofline was created. At the end of treatment, EnSite produced voltage maps of the LA and 4 PVs. We defined EIAs as those with a potential <0.1 mV. The average scar area ratio (ASAR) was defined as the ratio of the EIA formed by the cryoballoon to the total surface area of the LA and the 4PV within 10 mm of the antrum. This study investigated the details of the procedure and the analysis of the voltage maps immediately after treatment.

**Results:** There were 36 G31 cases (67% male, age 69.5±8.7 years, paroxysmal AF 64%, LA diameter 42.4±5.4 mm) and 31 G28 cases (58% male, age 68.9±11.3 years, paroxysmal AF 61%, LA diameter 41.7±7.3 mm. Two G‐31 cases and a G28‐A case required touch‐up ablation with a radiofrequency catheter. Moreover, 7.4% of PVs in G‐31 required a reduction of the balloon size to 28mm. The following results are presented for G31 and G28 in that order. Freezing time per case, excluding touch‐up cases, was 880±266 s vs. 834±188 s. Analysis of the voltage map of LA and PVs immediately after treatment, excluding touch‐up cases, showed that 20% vs. 36% of cases had residual potential in the carina only, ASAR was 40.5±9% vs. 40.3±12.8%. There were no significant differences in endpoints between the two groups.

**Conclusions:** The results show that the usability of the novel 31mm expandable cryoballoon and the size of the EIA of the LA with it are comparable to that of the conventional 28mm balloon.

## CORE TEMPERATURE MANAGEMENT USING HEAT INSULATION BLANKETS IN PATIENTS UNDERGOING CRYO BALLOON ABLATION FOR ATRIAL FIBRILLATION UNDER GENERAL ANESTHESIA

### ARISA YAMAGUCHI

#### STEEL MEMORIAL YAWATA HOSPITAL, Kitakyushu, Japan

**Introduction:** Inadvertent intraoperative hypothermia (core temperature <36°C) is a frequently preventable complication with several adverse consequences including longer time to hemostasis, delay awakening from anesthesia, and longer postoperative hospital days. This single‐center randomized clinical trial assessed the efficacy of the heat insulation blanket (HIB) in patients undergoing cryo balloon ablation (CBA) for atrial fibrillation (AF).

**Methods:** In all, 60 patients undergoing CBA for non‐valvular paroxysmal AF were prospectively assigned to either the HIB (n=30) or control (conventional towelette; n=30) group. The core temperature was measured in the esophagus using an esophageal temperature probe. The primary endpoints of this study were time to hemostasis, awakening time from anesthesia, and post operative complications. Secondary endpoint was the length of hospital stay.

**Results:** Core temperature was significantly higher in the HIB group than control group (mean [±SD] 35.0 ± 1.0 vs. 35.5 ± 0.5°C; p=0.037). There were no significantly differences in the time to hemostasis (17.7 ± 6.4 vs. 18.8 ± 10.6 μινυτεσ; p=0.634), awakening time from anesthesia (29.0 ± 23.1 vs. 23.2 ± 12.1 μινυτεσ; p=0.091), prevalence of the post operative complications (0% vs.7%; p=0.155), and between the 2 groups. However, the awakening time from anesthesia was significantly longer in the patients with the temperature differences more than 1.5°C (n=15) between pre‐ and post‐ CBA for AF than less than 1.5°C (n=45) (28.3 ± 15.0 vs. 25.3 ± 12.4 minutes; p=0.028).

**Conclusions:** The use of HIB might not be associated with the time to hemostasis, awakening time from anesthesia, prevalence of the post operative complications, in patients undergoing CBA for AF under general anesthesia. Finally, future investigations should focus on whether the use of HIB could affect those factors in the patients with the temperature differences more than 1.5°C between pre‐ and post‐ CBA for AF.

## PREDICTION OF REDUCED LEFT ATRIAL APPENDAGE FLOW VELOCITY USING LEFT ATRIAL APPENDAGE FILLING DEFECTS ON CARDIAC COMPUTED TOMOGRAPHY IN PATIENTS WITH ATRIAL FIBRILLATION

### **JUMPEI YAMAMOTO** ^1^, YOSHINARI ENOMOTO^2^, MASAYA YAMAMOTO^2^, HISAO HARA^2^, YUKIO HIROI^2^

#### ^1^Toho University Ohashi Medical Center, Meguro‐ku, Tokyo, Japan,^2^National Center for Global Health and Medicine, Shinjuku‐ku, Tokyo, Japan

**Introduction:** Patients with atrial fibrillation (AF) who undergo cardiac computed tomography (CCT) often show left atrial appendage (LAA) filling defects in the early contrast phase. This finding may increase stroke risk, but the relationship between LAA flow velocity (LAAFV) and LAA filling defects is not well understood.

**Methods:** We retrospectively analyzed the 138 patients (median age 69 years, 36 females) with AF who underwent transesophageal echocardiography (TEE) and CCT within 3 months from January 2016 to March 2024. CCT was performed in the supine position in both the early and delayed contrast phases, and patients with LAA thrombus were excluded. As in the previous study, LAA filling defect was defined as incomplete mixing of contrast and blood and appeared only in the early phase. LAAFV was defined as the average of the filling and emptying flow velocities measured at TEE. Low LAAFV was defined as LAAFV <40 cm/sec, and patients were divided into two groups by LAAFV.

**Results:** Low LAAFV was present in 57%. Mean CHA₂DS₂‐VASc score was 2.4, LAA filling defect was present in 34%, oral anticoagulant was taken in 94%, and persistent AF was present in 59%. The low LAAFV group had significantly more persistent AF, history of heart failure, LAA filling defects, increased CHA₂DS₂‐VASc score, E/e′ and left atrial diameter, and lower left ventricular ejection fraction. In multivariate analysis, left atrial diameter (odds ratio, 1.16; 95% confidence interval (CI), 1.05‐1.28; p = 0.004), persistent AF (OR, 4.55; 95%CI, 1.76‐11.8; p = 0.002), and LAA filling defect (OR, 7.60; 95%CI, 2.47‐23.4; p < 0.001) were independent low LAAFV factors, whereas CHA₂DS₂‐VASc score and E/e' were not. The results were consistent with no interaction in the subanalysis for gender or left ventricular ejection fraction 50%.

**Conclusions:** LAA filling defects in the early contrast phase of CCT in patients with atrial fibrillation may reflect reduced LAAFV. This finding may be useful for functional assessment of LAA thrombotic risk, which is difficult to assess with existing stroke prediction scoring.

## EFFICACY OF LASER BALLOON LEFT ATRIAL ROOF ABLATION FOR PATIENTS WITH ATRIAL FIBRILLATION

### **TAKASHI YAMASAKI**, KEN KAKITA, MISUN PAK, TETSUHISA HATTORI

#### Koseikai Takeda Hospital, Kyoto, Japan

**Introduction:** The roof area ablation beyond pulmonary vein (PV) isolation (PVI) using a cryoballoon has been described as an effective therapy for patients with atrial fibrillation (AF). However, the effect of roof area ablation performed with a visually guided laser balloon (VGLB) remains to be elucidated. This study aims to investigate the efficacy of roof area ablation using VGLB.

**Methods:** A total of 325 patients with AF (paroxysmal AF=199, persistent AF=126) who underwent VGLB ablation from July 2018 to May 2022 were analyzed retrospectively. After initial circular isolation, VGLB was inflated to a larger size on both sides of superior PVs, and the isolation areas were extended to the roof portion. At the end of the procedure, left atrial (LA) voltage mapping was obtained to assess the LA roof scar area, and differential pacing was performed to confirm the complete conduction block. Our study population was divided into the PVI group (LB‐PVI, n=228) and the roof line block beyond PVI group (LB‐Roof, n=97). The efficacy endpoint was defined as freedom from atrial tachyarrhythmia (ATa) between months 3 and 12.

**Results:** One‐year clinical follow‐up could be obtained in 170 patients with LB‐PVI and 81 patients with LB‐Roof. The Kaplan‐Meier survival curves are shown in Figure. At the end of the 12 months of follow‐up, freedom from ATa was 80.8% in patients with LB‐PVI and 91.2% in LB‐Roof. The ATa freedom rate was significantly higher in the LB‐Roof than in the LB‐PVI (P=0.03).

**Conclusions:** The roof area ablation using VGLB was proved to be effective for patients with AF.

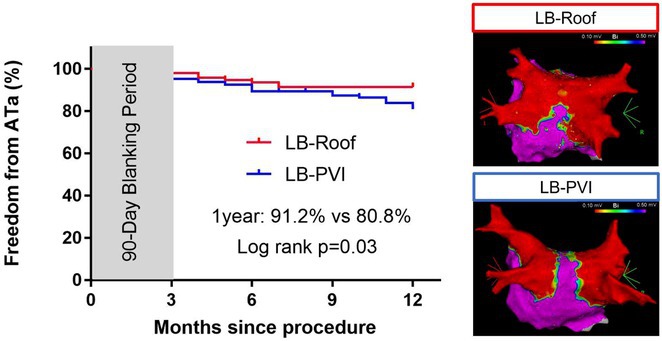

## ATRIAL FIBRILLATION ABLATION VIA SUPERIOR VENA CAVA IN PATIENTS WITH INTERRUPTED INFERIOR VENA CAVA USING REMOTE MAGNETIC NAVIGATION SYSTEM

### **KOHEI YAMASHIRO**, HIROKI SAKUMA, TOMOMI TANAKA

#### Takatsuki General Hospital, Takatsuki, Japan

**Introduction:** In patients with interruption of inferior vena cava (IVC), transseptal access for atrial fibrillation (AF) ablation cannot be obtained via femoral vein. The same is true in patients with a permanently implanted IVC filter. In these complex cases, ablation is performed via a superior approach. While in manual ablation, catheter manipulation is difficult and the operator is exposed to a large amount of radiation, remote magnetic navigation (RMN) can be used safely and effectively. This study describes the technique and outcomes of RMN‐guided AF ablation via superior vena cava (SVC).

**Methods:** Twenty cases of AF ablations via SVC were reviewed in 18 patients (mean age 64 + 12 years old; 50% female), performed between March 2018 and May 2023. AF type was paroxysmal AF (n=10), non‐paroxysmal AF (n=10) and recurrent AF (n=5). Due to interruption of the IVC (n=11), obstruction of the IVC (n=4) and IVC filter (n=5), the procedure via femoral vein was not possible.

**Results:** Transseptal puncture via SVC was successful in 19 cases, with the addition of a trans‐aortic approach in 2 of them. In one case, pulmonary vein isolation (PVI) had already performed. In 19 cases, PVI was performed and successfully, and additional procedures were performed in 15 cases. Mean total procedure time was 215.8 + 56.5 min (total fluoroscopy time 39.8 + 24.8 min). At a mean follow‐up of 13.4 + 14.7 months, 13 patients (72%) remained free from sustained arrhythmia recurrences. There was one case of pericardial effusion.

**Conclusions:** Remote magnetic navigation‐guided atrial fibrillation ablation by a superior approach is feasible alternative technique in patients who have difficulty via inferior vena cava.

## THE IMPLICATION OF PATIENT‐REPORTED HEALTH STATUS ON THE EFFICACY OF CATHETER ABLATION FOR ATRIAL FIBRILLATION

### **SHUHEI YAMASHITA**, YOSHINORI KATSUMATA, SHUHEI YANO, YUKIHIRO HIMENO, KOKI YAMAOKA, SUSUMU IBE, TAKAHIKO NISHIYAMA, TAKEHIRO KIMURA, IKUKO UEDA, SHUN KOHSAKA, SEIJI TAKATSUKI, MASAKI IEDA

#### Departmet of Cardiology, Keio University School of Medicine, Tokyo, Japan

**Introduction:** Although evaluating patient‐reported health status (HS) has been highly recommended for optimizing atrial fibrillation (AF) care, its clinical implication on the efficacy of catheter ablation (CA) remains unknown.

**Methods:** We extracted data from 2,883 patients from a multicenter Japanese AF registry, including information on patient‐reported HS via Atrial Fibrillation Effect on Quality‐of‐Life (AFEQT) survey. A composite outcome of death, heart failure hospitalization, and stroke was compared between patients undergoing CA and those receiving drug therapy after applying propensity overlap weighting in subgroups stratified by baseline AFEQT‐OS.

**Results:** In overall, median age was 68.3 years, 912 (31.6 %) were female. Lower baseline AFEQT‐OS was linearly associated with the more favorable cardiovascular outcomes for CA compared to medication. Based on the finding, the entire population was stratified into two subgroups; impaired HS (baseline AFEQT‐OS<80) and preserved HS (baseline AFEQT‐OS≥80). In the weighted cohort of impaired HS group, CA was associated with a lower incidence of composite outcome (hazard ratio, 0.49 [95%CI, 0.31 to 0.79], p=0.031) and heart failure hospitalization (HR 0.39 [95%CI, 0.19 to 0.79], p=0.0087) compared to drug therapy. Among patients with preserved HS, there was no significant difference in the risk of the composite outcome between two treatment groups.

**Conclusions:** Impaired baseline HS was associated with the favorable efficacy of CA over medication on cardiovascular outcomes. Our findings suggest that assessing patient‐reported HS in clinical practice can support optimal care delivery for AF patients.

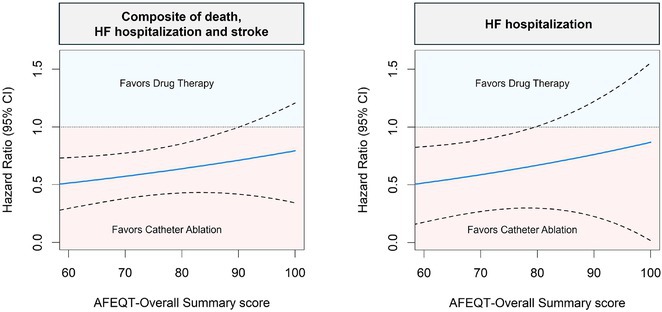

## DON’T’ STOP EFFECTIVE ATRIAL‐BASED ANTITACHYCARDIA PACING! INSIGHTS INTO EPISODE DURATION AND SUCCESS RATE FOR TERMINATION

### **SATOSHI YANAGISAWA** ^1^, YUKI SATO^2^, YASUYA INDEN^1^, YUJI NARITA^1^, ATSUYA SHIMIZU^3^, REI SHIBATA^1^, TOYOAKI MUROHARA^1^

#### ^1^Nagoya University Graduate School of Medicine, Nagoya, Japan,^2^Nagoya University Hospital, Nagoya, Japan,^3^National Center for Geriatrics and Gerontology, Obu, Japan

**Introduction:** Second‐generation atrial‐based anti‐tachycardia pacing (rATP; Medtronic) represents a therapeutic avenue for mitigating atrial fibrillation (AF) burden in patients with cardiac implantable electrical devices. Typically, the efficacy of rATP is confirmed by initiating the therapy against a frequent AF burden and subsequently observing a decline in AF. However, we present herein a unique case featuring an uncommon scenario wherein rATP effectiveness transitioned from on‐ to off‐operation inversely.

**Methods:** N/A

**Results:** A 74‐year‐old female presented with chest discomfort at an outpatient clinic. She had undergone dual‐chamber pacemaker implantation for sick sinus syndrome 7 years previously. The pacing mode was set with managed ventricular pacing to maintain intrinsic atrioventricular conduction. Due to a long interval of AP‐VS >400 ms in AAI mode, which was a possible cause of her symptoms, we changed the pacing mode to DDDR and discontinued rATP, introduced for AF 4 years ago. It is noteworthy that recent AF occurrences were infrequent, with short durations of a few minutes, and the success rate of rATP termination was 100%. Three months after cessation of rATP, the patient was hospitalized for heart failure (HF) alongside persistent AF lasting 3 weeks. HF management and administration of pilsicainide effectively terminated the AF. Subsequently, rATP was reactivated, resulting in AF suppression for 6 months post‐discharge. The development of persistent AF in this case may be precipitated by the cessation of rATP and subsequent increase in ventricular pacing rate, with HF exacerbation perpetuating the AF. Because of many AF episodes appearing short over the long term, and AF occurring infrequently in the last 6 months, the decision was made to discontinue rATP therapy. However, the remarkably high success rate of rATP played a pivotal role in suppressing AF progression during its early stages, effectively concealing all episodes within brief AF durations.

**Conclusions:** This unique scenario underscores the efficacy of rATP in managing numerous short AF episodes, which became evident upon cessation of therapy.

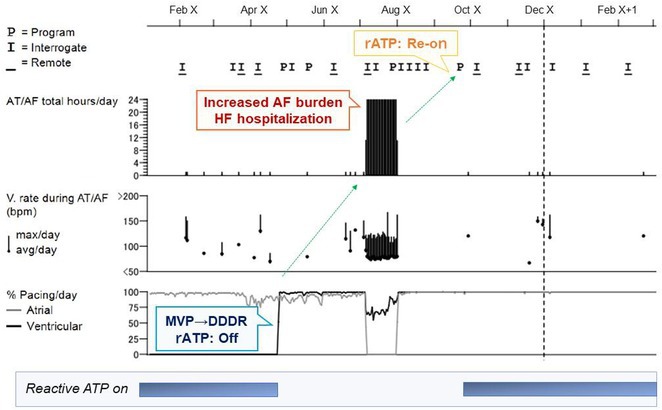

## CLINICAL IMPACTS OF LEFT ATRIAL SIZE AND ITS CHANGES AFTER CARDIAC RESYNCHRONIZATION THERAPY IN HEART FAILURE PATIENTS

### **JUWEI YANG**, YU YU, SIJING CHEN, HAO HUANG, TIANXIN LONG, BINGQI FU, WEI HUA

#### State Key Laboratory of Cardiovascular Disease, Fuwai Hospital, Chinese Academy of Medical Sciences & Peking Union Medical College, Beijing, China

**Introduction:** Limited evidence exists on the impact of left atrial (LA) diameter and its changes post‐cardiac resynchronization therapy (CRT) on the prognosis of heart failure (HF) patients. Thus, this study aimed to investigate the association between LA diameter and its changes with all‐cause mortality and HF hospitalization in HF patients with CRT.

**Methods:** This retrospective cohort study comprised 488 HF patients with CRT who underwent echocardiography at baseline and 6 months after CRT. The study used multivariate Cox proportional hazards regression models to investigate the association between LA diameter and its changes with all‐cause mortality and HF hospitalization.

**Results:** Non‐linear associations were found for LA diameter with all‐cause mortality (P = 0.006) and HF hospitalization (P = 0.02). Positive associations were observed for LA diameter <45 mm with all‐cause mortality (HR, 1.88; 95% CI, 1.42‐2.49) and HF hospitalization (HR, 1.72; 95% CI, 1.32‐2.22). Additionally, there was a positive association of LA diameter changes with all‐cause mortality (HR, 1.29; 95% CI, 1.12‐1.48) and HF hospitalization (HR, 1.28; 95% CI, 1.13‐1.45). LA adverse remodeling (LAAR) was associated with an increased risk of all‐cause mortality (HR, 2.35; 95% CI, 1.46‐3.79) and HF hospitalization (HR, 2.18; 95% CI, 1.40‐3.41).

**Conclusions:** The study found that LA enlargement and LAAR were associated with an unfavorable outcome in HF patients with CRT, indicating that monitoring LA diameter and its changes can have potential clinical implications for HF treatment.

## PRECLINICAL FEASIBILITY OF A NOVEL STEROID ELUTING ELECTRODE OF HOLLOW HELIX FOR HIS BUNDLE PACING

### **ZHONGPING YANG**, TERI WHITMAN

#### Medtronic plc, Mounds View, MN

**Introduction:** His‐bundle (HB) pacing threshold is higher than myocardial pacing threshold. Steroid‐eluting (SE) electrode is known to suppress inflammation therefore to lower chronic pacing thresholds. A direct steroid elution on the electrode/tissue interface is highly desired to lower pacing threshold at HB. This study investigated preclinical feasibility of a beclomethasone dipropionate (BDP) filled hollow helix electrode on the 4‐Fr lead body design for improvement of pacing threshold at the HB.

**Methods:** The 4 Fr pacing lead body with 2.3 mm hollow helix platinum/Iridium (90/10) electrode was designed and prototyped for the study. The hollow helix electrode was preloaded with ~20 ug of BDP. The BDP is eluted through 3 of ϕ0.002” holes through the wire and the hollow (ϕ0.004” ) at the distal hollow helix electrode. The hollow helix without steroid was used as a control. Leads were implanted by mapping the His‐wave using the helix unipolar electrogram (EGM) and fixating at the HB in canine model (n=8). Monitoring, including the collection of x‐rays and pacing electrical data, were performed on these leads throughout the 12‐week follow up.

**Results:** The novel BDP filled hollow helix electrode significantly lowered pacing thresholds at the HB (p <0.001) as compared to the non BDP control electrode in the canines. The pathohistological examination in the canine also demonstrated the suppression of inflammation surrounding the hollow helix electrodes at HB.

**Conclusions:** The study results demonstrated that the novel BDP filled hollow helix electrode provides lower and stable chronic pacing thresholds compared to a non‐steroid eluting 4‐Fr helix design at the HB.

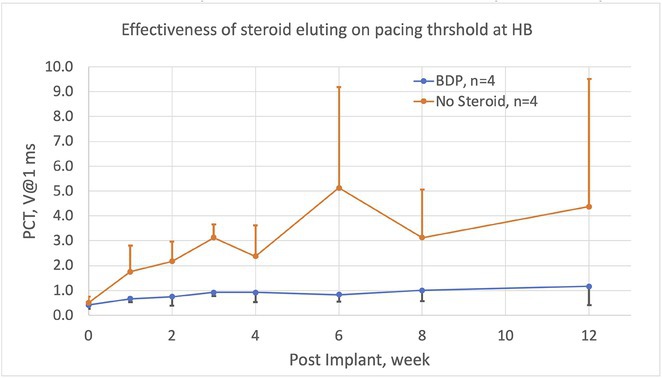

## EFFICACY OF PRONE POSITIONAL CONTRAST COMPUTED TOMOGRAPHY AND 20‐POLE THREE‐SITE MAPPING CATHETER WITH INNER LUMEN IN DETECTING THE VEIN OF MARSHALL

### **YUMI YASUI**, YASUTERU YAMAUCHI, ATSUHITO ODA, HIROFUMI ARAI, KAZUYA MURATA, YUICHIRO SAGAWA

#### Yokohama City Minato Red Cross Hospital, Yokohama, Japan

**Introduction:** Ethanol infusion into the vein of Marshall (VOM) has become increasingly common in atrial fibrillation (AF) ablation procedures. This study aimed to assess the feasibility of using contrast computed tomography (CT) for VOM detection.

**Methods:** We retrospectively examined consecutive 105 patients who underwent catheter ablation for AF. All patients underwent prone positional contrast CT before the procedure. Subsequently, retrograde coronary sinus (CS) venography was performed using a 6Fr 20‐pole three‐site mapping catheter with inner lumen (BeeAT‐IL). If VOM was not clearly visualized with BeeAT‐IL, balloon‐occluded CS venography was conducted.

**Results:** VOMs were detected in 83 (79%) of 105 patients. BeeAT‐IL successfully detected VOM in 80 patients (96%) and failed in 3 patients (4%). Contrast CT detected VOM in 60 patients (72%) out of 83 (with sensitivity=0.72, specificity=0.82, positive predictive value=0.94).

**Conclusions:** Our findings suggest that BeeAT‐IL and prone positional contrast CT could be effective for VOM detection in ablation for AF.

## EXPERIMENTAL STUDY COMPARING LESION FORMATION BY NONOCCLUSIVE CRYOBALLOON ABLATION BETWEEN POLARX FIT AND ARCTIC FRONT ADVANCE(AFA)‐ PRO

### **YUMI YASUI**, YASUTERU YAMAUCHI, ATSUHITO ODA, HIROFUMI ARAI, KAZUYA MURATA, YUICHIRO SAGAWA

#### Yokohama City Minato Red Cross Hospital, Yokohama, Japan

**Introduction:** Substrate modification on the left atrial roof area with cryoballoon has been described as an effective therapy for atrial fibrillation(AF). We aimed to evaluate lesion formation by nonocclusive cryoballoon ablation using northern hemisphere with two types of cryoballoon, POLARx Fit and AFA‐Pro.

**Methods:** We conducted an ex vivo experiment using novel models that turn red at ‐20°C for lesion visualization. We applied cryoablation for 3minutes by pressing northern hemisphere of cryoballoons against the model in a water tank and compared lesions produced by two cryoballoons.

**Results:** The depth was significantly larger in POLARx FIT (28mm:5.80±0.29mm,31mm:5.76±0.41mm) than in AFA‐Pro (4.34±0.32mm).(P<0.001) The length was significantly larger in POLARx FIT(28mm:23.26±0.99mm,31mm:23.50±1.31mm) than in AFA‐Pro(18.36±1.83mm).(P<0.001) The width had no significant difference(POLARx FIT 28mm:21.74±2.67mm, 31mm:23.64±2.05mm vs AFA‐Pro:21.66±1.03).

**Conclusions:** Nonocclusive cryoballoon ablation with POLARx FIT can produce a longer and deeper lesion, which is advantageous during left atrial roof ablation.

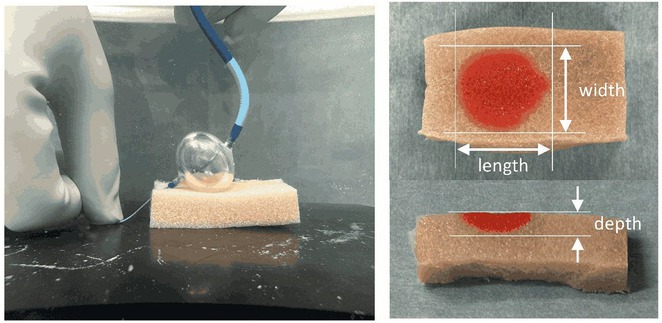

## TRAJECTORIES OF LIPID PROFILE CHANGES AND CARDIOVASCULAR OUTCOMES IN ASIAN ELDER PATIENTS WITH ATRIAL FIBRILLATION

### **JONG SHIUAN YEH** ^1,2^, YU‐HSUAN JONI SHAO^3^, CHIH‐CHIEH HUANG^4^, YU‐TING WANG^5^, JO‐HSIN CHEN^5^, GREGORY Y. H. LIP^6,7^

#### ^1^Taipei Medical University Wanfang Hospital, Taipei, Taiwan,^2^Division of Cardiology, Department of Internal Medicine, School of Medicine, College of Medicine, Taipei Medical University, Taipei, Taiwan,^3^Graduate Institute of Biomedical Informatics Taipei Medical University, Taipei, Taiwan,^4^Graduate Institute of Biomedical Informatics Taipei Medical University, Taipei, Taiwan,^5^Department of Pharmacy, Taipei Municipal Wanfang Hospital, Taipei Medical University, Taipei, Taiwan,^6^Liverpool Centre for Cardiovascular Science at University of Liverpool, Liverpool John Moores University and Liverpool Heart and Chest Hospital, Liverpool, United Kingdom,^7^Department of Clinical Medicine, Aalborg University, Aalborg, Denmark

**Introduction:** There is an association between ‘one off’ baseline total cholesterol and low‐density lipoprotein (LDL) cholesterol and cardiovascular outcomes, but an inverse link to incident atrial fibrillation (AF). Trajectories of lipid profile changes and cardiovascular outcomes in Asian patients with known AF have not been previously studied.

**Methods:** Using the Taipei Medical University Clinical Research Database (TMUCRD) from 2013 to 2020, we identified 1,926 patients with AF with 1‐year longitudinal lipid profiles at least 3 measurements a year, referred to as trajectories (Figure 1). We used accelerated failure time (AFT) model to examine the association of trajectories with the primary outcome of composite cardiovascular events (stroke, myocardial infarction and all‐cause mortality) until December 31, 2023.

**Results:** During follow‐up, a total 542 AF patients developed events (11.79 per 100 person‐years). In patients aged under 75 years, lipid profile trajectory was not associated with the primary outcome. Among patients aged over 75 years old, 3 trajectory groups for total cholesterol as group 1 (low); group 2 (high to low, ie. decreasing) and group 3 (middle) were identified (Figure 2). Compared to patients in group 1, patients in group 3 were associated with a mean 2.73‐year increase (95% confidence interval [CI]=1.05 to 7.11, p=0.0398) in survival time from the primary outcome. For LDL trajectories (Figure 3), group 3 (middle) was associated with a mean 2.43‐year increase (95% CI=1.11 to 4.94; p = 0.026) in the survival time from the primary outcome vs. group 1 (low). Total triglyceride trajectories were not significantly associated with the risk of the primary outcome.

**Conclusions:** In this hospital‐based cohort study, total cholesterol and low‐density lipoprotein trajectories are associated with composite cardiovascular outcomes in elderly Asian AF patients.

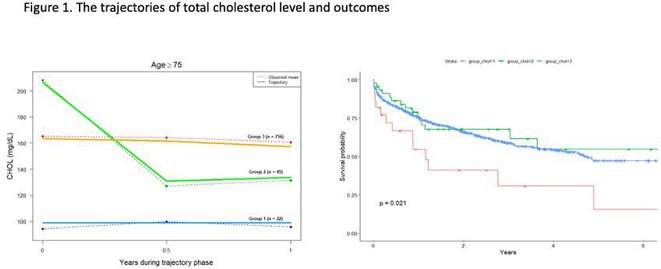

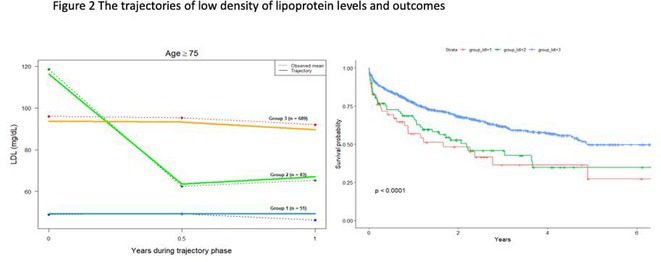

## PULSE OF THE PACIFIC: ANALYZING CARDIAC TACHYARRHYTHMIAS‐RELATED CARDIOVASCULAR MORTALITY TRENDS IN ASIAN AMERICANS

### **YONG HAO YEO** ^1^, TZE ERN ONG^2^, NISHAKI MEHTA^1^

#### ^1^Corewell Health William Beaumont University Hospital, Royal Oak, MI,^2^Melaka Manipal Medical College, Malacca, Malaysia

**Introduction:** Cardiac tachyarrhythmia management has advanced in recent decades, yet real‐world mortality data of cardiac tachyarrhythmia in Asian Americans remains limited.

**Methods:** We queried the CDC WONDER among patients ≥ 25 years old from 1999 to 2020. Cardiovascular diseases (CVD) were listed as the underlying cause of death, with cardiac tachyarrhythmia as the contributing cause, capturing patients where cardiac tachyarrhythmia was either the direct or proximate cause of death. Asian American subgroup categorization data was available between 2018 and 2020. We calculated age‐adjusted mortality rates (AAMR) per 100,000 individuals. We determined the trends over time by estimating the annual percent change (APC) using the Joinpoint regression program.

**Results:** In the 22‐year study period, there were 33,124 cardiac tachyarrhythmia‐related CVD deaths (174 (0.5%) supraventricular tachycardia, 26,662 (80.5%) atrial fibrillation/flutter [AF], and 5,911 (17.8%) ventricular arrhythmias [VA]) in Asian Americans. The overall AAMR in Asian Americans was lower than the other races (Asian 19.7 vs. American Indian 21.3 vs. African American 27.4 vs. White 37.8). In Asian Americans, the AAMR decreased from 22.8 in 1999 to 17.8 in 2007, with an APC of ‐2.2, but increased to 21.9 in 2020 with an AAPC of 1.2. The AAMR of VA decreased from 6.2 in 1999 to 2.8 in 2020, but the AAMR of AF increased (from 16.5 to 19.2). Similar trends were seen in other races. In Asian Americans, males also recorded higher AAMR compared to females (22.7 vs. 17.3). The top three Asian American subgroups with the highest percentage mortality were Filipino (25.5%), Chinese (24.1%), and Japanese (12.4%). Geographically, the West region had the highest AAMR (23.7). The AAMR was higher in rural than urban areas (21.9 vs. 19.6).

**Conclusions:** Cardiac tachyarrhythmia‐related CVD mortality among Asian Americans in the US declined in the first decade but increased in the second decade. Future effort is needed to address the disparities in sex, subgroup, and location.

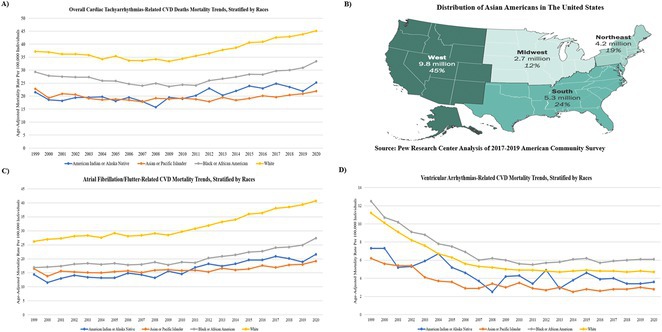

## TEMPORAL TRENDS OF HEART FAILURE RISK IN FEMALES WITH ATRIAL FIBRILLATION

### JIA‐YI HUANG^1^, AN‐PING CAI^2^, WEN‐LI GU^1^, RAN GUO^1^, JING‐NAN ZHANG^1^, GREGORY Y.H. LIP^3^, **KAI‐HANG YIU** ^1^

#### ^1^The university of Hong Kong‐ Shen Zhen Hospital, Shen Zhen, China,^2^Guang Dong Provicial People's Hospital, Guang Zhou, China,^3^The university of Liverpool, Liverpool, United Kingdom

**Introduction:** It is unclear whether there is a sex‐difference in the risk of HF among patients with AF. The present study aimed to examine HF risk in females with AF and its temporal trends.

**Methods:** Patients newly diagnosed with AF between 1997 and 2021 were stratified by sex. Annual incidence rate (IR), incidence rate ratio (IRR), and 95% confidence interval (CI) were estimated by Poisson regression analysis. Multivariate adjusted Fine‐Gray regression models were performed to examine the subdistribution hazard ratio (SHR) between females and HF, accounting for all‐cause mortality as a competing risk. The subsequent risk of ischaemic stroke (IS) and all‐cause mortality was further evaluated according to the occurrence of HF at two years following diagnosis of AF.

**Results:** The study cohort consisted of 130132 individuals with newly diagnosed AF (median age 76.6 years, 63181 [48.6%] females). The annual IR (per 1000 person‐years) of HF has declined from 69.6 to 16.7 in females and from 44.5 to 13.7 in males over the years. Nevertheless, females maintained a higher IR of HF than males for the entire follow‐up period (IR: 25.3 vs. 19.3 per 1000 person‐years) and were associated with higher risk of HF than males (IRR [95%CI]: 1.34 [1.31‐1.37]). When compared to males, females were older at the time of the first diagnosis of AF and HF following AF, had higher baseline CHA2DS2‐VASc scores, and were more likely to be prescribed reduced doses of direct oral anticoagulants. Among those with and without incident HF within 2 years following diagnosis of AF, females were consistently associated with higher risks of subsequent IS (SHR [95%CI]: 1.48 [1.41‐1.56]; 1.29 [1.25‐1.35]) and all‐cause mortality (SHR [95%CI]: 1.11 [1.04‐14.19]; 1.21 [1.17‐1.26]), compared to males.

**Conclusions:** Despite the IR of HF in AF declining over the past 20 years, females persist in having higher IR of HF than males, which is associated with a worse prognosis. Older age and inadequate cardiovascular prevention may contribute to the higher HF rate in females with AF.

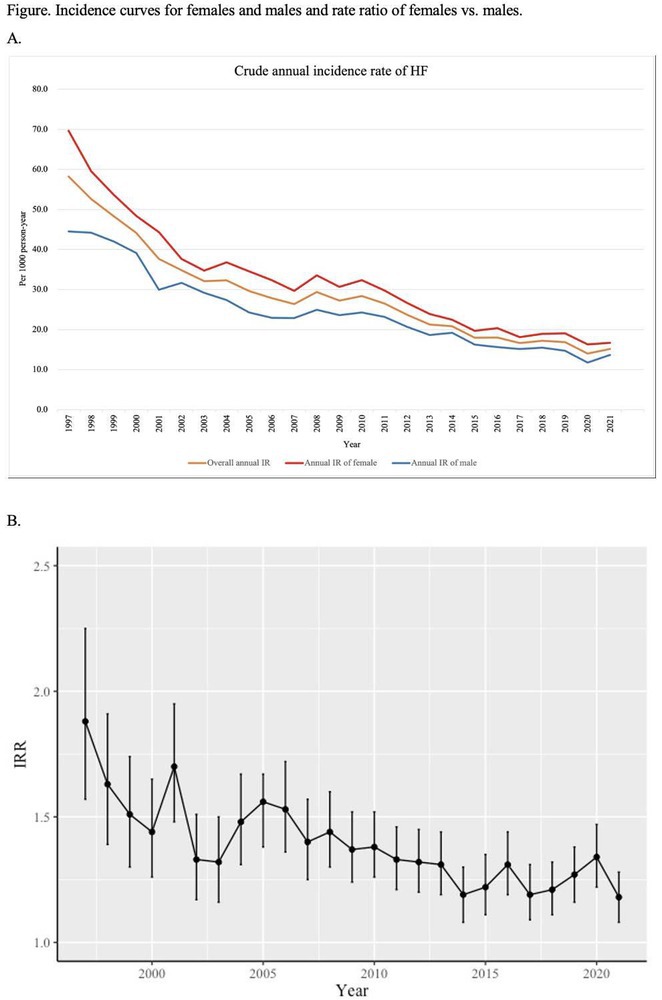

## THE INCIDENCE OF ASYMPTOMATIC CEREBRAL INFARCTION: COMPARISON BETWEEN CRYOBALLOON AND RADIOFREQUENCY ABLATION

### **MASUE YOH** ^1^, MASAHIKO TAKAGI^1^, HIROKI TAKAHASHI^1^, TAKASHI NISHIURA^1^, ICHIRO SHIOJIMA^2^

#### ^1^Kansai Medical University Medical Center, Moriguchi, Japan,^2^Kansai Medical University, Hirakata, Japan

**Introduction:** The management of atrial fibrillation (AF) has emerged pulmonary vein isolation (PVI) by catheter ablation (CA). The silent cerebral infarction (SCI) occurs occasionally during the CA for AF. We prospectively compared the risk of silent cerebral embolism between cryoballoon (CB) and radiofrequency (RF) ablation in patients undergoing the first procedure for all types (paroxysmal, persistent and long‐standing persistent) of AF.

**Methods:** We randomly assigned a total of 374 consecutive AF patients underwent either CB (N=142) or RF (N=232) ablation. Intravenous heparin was administered to keep 300‐400 activated clotting time during the procedure. PVI was considered mandatory for all patients undergoing CA of AF. All patients underwent brain magnetic resonance imaging (MRI) on the next day after the procedure to evaluate SCI.

**Results:** Mean age was 70±10 years, including 142 women (38%). The AF type was paroxysmal in 204 patients (54.5%), persistent in 93 (24.9%), and long‐standing persistent in 77 (20.6%). Mean age was older in the CB group than the RF group (71±11 years vs. 68±9 years, P=0.002, respectively). The median CHADS2 and CHA2DS2‐VASc scores were similar between the CB and RF group (1.6 vs. 1.5, P=0.44, 2.9 vs. 2.7, P=0.17, respectively). The median left ventricular ejection fraction (LVEF) was 63% in the CB group and 62% in the RF group (P=0.33). The incidence of SCI detected on diffusion‐weight imaging (DWI)‐MRI, was observed in 50 (35.2%) in the CB group and 56 patients (24.1%) in the RF group. The incidence of SCI was significantly higher in the CB group than RF group (P=0.02). In the CB group, SCI occurred in patients with higher median CHA2DS2‐VASc score (3.3±1.7 : 2.7±1.7, respectively, P=0.03), and lower LVEF (60.6±11.9% : 64.5±9.3%, respectively, P=0.04) than those without.

**Conclusions:** The CB ablation demonstrated more incidence of SCI detected by brain DWI‐MRI following the CA procedure than the RF ablation. The predictive factors of the occurrence of SCI during the CB ablation were higher CHA2DS2‐VASc score and lower LVEF.

## OBSERVATION OF REPETITIVE FOCAL ACTIVITIES DETECTED BY AUTOMATED ALGORITHM DURING ATRIAL FIBRILLATION AND CONTRALATERAL ATRIAL BURST PACING

### **HIROTAKE YOKOYAMA**, SUSUMU TAKASE, TSUKASA WATANABE, TOMOMI NAGAYAMA, KAZUO SAKAMOTO, SHUNSUKE KATSUKI, HIROYUKI TSUTSUI, KOHTARO ABE

#### Kyushu University, Fukuoka, Japan

**Introduction:** Focal activities (FAs) and rotors are thought to be the underlying mechanisms that perpetuate atrial fibrillation (AF). CARTO FINDER has been clinically used as an automated algorithm system to detect these; however, there is a concern that bystander FAs due to passive endocardial breakthrough may also be detected.

**Methods:** The study was conducted prospectively according to two mapping protocols in AF patients, and the spatial distribution and characteristics of FA sites detected by the automated algorithm were evaluated. In protocol 1, left and right atria (LA/RA) were mapped using CARTO FINDER while contralateral atrial burst (CLAB) pacing of 150 ppm was performed during sinus rhythm. In protocol 2, LA/RA were mapped during AF, and then we mapped the detected FA sites during CLAB pacing was performed. We verified whether FA was also detected during CLAB pacing and compared mean cycle length (mCL) and its standard deviation (Std) between overlapping and non‐overlapping sites. FA sites with 22 or more repetitions in 20 seconds were classified as grade 3.

**Results:** A total of 944 recordings were analyzed. In protocol 1, FA was detected during CLAB pacing in all 12 patients. Grade 3 FA was detected in more than half of the patients except in LA lateral and RA anterior, including LA septum and RAA (100%), and LAA and LA roof (91.6%). In protocol 2, the distribution of FA detected during AF in 9 patients was similar to that during CLAB pacing in protocol 1 except in LA lateral and roof. Among sites with grade 3 FA during AF, mCL was significantly longer and Std was higher at sites with FA during CLAB pacing compared to those without (mCL: 169.8±15.1 vs 161.8 ±12.7ms, p=0.0019; Std: 20.1±7.8 vs 17.1±5.0, p=0.019). However, mCL and Std could not predict FA sites during AF and CLAB pacing (AUC=0.53, 0.50).

**Conclusions:** FAs were frequently detected in LA/RA by automated algorithm during CLAB pacing, which potentially unveil passive endocardial breakthrough sites. The present study clearly demonstrated that care should be taken in interpreting the FAs in most parts of the atrium to find the essential FAs in AF perpetuation.

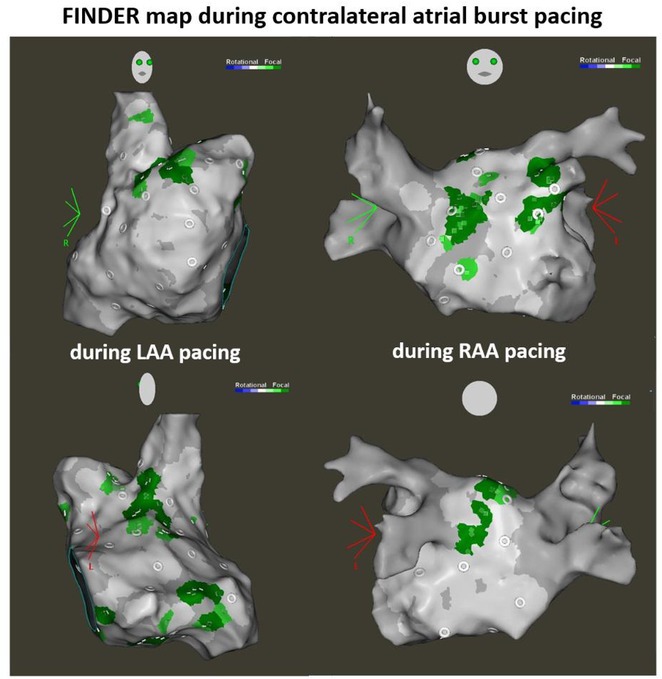

## POSTURE CHANGES DURING SYMPTOM AND ARRHYTHMIA EVENTS RECORDED BY AN INSERTABLE CARDIAC MONITOR

### **DALE YOO** ^1^, RAKESH GOPINATHANNAIR^2^, HARISH MANYAM^3^, DEVI NAIR^4^, FUJIAN QU^5^, JOY WONG^5^, JOHN GILL^5^, FADY DAWOUD^5^, KYUNGMOO RYU^5^, ROY GARDNER^6^, DHANUNJAYA LAKKIREDDY^2^

#### ^1^Heart Rhythm Specialists, Dallas, TX,^2^Kansas City Heart Rhythm Institute, Overland Park, KS,^3^Erlanger Health System, Chattanooga, TN,^4^St. Bernard's Medical Center, Jonesboro, AR,^5^Abbott, Sylmar, CA,^6^Golden Jubilee National Hospital, Clydebank, United Kingdom

**Introduction:** A recently released insertable cardiac monitor (ICM) system measures body posture every 4 minutes and classifies each measurement into 3 categories: lying down, reclined, or upright. The ICM makes on‐demand measurements when symptom or arrhythmia detection is triggered and compares the on‐demand measurements to the most recent automatic measurement to determine if posture remained steady or exhibited a downward change (e.g., upright to lying down). The aim of this study was to evaluate the pattern of body posture changes in symptom and ventricular arrhythmia episodes.

**Methods:** Randomly selected Abbott Assert‐IQ™ ICM devices with the Posture Evaluation feature activated were included in this analysis. Device‐recorded arrhythmia episodes were manually adjudicated. The distribution of 6 posture change categories (upright to lying down, reclined to lying down, upright to reclined, lying down steady, reclined steady, and upright steady) were calculated for each arrhythmia type and each patient symptom entry.

**Results:** A total of 232 patients were included in the analysis. Implant indications were syncope (35%), AF (35%), cryptogenic stroke (15%) and other (15%). During the post implant monitoring window of 67.9 ± 27.2 days, 1610 symptom, 1931 bradycardia, 368 pause, and 2078 tachycardia recordings occurred. Most bradycardia events (80%) and pause events (95%) were under steady lying down or reclined posture, whereas most tachycardia events (76%) were under steady upright posture. The majority of symptom episodes were under steady postures. The highest percentage of symptom episodes was at steady upright posture, suggesting patients were able to tolerate the symptoms to activate a recording.

**Conclusions:** ICM‐based body posture assessment provides additional insight to stratify onset of symptom and arrhythmia episodes beyond rhythm and heart rate. This novel feature can enhance diagnostic and therapeutic management of cardiac arrhythmias related to postural change.

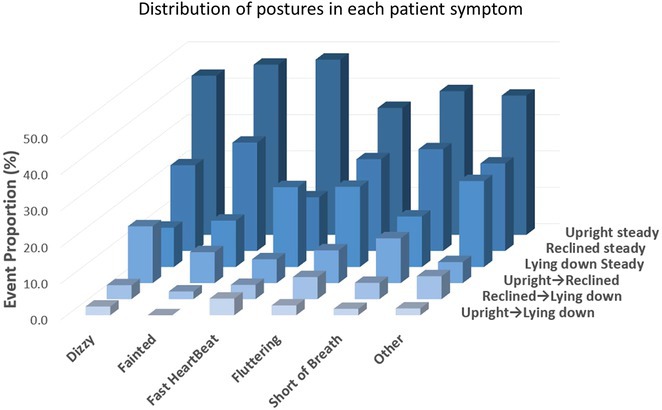

## TWO CASES OF BIATRIAL SINGLE‐LOOP REENTRANT TACHYCARDIA IN PATIENTS WHO UNDERWENT CATHETER ABLATION FOR ATRIAL FIBRILLATION AND FLUTTER

### **WON WOO YOO**, JAE‐SUN UHM, JI‐YOUN CHOI, GON LEE, DUK‐WOO PARK, HANJIN PARK, DAEHOON KIM, HEE TAE YU, TAE‐HOON KIM, BOYOUNG JOUNG, HUI‐NAM PAK, MOON‐HYOUNG LEE

#### Severance hospital, Seoul, Korea, Republic of

**Introduction:** N/A

**Methods:** N/A

**Results:** A 77‐year‐old female complained of palpitation and dyspnea of sudden onset. She underwent RFCA for paroxysmal atrial fibrillation 4months ago. Blood pressure and heart rate were 106/53 mmHg and 67/min. ECG showed atypical atrial flutter(Figure 1A). Because medical therapy was not effective, electrophysiological study was performed. The 3‐dimensional(3D) activation map showed left atrial(LA) macroreentrant tachycardia of which the critical isthmus was the septum. RFCA for the critical isthmus was performed between right inferior pulmonary vein(RIPV) and mitral annulus. During RFCA, tachycardia cycle length was prolonged(240 to 320 ms). The 3D activation map for the second tachycardia showed biatrial single‐loop macroreentrant tachycardia(Figure 1B). Entrainment pacing confirmed both atria were included in the reentry circuit. RFCA was performed at the right atrial(RA) insertion site of the Bachmann's bundle. During RFCA, tachycardia was terminated and not inducible. Tachycardia has not recurred for 3 months.A 54‐year‐old female complained of palpitation and dyspnea. She underwent mitral valve replacement for severe mitral stenosis 3 years ago. Blood pressure and heart rate were 102/62 mmHg and 66/min. ECG showed atypical atrial flutter(Figure 1C). Because medical therapy was not effective, electrophysiological study was performed. The 3D activation map showed LA macroreentrant tachycardia of which the critical isthmus was the septum. RFCA for the critical isthmus was performed between RIPV and mitral annulus. During RFCA, tachycardia cycle length was prolonged(196 to 226 ms). The 3D activation map for the second tachycardia showed biatrial single‐loop macroreentrant tachycardia(Figure 1D). RFCA was performed at RA insertion site of the Bachmann's bundle. During RFCA, tachycardia was terminated and not inducible. Tachycardia has not recurred for 3 years.

**Conclusions:** Biatrial single‐loop reentrant tachycardia can develop after catheter ablation of intraatrial reentrant tachycardia. The critical isthmus can be the RA‐LA connection point.

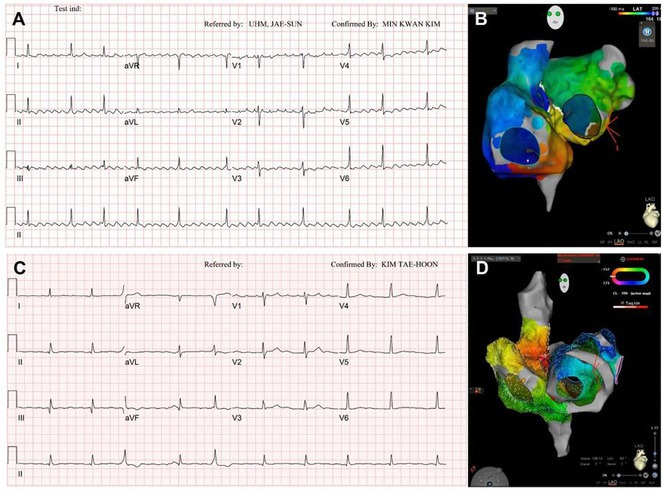

## EVALUATION OF THE ACTUAL IMPLANTATION SITES OF LEADLESS PACEMAKERS USING COMPUTED TOMOGRAPHY (CT) IMAGES

### KENTA YOSHIDA

#### Kurashiki Central Hospital, Kurashiki, Japan

**Introduction:** Although it is recommended that leadless pacemakers <LPs> be implanted in the right ventricular septum, there are few reports on the actual sites where LPs have been implanted. We analyzed patients in whom LPs were implanted into the septum under fluoroscopic guidance and whose implantation sites could be evaluated with CT images post‐operatively.

**Methods:** Between January 2021 and December 2023, 249 patients underwent leadless pacemaker implantation <LPI>, with 110 of these evaluated post‐LPI using CT imaging. Patients were categorized into two groups: those with devices implanted in the septum <septal group> and those with devices implanted outside the septum <non‐septal group>. We analyzed various parameters and complications, including comparisons between the Micra and Aveir subgroups.

**Results:** The study involved 110 patients, with an average age of 84 ± 8 years; 49 were female, weighing on average 54 ± 13 kg, and had a body mass index <BMI> of 22 ± 4 kg/m^2^. The Micra <Medtronic> was implanted in 91 patients, and the Aveir <Abbott> in 19. CT imaging showed that 69 patients had their devices implanted in non‐septal locations <refer to figure>. Patients with non‐septal implants exhibited higher impedance and R‐wave amplitudes than those with septal placements <778 ± 256 vs. 661 ± 186 ohms, p=0.12; 8.3 ± 5.2 vs. 6.7 ± 3.8 mV, p=0.09>. The QRS width during pacing was significantly narrower in the septal group compared to the non‐septal group <146 ± 11 vs. 161 ± 14 ms, p=0.001>. This trend was consistent across both device subgroups (Micra: 146 ± 2 vs. 161 ± 2 ms, p=0.001; Aveir: 140 ± 3 vs. 164 ± 6 ms, p=0.09>. One patient with an Aveir implant in the non‐septal group experienced cardiac tamponade.

**Conclusions:** Despite attempts to implant LPs into the septum guided by fluoroscopic images, more than half of the patients had their devices placed in non‐septal sites. Regardless of the device subgroup, patients with septal implantations exhibited narrower QRS widths compared to those with non‐septal placements.

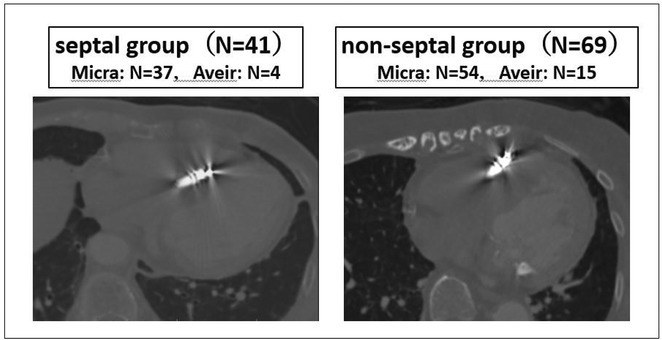

## ASSESSMENT OF ELECTROGRAM AND BI‐ATRIAL LONGITUDINAL STRAIN OF BACHMANN’S BUNDLE PACING USING A UNIPOLAR ATRIAL AMPLITUDE GUIDED APPROACH

### **DAISUKE YOSHIMOTO**, YUICHIRO SAKAMOTO, YUKOU UEMURA, RYO YAMAGUCHI, HIROKAZU NAGANAWA, TAKAHIKO SUZUKI

#### Toyohashi Heart Center, Toyohashi, Japan

**Introduction:** Bachmann's bundle pacing (BBp), which features P‐wave morphology similar to sinus rhythm and shortened P‐wave duration (PWD), was reported to significantly reduce permanent AF recurrences and occurrences compared with right atrial appendage pacing and non‐specific right septal pacing. However, the method of implantation of BBp has not been established.

**Methods:** BBp implantation was attempted using 3830 SelectSecure (Medtronic, Inc) through C315 S4/S5 delivery sheath (Medtronic, Inc) and the high right atrial septum was mapped with a unipolar amplitude recorded at the lead tip to recognize the superior vena cava (SVC)‐right atrium (RA) junction, where the lead was fixed into the RA septum. After implantation, P‐wave during atrial pacing was evaluated to assess if it met the BBp ECG criteria. In successful BBp patients, global longitudinal strain in simultaneous phases of both RA and left atrium (LA) was measured using Philips Ultrasound Workspace 2D Strain in apical 4‐chamber view during atrial pacing. The negative peak of strain rate during atrial systole was the starting point of atrial contraction, and time to peak (TTP) was measured using the end‐diastole of the left ventricle as the zero‐reference time point.

**Results:** From January 2023 to January 2024, 10 patients attempted BBp implantation and all patients achieved successful BBp on the ECG criteria. At implantation, bipolar atrial electrogram amplitude was 2.03±0.93 mV, atrial lead impedance was 632.7±157.6 Ω, and atrial pacing threshold was 0.7±0.2 V at 0.4 ms. The PWD during sinus rhythm was 132.8±10.6 ms, while during BBp it was 108.0±11.9 ms, which was significantly shorter in the BBp (p<0.001). In bi‐atrial longitudinal strain, the median difference in TTP between RA and LA during BBp was 1 ms (IQR 0‐15.25 ms).

**Conclusions:** A unipolar atrial amplitude guided approach using SelectSecure lead and C315 delivery sheath was feasible for BBp implantation. Bi‐atrial longitudinal strain results indicated that BBp achieved favorable inter‐atrial synchrony and might be effective in improving long‐term atrial function and suppressing AF.

## COMPARATIVE STUDY OF HEMOSTASIS DEVICES AFTER ARRHYTHMIA CATHETER ABLATION TREATMENT. PERCLOSE VS. VASCADE

### **MITSURU YOSHINO**, KENTA YOSHIDA, HIRONOBU SUMIYOSHI, RYUKI CHATANI, ATSUSHI SAKATA, HIROSHI TASAKA

#### Kurashiki Central Hospital, Kurashiki, Japan

**Introduction:** Incatheterization procedures for arrhythmia, multiple relatively large diametersheaths exceeding 8 Fr are often inserted through the femoral vein. Ashemostasis devices for femoral vein punctures, there are two types of devices:PERCLOSE, which sutures the vein directly, and VASCADE, which plugs thesubcutaneous tissue at the puncture site with collagen.We performed hemostasis at the femoralvein puncture site using PERCLOSE or VASCADE on patients after ablationprocedures at our hospital and compared their effectiveness and safety.

**Methods:** The efficacy and safety were compared inpatients who underwent ablation for arrhythmia between December 2023 and April2024 and used PERCLOSE or VASCADE to stop bleeding after the procedure. Afterthe ablation procedure, heparin antagonism with protamine sulfate was performedin all cases.After confirming hemostasis using eitherdevice, the patient was kept in a resting position for two hours, and wasallowed to walk after another two hours. We compared the time to achievehemostasis with either hemostasis device starting from the time all ablationdevices were removed from the sheath. We also compared the frequency ofrebleeding after returning to the ward, complications caused by hemostasisdevices, and the number of days until discharge.

**Results:** Therewere 50 patients in the PERCLOSE group and 51 patients in the VASCADE group. The time required toachieve hemostasis was 2.7±1.1minutes per sheath in the PERCLOSEgroup and 4.2±1.4 minutes in the VASCADE group (p<0.01).Rebleeding occurred in 7 patient in the PERCLOSE group and 8 patients in theVASCADE group after returning to the ward (p=1.0). There were no cases in either group where thenumber of days until discharge from hospital increased due to bleeding.

**Conclusions:** Bothdevices can reduce the time to achieve hemostasis. Although the time to hemostasis was slightlylonger in the VASCADE group, there were no significant differences in thefrequency of rebleeding or the number of days until discharge. Since both devices are effective forhemostasis, it was considered effective to use them depending on the case, suchas the diameter of the inserted sheath.

## ATRIAL FIBRILLATION ABLATION USING DIAMONDTEMP ABLATION SYSTEM: IS IT THE PINNACLE OF RADIOFREQUENCY CATHETER ABLATION?

### **KAZUYASU YOSHITANI**, YOSHIHIRO NAKATANI, YUKI YAMANAKA, KAZUNORI TERASHITA, DAISUKE TONOMURA, YOSHIHISA SHIMADA

#### Shiroyama Hospital, Habikino City, Japan

**Introduction:** Although the DiamondTemp (DT) ablation system lacks the ability to display contact force, it is expected to achieve high ablation efficiency and enable shorter ablation procedure durations. In this study, we report on the initial experience with DiamondTemp in pulmonary vein isolation (PVI) and linear ablation.

**Methods:** Atrial Fibrillation (AF) ablation using the DT system was performed on 83 patients with a point‐by‐point technique. The primary strategy was PVI, with ablation settings of a catheter‐tip temperature limit of 60°C, a temperature‐controlled power of 50W, and an application duration of 12 seconds. Aditionaly, for long‐standing persistent AF cases, linear ablations consisting of roof and mitral isthums(MI) lines, were performed, with each lesion applied for 15‐18 seconds. If endocardial MI ablation failed, ablation within the coronary sinus was performed with settings of 40W and 55°C. Following PVI, an adenosine triphosphate (ATP) test was performed to identify and ablate any gaps. After an induction study, extra‐pulmonary vein triggers were ablated as needed.

**Results:** All PVIs were performed successfully, with a first‐pass isolation rate of 50.6%. Notably, the anterior ridge of the left PV was the most frequent gap site (14.5%). Additionally, the non‐reconduction rate after the ATP test for each patient was 85.5%. Roof line ablation was successful in 95.4% of cases, whereas the success rate for MI ablation was 68.2%. The overall procedure time for all cases was 99.2 ± 29.6 minutes. For cases where only PVI was performed, the procedure time was 82.6 ± 15.3 minutes. Regarding complications, there was one case of transient ischemic attack.

**Conclusions:** Ablation using the DT system for AF ablation is promising due to its short procedure durations. However, to improve efficacy, further adjustments in application duration and other parameters might be necessary.

## A CASE OF CARDIAC AMYLOIDOSIS WITH RECURRENT SYNCOPE

### **HONGZHAO YOU**, YING WU, XIAOGANG GUO, WEI WANG, ZHE LI, RUOHAN CHEN, YAN DAI, KEPING CHEN

#### Fuwai Hospital, Beijing, China

**Introduction:** The manifestations of cardiac amyloidosis (CA) can be varied, and syncope is relatively rare. We report a 56‐year‐old male presented with repeated syncope and finally diagnosed as CA.

**Methods:** N/A

**Results:** On admission, his BP was 106/74mmHg, HR 59 bpm, without a significant change from supine to standing position. His HR dropped off 38bpm when he presented an amaurosis. His NT‐proBNP levels of 1216 pg/mL and cTNT levels of 0.049 ng/mL. ECG showed sinus bradycardia and CRBBB (Figure A). Echocardiography revealed enlargement of both atria, thickening of both ventricles, an interventricular septal thickness of 14 mm and LVEF of 52%. A 24‐hour Holter monitor showed sinus bradycardia with a minimum HR of 32 bpm, sinus arrest (longest RR interval 2.98 seconds) and second degree sinoarital block. Cardiac MRI revealed a prominent dust‐like enhancement mainly beneath the endocardium of LV on LGE scanning (Figure B). Serum and urine immunofixation electrophoresis (SUIE) showed free λ chains positive. Amyloidosis was diagnosed by an abdominal fat biopsy (Figure C). The patient was treated with anti‐HF drugs (torasemide, spirolactone and dapagliflozin) and BMD chemotherapy (bortezomib, melphalan and dexamethasone). After detailed discussion with the patient and his family member, he received implantation of a dual chamber defibrillator (Biotronik, Iforia 7DR‐T). During the follow‐up of 2 year, the patient reported no further syncope episode nor VT/VF event by pacemaker interrogation. His serial SUIEs were negative, cTNT 0.003ng/mL and NT‐proBNP level 950pg/mL, indicating an organ response.

**Conclusions:** Syncope in CA often attributed to ventricular tachyarrhythmias or high‐degree AVB rather than sick sinus syndrome. Greater attention should be paid to the untypical symptom, thereby prompt recognition of CA in patients investigated for syncope. AL‐type CA patients who met the criteria might be beneficial from ICD implantation for primary prevention, such as NYHA class I‐III, NT‐proBNP<8500pg/ml and systolic BP>90mmHg and a life expectancy for more than one year. An individualized comprehensive management when the above evidence was applied at the patient's will could yield a favorable result.

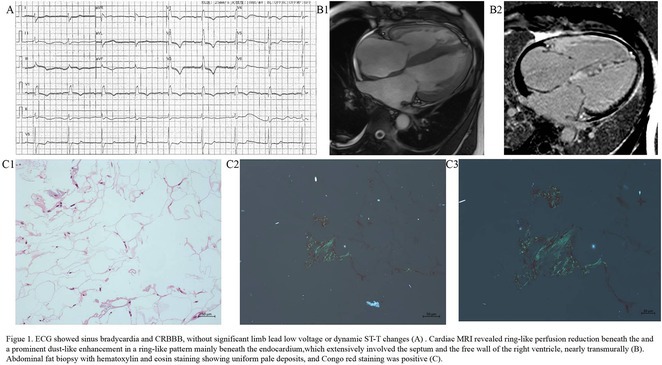

## INEFFECTIVE AND PRECARIOUS MANAGEMENT STRATEGIES OF VENTRICULAR TACHYCARDIA RECORDED ON AN IMPLANTABLE CARDIAC DEFIBRILLATOR

### **JULIET YOUNG**, ALEX DASHWOOD, HUI CHEN HAN, MOHAMMAD ALASTI, DAVID ADAM

#### Monash ‐ Victorian Heart Hospital, Clayton, Australia

**Introduction:** Interrogation of an implantable cardiac defibrillator (ICD) revealed regular artifacts across multiple electrogram (EGM) channels on a stored ventricular fibrillation episode. Further investigation revealed that external anti‐tachycardia pacing (ATP) via defibrillation pads, was unsuccessfully attempted to terminate ventricular tachycardia.

**Methods:** N/A

**Results:** ATP requires rapid pacing at a cycle length shorter than the VT, to penetrate a circuit and terminate reentrant ventricular tachycardia. There is lack of evidence to support the efficacy and safety of external ATP. Furthermore, in patients with ICDs, this intervention could trigger inappropriate detection of a ventricular arrhythmia, potentially leading to unnecessary shocks and device‐triggered ATP. Such events might escalate into a more critical arrhythmia. Fortunately, in this case example, no immediate adverse effects resulted from the external ATP.

**Conclusions:** This case study highlights the importance of involving specialised physicians and experienced cardiac device physiologists in the management of ICDs and ATP.

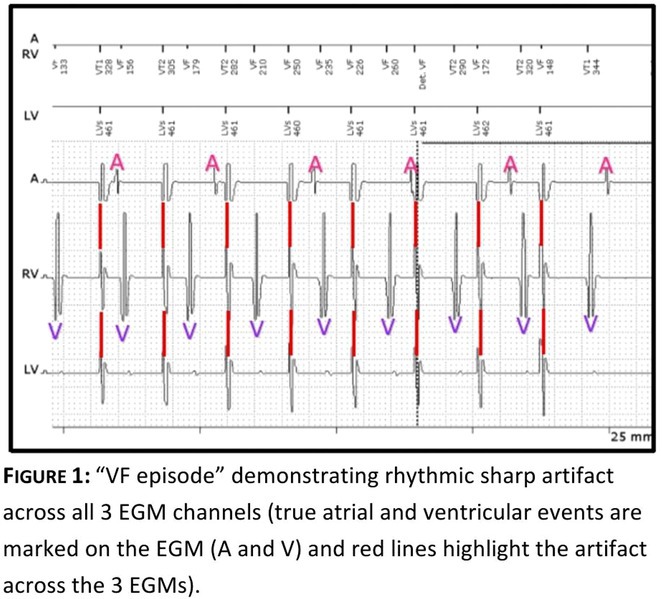

WAIT A MINUTE! AN IN‐DEPTH REVIEW OF THE MINUTE VENTILATION ALGORITHM

**JULIET YOUNG**, HUI CHEN HAN, MOHAMMAD ALASTI;

Monash ‐ Victorian Heart Hospital, Clayton, Australia.

**Introduction:** Review of two separate patients, each with a dual‐chamber pacemaker, revealed markedly different outcomes due to the functioning of the minute ventilation (MV) and signal artifact monitor (SAM) algorithms. Case study 1 illustrates the underperformance of the SAM algorithm, while case study 2 demonstrates scenarios in which the SAM algorithm exhibits excessive response.

**Methods:** N/A

**Results:** Case study 1 presented with dyspnoea and rapid ventricular pacing, attributed to excessive minute ventilation (MV) activity caused by a fractured atrial lead. The SAM algorithm failed to appropriately detect the evident atrial fracture noise, thus it did not disable MV, resulting in frequent and unnecessary periods at the maximum sensor rate. In Case Study 2, routine device interrogation revealed a false positive detection by the SAM algorithm. As a consequence, MV was inappropriately deactivated, leaving the patient without an active rate‐responsive algorithm. Unfortunately, disabling the SAM algorithm for this patient would expose them to other potential complications of the MV algorithm, such as pacing inhibition due to MV signal oversensing. Thus, it was deemed necessary to leave the SAM algorithm active in this instance.

**Conclusions:** These cases highlight the complexities associated with the MV and SAM algorithm functionalities and underscore their limitations. Future advancements in algorithm design could be explored to address the current device functionalities' shortcomings.

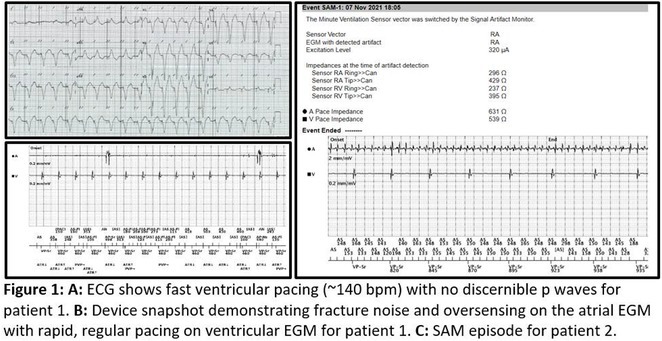

## EFFECT OF EMPAGLIFLOZIN ON ATRIAL FIBRILLATION BY ATTENUATING CARDIAC FIBROSIS VIA NA_V_1.5 REGULATION IN β‐ADRENERGIC STIMULATED KK/HIJ MOUSE

### **BYEONGIL YU** ^1^, MIN‐JU LEE^1^, JONG‐IL CHOI^1,2^

#### ^1^Department of Biomedical Sciences, Korea University College of Medicine, Seoul, Korea, Republic of,^2^Division of Cardiology, Department of Internal Medicine, Korea University College of Medicine and Korea University Anam Hospital, Seoul, Korea, Republic of

**Introduction:** Cardiac fibrosis is a disease in which extracellular matrix (ECM) such as collagen is accumulated in the heart. It is known that excessive accumulation of ECM causes myocardial stiffness and eventually interferes the electrical conduction system causing atrial fibrillation (AF). Empagliflozin (EMPA) is a drug for type 2 diabetes mellitus (T2DM) patients and known to lower glucose resorption by inhibiting sodium‐glucose co‐transporter 2 (SGLT2). Recent studies have reported that EMPA have an off‐target effect in which overall cardiac function is improved regardless of the presence or absence of T2DM. EMPA reported to alleviate cardiac fibrosis, however the exact mechanism is not known. We aimed to investigate the regulatory mechanism of cardiac fibrosis by EMPA.

**Methods:** The animal model with cardiac fibrosis was made by inducing β‐adrenergic stimulation through osmotic pump in KK/HIJ mouse. Electrophysiological, molecular biological and histological experiments were conducted in saline or isoproterenol (ISO, 30 mg/kg)‐induced C57BL/6 mouse and KK/HIJ mouse following intraperitoneally injection of vehicle (DMSO) or EMPA (10 mg/kg).

**Results:** After ISO induction in the KK/HIJ mouse (K‐I), 1) cardiac hypertrophy and severe cardiac fibrosis were occurred compare to other group, 2) the ejection fraction and fractional shortening were decreased, 3) QRS width and QTc were significantly prolonged and induced‐AF and AF duration also were increased, 4) the action potential duration (APD) was prolonged, 5) *I*_Na,Peak_ and *I*_Na,Late_ were increased, 6) Spontaneous Ca^2+^ releases were increased in K‐I group. After EMPA injection in the ISO‐induced KK/HIJ mouse (K‐I‐E), 1) Cardiac fibrosis was reduced although there was no change in cardiac hypertrophy, 2) ejection fraction and fractional shortening were increased, 3) AF duration was decreased although there was no change in induced‐AF, and QRS width and QTc were shortened in K‐I‐E group.

**Conclusions:** This finding suggests that EMPA is a potential antiarrhythmic drug for rhythm control in patients with AF, not directly affecting ion channel blocking.

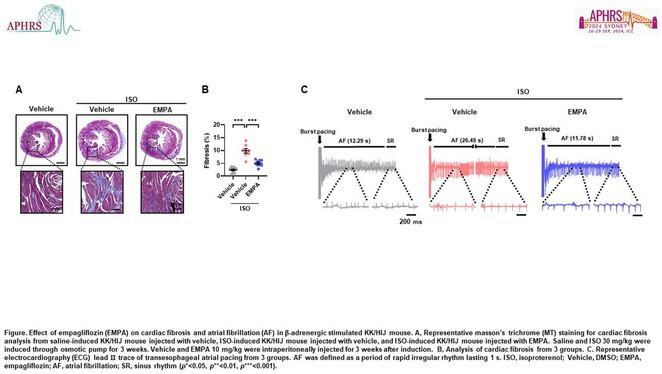

## ATRIAL FIBRILLATION ABLATION EXPERIENCE IN GOVERNMENT TERTIARY CENTRE, KLANG VALLEY MALAYSIA FOR 2023

### **ONG YU YING**, NOR HALWANI HABIZAL, EUNICE JING MUN LAI, POONAM DEVI A/P ANOOP KUMAR, ABDUL RAQIB ABD GHANI

#### Hospital Sultan Idris Shah, Serdang, Selangor, Malaysia

**Introduction:** Catheter Ablation (CA) is proven to be a superior option compared to medical therapy in Atrial Fibrillation. We wish to report the efficacy of both strategies‐ Wide Anterior Circumferential Ablation (WACA) vs Cryo Balloon Ablation (CBA) for Pulmonary Vein Isolation (PVI) implemented in our hospital in 2023.

**Methods:** We performed a retrospective study of 40 patients in our hospital who underwent CA (from Feb 2023 until Nov 2023) of which 16 patients underwent WACA under General Anaesthesia and 24 patients CBA under conscious sedation. Mean age was 59 years old. 22 patients were male & 18 female. Paroxysmal AF (n=18) & persistent AF (n=22). Mean LA size was 3.8cm. 39 patients were prescribed oral anticoagulation if their CHADS2VASc score is >/=0 and 1 patient was on LAA occluder.

**Results:** Of all 16 WACA, 2 unfortunately deceased due to catheter related complication and another hospital acquired infection. 1 of 24 CBA was aborted due to reversible phrenic nerve injury.

Recurrence rate after 6 months for WACA was 50% compared to CBA 37.5%.

All patients in sinus rhythm after CA reported symptoms relief and were able to stop anti‐arrhythmic medications.

**Conclusions:** In conclusion, both procedures were widely carried out almost concurrently in 2023, CBA for atrial fibrillation was found to offer an effective, safer, less operator‐dependent, alternative mean of achieving PVI with lesser recurrence rate compared to WACA. However, as WACA is still the cornerstone of the therapy and the only intervention with excellent supporting evidence, longer operator experience and skills for WACA learning curve were required for further comparison of ablation outcome in line with current best practice in our centre.

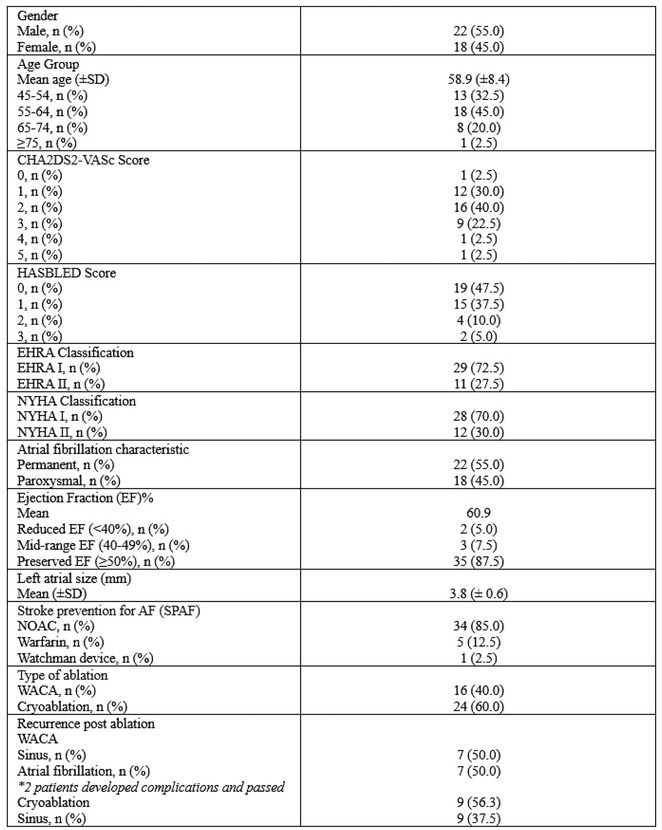

## THE POINT OF NO RETURN: IMPROVISATION SAVES THE DAY

### **ONG YU YING**, SIOW YOON KEE, ABDUL RAQIB ABDUL GHANI, NOR HALWANI HABIZAL

#### Hospital Sultan Idris Shah, Serdang, Selangor, Malaysia

**Introduction:** 83‐year‐old gentleman with a history of Hypertension was implanted a Dual Chamber Pacemaker for Sick Sinus Syndrome since 2014. Unfortunately just 1‐month after Pulse Generator (PG) change in February 2022, he required prolonged hospital stay from pocket infection and septicaemia. The new PG had to be removed but old leads were capped and closed (due to unavailability of extraction tool). Right Internal Jugular Temporary Pacemaker was inserted as bridging to successful MICRA insertion in the same admission. Throughout the year, patient had multiple admissions requiring antibiotics for recurrent discharge from previous pre‐pectoral incision site and was eventually re‐admitted for leads extraction in August 2023. Difficulty was encountered during old Right Ventricular lead extraction. However, it was overcome using TIGHTRAIL system with improvisation.

**Methods:** 12F sheath was placed via Right Femoral Vein and safety guidewire inserted to standby Bridge Occlusion Balloon (SVC tear prophylaxis). RA lead was removed with usual method by Lead Locking Device and Tightrail System. Difficulty encountered due to unravelling old RV lead insulation upon removal of stuck straight stylet. However, it was successfully extracted using improvised method by tying the folded proximal end of the stuck straight stylet with the “makeshift” lead locking stylet together as single unit using suture silk.

**Results:** Patient was discharged well after completion of IV antibiotics and pocket was dressed daily and left healing by secondary intention. Subsequent follow‐up revealed healed scar with no discharge.

**Conclusions:** Recurrent infection remains as part of the challenge in CIED implantation. Besides antibiotics administration and MICRA insertion, lead extraction serves as an integral skill for implanter to master in certain centre. It is vital to watch for unintended forceful traction that will commonly result in unravelling of the old lead. Besides that, the reported makeshift manoeuvre may be attempted in a compromised lumen with a stuck stylet. In this case, we also demonstrated the importance of complete device and leads extraction in the same setting as part of the CIED infection management.

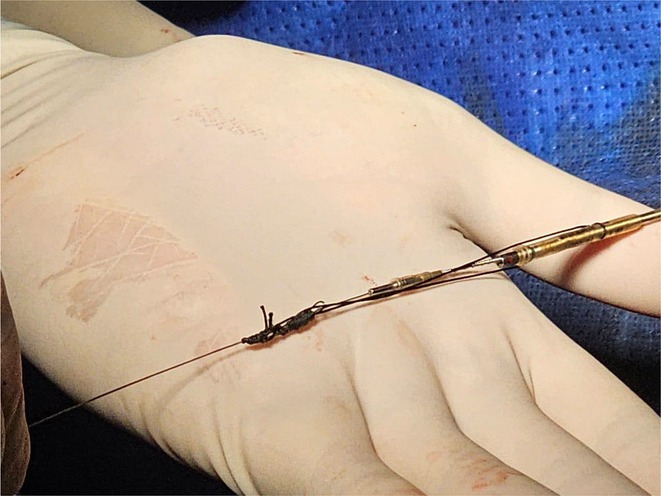

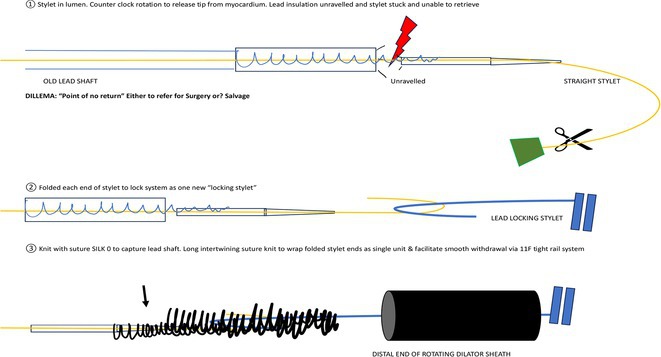

## A CASE OF TACHYCARDIA‐BRADYCARDIA SYNDROME IN A PATIENT WITH CHRONIC MYELOGENOUS LEUKEMIA ON IMATINIB‐CASE REPORT

### **CYRUS VIKTOR ZALAMEA**, ASTRID AMADOR, JENNIFER JEANNE VICERA

#### University of Santo Tomas Hospital, Manila, Philippines

**Introduction:** Chronic myelogenous leukemia (CML) is a hematopoietic stem cell disorder where patients are usually asymptomatic. Currently, there are no known cardiac arrhythmias which are related to CML. A first line agent used in the treatment of CML is imatinib, a first‐generation tyrosine kinase inhibitor. It is known to have cardiotoxic side effects such as development of heart failure and prolongation of QT interval. Development of tachycardia‐bradycardia syndrome hasn’t been reported as an adverse effect of imatinib.

**Methods:** N/A

**Results:** We report a case of a 38‐year‐old female, diagnosed as a case of CML and maintained on imatinib, who consulted due to recurrent syncopal episodes. She has no known risk factors predisposing her to developing cardiac arrhythmias. Her 24‐hour Holter monitoring showed sinus rhythm with episodes of narrow QRS tachycardia (Fig. 1A) and episodes of long pauses secondary to sinus pause with slow junctional rhythm with longest duration of 4.8 secs (Fig. 1B & Fig 1C). The patient was noted to have a seizure episode which coincided with an episode of bradycardia. These findings were consistent with sinus nodal dysfunction (tachycardia‐bradycardia syndrome). Further workup revealed unremarkable echocardiography. Dual chamber pacemaker was implanted to address her arrhythmia.

**Conclusions:** This is perhaps the first report of sinus nodal dysfunction (tachycardia‐bradycardia syndrome) in a patient with CML treated with imatinib. Further investigation may be warranted to confirm association of arrhythmia arising from either CML or the use of imatinib. Permanent pacemaker implantation is necessary until further cases are seen which may further give information on the correlation of imatinib and CML on the development of tachycardia‐bradycardia syndrome. Electrophysiologic studies +/‐ ablation therapy for the tachyarrhythmia should also be considered.

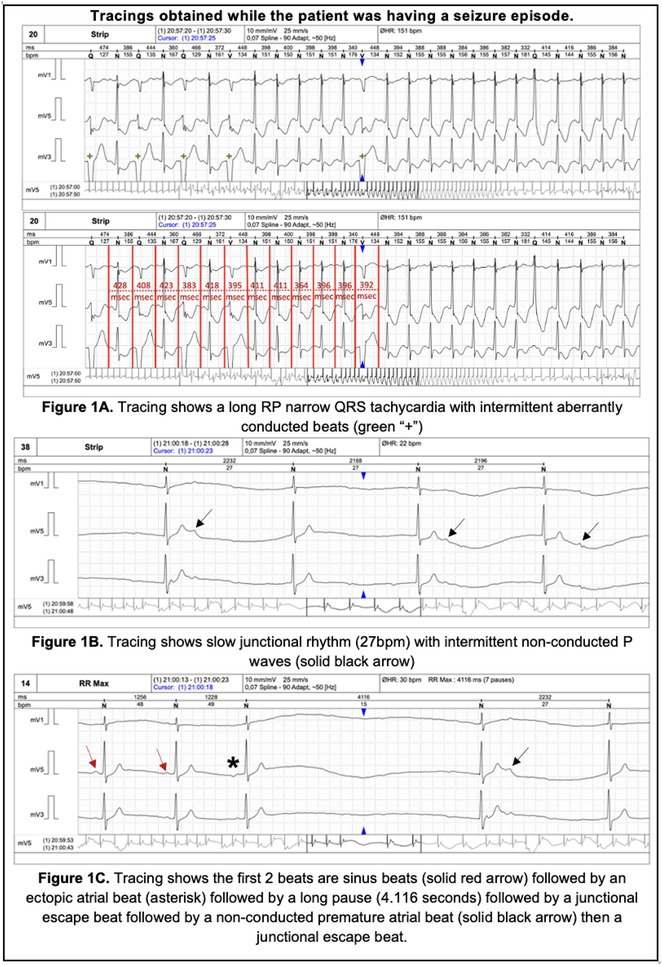

## CONCEALED CHAOS: UNMASKING A CASE OF COVERT VENTRICULAR TACHYCARDIA

### **CYRUS VIKTOR ZALAMEA**, MARCELLUS RAMIREZ, JENNIFER JEANNE VICERA

#### University of Santo Tomas Hospital, Manila, Philippines

**Introduction:** What is the ECG reading? Why are there varying QRS morphologies? Why are there varying PR intervals?

**Methods:** N/A

**Results:** A 70‐year‐old female presented with subarachnoid hemorrhage secondary to a fall for which she underwent emergency decompressive hemicraniectomy for evacuation of hematoma. During admission, she developed septic shock secondary to nosocomial pneumonia and urinary tract infection. This electrocardiogram (ECG) tracing was taken during hypotension.

**Conclusions:** This tracing has a constant atrial cycle length of 600 msec and variable ventricular cycle length of 530‐670 msec. The P wave is upright in leads I, II, III and V1 to V6 and occurs regularly at a rate of 100 bpm. The QRS complexes vary in rate and morphology. Sinus beats which were able to conduct down the AV node then to the ventricles are seen in beats 3, 9, and 16. With the other remaining beats, there are varying ventricular cycle lengths, varying QRS morphologies, and varying PR intervals. This can be explained by an intermittently faster ventricular beats which conducts retrogradely competing with the atrial signal in depolarizing the ventricle, causing intermittent fusion and capture beats. Beats 3, 9, and 16 are sinus beats which were able to conduct down the AV node then to the ventricles, these are consistent with capture beats. The remaining beats were sinus beats which were able to depolarize down; however, on the way down the beats meet and fuse with an infrahisian beat‐these are the fusion beats. Due to the presence of capture and fusion beats, this tracing is consistent with a ventricular tachycardia.

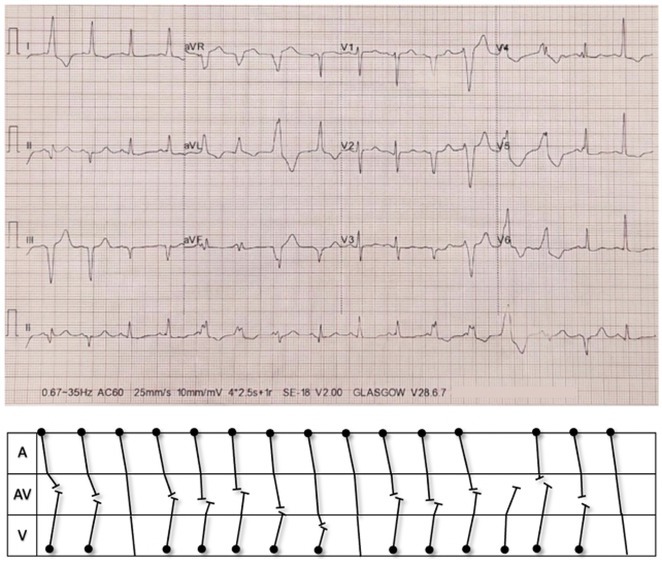

## CATHETER ABLATION VERSUS ANTIARRHYTHMIC DRUG THERAPY FOR SUSTAINED VENTRICULAR TACHYCARDIA IN PATIENTS WITH HYPERTROPHIC CARDIOMYOPATHY

### **FENGXIANG ZHANG** ^1^, YAN DONG^1^, XUDONG SONG^2^, DAN BO^1^, HONGTAO WANG^3^, BO YANG^4^, NISHANT YADAV^1^, QIUSHI CHEN^1^

#### ^1^The First Affiliated Hospital of Nanjing Medical University, Nanjing, China,^2^Department of Cardiology, ZhuJiang Hospital of Southern Medical University, Guangzhou, China,^3^Department of Cardiology, the Second Affiliated Hospital of Xi’an JiaoTong University, Xi’an, China,^4^Department of Cardiology, the First Hospital of Lanzhou University, Lanzhou, China

**Introduction:** Ventricular tachycardia (VT) is the primary cause of sudden cardiac death in patients with hypertrophic cardiomyopathy (HCM). However, the strategy for VT treatment in HCM patients remains unclear. This study is aimed to compare the effectiveness of catheter ablation versus antiarrhythmic drug (AAD) therapy for sustained VT in patients with HCM.

**Methods:** A total of 28 HCM patients with sustained VT at 4 different centers between December 2012 and December 2021 were enrolled. Twelve underwent catheter ablation (ablation group) and sixteen received AAD therapy (AAD group). The primary outcome was VT recurrence during follow‐up.

**Results:** Baseline characteristics were comparable between two groups. After a median follow‐up of 27 months, the primary outcome occurred in 35.7% of the ablation group and 90.6% of the AAD group (hazard ratio [HR], 0.29 [95%CI, 0.10 ‐ 0.89]; P = 0.021). No differences in hospital admission due to cardiovascular cause (25.0% vs. 71.0%; P = 0.138) and cardiovascular cause‐related mortality/heart transplantation (9.1% vs. 50.6%; P = 0.551) were observed. However, there was a significant reduction in the composite endpoint of VT recurrence, hospital admission due to cardiovascular cause, cardiovascular cause‐related mortality, or heart transplantation in ablation group as compared to that of AAD group (42.9% vs. 93.7%; HR, 0.34 [95% CI, 0.12 ‐ 0.95]; P = 0.029).

**Conclusions:** In HCM patients with sustained VT, catheter ablation reduced the VT recurrence, and the composite endpoint of VT recurrence, hospital admission due to cardiovascular cause, cardiovascular cause‐related mortality, or heart transplantation as compared to AAD.

## SUCCESS RATE OF ABLATION IN ATRIAL FIBRILLATION PATIENTS WITH RIGHT ATRIAL APPENDAGE TRIGGERS

### **FENGXIANG ZHANG**, SHOUMEI SHEN, DAN BO, YAN DONG, QIUSHI CHEN

#### The First Affiliated Hospital of Nanjing Medical University, Nanjing, China

**Introduction:** To explore the clinical features of right atrial appendage (RAA) triggered atrial fibrillation (AF) patients and the outcome of ablation on RAA triggers.

**Methods:** This study was a retrospective cohort study of patients with AF who underwent catheter ablation in the Department of Cardiology, the First Affiliated Hospital of Nanjing Medical University from October 2020 to October 2023. All patients underwent circumferential pulmonary vein isolation (CPVI) guided by 3D mapping system, and ablation was performed when non‐pulmonary vein triggers were detected. Patients with mapped RAA triggers were included in the RAA trigger group, and the rest were non‐RAA trigger group. The two groups were matched 1:1 propensity score matching (PSM).

**Results:** After PSM, 22 patients were included in the RAA trigger group and 22 patients in the non‐RAA trigger group. There were no significant differences in age, sex, comorbidities, AF type and AF course between the two groups. The right atrial volume (RAV) in the RAA‐triggered group was significantly higher than that in the non‐RAA‐triggered group [82.10 (68.30, 94.39) *vs*. 66.09 (50.72, 81.04) ml, P = 0.03]. The total operation time of RAA‐triggered group was also higher than that of non‐RAA‐triggered group [190.00 (172.50, 237.50) *vs*. 170.00 (150.00, 180.00) min, P < 0.01]. After 14.00±1.25 months follow‐up, there was no significant difference in the recurrence rate of AF between the two groups (P = 0.34).

**Conclusions:** AF patients with right auricular trigger have relatively larger RAV and longer operation time, but their long‐term prognosis is comparable to that of patients with non‐RAA trigger.

## THE OUTCOME OF CATHETER ABLATION FOR ATRIAL FIBRILLATION IN PATIENTS WITH CHRONIC KIDNEY DISEASE

### **NING ZHANG**, LINGJIE WANG, YUN XIE, YUE WEI, CHANGJIAN LIN, QINGZHI LUO, YANGYANG BAO, LIQUN WU

#### Ruijin Hospital, Shanghai Jiaotong University School of Medicine, Shanghai, China

**Introduction:** The efficacy and safety of catheter ablation for atrial fibrillation (AF) in patients with chronic kidney disease (CKD) remain to be elucidated.

**Methods:** Paroxysmal and persistent AF patients with AF and CKD who underwent catheter ablation in Ruijin Hospital from 2019 to 2021 were consecutively enrolled and underwent pulmonary vein isolation (PVI) or PVI‐plus (PVI combined with extra‐pulmonary vein triggers, linear, or substrate modification ablation); two groups were categorized according to eGFR: mild CKD (CKD stage 2, CKD2) and moderate‐to‐severe CKD (CKD stages 3‐5, CKD3‐5). The primary clinical endpoint is the rate of recurrence of AF at one‐year follow‐up (≥30s of any episode of atrial tachycardia after blanking period); Safety endpoints comprise perioperative events and long‐term events.

**Results:** A total of 156 patients with CKD were enrolled including 132 cases of CKD2, 24 cases of CKD3‐5 (including 5 cases of ESRD). Most baseline characteristics were not significantly different between two groups. However, CKD3‐5 patients had a higher prevalence of heart failure and hypertension, as well as a higher proportion of RAAS inhibitors application. During the ablation procedure, significantly increased incidence of PVI‐plus procedures was found in CKD3‐5 group (39.4% vs. 65.2%,P=0.021). Three perioperative safety events (1 pericardial effusion, 1 sedation‐related psycho‐neurologic symptom, 1 gastrointestinal symptom) and 1 (pericardial effusion) occurred in CKD2 and CKD3‐5 group respectively. Follow‐up at 12 months revealed no significant difference in the rate of recurrence of atrial fibrillation between the two groups (14.7% vs. 17.9%, P=0.689), no significant difference in the rate of long‐term safety events, and no further deterioration of heart failure or renal function in CKD3‐5 group. Logistic regression analysis revealed that the main factors affecting the recurrence of AF were left atrial size and AF type.

**Conclusions:** Catheter ablation can be used effectively and safely for rhythm control in AF patients combined with moderate‐to‐severe CKD.

## IMPACT OF LEFT ATRIAL LATERAL RIDGE SHAPE ON THE LONG‐TERM OUTCOME OF RADIOFREQUENCY CATHETER ABLATION FOR ATRIAL FIBRILLATION

### GUOQIANG ZHONG

#### The First Affiliated Hospital of Guangxi Medical University, Nanning, China

**Introduction:** The left atrial lateral ridge (LLR) has even been described as the most challenging site on ablation for atrial fibrillation (AF). The impact of LLR on high‐power short‐term radiofrequency ablation (HPSD‐RFA) is still unknown.

**Methods:** The morphological characteristics of LLR were analyzed. Bilateral HPSD‐RFA of LLR by the left pulmonary vein (LPV) side and left atrial appendage side or Marshall vein ethanol infusion (EI‐VOM) were used as alternative optimized strategies. The efficacy of two strategies was evaluated, the main endpoint was recurrence of atrial tachyarrhythmias (ATs) lasting more than 30S.

**Results:** In conventional ablation, 34 of 117 patients had recurrent ATs during follow‐up. Multivariate logistic regression analysis showed that LLR thickness and persistent AF remains an independent risk factor for ATs recurrence. Patients with LLR >5.05mm were more prone to ATs (sensitivity 52.9% and specificity 81.9%). 61 paroxysmal AF patients underwent bilateral HPSD‐RFA of LLR, 54 patients (88.5%) achieved complete first‐pass isolation of LPV, the main acute reconnection was in the posterior walls (71.4%). It did not significantly reduce the ATs recurrence compared to conventional ablation (11.48% VS. 18.33%, P=0.289). 112 persistent AF patients underwent bilateral HPSD‐RFA of LLR(n=52) or EI‐VOM (n=60), no significant difference in ATs recurrence between the two group (P=0.427). 48 patients (80%) successfully performed EI‐VOM. The LIPV‐LAA distance was the only independent risk factor (OR: 1.63, 95% CI: 1.131‐2.348, P=0.009), it > 12.65mm had a sensitivity of 75.0% and a specificity of 85.1% in predicting EI‐VOM failure.

**Conclusions:** AF patients with LLR >5.05mm are more likely to have ATs after HPSD‐RFA. Bilateral HPSD‐RFA of LLR can improve success rate for AF ablation and not inferior to EI‐VOM. It is reasonable as an alternative ablation who cannot perform EI‐VOM or have a low success rate of EI‐VOM with LIPV‐LAA distance >12.65mm.

## ADDRESSING OESOPHAGEAL INJURY RISKS IN CATHETER ABLATION OF ATRIAL FIBRILLATION: EVALUATING THE IMPACT OF OESOPHAGEAL TEMPERATURE PROBES

### SHAYEKH ABEDIN^1^, TONY BARRY^1^, STUART THOMAS^1^, PIERRE QIAN^1^, **JULIA ZHOU** ^2^

#### ^1^Westmead Applied Research Centre, Sydney, Australia,^2^Westmead Hospital, Sydney, Australia

**Introduction:** Catheter ablation procedures targeting the posterior left atrium pose a potential risk of oesophageal injury. Oesophageal temperature probes (OTP) equipped with multiple electrodes serve a pivotal role in overseeing and mitigating oesophageal overheating. This study seeks to scrutinize the temperature variations observed in oesophageal tissue and OTPs concerning two key factors: 1) high power, short duration (HPSD) versus low power, long duration (LPLD) ablation, and 2) uninsulated versus insulated OTPs.

**Methods:** Employing a gel and thermochromic phantom model mimicking the left atrium and oesophagus, we analyzed heating kinetics. An OTP commonly used in clinical settings was inserted into the simulated oesophagus to monitor temperatures during ablation. Comparative analyses were conducted between irrigated ablations at 30W/20s (LPLD) and 50W/5s (HPSD), alongside lesion temperature mapping via digital photography.

**Results:** HPSD ablation exhibited a notably lower peak esophageal temperature (Tpeak) (39.17 ± 0.06°C vs. 41.73 ± 0.12°C, p < 0.0001) and demonstrated a reduced discrepancy in OTP measurements (5.13 ± 0.23°C, p < 0.0001 vs. 7.20 ± 0.14°C, p < 0.0001) (Panel A, B). Moreover, HPSD ablation resulted in shallower lesions compared to LPLD (2.49 ± 0.04 mm vs. 3.74 ± 0.23 mm, p = 0.0008). Particularly, LPLD ablations exhibited heightened heating around the OTP, with significant overshoots above the 38.5°C threshold (3.23 ± 0.16°C vs. 0.67 ± 0.06°C, p < 0.0001), which insulation effectively mitigated (41.73 ± 0.12°C vs. 39.13 ± 0.25°C, p < 0.0001) (Panel B, C).

**Conclusions:** LPLD ablation poses a higher risk of oesophageal heating and poses challenges in OTP temperature monitoring. The insulation of OTP emerges as a promising strategy to alleviate oesophageal heating during ablation procedures.

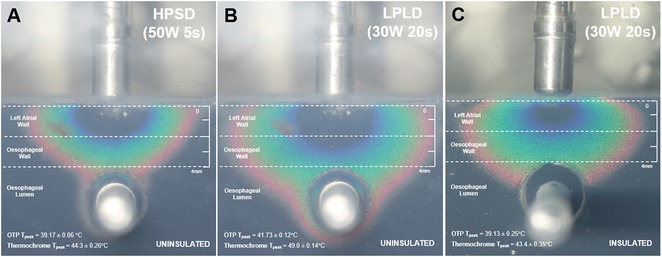

## FLUOROLESS PULMONARY VEIN ISOLATION USING FARAWAVE

### **YEHUAN ZHOU** ^1^, STUART THOMAS^1,2^, PIERRE QIAN^1,2^

#### ^1^Westmead Hospital, Westmead, Australia,^2^University of Sydney, Sydney, Australia

**Introduction:** Pulsed‐field ablation (PFA) for AF was associated with longer fluoroscopy times than radiofrequency ablation. We describe our fluoroless workflow using the FARAWAVE system.

**Methods:** Transoesophageal echocardiography (TOE) and 3D electroanatomical mapping with CARTO were used to achieve fluoroless PFA ablation. Procedures were performed under general anaesthesia. After TOE‐guided transeptal puncture, Pentaray was used to acquire left atrial and pulmonary branch anatomy. The Farawave catheter and Rosen wire were advanced into the left atrium, visualised using carto. The Rosen wire was connected via two alligator clips and visualised as 2mm bipolar catheter (Panel A) and manipulated to select the appropriate pulmonary vein branch. The Farawave catheter is rendered as a circular catheter on CARTO (Panel B), with its formation (flower vs basket) and relation to the Faradrive sheath controlled using tactile sensation and guided with TOE imaging (Panel C). Rotation of the Farawave catheter was visualised by rotational movement on CARTO (Panel B). Post ablation, Pentaray re‐mapping was performed to confirm antral pulmonary vein isolation (Panel D).

**Results:** A total of 8 patients with median age of 66 ± 9.4 underwent fluoroless PFA (3 persistents). Procedure time was 86±8 minutes. Left atrial dwelling time was 51±7 minutes and ablation time was 21±7 minutes. Acute PV isolation was achieved in all patients. There was one patient who experienced pericarditis responding to colchicine, otherwise no complications.

**Conclusions:** A fluoroless workflow for Farawave is feasible and associated with short procedure times.

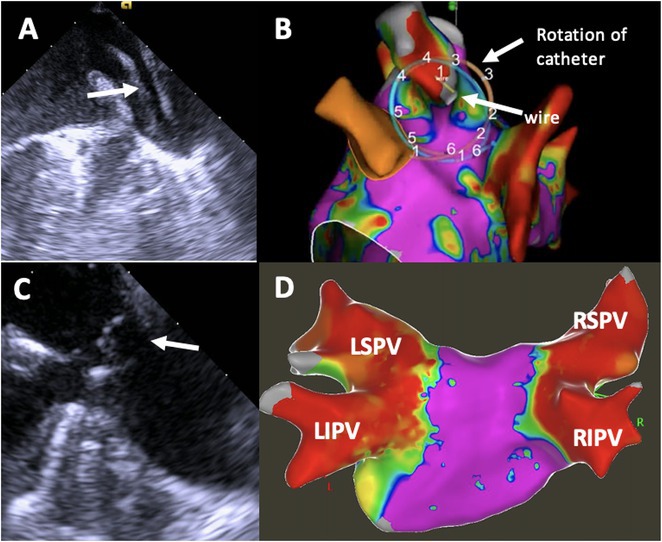

HAEMOLYSIS IN PATIENTS UNDERGONE PULMONARY VEIN ISOLATION USING FARAWAVE

**YEHUAN ZHOU** ^1^, STUART THOMAS^1,2^, LEONADO PASALIC^1^, PIERRE QIAN^1,2^;

^1^Westmead Hospital, Westmead, Australia,^2^University of Sydney, Sydney, Australia.

**Introduction:** Registry data reports haemolysis causing acute renal failure in patients undergone pulmonary vein isolation (PVI) using pulsed‐field ablation (PFA). We aim to determine the presence of haemolysis in patients undergone PFA using FARAWAVE system.

**Methods:** Consecutive patients with paroxysmal or persistent atrial fibrillation (AF) undergone PVI using FARAWAVE system were included. Blood samples were taken via the femoral venous sheath immediately upon gaining access and at end of ablation before venous sheath removal. Blood samples taken include bilirubin, haptoglobin, lactate dehydrogenase, haemoglobin, serum creatinine, free haemoglobin and urine analysis. The delta change of haemolysis markers were analysed using paired‐sample T‐ test with statistical significance defined as p <0.05.

**Results:** A total of 22 patients (median age 50±11.7; 10 persistent AF) were included for analysis. There was a statistically significant change in all haemolysis markers when comparing samples pre and post procedure (Panels A, B & C). The mean differences of bilirubin, lactate dehydrogenase and haptoglobin pre‐procedure and post‐procedure were ‐5.8 μmol/L (95% CI 0.7, 10.9, *p =* 0.029), 33.3 U/L (95% CI 7.5, 59.1, *p =* 0.01), ‐0.12 g/L (95% CI ‐0.17, ‐0.08, *p* <0.001) respectively. Haemoglobin change was statistically insignificant (2.1 g/L, 95% CI ‐1.3, 5.5, *p* = 0.2) (Panel D). There was no evidence of acute renal injury in all patients with an average eGFR difference of 2.65 mL/min/1.73m2 (95% CI ‐0.38, 5.7 *p* = 0.083) (Panel E). No patients had haematuria on urine analysis.

**Conclusions:** Haemolysis has been observed in patients undergoing PVI using Farawave. However, changes in haemoglobin and renal function post‐procedure were statistically insignificant.

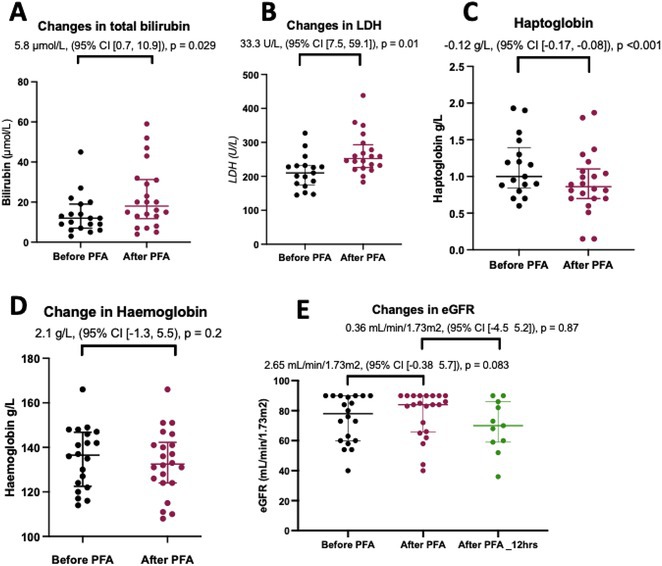

## UNVEILING HEART FAILURE RECOVERY: STANDARD MEDICATION REGIMENS AFTER CARDIAC PHYSIOLOGIC PACING

### **JIANGANG ZOU** ^1^, ENRUI ZHANG^1^, XIAOHAN FAN^2^, YIJIA TANG^3^, LANG HE^4^, FAYUAN FU^5^, BINNI CAI^6^, RUOGU LI^7^, XIAOLIN XUE^8^, RUIQIN XIE^9^, MIN SHEN^10^, LIANGRONG ZHENG^11^, YANGXIN CHEN^12^, LIN CAI^13^

#### ^1^Jiangsu Province Hospital, Nanjing, China,^2^Fuwai hospital, Beijing, China,^3^Sichuan Provincial People's Hospital, Sichuan, China,^4^Zhejiang Greentown Cardiovascular Hospital, Zhejiang, China,^5^Union Hospital, Fujian Medical University, Fujian, China,^6^Xiamen Cardiovascular Hospital, Xiamen, China,^7^Shanghai Chest Hospital, Shanghai, China,^8^the First Affiliated Hospital, Xi'an Jiaotong University, Xi'an, China,^9^The Second Hospital of Hebei Medical University, Shijiazhuang, China,^10^Xijing Hospital, Xian, China,^11^The First Affiliated Hospital, College of Medicine, Zhejiang University, Zhejiang, China,^12^Sun Yat‐sen Memorial Hospital, Guangzhou, China,^13^Third People's Hospital of Chengdu, Chengdu, China

**Introduction:** After resynchronizing conduction delays in heterogeneous heart failure (HF) patients, the potential benefits of standard medication regimens following cardiac physiologic pacing (CPP) remain elusive. This study aims to determine whether the standardization of post‐CPP medication regimens promotes prognosis in Class I and non‐Class I HF patients.

**Methods:** Patients who met resynchronization indication (QRS≥130ms, LVEF≤35%) undergoing CPP at 13 centers nationwide, were included and stratified into two groups: Class I (SR, LBBB, QRS≥150ms) and non‐Class I indication group. Post‐CPP medication regimen was categorized as 'standard' vs 'non‐standard' using a previously published standard HF medication score (≥4 vs <4) that is based on the dosage and class of the 4 pillars of HF medications. Primary outcome was major adverse cardiac events (MACE), including all‐cause mortality, HF hospitalization, and ventricular tachyarrhythmic events. Clinical metrics for cardiac function were also analyzed.

**Results:** A total of 1180 patients were screened and 529 were enrolled. In 316 Class I patients, standard medication regimen didn't significantly impact improvement in clinical outcomes and cardiac function (6m ΔLVEF, P=.09, P psm=.32; MACE, P unadj‐KM=.55, P iptw‐KM=.55, P psm‐KM=.89). CPP modality (LBBP vs BiVP) was the major determinant for improved outcomes (6m ΔLVEF, P<.01, P psm<.01; MACE, P unadj‐KM<.01, P iptw‐KM<.01, P psm‐KM<.01). In 213 non‐Class I patients, standard medication regimen significantly improved cardiac function and clinical outcomes (6m ΔLVEF, P<.01, P psm=.04; MACE, P unadj‐KM<.01, P iptw‐KM<.01, P psm‐KM<.01), while CPP modality didn't impact clinical outcomes (6m ΔLVEF, P=.31, P psm=.89; MACE, P unadj‐KM=.32, P iptw‐KM=.45, P psm‐KM=.49).

**Conclusions:** The implications of post‐CPP medication regimens differ among heterogeneous HF patients. Post‐CPP medication regimens don't affect prognosis for Class I patients undergoing CPP. For non‐Class I patients, regardless of CPP modality, standard medication regimen determines outcomes.